# Supplementary material for: Network Analysis Reveals the Molecular Bases of Statin Pleiotropy That Vary with Genetic Background
Source: Microbiol Spectr. 2023 Mar 22;11(2):e04148-22. doi: 10.1128/spectrum.04148-22 (PMC10100750; doi:10.1128/spectrum.04148-22)
Supplement: Supplemental file 1 — Supplemental material. Download spectrum.04148-22-s0001.pdf, PDF file, 9.3 MB [file spectrum.04148-22-s0001.pdf]

Supplemental Material.

Please refer to bookmarks to move between figures and tables.

Supplemental Material Figure legends

Figure S1. **Genetic interaction networks connecting validated interactions hypersensitive to atorvastatin.** Genetic interactions networks for the *HMG1* query (upper panel) and *BTS1* query (lower panel) were constructed using GeneMania and visualised using NetworkAnalyst. Darker nodes in each network are the input genes.

Figure S2. **Protein-protein interaction networks connecting validated interactions hypersensitive to atorvastatin.** Protein-protein interaction networks for the *HMG1* query (upper panel) and *BTS1* query (lower panel) were constructed using STRING and visualised using NetworkAnalyst. Darker nodes in each network are the input genes.

Figure S3. **Network topology centrality analyses of GINs and PPINs identify key *HMG1*/*BTS1* interactors for atorvastatin sensitivity.** Centrality measurements (degree, closeness and betweenness) were calculated for each gene and visualised in a 3D plot.

Figure S4. **Metabolic pathway enrichment of modules in protein-protein interaction networks for atorvastatin sensitivity.** Bubble plots showing enrichment for each of the modules (named for their genetic background) identified through community analysis for *HMG1* (top panel) and *BTS1* (bottom panel) interactions. The size of the bubbles is relative to the enrichment score for each pathway, while the intensity of the colours is relative to the adjusted *P*-value. The x axis labels show the genetic background followed by the number of modules. Numbers missing in the sequence are modules without significantly enriched pathways.

Figure S5. **Network centrality of genes behind hypersensitivity to atorvastatin for *HMG1* interactors overlap in three genetic backgrounds.** Genes that ranked in the top ten centrality measurements were found to confirm phenotypic findings. Centrality measurements (betweeness, closeness and degree) were calculated in NetworkAnalyzer app in Cytoscape (31) and networks were built in Cytoscape. The red outline points to highly central genes.

Figure S6. **Network centrality of genes behind hypersensitivity to atorvastatin for *BTS1* interactors overlap in three genetic backgrounds.** Genes that ranked in the top ten centrality measurements were found to confirm phenotypic findings. Centrality measurements (betweeness, closeness and degree) were calculated in NetworkAnalyzer app in Cytoscape (31) and networks were built in Cytoscape. The red outline points to highly central genes.

Figure S7. **Metabolic pathway enrichment of modules that did not overlap in single-layer and multi-layer network community analysis.** Bubble plots showing enrichment for each of the modules (named for their genetic background) identified through community analysis for *HMG1* (top panel) and *BTS1* (bottom panel) interactions that were unique to either single-layer (left panel) or multi-layer (right panel) analyses. The size of the bubbles is relative to the enrichment score for each pathway, while the intensity of the colours is relative to the adjusted *P* value. The x axis labels show the genetic background followed by the number of modules.

Figure S1

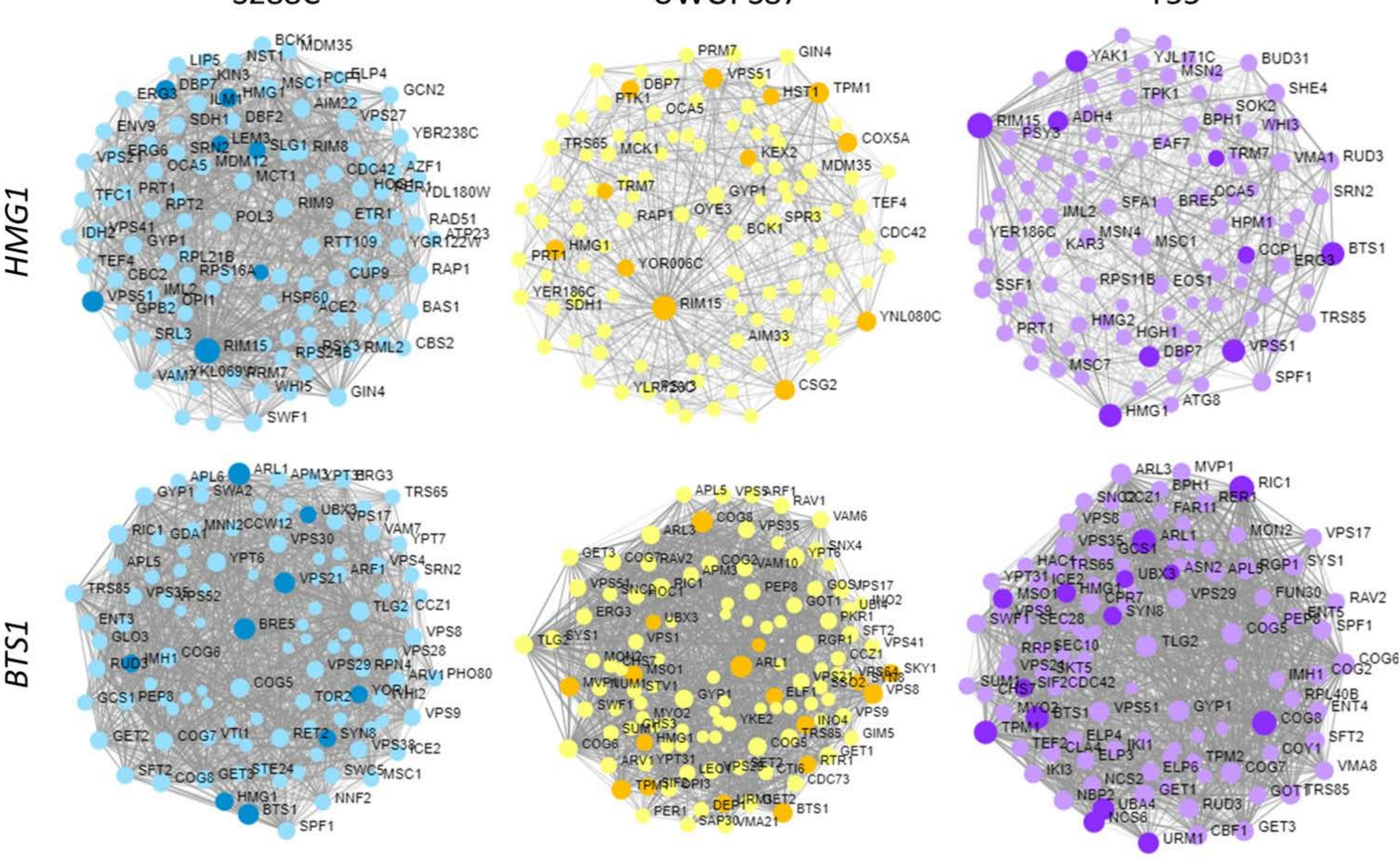

Figure S2

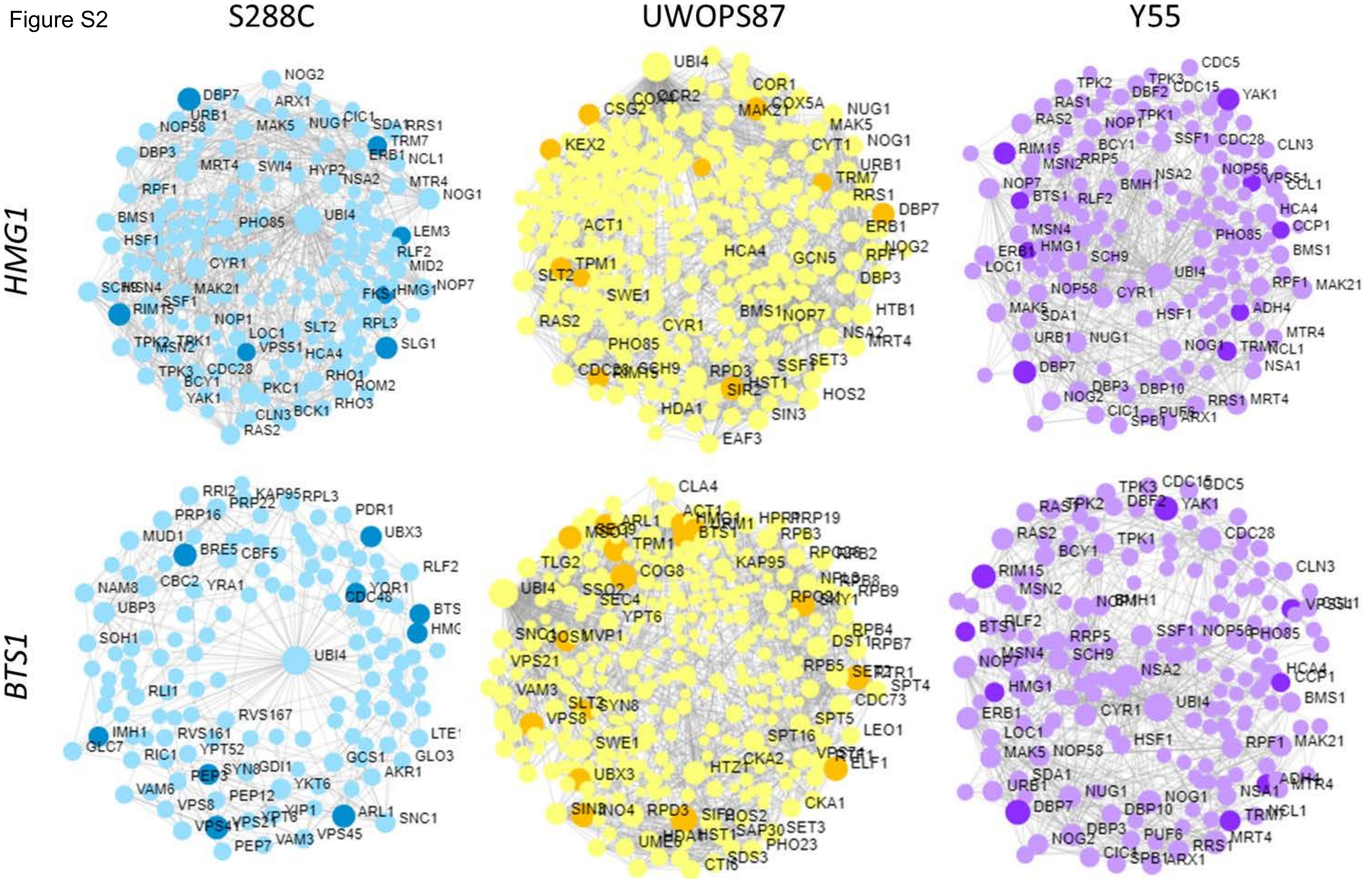

Figure S3

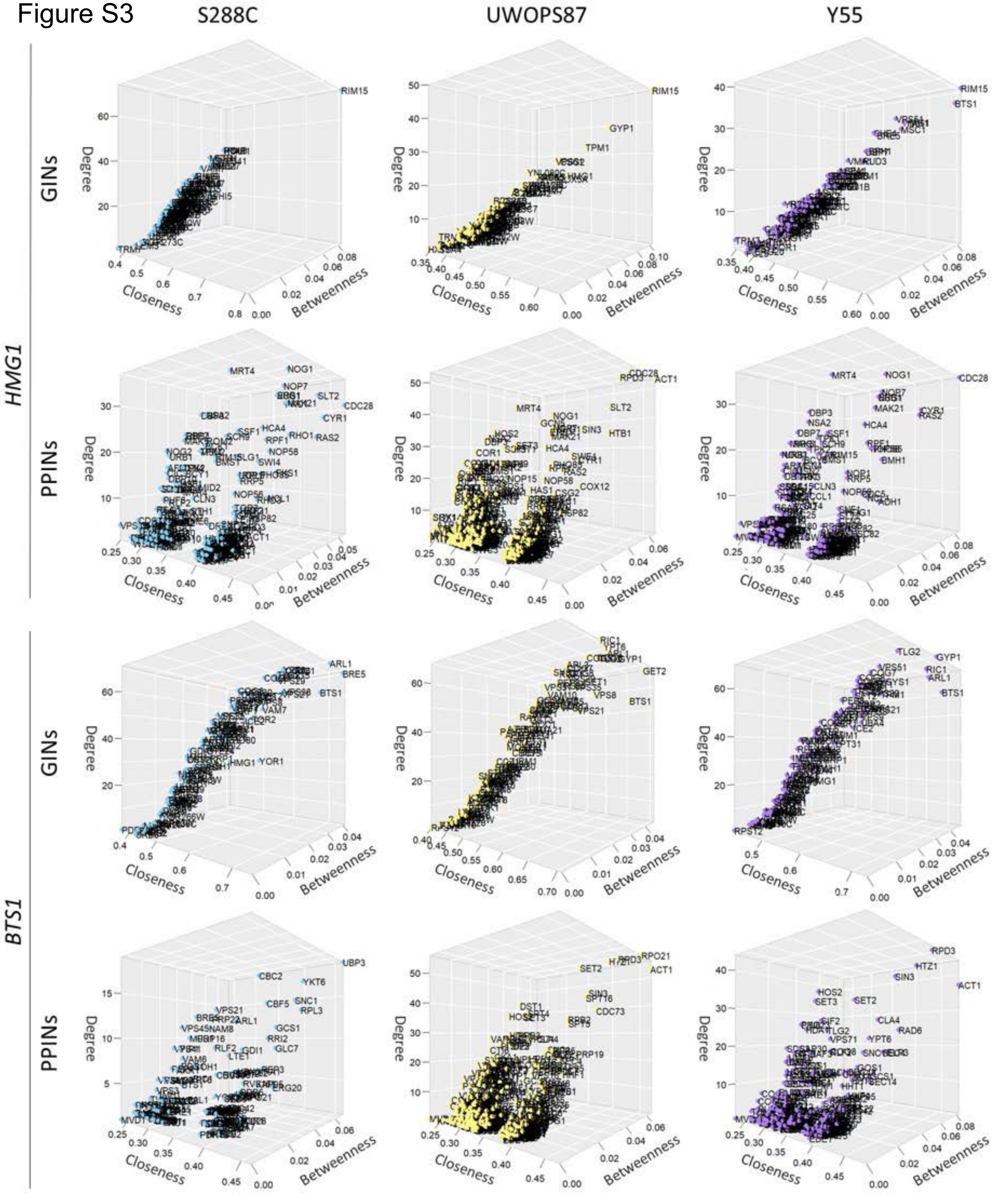

S288C
UWOPS87
Y55
HMG1
BTS1
GINs
PPINs
GINs
PPINs
Degree
Closeness
Betweenness

Figure S4

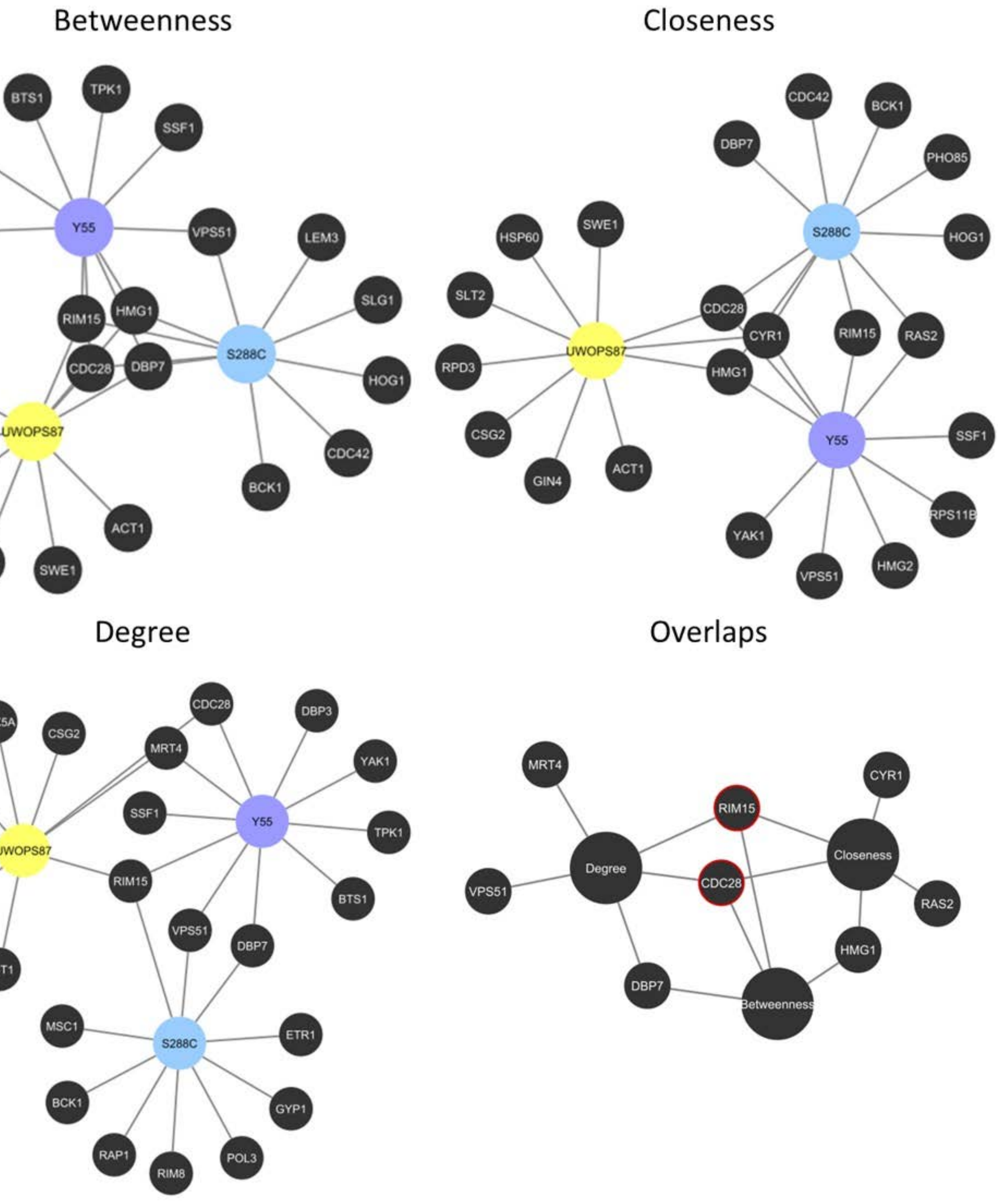

Betweenness
Closeness
Degree
Overlaps
Y55
S288C
UWOPS87
BTS1
TPK1
SSF1
YAK1
DBP3
VPS51
LEM3
SLG1
HOG1
CDC42
BCK1
RIM15
HMG1
CDC28
DBP7
HST1
TPM1
COX5A
CSG2
SWE1
ACT1
PHO85
HSP60
SLT2
RPD3
GIN4
CYR1
RAS2
RPS11B
HMG2
MRT4
MSC1
ETR1
GYP1
POL3
RIM8
RAP1
Degree
Closeness
Betweenness

Figure S5

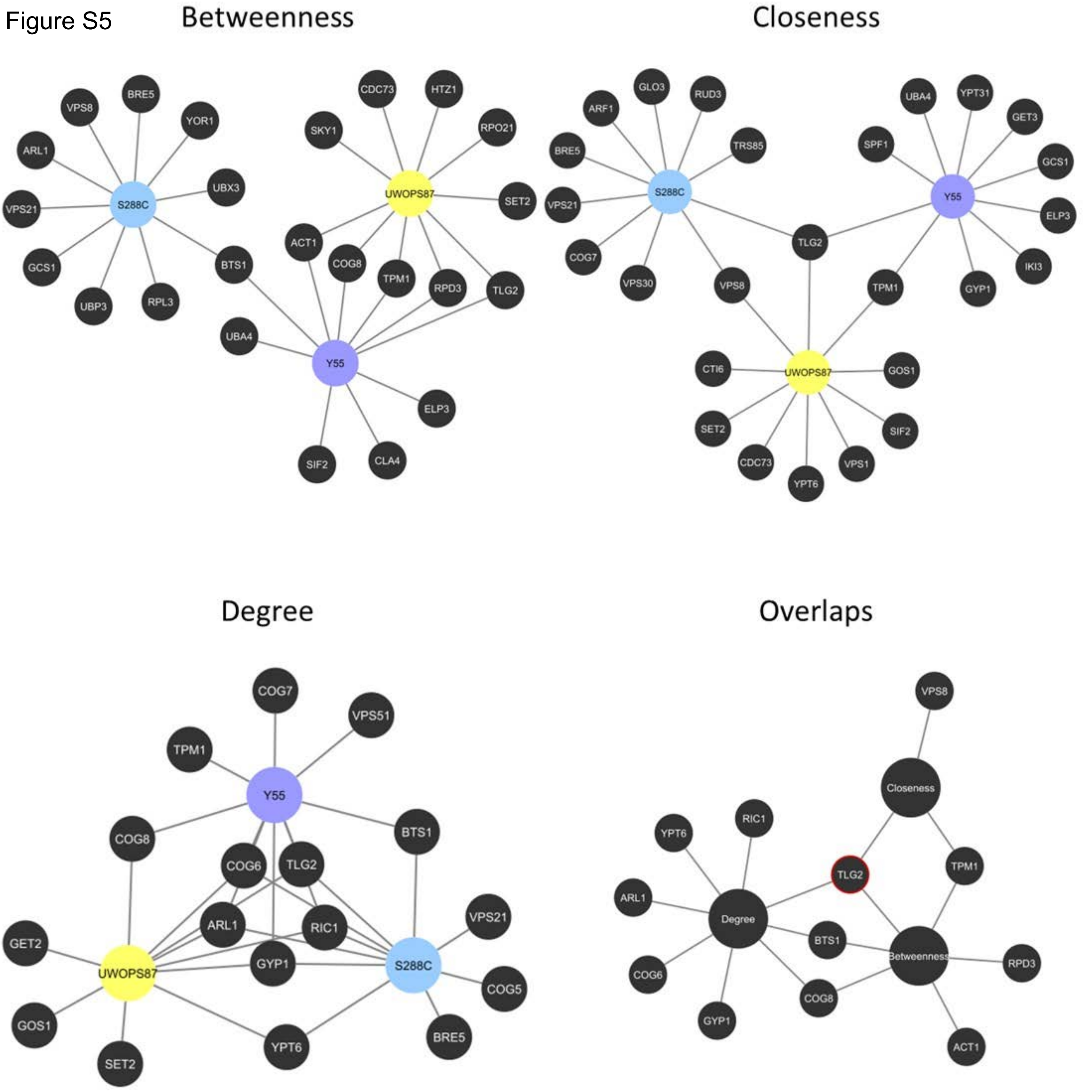
Betweenness
Closeness
Degree
Overlaps
S288C
UWOPS87
Y55
VPS8
BRE5
YOR1
ARL1
UBX3
VPS21
GCS1
UBP3
RPL3
BTS1
CDC73
HTZ1
SKY1
RPO21
SET2
ACT1
COG8
TPM1
RPD3
TLG2
UBA4
ELP3
SIF2
CLA4
GLO3
RUD3
ARF1
TRS85
COG7
VPS30
UBA4
YPT31
GET3
SPF1
GCS1
ELP3
IKI3
GYP1
CTI6
GOS1
YPT6
VPS1
VPS51
COG6
RIC1
GET2
COG5
Closeness
Degree
Betweenness

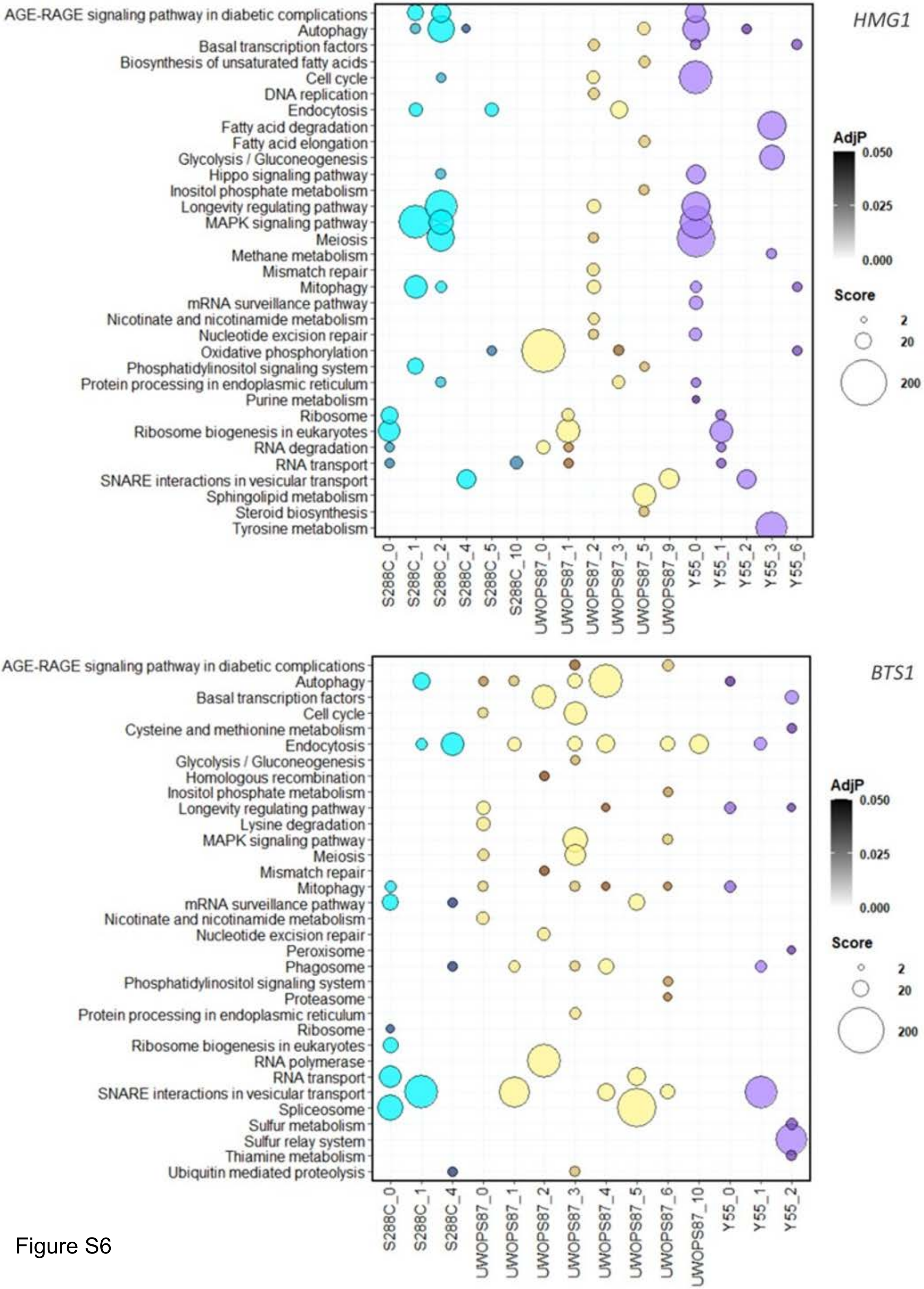
HMG1
AGE-RAGE signaling pathway in diabetic complications
Autophagy
Basal transcription factors
Biosynthesis of unsaturated fatty acids
Cell cycle
DNA replication
Endocytosis
Fatty acid degradation
Fatty acid elongation
Glycolysis / Gluconeogenesis
Hippo signaling pathway
Inositol phosphate metabolism
Longevity regulating pathway
MAPK signaling pathway
Meiosis
Methane metabolism
Mismatch repair
Mitophagy
mRNA surveillance pathway
Nicotinate and nicotinamide metabolism
Nucleotide excision repair
Oxidative phosphorylation
Phosphatidylinositol signaling system
Protein processing in endoplasmic reticulum
Purine metabolism
Ribosome
Ribosome biogenesis in eukaryotes
RNA degradation
RNA transport
SNARE interactions in vesicular transport
Sphingolipid metabolism
Steroid biosynthesis
Tyrosine metabolism
S288C_0
S288C_1
S288C_2
S288C_4
S288C_5
S288C_10
UWOPS87_0
UWOPS87_1
UWOPS87_2
UWOPS87_3
UWOPS87_5
UWOPS87_9
Y55_0
Y55_1
Y55_2
Y55_3
Y55_6
AdjP
0.050
0.025
0.000
Score
2
20
200
BTS1
AGE-RAGE signaling pathway in diabetic complications
Autophagy
Basal transcription factors
Cell cycle
Cysteine and methionine metabolism
Endocytosis
Glycolysis / Gluconeogenesis
Homologous recombination
Inositol phosphate metabolism
Longevity regulating pathway
Lysine degradation
MAPK signaling pathway
Meiosis
Mismatch repair
Mitophagy
mRNA surveillance pathway
Nicotinate and nicotinamide metabolism
Nucleotide excision repair
Peroxisome
Phagosome
Phosphatidylinositol signaling system
Proteasome
Protein processing in endoplasmic reticulum
Ribosome
Ribosome biogenesis in eukaryotes
RNA polymerase
RNA transport
SNARE interactions in vesicular transport
Spliceosome
Sulfur metabolism
Sulfur relay system
Thiamine metabolism
Ubiquitin mediated proteolysis
S288C_0
S288C_1
S288C_4
UWOPS87_0
UWOPS87_1
UWOPS87_2
UWOPS87_3
UWOPS87_4
UWOPS87_5
UWOPS87_6
UWOPS87_10
Y55_0
Y55_1
Y55_2
AdjP
0.050
0.025
0.000
Score
2
20
200

Figure S6

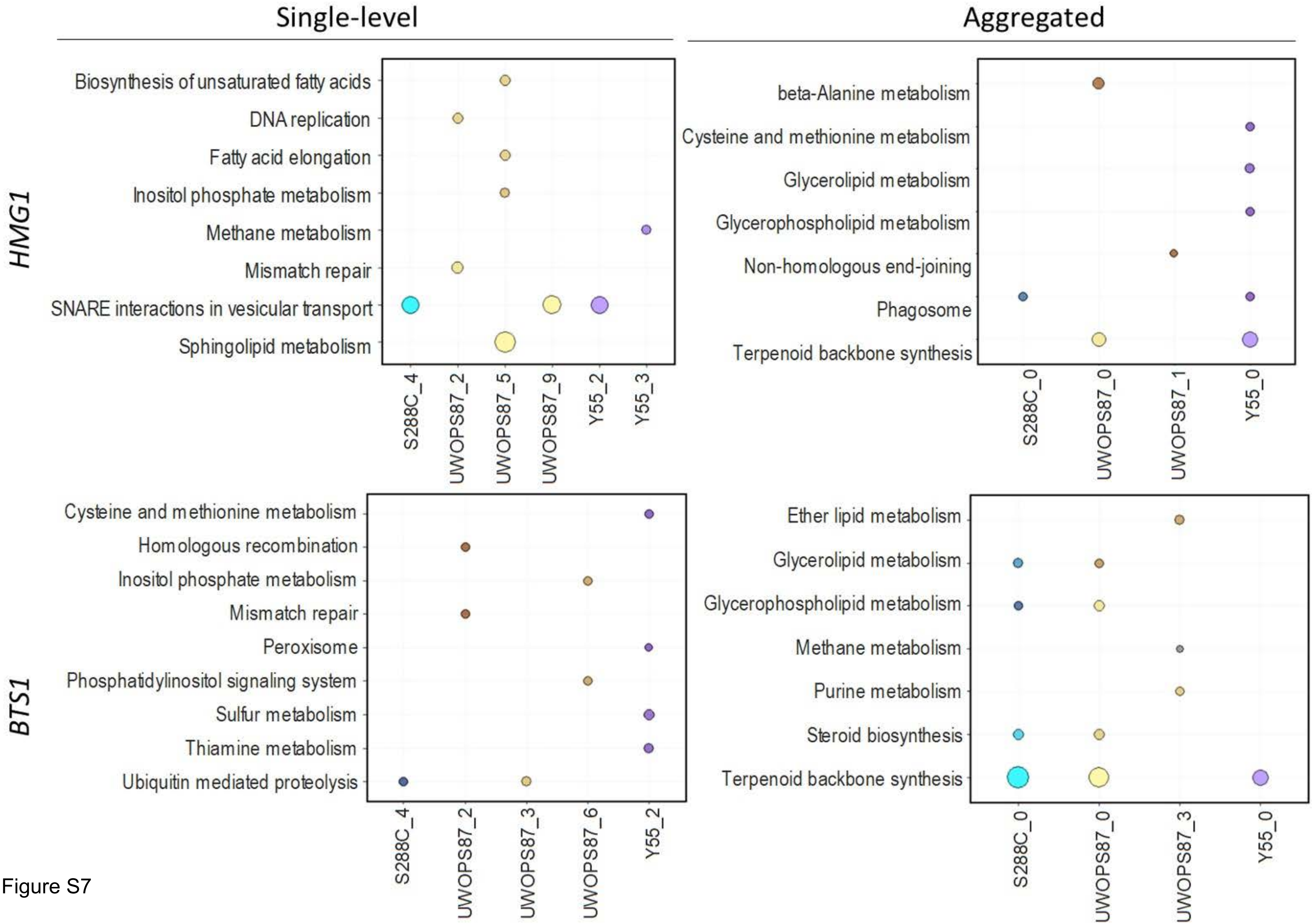
Single-level
Aggregated
HMG1
BTS1
Biosynthesis of unsaturated fatty acids
DNA replication
Fatty acid elongation
Inositol phosphate metabolism
Methane metabolism
Mismatch repair
SNARE interactions in vesicular transport
Sphingolipid metabolism
S288C_4
UWOPS87_2
UWOPS87_5
UWOPS87_9
Y55_2
Y55_3
beta-Alanine metabolism
Cysteine and methionine metabolism
Glycerolipid metabolism
Glycerophospholipid metabolism
Non-homologous end-joining
Phagosome
Terpenoid backbone synthesis
S288C_0
UWOPS87_0
UWOPS87_1
Y55_0
Cysteine and methionine metabolism
Homologous recombination
Inositol phosphate metabolism
Mismatch repair
Peroxisome
Phosphatidylinositol signaling system
Sulfur metabolism
Thiamine metabolism
Ubiquitin mediated proteolysis
S288C_4
UWOPS87_2
UWOPS87_3
UWOPS87_6
Y55_2
Ether lipid metabolism
Glycerolipid metabolism
Glycerophospholipid metabolism
Methane metabolism
Purine metabolism
Steroid biosynthesis
Terpenoid backbone synthesis
S288C_0
UWOPS87_0
UWOPS87_3
Y55_0

Figure S7

Supplemental Tables Legends

Table S1. List of atorvastatin *hmg1Δ xxxΔ* and *bts1Δ xxxΔ* that enhanced sensitivity to atorvastatin.

Table S2. Top betweenness centrality measurements for GINs and PPINs in three genetic backgrounds.

Table S3. Human orthologues of top validated hits and centralities used as input for enrichment analysis in Drug Signature Database.

Table S4. Most of the top 20 drugs that share signature genes with atorvastatin identified have anticancer activity but have not been investigated for synergy with statins.

Table S5. Strains used in this study.

Table S6. PCR primers and conditions used in this study.

Table S7. Raw dataset for *hmg1Δ xxxΔ* screening with atorvastatin.

Table S8. Raw dataset for *bts1Δ xxxΔ* screening with atorvastatin.

Table S9. Raw dataset for xxxΔ screening with atorvastatin.

Table S1. List of atorvastatin hmg1Δ xxxΔ and bts1Δ xxxΔ that enhanced sensitivity to atorvastatin

Query	Background	ORF	Gene	Name	Description	Human orthologue(s)	Cancer links	Diabetes	Myopathies	Rhabdomyolisis	Statins
HMG1	SUY	YKR024C	*DBP7*	Dead Box Protein	RNA helicase of the DEAD-box family involved in ribosomal biogenesis	DDX41, DDX46	Yes				
HMG1	SUY	YFL033C	*RIM15*	Regulator of IME2	Protein kinase involved in cell proliferation in response to nutrients	MAST1, MAST2, MAST3, MAST4, MASTL	Yes	Yes			
HMG1	SUY	YBR061C	*TRM7*	Transfer RNA Methyltransferase	Ribose methyltransferase that methylates the tRNA-Phe, -Trp, and -Leu at the anticodon loop	FTSJ1	Yes				
HMG1	SUY	YKR020W	*VPS51*	Vacuolar Protein Sorting	Required for the recycling of proteins from endosomes to the late Golgi	GARP complex	Yes				
HMG1	S	YNL323W	*LEM3*	Effect	translocation of phospholipids and alkylphosphocholine drugs	TMEM30B,	Yes	Yes			
HMG1	S	YOR008C	*SLG1*	Synthetic Lethal with Gap	Sensor-transducer of the stressactivated PKC1-MPK1 kinase pathway; involved in organization of the actin cytoskeleton	MUC15	Yes				
HMG1	U	YNL052W	*COX5A*	Cytochrome c OXidase	Subunit Va of cytochrome c oxidase, the terminal member of the mitochondrial inner membrane electron transport chain	COX4I1, COX4I2	Yes	Yes			
HMG1	U	YBR036C	*CSG2*	Calcium Sensitive Growth	ER membrane protein with a role in mannosylation of inositolphosphorylceramide required for growth at high calcium concentrations	None					
HMG1	U	YNL080C	*EOS1*	ER-localized and Oxidants Sensitive	Protein involved in N-glycosylation; deletion mutation confers sensitivity to oxidative stress	None					
HMG1	U	YOL068C	*HST1*	Homolog of SIR Two	NAD(+)-dependent histone deacetylase; involved in meiotic repression and telomere maintenance	SIRT1, SIRT4, SIRT5	Yes	Yes	Yes		Yes
HMG1	U	YNL238W	*KEX2*	Killer EXpression defective	Kexin, a calcium-dependent serine protease with a role in the secretory pathway	FURIN, PCSK6, PCSK5, PCSK4, PCSK1, PCSK2, PCSK7	Yes	Yes			Yes
HMG1	U	YNL079C	*TPM1*	TroPoMyosin	Major isoform of tropomyosin, which stabilizes actin cables and filaments	TPM1, TPM2, TPM3, TPM4	Yes	Yes	Yes		
HMG1	U	YOR006C	*TSR3*	Twenty S rRNA accumulation	Protein required for 20S pre-rRNA processing	TSR3					
HMG1	Y	YGL256W	*ADH4*	Alcohol DeHydrogenase	Alcohol32 dehydrogenase that has induced transcription upon zinc deficiency	ADHFE1	Yes				
HMG1	Y	YPL069C	*BTS1*	Bet Two Suppressor	Geranylgeranyl diphosphate synthase (GGPPS); suppressor of bet2 mutation that causes defective vesicular traffic	GGPS1	Yes	Yes	Yes		Yes
HMG1	Y	YKR066C	*CCP1*	Cytochrome c Peroxidase	Mitochondrial cytochrome-c peroxidase involved in the response to oxidative stress	None					
HMG1	Y	YJL141C	*YAK1*	Yet Another Kinase	Serine-threonine protein kinase sensitive to glucose that phosphorylates Crf1p in response to nutrient deprivation inhibiting transcription of ribosomal genes	HIPK3, HIPK4, DYRK1A, HIPK1, HIPK2, DYRK3, PRPF4B, DYRK1B	Yes	Yes	Yes		
BTS1	SUY	YBR164C	*ARL1*	ADP-Ribosylation	Soluble GTPase of the Ras superfamily that	ARL8A, ARL1,	Yes	Yes			
BTS1	SUY	YAL014C	*SYN8*	SYNtaxin	Endosomal SNARE related to mammalian syntaxin 8	STX6, STX8, STX10	Yes	Yes			
BTS1	SUY	YDL091C	*UBX3*	UBiquitin regulatory X	Vesicle component required for efficient clathrin-mediated endocytosis that interacts with CDC48	FAF1, UBXN10, FAF2, UBXN8	Yes	Yes			Yes
BTS1	UY	YML071C	*COG8*	Conserved Oligo-meric Golgi complex	Component of the oligomeric Golgi complex that mediates fusion of transport vesicles to Golgi compartments	COG8	Yes				
BTS1	UY	YNR049C	*MSO1*	Multicopy suppressor of Sec1	Lipid-interacting protein in SNARE complex assembly machinery with a role in late secretion	None					
BTS1	UY	YBR103W	*SIF2*	Sir4p-Interacting Factor	Subunit of Set3C histone deacetylase complex that antagonizes telomeric silencing	WDR17, TBL1X, TBL1XR1, THOC3, TBL1Y	Yes	Yes			
BTS1	UY	YNL079C	*TPM1*	TroPoMyosin	Major isoform of tropomyosin, which stabilises actin cables and filaments	TPM1, TPM2, TPM3, TPM4	Yes	Yes	Yes		

Query	Background	ORF	Gene	Name	Description	Human orthologue(s)	Cancer links	Diabetes	Myopathies	Rhabdomyolisis	Statins
BTS1	UY	YIL008W	*URM1*	Ubiquitin Related Modifier	Ubiquitin-like protein involved in thiolation of cytoplasmic tRNAs that also has roles in oxidative stress response	URM1	Yes				
BTS1	S	YNR051C	*BRE5*	BREfeldin A sensitivity	Ubiquitin protease cofactor; forms deubiquitination complex with UBP3 and deubiquitinate COPII and COPI vesicle coat constituents, SEC23 and SEC27	G3BP1, G3BP2	Yes	Yes	Yes		Yes
BTS1	S	YLR309C	*IMH1*	Integrins and Myosins significant Homology	Protein involved in vesicular transport between an endosome and the Golgi	None					
BTS1	S	YOR089C	*VPS21*	Vacuolar Protein Sorting	Endosomal Rab family GTPase required for endosomal localization of the CORVET complex and has a role in autophagy and ionic stress tolerance; geranylgeranylation required for membrane association	RAB31, RAB24, RAB20, RAB22A, RAB5A, RAB5B, RAB5C, RAB17	Yes	Yes			Yes
BTS1	S	YGR281W	*YOR1*	Yeast Oligomycin Resistance	Plasma membrane ATP-binding cassette (ABC) transporter of drugs	ABCC5, ABCC9, ABCC4, CFTR, ABCC2, ABCC6, ABCC1, ABCC8, ABCC11, ABCC3, ABCC10, ABCC12	Yes	Yes	Yes	Yes	Yes
BTS1	U	YKL160W	*ELF1*	ELongation Factor	Transcription elongation factor with a role in chromatin structure	ELOF1					
BTS1	U	YOL108C	*INO4*	INOsitol requiring	Transcription factor involved in phospholipid synthesis	TFEC, MITF, TFE3, USF1, USF2, TFEB	Yes	Yes	Yes	Yes	Yes
BTS1	U	YMR004W	*MVP1*	Multi-copy suppressor of vps1	Protein required for sorting proteins to the vacuole	SNX18, SNX32, SNX33, SNX5, SNX8, SNX10, SNX11, SNX12, SNX30, SNX7, SNX9, SNX6, SNX1, SNX2, SNX3	Yes	Yes			
BTS1	U	YER139C	*RTR1*	Regulator of TRanscription	Protein phosphatase that dephosphorylates T1 and S5 of RNA polymerase II largest subunit	RPAP2			Yes		
BTS1	U	YMR216C	*SKY1*	SRPK1-like Kinase in Yeast	SR protein kinase (SRPK) involved in mRNA 3' splice site recognition with PRP8 and CDC40 and has a role in cation uptake and homeostasis	SRPK1, SRPK2, SRPK3	Yes	Yes	Yes		
BTS1	U	YAL002W	*VPS8*	Vacuolar Protein Sorting	Component of the CORVET complex involved in endosomal vesicle tethering that interacts with VPS21	VPS8, VPS41	Yes	Yes			
BTS1	U	YOR172W	*YRM1*	Yeast Reveromy-cin resistance Modulator	Zinc finger transcription factor involved in multidrug resistance	None					
BTS1	Y	YGR124W	*ASN2*	ASparagiNe requiring	Asparagine synthetase; catalyzes the synthesis of L-asparagine from L-aspartate	ASNS	Yes				
BTS1	Y	YGL211W	*NCS6*	Needs Cla4 to Survive	Protein required for uridine thiolation of Gln, Lys, and Glu tRNAs with a role in urmylation	CTU1					
BTS1	Y	YLR039C	*RIC1*	RIbosome Control	Protein involved in retrograde transport to the cis-Golgi network involved in transcription of rRNA and ribosomal genes	RIC1		Yes			
BTS1	Y	YHR111W	*UBA4*	UBiquitin-Activating	E1-like protein that activates URM1 before urmylation	MOCS3, UBA5	Yes				
						TOTAL	29	21	10	2	8

Key

Query	Refers to query strain gene deletion
Backgroui	Refers to genetic background: S = S288C; U = UWOPS87; Y = Y55
ORF	Refers to the open reading frame of the second gene deletion
Gene	Refers to the second gene deletion
Name	Refers to the name of the second gene deletion

Query	Background	ORF	Gene	Name	Description	Human orthologue(s)	Cancer links	Diabetes	Myopathies	Rhabdomyolisis	Statins

Descriptic Refers to the description of the second gene deletion

Human or Refers to the human orthologue(s) of the second gene deletion

Type text here

Table S2. Top betweenness centrality measurements for GINs and PPINs in three genetic backgrounds

Query Gene	Genetic background	Interaction	Total number of nodes	Total number of edges	Gene name	Betweenness	Closeness	Degree
HMG1	S288C	GIN	117	1478	*WHI5*	0.03	0.6	25
					RAP1	0.02	0.6	44
					POL3	0.02	0.6	44
		PPIN	203	950	*PKC1*	0.04	0.4	31
					CYR1	0.04	0.5	27
					RAS2	0.4	0.5	23
	UWOPS87	GIN	112	702	*GYP1*	0.07	0.6	37
					TPM1	0.05	0.6	31
					HMG1	0.05	0.5	23
		PPIN	372	1858	*ACT1*	0.07	0.5	53
					HTB1	0.04	0.4	34
					RPD3	0.04	0.4	50
	Y55	GIN	109	732	*BTS1*	0.08	0.6	37
					YAK1	0.05	0.6	31
					HMG1	0.05	0.6	31
		PPIN	186	835	*CYR1*	0.05	0.5	28
					RAS2	0.05	0.5	27
					ADH1	0.04	0.4	9
BTS1	S288C	GIN	109	1873	*ARL1*	0.03	0.7	71
					BTS1	0.04	0.7	61
					YOR1	0.03	0.6	30
					VPS38	0.02	0.7	58
					VPS21	0.02	0.7	57
		PPIN	139	388	*ACT1*	0	0.4	4
					UBP3	0.07	0.5	19
					BRE5	0.04	0.7	70
					GYP1	0.01	0.7	61
					RIC1	0.02	0.7	65
					RPL3	0.04	0.4	13
					YKT6	0.04	0.4	16
					SNC1	0.04	0.4	14
	UWOPS87	GIN	116	1901	*ARL1*	0.02	0.7	66
					BTS1	0.05	0.6	55
					GET2	0.04	0.7	66
					VPS8	0.02	0.6	54
					VPS21	0.02	0.6	48
		PPIN	370	1944	*ACT1*	0.07	0.5	53
					RPD3	0.04	0.5	53
					GYP1	0.03	0.7	66
					RIC1	0.01	0.7	68
					RPO21	0.05	0.5	56
					HTZ1	0.05	0.4	54
					RPD3	0.04	0.5	53
	Y55	GIN	113	1834	*ARL1*	0.03	0.7	64
					BTS1	0.05	0.7	61
					VPS21	0.02	0.6	50
					TPM1	0.02	0.7	55
					UBA4	0.02	0.6	45
		PPIN	233	1018	*ACT1*	0.08	0.5	37
					RPD3	0.06	0.5	44
					GYP1	0.03	0.7	70
					RIC1	0.03	0.7	66
					HTZ1	0.06	0.4	41
					CLA4	0.05	0.4	27
					YPT6	0.04	0.4	22

Table S3. Human orthologues of top validated hits and centralities used as input for enrichment analysis in Drug Signature Database

Yeast query	Yeast interactor	Human orthologue	Yeast query	Yeast interactor	Human orthologue	Yeast query	Yeast interactor	Human orthologue	Yeast query	Yeast interactor	Human orthologue
HMG1/BTS1	DBP7	DDX41	HMG1	YAK1	HIPK2	BTS1	INO4	MITF	BTS1	SKY1	SRPK3
HMG1/BTS1	DBP7	DDX46	HMG1	YAK1	HIPK3	BTS1	INO4	TFE3	BTS1	SYN8	STX10
HMG1/BTS1	RIM15	MAST1	HMG1	YAK1	HIPK4	BTS1	INO4	TFEB	BTS1	SYN8	STX6
HMG1/BTS1	RIM15	MAST2	HMG1	YAK1	PRPF4B	BTS1	INO4	TFEC	BTS1	SYN8	STX8
HMG1/BTS1	RIM15	MAST3	BTS1	ACT1	ACTA1	BTS1	INO4	USF1	BTS1	TLG2	STX16
HMG1/BTS1	RIM15	MAST4	BTS1	ACT1	ACTA2	BTS1	INO4	USF2	BTS1	TLG2	STX16-NPEPL1
HMG1/BTS1	RIM15	MASTL	BTS1	ACT1	ACTB	BTS1	MVP1	SNX1	BTS1	UBA4	MOCS3
HMG1/BTS1	TPM1	TPM1	BTS1	ACT1	ACTBL2	BTS1	MVP1	SNX10	BTS1	UBA4	UBA5
HMG1/BTS1	TPM1	TPM2	BTS1	ACT1	ACTC1	BTS1	MVP1	SNX11	BTS1	UBX3	FAF1
HMG1/BTS1	TPM1	TPM3	BTS1	ACT1	ACTG1	BTS1	MVP1	SNX12	BTS1	UBX3	FAF2
HMG1/BTS1	TPM1	TPM4	BTS1	ACT1	ACTG2	BTS1	MVP1	SNX18	BTS1	UBX3	UBXN10
HMG1	ADH4	ADHFE1	BTS1	ACT1	ACTL8	BTS1	MVP1	SNX2	BTS1	UBX3	UBXN8
HMG1	BTS1	GGPS1	BTS1	ACT1	ACTR1A	BTS1	MVP1	SNX3	BTS1	URM1	URM1
HMG1	COX5A	COX4I1	BTS1	ACT1	ACTR1B	BTS1	MVP1	SNX30	BTS1	VPS21	RAB17
HMG1	COX5A	COX4I2	BTS1	ACT1	ACTRT1	BTS1	MVP1	SNX32	BTS1	VPS21	RAB20
HMG1	HST1	SIRT1	BTS1	ACT1	ACTRT2	BTS1	MVP1	SNX33	BTS1	VPS21	RAB22A
HMG1	HST1	SIRT4	BTS1	ACT1	ACTRT3	BTS1	MVP1	SNX5	BTS1	VPS21	RAB24
HMG1	HST1	SIRT5	BTS1	ACT1	POTEE	BTS1	MVP1	SNX6	BTS1	VPS21	RAB31
HMG1	KEX2	FURIN	BTS1	ACT1	POTEKP	BTS1	MVP1	SNX7	BTS1	VPS21	RAB5A
HMG1	KEX2	PCSK1	BTS1	ARL1	ARL1	BTS1	MVP1	SNX8	BTS1	VPS21	RAB5B
HMG1	KEX2	PCSK2	BTS1	ARL1	ARL15	BTS1	MVP1	SNX9	BTS1	VPS21	RAB5C
HMG1	KEX2	PCSK4	BTS1	ARL1	ARL6	BTS1	NCS6	CTU1	BTS1	VPS8	VPS41
HMG1	KEX2	PCSK5	BTS1	ARL1	ARL8A	BTS1	RIC1	RIC1	BTS1	VPS8	VPS8
HMG1	KEX2	PCSK6	BTS1	ARL1	ARL8B	BTS1	RPD3	HDAC1	BTS1	YOR1	ABCC1
HMG1	KEX2	PCSK7	BTS1	ASN2	ASNS	BTS1	RPD3	HDAC2	BTS1	YOR1	ABCC10
HMG1	LEM3	TMEM30A	BTS1	BRE5	G3BP1	BTS1	RPD3	HDAC3	BTS1	YOR1	ABCC11
HMG1	LEM3	TMEM30B	BTS1	BRE5	G3BP2	BTS1	RPD3	HDAC8	BTS1	YOR1	ABCC12
HMG1	LEM3	TMEM30C	BTS1	CDC28	CDK1	BTS1	RTR1	RPAP2	BTS1	YOR1	ABCC2
HMG1	SLG1	MUC15	BTS1	CDC28	CDK2	BTS1	SIF2	TBL1X	BTS1	YOR1	ABCC3
HMG1	TRM7	FTSJ1	BTS1	CDC28	CDK3	BTS1	SIF2	TBL1XR1	BTS1	YOR1	ABCC4
HMG1	TSR3	TSR3	BTS1	CDC28	CDK4	BTS1	SIF2	TBL1Y	BTS1	YOR1	ABCC5
HMG1	YAK1	DYRK1A	BTS1	CDC28	CDK6	BTS1	SIF2	THOC3	BTS1	YOR1	ABCC6
HMG1	YAK1	DYRK1B	BTS1	COG8	COG8	BTS1	SIF2	WDR17	BTS1	YOR1	ABCC8
HMG1	YAK1	DYRK3	BTS1	ELF1	ELOF1	BTS1	SKY1	SRPK1	BTS1	YOR1	ABCC9
HMG1	YAK1	HIPK1	BTS1	HMG1	HMGCR	BTS1	SKY1	SRPK2	BTS1	YOR1	CFTR

Table S4. Most of the top 20 drugs that share signature genes with atorvastatin identified have anticancer activity but have not been investigated for synergy with statins

Drug/ compound	Description	Approved	Intended/ approved use	Clinical trial(s)	Clinical trial(s) with statin
GW779439X	Pyrazolopyridazine with antibiotic synergistic properties	No	Antibiotic	No	No
Dinaciclib	UPR inhibitor via CDK1 and 5	No	Cancer	Yes	No
Docetaxel	Chemotherapeutic for breast cancer	Yes	Cancer	Yes	Yes
Lestaurtinib	Tyorine kinase inhibitor	Yes	Cancer	Yes	No
Vorinostat	Used to treat cutaneous T-cell lymphoma	Yes	Cancer	Yes	No
KW-2449	Multikinase inhibitor	No	Cancer	Yes	No
RO-31-8220	Protein kinase C inhibitor	No	Various	No	No
Palbociclib	Ibrance, inhibitor of the cyclin-dependent kinases CDK4 and 6	Yes	Cancer	Yes	No
AZD5438	Oral inhibitor of cyclin-dependent kinases 1, 2, and 9	No	Cancer	Yes	No
Probenecid	Probalan, increases uric acid excretion and inhibits drug renal excretion	Yes	Prevention of gout	Yes	Yes
GW5074	cRaf1 kinase inhibitor	No	Cancer, neurodegenerative disorders	Yes	No
CGP74514A	CDK1 inhibitor	No	Cancer	No	No
Sunitinib	Sutent, multi-targeted receptor tyrosine kinase (RTK) inhibitor	Yes	Cancer	Yes	No
Verlukast	selective inhibitor of leukotriene	No	Bronchodilator	No	No
JNK-9L	c-jun-N-terminal kinase (JNK) inhibitor	No	Cancer	No	No
Staurosporine	Protein kinases inhibitor	No	Cancer	Yes	No
PKR Inhibitor	C16, inhibitor of RNA-dependent protein kinase (PKR)	No	Cancer	No	No
Hesperetin	Cholesterol lowering flavanoid found in citrus juices	No	Lowering cholesterol, cancer, antioxidant, anti-inflammatory, vasoprotective	Yes	No
A-674563	AKT1 inhibitor that also suppresses CDK2 activity	No	Cancer	No	No
AS-59957	1H-Pyrrole-2,5-dione, 3,4-diphenyl	No	Various	No	No

Figure S2

Table S5. Strains used in this study

Background	Genotype	Description	Reference
Y7092 (S288C)	*Matα can1::STE2pr-Sp_his5 lyp1Δ his3Δ1 leu2Δ0 ura3Δ0 met15Δ0*	Query construction starting strain	Tong and Boone 2006
Y55	*Matα ho::HPH ura3Δ0 his3Δ0 [URA3_CEN]*	Query construction starting strain	Busby et al. 2019
UWOPS87	*Matα ho::HPH ura3Δ0 his3Δ0 [URA3_CEN]*	Query construction starting strain	Busby et al. 2019
Y7092 (S288C)	*Matα can1::STE2pr-Sp_his5 lyp1Δ his3Δ1 leu2Δ0 ura3Δ0 met15Δ0 hmg1::NatR*	*hmg1Δ* query strain	This study
Y55	*Matα ho::HPH ura3Δ0 his3Δ0 hmg1::NatR*	*hmg1Δ* query strain	This study
UWOPS87	*Matα ho::HPH ura3Δ0 his3Δ0 hmg1::NatR*	*hmg1Δ* query strain	This study
Y7092 (S288C)	*Matα can1::STE2pr-Sp_his5 lyp1Δ his3Δ1 leu2Δ0 ura3Δ0 met15Δ0 bts1::NatR*	*bts1Δ* query strain	This study
Y55	*Matα ho::HPH ura3Δ0 his3Δ0 bts1::NatR*	*bts1Δ* query strain	This study
UWOPS87	*Matα ho::HPH ura3Δ0 his3Δ0 bts1::NatR*	*bts1Δ* query strain	This study
Y7092 (S288C)	*MATa his3Δ1 leu2Δ0 met15Δ0 ura3Δ0 xxx::KanR*	Yeast deletion collection (DMA)	Tong and Boone 2006
Y55	*Mata can1::STE2pr-Sp_his5 lyp1Δ his3Δ1 ura3Δ0 xxx::KanR*	Yeast deletion collection (DMA)	Busby et al. 2019
UWOPS87	*Mata can1::STE2pr-Sp_his5 lyp1Δ his3Δ1 ura3Δ0 xxx::KanR*	Yeast deletion collection (DMA)	Busby et al. 2019
Y7092 (S288C)	*Mata can1::STE2pr-Sp_his5 lyp1Δ his3Δ1 leu2Δ0 ura3Δ0 met15Δ0 hmg1::NatR xxx::KanR*	*hmg1Δ xxxΔ* SGA	This study
Y55	*Mata can1::STE2pr-Sp_his5 lyp1Δ his3Δ1 ura3Δ0 ho::HPH hmg1::NatR xxx::KanR*	*hmg1Δ xxxΔ* SGA	This study
UWOPS87	*Mata can1::STE2pr-Sp_his5 lyp1Δ his3Δ1 ura3Δ0 ho::HPH hmg1::NatR xxx::KanR*	*hmg1Δ xxxΔ* SGA	This study
Y7092 (S288C)	*Mata can1::STE2pr-Sp_his5 lyp1Δ his3Δ1 leu2Δ0 ura3Δ0 met15Δ0 bts1::NatR xxx::KanR*	*bts1Δ xxxΔ* SGA	This study
Y55	*Mata can1::STE2pr-Sp_his5 lyp1Δ his3Δ1 ura3Δ0 ho::HPH bts1::NatR xxx::KanR*	*bts1Δ xxxΔ* SGA	This study
UWOPS87	*Mata can1::STE2pr-Sp_his5 lyp1Δ his3Δ1 ura3Δ0 ho::HPH bts1::NatR xxx::KanR*	*bts1Δ xxxΔ* SGA	This study

Table S6. PCR primers and conditions used in this study

Primer	Sequence	Description
hmg1Δ forward	ATAGTGTATCATTGTCTAATTGTTGATACAAAGTAGATAAATACA TAAAACAAGCACATGGAGGCCCAGAATACCCT	5' HMG1 loci KO with clonNAT resistance cassette
hmg1Δ reverse	ACATGGTGCTGTTGTGCTTCTTTTTCAAGAGAATACCAATGACGT ATGACTAAGTCAGTATAGAGCGACCAGCATTCAC	3' HMG1 loci KO with clonNAT resistance cassette
bts1Δ forward	TTCAAAGAAGCTACTAATAGAAAGAGAACAAAGCGTTTACGAG TCTGGAAAATCAACATGGAGGCCCAGAATACCCT	5' BTS1 loci KO with clonNAT resistance cassette
bts1Δ reverse	GAGAAGGCTTTATTTCTGACTATCTTCCTCCACTAATTTGATTGA TCAATTTATTCAGTATAGCGACCAGCATTCAC	3' BTS1 loci KO with clonNAT resistance cassette
hmg1Δ confirmation forward (A)	AGTCTCTACGCCCGCTCG	5' HMG1 loci KO confirmation A
hmg1Δ confirmation reverse (D)	CGCATGACTCAAGAGAAGC	3' HMG1 loci KO confirmation D
bts1Δ confirmation forward (A)	AGTCTCTACGCCCGCTCG	5' BTS1 loci KO confirmation A
bts1Δ confirmation reverse (D)	GGAGTTTCAGAAATCGTGG	3' BTS1 loci KO confirmation D
NAT confirmation reverse (B)	TACGAGATGACCACGAAGC	3' clonNAT resistance loci confirmation B
NAT confirmation forward (C)	TGGAACCGCCGGCTGACC	5' clonNAT resistance loci confirmation C

Component	Volume (µL)
ddH2O	17.625
10X Buffer	2.5
dNTPs	2
DMSO	1.25
Deletion primer Fwd	0.5
Deletion primer Rev	0.5
Taq	0.125
Template (plasmid)	0.5

PCR phase	Temperature (°C)	Time	# cycles
Initial denaturation	95	5 min	1
Denaturation	94	40 s	36
Annealing	58	1 min	36
Extension	68	2 min	36
Final extension	72	5 min	1

Table S7. Raw dataset for hmg1Δ xxxΔ screening with atorvastatin

Array	Array annotation	Plate id / file name	Raw avg. colony size	Raw avg. colony size std. dev.	Normalized colony size	Normalized colony size std. dev.	Score	Score std. dev.	p-Value
1	1	S288C_Ator08_hmg124h_4.JPG.dat	1221.3	75.7	1.1	0.01	-0.030	0.011	3.6E-05
2	2	S288C_Ator08_hmg124h_4.JPG.dat	1150.3	105.1	1.1	0.08	0.093	0.083	1.3E-04
3	3	S288C_Ator08_hmg124h_4.JPG.dat	1165.8	110.2	1.1	0.09	0.043	0.086	1.5E-04
4	4	S288C_Ator08_hmg124h_4.JPG.dat	1077.3	52.4	1.0	0.04	0.002	0.037	1.4E-05
YDR423C	YDR423C	S288C_Ator08_hmg124h_4.JPG.dat	994.8	57.3	1.0	0.06	-0.075	0.056	5.4E-05
YDR424C	YDR424C	S288C_Ator08_hmg124h_4.JPG.dat	1026.0	58.7	1.0	0.06	-0.022	0.058	5.6E-05
YDR425W	YDR425W	S288C_Ator08_hmg124h_4.JPG.dat	984.8	82.2	0.9	0.08	-0.044	0.077	1.6E-04
YDR426C	YDR426C	S288C_Ator08_hmg124h_4.JPG.dat	1026.5	49.0	1.0	0.05	0.024	0.048	3.4E-05
YDR428C	YDR428C	S288C_Ator08_hmg124h_4.JPG.dat	1114.8	6.1	1.0	0.00	0.002	0.004	1.6E-08
YDR430C	YDR430C	S288C_Ator08_hmg124h_4.JPG.dat	1090.0	13.3	1.0	0.00	0.005	0.004	5.7E-06
YDR431W	YDR431W	S288C_Ator08_hmg124h_4.JPG.dat	1101.0	52.4	1.0	0.01	-0.014	0.013	5.9E-05
YDR435C	YDR435C	S288C_Ator08_hmg124h_4.JPG.dat	1130.3	23.2	1.0	0.02	-0.010	0.023	3.2E-06
YDR436W	YDR436W	S288C_Ator08_hmg124h_4.JPG.dat	1120.8	42.8	1.0	0.03	-0.025	0.034	1.1E-05
YDR438W	YDR438W	S288C_Ator08_hmg124h_4.JPG.dat	1122.8	42.1	1.0	0.02	0.010	0.024	4.1E-06
YDR439W	YDR439W	S288C_Ator08_hmg124h_4.JPG.dat	1188.5	37.4	1.0	0.03	0.086	0.031	7.7E-06
YDR440W	YDR440W	S288C_Ator08_hmg124h_4.JPG.dat	1067.3	109.0	1.0	0.02	-0.078	0.022	1.6E-04
YDR441C	YDR441C	S288C_Ator08_hmg124h_4.JPG.dat	1120.8	18.4	1.0	0.00	0.040	0.002	8.2E-07
YDR445C	YDR445C	S288C_Ator08_hmg124h_4.JPG.dat	1125.5	37.9	1.0	0.03	0.051	0.029	6.0E-06
YDR446W	YDR446W	S288C_Ator08_hmg124h_4.JPG.dat	1107.5	23.1	1.0	0.02	0.009	0.020	2.2E-06
YDR447C	YDR447C	S288C_Ator08_hmg124h_4.JPG.dat	971.5	25.9	0.9	0.01	-0.012	0.007	1.9E-05
YDR451C	YDR451C	S288C_Ator08_hmg124h_4.JPG.dat	1020.5	99.2	1.0	0.01	-0.017	0.007	1.9E-05
YDR452W	YDR452W	S288C_Ator08_hmg124h_4.JPG.dat	1035.0	12.6	1.0	0.01	-0.027	0.012	4.8E-07
YDR453C	YDR453C	S288C_Ator08_hmg124h_4.JPG.dat	1080.0	13.0	1.0	0.01	0.001	0.014	6.8E-07
YDR455C	YDR455C	S288C_Ator08_hmg124h_4.JPG.dat	914.5	50.4	0.8	0.05	-0.025	0.046	4.6E-05
YDR458C	YDR458C	S288C_Ator08_hmg124h_4.JPG.dat	1107.5	22.6	1.0	0.01	-0.017	0.013	5.7E-07
YDR459C	YDR459C	S288C_Ator08_hmg124h_4.JPG.dat	1149.8	73.6	1.0	0.07	-0.014	0.065	6.8E-05
YDR463W	YDR463W	S288C_Ator08_hmg124h_4.JPG.dat	993.3	32.4	1.0	0.04	0.014	0.036	1.3E-05
YDR465C	YDR465C	S288C_Ator08_hmg124h_4.JPG.dat	1082.0	17.8	1.0	0.00	0.001	0.003	3.6E-06
YDR466W	YDR466W	S288C_Ator08_hmg124h_4.JPG.dat	1132.0	22.7	1.0	0.02	-0.009	0.023	2.8E-06
YDR467C	YDR467C	S288C_Ator08_hmg124h_4.JPG.dat	1065.8	27.4	1.0	0.02	-0.038	0.020	2.0E-06
YDR469W	YDR469W	S288C_Ator08_hmg124h_4.JPG.dat	1042.8	32.3	1.0	0.03	0.018	0.029	7.3E-06
YDR471W	YDR471W	S288C_Ator08_hmg124h_4.JPG.dat	1021.0	43.0	1.0	0.04	0.042	0.037	1.6E-05
YDR474C	YDR474C	S288C_Ator08_hmg124h_4.JPG.dat	1045.3	11.5	1.0	0.00	0.017	0.002	1.3E-06
YDR475C	YDR475C	S288C_Ator08_hmg124h_4.JPG.dat	1031.5	14.3	1.0	0.02	0.008	0.019	1.8E-06
YDR476C	YDR476C	S288C_Ator08_hmg124h_4.JPG.dat	1058.5	24.1	1.0	0.02	-0.003	0.020	2.2E-06
YDR479C	YDR479C	S288C_Ator08_hmg124h_4.JPG.dat	1047.0	9.6	1.0	0.01	-0.026	0.009	2.1E-07
YDR480W	YDR480W	S288C_Ator08_hmg124h_4.JPG.dat	1038.8	22.4	1.0	0.01	-0.070	0.009	2.0E-07
YDR481C	YDR481C	S288C_Ator08_hmg124h_4.JPG.dat	1065.8	25.2	1.0	0.01	-0.015	0.013	5.7E-07
YDR482C	YDR482C	S288C_Ator08_hmg124h_4.JPG.dat	1105.3	7.3	1.0	0.01	0.004	0.007	7.7E-08
YDR483W	YDR483W	S288C_Ator08_hmg124h_4.JPG.dat	1104.3	20.8	1.0	0.02	0.022	0.018	1.7E-06
YDR485C	YDR485C	S288C_Ator08_hmg124h_4.JPG.dat	917.8	17.6	0.8	0.02	-0.063	0.018	2.7E-06
YDR486C	YDR486C	S288C_Ator08_hmg124h_4.JPG.dat	1192.3	22.0	1.1	0.00	0.040	0.005	6.6E-06
YDR488C	YDR488C	S288C_Ator08_hmg124h_4.JPG.dat	1139.8	20.0	1.0	0.02	-0.009	0.018	1.6E-06
YDR490C	YDR490C	S288C_Ator08_hmg124h_4.JPG.dat	1039.0	28.6	1.0	0.02	-0.045	0.025	4.7E-06
YDR491C	YDR491C	S288C_Ator08_hmg124h_4.JPG.dat	1118.5	27.0	1.1	0.03	0.027	0.029	5.5E-06
YDR492W	YDR492W	S288C_Ator08_hmg124h_4.JPG.dat	1098.8	23.5	1.0	0.02	-0.054	0.021	2.2E-06
YDR494W	YDR494W	S288C_Ator08_hmg124h_4.JPG.dat	1114.5	30.9	1.0	0.03	0.040	0.031	7.3E-06
YDR496C	YDR496C	S288C_Ator08_hmg124h_4.JPG.dat	1045.3	50.5	1.0	0.04	0.094	0.037	1.5E-05
YDR497C	YDR497C	S288C_Ator08_hmg124h_4.JPG.dat	1119.8	31.0	1.0	0.00	-0.001	0.004	5.6E-06
YDR500C	YDR500C	S288C_Ator08_hmg124h_4.JPG.dat	1045.0	76.6	0.9	0.02	0.077	0.024	2.3E-04
YDR501W	YDR501W	S288C_Ator08_hmg124h_4.JPG.dat	1110.0	16.8	1.0	0.02	0.003	0.016	1.2E-06
YDR503C	YDR503C	S288C_Ator08_hmg124h_4.JPG.dat	1127.3	15.6	1.0	0.00	0.010	0.002	1.1E-06
YDR504C	YDR504C	S288C_Ator08_hmg124h_4.JPG.dat	1133.3	10.7	1.0	0.01	0.002	0.010	2.3E-07
YDR505C	YDR505C	S288C_Ator08_hmg124h_4.JPG.dat	1125.0	20.0	1.0	0.00	-0.013	0.005	8.3E-06
YDR506C	YDR506C	S288C_Ator08_hmg124h_4.JPG.dat	1014.8	91.4	0.9	0.08	-0.055	0.085	2.0E-04
YDR508C	YDR508C	S288C_Ator08_hmg124h_4.JPG.dat	1036.5	17.6	1.0	0.02	0.025	0.015	1.0E-06
YDR509W	YDR509W	S288C_Ator08_hmg124h_4.JPG.dat	960.0	33.1	0.9	0.01	-0.040	0.005	1.1E-05
YDR511W	YDR511W	S288C_Ator08_hmg124h_4.JPG.dat	1059.5	18.9	1.0	0.00	-0.003	0.004	4.9E-06
YDR512C	YDR512C	S288C_Ator08_hmg124h_4.JPG.dat	1063.5	107.5	1.0	0.01	0.089	0.009	2.3E-05
YDR513W	YDR513W	S288C_Ator08_hmg124h_4.JPG.dat	1065.3	18.7	1.0	0.01	0.050	0.011	3.6E-05
YDR514C	YDR514C	S288C_Ator08_hmg124h_4.JPG.dat	1120.5	15.2	1.0	0.02	0.030	0.015	8.7E-07
YDR516C	YDR516C	S288C_Ator08_hmg124h_4.JPG.dat	1123.3	19.5	1.0	0.02	-0.002	0.020	2.2E-06
YDR517W	YDR517W	S288C_Ator08_hmg124h_4.JPG.dat	1109.5	14.5	1.0	0.01	-0.020	0.011	3.8E-07
YDR519W	YDR519W	S288C_Ator08_hmg124h_4.JPG.dat	1142.3	36.9	1.0	0.03	0.026	0.031	7.7E-06
YDR520C	YDR520C	S288C_Ator08_hmg124h_4.JPG.dat	1114.8	23.5	1.0	0.01	0.002	0.008	1.9E-05

YDR522C	YDR522C	S288C_Ator08_hmg124h_4.JPG.dat	1067.0	83.5	1.0	0.08	-0.012	0.081	1.6E-04
YDR524C	YDR524C	S288C_Ator08_hmg124h_4.JPG.dat	1040.5	16.0	1.0	0.01	-0.044	0.006	7.8E-08
YDR525W	YDR525W	S288C_Ator08_hmg124h_4.JPG.dat	1041.5	4.0	1.0	0.01	-0.024	0.006	7.7E-08
YDR525W-	YDR525W-A	S288C_Ator08_hmg124h_4.JPG.dat	969.0	11.0	0.9	0.01	-0.028	0.011	5.6E-07
YDR528W	YDR528W	S288C_Ator08_hmg124h_4.JPG.dat	1127.5	29.0	1.1	0.06	0.059	0.061	4.4E-05
YDR530C	YDR530C	S288C_Ator08_hmg124h_4.JPG.dat	934.0	73.7	0.9	0.08	-0.141	0.078	2.0E-04
YDR532C	YDR532C	S288C_Ator08_hmg124h_4.JPG.dat	1059.5	49.5	1.0	0.03	-0.042	0.033	1.1E-05
YDR533C	YDR533C	S288C_Ator08_hmg124h_4.JPG.dat	1127.5	32.8	1.0	0.02	0.024	0.021	2.4E-06
YDR534C	YDR534C	S288C_Ator08_hmg124h_4.JPG.dat	1141.8	65.1	1.0	0.03	0.014	0.034	9.7E-06
YDR535C	YDR535C	S288C_Ator08_hmg124h_4.JPG.dat	1106.0	51.0	1.0	0.02	-0.022	0.021	2.5E-06
YDR536W	YDR536W	S288C_Ator08_hmg124h_4.JPG.dat	1113.8	34.5	1.0	0.01	0.004	0.012	4.2E-07
YDR537C	YDR537C	S288C_Ator08_hmg124h_4.JPG.dat	1120.8	31.5	1.0	0.03	0.073	0.027	5.1E-06
YDR538W	YDR538W	S288C_Ator08_hmg124h_4.JPG.dat	1106.8	25.1	1.0	0.01	0.042	0.006	1.4E-05
YDR539W	YDR539W	S288C_Ator08_hmg124h_4.JPG.dat	1086.5	30.7	1.0	0.00	-0.029	0.004	6.1E-06
YDR540C	YDR540C	S288C_Ator08_hmg124h_4.JPG.dat	1108.5	24.7	1.1	0.06	0.033	0.062	5.4E-05
YDR541C	YDR541C	S288C_Ator08_hmg124h_4.JPG.dat	509.8	603.4	0.6	0.74	0.055	0.737	1.8E-01
YEL001C	YEL001C	S288C_Ator08_hmg124h_4.JPG.dat	985.8	79.1	1.0	0.08	0.025	0.082	1.5E-04
YEL003W	YEL003W	S288C_Ator08_hmg124h_4.JPG.dat	1022.8	45.7	1.0	0.01	0.001	0.014	7.1E-05
YEL004W	YEL004W	S288C_Ator08_hmg124h_4.JPG.dat	1100.0	78.5	1.0	0.08	0.091	0.075	1.0E-04
YEL005C	YEL005C	S288C_Ator08_hmg124h_4.JPG.dat	1045.3	20.9	1.0	0.02	-0.010	0.020	2.3E-06
YEL006W	YEL006W	S288C_Ator08_hmg124h_4.JPG.dat	1054.8	11.6	1.0	0.02	-0.040	0.015	1.0E-06
YEL007W	YEL007W	S288C_Ator08_hmg124h_4.JPG.dat	1138.0	48.1	1.0	0.05	0.055	0.045	2.3E-05
YEL008W	YEL008W	S288C_Ator08_hmg124h_4.JPG.dat	1112.3	6.6	1.0	0.00	0.015	0.002	1.6E-06
YEL010W	YEL010W	S288C_Ator08_hmg124h_4.JPG.dat	1139.0	43.9	1.0	0.04	0.019	0.039	1.5E-05
YEL011W	YEL011W	S288C_Ator08_hmg124h_4.JPG.dat	1073.3	52.4	1.0	0.04	-0.047	0.043	2.3E-05
YEL012W	YEL012W	S288C_Ator08_hmg124h_4.JPG.dat	1078.5	7.4	1.0	0.01	-0.032	0.007	1.3E-07
YEL013W	YEL013W	S288C_Ator08_hmg124h_4.JPG.dat	812.3	133.3	0.7	0.12	0.063	0.119	1.4E-03
YEL014C	YEL014C	S288C_Ator08_hmg124h_4.JPG.dat	950.0	21.3	1.0	0.00	0.110	0.004	6.9E-06
YEL015W	YEL015W	S288C_Ator08_hmg124h_4.JPG.dat	1071.5	40.6	1.0	0.04	-0.009	0.037	1.4E-05
YEL016C	YEL016C	S288C_Ator08_hmg124h_4.JPG.dat	1104.5	35.7	1.0	0.01	-0.027	0.007	1.7E-05
YEL017C-A	YEL017C-A	S288C_Ator08_hmg124h_4.JPG.dat	1107.0	14.2	1.0	0.02	0.018	0.019	1.8E-06
YEL017W	YEL017W	S288C_Ator08_hmg124h_4.JPG.dat	1088.5	7.4	1.0	0.01	-0.004	0.013	6.5E-07
YEL020C	YEL020C	S288C_Ator08_hmg124h_4.JPG.dat	1099.3	50.4	1.0	0.02	-0.022	0.017	1.1E-04
YEL023C	YEL023C	S288C_Ator08_hmg124h_4.JPG.dat	1063.0	29.8	1.0	0.03	-0.045	0.028	6.3E-06
YEL025C	YEL025C	S288C_Ator08_hmg124h_4.JPG.dat	1037.8	83.2	1.0	0.01	0.041	0.012	4.8E-05
YEL028W	YEL028W	S288C_Ator08_hmg124h_4.JPG.dat	1121.5	58.0	1.0	0.05	0.010	0.053	3.6E-05
YEL030W	YEL030W	S288C_Ator08_hmg124h_4.JPG.dat	1073.0	24.9	1.0	0.02	-0.013	0.022	3.2E-06
YEL031W	YEL031W	S288C_Ator08_hmg124h_4.JPG.dat	5.5	11.0	0.0	0.00	0.000	0.000	
YEL033W	YEL033W	S288C_Ator08_hmg124h_4.JPG.dat	972.3	78.8	1.0	0.07	0.084	0.074	1.0E-04
YEL037C	YEL037C	S288C_Ator08_hmg124h_4.JPG.dat	1146.0	16.8	1.1	0.01	0.092	0.005	8.3E-06
YEL038W	YEL038W	S288C_Ator08_hmg124h_4.JPG.dat	1091.5	23.0	1.0	0.01	0.020	0.007	1.4E-05
YEL039C	YEL039C	S288C_Ator08_hmg124h_4.JPG.dat	1111.0	14.6	1.0	0.01	0.010	0.014	7.6E-07
YEL040W	YEL040W	S288C_Ator08_hmg124h_4.JPG.dat	1067.5	19.6	1.0	0.02	0.000	0.016	1.1E-06
YEL041W	YEL041W	S288C_Ator08_hmg124h_4.JPG.dat	1054.3	31.6	1.0	0.03	-0.001	0.027	5.7E-06
YEL042W	YEL042W	S288C_Ator08_hmg124h_4.JPG.dat	841.5	36.1	0.8	0.01	-0.144	0.010	5.6E-05
YEL043W	YEL043W	S288C_Ator08_hmg124h_4.JPG.dat	969.3	69.5	0.9	0.06	-0.020	0.065	9.1E-05
YEL047C	YEL047C	S288C_Ator08_hmg124h_4.JPG.dat	1034.3	25.7	1.0	0.03	-0.023	0.031	8.3E-06
YEL048C	YEL048C	S288C_Ator08_hmg124h_4.JPG.dat	1005.8	26.3	1.0	0.03	-0.035	0.025	5.0E-06
YEL049W	YEL049W	S288C_Ator08_hmg124h_4.JPG.dat	984.8	42.4	0.9	0.04	-0.017	0.041	2.4E-05
YEL052W	YEL052W	S288C_Ator08_hmg124h_4.JPG.dat	1032.0	56.7	1.0	0.05	0.034	0.046	2.3E-05
YEL053C	YEL053C	S288C_Ator08_hmg124h_4.JPG.dat	1019.8	29.9	0.9	0.00	-0.074	0.005	8.8E-06
YEL056W	YEL056W	S288C_Ator08_hmg124h_4.JPG.dat	1115.3	20.3	1.0	0.00	0.012	0.004	5.5E-06
YEL057C	YEL057C	S288C_Ator08_hmg124h_4.JPG.dat	816.3	545.6	1.0	0.04	-0.001	0.045	7.0E-04
YEL059W	YEL059W	S288C_Ator08_hmg124h_4.JPG.dat	706.8	471.2	0.9	0.01	-0.065	0.007	2.0E-05
YEL060C	YEL060C	S288C_Ator08_hmg124h_4.JPG.dat	300.8	601.5	0.0	0.00	0.000	0.000	
YEL061C	YEL061C	S288C_Ator08_hmg124h_4.JPG.dat	284.3	568.5	0.0	0.00	0.000	0.000	
YEL062W	YEL062W	S288C_Ator08_hmg124h_4.JPG.dat	424.3	520.8	0.4	0.48	-0.029	0.484	2.0E-01
YEL063C	YEL063C	S288C_Ator08_hmg124h_4.JPG.dat	0.0	0.0	0.0	0.00	0.000	0.000	
YEL064C	YEL064C	S288C_Ator08_hmg124h_4.JPG.dat	0.0	0.0	0.0	0.00	0.000	0.000	
YEL065W	YEL065W	S288C_Ator08_hmg124h_4.JPG.dat	835.8	557.2	1.0	0.01	-0.068	0.005	9.6E-06
YEL066W	YEL066W	S288C_Ator08_hmg124h_4.JPG.dat	1015.0	32.0	1.0	0.05	0.033	0.054	4.1E-05
YEL067C	YEL067C	S288C_Ator08_hmg124h_4.JPG.dat	1135.8	24.4	1.0	0.02	-0.032	0.017	1.3E-06
YEL068C	YEL068C	S288C_Ator08_hmg124h_4.JPG.dat	1099.8	24.5	1.0	0.02	-0.005	0.021	2.7E-06
YEL071W	YEL071W	S288C_Ator08_hmg124h_4.JPG.dat	1104.8	11.0	1.0	0.01	-0.021	0.011	3.6E-07
YER001W	YER001W	S288C_Ator08_hmg124h_4.JPG.dat	1111.5	19.6	1.0	0.02	0.013	0.023	3.1E-06
YER002W	YER002W	S288C_Ator08_hmg124h_4.JPG.dat	1167.8	22.6	1.0	0.00	0.035	0.003	3.6E-06
YER004W	YER004W	S288C_Ator08_hmg124h_4.JPG.dat	1097.8	19.4	1.0	0.02	-0.005	0.020	2.3E-06
YER005W	YER005W	S288C_Ator08_hmg124h_4.JPG.dat	1131.0	10.5	1.1	0.01	-0.004	0.010	2.5E-07
YER007C-A	YER007C-A	S288C_Ator08_hmg124h_4.JPG.dat	999.0	15.1	1.0	0.02	-0.028	0.016	1.2E-06
YER007W	YER007W	S288C_Ator08_hmg124h_4.JPG.dat	1059.3	22.0	1.0	0.02	-0.017	0.019	1.9E-06

YER010C	YER010C	S288C_Ator08_hmg124h_4.JPG.dat	1111.3	17.2	1.0	0.02	0.027	0.016	1.1E-06
YER011W	YER011W	S288C_Ator08_hmg124h_4.JPG.dat	1036.5	42.4	1.0	0.05	0.030	0.053	3.6E-05
YER016W	YER016W	S288C_Ator08_hmg124h_4.JPG.dat	1096.5	12.7	1.0	0.01	0.070	0.013	5.9E-07
YER019C-A	YER019C-A	S288C_Ator08_hmg124h_4.JPG.dat	1112.5	79.7	1.0	0.02	-0.018	0.021	1.5E-04
YER019W	YER019W	S288C_Ator08_hmg124h_4.JPG.dat	780.8	190.2	0.7	0.17	-0.076	0.172	3.8E-03
YER020W	YER020W	S288C_Ator08_hmg124h_4.JPG.dat	1098.0	84.8	1.0	0.01	0.095	0.011	4.0E-05
YER024W	YER024W	S288C_Ator08_hmg124h_4.JPG.dat	1147.5	20.4	1.0	0.02	0.010	0.019	2.0E-06
YER027C	YER027C	S288C_Ator08_hmg124h_4.JPG.dat	1123.8	11.0	1.0	0.00	-0.021	0.002	1.2E-06
YER028C	YER028C	S288C_Ator08_hmg124h_4.JPG.dat	1100.8	38.4	1.0	0.03	-0.013	0.033	9.5E-06
YER030W	YER030W	S288C_Ator08_hmg124h_4.JPG.dat	1018.8	25.6	1.0	0.02	-0.032	0.021	2.9E-06
YER031C	YER031C	S288C_Ator08_hmg124h_4.JPG.dat	904.8	22.1	0.9	0.02	-0.177	0.022	4.8E-06
YER032W	YER032W	S288C_Ator08_hmg124h_4.JPG.dat	1102.8	14.7	1.0	0.02	-0.010	0.018	1.5E-06
YER033C	YER033C	S288C_Ator08_hmg124h_4.JPG.dat	905.5	42.5	0.9	0.03	-0.153	0.035	1.7E-05
YER034W	YER034W	S288C_Ator08_hmg124h_4.JPG.dat	1095.3	40.5	1.0	0.02	-0.017	0.024	4.0E-06
YER035W	YER035W	S288C_Ator08_hmg124h_4.JPG.dat	1030.3	106.5	0.9	0.10	-0.042	0.099	3.1E-04
YER038W-A	YER038W-A	S288C_Ator08_hmg124h_4.JPG.dat	1090.3	38.0	1.0	0.03	-0.022	0.034	1.1E-05
YER039C	YER039C	S288C_Ator08_hmg124h_4.JPG.dat	1123.0	24.1	1.0	0.01	0.016	0.006	1.0E-05
YER039C-A	YER039C-A	S288C_Ator08_hmg124h_4.JPG.dat	1136.8	34.6	1.0	0.03	0.037	0.028	5.6E-06
YER041W	YER041W	S288C_Ator08_hmg124h_4.JPG.dat	1075.8	106.1	1.0	0.07	-0.084	0.070	1.1E-04
YER042W	YER042W	S288C_Ator08_hmg124h_4.JPG.dat	1089.5	44.1	1.0	0.01	0.008	0.007	1.9E-05
YER044C-A	YER044C-A	S288C_Ator08_hmg124h_4.JPG.dat	1099.5	51.9	1.0	0.04	0.014	0.043	2.2E-05
YER045C	YER045C	S288C_Ator08_hmg124h_4.JPG.dat	1078.3	56.7	1.0	0.07	-0.058	0.067	8.6E-05
YER046W	YER046W	S288C_Ator08_hmg124h_4.JPG.dat	1125.5	22.8	1.2	0.10	0.050	0.095	1.3E-04
YER046W-A	YER046W-A	S288C_Ator08_hmg124h_4.JPG.dat	967.8	17.8	1.0	0.02	-0.034	0.018	1.8E-06
YER047C	YER047C	S288C_Ator08_hmg124h_4.JPG.dat	900.5	27.9	0.9	0.03	-0.081	0.026	6.4E-06
YER048C	YER048C	S288C_Ator08_hmg124h_4.JPG.dat	961.0	41.6	1.0	0.01	-0.056	0.011	4.0E-05
YER049W	YER049W	S288C_Ator08_hmg124h_4.JPG.dat	923.0	61.8	0.9	0.06	-0.074	0.059	7.5E-05
YER051W	YER051W	S288C_Ator08_hmg124h_4.JPG.dat	1127.8	39.3	1.1	0.04	0.070	0.035	9.2E-06
YER052C	YER052C	S288C_Ator08_hmg124h_4.JPG.dat	1164.3	94.4	1.1	0.09	0.119	0.085	1.2E-04
YER053C	YER053C	S288C_Ator08_hmg124h_4.JPG.dat	1066.3	9.0	1.0	0.01	-0.010	0.008	1.3E-07
YER054C	YER054C	S288C_Ator08_hmg124h_4.JPG.dat	1184.5	45.2	1.1	0.04	0.075	0.042	1.4E-05
YER055C	YER055C	S288C_Ator08_hmg124h_4.JPG.dat	294.8	589.5	0.0	0.00	0.000	0.000	
YER056C	YER056C	S288C_Ator08_hmg124h_4.JPG.dat	1102.3	68.8	1.0	0.05	-0.016	0.053	4.3E-05
YER056C-A	YER056C-A	S288C_Ator08_hmg124h_4.JPG.dat	1142.3	45.8	1.0	0.04	0.134	0.044	2.3E-05
YER057C	YER057C	S288C_Ator08_hmg124h_4.JPG.dat	1007.8	50.8	1.0	0.05	0.009	0.046	2.7E-05
YER059W	YER059W	S288C_Ator08_hmg124h_4.JPG.dat	1055.0	39.2	1.0	0.04	-0.003	0.038	1.5E-05
YER060W	YER060W	S288C_Ator08_hmg124h_4.JPG.dat	1084.8	31.1	1.0	0.03	0.004	0.032	8.4E-06
YER060W-A	YER060W-A	S288C_Ator08_hmg124h_4.JPG.dat	1107.0	34.2	1.0	0.03	0.053	0.029	5.6E-06
YER061C	YER061C	S288C_Ator08_hmg124h_4.JPG.dat	1075.3	14.5	1.0	0.01	0.179	0.008	1.7E-07
YER062C	YER062C	S288C_Ator08_hmg124h_4.JPG.dat	1099.3	16.3	1.0	0.02	0.001	0.023	3.0E-06
YER063W	YER063W	S288C_Ator08_hmg124h_4.JPG.dat	1076.3	13.9	1.0	0.01	-0.033	0.009	1.8E-07
YER064C	YER064C	S288C_Ator08_hmg124h_4.JPG.dat	1113.0	14.0	1.0	0.01	0.031	0.012	4.5E-07
YER065C	YER065C	S288C_Ator08_hmg124h_4.JPG.dat	1080.5	17.0	1.0	0.02	-0.016	0.024	3.9E-06
YER066C-A	YER066C-A	S288C_Ator08_hmg124h_4.JPG.dat	1037.5	34.2	1.0	0.01	-0.002	0.012	5.1E-05
YER066W	YER066W	S288C_Ator08_hmg124h_4.JPG.dat	1107.8	82.9	1.0	0.06	-0.001	0.064	7.3E-05
YER067C-A	YER067C-A	S288C_Ator08_hmg124h_4.JPG.dat	1038.3	43.7	1.0	0.05	0.032	0.052	3.5E-05
YER067W	YER067W	S288C_Ator08_hmg124h_4.JPG.dat	1076.8	11.6	1.0	0.01	-0.019	0.006	5.9E-08
YER069W	YER069W	S288C_Ator08_hmg124h_4.JPG.dat	251.3	502.5	0.0	0.00	0.000	0.000	
YER071C	YER071C	S288C_Ator08_hmg124h_4.JPG.dat	1089.0	23.2	1.0	0.00	0.032	0.004	4.0E-06
YER072W	YER072W	S288C_Ator08_hmg124h_4.JPG.dat	1097.5	26.0	1.0	0.02	0.015	0.023	3.1E-06
YER073W	YER073W	S288C_Ator08_hmg124h_4.JPG.dat	1071.3	15.9	1.0	0.02	0.044	0.018	1.6E-06
YER074W	YER074W	S288C_Ator08_hmg124h_4.JPG.dat	1031.5	21.9	1.0	0.02	-0.022	0.021	2.7E-06
YER075C	YER075C	S288C_Ator08_hmg124h_4.JPG.dat	1028.0	21.4	1.0	0.02	-0.006	0.022	3.2E-06
YER077C	YER077C	S288C_Ator08_hmg124h_4.JPG.dat	1063.8	18.8	1.0	0.02	0.056	0.018	1.6E-06
YER078C	YER078C	S288C_Ator08_hmg124h_4.JPG.dat	1066.5	7.4	1.0	0.01	0.080	0.014	8.3E-07
YER079W	YER079W	S288C_Ator08_hmg124h_4.JPG.dat	1094.3	32.6	1.0	0.02	-0.001	0.024	3.6E-06
YER080W	YER080W	S288C_Ator08_hmg124h_4.JPG.dat	1047.3	31.0	1.0	0.02	-0.024	0.024	3.7E-06
YER081W	YER081W	S288C_Ator08_hmg124h_4.JPG.dat	1115.3	20.1	1.0	0.01	-0.003	0.007	1.6E-05
YER083C	YER083C	S288C_Ator08_hmg124h_4.JPG.dat	423.8	236.9	0.4	0.21	-0.135	0.214	3.7E-02
YER084W	YER084W	S288C_Ator08_hmg124h_4.JPG.dat	1119.0	23.8	1.0	0.02	0.016	0.020	1.9E-06
YER085C	YER085C	S288C_Ator08_hmg124h_4.JPG.dat	1105.0	20.6	1.0	0.00	-0.013	0.005	6.9E-06
YER087C-A	YER087C-A	S288C_Ator08_hmg124h_4.JPG.dat	1095.5	21.3	1.0	0.02	-0.020	0.016	1.1E-06
YER088C	YER088C	S288C_Ator08_hmg124h_4.JPG.dat	1106.3	95.1	1.0	0.09	0.008	0.092	1.9E-04
YER090W	YER090W	S288C_Ator08_hmg124h_4.JPG.dat	864.3	41.3	0.8	0.04	-0.131	0.040	3.2E-05
YER091C	YER091C	S288C_Ator08_hmg124h_4.JPG.dat	1063.3	16.4	1.0	0.01	0.026	0.014	8.0E-07
YER091C-A	YER091C-A	S288C_Ator08_hmg124h_4.JPG.dat	1091.0	16.5	1.0	0.02	0.004	0.022	2.6E-06
YER092W	YER092W	S288C_Ator08_hmg124h_4.JPG.dat	968.0	24.7	0.9	0.02	0.148	0.020	2.8E-06
YER093C-A	YER093C-A	S288C_Ator08_hmg124h_4.JPG.dat	1117.8	40.2	1.1	0.00	0.084	0.004	3.6E-06
YER095W	YER095W	S288C_Ator08_hmg124h_4.JPG.dat	958.8	31.8	0.9	0.03	0.061	0.029	9.6E-06
YER096W	YER096W	S288C_Ator08_hmg124h_4.JPG.dat	1105.3	20.3	1.0	0.00	0.023	0.004	6.4E-06

YER097W	YER097W	S288C_Ator08_hmg124h_4.JPG.dat	1099.3	8.9	1.0	0.01	0.027	0.007	8.2E-08
YER098W	YER098W	S288C_Ator08_hmg124h_4.JPG.dat	1056.0	47.1	0.9	0.01	-0.050	0.014	7.1E-05
YER101C	YER101C	S288C_Ator08_hmg124h_4.JPG.dat	1102.3	59.4	1.0	0.05	-0.022	0.052	3.8E-05
YER106W	YER106W	S288C_Ator08_hmg124h_4.JPG.dat	1088.0	13.4	1.0	0.01	-0.002	0.013	6.3E-07
YER108C	YER108C	S288C_Ator08_hmg124h_4.JPG.dat	1012.3	39.4	1.0	0.00	-0.020	0.003	3.2E-06
YER109C	YER109C	S288C_Ator08_hmg124h_4.JPG.dat	1054.5	33.9	1.0	0.03	-0.002	0.034	1.1E-05
YER111C	YER111C	S288C_Ator08_hmg124h_4.JPG.dat	1051.3	49.0	1.0	0.04	-0.010	0.043	2.2E-05
YER113C	YER113C	S288C_Ator08_hmg124h_4.JPG.dat	1080.3	51.2	1.0	0.04	0.036	0.044	2.2E-05
YER114C	YER114C	S288C_Ator08_hmg124h_4.JPG.dat	1129.5	23.0	1.1	0.00	0.041	0.005	6.1E-06
YER115C	YER115C	S288C_Ator08_hmg124h_4.JPG.dat	1113.3	23.7	1.0	0.02	0.009	0.023	3.0E-06
YER116C	YER116C	S288C_Ator08_hmg124h_4.JPG.dat	985.3	11.6	0.9	0.01	0.072	0.006	8.2E-08
YER117W	YER117W	S288C_Ator08_hmg124h_4.JPG.dat	1014.0	76.3	0.9	0.06	-0.058	0.064	9.2E-05
YER118C	YER118C	S288C_Ator08_hmg124h_4.JPG.dat	1102.8	35.0	1.0	0.00	0.031	0.004	5.3E-06
YER119C	YER119C	S288C_Ator08_hmg124h_4.JPG.dat	1097.0	13.5	1.0	0.01	-0.013	0.010	3.2E-07
YER119C-A	YER119C-A	S288C_Ator08_hmg124h_4.JPG.dat	1111.8	30.3	1.0	0.02	0.004	0.024	3.4E-06
YER120W	YER120W	S288C_Ator08_hmg124h_4.JPG.dat	1013.0	70.2	1.0	0.06	-0.029	0.059	6.5E-05
YER121W	YER121W	S288C_Ator08_hmg124h_4.JPG.dat	1040.8	25.6	1.0	0.03	0.003	0.025	4.5E-06
YER123W	YER123W	S288C_Ator08_hmg124h_4.JPG.dat	989.3	39.7	0.9	0.01	0.043	0.013	7.2E-05
YER124C	YER124C	S288C_Ator08_hmg124h_4.JPG.dat	1080.5	13.3	1.0	0.01	-0.009	0.014	6.7E-07
YER128W	YER128W	S288C_Ator08_hmg124h_4.JPG.dat	1068.5	15.9	1.0	0.01	0.017	0.010	2.4E-07
YER129W	YER129W	S288C_Ator08_hmg124h_4.JPG.dat	1079.8	17.2	1.0	0.02	0.016	0.016	1.1E-06
YER130C	YER130C	S288C_Ator08_hmg124h_4.JPG.dat	1131.0	31.9	1.0	0.03	0.003	0.030	7.0E-06
YER131W	YER131W	S288C_Ator08_hmg124h_4.JPG.dat	1026.3	11.2	0.9	0.01	0.040	0.011	4.8E-07
YER132C	YER132C	S288C_Ator08_hmg124h_4.JPG.dat	1125.0	26.7	1.0	0.01	0.018	0.009	2.9E-05
YER134C	YER134C	S288C_Ator08_hmg124h_4.JPG.dat	1044.8	82.2	0.9	0.07	-0.073	0.072	1.3E-04
YER135C	YER135C	S288C_Ator08_hmg124h_4.JPG.dat	1148.0	9.8	1.0	0.01	0.033	0.009	2.1E-07
YER137C	YER137C	S288C_Ator08_hmg124h_4.JPG.dat	1081.0	42.3	1.0	0.01	-0.024	0.007	1.6E-05
YER139C	YER139C	S288C_Ator08_hmg124h_4.JPG.dat	1054.0	29.6	1.0	0.02	0.030	0.019	2.1E-06
YER140W	YER140W	S288C_Ator08_hmg124h_4.JPG.dat	1059.0	14.9	1.0	0.00	-0.036	0.001	1.6E-07
YER142C	YER142C	S288C_Ator08_hmg124h_4.JPG.dat	1090.8	31.9	1.2	0.07	-0.051	0.071	5.4E-05
YER143W	YER143W	S288C_Ator08_hmg124h_4.JPG.dat	886.8	45.2	0.9	0.05	-0.035	0.053	5.2E-05
YER144C	YER144C	S288C_Ator08_hmg124h_4.JPG.dat	898.3	60.0	0.9	0.06	-0.112	0.060	8.4E-05
YER145C	YER145C	S288C_Ator08_hmg124h_4.JPG.dat	913.3	33.7	0.9	0.01	-0.062	0.007	1.8E-05
YER149C	YER149C	S288C_Ator08_hmg124h_4.JPG.dat	1051.0	37.5	1.0	0.03	0.015	0.033	8.6E-06
YER150W	YER150W	S288C_Ator08_hmg124h_4.JPG.dat	1082.5	63.0	1.1	0.05	0.030	0.051	3.0E-05
YER151C	YER151C	S288C_Ator08_hmg124h_4.JPG.dat	818.8	67.1	0.8	0.06	-0.038	0.061	1.3E-04
YER152C	YER152C	S288C_Ator08_hmg124h_4.JPG.dat	1066.5	44.4	1.0	0.04	-0.011	0.039	1.5E-05
YER153C	YER153C	S288C_Ator08_hmg124h_4.JPG.dat	678.8	396.4	0.8	0.07	0.168	0.070	2.3E-03
YER155C	YER155C	S288C_Ator08_hmg124h_4.JPG.dat	812.5	49.0	0.8	0.05	-0.207	0.047	6.5E-05
YER156C	YER156C	S288C_Ator08_hmg124h_4.JPG.dat	1153.3	43.9	1.1	0.04	0.136	0.044	2.0E-05
YER158C	YER158C	S288C_Ator08_hmg124h_4.JPG.dat	1182.8	25.5	1.0	0.01	0.019	0.005	9.4E-06
YER161C	YER161C	S288C_Ator08_hmg124h_4.JPG.dat	820.0	62.8	0.9	0.02	0.044	0.023	2.2E-04
YER162C	YER162C	S288C_Ator08_hmg124h_4.JPG.dat	860.8	584.7	1.1	0.14	0.027	0.142	5.4E-03
YER163C	YER163C	S288C_Ator08_hmg124h_4.JPG.dat	1063.0	29.0	1.0	0.03	-0.004	0.026	4.9E-06
YER164W	YER164W	S288C_Ator08_hmg124h_4.JPG.dat	952.3	28.0	0.9	0.03	-0.027	0.028	8.9E-06
YER166W	YER166W	S288C_Ator08_hmg124h_4.JPG.dat	1060.0	16.1	1.0	0.02	-0.046	0.019	1.8E-06
YER167W	YER167W	S288C_Ator08_hmg124h_4.JPG.dat	1095.5	68.9	1.0	0.02	0.070	0.017	9.4E-05
YER170W	YER170W	S288C_Ator08_hmg124h_4.JPG.dat	1067.0	65.3	1.0	0.06	-0.016	0.059	5.9E-05
YER173W	YER173W	S288C_Ator08_hmg124h_4.JPG.dat	1081.8	23.7	1.0	0.02	-0.004	0.024	3.8E-06
YER174C	YER174C	S288C_Ator08_hmg124h_4.JPG.dat	1133.3	14.1	1.1	0.01	0.033	0.009	1.6E-07
YER175C	YER175C	S288C_Ator08_hmg124h_4.JPG.dat	1081.0	20.8	1.0	0.01	-0.024	0.007	1.1E-07
YER176W	YER176W	S288C_Ator08_hmg124h_4.JPG.dat	1081.3	84.2	1.0	0.07	-0.066	0.068	1.0E-04
YER177W	YER177W	S288C_Ator08_hmg124h_4.JPG.dat	840.8	61.6	0.9	0.04	0.081	0.044	3.4E-05
YER178W	YER178W	S288C_Ator08_hmg124h_4.JPG.dat	1003.0	61.3	1.0	0.05	0.018	0.055	5.2E-05
YER179W	YER179W	S288C_Ator08_hmg124h_4.JPG.dat	1095.8	53.5	1.0	0.05	-0.019	0.048	2.8E-05
YER180C	YER180C	S288C_Ator08_hmg124h_4.JPG.dat	1105.8	19.3	1.0	0.02	-0.025	0.018	1.6E-06
YER181C	YER181C	S288C_Ator08_hmg124h_4.JPG.dat	1034.3	49.6	1.0	0.04	-0.049	0.042	2.2E-05
YER182W	YER182W	S288C_Ator08_hmg124h_4.JPG.dat	1051.8	36.9	1.0	0.03	-0.020	0.033	1.1E-05
YER183C	YER183C	S288C_Ator08_hmg124h_4.JPG.dat	1058.3	22.8	1.0	0.02	0.001	0.021	2.4E-06
YER184C	YER184C	S288C_Ator08_hmg124h_4.JPG.dat	1056.3	36.2	1.0	0.03	0.006	0.034	1.0E-05
YER185W	YER185W	S288C_Ator08_hmg124h_4.JPG.dat	1000.0	102.3	1.0	0.02	0.048	0.019	1.2E-04
YER186C	YER186C	S288C_Ator08_hmg124h_4.JPG.dat	1043.5	19.3	1.0	0.02	-0.015	0.019	1.8E-06
YER187W	YER187W	S288C_Ator08_hmg124h_4.JPG.dat	1099.0	20.6	1.0	0.02	0.002	0.018	1.4E-06
YER188W	YER188W	S288C_Ator08_hmg124h_4.JPG.dat	959.8	13.9	1.0	0.03	-0.021	0.032	9.8E-06
YFL006W	YFL006W	S288C_Ator08_hmg124h_4.JPG.dat	1072.8	35.3	1.0	0.03	0.021	0.030	7.7E-06
YFL011W	YFL011W	S288C_Ator08_hmg124h_4.JPG.dat	1142.5	31.0	1.0	0.02	0.009	0.025	3.8E-06
YFL013W-A	YFL013W-A	S288C_Ator08_hmg124h_4.JPG.dat	984.3	57.3	0.9	0.01	-0.032	0.011	4.7E-05
YFL014W	YFL014W	S288C_Ator08_hmg124h_4.JPG.dat	1079.3	36.2	1.0	0.01	0.015	0.009	2.6E-05
YFL015C	YFL015C	S288C_Ator08_hmg124h_4.JPG.dat	1151.5	24.9	1.0	0.01	0.010	0.006	1.1E-05
YFL018C	YFL018C	S288C_Ator08_hmg124h_4.JPG.dat	1097.5	8.3	1.0	0.00	0.049	0.001	4.8E-07

YFL019C	YFL019C	S288C_Ator08_hmg124h_4.JPG.dat	1043.5	38.5	1.0	0.01	-0.043	0.012	4.7E-05
YFL020C	YFL020C	S288C_Ator08_hmg124h_4.JPG.dat	1048.3	23.1	1.0	0.01	-0.040	0.005	8.6E-06
YFL021W	YFL021W	S288C_Ator08_hmg124h_4.JPG.dat	1091.0	31.5	1.0	0.03	0.022	0.028	5.8E-06
YFL023W	YFL023W	S288C_Ator08_hmg124h_4.JPG.dat	1047.0	52.4	1.0	0.05	0.128	0.049	3.7E-05
YFL025C	YFL025C	S288C_Ator08_hmg124h_4.JPG.dat	1045.0	26.0	1.1	0.03	0.026	0.030	6.1E-06
YFL026W	YFL026W	S288C_Ator08_hmg124h_4.JPG.dat	0.0	0.0	0.0	0.00	0.000	0.000	
YFL027C	YFL027C	S288C_Ator08_hmg124h_4.JPG.dat	1119.8	32.8	1.0	0.03	0.014	0.031	7.8E-06
YFL028C	YFL028C	S288C_Ator08_hmg124h_4.JPG.dat	1100.3	27.7	1.0	0.01	0.044	0.007	1.4E-05
YFL030W	YFL030W	S288C_Ator08_hmg124h_4.JPG.dat	1042.8	38.9	1.0	0.01	-0.009	0.012	4.8E-05
YFL031W	YFL031W	S288C_Ator08_hmg124h_4.JPG.dat	825.8	8.1	0.8	0.00	-0.219	0.002	2.9E-06
YFL032W	YFL032W	S288C_Ator08_hmg124h_4.JPG.dat	831.5	176.4	0.7	0.04	-0.222	0.037	9.6E-04
YFL034C-A	YFL034C-A	S288C_Ator08_hmg124h_4.JPG.dat	1043.8	16.1	1.0	0.01	-0.024	0.013	6.0E-07
YFL034W	YFL034W	S288C_Ator08_hmg124h_4.JPG.dat	1001.8	38.7	1.0	0.04	-0.075	0.036	1.4E-05
YFL035C-B	YFL035C-B	S288C_Ator08_hmg124h_4.JPG.dat	1072.0	41.6	1.0	0.01	0.030	0.011	3.8E-05
YFL036W	YFL036W	S288C_Ator08_hmg124h_4.JPG.dat	405.5	514.1	0.1	0.13	-0.044	0.134	2.0E-01
YFL040W	YFL040W	S288C_Ator08_hmg124h_4.JPG.dat	1048.0	55.1	1.1	0.04	-0.003	0.037	1.1E-05
YFL041W	YFL041W	S288C_Ator08_hmg124h_4.JPG.dat	1132.3	21.7	1.0	0.03	0.029	0.027	4.9E-06
YFL042C	YFL042C	S288C_Ator08_hmg124h_4.JPG.dat	1119.3	35.6	1.0	0.03	0.019	0.034	9.8E-06
YFL043C	YFL043C	S288C_Ator08_hmg124h_4.JPG.dat	1155.8	43.6	1.1	0.03	0.008	0.032	7.5E-06
YFL044C	YFL044C	S288C_Ator08_hmg124h_4.JPG.dat	1099.5	49.2	1.0	0.05	-0.019	0.049	3.2E-05
YFL046W	YFL046W	S288C_Ator08_hmg124h_4.JPG.dat	1097.8	42.5	1.0	0.04	-0.024	0.036	1.4E-05
YFL047W	YFL047W	S288C_Ator08_hmg124h_4.JPG.dat	1050.0	93.7	1.0	0.09	-0.051	0.088	2.1E-04
YFL048C	YFL048C	S288C_Ator08_hmg124h_4.JPG.dat	1066.3	26.2	1.0	0.01	-0.001	0.006	1.2E-05
YFL049W	YFL049W	S288C_Ator08_hmg124h_4.JPG.dat	1030.0	56.0	1.0	0.06	0.018	0.060	6.0E-05
YFL050C	YFL050C	S288C_Ator08_hmg124h_4.JPG.dat	1053.3	8.3	1.0	0.01	0.005	0.015	8.4E-07
YFL051C	YFL051C	S288C_Ator08_hmg124h_4.JPG.dat	1091.3	10.0	1.0	0.00	-0.005	0.002	1.2E-06
YFL052W	YFL052W	S288C_Ator08_hmg124h_4.JPG.dat	1013.8	28.4	1.0	0.02	-0.047	0.023	3.1E-06
YFL053W	YFL053W	S288C_Ator08_hmg124h_4.JPG.dat	1103.5	14.3	1.0	0.01	0.011	0.014	6.7E-07
YFL054C	YFL054C	S288C_Ator08_hmg124h_4.JPG.dat	1043.3	58.8	0.9	0.05	-0.055	0.054	5.1E-05
YFL055W	YFL055W	S288C_Ator08_hmg124h_4.JPG.dat	1132.5	25.9	1.0	0.00	-0.021	0.005	7.5E-06
YFL056C	YFL056C	S288C_Ator08_hmg124h_4.JPG.dat	1154.0	32.7	1.0	0.02	0.027	0.023	3.1E-06
YFR006W	YFR006W	S288C_Ator08_hmg124h_4.JPG.dat	1117.5	76.2	1.0	0.07	-0.007	0.065	8.0E-05
YFR007W	YFR007W	S288C_Ator08_hmg124h_4.JPG.dat	1093.3	86.8	1.0	0.02	0.020	0.018	1.0E-04
YFR008W	YFR008W	S288C_Ator08_hmg124h_4.JPG.dat	1113.0	30.3	1.0	0.03	0.025	0.026	4.6E-06
YFR009W	YFR009W	S288C_Ator08_hmg124h_4.JPG.dat	1067.5	34.9	1.0	0.03	0.002	0.033	1.0E-05
YFR010W	YFR010W	S288C_Ator08_hmg124h_4.JPG.dat	862.8	139.6	0.8	0.13	0.166	0.127	1.1E-03
YFR011C	YFR011C	S288C_Ator08_hmg124h_4.JPG.dat	1086.5	14.6	1.1	0.04	0.045	0.043	1.8E-05
YOR202W	YOR202W	S288C_Ator08_hmg124h_4.JPG.dat	961.8	227.1	1.0	0.22	-0.021	0.220	1.3E-180
1	1	S288C_Ator08_hmg124h_3.JPG.dat	1092.5	135.8	1.1	0.02	0.091	0.016	6.6E-05
2	2	S288C_Ator08_hmg124h_3.JPG.dat	929.3	79.4	1.0	0.02	0.042	0.015	8.0E-05
3	3	S288C_Ator08_hmg124h_3.JPG.dat	925.0	56.9	1.0	0.07	-0.062	0.068	9.7E-05
4	4	S288C_Ator08_hmg124h_3.JPG.dat	893.0	75.3	0.9	0.09	-0.075	0.095	2.7E-04
YDL185W	YDL185W	S288C_Ator08_hmg124h_3.JPG.dat	991.3	54.1	1.0	0.05	-0.043	0.052	3.9E-05
YDL186W	YDL186W	S288C_Ator08_hmg124h_3.JPG.dat	1123.3	37.4	1.1	0.04	0.107	0.036	1.0E-05
YDL187C	YDL187C	S288C_Ator08_hmg124h_3.JPG.dat	1087.8	15.9	1.0	0.00	-0.006	0.005	7.8E-06
YDL188C	YDL188C	S288C_Ator08_hmg124h_3.JPG.dat	1053.0	28.5	1.0	0.03	-0.018	0.027	5.3E-06
YDL189W	YDL189W	S288C_Ator08_hmg124h_3.JPG.dat	1066.5	12.7	1.0	0.01	-0.020	0.011	3.7E-07
YDL190C	YDL190C	S288C_Ator08_hmg124h_3.JPG.dat	1029.3	11.5	1.0	0.01	-0.036	0.015	9.4E-07
YDL191W	YDL191W	S288C_Ator08_hmg124h_3.JPG.dat	1014.0	47.8	1.0	0.05	0.016	0.046	2.9E-05
YDL192W	YDL192W	S288C_Ator08_hmg124h_3.JPG.dat	1011.5	10.0	1.0	0.01	0.019	0.007	1.3E-07
YDL194W	YDL194W	S288C_Ator08_hmg124h_3.JPG.dat	955.5	58.0	0.9	0.05	-0.108	0.052	5.3E-05
YDL197C	YDL197C	S288C_Ator08_hmg124h_3.JPG.dat	1068.3	17.4	1.0	0.01	0.045	0.007	1.4E-05
YDL199C	YDL199C	S288C_Ator08_hmg124h_3.JPG.dat	1032.0	34.0	1.0	0.04	-0.028	0.045	2.4E-05
YDL200C	YDL200C	S288C_Ator08_hmg124h_3.JPG.dat	962.3	68.1	1.0	0.01	0.043	0.010	3.4E-05
YDL201W	YDL201W	S288C_Ator08_hmg124h_3.JPG.dat	1065.8	32.9	1.0	0.03	0.024	0.031	8.3E-06
YDL203C	YDL203C	S288C_Ator08_hmg124h_3.JPG.dat	1027.5	36.6	1.0	0.03	-0.023	0.034	1.1E-05
YDL204W	YDL204W	S288C_Ator08_hmg124h_3.JPG.dat	1043.8	17.0	1.0	0.02	0.023	0.020	2.3E-06
YDL206W	YDL206W	S288C_Ator08_hmg124h_3.JPG.dat	1031.8	2.5	1.0	0.00	-0.032	0.002	9.5E-07
YDL210W	YDL210W	S288C_Ator08_hmg124h_3.JPG.dat	1040.0	23.3	1.0	0.02	0.027	0.022	3.0E-06
YDL211C	YDL211C	S288C_Ator08_hmg124h_3.JPG.dat	1043.8	12.0	1.0	0.01	-0.006	0.015	8.7E-07
YDL213C	YDL213C	S288C_Ator08_hmg124h_3.JPG.dat	1001.0	59.1	1.0	0.06	-0.029	0.058	6.2E-05
YDL214C	YDL214C	S288C_Ator08_hmg124h_3.JPG.dat	1120.0	22.1	1.0	0.00	-0.019	0.001	1.2E-07
YDL215C	YDL215C	S288C_Ator08_hmg124h_3.JPG.dat	1036.8	32.0	1.0	0.03	0.009	0.032	9.3E-06
YDL216C	YDL216C	S288C_Ator08_hmg124h_3.JPG.dat	1006.3	45.5	1.0	0.04	-0.033	0.040	1.8E-05
YDL218W	YDL218W	S288C_Ator08_hmg124h_3.JPG.dat	988.8	31.8	1.0	0.03	-0.042	0.034	1.1E-05
YDL219W	YDL219W	S288C_Ator08_hmg124h_3.JPG.dat	1031.3	42.6	1.0	0.04	-0.002	0.038	1.6E-05
YDL222C	YDL222C	S288C_Ator08_hmg124h_3.JPG.dat	987.5	31.1	1.0	0.04	-0.029	0.036	1.4E-05
YDL223C	YDL223C	S288C_Ator08_hmg124h_3.JPG.dat	999.8	22.0	1.0	0.02	-0.017	0.022	2.9E-06
YDL224C	YDL224C	S288C_Ator08_hmg124h_3.JPG.dat	1110.0	24.3	1.1	0.02	0.104	0.025	3.3E-06
YDL226C	YDL226C	S288C_Ator08_hmg124h_3.JPG.dat	971.0	32.6	0.9	0.04	-0.044	0.037	1.6E-05

YDL227C	YDL227C	S288C_Ator08_hmg124h_3.JPG.dat	1026.3	11.4	1.0	0.01	0.036	0.013	6.3E-07
YDL229W	YDL229W	S288C_Ator08_hmg124h_3.JPG.dat	1057.0	14.3	1.0	0.01	0.033	0.014	7.4E-07
YDL230W	YDL230W	S288C_Ator08_hmg124h_3.JPG.dat	1090.3	20.9	1.0	0.03	0.009	0.025	3.8E-06
YDL231C	YDL231C	S288C_Ator08_hmg124h_3.JPG.dat	992.8	45.9	1.0	0.04	-0.019	0.042	2.1E-05
YDL232W	YDL232W	S288C_Ator08_hmg124h_3.JPG.dat	674.8	339.3	0.7	0.36	-0.080	0.359	3.1E-02
YDL233W	YDL233W	S288C_Ator08_hmg124h_3.JPG.dat	1062.8	38.9	1.1	0.04	0.047	0.043	1.8E-05
YDL234C	YDL234C	S288C_Ator08_hmg124h_3.JPG.dat	1057.8	17.7	1.0	0.00	0.007	0.004	3.9E-06
YDL236W	YDL236W	S288C_Ator08_hmg124h_3.JPG.dat	982.0	19.4	1.0	0.02	-0.036	0.021	2.6E-06
YDL237W	YDL237W	S288C_Ator08_hmg124h_3.JPG.dat	1006.0	16.6	1.0	0.02	0.037	0.019	1.8E-06
YDL238C	YDL238C	S288C_Ator08_hmg124h_3.JPG.dat	1040.3	13.1	1.0	0.00	-0.003	0.004	4.7E-06
YDL239C	YDL239C	S288C_Ator08_hmg124h_3.JPG.dat	1027.8	10.6	1.0	0.01	-0.003	0.010	2.5E-07
YDL240W	YDL240W	S288C_Ator08_hmg124h_3.JPG.dat	1033.3	21.7	1.0	0.00	0.040	0.004	5.6E-06
YDL241W	YDL241W	S288C_Ator08_hmg124h_3.JPG.dat	961.8	52.0	0.9	0.01	-0.068	0.015	8.8E-05
YDL242W	YDL242W	S288C_Ator08_hmg124h_3.JPG.dat	975.3	16.8	0.9	0.01	-0.067	0.011	4.9E-07
YDL243C	YDL243C	S288C_Ator08_hmg124h_3.JPG.dat	1024.3	34.6	1.0	0.03	-0.007	0.032	8.8E-06
YDR001C	YDR001C	S288C_Ator08_hmg124h_3.JPG.dat	1013.3	33.1	1.0	0.01	0.007	0.013	4.8E-05
YDR003W	YDR003W	S288C_Ator08_hmg124h_3.JPG.dat	976.0	29.0	1.0	0.02	-0.007	0.023	3.4E-06
YDR004W	YDR004W	S288C_Ator08_hmg124h_3.JPG.dat	886.5	56.2	0.9	0.06	-0.032	0.057	8.1E-05
YDR005C	YDR005C	S288C_Ator08_hmg124h_3.JPG.dat	1082.5	31.8	1.0	0.01	-0.017	0.009	2.7E-05
YDR006C	YDR006C	S288C_Ator08_hmg124h_3.JPG.dat	964.0	10.5	1.0	0.02	0.000	0.017	1.5E-06
YDR007W	YDR007W	S288C_Ator08_hmg124h_3.JPG.dat	841.5	44.8	0.8	0.05	-0.111	0.046	4.4E-05
YDR008C	YDR008C	S288C_Ator08_hmg124h_3.JPG.dat	1041.5	19.8	1.0	0.03	0.007	0.030	6.5E-06
YDR009W	YDR009W	S288C_Ator08_hmg124h_3.JPG.dat	992.5	28.9	1.0	0.02	-0.005	0.024	3.9E-06
YDR010C	YDR010C	S288C_Ator08_hmg124h_3.JPG.dat	1042.3	22.4	1.0	0.01	0.036	0.005	8.7E-06
YDR011W	YDR011W	S288C_Ator08_hmg124h_3.JPG.dat	1022.5	54.9	1.0	0.05	-0.023	0.048	3.1E-05
YDR014W	YDR014W	S288C_Ator08_hmg124h_3.JPG.dat	939.3	20.6	0.9	0.00	-0.090	0.003	4.0E-06
YDR015C	YDR015C	S288C_Ator08_hmg124h_3.JPG.dat	1054.5	49.9	1.1	0.05	0.019	0.045	2.1E-05
YDR018C	YDR018C	S288C_Ator08_hmg124h_3.JPG.dat	1042.0	51.2	1.1	0.02	0.031	0.017	9.1E-05
YDR019C	YDR019C	S288C_Ator08_hmg124h_3.JPG.dat	1088.5	16.9	1.0	0.02	0.026	0.020	2.2E-06
YDR020C	YDR020C	S288C_Ator08_hmg124h_3.JPG.dat	1115.5	18.3	1.1	0.03	0.004	0.025	3.7E-06
YDR022C	YDR022C	S288C_Ator08_hmg124h_3.JPG.dat	1046.5	5.3	1.0	0.01	0.011	0.008	1.4E-07
YDR024W	YDR024W	S288C_Ator08_hmg124h_3.JPG.dat	968.8	53.4	1.0	0.02	-0.007	0.018	1.2E-04
YDR025W	YDR025W	S288C_Ator08_hmg124h_3.JPG.dat	995.8	28.6	1.0	0.03	0.000	0.028	6.3E-06
YDR048C	YDR048C	S288C_Ator08_hmg124h_3.JPG.dat	971.3	74.1	1.0	0.07	-0.041	0.071	1.1E-04
YDR049W	YDR049W	S288C_Ator08_hmg124h_3.JPG.dat	1038.3	19.4	1.0	0.02	0.017	0.018	1.6E-06
YDR051C	YDR051C	S288C_Ator08_hmg124h_3.JPG.dat	1096.3	28.7	1.1	0.01	0.038	0.012	4.2E-05
YDR055W	YDR055W	S288C_Ator08_hmg124h_3.JPG.dat	1021.8	46.0	1.0	0.05	-0.015	0.045	2.6E-05
YDR056C	YDR056C	S288C_Ator08_hmg124h_3.JPG.dat	1056.5	89.3	1.0	0.02	-0.001	0.023	1.8E-04
YDR057W	YDR057W	S288C_Ator08_hmg124h_3.JPG.dat	1111.0	110.8	1.1	0.12	0.038	0.117	3.7E-04
YDR058C	YDR058C	S288C_Ator08_hmg124h_3.JPG.dat	1026.3	67.2	0.9	0.06	-0.028	0.057	6.0E-05
YDR059C	YDR059C	S288C_Ator08_hmg124h_3.JPG.dat	1179.3	37.1	1.1	0.04	0.003	0.042	1.5E-05
YDR061W	YDR061W	S288C_Ator08_hmg124h_3.JPG.dat	1142.3	26.8	1.1	0.02	0.032	0.018	1.2E-06
YDR063W	YDR063W	S288C_Ator08_hmg124h_3.JPG.dat	1102.3	40.1	1.0	0.04	-0.019	0.038	1.3E-05
YDR066C	YDR066C	S288C_Ator08_hmg124h_3.JPG.dat	1066.3	33.7	1.0	0.01	-0.015	0.008	2.2E-05
YDR067C	YDR067C	S288C_Ator08_hmg124h_3.JPG.dat	1043.3	55.0	1.0	0.01	-0.032	0.010	3.7E-05
YDR068W	YDR068W	S288C_Ator08_hmg124h_3.JPG.dat	1053.3	33.6	1.0	0.03	-0.007	0.026	5.0E-06
YDR070C	YDR070C	S288C_Ator08_hmg124h_3.JPG.dat	1045.0	24.4	1.0	0.01	-0.008	0.010	3.7E-05
YDR071C	YDR071C	S288C_Ator08_hmg124h_3.JPG.dat	976.8	95.4	0.9	0.08	-0.111	0.080	1.8E-04
YDR072C	YDR072C	S288C_Ator08_hmg124h_3.JPG.dat	1054.0	57.5	0.9	0.06	0.029	0.060	7.2E-05
YDR073W	YDR073W	S288C_Ator08_hmg124h_3.JPG.dat	908.8	38.5	0.9	0.03	-0.084	0.026	6.0E-06
YDR074W	YDR074W	S288C_Ator08_hmg124h_3.JPG.dat	744.5	28.4	0.7	0.03	-0.042	0.026	1.3E-05
YDR075W	YDR075W	S288C_Ator08_hmg124h_3.JPG.dat	1168.3	20.2	1.1	0.02	0.076	0.019	1.5E-06
YDR076W	YDR076W	S288C_Ator08_hmg124h_3.JPG.dat	944.0	37.7	0.9	0.04	0.028	0.036	1.7E-05
YDR077W	YDR077W	S288C_Ator08_hmg124h_3.JPG.dat	1044.5	20.0	1.0	0.02	0.022	0.016	1.0E-06
YDR080W	YDR080W	S288C_Ator08_hmg124h_3.JPG.dat	823.5	29.1	0.8	0.02	-0.077	0.024	7.7E-06
YDR083W	YDR083W	S288C_Ator08_hmg124h_3.JPG.dat	853.3	122.7	0.8	0.00	-0.037	0.005	1.3E-05
YDR084C	YDR084C	S288C_Ator08_hmg124h_3.JPG.dat	1068.8	12.0	1.0	0.01	0.020	0.010	3.0E-07
YDR085C	YDR085C	S288C_Ator08_hmg124h_3.JPG.dat	1096.8	54.2	1.0	0.01	-0.014	0.010	3.5E-05
YDR089W	YDR089W	S288C_Ator08_hmg124h_3.JPG.dat	1091.0	11.9	1.0	0.01	-0.003	0.006	6.0E-08
YDR090C	YDR090C	S288C_Ator08_hmg124h_3.JPG.dat	1047.8	30.0	1.0	0.03	0.045	0.031	7.9E-06
YDR092W	YDR092W	S288C_Ator08_hmg124h_3.JPG.dat	998.5	99.0	1.0	0.11	0.032	0.107	3.1E-04
YDR093W	YDR093W	S288C_Ator08_hmg124h_3.JPG.dat	971.8	44.8	0.9	0.05	-0.068	0.046	3.1E-05
YDR094W	YDR094W	S288C_Ator08_hmg124h_3.JPG.dat	1087.5	18.2	1.0	0.02	0.006	0.016	1.1E-06
YDR095C	YDR095C	S288C_Ator08_hmg124h_3.JPG.dat	1052.0	24.5	1.0	0.01	-0.009	0.009	2.6E-05
YDR096W	YDR096W	S288C_Ator08_hmg124h_3.JPG.dat	1154.0	20.2	1.1	0.02	0.041	0.021	1.8E-06
YDR097C	YDR097C	S288C_Ator08_hmg124h_3.JPG.dat	980.0	47.6	1.0	0.05	-0.036	0.046	3.1E-05
YDR098C	YDR098C	S288C_Ator08_hmg124h_3.JPG.dat	1043.5	23.4	1.0	0.02	-0.030	0.023	3.1E-06
YDR099W	YDR099W	S288C_Ator08_hmg124h_3.JPG.dat	1039.3	24.8	1.0	0.02	0.003	0.021	2.8E-06
YDR100W	YDR100W	S288C_Ator08_hmg124h_3.JPG.dat	1075.3	19.8	1.0	0.02	-0.007	0.018	1.6E-06
YDR101C	YDR101C	S288C_Ator08_hmg124h_3.JPG.dat	979.0	43.9	0.9	0.04	0.025	0.044	2.8E-05

YDR102C	YDR102C	S288C_Ator08_hmg124h_3.JPG.dat	1068.8	50.1	1.0	0.05	0.016	0.049	2.9E-05
YDR103W	YDR103W	S288C_Ator08_hmg124h_3.JPG.dat	0.0	0.0	0.0	0.00	0.000	0.000	
YDR104C	YDR104C	S288C_Ator08_hmg124h_3.JPG.dat	915.0	50.2	1.0	0.06	-0.122	0.060	6.8E-05
YDR105C	YDR105C	S288C_Ator08_hmg124h_3.JPG.dat	1057.0	16.4	1.0	0.02	0.037	0.020	1.9E-06
YDR107C	YDR107C	S288C_Ator08_hmg124h_3.JPG.dat	1048.3	10.5	1.0	0.01	0.004	0.012	4.9E-07
YDR108W	YDR108W	S288C_Ator08_hmg124h_3.JPG.dat	939.8	18.3	0.9	0.01	0.023	0.005	1.0E-05
YDR109C	YDR109C	S288C_Ator08_hmg124h_3.JPG.dat	1003.3	49.2	1.0	0.05	-0.035	0.047	3.0E-05
YDR110W	YDR110W	S288C_Ator08_hmg124h_3.JPG.dat	1027.0	11.9	1.0	0.01	0.045	0.011	3.4E-07
YDR111C	YDR111C	S288C_Ator08_hmg124h_3.JPG.dat	1030.0	14.1	1.0	0.01	-0.007	0.009	1.9E-07
YDR112W	YDR112W	S288C_Ator08_hmg124h_3.JPG.dat	1044.5	53.0	1.0	0.05	0.034	0.053	4.0E-05
YDR116C	YDR116C	S288C_Ator08_hmg124h_3.JPG.dat	1019.3	47.7	1.0	0.05	0.011	0.049	3.2E-05
YDR117C	YDR117C	S288C_Ator08_hmg124h_3.JPG.dat	1034.3	21.2	1.0	0.02	0.027	0.021	2.4E-06
YDR119W	YDR119W	S288C_Ator08_hmg124h_3.JPG.dat	1012.0	85.7	1.1	0.09	0.074	0.088	1.5E-04
YDR120C	YDR120C	S288C_Ator08_hmg124h_3.JPG.dat	1098.3	52.8	1.1	0.05	0.043	0.049	2.5E-05
YDR121W	YDR121W	S288C_Ator08_hmg124h_3.JPG.dat	916.8	42.5	0.9	0.04	-0.062	0.042	2.7E-05
YDR122W	YDR122W	S288C_Ator08_hmg124h_3.JPG.dat	1022.5	33.7	1.0	0.03	0.003	0.034	9.6E-06
YDR123C	YDR123C	S288C_Ator08_hmg124h_3.JPG.dat	797.3	26.7	0.8	0.02	-0.025	0.024	7.2E-06
YDR124W	YDR124W	S288C_Ator08_hmg124h_3.JPG.dat	1031.3	9.8	1.0	0.00	0.014	0.004	2.0E-08
YDR125C	YDR125C	S288C_Ator08_hmg124h_3.JPG.dat	1005.5	17.2	1.0	0.01	0.015	0.014	8.2E-07
YDR126W	YDR126W	S288C_Ator08_hmg124h_3.JPG.dat	1011.0	12.5	1.0	0.01	0.008	0.012	5.5E-07
YDR127W	YDR127W	S288C_Ator08_hmg124h_3.JPG.dat	886.8	17.2	0.9	0.01	-0.047	0.015	1.4E-06
YDR128W	YDR128W	S288C_Ator08_hmg124h_3.JPG.dat	994.8	65.5	1.0	0.06	0.009	0.061	6.6E-05
YDR130C	YDR130C	S288C_Ator08_hmg124h_3.JPG.dat	1001.0	11.2	1.0	0.01	-0.040	0.013	5.7E-07
YDR131C	YDR131C	S288C_Ator08_hmg124h_3.JPG.dat	981.0	52.4	1.0	0.04	0.043	0.036	1.1E-05
YDR132C	YDR132C	S288C_Ator08_hmg124h_3.JPG.dat	1058.3	15.5	1.0	0.01	-0.020	0.008	1.4E-07
YDR133C	YDR133C	S288C_Ator08_hmg124h_3.JPG.dat	1046.3	34.0	1.0	0.01	0.015	0.014	6.3E-05
YDR134C	YDR134C	S288C_Ator08_hmg124h_3.JPG.dat	954.8	89.0	0.9	0.09	-0.077	0.088	2.2E-04
YDR135C	YDR135C	S288C_Ator08_hmg124h_3.JPG.dat	956.3	33.9	1.0	0.03	-0.019	0.027	6.0E-06
YDR139C	YDR139C	S288C_Ator08_hmg124h_3.JPG.dat	1027.8	28.5	1.0	0.03	-0.012	0.026	4.5E-06
YDR142C	YDR142C	S288C_Ator08_hmg124h_3.JPG.dat	985.8	11.9	1.0	0.01	-0.025	0.008	1.3E-07
YDR143C	YDR143C	S288C_Ator08_hmg124h_3.JPG.dat	1088.8	24.0	1.1	0.02	0.045	0.024	3.0E-06
YDR144C	YDR144C	S288C_Ator08_hmg124h_3.JPG.dat	1010.0	50.8	1.0	0.05	0.034	0.054	4.3E-05
YDR146C	YDR146C	S288C_Ator08_hmg124h_3.JPG.dat	991.8	47.6	1.0	0.04	0.012	0.044	2.6E-05
YDR147W	YDR147W	S288C_Ator08_hmg124h_3.JPG.dat	1034.8	43.6	1.0	0.04	0.020	0.041	1.9E-05
YDR148C	YDR148C	S288C_Ator08_hmg124h_3.JPG.dat	1008.8	30.0	1.0	0.03	0.033	0.033	9.0E-06
YDR149C	YDR149C	S288C_Ator08_hmg124h_3.JPG.dat	967.0	63.2	1.0	0.01	0.022	0.007	1.8E-05
YDR150W	YDR150W	S288C_Ator08_hmg124h_3.JPG.dat	1010.3	44.6	1.0	0.01	0.003	0.014	7.3E-05
YDR151C	YDR151C	S288C_Ator08_hmg124h_3.JPG.dat	1045.8	68.3	1.0	0.06	-0.042	0.062	6.5E-05
YDR152W	YDR152W	S288C_Ator08_hmg124h_3.JPG.dat	1031.5	16.3	1.0	0.02	0.027	0.016	1.0E-06
YDR153C	YDR153C	S288C_Ator08_hmg124h_3.JPG.dat	828.0	12.4	0.8	0.01	-0.156	0.009	3.4E-07
YDR154C	YDR154C	S288C_Ator08_hmg124h_3.JPG.dat	970.3	20.7	1.0	0.03	-0.086	0.026	5.4E-06
YDR155C	YDR155C	S288C_Ator08_hmg124h_3.JPG.dat	1023.5	7.3	1.0	0.00	-0.026	0.001	1.6E-07
YDR156W	YDR156W	S288C_Ator08_hmg124h_3.JPG.dat	956.5	30.1	0.9	0.03	0.022	0.025	5.6E-06
YDR157W	YDR157W	S288C_Ator08_hmg124h_3.JPG.dat	1060.3	12.0	1.0	0.00	-0.018	0.004	5.8E-06
YDR158W	YDR158W	S288C_Ator08_hmg124h_3.JPG.dat	1086.5	18.9	1.0	0.02	0.059	0.020	2.2E-06
YDR159W	YDR159W	S288C_Ator08_hmg124h_3.JPG.dat	171.5	198.0	0.2	0.19	-0.040	0.191	1.8E-01
YDR161W	YDR161W	S288C_Ator08_hmg124h_3.JPG.dat	1103.3	49.9	1.0	0.04	0.085	0.041	1.8E-05
YDR162C	YDR162C	S288C_Ator08_hmg124h_3.JPG.dat	896.5	184.2	0.8	0.17	-0.134	0.171	2.4E-03
YDR163W	YDR163W	S288C_Ator08_hmg124h_3.JPG.dat	1095.5	54.3	1.0	0.06	0.059	0.063	6.1E-05
YDR165W	YDR165W	S288C_Ator08_hmg124h_3.JPG.dat	1047.8	19.3	1.0	0.01	-0.008	0.011	3.6E-07
YDR169C	YDR169C	S288C_Ator08_hmg124h_3.JPG.dat	1025.5	22.3	1.0	0.01	-0.052	0.008	2.1E-05
YDR171W	YDR171W	S288C_Ator08_hmg124h_3.JPG.dat	1053.8	34.6	1.0	0.03	-0.031	0.026	5.0E-06
YDR173C	YDR173C	S288C_Ator08_hmg124h_3.JPG.dat	1047.3	22.9	1.0	0.00	-0.046	0.004	6.3E-06
YDR174W	YDR174W	S288C_Ator08_hmg124h_3.JPG.dat	1029.0	80.3	1.0	0.07	0.005	0.069	9.8E-05
YDR178W	YDR178W	S288C_Ator08_hmg124h_3.JPG.dat	1102.3	98.8	1.0	0.00	0.026	0.005	7.1E-06
YDR179C	YDR179C	S288C_Ator08_hmg124h_3.JPG.dat	1090.3	59.6	1.0	0.04	0.023	0.039	1.6E-05
YDR179W-	YDR179W-A	S288C_Ator08_hmg124h_3.JPG.dat	906.3	62.8	0.9	0.07	0.048	0.074	1.6E-04
YDR181C	YDR181C	S288C_Ator08_hmg124h_3.JPG.dat	1050.5	54.7	1.0	0.02	0.000	0.022	1.5E-04
YDR183W	YDR183W	S288C_Ator08_hmg124h_3.JPG.dat	1042.0	48.1	1.0	0.03	0.018	0.030	8.1E-06
YDR184C	YDR184C	S288C_Ator08_hmg124h_3.JPG.dat	1076.8	36.5	1.0	0.02	-0.037	0.023	3.5E-06
YDR185C	YDR185C	S288C_Ator08_hmg124h_3.JPG.dat	1069.5	7.5	1.0	0.02	-0.001	0.021	2.8E-06
YDR186C	YDR186C	S288C_Ator08_hmg124h_3.JPG.dat	990.3	45.1	0.9	0.00	-0.041	0.001	3.3E-07
YDR191W	YDR191W	S288C_Ator08_hmg124h_3.JPG.dat	1107.5	77.5	1.0	0.05	0.011	0.054	4.0E-05
YDR192C	YDR192C	S288C_Ator08_hmg124h_3.JPG.dat	1092.3	31.7	1.0	0.02	-0.021	0.017	1.3E-06
YDR193W	YDR193W	S288C_Ator08_hmg124h_3.JPG.dat	1124.0	35.5	1.0	0.05	-0.057	0.045	2.4E-05
YDR198C	YDR198C	S288C_Ator08_hmg124h_3.JPG.dat	1104.5	45.1	1.0	0.03	0.035	0.026	4.5E-06
YDR199W	YDR199W	S288C_Ator08_hmg124h_3.JPG.dat	1126.8	74.2	1.1	0.05	0.045	0.052	3.1E-05
YDR202C	YDR202C	S288C_Ator08_hmg124h_3.JPG.dat	1018.0	39.2	1.0	0.04	-0.001	0.041	1.8E-05
YDR203W	YDR203W	S288C_Ator08_hmg124h_3.JPG.dat	1121.5	43.7	1.1	0.05	0.038	0.046	2.2E-05
YDR205W	YDR205W	S288C_Ator08_hmg124h_3.JPG.dat	986.0	36.2	0.9	0.02	-0.042	0.024	4.6E-06

YDR206W	YDR206W	S288C_Ator08_hmg124h_3.JPG.dat	1117.0	42.7	1.1	0.04	0.031	0.041	1.6E-05
YDR207C	YDR207C	S288C_Ator08_hmg124h_3.JPG.dat	779.3	151.1	0.8	0.15	-0.059	0.149	2.1E-03
YDR209C	YDR209C	S288C_Ator08_hmg124h_3.JPG.dat	1045.8	18.6	1.0	0.01	0.044	0.014	7.2E-07
YDR210W	YDR210W	S288C_Ator08_hmg124h_3.JPG.dat	1068.5	36.0	1.0	0.03	-0.045	0.029	6.5E-06
YDR213W	YDR213W	S288C_Ator08_hmg124h_3.JPG.dat	922.3	24.0	0.9	0.00	-0.142	0.004	6.4E-06
YDR214W	YDR214W	S288C_Ator08_hmg124h_3.JPG.dat	1081.8	36.0	1.0	0.04	0.012	0.043	2.1E-05
YDR215C	YDR215C	S288C_Ator08_hmg124h_3.JPG.dat	1055.8	53.8	1.0	0.04	0.003	0.042	1.9E-05
YDR216W	YDR216W	S288C_Ator08_hmg124h_3.JPG.dat	1039.0	63.1	1.0	0.04	0.029	0.036	1.2E-05
YDR217C	YDR217C	S288C_Ator08_hmg124h_3.JPG.dat	952.3	54.4	1.0	0.06	-0.014	0.059	6.2E-05
YDR218C	YDR218C	S288C_Ator08_hmg124h_3.JPG.dat	1094.0	23.8	1.1	0.02	0.046	0.024	3.1E-06
YDR219C	YDR219C	S288C_Ator08_hmg124h_3.JPG.dat	1050.0	42.1	1.0	0.04	0.086	0.040	1.7E-05
YDR220C	YDR220C	S288C_Ator08_hmg124h_3.JPG.dat	1013.0	66.6	1.0	0.02	-0.047	0.024	2.0E-04
YDR221W	YDR221W	S288C_Ator08_hmg124h_3.JPG.dat	1035.5	12.2	1.0	0.01	-0.005	0.013	5.8E-07
YDR222W	YDR222W	S288C_Ator08_hmg124h_3.JPG.dat	1015.8	28.7	1.0	0.03	0.026	0.029	6.8E-06
YDR223W	YDR223W	S288C_Ator08_hmg124h_3.JPG.dat	1046.3	27.6	1.0	0.03	0.030	0.028	5.4E-06
YDR225W	YDR225W	S288C_Ator08_hmg124h_3.JPG.dat	909.5	59.3	0.9	0.01	0.048	0.013	7.9E-05
YDR227W	YDR227W	S288C_Ator08_hmg124h_3.JPG.dat	0.0	0.0	0.0	0.00	0.000	0.000	
YDR229W	YDR229W	S288C_Ator08_hmg124h_3.JPG.dat	978.3	25.2	1.0	0.00	-0.038	0.003	2.6E-06
YDR233C	YDR233C	S288C_Ator08_hmg124h_3.JPG.dat	997.3	57.2	1.0	0.06	-0.044	0.062	6.0E-05
YDR234W	YDR234W	S288C_Ator08_hmg124h_3.JPG.dat	0.0	0.0	0.0	0.00	0.000	0.000	
YDR239C	YDR239C	S288C_Ator08_hmg124h_3.JPG.dat	1041.8	14.0	1.0	0.02	-0.004	0.017	1.3E-06
YDR241W	YDR241W	S288C_Ator08_hmg124h_3.JPG.dat	932.0	17.3	0.9	0.01	0.011	0.014	9.3E-07
YDR244W	YDR244W	S288C_Ator08_hmg124h_3.JPG.dat	918.5	51.1	0.9	0.05	0.001	0.050	4.4E-05
YDR245W	YDR245W	S288C_Ator08_hmg124h_3.JPG.dat	728.0	214.9	0.7	0.21	-0.242	0.213	6.4E-03
YDR247W	YDR247W	S288C_Ator08_hmg124h_3.JPG.dat	1058.5	12.7	1.1	0.02	-0.003	0.016	1.1E-06
YDR248C	YDR248C	S288C_Ator08_hmg124h_3.JPG.dat	1044.3	10.9	1.0	0.00	0.006	0.003	2.2E-06
YDR249C	YDR249C	S288C_Ator08_hmg124h_3.JPG.dat	1086.0	4.2	1.1	0.01	0.016	0.007	6.8E-08
YDR250C	YDR250C	S288C_Ator08_hmg124h_3.JPG.dat	1038.5	28.9	1.0	0.03	0.019	0.030	7.5E-06
YDR251W	YDR251W	S288C_Ator08_hmg124h_3.JPG.dat	1074.5	83.9	1.1	0.09	0.006	0.085	1.4E-04
YDR252W	YDR252W	S288C_Ator08_hmg124h_3.JPG.dat	1014.0	33.6	1.0	0.05	0.104	0.053	3.6E-05
YDR253C	YDR253C	S288C_Ator08_hmg124h_3.JPG.dat	939.3	39.0	1.0	0.04	-0.044	0.036	1.5E-05
YDR254W	YDR254W	S288C_Ator08_hmg124h_3.JPG.dat	1003.0	28.1	1.0	0.03	-0.053	0.027	5.8E-06
YDR255C	YDR255C	S288C_Ator08_hmg124h_3.JPG.dat	1073.3	21.4	1.1	0.01	0.031	0.015	8.0E-07
YDR256C	YDR256C	S288C_Ator08_hmg124h_3.JPG.dat	1056.8	23.5	1.0	0.00	-0.032	0.004	4.1E-06
YDR257C	YDR257C	S288C_Ator08_hmg124h_3.JPG.dat	1013.5	47.1	1.0	0.05	0.029	0.047	2.8E-05
YDR258C	YDR258C	S288C_Ator08_hmg124h_3.JPG.dat	966.3	28.1	1.0	0.01	-0.007	0.010	3.4E-05
YDR259C	YDR259C	S288C_Ator08_hmg124h_3.JPG.dat	1033.3	20.5	1.0	0.02	-0.032	0.022	2.6E-06
YDR260C	YDR260C	S288C_Ator08_hmg124h_3.JPG.dat	1036.8	35.1	1.0	0.04	-0.015	0.035	1.1E-05
YDR261C	YDR261C	S288C_Ator08_hmg124h_3.JPG.dat	1054.5	32.4	1.0	0.03	-0.008	0.032	8.9E-06
YDR262W	YDR262W	S288C_Ator08_hmg124h_3.JPG.dat	1023.8	22.9	1.0	0.01	0.031	0.007	1.6E-05
YDR263C	YDR263C	S288C_Ator08_hmg124h_3.JPG.dat	1074.5	44.7	1.1	0.04	0.043	0.037	1.1E-05
YDR265W	YDR265W	S288C_Ator08_hmg124h_3.JPG.dat	983.0	22.9	1.0	0.02	0.056	0.021	2.4E-06
YDR266C	YDR266C	S288C_Ator08_hmg124h_3.JPG.dat	1033.5	53.2	1.0	0.05	-0.025	0.051	3.6E-05
YDR270W	YDR270W	S288C_Ator08_hmg124h_3.JPG.dat	1039.8	49.3	1.0	0.01	-0.019	0.013	5.3E-05
YDR272W	YDR272W	S288C_Ator08_hmg124h_3.JPG.dat	971.5	49.1	1.0	0.05	0.005	0.046	3.1E-05
YDR273W	YDR273W	S288C_Ator08_hmg124h_3.JPG.dat	893.8	66.1	0.9	0.07	-0.075	0.065	1.1E-04
YDR274C	YDR274C	S288C_Ator08_hmg124h_3.JPG.dat	1002.8	18.6	1.0	0.02	-0.023	0.018	1.5E-06
YDR275W	YDR275W	S288C_Ator08_hmg124h_3.JPG.dat	1020.5	9.9	1.0	0.01	0.035	0.008	1.3E-07
YDR276C	YDR276C	S288C_Ator08_hmg124h_3.JPG.dat	935.3	62.6	0.9	0.01	-0.077	0.006	1.3E-05
YDR277C	YDR277C	S288C_Ator08_hmg124h_3.JPG.dat	1010.0	44.4	1.0	0.01	-0.011	0.011	3.7E-05
YDR278C	YDR278C	S288C_Ator08_hmg124h_3.JPG.dat	1022.5	22.5	1.0	0.02	0.019	0.021	2.4E-06
YDR279W	YDR279W	S288C_Ator08_hmg124h_3.JPG.dat	1081.0	27.7	1.1	0.03	0.027	0.028	5.0E-06
YDR281C	YDR281C	S288C_Ator08_hmg124h_3.JPG.dat	1076.0	38.4	1.0	0.01	0.014	0.014	6.0E-05
YDR282C	YDR282C	S288C_Ator08_hmg124h_3.JPG.dat	1110.8	25.9	1.0	0.03	0.047	0.027	4.6E-06
YDR284C	YDR284C	S288C_Ator08_hmg124h_3.JPG.dat	1076.3	35.4	1.0	0.01	-0.035	0.009	2.7E-05
YDR285W	YDR285W	S288C_Ator08_hmg124h_3.JPG.dat	1094.3	54.7	1.0	0.05	0.021	0.051	3.2E-05
YDR286C	YDR286C	S288C_Ator08_hmg124h_3.JPG.dat	1013.3	17.4	1.0	0.01	0.028	0.014	8.0E-07
YDR287W	YDR287W	S288C_Ator08_hmg124h_3.JPG.dat	1003.3	23.8	1.0	0.03	0.044	0.025	4.6E-06
YDR289C	YDR289C	S288C_Ator08_hmg124h_3.JPG.dat	952.0	33.7	0.9	0.01	-0.016	0.012	5.8E-05
YDR291W	YDR291W	S288C_Ator08_hmg124h_3.JPG.dat	1053.3	16.8	1.0	0.02	0.013	0.015	8.6E-07
YDR293C	YDR293C	S288C_Ator08_hmg124h_3.JPG.dat	572.0	38.7	0.5	0.00	-0.399	0.001	7.6E-07
YDR294C	YDR294C	S288C_Ator08_hmg124h_3.JPG.dat	950.3	63.1	0.9	0.01	-0.079	0.008	2.2E-05
YDR297W	YDR297W	S288C_Ator08_hmg124h_3.JPG.dat	957.5	36.2	0.9	0.04	-0.145	0.044	3.4E-05
YDR304C	YDR304C	S288C_Ator08_hmg124h_3.JPG.dat	949.8	6.9	0.9	0.02	0.016	0.019	2.2E-06
YDR305C	YDR305C	S288C_Ator08_hmg124h_3.JPG.dat	950.3	75.5	0.9	0.01	-0.001	0.007	1.6E-05
YDR306C	YDR306C	S288C_Ator08_hmg124h_3.JPG.dat	1103.3	51.8	1.0	0.06	0.029	0.063	6.2E-05
YDR307W	YDR307W	S288C_Ator08_hmg124h_3.JPG.dat	1073.0	31.0	1.0	0.04	0.009	0.041	1.8E-05
YDR309C	YDR309C	S288C_Ator08_hmg124h_3.JPG.dat	1111.0	28.7	1.0	0.02	0.046	0.015	9.1E-07
YDR310C	YDR310C	S288C_Ator08_hmg124h_3.JPG.dat	1046.5	13.2	1.0	0.01	-0.027	0.009	2.3E-07
YDR312W	YDR312W	S288C_Ator08_hmg124h_3.JPG.dat	1096.0	52.0	1.0	0.04	-0.044	0.045	2.4E-05

YDR313C	YDR313C	S288C_Ator08_hmg124h_3.JPG.dat	1144.8	70.4	1.1	0.05	-0.032	0.046	2.2E-05
YDR314C	YDR314C	S288C_Ator08_hmg124h_3.JPG.dat	1110.0	29.0	1.0	0.03	0.006	0.027	5.1E-06
YDR315C	YDR315C	S288C_Ator08_hmg124h_3.JPG.dat	1048.0	63.0	1.0	0.05	0.105	0.053	4.4E-05
YDR316W	YDR316W	S288C_Ator08_hmg124h_3.JPG.dat	1083.8	24.0	1.0	0.01	0.028	0.007	1.7E-05
YDR317W	YDR317W	S288C_Ator08_hmg124h_3.JPG.dat	1005.8	27.9	1.0	0.01	0.076	0.014	6.3E-07
YDR318W	YDR318W	S288C_Ator08_hmg124h_3.JPG.dat	1024.8	72.5	1.0	0.06	-0.009	0.057	5.2E-05
YDR319C	YDR319C	S288C_Ator08_hmg124h_3.JPG.dat	991.3	51.9	0.9	0.05	-0.028	0.051	4.4E-05
YDR320C	YDR320C	S288C_Ator08_hmg124h_3.JPG.dat	915.3	15.8	0.9	0.01	-0.074	0.014	1.1E-06
YDR321W	YDR321W	S288C_Ator08_hmg124h_3.JPG.dat	1015.3	12.6	1.0	0.02	0.005	0.023	3.5E-06
YDR329C	YDR329C	S288C_Ator08_hmg124h_3.JPG.dat	1005.8	12.4	1.0	0.02	0.036	0.022	3.3E-06
YDR330W	YDR330W	S288C_Ator08_hmg124h_3.JPG.dat	1103.8	12.6	1.1	0.00	0.030	0.002	1.1E-06
YDR332W	YDR332W	S288C_Ator08_hmg124h_3.JPG.dat	234.0	468.0	0.0	0.00	0.000	0.000	
YDR333C	YDR333C	S288C_Ator08_hmg124h_3.JPG.dat	1106.0	21.5	1.0	0.02	0.008	0.016	1.1E-06
YDR334W	YDR334W	S288C_Ator08_hmg124h_3.JPG.dat	929.3	22.8	0.9	0.03	-0.063	0.030	1.1E-05
YDR335W	YDR335W	S288C_Ator08_hmg124h_3.JPG.dat	1277.0	102.5	1.3	0.03	0.232	0.027	1.5E-04
YDR336W	YDR336W	S288C_Ator08_hmg124h_3.JPG.dat	962.5	37.8	1.0	0.03	-0.029	0.032	8.7E-06
YDR338C	YDR338C	S288C_Ator08_hmg124h_3.JPG.dat	955.8	111.5	0.9	0.10	-0.079	0.101	3.4E-04
YDR340W	YDR340W	S288C_Ator08_hmg124h_3.JPG.dat	1056.0	17.6	1.0	0.02	0.036	0.016	1.1E-06
YDR344C	YDR344C	S288C_Ator08_hmg124h_3.JPG.dat	1078.5	16.2	1.1	0.01	0.066	0.014	5.5E-07
YDR345C	YDR345C	S288C_Ator08_hmg124h_3.JPG.dat	879.0	35.7	0.9	0.01	-0.115	0.011	5.0E-05
YDR346C	YDR346C	S288C_Ator08_hmg124h_3.JPG.dat	954.0	20.5	1.0	0.02	-0.058	0.021	3.0E-06
YDR348C	YDR348C	S288C_Ator08_hmg124h_3.JPG.dat	964.5	25.8	0.9	0.03	-0.023	0.025	5.1E-06
YDR349C	YDR349C	S288C_Ator08_hmg124h_3.JPG.dat	1033.0	33.0	1.0	0.03	0.029	0.035	1.1E-05
YDR351W	YDR351W	S288C_Ator08_hmg124h_3.JPG.dat	1069.8	20.7	1.0	0.02	-0.015	0.017	1.1E-06
YDR352W	YDR352W	S288C_Ator08_hmg124h_3.JPG.dat	963.5	37.6	1.0	0.04	-0.045	0.038	1.7E-05
YDR354W	YDR354W	S288C_Ator08_hmg124h_3.JPG.dat	912.0	68.3	0.9	0.07	-0.087	0.068	1.2E-04
YDR357C	YDR357C	S288C_Ator08_hmg124h_3.JPG.dat	950.3	59.0	1.0	0.01	-0.041	0.012	4.9E-05
YDR358W	YDR358W	S288C_Ator08_hmg124h_3.JPG.dat	1010.8	13.0	1.0	0.02	-0.033	0.019	1.9E-06
YDR359C	YDR359C	S288C_Ator08_hmg124h_3.JPG.dat	1008.8	247.2	0.9	0.09	-0.045	0.086	3.2E-03
YDR360W	YDR360W	S288C_Ator08_hmg124h_3.JPG.dat	898.0	29.5	0.9	0.03	-0.032	0.028	8.0E-06
YDR363W	YDR363W	S288C_Ator08_hmg124h_3.JPG.dat	884.8	45.3	0.9	0.05	-0.005	0.049	4.5E-05
YDR363W-	YDR363W-A	S288C_Ator08_hmg124h_3.JPG.dat	1062.5	22.8	1.1	0.03	0.086	0.026	3.8E-06
YDR368W	YDR368W	S288C_Ator08_hmg124h_3.JPG.dat	1011.8	24.0	1.0	0.01	0.011	0.008	2.2E-05
YDR369C	YDR369C	S288C_Ator08_hmg124h_3.JPG.dat	930.5	83.2	0.9	0.02	0.080	0.019	1.6E-04
YDR370C	YDR370C	S288C_Ator08_hmg124h_3.JPG.dat	1045.3	25.2	1.0	0.02	0.030	0.022	2.5E-06
YDR371W	YDR371W	S288C_Ator08_hmg124h_3.JPG.dat	1033.5	29.5	1.0	0.03	-0.018	0.030	6.6E-06
YDR372C	YDR372C	S288C_Ator08_hmg124h_3.JPG.dat	882.0	37.0	0.9	0.04	-0.104	0.036	1.8E-05
YDR374C	YDR374C	S288C_Ator08_hmg124h_3.JPG.dat	1022.8	81.4	1.1	0.08	0.067	0.076	9.9E-05
YDR375C	YDR375C	S288C_Ator08_hmg124h_3.JPG.dat	265.3	530.5	0.0	0.00	0.000	0.000	
YDR378C	YDR378C	S288C_Ator08_hmg124h_3.JPG.dat	868.0	64.7	0.9	0.00	0.058	0.005	1.0E-05
YDR379W	YDR379W	S288C_Ator08_hmg124h_3.JPG.dat	1039.8	8.3	1.0	0.00	0.042	0.002	1.7E-06
YDR380W	YDR380W	S288C_Ator08_hmg124h_3.JPG.dat	1016.8	4.5	1.0	0.00	-0.019	0.003	8.6E-09
YDR382W	YDR382W	S288C_Ator08_hmg124h_3.JPG.dat	866.3	106.9	0.9	0.11	0.114	0.105	4.9E-04
YDR383C	YDR383C	S288C_Ator08_hmg124h_3.JPG.dat	1015.5	7.0	1.0	0.01	0.040	0.007	1.1E-07
YDR384C	YDR384C	S288C_Ator08_hmg124h_3.JPG.dat	953.5	49.3	1.0	0.02	-0.008	0.015	8.5E-05
YDR385W	YDR385W	S288C_Ator08_hmg124h_3.JPG.dat	1030.0	10.7	1.0	0.01	0.067	0.014	6.8E-07
YDR386W	YDR386W	S288C_Ator08_hmg124h_3.JPG.dat	1005.3	23.7	1.0	0.01	0.048	0.007	1.6E-05
YDR387C	YDR387C	S288C_Ator08_hmg124h_3.JPG.dat	980.8	40.3	1.0	0.04	-0.055	0.038	1.8E-05
YDR388W	YDR388W	S288C_Ator08_hmg124h_3.JPG.dat	968.8	50.4	1.0	0.05	-0.017	0.052	3.6E-05
YDR389W	YDR389W	S288C_Ator08_hmg124h_3.JPG.dat	975.3	64.1	1.0	0.07	-0.020	0.065	8.6E-05
YDR391C	YDR391C	S288C_Ator08_hmg124h_3.JPG.dat	1086.8	14.9	1.0	0.01	0.034	0.013	5.4E-07
YDR392W	YDR392W	S288C_Ator08_hmg124h_3.JPG.dat	943.3	107.4	0.9	0.02	0.002	0.024	2.6E-04
YDR393W	YDR393W	S288C_Ator08_hmg124h_3.JPG.dat	917.8	54.4	0.9	0.01	0.022	0.014	7.6E-05
YDR395W	YDR395W	S288C_Ator08_hmg124h_3.JPG.dat	992.0	21.1	1.0	0.02	0.017	0.020	2.3E-06
YDR399W	YDR399W	S288C_Ator08_hmg124h_3.JPG.dat	1000.5	13.2	1.0	0.02	0.009	0.015	1.0E-06
YDR400W	YDR400W	S288C_Ator08_hmg124h_3.JPG.dat	1041.3	23.4	1.0	0.02	0.031	0.024	3.4E-06
YDR401W	YDR401W	S288C_Ator08_hmg124h_3.JPG.dat	1026.5	5.8	1.0	0.01	0.009	0.008	1.1E-07
YDR402C	YDR402C	S288C_Ator08_hmg124h_3.JPG.dat	1007.8	28.9	1.0	0.01	-0.009	0.011	3.8E-05
YDR403W	YDR403W	S288C_Ator08_hmg124h_3.JPG.dat	1065.8	49.6	1.0	0.05	-0.019	0.045	2.3E-05
YDR406W	YDR406W	S288C_Ator08_hmg124h_3.JPG.dat	1040.3	51.6	1.1	0.05	0.010	0.048	2.6E-05
YDR408C	YDR408C	S288C_Ator08_hmg124h_3.JPG.dat	1097.8	37.3	1.0	0.04	0.057	0.038	1.3E-05
YDR409W	YDR409W	S288C_Ator08_hmg124h_3.JPG.dat	1126.5	29.6	1.1	0.03	0.048	0.028	5.1E-06
YDR410C	YDR410C	S288C_Ator08_hmg124h_3.JPG.dat	0.0	0.0	0.0	0.00	0.000	0.000	
YDR411C	YDR411C	S288C_Ator08_hmg124h_3.JPG.dat	1063.5	19.0	1.0	0.00	0.037	0.005	6.5E-06
YDR414C	YDR414C	S288C_Ator08_hmg124h_3.JPG.dat	1023.0	35.1	1.0	0.03	-0.033	0.033	9.8E-06
YDR415C	YDR415C	S288C_Ator08_hmg124h_3.JPG.dat	976.5	29.1	1.0	0.03	-0.018	0.029	7.5E-06
YDR419W	YDR419W	S288C_Ator08_hmg124h_3.JPG.dat	1021.3	10.7	1.0	0.00	0.007	0.001	7.4E-07
YDR420W	YDR420W	S288C_Ator08_hmg124h_3.JPG.dat	958.0	27.0	0.9	0.03	-0.091	0.028	7.8E-06
YDR421W	YDR421W	S288C_Ator08_hmg124h_3.JPG.dat	1049.0	36.6	1.0	0.04	0.004	0.036	1.2E-05
YDR422C	YDR422C	S288C_Ator08_hmg124h_3.JPG.dat	1080.0	49.1	1.0	0.05	0.020	0.048	2.9E-05

YOR202W	YOR202W	S288C_Ator08_hmg124h_3.JPG.dat	1041.5	141.1	1.0	0.09	0.002	0.091	1.8E-289
1	1	S288C_Ator08_hmg124h_8.JPG.dat	1267.8	126.3	1.0	0.07	-0.171	0.069	8.6E-05
2	2	S288C_Ator08_hmg124h_8.JPG.dat	1100.8	103.4	0.9	0.07	0.037	0.073	1.3E-04
3	3	S288C_Ator08_hmg124h_8.JPG.dat	1105.8	54.8	1.0	0.04	0.028	0.038	1.6E-05
4	4	S288C_Ator08_hmg124h_8.JPG.dat	1107.0	44.0	1.0	0.03	-0.018	0.032	8.6E-06
YJR100C	YJR100C	S288C_Ator08_hmg124h_8.JPG.dat	1159.3	23.2	1.0	0.02	0.006	0.018	1.4E-06
YJR103W	YJR103W	S288C_Ator08_hmg124h_8.JPG.dat	1174.8	30.3	1.0	0.00	0.032	0.004	4.1E-06
YJR106W	YJR106W	S288C_Ator08_hmg124h_8.JPG.dat	1171.0	16.8	1.0	0.02	0.008	0.018	1.4E-06
YJR107W	YJR107W	S288C_Ator08_hmg124h_8.JPG.dat	1169.5	48.0	1.0	0.04	0.014	0.041	1.7E-05
YJR108W	YJR108W	S288C_Ator08_hmg124h_8.JPG.dat	987.3	81.2	0.9	0.07	-0.111	0.070	1.5E-04
YJR109C	YJR109C	S288C_Ator08_hmg124h_8.JPG.dat	0.0	0.0	0.0	0.00	0.000	0.000	
YJR110W	YJR110W	S288C_Ator08_hmg124h_8.JPG.dat	1074.0	19.3	0.9	0.02	-0.033	0.019	2.4E-06
YJR111C	YJR111C	S288C_Ator08_hmg124h_8.JPG.dat	1129.5	41.0	1.0	0.04	-0.006	0.043	2.2E-05
YJR115W	YJR115W	S288C_Ator08_hmg124h_8.JPG.dat	1178.0	12.2	1.0	0.00	0.007	0.004	4.4E-06
YJR116W	YJR116W	S288C_Ator08_hmg124h_8.JPG.dat	1153.5	35.3	1.0	0.00	0.007	0.003	3.0E-06
YJR117W	YJR117W	S288C_Ator08_hmg124h_8.JPG.dat	1063.5	18.3	0.9	0.01	-0.018	0.011	4.6E-07
YJR118C	YJR118C	S288C_Ator08_hmg124h_8.JPG.dat	996.3	39.4	0.9	0.04	0.018	0.038	2.1E-05
YJR119C	YJR119C	S288C_Ator08_hmg124h_8.JPG.dat	1144.5	42.2	1.0	0.04	-0.005	0.038	1.4E-05
YJR120W	YJR120W	S288C_Ator08_hmg124h_8.JPG.dat	1121.3	32.0	1.0	0.03	0.044	0.032	8.4E-06
YJR121W	YJR121W	S288C_Ator08_hmg124h_8.JPG.dat	1133.8	83.0	1.1	0.08	0.028	0.079	1.2E-04
YJR124C	YJR124C	S288C_Ator08_hmg124h_8.JPG.dat	1070.0	71.1	1.0	0.07	-0.005	0.065	7.7E-05
YJR125C	YJR125C	S288C_Ator08_hmg124h_8.JPG.dat	1025.0	61.2	0.9	0.06	-0.022	0.057	5.9E-05
YJR126C	YJR126C	S288C_Ator08_hmg124h_8.JPG.dat	985.5	22.5	0.9	0.02	-0.050	0.018	2.0E-06
YJR127C	YJR127C	S288C_Ator08_hmg124h_8.JPG.dat	1103.5	26.0	1.0	0.02	0.014	0.019	1.9E-06
YJR128W	YJR128W	S288C_Ator08_hmg124h_8.JPG.dat	1182.5	52.2	1.1	0.05	0.019	0.045	2.2E-05
YJR129C	YJR129C	S288C_Ator08_hmg124h_8.JPG.dat	1024.8	110.6	0.9	0.10	-0.070	0.098	3.2E-04
YJR130C	YJR130C	S288C_Ator08_hmg124h_8.JPG.dat	1116.3	60.4	1.0	0.06	-0.002	0.057	4.8E-05
YJR131W	YJR131W	S288C_Ator08_hmg124h_8.JPG.dat	1028.3	87.9	0.9	0.08	-0.059	0.080	1.7E-04
YJR133W	YJR133W	S288C_Ator08_hmg124h_8.JPG.dat	1052.5	37.8	1.0	0.03	-0.025	0.034	1.2E-05
YJR134C	YJR134C	S288C_Ator08_hmg124h_8.JPG.dat	1077.8	70.0	1.0	0.07	0.018	0.067	8.2E-05
YJR135C	YJR135C	S288C_Ator08_hmg124h_8.JPG.dat	1093.8	34.6	1.0	0.03	0.011	0.031	7.7E-06
YJR137C	YJR137C	S288C_Ator08_hmg124h_8.JPG.dat	1041.8	80.8	1.0	0.08	-0.038	0.077	1.4E-04
YJR139C	YJR139C	S288C_Ator08_hmg124h_8.JPG.dat	977.8	90.6	0.9	0.03	-0.002	0.025	2.4E-04
YJR140C	YJR140C	S288C_Ator08_hmg124h_8.JPG.dat	1011.0	64.8	1.0	0.01	0.003	0.008	2.4E-05
YJR142W	YJR142W	S288C_Ator08_hmg124h_8.JPG.dat	1053.0	61.2	0.9	0.06	-0.055	0.060	6.8E-05
YJR145C	YJR145C	S288C_Ator08_hmg124h_8.JPG.dat	978.5	87.4	0.9	0.08	-0.090	0.076	1.9E-04
YJR146W	YJR146W	S288C_Ator08_hmg124h_8.JPG.dat	1147.0	25.5	1.0	0.02	-0.014	0.022	2.7E-06
YJR147W	YJR147W	S288C_Ator08_hmg124h_8.JPG.dat	1123.3	12.6	1.0	0.01	-0.010	0.011	3.2E-07
YJR148W	YJR148W	S288C_Ator08_hmg124h_8.JPG.dat	1060.3	35.9	1.0	0.01	-0.047	0.006	1.4E-05
YJR149W	YJR149W	S288C_Ator08_hmg124h_8.JPG.dat	1023.0	50.7	0.9	0.05	-0.042	0.046	3.1E-05
YJR150C	YJR150C	S288C_Ator08_hmg124h_8.JPG.dat	993.8	134.4	0.9	0.13	-0.079	0.129	7.0E-04
YJR152W	YJR152W	S288C_Ator08_hmg124h_8.JPG.dat	1087.0	39.2	1.0	0.04	0.005	0.035	1.1E-05
YJR153W	YJR153W	S288C_Ator08_hmg124h_8.JPG.dat	1071.8	109.0	1.0	0.03	0.046	0.032	3.1E-04
YJR154W	YJR154W	S288C_Ator08_hmg124h_8.JPG.dat	1152.8	23.6	1.0	0.02	0.014	0.021	2.3E-06
YKL001C	YKL001C	S288C_Ator08_hmg124h_8.JPG.dat	1137.0	16.8	1.0	0.01	0.030	0.013	5.8E-07
YKL005C	YKL005C	S288C_Ator08_hmg124h_8.JPG.dat	1150.8	22.6	1.0	0.02	0.016	0.020	2.2E-06
YKL006W	YKL006W	S288C_Ator08_hmg124h_8.JPG.dat	569.8	468.1	0.5	0.41	-0.274	0.411	9.3E-02
YKL007W	YKL007W	S288C_Ator08_hmg124h_8.JPG.dat	1089.8	20.5	1.0	0.02	-0.046	0.023	3.4E-06
YKL008C	YKL008C	S288C_Ator08_hmg124h_8.JPG.dat	1116.5	41.8	1.0	0.01	-0.018	0.012	4.6E-05
YKL009W	YKL009W	S288C_Ator08_hmg124h_8.JPG.dat	911.8	32.3	0.9	0.00	-0.088	0.004	7.8E-06
YKL010C	YKL010C	S288C_Ator08_hmg124h_8.JPG.dat	1160.0	5.1	1.1	0.00	0.036	0.002	1.1E-06
YKL015W	YKL015W	S288C_Ator08_hmg124h_8.JPG.dat	1106.3	29.4	1.0	0.03	0.032	0.030	6.8E-06
YKL017C	YKL017C	S288C_Ator08_hmg124h_8.JPG.dat	1118.0	21.6	1.0	0.03	0.008	0.025	3.9E-06
YKL020C	YKL020C	S288C_Ator08_hmg124h_8.JPG.dat	1094.3	70.0	1.0	0.06	0.007	0.058	5.5E-05
YKL023W	YKL023W	S288C_Ator08_hmg124h_8.JPG.dat	951.0	191.5	0.8	0.17	-0.126	0.170	2.3E-03
YKL025C	YKL025C	S288C_Ator08_hmg124h_8.JPG.dat	1120.0	24.9	1.0	0.00	-0.045	0.002	1.1E-06
YKL026C	YKL026C	S288C_Ator08_hmg124h_8.JPG.dat	1120.8	122.2	1.0	0.11	-0.035	0.107	3.6E-04
YKL027W	YKL027W	S288C_Ator08_hmg124h_8.JPG.dat	1154.8	43.7	1.0	0.01	0.046	0.010	3.1E-05
YKL029C	YKL029C	S288C_Ator08_hmg124h_8.JPG.dat	1042.8	19.0	1.0	0.01	-0.034	0.013	7.4E-07
YKL030W	YKL030W	S288C_Ator08_hmg124h_8.JPG.dat	1093.8	14.5	1.0	0.01	-0.016	0.012	4.4E-07
YKL031W	YKL031W	S288C_Ator08_hmg124h_8.JPG.dat	1207.0	6.8	1.1	0.00	0.029	0.002	1.2E-06
YKL032C	YKL032C	S288C_Ator08_hmg124h_8.JPG.dat	1220.5	16.7	1.1	0.00	0.144	0.003	2.3E-06
YKL033W-/	YKL033W-A	S288C_Ator08_hmg124h_8.JPG.dat	1085.5	37.2	1.0	0.03	-0.037	0.034	1.2E-05
YKL034W	YKL034W	S288C_Ator08_hmg124h_8.JPG.dat	1132.3	52.5	1.0	0.05	0.002	0.047	2.8E-05
YKL037W	YKL037W	S288C_Ator08_hmg124h_8.JPG.dat	1080.5	11.6	1.0	0.01	0.012	0.010	3.0E-07
YKL038W	YKL038W	S288C_Ator08_hmg124h_8.JPG.dat	1129.8	45.8	1.0	0.01	0.002	0.007	1.9E-05
YKL039W	YKL039W	S288C_Ator08_hmg124h_8.JPG.dat	1174.5	13.5	1.0	0.01	-0.001	0.010	2.5E-07
YKL040C	YKL040C	S288C_Ator08_hmg124h_8.JPG.dat	1194.0	59.1	1.0	0.05	-0.001	0.050	3.4E-05
YKL041W	YKL041W	S288C_Ator08_hmg124h_8.JPG.dat	445.3	525.1	0.4	0.45	-0.114	0.454	1.9E-01
YKL043W	YKL043W	S288C_Ator08_hmg124h_8.JPG.dat	1126.5	12.9	1.0	0.01	0.019	0.007	9.3E-08

YKL044W	YKL044W	S288C_Ator08_hmg124h_8.JPG.dat	1061.5	7.4	1.0	0.01	-0.051	0.007	9.8E-08
YKL046C	YKL046C	S288C_Ator08_hmg124h_8.JPG.dat	1001.0	43.2	0.8	0.04	-0.087	0.043	3.7E-05
YKL047W	YKL047W	S288C_Ator08_hmg124h_8.JPG.dat	1211.3	63.0	1.0	0.05	0.019	0.046	2.4E-05
YKL048C	YKL048C	S288C_Ator08_hmg124h_8.JPG.dat	1120.8	53.1	1.0	0.05	-0.086	0.050	3.6E-05
YKL050C	YKL050C	S288C_Ator08_hmg124h_8.JPG.dat	1155.8	35.6	1.0	0.04	-0.050	0.036	1.2E-05
YKL051W	YKL051W	S288C_Ator08_hmg124h_8.JPG.dat	1170.5	44.0	1.0	0.01	-0.024	0.008	1.9E-05
YKL053C-A	YKL053C-A	S288C_Ator08_hmg124h_8.JPG.dat	1190.8	41.8	1.0	0.04	0.013	0.037	1.4E-05
YKL053W	YKL053W	S288C_Ator08_hmg124h_8.JPG.dat	1223.3	33.5	1.0	0.03	-0.015	0.027	4.7E-06
YKL055C	YKL055C	S288C_Ator08_hmg124h_8.JPG.dat	1080.5	150.6	0.9	0.13	0.029	0.128	7.5E-04
YKL056C	YKL056C	S288C_Ator08_hmg124h_8.JPG.dat	1050.5	179.8	0.9	0.15	-0.059	0.152	1.3E-03
YKL061W	YKL061W	S288C_Ator08_hmg124h_8.JPG.dat	1123.5	22.2	1.0	0.01	0.015	0.012	5.2E-07
YKL062W	YKL062W	S288C_Ator08_hmg124h_8.JPG.dat	1092.3	36.2	1.0	0.03	0.100	0.031	8.6E-06
YKL063C	YKL063C	S288C_Ator08_hmg124h_8.JPG.dat	1114.5	53.7	1.0	0.04	-0.019	0.045	2.5E-05
YKL064W	YKL064W	S288C_Ator08_hmg124h_8.JPG.dat	1141.5	33.8	1.0	0.03	0.012	0.034	1.1E-05
YKL065C	YKL065C	S288C_Ator08_hmg124h_8.JPG.dat	1085.5	9.3	1.0	0.01	-0.012	0.012	5.8E-07
YKL066W	YKL066W	S288C_Ator08_hmg124h_8.JPG.dat	1159.3	22.7	1.0	0.02	0.006	0.020	2.0E-06
YKL067W	YKL067W	S288C_Ator08_hmg124h_8.JPG.dat	1027.0	159.4	0.9	0.14	-0.089	0.139	9.9E-04
YKL068W	YKL068W	S288C_Ator08_hmg124h_8.JPG.dat	1225.3	62.3	1.1	0.05	0.030	0.047	2.5E-05
YKL069W	YKL069W	S288C_Ator08_hmg124h_8.JPG.dat	887.3	104.2	0.8	0.03	0.001	0.032	5.1E-04
YKL070W	YKL070W	S288C_Ator08_hmg124h_8.JPG.dat	1059.5	126.5	1.0	0.03	-0.019	0.028	2.6E-04
YKL071W	YKL071W	S288C_Ator08_hmg124h_8.JPG.dat	1167.5	7.9	1.0	0.01	0.005	0.013	5.8E-07
YKL072W	YKL072W	S288C_Ator08_hmg124h_8.JPG.dat	1207.8	61.6	1.0	0.05	0.029	0.053	3.6E-05
YKL073W	YKL073W	S288C_Ator08_hmg124h_8.JPG.dat	1187.5	39.4	1.1	0.04	-0.010	0.040	1.5E-05
YKL074C	YKL074C	S288C_Ator08_hmg124h_8.JPG.dat	1052.0	30.9	1.0	0.01	0.010	0.008	2.3E-05
YKL075C	YKL075C	S288C_Ator08_hmg124h_8.JPG.dat	1120.8	16.3	1.0	0.01	0.042	0.012	4.1E-07
YKL076C	YKL076C	S288C_Ator08_hmg124h_8.JPG.dat	1169.0	57.9	1.1	0.06	0.100	0.059	4.6E-05
YKL077W	YKL077W	S288C_Ator08_hmg124h_8.JPG.dat	1154.0	81.8	1.0	0.01	0.000	0.010	3.5E-05
YKL079W	YKL079W	S288C_Ator08_hmg124h_8.JPG.dat	1099.0	53.4	1.0	0.05	-0.024	0.050	3.3E-05
YKL081W	YKL081W	S288C_Ator08_hmg124h_8.JPG.dat	663.3	37.5	0.6	0.04	-0.051	0.038	6.6E-05
YKL084W	YKL084W	S288C_Ator08_hmg124h_8.JPG.dat	965.5	41.4	0.9	0.04	-0.086	0.041	2.6E-05
YKL085W	YKL085W	S288C_Ator08_hmg124h_8.JPG.dat	1081.8	5.9	1.0	0.01	0.006	0.009	1.9E-07
YKL086W	YKL086W	S288C_Ator08_hmg124h_8.JPG.dat	1147.5	16.4	1.0	0.00	-0.007	0.005	6.7E-06
YKL090W	YKL090W	S288C_Ator08_hmg124h_8.JPG.dat	1073.5	64.7	1.0	0.01	-0.040	0.011	4.1E-05
YKL091C	YKL091C	S288C_Ator08_hmg124h_8.JPG.dat	1089.0	53.4	1.0	0.01	0.012	0.014	6.2E-05
YKL092C	YKL092C	S288C_Ator08_hmg124h_8.JPG.dat	1149.3	16.8	1.1	0.01	0.003	0.012	3.8E-07
YKL093W	YKL093W	S288C_Ator08_hmg124h_8.JPG.dat	1115.3	8.7	1.0	0.01	0.046	0.008	1.3E-07
YKL094W	YKL094W	S288C_Ator08_hmg124h_8.JPG.dat	1138.0	5.4	1.0	0.00	0.022	0.003	8.4E-09
YKL096W	YKL096W	S288C_Ator08_hmg124h_8.JPG.dat	1111.8	35.5	1.0	0.03	0.048	0.035	1.0E-05
YKL096W-/	YKL096W-A	S288C_Ator08_hmg124h_8.JPG.dat	1068.5	47.0	1.0	0.02	0.005	0.015	7.3E-05
YKL097C	YKL097C	S288C_Ator08_hmg124h_8.JPG.dat	1064.0	29.7	1.0	0.03	-0.035	0.027	5.9E-06
YKL098W	YKL098W	S288C_Ator08_hmg124h_8.JPG.dat	1097.8	4.6	1.0	0.01	0.027	0.008	1.2E-07
YKL100C	YKL100C	S288C_Ator08_hmg124h_8.JPG.dat	1118.3	8.2	1.0	0.00	0.032	0.002	1.1E-06
YKL101W	YKL101W	S288C_Ator08_hmg124h_8.JPG.dat	1074.8	76.3	1.0	0.07	-0.097	0.069	1.0E-04
YKL102C	YKL102C	S288C_Ator08_hmg124h_8.JPG.dat	1145.3	8.9	1.0	0.01	-0.005	0.009	1.8E-07
YKL103C	YKL103C	S288C_Ator08_hmg124h_8.JPG.dat	1126.3	13.1	1.0	0.00	-0.002	0.003	2.1E-06
YKL105C	YKL105C	S288C_Ator08_hmg124h_8.JPG.dat	1112.5	39.2	1.0	0.04	0.000	0.036	1.1E-05
YKL106W	YKL106W	S288C_Ator08_hmg124h_8.JPG.dat	1089.8	25.6	1.0	0.02	0.002	0.024	3.9E-06
YKL107W	YKL107W	S288C_Ator08_hmg124h_8.JPG.dat	1010.8	30.6	0.9	0.03	-0.041	0.026	5.8E-06
YKL109W	YKL109W	S288C_Ator08_hmg124h_8.JPG.dat	1009.5	153.2	1.0	0.15	-0.010	0.146	9.7E-04
YKL110C	YKL110C	S288C_Ator08_hmg124h_8.JPG.dat	960.8	42.5	0.9	0.04	-0.009	0.038	2.0E-05
YKL113C	YKL113C	S288C_Ator08_hmg124h_8.JPG.dat	878.0	156.6	0.7	0.04	-0.240	0.041	1.0E-03
YKL114C	YKL114C	S288C_Ator08_hmg124h_8.JPG.dat	1069.3	55.2	1.0	0.05	-0.002	0.050	4.0E-05
YKL115C	YKL115C	S288C_Ator08_hmg124h_8.JPG.dat	1119.0	34.4	1.0	0.03	-0.003	0.032	8.9E-06
YKL116C	YKL116C	S288C_Ator08_hmg124h_8.JPG.dat	1175.3	15.2	1.0	0.01	0.036	0.013	5.8E-07
YKL117W	YKL117W	S288C_Ator08_hmg124h_8.JPG.dat	1177.8	39.2	1.0	0.03	0.033	0.031	7.3E-06
YKL120W	YKL120W	S288C_Ator08_hmg124h_8.JPG.dat	1124.0	13.0	1.0	0.02	-0.005	0.016	1.0E-06
YKL121W	YKL121W	S288C_Ator08_hmg124h_8.JPG.dat	1057.8	26.8	1.0	0.03	-0.027	0.032	8.9E-06
YKL123W	YKL123W	S288C_Ator08_hmg124h_8.JPG.dat	1119.8	43.8	1.0	0.04	0.008	0.041	1.8E-05
YKL124W	YKL124W	S288C_Ator08_hmg124h_8.JPG.dat	1089.0	23.4	1.0	0.02	0.004	0.023	3.1E-06
YKL127W	YKL127W	S288C_Ator08_hmg124h_8.JPG.dat	1054.5	44.2	1.0	0.04	-0.010	0.037	1.4E-05
YKL128C	YKL128C	S288C_Ator08_hmg124h_8.JPG.dat	1096.0	14.5	1.0	0.01	0.011	0.015	8.4E-07
YKL129C	YKL129C	S288C_Ator08_hmg124h_8.JPG.dat	1118.8	64.8	1.0	0.05	0.013	0.049	3.5E-05
YKL130C	YKL130C	S288C_Ator08_hmg124h_8.JPG.dat	1144.5	10.7	1.0	0.01	0.004	0.007	1.0E-07
YKL131W	YKL131W	S288C_Ator08_hmg124h_8.JPG.dat	1145.0	35.6	1.0	0.03	0.010	0.035	1.1E-05
YKL132C	YKL132C	S288C_Ator08_hmg124h_8.JPG.dat	1177.8	45.6	1.0	0.01	0.028	0.013	5.1E-05
YKL133C	YKL133C	S288C_Ator08_hmg124h_8.JPG.dat	1161.8	27.0	1.0	0.02	0.016	0.018	1.5E-06
YKL136W	YKL136W	S288C_Ator08_hmg124h_8.JPG.dat	926.0	41.6	0.9	0.04	-0.101	0.038	2.4E-05
YKL137W	YKL137W	S288C_Ator08_hmg124h_8.JPG.dat	1070.3	33.4	1.0	0.00	-0.040	0.005	8.6E-06
YKL139W	YKL139W	S288C_Ator08_hmg124h_8.JPG.dat	1128.8	28.5	1.0	0.03	-0.003	0.026	4.7E-06
YKL140W	YKL140W	S288C_Ator08_hmg124h_8.JPG.dat	1080.5	87.9	1.0	0.02	-0.009	0.020	1.4E-04

YKL142W	YKL142W	S288C_Ator08_hmg124h_8.JPG.dat	1152.3	44.3	1.0	0.04	-0.016	0.040	1.6E-05
YKL146W	YKL146W	S288C_Ator08_hmg124h_8.JPG.dat	1131.8	7.8	1.0	0.01	0.021	0.007	8.2E-08
YKL147C	YKL147C	S288C_Ator08_hmg124h_8.JPG.dat	1154.3	31.4	1.0	0.03	0.000	0.025	4.7E-06
YKL148C	YKL148C	S288C_Ator08_hmg124h_8.JPG.dat	1175.8	29.2	1.0	0.02	0.026	0.025	4.0E-06
YKL149C	YKL149C	S288C_Ator08_hmg124h_8.JPG.dat	1168.5	24.8	1.0	0.02	-0.005	0.020	2.4E-06
YKL150W	YKL150W	S288C_Ator08_hmg124h_8.JPG.dat	1211.0	40.3	1.0	0.04	0.007	0.039	1.5E-05
YKL151C	YKL151C	S288C_Ator08_hmg124h_8.JPG.dat	1180.8	27.5	1.0	0.02	0.033	0.023	3.0E-06
YKL156W	YKL156W	S288C_Ator08_hmg124h_8.JPG.dat	978.8	31.7	0.9	0.03	-0.041	0.030	9.6E-06
YKL157W	YKL157W	S288C_Ator08_hmg124h_8.JPG.dat	1145.0	88.4	0.9	0.07	-0.031	0.071	1.2E-04
YKL158W	YKL158W	S288C_Ator08_hmg124h_8.JPG.dat	1099.5	123.6	0.9	0.10	0.021	0.097	3.1E-04
YKL159C	YKL159C	S288C_Ator08_hmg124h_8.JPG.dat	1052.3	55.8	0.9	0.05	-0.105	0.047	3.8E-05
YKL160W	YKL160W	S288C_Ator08_hmg124h_8.JPG.dat	1176.3	72.9	1.0	0.07	0.014	0.069	8.2E-05
YKL161C	YKL161C	S288C_Ator08_hmg124h_8.JPG.dat	1201.5	21.8	1.0	0.02	0.000	0.017	1.2E-06
YKL162C	YKL162C	S288C_Ator08_hmg124h_8.JPG.dat	1224.0	38.6	1.0	0.01	-0.010	0.008	2.0E-05
YKL163W	YKL163W	S288C_Ator08_hmg124h_8.JPG.dat	1257.3	17.2	1.1	0.02	0.066	0.016	9.3E-07
YKL164C	YKL164C	S288C_Ator08_hmg124h_8.JPG.dat	1189.3	37.3	1.0	0.03	0.004	0.034	1.0E-05
YKL166C	YKL166C	S288C_Ator08_hmg124h_8.JPG.dat	1185.5	27.7	1.0	0.02	0.020	0.023	3.1E-06
YKL167C	YKL167C	S288C_Ator08_hmg124h_8.JPG.dat	1147.0	91.4	1.0	0.07	0.030	0.072	1.1E-04
YKL168C	YKL168C	S288C_Ator08_hmg124h_8.JPG.dat	1089.0	41.1	1.0	0.03	0.013	0.030	7.8E-06
YKL171W	YKL171W	S288C_Ator08_hmg124h_8.JPG.dat	1094.3	49.7	1.0	0.03	-0.046	0.033	1.2E-05
YKL174C	YKL174C	S288C_Ator08_hmg124h_8.JPG.dat	1177.0	65.1	1.0	0.04	0.033	0.042	1.9E-05
YKL175W	YKL175W	S288C_Ator08_hmg124h_8.JPG.dat	1142.3	13.4	1.0	0.03	0.055	0.027	5.4E-06
YKL176C	YKL176C	S288C_Ator08_hmg124h_8.JPG.dat	1113.5	54.4	1.0	0.01	0.048	0.012	4.9E-05
YKL177W	YKL177W	S288C_Ator08_hmg124h_8.JPG.dat	1209.8	24.1	1.0	0.03	0.038	0.032	8.1E-06
YKL178C	YKL178C	S288C_Ator08_hmg124h_8.JPG.dat	1276.8	32.6	1.1	0.04	0.023	0.045	2.0E-05
YKL179C	YKL179C	S288C_Ator08_hmg124h_8.JPG.dat	1269.8	27.3	1.1	0.01	0.033	0.009	1.4E-07
YKL183W	YKL183W	S288C_Ator08_hmg124h_8.JPG.dat	1208.3	31.1	1.0	0.03	0.006	0.032	8.0E-06
YKL184W	YKL184W	S288C_Ator08_hmg124h_8.JPG.dat	349.8	20.5	0.3	0.02	-0.064	0.018	6.1E-05
YKL185W	YKL185W	S288C_Ator08_hmg124h_8.JPG.dat	1091.8	77.4	0.9	0.06	-0.097	0.058	6.3E-05
YKL187C	YKL187C	S288C_Ator08_hmg124h_8.JPG.dat	1161.0	31.4	1.0	0.00	0.042	0.003	2.1E-06
YKL188C	YKL188C	S288C_Ator08_hmg124h_8.JPG.dat	1139.5	19.1	1.0	0.02	0.023	0.019	1.8E-06
YKL190W	YKL190W	S288C_Ator08_hmg124h_8.JPG.dat	964.8	39.4	0.9	0.04	-0.127	0.035	1.9E-05
YKL191W	YKL191W	S288C_Ator08_hmg124h_8.JPG.dat	1018.3	50.2	1.0	0.00	0.000	0.002	9.3E-07
YKL197C	YKL197C	S288C_Ator08_hmg124h_8.JPG.dat	1079.3	29.8	1.0	0.03	0.012	0.028	6.5E-06
YKL198C	YKL198C	S288C_Ator08_hmg124h_8.JPG.dat	1070.5	55.2	1.0	0.05	-0.021	0.052	4.0E-05
YKL199C	YKL199C	S288C_Ator08_hmg124h_8.JPG.dat	1110.5	56.5	1.0	0.04	-0.011	0.042	2.0E-05
YKL200C	YKL200C	S288C_Ator08_hmg124h_8.JPG.dat	1072.5	51.1	1.0	0.01	-0.050	0.012	4.6E-05
YKL201C	YKL201C	S288C_Ator08_hmg124h_8.JPG.dat	1124.0	36.7	1.0	0.01	0.011	0.011	3.6E-05
YKL202W	YKL202W	S288C_Ator08_hmg124h_8.JPG.dat	1163.5	13.3	1.0	0.01	-0.005	0.014	6.8E-07
YKL204W	YKL204W	S288C_Ator08_hmg124h_8.JPG.dat	536.3	623.1	0.5	0.57	-0.010	0.566	1.8E-01
YKL205W	YKL205W	S288C_Ator08_hmg124h_8.JPG.dat	1190.5	51.6	1.1	0.05	0.051	0.046	2.0E-05
YKL206C	YKL206C	S288C_Ator08_hmg124h_8.JPG.dat	1156.5	29.0	1.1	0.01	0.039	0.010	2.9E-05
YKL207W	YKL207W	S288C_Ator08_hmg124h_8.JPG.dat	1070.0	29.8	1.0	0.02	-0.043	0.019	2.0E-06
YKL208W	YKL208W	S288C_Ator08_hmg124h_8.JPG.dat	1115.3	15.5	1.0	0.01	0.041	0.011	3.0E-07
YKL211C	YKL211C	S288C_Ator08_hmg124h_8.JPG.dat	1020.5	58.6	0.9	0.04	-0.067	0.042	2.3E-05
YKL212W	YKL212W	S288C_Ator08_hmg124h_8.JPG.dat	0.0	0.0	0.0	0.00	0.000	0.000	
YKL213C	YKL213C	S288C_Ator08_hmg124h_8.JPG.dat	1106.5	10.9	1.0	0.00	0.001	0.003	3.7E-06
YKL214C	YKL214C	S288C_Ator08_hmg124h_8.JPG.dat	954.8	103.7	0.9	0.10	-0.090	0.104	4.5E-04
YKL215C	YKL215C	S288C_Ator08_hmg124h_8.JPG.dat	1093.8	30.3	1.0	0.03	-0.009	0.028	6.6E-06
YKL216W	YKL216W	S288C_Ator08_hmg124h_8.JPG.dat	1158.0	92.5	1.0	0.09	-0.014	0.092	2.0E-04
YKL217W	YKL217W	S288C_Ator08_hmg124h_8.JPG.dat	1070.3	61.1	1.0	0.05	-0.028	0.049	3.8E-05
YKL218C	YKL218C	S288C_Ator08_hmg124h_8.JPG.dat	1099.5	44.8	1.0	0.04	-0.034	0.042	2.1E-05
YKL220C	YKL220C	S288C_Ator08_hmg124h_8.JPG.dat	1090.0	14.0	1.0	0.01	0.041	0.013	5.0E-07
YKL221W	YKL221W	S288C_Ator08_hmg124h_8.JPG.dat	1065.5	71.7	1.0	0.07	0.019	0.068	8.3E-05
YKL222C	YKL222C	S288C_Ator08_hmg124h_8.JPG.dat	1053.0	30.3	1.0	0.01	0.015	0.014	6.9E-07
YKR001C	YKR001C	S288C_Ator08_hmg124h_8.JPG.dat	935.0	39.6	0.9	0.05	-0.082	0.050	4.5E-05
YKR003W	YKR003W	S288C_Ator08_hmg124h_8.JPG.dat	1042.0	39.7	1.0	0.04	0.030	0.040	1.7E-05
YKR005C	YKR005C	S288C_Ator08_hmg124h_8.JPG.dat	1129.8	38.1	1.1	0.04	0.022	0.043	1.8E-05
YKR007W	YKR007W	S288C_Ator08_hmg124h_8.JPG.dat	1005.5	57.4	0.9	0.02	0.045	0.015	9.1E-05
YKR009C	YKR009C	S288C_Ator08_hmg124h_8.JPG.dat	1097.3	25.8	1.0	0.01	0.002	0.012	4.6E-07
YKR010C	YKR010C	S288C_Ator08_hmg124h_8.JPG.dat	1009.8	151.2	0.9	0.13	-0.048	0.127	7.0E-04
YKR011C	YKR011C	S288C_Ator08_hmg124h_8.JPG.dat	1116.8	13.5	1.0	0.00	0.019	0.005	7.1E-06
YKR012C	YKR012C	S288C_Ator08_hmg124h_8.JPG.dat	1049.0	68.7	1.0	0.02	-0.070	0.018	1.0E-04
YKR013W	YKR013W	S288C_Ator08_hmg124h_8.JPG.dat	1092.0	31.9	1.0	0.01	0.006	0.007	1.6E-05
YKR014C	YKR014C	S288C_Ator08_hmg124h_8.JPG.dat	1112.8	29.8	1.0	0.02	-0.018	0.023	3.1E-06
YKR015C	YKR015C	S288C_Ator08_hmg124h_8.JPG.dat	988.0	106.9	0.9	0.10	-0.097	0.098	3.2E-04
YKR016W	YKR016W	S288C_Ator08_hmg124h_8.JPG.dat	1082.0	18.7	1.0	0.03	0.043	0.031	8.1E-06
YKR017C	YKR017C	S288C_Ator08_hmg124h_8.JPG.dat	1109.5	17.1	1.0	0.02	0.025	0.022	2.6E-06
YKR018C	YKR018C	S288C_Ator08_hmg124h_8.JPG.dat	1110.3	20.0	1.0	0.00	-0.009	0.002	1.6E-06
YKR019C	YKR019C	S288C_Ator08_hmg124h_8.JPG.dat	902.8	145.6	0.8	0.12	-0.116	0.117	8.4E-04

YKR020W	YKR020W	S288C_Ator08_hmg124h_8.JPG.dat	560.3	80.6	0.5	0.01	-0.332	0.012	2.2E-04
YKR021W	YKR021W	S288C_Ator08_hmg124h_8.JPG.dat	1134.3	48.8	1.0	0.03	0.001	0.031	8.2E-06
YKR023W	YKR023W	S288C_Ator08_hmg124h_8.JPG.dat	773.0	112.1	0.7	0.10	-0.239	0.096	6.8E-04
YKR024C	YKR024C	S288C_Ator08_hmg124h_8.JPG.dat	846.0	170.7	0.8	0.17	-0.177	0.166	2.4E-03
YKR026C	YKR026C	S288C_Ator08_hmg124h_8.JPG.dat	1130.8	24.3	1.1	0.01	0.048	0.007	1.5E-05
YKR027W	YKR027W	S288C_Ator08_hmg124h_8.JPG.dat	1078.3	9.4	1.0	0.01	0.006	0.014	8.2E-07
YKR028W	YKR028W	S288C_Ator08_hmg124h_8.JPG.dat	921.5	71.6	0.9	0.07	-0.066	0.067	1.3E-04
YKR029C	YKR029C	S288C_Ator08_hmg124h_8.JPG.dat	959.5	47.0	0.9	0.04	-0.063	0.039	2.4E-05
YKR030W	YKR030W	S288C_Ator08_hmg124h_8.JPG.dat	1018.8	70.8	0.9	0.06	-0.055	0.060	7.2E-05
YKR031C	YKR031C	S288C_Ator08_hmg124h_8.JPG.dat	1160.0	50.7	1.0	0.01	-0.036	0.010	3.6E-05
YKR032W	YKR032W	S288C_Ator08_hmg124h_8.JPG.dat	1160.8	16.1	1.0	0.02	0.014	0.020	2.1E-06
YKR033C	YKR033C	S288C_Ator08_hmg124h_8.JPG.dat	1033.5	184.6	0.9	0.16	-0.059	0.157	1.4E-03
YKR034W	YKR034W	S288C_Ator08_hmg124h_8.JPG.dat	996.3	198.3	0.9	0.18	-0.097	0.176	2.1E-03
YKR035C	YKR035C	S288C_Ator08_hmg124h_8.JPG.dat	916.5	171.9	0.9	0.17	-0.137	0.169	2.1E-03
YKR035W-A	YKR035W-A	S288C_Ator08_hmg124h_8.JPG.dat	895.0	81.9	0.9	0.07	-0.185	0.075	1.8E-04
YKR036C	YKR036C	S288C_Ator08_hmg124h_8.JPG.dat	1103.0	72.6	1.0	0.05	-0.035	0.049	3.5E-05
YKR039W	YKR039W	S288C_Ator08_hmg124h_8.JPG.dat	1090.5	40.0	1.0	0.04	-0.029	0.041	2.1E-05
YKR040C	YKR040C	S288C_Ator08_hmg124h_8.JPG.dat	1081.0	36.3	1.0	0.03	-0.067	0.029	7.2E-06
YKR041W	YKR041W	S288C_Ator08_hmg124h_8.JPG.dat	1130.8	23.9	1.0	0.02	0.026	0.023	2.9E-06
YKR042W	YKR042W	S288C_Ator08_hmg124h_8.JPG.dat	1128.8	20.2	1.0	0.03	0.037	0.026	4.5E-06
YKR043C	YKR043C	S288C_Ator08_hmg124h_8.JPG.dat	1163.3	32.1	1.0	0.02	0.030	0.018	1.6E-06
YKR044W	YKR044W	S288C_Ator08_hmg124h_8.JPG.dat	1187.3	29.0	1.0	0.01	0.051	0.006	1.2E-05
YKR045C	YKR045C	S288C_Ator08_hmg124h_8.JPG.dat	1169.0	23.5	1.0	0.02	-0.030	0.018	1.7E-06
YKR046C	YKR046C	S288C_Ator08_hmg124h_8.JPG.dat	1030.8	147.0	0.9	0.13	-0.104	0.129	7.8E-04
YKR047W	YKR047W	S288C_Ator08_hmg124h_8.JPG.dat	1098.8	36.9	1.0	0.03	-0.010	0.028	5.7E-06
YKR048C	YKR048C	S288C_Ator08_hmg124h_8.JPG.dat	1084.8	21.8	1.0	0.02	0.009	0.024	3.8E-06
YKR049C	YKR049C	S288C_Ator08_hmg124h_8.JPG.dat	1071.5	49.2	0.9	0.05	0.010	0.047	3.4E-05
YKR050W	YKR050W	S288C_Ator08_hmg124h_8.JPG.dat	1106.3	78.2	1.0	0.01	-0.011	0.015	7.7E-05
YKR051W	YKR051W	S288C_Ator08_hmg124h_8.JPG.dat	1237.0	21.2	1.1	0.02	0.065	0.025	3.4E-06
YKR052C	YKR052C	S288C_Ator08_hmg124h_8.JPG.dat	1234.3	53.9	1.1	0.04	0.042	0.042	1.8E-05
YKR053C	YKR053C	S288C_Ator08_hmg124h_8.JPG.dat	1176.8	41.4	1.0	0.03	-0.016	0.031	8.5E-06
YKR054C	YKR054C	S288C_Ator08_hmg124h_8.JPG.dat	1169.3	52.0	1.0	0.03	-0.047	0.025	5.0E-06
YKR055W	YKR055W	S288C_Ator08_hmg124h_8.JPG.dat	1158.8	40.4	1.0	0.05	-0.026	0.046	2.7E-05
YKR056W	YKR056W	S288C_Ator08_hmg124h_8.JPG.dat	1205.5	60.1	1.0	0.01	-0.009	0.006	1.2E-05
YKR057W	YKR057W	S288C_Ator08_hmg124h_8.JPG.dat	990.3	117.6	0.9	0.09	0.038	0.092	3.4E-04
YKR058W	YKR058W	S288C_Ator08_hmg124h_8.JPG.dat	1053.0	128.7	0.9	0.12	-0.108	0.119	6.4E-04
YKR059W	YKR059W	S288C_Ator08_hmg124h_8.JPG.dat	1085.5	45.8	0.9	0.01	-0.028	0.010	3.9E-05
YKR060W	YKR060W	S288C_Ator08_hmg124h_8.JPG.dat	1075.5	53.5	1.0	0.05	-0.026	0.048	3.1E-05
YKR061W	YKR061W	S288C_Ator08_hmg124h_8.JPG.dat	1138.0	32.4	1.0	0.03	0.012	0.028	5.4E-06
YKR064W	YKR064W	S288C_Ator08_hmg124h_8.JPG.dat	1097.8	34.5	1.0	0.04	0.007	0.035	1.2E-05
YKR065C	YKR065C	S288C_Ator08_hmg124h_8.JPG.dat	1049.5	38.3	1.0	0.03	0.001	0.034	1.2E-05
YKR066C	YKR066C	S288C_Ator08_hmg124h_8.JPG.dat	1063.5	37.1	1.0	0.04	-0.035	0.035	1.3E-05
YKR067W	YKR067W	S288C_Ator08_hmg124h_8.JPG.dat	1177.0	33.3	1.1	0.03	0.068	0.033	8.7E-06
YKR069W	YKR069W	S288C_Ator08_hmg124h_8.JPG.dat	1142.0	54.6	1.1	0.05	-0.020	0.047	2.4E-05
YKR070W	YKR070W	S288C_Ator08_hmg124h_8.JPG.dat	1063.3	30.1	1.0	0.03	-0.018	0.025	4.8E-06
YKR072C	YKR072C	S288C_Ator08_hmg124h_8.JPG.dat	1041.8	27.7	0.9	0.02	-0.003	0.025	5.2E-06
YKR074W	YKR074W	S288C_Ator08_hmg124h_8.JPG.dat	1138.0	18.4	1.0	0.01	0.077	0.014	8.2E-07
YKR076W	YKR076W	S288C_Ator08_hmg124h_8.JPG.dat	1103.5	118.5	1.0	0.11	-0.002	0.109	3.4E-04
YKR077W	YKR077W	S288C_Ator08_hmg124h_8.JPG.dat	1102.8	17.9	1.0	0.00	-0.004	0.003	2.8E-06
YKR078W	YKR078W	S288C_Ator08_hmg124h_8.JPG.dat	1142.8	24.4	1.0	0.01	-0.012	0.009	1.7E-07
YKR080W	YKR080W	S288C_Ator08_hmg124h_8.JPG.dat	1122.3	17.5	1.0	0.02	-0.013	0.021	2.6E-06
YKR082W	YKR082W	S288C_Ator08_hmg124h_8.JPG.dat	900.5	56.7	0.9	0.01	0.033	0.013	7.5E-05
YKR084C	YKR084C	S288C_Ator08_hmg124h_8.JPG.dat	1141.3	51.1	1.1	0.04	0.071	0.037	1.2E-05
YKR087C	YKR087C	S288C_Ator08_hmg124h_8.JPG.dat	1103.8	137.6	1.0	0.13	-0.008	0.129	5.7E-04
YKR088C	YKR088C	S288C_Ator08_hmg124h_8.JPG.dat	1103.3	70.6	1.0	0.06	0.026	0.056	4.6E-05
YKR089C	YKR089C	S288C_Ator08_hmg124h_8.JPG.dat	1084.3	60.0	1.0	0.01	-0.038	0.013	5.9E-05
YKR090W	YKR090W	S288C_Ator08_hmg124h_8.JPG.dat	1171.0	49.6	1.1	0.01	0.029	0.013	4.8E-05
YKR091W	YKR091W	S288C_Ator08_hmg124h_8.JPG.dat	742.3	500.7	0.9	0.08	-0.127	0.081	2.9E-03
YKR092C	YKR092C	S288C_Ator08_hmg124h_8.JPG.dat	1061.0	52.6	1.0	0.04	0.043	0.044	2.5E-05
YKR093W	YKR093W	S288C_Ator08_hmg124h_8.JPG.dat	894.3	87.8	0.9	0.09	-0.105	0.093	3.3E-04
YKR094C	YKR094C	S288C_Ator08_hmg124h_8.JPG.dat	1032.3	40.2	1.0	0.04	0.085	0.039	1.7E-05
YKR095W	YKR095W	S288C_Ator08_hmg124h_8.JPG.dat	921.0	63.2	0.9	0.02	-0.134	0.018	1.4E-04
YKR096W	YKR096W	S288C_Ator08_hmg124h_8.JPG.dat	1010.5	25.5	1.0	0.01	-0.018	0.015	8.9E-07
YKR097W	YKR097W	S288C_Ator08_hmg124h_8.JPG.dat	1054.3	61.1	1.0	0.05	0.002	0.048	2.8E-05
YKR098C	YKR098C	S288C_Ator08_hmg124h_8.JPG.dat	1076.8	119.8	1.1	0.04	0.037	0.037	4.0E-04
YKR099W	YKR099W	S288C_Ator08_hmg124h_8.JPG.dat	939.8	93.1	0.9	0.09	-0.064	0.088	2.7E-04
YKR100C	YKR100C	S288C_Ator08_hmg124h_8.JPG.dat	1126.0	10.9	1.1	0.02	0.026	0.021	2.3E-06
YKR101W	YKR101W	S288C_Ator08_hmg124h_8.JPG.dat	1088.3	39.8	1.0	0.03	0.018	0.033	9.7E-06
YKR102W	YKR102W	S288C_Ator08_hmg124h_8.JPG.dat	1073.3	74.2	1.0	0.07	-0.022	0.066	8.3E-05
YKR103W	YKR103W	S288C_Ator08_hmg124h_8.JPG.dat	1027.5	74.4	0.9	0.01	-0.076	0.011	4.5E-05

YKR105C	YKR105C	S288C_Ator08_hmg124h_8.JPG.dat	575.8	664.8	0.5	0.63	0.033	0.626	1.8E-01
YKR106W	YKR106W	S288C_Ator08_hmg124h_8.JPG.dat	1035.3	28.5	0.9	0.00	-0.060	0.005	8.2E-06
YLL001W	YLL001W	S288C_Ator08_hmg124h_8.JPG.dat	1083.8	15.1	1.0	0.00	-0.005	0.004	4.2E-06
YLL002W	YLL002W	S288C_Ator08_hmg124h_8.JPG.dat	812.0	52.6	0.8	0.06	-0.049	0.061	1.4E-04
YLL005C	YLL005C	S288C_Ator08_hmg124h_8.JPG.dat	1071.3	29.6	1.0	0.02	0.001	0.023	3.5E-06
YLL006W	YLL006W	S288C_Ator08_hmg124h_8.JPG.dat	1140.5	7.2	1.0	0.02	-0.006	0.016	1.2E-06
YLL007C	YLL007C	S288C_Ator08_hmg124h_8.JPG.dat	993.3	68.0	0.9	0.06	-0.041	0.059	7.9E-05
YLL009C	YLL009C	S288C_Ator08_hmg124h_8.JPG.dat	1154.0	24.0	1.0	0.01	0.010	0.011	3.0E-07
YLL010C	YLL010C	S288C_Ator08_hmg124h_8.JPG.dat	1097.0	54.5	1.0	0.01	-0.041	0.011	4.2E-05
YLL012W	YLL012W	S288C_Ator08_hmg124h_8.JPG.dat	1173.0	38.4	1.0	0.02	0.000	0.021	2.1E-06
YLL013C	YLL013C	S288C_Ator08_hmg124h_8.JPG.dat	1022.0	107.0	1.0	0.10	-0.031	0.102	3.3E-04
YLL014W	YLL014W	S288C_Ator08_hmg124h_8.JPG.dat	1048.0	38.8	1.0	0.03	-0.058	0.033	1.0E-05
YLL015W	YLL015W	S288C_Ator08_hmg124h_8.JPG.dat	1146.3	29.5	1.1	0.03	0.033	0.030	6.1E-06
YLL016W	YLL016W	S288C_Ator08_hmg124h_8.JPG.dat	1075.3	66.0	1.0	0.06	-0.014	0.056	4.9E-05
YLL017W	YLL017W	S288C_Ator08_hmg124h_8.JPG.dat	1104.5	38.6	1.0	0.01	0.009	0.015	6.6E-05
YLL019C	YLL019C	S288C_Ator08_hmg124h_8.JPG.dat	1022.3	84.1	0.9	0.03	-0.078	0.027	2.9E-04
YLL020C	YLL020C	S288C_Ator08_hmg124h_8.JPG.dat	1090.3	55.4	1.0	0.04	-0.061	0.042	2.3E-05
YLL021W	YLL021W	S288C_Ator08_hmg124h_8.JPG.dat	1178.5	40.4	1.0	0.03	-0.020	0.031	7.6E-06
YLL023C	YLL023C	S288C_Ator08_hmg124h_8.JPG.dat	1144.5	61.9	1.0	0.06	0.003	0.059	5.7E-05
YLL024C	YLL024C	S288C_Ator08_hmg124h_8.JPG.dat	1163.3	10.2	1.0	0.02	0.030	0.016	1.1E-06
YLL025W	YLL025W	S288C_Ator08_hmg124h_8.JPG.dat	1096.3	138.1	1.0	0.12	-0.030	0.124	5.4E-04
YLL026W	YLL026W	S288C_Ator08_hmg124h_8.JPG.dat	1095.5	40.6	1.1	0.01	0.045	0.006	1.2E-05
YLL028W	YLL028W	S288C_Ator08_hmg124h_8.JPG.dat	1217.5	20.5	1.1	0.03	0.026	0.035	9.4E-06
YLL029W	YLL029W	S288C_Ator08_hmg124h_8.JPG.dat	1111.5	5.3	1.0	0.01	0.020	0.013	5.8E-07
YLL032C	YLL032C	S288C_Ator08_hmg124h_8.JPG.dat	1148.5	34.3	1.0	0.04	-0.015	0.036	1.1E-05
YLL038C	YLL038C	S288C_Ator08_hmg124h_8.JPG.dat	1015.8	72.2	0.9	0.02	-0.046	0.021	1.7E-04
YLL039C	YLL039C	S288C_Ator08_hmg124h_8.JPG.dat	1164.0	11.0	1.0	0.02	0.034	0.016	1.0E-06
YLL040C	YLL040C	S288C_Ator08_hmg124h_8.JPG.dat	1087.5	140.3	1.0	0.03	0.011	0.030	3.0E-04
YLL041C	YLL041C	S288C_Ator08_hmg124h_8.JPG.dat	1129.3	78.8	1.0	0.06	0.002	0.061	6.6E-05
YLL042C	YLL042C	S288C_Ator08_hmg124h_8.JPG.dat	1127.0	67.3	0.9	0.02	-0.049	0.020	1.4E-04
YLL043W	YLL043W	S288C_Ator08_hmg124h_8.JPG.dat	1150.0	129.0	1.0	0.11	-0.035	0.112	3.8E-04
YLL044W	YLL044W	S288C_Ator08_hmg124h_8.JPG.dat	977.3	248.3	1.0	0.00	0.023	0.005	8.4E-06
YLL045C	YLL045C	S288C_Ator08_hmg124h_8.JPG.dat	1017.3	60.7	1.0	0.01	0.032	0.011	4.3E-05
YOR202W	YOR202W	S288C_Ator08_hmg124h_8.JPG.dat	1201.7	140.0	1.0	0.07	0.010	0.072	7.0651904[illegible]
1	1	S288C_Ator08_hmg124h_14.JPG.dat	1783.8	275.2	#NUM!		#NUM!		
2	2	S288C_Ator08_hmg124h_14.JPG.dat	1443.8	181.0	1.2	0.10	0.263	0.100	1.5E-04
3	3	S288C_Ator08_hmg124h_14.JPG.dat	1441.3	219.7	1.1	0.13	0.138	0.127	4.2E-04
4	4	S288C_Ator08_hmg124h_14.JPG.dat	1380.8	168.6	1.0	0.06	-0.044	0.055	4.6E-05
YAL024C	YAL024C	S288C_Ator08_hmg124h_14.JPG.dat	1012.8	53.0	1.0	0.06	-0.037	0.058	5.3E-05
YAL046C	YAL046C	S288C_Ator08_hmg124h_14.JPG.dat	1065.3	7.3	1.0	0.00	0.028	0.003	4.6E-09
YAL048C	YAL048C	S288C_Ator08_hmg124h_14.JPG.dat	1033.0	3.4	1.0	0.00	-0.036	0.000	4.0E-08
YBL103C	YBL103C	S288C_Ator08_hmg124h_14.JPG.dat	1010.5	29.6	0.9	0.02	-0.067	0.024	4.3E-06
YBR020W	YBR020W	S288C_Ator08_hmg124h_14.JPG.dat	994.8	41.6	0.9	0.03	-0.074	0.031	1.0E-05
YBR061C	YBR061C	S288C_Ator08_hmg124h_14.JPG.dat	974.8	34.9	1.0	0.03	-0.026	0.031	9.0E-06
YBR075W	YBR075W	S288C_Ator08_hmg124h_14.JPG.dat	1033.5	47.2	1.1	0.05	0.049	0.050	3.0E-05
YBR078W	YBR078W	S288C_Ator08_hmg124h_14.JPG.dat	1024.3	47.5	1.0	0.02	-0.196	0.017	1.1E-04
YBR082C	YBR082C	S288C_Ator08_hmg124h_14.JPG.dat	935.5	19.3	1.0	0.00	-0.017	0.003	2.3E-06
YBR085W	YBR085W	S288C_Ator08_hmg124h_14.JPG.dat	910.8	33.8	0.9	0.03	-0.052	0.031	9.4E-06
YBR086C	YBR086C	S288C_Ator08_hmg124h_14.JPG.dat	910.0	40.2	0.9	0.04	-0.050	0.043	2.6E-05
YBR095C	YBR095C	S288C_Ator08_hmg124h_14.JPG.dat	1036.0	29.3	1.1	0.03	0.048	0.031	7.0E-06
YBR115C	YBR115C	S288C_Ator08_hmg124h_14.JPG.dat	0.0	0.0	0.0	0.00	0.000	0.000	
YBR118W	YBR118W	S288C_Ator08_hmg124h_14.JPG.dat	1072.3	53.4	1.1	0.01	0.095	0.008	1.9E-05
YBR165W	YBR165W	S288C_Ator08_hmg124h_14.JPG.dat	1078.3	14.3	1.1	0.02	0.059	0.015	7.3E-07
YBR299W	YBR299W	S288C_Ator08_hmg124h_14.JPG.dat	950.0	37.3	1.0	0.04	-0.037	0.040	1.9E-05
YCR107W	YCR107W	S288C_Ator08_hmg124h_14.JPG.dat	930.8	42.9	1.0	0.05	-0.046	0.045	2.8E-05
YDL074C	YDL074C	S288C_Ator08_hmg124h_14.JPG.dat	901.8	27.0	0.9	0.03	-0.021	0.025	5.2E-06
YDR026C	YDR026C	S288C_Ator08_hmg124h_14.JPG.dat	1052.0	15.8	1.0	0.01	-0.016	0.014	7.5E-07
YDR029W	YDR029W	S288C_Ator08_hmg124h_14.JPG.dat	1028.8	37.0	1.0	0.04	0.035	0.036	1.3E-05
YDR030C	YDR030C	S288C_Ator08_hmg124h_14.JPG.dat	1071.8	19.4	1.0	0.02	-0.017	0.020	2.1E-06
YDR031W	YDR031W	S288C_Ator08_hmg124h_14.JPG.dat	1066.8	25.0	1.0	0.03	0.023	0.028	5.6E-06
YDR032C	YDR032C	S288C_Ator08_hmg124h_14.JPG.dat	1047.3	39.1	1.0	0.03	0.016	0.034	1.0E-05
YDR033W	YDR033W	S288C_Ator08_hmg124h_14.JPG.dat	1070.8	50.5	1.0	0.01	0.013	0.009	2.3E-05
YDR034C	YDR034C	S288C_Ator08_hmg124h_14.JPG.dat	194.5	389.0	0.0	0.00	0.000	0.000	
YDR035W	YDR035W	S288C_Ator08_hmg124h_14.JPG.dat	1048.3	19.3	1.0	0.02	-0.013	0.019	1.8E-06
YDR036C	YDR036C	S288C_Ator08_hmg124h_14.JPG.dat	1012.8	36.9	1.0	0.01	0.012	0.011	4.3E-05
YDR042C	YDR042C	S288C_Ator08_hmg124h_14.JPG.dat	1074.3	28.4	1.0	0.02	-0.003	0.024	3.6E-06
YDR043C	YDR043C	S288C_Ator08_hmg124h_14.JPG.dat	1002.3	45.5	0.9	0.06	-0.087	0.057	6.8E-05
YDR046C	YDR046C	S288C_Ator08_hmg124h_14.JPG.dat	1106.3	18.5	1.0	0.03	-0.056	0.032	9.2E-06
YDR242W	YDR242W	S288C_Ator08_hmg124h_14.JPG.dat	995.5	13.8	1.0	0.00	-0.023	0.004	4.5E-06
YDR269C	YDR269C	S288C_Ator08_hmg124h_14.JPG.dat	977.0	15.1	1.0	0.01	-0.023	0.006	1.1E-05

YDR271C	YDR271C	S288C_Ator08_hmg124h_14.JPG.dat	996.3	19.3	1.0	0.02	-0.007	0.019	2.0E-06
YDR290W	YDR290W	S288C_Ator08_hmg124h_14.JPG.dat	965.3	44.6	1.0	0.04	0.007	0.042	2.3E-05
YDR326C	YDR326C	S288C_Ator08_hmg124h_14.JPG.dat	967.0	36.1	1.0	0.05	-0.023	0.048	3.0E-05
YDR444W	YDR444W	S288C_Ator08_hmg124h_14.JPG.dat	966.5	20.2	1.0	0.03	0.028	0.025	4.1E-06
YDR461W	YDR461W	S288C_Ator08_hmg124h_14.JPG.dat	251.5	503.0	0.0	0.00	0.000	0.000	
YDR477W	YDR477W	S288C_Ator08_hmg124h_14.JPG.dat	941.5	5.9	1.0	0.02	0.009	0.018	1.5E-06
YDR483W	YDR483W	S288C_Ator08_hmg124h_14.JPG.dat	955.0	34.4	1.0	0.03	0.001	0.029	6.7E-06
YDR493W	YDR493W	S288C_Ator08_hmg124h_14.JPG.dat	1006.5	29.4	1.0	0.03	0.046	0.032	7.8E-06
YDR502C	YDR502C	S288C_Ator08_hmg124h_14.JPG.dat	974.8	38.9	1.0	0.04	0.014	0.040	1.8E-05
YDR515W	YDR515W	S288C_Ator08_hmg124h_14.JPG.dat	985.5	10.7	1.0	0.01	-0.043	0.014	7.2E-07
YER089C	YER089C	S288C_Ator08_hmg124h_14.JPG.dat	1055.3	30.0	1.1	0.03	0.064	0.032	7.5E-06
YFL001W	YFL001W	S288C_Ator08_hmg124h_14.JPG.dat	1007.8	24.5	0.9	0.00	0.058	0.004	8.0E-06
YFL003C	YFL003C	S288C_Ator08_hmg124h_14.JPG.dat	890.0	56.3	0.9	0.02	-0.254	0.018	1.5E-04
YFL004W	YFL004W	S288C_Ator08_hmg124h_14.JPG.dat	1113.8	26.3	1.0	0.00	0.002	0.003	3.6E-06
YFL007W	YFL007W	S288C_Ator08_hmg124h_14.JPG.dat	1041.3	121.1	0.9	0.10	-0.044	0.102	3.5E-04
YFL010C	YFL010C	S288C_Ator08_hmg124h_14.JPG.dat	1160.8	36.0	1.0	0.01	0.009	0.010	2.7E-07
YFL010W-/	YFL010W-A	S288C_Ator08_hmg124h_14.JPG.dat	1161.3	61.1	1.0	0.02	0.021	0.025	3.7E-06
YFL012W	YFL012W	S288C_Ator08_hmg124h_14.JPG.dat	1161.0	72.4	1.0	0.01	0.017	0.013	5.4E-05
YFL013C	YFL013C	S288C_Ator08_hmg124h_14.JPG.dat	1032.8	48.6	1.0	0.02	0.086	0.020	2.1E-06
YFL033C	YFL033C	S288C_Ator08_hmg124h_14.JPG.dat	982.0	28.3	0.9	0.02	-0.050	0.024	4.6E-06
YFL063W	YFL063W	S288C_Ator08_hmg124h_14.JPG.dat	986.3	99.5	1.0	0.02	-0.004	0.023	1.6E-04
YGL029W	YGL029W	S288C_Ator08_hmg124h_14.JPG.dat	833.3	555.7	1.0	0.02	0.025	0.023	1.7E-04
YGL037C	YGL037C	S288C_Ator08_hmg124h_14.JPG.dat	1059.8	58.9	1.0	0.05	-0.025	0.051	3.8E-05
YGL042C	YGL042C	S288C_Ator08_hmg124h_14.JPG.dat	1013.5	80.9	1.0	0.02	-0.029	0.024	2.0E-04
YGL049C	YGL049C	S288C_Ator08_hmg124h_14.JPG.dat	1066.5	56.2	1.0	0.03	0.099	0.031	7.3E-06
YGL063W	YGL063W	S288C_Ator08_hmg124h_14.JPG.dat	1158.5	70.5	1.0	0.06	0.088	0.058	4.7E-05
YGL071W	YGL071W	S288C_Ator08_hmg124h_14.JPG.dat	1129.8	44.3	1.0	0.03	-0.008	0.028	6.5E-06
YGL094C	YGL094C	S288C_Ator08_hmg124h_14.JPG.dat	1036.3	53.8	1.0	0.05	-0.072	0.053	4.2E-05
YGL096W	YGL096W	S288C_Ator08_hmg124h_14.JPG.dat	1031.0	40.7	1.0	0.04	-0.002	0.043	2.2E-05
YGR055W	YGR055W	S288C_Ator08_hmg124h_14.JPG.dat	1049.3	40.0	0.9	0.02	-0.087	0.023	3.7E-06
YGR061C	YGR061C	S288C_Ator08_hmg124h_14.JPG.dat	1087.0	21.5	1.0	0.01	-0.098	0.010	3.6E-05
YGR067C	YGR067C	S288C_Ator08_hmg124h_14.JPG.dat	1080.5	31.2	1.0	0.00	-0.049	0.004	4.2E-06
YGR068C	YGR068C	S288C_Ator08_hmg124h_14.JPG.dat	1072.3	33.2	1.0	0.03	-0.090	0.031	8.8E-06
YGR122C-/	YGR122C-A	S288C_Ator08_hmg124h_14.JPG.dat	1235.5	29.8	1.1	0.00	0.244	0.002	1.1E-06
YGR122W	YGR122W	S288C_Ator08_hmg124h_14.JPG.dat	904.3	20.9	0.8	0.01	-0.213	0.010	4.4E-07
YGR254W	YGR254W	S288C_Ator08_hmg124h_14.JPG.dat	1131.8	17.5	1.0	0.04	0.055	0.041	1.8E-05
YGR255C	YGR255C	S288C_Ator08_hmg124h_14.JPG.dat	1116.8	16.2	1.0	0.02	0.025	0.024	3.9E-06
YGR271W	YGR271W	S288C_Ator08_hmg124h_14.JPG.dat	1062.0	15.8	0.9	0.04	-0.064	0.037	1.9E-05
YGR272C	YGR272C	S288C_Ator08_hmg124h_14.JPG.dat	1067.0	23.0	1.0	0.02	-0.017	0.022	3.0E-06
YGR273C	YGR273C	S288C_Ator08_hmg124h_14.JPG.dat	1078.5	35.8	1.0	0.03	0.025	0.032	8.2E-06
YGR276C	YGR276C	S288C_Ator08_hmg124h_14.JPG.dat	1060.0	18.0	1.0	0.02	0.009	0.017	1.3E-06
YGR289C	YGR289C	S288C_Ator08_hmg124h_14.JPG.dat	1062.5	22.6	1.0	0.02	0.039	0.021	2.3E-06
YGR291C	YGR291C	S288C_Ator08_hmg124h_14.JPG.dat	1091.5	18.4	1.0	0.02	-0.018	0.019	1.6E-06
YGR292W	YGR292W	S288C_Ator08_hmg124h_14.JPG.dat	1115.5	20.2	1.1	0.01	0.060	0.015	7.7E-07
YHR008C	YHR008C	S288C_Ator08_hmg124h_14.JPG.dat	922.8	71.7	1.0	0.03	-0.039	0.025	2.2E-04
YIL085C	YIL085C	S288C_Ator08_hmg124h_14.JPG.dat	1118.0	25.4	1.0	0.02	0.023	0.023	2.9E-06
YIL094C	YIL094C	S288C_Ator08_hmg124h_14.JPG.dat	536.3	619.8	0.5	0.59	0.024	0.589	1.8E-01
YIL102C	YIL102C	S288C_Ator08_hmg124h_14.JPG.dat	1071.3	21.9	1.0	0.02	0.028	0.017	1.3E-06
YIL111W	YIL111W	S288C_Ator08_hmg124h_14.JPG.dat	1012.8	50.2	1.0	0.05	-0.032	0.046	2.9E-05
YIL122W	YIL122W	S288C_Ator08_hmg124h_14.JPG.dat	1055.3	33.6	1.0	0.01	0.042	0.009	2.3E-05
YIL125W	YIL125W	S288C_Ator08_hmg124h_14.JPG.dat	287.5	575.0	0.0	0.00	0.000	0.000	
YIL131C	YIL131C	S288C_Ator08_hmg124h_14.JPG.dat	1036.5	21.2	1.0	0.02	0.022	0.015	1.0E-06
YIL136W	YIL136W	S288C_Ator08_hmg124h_14.JPG.dat	1066.8	14.0	1.0	0.00	0.017	0.003	2.3E-06
YIL151C	YIL151C	S288C_Ator08_hmg124h_14.JPG.dat	1048.5	29.0	1.0	0.01	0.005	0.010	3.5E-05
YIL158W	YIL158W	S288C_Ator08_hmg124h_14.JPG.dat	999.3	42.1	1.0	0.04	-0.052	0.039	1.8E-05
YIR004W	YIR004W	S288C_Ator08_hmg124h_14.JPG.dat	1067.5	33.5	1.0	0.04	0.013	0.036	1.1E-05
YJL029C	YJL029C	S288C_Ator08_hmg124h_14.JPG.dat	819.5	172.4	0.7	0.05	-0.105	0.047	1.4E-03
YJL079C	YJL079C	S288C_Ator08_hmg124h_14.JPG.dat	1014.0	35.2	1.0	0.04	0.003	0.036	1.2E-05
YJL082W	YJL082W	S288C_Ator08_hmg124h_14.JPG.dat	925.8	46.4	1.0	0.01	-0.049	0.011	4.6E-05
YJL092W	YJL092W	S288C_Ator08_hmg124h_14.JPG.dat	1203.8	74.0	1.1	0.04	0.137	0.036	9.9E-06
YJL103C	YJL103C	S288C_Ator08_hmg124h_14.JPG.dat	1061.3	18.0	1.0	0.02	0.054	0.015	8.6E-07
YJL106W	YJL106W	S288C_Ator08_hmg124h_14.JPG.dat	1011.0	45.5	1.0	0.03	-0.032	0.035	1.3E-05
YJL107C	YJL107C	S288C_Ator08_hmg124h_14.JPG.dat	1077.3	45.7	1.0	0.03	0.022	0.028	5.2E-06
YJL117W	YJL117W	S288C_Ator08_hmg124h_14.JPG.dat	1009.0	36.9	1.0	0.01	-0.017	0.006	1.2E-05
YJL119C	YJL119C	S288C_Ator08_hmg124h_14.JPG.dat	959.8	26.3	1.0	0.01	-0.040	0.007	1.7E-05
YJL120W	YJL120W	S288C_Ator08_hmg124h_14.JPG.dat	924.3	16.7	0.9	0.03	-0.051	0.031	1.0E-05
YJL177W	YJL177W	S288C_Ator08_hmg124h_14.JPG.dat	1089.5	98.1	1.0	0.06	0.003	0.062	7.1E-05
YJR034W	YJR034W	S288C_Ator08_hmg124h_14.JPG.dat	918.8	62.0	0.9	0.05	-0.070	0.045	3.1E-05
YJR039W	YJR039W	S288C_Ator08_hmg124h_14.JPG.dat	1113.5	32.4	1.1	0.03	-0.001	0.035	9.3E-06
YJR044C	YJR044C	S288C_Ator08_hmg124h_14.JPG.dat	990.3	19.7	1.0	0.02	-0.019	0.019	2.1E-06

YJR066W	YJR066W	S288C_Ator08_hmg124h_14.JPG.dat	1013.0	18.9	1.0	0.02	0.068	0.016	1.3E-06
YKL087C	YKL087C	S288C_Ator08_hmg124h_14.JPG.dat	996.3	56.0	0.9	0.05	-0.008	0.052	4.7E-05
YKR104W	YKR104W	S288C_Ator08_hmg124h_14.JPG.dat	1025.0	27.4	1.0	0.03	-0.084	0.031	9.6E-06
YLR227C	YLR227C	S288C_Ator08_hmg124h_14.JPG.dat	1097.5	30.3	1.0	0.03	0.030	0.029	6.1E-06
YLR238W	YLR238W	S288C_Ator08_hmg124h_14.JPG.dat	1026.0	6.1	1.0	0.01	0.005	0.006	4.9E-08
YLR308W	YLR308W	S288C_Ator08_hmg124h_14.JPG.dat	963.0	44.7	1.0	0.00	-0.016	0.003	3.4E-06
YLR455W	YLR455W	S288C_Ator08_hmg124h_14.JPG.dat	994.3	78.2	1.0	0.07	-0.046	0.074	1.2E-04
YML050W	YML050W	S288C_Ator08_hmg124h_14.JPG.dat	917.8	73.9	0.9	0.07	-0.011	0.074	1.4E-04
YML111W	YML111W	S288C_Ator08_hmg124h_14.JPG.dat	1049.8	27.2	1.0	0.01	-0.040	0.008	2.2E-05
YML112W	YML112W	S288C_Ator08_hmg124h_14.JPG.dat	1059.0	12.9	1.0	0.00	0.046	0.003	2.9E-06
YML129C	YML129C	S288C_Ator08_hmg124h_14.JPG.dat	540.8	624.8	0.5	0.56	-0.038	0.563	1.8E-01
YMR074C	YMR074C	S288C_Ator08_hmg124h_14.JPG.dat	1132.3	37.7	0.9	0.02	0.007	0.019	2.1E-06
YMR118C	YMR118C	S288C_Ator08_hmg124h_14.JPG.dat	1023.8	25.9	1.0	0.02	0.011	0.023	3.3E-06
YMR119W	YMR119W	S288C_Ator08_hmg124h_14.JPG.dat	982.5	47.9	1.0	0.01	-0.018	0.009	3.0E-05
YMR165C	YMR165C	S288C_Ator08_hmg124h_14.JPG.dat	1031.8	57.3	1.0	0.06	0.033	0.056	4.7E-05
YMR194C-.	YMR194C-A	S288C_Ator08_hmg124h_14.JPG.dat	1074.0	40.8	1.0	0.05	-0.008	0.046	2.8E-05
YNL011C	YNL011C	S288C_Ator08_hmg124h_14.JPG.dat	1145.5	34.6	1.0	0.01	-0.079	0.008	2.6E-05
YNL014W	YNL014W	S288C_Ator08_hmg124h_14.JPG.dat	1093.0	51.4	1.0	0.05	0.023	0.051	3.4E-05
YNL042W	YNL042W	S288C_Ator08_hmg124h_14.JPG.dat	1111.8	26.8	1.0	0.02	0.004	0.022	2.5E-06
YNL047C	YNL047C	S288C_Ator08_hmg124h_14.JPG.dat	954.5	68.2	0.9	0.07	-0.047	0.067	1.0E-04
YNL053W	YNL053W	S288C_Ator08_hmg124h_14.JPG.dat	942.8	53.6	1.0	0.01	0.044	0.013	6.2E-05
YNL086W	YNL086W	S288C_Ator08_hmg124h_14.JPG.dat	1023.0	20.2	1.0	0.02	0.023	0.020	2.0E-06
YNL096C	YNL096C	S288C_Ator08_hmg124h_14.JPG.dat	920.3	46.4	0.9	0.04	-0.004	0.044	3.2E-05
YNL109W	YNL109W	S288C_Ator08_hmg124h_14.JPG.dat	943.5	77.1	0.9	0.07	-0.073	0.074	1.3E-04
YNL111C	YNL111C	S288C_Ator08_hmg124h_14.JPG.dat	572.0	660.5	0.5	0.59	-0.029	0.592	1.8E-01
YNL147W	YNL147W	S288C_Ator08_hmg124h_14.JPG.dat	977.3	62.5	0.9	0.06	0.041	0.064	8.3E-05
YNL180C	YNL180C	S288C_Ator08_hmg124h_14.JPG.dat	1017.3	35.6	1.0	0.00	-0.025	0.004	6.1E-06
YNL284C	YNL284C	S288C_Ator08_hmg124h_14.JPG.dat	1068.3	27.4	1.1	0.02	0.081	0.024	2.8E-06
YNR033W	YNR033W	S288C_Ator08_hmg124h_14.JPG.dat	989.3	11.0	1.0	0.01	-0.018	0.009	2.1E-07
YNR041C	YNR041C	S288C_Ator08_hmg124h_14.JPG.dat	981.8	40.6	1.0	0.03	-0.056	0.034	1.2E-05
YNR044W	YNR044W	S288C_Ator08_hmg124h_14.JPG.dat	926.3	46.8	1.0	0.05	-0.010	0.047	3.1E-05
YOL073C	YOL073C	S288C_Ator08_hmg124h_14.JPG.dat	1002.5	8.6	1.0	0.01	0.022	0.005	3.7E-08
YOL086C	YOL086C	S288C_Ator08_hmg124h_14.JPG.dat	957.8	62.4	0.9	0.06	-0.064	0.063	8.6E-05
YOL153C	YOL153C	S288C_Ator08_hmg124h_14.JPG.dat	987.8	18.6	1.0	0.02	0.038	0.016	1.1E-06
YOR054C	YOR054C	S288C_Ator08_hmg124h_14.JPG.dat	901.5	29.9	0.9	0.01	-0.085	0.013	8.1E-05
YOR125C	YOR125C	S288C_Ator08_hmg124h_14.JPG.dat	925.0	45.2	0.9	0.01	-0.036	0.011	4.3E-05
YOR128C	YOR128C	S288C_Ator08_hmg124h_14.JPG.dat	1187.5	18.3	1.0	0.04	-0.087	0.038	1.3E-05
YOR155C	YOR155C	S288C_Ator08_hmg124h_14.JPG.dat	1057.0	28.1	1.0	0.02	-0.039	0.016	1.4E-06
YOR158W	YOR158W	S288C_Ator08_hmg124h_14.JPG.dat	1053.0	14.9	0.9	0.03	-0.063	0.035	1.4E-05
YOR179C	YOR179C	S288C_Ator08_hmg124h_14.JPG.dat	1039.5	7.3	0.9	0.01	0.000	0.008	1.6E-07
YOR180C	YOR180C	S288C_Ator08_hmg124h_14.JPG.dat	1106.3	7.1	1.0	0.01	0.025	0.013	5.3E-07
YOR202W	YOR202W	S288C_Ator08_hmg124h_14.JPG.dat	1402.1	222.6	1.0	0.09	0.012	0.088	2.2E-299
YOR220W	YOR220W	S288C_Ator08_hmg124h_14.JPG.dat	1030.5	50.5	1.0	0.05	-0.044	0.047	3.1E-05
YOR240W	YOR240W	S288C_Ator08_hmg124h_14.JPG.dat	1079.3	28.1	1.0	0.02	0.038	0.024	3.4E-06
YOR248W	YOR248W	S288C_Ator08_hmg124h_14.JPG.dat	1066.3	17.4	1.0	0.02	0.012	0.015	9.4E-07
YOR300W	YOR300W	S288C_Ator08_hmg124h_14.JPG.dat	1178.3	62.6	1.1	0.05	0.019	0.055	3.1E-05
YOR306C	YOR306C	S288C_Ator08_hmg124h_14.JPG.dat	971.3	65.0	1.0	0.07	-0.022	0.066	8.3E-05
YOR309C	YOR309C	S288C_Ator08_hmg124h_14.JPG.dat	1047.5	39.0	1.1	0.01	0.103	0.008	2.0E-05
YOR325W	YOR325W	S288C_Ator08_hmg124h_14.JPG.dat	940.8	73.0	1.0	0.08	-0.002	0.079	1.5E-04
YOR333C	YOR333C	S288C_Ator08_hmg124h_14.JPG.dat	988.0	29.5	1.0	0.03	0.066	0.028	5.6E-06
YOR345C	YOR345C	S288C_Ator08_hmg124h_14.JPG.dat	955.0	32.3	1.0	0.03	-0.019	0.032	9.1E-06
YOR366W	YOR366W	S288C_Ator08_hmg124h_14.JPG.dat	1011.3	37.7	1.0	0.00	0.016	0.001	1.7E-07
YOR379C	YOR379C	S288C_Ator08_hmg124h_14.JPG.dat	975.5	18.5	1.0	0.02	-0.033	0.017	1.4E-06
YPL015C	YPL015C	S288C_Ator08_hmg124h_14.JPG.dat	968.3	33.2	1.0	0.03	-0.058	0.031	9.2E-06
YPL035C	YPL035C	S288C_Ator08_hmg124h_14.JPG.dat	998.8	20.6	1.0	0.02	0.009	0.021	2.4E-06
YPL072W	YPL072W	S288C_Ator08_hmg124h_14.JPG.dat	981.3	35.5	1.0	0.03	0.004	0.031	9.0E-06
YPL158C	YPL158C	S288C_Ator08_hmg124h_14.JPG.dat	838.5	75.4	0.9	0.08	0.052	0.081	2.1E-04
YPL261C	YPL261C	S288C_Ator08_hmg124h_14.JPG.dat	1137.0	9.8	1.0	0.01	0.052	0.015	8.4E-07
YPL262W	YPL262W	S288C_Ator08_hmg124h_14.JPG.dat	1021.5	36.9	0.9	0.03	-0.006	0.028	7.5E-06
YPL263C	YPL263C	S288C_Ator08_hmg124h_14.JPG.dat	1034.0	14.1	1.0	0.01	-0.017	0.011	4.4E-07
YPL264C	YPL264C	S288C_Ator08_hmg124h_14.JPG.dat	1065.0	13.0	1.0	0.01	-0.002	0.011	3.6E-07
YPL265W	YPL265W	S288C_Ator08_hmg124h_14.JPG.dat	1099.8	67.8	1.0	0.06	0.061	0.063	6.1E-05
YPL267W	YPL267W	S288C_Ator08_hmg124h_14.JPG.dat	1050.0	71.1	1.0	0.06	-0.067	0.062	6.8E-05
YPL269W	YPL269W	S288C_Ator08_hmg124h_14.JPG.dat	1123.5	10.2	1.0	0.00	0.017	0.002	1.5E-06
YPL270W	YPL270W	S288C_Ator08_hmg124h_14.JPG.dat	1101.3	24.6	1.0	0.02	0.000	0.020	2.2E-06
YPL272C	YPL272C	S288C_Ator08_hmg124h_14.JPG.dat	1107.3	23.8	1.0	0.02	0.014	0.021	2.5E-06
YPL273W	YPL273W	S288C_Ator08_hmg124h_14.JPG.dat	1085.8	40.0	1.0	0.01	0.093	0.009	2.4E-05
YPL274W	YPL274W	S288C_Ator08_hmg124h_14.JPG.dat	1030.0	46.3	0.9	0.01	-0.030	0.008	2.7E-05
YPR001W	YPR001W	S288C_Ator08_hmg124h_14.JPG.dat	1100.3	30.3	1.0	0.04	0.015	0.043	2.2E-05
YPR002W	YPR002W	S288C_Ator08_hmg124h_14.JPG.dat	1099.8	23.6	1.0	0.02	-0.016	0.019	1.7E-06

YPR003C	YPR003C	S288C_Ator08_hmg124h_14.JPG.dat	1080.5	13.9	1.0	0.01	0.014	0.008	1.2E-07
YPR004C	YPR004C	S288C_Ator08_hmg124h_14.JPG.dat	1072.8	6.2	1.0	0.01	0.005	0.009	1.8E-07
YPR005C	YPR005C	S288C_Ator08_hmg124h_14.JPG.dat	1055.3	6.7	1.0	0.00	0.010	0.004	1.5E-08
YPR006C	YPR006C	S288C_Ator08_hmg124h_14.JPG.dat	1047.0	12.7	1.0	0.01	0.008	0.010	2.5E-07
YPR007C	YPR007C	S288C_Ator08_hmg124h_14.JPG.dat	1003.8	44.3	1.0	0.04	-0.038	0.042	2.3E-05
YPR008W	YPR008W	S288C_Ator08_hmg124h_14.JPG.dat	1032.0	8.0	1.0	0.01	-0.035	0.013	6.4E-07
YPR009W	YPR009W	S288C_Ator08_hmg124h_14.JPG.dat	1080.0	17.2	1.0	0.01	0.033	0.015	8.0E-07
YPR011C	YPR011C	S288C_Ator08_hmg124h_14.JPG.dat	946.3	19.3	1.0	0.01	0.023	0.010	3.1E-05
YPR012W	YPR012W	S288C_Ator08_hmg124h_14.JPG.dat	1077.0	26.6	1.0	0.02	0.045	0.018	1.5E-06
YPR013C	YPR013C	S288C_Ator08_hmg124h_14.JPG.dat	1013.0	68.3	0.9	0.03	-0.166	0.032	1.2E-05
YPR014C	YPR014C	S288C_Ator08_hmg124h_14.JPG.dat	1086.8	16.5	1.0	0.00	0.011	0.004	4.4E-06
YPR015C	YPR015C	S288C_Ator08_hmg124h_14.JPG.dat	1104.3	22.6	1.1	0.01	0.048	0.006	1.2E-05
YPR017C	YPR017C	S288C_Ator08_hmg124h_14.JPG.dat	1037.3	61.5	1.0	0.06	-0.007	0.056	4.6E-05
YPR018W	YPR018W	S288C_Ator08_hmg124h_14.JPG.dat	1124.0	13.9	1.1	0.01	0.134	0.008	1.1E-07
YPR020W	YPR020W	S288C_Ator08_hmg124h_14.JPG.dat	1012.3	16.3	1.0	0.02	0.043	0.015	9.9E-07
YPR021C	YPR021C	S288C_Ator08_hmg124h_14.JPG.dat	906.3	61.8	1.0	0.02	0.009	0.018	1.1E-04
YPR022C	YPR022C	S288C_Ator08_hmg124h_14.JPG.dat	1018.8	17.5	1.0	0.00	0.118	0.004	5.6E-06
YPR023C	YPR023C	S288C_Ator08_hmg124h_14.JPG.dat	975.0	75.9	0.9	0.02	-0.069	0.015	9.2E-05
YPR024W	YPR024W	S288C_Ator08_hmg124h_14.JPG.dat	667.8	515.3	0.6	0.50	-0.123	0.500	8.1E-02
YPR026W	YPR026W	S288C_Ator08_hmg124h_14.JPG.dat	1027.0	10.6	1.0	0.00	0.012	0.001	4.3E-07
YPR027C	YPR027C	S288C_Ator08_hmg124h_14.JPG.dat	1077.8	16.6	1.1	0.01	0.039	0.015	7.6E-07
YPR028W	YPR028W	S288C_Ator08_hmg124h_14.JPG.dat	1055.5	37.2	1.0	0.00	-0.066	0.003	2.6E-06
YPR029C	YPR029C	S288C_Ator08_hmg124h_14.JPG.dat	931.0	7.7	0.9	0.01	-0.177	0.010	4.3E-07
YPR030W	YPR030W	S288C_Ator08_hmg124h_14.JPG.dat	1050.3	24.6	1.0	0.03	0.026	0.034	9.2E-06
YPR031W	YPR031W	S288C_Ator08_hmg124h_14.JPG.dat	958.0	18.6	1.0	0.02	-0.028	0.022	3.3E-06
YPR032W	YPR032W	S288C_Ator08_hmg124h_14.JPG.dat	875.5	34.1	0.9	0.03	-0.041	0.031	1.1E-05
YPR037C	YPR037C	S288C_Ator08_hmg124h_14.JPG.dat	1000.3	26.7	1.1	0.00	0.051	0.002	1.8E-06
YPR038W	YPR038W	S288C_Ator08_hmg124h_14.JPG.dat	1015.5	51.7	1.1	0.05	0.007	0.047	2.4E-05
YPR039W	YPR039W	S288C_Ator08_hmg124h_14.JPG.dat	1036.3	45.7	1.0	0.01	-0.006	0.013	5.5E-05
YPR040W	YPR040W	S288C_Ator08_hmg124h_14.JPG.dat	976.3	26.6	1.0	0.02	-0.005	0.025	4.4E-06
YPR042C	YPR042C	S288C_Ator08_hmg124h_14.JPG.dat	1012.5	33.3	1.0	0.01	0.056	0.011	3.8E-05
YPR043W	YPR043W	S288C_Ator08_hmg124h_14.JPG.dat	994.0	32.9	1.0	0.03	0.154	0.030	7.8E-06
YPR044C	YPR044C	S288C_Ator08_hmg124h_14.JPG.dat	1016.0	56.2	1.0	0.06	-0.035	0.057	4.9E-05
YPR045C	YPR045C	S288C_Ator08_hmg124h_14.JPG.dat	1073.3	18.3	1.0	0.03	-0.016	0.034	1.1E-05
YPR046W	YPR046W	S288C_Ator08_hmg124h_14.JPG.dat	1139.3	25.7	1.1	0.04	0.011	0.038	1.2E-05
YPR050C	YPR050C	S288C_Ator08_hmg124h_14.JPG.dat	919.5	42.9	0.9	0.04	-0.108	0.036	1.7E-05
YPR051W	YPR051W	S288C_Ator08_hmg124h_14.JPG.dat	917.3	19.4	0.9	0.03	-0.074	0.027	6.4E-06
YPR052C	YPR052C	S288C_Ator08_hmg124h_14.JPG.dat	991.0	31.2	1.0	0.03	0.030	0.029	6.0E-06
YPR053C	YPR053C	S288C_Ator08_hmg124h_14.JPG.dat	950.8	23.8	1.0	0.03	0.035	0.026	4.7E-06
YPR054W	YPR054W	S288C_Ator08_hmg124h_14.JPG.dat	943.5	38.5	1.0	0.04	0.012	0.040	1.7E-05
YPR057W	YPR057W	S288C_Ator08_hmg124h_14.JPG.dat	830.5	119.5	0.9	0.13	0.133	0.126	8.3E-04
YPR058W	YPR058W	S288C_Ator08_hmg124h_14.JPG.dat	983.3	35.5	1.0	0.04	-0.003	0.039	1.6E-05
YPR059C	YPR059C	S288C_Ator08_hmg124h_14.JPG.dat	1017.3	20.9	1.0	0.03	0.033	0.025	4.0E-06
YPR060C	YPR060C	S288C_Ator08_hmg124h_14.JPG.dat	921.5	11.6	0.9	0.01	-0.012	0.015	1.1E-06
YPR061C	YPR061C	S288C_Ator08_hmg124h_14.JPG.dat	1098.0	31.3	1.0	0.02	0.055	0.023	2.8E-06
YPR062W	YPR062W	S288C_Ator08_hmg124h_14.JPG.dat	1159.0	15.4	1.1	0.02	-0.003	0.019	1.4E-06
YPR063C	YPR063C	S288C_Ator08_hmg124h_14.JPG.dat	958.3	32.5	0.9	0.03	-0.017	0.032	1.1E-05
YPR064W	YPR064W	S288C_Ator08_hmg124h_14.JPG.dat	1047.8	42.3	1.1	0.04	0.051	0.041	1.6E-05
YPR065W	YPR065W	S288C_Ator08_hmg124h_14.JPG.dat	1026.8	23.6	1.1	0.02	0.051	0.019	1.6E-06
YPR066W	YPR066W	S288C_Ator08_hmg124h_14.JPG.dat	962.0	43.2	1.0	0.01	0.118	0.014	6.4E-05
YPR068C	YPR068C	S288C_Ator08_hmg124h_14.JPG.dat	909.3	43.8	1.0	0.05	0.000	0.048	3.3E-05
YPR069C	YPR069C	S288C_Ator08_hmg124h_14.JPG.dat	917.0	65.3	1.0	0.02	0.118	0.020	1.3E-04
YPR070W	YPR070W	S288C_Ator08_hmg124h_14.JPG.dat	772.5	44.0	0.8	0.04	-0.075	0.044	4.6E-05
YPR071W	YPR071W	S288C_Ator08_hmg124h_14.JPG.dat	963.3	73.8	1.0	0.07	-0.026	0.075	1.2E-04
YPR073C	YPR073C	S288C_Ator08_hmg124h_14.JPG.dat	1013.5	24.3	1.0	0.02	0.013	0.023	3.2E-06
YPR074C	YPR074C	S288C_Ator08_hmg124h_14.JPG.dat	997.5	26.0	1.0	0.01	-0.073	0.008	2.1E-05
YPR075C	YPR075C	S288C_Ator08_hmg124h_14.JPG.dat	1112.0	57.2	1.1	0.04	0.064	0.044	2.1E-05
YPR076W	YPR076W	S288C_Ator08_hmg124h_14.JPG.dat	1068.0	21.7	1.0	0.01	-0.019	0.009	2.8E-05
YPR077C	YPR077C	S288C_Ator08_hmg124h_14.JPG.dat	1130.5	27.1	1.1	0.01	0.027	0.006	9.4E-06
YPR078C	YPR078C	S288C_Ator08_hmg124h_14.JPG.dat	1025.0	26.0	1.0	0.02	-0.033	0.018	1.7E-06
YPR079W	YPR079W	S288C_Ator08_hmg124h_14.JPG.dat	961.8	30.5	1.0	0.03	-0.059	0.028	6.9E-06
YPR083W	YPR083W	S288C_Ator08_hmg124h_14.JPG.dat	975.5	32.5	1.0	0.04	0.021	0.043	2.1E-05
YPR084W	YPR084W	S288C_Ator08_hmg124h_14.JPG.dat	958.5	39.9	1.0	0.01	-0.062	0.009	2.7E-05
YPR089W	YPR089W	S288C_Ator08_hmg124h_14.JPG.dat	1013.8	20.6	1.0	0.01	0.022	0.012	4.3E-07
YPR090W	YPR090W	S288C_Ator08_hmg124h_14.JPG.dat	970.3	38.0	1.0	0.03	-0.039	0.030	8.7E-06
YPR091C	YPR091C	S288C_Ator08_hmg124h_14.JPG.dat	977.3	10.3	1.0	0.02	-0.025	0.017	1.3E-06
YPR092W	YPR092W	S288C_Ator08_hmg124h_14.JPG.dat	977.5	28.9	0.9	0.02	-0.063	0.023	4.1E-06
YPR093C	YPR093C	S288C_Ator08_hmg124h_14.JPG.dat	907.3	57.8	0.9	0.04	-0.071	0.040	2.4E-05
YPR095C	YPR095C	S288C_Ator08_hmg124h_14.JPG.dat	1046.0	56.5	1.1	0.05	0.106	0.047	2.4E-05
YPR096C	YPR096C	S288C_Ator08_hmg124h_14.JPG.dat	1149.0	33.9	1.0	0.03	-0.029	0.025	4.7E-06

YPR097W	YPR097W	S288C_Ator08_hmg124h_14.JPG.dat	1068.3	16.5	1.0	0.01	-0.017	0.010	3.1E-07
YPR098C	YPR098C	S288C_Ator08_hmg124h_14.JPG.dat	1025.3	34.3	0.9	0.01	-0.054	0.013	6.0E-05
YPR106W	YPR106W	S288C_Ator08_hmg124h_14.JPG.dat	1030.0	13.9	1.0	0.01	0.004	0.014	7.6E-07
YPR109W	YPR109W	S288C_Ator08_hmg124h_14.JPG.dat	1044.8	17.8	1.0	0.01	-0.039	0.014	8.3E-07
YPR111W	YPR111W	S288C_Ator08_hmg124h_14.JPG.dat	1000.8	50.8	0.9	0.04	-0.048	0.045	2.9E-05
YPR114W	YPR114W	S288C_Ator08_hmg124h_14.JPG.dat	1038.0	40.6	1.0	0.04	-0.057	0.044	2.5E-05
YPR115W	YPR115W	S288C_Ator08_hmg124h_14.JPG.dat	1113.5	33.3	1.0	0.03	0.021	0.033	9.5E-06
YPR117W	YPR117W	S288C_Ator08_hmg124h_14.JPG.dat	1106.0	65.5	1.0	0.06	-0.016	0.062	6.3E-05
YPR118W	YPR118W	S288C_Ator08_hmg124h_14.JPG.dat	834.0	60.5	0.9	0.07	-0.147	0.068	1.4E-04
YPR119W	YPR119W	S288C_Ator08_hmg124h_14.JPG.dat	1144.8	26.6	1.1	0.03	0.069	0.026	4.1E-06
YPR120C	YPR120C	S288C_Ator08_hmg124h_14.JPG.dat	942.0	44.0	0.9	0.06	-0.071	0.056	6.4E-05
YPR121W	YPR121W	S288C_Ator08_hmg124h_14.JPG.dat	1110.0	34.0	0.9	0.02	-0.009	0.017	1.8E-06
YPR122W	YPR122W	S288C_Ator08_hmg124h_14.JPG.dat	1123.5	25.9	1.0	0.03	0.036	0.028	5.7E-06
YPR123C	YPR123C	S288C_Ator08_hmg124h_14.JPG.dat	1083.8	36.5	1.0	0.04	0.020	0.040	1.6E-05
YPR125W	YPR125W	S288C_Ator08_hmg124h_14.JPG.dat	1075.3	11.3	1.0	0.01	0.013	0.015	8.1E-07
YPR126C	YPR126C	S288C_Ator08_hmg124h_14.JPG.dat	1039.3	15.4	1.0	0.02	0.012	0.019	1.8E-06
YPR127W	YPR127W	S288C_Ator08_hmg124h_14.JPG.dat	1021.0	105.6	0.9	0.02	-0.048	0.021	1.6E-04
YPR128C	YPR128C	S288C_Ator08_hmg124h_14.JPG.dat	1030.0	50.4	1.0	0.04	-0.010	0.042	2.2E-05
YPR129W	YPR129W	S288C_Ator08_hmg124h_14.JPG.dat	1040.0	12.5	1.0	0.00	0.021	0.003	3.1E-06
YPR130C	YPR130C	S288C_Ator08_hmg124h_14.JPG.dat	1053.8	33.5	1.0	0.03	-0.008	0.027	5.7E-06
YPR132W	YPR132W	S288C_Ator08_hmg124h_14.JPG.dat	1101.0	48.7	1.1	0.04	0.099	0.043	1.8E-05
YPR134W	YPR134W	S288C_Ator08_hmg124h_14.JPG.dat	763.8	246.3	0.9	0.07	0.012	0.072	2.4E-03
YPR135W	YPR135W	S288C_Ator08_hmg124h_14.JPG.dat	982.8	16.4	0.8	0.02	-0.045	0.024	6.4E-06
YPR138C	YPR138C	S288C_Ator08_hmg124h_14.JPG.dat	1083.0	26.8	1.0	0.02	-0.008	0.025	4.0E-06
YPR140W	YPR140W	S288C_Ator08_hmg124h_14.JPG.dat	1025.8	29.1	1.0	0.02	-0.028	0.021	2.5E-06
YPR141C	YPR141C	S288C_Ator08_hmg124h_14.JPG.dat	906.8	26.2	0.9	0.01	0.071	0.007	2.2E-05
YPR145W	YPR145W	S288C_Ator08_hmg124h_14.JPG.dat	966.8	20.3	1.0	0.02	-0.006	0.019	2.2E-06
YPR146C	YPR146C	S288C_Ator08_hmg124h_14.JPG.dat	1000.0	34.3	1.0	0.03	-0.032	0.027	5.9E-06
YPR147C	YPR147C	S288C_Ator08_hmg124h_14.JPG.dat	1008.5	25.6	1.0	0.02	-0.044	0.025	4.4E-06
YPR148C	YPR148C	S288C_Ator08_hmg124h_14.JPG.dat	1062.0	19.6	1.0	0.02	0.040	0.021	2.3E-06
YPR149W	YPR149W	S288C_Ator08_hmg124h_14.JPG.dat	1070.8	37.4	1.0	0.03	0.026	0.032	7.7E-06
YPR150W	YPR150W	S288C_Ator08_hmg124h_14.JPG.dat	987.5	17.2	1.0	0.02	-0.026	0.016	1.3E-06
YPR151C	YPR151C	S288C_Ator08_hmg124h_14.JPG.dat	1009.3	30.7	1.1	0.01	0.056	0.011	3.8E-05
YPR152C	YPR152C	S288C_Ator08_hmg124h_14.JPG.dat	1021.3	15.7	1.0	0.02	-0.007	0.020	2.2E-06
YPR153W	YPR153W	S288C_Ator08_hmg124h_14.JPG.dat	1073.0	50.3	0.9	0.03	-0.049	0.033	1.2E-05
YPR154W	YPR154W	S288C_Ator08_hmg124h_14.JPG.dat	1081.0	26.4	1.1	0.03	0.032	0.031	7.4E-06
YPR155C	YPR155C	S288C_Ator08_hmg124h_14.JPG.dat	897.8	28.4	0.9	0.04	-0.073	0.036	1.6E-05
YPR156C	YPR156C	S288C_Ator08_hmg124h_14.JPG.dat	975.8	17.6	1.0	0.01	-0.035	0.014	7.5E-07
YPR157W	YPR157W	S288C_Ator08_hmg124h_14.JPG.dat	972.3	6.6	1.0	0.00	0.065	0.004	1.7E-08
YPR158W	YPR158W	S288C_Ator08_hmg124h_14.JPG.dat	1006.3	27.0	1.0	0.02	0.014	0.022	2.5E-06
YPR160W	YPR160W	S288C_Ator08_hmg124h_14.JPG.dat	992.0	42.3	1.0	0.04	0.009	0.035	1.2E-05
YPR164W	YPR164W	S288C_Ator08_hmg124h_14.JPG.dat	957.0	22.9	1.0	0.02	0.016	0.023	3.8E-06
YPR167C	YPR167C	S288C_Ator08_hmg124h_14.JPG.dat	1095.5	28.6	1.1	0.03	0.055	0.031	6.3E-06
YPR170C	YPR170C	S288C_Ator08_hmg124h_14.JPG.dat	981.3	68.3	1.0	0.07	-0.050	0.068	9.5E-05
YPR171W	YPR171W	S288C_Ator08_hmg124h_14.JPG.dat	1044.8	25.2	1.0	0.03	0.024	0.029	5.8E-06
YPR172W	YPR172W	S288C_Ator08_hmg124h_14.JPG.dat	1084.3	92.5	1.0	0.09	-0.089	0.089	2.1E-04
YPR173C	YPR173C	S288C_Ator08_hmg124h_14.JPG.dat	423.8	399.9	0.2	0.13	-0.096	0.134	9.8E-02
YPR174C	YPR174C	S288C_Ator08_hmg124h_14.JPG.dat	911.5	30.5	0.9	0.03	-0.036	0.032	1.1E-05
YPR179C	YPR179C	S288C_Ator08_hmg124h_14.JPG.dat	989.0	27.8	1.0	0.03	0.042	0.032	8.1E-06
YPR184W	YPR184W	S288C_Ator08_hmg124h_14.JPG.dat	944.8	4.9	1.0	0.00	-0.004	0.003	7.7E-09
YPR185W	YPR185W	S288C_Ator08_hmg124h_14.JPG.dat	922.0	21.9	1.0	0.02	-0.003	0.019	2.0E-06
YPR188C	YPR188C	S288C_Ator08_hmg124h_14.JPG.dat	918.5	27.3	1.0	0.02	-0.021	0.023	3.6E-06
YPR189W	YPR189W	S288C_Ator08_hmg124h_14.JPG.dat	992.5	47.0	1.0	0.05	0.057	0.052	3.6E-05
YPR191W	YPR191W	S288C_Ator08_hmg124h_14.JPG.dat	985.5	94.7	1.0	0.10	0.037	0.097	2.5E-04
YPR192W	YPR192W	S288C_Ator08_hmg124h_14.JPG.dat	1080.0	18.1	1.1	0.00	0.063	0.005	5.8E-06
YPR193C	YPR193C	S288C_Ator08_hmg124h_14.JPG.dat	1058.5	14.7	1.1	0.02	0.033	0.021	2.0E-06
YPR194C	YPR194C	S288C_Ator08_hmg124h_14.JPG.dat	1111.3	117.6	1.0	0.08	-0.064	0.077	1.3E-04
YPR195C	YPR195C	S288C_Ator08_hmg124h_14.JPG.dat	985.8	43.9	1.0	0.04	-0.037	0.039	1.9E-05
YPR196W	YPR196W	S288C_Ator08_hmg124h_14.JPG.dat	1044.3	30.9	1.1	0.03	0.030	0.029	5.6E-06
YPR197C	YPR197C	S288C_Ator08_hmg124h_14.JPG.dat	672.3	451.3	0.9	0.07	-0.064	0.067	1.8E-03
YPR198W	YPR198W	S288C_Ator08_hmg124h_14.JPG.dat	977.5	7.6	1.0	0.00	0.040	0.002	3.2E-09
YPR199C	YPR199C	S288C_Ator08_hmg124h_14.JPG.dat	948.5	48.8	1.0	0.05	0.009	0.048	2.9E-05
YPR200C	YPR200C	S288C_Ator08_hmg124h_14.JPG.dat	976.0	18.7	1.0	0.01	0.062	0.006	1.2E-05
YPR201W	YPR201W	S288C_Ator08_hmg124h_14.JPG.dat	935.3	59.8	1.0	0.06	-0.026	0.062	7.4E-05
1	1	S288C_Ator08_hmg124h_1.JPG.dat	1172.0	69.1	1.0	0.04	-0.069	0.038	1.4E-05
2	2	S288C_Ator08_hmg124h_1.JPG.dat	1075.8	83.9	1.0	0.07	0.012	0.065	8.1E-05
3	3	S288C_Ator08_hmg124h_1.JPG.dat	1079.0	92.8	1.0	0.08	0.026	0.081	1.3E-04
4	4	S288C_Ator08_hmg124h_1.JPG.dat	1004.8	53.7	1.0	0.04	-0.013	0.042	2.2E-05
YAL002W	YAL002W	S288C_Ator08_hmg124h_1.JPG.dat	973.3	30.7	1.0	0.04	-0.125	0.041	2.1E-05
YAL004W	YAL004W	S288C_Ator08_hmg124h_1.JPG.dat	1039.5	49.9	1.0	0.01	-0.014	0.007	1.7E-05

YAL005C	YAL005C	S288C_Ator08_hmg124h_1.JPG.dat	1089.3	42.5	1.0	0.04	-0.012	0.039	1.6E-05
YAL007C	YAL007C	S288C_Ator08_hmg124h_1.JPG.dat	1178.8	20.4	1.0	0.01	0.037	0.014	6.8E-07
YAL008W	YAL008W	S288C_Ator08_hmg124h_1.JPG.dat	1184.8	43.7	1.0	0.02	0.009	0.016	8.4E-05
YAL010C	YAL010C	S288C_Ator08_hmg124h_1.JPG.dat	998.8	150.6	0.8	0.04	-0.028	0.036	6.4E-04
YAL011W	YAL011W	S288C_Ator08_hmg124h_1.JPG.dat	981.8	10.9	0.9	0.01	-0.071	0.011	5.8E-07
YAL013W	YAL013W	S288C_Ator08_hmg124h_1.JPG.dat	1134.8	32.9	1.0	0.03	0.115	0.026	5.2E-06
YAL014C	YAL014C	S288C_Ator08_hmg124h_1.JPG.dat	1170.8	14.8	1.0	0.02	0.022	0.015	1.0E-06
YAL015C	YAL015C	S288C_Ator08_hmg124h_1.JPG.dat	1136.3	23.4	1.0	0.02	-0.011	0.023	3.6E-06
YAL017W	YAL017W	S288C_Ator08_hmg124h_1.JPG.dat	1138.3	45.6	1.1	0.07	0.039	0.067	7.1E-05
YAL018C	YAL018C	S288C_Ator08_hmg124h_1.JPG.dat	1021.8	57.0	1.0	0.06	-0.014	0.058	4.7E-05
YAL019W	YAL019W	S288C_Ator08_hmg124h_1.JPG.dat	1020.5	28.4	1.0	0.00	-0.019	0.001	4.6E-07
YAL020C	YAL020C	S288C_Ator08_hmg124h_1.JPG.dat	1053.5	30.8	1.0	0.03	0.063	0.028	6.4E-06
YAL022C	YAL022C	S288C_Ator08_hmg124h_1.JPG.dat	1108.5	20.1	1.0	0.02	-0.007	0.019	1.9E-06
YAL023C	YAL023C	S288C_Ator08_hmg124h_1.JPG.dat	1128.8	27.1	1.0	0.02	-0.064	0.019	1.7E-06
YAL026C	YAL026C	S288C_Ator08_hmg124h_1.JPG.dat	448.3	77.4	0.4	0.03	-0.505	0.026	1.6E-03
YAL027W	YAL027W	S288C_Ator08_hmg124h_1.JPG.dat	1083.8	62.9	1.0	0.05	-0.027	0.049	3.4E-05
YAL028W	YAL028W	S288C_Ator08_hmg124h_1.JPG.dat	1105.5	44.6	1.0	0.04	-0.024	0.042	2.0E-05
YAL029C	YAL029C	S288C_Ator08_hmg124h_1.JPG.dat	1156.3	29.3	1.0	0.00	0.002	0.004	6.5E-06
YAL030W	YAL030W	S288C_Ator08_hmg124h_1.JPG.dat	1129.0	61.2	1.0	0.05	-0.028	0.046	2.6E-05
YAL031C	YAL031C	S288C_Ator08_hmg124h_1.JPG.dat	1096.3	35.3	1.1	0.02	0.059	0.021	2.2E-06
YAL034C	YAL034C	S288C_Ator08_hmg124h_1.JPG.dat	963.8	37.1	1.0	0.04	0.031	0.042	2.1E-05
YAL036C	YAL036C	S288C_Ator08_hmg124h_1.JPG.dat	1054.0	50.3	1.0	0.04	0.001	0.041	1.8E-05
YAL037W	YAL037W	S288C_Ator08_hmg124h_1.JPG.dat	1102.0	51.3	1.1	0.01	0.031	0.010	2.8E-05
YAL040C	YAL040C	S288C_Ator08_hmg124h_1.JPG.dat	1088.0	5.4	1.0	0.01	0.001	0.013	6.7E-07
YAL042W	YAL042W	S288C_Ator08_hmg124h_1.JPG.dat	1006.8	109.4	0.9	0.09	-0.061	0.094	2.8E-04
YAL043C-A	YAL043C-A	S288C_Ator08_hmg124h_1.JPG.dat	997.8	114.7	0.9	0.10	-0.058	0.105	4.0E-04
YAL045C	YAL045C	S288C_Ator08_hmg124h_1.JPG.dat	1044.5	40.1	1.0	0.01	-0.019	0.007	1.9E-05
YAL049C	YAL049C	S288C_Ator08_hmg124h_1.JPG.dat	1094.3	14.2	1.0	0.01	0.012	0.014	7.5E-07
YAL051W	YAL051W	S288C_Ator08_hmg124h_1.JPG.dat	1125.5	32.6	1.0	0.01	0.004	0.007	1.7E-05
YAL053W	YAL053W	S288C_Ator08_hmg124h_1.JPG.dat	1095.5	17.4	1.0	0.02	-0.006	0.017	1.3E-06
YAL054C	YAL054C	S288C_Ator08_hmg124h_1.JPG.dat	1010.0	23.2	1.0	0.02	-0.004	0.017	1.3E-06
YAL055W	YAL055W	S288C_Ator08_hmg124h_1.JPG.dat	934.5	54.3	1.0	0.00	0.020	0.002	1.3E-06
YAL056W	YAL056W	S288C_Ator08_hmg124h_1.JPG.dat	1059.0	38.3	1.0	0.04	-0.061	0.039	1.5E-05
YAL058C-A	YAL058C-A	S288C_Ator08_hmg124h_1.JPG.dat	985.5	59.7	0.9	0.06	-0.061	0.056	6.2E-05
YAL058W	YAL058W	S288C_Ator08_hmg124h_1.JPG.dat	1045.5	31.3	1.0	0.03	0.015	0.032	1.1E-05
YAL059W	YAL059W	S288C_Ator08_hmg124h_1.JPG.dat	1101.0	41.8	1.0	0.03	0.014	0.033	9.2E-06
YAL060W	YAL060W	S288C_Ator08_hmg124h_1.JPG.dat	1114.5	30.1	1.0	0.03	0.038	0.033	9.5E-06
YAL061W	YAL061W	S288C_Ator08_hmg124h_1.JPG.dat	1106.0	47.8	1.0	0.04	0.017	0.042	1.9E-05
YAL062W	YAL062W	S288C_Ator08_hmg124h_1.JPG.dat	1112.3	63.0	1.0	0.05	0.025	0.052	3.6E-05
YAL064C-A	YAL064C-A	S288C_Ator08_hmg124h_1.JPG.dat	1058.3	79.1	1.0	0.08	-0.051	0.075	1.4E-04
YAL065C	YAL065C	S288C_Ator08_hmg124h_1.JPG.dat	1105.5	63.0	1.0	0.05	-0.042	0.051	3.4E-05
YAL066W	YAL066W	S288C_Ator08_hmg124h_1.JPG.dat	1011.3	37.6	1.0	0.01	-0.005	0.014	8.3E-07
YAL067C	YAL067C	S288C_Ator08_hmg124h_1.JPG.dat	1006.8	44.9	1.0	0.04	-0.010	0.039	1.5E-05
YAL068C	YAL068C	S288C_Ator08_hmg124h_1.JPG.dat	1029.0	47.3	1.0	0.04	-0.024	0.043	2.4E-05
YAR002C-A	YAR002C-A	S288C_Ator08_hmg124h_1.JPG.dat	1164.8	41.8	1.1	0.04	0.058	0.043	1.7E-05
YAR002W	YAR002W	S288C_Ator08_hmg124h_1.JPG.dat	988.8	39.5	0.9	0.04	0.038	0.035	1.6E-05
YAR003W	YAR003W	S288C_Ator08_hmg124h_1.JPG.dat	1055.0	22.6	1.0	0.03	0.047	0.028	5.8E-06
YAR014C	YAR014C	S288C_Ator08_hmg124h_1.JPG.dat	1070.8	72.8	1.0	0.02	0.025	0.019	1.1E-04
YAR015W	YAR015W	S288C_Ator08_hmg124h_1.JPG.dat	1079.0	52.8	1.0	0.00	0.032	0.005	7.8E-06
YAR018C	YAR018C	S288C_Ator08_hmg124h_1.JPG.dat	1138.8	71.8	1.1	0.02	0.069	0.019	1.0E-04
YAR020C	YAR020C	S288C_Ator08_hmg124h_1.JPG.dat	1156.8	25.1	1.0	0.01	0.015	0.008	1.8E-05
YAR023C	YAR023C	S288C_Ator08_hmg124h_1.JPG.dat	1084.5	38.9	1.0	0.04	0.034	0.036	1.3E-05
YAR027W	YAR027W	S288C_Ator08_hmg124h_1.JPG.dat	1014.0	73.0	1.0	0.02	0.021	0.015	7.6E-05
YAR028W	YAR028W	S288C_Ator08_hmg124h_1.JPG.dat	1096.3	56.1	1.0	0.03	0.043	0.026	2.0E-04
YAR029W	YAR029W	S288C_Ator08_hmg124h_1.JPG.dat	1129.0	40.5	1.0	0.04	0.026	0.040	1.6E-05
YAR030C	YAR030C	S288C_Ator08_hmg124h_1.JPG.dat	1168.0	26.4	1.1	0.02	0.067	0.024	3.1E-06
YAR031W	YAR031W	S288C_Ator08_hmg124h_1.JPG.dat	1101.5	43.8	1.0	0.04	0.021	0.041	1.9E-05
YAR035W	YAR035W	S288C_Ator08_hmg124h_1.JPG.dat	1045.5	34.0	1.0	0.03	0.019	0.031	9.1E-06
YAR037W	YAR037W	S288C_Ator08_hmg124h_1.JPG.dat	1079.5	17.7	1.0	0.01	0.008	0.012	5.4E-07
YAR040C	YAR040C	S288C_Ator08_hmg124h_1.JPG.dat	1098.5	16.7	1.0	0.01	-0.033	0.014	8.3E-07
YAR042W	YAR042W	S288C_Ator08_hmg124h_1.JPG.dat	1054.5	40.8	1.0	0.04	-0.052	0.040	2.1E-05
YAR043C	YAR043C	S288C_Ator08_hmg124h_1.JPG.dat	1077.8	12.9	1.0	0.01	-0.043	0.010	3.0E-07
YAR044W	YAR044W	S288C_Ator08_hmg124h_1.JPG.dat	1031.5	59.8	1.0	0.05	-0.041	0.054	4.9E-05
YAR047C	YAR047C	S288C_Ator08_hmg124h_1.JPG.dat	978.0	45.8	1.0	0.05	-0.044	0.045	2.9E-05
YAR050W	YAR050W	S288C_Ator08_hmg124h_1.JPG.dat	1041.3	68.6	1.0	0.01	-0.007	0.007	1.4E-05
YBL001C	YBL001C	S288C_Ator08_hmg124h_1.JPG.dat	1129.8	64.2	1.0	0.05	0.014	0.054	4.0E-05
YBL003C	YBL003C	S288C_Ator08_hmg124h_1.JPG.dat	1059.5	62.1	1.0	0.05	-0.041	0.050	3.8E-05
YBL005W	YBL005W	S288C_Ator08_hmg124h_1.JPG.dat	1127.5	49.7	1.0	0.05	0.014	0.050	3.1E-05
YBL007C	YBL007C	S288C_Ator08_hmg124h_1.JPG.dat	1102.5	44.2	1.0	0.04	0.018	0.036	1.3E-05
YBL008W	YBL008W	S288C_Ator08_hmg124h_1.JPG.dat	1165.5	44.8	1.0	0.04	0.001	0.043	2.0E-05

YBL009W	YBL009W	S288C_Ator08_hmg124h_1.JPG.dat	1148.3	86.4	1.1	0.02	0.010	0.018	9.9E-05
YBL010C	YBL010C	S288C_Ator08_hmg124h_1.JPG.dat	1109.0	38.4	1.0	0.03	-0.033	0.028	6.1E-06
YBL011W	YBL011W	S288C_Ator08_hmg124h_1.JPG.dat	1070.0	71.9	0.9	0.02	-0.069	0.020	1.6E-04
YBL013W	YBL013W	S288C_Ator08_hmg124h_1.JPG.dat	1072.5	22.0	1.0	0.01	0.011	0.014	7.9E-07
YBL015W	YBL015W	S288C_Ator08_hmg124h_1.JPG.dat	980.0	79.1	0.9	0.01	-0.036	0.014	7.6E-05
YBL016W	YBL016W	S288C_Ator08_hmg124h_1.JPG.dat	923.8	36.2	0.9	0.02	-0.045	0.016	1.0E-04
YBL017C	YBL017C	S288C_Ator08_hmg124h_1.JPG.dat	1046.5	35.5	1.0	0.04	-0.051	0.036	1.2E-05
YBL019W	YBL019W	S288C_Ator08_hmg124h_1.JPG.dat	1075.0	21.2	1.0	0.01	0.018	0.005	8.3E-06
YBL021C	YBL021C	S288C_Ator08_hmg124h_1.JPG.dat	1120.3	58.8	1.0	0.02	0.047	0.016	8.3E-05
YBL024W	YBL024W	S288C_Ator08_hmg124h_1.JPG.dat	1154.5	11.9	1.0	0.01	-0.023	0.011	3.9E-07
YBL025W	YBL025W	S288C_Ator08_hmg124h_1.JPG.dat	233.8	467.5	0.0	0.00	0.000	0.000	
YBL027W	YBL027W	S288C_Ator08_hmg124h_1.JPG.dat	1021.0	89.4	0.9	0.08	-0.037	0.083	2.1E-04
YBL028C	YBL028C	S288C_Ator08_hmg124h_1.JPG.dat	1181.0	48.2	1.0	0.04	-0.019	0.037	1.3E-05
YBL029W	YBL029W	S288C_Ator08_hmg124h_1.JPG.dat	1168.0	33.4	1.0	0.03	-0.026	0.032	8.4E-06
YBL031W	YBL031W	S288C_Ator08_hmg124h_1.JPG.dat	1038.8	90.9	0.9	0.08	-0.090	0.079	1.9E-04
YBL032W	YBL032W	S288C_Ator08_hmg124h_1.JPG.dat	1187.5	18.5	1.0	0.01	0.081	0.008	1.4E-07
YBL036C	YBL036C	S288C_Ator08_hmg124h_1.JPG.dat	960.0	29.4	1.0	0.02	-0.010	0.022	2.9E-06
YBL037W	YBL037W	S288C_Ator08_hmg124h_1.JPG.dat	1004.8	20.1	1.0	0.01	0.068	0.007	1.5E-05
YBL039C	YBL039C	S288C_Ator08_hmg124h_1.JPG.dat	1078.5	23.2	1.0	0.02	0.016	0.020	2.1E-06
YBL042C	YBL042C	S288C_Ator08_hmg124h_1.JPG.dat	1115.3	54.8	1.0	0.04	0.019	0.042	2.0E-05
YBL043W	YBL043W	S288C_Ator08_hmg124h_1.JPG.dat	1205.8	16.3	1.1	0.02	0.056	0.015	8.4E-07
YBL046W	YBL046W	S288C_Ator08_hmg124h_1.JPG.dat	1090.5	87.9	1.0	0.08	0.001	0.081	1.5E-04
YBL047C	YBL047C	S288C_Ator08_hmg124h_1.JPG.dat	1120.0	10.7	1.0	0.00	-0.017	0.003	2.1E-06
YBL048W	YBL048W	S288C_Ator08_hmg124h_1.JPG.dat	1135.0	28.8	1.0	0.03	-0.013	0.025	4.4E-06
YBL049W	YBL049W	S288C_Ator08_hmg124h_1.JPG.dat	1167.5	24.9	1.0	0.02	-0.005	0.023	3.3E-06
YBL051C	YBL051C	S288C_Ator08_hmg124h_1.JPG.dat	1022.3	30.1	0.9	0.03	-0.060	0.028	8.1E-06
YBL052C	YBL052C	S288C_Ator08_hmg124h_1.JPG.dat	1073.0	35.5	1.0	0.04	-0.057	0.036	1.3E-05
YBL053W	YBL053W	S288C_Ator08_hmg124h_1.JPG.dat	900.5	24.7	1.0	0.01	-0.039	0.014	7.9E-07
YBL054W	YBL054W	S288C_Ator08_hmg124h_1.JPG.dat	1020.3	25.1	1.0	0.02	0.050	0.024	3.5E-06
YBL055C	YBL055C	S288C_Ator08_hmg124h_1.JPG.dat	1119.8	21.5	1.1	0.03	0.042	0.029	5.9E-06
YBL056W	YBL056W	S288C_Ator08_hmg124h_1.JPG.dat	1094.8	31.6	1.0	0.03	-0.017	0.028	5.7E-06
YBL057C	YBL057C	S288C_Ator08_hmg124h_1.JPG.dat	1145.8	45.1	1.0	0.05	0.004	0.048	2.8E-05
YBL059W	YBL059W	S288C_Ator08_hmg124h_1.JPG.dat	1107.3	28.6	1.0	0.03	0.022	0.028	5.6E-06
YBL060W	YBL060W	S288C_Ator08_hmg124h_1.JPG.dat	1078.8	24.0	1.0	0.03	0.012	0.026	4.7E-06
YBL061C	YBL061C	S288C_Ator08_hmg124h_1.JPG.dat	1034.8	63.1	1.0	0.01	-0.018	0.015	7.4E-05
YBL062W	YBL062W	S288C_Ator08_hmg124h_1.JPG.dat	1076.0	12.4	1.0	0.01	-0.037	0.006	7.6E-08
YBL063W	YBL063W	S288C_Ator08_hmg124h_1.JPG.dat	1097.8	24.7	1.0	0.02	-0.069	0.021	2.6E-06
YBL064C	YBL064C	S288C_Ator08_hmg124h_1.JPG.dat	849.0	25.4	0.8	0.03	-0.060	0.031	1.5E-05
YBL065W	YBL065W	S288C_Ator08_hmg124h_1.JPG.dat	966.0	25.9	1.0	0.04	0.039	0.041	1.7E-05
YBL066C	YBL066C	S288C_Ator08_hmg124h_1.JPG.dat	1014.8	45.5	1.0	0.05	-0.042	0.051	3.8E-05
YBL067C	YBL067C	S288C_Ator08_hmg124h_1.JPG.dat	1082.8	49.4	1.0	0.05	0.002	0.047	2.7E-05
YBL068W	YBL068W	S288C_Ator08_hmg124h_1.JPG.dat	1054.0	92.0	1.0	0.08	0.006	0.080	1.5E-04
YBL069W	YBL069W	S288C_Ator08_hmg124h_1.JPG.dat	1122.0	49.4	1.0	0.01	-0.039	0.014	6.6E-05
YBL070C	YBL070C	S288C_Ator08_hmg124h_1.JPG.dat	1090.0	35.3	1.0	0.03	-0.004	0.028	6.1E-06
YBL071C	YBL071C	S288C_Ator08_hmg124h_1.JPG.dat	1058.8	29.9	1.0	0.03	0.001	0.026	4.8E-06
YBL072C	YBL072C	S288C_Ator08_hmg124h_1.JPG.dat	1008.0	91.9	1.0	0.03	-0.015	0.027	2.7E-04
YBL075C	YBL075C	S288C_Ator08_hmg124h_1.JPG.dat	1134.5	32.0	1.0	0.03	0.024	0.030	7.1E-06
YBL078C	YBL078C	S288C_Ator08_hmg124h_1.JPG.dat	1154.8	12.3	1.0	0.01	0.018	0.010	2.3E-07
YBL079W	YBL079W	S288C_Ator08_hmg124h_1.JPG.dat	1085.5	45.2	1.0	0.01	-0.004	0.005	8.9E-06
YBL081W	YBL081W	S288C_Ator08_hmg124h_1.JPG.dat	937.0	21.3	1.0	0.02	0.018	0.024	3.8E-06
YBL082C	YBL082C	S288C_Ator08_hmg124h_1.JPG.dat	1102.0	37.1	1.1	0.04	0.011	0.043	1.8E-05
YBL083C	YBL083C	S288C_Ator08_hmg124h_1.JPG.dat	1095.5	84.4	1.0	0.08	-0.005	0.076	1.1E-04
YBL085W	YBL085W	S288C_Ator08_hmg124h_1.JPG.dat	1030.8	46.3	1.0	0.04	-0.029	0.041	2.1E-05
YBL086C	YBL086C	S288C_Ator08_hmg124h_1.JPG.dat	1058.0	63.5	1.0	0.06	0.012	0.058	5.7E-05
YBL087C	YBL087C	S288C_Ator08_hmg124h_1.JPG.dat	1003.8	25.8	1.0	0.02	0.119	0.022	3.2E-06
YBL088C	YBL088C	S288C_Ator08_hmg124h_1.JPG.dat	1073.8	21.5	1.0	0.02	-0.006	0.019	1.7E-06
YBL089W	YBL089W	S288C_Ator08_hmg124h_1.JPG.dat	1113.3	10.7	1.0	0.00	0.024	0.004	4.4E-06
YBL091C	YBL091C	S288C_Ator08_hmg124h_1.JPG.dat	1066.8	102.3	1.0	0.09	-0.070	0.093	2.4E-04
YBL091C-A	YBL091C-A	S288C_Ator08_hmg124h_1.JPG.dat	1116.0	38.8	1.0	0.04	-0.067	0.035	1.2E-05
YBL094C	YBL094C	S288C_Ator08_hmg124h_1.JPG.dat	1024.8	40.9	1.0	0.04	-0.017	0.042	2.3E-05
YBL095W	YBL095W	S288C_Ator08_hmg124h_1.JPG.dat	1045.8	29.4	1.1	0.02	0.048	0.024	2.9E-06
YBL096C	YBL096C	S288C_Ator08_hmg124h_1.JPG.dat	1088.5	42.2	1.0	0.04	-0.027	0.037	1.3E-05
YBL098W	YBL098W	S288C_Ator08_hmg124h_1.JPG.dat	1133.3	5.3	1.0	0.00	0.044	0.001	5.2E-07
YBL100C	YBL100C	S288C_Ator08_hmg124h_1.JPG.dat	842.0	561.6	1.0	0.01	0.048	0.015	6.8E-05
YBL101C	YBL101C	S288C_Ator08_hmg124h_1.JPG.dat	1059.8	32.4	1.0	0.03	-0.016	0.032	9.5E-06
YBL102W	YBL102W	S288C_Ator08_hmg124h_1.JPG.dat	1036.3	21.3	1.0	0.01	-0.013	0.005	1.0E-05
YBL103C	YBL103C	S288C_Ator08_hmg124h_1.JPG.dat	1061.5	27.9	1.0	0.02	-0.012	0.021	2.8E-06
YBL104C	YBL104C	S288C_Ator08_hmg124h_1.JPG.dat	1139.3	31.3	1.0	0.03	0.018	0.025	4.2E-06
YBL106C	YBL106C	S288C_Ator08_hmg124h_1.JPG.dat	1081.8	58.7	1.0	0.05	-0.028	0.053	4.5E-05
YBL107C	YBL107C	S288C_Ator08_hmg124h_1.JPG.dat	1134.8	21.6	1.0	0.02	-0.021	0.019	1.7E-06

YBR001C	YBR001C	S288C_Ator08_hmg124h_1.JPG.dat	1099.5	27.1	1.0	0.02	0.015	0.022	2.8E-06
YBR005W	YBR005W	S288C_Ator08_hmg124h_1.JPG.dat	1035.5	48.4	1.0	0.04	-0.008	0.038	1.5E-05
YBR006W	YBR006W	S288C_Ator08_hmg124h_1.JPG.dat	1106.8	55.6	1.0	0.01	0.060	0.010	2.8E-05
YBR007C	YBR007C	S288C_Ator08_hmg124h_1.JPG.dat	1055.3	39.5	1.0	0.04	-0.040	0.042	2.4E-05
YBR008C	YBR008C	S288C_Ator08_hmg124h_1.JPG.dat	1108.8	45.4	1.0	0.01	0.008	0.013	5.2E-05
YBR009C	YBR009C	S288C_Ator08_hmg124h_1.JPG.dat	1127.8	40.7	1.0	0.04	0.011	0.037	1.3E-05
YBR010W	YBR010W	S288C_Ator08_hmg124h_1.JPG.dat	1124.5	50.2	1.0	0.05	-0.011	0.054	4.1E-05
YBR012C	YBR012C	S288C_Ator08_hmg124h_1.JPG.dat	1188.0	9.4	1.1	0.01	0.011	0.005	8.1E-06
YBR013C	YBR013C	S288C_Ator08_hmg124h_1.JPG.dat	1201.8	26.4	1.0	0.01	0.011	0.005	7.9E-06
YBR014C	YBR014C	S288C_Ator08_hmg124h_1.JPG.dat	1125.0	10.2	1.0	0.01	-0.001	0.013	6.6E-07
YBR015C	YBR015C	S288C_Ator08_hmg124h_1.JPG.dat	1121.5	29.1	1.0	0.02	0.045	0.019	1.9E-06
YBR016W	YBR016W	S288C_Ator08_hmg124h_1.JPG.dat	1053.5	60.5	1.0	0.05	0.031	0.055	4.8E-05
YBR018C	YBR018C	S288C_Ator08_hmg124h_1.JPG.dat	980.3	10.3	0.9	0.04	-0.076	0.035	1.5E-05
YBR019C	YBR019C	S288C_Ator08_hmg124h_1.JPG.dat	1021.3	38.8	0.9	0.06	-0.071	0.055	5.6E-05
YBR020W	YBR020W	S288C_Ator08_hmg124h_1.JPG.dat	1127.3	64.2	1.0	0.06	0.010	0.062	6.2E-05
YBR022W	YBR022W	S288C_Ator08_hmg124h_1.JPG.dat	1193.0	75.6	1.0	0.05	0.002	0.047	2.5E-05
YBR023C	YBR023C	S288C_Ator08_hmg124h_1.JPG.dat	1157.0	49.9	1.0	0.02	-0.027	0.023	3.3E-06
YBR024W	YBR024W	S288C_Ator08_hmg124h_1.JPG.dat	1233.8	23.8	1.0	0.02	0.040	0.023	2.9E-06
YBR025C	YBR025C	S288C_Ator08_hmg124h_1.JPG.dat	1226.5	36.0	1.0	0.02	0.037	0.023	3.0E-06
YBR026C	YBR026C	S288C_Ator08_hmg124h_1.JPG.dat	1120.8	69.4	1.0	0.06	0.045	0.057	5.7E-05
YBR027C	YBR027C	S288C_Ator08_hmg124h_1.JPG.dat	1204.8	29.8	1.0	0.01	-0.058	0.012	4.3E-07
YBR028C	YBR028C	S288C_Ator08_hmg124h_1.JPG.dat	1204.5	46.5	1.0	0.03	0.012	0.026	4.6E-06
YBR030W	YBR030W	S288C_Ator08_hmg124h_1.JPG.dat	1187.3	54.0	1.1	0.01	0.137	0.005	8.4E-06
YBR031W	YBR031W	S288C_Ator08_hmg124h_1.JPG.dat	1035.3	56.2	1.0	0.06	0.107	0.062	5.9E-05
YBR032W	YBR032W	S288C_Ator08_hmg124h_1.JPG.dat	1012.5	13.7	1.0	0.01	-0.006	0.013	7.5E-07
YBR033W	YBR033W	S288C_Ator08_hmg124h_1.JPG.dat	1108.5	8.0	1.0	0.00	-0.047	0.004	5.4E-06
YBR034C	YBR034C	S288C_Ator08_hmg124h_1.JPG.dat	1167.0	35.8	1.0	0.03	0.001	0.027	4.9E-06
YBR036C	YBR036C	S288C_Ator08_hmg124h_1.JPG.dat	309.8	101.0	0.2	0.02	-0.417	0.021	2.7E-03
YBR040W	YBR040W	S288C_Ator08_hmg124h_1.JPG.dat	1101.0	101.6	1.0	0.08	-0.040	0.079	1.4E-04
YBR041W	YBR041W	S288C_Ator08_hmg124h_1.JPG.dat	1096.0	99.2	1.0	0.01	-0.014	0.009	2.9E-05
YBR042C	YBR042C	S288C_Ator08_hmg124h_1.JPG.dat	1067.0	99.9	0.9	0.08	-0.084	0.080	1.8E-04
YBR043C	YBR043C	S288C_Ator08_hmg124h_1.JPG.dat	1174.0	5.4	1.0	0.01	-0.024	0.007	7.5E-08
YBR044C	YBR044C	S288C_Ator08_hmg124h_1.JPG.dat	1140.0	45.3	1.0	0.03	0.005	0.033	1.1E-05
YBR045C	YBR045C	S288C_Ator08_hmg124h_1.JPG.dat	1161.0	43.1	1.1	0.07	0.117	0.071	7.7E-05
YBR046C	YBR046C	S288C_Ator08_hmg124h_1.JPG.dat	974.0	44.8	1.0	0.01	0.028	0.012	5.1E-05
YBR047W	YBR047W	S288C_Ator08_hmg124h_1.JPG.dat	1077.3	66.7	1.0	0.06	-0.012	0.059	5.1E-05
YBR048W	YBR048W	S288C_Ator08_hmg124h_1.JPG.dat	887.3	22.3	0.8	0.02	0.038	0.020	4.2E-06
YBR050C	YBR050C	S288C_Ator08_hmg124h_1.JPG.dat	1151.3	36.7	1.0	0.03	-0.024	0.035	1.1E-05
YBR051W	YBR051W	S288C_Ator08_hmg124h_1.JPG.dat	1077.3	26.2	1.0	0.03	0.032	0.032	9.5E-06
YBR052C	YBR052C	S288C_Ator08_hmg124h_1.JPG.dat	1092.5	50.0	1.0	0.04	0.012	0.045	2.5E-05
YBR053C	YBR053C	S288C_Ator08_hmg124h_1.JPG.dat	1032.8	106.4	1.0	0.09	-0.022	0.090	2.3E-04
YBR054W	YBR054W	S288C_Ator08_hmg124h_1.JPG.dat	1080.3	42.0	1.0	0.04	-0.015	0.042	2.2E-05
YBR056W	YBR056W	S288C_Ator08_hmg124h_1.JPG.dat	1154.3	36.5	1.0	0.01	0.017	0.009	2.5E-05
YBR057C	YBR057C	S288C_Ator08_hmg124h_1.JPG.dat	1175.0	44.9	1.1	0.01	0.023	0.009	2.2E-05
YBR058C	YBR058C	S288C_Ator08_hmg124h_1.JPG.dat	1013.8	17.6	1.0	0.02	-0.016	0.021	2.7E-06
YBR059C	YBR059C	S288C_Ator08_hmg124h_1.JPG.dat	910.5	53.2	1.0	0.02	0.049	0.016	9.3E-05
YBR061C	YBR061C	S288C_Ator08_hmg124h_1.JPG.dat	994.0	69.3	1.0	0.06	-0.017	0.055	5.4E-05
YBR062C	YBR062C	S288C_Ator08_hmg124h_1.JPG.dat	1098.5	46.2	1.0	0.04	-0.013	0.044	2.2E-05
YBR063C	YBR063C	S288C_Ator08_hmg124h_1.JPG.dat	1135.5	46.2	1.0	0.04	0.005	0.040	1.7E-05
YBR064W	YBR064W	S288C_Ator08_hmg124h_1.JPG.dat	1050.3	92.7	1.0	0.02	0.006	0.022	1.6E-04
YBR065C	YBR065C	S288C_Ator08_hmg124h_1.JPG.dat	1162.0	21.7	1.1	0.01	0.054	0.012	3.6E-07
YBR066C	YBR066C	S288C_Ator08_hmg124h_1.JPG.dat	1088.3	11.1	1.0	0.02	0.010	0.017	1.3E-06
YBR067C	YBR067C	S288C_Ator08_hmg124h_1.JPG.dat	1074.3	28.4	1.0	0.00	-0.033	0.005	7.4E-06
YBR068C	YBR068C	S288C_Ator08_hmg124h_1.JPG.dat	1066.8	68.4	1.0	0.06	-0.041	0.057	5.7E-05
YBR069C	YBR069C	S288C_Ator08_hmg124h_1.JPG.dat	1119.8	26.1	1.0	0.02	-0.024	0.016	1.1E-06
YBR071W	YBR071W	S288C_Ator08_hmg124h_1.JPG.dat	992.3	17.5	1.0	0.01	-0.049	0.007	1.9E-05
YBR072W	YBR072W	S288C_Ator08_hmg124h_1.JPG.dat	937.5	39.2	1.0	0.05	-0.064	0.050	3.8E-05
YBR073W	YBR073W	S288C_Ator08_hmg124h_1.JPG.dat	1043.3	26.0	1.0	0.02	0.004	0.021	2.7E-06
YBR074W	YBR074W	S288C_Ator08_hmg124h_1.JPG.dat	1074.8	73.7	1.1	0.01	0.056	0.012	4.4E-05
YBR075W	YBR075W	S288C_Ator08_hmg124h_1.JPG.dat	1081.8	34.5	1.0	0.03	0.007	0.034	1.0E-05
YBR076W	YBR076W	S288C_Ator08_hmg124h_1.JPG.dat	1074.5	13.6	1.0	0.00	0.060	0.002	7.9E-07
YBR077C	YBR077C	S288C_Ator08_hmg124h_1.JPG.dat	1002.8	61.8	0.9	0.06	-0.210	0.058	6.5E-05
YBR082C	YBR082C	S288C_Ator08_hmg124h_1.JPG.dat	977.0	75.2	0.9	0.07	-0.064	0.074	1.4E-04
YBR083W	YBR083W	S288C_Ator08_hmg124h_1.JPG.dat	1060.0	70.8	1.0	0.07	-0.028	0.067	8.6E-05
YBR084C-A	YBR084C-A	S288C_Ator08_hmg124h_1.JPG.dat	1004.3	53.8	0.9	0.02	0.054	0.016	1.0E-04
YBR084W	YBR084W	S288C_Ator08_hmg124h_1.JPG.dat	1076.5	31.0	1.0	0.04	-0.051	0.036	1.3E-05
YBR090C	YBR090C	S288C_Ator08_hmg124h_1.JPG.dat	1008.8	32.9	1.0	0.01	-0.039	0.014	7.2E-07
YBR090C-A	YBR090C-A	S288C_Ator08_hmg124h_1.JPG.dat	1058.8	136.7	1.1	0.12	0.082	0.123	4.1E-04
YBR092C	YBR092C	S288C_Ator08_hmg124h_1.JPG.dat	1150.5	33.5	1.1	0.03	0.050	0.029	5.3E-06
YBR093C	YBR093C	S288C_Ator08_hmg124h_1.JPG.dat	848.0	98.4	0.8	0.10	-0.056	0.097	4.9E-04

YBR094W	YBR094W	S288C_Ator08_hmg124h_1.JPG.dat	1036.0	57.5	1.0	0.06	0.025	0.056	5.4E-05
YBR095C	YBR095C	S288C_Ator08_hmg124h_1.JPG.dat	1067.5	24.1	1.0	0.03	0.066	0.025	4.2E-06
YBR098W	YBR098W	S288C_Ator08_hmg124h_1.JPG.dat	1015.5	30.2	1.0	0.02	-0.009	0.024	4.6E-06
YBR099C	YBR099C	S288C_Ator08_hmg124h_1.JPG.dat	1031.5	31.5	1.0	0.02	-0.005	0.025	4.7E-06
YBR100W	YBR100W	S288C_Ator08_hmg124h_1.JPG.dat	1007.5	65.5	0.9	0.02	-0.102	0.022	2.1E-04
YBR101C	YBR101C	S288C_Ator08_hmg124h_1.JPG.dat	1140.0	30.9	1.0	0.03	0.000	0.025	3.9E-06
YBR103W	YBR103W	S288C_Ator08_hmg124h_1.JPG.dat	932.8	61.0	0.9	0.05	-0.066	0.049	4.8E-05
YBR104W	YBR104W	S288C_Ator08_hmg124h_1.JPG.dat	1023.5	32.1	1.0	0.02	-0.087	0.020	2.0E-06
YBR105C	YBR105C	S288C_Ator08_hmg124h_1.JPG.dat	889.8	172.0	0.8	0.17	-0.125	0.174	2.3E-03
YBR106W	YBR106W	S288C_Ator08_hmg124h_1.JPG.dat	0.0	0.0	0.0	0.00	0.000	0.000	
YBR107C	YBR107C	S288C_Ator08_hmg124h_1.JPG.dat	1052.8	29.4	1.0	0.02	-0.091	0.021	2.7E-06
YBR108W	YBR108W	S288C_Ator08_hmg124h_1.JPG.dat	1097.0	13.4	1.0	0.01	-0.016	0.009	2.2E-07
YBR111C	YBR111C	S288C_Ator08_hmg124h_1.JPG.dat	1034.0	70.7	1.0	0.01	-0.007	0.012	4.8E-05
YBR113W	YBR113W	S288C_Ator08_hmg124h_1.JPG.dat	1096.0	59.0	1.0	0.00	-0.001	0.003	2.2E-06
YBR114W	YBR114W	S288C_Ator08_hmg124h_1.JPG.dat	1157.5	8.2	1.0	0.01	-0.006	0.010	2.7E-07
YBR115C	YBR115C	S288C_Ator08_hmg124h_1.JPG.dat	598.8	691.4	0.5	0.62	0.050	0.615	1.8E-01
YBR116C	YBR116C	S288C_Ator08_hmg124h_1.JPG.dat	1152.0	18.5	1.0	0.01	0.006	0.015	8.4E-07
YBR119W	YBR119W	S288C_Ator08_hmg124h_1.JPG.dat	1106.5	35.8	1.0	0.03	0.055	0.029	6.9E-06
YBR121C	YBR121C	S288C_Ator08_hmg124h_1.JPG.dat	524.8	609.8	0.5	0.59	0.016	0.589	1.8E-01
YBR122C	YBR122C	S288C_Ator08_hmg124h_1.JPG.dat	262.0	524.0	0.0	0.00	0.000	0.000	
YBR125C	YBR125C	S288C_Ator08_hmg124h_1.JPG.dat	1012.5	57.0	0.9	0.06	-0.041	0.058	6.4E-05
YBR126C	YBR126C	S288C_Ator08_hmg124h_1.JPG.dat	994.3	48.6	0.9	0.03	-0.112	0.033	1.4E-05
YBR128C	YBR128C	S288C_Ator08_hmg124h_1.JPG.dat	1141.5	72.3	1.0	0.00	-0.022	0.005	7.4E-06
YBR129C	YBR129C	S288C_Ator08_hmg124h_1.JPG.dat	1213.0	72.9	1.0	0.04	0.030	0.043	2.1E-05
YBR130C	YBR130C	S288C_Ator08_hmg124h_1.JPG.dat	1182.0	57.2	1.0	0.03	0.029	0.026	4.8E-06
YBR131W	YBR131W	S288C_Ator08_hmg124h_1.JPG.dat	823.3	550.7	0.9	0.04	0.032	0.040	6.1E-04
YBR132C	YBR132C	S288C_Ator08_hmg124h_1.JPG.dat	1229.5	35.3	1.0	0.02	-0.017	0.022	2.7E-06
YBR134W	YBR134W	S288C_Ator08_hmg124h_1.JPG.dat	1225.0	33.0	1.0	0.02	-0.013	0.016	1.1E-06
YBR137W	YBR137W	S288C_Ator08_hmg124h_1.JPG.dat	1237.5	36.0	1.0	0.01	0.044	0.009	1.9E-07
YBR138C	YBR138C	S288C_Ator08_hmg124h_1.JPG.dat	1191.3	19.2	1.0	0.01	0.031	0.008	2.2E-05
YBR139W	YBR139W	S288C_Ator08_hmg124h_1.JPG.dat	971.0	52.0	1.0	0.06	-0.036	0.056	4.9E-05
YBR141C	YBR141C	S288C_Ator08_hmg124h_1.JPG.dat	1051.3	51.1	1.0	0.04	0.014	0.042	2.0E-05
YBR144C	YBR144C	S288C_Ator08_hmg124h_1.JPG.dat	1016.3	54.2	1.0	0.01	0.000	0.015	7.6E-05
YBR145W	YBR145W	S288C_Ator08_hmg124h_1.JPG.dat	1137.5	56.7	1.0	0.05	0.033	0.054	4.0E-05
YBR146W	YBR146W	S288C_Ator08_hmg124h_1.JPG.dat	1172.3	34.8	1.0	0.04	0.053	0.037	1.4E-05
YBR147W	YBR147W	S288C_Ator08_hmg124h_1.JPG.dat	1016.0	175.4	1.0	0.05	0.038	0.047	7.5E-04
YBR148W	YBR148W	S288C_Ator08_hmg124h_1.JPG.dat	1142.8	36.8	1.0	0.03	0.004	0.028	5.9E-06
YBR149W	YBR149W	S288C_Ator08_hmg124h_1.JPG.dat	1148.0	49.6	1.0	0.04	0.011	0.036	1.3E-05
YBR150C	YBR150C	S288C_Ator08_hmg124h_1.JPG.dat	1161.3	18.6	1.0	0.02	-0.035	0.020	2.2E-06
YBR151W	YBR151W	S288C_Ator08_hmg124h_1.JPG.dat	1172.3	37.3	1.0	0.03	-0.030	0.029	6.4E-06
YBR156C	YBR156C	S288C_Ator08_hmg124h_1.JPG.dat	1140.3	25.6	1.0	0.03	-0.002	0.030	6.9E-06
YBR157C	YBR157C	S288C_Ator08_hmg124h_1.JPG.dat	925.8	35.5	1.0	0.03	0.003	0.033	1.0E-05
YBR158W	YBR158W	S288C_Ator08_hmg124h_1.JPG.dat	1030.8	62.8	1.0	0.06	0.067	0.061	6.1E-05
YBR159W	YBR159W	S288C_Ator08_hmg124h_1.JPG.dat	1094.5	60.7	1.0	0.06	-0.011	0.064	6.7E-05
YBR161W	YBR161W	S288C_Ator08_hmg124h_1.JPG.dat	1157.3	36.7	1.0	0.01	-0.026	0.009	2.3E-05
YBR162C	YBR162C	S288C_Ator08_hmg124h_1.JPG.dat	1168.8	63.8	1.1	0.02	0.025	0.018	9.1E-05
YBR162W-	YBR162W-A	S288C_Ator08_hmg124h_1.JPG.dat	1015.0	74.4	0.9	0.07	-0.045	0.069	1.1E-04
YBR164C	YBR164C	S288C_Ator08_hmg124h_1.JPG.dat	818.5	101.0	0.8	0.10	-0.216	0.096	5.4E-04
YBR165W	YBR165W	S288C_Ator08_hmg124h_1.JPG.dat	1131.3	28.0	1.0	0.03	0.057	0.026	4.8E-06
YBR166C	YBR166C	S288C_Ator08_hmg124h_1.JPG.dat	1128.0	29.4	1.0	0.03	0.016	0.026	4.7E-06
YBR168W	YBR168W	S288C_Ator08_hmg124h_1.JPG.dat	1013.8	42.9	0.9	0.04	0.076	0.037	1.9E-05
YBR169C	YBR169C	S288C_Ator08_hmg124h_1.JPG.dat	1096.5	22.8	1.0	0.03	-0.020	0.025	4.2E-06
YBR170C	YBR170C	S288C_Ator08_hmg124h_1.JPG.dat	922.8	76.1	1.0	0.07	-0.025	0.073	1.1E-04
YBR171W	YBR171W	S288C_Ator08_hmg124h_1.JPG.dat	1079.0	56.1	1.1	0.05	-0.098	0.045	2.2E-05
YBR172C	YBR172C	S288C_Ator08_hmg124h_1.JPG.dat	1078.0	19.1	1.0	0.00	0.040	0.003	2.9E-06
YBR174C	YBR174C	S288C_Ator08_hmg124h_1.JPG.dat	1007.0	30.5	0.9	0.01	0.024	0.007	2.1E-05
YBR175W	YBR175W	S288C_Ator08_hmg124h_1.JPG.dat	0.0	0.0	0.0	0.00	0.000	0.000	
YBR176W	YBR176W	S288C_Ator08_hmg124h_1.JPG.dat	1026.5	81.3	0.9	0.08	-0.097	0.077	1.5E-04
YBR177C	YBR177C	S288C_Ator08_hmg124h_1.JPG.dat	1070.8	33.1	1.0	0.03	0.003	0.027	5.3E-06
YBR178W	YBR178W	S288C_Ator08_hmg124h_1.JPG.dat	1103.0	38.9	1.0	0.03	0.008	0.029	7.0E-06
YBR180W	YBR180W	S288C_Ator08_hmg124h_1.JPG.dat	1174.8	24.9	1.0	0.01	0.030	0.010	3.1E-05
YBR181C	YBR181C	S288C_Ator08_hmg124h_1.JPG.dat	796.5	25.9	0.7	0.03	-0.059	0.029	1.8E-05
YBR182C	YBR182C	S288C_Ator08_hmg124h_1.JPG.dat	1097.8	49.9	1.0	0.04	-0.028	0.038	1.4E-05
YBR183W	YBR183W	S288C_Ator08_hmg124h_1.JPG.dat	945.8	27.6	1.0	0.03	0.049	0.032	8.7E-06
YBR184W	YBR184W	S288C_Ator08_hmg124h_1.JPG.dat	972.0	41.2	1.0	0.04	-0.046	0.041	2.1E-05
YBR185C	YBR185C	S288C_Ator08_hmg124h_1.JPG.dat	1049.0	15.9	1.0	0.02	-0.031	0.017	1.4E-06
YBR186W	YBR186W	S288C_Ator08_hmg124h_1.JPG.dat	1059.3	25.7	1.0	0.01	0.033	0.006	1.3E-05
YBR187W	YBR187W	S288C_Ator08_hmg124h_1.JPG.dat	1102.0	30.5	1.0	0.01	0.038	0.011	3.5E-05
YBR188C	YBR188C	S288C_Ator08_hmg124h_1.JPG.dat	1081.5	54.7	1.0	0.05	0.000	0.055	4.1E-05
YBR189W	YBR189W	S288C_Ator08_hmg124h_1.JPG.dat	793.0	314.7	0.8	0.30	-0.067	0.300	1.5E-02

YBR194W	YBR194W	S288C_Ator08_hmg124h_1.JPG.dat	694.5	147.9	0.6	0.14	-0.085	0.136	2.5E-03
YBR195C	YBR195C	S288C_Ator08_hmg124h_1.JPG.dat	1218.5	69.5	1.1	0.06	0.123	0.065	5.5E-05
YBR197C	YBR197C	S288C_Ator08_hmg124h_1.JPG.dat	1127.0	56.0	1.0	0.05	0.031	0.052	3.6E-05
YBR199W	YBR199W	S288C_Ator08_hmg124h_1.JPG.dat	1052.0	50.6	1.0	0.05	-0.014	0.047	2.8E-05
YBR200W	YBR200W	S288C_Ator08_hmg124h_1.JPG.dat	709.5	473.7	1.0	0.02	0.058	0.021	1.5E-04
YBR201W	YBR201W	S288C_Ator08_hmg124h_1.JPG.dat	1129.3	56.5	1.1	0.06	0.049	0.059	4.5E-05
YBR203W	YBR203W	S288C_Ator08_hmg124h_1.JPG.dat	1104.5	60.4	1.0	0.06	0.047	0.059	5.1E-05
YBR204C	YBR204C	S288C_Ator08_hmg124h_1.JPG.dat	998.8	45.6	0.9	0.05	-0.054	0.048	3.7E-05
YBR205W	YBR205W	S288C_Ator08_hmg124h_1.JPG.dat	1039.0	49.8	1.0	0.05	-0.047	0.051	3.9E-05
YBR206W	YBR206W	S288C_Ator08_hmg124h_1.JPG.dat	1063.0	21.9	1.0	0.03	0.008	0.029	6.3E-06
YBR207W	YBR207W	S288C_Ator08_hmg124h_1.JPG.dat	1026.0	56.0	1.0	0.05	-0.029	0.047	3.2E-05
YBR208C	YBR208C	S288C_Ator08_hmg124h_1.JPG.dat	1119.3	48.8	1.0	0.04	-0.009	0.039	1.5E-05
YBR209W	YBR209W	S288C_Ator08_hmg124h_1.JPG.dat	1087.8	44.4	1.0	0.04	-0.023	0.041	1.9E-05
YBR210W	YBR210W	S288C_Ator08_hmg124h_1.JPG.dat	1134.3	23.6	1.0	0.02	0.058	0.024	3.3E-06
YBR212W	YBR212W	S288C_Ator08_hmg124h_1.JPG.dat	1025.0	49.5	1.0	0.05	-0.060	0.046	2.8E-05
YBR213W	YBR213W	S288C_Ator08_hmg124h_1.JPG.dat	1041.3	82.3	1.0	0.07	0.021	0.068	7.9E-05
YBR214W	YBR214W	S288C_Ator08_hmg124h_1.JPG.dat	1079.5	47.8	1.0	0.02	-0.070	0.015	8.2E-05
YBR215W	YBR215W	S288C_Ator08_hmg124h_1.JPG.dat	1102.3	22.8	1.0	0.00	0.021	0.004	4.4E-06
YBR216C	YBR216C	S288C_Ator08_hmg124h_1.JPG.dat	1126.8	57.1	1.0	0.06	0.005	0.060	5.4E-05
YBR217W	YBR217W	S288C_Ator08_hmg124h_1.JPG.dat	1090.8	20.1	1.0	0.01	-0.040	0.008	2.3E-05
YBR218C	YBR218C	S288C_Ator08_hmg124h_1.JPG.dat	1040.0	61.3	1.0	0.04	-0.007	0.045	2.9E-05
YBR219C	YBR219C	S288C_Ator08_hmg124h_1.JPG.dat	1154.0	49.8	1.1	0.01	0.054	0.008	2.1E-05
YBR220C	YBR220C	S288C_Ator08_hmg124h_1.JPG.dat	1189.8	26.1	1.0	0.03	0.033	0.030	6.4E-06
YBR221C	YBR221C	S288C_Ator08_hmg124h_1.JPG.dat	1112.3	49.1	1.0	0.05	0.030	0.045	2.7E-05
YBR222C	YBR222C	S288C_Ator08_hmg124h_1.JPG.dat	1119.8	72.7	1.0	0.02	0.048	0.019	1.2E-04
YBR223C	YBR223C	S288C_Ator08_hmg124h_1.JPG.dat	1100.8	50.4	1.0	0.01	-0.060	0.010	3.1E-05
YOR202W	YOR202W	S288C_Ator08_hmg124h_1.JPG.dat	1086.7	148.3	1.0	0.08	0.001	0.082	8.8E-301
1	1	S288C_Ator08_hmg124h_11.JPG.dat	1247.8	151.4	1.1	0.02	0.060	0.024	1.8E-04
2	2	S288C_Ator08_hmg124h_11.JPG.dat	1272.8	79.5	1.1	0.05	-0.148	0.046	2.2E-05
3	3	S288C_Ator08_hmg124h_11.JPG.dat	1074.0	94.7	0.9	0.07	-0.146	0.066	1.0E-04
4	4	S288C_Ator08_hmg124h_11.JPG.dat	1126.0	75.4	1.0	0.05	-0.116	0.052	4.0E-05
YJR109C	YJR109C	S288C_Ator08_hmg124h_11.JPG.dat	0.0	0.0	0.0	0.00	0.000	0.000	
YMR244W	YMR244W	S288C_Ator08_hmg124h_11.JPG.dat	1072.5	27.7	1.0	0.02	-0.060	0.022	3.0E-06
YMR245W	YMR245W	S288C_Ator08_hmg124h_11.JPG.dat	1197.5	41.5	1.1	0.03	0.022	0.032	8.0E-06
YMR246W	YMR246W	S288C_Ator08_hmg124h_11.JPG.dat	1219.0	37.5	1.1	0.02	0.045	0.024	3.1E-06
YMR247C	YMR247C	S288C_Ator08_hmg124h_11.JPG.dat	1133.5	76.2	1.0	0.06	-0.049	0.064	7.9E-05
YMR250W	YMR250W	S288C_Ator08_hmg124h_11.JPG.dat	1197.5	22.5	1.0	0.02	0.026	0.019	1.8E-06
YMR251W	YMR251W	S288C_Ator08_hmg124h_11.JPG.dat	1186.0	27.6	1.0	0.00	0.000	0.003	3.5E-06
YMR251W	YMR251W-A	S288C_Ator08_hmg124h_11.JPG.dat	1180.8	17.4	1.0	0.02	0.000	0.015	1.0E-06
YMR252C	YMR252C	S288C_Ator08_hmg124h_11.JPG.dat	1216.8	5.1	1.0	0.01	0.021	0.013	5.2E-07
YMR253C	YMR253C	S288C_Ator08_hmg124h_11.JPG.dat	1235.8	31.9	1.0	0.02	0.073	0.025	3.7E-06
YMR254C	YMR254C	S288C_Ator08_hmg124h_11.JPG.dat	1201.0	17.3	1.0	0.01	0.106	0.014	6.9E-07
YMR255W	YMR255W	S288C_Ator08_hmg124h_11.JPG.dat	1178.0	55.4	1.0	0.04	0.107	0.040	1.6E-05
YMR256C	YMR256C	S288C_Ator08_hmg124h_11.JPG.dat	0.0	0.0	0.0	0.00	0.000	0.000	
YMR258C	YMR258C	S288C_Ator08_hmg124h_11.JPG.dat	1256.8	29.4	1.1	0.02	0.072	0.022	2.3E-06
YMR259C	YMR259C	S288C_Ator08_hmg124h_11.JPG.dat	1290.3	48.7	1.1	0.03	0.094	0.034	8.3E-06
YMR261C	YMR261C	S288C_Ator08_hmg124h_11.JPG.dat	1241.5	90.2	1.0	0.07	0.007	0.073	9.2E-05
YMR262W	YMR262W	S288C_Ator08_hmg124h_11.JPG.dat	1193.8	36.3	1.0	0.03	0.023	0.034	1.0E-05
YMR263W	YMR263W	S288C_Ator08_hmg124h_11.JPG.dat	1274.5	27.4	1.1	0.01	0.102	0.006	1.0E-05
YMR264W	YMR264W	S288C_Ator08_hmg124h_11.JPG.dat	1172.5	28.5	1.0	0.02	-0.037	0.016	1.2E-06
YMR265C	YMR265C	S288C_Ator08_hmg124h_11.JPG.dat	1242.0	35.0	1.0	0.03	0.054	0.025	4.1E-06
YMR266W	YMR266W	S288C_Ator08_hmg124h_11.JPG.dat	1249.3	16.6	1.0	0.01	0.065	0.010	2.4E-07
YMR269W	YMR269W	S288C_Ator08_hmg124h_11.JPG.dat	1184.5	32.7	1.0	0.01	0.033	0.009	2.9E-05
YMR271C	YMR271C	S288C_Ator08_hmg124h_11.JPG.dat	1159.8	49.3	1.0	0.00	0.020	0.004	4.7E-06
YMR272C	YMR272C	S288C_Ator08_hmg124h_11.JPG.dat	1066.8	96.1	0.9	0.01	-0.037	0.007	2.1E-05
YMR273C	YMR273C	S288C_Ator08_hmg124h_11.JPG.dat	1172.8	10.9	1.0	0.02	0.040	0.020	2.1E-06
YMR274C	YMR274C	S288C_Ator08_hmg124h_11.JPG.dat	1059.5	55.7	0.9	0.01	-0.088	0.011	4.6E-05
YMR275C	YMR275C	S288C_Ator08_hmg124h_11.JPG.dat	1155.8	96.4	1.0	0.00	0.016	0.002	1.3E-06
YMR276W	YMR276W	S288C_Ator08_hmg124h_11.JPG.dat	1104.8	62.6	1.0	0.05	-0.032	0.054	4.9E-05
YMR278W	YMR278W	S288C_Ator08_hmg124h_11.JPG.dat	1216.5	46.3	1.1	0.05	0.040	0.048	2.6E-05
YMR279C	YMR279C	S288C_Ator08_hmg124h_11.JPG.dat	1245.5	41.3	1.1	0.00	0.059	0.003	2.1E-06
YMR280C	YMR280C	S288C_Ator08_hmg124h_11.JPG.dat	1253.8	6.7	1.1	0.02	0.001	0.021	2.3E-06
YMR282C	YMR282C	S288C_Ator08_hmg124h_11.JPG.dat	286.0	572.0	0.0	0.00	0.000	0.000	
YMR283C	YMR283C	S288C_Ator08_hmg124h_11.JPG.dat	1160.8	22.6	1.0	0.01	0.027	0.010	3.0E-07
YMR284W	YMR284W	S288C_Ator08_hmg124h_11.JPG.dat	1175.0	75.0	1.1	0.07	0.010	0.068	7.4E-05
YMR285C	YMR285C	S288C_Ator08_hmg124h_11.JPG.dat	1137.3	21.9	1.0	0.03	0.045	0.026	4.4E-06
YMR289W	YMR289W	S288C_Ator08_hmg124h_11.JPG.dat	1141.3	48.9	1.0	0.01	-0.001	0.008	1.9E-05
YMR291W	YMR291W	S288C_Ator08_hmg124h_11.JPG.dat	1181.3	25.9	1.1	0.03	0.036	0.026	3.9E-06
YMR292W	YMR292W	S288C_Ator08_hmg124h_11.JPG.dat	1151.0	10.7	1.0	0.01	0.055	0.015	7.8E-07
YMR294W	YMR294W	S288C_Ator08_hmg124h_11.JPG.dat	1074.8	27.6	1.0	0.00	-0.082	0.004	5.6E-06

YMR294W	YMR294W-A	S288C_Ator08_hmg124h_11.JPG.dat	1038.5	21.7	1.0	0.01	-0.102	0.006	1.5E-05
YMR295C	YMR295C	S288C_Ator08_hmg124h_11.JPG.dat	936.0	98.6	0.8	0.09	-0.207	0.089	3.4E-04
YMR297W	YMR297W	S288C_Ator08_hmg124h_11.JPG.dat	1232.8	15.5	1.1	0.02	0.060	0.015	7.6E-07
YMR299C	YMR299C	S288C_Ator08_hmg124h_11.JPG.dat	1097.8	23.2	1.0	0.02	-0.044	0.021	2.7E-06
YMR300C	YMR300C	S288C_Ator08_hmg124h_11.JPG.dat	1141.0	12.2	1.1	0.00	0.017	0.005	6.2E-06
YMR302C	YMR302C	S288C_Ator08_hmg124h_11.JPG.dat	1058.8	34.3	1.0	0.03	0.015	0.026	4.8E-06
YMR303C	YMR303C	S288C_Ator08_hmg124h_11.JPG.dat	1123.5	28.2	1.0	0.03	0.040	0.031	7.6E-06
YMR304C-.	YMR304C-A	S288C_Ator08_hmg124h_11.JPG.dat	1198.5	15.8	1.1	0.02	0.040	0.021	2.1E-06
YMR304W	YMR304W	S288C_Ator08_hmg124h_11.JPG.dat	1119.8	26.8	1.0	0.03	0.041	0.027	5.5E-06
YMR305C	YMR305C	S288C_Ator08_hmg124h_11.JPG.dat	1179.3	32.8	1.1	0.03	0.035	0.025	3.7E-06
YMR306C-.	YMR306C-A	S288C_Ator08_hmg124h_11.JPG.dat	1075.0	54.7	0.9	0.02	-0.009	0.019	1.4E-04
YMR306W	YMR306W	S288C_Ator08_hmg124h_11.JPG.dat	1214.0	40.2	1.1	0.03	0.098	0.033	7.5E-06
YMR307W	YMR307W	S288C_Ator08_hmg124h_11.JPG.dat	932.5	90.0	0.8	0.08	-0.139	0.081	2.4E-04
YMR310C	YMR310C	S288C_Ator08_hmg124h_11.JPG.dat	1165.3	28.4	1.0	0.01	0.045	0.007	1.6E-05
YMR311C	YMR311C	S288C_Ator08_hmg124h_11.JPG.dat	993.3	33.8	0.9	0.04	-0.081	0.036	1.6E-05
YMR312W	YMR312W	S288C_Ator08_hmg124h_11.JPG.dat	947.0	33.6	0.9	0.03	0.040	0.035	1.5E-05
YMR313C	YMR313C	S288C_Ator08_hmg124h_11.JPG.dat	973.3	93.4	1.0	0.09	-0.063	0.087	2.0E-04
YMR315W	YMR315W	S288C_Ator08_hmg124h_11.JPG.dat	1207.3	7.2	1.0	0.01	0.091	0.006	6.2E-08
YMR316C-.	YMR316C-A	S288C_Ator08_hmg124h_11.JPG.dat	1183.8	25.2	1.0	0.02	-0.005	0.024	4.0E-06
YMR316C-	YMR316C-B	S288C_Ator08_hmg124h_11.JPG.dat	1189.3	57.2	1.0	0.05	-0.016	0.045	2.5E-05
YMR316W	YMR316W	S288C_Ator08_hmg124h_11.JPG.dat	1158.3	58.6	1.0	0.05	0.014	0.052	4.0E-05
YMR317W	YMR317W	S288C_Ator08_hmg124h_11.JPG.dat	1198.8	20.3	1.0	0.02	0.019	0.020	2.1E-06
YMR318C	YMR318C	S288C_Ator08_hmg124h_11.JPG.dat	1209.5	16.8	1.0	0.02	0.032	0.017	1.1E-06
YMR319C	YMR319C	S288C_Ator08_hmg124h_11.JPG.dat	1217.3	19.8	1.1	0.01	0.032	0.013	5.6E-07
YMR320W	YMR320W	S288C_Ator08_hmg124h_11.JPG.dat	1203.3	11.1	1.1	0.02	0.039	0.016	1.0E-06
YMR322C	YMR322C	S288C_Ator08_hmg124h_11.JPG.dat	1142.8	48.5	1.0	0.04	0.001	0.039	1.5E-05
YMR326C	YMR326C	S288C_Ator08_hmg124h_11.JPG.dat	1110.0	32.7	1.0	0.04	-0.073	0.035	1.2E-05
YNL001W	YNL001W	S288C_Ator08_hmg124h_11.JPG.dat	1075.8	35.9	1.0	0.03	-0.055	0.031	9.0E-06
YNL003C	YNL003C	S288C_Ator08_hmg124h_11.JPG.dat	1119.5	65.6	0.9	0.06	-0.108	0.059	6.7E-05
YNL004W	YNL004W	S288C_Ator08_hmg124h_11.JPG.dat	1187.0	126.0	0.9	0.02	0.017	0.016	9.7E-05
YNL008C	YNL008C	S288C_Ator08_hmg124h_11.JPG.dat	1180.0	27.0	1.0	0.01	0.013	0.008	2.4E-05
YNL009W	YNL009W	S288C_Ator08_hmg124h_11.JPG.dat	1179.5	61.9	1.0	0.05	0.021	0.054	4.4E-05
YNL010W	YNL010W	S288C_Ator08_hmg124h_11.JPG.dat	1204.0	31.6	1.0	0.03	-0.023	0.027	5.4E-06
YNL012W	YNL012W	S288C_Ator08_hmg124h_11.JPG.dat	1193.3	57.5	1.0	0.04	-0.031	0.045	2.4E-05
YNL013C	YNL013C	S288C_Ator08_hmg124h_11.JPG.dat	1219.8	17.7	1.1	0.01	0.036	0.010	2.6E-07
YNL015W	YNL015W	S288C_Ator08_hmg124h_11.JPG.dat	1086.5	78.3	1.0	0.06	-0.058	0.064	8.2E-05
YNL016W	YNL016W	S288C_Ator08_hmg124h_11.JPG.dat	1033.0	53.2	0.9	0.05	-0.054	0.047	3.5E-05
YNL020C	YNL020C	S288C_Ator08_hmg124h_11.JPG.dat	1151.3	70.1	1.0	0.06	0.077	0.065	6.6E-05
YNL021W	YNL021W	S288C_Ator08_hmg124h_11.JPG.dat	1111.0	58.7	1.0	0.06	0.040	0.060	5.1E-05
YNL022C	YNL022C	S288C_Ator08_hmg124h_11.JPG.dat	1002.8	48.8	0.9	0.04	-0.166	0.042	2.7E-05
YNL023C	YNL023C	S288C_Ator08_hmg124h_11.JPG.dat	1059.8	75.8	1.0	0.07	-0.080	0.066	8.6E-05
YNL024C	YNL024C	S288C_Ator08_hmg124h_11.JPG.dat	1127.0	14.0	1.0	0.00	-0.008	0.001	2.9E-07
YNL027W	YNL027W	S288C_Ator08_hmg124h_11.JPG.dat	1133.5	47.0	1.0	0.04	-0.010	0.039	1.7E-05
YNL028W	YNL028W	S288C_Ator08_hmg124h_11.JPG.dat	1170.3	48.8	1.0	0.04	-0.036	0.038	1.4E-05
YNL029C	YNL029C	S288C_Ator08_hmg124h_11.JPG.dat	1158.5	11.9	1.0	0.01	-0.045	0.008	1.5E-07
YNL030W	YNL030W	S288C_Ator08_hmg124h_11.JPG.dat	1127.8	66.8	1.0	0.06	-0.032	0.058	5.9E-05
YNL031C	YNL031C	S288C_Ator08_hmg124h_11.JPG.dat	1237.0	34.6	1.0	0.03	0.016	0.029	6.1E-06
YNL032W	YNL032W	S288C_Ator08_hmg124h_11.JPG.dat	1177.3	89.0	1.0	0.08	-0.012	0.079	1.3E-04
YNL034W	YNL034W	S288C_Ator08_hmg124h_11.JPG.dat	1175.8	46.1	1.0	0.00	0.041	0.003	3.5E-06
YNL035C	YNL035C	S288C_Ator08_hmg124h_11.JPG.dat	1146.3	55.3	1.0	0.05	0.072	0.048	3.2E-05
YNL037C	YNL037C	S288C_Ator08_hmg124h_11.JPG.dat	1050.3	61.2	0.9	0.07	-0.075	0.065	9.0E-05
YNL040W	YNL040W	S288C_Ator08_hmg124h_11.JPG.dat	1134.8	35.9	1.0	0.03	-0.043	0.030	7.0E-06
YNL041C	YNL041C	S288C_Ator08_hmg124h_11.JPG.dat	1143.8	92.8	1.0	0.08	-0.027	0.078	1.3E-04
YNL043C	YNL043C	S288C_Ator08_hmg124h_11.JPG.dat	1130.5	57.8	1.0	0.05	-0.047	0.049	3.4E-05
YNL044W	YNL044W	S288C_Ator08_hmg124h_11.JPG.dat	1099.8	63.0	0.9	0.05	-0.085	0.049	4.0E-05
YNL045W	YNL045W	S288C_Ator08_hmg124h_11.JPG.dat	1125.5	57.1	1.0	0.02	-0.035	0.019	1.3E-04
YNL046W	YNL046W	S288C_Ator08_hmg124h_11.JPG.dat	1169.5	37.2	1.0	0.03	-0.003	0.034	1.1E-05
YNL049C	YNL049C	S288C_Ator08_hmg124h_11.JPG.dat	1215.3	19.2	1.0	0.02	0.014	0.016	1.2E-06
YNL050C	YNL050C	S288C_Ator08_hmg124h_11.JPG.dat	1130.8	40.6	1.0	0.03	-0.056	0.031	9.4E-06
YNL051W	YNL051W	S288C_Ator08_hmg124h_11.JPG.dat	1170.3	24.5	1.0	0.02	-0.055	0.024	4.3E-06
YNL052W	YNL052W	S288C_Ator08_hmg124h_11.JPG.dat	1186.8	39.7	1.0	0.01	0.018	0.006	1.1E-05
YNL054W	YNL054W	S288C_Ator08_hmg124h_11.JPG.dat	1091.0	69.4	1.0	0.05	0.002	0.048	3.2E-05
YNL056W	YNL056W	S288C_Ator08_hmg124h_11.JPG.dat	1001.3	74.5	0.9	0.05	-0.093	0.049	4.5E-05
YNL057W	YNL057W	S288C_Ator08_hmg124h_11.JPG.dat	1138.5	89.5	1.0	0.07	-0.015	0.071	1.0E-04
YNL058C	YNL058C	S288C_Ator08_hmg124h_11.JPG.dat	1133.0	115.4	1.0	0.08	0.002	0.082	1.5E-04
YNL063W	YNL063W	S288C_Ator08_hmg124h_11.JPG.dat	1103.3	55.3	1.0	0.03	-0.057	0.032	1.0E-05
YNL064C	YNL064C	S288C_Ator08_hmg124h_11.JPG.dat	1195.8	5.7	1.0	0.02	0.032	0.023	2.9E-06
YNL065W	YNL065W	S288C_Ator08_hmg124h_11.JPG.dat	1139.0	10.9	1.0	0.01	-0.036	0.012	5.4E-07
YNL066W	YNL066W	S288C_Ator08_hmg124h_11.JPG.dat	1207.5	96.2	1.0	0.09	-0.003	0.086	1.5E-04
YNL067W	YNL067W	S288C_Ator08_hmg124h_11.JPG.dat	1169.3	41.4	1.0	0.02	0.035	0.021	2.3E-06

YNL068C	YNL068C	S288C_Ator08_hmg124h_11.JPG.dat	1098.8	93.1	1.0	0.09	-0.051	0.087	2.0E-04
YNL069C	YNL069C	S288C_Ator08_hmg124h_11.JPG.dat	1154.5	76.9	1.1	0.07	0.022	0.067	6.9E-05
YNL070W	YNL070W	S288C_Ator08_hmg124h_11.JPG.dat	1028.0	20.7	0.9	0.02	-0.082	0.022	3.7E-06
YNL071W	YNL071W	S288C_Ator08_hmg124h_11.JPG.dat	1125.5	96.9	1.0	0.09	0.027	0.085	1.6E-04
YNL072W	YNL072W	S288C_Ator08_hmg124h_11.JPG.dat	1031.8	66.6	0.9	0.07	-0.038	0.068	1.0E-04
YNL074C	YNL074C	S288C_Ator08_hmg124h_11.JPG.dat	1107.3	83.4	1.0	0.08	0.019	0.077	1.2E-04
YNL076W	YNL076W	S288C_Ator08_hmg124h_11.JPG.dat	1096.3	72.2	1.0	0.02	-0.067	0.024	2.0E-04
YNL077W	YNL077W	S288C_Ator08_hmg124h_11.JPG.dat	1069.5	31.6	1.0	0.03	0.018	0.027	6.4E-06
YNL078W	YNL078W	S288C_Ator08_hmg124h_11.JPG.dat	1161.0	57.3	1.0	0.01	0.054	0.014	5.9E-05
YNL079C	YNL079C	S288C_Ator08_hmg124h_11.JPG.dat	972.5	43.7	0.9	0.01	-0.082	0.014	8.5E-05
YNL080C	YNL080C	S288C_Ator08_hmg124h_11.JPG.dat	898.3	79.9	0.8	0.07	-0.013	0.074	1.9E-04
YNL081C	YNL081C	S288C_Ator08_hmg124h_11.JPG.dat	0.0	0.0	0.0	0.00	0.000	0.000	
YNL082W	YNL082W	S288C_Ator08_hmg124h_11.JPG.dat	1077.0	44.7	1.0	0.04	-0.043	0.042	2.0E-05
YNL083W	YNL083W	S288C_Ator08_hmg124h_11.JPG.dat	1103.0	63.3	1.0	0.05	-0.041	0.050	3.6E-05
YNL085W	YNL085W	S288C_Ator08_hmg124h_11.JPG.dat	1105.3	74.1	1.0	0.06	-0.038	0.059	6.0E-05
YNL087W	YNL087W	S288C_Ator08_hmg124h_11.JPG.dat	1069.8	35.3	1.0	0.03	-0.035	0.032	9.7E-06
YNL089C	YNL089C	S288C_Ator08_hmg124h_11.JPG.dat	1021.5	54.8	0.9	0.05	-0.050	0.047	3.7E-05
YNL090W	YNL090W	S288C_Ator08_hmg124h_11.JPG.dat	1222.5	25.6	1.1	0.02	0.094	0.024	2.9E-06
YNL091W	YNL091W	S288C_Ator08_hmg124h_11.JPG.dat	1115.3	44.8	1.0	0.04	0.039	0.042	2.0E-05
YNL092W	YNL092W	S288C_Ator08_hmg124h_11.JPG.dat	1246.8	12.6	1.1	0.02	0.055	0.023	2.3E-06
YNL093W	YNL093W	S288C_Ator08_hmg124h_11.JPG.dat	1056.8	36.2	1.0	0.02	-0.025	0.022	3.1E-06
YNL094W	YNL094W	S288C_Ator08_hmg124h_11.JPG.dat	1036.0	64.4	1.0	0.05	-0.003	0.054	4.5E-05
YNL095C	YNL095C	S288C_Ator08_hmg124h_11.JPG.dat	1022.8	33.2	1.0	0.01	-0.065	0.008	2.5E-05
YNL097C	YNL097C	S288C_Ator08_hmg124h_11.JPG.dat	1121.0	88.7	0.9	0.02	-0.012	0.024	2.2E-04
YNL098C	YNL098C	S288C_Ator08_hmg124h_11.JPG.dat	1135.8	60.1	1.0	0.06	0.021	0.059	6.2E-05
YNL099C	YNL099C	S288C_Ator08_hmg124h_11.JPG.dat	1075.0	60.8	0.9	0.05	-0.036	0.051	4.8E-05
YNL100W	YNL100W	S288C_Ator08_hmg124h_11.JPG.dat	1088.5	66.5	0.9	0.06	-0.034	0.063	8.2E-05
YNL101W	YNL101W	S288C_Ator08_hmg124h_11.JPG.dat	1191.0	21.0	1.0	0.02	0.004	0.017	1.3E-06
YNL104C	YNL104C	S288C_Ator08_hmg124h_11.JPG.dat	1177.5	10.6	1.0	0.01	-0.007	0.012	5.5E-07
YNL105W	YNL105W	S288C_Ator08_hmg124h_11.JPG.dat	1086.8	45.9	1.0	0.01	-0.039	0.006	1.4E-05
YNL106C	YNL106C	S288C_Ator08_hmg124h_11.JPG.dat	1109.3	62.4	1.0	0.05	0.000	0.054	4.6E-05
YNL107W	YNL107W	S288C_Ator08_hmg124h_11.JPG.dat	1000.3	35.9	0.9	0.03	-0.124	0.030	1.0E-05
YNL108C	YNL108C	S288C_Ator08_hmg124h_11.JPG.dat	1084.5	46.4	1.0	0.04	-0.021	0.042	2.1E-05
YNL115C	YNL115C	S288C_Ator08_hmg124h_11.JPG.dat	1151.8	46.2	1.1	0.04	-0.033	0.038	1.2E-05
YNL116W	YNL116W	S288C_Ator08_hmg124h_11.JPG.dat	1183.5	81.8	1.0	0.01	-0.082	0.008	2.3E-05
YNL117W	YNL117W	S288C_Ator08_hmg124h_11.JPG.dat	1239.5	88.2	1.0	0.08	0.067	0.077	1.1E-04
YNL119W	YNL119W	S288C_Ator08_hmg124h_11.JPG.dat	1250.8	100.0	1.1	0.08	0.033	0.081	1.2E-04
YNL120C	YNL120C	S288C_Ator08_hmg124h_11.JPG.dat	1208.0	24.1	1.0	0.02	0.086	0.022	2.5E-06
YNL121C	YNL121C	S288C_Ator08_hmg124h_11.JPG.dat	1154.0	24.5	1.0	0.01	0.053	0.008	2.4E-05
YNL122C	YNL122C	S288C_Ator08_hmg124h_11.JPG.dat	1216.3	13.2	1.0	0.01	-0.026	0.009	2.0E-07
YNL123W	YNL123W	S288C_Ator08_hmg124h_11.JPG.dat	1193.8	55.9	1.0	0.05	-0.019	0.050	3.1E-05
YNL125C	YNL125C	S288C_Ator08_hmg124h_11.JPG.dat	1208.8	14.1	1.1	0.01	0.003	0.012	4.4E-07
YNL127W	YNL127W	S288C_Ator08_hmg124h_11.JPG.dat	1076.0	32.2	1.0	0.03	-0.066	0.028	6.4E-06
YNL128W	YNL128W	S288C_Ator08_hmg124h_11.JPG.dat	1052.8	48.0	1.0	0.04	-0.037	0.041	2.1E-05
YNL129W	YNL129W	S288C_Ator08_hmg124h_11.JPG.dat	1036.3	61.0	1.0	0.05	-0.014	0.053	4.9E-05
YNL130C	YNL130C	S288C_Ator08_hmg124h_11.JPG.dat	1046.0	46.7	0.9	0.02	-0.135	0.020	2.6E-06
YNL134C	YNL134C	S288C_Ator08_hmg124h_11.JPG.dat	1086.8	83.9	0.9	0.08	-0.078	0.077	1.5E-04
YNL135C	YNL135C	S288C_Ator08_hmg124h_11.JPG.dat	1202.8	21.9	1.0	0.02	0.026	0.018	1.4E-06
YNL136W	YNL136W	S288C_Ator08_hmg124h_11.JPG.dat	1162.8	73.1	1.0	0.04	-0.035	0.040	1.9E-05
YNL140C	YNL140C	S288C_Ator08_hmg124h_11.JPG.dat	1220.3	26.4	1.0	0.03	0.044	0.026	4.4E-06
YNL141W	YNL141W	S288C_Ator08_hmg124h_11.JPG.dat	1095.3	51.1	0.9	0.06	0.040	0.063	8.8E-05
YNL142W	YNL142W	S288C_Ator08_hmg124h_11.JPG.dat	1262.3	50.8	1.0	0.03	-0.005	0.033	8.3E-06
YNL143C	YNL143C	S288C_Ator08_hmg124h_11.JPG.dat	1232.8	48.9	1.0	0.02	-0.056	0.025	4.0E-06
YNL144C	YNL144C	S288C_Ator08_hmg124h_11.JPG.dat	1319.5	65.4	1.1	0.03	0.018	0.027	4.3E-06
YNL145W	YNL145W	S288C_Ator08_hmg124h_11.JPG.dat	1300.3	49.9	1.1	0.01	0.134	0.012	3.7E-05
YNL146W	YNL146W	S288C_Ator08_hmg124h_11.JPG.dat	1177.0	90.1	1.0	0.06	0.037	0.063	6.4E-05
YNL153C	YNL153C	S288C_Ator08_hmg124h_11.JPG.dat	1093.8	60.2	1.0	0.05	-0.042	0.050	3.3E-05
YNL154C	YNL154C	S288C_Ator08_hmg124h_11.JPG.dat	1088.3	79.9	1.0	0.08	-0.049	0.080	1.5E-04
YNL155W	YNL155W	S288C_Ator08_hmg124h_11.JPG.dat	1097.0	79.1	1.0	0.07	-0.057	0.071	1.1E-04
YNL156C	YNL156C	S288C_Ator08_hmg124h_11.JPG.dat	1257.8	21.2	1.1	0.01	0.034	0.015	6.7E-07
YNL157W	YNL157W	S288C_Ator08_hmg124h_11.JPG.dat	1199.5	25.3	1.0	0.01	-0.017	0.006	1.0E-05
YNL159C	YNL159C	S288C_Ator08_hmg124h_11.JPG.dat	1129.0	21.6	1.0	0.00	-0.080	0.004	6.7E-06
YNL162W	YNL162W	S288C_Ator08_hmg124h_11.JPG.dat	1093.8	23.6	0.9	0.02	0.036	0.025	5.0E-06
YNL164C	YNL164C	S288C_Ator08_hmg124h_11.JPG.dat	1233.5	49.6	1.0	0.05	0.008	0.047	2.5E-05
YNL165W	YNL165W	S288C_Ator08_hmg124h_11.JPG.dat	1142.8	51.5	1.0	0.04	-0.043	0.040	2.0E-05
YNL166C	YNL166C	S288C_Ator08_hmg124h_11.JPG.dat	1181.5	34.9	1.0	0.02	0.018	0.024	3.9E-06
YNL167C	YNL167C	S288C_Ator08_hmg124h_11.JPG.dat	986.0	47.9	0.9	0.01	-0.105	0.012	6.1E-05
YNL168C	YNL168C	S288C_Ator08_hmg124h_11.JPG.dat	1177.8	24.3	1.1	0.02	-0.027	0.023	2.8E-06
YNL169C	YNL169C	S288C_Ator08_hmg124h_11.JPG.dat	1107.8	35.2	1.0	0.02	-0.005	0.025	4.6E-06
YNL170W	YNL170W	S288C_Ator08_hmg124h_11.JPG.dat	0.0	0.0	0.0	0.00	0.000	0.000	

YNL171C	YNL171C	S288C_Ator08_hmg124h_11.JPG.dat	981.0	179.2	0.9	0.16	-0.022	0.157	1.7E-03
YNL173C	YNL173C	S288C_Ator08_hmg124h_11.JPG.dat	1193.5	8.2	1.0	0.00	0.011	0.004	6.3E-06
YNL175C	YNL175C	S288C_Ator08_hmg124h_11.JPG.dat	1185.3	46.4	1.0	0.04	0.005	0.042	1.9E-05
YNL176C	YNL176C	S288C_Ator08_hmg124h_11.JPG.dat	1229.3	23.4	1.0	0.02	0.011	0.021	2.1E-06
YNL179C	YNL179C	S288C_Ator08_hmg124h_11.JPG.dat	1054.8	45.3	0.9	0.04	-0.078	0.036	1.8E-05
YNL183C	YNL183C	S288C_Ator08_hmg124h_11.JPG.dat	1121.5	107.0	1.0	0.09	-0.060	0.089	2.2E-04
YNL187W	YNL187W	S288C_Ator08_hmg124h_11.JPG.dat	1187.5	36.2	1.0	0.01	0.012	0.007	1.5E-05
YNL190W	YNL190W	S288C_Ator08_hmg124h_11.JPG.dat	1020.8	138.0	0.9	0.12	-0.129	0.116	5.5E-04
YNL191W	YNL191W	S288C_Ator08_hmg124h_11.JPG.dat	1137.5	71.0	1.0	0.06	0.022	0.064	6.6E-05
YNL192W	YNL192W	S288C_Ator08_hmg124h_11.JPG.dat	1101.8	72.8	1.0	0.06	-0.031	0.064	8.0E-05
YNL193W	YNL193W	S288C_Ator08_hmg124h_11.JPG.dat	1161.8	14.7	1.0	0.01	0.017	0.011	3.0E-07
YNL194C	YNL194C	S288C_Ator08_hmg124h_11.JPG.dat	1052.5	60.6	0.9	0.05	-0.061	0.055	5.4E-05
YNL195C	YNL195C	S288C_Ator08_hmg124h_11.JPG.dat	1167.0	48.9	1.1	0.00	0.019	0.003	2.5E-06
YNL196C	YNL196C	S288C_Ator08_hmg124h_11.JPG.dat	1071.0	131.8	0.9	0.02	-0.099	0.021	1.9E-04
YNL197C	YNL197C	S288C_Ator08_hmg124h_11.JPG.dat	608.8	704.1	0.5	0.61	0.025	0.612	1.8E-01
YNL198C	YNL198C	S288C_Ator08_hmg124h_11.JPG.dat	1094.8	65.1	0.9	0.05	0.146	0.054	5.2E-05
YNL199C	YNL199C	S288C_Ator08_hmg124h_11.JPG.dat	1195.3	43.8	1.1	0.04	0.152	0.044	1.9E-05
YNL200C	YNL200C	S288C_Ator08_hmg124h_11.JPG.dat	1155.5	63.5	1.0	0.05	-0.017	0.052	3.5E-05
YNL201C	YNL201C	S288C_Ator08_hmg124h_11.JPG.dat	1036.0	37.8	1.0	0.03	-0.084	0.028	6.9E-06
YNL202W	YNL202W	S288C_Ator08_hmg124h_11.JPG.dat	1076.0	75.4	1.0	0.00	-0.002	0.004	6.6E-06
YNL203C	YNL203C	S288C_Ator08_hmg124h_11.JPG.dat	1189.8	21.6	1.0	0.02	-0.011	0.023	2.8E-06
YNL204C	YNL204C	S288C_Ator08_hmg124h_11.JPG.dat	1091.5	71.9	1.0	0.06	-0.026	0.061	7.1E-05
YNL205C	YNL205C	S288C_Ator08_hmg124h_11.JPG.dat	1057.5	84.2	0.9	0.08	-0.091	0.076	1.4E-04
YNL206C	YNL206C	S288C_Ator08_hmg124h_11.JPG.dat	1095.8	62.5	1.0	0.02	-0.008	0.016	9.7E-05
YNL208W	YNL208W	S288C_Ator08_hmg124h_11.JPG.dat	1009.8	28.1	0.9	0.01	-0.044	0.007	1.8E-05
YNL211C	YNL211C	S288C_Ator08_hmg124h_11.JPG.dat	1181.8	24.6	1.0	0.01	0.033	0.006	1.3E-05
YNL212W	YNL212W	S288C_Ator08_hmg124h_11.JPG.dat	1190.5	26.1	1.0	0.03	0.028	0.029	6.2E-06
YNL214W	YNL214W	S288C_Ator08_hmg124h_11.JPG.dat	1123.0	43.1	1.0	0.01	0.058	0.010	2.9E-05
YNL215W	YNL215W	S288C_Ator08_hmg124h_11.JPG.dat	1032.0	67.3	1.0	0.06	-0.048	0.058	6.1E-05
YNL217W	YNL217W	S288C_Ator08_hmg124h_11.JPG.dat	1065.3	58.9	1.0	0.05	0.081	0.049	2.9E-05
YNL218W	YNL218W	S288C_Ator08_hmg124h_11.JPG.dat	1148.0	92.3	1.0	0.08	0.032	0.084	1.5E-04
YNL219C	YNL219C	S288C_Ator08_hmg124h_11.JPG.dat	1162.8	102.3	1.0	0.09	-0.062	0.092	2.0E-04
YNL223W	YNL223W	S288C_Ator08_hmg124h_11.JPG.dat	1109.5	129.0	1.0	0.11	-0.046	0.114	4.2E-04
YNL224C	YNL224C	S288C_Ator08_hmg124h_11.JPG.dat	1182.5	10.4	1.0	0.01	0.071	0.009	1.5E-07
YNL226W	YNL226W	S288C_Ator08_hmg124h_11.JPG.dat	1066.8	54.1	0.9	0.04	0.009	0.045	3.0E-05
YNL227C	YNL227C	S288C_Ator08_hmg124h_11.JPG.dat	1015.3	59.3	0.9	0.05	-0.030	0.050	4.6E-05
YNL228W	YNL228W	S288C_Ator08_hmg124h_11.JPG.dat	1044.5	34.2	1.0	0.01	0.154	0.008	2.2E-05
YNL229C	YNL229C	S288C_Ator08_hmg124h_11.JPG.dat	971.0	28.4	0.9	0.03	-0.038	0.034	1.6E-05
YNL230C	YNL230C	S288C_Ator08_hmg124h_11.JPG.dat	1096.8	17.6	1.0	0.00	0.012	0.004	6.3E-06
YNL231C	YNL231C	S288C_Ator08_hmg124h_11.JPG.dat	997.8	68.4	1.0	0.06	-0.084	0.062	7.4E-05
YNL233W	YNL233W	S288C_Ator08_hmg124h_11.JPG.dat	883.3	29.3	0.9	0.02	-0.191	0.022	4.2E-06
YNL234W	YNL234W	S288C_Ator08_hmg124h_11.JPG.dat	1165.5	186.3	1.0	0.14	0.012	0.143	8.2E-04
YNL235C	YNL235C	S288C_Ator08_hmg124h_11.JPG.dat	1314.5	40.6	1.1	0.02	0.082	0.017	1.0E-06
YNL236W	YNL236W	S288C_Ator08_hmg124h_11.JPG.dat	1173.0	57.8	1.0	0.04	0.007	0.043	2.2E-05
YNL237W	YNL237W	S288C_Ator08_hmg124h_11.JPG.dat	1227.0	44.2	1.0	0.03	0.037	0.035	1.0E-05
YNL238W	YNL238W	S288C_Ator08_hmg124h_11.JPG.dat	1173.0	12.1	1.0	0.01	-0.018	0.013	7.1E-07
YNL239W	YNL239W	S288C_Ator08_hmg124h_11.JPG.dat	1148.0	55.7	1.0	0.01	0.012	0.011	4.2E-05
YNL241C	YNL241C	S288C_Ator08_hmg124h_11.JPG.dat	1014.5	112.0	0.9	0.09	0.016	0.095	3.4E-04
YNL242W	YNL242W	S288C_Ator08_hmg124h_11.JPG.dat	861.5	575.4	1.0	0.03	-0.018	0.028	2.4E-04
YNL246W	YNL246W	S288C_Ator08_hmg124h_11.JPG.dat	1146.3	30.1	1.0	0.03	0.024	0.028	5.4E-06
YNL249C	YNL249C	S288C_Ator08_hmg124h_11.JPG.dat	1107.0	93.4	1.0	0.02	-0.008	0.020	1.2E-04
YNL253W	YNL253W	S288C_Ator08_hmg124h_11.JPG.dat	1072.5	98.0	1.0	0.09	0.083	0.087	1.7E-04
YNL254C	YNL254C	S288C_Ator08_hmg124h_11.JPG.dat	788.8	528.4	0.9	0.02	0.019	0.019	1.3E-04
YNL255C	YNL255C	S288C_Ator08_hmg124h_11.JPG.dat	987.8	93.9	0.9	0.07	-0.101	0.071	1.4E-04
YNL257C	YNL257C	S288C_Ator08_hmg124h_11.JPG.dat	1024.5	98.9	0.9	0.08	-0.095	0.082	2.1E-04
YNL259C	YNL259C	S288C_Ator08_hmg124h_11.JPG.dat	1077.5	81.3	0.9	0.05	-0.035	0.045	3.1E-05
YNL265C	YNL265C	S288C_Ator08_hmg124h_11.JPG.dat	586.5	684.6	0.5	0.57	-0.003	0.572	1.9E-01
YNL266W	YNL266W	S288C_Ator08_hmg124h_11.JPG.dat	310.3	620.5	0.0	0.00	0.000	0.000	
YNL268W	YNL268W	S288C_Ator08_hmg124h_11.JPG.dat	1252.3	63.6	1.0	0.01	-0.013	0.015	7.6E-07
YNL270C	YNL270C	S288C_Ator08_hmg124h_11.JPG.dat	258.5	517.0	0.0	0.00	0.000	0.000	
YNL271C	YNL271C	S288C_Ator08_hmg124h_11.JPG.dat	313.0	626.0	0.0	0.00	0.000	0.000	
YNL273W	YNL273W	S288C_Ator08_hmg124h_11.JPG.dat	898.8	616.5	1.0	0.12	0.027	0.119	4.6E-03
YNL274C	YNL274C	S288C_Ator08_hmg124h_11.JPG.dat	883.8	592.6	1.0	0.09	0.094	0.091	2.7E-03
YNL275W	YNL275W	S288C_Ator08_hmg124h_11.JPG.dat	777.0	518.9	1.0	0.04	-0.048	0.037	4.9E-04
YNL277W	YNL277W	S288C_Ator08_hmg124h_11.JPG.dat	1172.8	56.4	1.1	0.06	0.109	0.060	4.8E-05
YNL278W	YNL278W	S288C_Ator08_hmg124h_11.JPG.dat	1176.0	92.8	1.1	0.03	0.093	0.029	2.5E-04
YNL279W	YNL279W	S288C_Ator08_hmg124h_11.JPG.dat	1277.0	74.0	1.1	0.02	0.078	0.016	7.2E-05
YNL280C	YNL280C	S288C_Ator08_hmg124h_11.JPG.dat	1197.3	30.0	1.0	0.03	0.059	0.025	3.9E-06
YNL281W	YNL281W	S288C_Ator08_hmg124h_11.JPG.dat	1048.5	78.7	0.9	0.07	-0.098	0.070	1.3E-04
YNL283C	YNL283C	S288C_Ator08_hmg124h_11.JPG.dat	1183.3	25.5	1.0	0.02	0.036	0.023	3.3E-06

YNL285W	YNL285W	S288C_Ator08_hmg124h_11.JPG.dat	1041.8	58.9	0.9	0.05	-0.117	0.049	4.7E-05
YNL286W	YNL286W	S288C_Ator08_hmg124h_11.JPG.dat	1129.0	65.7	1.0	0.01	0.004	0.008	2.2E-05
YNL288W	YNL288W	S288C_Ator08_hmg124h_11.JPG.dat	1195.0	31.1	1.0	0.02	0.089	0.023	3.2E-06
YNL289W	YNL289W	S288C_Ator08_hmg124h_11.JPG.dat	1099.5	98.2	1.0	0.08	0.025	0.081	1.6E-04
YNL291C	YNL291C	S288C_Ator08_hmg124h_11.JPG.dat	817.3	59.5	0.7	0.05	-0.254	0.052	9.7E-05
YNL292W	YNL292W	S288C_Ator08_hmg124h_11.JPG.dat	1180.3	63.0	1.0	0.01	0.026	0.012	4.7E-05
YNL293W	YNL293W	S288C_Ator08_hmg124h_11.JPG.dat	1192.5	32.5	1.0	0.03	-0.036	0.029	6.1E-06
YNL294C	YNL294C	S288C_Ator08_hmg124h_11.JPG.dat	973.5	46.5	0.9	0.04	-0.152	0.041	3.1E-05
YNL295W	YNL295W	S288C_Ator08_hmg124h_11.JPG.dat	1133.5	75.6	1.0	0.02	0.044	0.020	1.3E-04
YNL296W	YNL296W	S288C_Ator08_hmg124h_11.JPG.dat	732.5	27.4	0.6	0.02	-0.287	0.023	1.3E-05
YNL297C	YNL297C	S288C_Ator08_hmg124h_11.JPG.dat	787.5	60.5	0.6	0.02	-0.278	0.016	2.0E-04
YNL298W	YNL298W	S288C_Ator08_hmg124h_11.JPG.dat	1051.0	59.4	0.9	0.05	-0.114	0.051	5.2E-05
YNL299W	YNL299W	S288C_Ator08_hmg124h_11.JPG.dat	1194.0	97.1	1.1	0.03	0.058	0.031	2.8E-04
YNL300W	YNL300W	S288C_Ator08_hmg124h_11.JPG.dat	1274.5	53.6	1.1	0.05	0.052	0.049	2.4E-05
YNL301C	YNL301C	S288C_Ator08_hmg124h_11.JPG.dat	1041.3	83.4	0.9	0.08	-0.080	0.077	1.7E-04
YNL302C	YNL302C	S288C_Ator08_hmg124h_11.JPG.dat	769.0	30.3	0.7	0.03	0.102	0.028	1.8E-05
YNL303W	YNL303W	S288C_Ator08_hmg124h_11.JPG.dat	1048.3	19.6	0.9	0.02	-0.046	0.017	1.6E-06
YNL304W	YNL304W	S288C_Ator08_hmg124h_11.JPG.dat	1094.5	86.6	1.0	0.08	-0.039	0.077	1.4E-04
YNL305C	YNL305C	S288C_Ator08_hmg124h_11.JPG.dat	1050.0	52.5	1.0	0.05	-0.041	0.048	3.4E-05
YNL307C	YNL307C	S288C_Ator08_hmg124h_11.JPG.dat	1031.5	14.2	0.9	0.01	-0.002	0.011	4.5E-07
YNL309W	YNL309W	S288C_Ator08_hmg124h_11.JPG.dat	1178.0	34.1	1.0	0.03	0.053	0.031	7.3E-06
YNL311C	YNL311C	S288C_Ator08_hmg124h_11.JPG.dat	1243.3	56.9	1.1	0.05	0.067	0.047	2.2E-05
YNL314W	YNL314W	S288C_Ator08_hmg124h_11.JPG.dat	1199.8	19.5	1.0	0.02	0.011	0.021	2.5E-06
YNL315C	YNL315C	S288C_Ator08_hmg124h_11.JPG.dat	726.0	131.0	0.6	0.12	-0.041	0.116	1.6E-03
YNL316C	YNL316C	S288C_Ator08_hmg124h_11.JPG.dat	1064.8	23.0	1.0	0.00	-0.003	0.002	1.3E-06
YNL318C	YNL318C	S288C_Ator08_hmg124h_11.JPG.dat	1129.8	29.0	1.0	0.03	0.012	0.033	8.8E-06
YNL319W	YNL319W	S288C_Ator08_hmg124h_11.JPG.dat	1065.3	69.8	1.0	0.06	0.043	0.063	7.8E-05
YNL320W	YNL320W	S288C_Ator08_hmg124h_11.JPG.dat	1079.8	60.3	1.0	0.06	-0.037	0.060	6.5E-05
YNL321W	YNL321W	S288C_Ator08_hmg124h_11.JPG.dat	1102.8	64.6	1.0	0.06	-0.039	0.055	5.1E-05
YNL322C	YNL322C	S288C_Ator08_hmg124h_11.JPG.dat	1141.5	19.4	1.0	0.02	0.047	0.019	1.8E-06
YNL323W	YNL323W	S288C_Ator08_hmg124h_11.JPG.dat	849.0	108.5	0.8	0.10	-0.261	0.098	5.6E-04
YNL324W	YNL324W	S288C_Ator08_hmg124h_11.JPG.dat	1038.5	84.7	0.9	0.07	-0.103	0.072	1.3E-04
YNL325C	YNL325C	S288C_Ator08_hmg124h_11.JPG.dat	1133.8	58.8	1.0	0.01	-0.043	0.009	2.5E-05
YNL326C	YNL326C	S288C_Ator08_hmg124h_11.JPG.dat	1165.0	15.7	1.0	0.02	0.129	0.015	9.0E-07
YNL327W	YNL327W	S288C_Ator08_hmg124h_11.JPG.dat	1166.3	39.4	1.1	0.04	0.021	0.035	1.0E-05
YNL328C	YNL328C	S288C_Ator08_hmg124h_11.JPG.dat	1106.8	31.5	1.0	0.03	0.025	0.026	4.6E-06
YNL329C	YNL329C	S288C_Ator08_hmg124h_11.JPG.dat	1092.8	45.9	1.0	0.04	0.007	0.040	1.6E-05
YNL330C	YNL330C	S288C_Ator08_hmg124h_11.JPG.dat	1172.8	9.4	1.1	0.01	0.077	0.009	1.5E-07
YNL332W	YNL332W	S288C_Ator08_hmg124h_11.JPG.dat	1189.0	22.2	1.1	0.02	0.032	0.021	2.1E-06
YNL333W	YNL333W	S288C_Ator08_hmg124h_11.JPG.dat	1107.5	73.7	1.0	0.07	-0.029	0.065	8.0E-05
YNL334C	YNL334C	S288C_Ator08_hmg124h_11.JPG.dat	1193.3	46.2	1.1	0.04	0.042	0.043	1.8E-05
YNL335W	YNL335W	S288C_Ator08_hmg124h_11.JPG.dat	1163.5	44.9	1.0	0.04	0.013	0.040	1.5E-05
YNL336W	YNL336W	S288C_Ator08_hmg124h_11.JPG.dat	1179.0	6.2	1.0	0.00	0.058	0.003	2.2E-06
YNL338W	YNL338W	S288C_Ator08_hmg124h_11.JPG.dat	1190.3	19.3	1.1	0.01	0.041	0.006	9.7E-06
YNL339C	YNL339C	S288C_Ator08_hmg124h_11.JPG.dat	1142.5	128.0	1.0	0.01	0.035	0.005	8.6E-06
YNR001C	YNR001C	S288C_Ator08_hmg124h_11.JPG.dat	1112.5	44.0	1.0	0.04	0.069	0.037	1.3E-05
YNR002C	YNR002C	S288C_Ator08_hmg124h_11.JPG.dat	1013.5	47.1	1.0	0.04	-0.065	0.044	2.5E-05
YNR004W	YNR004W	S288C_Ator08_hmg124h_11.JPG.dat	1012.8	29.6	1.0	0.01	-0.010	0.009	2.9E-05
YNR005C	YNR005C	S288C_Ator08_hmg124h_11.JPG.dat	1310.8	20.4	1.1	0.03	0.064	0.027	3.7E-06
YNR006W	YNR006W	S288C_Ator08_hmg124h_11.JPG.dat	452.3	271.2	0.4	0.22	-0.044	0.222	4.2E-02
YNR007C	YNR007C	S288C_Ator08_hmg124h_11.JPG.dat	1058.3	93.0	0.9	0.02	-0.071	0.023	2.3E-04
YNR008W	YNR008W	S288C_Ator08_hmg124h_11.JPG.dat	1109.5	62.0	0.9	0.02	-0.050	0.021	1.7E-04
YNR009W	YNR009W	S288C_Ator08_hmg124h_11.JPG.dat	1157.5	67.5	1.0	0.04	0.017	0.045	2.6E-05
YNR010W	YNR010W	S288C_Ator08_hmg124h_11.JPG.dat	1227.3	26.0	1.0	0.02	0.020	0.020	2.1E-06
YNR012W	YNR012W	S288C_Ator08_hmg124h_11.JPG.dat	1108.0	58.1	1.0	0.05	-0.076	0.049	3.8E-05
YNR013C	YNR013C	S288C_Ator08_hmg124h_11.JPG.dat	1095.5	18.4	1.0	0.03	-0.045	0.029	7.7E-06
YNR014W	YNR014W	S288C_Ator08_hmg124h_11.JPG.dat	1140.5	44.0	1.0	0.04	-0.017	0.042	1.8E-05
YNR015W	YNR015W	S288C_Ator08_hmg124h_11.JPG.dat	1024.0	106.1	0.9	0.10	-0.077	0.098	3.1E-04
YNR018W	YNR018W	S288C_Ator08_hmg124h_11.JPG.dat	1120.5	25.6	1.0	0.01	0.032	0.014	6.9E-07
YOR202W	YOR202W	S288C_Ator08_hmg124h_11.JPG.dat	1168.7	120.9	1.0	0.06	0.008	0.064	1.97626258
1	1	S288C_Ator08_hmg124h_7.JPG.dat	1239.0	55.7	1.1	0.10	0.012	0.097	2.0E-04
2	2	S288C_Ator08_hmg124h_7.JPG.dat	1148.5	53.8	1.0	0.04	0.103	0.041	1.9E-05
3	3	S288C_Ator08_hmg124h_7.JPG.dat	1135.0	98.3	1.1	0.11	0.094	0.106	2.7E-04
4	4	S288C_Ator08_hmg124h_7.JPG.dat	1074.8	43.6	1.0	0.04	0.020	0.039	1.4E-05
YIL060W	YIL060W	S288C_Ator08_hmg124h_7.JPG.dat	1078.3	56.5	1.0	0.05	0.013	0.054	4.8E-05
YIL064W	YIL064W	S288C_Ator08_hmg124h_7.JPG.dat	1160.5	97.3	1.1	0.02	0.075	0.019	9.7E-05
YIL065C	YIL065C	S288C_Ator08_hmg124h_7.JPG.dat	1070.0	11.9	1.0	0.01	0.014	0.010	3.5E-07
YIL066C	YIL066C	S288C_Ator08_hmg124h_7.JPG.dat	1129.5	12.5	1.0	0.01	-0.007	0.008	1.2E-07
YIL067C	YIL067C	S288C_Ator08_hmg124h_7.JPG.dat	1059.5	46.1	1.0	0.04	-0.002	0.041	2.0E-05
YIL070C	YIL070C	S288C_Ator08_hmg124h_7.JPG.dat	1048.8	67.8	1.0	0.01	-0.016	0.015	7.2E-05

YIL071C	YIL071C	S288C_Ator08_hmg124h_7.JPG.dat	1120.0	18.8	1.0	0.01	-0.027	0.012	4.8E-07
YIL072W	YIL072W	S288C_Ator08_hmg124h_7.JPG.dat	1135.3	9.2	1.0	0.00	0.010	0.002	1.6E-06
YIL073C	YIL073C	S288C_Ator08_hmg124h_7.JPG.dat	1100.5	52.5	1.0	0.01	0.023	0.008	2.3E-05
YIL074C	YIL074C	S288C_Ator08_hmg124h_7.JPG.dat	1078.5	44.3	1.0	0.04	-0.028	0.045	2.8E-05
YIL076W	YIL076W	S288C_Ator08_hmg124h_7.JPG.dat	1040.5	23.6	0.9	0.02	-0.082	0.024	4.8E-06
YIL077C	YIL077C	S288C_Ator08_hmg124h_7.JPG.dat	1098.0	53.5	1.0	0.02	-0.011	0.019	1.2E-04
YIL079C	YIL079C	S288C_Ator08_hmg124h_7.JPG.dat	1138.5	21.5	1.0	0.02	-0.003	0.019	1.6E-06
YIL084C	YIL084C	S288C_Ator08_hmg124h_7.JPG.dat	1059.3	18.0	1.0	0.02	-0.057	0.018	1.8E-06
YIL086C	YIL086C	S288C_Ator08_hmg124h_7.JPG.dat	1098.0	29.1	1.0	0.02	0.021	0.020	2.3E-06
YIL087C	YIL087C	S288C_Ator08_hmg124h_7.JPG.dat	1074.8	10.9	1.0	0.02	0.021	0.017	1.4E-06
YIL088C	YIL088C	S288C_Ator08_hmg124h_7.JPG.dat	988.5	37.8	1.0	0.03	-0.047	0.033	1.1E-05
YIL089W	YIL089W	S288C_Ator08_hmg124h_7.JPG.dat	1065.8	16.9	1.0	0.02	0.002	0.016	1.1E-06
YIL090W	YIL090W	S288C_Ator08_hmg124h_7.JPG.dat	0.0	0.0	0.0	0.00	0.000	0.000	
YIL092W	YIL092W	S288C_Ator08_hmg124h_7.JPG.dat	1135.3	17.0	1.0	0.02	-0.004	0.018	1.6E-06
YIL093C	YIL093C	S288C_Ator08_hmg124h_7.JPG.dat	1130.3	22.3	1.0	0.02	0.047	0.024	3.5E-06
YIL095W	YIL095W	S288C_Ator08_hmg124h_7.JPG.dat	1101.8	48.6	1.1	0.02	0.032	0.017	8.3E-05
YIL096C	YIL096C	S288C_Ator08_hmg124h_7.JPG.dat	1131.3	39.2	1.0	0.01	-0.008	0.014	6.0E-05
YIL097W	YIL097W	S288C_Ator08_hmg124h_7.JPG.dat	1000.0	22.3	0.9	0.02	-0.002	0.019	2.3E-06
YIL098C	YIL098C	S288C_Ator08_hmg124h_7.JPG.dat	1076.5	34.0	1.0	0.03	0.016	0.030	7.4E-06
YIL099W	YIL099W	S288C_Ator08_hmg124h_7.JPG.dat	1040.8	42.8	1.0	0.04	-0.012	0.038	1.5E-05
YIL100W	YIL100W	S288C_Ator08_hmg124h_7.JPG.dat	1030.3	21.5	1.0	0.00	0.016	0.004	6.2E-06
YIL101C	YIL101C	S288C_Ator08_hmg124h_7.JPG.dat	957.8	15.8	1.0	0.02	0.008	0.020	2.6E-06
YIL103W	YIL103W	S288C_Ator08_hmg124h_7.JPG.dat	895.0	60.1	0.9	0.06	-0.052	0.064	1.0E-04
YIL105C	YIL105C	S288C_Ator08_hmg124h_7.JPG.dat	1056.8	16.7	1.0	0.01	-0.051	0.011	3.7E-07
YIL107C	YIL107C	S288C_Ator08_hmg124h_7.JPG.dat	1093.8	6.9	1.0	0.01	0.001	0.010	2.7E-07
YIL108W	YIL108W	S288C_Ator08_hmg124h_7.JPG.dat	1107.5	12.4	1.0	0.01	-0.015	0.006	6.9E-08
YIL110W	YIL110W	S288C_Ator08_hmg124h_7.JPG.dat	830.3	182.7	0.8	0.18	0.123	0.182	2.9E-03
YIL112W	YIL112W	S288C_Ator08_hmg124h_7.JPG.dat	1024.8	75.2	1.0	0.02	0.038	0.023	1.8E-04
YIL113W	YIL113W	S288C_Ator08_hmg124h_7.JPG.dat	968.8	101.4	0.9	0.10	-0.103	0.096	3.1E-04
YIL114C	YIL114C	S288C_Ator08_hmg124h_7.JPG.dat	1077.5	21.4	1.0	0.02	0.009	0.020	2.1E-06
YIL116W	YIL116W	S288C_Ator08_hmg124h_7.JPG.dat	0.0	0.0	0.0	0.00	0.000	0.000	
YIL117C	YIL117C	S288C_Ator08_hmg124h_7.JPG.dat	993.8	26.0	1.0	0.03	-0.014	0.032	8.9E-06
YIL119C	YIL119C	S288C_Ator08_hmg124h_7.JPG.dat	1061.8	21.4	1.1	0.02	0.046	0.021	2.1E-06
YIL120W	YIL120W	S288C_Ator08_hmg124h_7.JPG.dat	981.0	41.3	1.0	0.04	-0.002	0.040	1.8E-05
YIL123W	YIL123W	S288C_Ator08_hmg124h_7.JPG.dat	1027.0	29.2	1.0	0.03	-0.001	0.025	4.6E-06
YIL124W	YIL124W	S288C_Ator08_hmg124h_7.JPG.dat	1044.3	71.8	1.0	0.07	0.005	0.069	9.0E-05
YIL128W	YIL128W	S288C_Ator08_hmg124h_7.JPG.dat	870.3	21.7	0.8	0.02	0.328	0.021	4.4E-06
YIL130W	YIL130W	S288C_Ator08_hmg124h_7.JPG.dat	1009.0	40.4	1.0	0.04	0.019	0.035	1.2E-05
YIL132C	YIL132C	S288C_Ator08_hmg124h_7.JPG.dat	1079.5	34.2	1.0	0.03	0.035	0.033	1.0E-05
YIL133C	YIL133C	S288C_Ator08_hmg124h_7.JPG.dat	1042.3	10.3	1.0	0.01	0.061	0.009	2.2E-07
YIL134W	YIL134W	S288C_Ator08_hmg124h_7.JPG.dat	1099.0	12.7	1.0	0.01	0.049	0.013	6.2E-07
YIL135C	YIL135C	S288C_Ator08_hmg124h_7.JPG.dat	1133.3	31.8	1.1	0.01	0.012	0.008	1.9E-05
YIL137C	YIL137C	S288C_Ator08_hmg124h_7.JPG.dat	975.8	34.2	1.0	0.04	-0.003	0.037	1.5E-05
YIL138C	YIL138C	S288C_Ator08_hmg124h_7.JPG.dat	1013.0	15.0	1.0	0.01	-0.021	0.012	5.2E-07
YIL139C	YIL139C	S288C_Ator08_hmg124h_7.JPG.dat	1005.5	31.5	1.0	0.04	-0.016	0.037	1.4E-05
YIL140W	YIL140W	S288C_Ator08_hmg124h_7.JPG.dat	1064.3	13.6	1.0	0.01	0.023	0.013	6.3E-07
YIL141W	YIL141W	S288C_Ator08_hmg124h_7.JPG.dat	1101.5	17.8	1.0	0.02	-0.041	0.015	8.9E-07
YIL145C	YIL145C	S288C_Ator08_hmg124h_7.JPG.dat	1078.8	24.6	1.0	0.00	0.026	0.004	6.2E-06
YIL146C	YIL146C	S288C_Ator08_hmg124h_7.JPG.dat	1025.3	38.9	1.0	0.00	-0.008	0.003	3.6E-06
YIL148W	YIL148W	S288C_Ator08_hmg124h_7.JPG.dat	927.3	194.9	0.9	0.01	0.076	0.014	6.8E-05
YIL149C	YIL149C	S288C_Ator08_hmg124h_7.JPG.dat	1097.3	17.2	1.0	0.00	-0.018	0.002	1.2E-06
YIL152W	YIL152W	S288C_Ator08_hmg124h_7.JPG.dat	1118.5	14.2	1.0	0.02	0.000	0.016	1.1E-06
YIL153W	YIL153W	S288C_Ator08_hmg124h_7.JPG.dat	936.3	51.5	0.9	0.05	0.050	0.052	5.7E-05
YIL154C	YIL154C	S288C_Ator08_hmg124h_7.JPG.dat	875.8	13.4	0.9	0.02	0.028	0.019	2.9E-06
YIL155C	YIL155C	S288C_Ator08_hmg124h_7.JPG.dat	984.8	41.8	1.0	0.05	0.039	0.050	3.7E-05
YIL156W	YIL156W	S288C_Ator08_hmg124h_7.JPG.dat	1050.0	35.9	1.0	0.02	-0.050	0.025	4.1E-06
YIL157C	YIL157C	S288C_Ator08_hmg124h_7.JPG.dat	1082.0	18.0	1.0	0.00	0.006	0.005	6.8E-06
YIL159W	YIL159W	S288C_Ator08_hmg124h_7.JPG.dat	1130.3	32.8	1.1	0.04	0.001	0.040	1.4E-05
YIL160C	YIL160C	S288C_Ator08_hmg124h_7.JPG.dat	1121.8	21.6	1.0	0.02	0.008	0.017	1.2E-06
YIL161W	YIL161W	S288C_Ator08_hmg124h_7.JPG.dat	1101.5	15.8	1.1	0.02	0.094	0.022	2.4E-06
YIL162W	YIL162W	S288C_Ator08_hmg124h_7.JPG.dat	1175.5	50.3	1.0	0.05	0.051	0.046	2.5E-05
YIL163C	YIL163C	S288C_Ator08_hmg124h_7.JPG.dat	1146.3	44.8	1.0	0.04	0.012	0.041	1.9E-05
YIL164C	YIL164C	S288C_Ator08_hmg124h_7.JPG.dat	1182.8	29.6	1.0	0.03	0.054	0.026	4.1E-06
YIL165C	YIL165C	S288C_Ator08_hmg124h_7.JPG.dat	1165.3	28.2	1.0	0.03	0.050	0.029	5.7E-06
YIL166C	YIL166C	S288C_Ator08_hmg124h_7.JPG.dat	1123.3	23.7	1.1	0.01	-0.007	0.010	3.0E-05
YIL167W	YIL167W	S288C_Ator08_hmg124h_7.JPG.dat	1049.3	36.6	1.0	0.04	-0.031	0.036	1.4E-05
YIL168W	YIL168W	S288C_Ator08_hmg124h_7.JPG.dat	1103.3	10.7	1.0	0.02	-0.004	0.017	1.4E-06
YIL170W	YIL170W	S288C_Ator08_hmg124h_7.JPG.dat	1103.0	18.7	1.0	0.02	-0.063	0.016	1.3E-06
YIL173W	YIL173W	S288C_Ator08_hmg124h_7.JPG.dat	1098.5	23.6	1.0	0.02	-0.011	0.021	2.9E-06
YIR001C	YIR001C	S288C_Ator08_hmg124h_7.JPG.dat	1070.0	101.4	1.0	0.03	-0.043	0.026	2.5E-04

YIR002C	YIR002C	S288C_Ator08_hmg124h_7.JPG.dat	1074.8	29.0	0.9	0.03	0.049	0.031	1.1E-05
YIR003W	YIR003W	S288C_Ator08_hmg124h_7.JPG.dat	1125.8	62.2	1.0	0.04	0.011	0.041	1.8E-05
YIR005W	YIR005W	S288C_Ator08_hmg124h_7.JPG.dat	999.5	40.3	0.9	0.04	0.013	0.036	1.7E-05
YIR007W	YIR007W	S288C_Ator08_hmg124h_7.JPG.dat	1145.3	4.8	1.0	0.00	0.021	0.001	5.9E-07
YIR009W	YIR009W	S288C_Ator08_hmg124h_7.JPG.dat	1104.8	14.0	1.0	0.01	-0.037	0.014	7.3E-07
YIR013C	YIR013C	S288C_Ator08_hmg124h_7.JPG.dat	1099.3	36.9	1.0	0.03	0.047	0.034	1.0E-05
YIR014W	YIR014W	S288C_Ator08_hmg124h_7.JPG.dat	1103.8	43.8	1.1	0.01	0.041	0.014	6.3E-05
YIR016W	YIR016W	S288C_Ator08_hmg124h_7.JPG.dat	1074.3	12.3	1.0	0.00	0.003	0.005	7.2E-06
YIR017C	YIR017C	S288C_Ator08_hmg124h_7.JPG.dat	1126.8	28.3	1.0	0.03	-0.019	0.028	5.7E-06
YIR018W	YIR018W	S288C_Ator08_hmg124h_7.JPG.dat	1161.3	19.0	1.0	0.02	0.017	0.017	1.2E-06
YIR019C	YIR019C	S288C_Ator08_hmg124h_7.JPG.dat	1097.5	45.0	1.0	0.04	-0.035	0.045	2.6E-05
YIR020C	YIR020C	S288C_Ator08_hmg124h_7.JPG.dat	1059.5	124.0	1.0	0.02	-0.051	0.021	1.5E-04
YIR020W-E	YIR020W-B	S288C_Ator08_hmg124h_7.JPG.dat	1065.5	9.9	1.0	0.02	-0.015	0.017	1.3E-06
YIR023W	YIR023W	S288C_Ator08_hmg124h_7.JPG.dat	1088.3	37.4	1.0	0.03	0.001	0.033	1.0E-05
YIR024C	YIR024C	S288C_Ator08_hmg124h_7.JPG.dat	1115.0	12.4	1.0	0.01	0.046	0.010	3.1E-07
YIR025W	YIR025W	S288C_Ator08_hmg124h_7.JPG.dat	1106.8	30.3	1.0	0.03	-0.010	0.026	4.8E-06
YIR027C	YIR027C	S288C_Ator08_hmg124h_7.JPG.dat	1034.8	102.2	1.0	0.09	-0.023	0.089	2.1E-04
YIR028W	YIR028W	S288C_Ator08_hmg124h_7.JPG.dat	996.0	32.8	1.0	0.03	-0.028	0.029	7.7E-06
YIR029W	YIR029W	S288C_Ator08_hmg124h_7.JPG.dat	1052.0	21.6	1.0	0.02	-0.013	0.021	2.5E-06
YIR030C	YIR030C	S288C_Ator08_hmg124h_7.JPG.dat	1086.3	19.6	1.0	0.00	-0.010	0.003	3.0E-06
YIR031C	YIR031C	S288C_Ator08_hmg124h_7.JPG.dat	1154.0	31.5	1.0	0.03	-0.010	0.031	7.7E-06
YIR032C	YIR032C	S288C_Ator08_hmg124h_7.JPG.dat	1109.8	13.7	1.0	0.00	-0.023	0.004	5.4E-06
YIR033W	YIR033W	S288C_Ator08_hmg124h_7.JPG.dat	1095.5	24.5	1.0	0.03	0.031	0.031	8.2E-06
YIR034C	YIR034C	S288C_Ator08_hmg124h_7.JPG.dat	0.0	0.0	0.0	0.00	0.000	0.000	
YIR035C	YIR035C	S288C_Ator08_hmg124h_7.JPG.dat	1086.0	18.1	1.0	0.00	0.004	0.004	5.1E-06
YIR036C	YIR036C	S288C_Ator08_hmg124h_7.JPG.dat	1086.0	22.4	1.0	0.02	-0.043	0.020	2.1E-06
YIR037W	YIR037W	S288C_Ator08_hmg124h_7.JPG.dat	1086.3	40.4	1.0	0.01	-0.023	0.007	1.5E-05
YIR038C	YIR038C	S288C_Ator08_hmg124h_7.JPG.dat	1043.8	17.8	1.0	0.02	0.013	0.022	2.7E-06
YIR039C	YIR039C	S288C_Ator08_hmg124h_7.JPG.dat	979.3	5.4	1.0	0.00	-0.035	0.000	2.0E-11
YIR042C	YIR042C	S288C_Ator08_hmg124h_7.JPG.dat	991.8	41.0	1.0	0.04	0.047	0.041	1.9E-05
YIR043C	YIR043C	S288C_Ator08_hmg124h_7.JPG.dat	1037.0	16.2	1.0	0.00	-0.033	0.001	7.2E-07
YIR044C	YIR044C	S288C_Ator08_hmg124h_7.JPG.dat	1028.8	50.3	1.0	0.04	0.008	0.041	2.1E-05
YJL004C	YJL004C	S288C_Ator08_hmg124h_7.JPG.dat	757.3	55.1	0.7	0.05	-0.085	0.052	1.1E-04
YJL007C	YJL007C	S288C_Ator08_hmg124h_7.JPG.dat	1097.3	48.5	1.0	0.04	0.010	0.043	1.9E-05
YJL012C	YJL012C	S288C_Ator08_hmg124h_7.JPG.dat	1061.3	34.2	1.0	0.03	0.027	0.026	4.4E-06
YJL013C	YJL013C	S288C_Ator08_hmg124h_7.JPG.dat	980.3	51.1	0.9	0.05	-0.028	0.049	4.1E-05
YJL016W	YJL016W	S288C_Ator08_hmg124h_7.JPG.dat	1044.8	56.2	1.0	0.05	-0.027	0.053	4.2E-05
YJL017W	YJL017W	S288C_Ator08_hmg124h_7.JPG.dat	1082.0	34.0	1.0	0.04	-0.008	0.035	1.1E-05
YJL020C	YJL020C	S288C_Ator08_hmg124h_7.JPG.dat	1011.0	23.9	1.0	0.02	0.032	0.019	1.9E-06
YJL021C	YJL021C	S288C_Ator08_hmg124h_7.JPG.dat	995.8	30.1	1.0	0.01	0.055	0.007	1.4E-05
YJL022W	YJL022W	S288C_Ator08_hmg124h_7.JPG.dat	951.5	9.5	1.0	0.00	-0.053	0.000	5.9E-08
YJL023C	YJL023C	S288C_Ator08_hmg124h_7.JPG.dat	1012.5	50.5	1.0	0.05	-0.003	0.055	4.5E-05
YJL024C	YJL024C	S288C_Ator08_hmg124h_7.JPG.dat	898.3	68.8	0.9	0.07	-0.095	0.065	1.2E-04
YJL027C	YJL027C	S288C_Ator08_hmg124h_7.JPG.dat	1059.0	66.6	1.0	0.01	0.057	0.010	3.1E-05
YJL028W	YJL028W	S288C_Ator08_hmg124h_7.JPG.dat	886.8	349.3	1.0	0.02	-0.036	0.017	9.4E-05
YJL030W	YJL030W	S288C_Ator08_hmg124h_7.JPG.dat	1081.3	37.3	1.0	0.02	0.007	0.024	3.4E-06
YJL036W	YJL036W	S288C_Ator08_hmg124h_7.JPG.dat	1093.5	10.6	1.0	0.01	0.069	0.010	2.4E-07
YJL037W	YJL037W	S288C_Ator08_hmg124h_7.JPG.dat	1070.3	20.7	1.0	0.02	-0.025	0.019	1.9E-06
YJL038C	YJL038C	S288C_Ator08_hmg124h_7.JPG.dat	1062.3	25.3	1.0	0.03	-0.036	0.026	5.0E-06
YJL042W	YJL042W	S288C_Ator08_hmg124h_7.JPG.dat	1014.3	33.1	1.0	0.04	-0.012	0.036	1.4E-05
YJL043W	YJL043W	S288C_Ator08_hmg124h_7.JPG.dat	1021.0	14.8	1.0	0.02	-0.008	0.016	1.0E-06
YJL044C	YJL044C	S288C_Ator08_hmg124h_7.JPG.dat	992.5	10.8	1.0	0.00	-0.007	0.003	3.2E-06
YJL045W	YJL045W	S288C_Ator08_hmg124h_7.JPG.dat	1051.3	22.3	1.0	0.02	0.023	0.019	1.8E-06
YJL046W	YJL046W	S288C_Ator08_hmg124h_7.JPG.dat	1041.3	22.3	1.0	0.01	0.265	0.007	1.7E-05
YJL047C	YJL047C	S288C_Ator08_hmg124h_7.JPG.dat	1052.5	13.8	1.0	0.01	0.053	0.014	8.1E-07
YJL048C	YJL048C	S288C_Ator08_hmg124h_7.JPG.dat	1066.8	31.4	1.0	0.03	-0.013	0.032	8.7E-06
YJL049W	YJL049W	S288C_Ator08_hmg124h_7.JPG.dat	1071.3	21.6	1.0	0.00	0.014	0.004	5.1E-06
YJL051W	YJL051W	S288C_Ator08_hmg124h_7.JPG.dat	1190.5	35.3	1.1	0.03	0.003	0.027	3.9E-06
YJL053W	YJL053W	S288C_Ator08_hmg124h_7.JPG.dat	922.0	26.6	0.8	0.00	-0.077	0.002	1.6E-06
YJL055W	YJL055W	S288C_Ator08_hmg124h_7.JPG.dat	1064.8	31.8	1.0	0.00	-0.040	0.004	6.8E-06
YJL057C	YJL057C	S288C_Ator08_hmg124h_7.JPG.dat	1079.5	10.6	1.0	0.01	-0.016	0.010	2.8E-07
YJL058C	YJL058C	S288C_Ator08_hmg124h_7.JPG.dat	1007.5	32.8	1.0	0.03	-0.057	0.030	7.8E-06
YJL059W	YJL059W	S288C_Ator08_hmg124h_7.JPG.dat	1016.3	11.6	1.0	0.00	-0.003	0.003	8.0E-09
YJL060W	YJL060W	S288C_Ator08_hmg124h_7.JPG.dat	1058.3	17.5	1.0	0.01	0.047	0.015	8.5E-07
YJL062W	YJL062W	S288C_Ator08_hmg124h_7.JPG.dat	935.0	11.6	0.9	0.02	0.872	0.016	1.9E-06
YJL064W	YJL064W	S288C_Ator08_hmg124h_7.JPG.dat	1025.8	7.6	1.0	0.00	-0.016	0.002	2.0E-06
YJL065C	YJL065C	S288C_Ator08_hmg124h_7.JPG.dat	993.8	22.4	0.9	0.03	-0.048	0.028	7.4E-06
YJL066C	YJL066C	S288C_Ator08_hmg124h_7.JPG.dat	1127.5	19.2	1.0	0.03	0.034	0.027	5.6E-06
YJL067W	YJL067W	S288C_Ator08_hmg124h_7.JPG.dat	1207.3	28.2	1.1	0.03	-0.003	0.025	3.6E-06
YJL068C	YJL068C	S288C_Ator08_hmg124h_7.JPG.dat	1184.0	24.9	1.0	0.02	0.003	0.023	3.1E-06

YJL070C	YJL070C	S288C_Ator08_hmg124h_7.JPG.dat	1204.5	24.3	1.1	0.02	0.050	0.023	2.9E-06
YJL071W	YJL071W	S288C_Ator08_hmg124h_7.JPG.dat	274.8	549.5	0.0	0.00	0.000	0.000	
YJL073W	YJL073W	S288C_Ator08_hmg124h_7.JPG.dat	1126.5	11.2	1.1	0.00	0.045	0.003	2.7E-06
YJL077C	YJL077C	S288C_Ator08_hmg124h_7.JPG.dat	1091.0	34.3	1.0	0.01	-0.004	0.011	3.9E-05
YJL078C	YJL078C	S288C_Ator08_hmg124h_7.JPG.dat	1113.0	46.4	1.0	0.04	-0.017	0.044	2.5E-05
YJL083W	YJL083W	S288C_Ator08_hmg124h_7.JPG.dat	1099.0	20.7	1.0	0.02	-0.078	0.018	2.0E-06
YJL084C	YJL084C	S288C_Ator08_hmg124h_7.JPG.dat	1083.0	25.1	0.9	0.02	-0.014	0.020	2.5E-06
YJL088W	YJL088W	S288C_Ator08_hmg124h_7.JPG.dat	545.3	630.2	0.5	0.54	-0.019	0.539	1.8E-01
YJL089W	YJL089W	S288C_Ator08_hmg124h_7.JPG.dat	1081.0	53.2	0.9	0.05	-0.096	0.045	3.0E-05
YJL093C	YJL093C	S288C_Ator08_hmg124h_7.JPG.dat	1119.3	61.3	1.0	0.06	-0.001	0.065	7.6E-05
YJL094C	YJL094C	S288C_Ator08_hmg124h_7.JPG.dat	1134.8	19.2	1.0	0.00	0.014	0.004	5.0E-06
YJL095W	YJL095W	S288C_Ator08_hmg124h_7.JPG.dat	781.0	258.2	0.7	0.23	-0.259	0.233	9.1E-03
YJL098W	YJL098W	S288C_Ator08_hmg124h_7.JPG.dat	1099.5	21.3	1.0	0.01	-0.035	0.010	3.1E-07
YJL099W	YJL099W	S288C_Ator08_hmg124h_7.JPG.dat	1116.3	35.6	1.0	0.00	-0.079	0.003	2.3E-06
YJL100W	YJL100W	S288C_Ator08_hmg124h_7.JPG.dat	1132.8	17.2	1.0	0.00	0.030	0.002	8.6E-10
YJL101C	YJL101C	S288C_Ator08_hmg124h_7.JPG.dat	1152.3	57.1	1.0	0.04	0.046	0.036	1.2E-05
YJL105W	YJL105W	S288C_Ator08_hmg124h_7.JPG.dat	1114.3	24.0	1.0	0.04	-0.026	0.036	1.4E-05
YJL108C	YJL108C	S288C_Ator08_hmg124h_7.JPG.dat	1034.0	139.4	0.9	0.10	0.016	0.100	4.0E-04
YJL110C	YJL110C	S288C_Ator08_hmg124h_7.JPG.dat	1063.3	32.8	0.9	0.00	-0.212	0.004	5.8E-06
YJL112W	YJL112W	S288C_Ator08_hmg124h_7.JPG.dat	1086.8	39.6	1.0	0.01	-0.022	0.006	1.1E-05
YJL115W	YJL115W	S288C_Ator08_hmg124h_7.JPG.dat	825.3	34.9	0.7	0.03	0.144	0.028	1.4E-05
YJL116C	YJL116C	S288C_Ator08_hmg124h_7.JPG.dat	1111.3	23.5	1.0	0.02	-0.025	0.019	1.8E-06
YJL121C	YJL121C	S288C_Ator08_hmg124h_7.JPG.dat	931.0	70.9	0.8	0.06	-0.042	0.063	1.1E-04
YJL122W	YJL122W	S288C_Ator08_hmg124h_7.JPG.dat	1099.5	18.5	1.0	0.01	0.037	0.009	2.1E-07
YJL123C	YJL123C	S288C_Ator08_hmg124h_7.JPG.dat	1073.8	26.1	1.0	0.02	0.054	0.023	3.1E-06
YJL124C	YJL124C	S288C_Ator08_hmg124h_7.JPG.dat	818.0	126.2	0.8	0.13	-0.119	0.126	1.2E-03
YJL126W	YJL126W	S288C_Ator08_hmg124h_7.JPG.dat	1127.0	20.3	1.0	0.03	-0.066	0.026	4.9E-06
YJL128C	YJL128C	S288C_Ator08_hmg124h_7.JPG.dat	1120.8	39.7	1.0	0.03	-0.006	0.032	8.7E-06
YJL130C	YJL130C	S288C_Ator08_hmg124h_7.JPG.dat	1080.3	41.7	1.0	0.04	-0.012	0.036	1.5E-05
YJL131C	YJL131C	S288C_Ator08_hmg124h_7.JPG.dat	1092.5	41.3	1.0	0.03	-0.069	0.034	1.1E-05
YJL132W	YJL132W	S288C_Ator08_hmg124h_7.JPG.dat	1107.3	80.0	1.0	0.01	-0.044	0.011	3.7E-05
YJL133W	YJL133W	S288C_Ator08_hmg124h_7.JPG.dat	1110.3	27.4	1.0	0.01	-0.002	0.014	7.0E-07
YJL134W	YJL134W	S288C_Ator08_hmg124h_7.JPG.dat	1171.3	26.3	1.1	0.01	0.089	0.012	4.2E-07
YJL135W	YJL135W	S288C_Ator08_hmg124h_7.JPG.dat	1119.0	32.1	1.0	0.02	0.017	0.019	1.6E-06
YJL136C	YJL136C	S288C_Ator08_hmg124h_7.JPG.dat	882.8	50.4	0.9	0.04	0.099	0.037	2.1E-05
YJL137C	YJL137C	S288C_Ator08_hmg124h_7.JPG.dat	1014.8	24.9	1.0	0.01	0.009	0.010	3.0E-05
YJL138C	YJL138C	S288C_Ator08_hmg124h_7.JPG.dat	970.5	74.4	1.0	0.06	0.045	0.057	5.8E-05
YJL139C	YJL139C	S288C_Ator08_hmg124h_7.JPG.dat	789.3	527.8	1.0	0.06	0.005	0.061	1.2E-03
YJL141C	YJL141C	S288C_Ator08_hmg124h_7.JPG.dat	1075.3	62.3	1.0	0.06	0.039	0.058	5.4E-05
YJL142C	YJL142C	S288C_Ator08_hmg124h_7.JPG.dat	1136.5	13.8	1.0	0.03	0.005	0.026	4.1E-06
YJL144W	YJL144W	S288C_Ator08_hmg124h_7.JPG.dat	1055.3	32.6	1.0	0.01	-0.045	0.014	7.0E-07
YJL145W	YJL145W	S288C_Ator08_hmg124h_7.JPG.dat	1076.5	43.0	1.0	0.04	0.014	0.043	2.1E-05
YJL146W	YJL146W	S288C_Ator08_hmg124h_7.JPG.dat	1067.3	42.4	1.0	0.04	-0.002	0.039	1.7E-05
YJL147C	YJL147C	S288C_Ator08_hmg124h_7.JPG.dat	1039.5	11.0	1.0	0.01	0.020	0.009	2.1E-07
YJL148W	YJL148W	S288C_Ator08_hmg124h_7.JPG.dat	1033.5	24.7	1.0	0.02	-0.002	0.021	2.5E-06
YJL149W	YJL149W	S288C_Ator08_hmg124h_7.JPG.dat	959.8	34.8	1.0	0.04	-0.010	0.040	2.0E-05
YJL150W	YJL150W	S288C_Ator08_hmg124h_7.JPG.dat	1002.0	24.5	1.0	0.02	-0.015	0.022	3.0E-06
YJL151C	YJL151C	S288C_Ator08_hmg124h_7.JPG.dat	945.8	23.6	0.9	0.02	-0.063	0.022	3.6E-06
YJL152W	YJL152W	S288C_Ator08_hmg124h_7.JPG.dat	1050.3	23.9	1.0	0.02	0.007	0.021	2.4E-06
YJL153C	YJL153C	S288C_Ator08_hmg124h_7.JPG.dat	1006.5	94.2	1.0	0.09	0.008	0.088	2.2E-04
YJL154C	YJL154C	S288C_Ator08_hmg124h_7.JPG.dat	1117.3	20.6	1.0	0.02	-0.065	0.021	2.4E-06
YJL155C	YJL155C	S288C_Ator08_hmg124h_7.JPG.dat	1066.0	43.0	1.1	0.03	0.032	0.030	6.5E-06
YJL157C	YJL157C	S288C_Ator08_hmg124h_7.JPG.dat	1030.8	47.9	1.0	0.01	-0.016	0.014	7.5E-05
YJL158C	YJL158C	S288C_Ator08_hmg124h_7.JPG.dat	1089.0	28.2	1.0	0.03	-0.012	0.027	4.5E-06
YJL159W	YJL159W	S288C_Ator08_hmg124h_7.JPG.dat	993.3	32.9	1.0	0.03	-0.103	0.034	1.3E-05
YJL160C	YJL160C	S288C_Ator08_hmg124h_7.JPG.dat	488.3	566.3	0.5	0.55	-0.013	0.553	1.8E-01
YJL161W	YJL161W	S288C_Ator08_hmg124h_7.JPG.dat	1002.5	20.8	1.0	0.03	-0.028	0.029	6.3E-06
YJL162C	YJL162C	S288C_Ator08_hmg124h_7.JPG.dat	977.8	27.4	1.0	0.03	0.000	0.028	6.2E-06
YJL163C	YJL163C	S288C_Ator08_hmg124h_7.JPG.dat	1006.3	30.2	1.0	0.02	0.034	0.024	3.6E-06
YJL164C	YJL164C	S288C_Ator08_hmg124h_7.JPG.dat	975.3	103.3	1.0	0.10	-0.021	0.096	2.8E-04
YJL165C	YJL165C	S288C_Ator08_hmg124h_7.JPG.dat	974.3	41.9	0.9	0.04	0.159	0.044	2.8E-05
YJL168C	YJL168C	S288C_Ator08_hmg124h_7.JPG.dat	940.3	27.6	0.9	0.02	0.032	0.023	4.7E-06
YJL169W	YJL169W	S288C_Ator08_hmg124h_7.JPG.dat	955.0	56.6	1.0	0.06	0.141	0.056	5.5E-05
YJL170C	YJL170C	S288C_Ator08_hmg124h_7.JPG.dat	1151.5	23.4	1.1	0.02	0.045	0.023	2.8E-06
YJL171C	YJL171C	S288C_Ator08_hmg124h_7.JPG.dat	1093.0	34.3	1.0	0.01	0.042	0.007	1.5E-05
YJL172W	YJL172W	S288C_Ator08_hmg124h_7.JPG.dat	1039.5	38.1	1.0	0.03	0.050	0.033	1.1E-05
YJL175W	YJL175W	S288C_Ator08_hmg124h_7.JPG.dat	735.3	83.8	0.7	0.08	0.043	0.078	3.8E-04
YJL176C	YJL176C	S288C_Ator08_hmg124h_7.JPG.dat	1047.8	17.6	1.0	0.02	-0.042	0.017	1.3E-06
YJL178C	YJL178C	S288C_Ator08_hmg124h_7.JPG.dat	1002.0	41.9	1.0	0.04	0.055	0.042	2.1E-05
YJL181W	YJL181W	S288C_Ator08_hmg124h_7.JPG.dat	1028.8	23.3	1.0	0.02	0.041	0.015	9.2E-07

YJL182C	YJL182C	S288C_Ator08_hmg124h_7.JPG.dat	1039.5	42.8	1.0	0.04	-0.037	0.042	2.2E-05
YJL183W	YJL183W	S288C_Ator08_hmg124h_7.JPG.dat	1059.5	14.3	1.0	0.00	-0.023	0.002	1.2E-06
YJL185C	YJL185C	S288C_Ator08_hmg124h_7.JPG.dat	1044.5	87.6	1.0	0.08	0.005	0.084	1.7E-04
YJL186W	YJL186W	S288C_Ator08_hmg124h_7.JPG.dat	1130.3	37.5	1.1	0.01	0.080	0.013	4.5E-05
YJL187C	YJL187C	S288C_Ator08_hmg124h_7.JPG.dat	846.8	564.5	1.0	0.01	-0.056	0.008	2.0E-05
YJL188C	YJL188C	S288C_Ator08_hmg124h_7.JPG.dat	1001.5	48.9	0.9	0.04	-0.059	0.041	2.6E-05
YJL190C	YJL190C	S288C_Ator08_hmg124h_7.JPG.dat	1150.0	50.1	1.1	0.04	0.041	0.038	1.3E-05
YJL191W	YJL191W	S288C_Ator08_hmg124h_7.JPG.dat	1077.8	12.8	1.0	0.01	0.019	0.010	2.6E-07
YJL192C	YJL192C	S288C_Ator08_hmg124h_7.JPG.dat	1057.8	51.3	1.0	0.04	-0.043	0.044	2.2E-05
YJL193W	YJL193W	S288C_Ator08_hmg124h_7.JPG.dat	1036.3	32.3	1.0	0.02	0.007	0.023	3.2E-06
YJL196C	YJL196C	S288C_Ator08_hmg124h_7.JPG.dat	1088.0	25.1	1.0	0.01	-0.009	0.015	7.8E-07
YJL197W	YJL197W	S288C_Ator08_hmg124h_7.JPG.dat	1062.8	25.1	1.0	0.00	0.018	0.002	1.3E-06
YJL198W	YJL198W	S288C_Ator08_hmg124h_7.JPG.dat	1031.5	140.9	1.0	0.03	-0.056	0.034	3.7E-04
YJL199C	YJL199C	S288C_Ator08_hmg124h_7.JPG.dat	834.8	556.7	1.0	0.02	-0.048	0.021	1.4E-04
YJL201W	YJL201W	S288C_Ator08_hmg124h_7.JPG.dat	1030.5	94.2	1.0	0.03	0.027	0.026	2.3E-04
YJL204C	YJL204C	S288C_Ator08_hmg124h_7.JPG.dat	453.3	560.8	0.2	0.05	-0.208	0.051	3.5E-02
YJL206C	YJL206C	S288C_Ator08_hmg124h_7.JPG.dat	1109.3	19.8	1.0	0.01	-0.051	0.010	3.3E-07
YJL206C-A	YJL206C-A	S288C_Ator08_hmg124h_7.JPG.dat	1129.5	19.5	1.0	0.01	0.013	0.015	8.3E-07
YJL207C	YJL207C	S288C_Ator08_hmg124h_7.JPG.dat	1179.0	16.4	1.1	0.00	0.054	0.002	1.3E-06
YJL208C	YJL208C	S288C_Ator08_hmg124h_7.JPG.dat	1167.8	23.7	1.1	0.01	0.034	0.005	8.6E-06
YJL210W	YJL210W	S288C_Ator08_hmg124h_7.JPG.dat	1104.0	34.4	1.0	0.01	0.034	0.005	8.9E-06
YJL211C	YJL211C	S288C_Ator08_hmg124h_7.JPG.dat	1098.8	42.4	1.0	0.02	0.058	0.024	3.5E-06
YJL212C	YJL212C	S288C_Ator08_hmg124h_7.JPG.dat	1129.0	99.9	1.0	0.00	0.021	0.003	2.9E-06
YJL213W	YJL213W	S288C_Ator08_hmg124h_7.JPG.dat	1172.0	41.9	1.0	0.01	0.013	0.007	1.8E-05
YJL214W	YJL214W	S288C_Ator08_hmg124h_7.JPG.dat	1122.8	50.1	1.0	0.03	-0.004	0.031	8.8E-06
YJL215C	YJL215C	S288C_Ator08_hmg124h_7.JPG.dat	1080.0	111.6	0.9	0.08	-0.087	0.082	2.0E-04
YJL216C	YJL216C	S288C_Ator08_hmg124h_7.JPG.dat	1100.5	65.3	1.0	0.07	0.009	0.065	7.2E-05
YJL217W	YJL217W	S288C_Ator08_hmg124h_7.JPG.dat	1125.8	30.3	1.0	0.02	-0.014	0.024	3.6E-06
YJL218W	YJL218W	S288C_Ator08_hmg124h_7.JPG.dat	1113.8	45.0	1.0	0.01	0.024	0.011	3.6E-05
YJR001W	YJR001W	S288C_Ator08_hmg124h_7.JPG.dat	1101.0	17.8	1.0	0.02	0.007	0.020	2.2E-06
YJR003C	YJR003C	S288C_Ator08_hmg124h_7.JPG.dat	1082.0	32.9	1.0	0.03	-0.004	0.032	8.8E-06
YJR005W	YJR005W	S288C_Ator08_hmg124h_7.JPG.dat	972.5	41.3	0.9	0.04	-0.071	0.038	2.0E-05
YJR008W	YJR008W	S288C_Ator08_hmg124h_7.JPG.dat	1097.8	20.8	1.0	0.02	-0.013	0.016	9.5E-07
YJR009C	YJR009C	S288C_Ator08_hmg124h_7.JPG.dat	1114.3	20.1	1.0	0.02	-0.032	0.016	1.1E-06
YJR010C-A	YJR010C-A	S288C_Ator08_hmg124h_7.JPG.dat	1125.5	22.2	1.0	0.02	-0.017	0.018	1.6E-06
YJR010W	YJR010W	S288C_Ator08_hmg124h_7.JPG.dat	1126.0	25.9	1.0	0.00	-0.017	0.004	6.7E-06
YJR011C	YJR011C	S288C_Ator08_hmg124h_7.JPG.dat	940.8	117.4	0.9	0.11	-0.094	0.113	6.3E-04
YJR014W	YJR014W	S288C_Ator08_hmg124h_7.JPG.dat	961.0	21.4	0.9	0.03	-0.027	0.029	8.2E-06
YJR015W	YJR015W	S288C_Ator08_hmg124h_7.JPG.dat	1087.0	20.8	1.0	0.02	0.010	0.018	1.5E-06
YJR019C	YJR019C	S288C_Ator08_hmg124h_7.JPG.dat	1114.0	15.9	1.0	0.03	0.006	0.027	5.0E-06
YJR020W	YJR020W	S288C_Ator08_hmg124h_7.JPG.dat	1074.3	38.6	1.0	0.03	0.005	0.028	6.1E-06
YJR021C	YJR021C	S288C_Ator08_hmg124h_7.JPG.dat	1033.8	17.3	1.0	0.02	-0.025	0.021	2.6E-06
YJR024C	YJR024C	S288C_Ator08_hmg124h_7.JPG.dat	960.8	60.1	1.0	0.05	-0.039	0.047	3.3E-05
YJR025C	YJR025C	S288C_Ator08_hmg124h_7.JPG.dat	975.3	81.8	1.0	0.02	0.052	0.024	1.9E-04
YJR026W	YJR026W	S288C_Ator08_hmg124h_7.JPG.dat	1054.8	25.3	1.0	0.01	-0.006	0.014	7.6E-07
YJR030C	YJR030C	S288C_Ator08_hmg124h_7.JPG.dat	1118.8	24.1	1.0	0.02	0.010	0.022	2.7E-06
YJR031C	YJR031C	S288C_Ator08_hmg124h_7.JPG.dat	1053.3	66.4	1.0	0.05	-0.052	0.054	4.7E-05
YJR032W	YJR032W	S288C_Ator08_hmg124h_7.JPG.dat	293.5	512.0	0.0	0.06	0.037	0.064	4.2E-01
YJR033C	YJR033C	S288C_Ator08_hmg124h_7.JPG.dat	1056.8	70.5	1.0	0.06	-0.001	0.059	5.3E-05
YJR035W	YJR035W	S288C_Ator08_hmg124h_7.JPG.dat	1015.8	29.7	0.9	0.01	-0.044	0.010	3.8E-05
YJR036C	YJR036C	S288C_Ator08_hmg124h_7.JPG.dat	1114.8	32.2	1.0	0.00	-0.003	0.004	4.4E-06
YJR037W	YJR037W	S288C_Ator08_hmg124h_7.JPG.dat	1104.5	13.9	1.0	0.01	-0.012	0.011	3.3E-07
YJR038C	YJR038C	S288C_Ator08_hmg124h_7.JPG.dat	1027.3	23.2	1.0	0.02	0.008	0.024	3.7E-06
YJR040W	YJR040W	S288C_Ator08_hmg124h_7.JPG.dat	1000.5	51.7	1.0	0.06	-0.043	0.055	4.5E-05
YJR043C	YJR043C	S288C_Ator08_hmg124h_7.JPG.dat	962.5	70.7	1.0	0.07	-0.037	0.068	9.4E-05
YJR047C	YJR047C	S288C_Ator08_hmg124h_7.JPG.dat	996.3	15.6	1.0	0.01	-0.045	0.009	2.3E-07
YJR048W	YJR048W	S288C_Ator08_hmg124h_7.JPG.dat	1037.3	9.5	1.0	0.01	-0.013	0.008	1.7E-07
YJR049C	YJR049C	S288C_Ator08_hmg124h_7.JPG.dat	834.5	557.3	1.0	0.04	-0.017	0.035	3.8E-04
YJR050W	YJR050W	S288C_Ator08_hmg124h_7.JPG.dat	1081.3	23.0	1.0	0.03	0.044	0.026	4.4E-06
YJR051W	YJR051W	S288C_Ator08_hmg124h_7.JPG.dat	1066.8	56.3	1.0	0.04	0.012	0.037	1.3E-05
YJR052W	YJR052W	S288C_Ator08_hmg124h_7.JPG.dat	1082.3	40.5	1.1	0.01	0.029	0.011	3.9E-05
YJR053W	YJR053W	S288C_Ator08_hmg124h_7.JPG.dat	1059.0	48.8	1.0	0.05	0.063	0.046	2.6E-05
YJR054W	YJR054W	S288C_Ator08_hmg124h_7.JPG.dat	1028.0	28.3	1.0	0.03	-0.007	0.028	6.0E-06
YJR058C	YJR058C	S288C_Ator08_hmg124h_7.JPG.dat	995.5	21.9	1.0	0.02	0.013	0.020	2.2E-06
YJR059W	YJR059W	S288C_Ator08_hmg124h_7.JPG.dat	1000.0	14.9	1.0	0.01	0.035	0.012	4.7E-07
YJR060W	YJR060W	S288C_Ator08_hmg124h_7.JPG.dat	846.3	41.3	0.9	0.05	-0.125	0.048	4.5E-05
YJR061W	YJR061W	S288C_Ator08_hmg124h_7.JPG.dat	1033.0	8.3	1.0	0.00	-0.011	0.003	3.4E-06
YJR062C	YJR062C	S288C_Ator08_hmg124h_7.JPG.dat	1033.3	31.8	1.0	0.03	-0.015	0.028	5.9E-06
YJR069C	YJR069C	S288C_Ator08_hmg124h_7.JPG.dat	1052.3	27.9	1.0	0.03	-0.006	0.030	6.9E-06
YJR070C	YJR070C	S288C_Ator08_hmg124h_7.JPG.dat	1015.0	85.2	1.0	0.03	0.050	0.027	2.3E-04

YJR073C	YJR073C	S288C_Ator08_hmg124h_7.JPG.dat	556.3	643.1	0.5	0.62	0.086	0.619	1.8E-01
YJR074W	YJR074W	S288C_Ator08_hmg124h_7.JPG.dat	1051.0	40.8	1.0	0.04	0.205	0.038	1.6E-05
YJR075W	YJR075W	S288C_Ator08_hmg124h_7.JPG.dat	1035.5	24.6	1.0	0.02	-0.140	0.021	2.8E-06
YJR077C	YJR077C	S288C_Ator08_hmg124h_7.JPG.dat	1039.8	47.2	1.0	0.01	0.041	0.014	6.2E-05
YJR078W	YJR078W	S288C_Ator08_hmg124h_7.JPG.dat	1002.8	42.8	1.0	0.01	-0.010	0.012	4.9E-05
YJR079W	YJR079W	S288C_Ator08_hmg124h_7.JPG.dat	1002.8	9.2	1.0	0.01	-0.005	0.010	3.1E-07
YJR080C	YJR080C	S288C_Ator08_hmg124h_7.JPG.dat	1002.3	5.6	1.0	0.00	0.055	0.004	1.3E-08
YJR082C	YJR082C	S288C_Ator08_hmg124h_7.JPG.dat	1028.3	46.1	1.0	0.04	0.016	0.040	1.9E-05
YJR083C	YJR083C	S288C_Ator08_hmg124h_7.JPG.dat	1062.8	16.9	1.0	0.01	-0.074	0.014	7.7E-07
YJR084W	YJR084W	S288C_Ator08_hmg124h_7.JPG.dat	521.8	607.1	0.5	0.58	0.117	0.575	1.8E-01
YJR087W	YJR087W	S288C_Ator08_hmg124h_7.JPG.dat	964.3	116.9	1.0	0.00	0.005	0.002	8.6E-07
YJR088C	YJR088C	S288C_Ator08_hmg124h_7.JPG.dat	1024.8	62.0	1.0	0.07	0.000	0.065	8.7E-05
YJR090C	YJR090C	S288C_Ator08_hmg124h_7.JPG.dat	265.0	530.0	0.0	0.00	0.000	0.000	
YJR091C	YJR091C	S288C_Ator08_hmg124h_7.JPG.dat	1076.0	38.3	1.0	0.01	-0.014	0.014	6.9E-05
YJR092W	YJR092W	S288C_Ator08_hmg124h_7.JPG.dat	1147.8	45.8	1.0	0.04	0.069	0.037	1.2E-05
YJR094C	YJR094C	S288C_Ator08_hmg124h_7.JPG.dat	1085.3	22.1	1.0	0.02	0.094	0.019	1.8E-06
YJR094W-/	YJR094W-A	S288C_Ator08_hmg124h_7.JPG.dat	1055.0	20.1	1.0	0.02	0.002	0.017	1.3E-06
YJR095W	YJR095W	S288C_Ator08_hmg124h_7.JPG.dat	1003.5	17.8	1.0	0.03	0.086	0.026	5.4E-06
YJR096W	YJR096W	S288C_Ator08_hmg124h_7.JPG.dat	1070.5	42.3	1.0	0.03	0.050	0.033	9.7E-06
YJR097W	YJR097W	S288C_Ator08_hmg124h_7.JPG.dat	1102.8	31.5	1.0	0.03	0.096	0.028	5.6E-06
YJR098C	YJR098C	S288C_Ator08_hmg124h_7.JPG.dat	1136.5	36.7	1.0	0.01	-0.029	0.011	4.0E-05
YJR099W	YJR099W	S288C_Ator08_hmg124h_7.JPG.dat	934.8	95.8	0.9	0.10	0.179	0.102	4.6E-04
YOR202W	YOR202W	S288C_Ator08_hmg124h_7.JPG.dat	1067.1	120.7	1.0	0.08	-0.001	0.085	4.5E-292
1	1	S288C_Ator08_hmg124h_12.JPG.dat	1343.8	83.4	1.1	0.00	0.025	0.004	4.3E-06
2	2	S288C_Ator08_hmg124h_12.JPG.dat	1148.3	39.9	1.0	0.04	0.017	0.035	1.3E-05
3	3	S288C_Ator08_hmg124h_12.JPG.dat	1102.0	29.3	1.0	0.02	-0.098	0.023	3.6E-06
4	4	S288C_Ator08_hmg124h_12.JPG.dat	1070.0	26.0	1.0	0.02	0.016	0.023	3.7E-06
YNR019W	YNR019W	S288C_Ator08_hmg124h_12.JPG.dat	1283.8	72.9	1.0	0.06	0.024	0.065	6.5E-05
YNR020C	YNR020C	S288C_Ator08_hmg124h_12.JPG.dat	1250.0	61.4	1.0	0.01	-0.002	0.011	4.1E-05
YNR021W	YNR021W	S288C_Ator08_hmg124h_12.JPG.dat	1220.5	22.0	1.0	0.02	-0.004	0.020	2.4E-06
YNR022C	YNR022C	S288C_Ator08_hmg124h_12.JPG.dat	1217.8	68.7	1.0	0.05	0.010	0.051	3.7E-05
YNR024W	YNR024W	S288C_Ator08_hmg124h_12.JPG.dat	1272.8	35.3	1.0	0.00	0.019	0.003	3.3E-06
YNR025C	YNR025C	S288C_Ator08_hmg124h_12.JPG.dat	1228.8	47.3	1.0	0.04	0.035	0.041	1.9E-05
YNR027W	YNR027W	S288C_Ator08_hmg124h_12.JPG.dat	1249.3	23.1	1.0	0.01	0.017	0.013	5.3E-07
YNR028W	YNR028W	S288C_Ator08_hmg124h_12.JPG.dat	1218.3	20.6	1.0	0.01	-0.007	0.013	6.4E-07
YNR029C	YNR029C	S288C_Ator08_hmg124h_12.JPG.dat	1190.0	34.7	1.0	0.03	0.029	0.033	1.2E-05
YNR030W	YNR030W	S288C_Ator08_hmg124h_12.JPG.dat	1313.3	37.8	1.0	0.03	0.032	0.035	1.1E-05
YNR031C	YNR031C	S288C_Ator08_hmg124h_12.JPG.dat	1250.5	9.5	1.1	0.03	0.009	0.029	5.8E-06
YNR032C-/	YNR032C-A	S288C_Ator08_hmg124h_12.JPG.dat	1212.8	59.7	1.0	0.04	0.019	0.040	1.7E-05
YNR032W	YNR032W	S288C_Ator08_hmg124h_12.JPG.dat	1307.0	16.1	1.1	0.01	0.005	0.014	6.3E-07
YNR034W	YNR034W	S288C_Ator08_hmg124h_12.JPG.dat	1256.3	41.7	1.0	0.03	-0.011	0.029	5.9E-06
YNR039C	YNR039C	S288C_Ator08_hmg124h_12.JPG.dat	1174.0	8.8	1.0	0.00	0.003	0.002	1.1E-06
YNR040W	YNR040W	S288C_Ator08_hmg124h_12.JPG.dat	1185.0	17.6	1.0	0.01	0.004	0.013	5.8E-07
YNR042W	YNR042W	S288C_Ator08_hmg124h_12.JPG.dat	1147.3	23.3	1.0	0.01	0.031	0.007	1.4E-05
YNR045W	YNR045W	S288C_Ator08_hmg124h_12.JPG.dat	1201.3	43.4	1.0	0.04	0.016	0.040	1.7E-05
YNR047W	YNR047W	S288C_Ator08_hmg124h_12.JPG.dat	1218.5	36.6	1.0	0.03	0.047	0.029	6.0E-06
YNR048W	YNR048W	S288C_Ator08_hmg124h_12.JPG.dat	1244.5	40.7	1.0	0.03	-0.014	0.029	5.9E-06
YNR049C	YNR049C	S288C_Ator08_hmg124h_12.JPG.dat	1053.0	329.4	1.0	0.04	0.015	0.036	4.5E-04
YNR050C	YNR050C	S288C_Ator08_hmg124h_12.JPG.dat	0.0	0.0	0.0	0.00	0.000	0.000	
YNR051C	YNR051C	S288C_Ator08_hmg124h_12.JPG.dat	1011.3	67.5	0.8	0.02	-0.056	0.022	2.2E-04
YNR055C	YNR055C	S288C_Ator08_hmg124h_12.JPG.dat	1166.5	72.1	1.0	0.05	-0.013	0.055	4.8E-05
YNR056C	YNR056C	S288C_Ator08_hmg124h_12.JPG.dat	1119.3	26.8	1.0	0.03	-0.027	0.027	6.0E-06
YNR057C	YNR057C	S288C_Ator08_hmg124h_12.JPG.dat	1149.3	18.2	1.0	0.02	-0.004	0.016	1.1E-06
YNR058W	YNR058W	S288C_Ator08_hmg124h_12.JPG.dat	1149.8	42.0	1.0	0.04	-0.019	0.037	1.3E-05
YNR059W	YNR059W	S288C_Ator08_hmg124h_12.JPG.dat	1102.8	41.3	1.0	0.04	0.044	0.037	1.5E-05
YNR060W	YNR060W	S288C_Ator08_hmg124h_12.JPG.dat	1159.3	32.5	1.0	0.03	0.041	0.032	8.3E-06
YNR061C	YNR061C	S288C_Ator08_hmg124h_12.JPG.dat	1183.0	36.4	1.0	0.01	-0.006	0.009	2.7E-05
YNR062C	YNR062C	S288C_Ator08_hmg124h_12.JPG.dat	1200.3	25.2	1.0	0.02	0.028	0.022	2.9E-06
YNR063W	YNR063W	S288C_Ator08_hmg124h_12.JPG.dat	1205.8	14.9	1.0	0.01	-0.005	0.014	6.7E-07
YNR064C	YNR064C	S288C_Ator08_hmg124h_12.JPG.dat	1141.0	27.5	1.0	0.00	-0.034	0.004	1.7E-08
YNR065C	YNR065C	S288C_Ator08_hmg124h_12.JPG.dat	1198.3	5.0	1.0	0.01	0.007	0.012	4.7E-07
YNR066C	YNR066C	S288C_Ator08_hmg124h_12.JPG.dat	1182.5	50.1	1.0	0.04	-0.008	0.036	1.3E-05
YNR067C	YNR067C	S288C_Ator08_hmg124h_12.JPG.dat	1152.3	47.7	1.0	0.04	0.035	0.037	1.3E-05
YNR069C	YNR069C	S288C_Ator08_hmg124h_12.JPG.dat	1082.5	11.8	1.0	0.00	0.005	0.004	4.4E-06
YNR071C	YNR071C	S288C_Ator08_hmg124h_12.JPG.dat	1145.8	44.2	1.0	0.01	0.013	0.006	1.3E-05
YNR072W	YNR072W	S288C_Ator08_hmg124h_12.JPG.dat	1109.8	25.4	1.0	0.03	-0.004	0.025	4.3E-06
YNR073C	YNR073C	S288C_Ator08_hmg124h_12.JPG.dat	1172.8	30.2	1.0	0.03	0.026	0.028	5.3E-06
YNR074C	YNR074C	S288C_Ator08_hmg124h_12.JPG.dat	1175.0	19.9	1.0	0.02	0.046	0.019	1.7E-06
YNR075W	YNR075W	S288C_Ator08_hmg124h_12.JPG.dat	1157.3	31.1	1.0	0.03	-0.007	0.028	6.1E-06
YOL001W	YOL001W	S288C_Ator08_hmg124h_12.JPG.dat	641.3	488.1	0.5	0.42	-0.157	0.417	7.8E-02

YOL002C	YOL002C	S288C_Ator08_hmg124h_12.JPG.dat	1110.3	54.6	1.0	0.05	-0.009	0.045	2.5E-05
YOL003C	YOL003C	S288C_Ator08_hmg124h_12.JPG.dat	1122.5	11.5	1.0	0.02	-0.085	0.019	2.2E-06
YOL004W	YOL004W	S288C_Ator08_hmg124h_12.JPG.dat	0.0	0.0	0.0	0.00	0.000	0.000	
YOL006C	YOL006C	S288C_Ator08_hmg124h_12.JPG.dat	1120.3	28.1	1.0	0.01	0.006	0.005	1.0E-05
YOL007C	YOL007C	S288C_Ator08_hmg124h_12.JPG.dat	1145.0	20.3	1.0	0.02	-0.010	0.019	2.0E-06
YOL008W	YOL008W	S288C_Ator08_hmg124h_12.JPG.dat	1150.3	16.9	1.0	0.00	0.052	0.003	3.9E-06
YOL009C	YOL009C	S288C_Ator08_hmg124h_12.JPG.dat	1131.5	11.3	1.0	0.00	0.004	0.002	1.0E-06
YOL011W	YOL011W	S288C_Ator08_hmg124h_12.JPG.dat	1173.5	30.0	1.0	0.02	0.013	0.025	4.1E-06
YOL012C	YOL012C	S288C_Ator08_hmg124h_12.JPG.dat	745.8	52.3	0.6	0.01	-0.196	0.006	3.0E-05
YOL013C	YOL013C	S288C_Ator08_hmg124h_12.JPG.dat	1214.5	44.0	1.0	0.04	-0.063	0.038	1.4E-05
YOL013W-	YOL013W-A	S288C_Ator08_hmg124h_12.JPG.dat	982.8	296.5	0.8	0.25	-0.088	0.250	7.1E-03
YOL014W	YOL014W	S288C_Ator08_hmg124h_12.JPG.dat	1045.5	116.2	0.9	0.08	-0.053	0.081	1.8E-04
YOL015W	YOL015W	S288C_Ator08_hmg124h_12.JPG.dat	1265.3	45.4	1.1	0.03	0.045	0.026	3.9E-06
YOL016C	YOL016C	S288C_Ator08_hmg124h_12.JPG.dat	1176.8	99.3	1.0	0.09	-0.063	0.086	1.9E-04
YOL017W	YOL017W	S288C_Ator08_hmg124h_12.JPG.dat	1185.5	18.2	1.0	0.02	0.023	0.019	1.9E-06
YOL018C	YOL018C	S288C_Ator08_hmg124h_12.JPG.dat	1178.8	12.3	1.0	0.01	0.014	0.006	5.3E-08
YOL019W	YOL019W	S288C_Ator08_hmg124h_12.JPG.dat	1198.3	25.0	1.0	0.02	0.010	0.018	1.6E-06
YOL020W	YOL020W	S288C_Ator08_hmg124h_12.JPG.dat	1158.3	17.5	1.0	0.01	0.018	0.011	3.8E-07
YOL024W	YOL024W	S288C_Ator08_hmg124h_12.JPG.dat	1142.8	54.4	1.0	0.04	-0.017	0.037	1.5E-05
YOL025W	YOL025W	S288C_Ator08_hmg124h_12.JPG.dat	1175.3	38.1	1.0	0.03	-0.018	0.026	4.7E-06
YOL027C	YOL027C	S288C_Ator08_hmg124h_12.JPG.dat	1192.3	38.2	1.0	0.03	0.015	0.026	5.0E-06
YOL028C	YOL028C	S288C_Ator08_hmg124h_12.JPG.dat	1245.8	36.1	1.0	0.03	-0.013	0.028	5.3E-06
YOL029C	YOL029C	S288C_Ator08_hmg124h_12.JPG.dat	776.8	541.7	0.7	0.49	-0.245	0.486	6.6E-02
YOL030W	YOL030W	S288C_Ator08_hmg124h_12.JPG.dat	1141.3	156.7	0.9	0.13	0.023	0.127	6.8E-04
YOL031C	YOL031C	S288C_Ator08_hmg124h_12.JPG.dat	1243.0	44.4	1.0	0.04	-0.003	0.037	1.4E-05
YOL032W	YOL032W	S288C_Ator08_hmg124h_12.JPG.dat	1240.3	23.1	1.0	0.02	-0.016	0.017	1.4E-06
YOL035C	YOL035C	S288C_Ator08_hmg124h_12.JPG.dat	1282.5	15.6	1.0	0.01	0.013	0.013	5.5E-07
YOL036W	YOL036W	S288C_Ator08_hmg124h_12.JPG.dat	1246.0	29.5	1.0	0.03	-0.016	0.026	4.5E-06
YOL037C	YOL037C	S288C_Ator08_hmg124h_12.JPG.dat	1262.8	38.3	1.1	0.04	0.026	0.035	1.0E-05
YOL039W	YOL039W	S288C_Ator08_hmg124h_12.JPG.dat	1192.3	36.1	1.0	0.03	-0.048	0.032	9.0E-06
YOL041C	YOL041C	S288C_Ator08_hmg124h_12.JPG.dat	1216.3	43.9	1.0	0.03	-0.040	0.035	1.1E-05
YOL042W	YOL042W	S288C_Ator08_hmg124h_12.JPG.dat	1215.8	37.3	1.0	0.03	-0.002	0.031	8.3E-06
YOL043C	YOL043C	S288C_Ator08_hmg124h_12.JPG.dat	1211.3	86.7	1.0	0.07	-0.003	0.069	9.6E-05
YOL044W	YOL044W	S288C_Ator08_hmg124h_12.JPG.dat	1020.3	115.5	0.9	0.10	-0.026	0.097	3.9E-04
YOL045W	YOL045W	S288C_Ator08_hmg124h_12.JPG.dat	1062.5	58.4	0.9	0.04	-0.053	0.037	1.8E-05
YOL046C	YOL046C	S288C_Ator08_hmg124h_12.JPG.dat	1240.8	62.4	1.0	0.05	-0.052	0.047	3.0E-05
YOL047C	YOL047C	S288C_Ator08_hmg124h_12.JPG.dat	1281.3	21.6	1.0	0.02	-0.006	0.016	1.1E-06
YOL048C	YOL048C	S288C_Ator08_hmg124h_12.JPG.dat	1273.0	22.4	1.0	0.02	-0.012	0.022	2.6E-06
YOL049W	YOL049W	S288C_Ator08_hmg124h_12.JPG.dat	908.5	68.0	0.7	0.06	-0.015	0.057	1.2E-04
YOL050C	YOL050C	S288C_Ator08_hmg124h_12.JPG.dat	1364.3	30.4	1.1	0.02	0.132	0.024	2.7E-06
YOL052C	YOL052C	S288C_Ator08_hmg124h_12.JPG.dat	641.0	345.5	0.4	0.03	-0.043	0.034	2.7E-03
YOL053C-A	YOL053C-A	S288C_Ator08_hmg124h_12.JPG.dat	1270.0	27.5	1.0	0.02	0.009	0.018	1.4E-06
YOL053W	YOL053W	S288C_Ator08_hmg124h_12.JPG.dat	1317.8	38.8	1.1	0.03	0.017	0.030	6.2E-06
YOL054W	YOL054W	S288C_Ator08_hmg124h_12.JPG.dat	1244.5	29.1	1.0	0.03	-0.006	0.029	6.6E-06
YOL055C	YOL055C	S288C_Ator08_hmg124h_12.JPG.dat	1251.0	44.5	1.0	0.04	-0.015	0.043	2.1E-05
YOL056W	YOL056W	S288C_Ator08_hmg124h_12.JPG.dat	1108.5	30.8	1.0	0.02	-0.033	0.025	4.5E-06
YOL057W	YOL057W	S288C_Ator08_hmg124h_12.JPG.dat	1308.3	12.8	1.1	0.01	0.022	0.014	6.9E-07
YOL058W	YOL058W	S288C_Ator08_hmg124h_12.JPG.dat	0.0	0.0	0.0	0.00	0.000	0.000	
YOL059W	YOL059W	S288C_Ator08_hmg124h_12.JPG.dat	1197.5	15.6	1.0	0.00	0.028	0.003	2.5E-06
YOL060C	YOL060C	S288C_Ator08_hmg124h_12.JPG.dat	1072.5	38.7	0.9	0.03	-0.046	0.034	1.4E-05
YOL061W	YOL061W	S288C_Ator08_hmg124h_12.JPG.dat	1151.8	18.0	1.0	0.02	-0.002	0.017	1.4E-06
YOL062C	YOL062C	S288C_Ator08_hmg124h_12.JPG.dat	1138.0	15.8	1.0	0.01	-0.012	0.010	3.4E-07
YOL063C	YOL063C	S288C_Ator08_hmg124h_12.JPG.dat	1112.5	127.3	0.9	0.11	-0.020	0.107	3.9E-04
YOL064C	YOL064C	S288C_Ator08_hmg124h_12.JPG.dat	1052.8	25.3	0.9	0.02	0.000	0.019	2.7E-06
YOL065C	YOL065C	S288C_Ator08_hmg124h_12.JPG.dat	1247.3	53.5	1.0	0.05	0.016	0.050	3.3E-05
YOL067C	YOL067C	S288C_Ator08_hmg124h_12.JPG.dat	1225.3	46.9	1.0	0.05	0.017	0.046	2.6E-05
YOL068C	YOL068C	S288C_Ator08_hmg124h_12.JPG.dat	1102.3	81.7	1.0	0.06	-0.020	0.060	6.0E-05
YOL070C	YOL070C	S288C_Ator08_hmg124h_12.JPG.dat	1252.0	36.1	1.0	0.04	-0.018	0.036	1.1E-05
YOL071W	YOL071W	S288C_Ator08_hmg124h_12.JPG.dat	1122.8	26.4	1.0	0.03	0.002	0.027	6.0E-06
YOL075C	YOL075C	S288C_Ator08_hmg124h_12.JPG.dat	1160.5	44.0	1.0	0.04	0.022	0.038	1.4E-05
YOL079W	YOL079W	S288C_Ator08_hmg124h_12.JPG.dat	1128.5	9.9	1.0	0.01	0.008	0.009	2.1E-07
YOL080C	YOL080C	S288C_Ator08_hmg124h_12.JPG.dat	1118.0	16.1	1.0	0.01	0.010	0.013	6.1E-07
YOL081W	YOL081W	S288C_Ator08_hmg124h_12.JPG.dat	1082.8	38.2	1.0	0.03	0.013	0.032	9.7E-06
YOL082W	YOL082W	S288C_Ator08_hmg124h_12.JPG.dat	989.8	47.1	0.9	0.04	-0.119	0.042	3.2E-05
YOL083W	YOL083W	S288C_Ator08_hmg124h_12.JPG.dat	1190.5	49.3	1.0	0.04	-0.003	0.039	1.5E-05
YOL084W	YOL084W	S288C_Ator08_hmg124h_12.JPG.dat	1188.3	40.1	1.0	0.03	0.015	0.032	8.9E-06
YOL085C	YOL085C	S288C_Ator08_hmg124h_12.JPG.dat	1207.8	5.7	1.0	0.00	0.013	0.003	6.3E-09
YOL087C	YOL087C	S288C_Ator08_hmg124h_12.JPG.dat	1090.3	46.8	1.0	0.01	0.001	0.010	3.6E-05
YOL088C	YOL088C	S288C_Ator08_hmg124h_12.JPG.dat	1217.3	40.9	1.0	0.01	0.011	0.012	4.5E-05
YOL089C	YOL089C	S288C_Ator08_hmg124h_12.JPG.dat	1165.3	63.5	1.0	0.05	0.030	0.050	3.2E-05

YOL090W	YOL090W	S288C_Ator08_hmg124h_12.JPG.dat	1006.8	30.2	0.9	0.00	-0.024	0.002	2.3E-06
YOL091W	YOL091W	S288C_Ator08_hmg124h_12.JPG.dat	1092.5	32.2	1.0	0.02	0.003	0.025	4.4E-06
YOL092W	YOL092W	S288C_Ator08_hmg124h_12.JPG.dat	1160.8	35.1	1.0	0.03	-0.020	0.029	6.2E-06
YOL093W	YOL093W	S288C_Ator08_hmg124h_12.JPG.dat	1085.5	45.6	1.0	0.02	0.011	0.015	7.9E-05
YOL095C	YOL095C	S288C_Ator08_hmg124h_12.JPG.dat	1136.8	49.4	1.0	0.04	0.023	0.043	2.2E-05
YOL098C	YOL098C	S288C_Ator08_hmg124h_12.JPG.dat	1161.8	39.8	1.0	0.03	-0.002	0.034	1.1E-05
YOL099C	YOL099C	S288C_Ator08_hmg124h_12.JPG.dat	1131.0	34.5	1.0	0.01	-0.057	0.005	1.0E-05
YOL101C	YOL101C	S288C_Ator08_hmg124h_12.JPG.dat	1243.8	63.0	1.1	0.05	0.025	0.052	3.1E-05
YOL103W	YOL103W	S288C_Ator08_hmg124h_12.JPG.dat	1142.3	84.0	1.0	0.02	0.005	0.021	1.4E-04
YOL104C	YOL104C	S288C_Ator08_hmg124h_12.JPG.dat	1256.0	28.0	1.0	0.02	-0.013	0.023	3.2E-06
YOL105C	YOL105C	S288C_Ator08_hmg124h_12.JPG.dat	1195.3	25.7	1.0	0.03	0.004	0.026	4.8E-06
YOL106W	YOL106W	S288C_Ator08_hmg124h_12.JPG.dat	1147.5	31.4	1.0	0.01	0.051	0.008	2.3E-05
YOL107W	YOL107W	S288C_Ator08_hmg124h_12.JPG.dat	856.5	571.4	1.0	0.02	-0.005	0.019	1.1E-04
YOL108C	YOL108C	S288C_Ator08_hmg124h_12.JPG.dat	1126.3	36.1	1.0	0.03	0.011	0.030	7.7E-06
YOL109W	YOL109W	S288C_Ator08_hmg124h_12.JPG.dat	1118.8	22.6	1.0	0.02	0.010	0.017	1.4E-06
YOL110W	YOL110W	S288C_Ator08_hmg124h_12.JPG.dat	1206.8	30.0	1.0	0.02	0.036	0.023	3.2E-06
YOL111C	YOL111C	S288C_Ator08_hmg124h_12.JPG.dat	1195.8	49.3	1.0	0.05	-0.004	0.047	2.7E-05
YOL112W	YOL112W	S288C_Ator08_hmg124h_12.JPG.dat	1060.5	366.7	1.0	0.05	0.028	0.049	7.2E-04
YOL113W	YOL113W	S288C_Ator08_hmg124h_12.JPG.dat	1179.8	45.0	1.0	0.04	0.010	0.042	2.0E-05
YOL114C	YOL114C	S288C_Ator08_hmg124h_12.JPG.dat	1147.8	61.1	1.1	0.01	0.006	0.013	5.1E-05
YOL115W	YOL115W	S288C_Ator08_hmg124h_12.JPG.dat	0.0	0.0	0.0	0.00	0.000	0.000	
YOL116W	YOL116W	S288C_Ator08_hmg124h_12.JPG.dat	1195.8	21.2	1.0	0.00	-0.018	0.003	2.3E-06
YOL117W	YOL117W	S288C_Ator08_hmg124h_12.JPG.dat	1176.5	34.6	1.0	0.02	0.007	0.024	3.6E-06
YOL118C	YOL118C	S288C_Ator08_hmg124h_12.JPG.dat	1185.5	30.1	1.0	0.03	0.026	0.026	4.6E-06
YOL119C	YOL119C	S288C_Ator08_hmg124h_12.JPG.dat	1156.8	39.8	1.0	0.03	-0.002	0.034	1.1E-05
YOL121C	YOL121C	S288C_Ator08_hmg124h_12.JPG.dat	1142.5	45.8	1.0	0.03	0.010	0.031	8.2E-06
YOL122C	YOL122C	S288C_Ator08_hmg124h_12.JPG.dat	1132.0	74.9	1.0	0.02	-0.004	0.017	9.5E-05
YOL124C	YOL124C	S288C_Ator08_hmg124h_12.JPG.dat	1161.0	41.7	1.0	0.03	-0.007	0.033	1.1E-05
YOL126C	YOL126C	S288C_Ator08_hmg124h_12.JPG.dat	1198.0	43.1	1.0	0.01	-0.034	0.010	3.7E-05
YOL128C	YOL128C	S288C_Ator08_hmg124h_12.JPG.dat	1055.3	439.4	1.1	0.02	0.017	0.023	1.5E-04
YOL129W	YOL129W	S288C_Ator08_hmg124h_12.JPG.dat	1141.5	27.1	1.0	0.01	0.013	0.012	5.3E-05
YOL131W	YOL131W	S288C_Ator08_hmg124h_12.JPG.dat	1252.8	33.0	1.0	0.01	0.033	0.005	9.6E-06
YOL132W	YOL132W	S288C_Ator08_hmg124h_12.JPG.dat	1253.5	44.9	1.0	0.04	0.019	0.038	1.4E-05
YOL136C	YOL136C	S288C_Ator08_hmg124h_12.JPG.dat	1179.0	191.5	1.0	0.02	0.029	0.023	1.6E-04
YOL137W	YOL137W	S288C_Ator08_hmg124h_12.JPG.dat	1262.8	20.5	1.0	0.02	0.025	0.019	1.6E-06
YOL138C	YOL138C	S288C_Ator08_hmg124h_12.JPG.dat	1238.3	20.4	1.0	0.02	-0.020	0.016	1.1E-06
YOL141W	YOL141W	S288C_Ator08_hmg124h_12.JPG.dat	1221.5	63.0	1.0	0.02	-0.036	0.018	1.0E-04
YOL147C	YOL147C	S288C_Ator08_hmg124h_12.JPG.dat	1246.8	58.1	1.0	0.05	-0.001	0.048	2.7E-05
YOL150C	YOL150C	S288C_Ator08_hmg124h_12.JPG.dat	1181.5	43.4	1.0	0.01	0.001	0.006	1.2E-05
YOL151W	YOL151W	S288C_Ator08_hmg124h_12.JPG.dat	1308.0	139.8	1.1	0.12	0.086	0.117	3.6E-04
YOL152W	YOL152W	S288C_Ator08_hmg124h_12.JPG.dat	1009.0	278.2	0.9	0.08	0.047	0.085	2.8E-03
YOL155C	YOL155C	S288C_Ator08_hmg124h_12.JPG.dat	1131.8	63.4	0.9	0.06	-0.086	0.061	8.6E-05
YOL158C	YOL158C	S288C_Ator08_hmg124h_12.JPG.dat	1325.5	15.4	1.0	0.02	0.038	0.023	3.0E-06
YOL159C	YOL159C	S288C_Ator08_hmg124h_12.JPG.dat	1259.3	26.6	1.0	0.02	-0.019	0.018	1.7E-06
YOL160W	YOL160W	S288C_Ator08_hmg124h_12.JPG.dat	1243.3	197.6	1.1	0.02	0.071	0.016	6.9E-05
YOL162W	YOL162W	S288C_Ator08_hmg124h_12.JPG.dat	1327.5	33.1	1.0	0.01	0.064	0.011	3.0E-07
YOL163W	YOL163W	S288C_Ator08_hmg124h_12.JPG.dat	1302.5	75.5	1.0	0.04	0.024	0.043	2.0E-05
YOR001W	YOR001W	S288C_Ator08_hmg124h_12.JPG.dat	1132.5	47.3	0.9	0.01	-0.001	0.009	3.3E-05
YOR002W	YOR002W	S288C_Ator08_hmg124h_12.JPG.dat	1300.0	13.3	1.0	0.02	-0.012	0.021	2.5E-06
YOR003W	YOR003W	S288C_Ator08_hmg124h_12.JPG.dat	1311.0	21.8	1.0	0.00	0.005	0.004	5.7E-06
YOR005C	YOR005C	S288C_Ator08_hmg124h_12.JPG.dat	1275.8	52.7	1.0	0.01	-0.046	0.006	1.4E-05
YOR006C	YOR006C	S288C_Ator08_hmg124h_12.JPG.dat	641.8	365.6	0.4	0.07	-0.141	0.074	1.2E-02
YOR007C	YOR007C	S288C_Ator08_hmg124h_12.JPG.dat	1244.8	31.3	1.1	0.01	0.020	0.007	1.4E-05
YOR008C	YOR008C	S288C_Ator08_hmg124h_12.JPG.dat	797.8	29.1	0.7	0.01	-0.296	0.009	7.0E-05
YOR008C-/	YOR008C-A	S288C_Ator08_hmg124h_12.JPG.dat	1251.3	47.2	1.0	0.03	-0.018	0.025	4.2E-06
YOR009W	YOR009W	S288C_Ator08_hmg124h_12.JPG.dat	1200.5	65.3	1.0	0.05	-0.045	0.048	2.9E-05
YOR010C	YOR010C	S288C_Ator08_hmg124h_12.JPG.dat	1063.3	203.9	1.0	0.04	0.011	0.037	4.8E-04
YOR011W	YOR011W	S288C_Ator08_hmg124h_12.JPG.dat	1213.0	28.2	1.0	0.02	0.012	0.018	1.6E-06
YOR012W	YOR012W	S288C_Ator08_hmg124h_12.JPG.dat	1230.3	31.1	1.0	0.04	0.023	0.041	1.7E-05
YOR013W	YOR013W	S288C_Ator08_hmg124h_12.JPG.dat	1171.3	16.9	1.0	0.00	0.009	0.004	2.0E-08
YOR014W	YOR014W	S288C_Ator08_hmg124h_12.JPG.dat	1055.5	135.7	0.9	0.02	0.022	0.020	1.5E-04
YOR015W	YOR015W	S288C_Ator08_hmg124h_12.JPG.dat	1027.0	328.3	1.0	0.03	0.005	0.034	4.0E-04
YOR016C	YOR016C	S288C_Ator08_hmg124h_12.JPG.dat	1264.0	53.4	1.1	0.03	0.033	0.034	8.0E-06
YOR017W	YOR017W	S288C_Ator08_hmg124h_12.JPG.dat	1212.3	27.9	1.0	0.01	0.004	0.011	3.7E-05
YOR018W	YOR018W	S288C_Ator08_hmg124h_12.JPG.dat	1290.8	47.6	1.1	0.04	-0.028	0.039	1.3E-05
YOR019W	YOR019W	S288C_Ator08_hmg124h_12.JPG.dat	1225.3	55.0	1.0	0.04	0.032	0.038	1.3E-05
YOR021C	YOR021C	S288C_Ator08_hmg124h_12.JPG.dat	864.0	165.5	0.8	0.14	-0.172	0.138	1.7E-03
YOR022C	YOR022C	S288C_Ator08_hmg124h_12.JPG.dat	1178.5	46.9	1.0	0.04	0.036	0.041	1.8E-05
YOR023C	YOR023C	S288C_Ator08_hmg124h_12.JPG.dat	1127.0	13.0	1.0	0.01	-0.043	0.013	6.5E-07
YOR024W	YOR024W	S288C_Ator08_hmg124h_12.JPG.dat	911.5	147.7	0.7	0.03	-0.156	0.030	5.4E-04

YOR025W	YOR025W	S288C_Ator08_hmg124h_12.JPG.dat	1005.8	271.8	0.9	0.23	-0.063	0.232	5.0E-03
YOR026W	YOR026W	S288C_Ator08_hmg124h_12.JPG.dat	953.8	142.5	0.8	0.12	0.078	0.118	8.7E-04
YOR027W	YOR027W	S288C_Ator08_hmg124h_12.JPG.dat	1228.3	46.7	1.0	0.01	0.022	0.009	2.5E-05
YOR028C	YOR028C	S288C_Ator08_hmg124h_12.JPG.dat	1169.5	31.4	1.0	0.02	-0.010	0.019	1.7E-06
YOR029W	YOR029W	S288C_Ator08_hmg124h_12.JPG.dat	1155.3	35.2	1.0	0.01	-0.047	0.007	1.8E-05
YOR030W	YOR030W	S288C_Ator08_hmg124h_12.JPG.dat	864.8	31.0	0.8	0.01	-0.199	0.009	4.4E-05
YOR031W	YOR031W	S288C_Ator08_hmg124h_12.JPG.dat	1182.8	59.3	1.1	0.05	0.027	0.051	3.2E-05
YOR032C	YOR032C	S288C_Ator08_hmg124h_12.JPG.dat	1008.5	190.3	1.0	0.03	0.033	0.032	3.4E-04
YOR033C	YOR033C	S288C_Ator08_hmg124h_12.JPG.dat	287.5	575.0	0.0	0.00	0.000	0.000	
YOR034C	YOR034C	S288C_Ator08_hmg124h_12.JPG.dat	1065.3	23.8	1.0	0.02	-0.038	0.019	2.2E-06
YOR035C	YOR035C	S288C_Ator08_hmg124h_12.JPG.dat	420.0	37.7	0.4	0.03	-0.397	0.033	1.9E-04
YOR037W	YOR037W	S288C_Ator08_hmg124h_12.JPG.dat	1094.5	22.6	1.0	0.02	0.039	0.019	2.3E-06
YOR038C	YOR038C	S288C_Ator08_hmg124h_12.JPG.dat	469.0	241.2	0.4	0.21	-0.236	0.207	3.0E-02
YOR039W	YOR039W	S288C_Ator08_hmg124h_12.JPG.dat	987.0	34.1	0.8	0.03	-0.012	0.030	1.2E-05
YOR040W	YOR040W	S288C_Ator08_hmg124h_12.JPG.dat	1132.0	46.8	1.0	0.02	-0.051	0.018	1.6E-06
YOR041C	YOR041C	S288C_Ator08_hmg124h_12.JPG.dat	1137.8	47.9	1.0	0.05	-0.025	0.047	3.2E-05
YOR042W	YOR042W	S288C_Ator08_hmg124h_12.JPG.dat	1221.8	41.5	1.0	0.01	-0.036	0.008	2.0E-05
YOR043W	YOR043W	S288C_Ator08_hmg124h_12.JPG.dat	855.3	144.1	0.7	0.04	-0.200	0.041	1.2E-03
YOR044W	YOR044W	S288C_Ator08_hmg124h_12.JPG.dat	1125.0	23.2	1.0	0.00	-0.024	0.004	4.3E-06
YOR045W	YOR045W	S288C_Ator08_hmg124h_12.JPG.dat	1107.5	23.7	1.0	0.02	0.003	0.022	3.3E-06
YOR047C	YOR047C	S288C_Ator08_hmg124h_12.JPG.dat	1138.3	22.8	1.0	0.00	0.025	0.005	7.5E-06
YOR049C	YOR049C	S288C_Ator08_hmg124h_12.JPG.dat	1149.0	17.8	1.0	0.00	-0.011	0.003	3.4E-06
YOR050C	YOR050C	S288C_Ator08_hmg124h_12.JPG.dat	1185.0	32.9	1.0	0.03	-0.009	0.029	6.3E-06
YOR051C	YOR051C	S288C_Ator08_hmg124h_12.JPG.dat	1258.8	40.6	1.1	0.03	0.057	0.034	9.3E-06
YOR052C	YOR052C	S288C_Ator08_hmg124h_12.JPG.dat	1257.0	41.3	1.1	0.03	0.014	0.034	8.7E-06
YOR053W	YOR053W	S288C_Ator08_hmg124h_12.JPG.dat	1137.3	107.7	1.0	0.03	-0.030	0.027	2.5E-04
YOR055W	YOR055W	S288C_Ator08_hmg124h_12.JPG.dat	1202.3	40.3	1.0	0.03	0.038	0.026	4.4E-06
YOR058C	YOR058C	S288C_Ator08_hmg124h_12.JPG.dat	1023.3	276.7	0.9	0.23	-0.124	0.233	5.1E-03
YOR059C	YOR059C	S288C_Ator08_hmg124h_12.JPG.dat	1121.3	15.8	1.0	0.01	-0.023	0.013	5.9E-07
YOR061W	YOR061W	S288C_Ator08_hmg124h_12.JPG.dat	799.3	14.4	0.7	0.01	-0.179	0.014	2.0E-06
YOR062C	YOR062C	S288C_Ator08_hmg124h_12.JPG.dat	964.8	194.1	0.9	0.04	-0.058	0.039	5.8E-04
YOR064C	YOR064C	S288C_Ator08_hmg124h_12.JPG.dat	1046.5	50.1	0.9	0.04	-0.048	0.040	2.2E-05
YOR065W	YOR065W	S288C_Ator08_hmg124h_12.JPG.dat	890.3	595.5	1.0	0.05	-0.008	0.052	8.4E-04
YOR066W	YOR066W	S288C_Ator08_hmg124h_12.JPG.dat	1155.5	49.4	1.0	0.05	-0.036	0.046	2.6E-05
YOR067C	YOR067C	S288C_Ator08_hmg124h_12.JPG.dat	1211.0	14.2	1.0	0.02	0.003	0.016	9.7E-07
YOR068C	YOR068C	S288C_Ator08_hmg124h_12.JPG.dat	829.0	555.2	0.9	0.05	-0.025	0.053	1.0E-03
YOR069W	YOR069W	S288C_Ator08_hmg124h_12.JPG.dat	1008.8	141.3	0.9	0.11	-0.057	0.107	4.4E-04
YOR070C	YOR070C	S288C_Ator08_hmg124h_12.JPG.dat	1011.8	37.1	0.9	0.01	-0.108	0.011	5.8E-05
YOR071C	YOR071C	S288C_Ator08_hmg124h_12.JPG.dat	1219.0	33.6	1.0	0.01	-0.001	0.007	1.5E-05
YOR072W	YOR072W	S288C_Ator08_hmg124h_12.JPG.dat	1241.5	87.2	1.0	0.07	-0.011	0.073	1.0E-04
YOR076C	YOR076C	S288C_Ator08_hmg124h_12.JPG.dat	1217.5	14.6	1.0	0.01	0.038	0.006	5.6E-08
YOR078W	YOR078W	S288C_Ator08_hmg124h_12.JPG.dat	778.8	553.8	0.6	0.46	-0.107	0.462	6.8E-02
YOR079C	YOR079C	S288C_Ator08_hmg124h_12.JPG.dat	1201.3	28.3	1.0	0.01	0.000	0.005	8.6E-06
YOR080W	YOR080W	S288C_Ator08_hmg124h_12.JPG.dat	1145.5	86.7	0.9	0.01	-0.078	0.013	6.5E-05
YOR081C	YOR081C	S288C_Ator08_hmg124h_12.JPG.dat	1202.0	44.0	1.0	0.03	-0.005	0.030	7.2E-06
YOR082C	YOR082C	S288C_Ator08_hmg124h_12.JPG.dat	1193.5	38.5	1.0	0.00	-0.020	0.004	4.7E-06
YOR083W	YOR083W	S288C_Ator08_hmg124h_12.JPG.dat	645.5	745.4	0.5	0.62	0.028	0.621	1.8E-01
YOR084W	YOR084W	S288C_Ator08_hmg124h_12.JPG.dat	1132.0	106.2	1.0	0.07	0.080	0.069	9.7E-05
YOR085W	YOR085W	S288C_Ator08_hmg124h_12.JPG.dat	1064.5	50.4	0.9	0.05	0.047	0.052	5.7E-05
YOR086C	YOR086C	S288C_Ator08_hmg124h_12.JPG.dat	1246.8	71.7	1.0	0.07	-0.032	0.067	9.3E-05
YOR087W	YOR087W	S288C_Ator08_hmg124h_12.JPG.dat	1320.0	57.6	1.1	0.02	0.062	0.015	7.0E-05
YOR088W	YOR088W	S288C_Ator08_hmg124h_12.JPG.dat	1299.5	34.5	1.0	0.02	-0.003	0.019	1.8E-06
YOR089C	YOR089C	S288C_Ator08_hmg124h_12.JPG.dat	1069.8	45.6	0.8	0.00	-0.127	0.001	4.4E-07
YOR090C	YOR090C	S288C_Ator08_hmg124h_12.JPG.dat	1337.5	54.7	1.1	0.03	0.021	0.025	3.8E-06
YOR091W	YOR091W	S288C_Ator08_hmg124h_12.JPG.dat	1347.8	29.3	1.1	0.02	0.030	0.019	1.7E-06
YOR092W	YOR092W	S288C_Ator08_hmg124h_12.JPG.dat	1320.0	57.0	1.0	0.03	0.001	0.032	8.0E-06
YOR093C	YOR093C	S288C_Ator08_hmg124h_12.JPG.dat	1225.3	43.9	1.0	0.03	-0.031	0.029	7.6E-06
YOR094W	YOR094W	S288C_Ator08_hmg124h_12.JPG.dat	1294.0	38.1	1.0	0.02	0.004	0.019	1.9E-06
YOR097C	YOR097C	S288C_Ator08_hmg124h_12.JPG.dat	1247.5	60.9	1.0	0.04	0.008	0.037	1.5E-05
YOR099W	YOR099W	S288C_Ator08_hmg124h_12.JPG.dat	1115.3	35.6	1.0	0.01	-0.004	0.008	2.2E-05
YOR100C	YOR100C	S288C_Ator08_hmg124h_12.JPG.dat	1305.0	19.2	1.0	0.01	0.040	0.011	3.1E-07
YOR101W	YOR101W	S288C_Ator08_hmg124h_12.JPG.dat	1317.3	45.3	1.1	0.04	0.040	0.036	1.1E-05
YOR104W	YOR104W	S288C_Ator08_hmg124h_12.JPG.dat	1196.3	49.2	1.0	0.03	0.004	0.028	6.4E-06
YOR105W	YOR105W	S288C_Ator08_hmg124h_12.JPG.dat	1114.8	21.0	1.0	0.03	-0.037	0.026	5.4E-06
YOR106W	YOR106W	S288C_Ator08_hmg124h_12.JPG.dat	804.3	47.4	0.7	0.04	-0.096	0.040	5.5E-05
YOR107W	YOR107W	S288C_Ator08_hmg124h_12.JPG.dat	1201.0	49.2	1.0	0.01	0.006	0.009	2.3E-05
YOR108W	YOR108W	S288C_Ator08_hmg124h_12.JPG.dat	1215.5	30.0	1.0	0.04	0.011	0.037	1.3E-05
YOR109W	YOR109W	S288C_Ator08_hmg124h_12.JPG.dat	1018.0	20.4	0.8	0.01	-0.177	0.010	4.2E-07
YOR111W	YOR111W	S288C_Ator08_hmg124h_12.JPG.dat	1155.3	85.0	0.9	0.07	0.051	0.069	1.1E-04
YOR112W	YOR112W	S288C_Ator08_hmg124h_12.JPG.dat	1224.8	9.2	1.0	0.02	-0.022	0.019	1.9E-06

YOR113W	YOR113W	S288C_Ator08_hmg124h_12.JPG.dat	1105.3	37.6	1.0	0.03	-0.042	0.033	1.0E-05
YOR114W	YOR114W	S288C_Ator08_hmg124h_12.JPG.dat	1219.3	38.4	1.0	0.02	-0.028	0.019	1.8E-06
YOR115C	YOR115C	S288C_Ator08_hmg124h_12.JPG.dat	1029.5	27.6	0.9	0.02	-0.142	0.024	5.6E-06
YOR118W	YOR118W	S288C_Ator08_hmg124h_12.JPG.dat	1108.3	82.3	1.0	0.06	-0.020	0.060	6.6E-05
YOR120W	YOR120W	S288C_Ator08_hmg124h_12.JPG.dat	1170.3	53.2	1.0	0.01	-0.032	0.012	4.5E-05
YOR121C	YOR121C	S288C_Ator08_hmg124h_12.JPG.dat	1102.5	38.0	1.0	0.03	-0.040	0.026	5.7E-06
YOR123C	YOR123C	S288C_Ator08_hmg124h_12.JPG.dat	969.5	38.7	0.9	0.03	-0.034	0.025	7.3E-06
YOR124C	YOR124C	S288C_Ator08_hmg124h_12.JPG.dat	1165.5	30.6	1.0	0.02	0.031	0.023	3.4E-06
YOR126C	YOR126C	S288C_Ator08_hmg124h_12.JPG.dat	1242.8	16.4	1.1	0.01	0.015	0.008	1.2E-07
YOR127W	YOR127W	S288C_Ator08_hmg124h_12.JPG.dat	1274.8	42.8	1.1	0.03	0.008	0.034	9.1E-06
YOR129C	YOR129C	S288C_Ator08_hmg124h_12.JPG.dat	1164.5	43.5	1.0	0.01	0.012	0.011	4.5E-05
YOR131C	YOR131C	S288C_Ator08_hmg124h_12.JPG.dat	1172.5	74.9	1.1	0.05	0.039	0.054	3.7E-05
YOR132W	YOR132W	S288C_Ator08_hmg124h_12.JPG.dat	1062.5	95.3	0.9	0.08	-0.071	0.084	2.2E-04
YOR133W	YOR133W	S288C_Ator08_hmg124h_12.JPG.dat	1120.3	40.0	1.0	0.04	-0.001	0.035	1.3E-05
YOR134W	YOR134W	S288C_Ator08_hmg124h_12.JPG.dat	1104.0	24.1	1.0	0.02	-0.019	0.019	2.0E-06
YOR135C	YOR135C	S288C_Ator08_hmg124h_12.JPG.dat	1081.5	21.5	1.0	0.02	-0.013	0.018	1.7E-06
YOR136W	YOR136W	S288C_Ator08_hmg124h_12.JPG.dat	1148.3	49.3	1.0	0.04	-0.032	0.043	2.0E-05
YOR137C	YOR137C	S288C_Ator08_hmg124h_12.JPG.dat	1141.0	40.3	1.0	0.04	0.018	0.037	1.3E-05
YOR138C	YOR138C	S288C_Ator08_hmg124h_12.JPG.dat	1198.8	32.2	1.1	0.03	0.038	0.032	7.6E-06
YOR139C	YOR139C	S288C_Ator08_hmg124h_12.JPG.dat	1196.0	38.3	1.1	0.00	0.025	0.004	4.8E-06
YOR140W	YOR140W	S288C_Ator08_hmg124h_12.JPG.dat	1085.8	120.8	0.9	0.10	-0.038	0.105	3.9E-04
YOR141C	YOR141C	S288C_Ator08_hmg124h_12.JPG.dat	415.5	533.6	0.4	0.46	0.018	0.461	2.2E-01
YOR142W	YOR142W	S288C_Ator08_hmg124h_12.JPG.dat	1192.5	65.2	1.1	0.06	0.059	0.057	4.3E-05
YOR144C	YOR144C	S288C_Ator08_hmg124h_12.JPG.dat	1255.8	16.4	1.0	0.02	0.028	0.019	1.8E-06
YOR152C	YOR152C	S288C_Ator08_hmg124h_12.JPG.dat	1177.0	27.2	1.0	0.02	-0.006	0.021	2.3E-06
YOR153W	YOR153W	S288C_Ator08_hmg124h_12.JPG.dat	1165.3	60.5	1.0	0.05	0.028	0.051	3.3E-05
YOR154W	YOR154W	S288C_Ator08_hmg124h_12.JPG.dat	1117.5	16.5	1.0	0.00	0.026	0.003	3.7E-06
YOR156C	YOR156C	S288C_Ator08_hmg124h_12.JPG.dat	1113.0	29.0	1.0	0.03	-0.013	0.026	5.3E-06
YOR161C	YOR161C	S288C_Ator08_hmg124h_12.JPG.dat	1128.0	15.3	1.0	0.02	-0.011	0.017	1.5E-06
YOR162C	YOR162C	S288C_Ator08_hmg124h_12.JPG.dat	1148.5	47.0	1.0	0.04	-0.018	0.041	2.0E-05
YOR163W	YOR163W	S288C_Ator08_hmg124h_12.JPG.dat	1176.8	35.5	1.0	0.03	-0.008	0.031	8.1E-06
YOR164C	YOR164C	S288C_Ator08_hmg124h_12.JPG.dat	1217.3	83.5	1.0	0.07	-0.001	0.070	8.4E-05
YOR165W	YOR165W	S288C_Ator08_hmg124h_12.JPG.dat	1099.3	51.2	0.9	0.01	-0.097	0.015	8.5E-05
YOR166C	YOR166C	S288C_Ator08_hmg124h_12.JPG.dat	1234.5	23.6	1.1	0.03	0.051	0.034	8.0E-06
YOR167C	YOR167C	S288C_Ator08_hmg124h_12.JPG.dat	1203.5	18.5	1.0	0.02	0.034	0.022	3.1E-06
YOR170W	YOR170W	S288C_Ator08_hmg124h_12.JPG.dat	1138.0	31.8	1.0	0.02	-0.029	0.024	3.9E-06
YOR171C	YOR171C	S288C_Ator08_hmg124h_12.JPG.dat	1026.8	35.1	0.9	0.03	-0.126	0.029	8.8E-06
YOR172W	YOR172W	S288C_Ator08_hmg124h_12.JPG.dat	1103.5	33.0	1.0	0.01	-0.023	0.008	2.1E-05
YOR173W	YOR173W	S288C_Ator08_hmg124h_12.JPG.dat	1112.5	29.8	1.0	0.03	-0.015	0.031	8.6E-06
YOR175C	YOR175C	S288C_Ator08_hmg124h_12.JPG.dat	1201.3	46.5	1.1	0.04	0.026	0.042	1.7E-05
YOR177C	YOR177C	S288C_Ator08_hmg124h_12.JPG.dat	1205.8	21.0	1.0	0.02	0.038	0.019	1.5E-06
YOR178C	YOR178C	S288C_Ator08_hmg124h_12.JPG.dat	1195.0	33.5	1.0	0.03	0.056	0.032	8.3E-06
YOR182C	YOR182C	S288C_Ator08_hmg124h_12.JPG.dat	1084.0	105.1	1.0	0.02	0.038	0.021	1.5E-04
YOR183W	YOR183W	S288C_Ator08_hmg124h_12.JPG.dat	1203.5	49.2	1.0	0.04	0.075	0.039	1.5E-05
YOR184W	YOR184W	S288C_Ator08_hmg124h_12.JPG.dat	1212.5	28.0	1.1	0.03	0.028	0.030	6.3E-06
YOR185C	YOR185C	S288C_Ator08_hmg124h_12.JPG.dat	1258.8	50.3	1.0	0.01	-0.028	0.013	5.3E-05
YOR186W	YOR186W	S288C_Ator08_hmg124h_12.JPG.dat	1194.3	16.7	1.0	0.02	0.004	0.016	1.2E-06
YOR188W	YOR188W	S288C_Ator08_hmg124h_12.JPG.dat	1208.3	40.3	1.0	0.02	-0.011	0.024	3.9E-06
YOR189W	YOR189W	S288C_Ator08_hmg124h_12.JPG.dat	1322.5	25.1	1.1	0.01	0.098	0.014	5.4E-07
YOR190W	YOR190W	S288C_Ator08_hmg124h_12.JPG.dat	1181.8	26.1	1.0	0.02	0.008	0.019	2.1E-06
YOR191W	YOR191W	S288C_Ator08_hmg124h_12.JPG.dat	1126.0	65.9	1.0	0.05	-0.024	0.050	3.8E-05
YOR192C	YOR192C	S288C_Ator08_hmg124h_12.JPG.dat	1112.8	57.5	0.9	0.04	-0.050	0.043	2.6E-05
YOR193W	YOR193W	S288C_Ator08_hmg124h_12.JPG.dat	1250.8	32.6	1.0	0.03	0.018	0.028	5.2E-06
YOR195W	YOR195W	S288C_Ator08_hmg124h_12.JPG.dat	1223.3	46.9	1.0	0.03	-0.022	0.033	9.8E-06
YOR196C	YOR196C	S288C_Ator08_hmg124h_12.JPG.dat	1084.0	157.1	0.9	0.14	0.051	0.138	9.6E-04
YOR202W	YOR202W	S288C_Ator08_hmg124h_12.JPG.dat	1201.6	132.6	1.0	0.07	0.003	0.072	4.00786447
1	1	S288C_Ator08_hmg124h_2.JPG.dat	1164.3	128.0	1.1	0.10	0.063	0.095	1.9E-04
2	2	S288C_Ator08_hmg124h_2.JPG.dat	981.0	65.7	1.0	0.08	-0.035	0.080	1.6E-04
3	3	S288C_Ator08_hmg124h_2.JPG.dat	1017.0	77.9	1.0	0.07	0.022	0.072	9.7E-05
4	4	S288C_Ator08_hmg124h_2.JPG.dat	928.3	83.6	0.9	0.11	-0.032	0.107	4.1E-04
YBR224W	YBR224W	S288C_Ator08_hmg124h_2.JPG.dat	990.8	44.5	1.0	0.04	-0.048	0.040	2.0E-05
YBR225W	YBR225W	S288C_Ator08_hmg124h_2.JPG.dat	1023.3	55.3	1.0	0.05	0.014	0.052	4.0E-05
YBR226C	YBR226C	S288C_Ator08_hmg124h_2.JPG.dat	1106.3	27.1	1.0	0.01	0.044	0.009	2.7E-05
YBR227C	YBR227C	S288C_Ator08_hmg124h_2.JPG.dat	1032.3	20.1	1.0	0.01	-0.044	0.013	5.9E-07
YBR228W	YBR228W	S288C_Ator08_hmg124h_2.JPG.dat	1098.8	26.6	1.0	0.03	-0.013	0.026	4.4E-06
YBR229C	YBR229C	S288C_Ator08_hmg124h_2.JPG.dat	1052.3	28.6	1.0	0.02	-0.071	0.018	1.8E-06
YBR230C	YBR230C	S288C_Ator08_hmg124h_2.JPG.dat	1070.8	27.8	1.0	0.01	0.010	0.007	1.8E-05
YBR231C	YBR231C	S288C_Ator08_hmg124h_2.JPG.dat	976.8	94.0	0.9	0.08	0.034	0.084	2.1E-04
YBR232C	YBR232C	S288C_Ator08_hmg124h_2.JPG.dat	1141.0	60.5	1.0	0.05	0.070	0.052	3.4E-05
YBR233W	YBR233W	S288C_Ator08_hmg124h_2.JPG.dat	1141.8	42.2	1.0	0.04	0.014	0.035	1.1E-05

YBR235W	YBR235W	S288C_Ator08_hmg124h_2.JPG.dat	1109.8	99.1	1.0	0.07	-0.118	0.073	1.0E-04
YBR238C	YBR238C	S288C_Ator08_hmg124h_2.JPG.dat	1026.0	84.0	1.0	0.08	0.124	0.081	1.4E-04
YBR239C	YBR239C	S288C_Ator08_hmg124h_2.JPG.dat	1068.5	41.9	1.0	0.04	0.069	0.042	1.8E-05
YBR240C	YBR240C	S288C_Ator08_hmg124h_2.JPG.dat	1072.5	28.2	1.0	0.01	-0.011	0.008	1.9E-05
YBR241C	YBR241C	S288C_Ator08_hmg124h_2.JPG.dat	1040.5	22.7	1.0	0.02	-0.001	0.024	3.7E-06
YBR242W	YBR242W	S288C_Ator08_hmg124h_2.JPG.dat	1061.5	30.3	1.0	0.03	0.015	0.026	4.2E-06
YBR244W	YBR244W	S288C_Ator08_hmg124h_2.JPG.dat	1057.5	13.2	1.0	0.02	-0.008	0.019	1.8E-06
YBR245C	YBR245C	S288C_Ator08_hmg124h_2.JPG.dat	1003.3	29.0	1.0	0.01	-0.031	0.007	1.5E-05
YBR246W	YBR246W	S288C_Ator08_hmg124h_2.JPG.dat	989.5	10.0	0.9	0.01	-0.037	0.011	4.9E-07
YBR248C	YBR248C	S288C_Ator08_hmg124h_2.JPG.dat	527.5	609.1	0.5	0.55	0.045	0.549	1.8E-01
YBR249C	YBR249C	S288C_Ator08_hmg124h_2.JPG.dat	1106.3	35.8	1.0	0.03	0.065	0.033	1.0E-05
YBR250W	YBR250W	S288C_Ator08_hmg124h_2.JPG.dat	1102.0	40.9	1.0	0.03	-0.001	0.029	6.4E-06
YBR255W	YBR255W	S288C_Ator08_hmg124h_2.JPG.dat	947.0	38.8	0.9	0.00	-0.109	0.002	1.1E-06
YBR258C	YBR258C	S288C_Ator08_hmg124h_2.JPG.dat	1018.5	29.1	1.0	0.02	-0.011	0.020	2.2E-06
YBR259W	YBR259W	S288C_Ator08_hmg124h_2.JPG.dat	1032.5	24.7	1.0	0.00	-0.054	0.004	5.8E-06
YBR260C	YBR260C	S288C_Ator08_hmg124h_2.JPG.dat	999.3	21.5	1.0	0.02	-0.070	0.024	4.2E-06
YBR261C	YBR261C	S288C_Ator08_hmg124h_2.JPG.dat	1013.3	52.1	1.0	0.04	0.045	0.044	2.3E-05
YBR262C	YBR262C	S288C_Ator08_hmg124h_2.JPG.dat	1004.0	13.3	1.0	0.01	-0.022	0.007	1.7E-05
YBR263W	YBR263W	S288C_Ator08_hmg124h_2.JPG.dat	1071.0	38.9	1.0	0.03	0.045	0.034	9.9E-06
YBR264C	YBR264C	S288C_Ator08_hmg124h_2.JPG.dat	1031.0	43.4	1.0	0.05	-0.015	0.051	3.7E-05
YBR266C	YBR266C	S288C_Ator08_hmg124h_2.JPG.dat	1044.8	110.3	1.0	0.02	0.122	0.020	1.4E-04
YBR267W	YBR267W	S288C_Ator08_hmg124h_2.JPG.dat	1040.5	150.4	0.9	0.13	0.129	0.133	7.8E-04
YBR269C	YBR269C	S288C_Ator08_hmg124h_2.JPG.dat	1224.3	19.9	1.1	0.06	0.289	0.056	3.1E-05
YBR270C	YBR270C	S288C_Ator08_hmg124h_2.JPG.dat	1018.0	40.8	1.0	0.04	0.035	0.039	1.6E-05
YBR271W	YBR271W	S288C_Ator08_hmg124h_2.JPG.dat	953.5	39.8	0.9	0.04	-0.112	0.037	1.7E-05
YBR272C	YBR272C	S288C_Ator08_hmg124h_2.JPG.dat	1066.8	33.1	1.0	0.03	0.021	0.028	6.2E-06
YBR273C	YBR273C	S288C_Ator08_hmg124h_2.JPG.dat	1019.0	25.7	1.0	0.02	-0.038	0.019	2.0E-06
YBR274W	YBR274W	S288C_Ator08_hmg124h_2.JPG.dat	994.8	46.5	1.0	0.04	-0.093	0.044	2.6E-05
YBR275C	YBR275C	S288C_Ator08_hmg124h_2.JPG.dat	1021.5	47.7	1.0	0.01	0.013	0.014	6.8E-05
YBR276C	YBR276C	S288C_Ator08_hmg124h_2.JPG.dat	1071.5	20.3	1.0	0.01	-0.038	0.007	1.8E-05
YBR277C	YBR277C	S288C_Ator08_hmg124h_2.JPG.dat	1061.8	37.2	1.0	0.04	0.074	0.036	1.2E-05
YBR278W	YBR278W	S288C_Ator08_hmg124h_2.JPG.dat	1090.0	31.3	1.0	0.01	0.060	0.010	3.6E-05
YBR280C	YBR280C	S288C_Ator08_hmg124h_2.JPG.dat	1110.0	26.0	1.0	0.02	-0.030	0.021	2.7E-06
YBR281C	YBR281C	S288C_Ator08_hmg124h_2.JPG.dat	1149.5	49.3	1.1	0.07	0.190	0.070	7.9E-05
YBR283C	YBR283C	S288C_Ator08_hmg124h_2.JPG.dat	1005.8	64.2	1.0	0.06	0.014	0.057	5.6E-05
YBR284W	YBR284W	S288C_Ator08_hmg124h_2.JPG.dat	1108.3	22.9	1.0	0.02	0.050	0.016	1.1E-06
YBR285W	YBR285W	S288C_Ator08_hmg124h_2.JPG.dat	1154.3	66.0	1.0	0.06	-0.025	0.057	4.4E-05
YBR286W	YBR286W	S288C_Ator08_hmg124h_2.JPG.dat	1084.5	21.0	1.0	0.02	0.071	0.023	3.1E-06
YBR287W	YBR287W	S288C_Ator08_hmg124h_2.JPG.dat	1048.0	17.7	1.0	0.02	0.050	0.019	1.8E-06
YBR288C	YBR288C	S288C_Ator08_hmg124h_2.JPG.dat	939.3	30.2	0.9	0.03	-0.056	0.027	7.2E-06
YBR289W	YBR289W	S288C_Ator08_hmg124h_2.JPG.dat	733.3	494.5	0.9	0.09	0.308	0.088	3.0E-03
YBR290W	YBR290W	S288C_Ator08_hmg124h_2.JPG.dat	934.0	51.7	0.9	0.04	-0.179	0.043	3.2E-05
YBR291C	YBR291C	S288C_Ator08_hmg124h_2.JPG.dat	1132.3	34.2	1.0	0.03	0.152	0.032	8.5E-06
YBR292C	YBR292C	S288C_Ator08_hmg124h_2.JPG.dat	1094.5	38.3	1.0	0.03	-0.034	0.033	1.1E-05
YBR293W	YBR293W	S288C_Ator08_hmg124h_2.JPG.dat	1164.8	27.1	1.1	0.05	0.089	0.047	2.4E-05
YBR294W	YBR294W	S288C_Ator08_hmg124h_2.JPG.dat	1155.8	28.9	1.1	0.02	-0.058	0.024	2.8E-06
YBR295W	YBR295W	S288C_Ator08_hmg124h_2.JPG.dat	1119.5	47.4	1.0	0.05	0.065	0.047	2.6E-05
YBR296C	YBR296C	S288C_Ator08_hmg124h_2.JPG.dat	1175.5	57.4	1.0	0.05	-0.003	0.048	2.7E-05
YBR297W	YBR297W	S288C_Ator08_hmg124h_2.JPG.dat	1096.0	30.8	1.0	0.01	0.015	0.006	1.2E-05
YBR298C	YBR298C	S288C_Ator08_hmg124h_2.JPG.dat	1084.3	11.6	1.0	0.01	0.030	0.010	2.8E-07
YBR300C	YBR300C	S288C_Ator08_hmg124h_2.JPG.dat	1071.3	25.6	1.0	0.03	0.010	0.025	4.4E-06
YBR301W	YBR301W	S288C_Ator08_hmg124h_2.JPG.dat	1070.8	26.4	1.0	0.03	-0.010	0.025	4.3E-06
YCL001W	YCL001W	S288C_Ator08_hmg124h_2.JPG.dat	1039.0	30.8	1.0	0.01	-0.050	0.008	2.1E-05
YCL001W-/	YCL001W-A	S288C_Ator08_hmg124h_2.JPG.dat	841.0	562.0	1.0	0.05	-0.083	0.049	7.6E-04
YCL002C	YCL002C	S288C_Ator08_hmg124h_2.JPG.dat	1078.8	49.8	1.0	0.05	-0.005	0.045	2.8E-05
YCL006C	YCL006C	S288C_Ator08_hmg124h_2.JPG.dat	1077.0	27.8	1.0	0.01	0.022	0.015	9.2E-07
YCL009C	YCL009C	S288C_Ator08_hmg124h_2.JPG.dat	1041.5	66.0	0.9	0.07	-0.047	0.067	1.1E-04
YCL010C	YCL010C	S288C_Ator08_hmg124h_2.JPG.dat	1070.0	73.3	0.9	0.06	-0.062	0.062	8.0E-05
YCL011C	YCL011C	S288C_Ator08_hmg124h_2.JPG.dat	1181.8	52.5	1.0	0.04	0.054	0.043	2.1E-05
YCL012W	YCL012W	S288C_Ator08_hmg124h_2.JPG.dat	1170.5	42.2	1.0	0.03	0.043	0.032	8.0E-06
YCL013W	YCL013W	S288C_Ator08_hmg124h_2.JPG.dat	1174.8	14.8	1.0	0.01	0.000	0.005	3.5E-08
YCL014W	YCL014W	S288C_Ator08_hmg124h_2.JPG.dat	1134.8	32.2	1.0	0.02	-0.047	0.018	1.7E-06
YCL016C	YCL016C	S288C_Ator08_hmg124h_2.JPG.dat	1213.0	52.8	1.1	0.04	0.180	0.038	1.2E-05
YCL022C	YCL022C	S288C_Ator08_hmg124h_2.JPG.dat	1191.5	61.3	1.1	0.02	0.083	0.019	1.0E-04
YCL023C	YCL023C	S288C_Ator08_hmg124h_2.JPG.dat	1097.8	56.9	1.0	0.05	-0.085	0.047	3.3E-05
YCL024W	YCL024W	S288C_Ator08_hmg124h_2.JPG.dat	1079.5	101.9	0.9	0.09	-0.115	0.085	2.1E-04
YCL025C	YCL025C	S288C_Ator08_hmg124h_2.JPG.dat	1079.5	67.4	0.9	0.08	-0.091	0.078	1.6E-04
YCL026C	YCL026C	S288C_Ator08_hmg124h_2.JPG.dat	1045.8	28.8	1.0	0.01	-0.055	0.014	6.2E-05
YCL026C-A	YCL026C-A	S288C_Ator08_hmg124h_2.JPG.dat	1030.0	35.9	1.0	0.01	-0.020	0.013	6.1E-05
YCL027W	YCL027W	S288C_Ator08_hmg124h_2.JPG.dat	1099.3	36.2	1.0	0.04	0.087	0.041	1.6E-05

YCL028W	YCL028W	S288C_Ator08_hmg124h_2.JPG.dat	1036.8	25.2	1.0	0.02	-0.049	0.019	2.0E-06
YCL029C	YCL029C	S288C_Ator08_hmg124h_2.JPG.dat	1015.8	15.6	1.0	0.02	0.014	0.023	3.8E-06
YCL030C	YCL030C	S288C_Ator08_hmg124h_2.JPG.dat	257.8	515.5	0.0	0.00	0.000	0.000	
YCL032W	YCL032W	S288C_Ator08_hmg124h_2.JPG.dat	1090.3	40.6	1.0	0.03	-0.003	0.032	8.8E-06
YCL033C	YCL033C	S288C_Ator08_hmg124h_2.JPG.dat	1089.5	45.6	1.0	0.04	0.017	0.040	1.7E-05
YCL034W	YCL034W	S288C_Ator08_hmg124h_2.JPG.dat	1132.8	31.8	1.0	0.03	0.027	0.031	7.3E-06
YCL035C	YCL035C	S288C_Ator08_hmg124h_2.JPG.dat	1088.0	10.7	1.0	0.01	0.049	0.012	5.2E-07
YCL036W	YCL036W	S288C_Ator08_hmg124h_2.JPG.dat	1171.8	34.0	1.0	0.03	0.021	0.029	5.8E-06
YCL037C	YCL037C	S288C_Ator08_hmg124h_2.JPG.dat	924.8	54.5	0.9	0.01	0.184	0.007	2.2E-05
YCL038C	YCL038C	S288C_Ator08_hmg124h_2.JPG.dat	985.8	13.0	1.0	0.01	-0.009	0.012	5.5E-07
YCL039W	YCL039W	S288C_Ator08_hmg124h_2.JPG.dat	1070.3	55.4	1.0	0.05	0.001	0.052	3.5E-05
YCL040W	YCL040W	S288C_Ator08_hmg124h_2.JPG.dat	1115.8	29.7	1.1	0.03	0.059	0.027	4.5E-06
YCL042W	YCL042W	S288C_Ator08_hmg124h_2.JPG.dat	1066.0	24.9	1.0	0.02	0.031	0.025	3.7E-06
YCL044C	YCL044C	S288C_Ator08_hmg124h_2.JPG.dat	1075.8	17.7	1.0	0.01	0.038	0.012	4.0E-07
YCL045C	YCL045C	S288C_Ator08_hmg124h_2.JPG.dat	995.8	22.2	1.0	0.01	-0.043	0.005	1.0E-05
YCL046W	YCL046W	S288C_Ator08_hmg124h_2.JPG.dat	971.5	38.1	0.9	0.01	-0.104	0.014	7.6E-05
YCL047C	YCL047C	S288C_Ator08_hmg124h_2.JPG.dat	1109.0	15.3	1.0	0.01	-0.036	0.008	1.5E-07
YCL048W	YCL048W	S288C_Ator08_hmg124h_2.JPG.dat	1129.5	39.3	1.0	0.04	-0.011	0.037	1.3E-05
YCL049C	YCL049C	S288C_Ator08_hmg124h_2.JPG.dat	1104.5	74.6	1.0	0.07	-0.010	0.069	9.5E-05
YCL050C	YCL050C	S288C_Ator08_hmg124h_2.JPG.dat	1085.5	39.4	1.1	0.02	0.105	0.019	1.5E-06
YCL051W	YCL051W	S288C_Ator08_hmg124h_2.JPG.dat	1050.0	25.1	1.0	0.03	0.015	0.032	7.9E-06
YCL055W	YCL055W	S288C_Ator08_hmg124h_2.JPG.dat	1047.5	34.8	1.0	0.00	0.051	0.001	1.2E-07
YCL056C	YCL056C	S288C_Ator08_hmg124h_2.JPG.dat	1087.0	28.0	1.0	0.02	0.016	0.016	1.1E-06
YCL057W	YCL057W	S288C_Ator08_hmg124h_2.JPG.dat	1024.0	14.0	1.0	0.00	0.006	0.005	2.6E-08
YCL060C	YCL060C	S288C_Ator08_hmg124h_2.JPG.dat	1002.5	29.0	1.0	0.02	0.038	0.022	2.8E-06
YCL061C	YCL061C	S288C_Ator08_hmg124h_2.JPG.dat	922.5	42.4	0.9	0.04	0.002	0.042	2.8E-05
YCL062W	YCL062W	S288C_Ator08_hmg124h_2.JPG.dat	966.3	36.8	0.9	0.03	0.087	0.032	1.1E-05
YCL063W	YCL063W	S288C_Ator08_hmg124h_2.JPG.dat	1016.3	30.4	0.9	0.03	0.156	0.026	5.4E-06
YCL064C	YCL064C	S288C_Ator08_hmg124h_2.JPG.dat	1129.5	36.7	1.0	0.03	-0.022	0.025	4.3E-06
YCL069W	YCL069W	S288C_Ator08_hmg124h_2.JPG.dat	1164.8	25.8	1.0	0.01	0.002	0.009	2.8E-05
YCL074W	YCL074W	S288C_Ator08_hmg124h_2.JPG.dat	1001.0	69.5	1.0	0.01	0.033	0.015	6.5E-05
YCL075W	YCL075W	S288C_Ator08_hmg124h_2.JPG.dat	1065.0	88.3	1.0	0.09	0.045	0.085	1.5E-04
YCL076W	YCL076W	S288C_Ator08_hmg124h_2.JPG.dat	997.5	49.3	1.0	0.05	-0.012	0.048	3.4E-05
YCR001W	YCR001W	S288C_Ator08_hmg124h_2.JPG.dat	1044.3	57.1	1.0	0.06	-0.035	0.060	5.9E-05
YCR005C	YCR005C	S288C_Ator08_hmg124h_2.JPG.dat	1054.3	17.3	1.0	0.02	0.022	0.017	1.2E-06
YCR006C	YCR006C	S288C_Ator08_hmg124h_2.JPG.dat	1068.5	20.2	1.0	0.02	0.007	0.020	1.8E-06
YCR007C	YCR007C	S288C_Ator08_hmg124h_2.JPG.dat	963.5	29.2	0.9	0.03	-0.020	0.027	6.2E-06
YCR008W	YCR008W	S288C_Ator08_hmg124h_2.JPG.dat	984.3	57.5	1.0	0.06	-0.001	0.056	5.6E-05
YCR009C	YCR009C	S288C_Ator08_hmg124h_2.JPG.dat	1122.5	31.4	1.0	0.03	0.033	0.034	9.9E-06
YCR010C	YCR010C	S288C_Ator08_hmg124h_2.JPG.dat	1119.3	17.2	1.0	0.02	0.018	0.018	1.5E-06
YCR011C	YCR011C	S288C_Ator08_hmg124h_2.JPG.dat	1166.5	37.2	1.0	0.04	-0.026	0.035	1.1E-05
YCR014C	YCR014C	S288C_Ator08_hmg124h_2.JPG.dat	1125.0	53.2	1.1	0.02	0.025	0.020	1.2E-04
YCR015C	YCR015C	S288C_Ator08_hmg124h_2.JPG.dat	1074.3	91.5	1.0	0.09	-0.007	0.093	2.0E-04
YCR016W	YCR016W	S288C_Ator08_hmg124h_2.JPG.dat	1118.3	22.2	1.0	0.02	0.046	0.018	1.5E-06
YCR017C	YCR017C	S288C_Ator08_hmg124h_2.JPG.dat	1106.3	61.6	1.0	0.02	0.058	0.016	7.9E-05
YCR019W	YCR019W	S288C_Ator08_hmg124h_2.JPG.dat	1045.8	19.7	1.0	0.02	-0.022	0.025	4.3E-06
YCR020C	YCR020C	S288C_Ator08_hmg124h_2.JPG.dat	1063.5	35.2	1.0	0.04	-0.014	0.039	1.6E-05
YCR020C-A	YCR020C-A	S288C_Ator08_hmg124h_2.JPG.dat	1019.5	23.0	1.0	0.00	0.023	0.002	2.0E-06
YCR021C	YCR021C	S288C_Ator08_hmg124h_2.JPG.dat	1046.0	23.9	1.0	0.02	-0.034	0.022	3.1E-06
YCR022C	YCR022C	S288C_Ator08_hmg124h_2.JPG.dat	1097.5	13.0	1.0	0.02	-0.014	0.016	1.1E-06
YCR023C	YCR023C	S288C_Ator08_hmg124h_2.JPG.dat	1137.5	26.7	1.0	0.01	-0.059	0.006	1.3E-05
YCR024C-A	YCR024C-A	S288C_Ator08_hmg124h_2.JPG.dat	1128.3	42.4	1.0	0.04	0.030	0.037	1.4E-05
YCR025C	YCR025C	S288C_Ator08_hmg124h_2.JPG.dat	1023.0	31.6	1.0	0.01	-0.063	0.015	9.1E-07
YCR026C	YCR026C	S288C_Ator08_hmg124h_2.JPG.dat	1118.8	67.6	1.0	0.07	-0.020	0.066	7.0E-05
YCR027C	YCR027C	S288C_Ator08_hmg124h_2.JPG.dat	1131.5	46.6	1.0	0.04	0.044	0.038	1.4E-05
YCR028C-A	YCR028C-A	S288C_Ator08_hmg124h_2.JPG.dat	0.0	0.0	0.0	0.00	0.000	0.000	
YCR030C	YCR030C	S288C_Ator08_hmg124h_2.JPG.dat	1074.8	21.5	1.0	0.02	-0.025	0.021	2.6E-06
YCR031C	YCR031C	S288C_Ator08_hmg124h_2.JPG.dat	977.8	35.3	0.9	0.03	0.196	0.034	1.4E-05
YCR032W	YCR032W	S288C_Ator08_hmg124h_2.JPG.dat	1016.0	37.8	1.0	0.04	0.028	0.036	1.5E-05
YCR033W	YCR033W	S288C_Ator08_hmg124h_2.JPG.dat	887.0	35.9	0.8	0.04	0.079	0.037	2.4E-05
YCR034W	YCR034W	S288C_Ator08_hmg124h_2.JPG.dat	715.5	31.5	0.7	0.03	-0.070	0.029	2.4E-05
YCR036W	YCR036W	S288C_Ator08_hmg124h_2.JPG.dat	1156.5	55.9	1.0	0.05	0.056	0.050	3.2E-05
YCR037C	YCR037C	S288C_Ator08_hmg124h_2.JPG.dat	1180.3	24.7	1.0	0.02	0.145	0.025	3.8E-06
YCR043C	YCR043C	S288C_Ator08_hmg124h_2.JPG.dat	1172.8	39.3	1.0	0.04	0.051	0.040	1.5E-05
YCR044C	YCR044C	S288C_Ator08_hmg124h_2.JPG.dat	1121.0	65.7	1.0	0.05	0.119	0.053	4.4E-05
YCR045C	YCR045C	S288C_Ator08_hmg124h_2.JPG.dat	1199.0	91.6	1.0	0.08	0.012	0.081	1.3E-04
YCR048W	YCR048W	S288C_Ator08_hmg124h_2.JPG.dat	1106.3	55.4	1.0	0.05	-0.047	0.053	4.9E-05
YCR049C	YCR049C	S288C_Ator08_hmg124h_2.JPG.dat	1133.5	42.1	1.0	0.04	-0.065	0.042	2.0E-05
YCR050C	YCR050C	S288C_Ator08_hmg124h_2.JPG.dat	1219.0	49.9	1.1	0.03	0.129	0.034	8.8E-06
YCR051W	YCR051W	S288C_Ator08_hmg124h_2.JPG.dat	1204.5	28.5	1.1	0.02	0.017	0.020	1.9E-06

YCR053W	YCR053W	S288C_Ator08_hmg124h_2.JPG.dat	1012.8	65.3	0.9	0.06	0.306	0.060	7.6E-05
YCR059C	YCR059C	S288C_Ator08_hmg124h_2.JPG.dat	1136.0	59.8	1.0	0.06	-0.010	0.056	4.9E-05
YCR060W	YCR060W	S288C_Ator08_hmg124h_2.JPG.dat	1191.0	45.3	1.0	0.04	0.011	0.040	1.6E-05
YCR061W	YCR061W	S288C_Ator08_hmg124h_2.JPG.dat	1162.8	84.6	1.0	0.08	-0.029	0.078	1.4E-04
YCR062W	YCR062W	S288C_Ator08_hmg124h_2.JPG.dat	948.5	39.0	0.9	0.04	-0.132	0.045	3.4E-05
YCR063W	YCR063W	S288C_Ator08_hmg124h_2.JPG.dat	1069.8	49.7	1.0	0.04	0.037	0.041	1.8E-05
YCR065W	YCR065W	S288C_Ator08_hmg124h_2.JPG.dat	1098.0	64.9	1.0	0.05	0.013	0.054	4.2E-05
YCR067C	YCR067C	S288C_Ator08_hmg124h_2.JPG.dat	1126.0	69.0	1.0	0.05	-0.006	0.054	3.8E-05
YCR068W	YCR068W	S288C_Ator08_hmg124h_2.JPG.dat	1085.3	35.1	1.0	0.02	-0.045	0.022	3.1E-06
YCR069W	YCR069W	S288C_Ator08_hmg124h_2.JPG.dat	1197.0	54.6	1.1	0.04	0.072	0.041	1.6E-05
YCR071C	YCR071C	S288C_Ator08_hmg124h_2.JPG.dat	1182.8	46.4	1.1	0.04	0.075	0.041	1.5E-05
YCR073C	YCR073C	S288C_Ator08_hmg124h_2.JPG.dat	1074.3	67.4	1.0	0.07	-0.014	0.067	8.1E-05
YCR073W-	YCR073W-A	S288C_Ator08_hmg124h_2.JPG.dat	1085.5	72.3	1.0	0.07	0.014	0.068	8.5E-05
YCR075C	YCR075C	S288C_Ator08_hmg124h_2.JPG.dat	1148.3	17.9	1.0	0.02	0.018	0.017	1.2E-06
YCR076C	YCR076C	S288C_Ator08_hmg124h_2.JPG.dat	1222.3	20.0	1.1	0.04	0.142	0.039	1.2E-05
YCR077C	YCR077C	S288C_Ator08_hmg124h_2.JPG.dat	591.3	20.2	0.6	0.02	-0.345	0.021	1.3E-05
YCR079W	YCR079W	S288C_Ator08_hmg124h_2.JPG.dat	978.3	25.8	0.9	0.03	-0.049	0.026	5.5E-06
YCR081W	YCR081W	S288C_Ator08_hmg124h_2.JPG.dat	739.8	166.3	0.6	0.01	0.077	0.010	9.0E-05
YCR082W	YCR082W	S288C_Ator08_hmg124h_2.JPG.dat	996.5	37.0	1.0	0.01	-0.039	0.011	4.3E-05
YCR083W	YCR083W	S288C_Ator08_hmg124h_2.JPG.dat	1074.3	9.9	1.0	0.01	-0.026	0.011	3.9E-07
YCR085W	YCR085W	S288C_Ator08_hmg124h_2.JPG.dat	1107.8	23.9	1.0	0.02	0.028	0.022	2.6E-06
YCR086W	YCR086W	S288C_Ator08_hmg124h_2.JPG.dat	1007.3	30.4	0.9	0.00	0.017	0.002	1.2E-06
YCR087C-A	YCR087C-A	S288C_Ator08_hmg124h_2.JPG.dat	1125.0	24.1	1.0	0.03	0.064	0.029	5.8E-06
YCR087W	YCR087W	S288C_Ator08_hmg124h_2.JPG.dat	1142.0	36.0	1.0	0.04	0.041	0.035	1.2E-05
YCR088W	YCR088W	S288C_Ator08_hmg124h_2.JPG.dat	1143.3	22.6	1.0	0.02	0.002	0.020	2.2E-06
YCR089W	YCR089W	S288C_Ator08_hmg124h_2.JPG.dat	1066.8	8.1	1.0	0.02	-0.037	0.024	4.0E-06
YCR090C	YCR090C	S288C_Ator08_hmg124h_2.JPG.dat	1064.8	73.7	1.0	0.08	0.053	0.078	1.1E-04
YCR091W	YCR091W	S288C_Ator08_hmg124h_2.JPG.dat	1028.3	27.8	1.0	0.03	-0.009	0.027	5.3E-06
YCR092C	YCR092C	S288C_Ator08_hmg124h_2.JPG.dat	1035.8	8.1	1.0	0.01	-0.051	0.011	4.0E-07
YCR095C	YCR095C	S288C_Ator08_hmg124h_2.JPG.dat	1056.5	51.1	1.0	0.01	0.056	0.008	2.0E-05
YCR098C	YCR098C	S288C_Ator08_hmg124h_2.JPG.dat	1004.0	14.7	1.0	0.02	-0.037	0.017	1.4E-06
YCR099C	YCR099C	S288C_Ator08_hmg124h_2.JPG.dat	1002.3	52.0	1.0	0.05	-0.026	0.051	3.9E-05
YCR100C	YCR100C	S288C_Ator08_hmg124h_2.JPG.dat	1036.3	26.0	1.0	0.02	-0.041	0.020	2.2E-06
YCR101C	YCR101C	S288C_Ator08_hmg124h_2.JPG.dat	1109.0	20.4	1.1	0.02	-0.016	0.021	2.2E-06
YCR102C	YCR102C	S288C_Ator08_hmg124h_2.JPG.dat	1163.0	42.3	1.1	0.03	0.008	0.035	1.0E-05
YCR102W-	YCR102W-A	S288C_Ator08_hmg124h_2.JPG.dat	1050.0	37.1	0.9	0.04	-0.084	0.035	1.5E-05
YCR105W	YCR105W	S288C_Ator08_hmg124h_2.JPG.dat	1120.8	49.8	1.0	0.06	0.028	0.059	5.1E-05
YCR106W	YCR106W	S288C_Ator08_hmg124h_2.JPG.dat	1049.3	45.5	1.1	0.04	0.020	0.044	2.0E-05
YDL001W	YDL001W	S288C_Ator08_hmg124h_2.JPG.dat	1028.5	23.0	1.0	0.01	0.067	0.009	2.8E-05
YDL002C	YDL002C	S288C_Ator08_hmg124h_2.JPG.dat	1004.8	41.8	1.0	0.04	0.077	0.037	1.5E-05
YDL006W	YDL006W	S288C_Ator08_hmg124h_2.JPG.dat	756.3	248.5	0.7	0.25	0.038	0.251	9.4E-03
YDL010W	YDL010W	S288C_Ator08_hmg124h_2.JPG.dat	982.3	61.3	1.0	0.06	0.015	0.062	6.9E-05
YDL011C	YDL011C	S288C_Ator08_hmg124h_2.JPG.dat	1025.8	13.4	1.0	0.01	-0.029	0.013	5.7E-07
YDL012C	YDL012C	S288C_Ator08_hmg124h_2.JPG.dat	1054.5	14.0	1.0	0.01	0.054	0.012	4.8E-07
YDL013W	YDL013W	S288C_Ator08_hmg124h_2.JPG.dat	860.5	140.6	0.8	0.13	0.187	0.130	1.0E-03
YDL018C	YDL018C	S288C_Ator08_hmg124h_2.JPG.dat	1052.5	68.3	1.0	0.06	-0.006	0.065	8.0E-05
YDL019C	YDL019C	S288C_Ator08_hmg124h_2.JPG.dat	1125.8	21.5	1.0	0.02	-0.028	0.022	2.8E-06
YDL020C	YDL020C	S288C_Ator08_hmg124h_2.JPG.dat	1135.8	21.2	1.1	0.04	0.377	0.044	1.9E-05
YDL023C	YDL023C	S288C_Ator08_hmg124h_2.JPG.dat	985.5	55.7	1.0	0.06	0.051	0.056	5.2E-05
YDL024C	YDL024C	S288C_Ator08_hmg124h_2.JPG.dat	964.8	11.9	0.9	0.01	-0.092	0.011	4.0E-07
YDL025C	YDL025C	S288C_Ator08_hmg124h_2.JPG.dat	1116.8	35.6	1.1	0.03	0.000	0.031	6.8E-06
YDL026W	YDL026W	S288C_Ator08_hmg124h_2.JPG.dat	982.8	78.4	1.0	0.07	-0.021	0.068	1.0E-04
YDL027C	YDL027C	S288C_Ator08_hmg124h_2.JPG.dat	1004.5	27.6	1.0	0.03	-0.031	0.026	5.1E-06
YDL033C	YDL033C	S288C_Ator08_hmg124h_2.JPG.dat	1044.8	29.4	1.0	0.03	0.020	0.028	5.5E-06
YDL034W	YDL034W	S288C_Ator08_hmg124h_2.JPG.dat	1064.3	37.9	1.0	0.04	0.002	0.038	1.4E-05
YDL035C	YDL035C	S288C_Ator08_hmg124h_2.JPG.dat	1006.3	22.0	1.0	0.02	-0.004	0.018	1.7E-06
YDL036C	YDL036C	S288C_Ator08_hmg124h_2.JPG.dat	1110.0	21.3	1.0	0.02	-0.009	0.021	2.2E-06
YDL037C	YDL037C	S288C_Ator08_hmg124h_2.JPG.dat	1112.5	27.7	1.0	0.01	-0.015	0.009	2.7E-05
YDL038C	YDL038C	S288C_Ator08_hmg124h_2.JPG.dat	1073.3	50.0	1.0	0.02	0.003	0.022	3.1E-06
YDL039C	YDL039C	S288C_Ator08_hmg124h_2.JPG.dat	1104.5	76.8	1.0	0.06	-0.078	0.064	6.4E-05
YDL040C	YDL040C	S288C_Ator08_hmg124h_2.JPG.dat	1030.5	13.5	0.9	0.02	-0.073	0.016	1.3E-06
YDL041W	YDL041W	S288C_Ator08_hmg124h_2.JPG.dat	0.0	0.0	0.0	0.00	0.000	0.000	
YDL042C	YDL042C	S288C_Ator08_hmg124h_2.JPG.dat	319.5	639.0	0.0	0.00	0.000	0.000	
YDL046W	YDL046W	S288C_Ator08_hmg124h_2.JPG.dat	1143.3	18.6	1.0	0.00	0.015	0.002	1.7E-06
YDL048C	YDL048C	S288C_Ator08_hmg124h_2.JPG.dat	1026.8	8.3	1.0	0.00	-0.048	0.004	1.6E-08
YDL050C	YDL050C	S288C_Ator08_hmg124h_2.JPG.dat	1068.3	35.2	1.0	0.04	-0.023	0.038	1.5E-05
YDL051W	YDL051W	S288C_Ator08_hmg124h_2.JPG.dat	1103.0	21.6	1.0	0.02	-0.018	0.020	2.0E-06
YDL052C	YDL052C	S288C_Ator08_hmg124h_2.JPG.dat	1086.8	53.5	1.0	0.05	0.059	0.052	3.8E-05
YDL053C	YDL053C	S288C_Ator08_hmg124h_2.JPG.dat	1144.5	71.3	1.0	0.02	0.061	0.017	8.6E-05
YDL054C	YDL054C	S288C_Ator08_hmg124h_2.JPG.dat	1180.0	83.3	1.1	0.08	0.077	0.082	1.3E-04

YDL056W	YDL056W	S288C_Ator08_hmg124h_2.JPG.dat	1121.0	107.6	1.0	0.10	0.046	0.102	2.6E-04
YDL059C	YDL059C	S288C_Ator08_hmg124h_2.JPG.dat	1128.0	33.5	1.0	0.03	-0.051	0.028	5.3E-06
YDL061C	YDL061C	S288C_Ator08_hmg124h_2.JPG.dat	1177.5	38.1	1.0	0.04	0.026	0.036	1.1E-05
YDL062W	YDL062W	S288C_Ator08_hmg124h_2.JPG.dat	1100.5	39.5	1.0	0.03	-0.014	0.031	8.2E-06
YDL065C	YDL065C	S288C_Ator08_hmg124h_2.JPG.dat	1089.0	14.0	1.0	0.01	0.076	0.009	2.1E-07
YDL066W	YDL066W	S288C_Ator08_hmg124h_2.JPG.dat	1088.3	8.7	1.0	0.01	-0.056	0.014	7.0E-07
YDL070W	YDL070W	S288C_Ator08_hmg124h_2.JPG.dat	1044.3	23.6	1.0	0.02	-0.038	0.018	1.7E-06
YDL071C	YDL071C	S288C_Ator08_hmg124h_2.JPG.dat	1082.8	58.2	1.0	0.06	0.001	0.057	4.8E-05
YDL074C	YDL074C	S288C_Ator08_hmg124h_2.JPG.dat	1011.3	31.5	0.9	0.03	0.144	0.027	6.9E-06
YDL076C	YDL076C	S288C_Ator08_hmg124h_2.JPG.dat	1229.3	21.4	1.1	0.02	0.007	0.022	2.3E-06
YDL077C	YDL077C	S288C_Ator08_hmg124h_2.JPG.dat	1068.8	53.8	1.0	0.04	0.181	0.039	1.9E-05
YDL078C	YDL078C	S288C_Ator08_hmg124h_2.JPG.dat	952.3	43.8	0.9	0.01	-0.141	0.013	6.8E-05
YDL079C	YDL079C	S288C_Ator08_hmg124h_2.JPG.dat	1032.0	44.4	1.0	0.04	-0.019	0.041	1.9E-05
YDL080C	YDL080C	S288C_Ator08_hmg124h_2.JPG.dat	956.3	48.6	0.9	0.04	-0.034	0.042	2.7E-05
YDL081C	YDL081C	S288C_Ator08_hmg124h_2.JPG.dat	983.3	21.6	0.9	0.01	0.171	0.006	1.7E-05
YDL082W	YDL082W	S288C_Ator08_hmg124h_2.JPG.dat	1048.0	31.6	1.0	0.03	0.149	0.033	1.1E-05
YDL083C	YDL083C	S288C_Ator08_hmg124h_2.JPG.dat	902.0	127.8	0.8	0.02	0.033	0.025	3.6E-04
YDL085W	YDL085W	S288C_Ator08_hmg124h_2.JPG.dat	1103.8	15.6	1.0	0.01	-0.022	0.012	4.2E-07
YDL086W	YDL086W	S288C_Ator08_hmg124h_2.JPG.dat	1095.5	33.9	1.0	0.02	0.007	0.024	3.7E-06
YDL088C	YDL088C	S288C_Ator08_hmg124h_2.JPG.dat	1161.3	61.2	1.1	0.06	0.104	0.059	4.6E-05
YDL089W	YDL089W	S288C_Ator08_hmg124h_2.JPG.dat	1095.3	85.9	1.0	0.08	0.021	0.079	1.4E-04
YDL090C	YDL090C	S288C_Ator08_hmg124h_2.JPG.dat	278.0	556.0	0.0	0.00	0.000	0.000	
YDL091C	YDL091C	S288C_Ator08_hmg124h_2.JPG.dat	1106.0	55.9	1.1	0.04	0.018	0.045	2.0E-05
YDL093W	YDL093W	S288C_Ator08_hmg124h_2.JPG.dat	1088.8	35.6	1.0	0.01	0.022	0.009	2.7E-05
YDL094C	YDL094C	S288C_Ator08_hmg124h_2.JPG.dat	1036.3	31.2	1.0	0.03	-0.025	0.030	7.6E-06
YDL095W	YDL095W	S288C_Ator08_hmg124h_2.JPG.dat	1063.5	18.4	1.0	0.02	-0.015	0.016	1.0E-06
YDL096C	YDL096C	S288C_Ator08_hmg124h_2.JPG.dat	1057.0	16.5	1.0	0.01	-0.023	0.014	7.0E-07
YDL099W	YDL099W	S288C_Ator08_hmg124h_2.JPG.dat	1042.5	16.2	1.0	0.02	-0.010	0.015	1.0E-06
YDL100C	YDL100C	S288C_Ator08_hmg124h_2.JPG.dat	1070.0	47.9	1.0	0.05	0.028	0.046	2.7E-05
YDL104C	YDL104C	S288C_Ator08_hmg124h_2.JPG.dat	1096.5	12.4	1.0	0.01	0.032	0.012	4.9E-07
YDL106C	YDL106C	S288C_Ator08_hmg124h_2.JPG.dat	1115.3	51.2	1.0	0.01	-0.054	0.006	1.2E-05
YDL109C	YDL109C	S288C_Ator08_hmg124h_2.JPG.dat	1130.3	51.8	1.0	0.04	-0.029	0.044	2.3E-05
YDL110C	YDL110C	S288C_Ator08_hmg124h_2.JPG.dat	1150.8	15.5	1.0	0.01	0.003	0.014	7.2E-07
YDL112W	YDL112W	S288C_Ator08_hmg124h_2.JPG.dat	1046.5	32.8	1.0	0.04	0.038	0.042	1.8E-05
YDL113C	YDL113C	S288C_Ator08_hmg124h_2.JPG.dat	1019.5	38.6	1.0	0.04	-0.056	0.040	1.8E-05
YDL114W	YDL114W	S288C_Ator08_hmg124h_2.JPG.dat	1053.0	21.6	1.0	0.02	0.009	0.017	1.4E-06
YDL117W	YDL117W	S288C_Ator08_hmg124h_2.JPG.dat	1049.8	37.1	1.0	0.03	0.090	0.034	1.0E-05
YDL118W	YDL118W	S288C_Ator08_hmg124h_2.JPG.dat	899.5	24.5	0.9	0.03	0.171	0.032	1.3E-05
YDL119C	YDL119C	S288C_Ator08_hmg124h_2.JPG.dat	961.3	25.1	0.9	0.01	-0.041	0.009	2.9E-05
YDL121C	YDL121C	S288C_Ator08_hmg124h_2.JPG.dat	1060.0	55.9	1.0	0.05	0.010	0.047	2.6E-05
YDL122W	YDL122W	S288C_Ator08_hmg124h_2.JPG.dat	1116.3	46.3	1.1	0.03	0.104	0.033	7.8E-06
YDL123W	YDL123W	S288C_Ator08_hmg124h_2.JPG.dat	1083.3	45.3	1.0	0.03	-0.010	0.032	8.8E-06
YDL124W	YDL124W	S288C_Ator08_hmg124h_2.JPG.dat	1153.8	40.0	1.0	0.04	0.023	0.038	1.4E-05
YDL125C	YDL125C	S288C_Ator08_hmg124h_2.JPG.dat	1116.8	20.7	1.0	0.01	0.013	0.010	2.5E-07
YDL127W	YDL127W	S288C_Ator08_hmg124h_2.JPG.dat	941.5	31.5	1.0	0.03	0.006	0.027	6.5E-06
YDL128W	YDL128W	S288C_Ator08_hmg124h_2.JPG.dat	1000.5	88.9	1.0	0.09	-0.008	0.086	1.8E-04
YDL129W	YDL129W	S288C_Ator08_hmg124h_2.JPG.dat	1058.0	47.7	1.0	0.04	-0.003	0.044	2.2E-05
YDL130W	YDL130W	S288C_Ator08_hmg124h_2.JPG.dat	973.8	27.3	0.9	0.03	0.177	0.032	1.0E-05
YDL130W-,	YDL130W-A	S288C_Ator08_hmg124h_2.JPG.dat	985.5	15.5	1.0	0.02	-0.012	0.018	1.7E-06
YDL131W	YDL131W	S288C_Ator08_hmg124h_2.JPG.dat	1019.8	43.5	1.0	0.05	-0.008	0.047	2.7E-05
YDL133C-A	YDL133C-A	S288C_Ator08_hmg124h_2.JPG.dat	995.8	56.4	1.0	0.01	0.023	0.014	6.7E-05
YDL133W	YDL133W	S288C_Ator08_hmg124h_2.JPG.dat	1038.0	22.4	1.0	0.02	-0.014	0.021	2.3E-06
YDL134C	YDL134C	S288C_Ator08_hmg124h_2.JPG.dat	1028.0	57.0	1.0	0.06	-0.027	0.062	7.2E-05
YDL134C-A	YDL134C-A	S288C_Ator08_hmg124h_2.JPG.dat	1127.5	38.3	1.0	0.04	0.001	0.038	1.4E-05
YDL135C	YDL135C	S288C_Ator08_hmg124h_2.JPG.dat	1124.8	22.9	1.0	0.02	0.007	0.017	1.3E-06
YDL136W	YDL136W	S288C_Ator08_hmg124h_2.JPG.dat	1005.5	82.9	1.0	0.08	0.247	0.079	1.3E-04
YDL137W	YDL137W	S288C_Ator08_hmg124h_2.JPG.dat	1026.5	65.9	1.0	0.06	-0.022	0.062	6.4E-05
YDL138W	YDL138W	S288C_Ator08_hmg124h_2.JPG.dat	1006.8	38.0	1.0	0.01	-0.054	0.012	5.1E-05
YDL142C	YDL142C	S288C_Ator08_hmg124h_2.JPG.dat	1012.8	32.5	1.0	0.03	0.074	0.033	1.1E-05
YDL144C	YDL144C	S288C_Ator08_hmg124h_2.JPG.dat	1010.5	14.5	1.0	0.00	0.038	0.005	8.4E-06
YDL146W	YDL146W	S288C_Ator08_hmg124h_2.JPG.dat	1031.3	61.9	1.0	0.06	-0.021	0.061	6.0E-05
YDL149W	YDL149W	S288C_Ator08_hmg124h_2.JPG.dat	1057.0	24.2	1.0	0.03	0.033	0.026	4.1E-06
YDL154W	YDL154W	S288C_Ator08_hmg124h_2.JPG.dat	1062.5	13.4	1.0	0.01	0.033	0.014	7.0E-07
YDL155W	YDL155W	S288C_Ator08_hmg124h_2.JPG.dat	1061.8	92.7	1.0	0.02	0.012	0.023	1.6E-04
YDL156W	YDL156W	S288C_Ator08_hmg124h_2.JPG.dat	1094.3	38.6	1.0	0.00	-0.059	0.005	8.4E-06
YDL157C	YDL157C	S288C_Ator08_hmg124h_2.JPG.dat	1107.5	24.5	1.0	0.02	0.007	0.022	2.9E-06
YDL159W	YDL159W	S288C_Ator08_hmg124h_2.JPG.dat	0.0	0.0	0.0	0.00	0.000	0.000	
YDL160C	YDL160C	S288C_Ator08_hmg124h_2.JPG.dat	0.0	0.0	0.0	0.00	0.000	0.000	
YDL161W	YDL161W	S288C_Ator08_hmg124h_2.JPG.dat	1107.8	48.5	1.0	0.05	-0.049	0.051	3.6E-05
YDL162C	YDL162C	S288C_Ator08_hmg124h_2.JPG.dat	1159.0	54.8	1.0	0.05	0.015	0.045	2.3E-05

YDL167C	YDL167C	S288C_Ator08_hmg124h_2.JPG.dat	1042.5	23.1	1.0	0.03	0.008	0.026	5.4E-06
YDL168W	YDL168W	S288C_Ator08_hmg124h_2.JPG.dat	1033.3	44.6	1.0	0.04	-0.019	0.042	2.3E-05
YDL169C	YDL169C	S288C_Ator08_hmg124h_2.JPG.dat	1050.8	66.3	1.0	0.06	0.013	0.060	6.2E-05
YDL170W	YDL170W	S288C_Ator08_hmg124h_2.JPG.dat	1119.3	45.5	1.0	0.04	0.030	0.041	1.7E-05
YDL171C	YDL171C	S288C_Ator08_hmg124h_2.JPG.dat	1070.3	46.9	1.0	0.04	-0.033	0.042	2.3E-05
YDL172C	YDL172C	S288C_Ator08_hmg124h_2.JPG.dat	1129.3	30.1	1.0	0.03	-0.047	0.029	7.2E-06
YDL173W	YDL173W	S288C_Ator08_hmg124h_2.JPG.dat	1089.8	42.8	1.0	0.01	0.066	0.008	2.5E-05
YDL174C	YDL174C	S288C_Ator08_hmg124h_2.JPG.dat	1094.5	39.8	1.0	0.01	0.004	0.006	1.2E-05
YDL175C	YDL175C	S288C_Ator08_hmg124h_2.JPG.dat	1086.3	22.4	1.0	0.02	-0.060	0.024	3.9E-06
YDL176W	YDL176W	S288C_Ator08_hmg124h_2.JPG.dat	1069.3	24.4	1.0	0.01	-0.022	0.009	3.0E-05
YDL177C	YDL177C	S288C_Ator08_hmg124h_2.JPG.dat	1159.0	40.5	1.0	0.03	-0.008	0.031	7.4E-06
YDL178W	YDL178W	S288C_Ator08_hmg124h_2.JPG.dat	1117.5	19.5	1.0	0.02	0.031	0.016	1.1E-06
YDL179W	YDL179W	S288C_Ator08_hmg124h_2.JPG.dat	1036.3	51.5	0.9	0.05	-0.040	0.050	4.1E-05
YDL180W	YDL180W	S288C_Ator08_hmg124h_2.JPG.dat	1111.0	59.9	1.0	0.05	0.019	0.050	3.1E-05
YDL181W	YDL181W	S288C_Ator08_hmg124h_2.JPG.dat	1091.0	51.5	1.0	0.05	-0.018	0.050	3.2E-05
YDL182W	YDL182W	S288C_Ator08_hmg124h_2.JPG.dat	1162.5	31.4	1.1	0.01	0.137	0.006	1.2E-05
YDL183C	YDL183C	S288C_Ator08_hmg124h_2.JPG.dat	1111.3	102.7	1.0	0.03	0.104	0.026	2.2E-04
YDL184C	YDL184C	S288C_Ator08_hmg124h_2.JPG.dat	1092.8	93.3	1.0	0.08	-0.024	0.082	1.7E-04
YOR202W	YOR202W	S288C_Ator08_hmg124h_2.JPG.dat	1057.7	134.5	1.0	0.07	-0.007	0.074	7.9E-308
1	1	S288C_Ator08_hmg124h_9.JPG.dat	364.3	728.5	0.0	0.00	0.000	0.000	
2	2	S288C_Ator08_hmg124h_9.JPG.dat	1344.5	302.4	1.2	0.26	0.205	0.255	2.5E-03
3	3	S288C_Ator08_hmg124h_9.JPG.dat	1100.0	112.4	1.0	0.08	0.000	0.082	1.5E-04
4	4	S288C_Ator08_hmg124h_9.JPG.dat	1154.8	27.8	1.1	0.06	0.156	0.057	3.6E-05
YLL046C	YLL046C	S288C_Ator08_hmg124h_9.JPG.dat	959.0	61.9	1.0	0.07	-0.075	0.073	1.2E-04
YLL047W	YLL047W	S288C_Ator08_hmg124h_9.JPG.dat	994.0	17.8	1.0	0.03	-0.022	0.030	8.0E-06
YLL048C	YLL048C	S288C_Ator08_hmg124h_9.JPG.dat	1073.3	41.2	1.0	0.03	0.064	0.030	6.6E-06
YLL049W	YLL049W	S288C_Ator08_hmg124h_9.JPG.dat	942.3	86.6	0.9	0.07	-0.079	0.075	1.5E-04
YLL051C	YLL051C	S288C_Ator08_hmg124h_9.JPG.dat	1052.8	45.3	1.0	0.04	0.010	0.035	1.2E-05
YLL052C	YLL052C	S288C_Ator08_hmg124h_9.JPG.dat	1039.5	55.2	1.0	0.05	-0.024	0.051	3.5E-05
YLL053C	YLL053C	S288C_Ator08_hmg124h_9.JPG.dat	1036.0	25.4	1.0	0.01	0.012	0.014	7.3E-07
YLL054C	YLL054C	S288C_Ator08_hmg124h_9.JPG.dat	1060.3	50.4	1.0	0.05	0.011	0.047	2.5E-05
YLL055W	YLL055W	S288C_Ator08_hmg124h_9.JPG.dat	996.3	27.3	1.0	0.03	0.013	0.034	1.1E-05
YLL056C	YLL056C	S288C_Ator08_hmg124h_9.JPG.dat	936.8	89.4	1.0	0.01	-0.005	0.009	3.0E-05
YLL057C	YLL057C	S288C_Ator08_hmg124h_9.JPG.dat	1048.3	22.3	1.0	0.01	-0.046	0.009	3.0E-05
YLL058W	YLL058W	S288C_Ator08_hmg124h_9.JPG.dat	993.5	33.9	1.0	0.01	-0.027	0.005	8.6E-06
YLL059C	YLL059C	S288C_Ator08_hmg124h_9.JPG.dat	1051.5	46.6	1.0	0.04	0.030	0.041	1.8E-05
YLL060C	YLL060C	S288C_Ator08_hmg124h_9.JPG.dat	1033.8	18.5	1.0	0.02	0.006	0.018	1.6E-06
YLL061W	YLL061W	S288C_Ator08_hmg124h_9.JPG.dat	1057.0	14.1	1.0	0.02	0.014	0.017	1.2E-06
YLL062C	YLL062C	S288C_Ator08_hmg124h_9.JPG.dat	1027.5	22.2	1.0	0.01	0.019	0.005	9.7E-06
YLL063C	YLL063C	S288C_Ator08_hmg124h_9.JPG.dat	1038.3	35.3	1.0	0.01	-0.009	0.015	7.6E-05
YLR001C	YLR001C	S288C_Ator08_hmg124h_9.JPG.dat	869.3	102.7	0.9	0.02	-0.104	0.019	1.4E-04
YLR003C	YLR003C	S288C_Ator08_hmg124h_9.JPG.dat	951.5	46.0	0.9	0.05	-0.051	0.047	3.5E-05
YLR004C	YLR004C	S288C_Ator08_hmg124h_9.JPG.dat	1039.0	42.2	1.0	0.04	0.029	0.038	1.3E-05
YLR006C	YLR006C	S288C_Ator08_hmg124h_9.JPG.dat	1002.0	27.3	1.0	0.01	-0.014	0.007	1.8E-05
YLR011W	YLR011W	S288C_Ator08_hmg124h_9.JPG.dat	981.3	57.4	1.0	0.06	-0.028	0.056	5.4E-05
YLR012C	YLR012C	S288C_Ator08_hmg124h_9.JPG.dat	984.8	69.3	1.0	0.06	0.037	0.060	5.7E-05
YLR013W	YLR013W	S288C_Ator08_hmg124h_9.JPG.dat	1003.0	25.0	1.0	0.03	0.026	0.028	6.4E-06
YLR014C	YLR014C	S288C_Ator08_hmg124h_9.JPG.dat	982.8	66.4	0.9	0.02	-0.101	0.016	9.9E-05
YLR015W	YLR015W	S288C_Ator08_hmg124h_9.JPG.dat	921.3	32.8	0.9	0.03	-0.101	0.034	1.5E-05
YLR016C	YLR016C	S288C_Ator08_hmg124h_9.JPG.dat	1064.5	14.9	1.0	0.02	-0.001	0.017	1.3E-06
YLR017W	YLR017W	S288C_Ator08_hmg124h_9.JPG.dat	954.3	45.8	0.9	0.05	-0.074	0.045	3.2E-05
YLR018C	YLR018C	S288C_Ator08_hmg124h_9.JPG.dat	989.3	71.3	1.0	0.07	-0.013	0.068	9.2E-05
YLR019W	YLR019W	S288C_Ator08_hmg124h_9.JPG.dat	944.3	66.2	0.9	0.07	-0.064	0.067	9.9E-05
YLR020C	YLR020C	S288C_Ator08_hmg124h_9.JPG.dat	951.8	62.3	1.0	0.06	-0.037	0.064	7.8E-05
YLR021W	YLR021W	S288C_Ator08_hmg124h_9.JPG.dat	1011.3	37.8	1.0	0.04	0.015	0.038	1.4E-05
YLR023C	YLR023C	S288C_Ator08_hmg124h_9.JPG.dat	1012.3	25.0	1.0	0.00	0.049	0.002	1.9E-06
YLR024C	YLR024C	S288C_Ator08_hmg124h_9.JPG.dat	922.5	102.4	1.0	0.12	0.005	0.120	5.5E-04
YLR028C	YLR028C	S288C_Ator08_hmg124h_9.JPG.dat	1090.8	22.9	1.1	0.02	0.055	0.022	2.4E-06
YLR030W	YLR030W	S288C_Ator08_hmg124h_9.JPG.dat	1078.8	49.3	1.0	0.04	0.036	0.044	2.1E-05
YLR031W	YLR031W	S288C_Ator08_hmg124h_9.JPG.dat	1092.5	46.2	1.0	0.01	0.044	0.013	5.5E-05
YLR032W	YLR032W	S288C_Ator08_hmg124h_9.JPG.dat	972.5	34.4	0.9	0.03	-0.094	0.031	1.0E-05
YLR034C	YLR034C	S288C_Ator08_hmg124h_9.JPG.dat	993.8	87.8	0.9	0.08	-0.049	0.084	1.9E-04
YLR035C	YLR035C	S288C_Ator08_hmg124h_9.JPG.dat	1020.5	50.3	1.0	0.04	0.002	0.040	1.7E-05
YLR036C	YLR036C	S288C_Ator08_hmg124h_9.JPG.dat	977.5	61.7	1.0	0.06	-0.039	0.064	7.9E-05
YLR037C	YLR037C	S288C_Ator08_hmg124h_9.JPG.dat	966.3	63.6	1.0	0.07	-0.037	0.067	8.3E-05
YLR038C	YLR038C	S288C_Ator08_hmg124h_9.JPG.dat	810.8	60.6	0.8	0.06	-0.012	0.061	1.1E-04
YLR039C	YLR039C	S288C_Ator08_hmg124h_9.JPG.dat	921.8	54.2	1.0	0.06	-0.025	0.058	5.7E-05
YLR040C	YLR040C	S288C_Ator08_hmg124h_9.JPG.dat	1026.0	46.9	1.0	0.04	0.036	0.044	2.0E-05
YLR041W	YLR041W	S288C_Ator08_hmg124h_9.JPG.dat	1055.3	18.8	1.0	0.01	0.042	0.015	7.8E-07
YLR042C	YLR042C	S288C_Ator08_hmg124h_9.JPG.dat	996.5	61.2	1.0	0.06	-0.057	0.058	5.7E-05

YLR043C	YLR043C	S288C_Ator08_hmg124h_9.JPG.dat	1060.3	24.5	1.0	0.02	0.037	0.018	1.6E-06
YLR044C	YLR044C	S288C_Ator08_hmg124h_9.JPG.dat	1062.5	27.1	1.0	0.02	0.052	0.025	4.0E-06
YLR046C	YLR046C	S288C_Ator08_hmg124h_9.JPG.dat	1054.3	33.3	1.0	0.03	0.013	0.033	9.7E-06
YLR047C	YLR047C	S288C_Ator08_hmg124h_9.JPG.dat	937.8	24.3	0.9	0.03	-0.081	0.027	6.9E-06
YLR048W	YLR048W	S288C_Ator08_hmg124h_9.JPG.dat	1035.3	18.0	1.0	0.01	-0.002	0.005	7.9E-06
YLR049C	YLR049C	S288C_Ator08_hmg124h_9.JPG.dat	958.3	25.1	1.0	0.03	-0.003	0.028	6.5E-06
YLR050C	YLR050C	S288C_Ator08_hmg124h_9.JPG.dat	951.5	44.0	1.0	0.01	0.075	0.011	3.6E-05
YLR053C	YLR053C	S288C_Ator08_hmg124h_9.JPG.dat	897.5	66.8	1.0	0.02	0.012	0.021	1.4E-04
YLR054C	YLR054C	S288C_Ator08_hmg124h_9.JPG.dat	1048.0	90.4	1.0	0.08	0.020	0.084	1.5E-04
YLR055C	YLR055C	S288C_Ator08_hmg124h_9.JPG.dat	1044.8	31.1	1.0	0.03	0.068	0.033	9.3E-06
YLR056W	YLR056W	S288C_Ator08_hmg124h_9.JPG.dat	1054.3	40.3	1.0	0.01	0.058	0.008	2.1E-05
YLR057W	YLR057W	S288C_Ator08_hmg124h_9.JPG.dat	1070.5	17.9	1.0	0.02	0.026	0.025	3.9E-06
YLR058C	YLR058C	S288C_Ator08_hmg124h_9.JPG.dat	1066.0	45.8	1.0	0.00	-0.055	0.003	4.2E-06
YLR059C	YLR059C	S288C_Ator08_hmg124h_9.JPG.dat	1015.0	58.1	1.0	0.06	-0.061	0.056	5.4E-05
YLR061W	YLR061W	S288C_Ator08_hmg124h_9.JPG.dat	1009.3	114.9	1.0	0.10	-0.037	0.096	2.5E-04
YLR062C	YLR062C	S288C_Ator08_hmg124h_9.JPG.dat	994.3	16.4	1.0	0.03	-0.006	0.027	5.7E-06
YLR063W	YLR063W	S288C_Ator08_hmg124h_9.JPG.dat	992.5	13.7	1.0	0.01	0.006	0.009	1.7E-07
YLR064W	YLR064W	S288C_Ator08_hmg124h_9.JPG.dat	920.3	53.7	1.0	0.05	-0.033	0.054	5.0E-05
YLR065C	YLR065C	S288C_Ator08_hmg124h_9.JPG.dat	768.5	513.0	1.1	0.03	0.058	0.028	2.3E-04
YLR070C	YLR070C	S288C_Ator08_hmg124h_9.JPG.dat	1033.3	77.1	1.0	0.07	-0.027	0.065	8.0E-05
YLR072W	YLR072W	S288C_Ator08_hmg124h_9.JPG.dat	1023.8	76.8	1.0	0.07	-0.052	0.074	1.2E-04
YLR073C	YLR073C	S288C_Ator08_hmg124h_9.JPG.dat	983.5	54.8	0.9	0.06	-0.053	0.058	6.5E-05
YLR077W	YLR077W	S288C_Ator08_hmg124h_9.JPG.dat	1010.0	79.3	0.9	0.07	-0.025	0.066	9.5E-05
YLR079W	YLR079W	S288C_Ator08_hmg124h_9.JPG.dat	1014.0	83.6	0.9	0.01	-0.124	0.014	7.6E-05
YLR080W	YLR080W	S288C_Ator08_hmg124h_9.JPG.dat	1021.8	89.0	1.0	0.09	-0.068	0.088	2.1E-04
YLR081W	YLR081W	S288C_Ator08_hmg124h_9.JPG.dat	1014.8	48.2	1.0	0.05	-0.003	0.045	2.5E-05
YLR082C	YLR082C	S288C_Ator08_hmg124h_9.JPG.dat	977.3	70.1	1.0	0.07	-0.060	0.073	1.1E-04
YLR083C	YLR083C	S288C_Ator08_hmg124h_9.JPG.dat	1036.5	37.8	1.1	0.04	0.078	0.040	1.5E-05
YLR084C	YLR084C	S288C_Ator08_hmg124h_9.JPG.dat	1023.3	51.4	1.1	0.02	0.109	0.016	6.6E-05
YLR085C	YLR085C	S288C_Ator08_hmg124h_9.JPG.dat	811.0	62.0	0.9	0.07	-0.115	0.067	1.2E-04
YLR087C	YLR087C	S288C_Ator08_hmg124h_9.JPG.dat	1065.8	19.5	1.1	0.02	0.106	0.017	1.0E-06
YLR089C	YLR089C	S288C_Ator08_hmg124h_9.JPG.dat	937.0	24.5	0.9	0.01	-0.001	0.011	4.7E-07
YLR090W	YLR090W	S288C_Ator08_hmg124h_9.JPG.dat	1079.5	50.8	1.0	0.04	0.064	0.039	1.4E-05
YLR091W	YLR091W	S288C_Ator08_hmg124h_9.JPG.dat	1069.3	37.2	1.0	0.03	0.049	0.026	4.4E-06
YLR092W	YLR092W	S288C_Ator08_hmg124h_9.JPG.dat	1077.0	61.2	1.0	0.06	-0.033	0.062	6.0E-05
YLR093C	YLR093C	S288C_Ator08_hmg124h_9.JPG.dat	1087.0	48.0	1.0	0.04	0.049	0.037	1.2E-05
YLR094C	YLR094C	S288C_Ator08_hmg124h_9.JPG.dat	1018.0	26.4	1.0	0.02	-0.031	0.022	3.2E-06
YLR095C	YLR095C	S288C_Ator08_hmg124h_9.JPG.dat	838.0	73.4	0.9	0.01	-0.130	0.013	7.9E-05
YLR096W	YLR096W	S288C_Ator08_hmg124h_9.JPG.dat	1013.8	44.7	1.0	0.04	-0.013	0.039	1.6E-05
YLR097C	YLR097C	S288C_Ator08_hmg124h_9.JPG.dat	981.3	22.9	1.0	0.03	-0.008	0.035	1.2E-05
YLR098C	YLR098C	S288C_Ator08_hmg124h_9.JPG.dat	871.3	72.0	0.8	0.03	-0.173	0.026	3.4E-04
YLR099C	YLR099C	S288C_Ator08_hmg124h_9.JPG.dat	966.8	32.3	1.0	0.01	0.030	0.006	1.2E-05
YLR102C	YLR102C	S288C_Ator08_hmg124h_9.JPG.dat	1043.8	47.7	1.0	0.04	0.029	0.043	2.0E-05
YLR104W	YLR104W	S288C_Ator08_hmg124h_9.JPG.dat	1132.0	44.6	1.1	0.04	0.069	0.043	1.7E-05
YLR107W	YLR107W	S288C_Ator08_hmg124h_9.JPG.dat	860.0	99.0	0.8	0.10	-0.182	0.099	4.5E-04
YLR108C	YLR108C	S288C_Ator08_hmg124h_9.JPG.dat	1070.0	26.8	1.0	0.01	0.040	0.007	1.5E-05
YLR109W	YLR109W	S288C_Ator08_hmg124h_9.JPG.dat	1067.3	24.5	1.0	0.03	0.024	0.026	4.6E-06
YLR110C	YLR110C	S288C_Ator08_hmg124h_9.JPG.dat	1071.5	47.6	1.0	0.05	0.035	0.049	3.0E-05
YLR111W	YLR111W	S288C_Ator08_hmg124h_9.JPG.dat	995.3	25.6	1.0	0.03	0.020	0.029	6.9E-06
YLR112W	YLR112W	S288C_Ator08_hmg124h_9.JPG.dat	1018.0	14.3	1.0	0.01	-0.002	0.013	5.4E-07
YLR113W	YLR113W	S288C_Ator08_hmg124h_9.JPG.dat	986.0	37.7	1.0	0.03	-0.017	0.034	1.1E-05
YLR114C	YLR114C	S288C_Ator08_hmg124h_9.JPG.dat	862.8	56.2	0.9	0.05	-0.110	0.054	6.7E-05
YLR118C	YLR118C	S288C_Ator08_hmg124h_9.JPG.dat	914.5	41.3	1.0	0.02	0.017	0.016	8.6E-05
YLR119W	YLR119W	S288C_Ator08_hmg124h_9.JPG.dat	1062.8	49.6	1.1	0.01	-0.059	0.015	6.4E-05
YLR120C	YLR120C	S288C_Ator08_hmg124h_9.JPG.dat	1010.0	44.7	1.0	0.04	-0.042	0.040	1.9E-05
YLR121C	YLR121C	S288C_Ator08_hmg124h_9.JPG.dat	1086.8	52.2	1.1	0.01	0.077	0.010	3.0E-05
YLR122C	YLR122C	S288C_Ator08_hmg124h_9.JPG.dat	1052.3	53.2	1.0	0.05	0.000	0.047	2.8E-05
YLR123C	YLR123C	S288C_Ator08_hmg124h_9.JPG.dat	1054.3	12.3	1.0	0.01	0.004	0.007	1.0E-07
YLR124W	YLR124W	S288C_Ator08_hmg124h_9.JPG.dat	1072.0	19.6	1.0	0.00	0.043	0.003	3.6E-06
YLR125W	YLR125W	S288C_Ator08_hmg124h_9.JPG.dat	1017.0	49.7	1.0	0.05	0.005	0.047	2.7E-05
YLR126C	YLR126C	S288C_Ator08_hmg124h_9.JPG.dat	1005.5	44.2	1.0	0.04	0.048	0.040	1.7E-05
YLR128W	YLR128W	S288C_Ator08_hmg124h_9.JPG.dat	1028.5	27.5	1.1	0.03	0.021	0.028	5.1E-06
YLR130C	YLR130C	S288C_Ator08_hmg124h_9.JPG.dat	974.3	29.0	1.0	0.03	0.003	0.028	6.3E-06
YLR131C	YLR131C	S288C_Ator08_hmg124h_9.JPG.dat	976.0	56.6	1.0	0.06	-0.026	0.063	6.1E-05
YLR133W	YLR133W	S288C_Ator08_hmg124h_9.JPG.dat	1080.5	34.8	1.1	0.04	0.068	0.042	1.7E-05
YLR134W	YLR134W	S288C_Ator08_hmg124h_9.JPG.dat	984.8	46.3	1.0	0.01	-0.069	0.014	7.1E-05
YLR135W	YLR135W	S288C_Ator08_hmg124h_9.JPG.dat	1067.5	31.0	1.0	0.00	0.072	0.004	6.1E-06
YLR136C	YLR136C	S288C_Ator08_hmg124h_9.JPG.dat	1102.0	12.0	1.0	0.01	0.043	0.011	3.3E-07
YLR137W	YLR137W	S288C_Ator08_hmg124h_9.JPG.dat	1079.3	55.3	1.0	0.01	-0.033	0.013	6.0E-05
YLR138W	YLR138W	S288C_Ator08_hmg124h_9.JPG.dat	1059.8	43.9	1.0	0.04	0.013	0.042	2.0E-05

YLR142W	YLR142W	S288C_Ator08_hmg124h_9.JPG.dat	1051.3	35.5	1.0	0.01	0.030	0.009	2.5E-05
YLR143W	YLR143W	S288C_Ator08_hmg124h_9.JPG.dat	977.3	26.2	1.0	0.03	0.039	0.031	8.1E-06
YLR144C	YLR144C	S288C_Ator08_hmg124h_9.JPG.dat	990.3	26.1	1.0	0.01	-0.019	0.008	2.3E-05
YLR146C	YLR146C	S288C_Ator08_hmg124h_9.JPG.dat	890.0	68.2	0.9	0.07	-0.056	0.071	1.2E-04
YLR149C	YLR149C	S288C_Ator08_hmg124h_9.JPG.dat	1038.8	58.9	1.1	0.07	0.079	0.073	8.0E-05
YLR150W	YLR150W	S288C_Ator08_hmg124h_9.JPG.dat	1148.5	13.1	1.1	0.01	0.089	0.006	3.6E-08
YLR151C	YLR151C	S288C_Ator08_hmg124h_9.JPG.dat	1039.5	15.6	1.0	0.00	0.020	0.004	6.0E-06
YLR152C	YLR152C	S288C_Ator08_hmg124h_9.JPG.dat	967.5	56.5	0.9	0.05	-0.088	0.054	4.9E-05
YLR154C	YLR154C	S288C_Ator08_hmg124h_9.JPG.dat	980.0	48.9	0.9	0.05	-0.075	0.048	3.7E-05
YLR164W	YLR164W	S288C_Ator08_hmg124h_9.JPG.dat	1074.8	43.9	1.0	0.05	0.047	0.046	2.5E-05
YLR165C	YLR165C	S288C_Ator08_hmg124h_9.JPG.dat	1092.8	25.7	1.0	0.02	0.052	0.020	1.9E-06
YLR168C	YLR168C	S288C_Ator08_hmg124h_9.JPG.dat	1052.5	105.1	1.1	0.10	0.053	0.103	2.5E-04
YLR169W	YLR169W	S288C_Ator08_hmg124h_9.JPG.dat	885.5	24.8	0.9	0.00	-0.086	0.004	6.7E-06
YLR170C	YLR170C	S288C_Ator08_hmg124h_9.JPG.dat	856.3	25.5	0.9	0.01	-0.052	0.007	2.4E-05
YLR171W	YLR171W	S288C_Ator08_hmg124h_9.JPG.dat	878.3	14.7	0.9	0.02	-0.030	0.018	1.9E-06
YLR172C	YLR172C	S288C_Ator08_hmg124h_9.JPG.dat	936.8	66.4	0.9	0.07	-0.112	0.066	9.7E-05
YLR173W	YLR173W	S288C_Ator08_hmg124h_9.JPG.dat	1018.3	21.3	1.0	0.03	-0.012	0.028	6.4E-06
YLR174W	YLR174W	S288C_Ator08_hmg124h_9.JPG.dat	1024.0	54.7	1.0	0.06	0.015	0.062	6.9E-05
YLR176C	YLR176C	S288C_Ator08_hmg124h_9.JPG.dat	1034.3	46.0	1.0	0.04	0.019	0.039	1.6E-05
YLR177W	YLR177W	S288C_Ator08_hmg124h_9.JPG.dat	1006.8	75.2	0.9	0.08	-0.050	0.076	1.4E-04
YLR178C	YLR178C	S288C_Ator08_hmg124h_9.JPG.dat	1085.8	60.1	1.0	0.06	0.029	0.061	6.0E-05
YLR179C	YLR179C	S288C_Ator08_hmg124h_9.JPG.dat	1073.3	33.4	1.0	0.03	0.014	0.034	1.1E-05
YLR180W	YLR180W	S288C_Ator08_hmg124h_9.JPG.dat	1008.5	16.5	1.0	0.01	0.042	0.012	4.5E-07
YLR181C	YLR181C	S288C_Ator08_hmg124h_9.JPG.dat	1013.5	103.4	1.1	0.03	0.036	0.028	2.2E-04
YLR182W	YLR182W	S288C_Ator08_hmg124h_9.JPG.dat	889.5	59.2	0.9	0.05	-0.060	0.052	5.1E-05
YLR183C	YLR183C	S288C_Ator08_hmg124h_9.JPG.dat	1011.8	25.7	1.1	0.04	0.037	0.038	1.2E-05
YLR184W	YLR184W	S288C_Ator08_hmg124h_9.JPG.dat	895.3	60.7	0.8	0.02	-0.083	0.022	2.4E-04
YLR185W	YLR185W	S288C_Ator08_hmg124h_9.JPG.dat	804.0	100.5	0.8	0.09	0.047	0.088	4.3E-04
YLR187W	YLR187W	S288C_Ator08_hmg124h_9.JPG.dat	1079.5	11.0	1.0	0.00	0.057	0.003	6.0E-09
YLR188W	YLR188W	S288C_Ator08_hmg124h_9.JPG.dat	1107.3	73.5	1.0	0.06	0.080	0.064	6.3E-05
YLR189C	YLR189C	S288C_Ator08_hmg124h_9.JPG.dat	1114.8	24.8	1.0	0.02	0.042	0.021	2.3E-06
YLR190W	YLR190W	S288C_Ator08_hmg124h_9.JPG.dat	1138.3	38.4	1.1	0.04	0.057	0.039	1.3E-05
YLR191W	YLR191W	S288C_Ator08_hmg124h_9.JPG.dat	1106.0	30.8	1.0	0.02	0.015	0.022	2.5E-06
YLR192C	YLR192C	S288C_Ator08_hmg124h_9.JPG.dat	1096.8	55.4	1.1	0.06	0.108	0.058	4.0E-05
YLR193C	YLR193C	S288C_Ator08_hmg124h_9.JPG.dat	917.0	53.9	0.9	0.00	-0.098	0.003	3.2E-06
YLR194C	YLR194C	S288C_Ator08_hmg124h_9.JPG.dat	943.8	85.9	1.0	0.09	0.007	0.090	2.0E-04
YLR199C	YLR199C	S288C_Ator08_hmg124h_9.JPG.dat	959.0	61.7	1.0	0.07	0.000	0.069	8.2E-05
YLR200W	YLR200W	S288C_Ator08_hmg124h_9.JPG.dat	909.8	64.5	0.9	0.02	0.073	0.017	1.1E-04
YLR205C	YLR205C	S288C_Ator08_hmg124h_9.JPG.dat	1062.8	53.3	1.0	0.05	0.030	0.052	3.6E-05
YLR206W	YLR206W	S288C_Ator08_hmg124h_9.JPG.dat	991.8	45.8	1.0	0.04	-0.065	0.042	2.4E-05
YLR207W	YLR207W	S288C_Ator08_hmg124h_9.JPG.dat	1101.5	137.3	1.0	0.02	-0.061	0.023	1.7E-04
YLR209C	YLR209C	S288C_Ator08_hmg124h_9.JPG.dat	1065.0	60.8	1.0	0.06	-0.005	0.059	5.3E-05
YLR210W	YLR210W	S288C_Ator08_hmg124h_9.JPG.dat	1082.8	17.0	1.0	0.02	0.040	0.017	1.3E-06
YLR211C	YLR211C	S288C_Ator08_hmg124h_9.JPG.dat	1054.5	39.8	1.0	0.03	0.102	0.032	7.9E-06
YLR213C	YLR213C	S288C_Ator08_hmg124h_9.JPG.dat	1060.8	41.2	1.0	0.04	0.035	0.040	1.5E-05
YLR214W	YLR214W	S288C_Ator08_hmg124h_9.JPG.dat	940.8	51.7	0.9	0.05	-0.019	0.050	4.2E-05
YLR216C	YLR216C	S288C_Ator08_hmg124h_9.JPG.dat	1028.3	36.2	1.0	0.03	0.025	0.029	6.6E-06
YLR217W	YLR217W	S288C_Ator08_hmg124h_9.JPG.dat	987.5	102.8	0.9	0.09	0.023	0.093	2.7E-04
YLR218C	YLR218C	S288C_Ator08_hmg124h_9.JPG.dat	228.3	456.5	0.0	0.00	0.000	0.000	
YLR219W	YLR219W	S288C_Ator08_hmg124h_9.JPG.dat	902.3	45.2	0.9	0.03	-0.087	0.034	1.4E-05
YLR220W	YLR220W	S288C_Ator08_hmg124h_9.JPG.dat	990.3	110.6	1.1	0.02	0.055	0.025	1.8E-04
YLR221C	YLR221C	S288C_Ator08_hmg124h_9.JPG.dat	1034.3	9.2	1.0	0.01	0.047	0.011	3.2E-07
YLR224W	YLR224W	S288C_Ator08_hmg124h_9.JPG.dat	1013.3	33.0	1.0	0.04	0.038	0.043	2.1E-05
YLR225C	YLR225C	S288C_Ator08_hmg124h_9.JPG.dat	980.5	37.3	1.0	0.03	0.017	0.026	4.8E-06
YLR228C	YLR228C	S288C_Ator08_hmg124h_9.JPG.dat	989.8	26.2	1.0	0.02	0.004	0.020	2.1E-06
YLR231C	YLR231C	S288C_Ator08_hmg124h_9.JPG.dat	971.8	50.2	1.0	0.04	-0.043	0.038	1.6E-05
YLR232W	YLR232W	S288C_Ator08_hmg124h_9.JPG.dat	987.5	21.0	1.0	0.03	0.010	0.033	9.2E-06
YLR233C	YLR233C	S288C_Ator08_hmg124h_9.JPG.dat	992.8	36.2	1.0	0.03	0.001	0.027	4.8E-06
YLR234W	YLR234W	S288C_Ator08_hmg124h_9.JPG.dat	888.0	28.3	0.9	0.01	-0.015	0.013	7.4E-07
YLR235C	YLR235C	S288C_Ator08_hmg124h_9.JPG.dat	903.5	74.1	1.0	0.08	-0.054	0.082	1.8E-04
YLR236C	YLR236C	S288C_Ator08_hmg124h_9.JPG.dat	1062.8	62.8	1.1	0.06	0.054	0.056	4.0E-05
YLR237W	YLR237W	S288C_Ator08_hmg124h_9.JPG.dat	922.8	88.8	1.0	0.01	-0.044	0.012	5.6E-05
YLR239C	YLR239C	S288C_Ator08_hmg124h_9.JPG.dat	1041.0	69.1	1.0	0.07	-0.015	0.069	8.2E-05
YLR241W	YLR241W	S288C_Ator08_hmg124h_9.JPG.dat	1024.3	38.5	1.0	0.04	0.025	0.041	2.0E-05
YLR242C	YLR242C	S288C_Ator08_hmg124h_9.JPG.dat	964.8	26.1	0.9	0.01	-0.056	0.005	1.0E-05
YLR246W	YLR246W	S288C_Ator08_hmg124h_9.JPG.dat	1016.5	32.2	1.0	0.03	0.044	0.026	4.6E-06
YLR247C	YLR247C	S288C_Ator08_hmg124h_9.JPG.dat	1035.0	34.6	1.1	0.04	0.052	0.038	1.3E-05
YLR248W	YLR248W	S288C_Ator08_hmg124h_9.JPG.dat	891.0	79.7	0.9	0.08	-0.084	0.079	1.8E-04
YLR250W	YLR250W	S288C_Ator08_hmg124h_9.JPG.dat	999.5	37.8	1.1	0.01	0.064	0.009	2.6E-05
YLR251W	YLR251W	S288C_Ator08_hmg124h_9.JPG.dat	973.0	109.7	1.1	0.01	0.025	0.013	5.4E-05

YLR252W	YLR252W	S288C_Ator08_hmg124h_9.JPG.dat	1021.3	42.1	1.0	0.01	0.011	0.014	6.2E-05
YLR253W	YLR253W	S288C_Ator08_hmg124h_9.JPG.dat	1051.5	45.1	1.0	0.04	0.025	0.039	1.5E-05
YLR254C	YLR254C	S288C_Ator08_hmg124h_9.JPG.dat	1083.8	36.4	1.1	0.03	0.047	0.030	6.6E-06
YLR255C	YLR255C	S288C_Ator08_hmg124h_9.JPG.dat	942.8	67.2	0.9	0.07	-0.086	0.067	1.1E-04
YLR257W	YLR257W	S288C_Ator08_hmg124h_9.JPG.dat	1062.0	29.9	1.0	0.03	0.005	0.027	5.4E-06
YLR258W	YLR258W	S288C_Ator08_hmg124h_9.JPG.dat	1010.0	58.7	1.0	0.01	-0.015	0.006	1.2E-05
YLR261C	YLR261C	S288C_Ator08_hmg124h_9.JPG.dat	1055.5	14.2	1.1	0.01	0.053	0.006	9.9E-06
YLR262C	YLR262C	S288C_Ator08_hmg124h_9.JPG.dat	693.8	19.6	0.7	0.00	-0.187	0.005	1.7E-05
YLR262C-A	YLR262C-A	S288C_Ator08_hmg124h_9.JPG.dat	949.8	59.3	1.0	0.06	-0.037	0.056	5.1E-05
YLR263W	YLR263W	S288C_Ator08_hmg124h_9.JPG.dat	979.0	39.8	1.0	0.04	-0.011	0.041	1.8E-05
YLR264W	YLR264W	S288C_Ator08_hmg124h_9.JPG.dat	246.3	492.5	0.0	0.00	0.000	0.000	
YLR265C	YLR265C	S288C_Ator08_hmg124h_9.JPG.dat	937.5	53.1	1.0	0.07	-0.048	0.066	8.7E-05
YLR266C	YLR266C	S288C_Ator08_hmg124h_9.JPG.dat	918.0	63.7	0.9	0.01	-0.114	0.014	8.8E-05
YLR267W	YLR267W	S288C_Ator08_hmg124h_9.JPG.dat	1045.0	20.5	1.0	0.00	0.018	0.003	3.5E-06
YLR268W	YLR268W	S288C_Ator08_hmg124h_9.JPG.dat	992.0	22.6	1.0	0.02	-0.037	0.017	1.5E-06
YLR269C	YLR269C	S288C_Ator08_hmg124h_9.JPG.dat	1055.0	47.2	1.0	0.04	-0.036	0.045	2.4E-05
YLR271W	YLR271W	S288C_Ator08_hmg124h_9.JPG.dat	1063.0	67.4	1.1	0.02	0.053	0.023	1.5E-04
YLR273C	YLR273C	S288C_Ator08_hmg124h_9.JPG.dat	966.5	50.7	1.0	0.06	-0.034	0.056	5.4E-05
YLR278C	YLR278C	S288C_Ator08_hmg124h_9.JPG.dat	1011.8	15.6	1.0	0.00	0.051	0.004	6.1E-06
YLR279W	YLR279W	S288C_Ator08_hmg124h_9.JPG.dat	936.5	30.0	1.0	0.04	-0.051	0.036	1.4E-05
YLR280C	YLR280C	S288C_Ator08_hmg124h_9.JPG.dat	991.0	20.5	1.0	0.02	0.068	0.020	2.0E-06
YLR281C	YLR281C	S288C_Ator08_hmg124h_9.JPG.dat	940.8	47.0	1.0	0.05	0.000	0.051	3.4E-05
YLR282C	YLR282C	S288C_Ator08_hmg124h_9.JPG.dat	1038.3	14.8	1.1	0.02	0.058	0.017	1.1E-06
YLR283W	YLR283W	S288C_Ator08_hmg124h_9.JPG.dat	895.3	107.0	0.9	0.10	-0.116	0.100	4.0E-04
YLR284C	YLR284C	S288C_Ator08_hmg124h_9.JPG.dat	912.3	102.5	0.9	0.09	-0.107	0.094	3.1E-04
YLR285W	YLR285W	S288C_Ator08_hmg124h_9.JPG.dat	1056.0	18.9	1.0	0.03	0.052	0.028	5.6E-06
YLR286C	YLR286C	S288C_Ator08_hmg124h_9.JPG.dat	1109.8	52.7	1.1	0.01	0.081	0.009	2.1E-05
YLR287C	YLR287C	S288C_Ator08_hmg124h_9.JPG.dat	991.0	60.2	1.0	0.05	-0.039	0.054	4.9E-05
YLR287C-A	YLR287C-A	S288C_Ator08_hmg124h_9.JPG.dat	983.3	80.3	1.0	0.07	-0.049	0.073	1.1E-04
YLR289W	YLR289W	S288C_Ator08_hmg124h_9.JPG.dat	1003.3	26.3	1.0	0.03	0.058	0.028	5.5E-06
YLR290C	YLR290C	S288C_Ator08_hmg124h_9.JPG.dat	953.0	94.4	1.0	0.10	0.037	0.101	2.9E-04
YLR292C	YLR292C	S288C_Ator08_hmg124h_9.JPG.dat	999.3	69.4	1.1	0.07	0.026	0.073	9.1E-05
YLR294C	YLR294C	S288C_Ator08_hmg124h_9.JPG.dat	942.5	74.4	1.0	0.09	0.026	0.091	1.9E-04
YLR296W	YLR296W	S288C_Ator08_hmg124h_9.JPG.dat	1018.8	90.9	1.0	0.03	-0.029	0.029	3.1E-04
YLR297W	YLR297W	S288C_Ator08_hmg124h_9.JPG.dat	990.8	35.5	1.0	0.01	-0.003	0.011	4.5E-05
YLR299W	YLR299W	S288C_Ator08_hmg124h_9.JPG.dat	1068.3	52.0	1.0	0.05	0.060	0.047	2.6E-05
YLR300W	YLR300W	S288C_Ator08_hmg124h_9.JPG.dat	1067.8	80.0	1.1	0.02	0.049	0.023	1.6E-04
YLR303W	YLR303W	S288C_Ator08_hmg124h_9.JPG.dat	1080.0	36.2	1.0	0.04	0.059	0.037	1.3E-05
YLR306W	YLR306W	S288C_Ator08_hmg124h_9.JPG.dat	1098.3	44.6	1.1	0.04	0.055	0.041	1.7E-05
YLR307W	YLR307W	S288C_Ator08_hmg124h_9.JPG.dat	937.5	38.6	0.9	0.03	-0.069	0.032	1.1E-05
YLR309C	YLR309C	S288C_Ator08_hmg124h_9.JPG.dat	918.8	80.8	0.9	0.02	-0.116	0.018	1.4E-04
YLR311C	YLR311C	S288C_Ator08_hmg124h_9.JPG.dat	939.3	61.2	1.0	0.06	-0.028	0.062	7.0E-05
YLR312C	YLR312C	S288C_Ator08_hmg124h_9.JPG.dat	938.5	58.1	1.0	0.06	-0.029	0.058	5.6E-05
YLR313C	YLR313C	S288C_Ator08_hmg124h_9.JPG.dat	983.8	54.4	1.1	0.06	0.042	0.061	5.2E-05
YLR315W	YLR315W	S288C_Ator08_hmg124h_9.JPG.dat	935.0	64.0	0.9	0.06	0.074	0.063	8.3E-05
YLR318W	YLR318W	S288C_Ator08_hmg124h_9.JPG.dat	1078.5	15.6	1.0	0.02	0.019	0.019	1.8E-06
YLR319C	YLR319C	S288C_Ator08_hmg124h_9.JPG.dat	1065.3	23.7	1.0	0.00	0.000	0.005	6.8E-06
YLR320W	YLR320W	S288C_Ator08_hmg124h_9.JPG.dat	1051.8	30.2	1.0	0.03	-0.009	0.029	6.4E-06
YLR324W	YLR324W	S288C_Ator08_hmg124h_9.JPG.dat	1028.8	57.3	1.0	0.05	-0.024	0.055	4.9E-05
YLR325C	YLR325C	S288C_Ator08_hmg124h_9.JPG.dat	1064.0	51.6	1.0	0.05	0.006	0.051	3.6E-05
YLR326W	YLR326W	S288C_Ator08_hmg124h_9.JPG.dat	1045.8	80.2	1.0	0.01	-0.031	0.010	3.7E-05
YLR327C	YLR327C	S288C_Ator08_hmg124h_9.JPG.dat	1093.0	53.6	1.1	0.02	0.095	0.020	1.1E-04
YLR328W	YLR328W	S288C_Ator08_hmg124h_9.JPG.dat	978.8	14.5	1.0	0.00	0.041	0.004	6.4E-06
YLR329W	YLR329W	S288C_Ator08_hmg124h_9.JPG.dat	1008.8	27.9	1.0	0.02	0.049	0.023	3.3E-06
YLR330W	YLR330W	S288C_Ator08_hmg124h_9.JPG.dat	897.8	125.0	0.9	0.12	-0.157	0.115	6.4E-04
YLR332W	YLR332W	S288C_Ator08_hmg124h_9.JPG.dat	819.3	103.1	0.9	0.11	-0.203	0.114	5.6E-04
YLR333C	YLR333C	S288C_Ator08_hmg124h_9.JPG.dat	871.8	72.2	0.9	0.06	-0.099	0.064	9.9E-05
YLR334C	YLR334C	S288C_Ator08_hmg124h_9.JPG.dat	903.8	29.1	0.9	0.04	-0.085	0.036	1.6E-05
YLR335W	YLR335W	S288C_Ator08_hmg124h_9.JPG.dat	1023.0	30.0	1.0	0.02	0.060	0.020	2.0E-06
YLR337C	YLR337C	S288C_Ator08_hmg124h_9.JPG.dat	906.5	239.1	1.0	0.01	0.044	0.012	5.1E-05
YLR338W	YLR338W	S288C_Ator08_hmg124h_9.JPG.dat	605.5	229.5	0.6	0.23	-0.170	0.230	1.4E-02
YLR341W	YLR341W	S288C_Ator08_hmg124h_9.JPG.dat	1047.8	36.5	1.0	0.02	0.027	0.023	2.8E-06
YLR342W	YLR342W	S288C_Ator08_hmg124h_9.JPG.dat	952.3	75.2	1.0	0.07	-0.009	0.072	1.1E-04
YLR343W	YLR343W	S288C_Ator08_hmg124h_9.JPG.dat	965.0	50.8	1.0	0.05	-0.025	0.050	3.5E-05
YLR344W	YLR344W	S288C_Ator08_hmg124h_9.JPG.dat	1002.3	28.7	1.0	0.04	0.051	0.041	1.7E-05
YLR345W	YLR345W	S288C_Ator08_hmg124h_9.JPG.dat	945.5	71.6	1.0	0.01	-0.013	0.008	2.0E-05
YLR346C	YLR346C	S288C_Ator08_hmg124h_9.JPG.dat	838.5	29.8	0.9	0.04	-0.094	0.036	1.6E-05
YLR348C	YLR348C	S288C_Ator08_hmg124h_9.JPG.dat	985.8	57.9	1.0	0.05	-0.025	0.053	4.1E-05
YLR349W	YLR349W	S288C_Ator08_hmg124h_9.JPG.dat	1031.3	66.4	1.0	0.07	0.028	0.066	7.5E-05
YLR350W	YLR350W	S288C_Ator08_hmg124h_9.JPG.dat	936.3	63.3	0.9	0.06	-0.053	0.063	8.4E-05

YLR351C	YLR351C	S288C_Ator08_hmg124h_9.JPG.dat	1021.0	22.9	1.0	0.02	0.015	0.022	2.9E-06
YLR352W	YLR352W	S288C_Ator08_hmg124h_9.JPG.dat	1048.3	8.7	1.0	0.01	0.040	0.014	7.4E-07
YLR353W	YLR353W	S288C_Ator08_hmg124h_9.JPG.dat	970.3	58.5	0.9	0.06	-0.067	0.056	5.6E-05
YLR354C	YLR354C	S288C_Ator08_hmg124h_9.JPG.dat	1009.3	13.0	1.0	0.01	0.052	0.014	7.6E-07
YLR356W	YLR356W	S288C_Ator08_hmg124h_9.JPG.dat	1030.3	54.3	1.1	0.06	0.047	0.060	5.0E-05
YLR357W	YLR357W	S288C_Ator08_hmg124h_9.JPG.dat	664.8	253.8	0.6	0.10	-0.046	0.101	1.0E-02
YLR360W	YLR360W	S288C_Ator08_hmg124h_9.JPG.dat	843.5	27.5	0.9	0.03	-0.043	0.031	1.2E-05
YLR361C	YLR361C	S288C_Ator08_hmg124h_9.JPG.dat	889.0	44.4	0.9	0.02	-0.099	0.015	9.2E-05
YLR362W	YLR362W	S288C_Ator08_hmg124h_9.JPG.dat	0.0	0.0	0.0	0.00	0.000	0.000	
YLR363C	YLR363C	S288C_Ator08_hmg124h_9.JPG.dat	1080.5	50.5	1.0	0.05	0.012	0.048	2.7E-05
YLR364W	YLR364W	S288C_Ator08_hmg124h_9.JPG.dat	1054.5	38.0	1.0	0.04	0.035	0.040	1.7E-05
YLR365W	YLR365W	S288C_Ator08_hmg124h_9.JPG.dat	1091.3	17.2	1.0	0.01	0.035	0.009	2.0E-07
YLR366W	YLR366W	S288C_Ator08_hmg124h_9.JPG.dat	1024.0	30.0	1.0	0.02	-0.003	0.021	2.9E-06
YLR367W	YLR367W	S288C_Ator08_hmg124h_9.JPG.dat	993.5	14.3	0.9	0.02	0.018	0.016	1.5E-06
YLR368W	YLR368W	S288C_Ator08_hmg124h_9.JPG.dat	953.3	48.8	1.0	0.04	-0.018	0.043	2.5E-05
YLR370C	YLR370C	S288C_Ator08_hmg124h_9.JPG.dat	896.8	38.8	0.9	0.04	-0.122	0.044	3.0E-05
YLR371W	YLR371W	S288C_Ator08_hmg124h_9.JPG.dat	337.5	124.2	0.4	0.13	0.035	0.130	1.3E-02
YLR372W	YLR372W	S288C_Ator08_hmg124h_9.JPG.dat	1014.5	21.3	1.1	0.03	0.072	0.026	4.0E-06
YLR373C	YLR373C	S288C_Ator08_hmg124h_9.JPG.dat	831.5	61.9	0.9	0.07	0.046	0.072	1.4E-04
YLR374C	YLR374C	S288C_Ator08_hmg124h_9.JPG.dat	952.0	34.8	1.0	0.04	-0.059	0.038	1.7E-05
YLR375W	YLR375W	S288C_Ator08_hmg124h_9.JPG.dat	997.3	20.6	1.0	0.02	-0.008	0.016	1.3E-06
YLR376C	YLR376C	S288C_Ator08_hmg124h_9.JPG.dat	1051.8	48.0	1.0	0.04	0.030	0.044	2.1E-05
YLR377C	YLR377C	S288C_Ator08_hmg124h_9.JPG.dat	1091.5	29.6	1.0	0.03	0.039	0.029	6.0E-06
YLR380W	YLR380W	S288C_Ator08_hmg124h_9.JPG.dat	1084.5	15.0	1.0	0.02	0.028	0.019	1.6E-06
YLR381W	YLR381W	S288C_Ator08_hmg124h_9.JPG.dat	1093.0	33.3	1.0	0.03	0.068	0.028	5.3E-06
YLR384C	YLR384C	S288C_Ator08_hmg124h_9.JPG.dat	1006.0	66.6	1.0	0.02	0.038	0.025	1.9E-04
YLR385C	YLR385C	S288C_Ator08_hmg124h_9.JPG.dat	1015.3	13.2	1.0	0.02	0.027	0.019	1.6E-06
YLR386W	YLR386W	S288C_Ator08_hmg124h_9.JPG.dat	868.8	33.9	0.9	0.04	-0.181	0.038	1.9E-05
YLR387C	YLR387C	S288C_Ator08_hmg124h_9.JPG.dat	973.8	37.7	1.1	0.04	0.046	0.035	1.0E-05
YLR388W	YLR388W	S288C_Ator08_hmg124h_9.JPG.dat	805.3	137.3	0.8	0.04	-0.088	0.042	9.8E-04
YLR389C	YLR389C	S288C_Ator08_hmg124h_9.JPG.dat	1051.0	62.8	1.0	0.05	0.035	0.049	2.9E-05
YLR390W	YLR390W	S288C_Ator08_hmg124h_9.JPG.dat	989.5	101.4	1.0	0.09	-0.053	0.089	2.1E-04
YLR390W-/	YLR390W-A	S288C_Ator08_hmg124h_9.JPG.dat	928.3	89.6	0.9	0.08	-0.091	0.077	1.6E-04
YLR391W	YLR391W	S288C_Ator08_hmg124h_9.JPG.dat	969.5	82.8	0.9	0.09	-0.082	0.089	2.4E-04
YLR392C	YLR392C	S288C_Ator08_hmg124h_9.JPG.dat	968.3	50.5	0.9	0.05	-0.087	0.049	4.1E-05
YLR393W	YLR393W	S288C_Ator08_hmg124h_9.JPG.dat	1069.8	29.4	1.0	0.04	0.028	0.036	1.2E-05
YLR394W	YLR394W	S288C_Ator08_hmg124h_9.JPG.dat	941.0	71.8	0.9	0.07	-0.070	0.073	1.3E-04
YLR395C	YLR395C	S288C_Ator08_hmg124h_9.JPG.dat	990.3	38.5	1.0	0.04	0.025	0.044	2.3E-05
YLR398C	YLR398C	S288C_Ator08_hmg124h_9.JPG.dat	949.3	113.7	1.0	0.11	0.052	0.108	3.4E-04
YLR400W	YLR400W	S288C_Ator08_hmg124h_9.JPG.dat	1007.5	13.1	1.1	0.03	0.081	0.028	4.8E-06
YLR401C	YLR401C	S288C_Ator08_hmg124h_9.JPG.dat	912.5	72.4	0.9	0.06	-0.155	0.061	8.9E-05
YLR402W	YLR402W	S288C_Ator08_hmg124h_9.JPG.dat	773.0	99.6	0.8	0.10	0.020	0.097	5.9E-04
YLR404W	YLR404W	S288C_Ator08_hmg124h_9.JPG.dat	864.8	158.7	0.8	0.15	-0.151	0.152	1.6E-03
YLR405W	YLR405W	S288C_Ator08_hmg124h_9.JPG.dat	1010.0	42.9	1.0	0.04	-0.001	0.040	1.8E-05
YLR406C	YLR406C	S288C_Ator08_hmg124h_9.JPG.dat	1020.5	81.4	1.0	0.01	0.011	0.012	5.1E-05
YLR407W	YLR407W	S288C_Ator08_hmg124h_9.JPG.dat	1015.8	61.4	1.0	0.05	-0.064	0.053	4.7E-05
YLR408C	YLR408C	S288C_Ator08_hmg124h_9.JPG.dat	1078.5	6.4	1.0	0.01	0.040	0.006	6.8E-08
YLR410W	YLR410W	S288C_Ator08_hmg124h_9.JPG.dat	988.8	56.1	1.0	0.05	-0.010	0.055	4.6E-05
YLR412W	YLR412W	S288C_Ator08_hmg124h_9.JPG.dat	1065.8	26.7	1.1	0.02	0.152	0.023	2.6E-06
YLR413W	YLR413W	S288C_Ator08_hmg124h_9.JPG.dat	964.3	85.7	1.0	0.09	0.024	0.088	1.8E-04
YLR414C	YLR414C	S288C_Ator08_hmg124h_9.JPG.dat	997.0	60.0	1.1	0.07	0.034	0.073	8.6E-05
YOR202W	YOR202W	S288C_Ator08_hmg124h_9.JPG.dat	1076.6	183.6	1.0	0.08	-0.002	0.077	2.0E-304
1	1	S288C_Ator08_hmg124h_13.JPG.dat	2016.5	418.2	#NUM!		#NUM!		
2	2	S288C_Ator08_hmg124h_13.JPG.dat	1437.8	217.0	1.2	0.16	0.231	0.159	7.0E-04
3	3	S288C_Ator08_hmg124h_13.JPG.dat	1442.3	266.8	1.1	0.19	0.190	0.185	1.1E-03
4	4	S288C_Ator08_hmg124h_13.JPG.dat	1314.3	74.6	1.0	0.04	0.030	0.041	1.7E-05
YOR197W	YOR197W	S288C_Ator08_hmg124h_13.JPG.dat	1212.0	29.3	1.0	0.02	0.014	0.019	1.9E-06
YOR202W	YOR202W	S288C_Ator08_hmg124h_13.JPG.dat	1331.1	203.8	1.0	0.11	0.006	0.109	1.4E-270
YOR208W	YOR208W	S288C_Ator08_hmg124h_13.JPG.dat	1208.0	6.4	1.0	0.01	-0.005	0.007	9.9E-08
YOR209C	YOR209C	S288C_Ator08_hmg124h_13.JPG.dat	1130.5	20.9	1.0	0.02	0.010	0.019	2.3E-06
YOR212W	YOR212W	S288C_Ator08_hmg124h_13.JPG.dat	309.8	619.5	0.0	0.00	0.000	0.000	
YOR213C	YOR213C	S288C_Ator08_hmg124h_13.JPG.dat	1173.0	47.8	1.0	0.04	-0.043	0.040	1.8E-05
YOR214C	YOR214C	S288C_Ator08_hmg124h_13.JPG.dat	1203.0	30.4	1.0	0.03	-0.010	0.028	5.6E-06
YOR215C	YOR215C	S288C_Ator08_hmg124h_13.JPG.dat	1197.5	18.3	1.0	0.00	-0.015	0.005	8.2E-06
YOR216C	YOR216C	S288C_Ator08_hmg124h_13.JPG.dat	1172.5	24.9	1.0	0.02	-0.069	0.021	2.9E-06
YOR219C	YOR219C	S288C_Ator08_hmg124h_13.JPG.dat	1164.5	24.8	1.0	0.01	-0.023	0.009	2.6E-05
YOR221C	YOR221C	S288C_Ator08_hmg124h_13.JPG.dat	1219.0	59.7	1.0	0.05	0.002	0.050	3.1E-05
YOR222W	YOR222W	S288C_Ator08_hmg124h_13.JPG.dat	1248.8	64.2	1.0	0.05	-0.007	0.047	2.7E-05
YOR223W	YOR223W	S288C_Ator08_hmg124h_13.JPG.dat	1305.8	37.6	1.1	0.03	0.023	0.027	4.6E-06
YOR225W	YOR225W	S288C_Ator08_hmg124h_13.JPG.dat	1192.0	20.2	1.0	0.02	0.012	0.018	1.8E-06

YOR226C	YOR226C	S288C_Ator08_hmg124h_13.JPG.dat	1208.8	17.6	1.0	0.01	0.020	0.014	7.2E-07
YOR227W	YOR227W	S288C_Ator08_hmg124h_13.JPG.dat	1195.3	28.0	1.0	0.00	-0.002	0.004	5.1E-06
YOR228C	YOR228C	S288C_Ator08_hmg124h_13.JPG.dat	1134.3	55.7	1.0	0.05	-0.051	0.047	3.1E-05
YOR229W	YOR229W	S288C_Ator08_hmg124h_13.JPG.dat	1215.3	55.6	1.0	0.00	0.021	0.005	7.2E-06
YOR230W	YOR230W	S288C_Ator08_hmg124h_13.JPG.dat	1179.3	23.2	1.0	0.00	0.006	0.005	7.3E-06
YOR231W	YOR231W	S288C_Ator08_hmg124h_13.JPG.dat	1186.5	37.1	1.0	0.03	-0.002	0.030	7.9E-06
YOR233W	YOR233W	S288C_Ator08_hmg124h_13.JPG.dat	1207.3	26.1	1.0	0.02	0.027	0.019	1.9E-06
YOR234C	YOR234C	S288C_Ator08_hmg124h_13.JPG.dat	1175.5	57.4	0.9	0.01	-0.007	0.009	2.8E-05
YOR235W	YOR235W	S288C_Ator08_hmg124h_13.JPG.dat	1081.5	37.5	0.9	0.01	0.078	0.012	5.9E-05
YOR237W	YOR237W	S288C_Ator08_hmg124h_13.JPG.dat	1213.5	44.4	1.0	0.04	-0.040	0.038	1.4E-05
YOR238W	YOR238W	S288C_Ator08_hmg124h_13.JPG.dat	1216.8	27.1	1.0	0.02	0.008	0.023	3.0E-06
YOR239W	YOR239W	S288C_Ator08_hmg124h_13.JPG.dat	1148.5	48.6	1.0	0.02	-0.037	0.016	8.7E-05
YOR242C	YOR242C	S288C_Ator08_hmg124h_13.JPG.dat	1116.3	12.0	1.0	0.01	0.029	0.011	3.5E-07
YOR243C	YOR243C	S288C_Ator08_hmg124h_13.JPG.dat	1097.8	37.6	1.0	0.03	0.042	0.033	1.0E-05
YOR245C	YOR245C	S288C_Ator08_hmg124h_13.JPG.dat	1093.0	11.5	1.0	0.00	-0.021	0.002	1.8E-06
YOR246C	YOR246C	S288C_Ator08_hmg124h_13.JPG.dat	1154.0	17.6	1.0	0.01	0.018	0.014	7.7E-07
YOR247W	YOR247W	S288C_Ator08_hmg124h_13.JPG.dat	1143.0	17.7	1.0	0.01	-0.007	0.013	6.7E-07
YOR251C	YOR251C	S288C_Ator08_hmg124h_13.JPG.dat	1201.5	32.2	1.0	0.03	-0.001	0.029	5.8E-06
YOR252W	YOR252W	S288C_Ator08_hmg124h_13.JPG.dat	1166.3	29.6	1.0	0.02	-0.077	0.025	4.4E-06
YOR253W	YOR253W	S288C_Ator08_hmg124h_13.JPG.dat	1127.8	47.2	1.0	0.04	-0.028	0.036	1.4E-05
YOR255W	YOR255W	S288C_Ator08_hmg124h_13.JPG.dat	1184.3	9.9	1.0	0.01	0.006	0.014	7.7E-07
YOR263C	YOR263C	S288C_Ator08_hmg124h_13.JPG.dat	1147.5	21.5	1.0	0.00	-0.006	0.005	7.0E-06
YOR264W	YOR264W	S288C_Ator08_hmg124h_13.JPG.dat	1109.3	46.4	1.0	0.03	0.003	0.033	9.1E-06
YOR265W	YOR265W	S288C_Ator08_hmg124h_13.JPG.dat	980.8	24.8	0.9	0.02	-0.093	0.020	3.1E-06
YOR266W	YOR266W	S288C_Ator08_hmg124h_13.JPG.dat	1105.8	32.7	1.0	0.01	-0.014	0.009	2.8E-05
YOR267C	YOR267C	S288C_Ator08_hmg124h_13.JPG.dat	1040.3	31.7	1.0	0.03	-0.003	0.029	7.6E-06
YOR268C	YOR268C	S288C_Ator08_hmg124h_13.JPG.dat	1129.3	4.3	1.0	0.01	0.068	0.010	2.5E-07
YOR269W	YOR269W	S288C_Ator08_hmg124h_13.JPG.dat	1086.3	59.5	1.0	0.05	-0.025	0.054	5.1E-05
YOR270C	YOR270C	S288C_Ator08_hmg124h_13.JPG.dat	1191.3	13.4	1.0	0.01	0.007	0.008	1.3E-07
YOR271C	YOR271C	S288C_Ator08_hmg124h_13.JPG.dat	1152.8	99.1	1.0	0.07	-0.025	0.075	1.3E-04
YOR273C	YOR273C	S288C_Ator08_hmg124h_13.JPG.dat	1183.8	12.1	1.0	0.01	0.010	0.015	8.9E-07
YOR274W	YOR274W	S288C_Ator08_hmg124h_13.JPG.dat	1191.8	10.1	1.0	0.00	0.016	0.002	1.1E-06
YOR275C	YOR275C	S288C_Ator08_hmg124h_13.JPG.dat	942.5	108.6	0.8	0.03	-0.127	0.030	4.9E-04
YOR276W	YOR276W	S288C_Ator08_hmg124h_13.JPG.dat	1090.8	7.9	1.0	0.01	0.006	0.005	4.5E-08
YOR277C	YOR277C	S288C_Ator08_hmg124h_13.JPG.dat	1069.3	59.7	1.0	0.05	-0.044	0.053	4.6E-05
YOR279C	YOR279C	S288C_Ator08_hmg124h_13.JPG.dat	1089.0	31.4	1.0	0.03	-0.004	0.028	6.6E-06
YOR280C	YOR280C	S288C_Ator08_hmg124h_13.JPG.dat	1129.5	22.5	1.0	0.01	0.011	0.006	1.2E-05
YOR283W	YOR283W	S288C_Ator08_hmg124h_13.JPG.dat	1167.3	17.0	1.0	0.01	0.015	0.013	5.8E-07
YOR284W	YOR284W	S288C_Ator08_hmg124h_13.JPG.dat	1150.0	31.7	1.0	0.03	-0.001	0.028	6.3E-06
YOR285W	YOR285W	S288C_Ator08_hmg124h_13.JPG.dat	1206.0	22.7	1.0	0.02	0.019	0.020	2.0E-06
YOR286W	YOR286W	S288C_Ator08_hmg124h_13.JPG.dat	1195.3	78.7	1.0	0.06	-0.023	0.062	7.0E-05
YOR288C	YOR288C	S288C_Ator08_hmg124h_13.JPG.dat	1220.5	26.8	1.0	0.01	-0.031	0.013	5.1E-07
YOR289W	YOR289W	S288C_Ator08_hmg124h_13.JPG.dat	1144.0	15.9	1.0	0.02	-0.023	0.018	1.6E-06
YOR291W	YOR291W	S288C_Ator08_hmg124h_13.JPG.dat	1186.8	16.5	1.0	0.02	-0.012	0.017	1.1E-06
YOR292C	YOR292C	S288C_Ator08_hmg124h_13.JPG.dat	1139.0	23.2	1.0	0.01	-0.006	0.007	8.2E-08
YOR293W	YOR293W	S288C_Ator08_hmg124h_13.JPG.dat	1142.8	35.5	1.0	0.01	-0.004	0.010	3.0E-05
YOR296W	YOR296W	S288C_Ator08_hmg124h_13.JPG.dat	1131.8	26.3	1.0	0.01	0.022	0.009	2.3E-07
YOR297C	YOR297C	S288C_Ator08_hmg124h_13.JPG.dat	1129.0	27.8	1.0	0.00	0.010	0.001	7.1E-07
YOR298C-/	YOR298C-A	S288C_Ator08_hmg124h_13.JPG.dat	1138.5	26.6	1.0	0.02	0.019	0.024	3.8E-06
YOR298W	YOR298W	S288C_Ator08_hmg124h_13.JPG.dat	1153.8	43.0	1.0	0.00	0.005	0.002	9.3E-07
YOR299W	YOR299W	S288C_Ator08_hmg124h_13.JPG.dat	1147.5	54.1	1.0	0.04	-0.021	0.037	1.5E-05
YOR300W	YOR300W	S288C_Ator08_hmg124h_13.JPG.dat	1250.0	95.4	1.1	0.01	0.043	0.012	4.5E-05
YOR301W	YOR301W	S288C_Ator08_hmg124h_13.JPG.dat	1315.5	36.6	1.0	0.02	0.097	0.019	1.9E-06
YOR302W	YOR302W	S288C_Ator08_hmg124h_13.JPG.dat	0.0	0.0	0.0	0.00	0.000	0.000	
YOR303W	YOR303W	S288C_Ator08_hmg124h_13.JPG.dat	1288.8	72.0	1.0	0.03	0.033	0.030	6.6E-06
YOR304C-/	YOR304C-A	S288C_Ator08_hmg124h_13.JPG.dat	1230.3	108.7	1.0	0.06	-0.044	0.059	6.0E-05
YOR304W	YOR304W	S288C_Ator08_hmg124h_13.JPG.dat	1028.5	46.3	0.8	0.03	-0.143	0.027	9.5E-06
YOR306C	YOR306C	S288C_Ator08_hmg124h_13.JPG.dat	1185.0	111.2	1.0	0.02	-0.045	0.015	7.6E-05
YOR307C	YOR307C	S288C_Ator08_hmg124h_13.JPG.dat	1263.8	46.0	1.0	0.01	-0.020	0.010	2.9E-07
YOR308C	YOR308C	S288C_Ator08_hmg124h_13.JPG.dat	1235.8	19.1	1.0	0.03	-0.009	0.030	7.7E-06
YOR309C	YOR309C	S288C_Ator08_hmg124h_13.JPG.dat	1223.0	16.5	1.0	0.03	-0.021	0.034	1.2E-05
YOR311C	YOR311C	S288C_Ator08_hmg124h_13.JPG.dat	1173.5	38.6	0.9	0.05	-0.032	0.053	5.2E-05
YOR312C	YOR312C	S288C_Ator08_hmg124h_13.JPG.dat	1373.3	37.7	1.1	0.01	0.197	0.014	5.7E-05
YOR313C	YOR313C	S288C_Ator08_hmg124h_13.JPG.dat	1240.5	58.2	1.0	0.05	0.049	0.046	2.6E-05
YOR314W	YOR314W	S288C_Ator08_hmg124h_13.JPG.dat	1227.3	22.2	1.0	0.02	0.045	0.020	2.1E-06
YOR315W	YOR315W	S288C_Ator08_hmg124h_13.JPG.dat	1181.8	16.2	1.0	0.01	-0.010	0.014	7.3E-07
YOR316C	YOR316C	S288C_Ator08_hmg124h_13.JPG.dat	1224.5	59.1	1.0	0.05	0.034	0.053	3.8E-05
YOR317W	YOR317W	S288C_Ator08_hmg124h_13.JPG.dat	1189.5	62.0	1.0	0.05	0.024	0.050	3.4E-05
YOR318C	YOR318C	S288C_Ator08_hmg124h_13.JPG.dat	1240.5	10.8	1.0	0.01	0.011	0.010	2.4E-07
YOR320C	YOR320C	S288C_Ator08_hmg124h_13.JPG.dat	1171.8	14.2	1.0	0.01	0.012	0.012	4.5E-07

YOR321W	YOR321W	S288C_Ator08_hmg124h_13.JPG.dat	1222.0	19.1	1.0	0.00	0.007	0.004	5.4E-06
YOR322C	YOR322C	S288C_Ator08_hmg124h_13.JPG.dat	1142.5	22.6	0.9	0.02	-0.019	0.019	2.1E-06
YOR324C	YOR324C	S288C_Ator08_hmg124h_13.JPG.dat	1244.5	15.4	1.0	0.01	0.028	0.015	8.3E-07
YOR327C	YOR327C	S288C_Ator08_hmg124h_13.JPG.dat	1167.5	50.2	1.0	0.04	-0.015	0.042	2.0E-05
YOR328W	YOR328W	S288C_Ator08_hmg124h_13.JPG.dat	1266.0	40.8	1.0	0.03	-0.006	0.028	5.9E-06
YOR334W	YOR334W	S288C_Ator08_hmg124h_13.JPG.dat	1199.5	29.7	1.0	0.00	-0.026	0.003	2.7E-06
YOR337W	YOR337W	S288C_Ator08_hmg124h_13.JPG.dat	1222.3	11.4	1.0	0.00	-0.021	0.003	2.4E-06
YOR338W	YOR338W	S288C_Ator08_hmg124h_13.JPG.dat	1204.8	24.8	1.0	0.01	-0.016	0.007	1.5E-05
YOR339C	YOR339C	S288C_Ator08_hmg124h_13.JPG.dat	1158.5	12.9	1.0	0.01	0.031	0.011	3.5E-07
YOR342C	YOR342C	S288C_Ator08_hmg124h_13.JPG.dat	1150.3	59.4	1.0	0.05	-0.031	0.049	3.5E-05
YOR343C	YOR343C	S288C_Ator08_hmg124h_13.JPG.dat	1145.3	15.2	1.0	0.01	0.024	0.013	6.7E-07
YOR344C	YOR344C	S288C_Ator08_hmg124h_13.JPG.dat	1169.0	20.9	1.0	0.02	0.008	0.019	2.1E-06
YOR346W	YOR346W	S288C_Ator08_hmg124h_13.JPG.dat	1196.5	12.7	1.0	0.01	0.005	0.009	2.3E-07
YOR347C	YOR347C	S288C_Ator08_hmg124h_13.JPG.dat	1198.0	13.9	1.0	0.01	0.007	0.011	3.7E-07
YOR348C	YOR348C	S288C_Ator08_hmg124h_13.JPG.dat	1167.8	23.3	1.0	0.02	-0.091	0.021	2.8E-06
YOR349W	YOR349W	S288C_Ator08_hmg124h_13.JPG.dat	1266.0	25.6	1.0	0.00	-0.008	0.003	3.7E-06
YOR350C	YOR350C	S288C_Ator08_hmg124h_13.JPG.dat	634.5	82.5	0.5	0.07	-0.004	0.069	5.9E-04
YOR351C	YOR351C	S288C_Ator08_hmg124h_13.JPG.dat	1230.5	10.7	1.0	0.01	0.017	0.008	1.1E-07
YOR352W	YOR352W	S288C_Ator08_hmg124h_13.JPG.dat	1137.3	8.7	1.0	0.01	-0.033	0.011	4.2E-07
YOR354C	YOR354C	S288C_Ator08_hmg124h_13.JPG.dat	1126.5	17.2	1.0	0.01	0.048	0.006	1.2E-05
YOR355W	YOR355W	S288C_Ator08_hmg124h_13.JPG.dat	1050.0	16.5	0.9	0.01	-0.002	0.014	1.0E-06
YOR356W	YOR356W	S288C_Ator08_hmg124h_13.JPG.dat	1143.0	29.2	1.0	0.03	0.019	0.026	4.7E-06
YOR357C	YOR357C	S288C_Ator08_hmg124h_13.JPG.dat	1090.0	32.8	1.0	0.03	-0.020	0.030	8.2E-06
YOR358W	YOR358W	S288C_Ator08_hmg124h_13.JPG.dat	1118.5	35.7	1.0	0.03	-0.016	0.028	6.8E-06
YOR359W	YOR359W	S288C_Ator08_hmg124h_13.JPG.dat	1248.5	21.7	1.1	0.02	0.061	0.018	1.3E-06
YOR360C	YOR360C	S288C_Ator08_hmg124h_13.JPG.dat	765.3	584.0	0.7	0.50	-0.167	0.496	7.8E-02
YOR363C	YOR363C	S288C_Ator08_hmg124h_13.JPG.dat	1191.3	19.9	1.0	0.02	-0.004	0.019	1.8E-06
YOR364W	YOR364W	S288C_Ator08_hmg124h_13.JPG.dat	1156.0	15.6	1.0	0.02	0.000	0.015	9.3E-07
YOR365C	YOR365C	S288C_Ator08_hmg124h_13.JPG.dat	1087.8	20.8	1.0	0.02	-0.007	0.022	3.3E-06
YOR367W	YOR367W	S288C_Ator08_hmg124h_13.JPG.dat	1091.5	41.0	1.0	0.01	0.023	0.010	3.6E-05
YOR368W	YOR368W	S288C_Ator08_hmg124h_13.JPG.dat	1061.0	4.8	1.0	0.01	0.002	0.009	1.9E-07
YOR371C	YOR371C	S288C_Ator08_hmg124h_13.JPG.dat	1118.5	34.5	1.0	0.03	0.014	0.027	5.2E-06
YOR374W	YOR374W	S288C_Ator08_hmg124h_13.JPG.dat	1113.0	20.5	1.0	0.02	-0.003	0.020	2.0E-06
YOR375C	YOR375C	S288C_Ator08_hmg124h_13.JPG.dat	1130.8	24.3	1.0	0.02	0.004	0.020	2.2E-06
YOR376W	YOR376W	S288C_Ator08_hmg124h_13.JPG.dat	1112.3	21.5	1.0	0.02	-0.028	0.018	1.8E-06
YOR377W	YOR377W	S288C_Ator08_hmg124h_13.JPG.dat	1189.0	40.5	1.0	0.03	0.026	0.034	1.0E-05
YOR378W	YOR378W	S288C_Ator08_hmg124h_13.JPG.dat	1182.3	16.7	1.0	0.02	-0.020	0.018	1.4E-06
YOR380W	YOR380W	S288C_Ator08_hmg124h_13.JPG.dat	1208.0	17.8	1.0	0.01	0.004	0.013	5.7E-07
YOR381W	YOR381W	S288C_Ator08_hmg124h_13.JPG.dat	1135.8	16.9	1.0	0.01	-0.034	0.014	7.3E-07
YOR382W	YOR382W	S288C_Ator08_hmg124h_13.JPG.dat	1140.0	18.9	1.0	0.02	0.011	0.019	1.9E-06
YOR383C	YOR383C	S288C_Ator08_hmg124h_13.JPG.dat	1116.0	8.3	1.0	0.01	0.005	0.010	3.2E-07
YOR384W	YOR384W	S288C_Ator08_hmg124h_13.JPG.dat	1080.8	17.7	1.0	0.02	-0.007	0.018	1.6E-06
YOR385W	YOR385W	S288C_Ator08_hmg124h_13.JPG.dat	1128.5	33.5	1.0	0.03	0.016	0.030	7.4E-06
YOR386W	YOR386W	S288C_Ator08_hmg124h_13.JPG.dat	1089.0	11.3	1.0	0.01	-0.017	0.009	1.8E-07
YPL001W	YPL001W	S288C_Ator08_hmg124h_13.JPG.dat	1111.0	39.5	1.0	0.04	0.037	0.036	1.3E-05
YPL003W	YPL003W	S288C_Ator08_hmg124h_13.JPG.dat	1143.3	26.5	1.0	0.02	-0.011	0.022	2.8E-06
YPL004C	YPL004C	S288C_Ator08_hmg124h_13.JPG.dat	1148.3	126.1	1.0	0.02	0.029	0.021	1.4E-04
YPL008W	YPL008W	S288C_Ator08_hmg124h_13.JPG.dat	1207.0	24.5	1.0	0.00	0.024	0.002	1.2E-06
YPL009C	YPL009C	S288C_Ator08_hmg124h_13.JPG.dat	1205.0	62.9	1.0	0.07	0.011	0.065	7.7E-05
YPL014W	YPL014W	S288C_Ator08_hmg124h_13.JPG.dat	1169.8	73.9	1.0	0.06	-0.011	0.060	6.1E-05
YPL017C	YPL017C	S288C_Ator08_hmg124h_13.JPG.dat	1133.0	13.0	1.0	0.02	0.003	0.020	2.2E-06
YPL018W	YPL018W	S288C_Ator08_hmg124h_13.JPG.dat	1137.8	20.5	1.0	0.01	-0.040	0.013	6.1E-07
YPL019C	YPL019C	S288C_Ator08_hmg124h_13.JPG.dat	1117.0	34.5	1.0	0.02	0.007	0.019	2.0E-06
YPL021W	YPL021W	S288C_Ator08_hmg124h_13.JPG.dat	1116.3	51.4	1.0	0.03	-0.007	0.032	9.6E-06
YPL022W	YPL022W	S288C_Ator08_hmg124h_13.JPG.dat	1121.0	28.4	1.0	0.01	-0.003	0.006	1.2E-05
YPL023C	YPL023C	S288C_Ator08_hmg124h_13.JPG.dat	1153.8	24.6	1.0	0.00	0.041	0.001	6.7E-07
YPL024W	YPL024W	S288C_Ator08_hmg124h_13.JPG.dat	994.0	23.3	0.9	0.01	0.022	0.013	9.0E-07
YPL025C	YPL025C	S288C_Ator08_hmg124h_13.JPG.dat	1162.5	32.1	1.0	0.03	-0.042	0.026	5.1E-06
YPL026C	YPL026C	S288C_Ator08_hmg124h_13.JPG.dat	1210.5	29.5	1.0	0.02	0.025	0.018	1.5E-06
YPL027W	YPL027W	S288C_Ator08_hmg124h_13.JPG.dat	1253.3	42.7	0.9	0.04	0.027	0.039	1.9E-05
YPL030W	YPL030W	S288C_Ator08_hmg124h_13.JPG.dat	1329.8	72.5	1.0	0.01	0.043	0.012	4.6E-05
YPL032C	YPL032C	S288C_Ator08_hmg124h_13.JPG.dat	1335.0	70.7	1.0	0.03	0.007	0.029	5.9E-06
YPL033C	YPL033C	S288C_Ator08_hmg124h_13.JPG.dat	1294.3	13.7	1.0	0.02	0.011	0.023	3.0E-06
YPL034W	YPL034W	S288C_Ator08_hmg124h_13.JPG.dat	1260.5	31.6	1.0	0.00	0.017	0.003	2.4E-06
YPL036W	YPL036W	S288C_Ator08_hmg124h_13.JPG.dat	1162.8	150.0	1.0	0.02	-0.043	0.023	1.8E-04
YPL037C	YPL037C	S288C_Ator08_hmg124h_13.JPG.dat	1244.0	36.3	1.0	0.01	-0.003	0.006	5.1E-08
YPL038W	YPL038W	S288C_Ator08_hmg124h_13.JPG.dat	1231.3	25.2	1.0	0.01	-0.035	0.010	3.4E-07
YPL039W	YPL039W	S288C_Ator08_hmg124h_13.JPG.dat	1236.8	32.5	1.0	0.01	-0.016	0.009	2.2E-07
YPL041C	YPL041C	S288C_Ator08_hmg124h_13.JPG.dat	1258.8	46.0	1.0	0.03	-0.002	0.031	8.4E-06
YPL046C	YPL046C	S288C_Ator08_hmg124h_13.JPG.dat	1274.0	83.2	1.0	0.05	0.011	0.046	2.8E-05

YPL047W	YPL047W	S288C_Ator08_hmg124h_13.JPG.dat	1288.0	32.8	1.0	0.00	0.049	0.005	2.9E-08
YPL048W	YPL048W	S288C_Ator08_hmg124h_13.JPG.dat	1282.5	37.5	1.0	0.02	0.020	0.020	2.0E-06
YPL051W	YPL051W	S288C_Ator08_hmg124h_13.JPG.dat	1214.5	46.6	1.0	0.02	-0.045	0.024	4.4E-06
YPL052W	YPL052W	S288C_Ator08_hmg124h_13.JPG.dat	1283.3	65.8	1.1	0.00	0.040	0.002	1.4E-06
YPL053C	YPL053C	S288C_Ator08_hmg124h_13.JPG.dat	1285.0	19.2	1.0	0.03	0.011	0.033	9.2E-06
YPL054W	YPL054W	S288C_Ator08_hmg124h_13.JPG.dat	1227.0	30.2	1.0	0.01	-0.017	0.009	2.8E-05
YPL055C	YPL055C	S288C_Ator08_hmg124h_13.JPG.dat	1270.0	44.1	1.0	0.00	-0.017	0.001	2.3E-07
YPL056C	YPL056C	S288C_Ator08_hmg124h_13.JPG.dat	1080.5	27.4	0.8	0.03	-0.168	0.030	1.2E-05
YPL057C	YPL057C	S288C_Ator08_hmg124h_13.JPG.dat	1207.5	106.1	0.9	0.07	-0.041	0.075	1.4E-04
YPL058C	YPL058C	S288C_Ator08_hmg124h_13.JPG.dat	1197.0	58.1	0.9	0.01	-0.065	0.012	5.5E-05
YPL060W	YPL060W	S288C_Ator08_hmg124h_13.JPG.dat	1222.3	37.1	1.0	0.04	0.141	0.044	2.2E-05
YPL061W	YPL061W	S288C_Ator08_hmg124h_13.JPG.dat	1131.5	30.6	0.9	0.03	-0.015	0.027	6.1E-06
YPL062W	YPL062W	S288C_Ator08_hmg124h_13.JPG.dat	1205.0	23.8	1.0	0.02	-0.022	0.020	2.0E-06
YPL064C	YPL064C	S288C_Ator08_hmg124h_13.JPG.dat	1192.3	15.0	1.0	0.01	0.015	0.010	3.0E-07
YPL066W	YPL066W	S288C_Ator08_hmg124h_13.JPG.dat	978.8	28.4	0.8	0.02	-0.133	0.024	6.4E-06
YPL067C	YPL067C	S288C_Ator08_hmg124h_13.JPG.dat	1215.0	43.8	1.0	0.03	0.036	0.033	8.7E-06
YPL068C	YPL068C	S288C_Ator08_hmg124h_13.JPG.dat	1168.3	33.0	1.0	0.03	-0.003	0.027	5.5E-06
YPL069C	YPL069C	S288C_Ator08_hmg124h_13.JPG.dat	1042.5	88.4	0.9	0.01	-0.046	0.013	6.8E-05
YPL070W	YPL070W	S288C_Ator08_hmg124h_13.JPG.dat	1136.3	68.7	1.0	0.02	-0.002	0.019	1.3E-04
YPL071C	YPL071C	S288C_Ator08_hmg124h_13.JPG.dat	1236.8	21.7	1.0	0.02	0.002	0.018	1.3E-06
YPL073C	YPL073C	S288C_Ator08_hmg124h_13.JPG.dat	1146.5	74.9	1.0	0.02	-0.006	0.019	1.1E-04
YPL074W	YPL074W	S288C_Ator08_hmg124h_13.JPG.dat	1205.5	34.9	1.0	0.03	-0.019	0.029	6.3E-06
YPL079W	YPL079W	S288C_Ator08_hmg124h_13.JPG.dat	1231.5	32.6	1.0	0.03	0.116	0.033	9.4E-06
YPL080C	YPL080C	S288C_Ator08_hmg124h_13.JPG.dat	1197.0	15.1	1.0	0.01	0.085	0.012	4.4E-07
YPL081W	YPL081W	S288C_Ator08_hmg124h_13.JPG.dat	1201.0	33.5	1.0	0.02	0.026	0.020	2.2E-06
YPL086C	YPL086C	S288C_Ator08_hmg124h_13.JPG.dat	1104.5	87.8	0.9	0.02	0.090	0.023	2.0E-04
YPL087W	YPL087W	S288C_Ator08_hmg124h_13.JPG.dat	1128.8	36.5	1.0	0.02	0.025	0.024	4.0E-06
YPL088W	YPL088W	S288C_Ator08_hmg124h_13.JPG.dat	1125.8	9.4	1.0	0.02	0.019	0.016	1.2E-06
YPL089C	YPL089C	S288C_Ator08_hmg124h_13.JPG.dat	1046.5	40.4	0.9	0.01	-0.067	0.009	3.1E-05
YPL090C	YPL090C	S288C_Ator08_hmg124h_13.JPG.dat	1104.3	31.4	1.0	0.02	-0.002	0.024	4.3E-06
YPL091W	YPL091W	S288C_Ator08_hmg124h_13.JPG.dat	1196.3	39.2	1.0	0.03	0.013	0.027	5.0E-06
YPL092W	YPL092W	S288C_Ator08_hmg124h_13.JPG.dat	1217.3	18.8	1.0	0.02	0.000	0.022	2.5E-06
YPL095C	YPL095C	S288C_Ator08_hmg124h_13.JPG.dat	1201.8	73.9	1.0	0.02	0.022	0.023	1.7E-04
YPL096W	YPL096W	S288C_Ator08_hmg124h_13.JPG.dat	1212.5	11.2	1.0	0.01	-0.041	0.014	6.7E-07
YPL098C	YPL098C	S288C_Ator08_hmg124h_13.JPG.dat	1158.0	18.1	1.0	0.02	0.062	0.017	1.5E-06
YPL099C	YPL099C	S288C_Ator08_hmg124h_13.JPG.dat	1140.0	27.2	1.0	0.02	-0.033	0.025	4.4E-06
YPL100W	YPL100W	S288C_Ator08_hmg124h_13.JPG.dat	1071.3	10.2	1.0	0.01	-0.017	0.009	1.9E-07
YPL101W	YPL101W	S288C_Ator08_hmg124h_13.JPG.dat	1117.5	29.1	1.0	0.02	-0.002	0.023	3.5E-06
YPL102C	YPL102C	S288C_Ator08_hmg124h_13.JPG.dat	1113.0	5.9	1.0	0.00	0.012	0.004	4.2E-06
YPL103C	YPL103C	S288C_Ator08_hmg124h_13.JPG.dat	1100.3	23.3	1.0	0.02	0.030	0.018	1.6E-06
YPL105C	YPL105C	S288C_Ator08_hmg124h_13.JPG.dat	1083.0	60.8	1.0	0.05	-0.039	0.052	4.5E-05
YPL106C	YPL106C	S288C_Ator08_hmg124h_13.JPG.dat	1138.8	22.6	1.0	0.02	-0.015	0.023	3.3E-06
YPL107W	YPL107W	S288C_Ator08_hmg124h_13.JPG.dat	1163.8	80.3	1.0	0.07	-0.013	0.066	8.0E-05
YPL108W	YPL108W	S288C_Ator08_hmg124h_13.JPG.dat	1152.3	23.7	1.0	0.01	-0.031	0.006	1.2E-05
YPL109C	YPL109C	S288C_Ator08_hmg124h_13.JPG.dat	1145.0	39.0	1.0	0.03	-0.051	0.034	1.2E-05
YPL110C	YPL110C	S288C_Ator08_hmg124h_13.JPG.dat	1159.0	4.2	1.0	0.00	0.009	0.005	2.5E-08
YPL111W	YPL111W	S288C_Ator08_hmg124h_13.JPG.dat	1118.8	26.0	1.0	0.03	0.007	0.027	5.5E-06
YPL112C	YPL112C	S288C_Ator08_hmg124h_13.JPG.dat	1082.0	21.3	1.0	0.02	0.015	0.022	3.0E-06
YPL113C	YPL113C	S288C_Ator08_hmg124h_13.JPG.dat	1076.0	55.4	1.0	0.05	-0.039	0.049	3.5E-05
YPL114W	YPL114W	S288C_Ator08_hmg124h_13.JPG.dat	1117.5	33.5	1.0	0.01	0.036	0.008	1.9E-05
YPL115C	YPL115C	S288C_Ator08_hmg124h_13.JPG.dat	1089.5	8.3	1.0	0.01	0.018	0.007	1.1E-07
YPL116W	YPL116W	S288C_Ator08_hmg124h_13.JPG.dat	1131.5	2.5	1.0	0.00	0.024	0.003	7.7E-09
YPL119C	YPL119C	S288C_Ator08_hmg124h_13.JPG.dat	1193.3	18.6	1.0	0.01	0.024	0.013	5.9E-07
YPL120W	YPL120W	S288C_Ator08_hmg124h_13.JPG.dat	1085.0	52.9	0.9	0.05	-0.033	0.046	3.3E-05
YPL121C	YPL121C	S288C_Ator08_hmg124h_13.JPG.dat	1179.8	54.7	1.0	0.05	-0.053	0.052	4.0E-05
YPL123C	YPL123C	S288C_Ator08_hmg124h_13.JPG.dat	1178.8	13.5	1.0	0.00	-0.017	0.003	2.1E-06
YPL125W	YPL125W	S288C_Ator08_hmg124h_13.JPG.dat	1123.0	125.8	1.0	0.11	-0.016	0.108	3.7E-04
YPL127C	YPL127C	S288C_Ator08_hmg124h_13.JPG.dat	1105.8	33.8	1.0	0.00	0.000	0.002	1.2E-06
YPL130W	YPL130W	S288C_Ator08_hmg124h_13.JPG.dat	1136.8	16.2	1.0	0.01	0.014	0.013	5.2E-07
YPL133C	YPL133C	S288C_Ator08_hmg124h_13.JPG.dat	1108.8	15.0	1.0	0.01	0.024	0.015	9.0E-07
YPL134C	YPL134C	S288C_Ator08_hmg124h_13.JPG.dat	1111.8	19.3	1.0	0.02	0.020	0.017	1.3E-06
YPL135W	YPL135W	S288C_Ator08_hmg124h_13.JPG.dat	1106.5	9.0	1.0	0.01	0.013	0.009	2.3E-07
YPL136W	YPL136W	S288C_Ator08_hmg124h_13.JPG.dat	1118.5	27.3	1.0	0.02	-0.024	0.024	3.9E-06
YPL137C	YPL137C	S288C_Ator08_hmg124h_13.JPG.dat	1122.3	35.5	1.0	0.03	0.000	0.032	1.0E-05
YPL138C	YPL138C	S288C_Ator08_hmg124h_13.JPG.dat	1136.3	41.8	1.0	0.03	0.000	0.034	1.2E-05
YPL139C	YPL139C	S288C_Ator08_hmg124h_13.JPG.dat	1352.0	80.1	1.1	0.07	0.108	0.066	5.6E-05
YPL140C	YPL140C	S288C_Ator08_hmg124h_13.JPG.dat	1109.5	72.9	0.9	0.07	-0.040	0.068	1.1E-04
YPL141C	YPL141C	S288C_Ator08_hmg124h_13.JPG.dat	1196.3	43.7	1.0	0.02	-0.022	0.022	2.7E-06
YPL144W	YPL144W	S288C_Ator08_hmg124h_13.JPG.dat	1189.0	30.9	1.0	0.02	-0.003	0.020	2.0E-06
YPL145C	YPL145C	S288C_Ator08_hmg124h_13.JPG.dat	1147.8	16.0	1.0	0.01	-0.010	0.008	1.3E-07

YPL147W	YPL147W	S288C_Ator08_hmg124h_13.JPG.dat	1170.0	31.1	1.0	0.01	0.006	0.012	4.5E-07
YPL149W	YPL149W	S288C_Ator08_hmg124h_13.JPG.dat	1143.5	18.9	1.0	0.00	-0.004	0.004	5.7E-06
YPL150W	YPL150W	S288C_Ator08_hmg124h_13.JPG.dat	1148.8	24.7	1.0	0.02	0.014	0.017	1.4E-06
YPL152W	YPL152W	S288C_Ator08_hmg124h_13.JPG.dat	1182.3	16.0	1.0	0.00	-0.007	0.003	3.6E-06
YPL154C	YPL154C	S288C_Ator08_hmg124h_13.JPG.dat	1188.3	32.0	1.0	0.03	0.024	0.033	9.3E-06
YPL155C	YPL155C	S288C_Ator08_hmg124h_13.JPG.dat	1178.8	43.2	1.0	0.04	-0.013	0.038	1.5E-05
YPL156C	YPL156C	S288C_Ator08_hmg124h_13.JPG.dat	1200.3	21.9	1.0	0.01	-0.016	0.007	1.9E-05
YPL157W	YPL157W	S288C_Ator08_hmg124h_13.JPG.dat	1155.0	42.9	0.9	0.03	0.130	0.029	9.4E-06
YPL159C	YPL159C	S288C_Ator08_hmg124h_13.JPG.dat	1273.0	32.0	1.0	0.01	0.078	0.009	1.7E-07
YPL161C	YPL161C	S288C_Ator08_hmg124h_13.JPG.dat	1337.5	59.1	1.1	0.01	0.090	0.009	2.5E-05
YPL162C	YPL162C	S288C_Ator08_hmg124h_13.JPG.dat	1281.0	14.0	1.0	0.01	0.044	0.012	4.5E-07
YPL163C	YPL163C	S288C_Ator08_hmg124h_13.JPG.dat	1309.5	18.9	1.0	0.01	0.022	0.013	5.2E-07
YPL164C	YPL164C	S288C_Ator08_hmg124h_13.JPG.dat	1241.8	16.5	1.0	0.02	0.027	0.021	2.7E-06
YPL165C	YPL165C	S288C_Ator08_hmg124h_13.JPG.dat	1316.8	55.3	1.0	0.02	0.016	0.021	2.2E-06
YPL166W	YPL166W	S288C_Ator08_hmg124h_13.JPG.dat	1252.0	66.5	1.0	0.03	-0.018	0.029	7.2E-06
YPL167C	YPL167C	S288C_Ator08_hmg124h_13.JPG.dat	1264.8	61.3	1.0	0.03	-0.048	0.025	4.5E-06
YPL168W	YPL168W	S288C_Ator08_hmg124h_13.JPG.dat	1205.5	71.1	0.9	0.01	-0.108	0.006	1.4E-05
YPL170W	YPL170W	S288C_Ator08_hmg124h_13.JPG.dat	1209.8	13.0	1.0	0.01	-0.016	0.007	1.8E-05
YPL171C	YPL171C	S288C_Ator08_hmg124h_13.JPG.dat	1216.8	78.4	1.0	0.06	0.015	0.064	7.0E-05
YPL172C	YPL172C	S288C_Ator08_hmg124h_13.JPG.dat	906.0	354.9	0.8	0.30	-0.072	0.297	1.5E-02
YPL174C	YPL174C	S288C_Ator08_hmg124h_13.JPG.dat	518.3	70.0	0.4	0.06	-0.081	0.058	6.6E-04
YPL176C	YPL176C	S288C_Ator08_hmg124h_13.JPG.dat	1158.8	24.0	1.0	0.02	0.004	0.024	3.8E-06
YPL177C	YPL177C	S288C_Ator08_hmg124h_13.JPG.dat	1140.5	18.1	1.0	0.01	-0.059	0.013	6.8E-07
YPL178W	YPL178W	S288C_Ator08_hmg124h_13.JPG.dat	731.8	56.3	0.6	0.05	0.034	0.045	1.1E-04
YPL179W	YPL179W	S288C_Ator08_hmg124h_13.JPG.dat	1181.8	20.1	1.0	0.02	0.038	0.020	2.2E-06
YPL180W	YPL180W	S288C_Ator08_hmg124h_13.JPG.dat	1214.5	21.4	1.0	0.01	-0.003	0.013	5.9E-07
YPL181W	YPL181W	S288C_Ator08_hmg124h_13.JPG.dat	1234.3	14.8	1.0	0.01	0.046	0.010	2.2E-07
YPL182C	YPL182C	S288C_Ator08_hmg124h_13.JPG.dat	1176.8	28.2	1.0	0.02	0.049	0.023	3.5E-06
YPL183C	YPL183C	S288C_Ator08_hmg124h_13.JPG.dat	1068.3	40.7	0.9	0.03	-0.059	0.034	1.5E-05
YPL183W-/	YPL183W-A	S288C_Ator08_hmg124h_13.JPG.dat	637.3	36.5	0.5	0.03	-0.037	0.027	3.9E-05
YPL184C	YPL184C	S288C_Ator08_hmg124h_13.JPG.dat	1179.3	23.4	1.0	0.02	-0.003	0.023	3.4E-06
YPL185W	YPL185W	S288C_Ator08_hmg124h_13.JPG.dat	1227.3	7.2	1.0	0.00	-0.007	0.001	4.5E-07
YPL186C	YPL186C	S288C_Ator08_hmg124h_13.JPG.dat	1173.3	29.5	1.0	0.02	0.016	0.019	1.8E-06
YPL187W	YPL187W	S288C_Ator08_hmg124h_13.JPG.dat	1137.3	25.3	1.0	0.03	-0.033	0.029	6.8E-06
YPL189W	YPL189W	S288C_Ator08_hmg124h_13.JPG.dat	1156.0	10.4	1.0	0.01	-0.030	0.006	5.7E-08
YPL191C	YPL191C	S288C_Ator08_hmg124h_13.JPG.dat	1139.0	31.1	1.0	0.02	-0.013	0.019	1.9E-06
YPL192C	YPL192C	S288C_Ator08_hmg124h_13.JPG.dat	1177.5	11.0	1.0	0.00	0.003	0.002	1.0E-06
YPL194W	YPL194W	S288C_Ator08_hmg124h_13.JPG.dat	1152.5	38.4	1.0	0.03	-0.016	0.027	5.7E-06
YPL195W	YPL195W	S288C_Ator08_hmg124h_13.JPG.dat	1089.8	11.5	0.9	0.01	-0.094	0.014	1.0E-06
YPL196W	YPL196W	S288C_Ator08_hmg124h_13.JPG.dat	1145.8	26.9	1.0	0.00	-0.060	0.004	6.2E-06
YPL197C	YPL197C	S288C_Ator08_hmg124h_13.JPG.dat	1273.8	21.0	1.1	0.02	0.050	0.015	8.1E-07
YPL198W	YPL198W	S288C_Ator08_hmg124h_13.JPG.dat	1189.5	54.6	1.0	0.02	0.032	0.015	7.5E-05
YPL199C	YPL199C	S288C_Ator08_hmg124h_13.JPG.dat	1171.5	10.1	1.0	0.02	-0.018	0.016	1.2E-06
YPL200W	YPL200W	S288C_Ator08_hmg124h_13.JPG.dat	1118.8	38.0	1.0	0.04	-0.017	0.039	1.7E-05
YPL201C	YPL201C	S288C_Ator08_hmg124h_13.JPG.dat	1131.8	34.1	1.0	0.02	0.006	0.024	3.7E-06
YPL202C	YPL202C	S288C_Ator08_hmg124h_13.JPG.dat	1187.8	7.8	1.1	0.01	0.012	0.010	2.6E-07
YPL203W	YPL203W	S288C_Ator08_hmg124h_13.JPG.dat	1104.0	40.7	1.0	0.03	-0.028	0.034	1.1E-05
YPL205C	YPL205C	S288C_Ator08_hmg124h_13.JPG.dat	1103.0	154.9	1.0	0.03	0.054	0.027	2.2E-04
YPL206C	YPL206C	S288C_Ator08_hmg124h_13.JPG.dat	1162.8	21.6	1.0	0.02	-0.006	0.025	4.0E-06
YPL207W	YPL207W	S288C_Ator08_hmg124h_13.JPG.dat	1081.3	42.8	0.9	0.03	-0.039	0.035	1.4E-05
YPL208W	YPL208W	S288C_Ator08_hmg124h_13.JPG.dat	1173.0	14.9	1.0	0.02	-0.016	0.018	1.7E-06
YPL212C	YPL212C	S288C_Ator08_hmg124h_13.JPG.dat	1138.3	45.6	1.0	0.04	-0.030	0.040	2.0E-05
YPL213W	YPL213W	S288C_Ator08_hmg124h_13.JPG.dat	1145.0	11.2	1.0	0.00	0.017	0.003	2.4E-06
YPL214C	YPL214C	S288C_Ator08_hmg124h_13.JPG.dat	1124.0	26.5	1.0	0.02	-0.012	0.023	3.7E-06
YPL216W	YPL216W	S288C_Ator08_hmg124h_13.JPG.dat	1107.0	23.1	1.0	0.02	-0.009	0.019	1.9E-06
YPL219W	YPL219W	S288C_Ator08_hmg124h_13.JPG.dat	1095.5	37.3	1.0	0.03	-0.007	0.032	8.6E-06
YPL220W	YPL220W	S288C_Ator08_hmg124h_13.JPG.dat	1124.8	8.1	1.0	0.01	0.006	0.007	1.0E-07
YPL221W	YPL221W	S288C_Ator08_hmg124h_13.JPG.dat	1089.3	19.2	1.0	0.02	-0.002	0.016	1.1E-06
YPL222W	YPL222W	S288C_Ator08_hmg124h_13.JPG.dat	1161.0	24.1	1.0	0.02	0.013	0.020	1.9E-06
YPL223C	YPL223C	S288C_Ator08_hmg124h_13.JPG.dat	1139.5	26.8	1.0	0.02	0.049	0.020	2.3E-06
YPL224C	YPL224C	S288C_Ator08_hmg124h_13.JPG.dat	1165.0	57.0	1.0	0.05	0.006	0.050	3.3E-05
YPL225W	YPL225W	S288C_Ator08_hmg124h_13.JPG.dat	1217.8	32.1	1.0	0.03	0.053	0.026	4.2E-06
YPL226W	YPL226W	S288C_Ator08_hmg124h_13.JPG.dat	1151.0	22.0	1.0	0.02	-0.020	0.019	2.0E-06
YPL227C	YPL227C	S288C_Ator08_hmg124h_13.JPG.dat	1183.0	79.5	1.0	0.07	0.025	0.068	8.2E-05
YPL229W	YPL229W	S288C_Ator08_hmg124h_13.JPG.dat	1146.3	14.1	1.0	0.01	0.000	0.011	3.5E-07
YPL230W	YPL230W	S288C_Ator08_hmg124h_13.JPG.dat	1150.0	26.1	1.0	0.02	0.017	0.024	3.5E-06
YPL232W	YPL232W	S288C_Ator08_hmg124h_13.JPG.dat	1088.8	47.8	1.0	0.04	-0.053	0.044	2.5E-05
YPL236C	YPL236C	S288C_Ator08_hmg124h_13.JPG.dat	1097.0	21.5	1.0	0.02	0.037	0.020	2.2E-06
YPL239W	YPL239W	S288C_Ator08_hmg124h_13.JPG.dat	1116.8	15.2	1.0	0.01	-0.013	0.014	8.3E-07
YPL240C	YPL240C	S288C_Ator08_hmg124h_13.JPG.dat	1163.5	17.4	1.0	0.01	0.000	0.014	7.7E-07

YPL241C	YPL241C	S288C_Ator08_hmg124h_13.JPG.dat	1131.5	88.6	1.0	0.08	-0.014	0.076	1.2E-04
YPL244C	YPL244C	S288C_Ator08_hmg124h_13.JPG.dat	1164.3	31.7	1.0	0.03	-0.031	0.026	4.9E-06
YPL245W	YPL245W	S288C_Ator08_hmg124h_13.JPG.dat	1209.5	45.9	1.0	0.04	0.027	0.039	1.5E-05
YPL246C	YPL246C	S288C_Ator08_hmg124h_13.JPG.dat	1235.0	42.3	1.0	0.02	0.007	0.020	2.2E-06
YPL247C	YPL247C	S288C_Ator08_hmg124h_13.JPG.dat	1171.0	24.2	1.0	0.02	-0.064	0.015	1.0E-06
YPL248C	YPL248C	S288C_Ator08_hmg124h_13.JPG.dat	1197.3	38.0	1.0	0.02	0.013	0.022	2.9E-06
YPL249C	YPL249C	S288C_Ator08_hmg124h_13.JPG.dat	1083.0	93.5	0.9	0.09	-0.027	0.090	2.4E-04
YPL250C	YPL250C	S288C_Ator08_hmg124h_13.JPG.dat	1174.0	40.7	1.0	0.02	0.044	0.020	2.2E-06
YPL253C	YPL253C	S288C_Ator08_hmg124h_13.JPG.dat	1161.5	19.9	1.0	0.01	0.000	0.010	2.6E-07
YPL256C	YPL256C	S288C_Ator08_hmg124h_13.JPG.dat	1146.3	42.9	1.0	0.01	-0.052	0.008	2.0E-05
YPL257W	YPL257W	S288C_Ator08_hmg124h_13.JPG.dat	1172.0	16.1	1.0	0.00	-0.003	0.003	2.9E-06
YPL258C	YPL258C	S288C_Ator08_hmg124h_13.JPG.dat	1197.5	16.4	1.0	0.01	-0.018	0.006	1.2E-05
YPL259C	YPL259C	S288C_Ator08_hmg124h_13.JPG.dat	986.3	29.2	0.8	0.00	-0.144	0.004	9.3E-06
YPL260W	YPL260W	S288C_Ator08_hmg124h_13.JPG.dat	1206.5	29.9	1.0	0.03	0.022	0.026	5.1E-06
1	1	S288C_Ator08_hmg124h_10.JPG.dat	1336.8	173.4	1.0	0.12	0.010	0.116	3.7E-04
2	2	S288C_Ator08_hmg124h_10.JPG.dat	1119.0	68.1	0.9	0.04	-0.136	0.041	2.5E-05
3	3	S288C_Ator08_hmg124h_10.JPG.dat	1124.8	41.9	1.0	0.02	-0.034	0.024	4.1E-06
4	4	S288C_Ator08_hmg124h_10.JPG.dat	1166.3	45.9	1.0	0.04	-0.006	0.039	1.6E-05
YLR415C	YLR415C	S288C_Ator08_hmg124h_10.JPG.dat	1077.5	56.9	0.9	0.05	-0.054	0.053	4.7E-05
YLR416C	YLR416C	S288C_Ator08_hmg124h_10.JPG.dat	1185.8	27.4	1.0	0.02	0.020	0.020	2.1E-06
YLR418C	YLR418C	S288C_Ator08_hmg124h_10.JPG.dat	859.0	30.4	0.7	0.03	-0.001	0.025	1.2E-05
YLR420W	YLR420W	S288C_Ator08_hmg124h_10.JPG.dat	1239.0	15.2	1.0	0.01	0.028	0.013	5.4E-07
YLR421C	YLR421C	S288C_Ator08_hmg124h_10.JPG.dat	1155.3	84.8	1.0	0.07	-0.037	0.074	1.1E-04
YLR422W	YLR422W	S288C_Ator08_hmg124h_10.JPG.dat	1261.3	40.1	1.1	0.03	-0.001	0.034	8.6E-06
YLR423C	YLR423C	S288C_Ator08_hmg124h_10.JPG.dat	1170.8	85.8	1.0	0.01	0.016	0.013	5.6E-05
YLR425W	YLR425W	S288C_Ator08_hmg124h_10.JPG.dat	988.0	95.6	0.8	0.08	-0.169	0.083	2.7E-04
YLR426W	YLR426W	S288C_Ator08_hmg124h_10.JPG.dat	1223.3	26.4	1.0	0.02	0.034	0.023	3.3E-06
YLR427W	YLR427W	S288C_Ator08_hmg124h_10.JPG.dat	1178.8	17.9	1.0	0.02	0.014	0.016	1.0E-06
YLR428C	YLR428C	S288C_Ator08_hmg124h_10.JPG.dat	1227.8	49.5	1.1	0.05	0.067	0.052	3.0E-05
YLR429W	YLR429W	S288C_Ator08_hmg124h_10.JPG.dat	1031.5	65.9	0.9	0.06	-0.075	0.060	7.8E-05
YLR431C	YLR431C	S288C_Ator08_hmg124h_10.JPG.dat	1148.5	46.8	1.0	0.05	-0.018	0.046	2.6E-05
YLR432W	YLR432W	S288C_Ator08_hmg124h_10.JPG.dat	1144.0	41.3	1.0	0.03	-0.049	0.034	1.1E-05
YLR433C	YLR433C	S288C_Ator08_hmg124h_10.JPG.dat	1205.3	32.8	1.0	0.01	0.024	0.010	3.3E-05
YLR434C	YLR434C	S288C_Ator08_hmg124h_10.JPG.dat	1134.0	30.3	1.0	0.03	-0.006	0.029	6.3E-06
YLR435W	YLR435W	S288C_Ator08_hmg124h_10.JPG.dat	962.5	28.5	0.9	0.01	0.063	0.005	1.2E-05
YLR436C	YLR436C	S288C_Ator08_hmg124h_10.JPG.dat	1203.3	39.2	1.1	0.03	0.062	0.033	8.8E-06
YLR437C	YLR437C	S288C_Ator08_hmg124h_10.JPG.dat	1253.3	77.9	1.1	0.07	0.044	0.066	6.4E-05
YLR438W	YLR438W	S288C_Ator08_hmg124h_10.JPG.dat	1255.5	3.7	1.1	0.01	0.016	0.006	5.7E-08
YLR441C	YLR441C	S288C_Ator08_hmg124h_10.JPG.dat	925.0	69.5	0.8	0.06	0.017	0.061	1.2E-04
YLR442C	YLR442C	S288C_Ator08_hmg124h_10.JPG.dat	0.0	0.0	0.0	0.00	0.000	0.000	
YLR443W	YLR443W	S288C_Ator08_hmg124h_10.JPG.dat	1043.3	53.8	0.9	0.05	-0.102	0.047	3.5E-05
YLR444C	YLR444C	S288C_Ator08_hmg124h_10.JPG.dat	1188.8	52.4	1.1	0.05	0.007	0.052	3.3E-05
YLR445W	YLR445W	S288C_Ator08_hmg124h_10.JPG.dat	1084.5	100.9	1.0	0.03	-0.023	0.034	3.8E-04
YLR446W	YLR446W	S288C_Ator08_hmg124h_10.JPG.dat	1098.0	21.4	1.0	0.02	-0.013	0.020	2.1E-06
YLR448W	YLR448W	S288C_Ator08_hmg124h_10.JPG.dat	1044.5	22.5	1.0	0.02	0.070	0.019	2.1E-06
YLR449W	YLR449W	S288C_Ator08_hmg124h_10.JPG.dat	845.8	87.9	0.8	0.08	-0.141	0.084	3.4E-04
YLR450W	YLR450W	S288C_Ator08_hmg124h_10.JPG.dat	0.0	0.0	0.0	0.00	0.000	0.000	
YLR451W	YLR451W	S288C_Ator08_hmg124h_10.JPG.dat	1031.5	22.4	0.9	0.02	-0.111	0.018	2.2E-06
YLR452C	YLR452C	S288C_Ator08_hmg124h_10.JPG.dat	1075.5	36.4	0.9	0.03	0.116	0.028	7.8E-06
YLR453C	YLR453C	S288C_Ator08_hmg124h_10.JPG.dat	1153.8	27.2	1.0	0.03	0.015	0.027	5.2E-06
YLR454W	YLR454W	S288C_Ator08_hmg124h_10.JPG.dat	1025.3	39.6	0.9	0.01	-0.131	0.011	4.3E-05
YLR455W	YLR455W	S288C_Ator08_hmg124h_10.JPG.dat	1109.0	76.4	1.0	0.07	-0.057	0.072	1.0E-04
YLR456W	YLR456W	S288C_Ator08_hmg124h_10.JPG.dat	1096.3	79.5	1.0	0.07	-0.019	0.072	1.1E-04
YLR460C	YLR460C	S288C_Ator08_hmg124h_10.JPG.dat	1082.8	59.3	1.0	0.06	-0.049	0.055	5.0E-05
YLR461W	YLR461W	S288C_Ator08_hmg124h_10.JPG.dat	1006.8	47.0	0.9	0.04	-0.071	0.044	3.0E-05
YML001W	YML001W	S288C_Ator08_hmg124h_10.JPG.dat	1010.5	30.9	0.9	0.02	0.008	0.023	4.3E-06
YML002W	YML002W	S288C_Ator08_hmg124h_10.JPG.dat	1124.0	28.3	1.0	0.02	0.053	0.025	3.8E-06
YML003W	YML003W	S288C_Ator08_hmg124h_10.JPG.dat	1112.5	8.7	1.0	0.01	0.006	0.009	2.1E-07
YML004C	YML004C	S288C_Ator08_hmg124h_10.JPG.dat	1125.5	14.2	1.0	0.01	0.014	0.010	2.8E-07
YML005W	YML005W	S288C_Ator08_hmg124h_10.JPG.dat	1135.3	20.6	1.0	0.02	-0.012	0.020	2.1E-06
YML006C	YML006C	S288C_Ator08_hmg124h_10.JPG.dat	1138.0	64.8	1.0	0.06	0.031	0.058	4.8E-05
YML007W	YML007W	S288C_Ator08_hmg124h_10.JPG.dat	1113.3	61.0	1.1	0.06	0.109	0.065	6.4E-05
YML008C	YML008C	S288C_Ator08_hmg124h_10.JPG.dat	1182.8	10.4	1.0	0.01	0.034	0.012	4.4E-07
YML009c	YML009c	S288C_Ator08_hmg124h_10.JPG.dat	1146.5	43.5	1.0	0.03	-0.014	0.034	1.1E-05
YML010C-I	YML010C-B	S288C_Ator08_hmg124h_10.JPG.dat	1141.3	34.2	1.0	0.02	0.043	0.024	4.0E-06
YML011C	YML011C	S288C_Ator08_hmg124h_10.JPG.dat	1129.0	18.2	1.0	0.02	0.030	0.019	2.0E-06
YML012W	YML012W	S288C_Ator08_hmg124h_10.JPG.dat	1194.8	34.2	1.1	0.03	0.033	0.027	4.4E-06
YML013C-/	YML013C-A	S288C_Ator08_hmg124h_10.JPG.dat	1074.3	69.9	1.0	0.06	-0.033	0.064	7.8E-05
YML013W	YML013W	S288C_Ator08_hmg124h_10.JPG.dat	1055.8	24.1	0.9	0.02	-0.040	0.023	3.8E-06
YML016C	YML016C	S288C_Ator08_hmg124h_10.JPG.dat	1178.8	31.9	1.0	0.03	-0.018	0.030	6.6E-06

YML017W	YML017W	S288C_Ator08_hmg124h_10.JPG.dat	1011.3	327.7	1.0	0.07	-0.092	0.070	1.5E-03
YML018C	YML018C	S288C_Ator08_hmg124h_10.JPG.dat	1134.0	23.5	1.0	0.02	-0.028	0.020	2.0E-06
YML019W	YML019W	S288C_Ator08_hmg124h_10.JPG.dat	1055.0	9.2	1.0	0.00	-0.011	0.004	1.6E-08
YML020W	YML020W	S288C_Ator08_hmg124h_10.JPG.dat	1215.3	36.9	1.1	0.03	0.052	0.026	4.2E-06
YML021C	YML021C	S288C_Ator08_hmg124h_10.JPG.dat	1092.5	113.8	0.9	0.10	-0.028	0.103	3.5E-04
YML022W	YML022W	S288C_Ator08_hmg124h_10.JPG.dat	1113.3	152.6	1.0	0.04	0.124	0.040	4.9E-04
YML026C	YML026C	S288C_Ator08_hmg124h_10.JPG.dat	883.3	26.9	0.8	0.03	0.014	0.027	1.1E-05
YML027W	YML027W	S288C_Ator08_hmg124h_10.JPG.dat	1073.0	46.9	1.0	0.04	-0.008	0.043	2.4E-05
YML028W	YML028W	S288C_Ator08_hmg124h_10.JPG.dat	937.3	57.5	0.9	0.05	-0.073	0.049	5.4E-05
YML029W	YML029W	S288C_Ator08_hmg124h_10.JPG.dat	1103.8	62.8	1.0	0.04	-0.062	0.044	2.4E-05
YML030W	YML030W	S288C_Ator08_hmg124h_10.JPG.dat	1120.5	11.3	1.0	0.01	0.026	0.011	3.5E-07
YML032C	YML032C	S288C_Ator08_hmg124h_10.JPG.dat	948.8	11.1	0.9	0.01	0.016	0.005	1.4E-05
YML033W	YML033W	S288C_Ator08_hmg124h_10.JPG.dat	1087.0	55.5	1.0	0.02	0.027	0.016	8.7E-05
YML034W	YML034W	S288C_Ator08_hmg124h_10.JPG.dat	1047.5	50.2	1.0	0.05	0.030	0.050	3.6E-05
YML035C	YML035C	S288C_Ator08_hmg124h_10.JPG.dat	795.5	49.9	0.7	0.01	0.009	0.009	5.8E-05
YML035C-/	YML035C-A	S288C_Ator08_hmg124h_10.JPG.dat	1184.5	33.8	1.0	0.03	0.029	0.026	4.5E-06
YML036W	YML036W	S288C_Ator08_hmg124h_10.JPG.dat	1123.0	61.3	1.0	0.05	-0.009	0.050	3.8E-05
YML037C	YML037C	S288C_Ator08_hmg124h_10.JPG.dat	1045.0	40.8	0.9	0.04	-0.093	0.035	1.6E-05
YML038C	YML038C	S288C_Ator08_hmg124h_10.JPG.dat	1055.5	15.7	0.9	0.00	-0.067	0.004	4.9E-06
YML041C	YML041C	S288C_Ator08_hmg124h_10.JPG.dat	1109.5	60.4	1.0	0.05	0.025	0.053	4.5E-05
YML042W	YML042W	S288C_Ator08_hmg124h_10.JPG.dat	1154.0	95.1	1.0	0.03	0.009	0.030	2.7E-04
YML047C	YML047C	S288C_Ator08_hmg124h_10.JPG.dat	999.3	95.9	0.8	0.02	-0.145	0.024	2.7E-04
YML048W	YML048W	S288C_Ator08_hmg124h_10.JPG.dat	975.3	124.4	0.9	0.11	-0.136	0.111	5.8E-04
YML048W·	YML048W-A	S288C_Ator08_hmg124h_10.JPG.dat	1083.3	114.4	1.0	0.10	-0.021	0.104	3.2E-04
YML051W	YML051W	S288C_Ator08_hmg124h_10.JPG.dat	1009.5	44.8	0.9	0.05	0.007	0.045	3.1E-05
YML052W	YML052W	S288C_Ator08_hmg124h_10.JPG.dat	915.3	31.8	0.8	0.03	-0.155	0.033	1.7E-05
YML053C	YML053C	S288C_Ator08_hmg124h_10.JPG.dat	1115.0	17.2	1.0	0.01	-0.003	0.015	9.3E-07
YML054C	YML054C	S288C_Ator08_hmg124h_10.JPG.dat	1140.3	60.8	1.0	0.05	-0.005	0.050	3.7E-05
YML055W	YML055W	S288C_Ator08_hmg124h_10.JPG.dat	1098.8	29.8	0.9	0.03	0.014	0.026	6.1E-06
YML056C	YML056C	S288C_Ator08_hmg124h_10.JPG.dat	1174.5	21.3	1.0	0.01	0.047	0.014	8.3E-07
YML057W	YML057W	S288C_Ator08_hmg124h_10.JPG.dat	1025.0	138.8	0.9	0.12	-0.124	0.118	6.7E-04
YML058C-/	YML058C-A	S288C_Ator08_hmg124h_10.JPG.dat	884.8	590.2	1.0	0.02	0.002	0.021	1.5E-04
YML058W	YML058W	S288C_Ator08_hmg124h_10.JPG.dat	1078.5	200.2	1.0	0.02	-0.053	0.020	1.4E-04
YML059C	YML059C	S288C_Ator08_hmg124h_10.JPG.dat	1190.0	92.9	1.0	0.08	-0.074	0.082	1.5E-04
YML060W	YML060W	S288C_Ator08_hmg124h_10.JPG.dat	1126.5	95.7	0.9	0.08	-0.057	0.080	1.7E-04
YML062C	YML062C	S288C_Ator08_hmg124h_10.JPG.dat	1226.5	14.4	1.1	0.02	0.071	0.019	1.6E-06
YML063W	YML063W	S288C_Ator08_hmg124h_10.JPG.dat	449.8	533.1	0.4	0.48	0.055	0.478	1.9E-01
YML066C	YML066C	S288C_Ator08_hmg124h_10.JPG.dat	1030.0	56.4	0.9	0.01	-0.074	0.006	1.7E-05
YML067C	YML067C	S288C_Ator08_hmg124h_10.JPG.dat	1189.0	9.6	1.0	0.00	0.089	0.004	6.0E-06
YML068W	YML068W	S288C_Ator08_hmg124h_10.JPG.dat	838.5	565.6	1.0	0.09	-0.035	0.090	2.9E-03
YML070W	YML070W	S288C_Ator08_hmg124h_10.JPG.dat	866.8	578.0	1.0	0.02	0.037	0.019	1.2E-04
YML071C	YML071C	S288C_Ator08_hmg124h_10.JPG.dat	789.3	526.7	0.9	0.02	-0.103	0.025	2.4E-04
YML072C	YML072C	S288C_Ator08_hmg124h_10.JPG.dat	627.3	724.3	0.6	0.64	0.037	0.637	1.8E-01
YML074C	YML074C	S288C_Ator08_hmg124h_10.JPG.dat	616.0	712.2	0.5	0.61	0.048	0.613	1.8E-01
YML075C	YML075C	S288C_Ator08_hmg124h_10.JPG.dat	313.5	627.0	0.0	0.00	0.000	0.000	
YML076C	YML076C	S288C_Ator08_hmg124h_10.JPG.dat	568.3	662.8	0.5	0.56	0.073	0.562	1.8E-01
YML081C-/	YML081C-A	S288C_Ator08_hmg124h_10.JPG.dat	808.0	558.2	0.9	0.15	-0.011	0.151	8.3E-03
YML090W	YML090W	S288C_Ator08_hmg124h_10.JPG.dat	1048.0	24.9	1.0	0.03	0.117	0.033	1.1E-05
YML094W	YML094W	S288C_Ator08_hmg124h_10.JPG.dat	296.5	593.0	0.0	0.00	0.000	0.000	
YML095C	YML095C	S288C_Ator08_hmg124h_10.JPG.dat	1174.5	28.5	1.0	0.01	0.018	0.010	3.3E-05
YML096W	YML096W	S288C_Ator08_hmg124h_10.JPG.dat	1140.3	30.0	1.0	0.03	0.007	0.027	5.1E-06
YML097C	YML097C	S288C_Ator08_hmg124h_10.JPG.dat	930.8	149.1	0.8	0.05	-0.150	0.052	1.5E-03
YML099C	YML099C	S288C_Ator08_hmg124h_10.JPG.dat	1109.3	28.3	1.0	0.01	0.035	0.009	2.6E-05
YML100W	YML100W	S288C_Ator08_hmg124h_10.JPG.dat	1084.3	94.1	1.0	0.09	-0.017	0.091	2.1E-04
YML100W·	YML100W-A	S288C_Ator08_hmg124h_10.JPG.dat	1144.3	82.6	1.0	0.08	0.008	0.075	1.1E-04
YML101C	YML101C	S288C_Ator08_hmg124h_10.JPG.dat	1184.0	13.0	1.0	0.01	0.059	0.013	5.5E-07
YML102C-/	YML102C-A	S288C_Ator08_hmg124h_10.JPG.dat	1203.3	36.5	1.0	0.01	0.115	0.010	3.4E-05
YML102W	YML102W	S288C_Ator08_hmg124h_10.JPG.dat	1138.0	32.6	1.0	0.03	0.023	0.026	4.6E-06
YML103C	YML103C	S288C_Ator08_hmg124h_10.JPG.dat	1080.0	19.1	1.0	0.01	0.003	0.012	4.5E-07
YML104C	YML104C	S288C_Ator08_hmg124h_10.JPG.dat	1065.8	78.4	1.0	0.07	-0.010	0.071	1.1E-04
YML106W	YML106W	S288C_Ator08_hmg124h_10.JPG.dat	1090.3	22.1	1.0	0.02	0.032	0.019	2.0E-06
YML107C	YML107C	S288C_Ator08_hmg124h_10.JPG.dat	1049.8	27.8	1.0	0.01	0.081	0.008	2.2E-05
YML108W	YML108W	S288C_Ator08_hmg124h_10.JPG.dat	1086.5	25.6	1.0	0.02	0.027	0.018	1.6E-06
YML109W	YML109W	S288C_Ator08_hmg124h_10.JPG.dat	1081.5	36.1	1.0	0.03	-0.065	0.029	6.7E-06
YML113W	YML113W	S288C_Ator08_hmg124h_10.JPG.dat	1097.8	38.1	1.0	0.03	-0.026	0.030	7.5E-06
YML116W	YML116W	S288C_Ator08_hmg124h_10.JPG.dat	1129.8	21.3	1.0	0.02	0.002	0.017	1.3E-06
YML117W	YML117W	S288C_Ator08_hmg124h_10.JPG.dat	1185.5	49.3	1.1	0.05	0.014	0.049	2.6E-05
YML117W·	YML117W-A	S288C_Ator08_hmg124h_10.JPG.dat	1113.3	78.1	1.0	0.07	-0.064	0.074	1.1E-04
YML118W	YML118W	S288C_Ator08_hmg124h_10.JPG.dat	1105.8	18.5	1.0	0.01	0.005	0.013	6.1E-07
YML119W	YML119W	S288C_Ator08_hmg124h_10.JPG.dat	1133.5	68.8	1.0	0.05	-0.038	0.050	3.2E-05

YML120C	YML120C	S288C_Ator08_hmg124h_10.JPG.dat	1206.3	22.3	1.0	0.02	0.012	0.020	1.9E-06
YML121W	YML121W	S288C_Ator08_hmg124h_10.JPG.dat	1055.8	21.0	0.9	0.02	0.041	0.021	3.2E-06
YML122C	YML122C	S288C_Ator08_hmg124h_10.JPG.dat	1097.5	32.1	1.0	0.03	0.032	0.027	5.9E-06
YML123C	YML123C	S288C_Ator08_hmg124h_10.JPG.dat	1088.8	56.5	1.0	0.05	0.037	0.047	3.0E-05
YML124C	YML124C	S288C_Ator08_hmg124h_10.JPG.dat	1157.8	15.7	1.0	0.01	0.027	0.014	7.5E-07
YML128C	YML128C	S288C_Ator08_hmg124h_10.JPG.dat	1047.5	128.2	0.9	0.11	-0.038	0.113	4.9E-04
YML131W	YML131W	S288C_Ator08_hmg124h_10.JPG.dat	1126.3	24.6	1.0	0.02	0.014	0.022	2.8E-06
YMR002W	YMR002W	S288C_Ator08_hmg124h_10.JPG.dat	1157.8	24.6	1.0	0.02	0.015	0.022	2.6E-06
YMR003W	YMR003W	S288C_Ator08_hmg124h_10.JPG.dat	1129.3	27.1	1.0	0.02	-0.025	0.024	3.8E-06
YMR004W	YMR004W	S288C_Ator08_hmg124h_10.JPG.dat	1060.3	42.3	1.0	0.04	-0.056	0.039	1.7E-05
YMR006C	YMR006C	S288C_Ator08_hmg124h_10.JPG.dat	1138.0	31.6	1.0	0.03	-0.086	0.030	7.0E-06
YMR007W	YMR007W	S288C_Ator08_hmg124h_10.JPG.dat	1158.5	13.2	1.0	0.02	0.019	0.020	2.3E-06
YMR008C	YMR008C	S288C_Ator08_hmg124h_10.JPG.dat	1199.8	21.9	1.0	0.01	0.010	0.015	7.7E-07
YMR009W	YMR009W	S288C_Ator08_hmg124h_10.JPG.dat	1173.0	26.2	1.0	0.02	0.041	0.017	1.1E-06
YMR010W	YMR010W	S288C_Ator08_hmg124h_10.JPG.dat	930.0	29.1	0.8	0.02	-0.143	0.017	2.4E-06
YMR011W	YMR011W	S288C_Ator08_hmg124h_10.JPG.dat	1117.3	30.2	1.0	0.01	0.001	0.012	4.3E-05
YMR012W	YMR012W	S288C_Ator08_hmg124h_10.JPG.dat	1142.0	26.9	1.0	0.03	0.013	0.026	4.8E-06
YMR015C	YMR015C	S288C_Ator08_hmg124h_10.JPG.dat	1145.0	41.9	1.0	0.03	-0.033	0.032	8.4E-06
YMR016C	YMR016C	S288C_Ator08_hmg124h_10.JPG.dat	935.0	36.4	0.8	0.04	-0.178	0.041	3.1E-05
YMR017W	YMR017W	S288C_Ator08_hmg124h_10.JPG.dat	1117.0	21.6	1.0	0.02	-0.018	0.025	4.0E-06
YMR018W	YMR018W	S288C_Ator08_hmg124h_10.JPG.dat	1121.5	51.6	1.0	0.05	0.003	0.050	3.0E-05
YMR019W	YMR019W	S288C_Ator08_hmg124h_10.JPG.dat	1153.5	34.0	1.0	0.03	0.018	0.027	5.3E-06
YMR020W	YMR020W	S288C_Ator08_hmg124h_10.JPG.dat	1253.5	41.7	1.1	0.04	0.074	0.037	1.1E-05
YMR021C	YMR021C	S288C_Ator08_hmg124h_10.JPG.dat	1104.8	69.1	1.0	0.05	-0.087	0.055	5.1E-05
YMR022W	YMR022W	S288C_Ator08_hmg124h_10.JPG.dat	1135.5	41.8	1.0	0.01	-0.042	0.007	1.8E-05
YMR023C	YMR023C	S288C_Ator08_hmg124h_10.JPG.dat	1210.3	67.7	1.1	0.02	0.077	0.025	1.7E-04
YMR025W	YMR025W	S288C_Ator08_hmg124h_10.JPG.dat	1242.8	19.9	1.1	0.02	0.092	0.018	1.2E-06
YMR026C	YMR026C	S288C_Ator08_hmg124h_10.JPG.dat	1235.8	16.4	1.1	0.02	0.091	0.015	7.5E-07
YMR027W	YMR027W	S288C_Ator08_hmg124h_10.JPG.dat	1170.3	30.8	1.0	0.03	0.034	0.027	5.2E-06
YMR029C	YMR029C	S288C_Ator08_hmg124h_10.JPG.dat	1134.5	23.1	1.0	0.01	-0.007	0.006	1.3E-05
YMR030W	YMR030W	S288C_Ator08_hmg124h_10.JPG.dat	1178.8	30.8	1.1	0.01	0.038	0.007	1.6E-05
YMR031C	YMR031C	S288C_Ator08_hmg124h_10.JPG.dat	989.8	59.1	0.9	0.05	-0.092	0.050	4.6E-05
YMR031W	YMR031W-A	S288C_Ator08_hmg124h_10.JPG.dat	359.0	414.6	0.3	0.36	-0.006	0.360	1.8E-01
YMR034C	YMR034C	S288C_Ator08_hmg124h_10.JPG.dat	1152.3	37.0	1.0	0.03	0.009	0.028	6.8E-06
YMR035W	YMR035W	S288C_Ator08_hmg124h_10.JPG.dat	1155.3	12.7	1.0	0.01	0.033	0.015	1.0E-06
YMR036C	YMR036C	S288C_Ator08_hmg124h_10.JPG.dat	1282.3	30.2	1.1	0.02	0.038	0.019	1.5E-06
YMR037C	YMR037C	S288C_Ator08_hmg124h_10.JPG.dat	1173.0	44.9	1.0	0.00	0.038	0.003	4.0E-06
YMR038C	YMR038C	S288C_Ator08_hmg124h_10.JPG.dat	0.0	0.0	0.0	0.00	0.000	0.000	
YMR039C	YMR039C	S288C_Ator08_hmg124h_10.JPG.dat	1382.3	75.9	1.1	0.07	0.144	0.068	5.8E-05
YMR040W	YMR040W	S288C_Ator08_hmg124h_10.JPG.dat	1212.3	53.4	1.0	0.04	0.034	0.038	1.5E-05
YMR041C	YMR041C	S288C_Ator08_hmg124h_10.JPG.dat	1211.8	86.9	1.0	0.06	0.000	0.064	7.5E-05
YMR042W	YMR042W	S288C_Ator08_hmg124h_10.JPG.dat	1200.3	30.4	1.0	0.03	0.030	0.029	6.5E-06
YMR044W	YMR044W	S288C_Ator08_hmg124h_10.JPG.dat	1174.5	25.0	1.0	0.01	0.010	0.007	1.6E-05
YMR048W	YMR048W	S288C_Ator08_hmg124h_10.JPG.dat	1141.5	28.7	1.0	0.03	0.029	0.027	5.4E-06
YMR052C-.	YMR052C-A	S288C_Ator08_hmg124h_10.JPG.dat	1188.0	27.4	1.0	0.00	0.012	0.002	1.8E-06
YMR052W	YMR052W	S288C_Ator08_hmg124h_10.JPG.dat	1221.3	28.1	1.0	0.02	0.017	0.022	2.8E-06
YMR053C	YMR053C	S288C_Ator08_hmg124h_10.JPG.dat	1183.8	29.1	1.0	0.01	-0.019	0.007	1.5E-05
YMR054W	YMR054W	S288C_Ator08_hmg124h_10.JPG.dat	1133.3	27.0	1.0	0.01	-0.043	0.005	9.6E-06
YMR055C	YMR055C	S288C_Ator08_hmg124h_10.JPG.dat	1213.0	29.1	1.0	0.02	0.031	0.024	3.2E-06
YMR056C	YMR056C	S288C_Ator08_hmg124h_10.JPG.dat	1188.3	26.6	1.0	0.02	0.003	0.020	2.2E-06
YMR057C	YMR057C	S288C_Ator08_hmg124h_10.JPG.dat	1244.0	20.0	1.0	0.02	0.027	0.016	9.9E-07
YMR058W	YMR058W	S288C_Ator08_hmg124h_10.JPG.dat	1283.3	26.2	1.1	0.02	0.062	0.020	2.0E-06
YMR060C	YMR060C	S288C_Ator08_hmg124h_10.JPG.dat	1021.8	49.1	0.9	0.04	-0.027	0.044	3.5E-05
YMR063W	YMR063W	S288C_Ator08_hmg124h_10.JPG.dat	1008.8	26.7	0.9	0.03	-0.116	0.030	1.1E-05
YMR065W	YMR065W	S288C_Ator08_hmg124h_10.JPG.dat	1174.0	55.3	1.1	0.01	0.061	0.010	3.0E-05
YMR067C	YMR067C	S288C_Ator08_hmg124h_10.JPG.dat	1192.8	19.0	1.1	0.00	0.026	0.003	2.4E-06
YMR068W	YMR068W	S288C_Ator08_hmg124h_10.JPG.dat	1102.8	82.4	0.9	0.07	-0.089	0.066	9.2E-05
YMR070W	YMR070W	S288C_Ator08_hmg124h_10.JPG.dat	1164.3	12.3	1.0	0.02	0.050	0.020	2.0E-06
YMR073C	YMR073C	S288C_Ator08_hmg124h_10.JPG.dat	1088.0	33.4	1.0	0.01	0.067	0.012	4.6E-05
YMR075C-.	YMR075C-A	S288C_Ator08_hmg124h_10.JPG.dat	1023.0	44.4	0.9	0.04	-0.024	0.036	1.6E-05
YMR075W	YMR075W	S288C_Ator08_hmg124h_10.JPG.dat	1051.0	29.7	0.9	0.02	-0.009	0.020	2.7E-06
YMR078C	YMR078C	S288C_Ator08_hmg124h_10.JPG.dat	1009.8	27.1	0.9	0.02	-0.080	0.024	5.9E-06
YMR080C	YMR080C	S288C_Ator08_hmg124h_10.JPG.dat	1309.0	44.3	1.1	0.03	0.074	0.029	4.9E-06
YMR085W	YMR085W	S288C_Ator08_hmg124h_10.JPG.dat	1079.0	98.2	0.9	0.09	-0.092	0.092	2.6E-04
YMR086C-.	YMR086C-A	S288C_Ator08_hmg124h_10.JPG.dat	1221.0	33.5	1.1	0.01	0.027	0.010	2.5E-05
YMR086W	YMR086W	S288C_Ator08_hmg124h_10.JPG.dat	1181.3	20.8	1.0	0.02	0.009	0.017	1.1E-06
YMR087W	YMR087W	S288C_Ator08_hmg124h_10.JPG.dat	1132.0	28.7	1.0	0.03	-0.014	0.025	4.6E-06
YMR088C	YMR088C	S288C_Ator08_hmg124h_10.JPG.dat	890.5	593.7	1.0	0.02	0.016	0.020	1.2E-04
YMR092C	YMR092C	S288C_Ator08_hmg124h_10.JPG.dat	1043.5	51.4	0.9	0.04	-0.020	0.040	2.2E-05
YMR095C	YMR095C	S288C_Ator08_hmg124h_10.JPG.dat	1138.8	16.9	1.0	0.00	0.030	0.001	6.6E-07

YMR096W	YMR096W	S288C_Ator08_hmg124h_10.JPG.dat	1144.8	58.4	1.0	0.05	-0.001	0.054	3.8E-05
YMR099C	YMR099C	S288C_Ator08_hmg124h_10.JPG.dat	1154.5	29.2	1.0	0.02	-0.001	0.022	2.7E-06
YMR100W	YMR100W	S288C_Ator08_hmg124h_10.JPG.dat	1212.3	29.5	1.1	0.01	0.143	0.011	3.6E-05
YMR101C	YMR101C	S288C_Ator08_hmg124h_10.JPG.dat	1150.0	22.1	1.0	0.01	0.011	0.012	5.2E-07
YMR102C	YMR102C	S288C_Ator08_hmg124h_10.JPG.dat	1124.0	67.2	1.0	0.06	-0.015	0.058	5.5E-05
YMR103C	YMR103C	S288C_Ator08_hmg124h_10.JPG.dat	1070.8	41.0	1.0	0.04	-0.021	0.045	2.6E-05
YMR104C	YMR104C	S288C_Ator08_hmg124h_10.JPG.dat	1152.0	49.9	1.0	0.04	-0.014	0.041	1.8E-05
YMR105C	YMR105C	S288C_Ator08_hmg124h_10.JPG.dat	1116.3	25.0	1.0	0.02	0.053	0.022	3.2E-06
YMR106C	YMR106C	S288C_Ator08_hmg124h_10.JPG.dat	1203.3	24.1	1.1	0.02	0.000	0.017	1.1E-06
YMR107W	YMR107W	S288C_Ator08_hmg124h_10.JPG.dat	1144.3	26.1	1.0	0.02	-0.005	0.024	3.6E-06
YMR109W	YMR109W	S288C_Ator08_hmg124h_10.JPG.dat	1107.8	17.4	1.0	0.02	-0.042	0.016	1.1E-06
YMR110C	YMR110C	S288C_Ator08_hmg124h_10.JPG.dat	1119.3	29.4	1.0	0.03	-0.010	0.025	4.3E-06
YMR111C	YMR111C	S288C_Ator08_hmg124h_10.JPG.dat	1157.3	17.3	1.0	0.00	0.029	0.004	4.6E-06
YMR114C	YMR114C	S288C_Ator08_hmg124h_10.JPG.dat	1148.8	21.2	1.0	0.02	0.034	0.016	1.2E-06
YMR115W	YMR115W	S288C_Ator08_hmg124h_10.JPG.dat	1109.8	28.9	1.0	0.01	-0.009	0.009	2.5E-05
YMR116C	YMR116C	S288C_Ator08_hmg124h_10.JPG.dat	444.8	552.0	0.4	0.50	0.158	0.503	2.1E-01
YMR119W	YMR119W	S288C_Ator08_hmg124h_10.JPG.dat	1052.8	40.2	1.0	0.03	-0.048	0.034	1.1E-05
YMR119W	YMR119W-A	S288C_Ator08_hmg124h_10.JPG.dat	1149.5	20.4	1.0	0.02	0.007	0.020	2.4E-06
YMR120C	YMR120C	S288C_Ator08_hmg124h_10.JPG.dat	1107.8	97.2	1.0	0.08	-0.045	0.085	1.9E-04
YMR121C	YMR121C	S288C_Ator08_hmg124h_10.JPG.dat	1167.8	31.4	1.0	0.03	-0.001	0.028	6.0E-06
YMR122C	YMR122C	S288C_Ator08_hmg124h_10.JPG.dat	1097.3	19.3	1.0	0.02	-0.008	0.015	1.1E-06
YMR123W	YMR123W	S288C_Ator08_hmg124h_10.JPG.dat	737.3	15.5	0.7	0.01	-0.136	0.015	3.0E-06
YMR124W	YMR124W	S288C_Ator08_hmg124h_10.JPG.dat	1101.0	29.0	1.0	0.03	-0.019	0.027	5.3E-06
YMR126C	YMR126C	S288C_Ator08_hmg124h_10.JPG.dat	1051.8	73.0	0.9	0.06	-0.051	0.063	8.6E-05
YMR127C	YMR127C	S288C_Ator08_hmg124h_10.JPG.dat	1114.0	36.1	1.0	0.03	-0.005	0.032	9.7E-06
YMR129W	YMR129W	S288C_Ator08_hmg124h_10.JPG.dat	1171.8	23.9	1.0	0.02	0.039	0.020	2.0E-06
YMR130W	YMR130W	S288C_Ator08_hmg124h_10.JPG.dat	1066.8	88.0	1.0	0.01	-0.034	0.007	1.8E-05
YMR132C	YMR132C	S288C_Ator08_hmg124h_10.JPG.dat	1099.5	45.2	1.0	0.05	0.004	0.047	2.7E-05
YMR133W	YMR133W	S288C_Ator08_hmg124h_10.JPG.dat	1212.5	19.1	1.0	0.02	0.007	0.019	1.9E-06
YMR135C	YMR135C	S288C_Ator08_hmg124h_10.JPG.dat	1206.3	46.6	1.0	0.01	0.041	0.014	6.0E-05
YMR135W	YMR135W-A	S288C_Ator08_hmg124h_10.JPG.dat	1225.0	49.9	1.0	0.03	0.002	0.035	1.0E-05
YMR136W	YMR136W	S288C_Ator08_hmg124h_10.JPG.dat	1183.8	32.9	1.0	0.01	0.021	0.007	1.5E-05
YMR137C	YMR137C	S288C_Ator08_hmg124h_10.JPG.dat	1117.0	40.0	1.0	0.04	-0.029	0.040	1.9E-05
YMR138W	YMR138W	S288C_Ator08_hmg124h_10.JPG.dat	1037.3	71.4	0.9	0.06	-0.073	0.062	8.9E-05
YMR139W	YMR139W	S288C_Ator08_hmg124h_10.JPG.dat	1047.8	64.2	0.9	0.05	-0.096	0.050	4.7E-05
YMR140W	YMR140W	S288C_Ator08_hmg124h_10.JPG.dat	1185.8	23.5	1.0	0.03	-0.017	0.028	5.7E-06
YMR141C	YMR141C	S288C_Ator08_hmg124h_10.JPG.dat	910.5	608.1	1.1	0.04	0.047	0.044	5.9E-04
YMR143W	YMR143W	S288C_Ator08_hmg124h_10.JPG.dat	1135.8	33.1	1.0	0.03	-0.018	0.034	1.0E-05
YMR144W	YMR144W	S288C_Ator08_hmg124h_10.JPG.dat	1019.0	95.1	0.9	0.09	-0.115	0.086	2.1E-04
YMR145C	YMR145C	S288C_Ator08_hmg124h_10.JPG.dat	1144.5	63.6	1.0	0.02	-0.013	0.015	7.9E-05
YMR147W	YMR147W	S288C_Ator08_hmg124h_10.JPG.dat	1084.0	34.2	0.9	0.01	-0.069	0.010	3.7E-05
YMR148W	YMR148W	S288C_Ator08_hmg124h_10.JPG.dat	1190.5	12.1	1.0	0.01	0.028	0.012	5.1E-07
YMR152W	YMR152W	S288C_Ator08_hmg124h_10.JPG.dat	1179.3	16.1	1.0	0.02	-0.026	0.018	1.8E-06
YMR153C-.	YMR153C-A	S288C_Ator08_hmg124h_10.JPG.dat	1234.8	40.0	1.0	0.01	0.029	0.012	4.6E-05
YMR153W	YMR153W	S288C_Ator08_hmg124h_10.JPG.dat	1288.3	38.0	1.1	0.03	0.040	0.031	6.7E-06
YMR154C	YMR154C	S288C_Ator08_hmg124h_10.JPG.dat	999.3	71.3	0.8	0.06	-0.189	0.063	1.2E-04
YMR155W	YMR155W	S288C_Ator08_hmg124h_10.JPG.dat	1136.3	103.3	1.0	0.02	-0.043	0.024	2.0E-04
YMR156C	YMR156C	S288C_Ator08_hmg124h_10.JPG.dat	1215.0	45.1	1.0	0.04	-0.039	0.044	2.2E-05
YMR157C	YMR157C	S288C_Ator08_hmg124h_10.JPG.dat	1160.0	59.5	1.0	0.05	-0.051	0.045	2.9E-05
YMR158C-	YMR158C-B	S288C_Ator08_hmg124h_10.JPG.dat	1228.0	20.5	1.0	0.00	-0.010	0.003	3.7E-06
YMR158W	YMR158W-A	S288C_Ator08_hmg124h_10.JPG.dat	1121.8	8.1	1.0	0.01	0.046	0.007	1.1E-07
YMR159C	YMR159C	S288C_Ator08_hmg124h_10.JPG.dat	1140.0	24.9	1.0	0.02	-0.034	0.025	4.3E-06
YMR160W	YMR160W	S288C_Ator08_hmg124h_10.JPG.dat	1227.5	26.1	1.1	0.01	0.040	0.007	1.4E-05
YMR161W	YMR161W	S288C_Ator08_hmg124h_10.JPG.dat	1189.8	22.5	1.0	0.02	-0.006	0.016	1.2E-06
YMR162C	YMR162C	S288C_Ator08_hmg124h_10.JPG.dat	1210.5	19.4	1.0	0.02	-0.065	0.023	3.1E-06
YMR163C	YMR163C	S288C_Ator08_hmg124h_10.JPG.dat	1037.8	29.8	0.9	0.02	-0.033	0.025	6.0E-06
YMR164C	YMR164C	S288C_Ator08_hmg124h_10.JPG.dat	1184.5	38.1	1.0	0.03	-0.026	0.035	1.1E-05
YMR166C	YMR166C	S288C_Ator08_hmg124h_10.JPG.dat	1111.3	105.4	0.9	0.09	-0.048	0.087	2.2E-04
YMR167W	YMR167W	S288C_Ator08_hmg124h_10.JPG.dat	985.5	102.1	0.9	0.02	-0.104	0.019	1.6E-04
YMR169c	YMR169c	S288C_Ator08_hmg124h_10.JPG.dat	1245.3	50.6	1.0	0.04	0.037	0.036	1.2E-05
YMR170C	YMR170C	S288C_Ator08_hmg124h_10.JPG.dat	1139.8	106.5	1.0	0.03	0.033	0.031	3.1E-04
YMR171C	YMR171C	S288C_Ator08_hmg124h_10.JPG.dat	1042.0	74.3	1.0	0.02	-0.013	0.015	8.2E-05
YMR172C-.	YMR172C-A	S288C_Ator08_hmg124h_10.JPG.dat	1084.5	70.5	0.9	0.03	-0.123	0.026	2.5E-04
YMR172W	YMR172W	S288C_Ator08_hmg124h_10.JPG.dat	1097.3	63.3	0.9	0.02	-0.060	0.019	1.4E-04
YMR173W	YMR173W	S288C_Ator08_hmg124h_10.JPG.dat	1140.3	79.1	1.0	0.01	-0.016	0.010	3.4E-05
YMR173W	YMR173W-A	S288C_Ator08_hmg124h_10.JPG.dat	1213.5	38.5	1.1	0.01	0.013	0.009	2.2E-05
YMR174C	YMR174C	S288C_Ator08_hmg124h_10.JPG.dat	1113.8	95.5	1.0	0.08	-0.012	0.080	1.4E-04
YMR175w	YMR175w	S288C_Ator08_hmg124h_10.JPG.dat	1154.3	59.4	1.0	0.04	0.002	0.045	2.3E-05
YMR176W	YMR176W	S288C_Ator08_hmg124h_10.JPG.dat	1163.0	45.2	1.0	0.04	0.030	0.037	1.4E-05
YMR177W	YMR177W	S288C_Ator08_hmg124h_10.JPG.dat	1167.3	53.6	1.0	0.06	-0.009	0.057	5.0E-05

YMR178W	YMR178W	S288C_Ator08_hmg124h_10.JPG.dat	1237.0	20.9	1.1	0.02	0.044	0.023	2.8E-06
YMR179W	YMR179W	S288C_Ator08_hmg124h_10.JPG.dat	1123.3	79.2	1.0	0.07	0.070	0.072	1.0E-04
YMR180C	YMR180C	S288C_Ator08_hmg124h_10.JPG.dat	1132.0	55.4	1.0	0.04	0.012	0.039	1.5E-05
YMR181C	YMR181C	S288C_Ator08_hmg124h_10.JPG.dat	1099.8	57.8	1.0	0.05	-0.082	0.047	3.2E-05
YMR182C	YMR182C	S288C_Ator08_hmg124h_10.JPG.dat	1171.8	20.1	1.0	0.02	0.070	0.023	3.2E-06
YMR183C	YMR183C	S288C_Ator08_hmg124h_10.JPG.dat	1072.5	21.2	1.0	0.02	-0.021	0.017	1.5E-06
YMR186W	YMR186W	S288C_Ator08_hmg124h_10.JPG.dat	988.8	67.8	0.9	0.06	-0.087	0.064	9.9E-05
YMR187C	YMR187C	S288C_Ator08_hmg124h_10.JPG.dat	1131.8	29.1	1.0	0.03	-0.047	0.027	5.2E-06
YMR188C	YMR188C	S288C_Ator08_hmg124h_10.JPG.dat	1121.8	38.9	1.0	0.03	-0.007	0.034	1.1E-05
YMR189W	YMR189W	S288C_Ator08_hmg124h_10.JPG.dat	1097.5	31.8	1.0	0.00	-0.032	0.003	4.5E-06
YMR190C	YMR190C	S288C_Ator08_hmg124h_10.JPG.dat	1076.5	37.4	0.9	0.03	0.020	0.028	7.5E-06
YMR191W	YMR191W	S288C_Ator08_hmg124h_10.JPG.dat	1078.8	96.1	0.9	0.08	-0.067	0.084	1.9E-04
YMR192W	YMR192W	S288C_Ator08_hmg124h_10.JPG.dat	1108.0	49.9	1.0	0.04	-0.025	0.036	1.3E-05
YMR193C-.	YMR193C-A	S288C_Ator08_hmg124h_10.JPG.dat	1104.0	38.8	1.0	0.02	-0.052	0.024	3.8E-06
YMR194W	YMR194W	S288C_Ator08_hmg124h_10.JPG.dat	1089.5	37.6	1.0	0.03	0.050	0.034	1.2E-05
YMR195W	YMR195W	S288C_Ator08_hmg124h_10.JPG.dat	1161.8	13.7	1.0	0.00	-0.026	0.005	7.0E-06
YMR196W	YMR196W	S288C_Ator08_hmg124h_10.JPG.dat	1117.3	26.5	1.0	0.02	0.003	0.023	3.4E-06
YMR198W	YMR198W	S288C_Ator08_hmg124h_10.JPG.dat	1013.3	58.9	0.9	0.05	-0.052	0.050	4.5E-05
YMR199W	YMR199W	S288C_Ator08_hmg124h_10.JPG.dat	1146.5	14.7	1.0	0.00	0.040	0.004	5.5E-06
YMR201C	YMR201C	S288C_Ator08_hmg124h_10.JPG.dat	836.8	559.4	1.0	0.04	0.003	0.043	6.1E-04
YMR202W	YMR202W	S288C_Ator08_hmg124h_10.JPG.dat	842.3	126.2	0.7	0.11	-0.178	0.112	9.0E-04
YMR204C	YMR204C	S288C_Ator08_hmg124h_10.JPG.dat	1042.0	35.1	0.9	0.03	-0.008	0.031	1.0E-05
YMR205C	YMR205C	S288C_Ator08_hmg124h_10.JPG.dat	1015.3	119.6	1.0	0.01	0.030	0.009	3.1E-05
YMR206W	YMR206W	S288C_Ator08_hmg124h_10.JPG.dat	1122.3	74.6	1.0	0.02	0.006	0.016	8.9E-05
YMR207C	YMR207C	S288C_Ator08_hmg124h_10.JPG.dat	1091.8	61.7	1.0	0.04	0.006	0.043	2.3E-05
YMR209C	YMR209C	S288C_Ator08_hmg124h_10.JPG.dat	1218.5	41.1	1.0	0.01	0.035	0.009	2.7E-05
YMR210W	YMR210W	S288C_Ator08_hmg124h_10.JPG.dat	1153.8	34.0	1.0	0.03	-0.058	0.031	8.0E-06
YMR214W	YMR214W	S288C_Ator08_hmg124h_10.JPG.dat	1140.3	38.3	1.0	0.03	0.024	0.031	8.2E-06
YMR215W	YMR215W	S288C_Ator08_hmg124h_10.JPG.dat	1084.0	53.8	1.0	0.05	-0.044	0.050	3.7E-05
YMR216C	YMR216C	S288C_Ator08_hmg124h_10.JPG.dat	1126.8	45.7	1.0	0.01	0.035	0.011	4.1E-05
YMR219W	YMR219W	S288C_Ator08_hmg124h_10.JPG.dat	1114.0	8.8	1.0	0.01	0.031	0.010	2.8E-07
YMR221C	YMR221C	S288C_Ator08_hmg124h_10.JPG.dat	1184.3	51.0	1.0	0.04	0.047	0.045	2.2E-05
YMR222C	YMR222C	S288C_Ator08_hmg124h_10.JPG.dat	1221.8	146.3	1.0	0.01	0.031	0.006	1.2E-05
YMR223W	YMR223W	S288C_Ator08_hmg124h_10.JPG.dat	1041.0	34.8	0.9	0.01	-0.005	0.007	1.7E-05
YMR224C	YMR224C	S288C_Ator08_hmg124h_10.JPG.dat	1056.8	72.7	1.0	0.01	-0.036	0.014	6.7E-05
YMR225C	YMR225C	S288C_Ator08_hmg124h_10.JPG.dat	1204.3	37.4	1.0	0.03	0.008	0.030	6.4E-06
YMR226C	YMR226C	S288C_Ator08_hmg124h_10.JPG.dat	1221.5	32.4	1.0	0.03	0.019	0.025	4.0E-06
YMR230W	YMR230W	S288C_Ator08_hmg124h_10.JPG.dat	940.3	74.7	0.8	0.06	-0.022	0.065	1.5E-04
YMR232W	YMR232W	S288C_Ator08_hmg124h_10.JPG.dat	1229.3	30.7	1.1	0.02	0.057	0.019	1.6E-06
YMR233W	YMR233W	S288C_Ator08_hmg124h_10.JPG.dat	1218.8	39.0	1.1	0.00	0.034	0.003	1.9E-06
YMR234W	YMR234W	S288C_Ator08_hmg124h_10.JPG.dat	1161.8	51.6	1.0	0.03	-0.001	0.035	1.2E-05
YMR237W	YMR237W	S288C_Ator08_hmg124h_10.JPG.dat	1106.0	132.7	1.0	0.02	-0.005	0.024	1.9E-04
YMR238W	YMR238W	S288C_Ator08_hmg124h_10.JPG.dat	1165.3	9.5	1.0	0.01	0.021	0.010	2.9E-07
YMR241W	YMR241W	S288C_Ator08_hmg124h_10.JPG.dat	852.0	568.0	1.0	0.01	0.013	0.009	2.7E-05
YMR243C	YMR243C	S288C_Ator08_hmg124h_10.JPG.dat	713.0	86.8	0.6	0.08	-0.161	0.076	4.8E-04
YMR244C-.	YMR244C-A	S288C_Ator08_hmg124h_10.JPG.dat	1085.3	11.9	1.0	0.01	-0.057	0.006	1.1E-05
YOR202W	YOR202W	S288C_Ator08_hmg124h_10.JPG.dat	1181.4	161.8	1.0	0.08	0.009	0.084	1.1E-290
1	1	S288C_Ator08_hmg124h_5.JPG.dat	1210.0	113.2	0.9	0.05	-0.161	0.045	8.4E-04
2	2	S288C_Ator08_hmg124h_5.JPG.dat	1191.8	40.4	1.1	0.03	0.111	0.026	3.6E-06
3	3	S288C_Ator08_hmg124h_5.JPG.dat	1152.8	30.4	1.1	0.04	0.067	0.042	1.7E-05
4	4	S288C_Ator08_hmg124h_5.JPG.dat	1140.8	65.0	1.0	0.07	0.038	0.068	8.5E-05
YFR012W	YFR012W	S288C_Ator08_hmg124h_5.JPG.dat	1153.3	42.2	1.0	0.03	-0.032	0.032	8.3E-06
YFR013W	YFR013W	S288C_Ator08_hmg124h_5.JPG.dat	1105.0	29.8	1.0	0.03	0.025	0.033	1.0E-05
YFR014C	YFR014C	S288C_Ator08_hmg124h_5.JPG.dat	1102.3	19.3	1.0	0.02	-0.004	0.024	3.8E-06
YFR015C	YFR015C	S288C_Ator08_hmg124h_5.JPG.dat	1114.3	28.6	1.0	0.02	0.022	0.022	2.7E-06
YFR016C	YFR016C	S288C_Ator08_hmg124h_5.JPG.dat	1052.0	8.4	1.0	0.01	0.019	0.007	8.8E-08
YFR017C	YFR017C	S288C_Ator08_hmg124h_5.JPG.dat	1041.5	15.1	1.0	0.02	-0.044	0.018	1.7E-06
YFR018C	YFR018C	S288C_Ator08_hmg124h_5.JPG.dat	1097.3	17.9	1.0	0.00	-0.026	0.001	1.1E-07
YFR019W	YFR019W	S288C_Ator08_hmg124h_5.JPG.dat	931.3	85.2	0.9	0.08	-0.128	0.077	1.9E-04
YFR020W	YFR020W	S288C_Ator08_hmg124h_5.JPG.dat	1059.0	19.9	1.0	0.02	0.019	0.022	2.8E-06
YFR021W	YFR021W	S288C_Ator08_hmg124h_5.JPG.dat	1001.0	38.2	1.0	0.01	0.001	0.006	1.3E-05
YFR022W	YFR022W	S288C_Ator08_hmg124h_5.JPG.dat	984.0	49.4	0.9	0.04	-0.087	0.037	1.7E-05
YFR023W	YFR023W	S288C_Ator08_hmg124h_5.JPG.dat	1100.3	26.1	1.0	0.00	0.036	0.004	6.1E-06
YFR024C	YFR024C	S288C_Ator08_hmg124h_5.JPG.dat	1123.8	52.6	1.0	0.05	-0.028	0.047	2.8E-05
YFR024C-A	YFR024C-A	S288C_Ator08_hmg124h_5.JPG.dat	1106.5	46.7	1.0	0.04	-0.009	0.043	2.2E-05
YFR025C	YFR025C	S288C_Ator08_hmg124h_5.JPG.dat	0.0	0.0	0.0	0.00	0.000	0.000	
YFR026C	YFR026C	S288C_Ator08_hmg124h_5.JPG.dat	1021.5	32.3	1.0	0.03	-0.089	0.028	7.2E-06
YFR030W	YFR030W	S288C_Ator08_hmg124h_5.JPG.dat	1052.3	11.4	1.0	0.00	-0.028	0.004	5.6E-06
YFR031C-A	YFR031C-A	S288C_Ator08_hmg124h_5.JPG.dat	1008.8	26.2	0.9	0.03	0.088	0.028	7.2E-06
YFR032C	YFR032C	S288C_Ator08_hmg124h_5.JPG.dat	1168.8	28.8	1.0	0.02	0.012	0.022	2.7E-06

YFR032C-A	YFR032C-A	S288C_Ator08_hmg124h_5.JPG.dat	1106.3	37.5	1.0	0.03	0.012	0.033	9.6E-06
YFR033C	YFR033C	S288C_Ator08_hmg124h_5.JPG.dat	1126.3	12.9	1.0	0.01	0.075	0.008	1.2E-07
YFR034C	YFR034C	S288C_Ator08_hmg124h_5.JPG.dat	1105.3	11.3	1.0	0.02	0.014	0.022	2.7E-06
YFR035C	YFR035C	S288C_Ator08_hmg124h_5.JPG.dat	1081.0	46.8	1.0	0.00	0.040	0.001	5.2E-07
YFR038W	YFR038W	S288C_Ator08_hmg124h_5.JPG.dat	1014.8	107.2	1.0	0.09	-0.030	0.090	2.3E-04
YFR039C	YFR039C	S288C_Ator08_hmg124h_5.JPG.dat	1026.5	90.7	1.0	0.10	-0.053	0.099	2.8E-04
YFR040W	YFR040W	S288C_Ator08_hmg124h_5.JPG.dat	921.8	46.7	0.9	0.05	-0.015	0.048	4.6E-05
YFR041C	YFR041C	S288C_Ator08_hmg124h_5.JPG.dat	1048.8	63.3	1.0	0.06	0.024	0.058	5.5E-05
YFR043C	YFR043C	S288C_Ator08_hmg124h_5.JPG.dat	967.3	35.5	0.9	0.01	-0.077	0.006	1.2E-05
YFR044C	YFR044C	S288C_Ator08_hmg124h_5.JPG.dat	1058.5	39.4	1.0	0.03	0.009	0.030	7.4E-06
YFR045W	YFR045W	S288C_Ator08_hmg124h_5.JPG.dat	1131.5	48.3	1.0	0.04	0.016	0.036	1.2E-05
YFR046C	YFR046C	S288C_Ator08_hmg124h_5.JPG.dat	1120.0	5.4	1.0	0.01	0.018	0.008	1.1E-07
YFR047C	YFR047C	S288C_Ator08_hmg124h_5.JPG.dat	1092.0	28.5	1.0	0.02	0.022	0.017	1.4E-06
YFR048W	YFR048W	S288C_Ator08_hmg124h_5.JPG.dat	1086.3	24.3	1.0	0.03	-0.036	0.031	7.8E-06
YFR049W	YFR049W	S288C_Ator08_hmg124h_5.JPG.dat	1077.5	16.8	1.0	0.01	-0.011	0.006	1.2E-05
YFR053C	YFR053C	S288C_Ator08_hmg124h_5.JPG.dat	1093.3	57.4	1.0	0.05	0.013	0.053	3.7E-05
YFR054C	YFR054C	S288C_Ator08_hmg124h_5.JPG.dat	930.3	60.9	0.9	0.06	-0.114	0.065	1.0E-04
YFR055W	YFR055W	S288C_Ator08_hmg124h_5.JPG.dat	973.5	140.5	1.0	0.03	0.031	0.027	2.4E-04
YFR056C	YFR056C	S288C_Ator08_hmg124h_5.JPG.dat	1043.0	44.4	1.0	0.04	0.012	0.042	2.0E-05
YFR057W	YFR057W	S288C_Ator08_hmg124h_5.JPG.dat	1064.0	17.5	1.0	0.02	-0.011	0.016	1.1E-06
YGL002W	YGL002W	S288C_Ator08_hmg124h_5.JPG.dat	1025.0	67.4	1.0	0.01	0.031	0.007	1.8E-05
YGL004C	YGL004C	S288C_Ator08_hmg124h_5.JPG.dat	1083.3	23.2	1.0	0.00	0.048	0.001	7.2E-07
YGL005C	YGL005C	S288C_Ator08_hmg124h_5.JPG.dat	1024.0	25.5	1.0	0.02	-0.089	0.020	2.5E-06
YGL006W	YGL006W	S288C_Ator08_hmg124h_5.JPG.dat	1068.8	23.1	1.0	0.02	-0.007	0.015	9.4E-07
YGL007W	YGL007W	S288C_Ator08_hmg124h_5.JPG.dat	872.8	42.3	0.8	0.03	-0.117	0.030	1.3E-05
YGL009C	YGL009C	S288C_Ator08_hmg124h_5.JPG.dat	1062.0	8.8	1.0	0.01	-0.011	0.011	3.8E-07
YGL010W	YGL010W	S288C_Ator08_hmg124h_5.JPG.dat	1067.5	19.6	1.0	0.02	-0.020	0.017	1.4E-06
YGL013C	YGL013C	S288C_Ator08_hmg124h_5.JPG.dat	1084.5	33.8	1.0	0.03	0.045	0.032	8.2E-06
YGL014W	YGL014W	S288C_Ator08_hmg124h_5.JPG.dat	1060.5	110.2	1.0	0.10	-0.022	0.099	2.6E-04
YGL015C	YGL015C	S288C_Ator08_hmg124h_5.JPG.dat	1071.0	47.9	1.0	0.04	-0.017	0.044	2.2E-05
YGL016W	YGL016W	S288C_Ator08_hmg124h_5.JPG.dat	993.3	13.2	0.9	0.01	0.067	0.013	7.4E-07
YGL017W	YGL017W	S288C_Ator08_hmg124h_5.JPG.dat	1019.8	28.6	1.0	0.03	-0.045	0.026	5.0E-06
YGL019W	YGL019W	S288C_Ator08_hmg124h_5.JPG.dat	969.8	104.6	0.8	0.03	-0.085	0.029	4.0E-04
YGL020C	YGL020C	S288C_Ator08_hmg124h_5.JPG.dat	991.0	47.3	0.9	0.05	-0.116	0.049	4.0E-05
YGL021W	YGL021W	S288C_Ator08_hmg124h_5.JPG.dat	1068.5	12.3	1.0	0.01	-0.015	0.012	4.4E-07
YGL023C	YGL023C	S288C_Ator08_hmg124h_5.JPG.dat	1090.3	40.3	1.0	0.03	0.069	0.027	5.3E-06
YGL024W	YGL024W	S288C_Ator08_hmg124h_5.JPG.dat	880.5	587.2	1.1	0.02	0.066	0.017	7.7E-05
YGL026C	YGL026C	S288C_Ator08_hmg124h_5.JPG.dat	0.0	0.0	0.0	0.00	0.000	0.000	
YGL027C	YGL027C	S288C_Ator08_hmg124h_5.JPG.dat	1084.8	2.6	1.0	0.01	0.001	0.012	4.3E-07
YGL028C	YGL028C	S288C_Ator08_hmg124h_5.JPG.dat	1059.5	9.2	1.0	0.01	-0.055	0.011	3.9E-07
YGL031C	YGL031C	S288C_Ator08_hmg124h_5.JPG.dat	1071.3	26.9	1.0	0.02	0.000	0.020	2.1E-06
YGL032C	YGL032C	S288C_Ator08_hmg124h_5.JPG.dat	1050.8	18.6	1.0	0.01	-0.002	0.012	4.8E-07
YGL034C	YGL034C	S288C_Ator08_hmg124h_5.JPG.dat	1076.8	25.4	1.0	0.03	0.010	0.027	5.6E-06
YGL035C	YGL035C	S288C_Ator08_hmg124h_5.JPG.dat	1035.5	17.5	0.9	0.02	-0.061	0.018	1.8E-06
YGL036W	YGL036W	S288C_Ator08_hmg124h_5.JPG.dat	1116.8	19.0	1.0	0.03	0.011	0.031	7.1E-06
YGL039W	YGL039W	S288C_Ator08_hmg124h_5.JPG.dat	1096.3	9.4	1.0	0.01	-0.011	0.008	1.4E-07
YGL041C	YGL041C	S288C_Ator08_hmg124h_5.JPG.dat	1100.5	29.9	1.0	0.01	-0.009	0.015	6.9E-05
YGL043W	YGL043W	S288C_Ator08_hmg124h_5.JPG.dat	886.5	54.6	0.8	0.05	-0.113	0.052	7.5E-05
YGL045W	YGL045W	S288C_Ator08_hmg124h_5.JPG.dat	850.3	29.5	0.8	0.03	-0.171	0.027	1.2E-05
YGL046W	YGL046W	S288C_Ator08_hmg124h_5.JPG.dat	831.8	31.4	0.8	0.03	-0.203	0.030	1.8E-05
YGL050W	YGL050W	S288C_Ator08_hmg124h_5.JPG.dat	1164.5	41.1	1.1	0.04	0.038	0.038	1.2E-05
YGL051W	YGL051W	S288C_Ator08_hmg124h_5.JPG.dat	1120.3	26.2	1.0	0.01	0.033	0.006	1.3E-05
YGL053W	YGL053W	S288C_Ator08_hmg124h_5.JPG.dat	1165.8	37.6	1.1	0.03	0.020	0.035	9.8E-06
YGL054C	YGL054C	S288C_Ator08_hmg124h_5.JPG.dat	1144.3	19.0	1.0	0.02	-0.023	0.018	1.6E-06
YGL056C	YGL056C	S288C_Ator08_hmg124h_5.JPG.dat	1117.3	20.3	1.0	0.02	-0.071	0.019	2.0E-06
YGL057C	YGL057C	S288C_Ator08_hmg124h_5.JPG.dat	1118.3	2.9	1.0	0.00	-0.047	0.005	3.4E-08
YGL058W	YGL058W	S288C_Ator08_hmg124h_5.JPG.dat	1068.0	59.5	0.9	0.04	-0.081	0.044	3.2E-05
YGL059W	YGL059W	S288C_Ator08_hmg124h_5.JPG.dat	1184.0	50.7	1.0	0.02	-0.009	0.023	3.9E-06
YGL060W	YGL060W	S288C_Ator08_hmg124h_5.JPG.dat	1166.8	11.6	1.0	0.00	-0.045	0.004	4.0E-06
YGL062W	YGL062W	S288C_Ator08_hmg124h_5.JPG.dat	1131.8	28.4	1.0	0.00	0.054	0.004	6.2E-06
YGL066W	YGL066W	S288C_Ator08_hmg124h_5.JPG.dat	1127.0	42.3	1.0	0.00	0.049	0.000	6.7E-08
YGL067W	YGL067W	S288C_Ator08_hmg124h_5.JPG.dat	1134.8	30.5	1.0	0.03	0.037	0.029	6.0E-06
YGL077C	YGL077C	S288C_Ator08_hmg124h_5.JPG.dat	1088.8	22.7	1.0	0.02	0.047	0.015	9.3E-07
YGL078C	YGL078C	S288C_Ator08_hmg124h_5.JPG.dat	1020.5	26.7	1.0	0.02	0.104	0.022	3.2E-06
YGL079W	YGL079W	S288C_Ator08_hmg124h_5.JPG.dat	1003.0	14.7	1.0	0.01	-0.074	0.014	8.2E-07
YGL080W	YGL080W	S288C_Ator08_hmg124h_5.JPG.dat	1019.0	36.3	0.9	0.01	-0.018	0.010	4.1E-05
YGL081W	YGL081W	S288C_Ator08_hmg124h_5.JPG.dat	1088.0	20.5	1.0	0.02	0.015	0.017	1.4E-06
YGL082W	YGL082W	S288C_Ator08_hmg124h_5.JPG.dat	1040.3	35.8	1.0	0.03	0.017	0.029	6.5E-06
YGL083W	YGL083W	S288C_Ator08_hmg124h_5.JPG.dat	942.8	24.6	0.9	0.01	-0.072	0.005	1.1E-05
YGL084C	YGL084C	S288C_Ator08_hmg124h_5.JPG.dat	900.5	159.9	0.8	0.15	-0.127	0.148	1.7E-03

YGL085W	YGL085W	S288C_Ator08_hmg124h_5.JPG.dat	1078.8	30.7	1.0	0.03	-0.034	0.028	6.4E-06
YGL086W	YGL086W	S288C_Ator08_hmg124h_5.JPG.dat	1162.0	54.4	1.0	0.05	0.068	0.050	2.9E-05
YGL087C	YGL087C	S288C_Ator08_hmg124h_5.JPG.dat	1147.3	27.8	1.1	0.03	-0.017	0.027	4.8E-06
YGL089C	YGL089C	S288C_Ator08_hmg124h_5.JPG.dat	1088.8	45.5	1.0	0.04	-0.052	0.041	1.9E-05
YGL090W	YGL090W	S288C_Ator08_hmg124h_5.JPG.dat	1019.8	92.4	1.0	0.09	-0.039	0.085	2.0E-04
YGL101W	YGL101W	S288C_Ator08_hmg124h_5.JPG.dat	1064.0	28.9	1.0	0.01	0.002	0.007	1.5E-05
YGL104C	YGL104C	S288C_Ator08_hmg124h_5.JPG.dat	1049.5	17.9	1.0	0.01	-0.022	0.012	6.2E-07
YGL105W	YGL105W	S288C_Ator08_hmg124h_5.JPG.dat	599.5	693.6	0.5	0.62	0.026	0.618	1.8E-01
YGL108C	YGL108C	S288C_Ator08_hmg124h_5.JPG.dat	1128.8	21.1	1.0	0.02	0.025	0.019	1.7E-06
YGL109W	YGL109W	S288C_Ator08_hmg124h_5.JPG.dat	1088.0	18.6	1.0	0.01	0.026	0.013	6.3E-07
YGL110C	YGL110C	S288C_Ator08_hmg124h_5.JPG.dat	1132.5	15.9	1.0	0.00	0.053	0.004	1.2E-08
YGL114W	YGL114W	S288C_Ator08_hmg124h_5.JPG.dat	1063.8	81.4	1.0	0.07	-0.025	0.066	7.8E-05
YGL115W	YGL115W	S288C_Ator08_hmg124h_5.JPG.dat	800.8	81.1	0.8	0.08	-0.139	0.076	2.8E-04
YGL117W	YGL117W	S288C_Ator08_hmg124h_5.JPG.dat	1079.5	45.2	1.0	0.04	-0.028	0.045	2.2E-05
YGL118C	YGL118C	S288C_Ator08_hmg124h_5.JPG.dat	1078.0	17.3	1.0	0.01	0.000	0.011	3.7E-07
YGL121C	YGL121C	S288C_Ator08_hmg124h_5.JPG.dat	1071.8	25.1	1.0	0.03	-0.024	0.031	7.5E-06
YGL124C	YGL124C	S288C_Ator08_hmg124h_5.JPG.dat	804.5	189.5	0.8	0.18	0.017	0.185	3.7E-03
YGL125W	YGL125W	S288C_Ator08_hmg124h_5.JPG.dat	1095.8	29.8	1.0	0.02	-0.026	0.017	1.4E-06
YGL126W	YGL126W	S288C_Ator08_hmg124h_5.JPG.dat	1081.3	16.8	1.0	0.01	-0.031	0.008	1.8E-07
YGL127C	YGL127C	S288C_Ator08_hmg124h_5.JPG.dat	971.0	13.6	0.9	0.01	0.042	0.005	5.8E-08
YGL131C	YGL131C	S288C_Ator08_hmg124h_5.JPG.dat	1097.8	11.8	1.0	0.01	0.031	0.005	3.4E-08
YGL132W	YGL132W	S288C_Ator08_hmg124h_5.JPG.dat	1101.0	44.9	1.0	0.01	0.015	0.011	3.5E-05
YGL133W	YGL133W	S288C_Ator08_hmg124h_5.JPG.dat	893.5	13.1	0.9	0.02	-0.122	0.017	2.2E-06
YGL136C	YGL136C	S288C_Ator08_hmg124h_5.JPG.dat	644.5	500.4	0.6	0.49	0.059	0.486	8.3E-02
YGL138C	YGL138C	S288C_Ator08_hmg124h_5.JPG.dat	989.5	137.2	1.0	0.02	0.052	0.018	1.1E-04
YGL139W	YGL139W	S288C_Ator08_hmg124h_5.JPG.dat	1055.8	60.0	1.0	0.05	0.017	0.054	4.4E-05
YGL140C	YGL140C	S288C_Ator08_hmg124h_5.JPG.dat	1018.5	32.8	1.0	0.01	-0.073	0.008	2.4E-05
YGL141W	YGL141W	S288C_Ator08_hmg124h_5.JPG.dat	1070.0	13.0	1.0	0.00	0.026	0.004	4.4E-06
YGL144C	YGL144C	S288C_Ator08_hmg124h_5.JPG.dat	1042.8	18.8	1.0	0.01	-0.029	0.012	5.1E-07
YGL146C	YGL146C	S288C_Ator08_hmg124h_5.JPG.dat	1095.3	33.6	1.0	0.04	0.049	0.037	1.4E-05
YGL147C	YGL147C	S288C_Ator08_hmg124h_5.JPG.dat	279.0	558.0	0.0	0.00	0.000	0.000	
YGL148W	YGL148W	S288C_Ator08_hmg124h_5.JPG.dat	1053.5	12.9	1.0	0.01	-0.020	0.011	3.3E-07
YGL149W	YGL149W	S288C_Ator08_hmg124h_5.JPG.dat	1102.8	3.4	1.0	0.01	0.081	0.012	4.5E-07
YGL151W	YGL151W	S288C_Ator08_hmg124h_5.JPG.dat	1065.8	17.3	1.0	0.02	0.004	0.018	1.6E-06
YGL152C	YGL152C	S288C_Ator08_hmg124h_5.JPG.dat	1022.0	20.8	1.0	0.02	0.010	0.021	2.8E-06
YGL153W	YGL153W	S288C_Ator08_hmg124h_5.JPG.dat	1001.0	21.9	1.0	0.02	0.006	0.018	2.0E-06
YGL154C	YGL154C	S288C_Ator08_hmg124h_5.JPG.dat	269.0	538.0	0.0	0.00	0.000	0.000	
YGL156W	YGL156W	S288C_Ator08_hmg124h_5.JPG.dat	1115.0	13.8	1.1	0.01	-0.012	0.012	3.9E-07
YGL157W	YGL157W	S288C_Ator08_hmg124h_5.JPG.dat	1028.5	24.4	1.0	0.00	-0.043	0.003	2.9E-06
YGL158W	YGL158W	S288C_Ator08_hmg124h_5.JPG.dat	1079.8	12.5	1.0	0.01	-0.025	0.009	1.9E-07
YGL159W	YGL159W	S288C_Ator08_hmg124h_5.JPG.dat	1099.5	46.5	1.0	0.04	-0.006	0.044	2.2E-05
YGL160W	YGL160W	S288C_Ator08_hmg124h_5.JPG.dat	1079.0	5.7	1.0	0.01	0.058	0.005	4.1E-08
YGL161C	YGL161C	S288C_Ator08_hmg124h_5.JPG.dat	1082.0	9.3	1.0	0.01	0.002	0.010	2.5E-07
YGL162W	YGL162W	S288C_Ator08_hmg124h_5.JPG.dat	1084.5	41.9	1.0	0.03	-0.046	0.026	5.0E-06
YGL163C	YGL163C	S288C_Ator08_hmg124h_5.JPG.dat	924.5	25.7	0.9	0.03	-0.032	0.027	8.4E-06
YGL164C	YGL164C	S288C_Ator08_hmg124h_5.JPG.dat	1129.5	60.2	1.1	0.06	0.008	0.056	4.2E-05
YGL165C	YGL165C	S288C_Ator08_hmg124h_5.JPG.dat	1060.0	48.8	1.0	0.04	-0.016	0.043	2.1E-05
YGL166W	YGL166W	S288C_Ator08_hmg124h_5.JPG.dat	1094.8	10.0	1.0	0.00	0.004	0.005	7.1E-06
YGL167C	YGL167C	S288C_Ator08_hmg124h_5.JPG.dat	944.5	91.7	0.8	0.02	-0.035	0.025	2.8E-04
YGL168W	YGL168W	S288C_Ator08_hmg124h_5.JPG.dat	955.0	54.0	0.9	0.00	-0.012	0.003	4.1E-06
YGL170C	YGL170C	S288C_Ator08_hmg124h_5.JPG.dat	1114.5	35.5	1.0	0.01	0.029	0.006	1.1E-05
YGL173C	YGL173C	S288C_Ator08_hmg124h_5.JPG.dat	896.3	36.5	0.8	0.04	0.036	0.040	3.1E-05
YGL174W	YGL174W	S288C_Ator08_hmg124h_5.JPG.dat	970.0	15.9	0.9	0.02	0.024	0.022	3.9E-06
YGL175C	YGL175C	S288C_Ator08_hmg124h_5.JPG.dat	1087.5	18.1	1.0	0.00	-0.015	0.002	2.1E-06
YGL176C	YGL176C	S288C_Ator08_hmg124h_5.JPG.dat	1161.8	40.6	1.0	0.02	0.021	0.017	8.8E-05
YGL177W	YGL177W	S288C_Ator08_hmg124h_5.JPG.dat	1122.3	13.9	1.0	0.02	0.063	0.016	9.8E-07
YGL179C	YGL179C	S288C_Ator08_hmg124h_5.JPG.dat	1135.5	36.3	1.0	0.03	-0.026	0.031	7.6E-06
YGL180W	YGL180W	S288C_Ator08_hmg124h_5.JPG.dat	1106.5	33.8	1.0	0.03	-0.002	0.033	9.4E-06
YGL181W	YGL181W	S288C_Ator08_hmg124h_5.JPG.dat	1160.8	22.8	1.0	0.02	0.012	0.025	3.6E-06
YGL194C	YGL194C	S288C_Ator08_hmg124h_5.JPG.dat	1075.8	14.6	1.0	0.00	0.037	0.002	1.4E-06
YGL195W	YGL195W	S288C_Ator08_hmg124h_5.JPG.dat	1142.3	18.4	1.0	0.02	0.030	0.017	1.3E-06
YGL196W	YGL196W	S288C_Ator08_hmg124h_5.JPG.dat	1160.0	31.6	1.0	0.03	-0.024	0.026	4.5E-06
YGL197W	YGL197W	S288C_Ator08_hmg124h_5.JPG.dat	1150.8	25.3	1.0	0.03	-0.006	0.025	4.6E-06
YGL198W	YGL198W	S288C_Ator08_hmg124h_5.JPG.dat	1129.3	34.3	1.0	0.02	-0.039	0.023	3.6E-06
YGL199C	YGL199C	S288C_Ator08_hmg124h_5.JPG.dat	1106.0	30.9	0.9	0.02	-0.039	0.022	3.4E-06
YGL202W	YGL202W	S288C_Ator08_hmg124h_5.JPG.dat	1186.0	17.1	1.0	0.03	0.205	0.035	1.1E-05
YGL203C	YGL203C	S288C_Ator08_hmg124h_5.JPG.dat	1094.0	36.8	0.9	0.05	-0.028	0.051	4.5E-05
YGL205W	YGL205W	S288C_Ator08_hmg124h_5.JPG.dat	1139.3	42.1	1.0	0.01	-0.016	0.014	8.0E-07
YGL208W	YGL208W	S288C_Ator08_hmg124h_5.JPG.dat	1143.0	29.1	1.0	0.01	-0.032	0.012	4.8E-05
YGL209W	YGL209W	S288C_Ator08_hmg124h_5.JPG.dat	1089.0	43.5	1.0	0.02	-0.017	0.023	3.6E-06

YGL210W	YGL210W	S288C_Ator08_hmg124h_5.JPG.dat	1088.8	24.1	1.0	0.02	-0.027	0.017	1.2E-06
YGL211W	YGL211W	S288C_Ator08_hmg124h_5.JPG.dat	1029.5	43.1	0.9	0.01	0.057	0.007	1.8E-05
YGL212W	YGL212W	S288C_Ator08_hmg124h_5.JPG.dat	923.8	225.5	0.9	0.20	-0.031	0.204	3.6E-03
YGL213C	YGL213C	S288C_Ator08_hmg124h_5.JPG.dat	1090.3	56.9	1.0	0.04	0.028	0.038	1.3E-05
YGL214W	YGL214W	S288C_Ator08_hmg124h_5.JPG.dat	787.3	526.0	1.0	0.03	0.070	0.027	2.3E-04
YGL215W	YGL215W	S288C_Ator08_hmg124h_5.JPG.dat	970.5	39.1	0.9	0.02	-0.117	0.025	5.2E-06
YGL216W	YGL216W	S288C_Ator08_hmg124h_5.JPG.dat	1099.5	62.4	1.0	0.05	-0.043	0.053	4.3E-05
YGL217C	YGL217C	S288C_Ator08_hmg124h_5.JPG.dat	1140.3	35.9	1.0	0.03	0.000	0.030	7.4E-06
YGL218W	YGL218W	S288C_Ator08_hmg124h_5.JPG.dat	313.0	520.1	0.1	0.09	0.044	0.087	4.2E-01
YGL219C	YGL219C	S288C_Ator08_hmg124h_5.JPG.dat	1014.0	27.9	0.9	0.03	0.044	0.027	7.2E-06
YGL221C	YGL221C	S288C_Ator08_hmg124h_5.JPG.dat	1058.8	33.9	1.0	0.03	0.002	0.031	8.8E-06
YGL222C	YGL222C	S288C_Ator08_hmg124h_5.JPG.dat	1067.0	21.9	1.0	0.02	0.016	0.021	2.4E-06
YGL224C	YGL224C	S288C_Ator08_hmg124h_5.JPG.dat	1104.3	15.5	1.0	0.01	-0.012	0.013	5.2E-07
YGL226C-A	YGL226C-A	S288C_Ator08_hmg124h_5.JPG.dat	1157.3	7.3	1.0	0.01	0.026	0.010	2.6E-07
YGL226W	YGL226W	S288C_Ator08_hmg124h_5.JPG.dat	1077.3	53.2	1.0	0.05	0.021	0.045	2.7E-05
YGL227W	YGL227W	S288C_Ator08_hmg124h_5.JPG.dat	1081.0	9.3	1.0	0.01	0.019	0.007	8.9E-08
YGL228W	YGL228W	S288C_Ator08_hmg124h_5.JPG.dat	1061.0	35.4	1.0	0.02	-0.038	0.022	3.0E-06
YGL229C	YGL229C	S288C_Ator08_hmg124h_5.JPG.dat	1062.8	39.8	1.0	0.04	-0.018	0.037	1.5E-05
YGL230C	YGL230C	S288C_Ator08_hmg124h_5.JPG.dat	1099.3	22.5	1.0	0.02	-0.001	0.023	3.3E-06
YGL231C	YGL231C	S288C_Ator08_hmg124h_5.JPG.dat	1045.0	26.3	1.0	0.03	-0.011	0.026	4.9E-06
YGL232W	YGL232W	S288C_Ator08_hmg124h_5.JPG.dat	1141.8	42.3	1.1	0.01	0.041	0.015	6.5E-05
YGL234W	YGL234W	S288C_Ator08_hmg124h_5.JPG.dat	1028.8	41.2	1.0	0.04	-0.036	0.038	1.6E-05
YGL235W	YGL235W	S288C_Ator08_hmg124h_5.JPG.dat	1049.3	13.1	1.0	0.01	0.049	0.010	2.8E-07
YGL236C	YGL236C	S288C_Ator08_hmg124h_5.JPG.dat	1093.3	21.2	1.0	0.02	0.029	0.017	1.3E-06
YGL237C	YGL237C	S288C_Ator08_hmg124h_5.JPG.dat	294.5	589.0	0.0	0.00	0.000	0.000	
YGL241W	YGL241W	S288C_Ator08_hmg124h_5.JPG.dat	1109.5	9.3	1.0	0.00	0.068	0.003	2.1E-06
YGL242C	YGL242C	S288C_Ator08_hmg124h_5.JPG.dat	1042.0	32.1	1.0	0.03	-0.027	0.032	9.5E-06
YGL243W	YGL243W	S288C_Ator08_hmg124h_5.JPG.dat	1096.3	25.8	1.0	0.02	-0.019	0.021	2.2E-06
YGL244W	YGL244W	S288C_Ator08_hmg124h_5.JPG.dat	898.3	15.1	0.8	0.01	-0.042	0.014	1.3E-06
YGL248W	YGL248W	S288C_Ator08_hmg124h_5.JPG.dat	978.0	52.6	0.9	0.05	-0.079	0.050	4.3E-05
YGL249W	YGL249W	S288C_Ator08_hmg124h_5.JPG.dat	1025.0	75.2	1.0	0.07	-0.013	0.070	9.7E-05
YGL250W	YGL250W	S288C_Ator08_hmg124h_5.JPG.dat	1031.3	20.3	1.0	0.01	-0.036	0.005	1.1E-05
YGL251C	YGL251C	S288C_Ator08_hmg124h_5.JPG.dat	1042.0	49.7	1.0	0.01	0.025	0.008	2.0E-05
YGL252C	YGL252C	S288C_Ator08_hmg124h_5.JPG.dat	1055.5	31.4	1.0	0.03	0.024	0.030	7.5E-06
YGL253W	YGL253W	S288C_Ator08_hmg124h_5.JPG.dat	963.5	18.4	0.9	0.02	-0.070	0.018	2.1E-06
YGL254W	YGL254W	S288C_Ator08_hmg124h_5.JPG.dat	1045.8	102.9	1.0	0.01	-0.002	0.008	2.3E-05
YGL255W	YGL255W	S288C_Ator08_hmg124h_5.JPG.dat	1131.0	32.9	1.0	0.03	-0.022	0.035	1.1E-05
YGL256W	YGL256W	S288C_Ator08_hmg124h_5.JPG.dat	1107.3	24.0	1.0	0.02	-0.005	0.021	2.5E-06
YGL257C	YGL257C	S288C_Ator08_hmg124h_5.JPG.dat	1114.5	21.1	1.0	0.01	0.080	0.014	6.7E-07
YGL258W	YGL258W	S288C_Ator08_hmg124h_5.JPG.dat	1083.5	18.9	1.0	0.01	-0.020	0.013	6.1E-07
YGL259W	YGL259W	S288C_Ator08_hmg124h_5.JPG.dat	1098.3	37.2	1.0	0.04	-0.005	0.036	1.1E-05
YGL260W	YGL260W	S288C_Ator08_hmg124h_5.JPG.dat	1050.0	35.6	1.0	0.03	0.005	0.034	1.1E-05
YGL261C	YGL261C	S288C_Ator08_hmg124h_5.JPG.dat	1071.5	25.6	1.0	0.02	0.007	0.020	2.0E-06
YGL262W	YGL262W	S288C_Ator08_hmg124h_5.JPG.dat	1070.8	14.4	1.0	0.02	0.004	0.015	9.6E-07
YGL263W	YGL263W	S288C_Ator08_hmg124h_5.JPG.dat	1059.8	42.3	1.0	0.04	0.022	0.036	1.3E-05
YGR001C	YGR001C	S288C_Ator08_hmg124h_5.JPG.dat	1032.8	59.3	1.0	0.01	0.002	0.010	3.1E-05
YGR003W	YGR003W	S288C_Ator08_hmg124h_5.JPG.dat	1071.5	27.4	1.0	0.03	-0.007	0.027	6.1E-06
YGR004W	YGR004W	S288C_Ator08_hmg124h_5.JPG.dat	1074.0	28.2	1.0	0.03	0.013	0.033	9.7E-06
YGR007W	YGR007W	S288C_Ator08_hmg124h_5.JPG.dat	1070.3	49.7	1.0	0.04	-0.007	0.044	2.4E-05
YGR008C	YGR008C	S288C_Ator08_hmg124h_5.JPG.dat	1094.5	29.7	1.0	0.02	-0.002	0.021	2.5E-06
YGR010W	YGR010W	S288C_Ator08_hmg124h_5.JPG.dat	1078.0	23.1	1.0	0.02	-0.044	0.023	3.4E-06
YGR011W	YGR011W	S288C_Ator08_hmg124h_5.JPG.dat	1051.0	48.8	1.0	0.05	-0.057	0.047	3.0E-05
YGR012W	YGR012W	S288C_Ator08_hmg124h_5.JPG.dat	1096.3	28.0	1.0	0.01	0.042	0.010	2.9E-05
YGR014W	YGR014W	S288C_Ator08_hmg124h_5.JPG.dat	1086.0	48.0	1.0	0.04	-0.018	0.041	1.8E-05
YGR015C	YGR015C	S288C_Ator08_hmg124h_5.JPG.dat	1060.3	26.0	1.0	0.02	0.006	0.023	3.5E-06
YGR016W	YGR016W	S288C_Ator08_hmg124h_5.JPG.dat	1074.0	42.2	1.0	0.04	0.010	0.039	1.5E-05
YGR017W	YGR017W	S288C_Ator08_hmg124h_5.JPG.dat	1056.0	39.8	1.0	0.04	0.005	0.039	1.7E-05
YGR018C	YGR018C	S288C_Ator08_hmg124h_5.JPG.dat	1102.5	14.8	1.0	0.01	-0.018	0.013	5.4E-07
YGR019W	YGR019W	S288C_Ator08_hmg124h_5.JPG.dat	1080.0	26.3	1.0	0.03	-0.003	0.029	6.4E-06
YGR021W	YGR021W	S288C_Ator08_hmg124h_5.JPG.dat	1090.3	2.9	1.0	0.00	-0.033	0.004	1.5E-08
YGR022C	YGR022C	S288C_Ator08_hmg124h_5.JPG.dat	1084.3	33.5	1.0	0.02	-0.048	0.020	2.1E-06
YGR023W	YGR023W	S288C_Ator08_hmg124h_5.JPG.dat	978.8	66.1	0.9	0.06	-0.099	0.057	6.9E-05
YGR025W	YGR025W	S288C_Ator08_hmg124h_5.JPG.dat	1103.5	39.3	1.0	0.04	-0.010	0.041	1.8E-05
YGR026W	YGR026W	S288C_Ator08_hmg124h_5.JPG.dat	1109.0	41.3	1.0	0.03	-0.034	0.031	7.7E-06
YGR027C	YGR027C	S288C_Ator08_hmg124h_5.JPG.dat	1054.3	42.6	1.0	0.04	0.065	0.036	1.4E-05
YGR028W	YGR028W	S288C_Ator08_hmg124h_5.JPG.dat	1080.8	30.0	1.0	0.01	0.044	0.012	4.9E-05
YGR031W	YGR031W	S288C_Ator08_hmg124h_5.JPG.dat	1103.0	23.2	1.0	0.00	0.022	0.005	7.4E-06
YGR032W	YGR032W	S288C_Ator08_hmg124h_5.JPG.dat	1085.3	14.5	1.0	0.00	0.014	0.002	1.1E-06
YGR033C	YGR033C	S288C_Ator08_hmg124h_5.JPG.dat	1095.8	47.5	1.0	0.01	-0.014	0.012	5.2E-05
YGR034W	YGR034W	S288C_Ator08_hmg124h_5.JPG.dat	1067.8	46.4	1.0	0.04	0.029	0.039	1.8E-05

YGR035C	YGR035C	S288C_Ator08_hmg124h_5.JPG.dat	1051.8	102.9	1.0	0.11	-0.084	0.107	3.8E-04
YGR037C	YGR037C	S288C_Ator08_hmg124h_5.JPG.dat	1041.0	33.6	0.9	0.01	-0.064	0.011	4.9E-07
YGR038W	YGR038W	S288C_Ator08_hmg124h_5.JPG.dat	1221.0	37.4	1.0	0.01	-0.059	0.008	1.2E-07
YGR039W	YGR039W	S288C_Ator08_hmg124h_5.JPG.dat	1141.5	33.8	1.0	0.04	-0.007	0.042	2.1E-05
YGR040W	YGR040W	S288C_Ator08_hmg124h_5.JPG.dat	1108.5	9.8	1.0	0.02	-0.036	0.020	2.6E-06
YGR041W	YGR041W	S288C_Ator08_hmg124h_5.JPG.dat	1155.0	22.5	1.0	0.02	0.036	0.022	2.9E-06
YGR042W	YGR042W	S288C_Ator08_hmg124h_5.JPG.dat	1136.8	22.2	1.0	0.03	0.011	0.030	7.6E-06
YGR043C	YGR043C	S288C_Ator08_hmg124h_5.JPG.dat	1102.0	24.2	1.0	0.00	0.037	0.002	1.7E-06
YGR044C	YGR044C	S288C_Ator08_hmg124h_5.JPG.dat	1111.5	43.1	1.0	0.02	0.044	0.020	2.1E-06
YGR045C	YGR045C	S288C_Ator08_hmg124h_5.JPG.dat	1139.8	28.9	1.1	0.01	0.006	0.010	2.1E-07
YGR049W	YGR049W	S288C_Ator08_hmg124h_5.JPG.dat	1094.0	46.8	1.0	0.03	0.023	0.029	6.3E-06
YGR050C	YGR050C	S288C_Ator08_hmg124h_5.JPG.dat	975.3	50.1	0.9	0.04	-0.097	0.043	2.7E-05
YGR051C	YGR051C	S288C_Ator08_hmg124h_5.JPG.dat	980.3	25.7	0.9	0.00	-0.084	0.005	8.4E-06
YGR052W	YGR052W	S288C_Ator08_hmg124h_5.JPG.dat	1143.0	39.2	1.0	0.03	-0.019	0.027	5.5E-06
YGR053C	YGR053C	S288C_Ator08_hmg124h_5.JPG.dat	1082.0	23.0	1.0	0.02	-0.053	0.020	2.5E-06
YGR054W	YGR054W	S288C_Ator08_hmg124h_5.JPG.dat	1102.3	49.3	1.0	0.05	-0.041	0.047	3.0E-05
YGR058W	YGR058W	S288C_Ator08_hmg124h_5.JPG.dat	1108.5	25.2	1.0	0.02	-0.011	0.021	2.6E-06
YGR059W	YGR059W	S288C_Ator08_hmg124h_5.JPG.dat	1078.3	41.2	1.0	0.04	-0.020	0.037	1.5E-05
YGR066C	YGR066C	S288C_Ator08_hmg124h_5.JPG.dat	1093.0	31.6	1.0	0.03	0.035	0.028	6.0E-06
YGR069W	YGR069W	S288C_Ator08_hmg124h_5.JPG.dat	1072.0	36.9	1.0	0.01	0.034	0.009	2.7E-05
YGR070W	YGR070W	S288C_Ator08_hmg124h_5.JPG.dat	1123.8	15.7	1.0	0.01	0.026	0.013	5.8E-07
YGR071C	YGR071C	S288C_Ator08_hmg124h_5.JPG.dat	1156.8	17.7	1.0	0.02	-0.023	0.021	2.4E-06
YGR072W	YGR072W	S288C_Ator08_hmg124h_5.JPG.dat	1184.8	35.6	1.1	0.01	0.102	0.010	3.1E-05
YGR077C	YGR077C	S288C_Ator08_hmg124h_5.JPG.dat	980.5	36.8	0.9	0.01	0.009	0.007	1.7E-05
YGR078C	YGR078C	S288C_Ator08_hmg124h_5.JPG.dat	991.3	46.0	0.9	0.04	0.040	0.040	2.3E-05
YGR079W	YGR079W	S288C_Ator08_hmg124h_5.JPG.dat	1049.5	28.3	1.0	0.03	-0.018	0.028	6.4E-06
YGR080W	YGR080W	S288C_Ator08_hmg124h_5.JPG.dat	1072.3	64.3	1.0	0.06	-0.019	0.063	6.9E-05
YGR081C	YGR081C	S288C_Ator08_hmg124h_5.JPG.dat	1016.0	43.0	1.0	0.04	0.089	0.042	2.2E-05
YGR084C	YGR084C	S288C_Ator08_hmg124h_5.JPG.dat	1071.8	31.8	1.0	0.03	0.031	0.026	4.6E-06
YGR085C	YGR085C	S288C_Ator08_hmg124h_5.JPG.dat	1040.8	42.2	1.0	0.04	-0.013	0.039	1.7E-05
YGR086C	YGR086C	S288C_Ator08_hmg124h_5.JPG.dat	991.0	38.4	1.0	0.01	-0.033	0.013	6.1E-05
YGR087C	YGR087C	S288C_Ator08_hmg124h_5.JPG.dat	1081.3	38.7	1.0	0.03	-0.032	0.032	9.1E-06
YGR088W	YGR088W	S288C_Ator08_hmg124h_5.JPG.dat	1079.0	74.1	1.0	0.02	-0.009	0.022	1.6E-04
YGR089W	YGR089W	S288C_Ator08_hmg124h_5.JPG.dat	1042.8	35.2	1.0	0.03	-0.036	0.033	1.1E-05
YGR092W	YGR092W	S288C_Ator08_hmg124h_5.JPG.dat	676.5	541.6	0.6	0.52	-0.072	0.516	8.8E-02
YGR093W	YGR093W	S288C_Ator08_hmg124h_5.JPG.dat	1095.8	41.0	1.0	0.03	0.013	0.034	1.0E-05
YGR096W	YGR096W	S288C_Ator08_hmg124h_5.JPG.dat	1068.8	12.9	1.0	0.00	-0.022	0.005	7.0E-06
YGR097W	YGR097W	S288C_Ator08_hmg124h_5.JPG.dat	802.8	537.3	1.0	0.05	-0.034	0.052	8.7E-04
YGR100W	YGR100W	S288C_Ator08_hmg124h_5.JPG.dat	957.8	133.4	1.0	0.04	0.003	0.037	4.8E-04
YGR101W	YGR101W	S288C_Ator08_hmg124h_5.JPG.dat	860.5	16.8	0.8	0.02	0.197	0.016	2.1E-06
YGR105W	YGR105W	S288C_Ator08_hmg124h_5.JPG.dat	1093.3	19.9	1.0	0.02	0.022	0.018	1.5E-06
YGR106C	YGR106C	S288C_Ator08_hmg124h_5.JPG.dat	1046.3	38.2	1.0	0.04	-0.017	0.037	1.4E-05
YGR107W	YGR107W	S288C_Ator08_hmg124h_5.JPG.dat	1074.8	47.6	1.0	0.04	0.011	0.037	1.4E-05
YGR108W	YGR108W	S288C_Ator08_hmg124h_5.JPG.dat	1122.8	20.2	1.0	0.02	0.033	0.018	1.6E-06
YGR109C	YGR109C	S288C_Ator08_hmg124h_5.JPG.dat	1075.8	25.1	1.0	0.02	-0.060	0.024	3.9E-06
YGR110W	YGR110W	S288C_Ator08_hmg124h_5.JPG.dat	1090.0	32.8	1.0	0.03	-0.025	0.030	6.7E-06
YGR111W	YGR111W	S288C_Ator08_hmg124h_5.JPG.dat	1051.3	12.7	1.0	0.01	-0.010	0.013	7.5E-07
YGR117C	YGR117C	S288C_Ator08_hmg124h_5.JPG.dat	1089.8	31.1	1.0	0.03	-0.008	0.034	1.0E-05
YGR118W	YGR118W	S288C_Ator08_hmg124h_5.JPG.dat	884.5	50.1	0.8	0.04	-0.040	0.045	4.1E-05
YGR121C	YGR121C	S288C_Ator08_hmg124h_5.JPG.dat	1043.8	26.3	1.0	0.02	-0.034	0.019	1.8E-06
YGR123C	YGR123C	S288C_Ator08_hmg124h_5.JPG.dat	1059.8	44.3	1.0	0.04	0.004	0.039	1.7E-05
YGR124W	YGR124W	S288C_Ator08_hmg124h_5.JPG.dat	1084.3	37.5	1.0	0.03	0.018	0.031	7.9E-06
YGR125W	YGR125W	S288C_Ator08_hmg124h_5.JPG.dat	1046.0	18.6	1.0	0.02	-0.015	0.017	1.4E-06
YGR126W	YGR126W	S288C_Ator08_hmg124h_5.JPG.dat	1080.5	28.9	1.0	0.00	0.024	0.002	1.9E-06
YGR127W	YGR127W	S288C_Ator08_hmg124h_5.JPG.dat	1099.3	9.5	1.0	0.01	0.012	0.011	3.3E-07
YGR129W	YGR129W	S288C_Ator08_hmg124h_5.JPG.dat	1074.5	17.7	1.0	0.01	0.019	0.007	1.4E-05
YGR130C	YGR130C	S288C_Ator08_hmg124h_5.JPG.dat	1085.3	18.6	1.0	0.02	0.017	0.019	1.7E-06
YGR131W	YGR131W	S288C_Ator08_hmg124h_5.JPG.dat	1100.3	25.9	1.0	0.01	-0.034	0.014	8.0E-07
YGR132C	YGR132C	S288C_Ator08_hmg124h_5.JPG.dat	1083.3	35.6	1.0	0.03	-0.002	0.033	9.4E-06
YGR133W	YGR133W	S288C_Ator08_hmg124h_5.JPG.dat	1040.3	35.3	1.0	0.01	0.016	0.010	3.4E-05
YGR134W	YGR134W	S288C_Ator08_hmg124h_5.JPG.dat	1001.8	41.8	0.9	0.04	0.005	0.037	1.7E-05
YGR135W	YGR135W	S288C_Ator08_hmg124h_5.JPG.dat	1085.5	43.8	1.0	0.04	0.073	0.043	2.2E-05
YGR136W	YGR136W	S288C_Ator08_hmg124h_5.JPG.dat	1071.5	21.4	1.0	0.01	0.039	0.005	8.5E-06
YGR137W	YGR137W	S288C_Ator08_hmg124h_5.JPG.dat	1102.0	39.4	1.0	0.03	0.043	0.035	1.0E-05
YGR138C	YGR138C	S288C_Ator08_hmg124h_5.JPG.dat	1099.5	29.6	1.0	0.03	0.012	0.031	8.1E-06
YGR139W	YGR139W	S288C_Ator08_hmg124h_5.JPG.dat	1090.0	16.8	1.0	0.00	-0.004	0.004	6.4E-06
YGR141W	YGR141W	S288C_Ator08_hmg124h_5.JPG.dat	1022.5	18.9	1.0	0.02	-0.013	0.018	1.7E-06
YGR142W	YGR142W	S288C_Ator08_hmg124h_5.JPG.dat	1065.5	28.5	1.0	0.03	-0.001	0.029	6.8E-06
YGR143W	YGR143W	S288C_Ator08_hmg124h_5.JPG.dat	1074.5	13.4	1.0	0.00	-0.056	0.004	4.6E-06
YGR144W	YGR144W	S288C_Ator08_hmg124h_5.JPG.dat	1106.3	8.8	1.0	0.01	-0.022	0.007	9.3E-08

YGR146C	YGR146C	S288C_Ator08_hmg124h_5.JPG.dat	1085.0	38.7	1.0	0.03	-0.010	0.029	6.7E-06
YGR148C	YGR148C	S288C_Ator08_hmg124h_5.JPG.dat	1063.8	15.1	1.0	0.02	0.077	0.016	1.1E-06
YGR149W	YGR149W	S288C_Ator08_hmg124h_5.JPG.dat	1125.3	31.4	1.0	0.02	0.040	0.024	3.4E-06
YGR151C	YGR151C	S288C_Ator08_hmg124h_5.JPG.dat	1106.8	37.1	1.0	0.03	0.005	0.029	6.3E-06
YGR152C	YGR152C	S288C_Ator08_hmg124h_5.JPG.dat	1133.8	50.9	1.1	0.01	0.078	0.013	4.6E-05
YGR153W	YGR153W	S288C_Ator08_hmg124h_5.JPG.dat	1084.8	14.0	1.0	0.01	0.001	0.008	2.0E-05
YGR154C	YGR154C	S288C_Ator08_hmg124h_5.JPG.dat	1111.5	12.1	1.0	0.02	-0.012	0.016	1.2E-06
YGR157W	YGR157W	S288C_Ator08_hmg124h_5.JPG.dat	590.3	375.1	0.7	0.10	0.014	0.098	6.3E-03
YGR161C	YGR161C	S288C_Ator08_hmg124h_5.JPG.dat	1078.8	18.4	1.0	0.03	-0.050	0.028	6.5E-06
YOR202W	YOR202W	S288C_Ator08_hmg124h_5.JPG.dat	1205.1	131.8	1.0	0.07	0.008	0.072	1.93510533
1	1	S288C_Ator08_hmg124h_6.JPG.dat	1533.3	228.3	1.1	0.11	-0.004	0.106	2.7E-04
2	2	S288C_Ator08_hmg124h_6.JPG.dat	1354.8	156.8	1.1	0.10	0.115	0.104	2.2E-04
3	3	S288C_Ator08_hmg124h_6.JPG.dat	1174.3	103.5	1.0	0.07	0.018	0.069	9.0E-05
4	4	S288C_Ator08_hmg124h_6.JPG.dat	1106.3	100.7	1.0	0.09	-0.008	0.090	2.1E-04
YGR163W	YGR163W	S288C_Ator08_hmg124h_6.JPG.dat	1105.5	27.6	1.0	0.02	0.104	0.025	4.2E-06
YGR164W	YGR164W	S288C_Ator08_hmg124h_6.JPG.dat	1118.3	13.0	1.0	0.01	0.022	0.010	2.3E-07
YGR166W	YGR166W	S288C_Ator08_hmg124h_6.JPG.dat	1020.8	33.0	0.9	0.01	-0.049	0.009	2.9E-05
YGR168C	YGR168C	S288C_Ator08_hmg124h_6.JPG.dat	1096.3	39.6	1.0	0.03	-0.029	0.034	1.1E-05
YGR169C	YGR169C	S288C_Ator08_hmg124h_6.JPG.dat	1130.3	36.0	1.0	0.03	0.026	0.033	9.3E-06
YGR170W	YGR170W	S288C_Ator08_hmg124h_6.JPG.dat	1077.8	38.8	1.0	0.04	-0.022	0.035	1.3E-05
YGR173W	YGR173W	S288C_Ator08_hmg124h_6.JPG.dat	1138.3	49.0	1.0	0.04	0.001	0.044	2.1E-05
YGR174C	YGR174C	S288C_Ator08_hmg124h_6.JPG.dat	1086.5	17.4	1.0	0.02	0.055	0.016	1.2E-06
YGR176W	YGR176W	S288C_Ator08_hmg124h_6.JPG.dat	1083.8	16.1	1.0	0.02	-0.031	0.016	1.2E-06
YGR177C	YGR177C	S288C_Ator08_hmg124h_6.JPG.dat	1102.0	19.3	1.0	0.01	0.028	0.007	1.6E-05
YGR178C	YGR178C	S288C_Ator08_hmg124h_6.JPG.dat	1150.5	28.8	1.0	0.02	-0.013	0.024	3.5E-06
YGR181W	YGR181W	S288C_Ator08_hmg124h_6.JPG.dat	1052.8	21.4	1.0	0.01	0.009	0.011	3.3E-07
YGR182C	YGR182C	S288C_Ator08_hmg124h_6.JPG.dat	1069.5	18.6	1.0	0.02	0.047	0.020	1.9E-06
YGR183C	YGR183C	S288C_Ator08_hmg124h_6.JPG.dat	635.3	428.4	0.8	0.07	0.018	0.072	2.7E-03
YGR184C	YGR184C	S288C_Ator08_hmg124h_6.JPG.dat	1115.3	44.1	1.0	0.01	0.078	0.011	3.5E-05
YGR187C	YGR187C	S288C_Ator08_hmg124h_6.JPG.dat	1073.0	25.0	1.0	0.02	0.025	0.024	4.2E-06
YGR188C	YGR188C	S288C_Ator08_hmg124h_6.JPG.dat	962.3	79.0	0.9	0.01	0.053	0.015	8.6E-05
YGR189C	YGR189C	S288C_Ator08_hmg124h_6.JPG.dat	1104.3	9.9	1.0	0.01	0.004	0.012	4.9E-07
YGR192C	YGR192C	S288C_Ator08_hmg124h_6.JPG.dat	1087.3	54.5	1.0	0.05	0.029	0.051	3.5E-05
YGR193C	YGR193C	S288C_Ator08_hmg124h_6.JPG.dat	1088.5	16.0	1.0	0.02	-0.017	0.021	2.2E-06
YGR194C	YGR194C	S288C_Ator08_hmg124h_6.JPG.dat	1081.5	46.3	1.0	0.04	-0.014	0.043	1.9E-05
YGR196C	YGR196C	S288C_Ator08_hmg124h_6.JPG.dat	1071.3	22.4	1.0	0.00	0.002	0.005	6.5E-06
YGR197C	YGR197C	S288C_Ator08_hmg124h_6.JPG.dat	1056.5	52.5	1.0	0.06	-0.014	0.056	5.0E-05
YGR199W	YGR199W	S288C_Ator08_hmg124h_6.JPG.dat	1094.5	49.8	1.0	0.04	0.003	0.042	1.8E-05
YGR200C	YGR200C	S288C_Ator08_hmg124h_6.JPG.dat	1033.5	64.0	1.0	0.06	0.111	0.060	6.6E-05
YGR201C	YGR201C	S288C_Ator08_hmg124h_6.JPG.dat	1121.5	54.0	1.0	0.05	0.015	0.049	3.1E-05
YGR202C	YGR202C	S288C_Ator08_hmg124h_6.JPG.dat	1154.3	38.6	1.0	0.04	0.017	0.036	1.1E-05
YGR203W	YGR203W	S288C_Ator08_hmg124h_6.JPG.dat	1118.8	50.1	1.0	0.04	0.020	0.043	2.1E-05
YGR205W	YGR205W	S288C_Ator08_hmg124h_6.JPG.dat	1099.8	23.0	1.0	0.02	-0.002	0.022	2.8E-06
YGR206W	YGR206W	S288C_Ator08_hmg124h_6.JPG.dat	1078.5	27.2	1.0	0.03	-0.013	0.026	5.0E-06
YGR207C	YGR207C	S288C_Ator08_hmg124h_6.JPG.dat	1063.3	37.6	1.0	0.01	0.017	0.005	8.7E-06
YGR208W	YGR208W	S288C_Ator08_hmg124h_6.JPG.dat	1078.0	60.3	1.0	0.01	0.017	0.006	1.1E-05
YGR209C	YGR209C	S288C_Ator08_hmg124h_6.JPG.dat	1047.3	21.2	1.0	0.02	-0.005	0.015	9.6E-07
YGR210C	YGR210C	S288C_Ator08_hmg124h_6.JPG.dat	1082.0	44.0	1.0	0.03	-0.033	0.032	8.7E-06
YGR212W	YGR212W	S288C_Ator08_hmg124h_6.JPG.dat	1114.3	115.7	1.1	0.10	0.034	0.102	2.4E-04
YGR213C	YGR213C	S288C_Ator08_hmg124h_6.JPG.dat	1101.3	27.6	1.0	0.03	0.024	0.027	4.8E-06
YGR214W	YGR214W	S288C_Ator08_hmg124h_6.JPG.dat	770.0	192.5	0.7	0.18	-0.018	0.178	4.1E-03
YGR217W	YGR217W	S288C_Ator08_hmg124h_6.JPG.dat	1000.0	33.7	0.9	0.04	-0.091	0.038	1.9E-05
YGR221C	YGR221C	S288C_Ator08_hmg124h_6.JPG.dat	1117.8	91.6	1.0	0.08	0.003	0.081	1.4E-04
YGR223C	YGR223C	S288C_Ator08_hmg124h_6.JPG.dat	1009.0	151.0	0.9	0.14	-0.058	0.138	9.3E-04
YGR224W	YGR224W	S288C_Ator08_hmg124h_6.JPG.dat	1084.3	51.9	1.0	0.01	0.020	0.010	3.4E-05
YGR225W	YGR225W	S288C_Ator08_hmg124h_6.JPG.dat	977.8	103.0	1.0	0.01	-0.032	0.006	1.4E-05
YGR226C	YGR226C	S288C_Ator08_hmg124h_6.JPG.dat	1010.0	106.8	1.0	0.03	-0.031	0.031	3.1E-04
YGR227W	YGR227W	S288C_Ator08_hmg124h_6.JPG.dat	1060.0	34.3	1.0	0.01	-0.017	0.007	1.6E-05
YGR228W	YGR228W	S288C_Ator08_hmg124h_6.JPG.dat	1031.0	9.0	1.0	0.00	-0.058	0.003	3.1E-06
YGR229C	YGR229C	S288C_Ator08_hmg124h_6.JPG.dat	856.5	199.2	0.8	0.19	-0.125	0.195	3.5E-03
YGR230W	YGR230W	S288C_Ator08_hmg124h_6.JPG.dat	1091.3	30.1	1.0	0.03	-0.024	0.029	6.2E-06
YGR231C	YGR231C	S288C_Ator08_hmg124h_6.JPG.dat	1041.8	69.7	1.0	0.01	0.099	0.013	5.9E-05
YGR232W	YGR232W	S288C_Ator08_hmg124h_6.JPG.dat	1101.5	30.7	1.0	0.03	0.006	0.030	7.0E-06
YGR233C	YGR233C	S288C_Ator08_hmg124h_6.JPG.dat	1154.0	30.0	1.0	0.03	0.026	0.031	7.3E-06
YGR234W	YGR234W	S288C_Ator08_hmg124h_6.JPG.dat	1066.8	144.8	1.0	0.01	0.038	0.012	4.3E-05
YGR235C	YGR235C	S288C_Ator08_hmg124h_6.JPG.dat	1101.8	33.3	1.0	0.01	-0.003	0.009	2.6E-05
YGR236C	YGR236C	S288C_Ator08_hmg124h_6.JPG.dat	1056.5	38.6	1.0	0.03	-0.011	0.032	8.7E-06
YGR237C	YGR237C	S288C_Ator08_hmg124h_6.JPG.dat	1047.0	26.8	1.0	0.01	0.019	0.006	9.9E-06
YGR238C	YGR238C	S288C_Ator08_hmg124h_6.JPG.dat	1033.8	45.0	1.0	0.03	-0.010	0.033	1.1E-05
YGR239C	YGR239C	S288C_Ator08_hmg124h_6.JPG.dat	1059.3	10.2	1.0	0.01	-0.005	0.014	7.2E-07

YGR241C	YGR241C	S288C_Ator08_hmg124h_6.JPG.dat	1077.5	32.3	1.0	0.03	0.035	0.031	7.1E-06
YGR242W	YGR242W	S288C_Ator08_hmg124h_6.JPG.dat	1046.3	53.7	1.0	0.05	-0.001	0.052	4.2E-05
YGR243W	YGR243W	S288C_Ator08_hmg124h_6.JPG.dat	1144.5	51.5	1.0	0.04	0.026	0.044	2.0E-05
YGR244C	YGR244C	S288C_Ator08_hmg124h_6.JPG.dat	1157.8	38.2	1.0	0.03	0.011	0.032	7.9E-06
YGR247W	YGR247W	S288C_Ator08_hmg124h_6.JPG.dat	1100.0	31.8	1.0	0.03	-0.007	0.031	8.3E-06
YGR248W	YGR248W	S288C_Ator08_hmg124h_6.JPG.dat	1013.3	172.8	1.0	0.03	-0.019	0.034	3.9E-04
YGR249W	YGR249W	S288C_Ator08_hmg124h_6.JPG.dat	1100.0	13.6	1.0	0.01	-0.009	0.015	8.1E-07
YGR250C	YGR250C	S288C_Ator08_hmg124h_6.JPG.dat	1025.5	91.3	1.0	0.08	-0.025	0.078	1.4E-04
YGR256W	YGR256W	S288C_Ator08_hmg124h_6.JPG.dat	990.5	55.1	1.0	0.01	0.042	0.010	3.5E-05
YGR259C	YGR259C	S288C_Ator08_hmg124h_6.JPG.dat	951.8	41.0	0.9	0.02	-0.060	0.024	5.7E-06
YGR260W	YGR260W	S288C_Ator08_hmg124h_6.JPG.dat	1017.5	39.3	1.0	0.01	0.125	0.014	6.9E-05
YGR261C	YGR261C	S288C_Ator08_hmg124h_6.JPG.dat	1012.3	42.8	1.0	0.04	-0.024	0.039	1.7E-05
YGR263C	YGR263C	S288C_Ator08_hmg124h_6.JPG.dat	1084.8	27.8	1.0	0.01	-0.026	0.011	3.6E-07
YGR266W	YGR266W	S288C_Ator08_hmg124h_6.JPG.dat	1106.5	20.9	1.0	0.02	0.011	0.022	2.7E-06
YGR268C	YGR268C	S288C_Ator08_hmg124h_6.JPG.dat	1066.3	96.0	1.0	0.07	-0.014	0.072	1.1E-04
YGR269W	YGR269W	S288C_Ator08_hmg124h_6.JPG.dat	1099.8	29.3	1.0	0.05	-0.006	0.046	2.6E-05
YGR270W	YGR270W	S288C_Ator08_hmg124h_6.JPG.dat	927.3	62.6	0.9	0.05	0.010	0.054	6.9E-05
YGR275W	YGR275W	S288C_Ator08_hmg124h_6.JPG.dat	1082.8	50.9	1.0	0.01	-0.032	0.007	1.9E-05
YGR279C	YGR279C	S288C_Ator08_hmg124h_6.JPG.dat	1075.5	23.1	1.0	0.04	-0.027	0.036	1.3E-05
YGR281W	YGR281W	S288C_Ator08_hmg124h_6.JPG.dat	498.3	9.6	0.5	0.02	-0.538	0.017	1.3E-05
YGR282C	YGR282C	S288C_Ator08_hmg124h_6.JPG.dat	1069.5	49.1	1.0	0.03	0.109	0.033	1.1E-05
YGR283C	YGR283C	S288C_Ator08_hmg124h_6.JPG.dat	1136.8	26.2	1.0	0.02	0.030	0.025	4.3E-06
YGR284C	YGR284C	S288C_Ator08_hmg124h_6.JPG.dat	1118.0	14.5	1.0	0.01	0.009	0.010	2.4E-07
YGR286C	YGR286C	S288C_Ator08_hmg124h_6.JPG.dat	1118.3	58.8	1.0	0.05	0.019	0.047	2.8E-05
YGR287C	YGR287C	S288C_Ator08_hmg124h_6.JPG.dat	1127.3	65.7	1.0	0.06	0.012	0.059	5.1E-05
YGR288W	YGR288W	S288C_Ator08_hmg124h_6.JPG.dat	1141.0	107.8	1.0	0.04	-0.028	0.038	4.8E-04
YGR290W	YGR290W	S288C_Ator08_hmg124h_6.JPG.dat	1057.3	10.0	1.0	0.01	-0.062	0.009	2.2E-07
YGR295C	YGR295C	S288C_Ator08_hmg124h_6.JPG.dat	1094.8	27.6	1.0	0.03	-0.018	0.026	4.8E-06
YHL002W	YHL002W	S288C_Ator08_hmg124h_6.JPG.dat	1133.3	37.4	1.0	0.03	-0.005	0.034	1.0E-05
YHL003C	YHL003C	S288C_Ator08_hmg124h_6.JPG.dat	991.8	98.8	0.9	0.09	-0.056	0.091	2.7E-04
YHL005C	YHL005C	S288C_Ator08_hmg124h_6.JPG.dat	957.0	123.6	0.9	0.11	-0.038	0.114	5.6E-04
YHL006C	YHL006C	S288C_Ator08_hmg124h_6.JPG.dat	1035.5	69.6	1.0	0.01	-0.007	0.014	6.5E-05
YHL007C	YHL007C	S288C_Ator08_hmg124h_6.JPG.dat	425.0	497.4	0.4	0.46	0.016	0.457	1.9E-01
YHL008C	YHL008C	S288C_Ator08_hmg124h_6.JPG.dat	989.0	46.0	1.0	0.05	-0.050	0.047	3.1E-05
YHL009C	YHL009C	S288C_Ator08_hmg124h_6.JPG.dat	1000.5	64.0	1.0	0.02	-0.050	0.021	1.5E-04
YHL010C	YHL010C	S288C_Ator08_hmg124h_6.JPG.dat	1035.3	117.8	1.0	0.10	-0.079	0.104	3.4E-04
YHL012W	YHL012W	S288C_Ator08_hmg124h_6.JPG.dat	1108.3	4.9	1.0	0.00	0.003	0.001	2.3E-07
YHL013C	YHL013C	S288C_Ator08_hmg124h_6.JPG.dat	1102.8	125.5	1.0	0.12	0.059	0.116	4.0E-04
YHL014C	YHL014C	S288C_Ator08_hmg124h_6.JPG.dat	1062.0	53.6	1.0	0.05	-0.003	0.046	2.9E-05
YHL016C	YHL016C	S288C_Ator08_hmg124h_6.JPG.dat	1057.0	54.0	1.0	0.05	-0.015	0.051	3.8E-05
YHL017W	YHL017W	S288C_Ator08_hmg124h_6.JPG.dat	1077.5	81.0	1.0	0.07	0.015	0.075	1.1E-04
YHL019C	YHL019C	S288C_Ator08_hmg124h_6.JPG.dat	989.5	91.9	1.0	0.09	-0.019	0.091	2.3E-04
YHL020C	YHL020C	S288C_Ator08_hmg124h_6.JPG.dat	1004.0	10.4	1.0	0.01	-0.004	0.010	2.9E-07
YHL021C	YHL021C	S288C_Ator08_hmg124h_6.JPG.dat	1086.0	44.0	1.0	0.04	-0.075	0.042	2.0E-05
YHL022C	YHL022C	S288C_Ator08_hmg124h_6.JPG.dat	1029.0	70.7	1.0	0.01	-0.022	0.010	3.5E-05
YHL023C	YHL023C	S288C_Ator08_hmg124h_6.JPG.dat	866.3	239.0	0.8	0.23	-0.019	0.226	5.7E-03
YHL024W	YHL024W	S288C_Ator08_hmg124h_6.JPG.dat	1075.3	59.2	1.0	0.06	-0.015	0.056	5.0E-05
YHL025W	YHL025W	S288C_Ator08_hmg124h_6.JPG.dat	565.0	244.3	0.4	0.04	-0.198	0.044	3.8E-03
YHL026C	YHL026C	S288C_Ator08_hmg124h_6.JPG.dat	1036.5	89.5	0.9	0.08	-0.025	0.082	1.8E-04
YHL027W	YHL027W	S288C_Ator08_hmg124h_6.JPG.dat	963.8	143.5	0.9	0.13	-0.029	0.131	9.0E-04
YHL028W	YHL028W	S288C_Ator08_hmg124h_6.JPG.dat	1123.0	36.7	1.0	0.03	0.017	0.034	1.0E-05
YHL029C	YHL029C	S288C_Ator08_hmg124h_6.JPG.dat	1036.3	36.5	1.0	0.03	0.024	0.035	1.3E-05
YHL030W	YHL030W	S288C_Ator08_hmg124h_6.JPG.dat	1073.3	34.7	1.0	0.03	0.012	0.033	9.1E-06
YHL031C	YHL031C	S288C_Ator08_hmg124h_6.JPG.dat	707.5	78.8	0.7	0.07	-0.023	0.073	3.2E-04
YHL032C	YHL032C	S288C_Ator08_hmg124h_6.JPG.dat	1070.8	50.5	1.0	0.05	-0.064	0.050	3.6E-05
YHL033C	YHL033C	S288C_Ator08_hmg124h_6.JPG.dat	1057.8	76.4	1.1	0.01	0.222	0.007	1.6E-05
YHL034C	YHL034C	S288C_Ator08_hmg124h_6.JPG.dat	965.0	92.6	0.9	0.09	-0.007	0.086	2.4E-04
YHL035C	YHL035C	S288C_Ator08_hmg124h_6.JPG.dat	1041.8	48.4	1.0	0.04	-0.012	0.038	1.7E-05
YHL036W	YHL036W	S288C_Ator08_hmg124h_6.JPG.dat	1067.0	128.5	1.0	0.03	0.053	0.031	2.9E-04
YHL037C	YHL037C	S288C_Ator08_hmg124h_6.JPG.dat	1029.3	191.4	0.9	0.18	-0.079	0.181	1.9E-03
YHL039W	YHL039W	S288C_Ator08_hmg124h_6.JPG.dat	1088.0	45.6	1.0	0.04	-0.013	0.038	1.5E-05
YHL040C	YHL040C	S288C_Ator08_hmg124h_6.JPG.dat	744.0	517.7	0.9	0.17	-0.067	0.173	1.2E-02
YHL041W	YHL041W	S288C_Ator08_hmg124h_6.JPG.dat	921.8	146.8	0.9	0.14	-0.056	0.144	1.2E-03
YHL042W	YHL042W	S288C_Ator08_hmg124h_6.JPG.dat	990.5	119.2	1.0	0.02	-0.005	0.018	1.0E-04
YHL043W	YHL043W	S288C_Ator08_hmg124h_6.JPG.dat	1023.8	20.5	1.0	0.02	-0.004	0.022	2.8E-06
YHL044W	YHL044W	S288C_Ator08_hmg124h_6.JPG.dat	1055.8	78.0	1.0	0.06	-0.086	0.061	6.9E-05
YHL045W	YHL045W	S288C_Ator08_hmg124h_6.JPG.dat	1030.0	80.2	1.0	0.08	-0.080	0.078	1.3E-04
YHL046C	YHL046C	S288C_Ator08_hmg124h_6.JPG.dat	1108.0	29.3	1.0	0.02	-0.003	0.021	2.3E-06
YHL047C	YHL047C	S288C_Ator08_hmg124h_6.JPG.dat	1014.5	130.8	1.0	0.04	0.006	0.039	5.1E-04
YHR001W-	YHR001W-A	S288C_Ator08_hmg124h_6.JPG.dat	1063.3	23.7	1.0	0.02	-0.032	0.022	3.2E-06

YHR003C	YHR003C	S288C_Ator08_hmg124h_6.JPG.dat	1096.0	48.9	1.0	0.04	0.013	0.045	2.6E-05
YHR004C	YHR004C	S288C_Ator08_hmg124h_6.JPG.dat	1044.5	194.5	1.0	0.02	0.038	0.020	1.3E-04
YHR005C	YHR005C	S288C_Ator08_hmg124h_6.JPG.dat	1049.0	103.6	1.0	0.10	-0.045	0.096	2.8E-04
YHR006W	YHR006W	S288C_Ator08_hmg124h_6.JPG.dat	1110.5	23.4	1.0	0.02	-0.023	0.021	2.2E-06
YHR009C	YHR009C	S288C_Ator08_hmg124h_6.JPG.dat	1068.0	15.0	1.0	0.00	0.014	0.003	2.6E-06
YHR012W	YHR012W	S288C_Ator08_hmg124h_6.JPG.dat	747.8	257.6	0.9	0.02	0.063	0.018	1.4E-04
YHR014W	YHR014W	S288C_Ator08_hmg124h_6.JPG.dat	1072.0	48.0	1.0	0.03	-0.026	0.034	1.1E-05
YHR015W	YHR015W	S288C_Ator08_hmg124h_6.JPG.dat	1021.8	72.4	1.0	0.07	-0.009	0.073	1.1E-04
YHR016C	YHR016C	S288C_Ator08_hmg124h_6.JPG.dat	1063.5	28.1	1.0	0.01	-0.015	0.007	1.7E-05
YHR017W	YHR017W	S288C_Ator08_hmg124h_6.JPG.dat	1102.5	28.5	1.0	0.03	0.042	0.027	4.9E-06
YHR018C	YHR018C	S288C_Ator08_hmg124h_6.JPG.dat	0.0	0.0	0.0	0.00	0.000	0.000	
YHR021C	YHR021C	S288C_Ator08_hmg124h_6.JPG.dat	722.3	271.8	0.5	0.08	-0.026	0.076	6.6E-03
YHR021W-	YHR021W-A	S288C_Ator08_hmg124h_6.JPG.dat	844.8	307.5	0.8	0.28	-0.117	0.277	1.2E-02
YHR022C	YHR022C	S288C_Ator08_hmg124h_6.JPG.dat	993.3	198.0	1.0	0.02	0.014	0.017	1.0E-04
YHR025W	YHR025W	S288C_Ator08_hmg124h_6.JPG.dat	593.8	52.9	0.5	0.01	0.066	0.012	1.7E-04
YHR028C	YHR028C	S288C_Ator08_hmg124h_6.JPG.dat	996.0	6.9	1.0	0.00	-0.034	0.001	2.8E-07
YHR029C	YHR029C	S288C_Ator08_hmg124h_6.JPG.dat	1036.5	28.5	1.0	0.03	-0.048	0.029	6.4E-06
YHR030C	YHR030C	S288C_Ator08_hmg124h_6.JPG.dat	735.5	371.2	0.5	0.02	-0.520	0.019	4.6E-04
YHR031C	YHR031C	S288C_Ator08_hmg124h_6.JPG.dat	1122.5	30.0	1.1	0.05	0.061	0.049	2.5E-05
YHR032W	YHR032W	S288C_Ator08_hmg124h_6.JPG.dat	1086.8	32.3	1.0	0.03	-0.004	0.029	6.6E-06
YHR033W	YHR033W	S288C_Ator08_hmg124h_6.JPG.dat	1086.3	33.2	1.0	0.04	0.002	0.037	1.3E-05
YHR034C	YHR034C	S288C_Ator08_hmg124h_6.JPG.dat	1110.8	59.0	1.0	0.05	0.064	0.052	3.5E-05
YHR035W	YHR035W	S288C_Ator08_hmg124h_6.JPG.dat	1130.0	19.0	1.1	0.01	0.055	0.007	8.8E-08
YHR037W	YHR037W	S288C_Ator08_hmg124h_6.JPG.dat	1112.5	7.9	1.0	0.02	0.011	0.018	1.5E-06
YHR039C	YHR039C	S288C_Ator08_hmg124h_6.JPG.dat	1114.0	32.9	1.0	0.01	0.034	0.011	3.1E-07
YHR041C	YHR041C	S288C_Ator08_hmg124h_6.JPG.dat	1050.5	48.0	1.0	0.03	0.005	0.028	6.3E-06
YHR043C	YHR043C	S288C_Ator08_hmg124h_6.JPG.dat	912.8	32.8	0.9	0.02	-0.065	0.023	5.0E-06
YHR044C	YHR044C	S288C_Ator08_hmg124h_6.JPG.dat	1019.0	55.8	0.9	0.04	-0.036	0.037	1.7E-05
YHR045W	YHR045W	S288C_Ator08_hmg124h_6.JPG.dat	1188.8	56.7	1.1	0.04	0.065	0.040	1.5E-05
YHR046C	YHR046C	S288C_Ator08_hmg124h_6.JPG.dat	1100.3	45.5	1.0	0.04	-0.051	0.044	2.4E-05
YHR047C	YHR047C	S288C_Ator08_hmg124h_6.JPG.dat	1143.5	8.1	1.0	0.02	0.035	0.025	3.9E-06
YHR048W	YHR048W	S288C_Ator08_hmg124h_6.JPG.dat	1112.5	41.3	1.0	0.04	-0.019	0.038	1.6E-05
YHR049C-/	YHR049C-A	S288C_Ator08_hmg124h_6.JPG.dat	1077.5	24.5	1.0	0.01	-0.040	0.012	5.5E-07
YHR049W	YHR049W	S288C_Ator08_hmg124h_6.JPG.dat	1148.8	33.7	1.0	0.02	0.020	0.018	1.5E-06
YHR050W	YHR050W	S288C_Ator08_hmg124h_6.JPG.dat	1188.3	84.8	1.0	0.02	-0.031	0.017	9.0E-05
YHR057C	YHR057C	S288C_Ator08_hmg124h_6.JPG.dat	1118.3	27.6	1.0	0.02	-0.024	0.023	3.1E-06
YHR059W	YHR059W	S288C_Ator08_hmg124h_6.JPG.dat	222.0	444.0	0.0	0.00	0.000	0.000	
YHR061C	YHR061C	S288C_Ator08_hmg124h_6.JPG.dat	1061.0	114.0	1.0	0.01	0.049	0.015	7.2E-05
YHR066W	YHR066W	S288C_Ator08_hmg124h_6.JPG.dat	979.3	17.5	0.9	0.02	-0.030	0.019	2.8E-06
YHR067W	YHR067W	S288C_Ator08_hmg124h_6.JPG.dat	770.0	173.8	0.7	0.16	0.056	0.157	2.9E-03
YHR073W	YHR073W	S288C_Ator08_hmg124h_6.JPG.dat	906.8	34.5	0.9	0.03	-0.126	0.034	1.7E-05
YHR075C	YHR075C	S288C_Ator08_hmg124h_6.JPG.dat	1084.8	35.0	1.0	0.03	-0.033	0.034	1.1E-05
YHR076W	YHR076W	S288C_Ator08_hmg124h_6.JPG.dat	1107.3	51.6	1.0	0.05	-0.004	0.048	3.0E-05
YHR077C	YHR077C	S288C_Ator08_hmg124h_6.JPG.dat	1176.3	70.0	1.1	0.01	0.067	0.008	1.7E-05
YHR078W	YHR078W	S288C_Ator08_hmg124h_6.JPG.dat	1181.3	66.6	1.1	0.06	-0.030	0.063	5.7E-05
YHR079C	YHR079C	S288C_Ator08_hmg124h_6.JPG.dat	880.0	55.2	0.8	0.05	-0.149	0.050	6.6E-05
YHR079C-E	YHR079C-B	S288C_Ator08_hmg124h_6.JPG.dat	1078.3	26.9	1.0	0.01	-0.049	0.008	2.2E-05
YHR080C	YHR080C	S288C_Ator08_hmg124h_6.JPG.dat	1085.3	13.4	1.0	0.01	0.006	0.014	7.5E-07
YHR081W	YHR081W	S288C_Ator08_hmg124h_6.JPG.dat	821.5	190.1	0.8	0.18	0.090	0.178	3.3E-03
YHR082C	YHR082C	S288C_Ator08_hmg124h_6.JPG.dat	1150.8	24.0	1.1	0.01	0.041	0.013	5.0E-07
YHR086W	YHR086W	S288C_Ator08_hmg124h_6.JPG.dat	1092.0	20.5	1.0	0.02	0.029	0.025	4.0E-06
YHR087W	YHR087W	S288C_Ator08_hmg124h_6.JPG.dat	1078.3	71.5	1.0	0.06	0.005	0.058	4.9E-05
YHR092C	YHR092C	S288C_Ator08_hmg124h_6.JPG.dat	1157.8	44.7	1.1	0.01	0.061	0.013	4.6E-05
YHR093W	YHR093W	S288C_Ator08_hmg124h_6.JPG.dat	1145.8	46.6	1.0	0.04	0.006	0.036	1.2E-05
YHR094C	YHR094C	S288C_Ator08_hmg124h_6.JPG.dat	1123.5	21.2	1.0	0.02	0.010	0.019	1.8E-06
YHR095W	YHR095W	S288C_Ator08_hmg124h_6.JPG.dat	1127.0	9.0	1.0	0.01	0.008	0.013	6.1E-07
YHR096C	YHR096C	S288C_Ator08_hmg124h_6.JPG.dat	1108.8	43.1	1.0	0.04	-0.027	0.039	1.6E-05
YHR097C	YHR097C	S288C_Ator08_hmg124h_6.JPG.dat	1093.0	34.4	1.0	0.03	-0.008	0.028	6.2E-06
YHR103W	YHR103W	S288C_Ator08_hmg124h_6.JPG.dat	1040.3	30.3	1.0	0.03	-0.020	0.032	9.4E-06
YHR104W	YHR104W	S288C_Ator08_hmg124h_6.JPG.dat	1035.8	30.5	1.0	0.03	-0.046	0.028	6.7E-06
YHR105W	YHR105W	S288C_Ator08_hmg124h_6.JPG.dat	1052.5	33.8	1.0	0.03	0.005	0.025	4.4E-06
YHR106W	YHR106W	S288C_Ator08_hmg124h_6.JPG.dat	1003.3	68.9	1.0	0.06	-0.072	0.060	6.8E-05
YHR108W	YHR108W	S288C_Ator08_hmg124h_6.JPG.dat	977.0	38.8	0.9	0.04	-0.044	0.042	2.5E-05
YHR109W	YHR109W	S288C_Ator08_hmg124h_6.JPG.dat	1085.8	88.3	1.0	0.09	0.034	0.085	1.6E-04
YHR110W	YHR110W	S288C_Ator08_hmg124h_6.JPG.dat	1068.0	120.0	1.0	0.04	0.046	0.042	5.4E-04
YHR111W	YHR111W	S288C_Ator08_hmg124h_6.JPG.dat	1144.8	85.9	1.1	0.09	0.010	0.085	1.5E-04
YHR112C	YHR112C	S288C_Ator08_hmg124h_6.JPG.dat	1105.3	134.7	1.0	0.12	0.000	0.118	4.4E-04
YHR113W	YHR113W	S288C_Ator08_hmg124h_6.JPG.dat	1088.3	20.2	1.0	0.02	-0.006	0.021	2.5E-06
YHR114W	YHR114W	S288C_Ator08_hmg124h_6.JPG.dat	1120.3	13.9	1.0	0.01	0.031	0.015	8.1E-07
YHR115C	YHR115C	S288C_Ator08_hmg124h_6.JPG.dat	1039.8	19.2	1.0	0.01	0.010	0.014	7.3E-07

YHR116W	YHR116W	S288C_Ator08_hmg124h_6.JPG.dat	142.8	285.5	0.0	0.00	0.000	0.000	
YHR117W	YHR117W	S288C_Ator08_hmg124h_6.JPG.dat	1012.8	22.2	1.0	0.02	0.009	0.018	1.8E-06
YHR121W	YHR121W	S288C_Ator08_hmg124h_6.JPG.dat	1103.3	9.9	1.0	0.01	-0.014	0.005	8.6E-06
YHR123W	YHR123W	S288C_Ator08_hmg124h_6.JPG.dat	1122.3	36.5	1.1	0.04	0.008	0.036	1.0E-05
YHR124W	YHR124W	S288C_Ator08_hmg124h_6.JPG.dat	1053.3	83.8	1.0	0.01	-0.003	0.009	2.4E-05
YHR125W	YHR125W	S288C_Ator08_hmg124h_6.JPG.dat	1052.3	38.4	1.0	0.03	-0.049	0.029	7.2E-06
YHR126C	YHR126C	S288C_Ator08_hmg124h_6.JPG.dat	1041.8	86.3	0.9	0.08	-0.008	0.080	1.7E-04
YHR127W	YHR127W	S288C_Ator08_hmg124h_6.JPG.dat	1133.5	10.7	1.0	0.02	0.004	0.016	1.2E-06
YHR129C	YHR129C	S288C_Ator08_hmg124h_6.JPG.dat	1063.0	79.3	1.0	0.03	-0.011	0.028	2.7E-04
YHR130C	YHR130C	S288C_Ator08_hmg124h_6.JPG.dat	969.8	87.0	0.9	0.08	-0.065	0.084	2.3E-04
YHR131C	YHR131C	S288C_Ator08_hmg124h_6.JPG.dat	1061.8	32.4	1.0	0.01	-0.004	0.007	1.7E-05
YHR132C	YHR132C	S288C_Ator08_hmg124h_6.JPG.dat	965.5	105.7	1.0	0.02	0.027	0.019	1.3E-04
YHR132W-	YHR132W-A	S288C_Ator08_hmg124h_6.JPG.dat	1060.8	21.3	1.0	0.02	0.013	0.019	1.7E-06
YHR133C	YHR133C	S288C_Ator08_hmg124h_6.JPG.dat	875.3	163.9	0.8	0.16	-0.157	0.159	1.9E-03
YHR134W	YHR134W	S288C_Ator08_hmg124h_6.JPG.dat	939.8	28.6	0.9	0.03	0.019	0.030	1.1E-05
YHR135C	YHR135C	S288C_Ator08_hmg124h_6.JPG.dat	1076.8	80.1	1.0	0.01	0.043	0.013	5.5E-05
YHR136C	YHR136C	S288C_Ator08_hmg124h_6.JPG.dat	1126.0	28.9	1.0	0.02	0.026	0.020	2.2E-06
YHR137W	YHR137W	S288C_Ator08_hmg124h_6.JPG.dat	1116.3	23.7	1.0	0.03	-0.020	0.026	4.7E-06
YHR138C	YHR138C	S288C_Ator08_hmg124h_6.JPG.dat	1136.8	28.2	1.0	0.03	-0.008	0.030	7.4E-06
YHR139C	YHR139C	S288C_Ator08_hmg124h_6.JPG.dat	1161.3	11.6	1.0	0.01	0.039	0.013	5.6E-07
YHR139C-/	YHR139C-A	S288C_Ator08_hmg124h_6.JPG.dat	1097.3	32.0	1.0	0.00	-0.004	0.000	1.6E-08
YHR140W	YHR140W	S288C_Ator08_hmg124h_6.JPG.dat	1071.0	33.1	1.0	0.03	0.037	0.030	7.1E-06
YHR142W	YHR142W	S288C_Ator08_hmg124h_6.JPG.dat	1045.5	38.9	1.0	0.04	-0.062	0.037	1.4E-05
YHR143W	YHR143W	S288C_Ator08_hmg124h_6.JPG.dat	1082.0	29.0	1.0	0.00	-0.057	0.004	4.2E-06
YHR146W	YHR146W	S288C_Ator08_hmg124h_6.JPG.dat	1075.5	47.0	1.0	0.01	0.021	0.012	4.9E-05
YHR150W	YHR150W	S288C_Ator08_hmg124h_6.JPG.dat	1040.8	33.9	1.0	0.03	0.009	0.025	4.2E-06
YHR151C	YHR151C	S288C_Ator08_hmg124h_6.JPG.dat	1024.3	79.1	1.0	0.02	0.014	0.021	1.5E-04
YHR152W	YHR152W	S288C_Ator08_hmg124h_6.JPG.dat	1105.5	10.0	1.0	0.01	0.003	0.008	1.3E-07
YHR153C	YHR153C	S288C_Ator08_hmg124h_6.JPG.dat	1107.0	27.5	1.0	0.03	0.060	0.025	4.0E-06
YHR154W	YHR154W	S288C_Ator08_hmg124h_6.JPG.dat	1109.0	12.2	1.0	0.01	0.014	0.011	3.2E-07
YHR155W	YHR155W	S288C_Ator08_hmg124h_6.JPG.dat	1099.3	8.8	1.0	0.01	-0.005	0.010	2.9E-07
YHR156C	YHR156C	S288C_Ator08_hmg124h_6.JPG.dat	1055.5	21.1	1.0	0.01	-0.015	0.010	3.6E-05
YHR157W	YHR157W	S288C_Ator08_hmg124h_6.JPG.dat	1013.8	36.9	1.0	0.03	-0.033	0.028	6.8E-06
YHR158C	YHR158C	S288C_Ator08_hmg124h_6.JPG.dat	1032.3	20.2	1.0	0.02	-0.067	0.020	2.1E-06
YHR159W	YHR159W	S288C_Ator08_hmg124h_6.JPG.dat	937.5	134.2	0.9	0.12	-0.043	0.123	7.8E-04
YHR160C	YHR160C	S288C_Ator08_hmg124h_6.JPG.dat	1166.8	70.5	1.0	0.02	0.014	0.017	9.7E-05
YHR161C	YHR161C	S288C_Ator08_hmg124h_6.JPG.dat	1127.5	38.4	1.0	0.00	0.013	0.003	2.2E-06
YHR162W	YHR162W	S288C_Ator08_hmg124h_6.JPG.dat	1130.5	14.2	1.0	0.03	0.025	0.028	6.0E-06
YHR163W	YHR163W	S288C_Ator08_hmg124h_6.JPG.dat	1098.5	41.6	1.0	0.02	0.015	0.015	7.6E-05
YHR167W	YHR167W	S288C_Ator08_hmg124h_6.JPG.dat	1078.5	50.8	1.0	0.05	0.001	0.047	3.2E-05
YHR171W	YHR171W	S288C_Ator08_hmg124h_6.JPG.dat	985.8	81.4	0.9	0.02	-0.104	0.022	2.3E-04
YHR176W	YHR176W	S288C_Ator08_hmg124h_6.JPG.dat	1122.8	23.7	1.0	0.03	-0.015	0.032	8.4E-06
YHR178W	YHR178W	S288C_Ator08_hmg124h_6.JPG.dat	964.8	128.6	0.9	0.11	0.063	0.109	5.3E-04
YHR179W	YHR179W	S288C_Ator08_hmg124h_6.JPG.dat	1119.8	33.5	1.0	0.03	0.003	0.027	4.9E-06
YHR180W	YHR180W	S288C_Ator08_hmg124h_6.JPG.dat	1087.0	29.5	1.0	0.02	0.006	0.016	1.1E-06
YHR181W	YHR181W	S288C_Ator08_hmg124h_6.JPG.dat	1002.0	134.0	1.0	0.01	0.001	0.010	3.5E-05
YHR182W	YHR182W	S288C_Ator08_hmg124h_6.JPG.dat	1150.0	29.7	1.0	0.01	0.002	0.015	8.9E-07
YHR184W	YHR184W	S288C_Ator08_hmg124h_6.JPG.dat	1033.5	70.4	1.0	0.07	-0.049	0.066	8.7E-05
YHR185C	YHR185C	S288C_Ator08_hmg124h_6.JPG.dat	1076.5	65.6	1.0	0.02	0.004	0.024	1.8E-04
YHR189W	YHR189W	S288C_Ator08_hmg124h_6.JPG.dat	352.3	144.9	0.4	0.05	-0.198	0.048	5.2E-03
YHR191C	YHR191C	S288C_Ator08_hmg124h_6.JPG.dat	1040.8	55.3	0.9	0.01	-0.050	0.007	1.8E-05
YHR193C	YHR193C	S288C_Ator08_hmg124h_6.JPG.dat	266.0	532.0	0.0	0.00	0.000	0.000	
YHR194W	YHR194W	S288C_Ator08_hmg124h_6.JPG.dat	509.0	599.2	0.5	0.55	-0.035	0.545	1.9E-01
YHR195W	YHR195W	S288C_Ator08_hmg124h_6.JPG.dat	1117.8	57.2	1.0	0.05	0.003	0.053	4.0E-05
YHR198C	YHR198C	S288C_Ator08_hmg124h_6.JPG.dat	824.8	199.4	0.8	0.18	-0.147	0.184	3.7E-03
YHR199C	YHR199C	S288C_Ator08_hmg124h_6.JPG.dat	1049.3	96.2	1.0	0.00	0.034	0.003	2.0E-06
YHR200W	YHR200W	S288C_Ator08_hmg124h_6.JPG.dat	1118.0	11.5	1.0	0.00	0.112	0.002	7.9E-07
YHR202W	YHR202W	S288C_Ator08_hmg124h_6.JPG.dat	1109.0	52.2	1.0	0.06	0.076	0.058	5.5E-05
YHR203C	YHR203C	S288C_Ator08_hmg124h_6.JPG.dat	893.8	43.8	0.9	0.04	0.072	0.039	2.6E-05
YHR204W	YHR204W	S288C_Ator08_hmg124h_6.JPG.dat	1128.0	44.0	1.0	0.04	0.012	0.041	1.7E-05
YHR206W	YHR206W	S288C_Ator08_hmg124h_6.JPG.dat	1150.3	15.3	1.1	0.01	0.068	0.014	7.1E-07
YHR207C	YHR207C	S288C_Ator08_hmg124h_6.JPG.dat	1104.8	1.9	1.0	0.01	-0.029	0.007	9.2E-08
YHR209W	YHR209W	S288C_Ator08_hmg124h_6.JPG.dat	1085.5	26.4	1.0	0.02	-0.030	0.022	2.9E-06
YHR210C	YHR210C	S288C_Ator08_hmg124h_6.JPG.dat	1060.5	35.5	1.0	0.03	-0.045	0.027	6.2E-06
YIL001W	YIL001W	S288C_Ator08_hmg124h_6.JPG.dat	1109.5	19.8	1.0	0.01	0.003	0.006	1.1E-05
YIL002C	YIL002C	S288C_Ator08_hmg124h_6.JPG.dat	1028.8	46.7	1.0	0.04	-0.045	0.044	2.7E-05
YIL005W	YIL005W	S288C_Ator08_hmg124h_6.JPG.dat	1019.5	51.3	1.0	0.05	-0.001	0.054	4.7E-05
YIL006W	YIL006W	S288C_Ator08_hmg124h_6.JPG.dat	1065.0	33.9	1.0	0.03	0.075	0.030	6.9E-06
YIL007C	YIL007C	S288C_Ator08_hmg124h_6.JPG.dat	1048.8	21.2	1.0	0.01	-0.041	0.006	1.3E-05
YIL008W	YIL008W	S288C_Ator08_hmg124h_6.JPG.dat	975.0	93.9	0.9	0.09	0.046	0.091	2.4E-04

YIL009C-A	YIL009C-A	S288C_Ator08_hmg124h_6.JPG.dat	1195.0	19.2	1.1	0.00	0.101	0.004	3.3E-06
YIL010W	YIL010W	S288C_Ator08_hmg124h_6.JPG.dat	1074.0	24.9	1.0	0.02	-0.006	0.022	3.0E-06
YIL011W	YIL011W	S288C_Ator08_hmg124h_6.JPG.dat	411.8	490.1	0.4	0.45	0.062	0.450	1.9E-01
YIL012W	YIL012W	S288C_Ator08_hmg124h_6.JPG.dat	300.8	601.5	0.0	0.00	0.000	0.000	
YIL013C	YIL013C	S288C_Ator08_hmg124h_6.JPG.dat	1077.0	91.4	1.0	0.08	0.009	0.081	1.5E-04
YIL014W	YIL014W	S288C_Ator08_hmg124h_6.JPG.dat	1082.5	42.5	1.0	0.04	-0.037	0.036	1.3E-05
YIL015C-A	YIL015C-A	S288C_Ator08_hmg124h_6.JPG.dat	996.8	114.3	1.0	0.02	0.015	0.019	1.2E-04
YIL015W	YIL015W	S288C_Ator08_hmg124h_6.JPG.dat	1051.3	60.0	1.0	0.06	0.006	0.057	4.7E-05
YIL016W	YIL016W	S288C_Ator08_hmg124h_6.JPG.dat	1097.8	36.5	1.1	0.03	0.008	0.029	5.1E-06
YIL017C	YIL017C	S288C_Ator08_hmg124h_6.JPG.dat	1077.3	49.9	1.0	0.05	0.000	0.053	4.4E-05
YIL020C	YIL020C	S288C_Ator08_hmg124h_6.JPG.dat	0.0	0.0	0.0	0.00	0.000	0.000	
YIL023C	YIL023C	S288C_Ator08_hmg124h_6.JPG.dat	1137.5	42.5	1.0	0.03	0.020	0.031	7.2E-06
YIL024C	YIL024C	S288C_Ator08_hmg124h_6.JPG.dat	1059.0	8.3	1.0	0.00	-0.005	0.002	2.1E-06
YIL025C	YIL025C	S288C_Ator08_hmg124h_6.JPG.dat	1091.0	23.3	1.0	0.03	-0.008	0.028	6.2E-06
YIL027C	YIL027C	S288C_Ator08_hmg124h_6.JPG.dat	1073.3	38.5	1.0	0.03	-0.017	0.031	9.0E-06
YIL028W	YIL028W	S288C_Ator08_hmg124h_6.JPG.dat	1077.5	120.0	1.0	0.04	0.056	0.035	4.0E-04
YIL029C	YIL029C	S288C_Ator08_hmg124h_6.JPG.dat	988.0	196.8	0.9	0.18	-0.075	0.178	2.1E-03
YIL030C	YIL030C	S288C_Ator08_hmg124h_6.JPG.dat	1072.0	18.8	1.0	0.01	-0.014	0.011	3.4E-07
YIL032C	YIL032C	S288C_Ator08_hmg124h_6.JPG.dat	1065.0	27.5	1.0	0.03	0.014	0.026	4.3E-06
YIL034C	YIL034C	S288C_Ator08_hmg124h_6.JPG.dat	1023.8	24.8	1.0	0.01	-0.087	0.006	1.2E-05
YIL035C	YIL035C	S288C_Ator08_hmg124h_6.JPG.dat	1057.5	34.1	1.0	0.03	-0.021	0.033	1.0E-05
YIL036W	YIL036W	S288C_Ator08_hmg124h_6.JPG.dat	1077.8	6.4	1.0	0.00	0.028	0.002	1.9E-09
YIL037C	YIL037C	S288C_Ator08_hmg124h_6.JPG.dat	1103.3	44.8	1.0	0.04	0.079	0.038	1.4E-05
YIL038C	YIL038C	S288C_Ator08_hmg124h_6.JPG.dat	1071.0	32.9	1.0	0.01	0.033	0.008	2.1E-05
YIL039W	YIL039W	S288C_Ator08_hmg124h_6.JPG.dat	1130.8	64.9	1.0	0.06	0.008	0.058	5.0E-05
YIL040W	YIL040W	S288C_Ator08_hmg124h_6.JPG.dat	897.3	286.0	0.8	0.26	-0.028	0.257	8.1E-03
YIL041W	YIL041W	S288C_Ator08_hmg124h_6.JPG.dat	1095.0	31.1	1.0	0.03	-0.035	0.029	6.9E-06
YIL042C	YIL042C	S288C_Ator08_hmg124h_6.JPG.dat	1014.8	119.5	1.0	0.03	-0.016	0.031	3.3E-04
YIL043C	YIL043C	S288C_Ator08_hmg124h_6.JPG.dat	1079.3	11.8	1.0	0.01	0.022	0.008	1.2E-07
YIL044C	YIL044C	S288C_Ator08_hmg124h_6.JPG.dat	918.0	28.7	0.9	0.03	0.009	0.028	8.1E-06
YIL045W	YIL045W	S288C_Ator08_hmg124h_6.JPG.dat	1024.5	20.0	1.0	0.01	-0.041	0.007	1.5E-05
YIL047C	YIL047C	S288C_Ator08_hmg124h_6.JPG.dat	841.3	561.6	1.1	0.03	0.031	0.031	2.8E-04
YIL049W	YIL049W	S288C_Ator08_hmg124h_6.JPG.dat	977.3	91.3	1.0	0.09	-0.028	0.087	2.1E-04
YIL050W	YIL050W	S288C_Ator08_hmg124h_6.JPG.dat	981.0	41.3	0.9	0.04	-0.068	0.041	2.4E-05
YIL052C	YIL052C	S288C_Ator08_hmg124h_6.JPG.dat	1010.5	54.2	1.0	0.05	-0.066	0.048	3.5E-05
YIL053W	YIL053W	S288C_Ator08_hmg124h_6.JPG.dat	1115.5	53.0	1.0	0.05	0.069	0.050	3.1E-05
YIL054W	YIL054W	S288C_Ator08_hmg124h_6.JPG.dat	1072.0	38.5	1.0	0.01	0.028	0.011	3.9E-05
YIL055C	YIL055C	S288C_Ator08_hmg124h_6.JPG.dat	1107.8	29.0	1.0	0.01	-0.006	0.011	4.0E-05
YIL056W	YIL056W	S288C_Ator08_hmg124h_6.JPG.dat	1097.0	21.0	1.0	0.02	0.020	0.018	1.5E-06
YIL057C	YIL057C	S288C_Ator08_hmg124h_6.JPG.dat	1052.5	15.2	1.0	0.01	-0.021	0.014	8.2E-07
YIL058W	YIL058W	S288C_Ator08_hmg124h_6.JPG.dat	949.0	50.9	1.0	0.00	-0.027	0.004	6.1E-06
YIL059C	YIL059C	S288C_Ator08_hmg124h_6.JPG.dat	1013.0	26.4	1.0	0.03	-0.039	0.033	1.0E-05
YOR202W	YOR202W	S288C_Ator08_hmg124h_6.JPG.dat	1240.1	169.6	1.0	0.08	0.004	0.076	1.6E-299
1	1	S288C_ctrl_hmg124h_2.JPG.dat	1711.3	218.7	1.0	0.08	#NUM!		1.3E-04
2	2	S288C_ctrl_hmg124h_2.JPG.dat	1485.5	77.5	1.0	0.02	#NUM!		2.2E-06
3	3	S288C_ctrl_hmg124h_2.JPG.dat	1487.5	75.0	1.0	0.03	#NUM!		3.6E-04
4	4	S288C_ctrl_hmg124h_2.JPG.dat	1316.5	100.7	1.0	0.03	#NUM!		9.6E-06
YBR224W	YBR224W	S288C_ctrl_hmg124h_2.JPG.dat	1287.5	56.5	1.0	0.06	#NUM!		4.4E-05
YBR225W	YBR225W	S288C_ctrl_hmg124h_2.JPG.dat	1277.3	42.0	1.0	0.03	#NUM!		1.1E-05
YBR226C	YBR226C	S288C_ctrl_hmg124h_2.JPG.dat	1301.8	39.7	1.0	0.03	#NUM!		5.1E-06
YBR227C	YBR227C	S288C_ctrl_hmg124h_2.JPG.dat	1316.8	31.1	1.0	0.00	#NUM!		7.0E-06
YBR228W	YBR228W	S288C_ctrl_hmg124h_2.JPG.dat	1315.8	29.8	1.0	0.02	#NUM!		2.6E-06
YBR229C	YBR229C	S288C_ctrl_hmg124h_2.JPG.dat	1307.3	37.9	1.0	0.03	#NUM!		4.2E-06
YBR230C	YBR230C	S288C_ctrl_hmg124h_2.JPG.dat	1243.0	35.7	1.0	0.01	#NUM!		1.1E-05
YBR231C	YBR231C	S288C_ctrl_hmg124h_2.JPG.dat	1095.0	100.0	0.9	0.08	#NUM!		2.0E-04
YBR232C	YBR232C	S288C_ctrl_hmg124h_2.JPG.dat	1247.0	29.5	1.0	0.03	#NUM!		7.4E-06
YBR233W	YBR233W	S288C_ctrl_hmg124h_2.JPG.dat	1317.8	28.9	1.0	0.02	#NUM!		3.1E-06
YBR235W	YBR235W	S288C_ctrl_hmg124h_2.JPG.dat	1423.8	72.9	1.1	0.05	#NUM!		3.2E-05
YBR238C	YBR238C	S288C_ctrl_hmg124h_2.JPG.dat	1042.8	82.6	0.9	0.07	#NUM!		1.4E-04
YBR239C	YBR239C	S288C_ctrl_hmg124h_2.JPG.dat	1218.0	12.3	1.0	0.01	#NUM!		3.4E-07
YBR240C	YBR240C	S288C_ctrl_hmg124h_2.JPG.dat	1272.8	29.7	1.0	0.02	#NUM!		3.3E-06
YBR241C	YBR241C	S288C_ctrl_hmg124h_2.JPG.dat	1229.3	48.3	1.0	0.01	#NUM!		3.0E-05
YBR242W	YBR242W	S288C_ctrl_hmg124h_2.JPG.dat	1184.0	25.6	1.0	0.02	#NUM!		3.7E-06
YBR244W	YBR244W	S288C_ctrl_hmg124h_2.JPG.dat	1197.3	36.7	1.0	0.03	#NUM!		4.0E-06
YBR245C	YBR245C	S288C_ctrl_hmg124h_2.JPG.dat	1161.5	23.3	1.0	0.02	#NUM!		2.9E-06
YBR246W	YBR246W	S288C_ctrl_hmg124h_2.JPG.dat	1114.0	25.0	1.0	0.01	#NUM!		2.4E-05
YBR248C	YBR248C	S288C_ctrl_hmg124h_2.JPG.dat	586.8	685.1	0.5	0.56	#NUM!		1.8E-01
YBR249C	YBR249C	S288C_ctrl_hmg124h_2.JPG.dat	1164.8	74.4	0.9	0.01	#NUM!		7.5E-05
YBR250W	YBR250W	S288C_ctrl_hmg124h_2.JPG.dat	1217.5	26.2	1.0	0.02	#NUM!		1.5E-06
YBR255W	YBR255W	S288C_ctrl_hmg124h_2.JPG.dat	1196.5	54.4	1.0	0.04	#NUM!		2.3E-05

YBR258C	YBR258C	S288C_ctrl_hmg124h_2.JPG.dat	1268.8	51.6	1.0	0.05	#NUM!	2.8E-05
YBR259W	YBR259W	S288C_ctrl_hmg124h_2.JPG.dat	1281.3	17.4	1.0	0.02	#NUM!	1.7E-06
YBR260C	YBR260C	S288C_ctrl_hmg124h_2.JPG.dat	1253.0	2.6	1.1	0.00	#NUM!	2.9E-08
YBR261C	YBR261C	S288C_ctrl_hmg124h_2.JPG.dat	1111.3	24.6	1.0	0.03	#NUM!	5.4E-06
YBR262C	YBR262C	S288C_ctrl_hmg124h_2.JPG.dat	1144.5	30.2	1.0	0.02	#NUM!	1.9E-06
YBR263W	YBR263W	S288C_ctrl_hmg124h_2.JPG.dat	1134.0	16.3	1.0	0.02	#NUM!	2.1E-06
YBR264C	YBR264C	S288C_ctrl_hmg124h_2.JPG.dat	1154.8	60.7	1.0	0.05	#NUM!	4.0E-05
YBR266C	YBR266C	S288C_ctrl_hmg124h_2.JPG.dat	1063.3	238.1	0.9	0.20	#NUM!	3.0E-03
YBR267W	YBR267W	S288C_ctrl_hmg124h_2.JPG.dat	937.3	294.0	0.8	0.26	#NUM!	8.4E-03
YBR269C	YBR269C	S288C_ctrl_hmg124h_2.JPG.dat	1012.3	36.8	0.9	0.01	#NUM!	3.2E-05
YBR270C	YBR270C	S288C_ctrl_hmg124h_2.JPG.dat	1177.8	34.4	1.0	0.03	#NUM!	6.4E-06
YBR271W	YBR271W	S288C_ctrl_hmg124h_2.JPG.dat	1308.8	44.2	1.0	0.01	#NUM!	4.1E-05
YBR272C	YBR272C	S288C_ctrl_hmg124h_2.JPG.dat	1238.3	36.8	1.0	0.03	#NUM!	5.1E-06
YBR273C	YBR273C	S288C_ctrl_hmg124h_2.JPG.dat	1256.5	53.2	1.0	0.04	#NUM!	1.7E-05
YBR274W	YBR274W	S288C_ctrl_hmg124h_2.JPG.dat	1254.3	42.7	1.1	0.03	#NUM!	9.2E-06
YBR275C	YBR275C	S288C_ctrl_hmg124h_2.JPG.dat	1133.0	27.1	1.0	0.02	#NUM!	3.1E-06
YBR276C	YBR276C	S288C_ctrl_hmg124h_2.JPG.dat	1239.3	27.5	1.1	0.02	#NUM!	3.3E-06
YBR277C	YBR277C	S288C_ctrl_hmg124h_2.JPG.dat	1093.5	50.7	0.9	0.04	#NUM!	2.9E-05
YBR278W	YBR278W	S288C_ctrl_hmg124h_2.JPG.dat	1147.0	46.2	1.0	0.04	#NUM!	1.5E-05
YBR280C	YBR280C	S288C_ctrl_hmg124h_2.JPG.dat	1214.0	34.9	1.0	0.02	#NUM!	2.2E-06
YBR281C	YBR281C	S288C_ctrl_hmg124h_2.JPG.dat	1011.3	36.3	0.9	0.04	#NUM!	2.0E-05
YBR283C	YBR283C	S288C_ctrl_hmg124h_2.JPG.dat	1200.0	56.6	1.0	0.05	#NUM!	3.9E-05
YBR284W	YBR284W	S288C_ctrl_hmg124h_2.JPG.dat	1308.5	40.0	1.0	0.03	#NUM!	5.9E-06
YBR285W	YBR285W	S288C_ctrl_hmg124h_2.JPG.dat	1386.3	62.5	1.1	0.04	#NUM!	1.6E-05
YBR286W	YBR286W	S288C_ctrl_hmg124h_2.JPG.dat	1170.8	31.7	0.9	0.02	#NUM!	5.0E-06
YBR287W	YBR287W	S288C_ctrl_hmg124h_2.JPG.dat	1157.3	70.2	1.0	0.05	#NUM!	4.5E-05
YBR288C	YBR288C	S288C_ctrl_hmg124h_2.JPG.dat	1144.0	12.9	1.0	0.01	#NUM!	2.5E-07
YBR289W	YBR289W	S288C_ctrl_hmg124h_2.JPG.dat	695.5	535.7	0.6	0.45	#NUM!	8.1E-02
YBR290W	YBR290W	S288C_ctrl_hmg124h_2.JPG.dat	1237.0	23.1	1.1	0.03	#NUM!	4.2E-06
YBR291C	YBR291C	S288C_ctrl_hmg124h_2.JPG.dat	1059.8	127.9	0.9	0.11	#NUM!	4.9E-04
YBR292C	YBR292C	S288C_ctrl_hmg124h_2.JPG.dat	1190.3	19.9	1.0	0.00	#NUM!	5.8E-06
YBR293W	YBR293W	S288C_ctrl_hmg124h_2.JPG.dat	1125.5	86.2	1.0	0.08	#NUM!	1.3E-04
YBR294W	YBR294W	S288C_ctrl_hmg124h_2.JPG.dat	1400.5	69.0	1.1	0.01	#NUM!	1.6E-05
YBR295W	YBR295W	S288C_ctrl_hmg124h_2.JPG.dat	1360.8	270.1	0.9	0.07	#NUM!	1.9E-03
YBR296C	YBR296C	S288C_ctrl_hmg124h_2.JPG.dat	1335.5	77.8	1.0	0.06	#NUM!	4.9E-05
YBR297W	YBR297W	S288C_ctrl_hmg124h_2.JPG.dat	1230.3	22.3	1.0	0.01	#NUM!	8.1E-07
YBR298C	YBR298C	S288C_ctrl_hmg124h_2.JPG.dat	1188.0	29.7	1.0	0.03	#NUM!	5.9E-06
YBR300C	YBR300C	S288C_ctrl_hmg124h_2.JPG.dat	1197.0	31.4	1.0	0.03	#NUM!	8.4E-06
YBR301W	YBR301W	S288C_ctrl_hmg124h_2.JPG.dat	1220.0	31.3	1.0	0.03	#NUM!	7.4E-06
YCL001W	YCL001W	S288C_ctrl_hmg124h_2.JPG.dat	1204.0	42.0	1.0	0.01	#NUM!	3.0E-05
YCL001W-/	YCL001W-A	S288C_ctrl_hmg124h_2.JPG.dat	934.8	628.4	1.1	0.08	#NUM!	1.9E-03
YCL002C	YCL002C	S288C_ctrl_hmg124h_2.JPG.dat	1102.5	110.1	1.0	0.09	#NUM!	2.1E-04
YCL006C	YCL006C	S288C_ctrl_hmg124h_2.JPG.dat	1104.8	52.5	1.0	0.04	#NUM!	2.7E-05
YCL009C	YCL009C	S288C_ctrl_hmg124h_2.JPG.dat	1219.8	16.2	1.0	0.03	#NUM!	5.4E-06
YCL010C	YCL010C	S288C_ctrl_hmg124h_2.JPG.dat	1308.5	207.5	1.0	0.15	#NUM!	9.3E-04
YCL011C	YCL011C	S288C_ctrl_hmg124h_2.JPG.dat	1245.5	79.2	1.0	0.04	#NUM!	2.8E-05
YCL012W	YCL012W	S288C_ctrl_hmg124h_2.JPG.dat	1248.8	93.3	1.0	0.06	#NUM!	5.1E-05
YCL013W	YCL013W	S288C_ctrl_hmg124h_2.JPG.dat	1293.0	47.8	1.0	0.01	#NUM!	1.7E-05
YCL014W	YCL014W	S288C_ctrl_hmg124h_2.JPG.dat	1287.8	15.8	1.0	0.03	#NUM!	9.8E-06
YCL016C	YCL016C	S288C_ctrl_hmg124h_2.JPG.dat	1091.8	53.7	0.9	0.03	#NUM!	6.7E-06
YCL022C	YCL022C	S288C_ctrl_hmg124h_2.JPG.dat	1237.8	70.4	1.0	0.06	#NUM!	4.6E-05
YCL023C	YCL023C	S288C_ctrl_hmg124h_2.JPG.dat	1250.3	93.5	1.0	0.07	#NUM!	8.0E-05
YCL024W	YCL024W	S288C_ctrl_hmg124h_2.JPG.dat	1238.0	66.7	1.0	0.07	#NUM!	8.6E-05
YCL025C	YCL025C	S288C_ctrl_hmg124h_2.JPG.dat	1189.0	96.8	1.0	0.10	#NUM!	2.6E-04
YCL026C	YCL026C	S288C_ctrl_hmg124h_2.JPG.dat	1361.5	28.0	1.1	0.01	#NUM!	3.2E-07
YCL026C-A	YCL026C-A	S288C_ctrl_hmg124h_2.JPG.dat	1317.8	19.8	1.0	0.01	#NUM!	8.2E-07
YCL027W	YCL027W	S288C_ctrl_hmg124h_2.JPG.dat	1268.8	52.0	1.0	0.00	#NUM!	7.6E-06
YCL028W	YCL028W	S288C_ctrl_hmg124h_2.JPG.dat	1310.3	49.5	1.0	0.03	#NUM!	1.0E-05
YCL029C	YCL029C	S288C_ctrl_hmg124h_2.JPG.dat	1207.3	11.1	1.0	0.02	#NUM!	1.3E-06
YCL030C	YCL030C	S288C_ctrl_hmg124h_2.JPG.dat	196.3	392.5	0.0	0.00	#NUM!	
YCL032W	YCL032W	S288C_ctrl_hmg124h_2.JPG.dat	1266.3	37.6	1.0	0.03	#NUM!	5.2E-06
YCL033C	YCL033C	S288C_ctrl_hmg124h_2.JPG.dat	1264.3	33.7	1.0	0.03	#NUM!	5.4E-06
YCL034W	YCL034W	S288C_ctrl_hmg124h_2.JPG.dat	1288.5	31.8	1.0	0.02	#NUM!	1.3E-06
YCL035C	YCL035C	S288C_ctrl_hmg124h_2.JPG.dat	1227.8	31.6	0.9	0.03	#NUM!	5.8E-06
YCL036W	YCL036W	S288C_ctrl_hmg124h_2.JPG.dat	1318.8	104.8	1.0	0.07	#NUM!	1.0E-04
YCL037C	YCL037C	S288C_ctrl_hmg124h_2.JPG.dat	889.3	54.2	0.7	0.01	#NUM!	7.1E-05
YCL038C	YCL038C	S288C_ctrl_hmg124h_2.JPG.dat	1196.0	26.8	1.0	0.00	#NUM!	1.8E-06
YCL039W	YCL039W	S288C_ctrl_hmg124h_2.JPG.dat	1246.5	41.4	1.0	0.01	#NUM!	1.7E-05
YCL040W	YCL040W	S288C_ctrl_hmg124h_2.JPG.dat	1219.8	25.8	1.0	0.02	#NUM!	2.2E-06
YCL042W	YCL042W	S288C_ctrl_hmg124h_2.JPG.dat	1191.3	19.3	1.0	0.01	#NUM!	3.9E-07

YCL044C	YCL044C	S288C_ctrl_hmg124h_2.JPG.dat	1164.8	17.0	1.0	0.00	#NUM!	3.0E-06
YCL045C	YCL045C	S288C_ctrl_hmg124h_2.JPG.dat	1144.3	25.1	1.0	0.02	#NUM!	1.7E-06
YCL046W	YCL046W	S288C_ctrl_hmg124h_2.JPG.dat	1175.5	52.4	1.0	0.05	#NUM!	3.1E-05
YCL047C	YCL047C	S288C_ctrl_hmg124h_2.JPG.dat	1270.0	54.4	1.1	0.03	#NUM!	8.4E-06
YCL048W	YCL048W	S288C_ctrl_hmg124h_2.JPG.dat	1237.8	52.1	1.0	0.04	#NUM!	1.7E-05
YCL049C	YCL049C	S288C_ctrl_hmg124h_2.JPG.dat	1193.3	68.8	1.0	0.06	#NUM!	5.8E-05
YCL050C	YCL050C	S288C_ctrl_hmg124h_2.JPG.dat	1188.3	17.0	1.0	0.01	#NUM!	6.3E-07
YCL051W	YCL051W	S288C_ctrl_hmg124h_2.JPG.dat	1260.8	35.8	1.0	0.03	#NUM!	5.6E-06
YCL055W	YCL055W	S288C_ctrl_hmg124h_2.JPG.dat	1222.0	64.5	1.0	0.05	#NUM!	2.8E-05
YCL056C	YCL056C	S288C_ctrl_hmg124h_2.JPG.dat	1246.3	38.8	1.0	0.03	#NUM!	4.3E-06
YCL057W	YCL057W	S288C_ctrl_hmg124h_2.JPG.dat	1172.8	31.4	1.0	0.00	#NUM!	1.8E-06
YCL060C	YCL060C	S288C_ctrl_hmg124h_2.JPG.dat	1095.8	25.4	0.9	0.02	#NUM!	1.7E-06
YCL061C	YCL061C	S288C_ctrl_hmg124h_2.JPG.dat	1034.3	48.1	0.9	0.05	#NUM!	3.9E-05
YCL062W	YCL062W	S288C_ctrl_hmg124h_2.JPG.dat	980.3	39.3	0.8	0.01	#NUM!	7.5E-05
YCL063W	YCL063W	S288C_ctrl_hmg124h_2.JPG.dat	954.3	35.3	0.8	0.01	#NUM!	1.2E-04
YCL064C	YCL064C	S288C_ctrl_hmg124h_2.JPG.dat	1259.3	49.4	1.0	0.04	#NUM!	1.8E-05
YCL069W	YCL069W	S288C_ctrl_hmg124h_2.JPG.dat	1198.3	32.1	1.0	0.01	#NUM!	1.2E-05
YCL074W	YCL074W	S288C_ctrl_hmg124h_2.JPG.dat	1233.0	19.4	1.0	0.03	#NUM!	4.9E-06
YCL075W	YCL075W	S288C_ctrl_hmg124h_2.JPG.dat	1280.5	83.9	1.0	0.06	#NUM!	4.6E-05
YCL076W	YCL076W	S288C_ctrl_hmg124h_2.JPG.dat	1237.5	53.6	1.0	0.04	#NUM!	2.0E-05
YCR001W	YCR001W	S288C_ctrl_hmg124h_2.JPG.dat	1279.0	27.4	1.0	0.01	#NUM!	1.1E-05
YCR005C	YCR005C	S288C_ctrl_hmg124h_2.JPG.dat	1215.0	22.5	1.0	0.02	#NUM!	1.2E-06
YCR006C	YCR006C	S288C_ctrl_hmg124h_2.JPG.dat	1231.0	25.6	1.0	0.02	#NUM!	2.9E-06
YCR007C	YCR007C	S288C_ctrl_hmg124h_2.JPG.dat	1131.5	84.6	1.0	0.07	#NUM!	1.1E-04
YCR008W	YCR008W	S288C_ctrl_hmg124h_2.JPG.dat	1103.0	53.3	1.0	0.05	#NUM!	3.3E-05
YCR009C	YCR009C	S288C_ctrl_hmg124h_2.JPG.dat	1189.0	2.4	1.0	0.01	#NUM!	1.3E-07
YCR010C	YCR010C	S288C_ctrl_hmg124h_2.JPG.dat	1189.3	29.4	1.0	0.02	#NUM!	3.7E-06
YCR011C	YCR011C	S288C_ctrl_hmg124h_2.JPG.dat	1213.5	32.4	1.1	0.03	#NUM!	7.4E-06
YCR014C	YCR014C	S288C_ctrl_hmg124h_2.JPG.dat	1319.5	63.0	1.1	0.02	#NUM!	8.8E-05
YCR015C	YCR015C	S288C_ctrl_hmg124h_2.JPG.dat	1339.3	39.8	1.0	0.03	#NUM!	9.7E-06
YCR016W	YCR016W	S288C_ctrl_hmg124h_2.JPG.dat	1273.8	48.9	1.0	0.04	#NUM!	1.3E-05
YCR017C	YCR017C	S288C_ctrl_hmg124h_2.JPG.dat	1229.8	33.0	1.0	0.02	#NUM!	1.5E-06
YCR019W	YCR019W	S288C_ctrl_hmg124h_2.JPG.dat	1229.8	44.6	1.0	0.03	#NUM!	7.3E-06
YCR020C	YCR020C	S288C_ctrl_hmg124h_2.JPG.dat	1240.8	9.4	1.0	0.01	#NUM!	5.0E-07
YCR020C-A	YCR020C-A	S288C_ctrl_hmg124h_2.JPG.dat	1138.8	12.5	1.0	0.01	#NUM!	5.4E-07
YCR021C	YCR021C	S288C_ctrl_hmg124h_2.JPG.dat	1224.0	27.1	1.0	0.02	#NUM!	1.2E-06
YCR022C	YCR022C	S288C_ctrl_hmg124h_2.JPG.dat	1214.5	49.2	1.0	0.03	#NUM!	1.1E-05
YCR023C	YCR023C	S288C_ctrl_hmg124h_2.JPG.dat	1260.5	40.9	1.1	0.04	#NUM!	1.3E-05
YCR024C-A	YCR024C-A	S288C_ctrl_hmg124h_2.JPG.dat	1082.5	103.3	0.9	0.09	#NUM!	2.1E-04
YCR025C	YCR025C	S288C_ctrl_hmg124h_2.JPG.dat	1335.8	36.3	1.1	0.02	#NUM!	2.3E-06
YCR026C	YCR026C	S288C_ctrl_hmg124h_2.JPG.dat	1261.0	186.2	1.0	0.03	#NUM!	3.3E-04
YCR027C	YCR027C	S288C_ctrl_hmg124h_2.JPG.dat	1241.5	89.0	1.0	0.07	#NUM!	1.0E-04
YCR028C-A	YCR028C-A	S288C_ctrl_hmg124h_2.JPG.dat	0.0	0.0	0.0	0.00	#NUM!	
YCR030C	YCR030C	S288C_ctrl_hmg124h_2.JPG.dat	1248.0	46.3	1.0	0.04	#NUM!	1.2E-05
YCR031C	YCR031C	S288C_ctrl_hmg124h_2.JPG.dat	859.8	89.0	0.7	0.07	#NUM!	3.0E-04
YCR032W	YCR032W	S288C_ctrl_hmg124h_2.JPG.dat	1114.8	55.2	0.9	0.05	#NUM!	4.0E-05
YCR033W	YCR033W	S288C_ctrl_hmg124h_2.JPG.dat	912.8	62.6	0.8	0.05	#NUM!	8.2E-05
YCR034W	YCR034W	S288C_ctrl_hmg124h_2.JPG.dat	835.0	28.8	0.7	0.01	#NUM!	6.7E-05
YCR036W	YCR036W	S288C_ctrl_hmg124h_2.JPG.dat	1049.8	215.7	1.0	0.03	#NUM!	2.3E-04
YCR037C	YCR037C	S288C_ctrl_hmg124h_2.JPG.dat	1029.8	67.0	0.9	0.06	#NUM!	6.7E-05
YCR043C	YCR043C	S288C_ctrl_hmg124h_2.JPG.dat	1295.5	52.9	1.0	0.05	#NUM!	4.0E-05
YCR044C	YCR044C	S288C_ctrl_hmg124h_2.JPG.dat	1302.3	254.8	0.9	0.07	#NUM!	1.8E-03
YCR045C	YCR045C	S288C_ctrl_hmg124h_2.JPG.dat	1331.8	95.6	1.0	0.06	#NUM!	4.7E-05
YCR048W	YCR048W	S288C_ctrl_hmg124h_2.JPG.dat	1288.0	32.5	1.0	0.03	#NUM!	4.6E-06
YCR049C	YCR049C	S288C_ctrl_hmg124h_2.JPG.dat	1329.5	69.9	1.1	0.04	#NUM!	1.2E-05
YCR050C	YCR050C	S288C_ctrl_hmg124h_2.JPG.dat	1156.8	93.7	0.9	0.06	#NUM!	6.0E-05
YCR051W	YCR051W	S288C_ctrl_hmg124h_2.JPG.dat	1277.5	16.6	1.0	0.04	#NUM!	1.1E-05
YCR053W	YCR053W	S288C_ctrl_hmg124h_2.JPG.dat	857.5	260.6	0.6	0.01	#NUM!	1.9E-04
YCR059C	YCR059C	S288C_ctrl_hmg124h_2.JPG.dat	1218.5	41.5	1.0	0.01	#NUM!	5.4E-07
YCR060W	YCR060W	S288C_ctrl_hmg124h_2.JPG.dat	1199.8	72.3	1.0	0.06	#NUM!	7.0E-05
YCR061W	YCR061W	S288C_ctrl_hmg124h_2.JPG.dat	1196.8	155.5	1.0	0.14	#NUM!	6.3E-04
YCR062W	YCR062W	S288C_ctrl_hmg124h_2.JPG.dat	1382.8	7.0	1.0	0.03	#NUM!	6.1E-06
YCR063W	YCR063W	S288C_ctrl_hmg124h_2.JPG.dat	1362.0	44.7	1.0	0.00	#NUM!	1.6E-07
YCR065W	YCR065W	S288C_ctrl_hmg124h_2.JPG.dat	1341.3	66.2	1.0	0.03	#NUM!	1.2E-05
YCR067C	YCR067C	S288C_ctrl_hmg124h_2.JPG.dat	1394.0	71.6	1.0	0.02	#NUM!	3.1E-06
YCR068W	YCR068W	S288C_ctrl_hmg124h_2.JPG.dat	1351.8	51.3	1.0	0.01	#NUM!	3.9E-07
YCR069W	YCR069W	S288C_ctrl_hmg124h_2.JPG.dat	1323.3	41.1	1.0	0.04	#NUM!	1.5E-05
YCR071C	YCR071C	S288C_ctrl_hmg124h_2.JPG.dat	1321.0	46.2	1.0	0.01	#NUM!	2.2E-07
YCR073C	YCR073C	S288C_ctrl_hmg124h_2.JPG.dat	1330.3	63.1	1.0	0.02	#NUM!	2.1E-06
YCR073W-	YCR073W-A	S288C_ctrl_hmg124h_2.JPG.dat	1334.5	57.8	1.0	0.01	#NUM!	9.0E-07

YCR075C	YCR075C	S288C_ctrl_hmg124h_2.JPG.dat	1403.8	62.7	1.0	0.05	#NUM!	3.0E-05
YCR076C	YCR076C	S288C_ctrl_hmg124h_2.JPG.dat	1321.0	81.9	1.0	0.08	#NUM!	1.7E-04
YCR077C	YCR077C	S288C_ctrl_hmg124h_2.JPG.dat	1102.8	49.6	0.9	0.04	#NUM!	2.3E-05
YCR079W	YCR079W	S288C_ctrl_hmg124h_2.JPG.dat	1255.5	21.4	1.0	0.02	#NUM!	1.7E-06
YCR081W	YCR081W	S288C_ctrl_hmg124h_2.JPG.dat	721.8	139.9	0.5	0.02	#NUM!	6.5E-04
YCR082W	YCR082W	S288C_ctrl_hmg124h_2.JPG.dat	1220.5	26.7	1.0	0.01	#NUM!	8.1E-07
YCR083W	YCR083W	S288C_ctrl_hmg124h_2.JPG.dat	1249.3	53.8	1.0	0.04	#NUM!	1.2E-05
YCR085W	YCR085W	S288C_ctrl_hmg124h_2.JPG.dat	1210.8	26.0	1.0	0.00	#NUM!	6.6E-06
YCR086W	YCR086W	S288C_ctrl_hmg124h_2.JPG.dat	1116.5	29.7	0.9	0.02	#NUM!	2.2E-06
YCR087C-A	YCR087C-A	S288C_ctrl_hmg124h_2.JPG.dat	1184.5	50.7	1.0	0.05	#NUM!	3.1E-05
YCR087W	YCR087W	S288C_ctrl_hmg124h_2.JPG.dat	1233.8	37.3	1.0	0.03	#NUM!	1.1E-05
YCR088W	YCR088W	S288C_ctrl_hmg124h_2.JPG.dat	1247.3	54.8	1.0	0.04	#NUM!	2.4E-05
YCR089W	YCR089W	S288C_ctrl_hmg124h_2.JPG.dat	1246.3	43.2	1.0	0.03	#NUM!	6.8E-06
YCR090C	YCR090C	S288C_ctrl_hmg124h_2.JPG.dat	1214.5	57.0	1.0	0.00	#NUM!	3.4E-06
YCR091W	YCR091W	S288C_ctrl_hmg124h_2.JPG.dat	1276.5	40.7	1.0	0.03	#NUM!	9.0E-06
YCR092C	YCR092C	S288C_ctrl_hmg124h_2.JPG.dat	1267.3	61.9	1.0	0.05	#NUM!	2.7E-05
YCR095C	YCR095C	S288C_ctrl_hmg124h_2.JPG.dat	1158.8	36.7	1.0	0.04	#NUM!	1.4E-05
YCR098C	YCR098C	S288C_ctrl_hmg124h_2.JPG.dat	1199.0	14.8	1.0	0.01	#NUM!	3.2E-07
YCR099C	YCR099C	S288C_ctrl_hmg124h_2.JPG.dat	1141.3	41.6	1.0	0.00	#NUM!	7.9E-06
YCR100C	YCR100C	S288C_ctrl_hmg124h_2.JPG.dat	1200.3	15.2	1.0	0.02	#NUM!	1.6E-06
YCR101C	YCR101C	S288C_ctrl_hmg124h_2.JPG.dat	1232.8	28.8	1.1	0.00	#NUM!	5.7E-06
YCR102C	YCR102C	S288C_ctrl_hmg124h_2.JPG.dat	1279.0	49.2	1.0	0.04	#NUM!	1.3E-05
YCR102W-	YCR102W-A	S288C_ctrl_hmg124h_2.JPG.dat	1227.0	44.4	1.0	0.04	#NUM!	1.4E-05
YCR105W	YCR105W	S288C_ctrl_hmg124h_2.JPG.dat	1209.0	35.3	1.0	0.03	#NUM!	7.7E-06
YCR106W	YCR106W	S288C_ctrl_hmg124h_2.JPG.dat	1221.8	36.7	1.0	0.03	#NUM!	5.0E-06
YDL001W	YDL001W	S288C_ctrl_hmg124h_2.JPG.dat	1225.8	48.9	1.0	0.04	#NUM!	1.7E-05
YDL002C	YDL002C	S288C_ctrl_hmg124h_2.JPG.dat	1110.3	42.9	0.9	0.03	#NUM!	1.3E-05
YDL006W	YDL006W	S288C_ctrl_hmg124h_2.JPG.dat	826.8	519.3	0.7	0.43	#NUM!	5.0E-02
YDL010W	YDL010W	S288C_ctrl_hmg124h_2.JPG.dat	1134.8	17.7	1.0	0.01	#NUM!	1.0E-06
YDL011C	YDL011C	S288C_ctrl_hmg124h_2.JPG.dat	1208.5	46.8	1.0	0.03	#NUM!	9.8E-06
YDL012C	YDL012C	S288C_ctrl_hmg124h_2.JPG.dat	1148.8	35.2	1.0	0.01	#NUM!	1.7E-05
YDL013W	YDL013W	S288C_ctrl_hmg124h_2.JPG.dat	736.0	446.9	0.6	0.39	#NUM!	4.6E-02
YDL018C	YDL018C	S288C_ctrl_hmg124h_2.JPG.dat	1175.5	34.1	1.0	0.03	#NUM!	8.2E-06
YDL019C	YDL019C	S288C_ctrl_hmg124h_2.JPG.dat	1242.5	8.7	1.0	0.01	#NUM!	3.1E-08
YDL020C	YDL020C	S288C_ctrl_hmg124h_2.JPG.dat	804.3	83.8	0.7	0.07	#NUM!	3.0E-04
YDL023C	YDL023C	S288C_ctrl_hmg124h_2.JPG.dat	1162.3	67.3	0.9	0.05	#NUM!	4.0E-05
YDL024C	YDL024C	S288C_ctrl_hmg124h_2.JPG.dat	1329.0	34.1	1.0	0.03	#NUM!	4.7E-06
YDL025C	YDL025C	S288C_ctrl_hmg124h_2.JPG.dat	1338.3	70.9	1.1	0.05	#NUM!	3.2E-05
YDL026W	YDL026W	S288C_ctrl_hmg124h_2.JPG.dat	1168.3	42.3	1.0	0.01	#NUM!	3.1E-05
YDL027C	YDL027C	S288C_ctrl_hmg124h_2.JPG.dat	1220.5	11.8	1.0	0.01	#NUM!	1.1E-07
YDL033C	YDL033C	S288C_ctrl_hmg124h_2.JPG.dat	1195.5	22.7	1.0	0.02	#NUM!	3.1E-06
YDL034W	YDL034W	S288C_ctrl_hmg124h_2.JPG.dat	1221.0	27.4	1.0	0.02	#NUM!	2.0E-06
YDL035C	YDL035C	S288C_ctrl_hmg124h_2.JPG.dat	1135.3	20.7	1.0	0.02	#NUM!	1.4E-06
YDL036C	YDL036C	S288C_ctrl_hmg124h_2.JPG.dat	1246.0	10.3	1.0	0.01	#NUM!	1.5E-07
YDL037C	YDL037C	S288C_ctrl_hmg124h_2.JPG.dat	1211.0	24.2	1.0	0.01	#NUM!	1.2E-05
YDL038C	YDL038C	S288C_ctrl_hmg124h_2.JPG.dat	1154.3	56.0	1.0	0.05	#NUM!	3.7E-05
YDL039C	YDL039C	S288C_ctrl_hmg124h_2.JPG.dat	1461.5	39.7	1.1	0.01	#NUM!	4.5E-05
YDL040C	YDL040C	S288C_ctrl_hmg124h_2.JPG.dat	1356.3	109.3	1.0	0.08	#NUM!	1.4E-04
YDL041W	YDL041W	S288C_ctrl_hmg124h_2.JPG.dat	0.0	0.0	0.0	0.00	#NUM!	
YDL042C	YDL042C	S288C_ctrl_hmg124h_2.JPG.dat	301.8	603.5	0.0	0.00	#NUM!	
YDL046W	YDL046W	S288C_ctrl_hmg124h_2.JPG.dat	1299.5	22.3	1.0	0.01	#NUM!	7.0E-07
YDL048C	YDL048C	S288C_ctrl_hmg124h_2.JPG.dat	1228.8	17.8	1.0	0.02	#NUM!	1.6E-06
YDL050C	YDL050C	S288C_ctrl_hmg124h_2.JPG.dat	1245.5	25.2	1.0	0.02	#NUM!	1.9E-06
YDL051W	YDL051W	S288C_ctrl_hmg124h_2.JPG.dat	1269.5	18.9	1.0	0.02	#NUM!	2.3E-06
YDL052C	YDL052C	S288C_ctrl_hmg124h_2.JPG.dat	1144.5	18.3	1.0	0.00	#NUM!	6.3E-06
YDL053C	YDL053C	S288C_ctrl_hmg124h_2.JPG.dat	1150.5	53.1	1.0	0.05	#NUM!	4.2E-05
YDL054C	YDL054C	S288C_ctrl_hmg124h_2.JPG.dat	1218.8	107.7	1.0	0.03	#NUM!	4.0E-04
YDL056W	YDL056W	S288C_ctrl_hmg124h_2.JPG.dat	1220.8	88.8	1.0	0.07	#NUM!	8.2E-05
YDL059C	YDL059C	S288C_ctrl_hmg124h_2.JPG.dat	1406.0	91.0	1.1	0.07	#NUM!	6.0E-05
YDL061C	YDL061C	S288C_ctrl_hmg124h_2.JPG.dat	1282.3	49.7	1.0	0.03	#NUM!	7.0E-06
YDL062W	YDL062W	S288C_ctrl_hmg124h_2.JPG.dat	1244.8	28.4	1.0	0.01	#NUM!	6.5E-07
YDL065C	YDL065C	S288C_ctrl_hmg124h_2.JPG.dat	1097.0	33.3	0.9	0.03	#NUM!	1.3E-05
YDL066W	YDL066W	S288C_ctrl_hmg124h_2.JPG.dat	1272.8	31.5	1.1	0.03	#NUM!	3.9E-06
YDL070W	YDL070W	S288C_ctrl_hmg124h_2.JPG.dat	1202.3	49.0	1.0	0.04	#NUM!	2.0E-05
YDL071C	YDL071C	S288C_ctrl_hmg124h_2.JPG.dat	1170.3	47.3	1.0	0.04	#NUM!	1.5E-05
YDL074C	YDL074C	S288C_ctrl_hmg124h_2.JPG.dat	908.0	37.5	0.8	0.03	#NUM!	2.4E-05
YDL076C	YDL076C	S288C_ctrl_hmg124h_2.JPG.dat	1197.8	96.4	1.1	0.02	#NUM!	1.6E-04
YDL077C	YDL077C	S288C_ctrl_hmg124h_2.JPG.dat	891.5	325.9	0.8	0.29	#NUM!	1.3E-02
YDL078C	YDL078C	S288C_ctrl_hmg124h_2.JPG.dat	1376.8	56.4	1.0	0.04	#NUM!	1.2E-05
YDL079C	YDL079C	S288C_ctrl_hmg124h_2.JPG.dat	1399.0	24.0	1.0	0.04	#NUM!	1.5E-05

YDL080C	YDL080C	S288C_ctrl_hmg124h_2.JPG.dat	1271.8	17.3	0.9	0.02	#NUM!	2.9E-06
YDL081C	YDL081C	S288C_ctrl_hmg124h_2.JPG.dat	989.8	40.9	0.7	0.02	#NUM!	1.0E-05
YDL082W	YDL082W	S288C_ctrl_hmg124h_2.JPG.dat	1078.0	53.2	0.8	0.03	#NUM!	1.8E-05
YDL083C	YDL083C	S288C_ctrl_hmg124h_2.JPG.dat	1027.3	138.8	0.7	0.02	#NUM!	2.6E-04
YDL085W	YDL085W	S288C_ctrl_hmg124h_2.JPG.dat	1351.0	69.3	1.0	0.04	#NUM!	1.0E-05
YDL086W	YDL086W	S288C_ctrl_hmg124h_2.JPG.dat	1330.5	89.5	1.0	0.04	#NUM!	1.6E-05
YDL088C	YDL088C	S288C_ctrl_hmg124h_2.JPG.dat	1252.8	112.2	1.0	0.02	#NUM!	1.3E-04
YDL089W	YDL089W	S288C_ctrl_hmg124h_2.JPG.dat	1329.8	46.1	1.0	0.02	#NUM!	2.1E-06
YDL090C	YDL090C	S288C_ctrl_hmg124h_2.JPG.dat	339.0	678.0	0.0	0.00	#NUM!	
YDL091C	YDL091C	S288C_ctrl_hmg124h_2.JPG.dat	1319.5	45.7	1.1	0.04	#NUM!	1.7E-05
YDL093W	YDL093W	S288C_ctrl_hmg124h_2.JPG.dat	1276.0	25.5	1.0	0.02	#NUM!	1.6E-06
YDL094C	YDL094C	S288C_ctrl_hmg124h_2.JPG.dat	1272.0	41.7	1.0	0.03	#NUM!	8.3E-06
YDL095W	YDL095W	S288C_ctrl_hmg124h_2.JPG.dat	1250.0	31.6	1.0	0.02	#NUM!	2.5E-06
YDL096C	YDL096C	S288C_ctrl_hmg124h_2.JPG.dat	1260.8	39.3	1.0	0.04	#NUM!	1.4E-05
YDL099W	YDL099W	S288C_ctrl_hmg124h_2.JPG.dat	1191.8	39.9	1.0	0.03	#NUM!	8.4E-06
YDL100C	YDL100C	S288C_ctrl_hmg124h_2.JPG.dat	1166.5	35.6	1.0	0.03	#NUM!	1.2E-05
YDL104C	YDL104C	S288C_ctrl_hmg124h_2.JPG.dat	1185.8	23.9	1.0	0.02	#NUM!	1.2E-06
YDL106C	YDL106C	S288C_ctrl_hmg124h_2.JPG.dat	1276.3	65.5	1.0	0.07	#NUM!	7.4E-05
YDL109C	YDL109C	S288C_ctrl_hmg124h_2.JPG.dat	1286.5	25.7	1.0	0.03	#NUM!	4.2E-06
YDL110C	YDL110C	S288C_ctrl_hmg124h_2.JPG.dat	1245.3	58.1	1.0	0.05	#NUM!	2.5E-05
YDL112W	YDL112W	S288C_ctrl_hmg124h_2.JPG.dat	1235.0	53.7	1.0	0.03	#NUM!	7.9E-06
YDL113C	YDL113C	S288C_ctrl_hmg124h_2.JPG.dat	1354.0	105.2	1.0	0.02	#NUM!	7.8E-05
YDL114W	YDL114W	S288C_ctrl_hmg124h_2.JPG.dat	1267.3	45.2	1.0	0.03	#NUM!	9.4E-06
YDL117W	YDL117W	S288C_ctrl_hmg124h_2.JPG.dat	1171.8	95.3	0.9	0.02	#NUM!	1.3E-04
YDL118W	YDL118W	S288C_ctrl_hmg124h_2.JPG.dat	914.0	135.4	0.7	0.02	#NUM!	2.2E-04
YDL119C	YDL119C	S288C_ctrl_hmg124h_2.JPG.dat	1120.5	64.3	1.0	0.06	#NUM!	5.9E-05
YDL121C	YDL121C	S288C_ctrl_hmg124h_2.JPG.dat	1162.8	56.6	1.0	0.04	#NUM!	2.1E-05
YDL122W	YDL122W	S288C_ctrl_hmg124h_2.JPG.dat	1106.0	58.8	1.0	0.01	#NUM!	7.6E-05
YDL123W	YDL123W	S288C_ctrl_hmg124h_2.JPG.dat	1212.3	49.3	1.0	0.03	#NUM!	7.4E-06
YDL124W	YDL124W	S288C_ctrl_hmg124h_2.JPG.dat	1236.5	25.7	1.0	0.03	#NUM!	5.3E-06
YDL125C	YDL125C	S288C_ctrl_hmg124h_2.JPG.dat	1168.0	47.4	1.0	0.03	#NUM!	9.8E-06
YDL127W	YDL127W	S288C_ctrl_hmg124h_2.JPG.dat	1159.5	15.0	1.0	0.01	#NUM!	5.5E-07
YDL128W	YDL128W	S288C_ctrl_hmg124h_2.JPG.dat	1262.8	16.6	1.0	0.00	#NUM!	4.3E-06
YDL129W	YDL129W	S288C_ctrl_hmg124h_2.JPG.dat	1307.0	61.0	1.0	0.05	#NUM!	3.1E-05
YDL130W	YDL130W	S288C_ctrl_hmg124h_2.JPG.dat	961.5	43.6	0.8	0.01	#NUM!	6.8E-05
YDL130W-,	YDL130W-A	S288C_ctrl_hmg124h_2.JPG.dat	1196.5	30.2	1.0	0.02	#NUM!	3.2E-06
YDL131W	YDL131W	S288C_ctrl_hmg124h_2.JPG.dat	1202.8	13.4	1.0	0.01	#NUM!	3.7E-07
YDL133C-A	YDL133C-A	S288C_ctrl_hmg124h_2.JPG.dat	1115.5	49.0	1.0	0.04	#NUM!	2.2E-05
YDL133W	YDL133W	S288C_ctrl_hmg124h_2.JPG.dat	1194.5	34.8	1.0	0.03	#NUM!	7.0E-06
YDL134C	YDL134C	S288C_ctrl_hmg124h_2.JPG.dat	1173.8	51.8	1.0	0.03	#NUM!	1.1E-05
YDL134C-A	YDL134C-A	S288C_ctrl_hmg124h_2.JPG.dat	1224.5	25.1	1.0	0.01	#NUM!	9.8E-06
YDL135C	YDL135C	S288C_ctrl_hmg124h_2.JPG.dat	1173.5	49.1	1.0	0.04	#NUM!	2.1E-05
YDL136W	YDL136W	S288C_ctrl_hmg124h_2.JPG.dat	991.3	53.8	0.8	0.02	#NUM!	1.7E-04
YDL137W	YDL137W	S288C_ctrl_hmg124h_2.JPG.dat	1337.5	26.7	1.0	0.01	#NUM!	1.1E-05
YDL138W	YDL138W	S288C_ctrl_hmg124h_2.JPG.dat	1284.0	38.5	1.0	0.03	#NUM!	8.2E-06
YDL142C	YDL142C	S288C_ctrl_hmg124h_2.JPG.dat	1118.8	28.5	0.9	0.01	#NUM!	2.4E-05
YDL144C	YDL144C	S288C_ctrl_hmg124h_2.JPG.dat	1159.8	58.6	1.0	0.04	#NUM!	2.8E-05
YDL146W	YDL146W	S288C_ctrl_hmg124h_2.JPG.dat	1208.3	56.9	1.0	0.01	#NUM!	4.3E-05
YDL149W	YDL149W	S288C_ctrl_hmg124h_2.JPG.dat	1169.5	11.8	1.0	0.00	#NUM!	5.2E-06
YDL154W	YDL154W	S288C_ctrl_hmg124h_2.JPG.dat	1174.3	23.6	1.0	0.02	#NUM!	1.5E-06
YDL155W	YDL155W	S288C_ctrl_hmg124h_2.JPG.dat	1221.3	55.3	1.0	0.05	#NUM!	4.0E-05
YDL156W	YDL156W	S288C_ctrl_hmg124h_2.JPG.dat	1240.5	34.4	1.0	0.03	#NUM!	6.8E-06
YDL157C	YDL157C	S288C_ctrl_hmg124h_2.JPG.dat	1134.0	11.2	1.0	0.01	#NUM!	2.3E-07
YDL159W	YDL159W	S288C_ctrl_hmg124h_2.JPG.dat	0.0	0.0	0.0	0.00	#NUM!	
YDL160C	YDL160C	S288C_ctrl_hmg124h_2.JPG.dat	0.0	0.0	0.0	0.00	#NUM!	
YDL161W	YDL161W	S288C_ctrl_hmg124h_2.JPG.dat	1367.0	23.7	1.0	0.00	#NUM!	3.5E-07
YDL162C	YDL162C	S288C_ctrl_hmg124h_2.JPG.dat	1299.5	34.6	1.0	0.02	#NUM!	3.0E-06
YDL167C	YDL167C	S288C_ctrl_hmg124h_2.JPG.dat	1181.8	19.3	1.0	0.00	#NUM!	7.4E-06
YDL168W	YDL168W	S288C_ctrl_hmg124h_2.JPG.dat	1188.0	56.9	1.0	0.05	#NUM!	4.0E-05
YDL169C	YDL169C	S288C_ctrl_hmg124h_2.JPG.dat	1176.0	17.6	1.0	0.01	#NUM!	8.5E-07
YDL170W	YDL170W	S288C_ctrl_hmg124h_2.JPG.dat	1225.5	19.0	1.0	0.00	#NUM!	3.1E-06
YDL171C	YDL171C	S288C_ctrl_hmg124h_2.JPG.dat	1210.0	28.9	1.0	0.02	#NUM!	1.8E-06
YDL172C	YDL172C	S288C_ctrl_hmg124h_2.JPG.dat	1249.8	32.3	1.0	0.02	#NUM!	3.3E-06
YDL173W	YDL173W	S288C_ctrl_hmg124h_2.JPG.dat	1070.3	60.3	0.9	0.01	#NUM!	5.9E-05
YDL174C	YDL174C	S288C_ctrl_hmg124h_2.JPG.dat	1263.8	28.5	1.0	0.01	#NUM!	1.2E-05
YDL175C	YDL175C	S288C_ctrl_hmg124h_2.JPG.dat	1328.0	48.6	1.0	0.01	#NUM!	4.3E-05
YDL176W	YDL176W	S288C_ctrl_hmg124h_2.JPG.dat	1273.5	30.0	1.0	0.02	#NUM!	3.4E-06
YDL177C	YDL177C	S288C_ctrl_hmg124h_2.JPG.dat	1311.8	72.8	1.0	0.05	#NUM!	2.7E-05
YDL178W	YDL178W	S288C_ctrl_hmg124h_2.JPG.dat	1205.0	34.6	1.0	0.04	#NUM!	1.3E-05
YDL179W	YDL179W	S288C_ctrl_hmg124h_2.JPG.dat	1176.3	35.9	1.0	0.01	#NUM!	1.8E-05

YDL180W	YDL180W	S288C_ctrl_hmg124h_2.JPG.dat	1208.0	33.1	1.0	0.03	#NUM!	9.4E-06
YDL181W	YDL181W	S288C_ctrl_hmg124h_2.JPG.dat	1235.8	54.3	1.0	0.04	#NUM!	1.4E-05
YDL182W	YDL182W	S288C_ctrl_hmg124h_2.JPG.dat	1088.8	34.7	0.9	0.03	#NUM!	5.9E-06
YDL183C	YDL183C	S288C_ctrl_hmg124h_2.JPG.dat	1082.3	93.9	0.9	0.08	#NUM!	2.0E-04
YDL184C	YDL184C	S288C_ctrl_hmg124h_2.JPG.dat	1098.5	72.9	1.0	0.07	#NUM!	1.0E-04
YOR202W	YOR202W	S288C_ctrl_hmg124h_2.JPG.dat	1478.9	171.6	1.0	0.06	#NUM!	0.0E+00
1	1	S288C_ctrl_hmg124h_5.JPG.dat	1504.3	232.4	1.0	0.12	#NUM!	4.2E-04
2	2	S288C_ctrl_hmg124h_5.JPG.dat	1271.3	98.8	1.0	0.06	#NUM!	7.9E-05
3	3	S288C_ctrl_hmg124h_5.JPG.dat	1255.3	74.3	1.0	0.05	#NUM!	3.7E-05
4	4	S288C_ctrl_hmg124h_5.JPG.dat	1246.5	86.9	1.0	0.06	#NUM!	6.6E-05
YFR012W	YFR012W	S288C_ctrl_hmg124h_5.JPG.dat	1426.3	45.7	1.1	0.01	#NUM!	1.1E-05
YFR013W	YFR013W	S288C_ctrl_hmg124h_5.JPG.dat	1320.3	23.5	1.0	0.02	#NUM!	3.0E-06
YFR014C	YFR014C	S288C_ctrl_hmg124h_5.JPG.dat	1345.0	43.9	1.0	0.03	#NUM!	9.7E-06
YFR015C	YFR015C	S288C_ctrl_hmg124h_5.JPG.dat	1300.3	32.4	1.0	0.01	#NUM!	2.3E-05
YFR016C	YFR016C	S288C_ctrl_hmg124h_5.JPG.dat	1221.8	19.6	1.0	0.02	#NUM!	1.8E-06
YFR017C	YFR017C	S288C_ctrl_hmg124h_5.JPG.dat	1294.0	25.3	1.0	0.02	#NUM!	1.4E-06
YFR018C	YFR018C	S288C_ctrl_hmg124h_5.JPG.dat	1352.3	14.4	1.1	0.01	#NUM!	1.4E-07
YFR019W	YFR019W	S288C_ctrl_hmg124h_5.JPG.dat	1300.0	55.0	1.0	0.04	#NUM!	2.1E-05
YFR020W	YFR020W	S288C_ctrl_hmg124h_5.JPG.dat	1274.5	32.4	1.0	0.02	#NUM!	4.0E-06
YFR021W	YFR021W	S288C_ctrl_hmg124h_5.JPG.dat	1269.8	8.0	1.0	0.00	#NUM!	2.4E-08
YFR022W	YFR022W	S288C_ctrl_hmg124h_5.JPG.dat	1276.5	37.1	1.0	0.03	#NUM!	8.5E-06
YFR023W	YFR023W	S288C_ctrl_hmg124h_5.JPG.dat	1350.8	43.7	1.0	0.04	#NUM!	1.6E-05
YFR024C	YFR024C	S288C_ctrl_hmg124h_5.JPG.dat	1309.8	480.4	1.1	0.07	#NUM!	1.3E-03
YFR024C-A	YFR024C-A	S288C_ctrl_hmg124h_5.JPG.dat	1412.5	24.1	1.0	0.02	#NUM!	2.2E-06
YFR025C	YFR025C	S288C_ctrl_hmg124h_5.JPG.dat	0.0	0.0	0.0	0.00	#NUM!	
YFR026C	YFR026C	S288C_ctrl_hmg124h_5.JPG.dat	1317.3	37.9	1.0	0.01	#NUM!	1.3E-05
YFR030W	YFR030W	S288C_ctrl_hmg124h_5.JPG.dat	1275.8	71.8	1.0	0.06	#NUM!	5.0E-05
YFR031C-A	YFR031C-A	S288C_ctrl_hmg124h_5.JPG.dat	1103.5	24.3	0.8	0.01	#NUM!	1.6E-05
YFR032C	YFR032C	S288C_ctrl_hmg124h_5.JPG.dat	1404.3	37.1	1.0	0.03	#NUM!	4.6E-06
YFR032C-A	YFR032C-A	S288C_ctrl_hmg124h_5.JPG.dat	1330.5	38.1	1.0	0.02	#NUM!	4.5E-06
YFR033C	YFR033C	S288C_ctrl_hmg124h_5.JPG.dat	1309.8	43.6	1.0	0.03	#NUM!	9.4E-06
YFR034C	YFR034C	S288C_ctrl_hmg124h_5.JPG.dat	1297.0	42.9	1.0	0.03	#NUM!	1.2E-05
YFR035C	YFR035C	S288C_ctrl_hmg124h_5.JPG.dat	1312.5	20.6	1.0	0.02	#NUM!	1.0E-06
YFR038W	YFR038W	S288C_ctrl_hmg124h_5.JPG.dat	1337.8	65.1	1.0	0.05	#NUM!	3.0E-05
YFR039C	YFR039C	S288C_ctrl_hmg124h_5.JPG.dat	1373.3	56.0	1.0	0.03	#NUM!	1.1E-05
YFR040W	YFR040W	S288C_ctrl_hmg124h_5.JPG.dat	1126.3	50.6	0.9	0.04	#NUM!	1.9E-05
YFR041C	YFR041C	S288C_ctrl_hmg124h_5.JPG.dat	1200.5	31.1	1.0	0.02	#NUM!	4.1E-06
YFR043C	YFR043C	S288C_ctrl_hmg124h_5.JPG.dat	1239.5	46.4	1.0	0.04	#NUM!	1.4E-05
YFR044C	YFR044C	S288C_ctrl_hmg124h_5.JPG.dat	1254.8	26.5	1.0	0.00	#NUM!	1.7E-06
YFR045W	YFR045W	S288C_ctrl_hmg124h_5.JPG.dat	1305.3	39.1	1.0	0.01	#NUM!	1.1E-05
YFR046C	YFR046C	S288C_ctrl_hmg124h_5.JPG.dat	1327.8	50.0	1.0	0.04	#NUM!	1.7E-05
YFR047C	YFR047C	S288C_ctrl_hmg124h_5.JPG.dat	1299.0	32.5	1.0	0.00	#NUM!	2.5E-06
YFR048W	YFR048W	S288C_ctrl_hmg124h_5.JPG.dat	1307.8	49.5	1.0	0.01	#NUM!	2.9E-05
YFR049W	YFR049W	S288C_ctrl_hmg124h_5.JPG.dat	1312.8	50.8	1.0	0.01	#NUM!	1.1E-05
YFR053C	YFR053C	S288C_ctrl_hmg124h_5.JPG.dat	1394.5	44.0	1.0	0.04	#NUM!	1.8E-05
YFR054C	YFR054C	S288C_ctrl_hmg124h_5.JPG.dat	1343.3	13.1	1.0	0.02	#NUM!	8.9E-07
YFR055W	YFR055W	S288C_ctrl_hmg124h_5.JPG.dat	1144.0	151.2	1.0	0.02	#NUM!	1.5E-04
YFR056C	YFR056C	S288C_ctrl_hmg124h_5.JPG.dat	1210.5	53.1	1.0	0.04	#NUM!	2.4E-05
YFR057W	YFR057W	S288C_ctrl_hmg124h_5.JPG.dat	1261.3	41.2	1.0	0.03	#NUM!	5.9E-06
YGL002W	YGL002W	S288C_ctrl_hmg124h_5.JPG.dat	1205.5	23.2	1.0	0.00	#NUM!	6.5E-06
YGL004C	YGL004C	S288C_ctrl_hmg124h_5.JPG.dat	1240.3	33.9	1.0	0.02	#NUM!	2.1E-06
YGL005C	YGL005C	S288C_ctrl_hmg124h_5.JPG.dat	1384.5	20.6	1.0	0.01	#NUM!	4.5E-07
YGL006W	YGL006W	S288C_ctrl_hmg124h_5.JPG.dat	1383.0	45.2	1.0	0.00	#NUM!	2.4E-06
YGL007W	YGL007W	S288C_ctrl_hmg124h_5.JPG.dat	1213.3	43.1	0.9	0.01	#NUM!	2.4E-05
YGL009C	YGL009C	S288C_ctrl_hmg124h_5.JPG.dat	1314.8	32.7	1.0	0.02	#NUM!	3.0E-06
YGL010W	YGL010W	S288C_ctrl_hmg124h_5.JPG.dat	1378.3	5.0	1.0	0.01	#NUM!	1.9E-07
YGL013C	YGL013C	S288C_ctrl_hmg124h_5.JPG.dat	1277.5	27.9	1.0	0.02	#NUM!	3.0E-06
YGL014W	YGL014W	S288C_ctrl_hmg124h_5.JPG.dat	1284.0	41.6	1.0	0.00	#NUM!	7.4E-06
YGL015C	YGL015C	S288C_ctrl_hmg124h_5.JPG.dat	1255.3	21.9	1.0	0.00	#NUM!	8.4E-07
YGL016W	YGL016W	S288C_ctrl_hmg124h_5.JPG.dat	1049.0	16.1	0.9	0.00	#NUM!	9.4E-06
YGL017W	YGL017W	S288C_ctrl_hmg124h_5.JPG.dat	1242.0	48.6	1.0	0.03	#NUM!	1.1E-05
YGL019W	YGL019W	S288C_ctrl_hmg124h_5.JPG.dat	1229.0	62.5	0.9	0.05	#NUM!	4.2E-05
YGL020C	YGL020C	S288C_ctrl_hmg124h_5.JPG.dat	1381.3	15.8	1.0	0.01	#NUM!	5.2E-07
YGL021W	YGL021W	S288C_ctrl_hmg124h_5.JPG.dat	1377.8	25.0	1.0	0.02	#NUM!	1.3E-06
YGL023C	YGL023C	S288C_ctrl_hmg124h_5.JPG.dat	1229.8	51.9	1.0	0.03	#NUM!	1.2E-05
YGL024W	YGL024W	S288C_ctrl_hmg124h_5.JPG.dat	1023.3	683.2	1.0	0.04	#NUM!	5.1E-04
YGL026C	YGL026C	S288C_ctrl_hmg124h_5.JPG.dat	0.0	0.0	0.0	0.00	#NUM!	
YGL027C	YGL027C	S288C_ctrl_hmg124h_5.JPG.dat	1355.5	35.6	1.0	0.01	#NUM!	9.3E-06
YGL028C	YGL028C	S288C_ctrl_hmg124h_5.JPG.dat	1307.3	19.8	1.0	0.00	#NUM!	2.4E-06
YGL031C	YGL031C	S288C_ctrl_hmg124h_5.JPG.dat	1237.3	21.2	1.0	0.02	#NUM!	1.7E-06

YGL032C	YGL032C	S288C_ctrl_hmg124h_5.JPG.dat	1205.5	33.4	1.0	0.00	#NUM!	9.8E-07
YGL034C	YGL034C	S288C_ctrl_hmg124h_5.JPG.dat	1263.5	37.2	1.0	0.02	#NUM!	2.2E-06
YGL035C	YGL035C	S288C_ctrl_hmg124h_5.JPG.dat	1343.5	34.5	1.0	0.02	#NUM!	3.8E-06
YGL036W	YGL036W	S288C_ctrl_hmg124h_5.JPG.dat	1400.5	24.1	1.0	0.01	#NUM!	9.5E-06
YGL039W	YGL039W	S288C_ctrl_hmg124h_5.JPG.dat	1411.5	18.0	1.0	0.02	#NUM!	1.0E-06
YGL041C	YGL041C	S288C_ctrl_hmg124h_5.JPG.dat	1348.8	31.1	1.0	0.04	#NUM!	1.4E-05
YGL043W	YGL043W	S288C_ctrl_hmg124h_5.JPG.dat	1295.5	65.6	0.9	0.03	#NUM!	6.5E-06
YGL045W	YGL045W	S288C_ctrl_hmg124h_5.JPG.dat	1373.0	56.6	0.9	0.03	#NUM!	9.1E-06
YGL046W	YGL046W	S288C_ctrl_hmg124h_5.JPG.dat	1361.8	68.4	1.0	0.03	#NUM!	8.2E-06
YGL050W	YGL050W	S288C_ctrl_hmg124h_5.JPG.dat	1388.5	59.5	1.0	0.03	#NUM!	5.2E-06
YGL051W	YGL051W	S288C_ctrl_hmg124h_5.JPG.dat	1350.5	54.8	1.0	0.02	#NUM!	1.7E-06
YGL053W	YGL053W	S288C_ctrl_hmg124h_5.JPG.dat	1413.0	107.6	1.0	0.06	#NUM!	4.4E-05
YGL054C	YGL054C	S288C_ctrl_hmg124h_5.JPG.dat	1457.5	65.1	1.0	0.00	#NUM!	5.5E-06
YGL056C	YGL056C	S288C_ctrl_hmg124h_5.JPG.dat	1494.0	95.1	1.0	0.05	#NUM!	2.7E-05
YGL057C	YGL057C	S288C_ctrl_hmg124h_5.JPG.dat	1453.8	39.6	1.0	0.00	#NUM!	3.3E-06
YGL058W	YGL058W	S288C_ctrl_hmg124h_5.JPG.dat	1437.5	41.0	1.0	0.00	#NUM!	6.6E-06
YGL059W	YGL059W	S288C_ctrl_hmg124h_5.JPG.dat	1366.3	43.9	1.0	0.05	#NUM!	2.8E-05
YGL060W	YGL060W	S288C_ctrl_hmg124h_5.JPG.dat	1432.8	75.6	1.1	0.06	#NUM!	4.9E-05
YGL062W	YGL062W	S288C_ctrl_hmg124h_5.JPG.dat	1364.8	25.3	1.0	0.00	#NUM!	5.6E-06
YGL066W	YGL066W	S288C_ctrl_hmg124h_5.JPG.dat	1316.3	8.8	1.0	0.01	#NUM!	4.0E-07
YGL067W	YGL067W	S288C_ctrl_hmg124h_5.JPG.dat	1302.3	36.9	1.0	0.03	#NUM!	1.2E-05
YGL077C	YGL077C	S288C_ctrl_hmg124h_5.JPG.dat	1218.8	13.9	1.0	0.01	#NUM!	3.2E-07
YGL078C	YGL078C	S288C_ctrl_hmg124h_5.JPG.dat	1082.0	16.5	0.9	0.01	#NUM!	1.3E-05
YGL079W	YGL079W	S288C_ctrl_hmg124h_5.JPG.dat	1287.3	28.1	1.0	0.03	#NUM!	4.0E-06
YGL080W	YGL080W	S288C_ctrl_hmg124h_5.JPG.dat	1238.8	44.4	1.0	0.03	#NUM!	1.3E-05
YGL081W	YGL081W	S288C_ctrl_hmg124h_5.JPG.dat	1301.0	22.2	1.0	0.02	#NUM!	1.3E-06
YGL082W	YGL082W	S288C_ctrl_hmg124h_5.JPG.dat	1274.0	26.5	1.0	0.02	#NUM!	2.9E-06
YGL083W	YGL083W	S288C_ctrl_hmg124h_5.JPG.dat	1289.0	45.4	1.0	0.04	#NUM!	1.2E-05
YGL084C	YGL084C	S288C_ctrl_hmg124h_5.JPG.dat	1278.0	53.2	0.9	0.04	#NUM!	2.5E-05
YGL085W	YGL085W	S288C_ctrl_hmg124h_5.JPG.dat	1454.5	29.8	1.0	0.02	#NUM!	3.1E-06
YGL086W	YGL086W	S288C_ctrl_hmg124h_5.JPG.dat	1381.5	59.4	1.0	0.04	#NUM!	2.0E-05
YGL087C	YGL087C	S288C_ctrl_hmg124h_5.JPG.dat	1441.8	20.5	1.1	0.03	#NUM!	4.2E-06
YGL089C	YGL089C	S288C_ctrl_hmg124h_5.JPG.dat	1359.8	29.6	1.0	0.02	#NUM!	1.7E-06
YGL090W	YGL090W	S288C_ctrl_hmg124h_5.JPG.dat	1280.8	68.2	1.0	0.05	#NUM!	4.1E-05
YGL101W	YGL101W	S288C_ctrl_hmg124h_5.JPG.dat	1276.0	26.0	1.0	0.01	#NUM!	1.2E-05
YGL104C	YGL104C	S288C_ctrl_hmg124h_5.JPG.dat	1303.0	26.4	1.0	0.02	#NUM!	3.3E-06
YGL105W	YGL105W	S288C_ctrl_hmg124h_5.JPG.dat	719.3	831.6	0.5	0.61	#NUM!	1.8E-01
YGL108C	YGL108C	S288C_ctrl_hmg124h_5.JPG.dat	1363.0	16.1	1.0	0.00	#NUM!	2.0E-06
YGL109W	YGL109W	S288C_ctrl_hmg124h_5.JPG.dat	1325.3	7.1	1.0	0.01	#NUM!	2.3E-07
YGL110C	YGL110C	S288C_ctrl_hmg124h_5.JPG.dat	1277.0	27.4	1.0	0.00	#NUM!	4.2E-06
YGL114W	YGL114W	S288C_ctrl_hmg124h_5.JPG.dat	1398.8	24.4	1.0	0.01	#NUM!	3.2E-07
YGL115W	YGL115W	S288C_ctrl_hmg124h_5.JPG.dat	1199.5	40.6	0.9	0.03	#NUM!	1.4E-05
YGL117W	YGL117W	S288C_ctrl_hmg124h_5.JPG.dat	1369.5	10.8	1.1	0.01	#NUM!	5.2E-08
YGL118C	YGL118C	S288C_ctrl_hmg124h_5.JPG.dat	1265.5	14.2	1.0	0.02	#NUM!	1.2E-06
YGL121C	YGL121C	S288C_ctrl_hmg124h_5.JPG.dat	1270.5	32.5	1.0	0.03	#NUM!	7.0E-06
YGL124C	YGL124C	S288C_ctrl_hmg124h_5.JPG.dat	932.5	235.1	0.8	0.19	#NUM!	4.3E-03
YGL125W	YGL125W	S288C_ctrl_hmg124h_5.JPG.dat	1320.8	36.0	1.0	0.01	#NUM!	1.1E-05
YGL126W	YGL126W	S288C_ctrl_hmg124h_5.JPG.dat	1326.5	16.0	1.0	0.01	#NUM!	6.0E-07
YGL127C	YGL127C	S288C_ctrl_hmg124h_5.JPG.dat	1141.5	30.9	0.9	0.02	#NUM!	4.7E-06
YGL131C	YGL131C	S288C_ctrl_hmg124h_5.JPG.dat	1310.5	13.5	1.0	0.01	#NUM!	2.9E-07
YGL132W	YGL132W	S288C_ctrl_hmg124h_5.JPG.dat	1291.5	22.7	1.0	0.02	#NUM!	1.7E-06
YGL133W	YGL133W	S288C_ctrl_hmg124h_5.JPG.dat	1306.5	35.9	1.0	0.03	#NUM!	1.2E-05
YGL136C	YGL136C	S288C_ctrl_hmg124h_5.JPG.dat	742.8	620.8	0.6	0.48	#NUM!	9.8E-02
YGL138C	YGL138C	S288C_ctrl_hmg124h_5.JPG.dat	1264.3	23.7	1.0	0.01	#NUM!	2.0E-05
YGL139W	YGL139W	S288C_ctrl_hmg124h_5.JPG.dat	1219.3	23.2	1.0	0.00	#NUM!	8.3E-06
YGL140C	YGL140C	S288C_ctrl_hmg124h_5.JPG.dat	1256.5	18.2	1.0	0.02	#NUM!	1.1E-06
YGL141W	YGL141W	S288C_ctrl_hmg124h_5.JPG.dat	1209.3	6.7	1.0	0.00	#NUM!	2.9E-07
YGL144C	YGL144C	S288C_ctrl_hmg124h_5.JPG.dat	1260.3	28.9	1.0	0.01	#NUM!	1.5E-05
YGL146C	YGL146C	S288C_ctrl_hmg124h_5.JPG.dat	1285.0	37.4	1.0	0.01	#NUM!	3.4E-05
YGL147C	YGL147C	S288C_ctrl_hmg124h_5.JPG.dat	337.0	674.0	0.0	0.00	#NUM!	
YGL148W	YGL148W	S288C_ctrl_hmg124h_5.JPG.dat	1356.8	49.0	1.0	0.03	#NUM!	1.0E-05
YGL149W	YGL149W	S288C_ctrl_hmg124h_5.JPG.dat	1185.5	62.3	0.9	0.04	#NUM!	2.1E-05
YGL151W	YGL151W	S288C_ctrl_hmg124h_5.JPG.dat	1354.8	43.7	1.0	0.03	#NUM!	5.6E-06
YGL152C	YGL152C	S288C_ctrl_hmg124h_5.JPG.dat	1250.0	22.1	1.0	0.01	#NUM!	4.1E-07
YGL153W	YGL153W	S288C_ctrl_hmg124h_5.JPG.dat	1192.5	13.5	1.0	0.01	#NUM!	8.8E-07
YGL154C	YGL154C	S288C_ctrl_hmg124h_5.JPG.dat	289.3	578.5	0.0	0.00	#NUM!	
YGL156W	YGL156W	S288C_ctrl_hmg124h_5.JPG.dat	1271.8	61.4	1.1	0.05	#NUM!	3.1E-05
YGL157W	YGL157W	S288C_ctrl_hmg124h_5.JPG.dat	1218.5	19.8	1.0	0.02	#NUM!	1.8E-06
YGL158W	YGL158W	S288C_ctrl_hmg124h_5.JPG.dat	1305.5	35.8	1.0	0.02	#NUM!	2.5E-06
YGL159W	YGL159W	S288C_ctrl_hmg124h_5.JPG.dat	1345.0	10.4	1.0	0.01	#NUM!	6.9E-08

YGL160W	YGL160W	S288C_ctrl_hmg124h_5.JPG.dat	1269.8	45.6	1.0	0.01	#NUM!		2.9E-05
YGL161C	YGL161C	S288C_ctrl_hmg124h_5.JPG.dat	1364.0	25.5	1.0	0.01	#NUM!		7.4E-07
YGL162W	YGL162W	S288C_ctrl_hmg124h_5.JPG.dat	1322.8	25.6	1.0	0.02	#NUM!		9.7E-07
YGL163C	YGL163C	S288C_ctrl_hmg124h_5.JPG.dat	1227.0	13.9	0.9	0.01	#NUM!		5.5E-07
YGL164C	YGL164C	S288C_ctrl_hmg124h_5.JPG.dat	1411.5	61.8	1.1	0.04	#NUM!		2.0E-05
YGL165C	YGL165C	S288C_ctrl_hmg124h_5.JPG.dat	1304.0	18.8	1.0	0.00	#NUM!		2.6E-10
YGL166W	YGL166W	S288C_ctrl_hmg124h_5.JPG.dat	1253.3	9.6	1.0	0.02	#NUM!		1.5E-06
YGL167C	YGL167C	S288C_ctrl_hmg124h_5.JPG.dat	1119.8	95.2	0.9	0.02	#NUM!		1.3E-04
YGL168W	YGL168W	S288C_ctrl_hmg124h_5.JPG.dat	1102.8	85.8	0.9	0.01	#NUM!		3.1E-05
YGL170C	YGL170C	S288C_ctrl_hmg124h_5.JPG.dat	1283.8	41.2	1.0	0.02	#NUM!		4.0E-06
YGL173C	YGL173C	S288C_ctrl_hmg124h_5.JPG.dat	1054.5	46.9	0.8	0.04	#NUM!		2.5E-05
YGL174W	YGL174W	S288C_ctrl_hmg124h_5.JPG.dat	1207.8	17.1	0.9	0.01	#NUM!		7.7E-07
YGL175C	YGL175C	S288C_ctrl_hmg124h_5.JPG.dat	1376.0	22.1	1.0	0.00	#NUM!		5.8E-07
YGL176C	YGL176C	S288C_ctrl_hmg124h_5.JPG.dat	1363.5	28.5	1.0	0.02	#NUM!		1.0E-06
YGL177W	YGL177W	S288C_ctrl_hmg124h_5.JPG.dat	1407.0	34.5	1.0	0.04	#NUM!		2.5E-05
YGL179C	YGL179C	S288C_ctrl_hmg124h_5.JPG.dat	1486.5	57.8	1.0	0.02	#NUM!		1.5E-06
YGL180W	YGL180W	S288C_ctrl_hmg124h_5.JPG.dat	1391.5	38.0	1.0	0.01	#NUM!		9.2E-06
YGL181W	YGL181W	S288C_ctrl_hmg124h_5.JPG.dat	1394.5	47.9	1.0	0.04	#NUM!		1.2E-05
YGL194C	YGL194C	S288C_ctrl_hmg124h_5.JPG.dat	1270.8	35.2	0.9	0.00	#NUM!		2.1E-08
YGL195W	YGL195W	S288C_ctrl_hmg124h_5.JPG.dat	1344.5	81.7	1.0	0.04	#NUM!		1.4E-05
YGL196W	YGL196W	S288C_ctrl_hmg124h_5.JPG.dat	1113.3	745.2	1.1	0.04	#NUM!		4.2E-04
YGL197W	YGL197W	S288C_ctrl_hmg124h_5.JPG.dat	1425.5	37.8	1.0	0.01	#NUM!		7.9E-07
YGL198W	YGL198W	S288C_ctrl_hmg124h_5.JPG.dat	1456.5	10.6	1.0	0.01	#NUM!		6.9E-07
YGL199C	YGL199C	S288C_ctrl_hmg124h_5.JPG.dat	1403.8	69.9	1.0	0.07	#NUM!		8.3E-05
YGL202W	YGL202W	S288C_ctrl_hmg124h_5.JPG.dat	1202.3	51.0	0.8	0.00	#NUM!		9.4E-06
YGL203C	YGL203C	S288C_ctrl_hmg124h_5.JPG.dat	1371.5	36.8	1.0	0.01	#NUM!		1.2E-05
YGL205W	YGL205W	S288C_ctrl_hmg124h_5.JPG.dat	1401.0	61.4	1.0	0.03	#NUM!		8.5E-06
YGL208W	YGL208W	S288C_ctrl_hmg124h_5.JPG.dat	1383.8	13.0	1.0	0.03	#NUM!		5.7E-06
YGL209W	YGL209W	S288C_ctrl_hmg124h_5.JPG.dat	1336.8	51.9	1.0	0.02	#NUM!		1.0E-06
YGL210W	YGL210W	S288C_ctrl_hmg124h_5.JPG.dat	1350.3	16.9	1.0	0.03	#NUM!		7.5E-06
YGL211W	YGL211W	S288C_ctrl_hmg124h_5.JPG.dat	1174.3	86.8	0.9	0.04	#NUM!		3.2E-05
YGL212W	YGL212W	S288C_ctrl_hmg124h_5.JPG.dat	1188.3	238.9	0.9	0.16	#NUM!		1.8E-03
YGL213C	YGL213C	S288C_ctrl_hmg124h_5.JPG.dat	1346.8	61.7	1.0	0.02	#NUM!		2.4E-06
YGL214W	YGL214W	S288C_ctrl_hmg124h_5.JPG.dat	944.5	629.9	1.0	0.03	#NUM!		2.3E-04
YGL215W	YGL215W	S288C_ctrl_hmg124h_5.JPG.dat	1375.8	34.1	1.0	0.01	#NUM!		3.4E-05
YGL216W	YGL216W	S288C_ctrl_hmg124h_5.JPG.dat	1412.8	84.0	1.0	0.06	#NUM!		6.6E-05
YGL217C	YGL217C	S288C_ctrl_hmg124h_5.JPG.dat	1441.3	48.9	1.0	0.03	#NUM!		5.0E-06
YGL218W	YGL218W	S288C_ctrl_hmg124h_5.JPG.dat	422.5	681.1	0.1	0.10	#NUM!		4.0E-01
YGL219C	YGL219C	S288C_ctrl_hmg124h_5.JPG.dat	1149.3	12.8	0.9	0.01	#NUM!		8.1E-08
YGL221C	YGL221C	S288C_ctrl_hmg124h_5.JPG.dat	1252.3	26.1	1.0	0.02	#NUM!		1.4E-06
YGL222C	YGL222C	S288C_ctrl_hmg124h_5.JPG.dat	1253.5	63.7	1.0	0.05	#NUM!		3.6E-05
YGL224C	YGL224C	S288C_ctrl_hmg124h_5.JPG.dat	1350.3	15.7	1.0	0.00	#NUM!		1.7E-07
YGL226C-A	YGL226C-A	S288C_ctrl_hmg124h_5.JPG.dat	1351.3	19.1	1.0	0.01	#NUM!		5.3E-07
YGL226W	YGL226W	S288C_ctrl_hmg124h_5.JPG.dat	1282.0	38.4	1.0	0.02	#NUM!		3.2E-06
YGL227W	YGL227W	S288C_ctrl_hmg124h_5.JPG.dat	1320.8	22.8	1.0	0.02	#NUM!		4.0E-06
YGL228W	YGL228W	S288C_ctrl_hmg124h_5.JPG.dat	1328.0	98.9	1.0	0.08	#NUM!		1.4E-04
YGL229C	YGL229C	S288C_ctrl_hmg124h_5.JPG.dat	1377.0	39.7	1.0	0.03	#NUM!		7.9E-06
YGL230C	YGL230C	S288C_ctrl_hmg124h_5.JPG.dat	1441.0	33.0	1.0	0.03	#NUM!		4.6E-06
YGL231C	YGL231C	S288C_ctrl_hmg124h_5.JPG.dat	1372.8	14.4	1.0	0.01	#NUM!		8.1E-07
YGL232W	YGL232W	S288C_ctrl_hmg124h_5.JPG.dat	1322.3	41.9	1.0	0.02	#NUM!		1.4E-06
YGL234W	YGL234W	S288C_ctrl_hmg124h_5.JPG.dat	1278.5	41.5	1.0	0.03	#NUM!		8.8E-06
YGL235W	YGL235W	S288C_ctrl_hmg124h_5.JPG.dat	1221.8	25.3	1.0	0.01	#NUM!		1.2E-05
YGL236C	YGL236C	S288C_ctrl_hmg124h_5.JPG.dat	1312.0	7.5	1.0	0.01	#NUM!		1.1E-05
YGL237C	YGL237C	S288C_ctrl_hmg124h_5.JPG.dat	358.3	716.5	0.0	0.00	#NUM!		
YGL241W	YGL241W	S288C_ctrl_hmg124h_5.JPG.dat	1276.3	19.1	1.0	0.02	#NUM!		4.8E-06
YGL242C	YGL242C	S288C_ctrl_hmg124h_5.JPG.dat	1334.3	32.7	1.0	0.03	#NUM!		7.6E-06
YGL243W	YGL243W	S288C_ctrl_hmg124h_5.JPG.dat	1331.0	90.8	1.0	0.06	#NUM!		4.5E-05
YGL244W	YGL244W	S288C_ctrl_hmg124h_5.JPG.dat	1176.3	25.8	0.9	0.01	#NUM!		1.0E-06
YGL248W	YGL248W	S288C_ctrl_hmg124h_5.JPG.dat	1393.3	36.4	1.0	0.03	#NUM!		5.1E-06
YGL249W	YGL249W	S288C_ctrl_hmg124h_5.JPG.dat	1337.8	42.5	1.0	0.03	#NUM!		8.9E-06
YGL250W	YGL250W	S288C_ctrl_hmg124h_5.JPG.dat	1289.8	17.2	1.0	0.01	#NUM!		3.8E-07
YGL251C	YGL251C	S288C_ctrl_hmg124h_5.JPG.dat	1225.3	18.6	1.0	0.02	#NUM!		1.3E-06
YGL252C	YGL252C	S288C_ctrl_hmg124h_5.JPG.dat	1228.3	47.2	1.0	0.04	#NUM!		1.5E-05
YGL253W	YGL253W	S288C_ctrl_hmg124h_5.JPG.dat	1248.0	14.7	1.0	0.01	#NUM!		2.7E-07
YGL254W	YGL254W	S288C_ctrl_hmg124h_5.JPG.dat	1308.8	39.2	1.0	0.01	#NUM!		3.8E-05
YGL255W	YGL255W	S288C_ctrl_hmg124h_5.JPG.dat	1422.8	60.6	1.1	0.05	#NUM!		2.2E-05
YGL256W	YGL256W	S288C_ctrl_hmg124h_5.JPG.dat	1400.0	37.7	1.0	0.03	#NUM!		5.3E-06
YGL257C	YGL257C	S288C_ctrl_hmg124h_5.JPG.dat	1242.5	42.2	1.0	0.03	#NUM!		1.2E-05
YGL258W	YGL258W	S288C_ctrl_hmg124h_5.JPG.dat	1353.0	63.4	1.0	0.01	#NUM!		5.4E-05
YGL259W	YGL259W	S288C_ctrl_hmg124h_5.JPG.dat	1409.8	19.1	1.0	0.00	#NUM!		2.0E-06

YGL260W	YGL260W	S288C_ctrl_hmg124h_5.JPG.dat	1330.3	18.0	1.0	0.00	#NUM!	2.8E-06
YGL261C	YGL261C	S288C_ctrl_hmg124h_5.JPG.dat	1260.8	19.6	1.0	0.00	#NUM!	3.7E-06
YGL262W	YGL262W	S288C_ctrl_hmg124h_5.JPG.dat	1236.3	23.9	1.0	0.02	#NUM!	1.9E-06
YGL263W	YGL263W	S288C_ctrl_hmg124h_5.JPG.dat	1199.3	42.6	1.0	0.03	#NUM!	1.2E-05
YGR001C	YGR001C	S288C_ctrl_hmg124h_5.JPG.dat	1262.8	10.4	1.0	0.00	#NUM!	1.7E-06
YGR003W	YGR003W	S288C_ctrl_hmg124h_5.JPG.dat	1300.8	40.0	1.0	0.02	#NUM!	4.1E-06
YGR004W	YGR004W	S288C_ctrl_hmg124h_5.JPG.dat	1323.8	9.9	1.0	0.01	#NUM!	5.7E-07
YGR007W	YGR007W	S288C_ctrl_hmg124h_5.JPG.dat	1382.3	19.5	1.0	0.02	#NUM!	9.5E-07
YGR008C	YGR008C	S288C_ctrl_hmg124h_5.JPG.dat	1335.5	45.3	1.0	0.03	#NUM!	6.7E-06
YGR010W	YGR010W	S288C_ctrl_hmg124h_5.JPG.dat	1396.3	55.4	1.0	0.03	#NUM!	9.1E-06
YGR011W	YGR011W	S288C_ctrl_hmg124h_5.JPG.dat	1401.0	68.7	1.0	0.05	#NUM!	2.8E-05
YGR012W	YGR012W	S288C_ctrl_hmg124h_5.JPG.dat	1286.5	30.6	1.0	0.02	#NUM!	2.9E-06
YGR014W	YGR014W	S288C_ctrl_hmg124h_5.JPG.dat	1281.8	38.3	1.0	0.01	#NUM!	1.9E-05
YGR015C	YGR015C	S288C_ctrl_hmg124h_5.JPG.dat	1210.8	29.4	1.0	0.02	#NUM!	3.4E-06
YGR016W	YGR016W	S288C_ctrl_hmg124h_5.JPG.dat	1228.8	31.4	1.0	0.03	#NUM!	4.4E-06
YGR017W	YGR017W	S288C_ctrl_hmg124h_5.JPG.dat	1242.8	30.0	1.0	0.01	#NUM!	8.3E-07
YGR018C	YGR018C	S288C_ctrl_hmg124h_5.JPG.dat	1366.8	34.1	1.0	0.02	#NUM!	2.8E-06
YGR019W	YGR019W	S288C_ctrl_hmg124h_5.JPG.dat	1369.5	38.1	1.0	0.03	#NUM!	5.4E-06
YGR021W	YGR021W	S288C_ctrl_hmg124h_5.JPG.dat	1445.5	14.5	1.0	0.01	#NUM!	3.1E-07
YGR022C	YGR022C	S288C_ctrl_hmg124h_5.JPG.dat	1410.8	56.0	1.0	0.02	#NUM!	8.5E-05
YGR023W	YGR023W	S288C_ctrl_hmg124h_5.JPG.dat	1353.3	12.4	1.0	0.02	#NUM!	1.4E-06
YGR025W	YGR025W	S288C_ctrl_hmg124h_5.JPG.dat	1436.5	30.3	1.0	0.02	#NUM!	1.3E-06
YGR026W	YGR026W	S288C_ctrl_hmg124h_5.JPG.dat	1414.3	54.9	1.1	0.01	#NUM!	1.1E-05
YGR027C	YGR027C	S288C_ctrl_hmg124h_5.JPG.dat	1185.0	48.9	0.9	0.04	#NUM!	3.1E-05
YGR028W	YGR028W	S288C_ctrl_hmg124h_5.JPG.dat	1241.0	25.5	1.0	0.02	#NUM!	1.9E-06
YGR031W	YGR031W	S288C_ctrl_hmg124h_5.JPG.dat	1269.5	47.0	1.0	0.03	#NUM!	5.2E-06
YGR032W	YGR032W	S288C_ctrl_hmg124h_5.JPG.dat	1282.0	41.6	1.0	0.03	#NUM!	1.1E-05
YGR033C	YGR033C	S288C_ctrl_hmg124h_5.JPG.dat	1373.8	38.4	1.0	0.02	#NUM!	2.6E-06
YGR034W	YGR034W	S288C_ctrl_hmg124h_5.JPG.dat	1288.3	10.0	0.9	0.01	#NUM!	1.0E-07
YGR035C	YGR035C	S288C_ctrl_hmg124h_5.JPG.dat	1410.0	47.7	1.0	0.01	#NUM!	4.6E-05
YGR037C	YGR037C	S288C_ctrl_hmg124h_5.JPG.dat	1282.8	75.7	1.0	0.05	#NUM!	3.4E-05
YGR038W	YGR038W	S288C_ctrl_hmg124h_5.JPG.dat	1539.0	43.7	1.1	0.00	#NUM!	5.0E-06
YGR039W	YGR039W	S288C_ctrl_hmg124h_5.JPG.dat	1440.3	18.7	1.0	0.03	#NUM!	5.6E-06
YGR040W	YGR040W	S288C_ctrl_hmg124h_5.JPG.dat	1385.0	34.2	1.0	0.01	#NUM!	6.5E-07
YGR041W	YGR041W	S288C_ctrl_hmg124h_5.JPG.dat	1344.3	37.6	1.0	0.00	#NUM!	8.7E-09
YGR042W	YGR042W	S288C_ctrl_hmg124h_5.JPG.dat	1343.8	65.4	1.0	0.02	#NUM!	1.9E-06
YGR043C	YGR043C	S288C_ctrl_hmg124h_5.JPG.dat	1276.8	33.4	1.0	0.00	#NUM!	2.7E-06
YGR044C	YGR044C	S288C_ctrl_hmg124h_5.JPG.dat	1295.3	31.9	1.0	0.02	#NUM!	1.3E-06
YGR045C	YGR045C	S288C_ctrl_hmg124h_5.JPG.dat	1420.8	82.1	1.0	0.04	#NUM!	1.2E-05
YGR049W	YGR049W	S288C_ctrl_hmg124h_5.JPG.dat	1347.0	63.2	1.0	0.02	#NUM!	3.2E-06
YGR050C	YGR050C	S288C_ctrl_hmg124h_5.JPG.dat	1387.8	64.6	1.0	0.02	#NUM!	3.8E-06
YGR051C	YGR051C	S288C_ctrl_hmg124h_5.JPG.dat	1369.0	34.5	1.0	0.01	#NUM!	2.3E-07
YGR052W	YGR052W	S288C_ctrl_hmg124h_5.JPG.dat	1387.5	33.0	1.0	0.03	#NUM!	9.3E-06
YGR053C	YGR053C	S288C_ctrl_hmg124h_5.JPG.dat	1451.0	32.8	1.0	0.02	#NUM!	1.6E-06
YGR054W	YGR054W	S288C_ctrl_hmg124h_5.JPG.dat	1406.8	43.8	1.0	0.04	#NUM!	1.4E-05
YGR058W	YGR058W	S288C_ctrl_hmg124h_5.JPG.dat	1353.0	44.4	1.0	0.02	#NUM!	4.1E-06
YGR059W	YGR059W	S288C_ctrl_hmg124h_5.JPG.dat	1308.8	56.8	1.0	0.01	#NUM!	4.7E-05
YGR066C	YGR066C	S288C_ctrl_hmg124h_5.JPG.dat	1237.5	25.6	1.0	0.02	#NUM!	2.8E-06
YGR069W	YGR069W	S288C_ctrl_hmg124h_5.JPG.dat	1250.5	22.0	1.0	0.02	#NUM!	1.3E-06
YGR070W	YGR070W	S288C_ctrl_hmg124h_5.JPG.dat	1329.5	37.8	1.0	0.03	#NUM!	7.1E-06
YGR071C	YGR071C	S288C_ctrl_hmg124h_5.JPG.dat	1409.0	16.3	1.1	0.01	#NUM!	7.7E-07
YGR072W	YGR072W	S288C_ctrl_hmg124h_5.JPG.dat	1301.8	75.5	1.0	0.05	#NUM!	4.1E-05
YGR077C	YGR077C	S288C_ctrl_hmg124h_5.JPG.dat	1229.3	49.3	0.9	0.03	#NUM!	9.9E-06
YGR078C	YGR078C	S288C_ctrl_hmg124h_5.JPG.dat	1170.5	43.7	0.9	0.04	#NUM!	2.4E-05
YGR079W	YGR079W	S288C_ctrl_hmg124h_5.JPG.dat	1406.3	54.3	1.0	0.03	#NUM!	8.2E-06
YGR080W	YGR080W	S288C_ctrl_hmg124h_5.JPG.dat	1392.3	28.2	1.0	0.01	#NUM!	9.1E-06
YGR081C	YGR081C	S288C_ctrl_hmg124h_5.JPG.dat	1161.8	22.7	0.9	0.01	#NUM!	5.1E-07
YGR084C	YGR084C	S288C_ctrl_hmg124h_5.JPG.dat	1250.0	45.6	1.0	0.03	#NUM!	1.1E-05
YGR085C	YGR085C	S288C_ctrl_hmg124h_5.JPG.dat	1226.3	70.9	1.0	0.01	#NUM!	6.0E-05
YGR086C	YGR086C	S288C_ctrl_hmg124h_5.JPG.dat	1260.5	39.7	1.0	0.02	#NUM!	1.8E-06
YGR087C	YGR087C	S288C_ctrl_hmg124h_5.JPG.dat	1355.5	62.3	1.0	0.04	#NUM!	1.3E-05
YGR088W	YGR088W	S288C_ctrl_hmg124h_5.JPG.dat	1384.0	75.5	1.0	0.05	#NUM!	2.5E-05
YGR089W	YGR089W	S288C_ctrl_hmg124h_5.JPG.dat	1359.3	3.5	1.0	0.01	#NUM!	2.0E-07
YGR092W	YGR092W	S288C_ctrl_hmg124h_5.JPG.dat	824.8	690.6	0.6	0.52	#NUM!	9.7E-02
YGR093W	YGR093W	S288C_ctrl_hmg124h_5.JPG.dat	1282.8	19.7	1.0	0.02	#NUM!	1.7E-06
YGR096W	YGR096W	S288C_ctrl_hmg124h_5.JPG.dat	1425.3	19.2	1.0	0.01	#NUM!	8.4E-07
YGR097W	YGR097W	S288C_ctrl_hmg124h_5.JPG.dat	1066.5	712.0	1.0	0.03	#NUM!	2.1E-04
YGR100W	YGR100W	S288C_ctrl_hmg124h_5.JPG.dat	1274.0	18.4	1.0	0.01	#NUM!	1.2E-05
YGR101W	YGR101W	S288C_ctrl_hmg124h_5.JPG.dat	782.3	65.1	0.6	0.05	#NUM!	1.8E-04
YGR105W	YGR105W	S288C_ctrl_hmg124h_5.JPG.dat	1262.3	58.4	1.0	0.05	#NUM!	2.9E-05

YGR106C	YGR106C	S288C_ctrl_hmg124h_5.JPG.dat	1255.5	31.8	1.0	0.03	#NUM!	5.5E-06
YGR107W	YGR107W	S288C_ctrl_hmg124h_5.JPG.dat	1288.8	18.0	1.0	0.00	#NUM!	2.4E-06
YGR108W	YGR108W	S288C_ctrl_hmg124h_5.JPG.dat	1341.3	65.2	1.0	0.05	#NUM!	2.9E-05
YGR109C	YGR109C	S288C_ctrl_hmg124h_5.JPG.dat	1444.0	54.2	1.1	0.04	#NUM!	1.3E-05
YGR110W	YGR110W	S288C_ctrl_hmg124h_5.JPG.dat	1411.8	26.6	1.0	0.02	#NUM!	1.4E-06
YGR111W	YGR111W	S288C_ctrl_hmg124h_5.JPG.dat	1257.0	91.2	1.0	0.06	#NUM!	7.2E-05
YGR117C	YGR117C	S288C_ctrl_hmg124h_5.JPG.dat	1428.0	20.8	1.0	0.01	#NUM!	2.0E-05
YGR118W	YGR118W	S288C_ctrl_hmg124h_5.JPG.dat	1164.5	38.2	0.9	0.03	#NUM!	9.5E-06
YGR121C	YGR121C	S288C_ctrl_hmg124h_5.JPG.dat	1326.0	36.5	1.0	0.02	#NUM!	1.2E-06
YGR123C	YGR123C	S288C_ctrl_hmg124h_5.JPG.dat	1247.3	46.8	1.0	0.01	#NUM!	4.6E-05
YGR124W	YGR124W	S288C_ctrl_hmg124h_5.JPG.dat	1242.5	38.1	1.0	0.01	#NUM!	1.3E-05
YGR125W	YGR125W	S288C_ctrl_hmg124h_5.JPG.dat	1247.3	32.6	1.0	0.01	#NUM!	3.6E-05
YGR126W	YGR126W	S288C_ctrl_hmg124h_5.JPG.dat	1241.8	15.0	1.0	0.01	#NUM!	6.4E-07
YGR127W	YGR127W	S288C_ctrl_hmg124h_5.JPG.dat	1329.5	28.5	1.0	0.02	#NUM!	1.8E-06
YGR129W	YGR129W	S288C_ctrl_hmg124h_5.JPG.dat	1353.0	44.0	1.0	0.04	#NUM!	1.4E-05
YGR130C	YGR130C	S288C_ctrl_hmg124h_5.JPG.dat	1361.3	7.5	1.0	0.01	#NUM!	8.4E-07
YGR131W	YGR131W	S288C_ctrl_hmg124h_5.JPG.dat	1333.8	35.1	1.0	0.03	#NUM!	4.7E-06
YGR132C	YGR132C	S288C_ctrl_hmg124h_5.JPG.dat	1390.5	33.5	1.0	0.02	#NUM!	3.0E-06
YGR133W	YGR133W	S288C_ctrl_hmg124h_5.JPG.dat	1254.0	35.8	0.9	0.02	#NUM!	3.2E-06
YGR134W	YGR134W	S288C_ctrl_hmg124h_5.JPG.dat	1216.5	19.8	0.9	0.01	#NUM!	2.3E-05
YGR135W	YGR135W	S288C_ctrl_hmg124h_5.JPG.dat	1120.8	134.6	0.9	0.11	#NUM!	5.2E-04
YGR136W	YGR136W	S288C_ctrl_hmg124h_5.JPG.dat	1201.8	41.5	1.0	0.03	#NUM!	1.2E-05
YGR137W	YGR137W	S288C_ctrl_hmg124h_5.JPG.dat	1221.3	43.2	1.0	0.03	#NUM!	1.2E-05
YGR138C	YGR138C	S288C_ctrl_hmg124h_5.JPG.dat	1308.8	30.1	1.0	0.02	#NUM!	1.8E-06
YGR139W	YGR139W	S288C_ctrl_hmg124h_5.JPG.dat	1359.5	25.8	1.0	0.01	#NUM!	1.8E-05
YGR141W	YGR141W	S288C_ctrl_hmg124h_5.JPG.dat	1341.0	35.6	1.0	0.02	#NUM!	3.2E-06
YGR142W	YGR142W	S288C_ctrl_hmg124h_5.JPG.dat	1367.5	19.9	1.0	0.02	#NUM!	1.8E-06
YGR143W	YGR143W	S288C_ctrl_hmg124h_5.JPG.dat	1361.0	43.1	1.0	0.04	#NUM!	1.3E-05
YGR144W	YGR144W	S288C_ctrl_hmg124h_5.JPG.dat	1460.8	29.8	1.1	0.03	#NUM!	8.4E-06
YGR146C	YGR146C	S288C_ctrl_hmg124h_5.JPG.dat	1378.0	27.4	1.0	0.02	#NUM!	1.1E-06
YGR148C	YGR148C	S288C_ctrl_hmg124h_5.JPG.dat	1201.5	40.5	0.9	0.02	#NUM!	4.5E-06
YGR149W	YGR149W	S288C_ctrl_hmg124h_5.JPG.dat	1277.3	35.1	1.0	0.02	#NUM!	1.4E-06
YGR151C	YGR151C	S288C_ctrl_hmg124h_5.JPG.dat	1288.3	43.2	1.0	0.02	#NUM!	2.8E-06
YGR152C	YGR152C	S288C_ctrl_hmg124h_5.JPG.dat	1265.0	62.5	1.0	0.04	#NUM!	1.9E-05
YGR153W	YGR153W	S288C_ctrl_hmg124h_5.JPG.dat	1327.3	25.0	1.0	0.02	#NUM!	3.6E-06
YGR154C	YGR154C	S288C_ctrl_hmg124h_5.JPG.dat	1376.8	35.3	1.0	0.02	#NUM!	3.6E-06
YGR157W	YGR157W	S288C_ctrl_hmg124h_5.JPG.dat	768.5	478.9	0.7	0.07	#NUM!	3.4E-03
YGR161C	YGR161C	S288C_ctrl_hmg124h_5.JPG.dat	1407.8	22.9	1.0	0.02	#NUM!	1.4E-06
YOR202W	YOR202W	S288C_ctrl_hmg124h_5.JPG.dat	1455.6	182.9	1.0	0.07	#NUM!	2.4238860[illegible]
1	1	S288C_ctrl_hmg124h_6.JPG.dat	1496.8	142.9	1.1	0.01	#NUM!	3.7E-05
2	2	S288C_ctrl_hmg124h_6.JPG.dat	1327.3	80.1	1.0	0.04	#NUM!	1.5E-05
3	3	S288C_ctrl_hmg124h_6.JPG.dat	1245.0	109.5	1.0	0.07	#NUM!	9.6E-05
4	4	S288C_ctrl_hmg124h_6.JPG.dat	1239.5	83.7	1.0	0.05	#NUM!	3.3E-05
YGR163W	YGR163W	S288C_ctrl_hmg124h_6.JPG.dat	1162.3	60.0	0.9	0.04	#NUM!	2.6E-05
YGR164W	YGR164W	S288C_ctrl_hmg124h_6.JPG.dat	1358.3	97.6	1.0	0.07	#NUM!	9.0E-05
YGR166W	YGR166W	S288C_ctrl_hmg124h_6.JPG.dat	1324.5	22.7	1.0	0.01	#NUM!	5.1E-07
YGR168C	YGR168C	S288C_ctrl_hmg124h_6.JPG.dat	1334.5	22.4	1.0	0.01	#NUM!	3.4E-07
YGR169C	YGR169C	S288C_ctrl_hmg124h_6.JPG.dat	1311.3	18.2	1.0	0.01	#NUM!	1.1E-07
YGR170W	YGR170W	S288C_ctrl_hmg124h_6.JPG.dat	1302.3	19.3	1.0	0.01	#NUM!	1.5E-07
YGR173W	YGR173W	S288C_ctrl_hmg124h_6.JPG.dat	1341.8	20.2	1.0	0.01	#NUM!	2.7E-07
YGR174C	YGR174C	S288C_ctrl_hmg124h_6.JPG.dat	1213.5	9.1	0.9	0.01	#NUM!	1.0E-06
YGR176W	YGR176W	S288C_ctrl_hmg124h_6.JPG.dat	1347.5	55.0	1.0	0.05	#NUM!	2.4E-05
YGR177C	YGR177C	S288C_ctrl_hmg124h_6.JPG.dat	1270.3	16.8	1.0	0.01	#NUM!	5.1E-07
YGR178C	YGR178C	S288C_ctrl_hmg124h_6.JPG.dat	1282.0	21.4	1.1	0.00	#NUM!	1.1E-06
YGR181W	YGR181W	S288C_ctrl_hmg124h_6.JPG.dat	1305.3	105.8	1.0	0.02	#NUM!	1.4E-04
YGR182C	YGR182C	S288C_ctrl_hmg124h_6.JPG.dat	1312.8	4.4	1.0	0.01	#NUM!	3.7E-07
YGR183C	YGR183C	S288C_ctrl_hmg124h_6.JPG.dat	803.3	541.7	0.8	0.07	#NUM!	2.6E-03
YGR184C	YGR184C	S288C_ctrl_hmg124h_6.JPG.dat	1267.3	25.5	1.0	0.01	#NUM!	3.5E-07
YGR187C	YGR187C	S288C_ctrl_hmg124h_6.JPG.dat	1241.8	31.7	1.0	0.00	#NUM!	7.9E-06
YGR188C	YGR188C	S288C_ctrl_hmg124h_6.JPG.dat	1102.5	73.4	0.9	0.06	#NUM!	1.1E-04
YGR189C	YGR189C	S288C_ctrl_hmg124h_6.JPG.dat	1313.5	18.9	1.0	0.00	#NUM!	3.5E-06
YGR192C	YGR192C	S288C_ctrl_hmg124h_6.JPG.dat	1264.0	22.8	1.0	0.01	#NUM!	8.2E-07
YGR193C	YGR193C	S288C_ctrl_hmg124h_6.JPG.dat	1360.5	44.0	1.1	0.03	#NUM!	4.3E-06
YGR194C	YGR194C	S288C_ctrl_hmg124h_6.JPG.dat	1291.3	69.0	1.1	0.02	#NUM!	6.7E-05
YGR196C	YGR196C	S288C_ctrl_hmg124h_6.JPG.dat	1240.5	44.4	1.0	0.02	#NUM!	2.8E-06
YGR197C	YGR197C	S288C_ctrl_hmg124h_6.JPG.dat	1309.5	41.4	1.0	0.03	#NUM!	4.9E-06
YGR199W	YGR199W	S288C_ctrl_hmg124h_6.JPG.dat	1388.3	18.9	1.0	0.02	#NUM!	1.8E-06
YGR200C	YGR200C	S288C_ctrl_hmg124h_6.JPG.dat	1127.5	16.9	0.8	0.00	#NUM!	6.2E-06
YGR201C	YGR201C	S288C_ctrl_hmg124h_6.JPG.dat	1305.5	16.9	1.0	0.01	#NUM!	5.5E-07
YGR202C	YGR202C	S288C_ctrl_hmg124h_6.JPG.dat	1319.8	27.0	1.0	0.02	#NUM!	2.0E-06

YGR203W	YGR203W	S288C_ctrl_hmg124h_6.JPG.dat	1246.8	50.2	1.0	0.04	#NUM!	1.8E-05
YGR205W	YGR205W	S288C_ctrl_hmg124h_6.JPG.dat	1257.0	12.6	1.0	0.01	#NUM!	5.5E-07
YGR206W	YGR206W	S288C_ctrl_hmg124h_6.JPG.dat	1279.8	30.6	1.0	0.03	#NUM!	4.4E-06
YGR207C	YGR207C	S288C_ctrl_hmg124h_6.JPG.dat	1285.5	23.6	1.0	0.02	#NUM!	3.0E-06
YGR208W	YGR208W	S288C_ctrl_hmg124h_6.JPG.dat	1237.8	29.2	1.0	0.02	#NUM!	1.9E-06
YGR209C	YGR209C	S288C_ctrl_hmg124h_6.JPG.dat	1246.0	36.9	1.0	0.02	#NUM!	1.7E-06
YGR210C	YGR210C	S288C_ctrl_hmg124h_6.JPG.dat	1357.0	45.1	1.0	0.04	#NUM!	1.1E-05
YGR212W	YGR212W	S288C_ctrl_hmg124h_6.JPG.dat	1361.0	21.3	1.0	0.02	#NUM!	2.7E-06
YGR213C	YGR213C	S288C_ctrl_hmg124h_6.JPG.dat	1287.8	30.7	1.0	0.03	#NUM!	6.6E-06
YGR214W	YGR214W	S288C_ctrl_hmg124h_6.JPG.dat	924.3	73.1	0.7	0.06	#NUM!	1.3E-04
YGR217W	YGR217W	S288C_ctrl_hmg124h_6.JPG.dat	1269.5	28.4	1.0	0.01	#NUM!	8.5E-06
YGR221C	YGR221C	S288C_ctrl_hmg124h_6.JPG.dat	1241.3	26.6	1.0	0.01	#NUM!	6.5E-07
YGR223C	YGR223C	S288C_ctrl_hmg124h_6.JPG.dat	1162.3	56.3	1.0	0.02	#NUM!	9.8E-05
YGR224W	YGR224W	S288C_ctrl_hmg124h_6.JPG.dat	1226.3	41.0	1.0	0.01	#NUM!	3.6E-05
YGR225W	YGR225W	S288C_ctrl_hmg124h_6.JPG.dat	1251.0	50.2	1.0	0.00	#NUM!	5.9E-07
YGR226C	YGR226C	S288C_ctrl_hmg124h_6.JPG.dat	1315.0	69.6	1.0	0.06	#NUM!	4.5E-05
YGR227W	YGR227W	S288C_ctrl_hmg124h_6.JPG.dat	1267.0	69.0	1.0	0.04	#NUM!	2.1E-05
YGR228W	YGR228W	S288C_ctrl_hmg124h_6.JPG.dat	1308.8	17.8	1.0	0.02	#NUM!	2.6E-06
YGR229C	YGR229C	S288C_ctrl_hmg124h_6.JPG.dat	1180.0	69.1	0.9	0.02	#NUM!	1.2E-04
YGR230W	YGR230W	S288C_ctrl_hmg124h_6.JPG.dat	1302.8	29.0	1.0	0.01	#NUM!	1.3E-05
YGR231C	YGR231C	S288C_ctrl_hmg124h_6.JPG.dat	1060.8	49.0	0.9	0.04	#NUM!	2.7E-05
YGR232W	YGR232W	S288C_ctrl_hmg124h_6.JPG.dat	1230.8	36.9	1.0	0.03	#NUM!	7.7E-06
YGR233C	YGR233C	S288C_ctrl_hmg124h_6.JPG.dat	1209.8	28.1	1.0	0.02	#NUM!	2.5E-06
YGR234W	YGR234W	S288C_ctrl_hmg124h_6.JPG.dat	1172.3	97.3	1.0	0.08	#NUM!	1.8E-04
YGR235C	YGR235C	S288C_ctrl_hmg124h_6.JPG.dat	1222.0	37.6	1.0	0.03	#NUM!	9.1E-06
YGR236C	YGR236C	S288C_ctrl_hmg124h_6.JPG.dat	1266.0	34.0	1.0	0.02	#NUM!	1.7E-06
YGR237C	YGR237C	S288C_ctrl_hmg124h_6.JPG.dat	1235.5	65.1	1.0	0.05	#NUM!	3.2E-05
YGR238C	YGR238C	S288C_ctrl_hmg124h_6.JPG.dat	1200.5	43.4	1.0	0.03	#NUM!	1.0E-05
YGR239C	YGR239C	S288C_ctrl_hmg124h_6.JPG.dat	1311.5	7.0	1.0	0.00	#NUM!	1.6E-08
YGR241C	YGR241C	S288C_ctrl_hmg124h_6.JPG.dat	1314.5	31.8	1.0	0.01	#NUM!	1.7E-05
YGR242W	YGR242W	S288C_ctrl_hmg124h_6.JPG.dat	1281.8	37.6	1.0	0.02	#NUM!	2.3E-06
YGR243W	YGR243W	S288C_ctrl_hmg124h_6.JPG.dat	1308.0	52.8	1.0	0.04	#NUM!	1.3E-05
YGR244C	YGR244C	S288C_ctrl_hmg124h_6.JPG.dat	1314.3	16.8	1.0	0.01	#NUM!	7.0E-07
YGR247W	YGR247W	S288C_ctrl_hmg124h_6.JPG.dat	1246.0	12.5	1.0	0.02	#NUM!	1.1E-06
YGR248W	YGR248W	S288C_ctrl_hmg124h_6.JPG.dat	1207.5	83.3	1.0	0.02	#NUM!	1.7E-04
YGR249W	YGR249W	S288C_ctrl_hmg124h_6.JPG.dat	1287.5	11.7	1.0	0.01	#NUM!	6.2E-08
YGR250C	YGR250C	S288C_ctrl_hmg124h_6.JPG.dat	1245.5	76.4	1.0	0.01	#NUM!	6.7E-05
YGR256W	YGR256W	S288C_ctrl_hmg124h_6.JPG.dat	1188.5	92.3	0.9	0.08	#NUM!	1.4E-04
YGR259C	YGR259C	S288C_ctrl_hmg124h_6.JPG.dat	1161.5	48.6	0.9	0.04	#NUM!	1.7E-05
YGR260W	YGR260W	S288C_ctrl_hmg124h_6.JPG.dat	1261.8	120.3	0.9	0.03	#NUM!	3.3E-04
YGR261C	YGR261C	S288C_ctrl_hmg124h_6.JPG.dat	1420.5	52.2	1.0	0.02	#NUM!	3.6E-06
YGR263C	YGR263C	S288C_ctrl_hmg124h_6.JPG.dat	1499.8	44.8	1.0	0.01	#NUM!	7.1E-07
YGR266W	YGR266W	S288C_ctrl_hmg124h_6.JPG.dat	1420.5	97.7	1.0	0.05	#NUM!	3.3E-05
YGR268C	YGR268C	S288C_ctrl_hmg124h_6.JPG.dat	1382.3	53.3	1.0	0.03	#NUM!	7.5E-06
YGR269W	YGR269W	S288C_ctrl_hmg124h_6.JPG.dat	1370.5	26.3	1.0	0.02	#NUM!	2.1E-06
YGR270W	YGR270W	S288C_ctrl_hmg124h_6.JPG.dat	1169.5	118.8	0.8	0.01	#NUM!	2.1E-05
YGR275W	YGR275W	S288C_ctrl_hmg124h_6.JPG.dat	1344.0	57.2	1.0	0.02	#NUM!	4.1E-06
YGR279C	YGR279C	S288C_ctrl_hmg124h_6.JPG.dat	1397.0	21.2	1.0	0.02	#NUM!	1.4E-06
YGR281W	YGR281W	S288C_ctrl_hmg124h_6.JPG.dat	1321.0	47.9	1.0	0.01	#NUM!	3.9E-05
YGR282C	YGR282C	S288C_ctrl_hmg124h_6.JPG.dat	1135.0	60.0	0.9	0.06	#NUM!	9.6E-05
YGR283C	YGR283C	S288C_ctrl_hmg124h_6.JPG.dat	1210.3	27.4	1.0	0.01	#NUM!	3.0E-07
YGR284C	YGR284C	S288C_ctrl_hmg124h_6.JPG.dat	1343.5	25.7	1.0	0.02	#NUM!	1.5E-06
YGR286C	YGR286C	S288C_ctrl_hmg124h_6.JPG.dat	1335.3	21.3	1.0	0.01	#NUM!	2.0E-05
YGR287C	YGR287C	S288C_ctrl_hmg124h_6.JPG.dat	1352.0	39.4	1.0	0.01	#NUM!	1.5E-05
YGR288W	YGR288W	S288C_ctrl_hmg124h_6.JPG.dat	1328.3	13.5	1.0	0.01	#NUM!	1.6E-07
YGR290W	YGR290W	S288C_ctrl_hmg124h_6.JPG.dat	1339.5	13.7	1.0	0.02	#NUM!	8.8E-07
YGR295C	YGR295C	S288C_ctrl_hmg124h_6.JPG.dat	1318.8	26.2	1.0	0.01	#NUM!	6.5E-07
YHL002W	YHL002W	S288C_ctrl_hmg124h_6.JPG.dat	1338.5	7.9	1.0	0.01	#NUM!	7.9E-08
YHL003C	YHL003C	S288C_ctrl_hmg124h_6.JPG.dat	1261.3	49.8	1.0	0.03	#NUM!	8.7E-06
YHL005C	YHL005C	S288C_ctrl_hmg124h_6.JPG.dat	1181.5	74.0	0.9	0.06	#NUM!	7.4E-05
YHL006C	YHL006C	S288C_ctrl_hmg124h_6.JPG.dat	1250.3	12.5	1.0	0.01	#NUM!	4.8E-07
YHL007C	YHL007C	S288C_ctrl_hmg124h_6.JPG.dat	615.3	746.9	0.5	0.60	#NUM!	2.0E-01
YHL008C	YHL008C	S288C_ctrl_hmg124h_6.JPG.dat	1335.3	47.3	1.0	0.04	#NUM!	1.4E-05
YHL009C	YHL009C	S288C_ctrl_hmg124h_6.JPG.dat	1369.3	19.6	1.0	0.01	#NUM!	1.7E-05
YHL010C	YHL010C	S288C_ctrl_hmg124h_6.JPG.dat	1366.8	27.0	1.0	0.02	#NUM!	9.9E-07
YHL012W	YHL012W	S288C_ctrl_hmg124h_6.JPG.dat	1315.0	33.7	1.0	0.02	#NUM!	4.0E-06
YHL013C	YHL013C	S288C_ctrl_hmg124h_6.JPG.dat	1262.5	70.2	1.0	0.01	#NUM!	6.8E-05
YHL014C	YHL014C	S288C_ctrl_hmg124h_6.JPG.dat	1259.0	30.8	1.0	0.02	#NUM!	1.4E-06
YHL016C	YHL016C	S288C_ctrl_hmg124h_6.JPG.dat	1283.8	70.4	1.0	0.05	#NUM!	3.3E-05
YHL017W	YHL017W	S288C_ctrl_hmg124h_6.JPG.dat	1292.5	38.6	1.0	0.03	#NUM!	9.3E-06

YHL019C	YHL019C	S288C_ctrl_hmg124h_6.JPG.dat	1236.5	51.3	1.0	0.03	#NUM!	1.2E-05
YHL020C	YHL020C	S288C_ctrl_hmg124h_6.JPG.dat	1196.0	37.5	1.0	0.03	#NUM!	1.0E-05
YHL021C	YHL021C	S288C_ctrl_hmg124h_6.JPG.dat	1319.0	56.7	1.0	0.06	#NUM!	4.2E-05
YHL022C	YHL022C	S288C_ctrl_hmg124h_6.JPG.dat	1383.3	31.2	1.0	0.02	#NUM!	2.9E-06
YHL023C	YHL023C	S288C_ctrl_hmg124h_6.JPG.dat	1110.3	59.6	0.8	0.05	#NUM!	5.0E-05
YHL024W	YHL024W	S288C_ctrl_hmg124h_6.JPG.dat	1310.8	25.5	1.0	0.02	#NUM!	1.4E-06
YHL025W	YHL025W	S288C_ctrl_hmg124h_6.JPG.dat	842.5	226.3	0.7	0.18	#NUM!	5.2E-03
YHL026C	YHL026C	S288C_ctrl_hmg124h_6.JPG.dat	1216.0	50.5	1.0	0.04	#NUM!	1.7E-05
YHL027W	YHL027W	S288C_ctrl_hmg124h_6.JPG.dat	1137.3	119.2	0.9	0.10	#NUM!	3.3E-04
YHL028W	YHL028W	S288C_ctrl_hmg124h_6.JPG.dat	1258.0	16.8	1.0	0.00	#NUM!	5.7E-06
YHL029C	YHL029C	S288C_ctrl_hmg124h_6.JPG.dat	1200.5	21.9	0.9	0.01	#NUM!	1.1E-06
YHL030W	YHL030W	S288C_ctrl_hmg124h_6.JPG.dat	1284.3	50.4	1.0	0.04	#NUM!	1.3E-05
YHL031C	YHL031C	S288C_ctrl_hmg124h_6.JPG.dat	909.5	91.7	0.7	0.07	#NUM!	2.5E-04
YHL032C	YHL032C	S288C_ctrl_hmg124h_6.JPG.dat	1307.8	23.2	1.0	0.04	#NUM!	1.2E-05
YHL033C	YHL033C	S288C_ctrl_hmg124h_6.JPG.dat	1114.3	54.2	0.8	0.01	#NUM!	2.3E-05
YHL034C	YHL034C	S288C_ctrl_hmg124h_6.JPG.dat	1180.3	7.9	0.9	0.01	#NUM!	7.8E-07
YHL035C	YHL035C	S288C_ctrl_hmg124h_6.JPG.dat	1233.5	60.3	1.0	0.05	#NUM!	4.9E-05
YHL036W	YHL036W	S288C_ctrl_hmg124h_6.JPG.dat	1249.0	32.3	1.0	0.02	#NUM!	3.4E-06
YHL037C	YHL037C	S288C_ctrl_hmg124h_6.JPG.dat	1245.5	101.2	1.0	0.08	#NUM!	1.2E-04
YHL039W	YHL039W	S288C_ctrl_hmg124h_6.JPG.dat	1227.5	9.5	1.0	0.01	#NUM!	1.2E-07
YHL040C	YHL040C	S288C_ctrl_hmg124h_6.JPG.dat	896.8	600.1	1.0	0.04	#NUM!	6.8E-04
YHL041W	YHL041W	S288C_ctrl_hmg124h_6.JPG.dat	1161.0	72.7	0.9	0.05	#NUM!	4.6E-05
YHL042W	YHL042W	S288C_ctrl_hmg124h_6.JPG.dat	1287.0	44.3	1.0	0.03	#NUM!	1.1E-05
YHL043W	YHL043W	S288C_ctrl_hmg124h_6.JPG.dat	1262.3	29.1	1.0	0.03	#NUM!	4.5E-06
YHL044W	YHL044W	S288C_ctrl_hmg124h_6.JPG.dat	1294.8	45.6	1.1	0.04	#NUM!	1.2E-05
YHL045W	YHL045W	S288C_ctrl_hmg124h_6.JPG.dat	1354.5	100.6	1.1	0.03	#NUM!	2.1E-04
YHL046C	YHL046C	S288C_ctrl_hmg124h_6.JPG.dat	1314.5	33.4	1.0	0.02	#NUM!	1.3E-06
YHL047C	YHL047C	S288C_ctrl_hmg124h_6.JPG.dat	1213.8	39.8	1.0	0.04	#NUM!	1.9E-05
YHR001W-	YHR001W-A	S288C_ctrl_hmg124h_6.JPG.dat	1224.0	18.1	1.0	0.01	#NUM!	1.6E-07
YHR003C	YHR003C	S288C_ctrl_hmg124h_6.JPG.dat	1177.3	18.0	1.0	0.01	#NUM!	1.4E-07
YHR004C	YHR004C	S288C_ctrl_hmg124h_6.JPG.dat	1173.3	53.3	1.0	0.04	#NUM!	2.6E-05
YHR005C	YHR005C	S288C_ctrl_hmg124h_6.JPG.dat	1173.5	59.4	1.0	0.02	#NUM!	9.8E-05
YHR006W	YHR006W	S288C_ctrl_hmg124h_6.JPG.dat	1298.0	21.3	1.1	0.02	#NUM!	3.5E-06
YHR009C	YHR009C	S288C_ctrl_hmg124h_6.JPG.dat	1282.5	55.5	1.0	0.02	#NUM!	1.0E-04
YHR012W	YHR012W	S288C_ctrl_hmg124h_6.JPG.dat	947.8	254.3	0.8	0.21	#NUM!	5.5E-03
YHR014W	YHR014W	S288C_ctrl_hmg124h_6.JPG.dat	1276.3	13.3	1.0	0.00	#NUM!	5.8E-06
YHR015W	YHR015W	S288C_ctrl_hmg124h_6.JPG.dat	1319.5	13.5	1.0	0.01	#NUM!	4.9E-08
YHR016C	YHR016C	S288C_ctrl_hmg124h_6.JPG.dat	1312.5	24.8	1.0	0.02	#NUM!	1.0E-06
YHR017W	YHR017W	S288C_ctrl_hmg124h_6.JPG.dat	1265.3	14.2	1.0	0.01	#NUM!	6.9E-07
YHR018C	YHR018C	S288C_ctrl_hmg124h_6.JPG.dat	0.0	0.0	0.0	0.00	#NUM!	
YHR021C	YHR021C	S288C_ctrl_hmg124h_6.JPG.dat	824.3	297.1	0.5	0.05	#NUM!	3.0E-03
YHR021W-	YHR021W-A	S288C_ctrl_hmg124h_6.JPG.dat	1098.5	162.7	0.9	0.13	#NUM!	8.1E-04
YHR022C	YHR022C	S288C_ctrl_hmg124h_6.JPG.dat	1201.0	89.8	1.0	0.07	#NUM!	9.9E-05
YHR025W	YHR025W	S288C_ctrl_hmg124h_6.JPG.dat	604.3	62.8	0.5	0.05	#NUM!	3.3E-04
YHR028C	YHR028C	S288C_ctrl_hmg124h_6.JPG.dat	1282.5	26.6	1.0	0.02	#NUM!	3.3E-06
YHR029C	YHR029C	S288C_ctrl_hmg124h_6.JPG.dat	1311.3	26.1	1.0	0.02	#NUM!	1.5E-06
YHR030C	YHR030C	S288C_ctrl_hmg124h_6.JPG.dat	1382.8	98.3	1.0	0.05	#NUM!	3.8E-05
YHR031C	YHR031C	S288C_ctrl_hmg124h_6.JPG.dat	1422.8	108.8	1.0	0.08	#NUM!	1.3E-04
YHR032W	YHR032W	S288C_ctrl_hmg124h_6.JPG.dat	1469.0	25.2	1.0	0.02	#NUM!	2.2E-06
YHR033W	YHR033W	S288C_ctrl_hmg124h_6.JPG.dat	1443.0	69.0	1.0	0.02	#NUM!	1.4E-06
YHR034C	YHR034C	S288C_ctrl_hmg124h_6.JPG.dat	1366.0	92.9	1.0	0.07	#NUM!	9.7E-05
YHR035W	YHR035W	S288C_ctrl_hmg124h_6.JPG.dat	1363.5	31.7	1.0	0.00	#NUM!	1.8E-06
YHR037W	YHR037W	S288C_ctrl_hmg124h_6.JPG.dat	1357.5	38.5	1.0	0.01	#NUM!	6.5E-07
YHR039C	YHR039C	S288C_ctrl_hmg124h_6.JPG.dat	1304.8	69.1	1.0	0.03	#NUM!	7.1E-06
YHR041C	YHR041C	S288C_ctrl_hmg124h_6.JPG.dat	1325.8	32.8	1.0	0.01	#NUM!	5.5E-05
YHR043C	YHR043C	S288C_ctrl_hmg124h_6.JPG.dat	1248.5	75.1	0.9	0.05	#NUM!	4.6E-05
YHR044C	YHR044C	S288C_ctrl_hmg124h_6.JPG.dat	1220.5	186.6	1.0	0.02	#NUM!	1.6E-04
YHR045W	YHR045W	S288C_ctrl_hmg124h_6.JPG.dat	1306.8	10.0	1.0	0.02	#NUM!	1.3E-06
YHR046C	YHR046C	S288C_ctrl_hmg124h_6.JPG.dat	1389.8	42.7	1.0	0.00	#NUM!	6.8E-06
YHR047C	YHR047C	S288C_ctrl_hmg124h_6.JPG.dat	1341.3	59.5	1.0	0.01	#NUM!	5.4E-05
YHR048W	YHR048W	S288C_ctrl_hmg124h_6.JPG.dat	1342.8	16.5	1.0	0.01	#NUM!	5.0E-07
YHR049C-/	YHR049C-A	S288C_ctrl_hmg124h_6.JPG.dat	1338.3	21.7	1.0	0.00	#NUM!	1.9E-06
YHR049W	YHR049W	S288C_ctrl_hmg124h_6.JPG.dat	1344.5	20.6	1.0	0.02	#NUM!	3.0E-06
YHR050W	YHR050W	S288C_ctrl_hmg124h_6.JPG.dat	1397.8	16.2	1.1	0.02	#NUM!	1.2E-06
YHR057C	YHR057C	S288C_ctrl_hmg124h_6.JPG.dat	1361.8	36.6	1.0	0.02	#NUM!	1.1E-06
YHR059W	YHR059W	S288C_ctrl_hmg124h_6.JPG.dat	303.3	606.5	0.0	0.00	#NUM!	
YHR061C	YHR061C	S288C_ctrl_hmg124h_6.JPG.dat	1240.5	112.8	1.0	0.02	#NUM!	1.0E-04
YHR066W	YHR066W	S288C_ctrl_hmg124h_6.JPG.dat	1124.3	22.5	0.9	0.01	#NUM!	1.8E-05
YHR067W	YHR067W	S288C_ctrl_hmg124h_6.JPG.dat	832.5	88.1	0.6	0.07	#NUM!	2.8E-04
YHR073W	YHR073W	S288C_ctrl_hmg124h_6.JPG.dat	1310.8	32.8	1.0	0.03	#NUM!	7.3E-06

YHR075C	YHR075C	S288C_ctrl_hmg124h_6.JPG.dat	1384.5	61.4	1.0	0.05	#NUM!	3.0E-05
YHR076W	YHR076W	S288C_ctrl_hmg124h_6.JPG.dat	1333.0	28.2	1.0	0.00	#NUM!	7.4E-06
YHR077C	YHR077C	S288C_ctrl_hmg124h_6.JPG.dat	1338.3	32.0	1.0	0.03	#NUM!	4.1E-06
YHR078W	YHR078W	S288C_ctrl_hmg124h_6.JPG.dat	1424.3	28.8	1.1	0.02	#NUM!	2.6E-06
YHR079C	YHR079C	S288C_ctrl_hmg124h_6.JPG.dat	1243.8	29.0	0.9	0.00	#NUM!	4.7E-06
YHR079C-E	YHR079C-B	S288C_ctrl_hmg124h_6.JPG.dat	1332.3	14.2	1.0	0.02	#NUM!	9.3E-07
YHR080C	YHR080C	S288C_ctrl_hmg124h_6.JPG.dat	1294.0	13.4	1.0	0.01	#NUM!	7.2E-07
YHR081W	YHR081W	S288C_ctrl_hmg124h_6.JPG.dat	885.5	177.2	0.7	0.15	#NUM!	2.4E-03
YHR082C	YHR082C	S288C_ctrl_hmg124h_6.JPG.dat	1255.5	39.7	1.0	0.05	#NUM!	2.4E-05
YHR086W	YHR086W	S288C_ctrl_hmg124h_6.JPG.dat	1296.0	28.9	1.0	0.02	#NUM!	1.6E-06
YHR087W	YHR087W	S288C_ctrl_hmg124h_6.JPG.dat	1367.3	42.8	1.0	0.03	#NUM!	7.5E-06
YHR092C	YHR092C	S288C_ctrl_hmg124h_6.JPG.dat	1388.5	28.3	1.0	0.00	#NUM!	7.1E-06
YHR093W	YHR093W	S288C_ctrl_hmg124h_6.JPG.dat	1337.3	34.5	1.0	0.01	#NUM!	2.8E-05
YHR094C	YHR094C	S288C_ctrl_hmg124h_6.JPG.dat	1304.8	28.7	1.0	0.03	#NUM!	4.6E-06
YHR095W	YHR095W	S288C_ctrl_hmg124h_6.JPG.dat	1297.5	26.9	1.0	0.02	#NUM!	1.4E-06
YHR096C	YHR096C	S288C_ctrl_hmg124h_6.JPG.dat	1316.5	6.5	1.0	0.01	#NUM!	3.0E-07
YHR097C	YHR097C	S288C_ctrl_hmg124h_6.JPG.dat	1300.5	23.5	1.0	0.01	#NUM!	6.6E-07
YHR103W	YHR103W	S288C_ctrl_hmg124h_6.JPG.dat	1289.8	13.8	1.0	0.01	#NUM!	2.8E-07
YHR104W	YHR104W	S288C_ctrl_hmg124h_6.JPG.dat	1294.8	37.6	1.0	0.02	#NUM!	3.8E-06
YHR105W	YHR105W	S288C_ctrl_hmg124h_6.JPG.dat	1228.0	13.3	1.0	0.01	#NUM!	8.3E-08
YHR106W	YHR106W	S288C_ctrl_hmg124h_6.JPG.dat	1326.3	7.3	1.0	0.00	#NUM!	4.3E-07
YHR108W	YHR108W	S288C_ctrl_hmg124h_6.JPG.dat	1292.3	32.7	1.0	0.02	#NUM!	4.3E-06
YHR109W	YHR109W	S288C_ctrl_hmg124h_6.JPG.dat	1280.5	20.7	1.0	0.02	#NUM!	1.0E-06
YHR110W	YHR110W	S288C_ctrl_hmg124h_6.JPG.dat	1267.3	70.0	1.0	0.06	#NUM!	4.8E-05
YHR111W	YHR111W	S288C_ctrl_hmg124h_6.JPG.dat	1310.8	36.2	1.0	0.03	#NUM!	6.4E-06
YHR112C	YHR112C	S288C_ctrl_hmg124h_6.JPG.dat	1250.0	22.0	1.0	0.02	#NUM!	1.5E-06
YHR113W	YHR113W	S288C_ctrl_hmg124h_6.JPG.dat	1242.3	14.5	1.0	0.01	#NUM!	2.6E-07
YHR114W	YHR114W	S288C_ctrl_hmg124h_6.JPG.dat	1266.0	16.6	1.0	0.01	#NUM!	8.7E-07
YHR115C	YHR115C	S288C_ctrl_hmg124h_6.JPG.dat	1251.5	26.6	1.0	0.02	#NUM!	2.3E-06
YHR116W	YHR116W	S288C_ctrl_hmg124h_6.JPG.dat	146.3	292.5	0.0	0.00	#NUM!	
YHR117W	YHR117W	S288C_ctrl_hmg124h_6.JPG.dat	1221.8	60.4	1.0	0.01	#NUM!	7.3E-05
YHR121W	YHR121W	S288C_ctrl_hmg124h_6.JPG.dat	1346.3	40.7	1.1	0.01	#NUM!	1.7E-05
YHR123W	YHR123W	S288C_ctrl_hmg124h_6.JPG.dat	1381.8	25.9	1.1	0.02	#NUM!	1.5E-06
YHR124W	YHR124W	S288C_ctrl_hmg124h_6.JPG.dat	1284.8	34.5	1.0	0.00	#NUM!	3.4E-07
YHR125W	YHR125W	S288C_ctrl_hmg124h_6.JPG.dat	1268.5	7.5	1.0	0.01	#NUM!	4.5E-08
YHR126C	YHR126C	S288C_ctrl_hmg124h_6.JPG.dat	1176.3	44.9	1.0	0.04	#NUM!	1.4E-05
YHR127W	YHR127W	S288C_ctrl_hmg124h_6.JPG.dat	1225.8	25.0	1.0	0.02	#NUM!	2.9E-06
YHR129C	YHR129C	S288C_ctrl_hmg124h_6.JPG.dat	1214.3	52.7	1.0	0.04	#NUM!	2.1E-05
YHR130C	YHR130C	S288C_ctrl_hmg124h_6.JPG.dat	1187.0	66.0	1.0	0.05	#NUM!	4.3E-05
YHR131C	YHR131C	S288C_ctrl_hmg124h_6.JPG.dat	1301.3	27.9	1.0	0.01	#NUM!	1.5E-05
YHR132C	YHR132C	S288C_ctrl_hmg124h_6.JPG.dat	1191.5	48.0	0.9	0.04	#NUM!	1.6E-05
YHR132W-	YHR132W-A	S288C_ctrl_hmg124h_6.JPG.dat	1237.3	35.5	1.0	0.01	#NUM!	8.6E-06
YHR133C	YHR133C	S288C_ctrl_hmg124h_6.JPG.dat	1277.0	32.3	1.0	0.03	#NUM!	5.3E-06
YHR134W	YHR134W	S288C_ctrl_hmg124h_6.JPG.dat	1138.5	41.7	0.9	0.03	#NUM!	1.4E-05
YHR135C	YHR135C	S288C_ctrl_hmg124h_6.JPG.dat	1274.5	19.8	1.0	0.01	#NUM!	3.0E-07
YHR136C	YHR136C	S288C_ctrl_hmg124h_6.JPG.dat	1251.0	48.2	1.0	0.04	#NUM!	1.6E-05
YHR137W	YHR137W	S288C_ctrl_hmg124h_6.JPG.dat	1270.8	9.6	1.0	0.00	#NUM!	4.1E-07
YHR138C	YHR138C	S288C_ctrl_hmg124h_6.JPG.dat	1254.5	26.2	1.0	0.02	#NUM!	1.6E-06
YHR139C	YHR139C	S288C_ctrl_hmg124h_6.JPG.dat	1245.5	26.7	1.0	0.01	#NUM!	2.2E-05
YHR139C-/	YHR139C-A	S288C_ctrl_hmg124h_6.JPG.dat	1267.5	26.0	1.0	0.02	#NUM!	2.0E-06
YHR140W	YHR140W	S288C_ctrl_hmg124h_6.JPG.dat	1251.8	25.1	1.0	0.01	#NUM!	1.6E-05
YHR142W	YHR142W	S288C_ctrl_hmg124h_6.JPG.dat	1356.5	13.5	1.1	0.00	#NUM!	2.4E-06
YHR143W	YHR143W	S288C_ctrl_hmg124h_6.JPG.dat	1315.0	33.7	1.1	0.04	#NUM!	1.1E-05
YHR146W	YHR146W	S288C_ctrl_hmg124h_6.JPG.dat	1337.0	21.4	1.0	0.00	#NUM!	7.1E-06
YHR150W	YHR150W	S288C_ctrl_hmg124h_6.JPG.dat	1358.0	42.4	1.0	0.01	#NUM!	3.6E-05
YHR151C	YHR151C	S288C_ctrl_hmg124h_6.JPG.dat	1347.3	24.1	1.0	0.01	#NUM!	7.5E-07
YHR152W	YHR152W	S288C_ctrl_hmg124h_6.JPG.dat	1363.0	19.5	1.0	0.01	#NUM!	1.5E-07
YHR153C	YHR153C	S288C_ctrl_hmg124h_6.JPG.dat	1278.5	27.6	1.0	0.01	#NUM!	1.1E-05
YHR154W	YHR154W	S288C_ctrl_hmg124h_6.JPG.dat	1297.5	19.4	1.0	0.02	#NUM!	1.0E-06
YHR155W	YHR155W	S288C_ctrl_hmg124h_6.JPG.dat	1301.5	25.1	1.0	0.02	#NUM!	1.5E-06
YHR156C	YHR156C	S288C_ctrl_hmg124h_6.JPG.dat	1262.0	18.9	1.0	0.00	#NUM!	6.1E-06
YHR157W	YHR157W	S288C_ctrl_hmg124h_6.JPG.dat	1266.8	90.4	1.0	0.01	#NUM!	2.3E-05
YHR158C	YHR158C	S288C_ctrl_hmg124h_6.JPG.dat	1356.5	74.7	1.1	0.06	#NUM!	4.3E-05
YHR159W	YHR159W	S288C_ctrl_hmg124h_6.JPG.dat	1121.3	193.5	0.9	0.16	#NUM!	1.5E-03
YHR160C	YHR160C	S288C_ctrl_hmg124h_6.JPG.dat	1281.8	69.7	1.0	0.03	#NUM!	1.1E-05
YHR161C	YHR161C	S288C_ctrl_hmg124h_6.JPG.dat	1340.3	12.3	1.0	0.01	#NUM!	2.0E-07
YHR162W	YHR162W	S288C_ctrl_hmg124h_6.JPG.dat	1278.8	21.1	1.0	0.00	#NUM!	5.8E-07
YHR163W	YHR163W	S288C_ctrl_hmg124h_6.JPG.dat	1310.3	39.3	1.0	0.03	#NUM!	4.8E-06
YHR167W	YHR167W	S288C_ctrl_hmg124h_6.JPG.dat	1288.0	3.6	1.0	0.02	#NUM!	1.2E-06
YHR171W	YHR171W	S288C_ctrl_hmg124h_6.JPG.dat	1268.3	64.3	1.0	0.04	#NUM!	2.7E-05

YHR176W	YHR176W	S288C_ctrl_hmg124h_6.JPG.dat	1353.8	59.6	1.0	0.03	#NUM!	6.5E-06
YHR178W	YHR178W	S288C_ctrl_hmg124h_6.JPG.dat	1119.5	163.1	0.8	0.03	#NUM!	5.5E-04
YHR179W	YHR179W	S288C_ctrl_hmg124h_6.JPG.dat	1335.8	39.7	1.0	0.02	#NUM!	1.9E-06
YHR180W	YHR180W	S288C_ctrl_hmg124h_6.JPG.dat	1326.5	27.0	1.0	0.03	#NUM!	7.3E-06
YHR181W	YHR181W	S288C_ctrl_hmg124h_6.JPG.dat	1249.5	38.8	1.0	0.04	#NUM!	2.7E-05
YHR182W	YHR182W	S288C_ctrl_hmg124h_6.JPG.dat	1259.5	52.2	1.0	0.04	#NUM!	1.9E-05
YHR184W	YHR184W	S288C_ctrl_hmg124h_6.JPG.dat	1343.5	25.2	1.0	0.02	#NUM!	1.1E-06
YHR185C	YHR185C	S288C_ctrl_hmg124h_6.JPG.dat	1345.5	15.3	1.0	0.00	#NUM!	2.7E-06
YHR189W	YHR189W	S288C_ctrl_hmg124h_6.JPG.dat	649.5	259.3	0.6	0.05	#NUM!	2.8E-03
YHR191C	YHR191C	S288C_ctrl_hmg124h_6.JPG.dat	1248.8	39.0	1.0	0.01	#NUM!	4.3E-05
YHR193C	YHR193C	S288C_ctrl_hmg124h_6.JPG.dat	334.8	669.5	0.0	0.00	#NUM!	
YHR194W	YHR194W	S288C_ctrl_hmg124h_6.JPG.dat	654.0	755.5	0.5	0.58	#NUM!	1.8E-01
YHR195W	YHR195W	S288C_ctrl_hmg124h_6.JPG.dat	1293.0	28.6	1.0	0.01	#NUM!	1.7E-05
YHR198C	YHR198C	S288C_ctrl_hmg124h_6.JPG.dat	1167.8	90.6	0.9	0.07	#NUM!	1.2E-04
YHR199C	YHR199C	S288C_ctrl_hmg124h_6.JPG.dat	1260.8	48.7	1.0	0.04	#NUM!	1.6E-05
YHR200W	YHR200W	S288C_ctrl_hmg124h_6.JPG.dat	1155.3	40.5	0.9	0.04	#NUM!	1.5E-05
YHR202W	YHR202W	S288C_ctrl_hmg124h_6.JPG.dat	1162.3	58.3	0.9	0.03	#NUM!	1.5E-05
YHR203C	YHR203C	S288C_ctrl_hmg124h_6.JPG.dat	1029.8	54.2	0.8	0.04	#NUM!	3.1E-05
YHR204W	YHR204W	S288C_ctrl_hmg124h_6.JPG.dat	1383.8	11.0	1.0	0.01	#NUM!	7.0E-08
YHR206W	YHR206W	S288C_ctrl_hmg124h_6.JPG.dat	1299.8	29.0	1.0	0.02	#NUM!	1.6E-06
YHR207C	YHR207C	S288C_ctrl_hmg124h_6.JPG.dat	1340.5	5.9	1.0	0.01	#NUM!	3.0E-07
YHR209W	YHR209W	S288C_ctrl_hmg124h_6.JPG.dat	1291.5	44.7	1.0	0.03	#NUM!	7.6E-06
YHR210C	YHR210C	S288C_ctrl_hmg124h_6.JPG.dat	1262.8	48.9	1.0	0.02	#NUM!	8.4E-05
YIL001W	YIL001W	S288C_ctrl_hmg124h_6.JPG.dat	1298.0	16.7	1.0	0.01	#NUM!	3.3E-07
YIL002C	YIL002C	S288C_ctrl_hmg124h_6.JPG.dat	1284.8	18.7	1.0	0.01	#NUM!	6.3E-07
YIL005W	YIL005W	S288C_ctrl_hmg124h_6.JPG.dat	1238.5	34.8	1.0	0.03	#NUM!	8.0E-06
YIL006W	YIL006W	S288C_ctrl_hmg124h_6.JPG.dat	1174.3	31.9	0.9	0.03	#NUM!	7.8E-06
YIL007C	YIL007C	S288C_ctrl_hmg124h_6.JPG.dat	1282.0	34.7	1.0	0.00	#NUM!	1.1E-06
YIL008W	YIL008W	S288C_ctrl_hmg124h_6.JPG.dat	1184.0	20.6	0.9	0.02	#NUM!	1.7E-06
YIL009C-A	YIL009C-A	S288C_ctrl_hmg124h_6.JPG.dat	1339.0	16.2	1.0	0.01	#NUM!	3.1E-07
YIL010W	YIL010W	S288C_ctrl_hmg124h_6.JPG.dat	1296.0	13.8	1.0	0.01	#NUM!	7.6E-08
YIL011W	YIL011W	S288C_ctrl_hmg124h_6.JPG.dat	492.5	588.8	0.4	0.47	#NUM!	1.9E-01
YIL012W	YIL012W	S288C_ctrl_hmg124h_6.JPG.dat	341.0	682.0	0.0	0.00	#NUM!	
YIL013C	YIL013C	S288C_ctrl_hmg124h_6.JPG.dat	1197.0	59.5	1.0	0.05	#NUM!	2.9E-05
YIL014W	YIL014W	S288C_ctrl_hmg124h_6.JPG.dat	1276.0	16.9	1.0	0.01	#NUM!	7.1E-07
YIL015C-A	YIL015C-A	S288C_ctrl_hmg124h_6.JPG.dat	1231.3	44.8	1.0	0.03	#NUM!	1.2E-05
YIL015W	YIL015W	S288C_ctrl_hmg124h_6.JPG.dat	1277.0	18.7	1.0	0.02	#NUM!	1.5E-06
YIL016W	YIL016W	S288C_ctrl_hmg124h_6.JPG.dat	1328.3	37.2	1.1	0.03	#NUM!	6.0E-06
YIL017C	YIL017C	S288C_ctrl_hmg124h_6.JPG.dat	1221.0	26.5	1.0	0.02	#NUM!	4.1E-06
YIL020C	YIL020C	S288C_ctrl_hmg124h_6.JPG.dat	0.0	0.0	0.0	0.00	#NUM!	
YIL023C	YIL023C	S288C_ctrl_hmg124h_6.JPG.dat	1334.3	10.9	1.0	0.01	#NUM!	9.1E-08
YIL024C	YIL024C	S288C_ctrl_hmg124h_6.JPG.dat	1233.5	14.3	1.0	0.01	#NUM!	1.7E-07
YIL025C	YIL025C	S288C_ctrl_hmg124h_6.JPG.dat	1247.8	25.3	1.0	0.02	#NUM!	1.4E-06
YIL027C	YIL027C	S288C_ctrl_hmg124h_6.JPG.dat	1197.0	22.1	1.0	0.00	#NUM!	1.2E-07
YIL028W	YIL028W	S288C_ctrl_hmg124h_6.JPG.dat	1173.0	38.1	1.0	0.03	#NUM!	7.0E-06
YIL029C	YIL029C	S288C_ctrl_hmg124h_6.JPG.dat	1177.5	83.8	1.0	0.07	#NUM!	9.7E-05
YIL030C	YIL030C	S288C_ctrl_hmg124h_6.JPG.dat	1257.3	12.5	1.0	0.01	#NUM!	6.0E-07
YIL032C	YIL032C	S288C_ctrl_hmg124h_6.JPG.dat	1277.5	29.2	1.0	0.02	#NUM!	3.2E-06
YIL034C	YIL034C	S288C_ctrl_hmg124h_6.JPG.dat	1333.8	20.3	1.1	0.02	#NUM!	1.0E-06
YIL035C	YIL035C	S288C_ctrl_hmg124h_6.JPG.dat	1236.3	29.5	1.0	0.00	#NUM!	1.6E-06
YIL036W	YIL036W	S288C_ctrl_hmg124h_6.JPG.dat	1323.5	10.3	1.0	0.00	#NUM!	1.7E-06
YIL037C	YIL037C	S288C_ctrl_hmg124h_6.JPG.dat	1259.3	54.0	0.9	0.01	#NUM!	3.9E-05
YIL038C	YIL038C	S288C_ctrl_hmg124h_6.JPG.dat	1231.3	26.9	1.0	0.02	#NUM!	1.7E-06
YIL039W	YIL039W	S288C_ctrl_hmg124h_6.JPG.dat	1254.8	45.4	1.0	0.04	#NUM!	1.7E-05
YIL040W	YIL040W	S288C_ctrl_hmg124h_6.JPG.dat	990.0	269.3	0.8	0.22	#NUM!	5.3E-03
YIL041W	YIL041W	S288C_ctrl_hmg124h_6.JPG.dat	1235.8	17.6	1.0	0.00	#NUM!	4.7E-06
YIL042C	YIL042C	S288C_ctrl_hmg124h_6.JPG.dat	1212.0	48.1	1.0	0.04	#NUM!	1.7E-05
YIL043C	YIL043C	S288C_ctrl_hmg124h_6.JPG.dat	1233.0	11.9	1.0	0.01	#NUM!	8.8E-08
YIL044C	YIL044C	S288C_ctrl_hmg124h_6.JPG.dat	1114.8	102.4	0.9	0.08	#NUM!	2.1E-04
YIL045W	YIL045W	S288C_ctrl_hmg124h_6.JPG.dat	1272.3	68.8	1.0	0.02	#NUM!	1.1E-04
YIL047C	YIL047C	S288C_ctrl_hmg124h_6.JPG.dat	993.3	663.7	1.0	0.04	#NUM!	4.6E-04
YIL049W	YIL049W	S288C_ctrl_hmg124h_6.JPG.dat	1336.3	34.1	1.0	0.02	#NUM!	2.2E-06
YIL050W	YIL050W	S288C_ctrl_hmg124h_6.JPG.dat	1361.3	14.5	1.0	0.01	#NUM!	9.9E-08
YIL052C	YIL052C	S288C_ctrl_hmg124h_6.JPG.dat	1344.3	61.0	1.0	0.01	#NUM!	4.4E-05
YIL053W	YIL053W	S288C_ctrl_hmg124h_6.JPG.dat	1292.5	42.9	1.0	0.04	#NUM!	1.7E-05
YIL054W	YIL054W	S288C_ctrl_hmg124h_6.JPG.dat	1263.0	20.7	1.0	0.02	#NUM!	2.6E-06
YIL055C	YIL055C	S288C_ctrl_hmg124h_6.JPG.dat	1281.0	55.6	1.0	0.04	#NUM!	2.5E-05
YIL056W	YIL056W	S288C_ctrl_hmg124h_6.JPG.dat	1273.0	12.5	1.0	0.01	#NUM!	3.0E-07
YIL057C	YIL057C	S288C_ctrl_hmg124h_6.JPG.dat	1313.5	15.5	1.0	0.02	#NUM!	8.8E-07
YIL058W	YIL058W	S288C_ctrl_hmg124h_6.JPG.dat	1227.0	178.8	1.0	0.14	#NUM!	8.4E-04

YIL059C	YIL059C	S288C_ctrl_hmg124h_6.JPG.dat	1262.5	108.7	1.0	0.08	#NUM!	1.7E-04
YOR202W	YOR202W	S288C_ctrl_hmg124h_6.JPG.dat	1372.3	155.2	1.0	0.07	#NUM!	1.4920782!
1	1	S288C_ctrl_hmg124h_1.JPG.dat	1566.0	173.7	1.1	0.07	#NUM!	6.6E-05
2	2	S288C_ctrl_hmg124h_1.JPG.dat	1351.8	131.7	1.0	0.06	#NUM!	5.7E-05
3	3	S288C_ctrl_hmg124h_1.JPG.dat	1326.0	110.2	1.0	0.06	#NUM!	4.5E-05
4	4	S288C_ctrl_hmg124h_1.JPG.dat	1259.3	101.7	1.0	0.05	#NUM!	3.5E-05
YAL002W	YAL002W	S288C_ctrl_hmg124h_1.JPG.dat	1490.5	37.4	1.1	0.03	#NUM!	5.9E-06
YAL004W	YAL004W	S288C_ctrl_hmg124h_1.JPG.dat	1474.5	60.5	1.0	0.04	#NUM!	1.1E-05
YAL005C	YAL005C	S288C_ctrl_hmg124h_1.JPG.dat	1397.8	48.0	1.0	0.03	#NUM!	1.1E-05
YAL007C	YAL007C	S288C_ctrl_hmg124h_1.JPG.dat	1356.3	30.0	1.0	0.02	#NUM!	3.2E-06
YAL008W	YAL008W	S288C_ctrl_hmg124h_1.JPG.dat	1393.5	60.5	1.0	0.05	#NUM!	3.2E-05
YAL010C	YAL010C	S288C_ctrl_hmg124h_1.JPG.dat	1179.0	155.0	0.8	0.03	#NUM!	4.9E-04
YAL011W	YAL011W	S288C_ctrl_hmg124h_1.JPG.dat	1245.3	33.7	0.9	0.02	#NUM!	4.9E-06
YAL013W	YAL013W	S288C_ctrl_hmg124h_1.JPG.dat	1210.3	37.0	0.9	0.01	#NUM!	2.3E-05
YAL014C	YAL014C	S288C_ctrl_hmg124h_1.JPG.dat	1357.0	39.1	1.0	0.03	#NUM!	4.9E-06
YAL015C	YAL015C	S288C_ctrl_hmg124h_1.JPG.dat	1302.3	25.6	1.0	0.02	#NUM!	3.3E-06
YAL017W	YAL017W	S288C_ctrl_hmg124h_1.JPG.dat	1234.5	46.0	1.0	0.03	#NUM!	1.0E-05
YAL018C	YAL018C	S288C_ctrl_hmg124h_1.JPG.dat	1432.3	41.7	1.1	0.01	#NUM!	2.1E-05
YAL019W	YAL019W	S288C_ctrl_hmg124h_1.JPG.dat	1388.5	16.4	1.0	0.01	#NUM!	1.6E-07
YAL020C	YAL020C	S288C_ctrl_hmg124h_1.JPG.dat	1246.5	21.2	0.9	0.02	#NUM!	1.7E-06
YAL022C	YAL022C	S288C_ctrl_hmg124h_1.JPG.dat	1314.0	28.6	1.0	0.02	#NUM!	3.0E-06
YAL023C	YAL023C	S288C_ctrl_hmg124h_1.JPG.dat	1414.5	17.9	1.1	0.01	#NUM!	1.6E-07
YAL026C	YAL026C	S288C_ctrl_hmg124h_1.JPG.dat	1121.8	21.0	0.9	0.02	#NUM!	2.9E-06
YAL027W	YAL027W	S288C_ctrl_hmg124h_1.JPG.dat	1301.5	34.2	1.0	0.02	#NUM!	3.3E-06
YAL028W	YAL028W	S288C_ctrl_hmg124h_1.JPG.dat	1368.0	23.3	1.0	0.01	#NUM!	4.9E-07
YAL029C	YAL029C	S288C_ctrl_hmg124h_1.JPG.dat	1382.5	7.2	1.0	0.00	#NUM!	6.6E-09
YAL030W	YAL030W	S288C_ctrl_hmg124h_1.JPG.dat	1373.8	32.8	1.0	0.01	#NUM!	7.1E-07
YAL031C	YAL031C	S288C_ctrl_hmg124h_1.JPG.dat	1204.8	18.8	1.0	0.00	#NUM!	7.4E-06
YAL034C	YAL034C	S288C_ctrl_hmg124h_1.JPG.dat	1258.3	31.0	1.0	0.02	#NUM!	3.4E-06
YAL036C	YAL036C	S288C_ctrl_hmg124h_1.JPG.dat	1413.0	63.4	1.0	0.04	#NUM!	1.9E-05
YAL037W	YAL037W	S288C_ctrl_hmg124h_1.JPG.dat	1363.0	27.3	1.0	0.02	#NUM!	1.2E-06
YAL040C	YAL040C	S288C_ctrl_hmg124h_1.JPG.dat	1257.5	26.6	1.0	0.00	#NUM!	2.3E-07
YAL042W	YAL042W	S288C_ctrl_hmg124h_1.JPG.dat	1244.5	59.1	1.0	0.04	#NUM!	2.5E-05
YAL043C-A	YAL043C-A	S288C_ctrl_hmg124h_1.JPG.dat	1199.3	51.7	1.0	0.04	#NUM!	2.0E-05
YAL045C	YAL045C	S288C_ctrl_hmg124h_1.JPG.dat	1210.0	28.1	1.0	0.02	#NUM!	1.3E-06
YAL049C	YAL049C	S288C_ctrl_hmg124h_1.JPG.dat	1301.5	16.7	1.0	0.01	#NUM!	7.8E-07
YAL051W	YAL051W	S288C_ctrl_hmg124h_1.JPG.dat	1369.5	29.1	1.0	0.02	#NUM!	3.0E-06
YAL053W	YAL053W	S288C_ctrl_hmg124h_1.JPG.dat	1347.5	38.1	1.0	0.02	#NUM!	1.7E-06
YAL054C	YAL054C	S288C_ctrl_hmg124h_1.JPG.dat	1223.8	9.1	1.0	0.02	#NUM!	1.3E-06
YAL055W	YAL055W	S288C_ctrl_hmg124h_1.JPG.dat	1254.5	37.0	1.0	0.03	#NUM!	6.4E-06
YAL056W	YAL056W	S288C_ctrl_hmg124h_1.JPG.dat	1478.0	24.6	1.1	0.02	#NUM!	1.5E-06
YAL058C-A	YAL058C-A	S288C_ctrl_hmg124h_1.JPG.dat	1273.0	28.6	1.0	0.00	#NUM!	2.7E-06
YAL058W	YAL058W	S288C_ctrl_hmg124h_1.JPG.dat	1170.3	22.4	0.9	0.02	#NUM!	2.0E-06
YAL059W	YAL059W	S288C_ctrl_hmg124h_1.JPG.dat	1234.5	39.3	1.0	0.03	#NUM!	6.5E-06
YAL060W	YAL060W	S288C_ctrl_hmg124h_1.JPG.dat	1193.0	37.9	1.0	0.03	#NUM!	7.2E-06
YAL061W	YAL061W	S288C_ctrl_hmg124h_1.JPG.dat	1222.5	28.2	1.0	0.03	#NUM!	4.6E-06
YAL062W	YAL062W	S288C_ctrl_hmg124h_1.JPG.dat	1280.0	9.4	1.0	0.01	#NUM!	6.0E-07
YAL064C-A	YAL064C-A	S288C_ctrl_hmg124h_1.JPG.dat	1343.5	51.8	1.0	0.04	#NUM!	1.5E-05
YAL065C	YAL065C	S288C_ctrl_hmg124h_1.JPG.dat	1392.3	21.9	1.1	0.01	#NUM!	1.3E-05
YAL066W	YAL066W	S288C_ctrl_hmg124h_1.JPG.dat	1227.8	45.2	1.0	0.04	#NUM!	2.1E-05
YAL067C	YAL067C	S288C_ctrl_hmg124h_1.JPG.dat	1405.8	81.7	1.0	0.06	#NUM!	5.5E-05
YAL068C	YAL068C	S288C_ctrl_hmg124h_1.JPG.dat	1412.5	48.6	1.0	0.03	#NUM!	6.6E-06
YAR002C-A	YAR002C-A	S288C_ctrl_hmg124h_1.JPG.dat	1396.5	23.7	1.0	0.00	#NUM!	5.5E-06
YAR002W	YAR002W	S288C_ctrl_hmg124h_1.JPG.dat	1103.3	35.9	0.9	0.03	#NUM!	8.2E-06
YAR003W	YAR003W	S288C_ctrl_hmg124h_1.JPG.dat	1179.3	17.4	0.9	0.00	#NUM!	2.0E-06
YAR014C	YAR014C	S288C_ctrl_hmg124h_1.JPG.dat	1242.5	63.7	1.0	0.05	#NUM!	3.5E-05
YAR015W	YAR015W	S288C_ctrl_hmg124h_1.JPG.dat	1249.5	15.8	1.0	0.00	#NUM!	1.8E-08
YAR018C	YAR018C	S288C_ctrl_hmg124h_1.JPG.dat	1325.0	25.0	1.0	0.02	#NUM!	1.8E-06
YAR020C	YAR020C	S288C_ctrl_hmg124h_1.JPG.dat	1397.5	15.9	1.0	0.01	#NUM!	2.8E-07
YAR023C	YAR023C	S288C_ctrl_hmg124h_1.JPG.dat	1306.8	48.9	1.0	0.03	#NUM!	1.2E-05
YAR027W	YAR027W	S288C_ctrl_hmg124h_1.JPG.dat	1244.3	65.9	1.0	0.04	#NUM!	1.9E-05
YAR028W	YAR028W	S288C_ctrl_hmg124h_1.JPG.dat	1387.3	32.5	1.0	0.01	#NUM!	8.4E-07
YAR029W	YAR029W	S288C_ctrl_hmg124h_1.JPG.dat	1475.5	36.6	1.0	0.02	#NUM!	1.2E-06
YAR030C	YAR030C	S288C_ctrl_hmg124h_1.JPG.dat	1410.3	19.4	1.0	0.02	#NUM!	1.5E-06
YAR031W	YAR031W	S288C_ctrl_hmg124h_1.JPG.dat	1309.8	32.9	1.0	0.03	#NUM!	5.4E-06
YAR035W	YAR035W	S288C_ctrl_hmg124h_1.JPG.dat	1246.5	27.1	1.0	0.02	#NUM!	1.7E-06
YAR037W	YAR037W	S288C_ctrl_hmg124h_1.JPG.dat	1268.5	44.4	1.0	0.03	#NUM!	1.0E-05
YAR040C	YAR040C	S288C_ctrl_hmg124h_1.JPG.dat	1349.3	20.7	1.0	0.00	#NUM!	5.1E-06
YAR042W	YAR042W	S288C_ctrl_hmg124h_1.JPG.dat	1357.8	39.9	1.0	0.01	#NUM!	2.5E-05
YAR043C	YAR043C	S288C_ctrl_hmg124h_1.JPG.dat	1406.0	38.8	1.0	0.03	#NUM!	1.0E-05

YAR044W	YAR044W	S288C_ctrl_hmg124h_1.JPG.dat	1351.3	64.6	1.0	0.04	#NUM!	1.9E-05
YAR047C	YAR047C	S288C_ctrl_hmg124h_1.JPG.dat	1271.3	22.0	1.0	0.03	#NUM!	4.7E-06
YAR050W	YAR050W	S288C_ctrl_hmg124h_1.JPG.dat	1474.5	50.5	1.0	0.03	#NUM!	5.6E-06
YBL001C	YBL001C	S288C_ctrl_hmg124h_1.JPG.dat	1525.5	53.7	1.0	0.01	#NUM!	2.8E-05
YBL003C	YBL003C	S288C_ctrl_hmg124h_1.JPG.dat	1490.3	51.0	1.0	0.03	#NUM!	1.0E-05
YBL005W	YBL005W	S288C_ctrl_hmg124h_1.JPG.dat	1447.8	44.3	1.0	0.03	#NUM!	7.7E-06
YBL007C	YBL007C	S288C_ctrl_hmg124h_1.JPG.dat	1435.5	78.1	1.0	0.02	#NUM!	1.1E-04
YBL008W	YBL008W	S288C_ctrl_hmg124h_1.JPG.dat	1446.8	53.5	1.0	0.04	#NUM!	1.3E-05
YBL009W	YBL009W	S288C_ctrl_hmg124h_1.JPG.dat	1501.0	62.5	1.1	0.04	#NUM!	2.0E-05
YBL010C	YBL010C	S288C_ctrl_hmg124h_1.JPG.dat	1485.5	39.9	1.0	0.03	#NUM!	5.1E-06
YBL011W	YBL011W	S288C_ctrl_hmg124h_1.JPG.dat	1451.3	35.8	1.0	0.02	#NUM!	2.6E-06
YBL013W	YBL013W	S288C_ctrl_hmg124h_1.JPG.dat	1394.0	57.3	1.0	0.04	#NUM!	1.6E-05
YBL015W	YBL015W	S288C_ctrl_hmg124h_1.JPG.dat	1284.8	45.4	0.9	0.03	#NUM!	8.8E-06
YBL016W	YBL016W	S288C_ctrl_hmg124h_1.JPG.dat	1280.0	62.5	1.0	0.04	#NUM!	2.7E-05
YBL017C	YBL017C	S288C_ctrl_hmg124h_1.JPG.dat	1502.3	11.2	1.1	0.02	#NUM!	9.9E-07
YBL019W	YBL019W	S288C_ctrl_hmg124h_1.JPG.dat	1396.8	24.0	1.0	0.00	#NUM!	2.5E-06
YBL021C	YBL021C	S288C_ctrl_hmg124h_1.JPG.dat	1347.5	28.9	1.0	0.03	#NUM!	4.5E-06
YBL024W	YBL024W	S288C_ctrl_hmg124h_1.JPG.dat	1395.3	17.9	1.0	0.01	#NUM!	6.3E-07
YBL025W	YBL025W	S288C_ctrl_hmg124h_1.JPG.dat	278.8	557.5	0.0	0.00	#NUM!	
YBL027W	YBL027W	S288C_ctrl_hmg124h_1.JPG.dat	1230.8	48.2	0.9	0.03	#NUM!	1.2E-05
YBL028C	YBL028C	S288C_ctrl_hmg124h_1.JPG.dat	1401.0	22.3	1.0	0.02	#NUM!	1.2E-06
YBL029W	YBL029W	S288C_ctrl_hmg124h_1.JPG.dat	1395.0	43.7	1.0	0.03	#NUM!	1.1E-05
YBL031W	YBL031W	S288C_ctrl_hmg124h_1.JPG.dat	1327.3	34.1	1.0	0.02	#NUM!	4.3E-06
YBL032W	YBL032W	S288C_ctrl_hmg124h_1.JPG.dat	1234.0	13.4	1.0	0.02	#NUM!	3.3E-06
YBL036C	YBL036C	S288C_ctrl_hmg124h_1.JPG.dat	1342.0	25.0	1.0	0.02	#NUM!	1.7E-06
YBL037W	YBL037W	S288C_ctrl_hmg124h_1.JPG.dat	1304.3	36.9	0.9	0.03	#NUM!	8.6E-06
YBL039C	YBL039C	S288C_ctrl_hmg124h_1.JPG.dat	1376.0	26.1	1.0	0.02	#NUM!	3.2E-06
YBL042C	YBL042C	S288C_ctrl_hmg124h_1.JPG.dat	1342.8	15.2	1.0	0.01	#NUM!	2.3E-07
YBL043W	YBL043W	S288C_ctrl_hmg124h_1.JPG.dat	1341.5	16.1	1.0	0.01	#NUM!	4.1E-07
YBL046W	YBL046W	S288C_ctrl_hmg124h_1.JPG.dat	1308.8	31.2	1.0	0.00	#NUM!	7.9E-06
YBL047C	YBL047C	S288C_ctrl_hmg124h_1.JPG.dat	1324.8	18.4	1.0	0.02	#NUM!	8.6E-07
YBL048W	YBL048W	S288C_ctrl_hmg124h_1.JPG.dat	1345.3	33.3	1.0	0.03	#NUM!	4.9E-06
YBL049W	YBL049W	S288C_ctrl_hmg124h_1.JPG.dat	1407.0	26.6	1.0	0.02	#NUM!	1.2E-06
YBL051C	YBL051C	S288C_ctrl_hmg124h_1.JPG.dat	1312.5	22.6	1.0	0.02	#NUM!	2.2E-06
YBL052C	YBL052C	S288C_ctrl_hmg124h_1.JPG.dat	1326.3	30.9	1.0	0.02	#NUM!	3.4E-06
YBL053W	YBL053W	S288C_ctrl_hmg124h_1.JPG.dat	1276.8	102.6	1.0	0.08	#NUM!	1.3E-04
YBL054W	YBL054W	S288C_ctrl_hmg124h_1.JPG.dat	1306.8	36.4	1.0	0.02	#NUM!	4.1E-06
YBL055C	YBL055C	S288C_ctrl_hmg124h_1.JPG.dat	1363.5	18.4	1.0	0.02	#NUM!	1.4E-06
YBL056W	YBL056W	S288C_ctrl_hmg124h_1.JPG.dat	1348.5	33.5	1.0	0.02	#NUM!	2.7E-06
YBL057C	YBL057C	S288C_ctrl_hmg124h_1.JPG.dat	1322.0	27.7	1.0	0.02	#NUM!	1.4E-06
YBL059W	YBL059W	S288C_ctrl_hmg124h_1.JPG.dat	1264.8	86.0	1.0	0.07	#NUM!	1.1E-04
YBL060W	YBL060W	S288C_ctrl_hmg124h_1.JPG.dat	1233.8	22.2	1.0	0.01	#NUM!	8.9E-06
YBL061C	YBL061C	S288C_ctrl_hmg124h_1.JPG.dat	1273.0	15.9	1.0	0.01	#NUM!	7.4E-08
YBL062W	YBL062W	S288C_ctrl_hmg124h_1.JPG.dat	1351.8	29.9	1.0	0.03	#NUM!	4.4E-06
YBL063W	YBL063W	S288C_ctrl_hmg124h_1.JPG.dat	1432.0	35.4	1.1	0.03	#NUM!	6.3E-06
YBL064C	YBL064C	S288C_ctrl_hmg124h_1.JPG.dat	1075.5	41.9	0.9	0.01	#NUM!	1.5E-05
YBL065W	YBL065W	S288C_ctrl_hmg124h_1.JPG.dat	1277.0	23.2	1.0	0.02	#NUM!	1.2E-06
YBL066C	YBL066C	S288C_ctrl_hmg124h_1.JPG.dat	1408.5	46.0	1.0	0.03	#NUM!	7.5E-06
YBL067C	YBL067C	S288C_ctrl_hmg124h_1.JPG.dat	1350.8	41.6	1.0	0.02	#NUM!	4.0E-06
YBL068W	YBL068W	S288C_ctrl_hmg124h_1.JPG.dat	1270.8	41.9	1.0	0.01	#NUM!	4.0E-05
YBL069W	YBL069W	S288C_ctrl_hmg124h_1.JPG.dat	1272.5	35.7	1.0	0.01	#NUM!	2.3E-05
YBL070C	YBL070C	S288C_ctrl_hmg124h_1.JPG.dat	1249.5	18.9	1.0	0.00	#NUM!	6.2E-07
YBL071C	YBL071C	S288C_ctrl_hmg124h_1.JPG.dat	1193.3	23.2	1.0	0.00	#NUM!	1.2E-06
YBL072C	YBL072C	S288C_ctrl_hmg124h_1.JPG.dat	1217.5	52.9	1.0	0.04	#NUM!	2.0E-05
YBL075C	YBL075C	S288C_ctrl_hmg124h_1.JPG.dat	1309.8	31.5	1.0	0.02	#NUM!	3.9E-06
YBL078C	YBL078C	S288C_ctrl_hmg124h_1.JPG.dat	1374.0	20.8	1.0	0.02	#NUM!	1.7E-06
YBL079W	YBL079W	S288C_ctrl_hmg124h_1.JPG.dat	1275.0	79.8	1.0	0.07	#NUM!	9.3E-05
YBL081W	YBL081W	S288C_ctrl_hmg124h_1.JPG.dat	1301.5	26.9	1.0	0.02	#NUM!	2.4E-06
YBL082C	YBL082C	S288C_ctrl_hmg124h_1.JPG.dat	1479.0	42.6	1.1	0.03	#NUM!	6.4E-06
YBL083C	YBL083C	S288C_ctrl_hmg124h_1.JPG.dat	1414.5	15.3	1.0	0.02	#NUM!	2.0E-06
YBL085W	YBL085W	S288C_ctrl_hmg124h_1.JPG.dat	1291.8	32.0	1.0	0.03	#NUM!	8.3E-06
YBL086C	YBL086C	S288C_ctrl_hmg124h_1.JPG.dat	1209.8	21.7	1.0	0.02	#NUM!	1.2E-06
YBL087C	YBL087C	S288C_ctrl_hmg124h_1.JPG.dat	1025.0	53.4	0.8	0.04	#NUM!	3.9E-05
YBL088C	YBL088C	S288C_ctrl_hmg124h_1.JPG.dat	1243.0	27.4	1.0	0.02	#NUM!	2.1E-06
YBL089W	YBL089W	S288C_ctrl_hmg124h_1.JPG.dat	1271.5	25.5	1.0	0.02	#NUM!	2.0E-06
YBL091C	YBL091C	S288C_ctrl_hmg124h_1.JPG.dat	1381.0	19.7	1.0	0.02	#NUM!	1.6E-06
YBL091C-A	YBL091C-A	S288C_ctrl_hmg124h_1.JPG.dat	1480.5	15.8	1.1	0.01	#NUM!	2.2E-07
YBL094C	YBL094C	S288C_ctrl_hmg124h_1.JPG.dat	1299.0	50.4	1.0	0.05	#NUM!	3.1E-05
YBL095W	YBL095W	S288C_ctrl_hmg124h_1.JPG.dat	1416.8	42.1	1.0	0.04	#NUM!	1.2E-05
YBL096C	YBL096C	S288C_ctrl_hmg124h_1.JPG.dat	1502.5	30.1	1.0	0.03	#NUM!	6.3E-06

YBL098W	YBL098W	S288C_ctrl_hmg124h_1.JPG.dat	1410.3	31.3	1.0	0.02	#NUM!	9.8E-07
YBL100C	YBL100C	S288C_ctrl_hmg124h_1.JPG.dat	1003.3	669.4	1.0	0.01	#NUM!	6.1E-05
YBL101C	YBL101C	S288C_ctrl_hmg124h_1.JPG.dat	1302.8	6.6	1.0	0.02	#NUM!	1.6E-06
YBL102W	YBL102W	S288C_ctrl_hmg124h_1.JPG.dat	1291.3	38.7	1.0	0.02	#NUM!	1.5E-06
YBL103C	YBL103C	S288C_ctrl_hmg124h_1.JPG.dat	1274.0	46.5	1.0	0.03	#NUM!	6.3E-06
YBL104C	YBL104C	S288C_ctrl_hmg124h_1.JPG.dat	1341.3	71.5	1.0	0.04	#NUM!	2.1E-05
YBL106C	YBL106C	S288C_ctrl_hmg124h_1.JPG.dat	1400.8	44.3	1.0	0.01	#NUM!	4.6E-05
YBL107C	YBL107C	S288C_ctrl_hmg124h_1.JPG.dat	1445.8	41.5	1.0	0.03	#NUM!	9.6E-06
YBR001C	YBR001C	S288C_ctrl_hmg124h_1.JPG.dat	1345.3	43.6	1.0	0.03	#NUM!	7.6E-06
YBR005W	YBR005W	S288C_ctrl_hmg124h_1.JPG.dat	1467.8	50.3	1.0	0.04	#NUM!	1.1E-05
YBR006W	YBR006W	S288C_ctrl_hmg124h_1.JPG.dat	1464.8	53.7	1.0	0.03	#NUM!	1.0E-05
YBR007C	YBR007C	S288C_ctrl_hmg124h_1.JPG.dat	1477.8	132.0	1.0	0.09	#NUM!	1.9E-04
YBR008C	YBR008C	S288C_ctrl_hmg124h_1.JPG.dat	1498.0	59.0	1.0	0.04	#NUM!	1.7E-05
YBR009C	YBR009C	S288C_ctrl_hmg124h_1.JPG.dat	1467.8	59.5	1.0	0.02	#NUM!	8.8E-05
YBR010W	YBR010W	S288C_ctrl_hmg124h_1.JPG.dat	1462.5	46.8	1.0	0.03	#NUM!	8.3E-06
YBR012C	YBR012C	S288C_ctrl_hmg124h_1.JPG.dat	1483.5	91.1	1.1	0.06	#NUM!	6.1E-05
YBR013C	YBR013C	S288C_ctrl_hmg124h_1.JPG.dat	1504.5	75.1	1.0	0.05	#NUM!	3.6E-05
YBR014C	YBR014C	S288C_ctrl_hmg124h_1.JPG.dat	1455.0	27.0	1.0	0.02	#NUM!	1.4E-06
YBR015C	YBR015C	S288C_ctrl_hmg124h_1.JPG.dat	1411.8	60.3	1.0	0.04	#NUM!	2.1E-05
YBR016W	YBR016W	S288C_ctrl_hmg124h_1.JPG.dat	1326.0	36.6	0.9	0.03	#NUM!	8.1E-06
YBR018C	YBR018C	S288C_ctrl_hmg124h_1.JPG.dat	1447.3	26.6	1.0	0.01	#NUM!	3.1E-07
YBR019C	YBR019C	S288C_ctrl_hmg124h_1.JPG.dat	1509.0	49.1	1.0	0.03	#NUM!	8.3E-06
YBR020W	YBR020W	S288C_ctrl_hmg124h_1.JPG.dat	1448.0	32.8	1.0	0.03	#NUM!	7.3E-06
YBR022W	YBR022W	S288C_ctrl_hmg124h_1.JPG.dat	1469.0	40.0	1.0	0.01	#NUM!	1.8E-07
YBR023C	YBR023C	S288C_ctrl_hmg124h_1.JPG.dat	1474.3	41.3	1.0	0.05	#NUM!	3.3E-05
YBR024W	YBR024W	S288C_ctrl_hmg124h_1.JPG.dat	1409.5	44.4	1.0	0.02	#NUM!	1.6E-06
YBR025C	YBR025C	S288C_ctrl_hmg124h_1.JPG.dat	1390.3	41.3	1.0	0.04	#NUM!	1.4E-05
YBR026C	YBR026C	S288C_ctrl_hmg124h_1.JPG.dat	1340.3	264.1	1.0	0.18	#NUM!	1.8E-03
YBR027C	YBR027C	S288C_ctrl_hmg124h_1.JPG.dat	1501.3	90.0	1.1	0.05	#NUM!	2.5E-05
YBR028C	YBR028C	S288C_ctrl_hmg124h_1.JPG.dat	1355.8	36.8	1.0	0.01	#NUM!	2.3E-05
YBR030W	YBR030W	S288C_ctrl_hmg124h_1.JPG.dat	1217.8	25.9	0.9	0.02	#NUM!	1.8E-06
YBR031W	YBR031W	S288C_ctrl_hmg124h_1.JPG.dat	1255.3	9.5	0.9	0.00	#NUM!	2.3E-06
YBR032W	YBR032W	S288C_ctrl_hmg124h_1.JPG.dat	1364.3	69.0	1.0	0.04	#NUM!	2.4E-05
YBR033W	YBR033W	S288C_ctrl_hmg124h_1.JPG.dat	1441.3	27.2	1.1	0.02	#NUM!	1.1E-06
YBR034C	YBR034C	S288C_ctrl_hmg124h_1.JPG.dat	1370.8	41.2	1.0	0.03	#NUM!	8.3E-06
YBR036C	YBR036C	S288C_ctrl_hmg124h_1.JPG.dat	878.3	81.2	0.6	0.02	#NUM!	3.4E-04
YBR040W	YBR040W	S288C_ctrl_hmg124h_1.JPG.dat	1307.5	21.0	1.0	0.01	#NUM!	5.0E-07
YBR041W	YBR041W	S288C_ctrl_hmg124h_1.JPG.dat	1331.8	60.5	1.0	0.04	#NUM!	1.3E-05
YBR042C	YBR042C	S288C_ctrl_hmg124h_1.JPG.dat	1356.8	54.5	1.0	0.03	#NUM!	1.1E-05
YBR043C	YBR043C	S288C_ctrl_hmg124h_1.JPG.dat	1404.3	39.4	1.0	0.03	#NUM!	4.4E-06
YBR044C	YBR044C	S288C_ctrl_hmg124h_1.JPG.dat	1278.5	37.9	1.0	0.03	#NUM!	1.0E-05
YBR045C	YBR045C	S288C_ctrl_hmg124h_1.JPG.dat	1183.3	39.9	1.0	0.01	#NUM!	1.5E-05
YBR046C	YBR046C	S288C_ctrl_hmg124h_1.JPG.dat	1252.0	7.0	0.9	0.01	#NUM!	2.5E-07
YBR047W	YBR047W	S288C_ctrl_hmg124h_1.JPG.dat	1465.5	13.5	1.0	0.01	#NUM!	3.5E-07
YBR048W	YBR048W	S288C_ctrl_hmg124h_1.JPG.dat	1053.8	16.8	0.8	0.00	#NUM!	1.3E-05
YBR050C	YBR050C	S288C_ctrl_hmg124h_1.JPG.dat	1391.8	13.9	1.1	0.01	#NUM!	3.5E-07
YBR051W	YBR051W	S288C_ctrl_hmg124h_1.JPG.dat	1229.3	32.4	1.0	0.02	#NUM!	2.9E-06
YBR052C	YBR052C	S288C_ctrl_hmg124h_1.JPG.dat	1251.3	19.9	1.0	0.02	#NUM!	1.3E-06
YBR053C	YBR053C	S288C_ctrl_hmg124h_1.JPG.dat	1240.5	41.6	1.0	0.03	#NUM!	6.6E-06
YBR054W	YBR054W	S288C_ctrl_hmg124h_1.JPG.dat	1325.0	8.1	1.0	0.00	#NUM!	2.5E-08
YBR056W	YBR056W	S288C_ctrl_hmg124h_1.JPG.dat	1417.8	27.6	1.0	0.02	#NUM!	1.8E-06
YBR057C	YBR057C	S288C_ctrl_hmg124h_1.JPG.dat	1371.3	29.0	1.0	0.01	#NUM!	6.7E-07
YBR058C	YBR058C	S288C_ctrl_hmg124h_1.JPG.dat	1242.3	24.0	1.0	0.03	#NUM!	8.6E-06
YBR059C	YBR059C	S288C_ctrl_hmg124h_1.JPG.dat	1203.5	23.4	0.9	0.02	#NUM!	1.5E-06
YBR061C	YBR061C	S288C_ctrl_hmg124h_1.JPG.dat	1350.5	28.3	1.0	0.02	#NUM!	2.7E-06
YBR062C	YBR062C	S288C_ctrl_hmg124h_1.JPG.dat	1379.3	32.6	1.0	0.02	#NUM!	3.8E-06
YBR063C	YBR063C	S288C_ctrl_hmg124h_1.JPG.dat	1313.8	47.6	1.0	0.04	#NUM!	1.7E-05
YBR064W	YBR064W	S288C_ctrl_hmg124h_1.JPG.dat	1258.0	73.6	1.0	0.05	#NUM!	3.8E-05
YBR065C	YBR065C	S288C_ctrl_hmg124h_1.JPG.dat	1251.8	57.2	1.0	0.05	#NUM!	3.0E-05
YBR066C	YBR066C	S288C_ctrl_hmg124h_1.JPG.dat	1241.3	31.1	1.0	0.02	#NUM!	1.5E-06
YBR067C	YBR067C	S288C_ctrl_hmg124h_1.JPG.dat	1326.8	30.2	1.0	0.01	#NUM!	1.8E-05
YBR068C	YBR068C	S288C_ctrl_hmg124h_1.JPG.dat	1364.3	49.8	1.0	0.03	#NUM!	9.2E-06
YBR069C	YBR069C	S288C_ctrl_hmg124h_1.JPG.dat	1401.8	29.2	1.1	0.00	#NUM!	2.6E-06
YBR071W	YBR071W	S288C_ctrl_hmg124h_1.JPG.dat	1244.5	24.2	1.0	0.03	#NUM!	1.1E-05
YBR072W	YBR072W	S288C_ctrl_hmg124h_1.JPG.dat	1368.8	35.5	1.0	0.03	#NUM!	4.6E-06
YBR073W	YBR073W	S288C_ctrl_hmg124h_1.JPG.dat	1416.0	34.0	1.0	0.02	#NUM!	2.2E-06
YBR074W	YBR074W	S288C_ctrl_hmg124h_1.JPG.dat	1325.5	39.4	1.0	0.02	#NUM!	3.4E-06
YBR075W	YBR075W	S288C_ctrl_hmg124h_1.JPG.dat	1271.5	61.2	1.0	0.05	#NUM!	3.1E-05
YBR076W	YBR076W	S288C_ctrl_hmg124h_1.JPG.dat	1182.3	45.0	1.0	0.04	#NUM!	1.6E-05
YBR077C	YBR077C	S288C_ctrl_hmg124h_1.JPG.dat	1406.3	46.2	1.2	0.04	#NUM!	8.0E-06

YBR082C	YBR082C	S288C_ctrl_hmg124h_1.JPG.dat	1223.5	28.9	1.0	0.03	#NUM!	6.6E-06
YBR083W	YBR083W	S288C_ctrl_hmg124h_1.JPG.dat	1318.8	25.8	1.0	0.02	#NUM!	2.6E-06
YBR084C-A	YBR084C-A	S288C_ctrl_hmg124h_1.JPG.dat	1224.5	31.6	0.9	0.01	#NUM!	2.2E-05
YBR084W	YBR084W	S288C_ctrl_hmg124h_1.JPG.dat	1422.8	33.0	1.1	0.02	#NUM!	3.2E-06
YBR090C	YBR090C	S288C_ctrl_hmg124h_1.JPG.dat	1300.0	100.4	1.0	0.07	#NUM!	7.9E-05
YBR090C-A	YBR090C-A	S288C_ctrl_hmg124h_1.JPG.dat	1387.3	91.6	1.0	0.02	#NUM!	1.5E-04
YBR092C	YBR092C	S288C_ctrl_hmg124h_1.JPG.dat	1466.0	19.6	1.0	0.00	#NUM!	3.3E-06
YBR093C	YBR093C	S288C_ctrl_hmg124h_1.JPG.dat	1150.3	35.6	0.9	0.03	#NUM!	8.5E-06
YBR094W	YBR094W	S288C_ctrl_hmg124h_1.JPG.dat	1213.5	64.9	1.0	0.05	#NUM!	4.5E-05
YBR095C	YBR095C	S288C_ctrl_hmg124h_1.JPG.dat	1171.0	29.5	0.9	0.02	#NUM!	4.0E-06
YBR098W	YBR098W	S288C_ctrl_hmg124h_1.JPG.dat	1199.5	30.8	1.0	0.03	#NUM!	6.0E-06
YBR099C	YBR099C	S288C_ctrl_hmg124h_1.JPG.dat	1228.0	23.0	1.0	0.02	#NUM!	2.0E-06
YBR100W	YBR100W	S288C_ctrl_hmg124h_1.JPG.dat	1321.5	22.8	1.0	0.01	#NUM!	4.5E-07
YBR101C	YBR101C	S288C_ctrl_hmg124h_1.JPG.dat	1415.5	32.0	1.0	0.02	#NUM!	9.0E-07
YBR103W	YBR103W	S288C_ctrl_hmg124h_1.JPG.dat	1254.0	47.9	0.9	0.03	#NUM!	1.2E-05
YBR104W	YBR104W	S288C_ctrl_hmg124h_1.JPG.dat	1376.8	20.8	1.1	0.02	#NUM!	1.5E-06
YBR105C	YBR105C	S288C_ctrl_hmg124h_1.JPG.dat	1390.8	75.1	1.0	0.06	#NUM!	6.1E-05
YBR106W	YBR106W	S288C_ctrl_hmg124h_1.JPG.dat	0.3	0.5	0.0	0.00	#NUM!	
YBR107C	YBR107C	S288C_ctrl_hmg124h_1.JPG.dat	1488.5	31.6	1.0	0.01	#NUM!	1.3E-07
YBR108W	YBR108W	S288C_ctrl_hmg124h_1.JPG.dat	1364.5	50.8	1.0	0.01	#NUM!	3.7E-05
YBR111C	YBR111C	S288C_ctrl_hmg124h_1.JPG.dat	1324.8	31.6	1.0	0.02	#NUM!	2.8E-06
YBR113W	YBR113W	S288C_ctrl_hmg124h_1.JPG.dat	1331.8	58.9	1.0	0.03	#NUM!	1.1E-05
YBR114W	YBR114W	S288C_ctrl_hmg124h_1.JPG.dat	1404.8	21.3	1.0	0.01	#NUM!	6.8E-07
YBR115C	YBR115C	S288C_ctrl_hmg124h_1.JPG.dat	677.0	781.8	0.5	0.56	#NUM!	1.8E-01
YBR116C	YBR116C	S288C_ctrl_hmg124h_1.JPG.dat	1452.5	66.4	1.0	0.04	#NUM!	1.6E-05
YBR119W	YBR119W	S288C_ctrl_hmg124h_1.JPG.dat	1331.0	32.8	1.0	0.03	#NUM!	1.1E-05
YBR121C	YBR121C	S288C_ctrl_hmg124h_1.JPG.dat	656.0	759.5	0.5	0.59	#NUM!	1.8E-01
YBR122C	YBR122C	S288C_ctrl_hmg124h_1.JPG.dat	388.5	777.0	0.0	0.00	#NUM!	
YBR125C	YBR125C	S288C_ctrl_hmg124h_1.JPG.dat	1467.5	98.7	1.0	0.05	#NUM!	3.1E-05
YBR126C	YBR126C	S288C_ctrl_hmg124h_1.JPG.dat	1484.3	53.6	1.0	0.02	#NUM!	3.7E-06
YBR128C	YBR128C	S288C_ctrl_hmg124h_1.JPG.dat	1429.0	21.3	1.0	0.00	#NUM!	1.5E-06
YBR129C	YBR129C	S288C_ctrl_hmg124h_1.JPG.dat	1437.8	48.7	1.0	0.03	#NUM!	5.1E-06
YBR130C	YBR130C	S288C_ctrl_hmg124h_1.JPG.dat	1408.0	37.3	1.0	0.02	#NUM!	1.1E-06
YBR131W	YBR131W	S288C_ctrl_hmg124h_1.JPG.dat	993.5	673.8	0.9	0.09	#NUM!	3.3E-03
YBR132C	YBR132C	S288C_ctrl_hmg124h_1.JPG.dat	1480.3	32.9	1.1	0.00	#NUM!	4.7E-07
YBR134W	YBR134W	S288C_ctrl_hmg124h_1.JPG.dat	1474.8	63.8	1.0	0.03	#NUM!	8.2E-06
YBR137W	YBR137W	S288C_ctrl_hmg124h_1.JPG.dat	1388.8	37.6	1.0	0.02	#NUM!	2.3E-06
YBR138C	YBR138C	S288C_ctrl_hmg124h_1.JPG.dat	1300.5	40.4	1.0	0.03	#NUM!	1.2E-05
YBR139W	YBR139W	S288C_ctrl_hmg124h_1.JPG.dat	1366.3	25.3	1.0	0.02	#NUM!	1.3E-06
YBR141C	YBR141C	S288C_ctrl_hmg124h_1.JPG.dat	1433.5	86.9	1.0	0.02	#NUM!	1.3E-04
YBR144C	YBR144C	S288C_ctrl_hmg124h_1.JPG.dat	1325.8	30.5	1.0	0.02	#NUM!	4.0E-06
YBR145W	YBR145W	S288C_ctrl_hmg124h_1.JPG.dat	1357.5	52.2	1.0	0.01	#NUM!	5.6E-05
YBR146W	YBR146W	S288C_ctrl_hmg124h_1.JPG.dat	1283.5	17.7	1.0	0.01	#NUM!	9.9E-08
YBR147W	YBR147W	S288C_ctrl_hmg124h_1.JPG.dat	1230.0	112.8	0.9	0.08	#NUM!	1.6E-04
YBR148W	YBR148W	S288C_ctrl_hmg124h_1.JPG.dat	1302.3	1.5	1.0	0.01	#NUM!	5.1E-07
YBR149W	YBR149W	S288C_ctrl_hmg124h_1.JPG.dat	1305.8	36.8	1.0	0.02	#NUM!	1.5E-06
YBR150C	YBR150C	S288C_ctrl_hmg124h_1.JPG.dat	1405.5	18.0	1.0	0.02	#NUM!	2.1E-06
YBR151W	YBR151W	S288C_ctrl_hmg124h_1.JPG.dat	1394.8	25.0	1.0	0.02	#NUM!	2.3E-06
YBR156C	YBR156C	S288C_ctrl_hmg124h_1.JPG.dat	1276.3	17.1	1.0	0.01	#NUM!	5.1E-07
YBR157C	YBR157C	S288C_ctrl_hmg124h_1.JPG.dat	1295.3	39.0	1.0	0.03	#NUM!	9.3E-06
YBR158W	YBR158W	S288C_ctrl_hmg124h_1.JPG.dat	1313.8	49.0	1.0	0.03	#NUM!	9.6E-06
YBR159W	YBR159W	S288C_ctrl_hmg124h_1.JPG.dat	1405.3	35.6	1.0	0.01	#NUM!	1.7E-05
YBR161W	YBR161W	S288C_ctrl_hmg124h_1.JPG.dat	1432.8	14.2	1.1	0.01	#NUM!	2.2E-07
YBR162C	YBR162C	S288C_ctrl_hmg124h_1.JPG.dat	1371.3	21.8	1.0	0.02	#NUM!	9.5E-07
YBR162W-	YBR162W-A	S288C_ctrl_hmg124h_1.JPG.dat	1271.0	51.3	1.0	0.04	#NUM!	1.6E-05
YBR164C	YBR164C	S288C_ctrl_hmg124h_1.JPG.dat	1216.8	61.9	1.0	0.05	#NUM!	3.0E-05
YBR165W	YBR165W	S288C_ctrl_hmg124h_1.JPG.dat	1258.5	24.9	1.0	0.01	#NUM!	8.5E-07
YBR166C	YBR166C	S288C_ctrl_hmg124h_1.JPG.dat	1331.0	18.2	1.0	0.00	#NUM!	1.6E-06
YBR168W	YBR168W	S288C_ctrl_hmg124h_1.JPG.dat	1127.5	59.7	0.8	0.04	#NUM!	3.6E-05
YBR169C	YBR169C	S288C_ctrl_hmg124h_1.JPG.dat	1339.3	56.4	1.0	0.05	#NUM!	3.0E-05
YBR170C	YBR170C	S288C_ctrl_hmg124h_1.JPG.dat	1318.8	48.8	1.0	0.04	#NUM!	1.6E-05
YBR171W	YBR171W	S288C_ctrl_hmg124h_1.JPG.dat	1575.0	68.2	1.1	0.06	#NUM!	4.3E-05
YBR172C	YBR172C	S288C_ctrl_hmg124h_1.JPG.dat	1333.0	13.1	1.0	0.01	#NUM!	8.3E-08
YBR174C	YBR174C	S288C_ctrl_hmg124h_1.JPG.dat	1207.5	21.4	0.9	0.00	#NUM!	7.2E-06
YBR175W	YBR175W	S288C_ctrl_hmg124h_1.JPG.dat	0.0	0.0	0.0	0.00	#NUM!	
YBR176W	YBR176W	S288C_ctrl_hmg124h_1.JPG.dat	1303.5	35.3	1.0	0.03	#NUM!	9.6E-06
YBR177C	YBR177C	S288C_ctrl_hmg124h_1.JPG.dat	1201.3	24.0	1.0	0.00	#NUM!	2.4E-07
YBR178W	YBR178W	S288C_ctrl_hmg124h_1.JPG.dat	1271.5	39.7	1.0	0.02	#NUM!	3.6E-06
YBR180W	YBR180W	S288C_ctrl_hmg124h_1.JPG.dat	1350.0	19.6	1.0	0.02	#NUM!	2.9E-06
YBR181C	YBR181C	S288C_ctrl_hmg124h_1.JPG.dat	1071.0	45.4	0.8	0.03	#NUM!	1.9E-05

YBR182C	YBR182C	S288C_ctrl_hmg124h_1.JPG.dat	1376.5	53.4	1.1	0.04	#NUM!	1.6E-05
YBR183W	YBR183W	S288C_ctrl_hmg124h_1.JPG.dat	1282.8	19.3	1.0	0.02	#NUM!	1.1E-06
YBR184W	YBR184W	S288C_ctrl_hmg124h_1.JPG.dat	1383.8	43.4	1.0	0.03	#NUM!	1.1E-05
YBR185C	YBR185C	S288C_ctrl_hmg124h_1.JPG.dat	1375.8	34.7	1.0	0.00	#NUM!	5.5E-06
YBR186W	YBR186W	S288C_ctrl_hmg124h_1.JPG.dat	1266.5	19.4	1.0	0.01	#NUM!	6.5E-07
YBR187W	YBR187W	S288C_ctrl_hmg124h_1.JPG.dat	1248.5	22.2	1.0	0.02	#NUM!	1.0E-06
YBR188C	YBR188C	S288C_ctrl_hmg124h_1.JPG.dat	1266.5	37.2	1.0	0.03	#NUM!	8.8E-06
YBR189W	YBR189W	S288C_ctrl_hmg124h_1.JPG.dat	932.0	301.0	0.8	0.25	#NUM!	8.4E-03
YBR194W	YBR194W	S288C_ctrl_hmg124h_1.JPG.dat	929.8	76.0	0.7	0.06	#NUM!	1.8E-04
YBR195C	YBR195C	S288C_ctrl_hmg124h_1.JPG.dat	1317.3	24.1	1.0	0.02	#NUM!	2.1E-06
YBR197C	YBR197C	S288C_ctrl_hmg124h_1.JPG.dat	1387.5	37.8	1.0	0.00	#NUM!	9.4E-07
YBR199W	YBR199W	S288C_ctrl_hmg124h_1.JPG.dat	1318.5	40.4	1.0	0.03	#NUM!	5.8E-06
YBR200W	YBR200W	S288C_ctrl_hmg124h_1.JPG.dat	959.3	640.4	1.0	0.03	#NUM!	2.4E-04
YBR201W	YBR201W	S288C_ctrl_hmg124h_1.JPG.dat	1455.3	54.8	1.0	0.03	#NUM!	7.0E-06
YBR203W	YBR203W	S288C_ctrl_hmg124h_1.JPG.dat	1372.0	27.4	1.0	0.01	#NUM!	1.7E-05
YBR204C	YBR204C	S288C_ctrl_hmg124h_1.JPG.dat	1298.5	41.2	1.0	0.02	#NUM!	2.5E-06
YBR205W	YBR205W	S288C_ctrl_hmg124h_1.JPG.dat	1257.8	45.3	1.0	0.00	#NUM!	4.1E-07
YBR206W	YBR206W	S288C_ctrl_hmg124h_1.JPG.dat	1244.3	16.2	1.0	0.00	#NUM!	6.4E-06
YBR207W	YBR207W	S288C_ctrl_hmg124h_1.JPG.dat	1232.0	24.2	1.0	0.02	#NUM!	2.5E-06
YBR208C	YBR208C	S288C_ctrl_hmg124h_1.JPG.dat	1341.5	37.9	1.0	0.02	#NUM!	3.3E-06
YBR209W	YBR209W	S288C_ctrl_hmg124h_1.JPG.dat	1360.3	10.2	1.0	0.01	#NUM!	3.8E-08
YBR210W	YBR210W	S288C_ctrl_hmg124h_1.JPG.dat	1345.3	85.0	1.0	0.06	#NUM!	7.1E-05
YBR212W	YBR212W	S288C_ctrl_hmg124h_1.JPG.dat	1364.3	54.9	1.1	0.05	#NUM!	2.4E-05
YBR213W	YBR213W	S288C_ctrl_hmg124h_1.JPG.dat	1403.0	17.5	1.0	0.02	#NUM!	3.1E-06
YBR214W	YBR214W	S288C_ctrl_hmg124h_1.JPG.dat	1523.3	58.1	1.0	0.03	#NUM!	4.2E-06
YBR215W	YBR215W	S288C_ctrl_hmg124h_1.JPG.dat	1390.0	38.8	1.0	0.01	#NUM!	8.7E-07
YBR216C	YBR216C	S288C_ctrl_hmg124h_1.JPG.dat	1433.3	57.2	1.0	0.03	#NUM!	8.6E-06
YBR217W	YBR217W	S288C_ctrl_hmg124h_1.JPG.dat	1414.3	50.1	1.0	0.02	#NUM!	2.5E-06
YBR218C	YBR218C	S288C_ctrl_hmg124h_1.JPG.dat	1301.0	54.1	1.0	0.03	#NUM!	7.6E-06
YBR219C	YBR219C	S288C_ctrl_hmg124h_1.JPG.dat	1295.0	90.0	1.0	0.01	#NUM!	5.1E-05
YBR220C	YBR220C	S288C_ctrl_hmg124h_1.JPG.dat	1393.8	30.6	1.0	0.02	#NUM!	2.0E-06
YBR221C	YBR221C	S288C_ctrl_hmg124h_1.JPG.dat	1336.0	17.5	1.0	0.01	#NUM!	9.2E-08
YBR222C	YBR222C	S288C_ctrl_hmg124h_1.JPG.dat	1379.3	41.0	1.0	0.02	#NUM!	4.3E-06
YBR223C	YBR223C	S288C_ctrl_hmg124h_1.JPG.dat	1416.0	42.3	1.1	0.03	#NUM!	5.8E-06
YOR202W	YOR202W	S288C_ctrl_hmg124h_1.JPG.dat	1448.9	164.8	1.0	0.06	#NUM!	0.0E+00
1	1	S288C_ctrl_hmg124h_10.JPG.dat	1358.5	96.1	1.0	0.02	#NUM!	1.7E-04
2	2	S288C_ctrl_hmg124h_10.JPG.dat	1264.5	71.1	1.0	0.05	#NUM!	3.6E-05
3	3	S288C_ctrl_hmg124h_10.JPG.dat	1181.5	67.9	1.0	0.05	#NUM!	3.0E-05
4	4	S288C_ctrl_hmg124h_10.JPG.dat	1192.3	64.5	1.0	0.05	#NUM!	3.1E-05
YLR415C	YLR415C	S288C_ctrl_hmg124h_10.JPG.dat	1302.8	45.0	1.0	0.04	#NUM!	1.5E-05
YLR416C	YLR416C	S288C_ctrl_hmg124h_10.JPG.dat	1270.5	31.8	1.0	0.03	#NUM!	5.3E-06
YLR418C	YLR418C	S288C_ctrl_hmg124h_10.JPG.dat	920.3	30.1	0.7	0.02	#NUM!	9.4E-06
YLR420W	YLR420W	S288C_ctrl_hmg124h_10.JPG.dat	1245.8	30.4	1.0	0.02	#NUM!	3.2E-06
YLR421C	YLR421C	S288C_ctrl_hmg124h_10.JPG.dat	1195.5	52.4	1.0	0.00	#NUM!	4.8E-06
YLR422W	YLR422W	S288C_ctrl_hmg124h_10.JPG.dat	1270.3	25.4	1.1	0.02	#NUM!	2.3E-06
YLR423C	YLR423C	S288C_ctrl_hmg124h_10.JPG.dat	1210.8	60.4	1.0	0.05	#NUM!	3.0E-05
YLR425W	YLR425W	S288C_ctrl_hmg124h_10.JPG.dat	1220.5	41.9	1.0	0.03	#NUM!	1.0E-05
YLR426W	YLR426W	S288C_ctrl_hmg124h_10.JPG.dat	1203.0	34.2	1.0	0.03	#NUM!	5.5E-06
YLR427W	YLR427W	S288C_ctrl_hmg124h_10.JPG.dat	1238.5	22.2	1.0	0.01	#NUM!	8.5E-07
YLR428C	YLR428C	S288C_ctrl_hmg124h_10.JPG.dat	1241.5	29.6	1.0	0.04	#NUM!	1.2E-05
YLR429W	YLR429W	S288C_ctrl_hmg124h_10.JPG.dat	1279.3	25.5	1.0	0.02	#NUM!	1.6E-06
YLR431C	YLR431C	S288C_ctrl_hmg124h_10.JPG.dat	1306.3	21.6	1.0	0.02	#NUM!	1.9E-06
YLR432W	YLR432W	S288C_ctrl_hmg124h_10.JPG.dat	1291.0	6.5	1.0	0.00	#NUM!	1.9E-08
YLR433C	YLR433C	S288C_ctrl_hmg124h_10.JPG.dat	1204.3	25.0	1.0	0.02	#NUM!	9.7E-07
YLR434C	YLR434C	S288C_ctrl_hmg124h_10.JPG.dat	1165.3	16.8	1.0	0.02	#NUM!	1.1E-06
YLR435W	YLR435W	S288C_ctrl_hmg124h_10.JPG.dat	926.5	23.2	0.8	0.03	#NUM!	8.1E-06
YLR436C	YLR436C	S288C_ctrl_hmg124h_10.JPG.dat	1158.0	37.2	1.0	0.03	#NUM!	8.1E-06
YLR437C	YLR437C	S288C_ctrl_hmg124h_10.JPG.dat	1221.0	15.9	1.0	0.01	#NUM!	3.3E-07
YLR438W	YLR438W	S288C_ctrl_hmg124h_10.JPG.dat	1260.0	19.6	1.0	0.01	#NUM!	7.9E-07
YLR441C	YLR441C	S288C_ctrl_hmg124h_10.JPG.dat	988.5	27.6	0.8	0.01	#NUM!	3.3E-05
YLR442C	YLR442C	S288C_ctrl_hmg124h_10.JPG.dat	0.0	0.0	0.0	0.00	#NUM!	
YLR443W	YLR443W	S288C_ctrl_hmg124h_10.JPG.dat	1317.0	45.7	1.0	0.03	#NUM!	8.5E-06
YLR444C	YLR444C	S288C_ctrl_hmg124h_10.JPG.dat	1317.3	55.9	1.1	0.04	#NUM!	1.4E-05
YLR445W	YLR445W	S288C_ctrl_hmg124h_10.JPG.dat	1242.3	22.8	1.0	0.02	#NUM!	2.2E-06
YLR446W	YLR446W	S288C_ctrl_hmg124h_10.JPG.dat	1168.0	38.2	1.0	0.04	#NUM!	1.5E-05
YLR448W	YLR448W	S288C_ctrl_hmg124h_10.JPG.dat	990.0	20.9	0.9	0.01	#NUM!	1.1E-05
YLR449W	YLR449W	S288C_ctrl_hmg124h_10.JPG.dat	1001.0	30.3	0.9	0.03	#NUM!	1.0E-05
YLR450W	YLR450W	S288C_ctrl_hmg124h_10.JPG.dat	0.0	0.0	0.0	0.00	#NUM!	
YLR451W	YLR451W	S288C_ctrl_hmg124h_10.JPG.dat	1172.3	21.5	1.0	0.02	#NUM!	1.2E-06
YLR452C	YLR452C	S288C_ctrl_hmg124h_10.JPG.dat	998.8	57.1	0.8	0.01	#NUM!	3.8E-05

YLR453C	YLR453C	S288C_ctrl_hmg124h_10.JPG.dat	1245.3	30.2	1.0	0.03	#NUM!	4.8E-06
YLR454W	YLR454W	S288C_ctrl_hmg124h_10.JPG.dat	1263.8	34.3	1.1	0.01	#NUM!	3.6E-05
YLR455W	YLR455W	S288C_ctrl_hmg124h_10.JPG.dat	1295.5	43.5	1.1	0.04	#NUM!	1.5E-05
YLR456W	YLR456W	S288C_ctrl_hmg124h_10.JPG.dat	1209.0	28.1	1.0	0.02	#NUM!	1.6E-06
YLR460C	YLR460C	S288C_ctrl_hmg124h_10.JPG.dat	1192.0	25.6	1.0	0.02	#NUM!	3.4E-06
YLR461W	YLR461W	S288C_ctrl_hmg124h_10.JPG.dat	1108.0	26.8	1.0	0.02	#NUM!	2.0E-06
YML001W	YML001W	S288C_ctrl_hmg124h_10.JPG.dat	994.3	8.1	0.9	0.01	#NUM!	3.2E-07
YML002W	YML002W	S288C_ctrl_hmg124h_10.JPG.dat	1073.8	41.7	1.0	0.02	#NUM!	8.1E-05
YML003W	YML003W	S288C_ctrl_hmg124h_10.JPG.dat	1085.5	38.7	1.0	0.03	#NUM!	1.0E-05
YML004C	YML004C	S288C_ctrl_hmg124h_10.JPG.dat	1103.8	32.2	1.0	0.03	#NUM!	4.3E-06
YML005W	YML005W	S288C_ctrl_hmg124h_10.JPG.dat	1160.3	24.6	1.0	0.01	#NUM!	2.7E-05
YML006C	YML006C	S288C_ctrl_hmg124h_10.JPG.dat	1185.3	58.7	1.0	0.05	#NUM!	3.0E-05
YML007W	YML007W	S288C_ctrl_hmg124h_10.JPG.dat	1067.3	49.6	0.9	0.04	#NUM!	1.6E-05
YML008C	YML008C	S288C_ctrl_hmg124h_10.JPG.dat	1239.3	15.6	1.0	0.01	#NUM!	8.0E-07
YML009c	YML009c	S288C_ctrl_hmg124h_10.JPG.dat	1225.3	18.3	1.0	0.01	#NUM!	1.1E-05
YML010C-I	YML010C-B	S288C_ctrl_hmg124h_10.JPG.dat	1121.0	37.3	1.0	0.03	#NUM!	1.1E-05
YML011C	YML011C	S288C_ctrl_hmg124h_10.JPG.dat	1086.5	47.6	1.0	0.04	#NUM!	1.5E-05
YML012W	YML012W	S288C_ctrl_hmg124h_10.JPG.dat	1126.5	32.1	1.0	0.01	#NUM!	2.9E-05
YML013C-/	YML013C-A	S288C_ctrl_hmg124h_10.JPG.dat	1086.8	25.8	1.0	0.03	#NUM!	4.9E-06
YML013W	YML013W	S288C_ctrl_hmg124h_10.JPG.dat	1066.8	45.7	1.0	0.05	#NUM!	3.0E-05
YML016C	YML016C	S288C_ctrl_hmg124h_10.JPG.dat	1192.5	11.8	1.1	0.01	#NUM!	7.1E-07
YML017W	YML017W	S288C_ctrl_hmg124h_10.JPG.dat	1140.3	307.9	1.1	0.03	#NUM!	2.7E-04
YML018C	YML018C	S288C_ctrl_hmg124h_10.JPG.dat	1260.8	53.7	1.1	0.05	#NUM!	2.4E-05
YML019W	YML019W	S288C_ctrl_hmg124h_10.JPG.dat	1139.0	56.4	1.0	0.04	#NUM!	1.9E-05
YML020W	YML020W	S288C_ctrl_hmg124h_10.JPG.dat	1293.3	30.2	1.0	0.00	#NUM!	7.2E-06
YML021C	YML021C	S288C_ctrl_hmg124h_10.JPG.dat	1222.8	37.9	1.0	0.04	#NUM!	1.4E-05
YML022W	YML022W	S288C_ctrl_hmg124h_10.JPG.dat	1150.8	80.0	0.9	0.02	#NUM!	9.5E-05
YML026C	YML026C	S288C_ctrl_hmg124h_10.JPG.dat	920.0	32.4	0.8	0.02	#NUM!	4.7E-06
YML027W	YML027W	S288C_ctrl_hmg124h_10.JPG.dat	1140.3	22.6	1.0	0.01	#NUM!	2.7E-05
YML028W	YML028W	S288C_ctrl_hmg124h_10.JPG.dat	1062.5	33.3	0.9	0.00	#NUM!	2.9E-06
YML029W	YML029W	S288C_ctrl_hmg124h_10.JPG.dat	1200.0	30.5	1.0	0.00	#NUM!	5.5E-07
YML030W	YML030W	S288C_ctrl_hmg124h_10.JPG.dat	1136.8	23.7	1.0	0.02	#NUM!	1.7E-06
YML032C	YML032C	S288C_ctrl_hmg124h_10.JPG.dat	1009.3	12.7	0.8	0.01	#NUM!	1.3E-06
YML033W	YML033W	S288C_ctrl_hmg124h_10.JPG.dat	1181.0	53.6	1.0	0.04	#NUM!	1.7E-05
YML034W	YML034W	S288C_ctrl_hmg124h_10.JPG.dat	1135.3	72.8	1.0	0.07	#NUM!	8.7E-05
YML035C	YML035C	S288C_ctrl_hmg124h_10.JPG.dat	952.3	123.7	0.7	0.10	#NUM!	8.9E-04
YML035C-/	YML035C-A	S288C_ctrl_hmg124h_10.JPG.dat	1372.5	79.1	1.0	0.04	#NUM!	1.3E-05
YML036W	YML036W	S288C_ctrl_hmg124h_10.JPG.dat	1357.0	48.4	1.0	0.02	#NUM!	1.2E-06
YML037C	YML037C	S288C_ctrl_hmg124h_10.JPG.dat	1347.0	33.9	1.0	0.01	#NUM!	2.6E-05
YML038C	YML038C	S288C_ctrl_hmg124h_10.JPG.dat	1307.8	23.6	1.0	0.01	#NUM!	4.7E-07
YML041C	YML041C	S288C_ctrl_hmg124h_10.JPG.dat	1231.8	44.6	1.0	0.02	#NUM!	2.7E-06
YML042W	YML042W	S288C_ctrl_hmg124h_10.JPG.dat	1346.0	93.6	1.0	0.05	#NUM!	2.9E-05
YML047C	YML047C	S288C_ctrl_hmg124h_10.JPG.dat	1265.5	34.5	1.0	0.00	#NUM!	7.0E-06
YML048W	YML048W	S288C_ctrl_hmg124h_10.JPG.dat	1297.8	24.0	1.0	0.00	#NUM!	7.9E-06
YML048W-	YML048W-A	S288C_ctrl_hmg124h_10.JPG.dat	1327.3	37.1	1.0	0.02	#NUM!	1.9E-06
YML051W	YML051W	S288C_ctrl_hmg124h_10.JPG.dat	1228.3	179.8	0.9	0.12	#NUM!	5.7E-04
YML052W	YML052W	S288C_ctrl_hmg124h_10.JPG.dat	1266.8	48.9	1.0	0.05	#NUM!	2.6E-05
YML053C	YML053C	S288C_ctrl_hmg124h_10.JPG.dat	1235.5	70.2	1.0	0.05	#NUM!	4.1E-05
YML054C	YML054C	S288C_ctrl_hmg124h_10.JPG.dat	1228.3	34.0	1.0	0.03	#NUM!	8.1E-06
YML055W	YML055W	S288C_ctrl_hmg124h_10.JPG.dat	1151.8	19.3	0.9	0.02	#NUM!	3.0E-06
YML056C	YML056C	S288C_ctrl_hmg124h_10.JPG.dat	1165.8	68.4	0.9	0.01	#NUM!	4.1E-05
YML057W	YML057W	S288C_ctrl_hmg124h_10.JPG.dat	1162.3	26.7	1.0	0.02	#NUM!	3.2E-06
YML058C-/	YML058C-A	S288C_ctrl_hmg124h_10.JPG.dat	903.5	602.6	1.0	0.02	#NUM!	1.9E-04
YML058W	YML058W	S288C_ctrl_hmg124h_10.JPG.dat	1263.8	74.5	1.0	0.06	#NUM!	6.5E-05
YML059C	YML059C	S288C_ctrl_hmg124h_10.JPG.dat	1313.8	34.6	1.1	0.03	#NUM!	6.7E-06
YML060W	YML060W	S288C_ctrl_hmg124h_10.JPG.dat	1235.0	23.7	1.0	0.02	#NUM!	2.0E-06
YML062C	YML062C	S288C_ctrl_hmg124h_10.JPG.dat	1237.0	25.2	1.0	0.02	#NUM!	1.9E-06
YML063W	YML063W	S288C_ctrl_hmg124h_10.JPG.dat	546.5	652.7	0.4	0.51	#NUM!	1.9E-01
YML066C	YML066C	S288C_ctrl_hmg124h_10.JPG.dat	1234.5	55.1	1.0	0.04	#NUM!	2.6E-05
YML067C	YML067C	S288C_ctrl_hmg124h_10.JPG.dat	1185.0	62.7	0.9	0.05	#NUM!	4.3E-05
YML068W	YML068W	S288C_ctrl_hmg124h_10.JPG.dat	933.3	622.4	1.0	0.01	#NUM!	5.5E-05
YML070W	YML070W	S288C_ctrl_hmg124h_10.JPG.dat	861.8	576.1	1.0	0.05	#NUM!	7.6E-04
YML071C	YML071C	S288C_ctrl_hmg124h_10.JPG.dat	854.8	571.8	1.0	0.05	#NUM!	8.9E-04
YML072C	YML072C	S288C_ctrl_hmg124h_10.JPG.dat	610.5	705.4	0.5	0.61	#NUM!	1.8E-01
YML074C	YML074C	S288C_ctrl_hmg124h_10.JPG.dat	585.5	676.4	0.5	0.57	#NUM!	1.8E-01
YML075C	YML075C	S288C_ctrl_hmg124h_10.JPG.dat	306.8	613.5	0.0	0.00	#NUM!	
YML076C	YML076C	S288C_ctrl_hmg124h_10.JPG.dat	561.8	656.2	0.5	0.54	#NUM!	1.9E-01
YML081C-/	YML081C-A	S288C_ctrl_hmg124h_10.JPG.dat	836.5	566.4	0.9	0.10	#NUM!	3.7E-03
YML090W	YML090W	S288C_ctrl_hmg124h_10.JPG.dat	1037.3	71.0	0.8	0.07	#NUM!	1.5E-04
YML094W	YML094W	S288C_ctrl_hmg124h_10.JPG.dat	317.3	634.5	0.0	0.00	#NUM!	

YML095C	YML095C	S288C_ctrl_hmg124h_10.JPG.dat	1240.5	42.9	1.0	0.03	#NUM!	8.7E-06
YML096W	YML096W	S288C_ctrl_hmg124h_10.JPG.dat	1206.8	38.2	1.0	0.02	#NUM!	2.9E-06
YML097C	YML097C	S288C_ctrl_hmg124h_10.JPG.dat	1046.8	66.5	0.9	0.06	#NUM!	8.8E-05
YML099C	YML099C	S288C_ctrl_hmg124h_10.JPG.dat	1059.5	44.0	1.0	0.04	#NUM!	1.7E-05
YML100W	YML100W	S288C_ctrl_hmg124h_10.JPG.dat	1105.5	36.3	1.0	0.04	#NUM!	1.3E-05
YML100W·	YML100W-A	S288C_ctrl_hmg124h_10.JPG.dat	1163.3	39.0	1.0	0.00	#NUM!	7.0E-06
YML101C	YML101C	S288C_ctrl_hmg124h_10.JPG.dat	1145.8	17.3	1.0	0.01	#NUM!	3.3E-07
YML102C-/	YML102C-A	S288C_ctrl_hmg124h_10.JPG.dat	1108.5	33.6	0.9	0.03	#NUM!	8.6E-06
YML102W	YML102W	S288C_ctrl_hmg124h_10.JPG.dat	1219.5	12.4	1.0	0.01	#NUM!	1.2E-07
YML103C	YML103C	S288C_ctrl_hmg124h_10.JPG.dat	1180.5	25.5	1.0	0.01	#NUM!	4.1E-05
YML104C	YML104C	S288C_ctrl_hmg124h_10.JPG.dat	1182.0	17.1	1.0	0.01	#NUM!	1.8E-07
YML106W	YML106W	S288C_ctrl_hmg124h_10.JPG.dat	1116.0	25.2	1.0	0.02	#NUM!	3.3E-06
YML107C	YML107C	S288C_ctrl_hmg124h_10.JPG.dat	1023.8	37.1	0.9	0.01	#NUM!	1.4E-05
YML108W	YML108W	S288C_ctrl_hmg124h_10.JPG.dat	1052.5	24.9	1.0	0.03	#NUM!	7.7E-06
YML109W	YML109W	S288C_ctrl_hmg124h_10.JPG.dat	1118.5	49.8	1.1	0.04	#NUM!	2.0E-05
YML113W	YML113W	S288C_ctrl_hmg124h_10.JPG.dat	1099.5	28.1	1.0	0.03	#NUM!	6.6E-06
YML116W	YML116W	S288C_ctrl_hmg124h_10.JPG.dat	1106.0	18.0	1.0	0.02	#NUM!	1.8E-06
YML117W	YML117W	S288C_ctrl_hmg124h_10.JPG.dat	1185.0	23.2	1.1	0.01	#NUM!	6.6E-07
YML117W·	YML117W-A	S288C_ctrl_hmg124h_10.JPG.dat	1237.0	24.4	1.1	0.02	#NUM!	9.6E-07
YML118W	YML118W	S288C_ctrl_hmg124h_10.JPG.dat	1191.8	47.8	1.0	0.03	#NUM!	1.1E-05
YML119W	YML119W	S288C_ctrl_hmg124h_10.JPG.dat	1260.3	60.5	1.1	0.01	#NUM!	4.2E-05
YML120C	YML120C	S288C_ctrl_hmg124h_10.JPG.dat	1263.5	23.7	1.0	0.00	#NUM!	1.2E-06
YML121W	YML121W	S288C_ctrl_hmg124h_10.JPG.dat	1043.0	31.1	0.9	0.03	#NUM!	8.5E-06
YML122C	YML122C	S288C_ctrl_hmg124h_10.JPG.dat	1079.0	69.0	0.9	0.06	#NUM!	5.6E-05
YML123C	YML123C	S288C_ctrl_hmg124h_10.JPG.dat	1021.0	54.1	0.9	0.05	#NUM!	3.5E-05
YML124C	YML124C	S288C_ctrl_hmg124h_10.JPG.dat	1089.3	49.7	1.0	0.04	#NUM!	2.4E-05
YML128C	YML128C	S288C_ctrl_hmg124h_10.JPG.dat	1046.8	66.0	1.0	0.06	#NUM!	7.4E-05
YML131W	YML131W	S288C_ctrl_hmg124h_10.JPG.dat	1093.0	38.3	1.0	0.03	#NUM!	7.4E-06
YMR002W	YMR002W	S288C_ctrl_hmg124h_10.JPG.dat	1144.3	63.3	1.0	0.05	#NUM!	3.4E-05
YMR003W	YMR003W	S288C_ctrl_hmg124h_10.JPG.dat	1215.5	23.4	1.0	0.02	#NUM!	1.4E-06
YMR004W	YMR004W	S288C_ctrl_hmg124h_10.JPG.dat	1202.8	72.4	1.0	0.06	#NUM!	5.1E-05
YMR006C	YMR006C	S288C_ctrl_hmg124h_10.JPG.dat	1364.8	33.2	1.1	0.04	#NUM!	1.0E-05
YMR007W	YMR007W	S288C_ctrl_hmg124h_10.JPG.dat	1236.5	36.6	1.0	0.04	#NUM!	1.6E-05
YMR008C	YMR008C	S288C_ctrl_hmg124h_10.JPG.dat	1256.8	33.3	1.0	0.03	#NUM!	7.5E-06
YMR009W	YMR009W	S288C_ctrl_hmg124h_10.JPG.dat	1209.0	18.5	1.0	0.01	#NUM!	8.7E-07
YMR010W	YMR010W	S288C_ctrl_hmg124h_10.JPG.dat	1138.8	40.0	1.0	0.04	#NUM!	2.1E-05
YMR011W	YMR011W	S288C_ctrl_hmg124h_10.JPG.dat	1178.8	71.9	1.0	0.05	#NUM!	2.8E-05
YMR012W	YMR012W	S288C_ctrl_hmg124h_10.JPG.dat	1156.3	45.8	1.0	0.03	#NUM!	1.1E-05
YMR015C	YMR015C	S288C_ctrl_hmg124h_10.JPG.dat	1214.3	50.6	1.1	0.03	#NUM!	8.0E-06
YMR016C	YMR016C	S288C_ctrl_hmg124h_10.JPG.dat	1201.0	31.6	1.0	0.02	#NUM!	1.4E-06
YMR017W	YMR017W	S288C_ctrl_hmg124h_10.JPG.dat	1245.8	11.9	1.0	0.01	#NUM!	1.5E-07
YMR018W	YMR018W	S288C_ctrl_hmg124h_10.JPG.dat	1271.0	27.9	1.0	0.02	#NUM!	3.2E-06
YMR019W	YMR019W	S288C_ctrl_hmg124h_10.JPG.dat	1364.0	49.3	1.0	0.05	#NUM!	3.4E-05
YMR020W	YMR020W	S288C_ctrl_hmg124h_10.JPG.dat	1401.3	38.1	1.0	0.02	#NUM!	9.4E-07
YMR021C	YMR021C	S288C_ctrl_hmg124h_10.JPG.dat	1423.8	28.3	1.0	0.01	#NUM!	2.7E-07
YMR022W	YMR022W	S288C_ctrl_hmg124h_10.JPG.dat	1400.0	39.3	1.0	0.01	#NUM!	3.7E-07
YMR023C	YMR023C	S288C_ctrl_hmg124h_10.JPG.dat	1341.3	18.3	1.0	0.03	#NUM!	5.2E-06
YMR025W	YMR025W	S288C_ctrl_hmg124h_10.JPG.dat	1289.5	58.3	1.0	0.02	#NUM!	3.0E-06
YMR026C	YMR026C	S288C_ctrl_hmg124h_10.JPG.dat	1285.0	22.2	1.0	0.00	#NUM!	8.4E-06
YMR027W	YMR027W	S288C_ctrl_hmg124h_10.JPG.dat	1267.0	35.1	1.0	0.01	#NUM!	8.7E-07
YMR029C	YMR029C	S288C_ctrl_hmg124h_10.JPG.dat	1282.8	14.4	1.0	0.02	#NUM!	1.2E-06
YMR030W	YMR030W	S288C_ctrl_hmg124h_10.JPG.dat	1342.0	17.5	1.0	0.01	#NUM!	8.2E-06
YMR031C	YMR031C	S288C_ctrl_hmg124h_10.JPG.dat	1303.8	36.9	1.0	0.01	#NUM!	1.8E-05
YMR031W	YMR031W-A	S288C_ctrl_hmg124h_10.JPG.dat	519.8	622.1	0.4	0.47	#NUM!	1.9E-01
YMR034C	YMR034C	S288C_ctrl_hmg124h_10.JPG.dat	1231.0	34.0	1.0	0.03	#NUM!	9.2E-06
YMR035W	YMR035W	S288C_ctrl_hmg124h_10.JPG.dat	1170.3	34.7	0.9	0.03	#NUM!	6.3E-06
YMR036C	YMR036C	S288C_ctrl_hmg124h_10.JPG.dat	1261.5	32.6	1.0	0.03	#NUM!	8.1E-06
YMR037C	YMR037C	S288C_ctrl_hmg124h_10.JPG.dat	1152.5	38.0	1.0	0.03	#NUM!	1.0E-05
YMR038C	YMR038C	S288C_ctrl_hmg124h_10.JPG.dat	0.0	0.0	0.0	0.00	#NUM!	
YMR039C	YMR039C	S288C_ctrl_hmg124h_10.JPG.dat	1237.5	56.7	1.0	0.01	#NUM!	9.6E-06
YMR040W	YMR040W	S288C_ctrl_hmg124h_10.JPG.dat	1192.3	16.3	1.0	0.02	#NUM!	1.1E-06
YMR041C	YMR041C	S288C_ctrl_hmg124h_10.JPG.dat	1233.8	106.8	1.0	0.09	#NUM!	1.9E-04
YMR042W	YMR042W	S288C_ctrl_hmg124h_10.JPG.dat	1221.8	13.2	1.0	0.01	#NUM!	4.9E-08
YMR044W	YMR044W	S288C_ctrl_hmg124h_10.JPG.dat	1241.0	47.2	1.0	0.04	#NUM!	2.2E-05
YMR048W	YMR048W	S288C_ctrl_hmg124h_10.JPG.dat	1269.0	75.6	1.0	0.06	#NUM!	7.1E-05
YMR052C-.	YMR052C-A	S288C_ctrl_hmg124h_10.JPG.dat	1295.0	38.2	1.0	0.03	#NUM!	6.4E-06
YMR052W	YMR052W	S288C_ctrl_hmg124h_10.JPG.dat	1274.8	23.2	1.0	0.02	#NUM!	1.2E-06
YMR053C	YMR053C	S288C_ctrl_hmg124h_10.JPG.dat	1258.5	14.4	1.0	0.01	#NUM!	1.2E-07
YMR054W	YMR054W	S288C_ctrl_hmg124h_10.JPG.dat	1188.0	39.9	1.0	0.03	#NUM!	1.1E-05
YMR055C	YMR055C	S288C_ctrl_hmg124h_10.JPG.dat	1198.3	44.1	1.0	0.03	#NUM!	9.9E-06

YMR056C	YMR056C	S288C_ctrl_hmg124h_10.JPG.dat	1199.3	32.0	1.0	0.03	#NUM!	5.3E-06
YMR057C	YMR057C	S288C_ctrl_hmg124h_10.JPG.dat	1233.5	33.5	1.0	0.03	#NUM!	5.4E-06
YMR058W	YMR058W	S288C_ctrl_hmg124h_10.JPG.dat	1229.3	9.6	1.0	0.01	#NUM!	1.1E-07
YMR060C	YMR060C	S288C_ctrl_hmg124h_10.JPG.dat	1116.8	25.1	0.9	0.02	#NUM!	1.7E-06
YMR063W	YMR063W	S288C_ctrl_hmg124h_10.JPG.dat	1224.0	17.6	1.0	0.03	#NUM!	5.8E-06
YMR065W	YMR065W	S288C_ctrl_hmg124h_10.JPG.dat	1326.3	38.5	1.0	0.00	#NUM!	5.2E-06
YMR067C	YMR067C	S288C_ctrl_hmg124h_10.JPG.dat	1338.0	29.1	1.0	0.03	#NUM!	4.9E-06
YMR068W	YMR068W	S288C_ctrl_hmg124h_10.JPG.dat	1323.0	82.4	1.0	0.05	#NUM!	4.0E-05
YMR070W	YMR070W	S288C_ctrl_hmg124h_10.JPG.dat	1168.3	16.9	1.0	0.01	#NUM!	7.1E-07
YMR073C	YMR073C	S288C_ctrl_hmg124h_10.JPG.dat	1056.3	43.2	0.9	0.03	#NUM!	7.2E-06
YMR075C-A	YMR075C-A	S288C_ctrl_hmg124h_10.JPG.dat	1066.3	41.2	0.9	0.04	#NUM!	1.5E-05
YMR075W	YMR075W	S288C_ctrl_hmg124h_10.JPG.dat	1102.0	53.7	0.9	0.04	#NUM!	1.4E-05
YMR078C	YMR078C	S288C_ctrl_hmg124h_10.JPG.dat	1126.5	36.2	0.9	0.02	#NUM!	3.6E-06
YMR080C	YMR080C	S288C_ctrl_hmg124h_10.JPG.dat	1261.8	10.4	1.0	0.02	#NUM!	1.9E-06
YMR085W	YMR085W	S288C_ctrl_hmg124h_10.JPG.dat	1294.3	23.7	1.0	0.01	#NUM!	2.0E-07
YMR086C-A	YMR086C-A	S288C_ctrl_hmg124h_10.JPG.dat	1331.0	41.8	1.1	0.03	#NUM!	3.3E-06
YMR086W	YMR086W	S288C_ctrl_hmg124h_10.JPG.dat	1312.5	23.2	1.0	0.02	#NUM!	1.3E-06
YMR087W	YMR087W	S288C_ctrl_hmg124h_10.JPG.dat	1246.8	23.4	1.0	0.02	#NUM!	1.1E-06
YMR088C	YMR088C	S288C_ctrl_hmg124h_10.JPG.dat	920.0	614.0	1.0	0.02	#NUM!	2.0E-04
YMR092C	YMR092C	S288C_ctrl_hmg124h_10.JPG.dat	1087.8	41.5	1.0	0.04	#NUM!	1.5E-05
YMR095C	YMR095C	S288C_ctrl_hmg124h_10.JPG.dat	1119.5	40.4	1.0	0.04	#NUM!	1.5E-05
YMR096W	YMR096W	S288C_ctrl_hmg124h_10.JPG.dat	1141.8	36.2	1.0	0.01	#NUM!	5.0E-05
YMR099C	YMR099C	S288C_ctrl_hmg124h_10.JPG.dat	1132.5	22.8	1.0	0.01	#NUM!	9.9E-06
YMR100W	YMR100W	S288C_ctrl_hmg124h_10.JPG.dat	1037.8	19.7	0.9	0.01	#NUM!	1.1E-05
YMR101C	YMR101C	S288C_ctrl_hmg124h_10.JPG.dat	1154.0	43.3	1.0	0.03	#NUM!	9.6E-06
YMR102C	YMR102C	S288C_ctrl_hmg124h_10.JPG.dat	1228.5	12.1	1.0	0.01	#NUM!	5.3E-07
YMR103C	YMR103C	S288C_ctrl_hmg124h_10.JPG.dat	1170.8	92.5	1.0	0.08	#NUM!	1.4E-04
YMR104C	YMR104C	S288C_ctrl_hmg124h_10.JPG.dat	1309.0	33.6	1.0	0.03	#NUM!	4.3E-06
YMR105C	YMR105C	S288C_ctrl_hmg124h_10.JPG.dat	1149.0	52.2	0.9	0.05	#NUM!	3.5E-05
YMR106C	YMR106C	S288C_ctrl_hmg124h_10.JPG.dat	1261.5	19.8	1.1	0.02	#NUM!	1.0E-06
YMR107W	YMR107W	S288C_ctrl_hmg124h_10.JPG.dat	1176.0	21.1	1.0	0.02	#NUM!	1.5E-06
YMR109W	YMR109W	S288C_ctrl_hmg124h_10.JPG.dat	1146.5	17.2	1.1	0.00	#NUM!	5.7E-07
YMR110C	YMR110C	S288C_ctrl_hmg124h_10.JPG.dat	1117.0	30.8	1.0	0.03	#NUM!	7.0E-06
YMR111C	YMR111C	S288C_ctrl_hmg124h_10.JPG.dat	1121.8	33.5	1.0	0.03	#NUM!	8.4E-06
YMR114C	YMR114C	S288C_ctrl_hmg124h_10.JPG.dat	1114.5	25.6	1.0	0.02	#NUM!	2.6E-06
YMR115W	YMR115W	S288C_ctrl_hmg124h_10.JPG.dat	1171.8	23.5	1.0	0.02	#NUM!	1.1E-06
YMR116C	YMR116C	S288C_ctrl_hmg124h_10.JPG.dat	478.3	625.9	0.4	0.52	#NUM!	2.2E-01
YMR119W	YMR119W	S288C_ctrl_hmg124h_10.JPG.dat	1191.3	45.0	1.0	0.02	#NUM!	2.5E-06
YMR119W	YMR119W-A	S288C_ctrl_hmg124h_10.JPG.dat	1250.3	10.2	1.0	0.01	#NUM!	1.3E-07
YMR120C	YMR120C	S288C_ctrl_hmg124h_10.JPG.dat	1219.5	69.8	1.0	0.05	#NUM!	3.6E-05
YMR121C	YMR121C	S288C_ctrl_hmg124h_10.JPG.dat	1220.0	26.4	1.0	0.02	#NUM!	2.5E-06
YMR122C	YMR122C	S288C_ctrl_hmg124h_10.JPG.dat	1144.8	30.1	1.0	0.03	#NUM!	7.0E-06
YMR123W	YMR123W	S288C_ctrl_hmg124h_10.JPG.dat	900.8	12.4	0.8	0.01	#NUM!	1.3E-06
YMR124W	YMR124W	S288C_ctrl_hmg124h_10.JPG.dat	1129.0	22.2	1.0	0.02	#NUM!	1.4E-06
YMR126C	YMR126C	S288C_ctrl_hmg124h_10.JPG.dat	1118.8	26.8	1.0	0.02	#NUM!	3.5E-06
YMR127C	YMR127C	S288C_ctrl_hmg124h_10.JPG.dat	1142.5	26.7	1.0	0.02	#NUM!	3.9E-06
YMR129W	YMR129W	S288C_ctrl_hmg124h_10.JPG.dat	1182.5	29.6	1.0	0.02	#NUM!	2.9E-06
YMR130W	YMR130W	S288C_ctrl_hmg124h_10.JPG.dat	1268.5	32.8	1.0	0.02	#NUM!	3.6E-06
YMR132C	YMR132C	S288C_ctrl_hmg124h_10.JPG.dat	1189.3	30.2	1.0	0.04	#NUM!	1.7E-05
YMR133W	YMR133W	S288C_ctrl_hmg124h_10.JPG.dat	1330.5	28.8	1.0	0.01	#NUM!	2.8E-07
YMR135C	YMR135C	S288C_ctrl_hmg124h_10.JPG.dat	1267.5	29.3	1.0	0.01	#NUM!	1.1E-05
YMR135W	YMR135W-A	S288C_ctrl_hmg124h_10.JPG.dat	1309.3	34.8	1.0	0.03	#NUM!	5.7E-06
YMR136W	YMR136W	S288C_ctrl_hmg124h_10.JPG.dat	1250.5	28.9	1.0	0.01	#NUM!	6.6E-07
YMR137C	YMR137C	S288C_ctrl_hmg124h_10.JPG.dat	1212.5	35.2	1.0	0.02	#NUM!	3.9E-06
YMR138W	YMR138W	S288C_ctrl_hmg124h_10.JPG.dat	1180.0	52.7	1.0	0.05	#NUM!	3.4E-05
YMR139W	YMR139W	S288C_ctrl_hmg124h_10.JPG.dat	1200.5	13.8	1.0	0.01	#NUM!	4.4E-07
YMR140W	YMR140W	S288C_ctrl_hmg124h_10.JPG.dat	1247.8	43.5	1.0	0.04	#NUM!	1.8E-05
YMR141C	YMR141C	S288C_ctrl_hmg124h_10.JPG.dat	1236.8	9.1	1.0	0.00	#NUM!	4.3E-06
YMR143W	YMR143W	S288C_ctrl_hmg124h_10.JPG.dat	1281.5	13.4	1.0	0.00	#NUM!	4.8E-07
YMR144W	YMR144W	S288C_ctrl_hmg124h_10.JPG.dat	1251.0	45.2	1.0	0.01	#NUM!	2.3E-05
YMR145C	YMR145C	S288C_ctrl_hmg124h_10.JPG.dat	1306.3	50.7	1.0	0.04	#NUM!	2.4E-05
YMR147W	YMR147W	S288C_ctrl_hmg124h_10.JPG.dat	1311.8	60.9	1.0	0.01	#NUM!	6.5E-05
YMR148W	YMR148W	S288C_ctrl_hmg124h_10.JPG.dat	1223.5	33.1	1.0	0.03	#NUM!	6.1E-06
YMR152W	YMR152W	S288C_ctrl_hmg124h_10.JPG.dat	1280.5	24.7	1.0	0.01	#NUM!	1.0E-05
YMR153C-A	YMR153C-A	S288C_ctrl_hmg124h_10.JPG.dat	1182.0	30.9	1.0	0.03	#NUM!	4.8E-06
YMR153W	YMR153W	S288C_ctrl_hmg124h_10.JPG.dat	1216.5	22.2	1.0	0.02	#NUM!	2.3E-06
YMR154C	YMR154C	S288C_ctrl_hmg124h_10.JPG.dat	1227.8	40.7	1.0	0.03	#NUM!	1.0E-05
YMR155W	YMR155W	S288C_ctrl_hmg124h_10.JPG.dat	1279.0	110.7	1.1	0.09	#NUM!	1.6E-04
YMR156C	YMR156C	S288C_ctrl_hmg124h_10.JPG.dat	1297.5	55.7	1.1	0.04	#NUM!	1.7E-05
YMR157C	YMR157C	S288C_ctrl_hmg124h_10.JPG.dat	1254.8	14.3	1.0	0.00	#NUM!	1.0E-06

YMR158C-	YMR158C-B	S288C_ctrl_hmg124h_10.JPG.dat	1307.3	23.2	1.0	0.00	#NUM!	4.4E-06
YMR158W	YMR158W-A	S288C_ctrl_hmg124h_10.JPG.dat	1228.3	70.7	1.0	0.06	#NUM!	6.2E-05
YMR159C	YMR159C	S288C_ctrl_hmg124h_10.JPG.dat	1300.8	48.4	1.0	0.01	#NUM!	4.1E-05
YMR160W	YMR160W	S288C_ctrl_hmg124h_10.JPG.dat	1283.0	12.7	1.0	0.01	#NUM!	1.8E-07
YMR161W	YMR161W	S288C_ctrl_hmg124h_10.JPG.dat	1256.3	37.8	1.0	0.03	#NUM!	5.3E-06
YMR162C	YMR162C	S288C_ctrl_hmg124h_10.JPG.dat	1274.3	51.3	1.1	0.02	#NUM!	9.3E-05
YMR163C	YMR163C	S288C_ctrl_hmg124h_10.JPG.dat	1073.5	15.0	0.9	0.01	#NUM!	6.0E-07
YMR164C	YMR164C	S288C_ctrl_hmg124h_10.JPG.dat	1226.0	16.4	1.0	0.01	#NUM!	6.6E-07
YMR166C	YMR166C	S288C_ctrl_hmg124h_10.JPG.dat	1187.3	37.9	1.0	0.03	#NUM!	7.1E-06
YMR167W	YMR167W	S288C_ctrl_hmg124h_10.JPG.dat	1190.5	21.6	1.0	0.02	#NUM!	2.5E-06
YMR169c	YMR169c	S288C_ctrl_hmg124h_10.JPG.dat	1235.8	17.1	1.0	0.01	#NUM!	8.4E-07
YMR170C	YMR170C	S288C_ctrl_hmg124h_10.JPG.dat	1234.8	22.3	1.0	0.00	#NUM!	1.2E-06
YMR171C	YMR171C	S288C_ctrl_hmg124h_10.JPG.dat	1238.0	77.2	1.0	0.05	#NUM!	4.0E-05
YMR172C-.	YMR172C-A	S288C_ctrl_hmg124h_10.JPG.dat	1369.5	70.3	1.0	0.07	#NUM!	6.6E-05
YMR172W	YMR172W	S288C_ctrl_hmg124h_10.JPG.dat	1251.0	21.1	1.0	0.01	#NUM!	7.7E-07
YMR173W	YMR173W	S288C_ctrl_hmg124h_10.JPG.dat	1283.3	49.1	1.0	0.02	#NUM!	3.4E-06
YMR173W	YMR173W-A	S288C_ctrl_hmg124h_10.JPG.dat	1224.5	24.7	1.1	0.02	#NUM!	1.4E-06
YMR174C	YMR174C	S288C_ctrl_hmg124h_10.JPG.dat	1130.0	60.4	1.0	0.04	#NUM!	2.3E-05
YMR175w	YMR175w	S288C_ctrl_hmg124h_10.JPG.dat	1182.0	34.0	1.0	0.02	#NUM!	2.3E-06
YMR176W	YMR176W	S288C_ctrl_hmg124h_10.JPG.dat	1150.8	56.1	1.0	0.04	#NUM!	1.3E-05
YMR177W	YMR177W	S288C_ctrl_hmg124h_10.JPG.dat	1217.3	24.1	1.0	0.01	#NUM!	5.9E-07
YMR178W	YMR178W	S288C_ctrl_hmg124h_10.JPG.dat	1252.8	24.1	1.0	0.03	#NUM!	7.7E-06
YMR179W	YMR179W	S288C_ctrl_hmg124h_10.JPG.dat	1153.0	116.3	0.9	0.10	#NUM!	3.3E-04
YMR180C	YMR180C	S288C_ctrl_hmg124h_10.JPG.dat	1251.8	55.7	1.0	0.03	#NUM!	9.1E-06
YMR181C	YMR181C	S288C_ctrl_hmg124h_10.JPG.dat	1320.5	35.1	1.1	0.02	#NUM!	3.2E-06
YMR182C	YMR182C	S288C_ctrl_hmg124h_10.JPG.dat	1157.8	32.0	1.0	0.02	#NUM!	4.2E-06
YMR183C	YMR183C	S288C_ctrl_hmg124h_10.JPG.dat	1138.5	28.1	1.0	0.02	#NUM!	3.4E-06
YMR186W	YMR186W	S288C_ctrl_hmg124h_10.JPG.dat	1082.0	33.0	1.0	0.03	#NUM!	1.2E-05
YMR187C	YMR187C	S288C_ctrl_hmg124h_10.JPG.dat	1150.3	27.0	1.1	0.03	#NUM!	3.7E-06
YMR188C	YMR188C	S288C_ctrl_hmg124h_10.JPG.dat	1111.5	55.8	1.0	0.05	#NUM!	3.4E-05
YMR189W	YMR189W	S288C_ctrl_hmg124h_10.JPG.dat	1117.3	24.6	1.0	0.00	#NUM!	1.8E-06
YMR190C	YMR190C	S288C_ctrl_hmg124h_10.JPG.dat	1060.3	6.8	0.9	0.01	#NUM!	5.5E-07
YMR191W	YMR191W	S288C_ctrl_hmg124h_10.JPG.dat	1224.5	50.9	1.0	0.01	#NUM!	4.8E-05
YMR192W	YMR192W	S288C_ctrl_hmg124h_10.JPG.dat	1223.0	21.2	1.0	0.00	#NUM!	9.7E-07
YMR193C-.	YMR193C-A	S288C_ctrl_hmg124h_10.JPG.dat	1291.5	33.9	1.0	0.03	#NUM!	6.6E-06
YMR194W	YMR194W	S288C_ctrl_hmg124h_10.JPG.dat	1131.5	9.0	0.9	0.01	#NUM!	1.9E-07
YMR195W	YMR195W	S288C_ctrl_hmg124h_10.JPG.dat	1247.3	35.5	1.0	0.03	#NUM!	5.8E-06
YMR196W	YMR196W	S288C_ctrl_hmg124h_10.JPG.dat	1165.0	21.4	1.0	0.01	#NUM!	4.9E-07
YMR198W	YMR198W	S288C_ctrl_hmg124h_10.JPG.dat	1087.8	45.6	1.0	0.04	#NUM!	1.4E-05
YMR199W	YMR199W	S288C_ctrl_hmg124h_10.JPG.dat	1086.0	32.6	1.0	0.03	#NUM!	9.1E-06
YMR201C	YMR201C	S288C_ctrl_hmg124h_10.JPG.dat	820.5	547.8	1.0	0.03	#NUM!	3.7E-04
YMR202W	YMR202W	S288C_ctrl_hmg124h_10.JPG.dat	1043.8	31.0	0.9	0.02	#NUM!	5.3E-06
YMR204C	YMR204C	S288C_ctrl_hmg124h_10.JPG.dat	1079.3	19.9	0.9	0.02	#NUM!	1.8E-06
YMR205C	YMR205C	S288C_ctrl_hmg124h_10.JPG.dat	1108.3	52.4	0.9	0.04	#NUM!	2.2E-05
YMR206W	YMR206W	S288C_ctrl_hmg124h_10.JPG.dat	1195.5	72.3	1.0	0.06	#NUM!	5.5E-05
YMR207C	YMR207C	S288C_ctrl_hmg124h_10.JPG.dat	1188.0	30.5	1.0	0.02	#NUM!	3.4E-06
YMR209C	YMR209C	S288C_ctrl_hmg124h_10.JPG.dat	1255.8	22.0	1.0	0.02	#NUM!	1.1E-06
YMR210W	YMR210W	S288C_ctrl_hmg124h_10.JPG.dat	1254.3	27.1	1.1	0.01	#NUM!	1.9E-05
YMR214W	YMR214W	S288C_ctrl_hmg124h_10.JPG.dat	1161.5	12.9	1.0	0.01	#NUM!	1.6E-07
YMR215W	YMR215W	S288C_ctrl_hmg124h_10.JPG.dat	1130.0	51.2	1.0	0.05	#NUM!	3.7E-05
YMR216C	YMR216C	S288C_ctrl_hmg124h_10.JPG.dat	1102.8	15.5	1.0	0.01	#NUM!	3.7E-07
YMR219W	YMR219W	S288C_ctrl_hmg124h_10.JPG.dat	1079.3	28.4	1.0	0.03	#NUM!	5.9E-06
YMR221C	YMR221C	S288C_ctrl_hmg124h_10.JPG.dat	1145.8	34.2	1.0	0.03	#NUM!	6.3E-06
YMR222C	YMR222C	S288C_ctrl_hmg124h_10.JPG.dat	1155.3	28.9	1.0	0.02	#NUM!	3.2E-06
YMR223W	YMR223W	S288C_ctrl_hmg124h_10.JPG.dat	1150.8	27.4	1.0	0.01	#NUM!	1.5E-05
YMR224C	YMR224C	S288C_ctrl_hmg124h_10.JPG.dat	1228.5	53.9	1.0	0.01	#NUM!	6.6E-05
YMR225C	YMR225C	S288C_ctrl_hmg124h_10.JPG.dat	1339.0	24.4	1.0	0.00	#NUM!	1.1E-06
YMR226C	YMR226C	S288C_ctrl_hmg124h_10.JPG.dat	1308.0	39.1	1.0	0.03	#NUM!	4.7E-06
YMR230W	YMR230W	S288C_ctrl_hmg124h_10.JPG.dat	1036.8	29.6	0.8	0.02	#NUM!	4.4E-06
YMR232W	YMR232W	S288C_ctrl_hmg124h_10.JPG.dat	1258.3	44.1	1.0	0.03	#NUM!	5.8E-06
YMR233W	YMR233W	S288C_ctrl_hmg124h_10.JPG.dat	1258.8	26.2	1.0	0.02	#NUM!	1.3E-06
YMR234W	YMR234W	S288C_ctrl_hmg124h_10.JPG.dat	1193.5	53.4	1.0	0.03	#NUM!	6.2E-06
YMR237W	YMR237W	S288C_ctrl_hmg124h_10.JPG.dat	1202.5	51.7	1.0	0.04	#NUM!	1.3E-05
YMR238W	YMR238W	S288C_ctrl_hmg124h_10.JPG.dat	1175.0	27.3	1.0	0.02	#NUM!	1.3E-06
YMR241W	YMR241W	S288C_ctrl_hmg124h_10.JPG.dat	1183.0	36.2	1.0	0.02	#NUM!	3.4E-06
YMR243C	YMR243C	S288C_ctrl_hmg124h_10.JPG.dat	973.0	63.6	0.8	0.05	#NUM!	7.0E-05
YMR244C-.	YMR244C-A	S288C_ctrl_hmg124h_10.JPG.dat	1265.8	50.0	1.0	0.00	#NUM!	5.3E-06
YOR202W	YOR202W	S288C_ctrl_hmg124h_10.JPG.dat	1250.8	169.8	1.0	0.09	#NUM!	7.8E-290
1	1	S288C_ctrl_hmg124h_11.JPG.dat	1365.8	83.5	1.0	0.06	#NUM!	7.3E-05
2	2	S288C_ctrl_hmg124h_11.JPG.dat	1548.0	73.6	1.2	0.05	#NUM!	1.9E-05

3	3	S288C_ctrl_hmg124h_11.JPG.dat	1283.5	55.2	1.1	0.01	#NUM!	1.3E-05
4	4	S288C_ctrl_hmg124h_11.JPG.dat	1352.5	91.8	1.1	0.08	#NUM!	1.1E-04
YJR109C	YJR109C	S288C_ctrl_hmg124h_11.JPG.dat	0.0	0.0	0.0	0.00	#NUM!	
YMR244W	YMR244W	S288C_ctrl_hmg124h_11.JPG.dat	1349.0	44.9	1.0	0.03	#NUM!	9.5E-06
YMR245W	YMR245W	S288C_ctrl_hmg124h_11.JPG.dat	1324.8	37.8	1.0	0.01	#NUM!	1.5E-05
YMR246W	YMR246W	S288C_ctrl_hmg124h_11.JPG.dat	1298.8	19.4	1.0	0.01	#NUM!	3.5E-08
YMR247C	YMR247C	S288C_ctrl_hmg124h_11.JPG.dat	1296.8	32.0	1.0	0.02	#NUM!	1.3E-06
YMR250W	YMR250W	S288C_ctrl_hmg124h_11.JPG.dat	1278.0	25.6	1.0	0.01	#NUM!	2.1E-05
YMR251W	YMR251W	S288C_ctrl_hmg124h_11.JPG.dat	1260.3	29.5	1.0	0.02	#NUM!	3.3E-06
YMR251W	YMR251W-A	S288C_ctrl_hmg124h_11.JPG.dat	1242.8	13.7	1.0	0.01	#NUM!	2.4E-07
YMR252C	YMR252C	S288C_ctrl_hmg124h_11.JPG.dat	1246.8	7.9	1.0	0.01	#NUM!	1.5E-07
YMR253C	YMR253C	S288C_ctrl_hmg124h_11.JPG.dat	1180.0	40.3	1.0	0.03	#NUM!	6.6E-06
YMR254C	YMR254C	S288C_ctrl_hmg124h_11.JPG.dat	1085.8	23.7	0.9	0.02	#NUM!	3.2E-06
YMR255W	YMR255W	S288C_ctrl_hmg124h_11.JPG.dat	1127.5	58.1	0.9	0.05	#NUM!	4.2E-05
YMR256C	YMR256C	S288C_ctrl_hmg124h_11.JPG.dat	0.0	0.0	0.0	0.00	#NUM!	
YMR258C	YMR258C	S288C_ctrl_hmg124h_11.JPG.dat	1368.5	45.7	1.0	0.04	#NUM!	1.2E-05
YMR259C	YMR259C	S288C_ctrl_hmg124h_11.JPG.dat	1368.0	45.4	1.0	0.03	#NUM!	9.8E-06
YMR261C	YMR261C	S288C_ctrl_hmg124h_11.JPG.dat	1392.3	55.0	1.0	0.04	#NUM!	1.7E-05
YMR262W	YMR262W	S288C_ctrl_hmg124h_11.JPG.dat	1301.3	36.4	1.0	0.01	#NUM!	1.3E-05
YMR263W	YMR263W	S288C_ctrl_hmg124h_11.JPG.dat	1238.8	14.4	1.0	0.01	#NUM!	3.3E-07
YMR264W	YMR264W	S288C_ctrl_hmg124h_11.JPG.dat	1307.5	16.9	1.0	0.01	#NUM!	5.1E-07
YMR265C	YMR265C	S288C_ctrl_hmg124h_11.JPG.dat	1255.3	10.6	1.0	0.00	#NUM!	3.5E-06
YMR266W	YMR266W	S288C_ctrl_hmg124h_11.JPG.dat	1218.8	27.0	1.0	0.02	#NUM!	1.8E-06
YMR269W	YMR269W	S288C_ctrl_hmg124h_11.JPG.dat	1192.8	7.5	1.0	0.00	#NUM!	7.4E-09
YMR271C	YMR271C	S288C_ctrl_hmg124h_11.JPG.dat	1252.5	74.7	1.0	0.06	#NUM!	7.0E-05
YMR272C	YMR272C	S288C_ctrl_hmg124h_11.JPG.dat	1236.0	54.2	0.9	0.01	#NUM!	4.3E-05
YMR273C	YMR273C	S288C_ctrl_hmg124h_11.JPG.dat	1328.5	39.2	1.0	0.02	#NUM!	1.9E-06
YMR274C	YMR274C	S288C_ctrl_hmg124h_11.JPG.dat	1386.3	60.4	1.0	0.03	#NUM!	7.7E-06
YMR275C	YMR275C	S288C_ctrl_hmg124h_11.JPG.dat	1357.8	39.2	1.0	0.03	#NUM!	4.9E-06
YMR276W	YMR276W	S288C_ctrl_hmg124h_11.JPG.dat	1295.5	55.5	1.0	0.03	#NUM!	8.6E-06
YMR278W	YMR278W	S288C_ctrl_hmg124h_11.JPG.dat	1299.5	40.6	1.0	0.03	#NUM!	8.4E-06
YMR279C	YMR279C	S288C_ctrl_hmg124h_11.JPG.dat	1293.0	27.1	1.0	0.01	#NUM!	2.7E-05
YMR280C	YMR280C	S288C_ctrl_hmg124h_11.JPG.dat	1372.5	58.5	1.1	0.01	#NUM!	1.4E-05
YMR282C	YMR282C	S288C_ctrl_hmg124h_11.JPG.dat	290.5	581.0	0.0	0.00	#NUM!	
YMR283C	YMR283C	S288C_ctrl_hmg124h_11.JPG.dat	1214.8	14.2	1.0	0.02	#NUM!	2.0E-06
YMR284W	YMR284W	S288C_ctrl_hmg124h_11.JPG.dat	1321.0	18.2	1.1	0.01	#NUM!	1.8E-05
YMR285C	YMR285C	S288C_ctrl_hmg124h_11.JPG.dat	1314.0	28.9	1.0	0.02	#NUM!	3.3E-06
YMR289W	YMR289W	S288C_ctrl_hmg124h_11.JPG.dat	1366.3	40.2	1.0	0.03	#NUM!	4.3E-06
YMR291W	YMR291W	S288C_ctrl_hmg124h_11.JPG.dat	1331.3	23.7	1.0	0.00	#NUM!	6.5E-06
YMR292W	YMR292W	S288C_ctrl_hmg124h_11.JPG.dat	1229.3	15.2	1.0	0.00	#NUM!	4.1E-06
YMR294W	YMR294W	S288C_ctrl_hmg124h_11.JPG.dat	1321.8	91.0	1.1	0.08	#NUM!	1.1E-04
YMR294W	YMR294W-A	S288C_ctrl_hmg124h_11.JPG.dat	1293.3	11.1	1.1	0.00	#NUM!	2.4E-06
YMR295C	YMR295C	S288C_ctrl_hmg124h_11.JPG.dat	1247.8	49.9	1.0	0.01	#NUM!	3.5E-05
YMR297W	YMR297W	S288C_ctrl_hmg124h_11.JPG.dat	1259.0	37.2	1.0	0.03	#NUM!	9.4E-06
YMR299C	YMR299C	S288C_ctrl_hmg124h_11.JPG.dat	1273.5	21.5	1.0	0.02	#NUM!	2.2E-06
YMR300C	YMR300C	S288C_ctrl_hmg124h_11.JPG.dat	1266.5	26.9	1.0	0.02	#NUM!	3.0E-06
YMR302C	YMR302C	S288C_ctrl_hmg124h_11.JPG.dat	1190.8	104.1	1.0	0.08	#NUM!	1.5E-04
YMR303C	YMR303C	S288C_ctrl_hmg124h_11.JPG.dat	1306.0	24.2	1.0	0.02	#NUM!	1.7E-06
YMR304C-.	YMR304C-A	S288C_ctrl_hmg124h_11.JPG.dat	1314.8	78.4	1.0	0.06	#NUM!	4.8E-05
YMR304W	YMR304W	S288C_ctrl_hmg124h_11.JPG.dat	1197.8	31.2	1.0	0.03	#NUM!	8.1E-06
YMR305C	YMR305C	S288C_ctrl_hmg124h_11.JPG.dat	1244.3	26.3	1.0	0.01	#NUM!	2.0E-05
YMR306C-.	YMR306C-A	S288C_ctrl_hmg124h_11.JPG.dat	1138.8	32.1	1.0	0.03	#NUM!	5.8E-06
YMR306W	YMR306W	S288C_ctrl_hmg124h_11.JPG.dat	1187.5	16.5	1.0	0.00	#NUM!	4.8E-06
YMR307W	YMR307W	S288C_ctrl_hmg124h_11.JPG.dat	1177.3	35.6	1.0	0.03	#NUM!	1.1E-05
YMR310C	YMR310C	S288C_ctrl_hmg124h_11.JPG.dat	1222.5	15.2	1.0	0.01	#NUM!	2.4E-07
YMR311C	YMR311C	S288C_ctrl_hmg124h_11.JPG.dat	1234.0	24.1	1.0	0.02	#NUM!	2.4E-06
YMR312W	YMR312W	S288C_ctrl_hmg124h_11.JPG.dat	1050.5	7.0	0.9	0.01	#NUM!	1.2E-07
YMR313C	YMR313C	S288C_ctrl_hmg124h_11.JPG.dat	1207.8	57.7	1.0	0.04	#NUM!	1.4E-05
YMR315W	YMR315W	S288C_ctrl_hmg124h_11.JPG.dat	1299.5	45.3	1.0	0.03	#NUM!	8.0E-06
YMR316C-.	YMR316C-A	S288C_ctrl_hmg124h_11.JPG.dat	1291.5	18.0	1.0	0.02	#NUM!	3.7E-06
YMR316C-	YMR316C-B	S288C_ctrl_hmg124h_11.JPG.dat	1281.0	75.7	1.0	0.05	#NUM!	2.8E-05
YMR316W	YMR316W	S288C_ctrl_hmg124h_11.JPG.dat	1167.0	41.8	1.0	0.03	#NUM!	6.8E-06
YMR317W	YMR317W	S288C_ctrl_hmg124h_11.JPG.dat	1193.5	24.0	1.0	0.01	#NUM!	2.2E-07
YMR318C	YMR318C	S288C_ctrl_hmg124h_11.JPG.dat	1209.8	33.6	1.0	0.02	#NUM!	3.3E-06
YMR319C	YMR319C	S288C_ctrl_hmg124h_11.JPG.dat	1259.0	20.2	1.0	0.01	#NUM!	2.0E-07
YMR320W	YMR320W	S288C_ctrl_hmg124h_11.JPG.dat	1278.5	32.2	1.0	0.02	#NUM!	3.2E-06
YMR322C	YMR322C	S288C_ctrl_hmg124h_11.JPG.dat	1301.0	39.7	1.0	0.02	#NUM!	3.8E-06
YMR326C	YMR326C	S288C_ctrl_hmg124h_11.JPG.dat	1320.8	23.5	1.1	0.00	#NUM!	3.5E-06
YNL001W	YNL001W	S288C_ctrl_hmg124h_11.JPG.dat	1234.5	24.3	1.0	0.03	#NUM!	4.2E-06
YNL003C	YNL003C	S288C_ctrl_hmg124h_11.JPG.dat	1502.0	72.4	1.0	0.03	#NUM!	6.4E-06

YNL004W	YNL004W	S288C_ctrl_hmg124h_11.JPG.dat	1300.8	101.4	0.9	0.02	#NUM!	1.6E-04
YNL008C	YNL008C	S288C_ctrl_hmg124h_11.JPG.dat	1334.3	64.5	1.0	0.03	#NUM!	5.2E-06
YNL009W	YNL009W	S288C_ctrl_hmg124h_11.JPG.dat	1323.8	99.8	1.0	0.02	#NUM!	9.6E-05
YNL010W	YNL010W	S288C_ctrl_hmg124h_11.JPG.dat	1331.8	66.7	1.0	0.01	#NUM!	3.1E-05
YNL012W	YNL012W	S288C_ctrl_hmg124h_11.JPG.dat	1371.0	83.2	1.1	0.04	#NUM!	1.1E-05
YNL013C	YNL013C	S288C_ctrl_hmg124h_11.JPG.dat	1338.3	73.0	1.0	0.03	#NUM!	8.8E-06
YNL015W	YNL015W	S288C_ctrl_hmg124h_11.JPG.dat	1366.3	96.3	1.0	0.02	#NUM!	1.9E-04
YNL016W	YNL016W	S288C_ctrl_hmg124h_11.JPG.dat	1277.5	33.2	1.0	0.05	#NUM!	2.7E-05
YNL020C	YNL020C	S288C_ctrl_hmg124h_11.JPG.dat	1189.3	92.1	0.9	0.09	#NUM!	2.3E-04
YNL021W	YNL021W	S288C_ctrl_hmg124h_11.JPG.dat	1198.3	40.0	1.0	0.02	#NUM!	1.0E-04
YNL022C	YNL022C	S288C_ctrl_hmg124h_11.JPG.dat	1367.5	111.4	1.1	0.08	#NUM!	1.3E-04
YNL023C	YNL023C	S288C_ctrl_hmg124h_11.JPG.dat	1354.0	13.1	1.0	0.00	#NUM!	2.9E-07
YNL024C	YNL024C	S288C_ctrl_hmg124h_11.JPG.dat	1281.8	27.4	1.0	0.01	#NUM!	4.9E-07
YNL027W	YNL027W	S288C_ctrl_hmg124h_11.JPG.dat	1290.0	39.6	1.0	0.00	#NUM!	6.8E-06
YNL028W	YNL028W	S288C_ctrl_hmg124h_11.JPG.dat	1328.3	22.9	1.1	0.02	#NUM!	3.1E-06
YNL029C	YNL029C	S288C_ctrl_hmg124h_11.JPG.dat	1297.8	14.6	1.0	0.01	#NUM!	4.9E-07
YNL030W	YNL030W	S288C_ctrl_hmg124h_11.JPG.dat	1246.5	60.8	1.0	0.05	#NUM!	3.2E-05
YNL031C	YNL031C	S288C_ctrl_hmg124h_11.JPG.dat	1269.0	14.4	1.0	0.02	#NUM!	1.0E-06
YNL032W	YNL032W	S288C_ctrl_hmg124h_11.JPG.dat	1257.3	30.2	1.0	0.03	#NUM!	5.5E-06
YNL034W	YNL034W	S288C_ctrl_hmg124h_11.JPG.dat	1173.3	37.0	1.0	0.03	#NUM!	6.9E-06
YNL035C	YNL035C	S288C_ctrl_hmg124h_11.JPG.dat	1093.5	61.5	0.9	0.05	#NUM!	5.3E-05
YNL037C	YNL037C	S288C_ctrl_hmg124h_11.JPG.dat	1280.0	109.6	1.0	0.00	#NUM!	6.8E-06
YNL040W	YNL040W	S288C_ctrl_hmg124h_11.JPG.dat	1418.5	88.2	1.1	0.07	#NUM!	7.4E-05
YNL041C	YNL041C	S288C_ctrl_hmg124h_11.JPG.dat	1373.5	17.1	1.0	0.00	#NUM!	3.2E-06
YNL043C	YNL043C	S288C_ctrl_hmg124h_11.JPG.dat	1367.5	20.4	1.0	0.01	#NUM!	6.8E-07
YNL044W	YNL044W	S288C_ctrl_hmg124h_11.JPG.dat	1350.0	30.9	1.0	0.02	#NUM!	3.5E-06
YNL045W	YNL045W	S288C_ctrl_hmg124h_11.JPG.dat	1314.5	31.8	1.0	0.00	#NUM!	4.3E-06
YNL046W	YNL046W	S288C_ctrl_hmg124h_11.JPG.dat	1278.5	30.6	1.0	0.01	#NUM!	1.8E-05
YNL049C	YNL049C	S288C_ctrl_hmg124h_11.JPG.dat	1277.3	23.6	1.0	0.02	#NUM!	1.5E-06
YNL050C	YNL050C	S288C_ctrl_hmg124h_11.JPG.dat	1274.5	30.4	1.0	0.00	#NUM!	4.9E-06
YNL051W	YNL051W	S288C_ctrl_hmg124h_11.JPG.dat	1283.8	8.9	1.0	0.00	#NUM!	1.6E-07
YNL052W	YNL052W	S288C_ctrl_hmg124h_11.JPG.dat	1237.3	27.7	1.0	0.02	#NUM!	1.8E-06
YNL054W	YNL054W	S288C_ctrl_hmg124h_11.JPG.dat	1263.5	31.2	1.0	0.02	#NUM!	1.9E-06
YNL056W	YNL056W	S288C_ctrl_hmg124h_11.JPG.dat	1329.8	24.2	1.0	0.01	#NUM!	2.4E-07
YNL057W	YNL057W	S288C_ctrl_hmg124h_11.JPG.dat	1358.8	66.7	1.0	0.04	#NUM!	2.3E-05
YNL058C	YNL058C	S288C_ctrl_hmg124h_11.JPG.dat	1333.8	29.8	1.0	0.01	#NUM!	2.7E-07
YNL063W	YNL063W	S288C_ctrl_hmg124h_11.JPG.dat	1341.3	53.9	1.0	0.03	#NUM!	5.5E-06
YNL064C	YNL064C	S288C_ctrl_hmg124h_11.JPG.dat	1298.5	34.0	1.0	0.03	#NUM!	6.0E-06
YNL065W	YNL065W	S288C_ctrl_hmg124h_11.JPG.dat	1264.0	11.4	1.0	0.00	#NUM!	6.9E-06
YNL066W	YNL066W	S288C_ctrl_hmg124h_11.JPG.dat	1340.8	42.5	1.1	0.03	#NUM!	6.7E-06
YNL067W	YNL067W	S288C_ctrl_hmg124h_11.JPG.dat	1221.0	67.5	1.0	0.05	#NUM!	4.5E-05
YNL068C	YNL068C	S288C_ctrl_hmg124h_11.JPG.dat	1273.0	28.5	1.0	0.03	#NUM!	9.6E-06
YNL069C	YNL069C	S288C_ctrl_hmg124h_11.JPG.dat	1327.8	94.8	1.0	0.07	#NUM!	9.1E-05
YNL070W	YNL070W	S288C_ctrl_hmg124h_11.JPG.dat	1326.3	32.1	1.0	0.01	#NUM!	3.0E-05
YNL071W	YNL071W	S288C_ctrl_hmg124h_11.JPG.dat	1274.8	77.3	1.0	0.06	#NUM!	5.7E-05
YNL072W	YNL072W	S288C_ctrl_hmg124h_11.JPG.dat	1232.8	50.4	1.0	0.04	#NUM!	1.5E-05
YNL074C	YNL074C	S288C_ctrl_hmg124h_11.JPG.dat	1239.0	34.6	1.0	0.03	#NUM!	5.7E-06
YNL076W	YNL076W	S288C_ctrl_hmg124h_11.JPG.dat	1264.5	35.0	1.0	0.02	#NUM!	2.3E-06
YNL077W	YNL077W	S288C_ctrl_hmg124h_11.JPG.dat	1134.3	59.0	0.9	0.01	#NUM!	4.8E-05
YNL078W	YNL078W	S288C_ctrl_hmg124h_11.JPG.dat	1206.3	27.4	1.0	0.02	#NUM!	2.1E-06
YNL079C	YNL079C	S288C_ctrl_hmg124h_11.JPG.dat	1166.5	17.3	0.9	0.02	#NUM!	1.5E-06
YNL080C	YNL080C	S288C_ctrl_hmg124h_11.JPG.dat	1012.5	68.9	0.9	0.02	#NUM!	1.7E-04
YNL081C	YNL081C	S288C_ctrl_hmg124h_11.JPG.dat	0.0	0.0	0.0	0.00	#NUM!	
YNL082W	YNL082W	S288C_ctrl_hmg124h_11.JPG.dat	1369.3	19.2	1.0	0.02	#NUM!	2.2E-06
YNL083W	YNL083W	S288C_ctrl_hmg124h_11.JPG.dat	1175.3	391.0	1.0	0.05	#NUM!	6.5E-04
YNL085W	YNL085W	S288C_ctrl_hmg124h_11.JPG.dat	1284.8	55.7	1.0	0.04	#NUM!	1.7E-05
YNL087W	YNL087W	S288C_ctrl_hmg124h_11.JPG.dat	1238.0	24.3	1.0	0.03	#NUM!	6.5E-06
YNL089C	YNL089C	S288C_ctrl_hmg124h_11.JPG.dat	1164.0	16.7	1.0	0.01	#NUM!	5.8E-07
YNL090W	YNL090W	S288C_ctrl_hmg124h_11.JPG.dat	1190.5	28.0	1.0	0.02	#NUM!	2.9E-06
YNL091W	YNL091W	S288C_ctrl_hmg124h_11.JPG.dat	1154.8	66.2	1.0	0.05	#NUM!	4.6E-05
YNL092W	YNL092W	S288C_ctrl_hmg124h_11.JPG.dat	1285.8	21.9	1.1	0.02	#NUM!	9.3E-07
YNL093W	YNL093W	S288C_ctrl_hmg124h_11.JPG.dat	1246.0	16.6	1.0	0.01	#NUM!	5.8E-07
YNL094W	YNL094W	S288C_ctrl_hmg124h_11.JPG.dat	1228.5	60.1	1.0	0.05	#NUM!	2.7E-05
YNL095C	YNL095C	S288C_ctrl_hmg124h_11.JPG.dat	1262.5	19.5	1.1	0.01	#NUM!	4.4E-07
YNL097C	YNL097C	S288C_ctrl_hmg124h_11.JPG.dat	1294.3	75.2	1.0	0.05	#NUM!	5.2E-05
YNL098C	YNL098C	S288C_ctrl_hmg124h_11.JPG.dat	1254.5	34.3	0.9	0.03	#NUM!	7.8E-06
YNL099C	YNL099C	S288C_ctrl_hmg124h_11.JPG.dat	1221.3	32.5	0.9	0.01	#NUM!	6.0E-05
YNL100W	YNL100W	S288C_ctrl_hmg124h_11.JPG.dat	1200.8	34.1	1.0	0.03	#NUM!	1.0E-05
YNL101W	YNL101W	S288C_ctrl_hmg124h_11.JPG.dat	1205.3	9.3	1.0	0.01	#NUM!	3.9E-07
YNL104C	YNL104C	S288C_ctrl_hmg124h_11.JPG.dat	1201.3	37.9	1.0	0.02	#NUM!	2.6E-06

YNL105W	YNL105W	S288C_ctrl_hmg124h_11.JPG.dat	1208.0	47.6	1.0	0.03	#NUM!	5.5E-06
YNL106C	YNL106C	S288C_ctrl_hmg124h_11.JPG.dat	1205.8	26.6	1.0	0.02	#NUM!	1.4E-06
YNL107W	YNL107W	S288C_ctrl_hmg124h_11.JPG.dat	1298.5	34.3	1.0	0.02	#NUM!	1.7E-06
YNL108C	YNL108C	S288C_ctrl_hmg124h_11.JPG.dat	1278.5	31.8	1.0	0.00	#NUM!	4.3E-06
YNL115C	YNL115C	S288C_ctrl_hmg124h_11.JPG.dat	1327.8	21.6	1.1	0.01	#NUM!	1.3E-07
YNL116W	YNL116W	S288C_ctrl_hmg124h_11.JPG.dat	1497.8	80.6	1.0	0.03	#NUM!	8.3E-06
YNL117W	YNL117W	S288C_ctrl_hmg124h_11.JPG.dat	1383.8	88.4	1.0	0.04	#NUM!	2.3E-05
YNL119W	YNL119W	S288C_ctrl_hmg124h_11.JPG.dat	1399.8	115.1	1.0	0.07	#NUM!	7.5E-05
YNL120C	YNL120C	S288C_ctrl_hmg124h_11.JPG.dat	1287.8	42.4	0.9	0.01	#NUM!	1.6E-07
YNL121C	YNL121C	S288C_ctrl_hmg124h_11.JPG.dat	1217.3	62.6	0.9	0.05	#NUM!	3.9E-05
YNL122C	YNL122C	S288C_ctrl_hmg124h_11.JPG.dat	1363.0	71.9	1.0	0.03	#NUM!	4.1E-06
YNL123W	YNL123W	S288C_ctrl_hmg124h_11.JPG.dat	1360.0	39.6	1.0	0.00	#NUM!	1.8E-08
YNL125C	YNL125C	S288C_ctrl_hmg124h_11.JPG.dat	1373.0	60.0	1.1	0.03	#NUM!	8.3E-06
YNL127W	YNL127W	S288C_ctrl_hmg124h_11.JPG.dat	1353.8	37.8	1.0	0.03	#NUM!	7.3E-06
YNL128W	YNL128W	S288C_ctrl_hmg124h_11.JPG.dat	1285.0	72.3	1.0	0.08	#NUM!	1.2E-04
YNL129W	YNL129W	S288C_ctrl_hmg124h_11.JPG.dat	1164.3	65.9	1.0	0.02	#NUM!	1.9E-04
YNL130C	YNL130C	S288C_ctrl_hmg124h_11.JPG.dat	1417.8	41.1	1.1	0.06	#NUM!	4.2E-05
YNL134C	YNL134C	S288C_ctrl_hmg124h_11.JPG.dat	1348.0	59.2	1.0	0.01	#NUM!	1.5E-05
YNL135C	YNL135C	S288C_ctrl_hmg124h_11.JPG.dat	1326.3	37.4	1.0	0.02	#NUM!	9.9E-07
YNL136W	YNL136W	S288C_ctrl_hmg124h_11.JPG.dat	1354.0	38.8	1.0	0.01	#NUM!	3.1E-05
YNL140C	YNL140C	S288C_ctrl_hmg124h_11.JPG.dat	1289.5	67.6	1.0	0.03	#NUM!	6.1E-06
YNL141W	YNL141W	S288C_ctrl_hmg124h_11.JPG.dat	1152.0	84.6	0.9	0.09	#NUM!	3.0E-04
YNL142W	YNL142W	S288C_ctrl_hmg124h_11.JPG.dat	1362.8	85.2	1.1	0.06	#NUM!	5.9E-05
YNL143C	YNL143C	S288C_ctrl_hmg124h_11.JPG.dat	1395.3	76.2	1.1	0.03	#NUM!	4.6E-06
YNL144C	YNL144C	S288C_ctrl_hmg124h_11.JPG.dat	1379.8	100.2	1.1	0.03	#NUM!	8.0E-06
YNL145W	YNL145W	S288C_ctrl_hmg124h_11.JPG.dat	1231.0	70.0	1.0	0.01	#NUM!	1.1E-05
YNL146W	YNL146W	S288C_ctrl_hmg124h_11.JPG.dat	1257.0	79.3	1.0	0.04	#NUM!	2.2E-05
YNL153C	YNL153C	S288C_ctrl_hmg124h_11.JPG.dat	1413.5	56.6	1.1	0.01	#NUM!	1.5E-05
YNL154C	YNL154C	S288C_ctrl_hmg124h_11.JPG.dat	1339.3	39.9	1.0	0.03	#NUM!	5.1E-06
YNL155W	YNL155W	S288C_ctrl_hmg124h_11.JPG.dat	1355.8	28.6	1.0	0.01	#NUM!	2.6E-05
YNL156C	YNL156C	S288C_ctrl_hmg124h_11.JPG.dat	1357.3	39.3	1.0	0.03	#NUM!	4.4E-06
YNL157W	YNL157W	S288C_ctrl_hmg124h_11.JPG.dat	1323.5	17.5	1.0	0.02	#NUM!	2.1E-06
YNL159C	YNL159C	S288C_ctrl_hmg124h_11.JPG.dat	1334.8	16.3	1.0	0.00	#NUM!	7.1E-06
YNL162W	YNL162W	S288C_ctrl_hmg124h_11.JPG.dat	1149.0	28.9	0.9	0.03	#NUM!	9.2E-06
YNL164C	YNL164C	S288C_ctrl_hmg124h_11.JPG.dat	1316.8	20.2	1.0	0.02	#NUM!	2.7E-06
YNL165W	YNL165W	S288C_ctrl_hmg124h_11.JPG.dat	1244.5	26.1	1.0	0.02	#NUM!	1.0E-06
YNL166C	YNL166C	S288C_ctrl_hmg124h_11.JPG.dat	1170.5	33.2	1.0	0.01	#NUM!	1.5E-05
YNL167C	YNL167C	S288C_ctrl_hmg124h_11.JPG.dat	1178.5	31.9	1.0	0.03	#NUM!	5.9E-06
YNL168C	YNL168C	S288C_ctrl_hmg124h_11.JPG.dat	1446.8	62.1	1.1	0.04	#NUM!	1.9E-05
YNL169C	YNL169C	S288C_ctrl_hmg124h_11.JPG.dat	1304.8	50.0	1.0	0.04	#NUM!	1.8E-05
YNL170W	YNL170W	S288C_ctrl_hmg124h_11.JPG.dat	0.0	0.0	0.0	0.00	#NUM!	
YNL171C	YNL171C	S288C_ctrl_hmg124h_11.JPG.dat	1125.5	149.8	0.9	0.11	#NUM!	6.0E-04
YNL173C	YNL173C	S288C_ctrl_hmg124h_11.JPG.dat	1325.5	27.2	1.0	0.02	#NUM!	2.3E-06
YNL175C	YNL175C	S288C_ctrl_hmg124h_11.JPG.dat	1301.0	44.5	1.0	0.04	#NUM!	1.3E-05
YNL176C	YNL176C	S288C_ctrl_hmg124h_11.JPG.dat	1318.0	31.2	1.0	0.02	#NUM!	3.8E-06
YNL179C	YNL179C	S288C_ctrl_hmg124h_11.JPG.dat	1244.0	24.8	1.0	0.02	#NUM!	2.3E-06
YNL183C	YNL183C	S288C_ctrl_hmg124h_11.JPG.dat	1277.8	49.6	1.0	0.04	#NUM!	1.6E-05
YNL187W	YNL187W	S288C_ctrl_hmg124h_11.JPG.dat	1234.3	45.8	1.0	0.04	#NUM!	1.5E-05
YNL190W	YNL190W	S288C_ctrl_hmg124h_11.JPG.dat	1261.8	47.4	1.0	0.01	#NUM!	2.0E-05
YNL191W	YNL191W	S288C_ctrl_hmg124h_11.JPG.dat	1352.5	32.3	1.0	0.02	#NUM!	2.8E-06
YNL192W	YNL192W	S288C_ctrl_hmg124h_11.JPG.dat	1338.3	45.9	1.0	0.04	#NUM!	1.2E-05
YNL193W	YNL193W	S288C_ctrl_hmg124h_11.JPG.dat	1346.8	47.8	1.0	0.04	#NUM!	1.3E-05
YNL194C	YNL194C	S288C_ctrl_hmg124h_11.JPG.dat	1298.0	12.6	1.0	0.00	#NUM!	1.8E-06
YNL195C	YNL195C	S288C_ctrl_hmg124h_11.JPG.dat	1336.8	33.5	1.0	0.03	#NUM!	4.6E-06
YNL196C	YNL196C	S288C_ctrl_hmg124h_11.JPG.dat	1249.8	49.9	1.0	0.04	#NUM!	2.0E-05
YNL197C	YNL197C	S288C_ctrl_hmg124h_11.JPG.dat	640.5	739.6	0.5	0.58	#NUM!	1.8E-01
YNL198C	YNL198C	S288C_ctrl_hmg124h_11.JPG.dat	1025.8	31.5	0.8	0.01	#NUM!	3.2E-05
YNL199C	YNL199C	S288C_ctrl_hmg124h_11.JPG.dat	1195.8	103.5	0.9	0.02	#NUM!	1.3E-04
YNL200C	YNL200C	S288C_ctrl_hmg124h_11.JPG.dat	1308.8	22.7	1.0	0.02	#NUM!	1.5E-06
YNL201C	YNL201C	S288C_ctrl_hmg124h_11.JPG.dat	1294.5	6.6	1.1	0.01	#NUM!	3.8E-07
YNL202W	YNL202W	S288C_ctrl_hmg124h_11.JPG.dat	1347.0	47.0	1.0	0.03	#NUM!	9.3E-06
YNL203C	YNL203C	S288C_ctrl_hmg124h_11.JPG.dat	1371.8	28.5	1.0	0.02	#NUM!	2.7E-06
YNL204C	YNL204C	S288C_ctrl_hmg124h_11.JPG.dat	1264.8	32.5	1.0	0.03	#NUM!	4.7E-06
YNL205C	YNL205C	S288C_ctrl_hmg124h_11.JPG.dat	1269.5	25.7	1.0	0.02	#NUM!	1.2E-06
YNL206C	YNL206C	S288C_ctrl_hmg124h_11.JPG.dat	1200.3	39.8	1.0	0.01	#NUM!	2.1E-05
YNL208W	YNL208W	S288C_ctrl_hmg124h_11.JPG.dat	1146.8	18.6	0.9	0.01	#NUM!	6.9E-07
YNL211C	YNL211C	S288C_ctrl_hmg124h_11.JPG.dat	1207.0	26.9	1.0	0.00	#NUM!	2.4E-06
YNL212W	YNL212W	S288C_ctrl_hmg124h_11.JPG.dat	1239.3	66.0	1.0	0.05	#NUM!	4.1E-05
YNL214W	YNL214W	S288C_ctrl_hmg124h_11.JPG.dat	1216.8	49.0	1.0	0.04	#NUM!	1.5E-05
YNL215W	YNL215W	S288C_ctrl_hmg124h_11.JPG.dat	1237.3	26.9	1.0	0.02	#NUM!	3.4E-06

YNL217W	YNL217W	S288C_ctrl_hmg124h_11.JPG.dat	1154.5	136.7	1.0	0.11	#NUM!	3.6E-04
YNL218W	YNL218W	S288C_ctrl_hmg124h_11.JPG.dat	1335.3	53.1	1.0	0.04	#NUM!	1.8E-05
YNL219C	YNL219C	S288C_ctrl_hmg124h_11.JPG.dat	1323.0	94.3	1.1	0.01	#NUM!	4.9E-05
YNL223W	YNL223W	S288C_ctrl_hmg124h_11.JPG.dat	1252.0	90.8	1.0	0.06	#NUM!	6.3E-05
YNL224C	YNL224C	S288C_ctrl_hmg124h_11.JPG.dat	1151.0	44.5	1.0	0.03	#NUM!	6.8E-06
YNL226W	YNL226W	S288C_ctrl_hmg124h_11.JPG.dat	1099.3	27.1	0.9	0.03	#NUM!	7.7E-06
YNL227C	YNL227C	S288C_ctrl_hmg124h_11.JPG.dat	1111.0	13.5	0.9	0.00	#NUM!	5.1E-06
YNL228W	YNL228W	S288C_ctrl_hmg124h_11.JPG.dat	948.3	21.4	0.8	0.02	#NUM!	7.6E-06
YNL229C	YNL229C	S288C_ctrl_hmg124h_11.JPG.dat	1116.0	20.3	0.9	0.00	#NUM!	4.5E-06
YNL230C	YNL230C	S288C_ctrl_hmg124h_11.JPG.dat	1223.5	26.2	1.0	0.03	#NUM!	7.8E-06
YNL231C	YNL231C	S288C_ctrl_hmg124h_11.JPG.dat	1243.3	41.1	1.0	0.03	#NUM!	5.9E-06
YNL233W	YNL233W	S288C_ctrl_hmg124h_11.JPG.dat	1237.8	89.0	1.1	0.06	#NUM!	5.8E-05
YNL234W	YNL234W	S288C_ctrl_hmg124h_11.JPG.dat	1339.3	92.5	1.0	0.05	#NUM!	4.2E-05
YNL235C	YNL235C	S288C_ctrl_hmg124h_11.JPG.dat	1333.0	54.7	1.0	0.02	#NUM!	3.2E-06
YNL236W	YNL236W	S288C_ctrl_hmg124h_11.JPG.dat	1271.5	85.8	1.0	0.05	#NUM!	2.7E-05
YNL237W	YNL237W	S288C_ctrl_hmg124h_11.JPG.dat	1237.8	4.9	1.0	0.02	#NUM!	2.3E-06
YNL238W	YNL238W	S288C_ctrl_hmg124h_11.JPG.dat	1216.8	19.0	1.0	0.00	#NUM!	2.1E-06
YNL239W	YNL239W	S288C_ctrl_hmg124h_11.JPG.dat	1220.5	44.8	1.0	0.01	#NUM!	1.5E-05
YNL241C	YNL241C	S288C_ctrl_hmg124h_11.JPG.dat	1066.5	54.5	0.9	0.05	#NUM!	3.9E-05
YNL242W	YNL242W	S288C_ctrl_hmg124h_11.JPG.dat	999.3	668.7	1.1	0.04	#NUM!	5.5E-04
YNL246W	YNL246W	S288C_ctrl_hmg124h_11.JPG.dat	1258.3	32.7	1.0	0.02	#NUM!	2.0E-06
YNL249C	YNL249C	S288C_ctrl_hmg124h_11.JPG.dat	1284.8	52.0	1.0	0.01	#NUM!	3.2E-05
YNL253W	YNL253W	S288C_ctrl_hmg124h_11.JPG.dat	1114.0	67.9	1.0	0.07	#NUM!	8.9E-05
YNL254C	YNL254C	S288C_ctrl_hmg124h_11.JPG.dat	906.5	606.5	0.9	0.01	#NUM!	5.6E-05
YNL255C	YNL255C	S288C_ctrl_hmg124h_11.JPG.dat	1256.8	83.3	1.0	0.02	#NUM!	1.2E-04
YNL257C	YNL257C	S288C_ctrl_hmg124h_11.JPG.dat	1295.0	47.4	1.0	0.00	#NUM!	6.3E-10
YNL259C	YNL259C	S288C_ctrl_hmg124h_11.JPG.dat	1264.3	36.8	1.0	0.03	#NUM!	6.3E-06
YNL265C	YNL265C	S288C_ctrl_hmg124h_11.JPG.dat	664.0	766.7	0.5	0.57	#NUM!	1.8E-01
YNL266W	YNL266W	S288C_ctrl_hmg124h_11.JPG.dat	337.8	675.5	0.0	0.00	#NUM!	
YNL268W	YNL268W	S288C_ctrl_hmg124h_11.JPG.dat	1368.0	94.2	1.1	0.03	#NUM!	7.6E-06
YNL270C	YNL270C	S288C_ctrl_hmg124h_11.JPG.dat	319.8	639.5	0.0	0.00	#NUM!	
YNL271C	YNL271C	S288C_ctrl_hmg124h_11.JPG.dat	330.5	661.0	0.0	0.00	#NUM!	
YNL273W	YNL273W	S288C_ctrl_hmg124h_11.JPG.dat	922.5	622.4	1.0	0.05	#NUM!	9.6E-04
YNL274C	YNL274C	S288C_ctrl_hmg124h_11.JPG.dat	873.5	583.6	0.9	0.02	#NUM!	1.8E-04
YNL275W	YNL275W	S288C_ctrl_hmg124h_11.JPG.dat	985.3	659.4	1.0	0.05	#NUM!	6.8E-04
YNL277W	YNL277W	S288C_ctrl_hmg124h_11.JPG.dat	1294.3	34.0	1.0	0.03	#NUM!	6.6E-06
YNL278W	YNL278W	S288C_ctrl_hmg124h_11.JPG.dat	1298.8	38.3	1.0	0.02	#NUM!	1.9E-06
YNL279W	YNL279W	S288C_ctrl_hmg124h_11.JPG.dat	1327.8	41.7	1.0	0.02	#NUM!	2.3E-06
YNL280C	YNL280C	S288C_ctrl_hmg124h_11.JPG.dat	1250.3	59.8	1.0	0.04	#NUM!	1.7E-05
YNL281W	YNL281W	S288C_ctrl_hmg124h_11.JPG.dat	1280.0	15.7	1.0	0.00	#NUM!	2.8E-06
YNL283C	YNL283C	S288C_ctrl_hmg124h_11.JPG.dat	1249.3	49.0	1.0	0.04	#NUM!	1.3E-05
YNL285W	YNL285W	S288C_ctrl_hmg124h_11.JPG.dat	1261.8	20.0	1.0	0.01	#NUM!	6.6E-07
YNL286W	YNL286W	S288C_ctrl_hmg124h_11.JPG.dat	1246.5	36.4	1.0	0.03	#NUM!	4.4E-06
YNL288W	YNL288W	S288C_ctrl_hmg124h_11.JPG.dat	1141.0	26.6	0.9	0.00	#NUM!	6.3E-06
YNL289W	YNL289W	S288C_ctrl_hmg124h_11.JPG.dat	1109.0	28.2	0.9	0.01	#NUM!	2.2E-05
YNL291C	YNL291C	S288C_ctrl_hmg124h_11.JPG.dat	1303.0	73.3	1.0	0.06	#NUM!	5.7E-05
YNL292W	YNL292W	S288C_ctrl_hmg124h_11.JPG.dat	1311.0	76.9	1.0	0.00	#NUM!	4.8E-06
YNL293W	YNL293W	S288C_ctrl_hmg124h_11.JPG.dat	1452.0	22.0	1.1	0.02	#NUM!	8.7E-07
YNL294C	YNL294C	S288C_ctrl_hmg124h_11.JPG.dat	1338.3	57.9	1.0	0.04	#NUM!	1.7E-05
YNL295W	YNL295W	S288C_ctrl_hmg124h_11.JPG.dat	1278.8	67.6	1.0	0.05	#NUM!	3.3E-05
YNL296W	YNL296W	S288C_ctrl_hmg124h_11.JPG.dat	1180.0	14.2	0.9	0.01	#NUM!	2.3E-07
YNL297C	YNL297C	S288C_ctrl_hmg124h_11.JPG.dat	1187.8	30.4	0.9	0.03	#NUM!	5.7E-06
YNL298W	YNL298W	S288C_ctrl_hmg124h_11.JPG.dat	1279.0	39.3	1.0	0.03	#NUM!	9.8E-06
YNL299W	YNL299W	S288C_ctrl_hmg124h_11.JPG.dat	1233.8	84.7	1.0	0.03	#NUM!	2.4E-04
YNL300W	YNL300W	S288C_ctrl_hmg124h_11.JPG.dat	1303.0	40.9	1.1	0.03	#NUM!	8.3E-06
YNL301C	YNL301C	S288C_ctrl_hmg124h_11.JPG.dat	1226.8	39.1	1.0	0.03	#NUM!	8.5E-06
YNL302C	YNL302C	S288C_ctrl_hmg124h_11.JPG.dat	773.0	52.0	0.6	0.03	#NUM!	4.5E-05
YNL303W	YNL303W	S288C_ctrl_hmg124h_11.JPG.dat	1330.5	15.6	1.0	0.01	#NUM!	7.2E-07
YNL304W	YNL304W	S288C_ctrl_hmg124h_11.JPG.dat	1361.5	34.7	1.0	0.03	#NUM!	4.9E-06
YNL305C	YNL305C	S288C_ctrl_hmg124h_11.JPG.dat	1306.3	33.7	1.0	0.02	#NUM!	2.5E-06
YNL307C	YNL307C	S288C_ctrl_hmg124h_11.JPG.dat	1190.5	28.6	0.9	0.00	#NUM!	2.6E-06
YNL309W	YNL309W	S288C_ctrl_hmg124h_11.JPG.dat	1279.3	57.2	1.0	0.05	#NUM!	2.6E-05
YNL311C	YNL311C	S288C_ctrl_hmg124h_11.JPG.dat	1280.0	101.5	1.0	0.08	#NUM!	1.3E-04
YNL314W	YNL314W	S288C_ctrl_hmg124h_11.JPG.dat	1276.5	53.1	1.0	0.04	#NUM!	1.9E-05
YNL315C	YNL315C	S288C_ctrl_hmg124h_11.JPG.dat	856.0	81.8	0.7	0.06	#NUM!	2.4E-04
YNL316C	YNL316C	S288C_ctrl_hmg124h_11.JPG.dat	1226.5	32.4	1.0	0.01	#NUM!	1.9E-05
YNL318C	YNL318C	S288C_ctrl_hmg124h_11.JPG.dat	1276.0	42.2	1.0	0.03	#NUM!	1.1E-05
YNL319W	YNL319W	S288C_ctrl_hmg124h_11.JPG.dat	1226.5	48.6	0.9	0.01	#NUM!	1.2E-05
YNL320W	YNL320W	S288C_ctrl_hmg124h_11.JPG.dat	1332.8	45.8	1.0	0.03	#NUM!	9.8E-06
YNL321W	YNL321W	S288C_ctrl_hmg124h_11.JPG.dat	1291.5	38.9	1.0	0.01	#NUM!	2.9E-05

YNL322C	YNL322C	S288C_ctrl_hmg124h_11.JPG.dat	1227.3	12.6	1.0	0.01	#NUM!	6.1E-07
YNL323W	YNL323W	S288C_ctrl_hmg124h_11.JPG.dat	1241.5	39.2	1.0	0.03	#NUM!	6.6E-06
YNL324W	YNL324W	S288C_ctrl_hmg124h_11.JPG.dat	1259.0	31.0	1.0	0.03	#NUM!	5.6E-06
YNL325C	YNL325C	S288C_ctrl_hmg124h_11.JPG.dat	1312.0	17.3	1.1	0.01	#NUM!	3.3E-07
YNL326C	YNL326C	S288C_ctrl_hmg124h_11.JPG.dat	1093.0	46.3	0.9	0.04	#NUM!	1.8E-05
YNL327W	YNL327W	S288C_ctrl_hmg124h_11.JPG.dat	1277.5	28.2	1.0	0.02	#NUM!	2.3E-06
YNL328C	YNL328C	S288C_ctrl_hmg124h_11.JPG.dat	1249.0	31.4	1.0	0.01	#NUM!	2.6E-05
YNL329C	YNL329C	S288C_ctrl_hmg124h_11.JPG.dat	1252.5	47.5	1.0	0.04	#NUM!	1.7E-05
YNL330C	YNL330C	S288C_ctrl_hmg124h_11.JPG.dat	1315.8	13.7	1.0	0.01	#NUM!	3.7E-07
YNL332W	YNL332W	S288C_ctrl_hmg124h_11.JPG.dat	1336.3	27.2	1.0	0.01	#NUM!	2.1E-07
YNL333W	YNL333W	S288C_ctrl_hmg124h_11.JPG.dat	1253.8	43.1	1.0	0.03	#NUM!	7.9E-06
YNL334C	YNL334C	S288C_ctrl_hmg124h_11.JPG.dat	1247.5	66.6	1.0	0.05	#NUM!	2.2E-05
YNL335W	YNL335W	S288C_ctrl_hmg124h_11.JPG.dat	1171.3	50.3	1.0	0.01	#NUM!	3.3E-05
YNL336W	YNL336W	S288C_ctrl_hmg124h_11.JPG.dat	1152.0	31.3	1.0	0.03	#NUM!	6.6E-06
YNL338W	YNL338W	S288C_ctrl_hmg124h_11.JPG.dat	1202.0	31.4	1.0	0.03	#NUM!	5.9E-06
YNL339C	YNL339C	S288C_ctrl_hmg124h_11.JPG.dat	1177.3	52.9	1.0	0.04	#NUM!	1.3E-05
YNR001C	YNR001C	S288C_ctrl_hmg124h_11.JPG.dat	1192.3	23.9	1.0	0.02	#NUM!	1.7E-06
YNR002C	YNR002C	S288C_ctrl_hmg124h_11.JPG.dat	1251.3	16.5	1.0	0.01	#NUM!	4.5E-07
YNR004W	YNR004W	S288C_ctrl_hmg124h_11.JPG.dat	1162.5	15.9	1.0	0.01	#NUM!	1.3E-07
YNR005C	YNR005C	S288C_ctrl_hmg124h_11.JPG.dat	1457.5	38.8	1.1	0.03	#NUM!	6.5E-06
YNR006W	YNR006W	S288C_ctrl_hmg124h_11.JPG.dat	601.0	322.8	0.4	0.24	#NUM!	3.3E-02
YNR007C	YNR007C	S288C_ctrl_hmg124h_11.JPG.dat	1210.0	48.3	0.9	0.04	#NUM!	2.0E-05
YNR008W	YNR008W	S288C_ctrl_hmg124h_11.JPG.dat	1228.0	27.4	1.0	0.02	#NUM!	4.5E-06
YNR009W	YNR009W	S288C_ctrl_hmg124h_11.JPG.dat	1181.0	35.9	1.0	0.02	#NUM!	3.2E-06
YNR010W	YNR010W	S288C_ctrl_hmg124h_11.JPG.dat	1234.0	24.1	1.0	0.01	#NUM!	7.2E-08
YNR012W	YNR012W	S288C_ctrl_hmg124h_11.JPG.dat	1221.8	96.0	1.0	0.02	#NUM!	8.8E-05
YNR013C	YNR013C	S288C_ctrl_hmg124h_11.JPG.dat	1252.3	17.9	1.0	0.01	#NUM!	4.3E-07
YNR014W	YNR014W	S288C_ctrl_hmg124h_11.JPG.dat	1319.8	38.7	1.1	0.04	#NUM!	1.3E-05
YNR015W	YNR015W	S288C_ctrl_hmg124h_11.JPG.dat	1251.0	37.8	1.0	0.04	#NUM!	1.3E-05
YNR018W	YNR018W	S288C_ctrl_hmg124h_11.JPG.dat	1205.0	44.6	1.0	0.04	#NUM!	1.5E-05
YOR202W	YOR202W	S288C_ctrl_hmg124h_11.JPG.dat	1343.7	152.4	1.0	0.08	#NUM!	3.2E-303
1	1	S288C_ctrl_hmg124h_8.JPG.dat	1298.0	169.4	1.2	0.04	#NUM!	4.6E-04
2	2	S288C_ctrl_hmg124h_8.JPG.dat	1097.0	72.7	0.9	0.02	#NUM!	1.4E-04
3	3	S288C_ctrl_hmg124h_8.JPG.dat	1048.8	45.8	0.9	0.06	#NUM!	6.4E-05
4	4	S288C_ctrl_hmg124h_8.JPG.dat	1098.8	51.3	1.0	0.05	#NUM!	2.7E-05
YJR100C	YJR100C	S288C_ctrl_hmg124h_8.JPG.dat	1238.5	29.6	1.0	0.02	#NUM!	2.4E-06
YJR103W	YJR103W	S288C_ctrl_hmg124h_8.JPG.dat	1195.3	31.6	1.0	0.03	#NUM!	5.5E-06
YJR106W	YJR106W	S288C_ctrl_hmg124h_8.JPG.dat	1221.5	25.7	1.0	0.02	#NUM!	3.1E-06
YJR107W	YJR107W	S288C_ctrl_hmg124h_8.JPG.dat	1205.0	11.2	1.0	0.01	#NUM!	5.4E-07
YJR108W	YJR108W	S288C_ctrl_hmg124h_8.JPG.dat	1167.5	28.3	1.0	0.03	#NUM!	6.3E-06
YJR109C	YJR109C	S288C_ctrl_hmg124h_8.JPG.dat	0.0	0.0	0.0	0.00	#NUM!	
YJR110W	YJR110W	S288C_ctrl_hmg124h_8.JPG.dat	1180.8	9.3	1.0	0.01	#NUM!	1.1E-07
YJR111C	YJR111C	S288C_ctrl_hmg124h_8.JPG.dat	1199.0	2.4	1.0	0.00	#NUM!	3.0E-08
YJR115W	YJR115W	S288C_ctrl_hmg124h_8.JPG.dat	1245.5	13.5	1.0	0.00	#NUM!	2.0E-06
YJR116W	YJR116W	S288C_ctrl_hmg124h_8.JPG.dat	1237.8	34.6	1.0	0.03	#NUM!	6.6E-06
YJR117W	YJR117W	S288C_ctrl_hmg124h_8.JPG.dat	1152.8	16.5	1.0	0.03	#NUM!	5.4E-06
YJR118C	YJR118C	S288C_ctrl_hmg124h_8.JPG.dat	1084.8	51.3	0.9	0.01	#NUM!	5.8E-05
YJR119C	YJR119C	S288C_ctrl_hmg124h_8.JPG.dat	1226.8	12.8	1.0	0.01	#NUM!	3.0E-07
YJR120W	YJR120W	S288C_ctrl_hmg124h_8.JPG.dat	1146.3	33.2	1.0	0.03	#NUM!	7.2E-06
YJR121W	YJR121W	S288C_ctrl_hmg124h_8.JPG.dat	1177.3	35.1	1.0	0.03	#NUM!	5.8E-06
YJR124C	YJR124C	S288C_ctrl_hmg124h_8.JPG.dat	1142.5	41.0	1.0	0.04	#NUM!	1.6E-05
YJR125C	YJR125C	S288C_ctrl_hmg124h_8.JPG.dat	1115.5	46.4	1.0	0.04	#NUM!	1.7E-05
YJR126C	YJR126C	S288C_ctrl_hmg124h_8.JPG.dat	1108.8	27.8	1.0	0.02	#NUM!	2.0E-06
YJR127C	YJR127C	S288C_ctrl_hmg124h_8.JPG.dat	1139.3	26.6	1.0	0.03	#NUM!	4.6E-06
YJR128W	YJR128W	S288C_ctrl_hmg124h_8.JPG.dat	1227.0	15.3	1.0	0.01	#NUM!	3.4E-07
YJR129C	YJR129C	S288C_ctrl_hmg124h_8.JPG.dat	1180.0	45.1	1.0	0.04	#NUM!	1.4E-05
YJR130C	YJR130C	S288C_ctrl_hmg124h_8.JPG.dat	1193.0	24.1	1.0	0.00	#NUM!	5.0E-07
YJR131W	YJR131W	S288C_ctrl_hmg124h_8.JPG.dat	1192.8	31.3	1.0	0.03	#NUM!	5.4E-06
YJR133W	YJR133W	S288C_ctrl_hmg124h_8.JPG.dat	1191.3	34.9	1.0	0.00	#NUM!	6.3E-06
YJR134C	YJR134C	S288C_ctrl_hmg124h_8.JPG.dat	1158.8	28.2	1.0	0.03	#NUM!	4.8E-06
YJR135C	YJR135C	S288C_ctrl_hmg124h_8.JPG.dat	1160.8	22.5	1.0	0.02	#NUM!	1.4E-06
YJR137C	YJR137C	S288C_ctrl_hmg124h_8.JPG.dat	1152.0	21.3	1.0	0.02	#NUM!	1.6E-06
YJR139C	YJR139C	S288C_ctrl_hmg124h_8.JPG.dat	1019.8	72.6	0.9	0.01	#NUM!	4.8E-05
YJR140C	YJR140C	S288C_ctrl_hmg124h_8.JPG.dat	1087.0	22.8	1.0	0.02	#NUM!	1.7E-06
YJR142W	YJR142W	S288C_ctrl_hmg124h_8.JPG.dat	1159.3	24.7	1.0	0.02	#NUM!	1.5E-06
YJR145C	YJR145C	S288C_ctrl_hmg124h_8.JPG.dat	1136.5	10.5	1.0	0.01	#NUM!	7.2E-07
YJR146W	YJR146W	S288C_ctrl_hmg124h_8.JPG.dat	1264.8	46.0	1.0	0.04	#NUM!	1.3E-05
YJR147W	YJR147W	S288C_ctrl_hmg124h_8.JPG.dat	1216.0	40.0	1.0	0.01	#NUM!	2.4E-05
YJR148W	YJR148W	S288C_ctrl_hmg124h_8.JPG.dat	1203.0	11.9	1.0	0.01	#NUM!	1.4E-07
YJR149W	YJR149W	S288C_ctrl_hmg124h_8.JPG.dat	1168.8	18.5	1.0	0.01	#NUM!	7.5E-07

YJR150C	YJR150C	S288C_ctrl_hmg124h_8.JPG.dat	1165.3	19.8	1.0	0.02	#NUM!	2.1E-06
YJR152W	YJR152W	S288C_ctrl_hmg124h_8.JPG.dat	1138.5	14.7	1.0	0.01	#NUM!	8.5E-07
YJR153W	YJR153W	S288C_ctrl_hmg124h_8.JPG.dat	1114.5	26.5	1.0	0.00	#NUM!	5.6E-06
YJR154W	YJR154W	S288C_ctrl_hmg124h_8.JPG.dat	1134.8	25.7	1.0	0.02	#NUM!	3.5E-06
YKL001C	YKL001C	S288C_ctrl_hmg124h_8.JPG.dat	1137.3	61.1	1.0	0.01	#NUM!	6.0E-05
YKL005C	YKL005C	S288C_ctrl_hmg124h_8.JPG.dat	1134.0	16.9	1.0	0.01	#NUM!	5.8E-07
YKL006W	YKL006W	S288C_ctrl_hmg124h_8.JPG.dat	643.8	441.5	0.7	0.11	#NUM!	7.2E-03
YKL007W	YKL007W	S288C_ctrl_hmg124h_8.JPG.dat	1225.5	36.9	1.0	0.03	#NUM!	8.3E-06
YKL008C	YKL008C	S288C_ctrl_hmg124h_8.JPG.dat	1235.3	44.8	1.1	0.01	#NUM!	3.8E-05
YKL009W	YKL009W	S288C_ctrl_hmg124h_8.JPG.dat	1146.3	19.8	0.9	0.02	#NUM!	1.3E-06
YKL010C	YKL010C	S288C_ctrl_hmg124h_8.JPG.dat	1237.0	8.9	1.0	0.00	#NUM!	1.6E-06
YKL015W	YKL015W	S288C_ctrl_hmg124h_8.JPG.dat	1174.5	15.9	1.0	0.01	#NUM!	3.4E-07
YKL017C	YKL017C	S288C_ctrl_hmg124h_8.JPG.dat	1174.3	10.6	1.0	0.00	#NUM!	3.3E-06
YKL020C	YKL020C	S288C_ctrl_hmg124h_8.JPG.dat	1114.8	43.5	1.0	0.04	#NUM!	1.6E-05
YKL023W	YKL023W	S288C_ctrl_hmg124h_8.JPG.dat	1090.0	25.8	1.0	0.02	#NUM!	3.3E-06
YKL025C	YKL025C	S288C_ctrl_hmg124h_8.JPG.dat	1195.0	14.0	1.0	0.00	#NUM!	3.8E-06
YKL026C	YKL026C	S288C_ctrl_hmg124h_8.JPG.dat	1161.3	27.6	1.0	0.02	#NUM!	3.3E-06
YKL027W	YKL027W	S288C_ctrl_hmg124h_8.JPG.dat	1146.0	19.6	1.0	0.02	#NUM!	1.7E-06
YKL029C	YKL029C	S288C_ctrl_hmg124h_8.JPG.dat	1163.3	16.9	1.0	0.01	#NUM!	4.5E-07
YKL030W	YKL030W	S288C_ctrl_hmg124h_8.JPG.dat	1241.8	13.3	1.0	0.01	#NUM!	6.8E-07
YKL031W	YKL031W	S288C_ctrl_hmg124h_8.JPG.dat	1273.5	27.1	1.0	0.02	#NUM!	1.8E-06
YKL032C	YKL032C	S288C_ctrl_hmg124h_8.JPG.dat	1148.0	10.4	0.9	0.00	#NUM!	1.5E-08
YKL033W-/	YKL033W-A	S288C_ctrl_hmg124h_8.JPG.dat	1236.0	24.0	1.0	0.00	#NUM!	4.3E-06
YKL034W	YKL034W	S288C_ctrl_hmg124h_8.JPG.dat	1175.8	43.7	1.0	0.01	#NUM!	2.2E-05
YKL037W	YKL037W	S288C_ctrl_hmg124h_8.JPG.dat	1088.5	30.9	0.9	0.02	#NUM!	2.2E-06
YKL038W	YKL038W	S288C_ctrl_hmg124h_8.JPG.dat	1145.5	23.0	1.0	0.02	#NUM!	1.2E-06
YKL039W	YKL039W	S288C_ctrl_hmg124h_8.JPG.dat	1180.5	3.9	1.0	0.01	#NUM!	2.8E-07
YKL040C	YKL040C	S288C_ctrl_hmg124h_8.JPG.dat	1199.5	16.9	1.0	0.02	#NUM!	1.5E-06
YKL041W	YKL041W	S288C_ctrl_hmg124h_8.JPG.dat	600.0	693.1	0.5	0.58	#NUM!	1.8E-01
YKL043W	YKL043W	S288C_ctrl_hmg124h_8.JPG.dat	1187.5	19.7	1.0	0.01	#NUM!	7.4E-07
YKL044W	YKL044W	S288C_ctrl_hmg124h_8.JPG.dat	1232.0	32.6	1.0	0.03	#NUM!	9.0E-06
YKL046C	YKL046C	S288C_ctrl_hmg124h_8.JPG.dat	1251.5	77.3	0.9	0.08	#NUM!	1.7E-04
YKL047W	YKL047W	S288C_ctrl_hmg124h_8.JPG.dat	1338.8	56.0	1.0	0.02	#NUM!	9.7E-05
YKL048C	YKL048C	S288C_ctrl_hmg124h_8.JPG.dat	1409.0	46.8	1.1	0.04	#NUM!	1.1E-05
YKL050C	YKL050C	S288C_ctrl_hmg124h_8.JPG.dat	1371.8	51.4	1.1	0.02	#NUM!	1.4E-06
YKL051W	YKL051W	S288C_ctrl_hmg124h_8.JPG.dat	1315.5	24.7	1.0	0.01	#NUM!	6.2E-07
YKL053C-A	YKL053C-A	S288C_ctrl_hmg124h_8.JPG.dat	1293.8	53.5	1.0	0.02	#NUM!	1.0E-06
YKL053W	YKL053W	S288C_ctrl_hmg124h_8.JPG.dat	1370.5	57.7	1.0	0.03	#NUM!	4.9E-06
YKL055C	YKL055C	S288C_ctrl_hmg124h_8.JPG.dat	1168.3	69.9	0.9	0.06	#NUM!	8.4E-05
YKL056C	YKL056C	S288C_ctrl_hmg124h_8.JPG.dat	1240.3	39.3	1.0	0.02	#NUM!	3.0E-06
YKL061W	YKL061W	S288C_ctrl_hmg124h_8.JPG.dat	1258.5	35.5	1.0	0.00	#NUM!	2.4E-07
YKL062W	YKL062W	S288C_ctrl_hmg124h_8.JPG.dat	1160.8	28.6	0.9	0.04	#NUM!	2.3E-05
YKL063C	YKL063C	S288C_ctrl_hmg124h_8.JPG.dat	1202.3	27.1	1.0	0.00	#NUM!	2.7E-08
YKL064W	YKL064W	S288C_ctrl_hmg124h_8.JPG.dat	1188.3	26.0	1.0	0.01	#NUM!	1.3E-05
YKL065C	YKL065C	S288C_ctrl_hmg124h_8.JPG.dat	1159.3	22.8	1.0	0.02	#NUM!	3.2E-06
YKL066W	YKL066W	S288C_ctrl_hmg124h_8.JPG.dat	1195.8	20.0	1.0	0.00	#NUM!	6.9E-06
YKL067W	YKL067W	S288C_ctrl_hmg124h_8.JPG.dat	1176.0	8.3	1.0	0.00	#NUM!	5.5E-07
YKL068W	YKL068W	S288C_ctrl_hmg124h_8.JPG.dat	1226.0	40.9	1.0	0.03	#NUM!	9.9E-06
YKL069W	YKL069W	S288C_ctrl_hmg124h_8.JPG.dat	940.8	94.6	0.8	0.01	#NUM!	3.1E-05
YKL070W	YKL070W	S288C_ctrl_hmg124h_8.JPG.dat	1210.3	21.9	1.0	0.01	#NUM!	7.2E-07
YKL071W	YKL071W	S288C_ctrl_hmg124h_8.JPG.dat	1240.3	27.9	1.0	0.01	#NUM!	1.3E-05
YKL072W	YKL072W	S288C_ctrl_hmg124h_8.JPG.dat	1228.3	21.1	1.0	0.02	#NUM!	1.6E-06
YKL073W	YKL073W	S288C_ctrl_hmg124h_8.JPG.dat	1283.8	32.0	1.1	0.03	#NUM!	4.3E-06
YKL074C	YKL074C	S288C_ctrl_hmg124h_8.JPG.dat	1119.0	38.1	1.0	0.03	#NUM!	7.1E-06
YKL075C	YKL075C	S288C_ctrl_hmg124h_8.JPG.dat	1142.5	13.0	1.0	0.01	#NUM!	5.2E-07
YKL076C	YKL076C	S288C_ctrl_hmg124h_8.JPG.dat	1126.8	15.0	1.0	0.02	#NUM!	1.4E-06
YKL077W	YKL077W	S288C_ctrl_hmg124h_8.JPG.dat	1177.0	25.5	1.0	0.02	#NUM!	2.1E-06
YKL079W	YKL079W	S288C_ctrl_hmg124h_8.JPG.dat	1197.5	16.2	1.0	0.01	#NUM!	5.6E-07
YKL081W	YKL081W	S288C_ctrl_hmg124h_8.JPG.dat	755.0	32.4	0.7	0.03	#NUM!	1.7E-05
YKL084W	YKL084W	S288C_ctrl_hmg124h_8.JPG.dat	1124.0	42.8	1.0	0.03	#NUM!	1.1E-05
YKL085W	YKL085W	S288C_ctrl_hmg124h_8.JPG.dat	1139.8	20.1	1.0	0.02	#NUM!	3.2E-06
YKL086W	YKL086W	S288C_ctrl_hmg124h_8.JPG.dat	1212.8	45.9	1.0	0.04	#NUM!	1.2E-05
YKL090W	YKL090W	S288C_ctrl_hmg124h_8.JPG.dat	1202.3	39.7	1.0	0.01	#NUM!	3.7E-05
YKL091C	YKL091C	S288C_ctrl_hmg124h_8.JPG.dat	1189.5	42.9	1.0	0.04	#NUM!	1.2E-05
YKL092C	YKL092C	S288C_ctrl_hmg124h_8.JPG.dat	1227.0	26.2	1.1	0.01	#NUM!	9.3E-06
YKL093W	YKL093W	S288C_ctrl_hmg124h_8.JPG.dat	1152.5	12.8	1.0	0.01	#NUM!	6.0E-07
YKL094W	YKL094W	S288C_ctrl_hmg124h_8.JPG.dat	1229.0	16.8	1.0	0.00	#NUM!	3.0E-06
YKL096W	YKL096W	S288C_ctrl_hmg124h_8.JPG.dat	1145.5	3.4	1.0	0.01	#NUM!	4.0E-08
YKL096W-/	YKL096W-A	S288C_ctrl_hmg124h_8.JPG.dat	1166.8	11.9	1.0	0.00	#NUM!	6.8E-06
YKL097C	YKL097C	S288C_ctrl_hmg124h_8.JPG.dat	1147.3	17.8	1.0	0.01	#NUM!	8.8E-07

YKL098W	YKL098W	S288C_ctrl_hmg124h_8.JPG.dat	1103.8	19.5	1.0	0.00	#NUM!	2.6E-07
YKL100C	YKL100C	S288C_ctrl_hmg124h_8.JPG.dat	1123.8	14.9	1.0	0.01	#NUM!	7.7E-07
YKL101W	YKL101W	S288C_ctrl_hmg124h_8.JPG.dat	1211.5	48.4	1.0	0.01	#NUM!	2.1E-05
YKL102C	YKL102C	S288C_ctrl_hmg124h_8.JPG.dat	1227.0	24.9	1.0	0.02	#NUM!	1.2E-06
YKL103C	YKL103C	S288C_ctrl_hmg124h_8.JPG.dat	1235.8	12.2	1.0	0.01	#NUM!	3.1E-07
YKL105C	YKL105C	S288C_ctrl_hmg124h_8.JPG.dat	1219.8	45.3	1.1	0.05	#NUM!	3.1E-05
YKL106W	YKL106W	S288C_ctrl_hmg124h_8.JPG.dat	1180.3	30.9	1.0	0.02	#NUM!	4.2E-06
YKL107W	YKL107W	S288C_ctrl_hmg124h_8.JPG.dat	1156.0	9.8	1.0	0.01	#NUM!	3.3E-07
YKL109W	YKL109W	S288C_ctrl_hmg124h_8.JPG.dat	1085.8	36.8	1.0	0.04	#NUM!	1.4E-05
YKL110C	YKL110C	S288C_ctrl_hmg124h_8.JPG.dat	1008.0	26.0	0.9	0.02	#NUM!	4.8E-06
YKL113C	YKL113C	S288C_ctrl_hmg124h_8.JPG.dat	1072.8	22.1	1.0	0.02	#NUM!	2.5E-06
YKL114C	YKL114C	S288C_ctrl_hmg124h_8.JPG.dat	1076.0	28.1	1.0	0.03	#NUM!	5.9E-06
YKL115C	YKL115C	S288C_ctrl_hmg124h_8.JPG.dat	1125.0	27.8	1.0	0.02	#NUM!	2.7E-06
YKL116C	YKL116C	S288C_ctrl_hmg124h_8.JPG.dat	1140.5	13.2	1.0	0.01	#NUM!	4.0E-07
YKL117W	YKL117W	S288C_ctrl_hmg124h_8.JPG.dat	1183.3	33.6	1.0	0.03	#NUM!	7.1E-06
YKL120W	YKL120W	S288C_ctrl_hmg124h_8.JPG.dat	1227.3	30.4	1.0	0.02	#NUM!	2.5E-06
YKL121W	YKL121W	S288C_ctrl_hmg124h_8.JPG.dat	1196.0	24.0	1.0	0.04	#NUM!	1.1E-05
YKL123W	YKL123W	S288C_ctrl_hmg124h_8.JPG.dat	1219.0	18.5	1.0	0.02	#NUM!	9.3E-07
YKL124W	YKL124W	S288C_ctrl_hmg124h_8.JPG.dat	1192.0	13.3	1.0	0.00	#NUM!	1.4E-06
YKL127W	YKL127W	S288C_ctrl_hmg124h_8.JPG.dat	1146.5	21.9	1.0	0.02	#NUM!	1.7E-06
YKL128C	YKL128C	S288C_ctrl_hmg124h_8.JPG.dat	1123.0	17.9	1.0	0.01	#NUM!	7.6E-07
YKL129C	YKL129C	S288C_ctrl_hmg124h_8.JPG.dat	1099.3	38.2	1.0	0.03	#NUM!	1.0E-05
YKL130C	YKL130C	S288C_ctrl_hmg124h_8.JPG.dat	1142.3	20.6	1.0	0.02	#NUM!	1.3E-06
YKL131W	YKL131W	S288C_ctrl_hmg124h_8.JPG.dat	1134.3	15.0	1.0	0.01	#NUM!	5.1E-07
YKL132C	YKL132C	S288C_ctrl_hmg124h_8.JPG.dat	1173.8	13.7	1.0	0.01	#NUM!	3.9E-07
YKL133C	YKL133C	S288C_ctrl_hmg124h_8.JPG.dat	1179.8	23.3	1.0	0.02	#NUM!	2.4E-06
YKL136W	YKL136W	S288C_ctrl_hmg124h_8.JPG.dat	1141.0	11.5	1.0	0.02	#NUM!	1.0E-06
YKL137W	YKL137W	S288C_ctrl_hmg124h_8.JPG.dat	1220.5	31.8	1.0	0.01	#NUM!	1.5E-05
YKL139W	YKL139W	S288C_ctrl_hmg124h_8.JPG.dat	1224.8	7.4	1.0	0.01	#NUM!	3.7E-07
YKL140W	YKL140W	S288C_ctrl_hmg124h_8.JPG.dat	1235.5	22.2	1.0	0.02	#NUM!	2.5E-06
YKL142W	YKL142W	S288C_ctrl_hmg124h_8.JPG.dat	1249.3	29.4	1.0	0.02	#NUM!	2.0E-06
YKL146W	YKL146W	S288C_ctrl_hmg124h_8.JPG.dat	1145.5	14.8	1.0	0.01	#NUM!	3.2E-07
YKL147C	YKL147C	S288C_ctrl_hmg124h_8.JPG.dat	1152.3	17.7	1.0	0.02	#NUM!	1.5E-06
YKL148C	YKL148C	S288C_ctrl_hmg124h_8.JPG.dat	1172.3	46.1	1.0	0.01	#NUM!	1.8E-05
YKL149C	YKL149C	S288C_ctrl_hmg124h_8.JPG.dat	1171.0	24.7	1.0	0.02	#NUM!	1.6E-06
YKL150W	YKL150W	S288C_ctrl_hmg124h_8.JPG.dat	1216.0	26.7	1.0	0.02	#NUM!	1.1E-06
YKL151C	YKL151C	S288C_ctrl_hmg124h_8.JPG.dat	1188.0	38.3	1.0	0.04	#NUM!	1.4E-05
YKL156W	YKL156W	S288C_ctrl_hmg124h_8.JPG.dat	1120.3	21.1	0.9	0.02	#NUM!	1.1E-06
YKL157W	YKL157W	S288C_ctrl_hmg124h_8.JPG.dat	1230.0	71.6	1.0	0.02	#NUM!	1.7E-04
YKL158W	YKL158W	S288C_ctrl_hmg124h_8.JPG.dat	1221.0	145.5	0.9	0.13	#NUM!	7.7E-04
YKL159C	YKL159C	S288C_ctrl_hmg124h_8.JPG.dat	1358.5	31.5	1.0	0.01	#NUM!	3.6E-08
YKL160W	YKL160W	S288C_ctrl_hmg124h_8.JPG.dat	1321.0	67.3	1.0	0.05	#NUM!	2.4E-05
YKL161C	YKL161C	S288C_ctrl_hmg124h_8.JPG.dat	1358.5	64.8	1.1	0.04	#NUM!	1.3E-05
YKL162C	YKL162C	S288C_ctrl_hmg124h_8.JPG.dat	1335.5	36.7	1.0	0.01	#NUM!	1.3E-07
YKL163W	YKL163W	S288C_ctrl_hmg124h_8.JPG.dat	1307.8	53.1	1.0	0.03	#NUM!	5.3E-06
YKL164C	YKL164C	S288C_ctrl_hmg124h_8.JPG.dat	1304.0	26.4	1.0	0.03	#NUM!	7.6E-06
YKL166C	YKL166C	S288C_ctrl_hmg124h_8.JPG.dat	1265.5	33.8	1.0	0.04	#NUM!	2.6E-05
YKL167C	YKL167C	S288C_ctrl_hmg124h_8.JPG.dat	1245.3	39.8	1.0	0.00	#NUM!	4.7E-06
YKL168C	YKL168C	S288C_ctrl_hmg124h_8.JPG.dat	1252.8	40.1	1.0	0.02	#NUM!	1.2E-06
YKL171W	YKL171W	S288C_ctrl_hmg124h_8.JPG.dat	1261.3	8.3	1.0	0.02	#NUM!	1.5E-06
YKL174C	YKL174C	S288C_ctrl_hmg124h_8.JPG.dat	1250.0	66.8	1.0	0.01	#NUM!	2.3E-05
YKL175W	YKL175W	S288C_ctrl_hmg124h_8.JPG.dat	1164.3	19.2	0.9	0.00	#NUM!	8.1E-06
YKL176C	YKL176C	S288C_ctrl_hmg124h_8.JPG.dat	1144.8	37.6	0.9	0.02	#NUM!	1.6E-06
YKL177W	YKL177W	S288C_ctrl_hmg124h_8.JPG.dat	1251.8	38.6	1.0	0.03	#NUM!	5.8E-06
YKL178C	YKL178C	S288C_ctrl_hmg124h_8.JPG.dat	1314.3	15.2	1.0	0.02	#NUM!	2.6E-06
YKL179C	YKL179C	S288C_ctrl_hmg124h_8.JPG.dat	1331.0	57.0	1.1	0.02	#NUM!	2.6E-06
YKL183W	YKL183W	S288C_ctrl_hmg124h_8.JPG.dat	1295.8	60.5	1.0	0.03	#NUM!	8.6E-06
YKL184W	YKL184W	S288C_ctrl_hmg124h_8.JPG.dat	455.8	14.1	0.4	0.01	#NUM!	1.9E-05
YKL185W	YKL185W	S288C_ctrl_hmg124h_8.JPG.dat	1299.3	68.5	1.0	0.04	#NUM!	1.1E-05
YKL187C	YKL187C	S288C_ctrl_hmg124h_8.JPG.dat	1214.3	34.6	1.0	0.04	#NUM!	2.3E-05
YKL188C	YKL188C	S288C_ctrl_hmg124h_8.JPG.dat	1213.5	14.2	1.0	0.01	#NUM!	3.6E-07
YKL190W	YKL190W	S288C_ctrl_hmg124h_8.JPG.dat	1190.3	14.9	1.0	0.01	#NUM!	6.0E-07
YKL191W	YKL191W	S288C_ctrl_hmg124h_8.JPG.dat	1115.8	15.7	1.0	0.01	#NUM!	2.8E-07
YKL197C	YKL197C	S288C_ctrl_hmg124h_8.JPG.dat	1137.0	11.9	1.0	0.01	#NUM!	3.3E-07
YKL198C	YKL198C	S288C_ctrl_hmg124h_8.JPG.dat	1179.0	22.4	1.0	0.01	#NUM!	2.6E-05
YKL199C	YKL199C	S288C_ctrl_hmg124h_8.JPG.dat	1189.0	26.4	1.0	0.02	#NUM!	1.2E-06
YKL200C	YKL200C	S288C_ctrl_hmg124h_8.JPG.dat	1236.0	24.8	1.1	0.00	#NUM!	1.5E-06
YKL201C	YKL201C	S288C_ctrl_hmg124h_8.JPG.dat	1189.5	28.6	1.0	0.01	#NUM!	1.7E-05
YKL202W	YKL202W	S288C_ctrl_hmg124h_8.JPG.dat	1242.8	25.7	1.0	0.02	#NUM!	1.7E-06
YKL204W	YKL204W	S288C_ctrl_hmg124h_8.JPG.dat	605.5	699.4	0.5	0.59	#NUM!	1.8E-01

YKL205W	YKL205W	S288C_ctrl_hmg124h_8.JPG.dat	1219.0	11.9	1.0	0.03	#NUM!	4.5E-06
YKL206C	YKL206C	S288C_ctrl_hmg124h_8.JPG.dat	1221.0	15.6	1.0	0.01	#NUM!	5.6E-07
YKL207W	YKL207W	S288C_ctrl_hmg124h_8.JPG.dat	1194.3	10.6	1.0	0.01	#NUM!	2.5E-07
YKL208W	YKL208W	S288C_ctrl_hmg124h_8.JPG.dat	1142.5	20.8	1.0	0.02	#NUM!	1.6E-06
YKL211C	YKL211C	S288C_ctrl_hmg124h_8.JPG.dat	1155.5	40.7	1.0	0.03	#NUM!	1.0E-05
YKL212W	YKL212W	S288C_ctrl_hmg124h_8.JPG.dat	0.0	0.0	0.0	0.00	#NUM!	
YKL213C	YKL213C	S288C_ctrl_hmg124h_8.JPG.dat	1126.5	15.6	1.0	0.01	#NUM!	9.8E-06
YKL214C	YKL214C	S288C_ctrl_hmg124h_8.JPG.dat	1100.5	23.2	1.0	0.02	#NUM!	4.3E-06
YKL215C	YKL215C	S288C_ctrl_hmg124h_8.JPG.dat	1147.8	20.7	1.0	0.02	#NUM!	2.9E-06
YKL216W	YKL216W	S288C_ctrl_hmg124h_8.JPG.dat	1231.0	45.5	1.0	0.04	#NUM!	1.1E-05
YKL217W	YKL217W	S288C_ctrl_hmg124h_8.JPG.dat	1183.8	36.3	1.0	0.03	#NUM!	8.0E-06
YKL218C	YKL218C	S288C_ctrl_hmg124h_8.JPG.dat	1214.5	29.2	1.0	0.03	#NUM!	4.6E-06
YKL220C	YKL220C	S288C_ctrl_hmg124h_8.JPG.dat	1175.5	16.8	1.0	0.01	#NUM!	6.6E-07
YKL221W	YKL221W	S288C_ctrl_hmg124h_8.JPG.dat	1164.0	34.2	1.0	0.03	#NUM!	7.1E-06
YKL222C	YKL222C	S288C_ctrl_hmg124h_8.JPG.dat	1152.0	17.5	1.0	0.01	#NUM!	7.5E-07
YKR001C	YKR001C	S288C_ctrl_hmg124h_8.JPG.dat	1111.8	26.3	1.0	0.02	#NUM!	3.3E-06
YKR003W	YKR003W	S288C_ctrl_hmg124h_8.JPG.dat	1076.0	5.6	1.0	0.01	#NUM!	5.2E-08
YKR005C	YKR005C	S288C_ctrl_hmg124h_8.JPG.dat	1148.5	36.2	1.0	0.03	#NUM!	9.4E-06
YKR007W	YKR007W	S288C_ctrl_hmg124h_8.JPG.dat	974.3	14.8	0.9	0.01	#NUM!	7.4E-07
YKR009C	YKR009C	S288C_ctrl_hmg124h_8.JPG.dat	1138.0	20.2	1.0	0.01	#NUM!	6.9E-07
YKR010C	YKR010C	S288C_ctrl_hmg124h_8.JPG.dat	1123.3	8.7	1.0	0.00	#NUM!	1.5E-08
YKR011C	YKR011C	S288C_ctrl_hmg124h_8.JPG.dat	1187.5	13.9	1.0	0.01	#NUM!	2.1E-07
YKR012C	YKR012C	S288C_ctrl_hmg124h_8.JPG.dat	1247.3	41.0	1.1	0.01	#NUM!	7.9E-06
YKR013W	YKR013W	S288C_ctrl_hmg124h_8.JPG.dat	1187.0	23.1	1.0	0.01	#NUM!	5.9E-07
YKR014C	YKR014C	S288C_ctrl_hmg124h_8.JPG.dat	1249.3	20.6	1.0	0.00	#NUM!	2.4E-06
YKR015C	YKR015C	S288C_ctrl_hmg124h_8.JPG.dat	1180.0	33.0	1.0	0.02	#NUM!	3.5E-06
YKR016W	YKR016W	S288C_ctrl_hmg124h_8.JPG.dat	1079.3	19.6	1.0	0.01	#NUM!	9.8E-06
YKR017C	YKR017C	S288C_ctrl_hmg124h_8.JPG.dat	1105.8	14.2	1.0	0.00	#NUM!	1.1E-06
YKR018C	YKR018C	S288C_ctrl_hmg124h_8.JPG.dat	1096.8	13.3	1.0	0.01	#NUM!	9.4E-07
YKR019C	YKR019C	S288C_ctrl_hmg124h_8.JPG.dat	1041.3	24.9	0.9	0.00	#NUM!	7.6E-06
YKR020W	YKR020W	S288C_ctrl_hmg124h_8.JPG.dat	898.8	21.9	0.8	0.02	#NUM!	6.0E-06
YKR021W	YKR021W	S288C_ctrl_hmg124h_8.JPG.dat	1161.5	32.3	1.0	0.01	#NUM!	1.4E-05
YKR023W	YKR023W	S288C_ctrl_hmg124h_8.JPG.dat	1096.5	36.9	0.9	0.03	#NUM!	1.3E-05
YKR024C	YKR024C	S288C_ctrl_hmg124h_8.JPG.dat	1127.8	84.5	1.0	0.08	#NUM!	1.7E-04
YKR026C	YKR026C	S288C_ctrl_hmg124h_8.JPG.dat	1210.0	16.8	1.0	0.01	#NUM!	6.5E-07
YKR027W	YKR027W	S288C_ctrl_hmg124h_8.JPG.dat	1170.0	53.8	1.0	0.04	#NUM!	2.6E-05
YKR028W	YKR028W	S288C_ctrl_hmg124h_8.JPG.dat	1111.3	55.4	0.9	0.02	#NUM!	1.0E-04
YKR029C	YKR029C	S288C_ctrl_hmg124h_8.JPG.dat	1078.5	22.6	0.9	0.02	#NUM!	2.3E-06
YKR030W	YKR030W	S288C_ctrl_hmg124h_8.JPG.dat	1097.0	19.2	1.0	0.02	#NUM!	1.5E-06
YKR031C	YKR031C	S288C_ctrl_hmg124h_8.JPG.dat	1175.3	36.8	1.0	0.03	#NUM!	8.1E-06
YKR032W	YKR032W	S288C_ctrl_hmg124h_8.JPG.dat	1155.3	27.5	1.0	0.02	#NUM!	3.7E-06
YKR033C	YKR033C	S288C_ctrl_hmg124h_8.JPG.dat	1115.0	27.2	1.0	0.00	#NUM!	7.7E-06
YKR034W	YKR034W	S288C_ctrl_hmg124h_8.JPG.dat	1135.8	39.3	1.0	0.03	#NUM!	1.1E-05
YKR035C	YKR035C	S288C_ctrl_hmg124h_8.JPG.dat	1147.0	25.9	1.0	0.02	#NUM!	3.6E-06
YKR035W-,	YKR035W-A	S288C_ctrl_hmg124h_8.JPG.dat	1220.0	31.5	1.0	0.03	#NUM!	9.7E-06
YKR036C	YKR036C	S288C_ctrl_hmg124h_8.JPG.dat	1258.5	46.0	1.0	0.02	#NUM!	3.1E-06
YKR039W	YKR039W	S288C_ctrl_hmg124h_8.JPG.dat	1255.0	34.2	1.0	0.02	#NUM!	2.4E-06
YKR040C	YKR040C	S288C_ctrl_hmg124h_8.JPG.dat	1284.0	44.9	1.0	0.03	#NUM!	4.7E-06
YKR041W	YKR041W	S288C_ctrl_hmg124h_8.JPG.dat	1205.8	42.3	1.0	0.02	#NUM!	4.1E-06
YKR042W	YKR042W	S288C_ctrl_hmg124h_8.JPG.dat	1170.3	53.5	1.0	0.01	#NUM!	1.7E-05
YKR043C	YKR043C	S288C_ctrl_hmg124h_8.JPG.dat	1167.0	30.9	1.0	0.01	#NUM!	9.7E-07
YKR044W	YKR044W	S288C_ctrl_hmg124h_8.JPG.dat	1172.8	33.3	1.0	0.01	#NUM!	4.3E-07
YKR045C	YKR045C	S288C_ctrl_hmg124h_8.JPG.dat	1241.5	36.2	1.0	0.00	#NUM!	4.7E-06
YKR046C	YKR046C	S288C_ctrl_hmg124h_8.JPG.dat	1215.5	21.8	1.0	0.02	#NUM!	2.9E-06
YKR047W	YKR047W	S288C_ctrl_hmg124h_8.JPG.dat	1226.5	16.3	1.0	0.00	#NUM!	1.1E-06
YKR048C	YKR048C	S288C_ctrl_hmg124h_8.JPG.dat	1217.5	40.6	1.0	0.02	#NUM!	1.8E-06
YKR049C	YKR049C	S288C_ctrl_hmg124h_8.JPG.dat	1128.0	125.2	0.9	0.10	#NUM!	3.6E-04
YKR050W	YKR050W	S288C_ctrl_hmg124h_8.JPG.dat	1245.8	42.0	1.0	0.03	#NUM!	1.1E-05
YKR051W	YKR051W	S288C_ctrl_hmg124h_8.JPG.dat	1250.5	31.5	1.0	0.02	#NUM!	2.7E-06
YKR052C	YKR052C	S288C_ctrl_hmg124h_8.JPG.dat	1274.0	60.4	1.0	0.04	#NUM!	1.1E-05
YKR053C	YKR053C	S288C_ctrl_hmg124h_8.JPG.dat	1269.8	48.9	1.0	0.02	#NUM!	1.0E-06
YKR054C	YKR054C	S288C_ctrl_hmg124h_8.JPG.dat	1291.0	50.4	1.0	0.02	#NUM!	1.3E-06
YKR055W	YKR055W	S288C_ctrl_hmg124h_8.JPG.dat	1267.0	26.3	1.0	0.01	#NUM!	2.1E-05
YKR056W	YKR056W	S288C_ctrl_hmg124h_8.JPG.dat	1294.0	66.7	1.0	0.03	#NUM!	9.0E-06
YKR057W	YKR057W	S288C_ctrl_hmg124h_8.JPG.dat	1078.8	125.7	0.8	0.01	#NUM!	3.5E-05
YKR058W	YKR058W	S288C_ctrl_hmg124h_8.JPG.dat	1259.3	13.6	1.0	0.02	#NUM!	1.3E-06
YKR059W	YKR059W	S288C_ctrl_hmg124h_8.JPG.dat	1185.3	23.4	1.0	0.01	#NUM!	9.3E-06
YKR060W	YKR060W	S288C_ctrl_hmg124h_8.JPG.dat	1170.5	24.5	1.0	0.00	#NUM!	5.7E-06
YKR061W	YKR061W	S288C_ctrl_hmg124h_8.JPG.dat	1210.5	9.1	1.0	0.01	#NUM!	3.8E-07
YKR064W	YKR064W	S288C_ctrl_hmg124h_8.JPG.dat	1175.0	22.3	1.0	0.02	#NUM!	3.8E-06

YKR065C	YKR065C	S288C_ctrl_hmg124h_8.JPG.dat	1116.3	11.5	1.0	0.01	#NUM!	1.9E-07
YKR066C	YKR066C	S288C_ctrl_hmg124h_8.JPG.dat	1167.3	15.3	1.0	0.00	#NUM!	1.8E-06
YKR067W	YKR067W	S288C_ctrl_hmg124h_8.JPG.dat	1169.0	29.0	1.0	0.03	#NUM!	8.8E-06
YKR069W	YKR069W	S288C_ctrl_hmg124h_8.JPG.dat	1252.8	23.2	1.1	0.02	#NUM!	1.4E-06
YKR070W	YKR070W	S288C_ctrl_hmg124h_8.JPG.dat	1147.5	25.0	1.0	0.01	#NUM!	8.8E-06
YKR072C	YKR072C	S288C_ctrl_hmg124h_8.JPG.dat	1119.0	31.8	0.9	0.02	#NUM!	5.0E-06
YKR074W	YKR074W	S288C_ctrl_hmg124h_8.JPG.dat	1131.0	15.3	0.9	0.01	#NUM!	1.1E-05
YKR076W	YKR076W	S288C_ctrl_hmg124h_8.JPG.dat	1178.5	61.4	1.0	0.01	#NUM!	6.1E-05
YKR077W	YKR077W	S288C_ctrl_hmg124h_8.JPG.dat	1172.8	23.7	1.0	0.01	#NUM!	1.3E-05
YKR078W	YKR078W	S288C_ctrl_hmg124h_8.JPG.dat	1229.3	7.0	1.0	0.01	#NUM!	4.8E-08
YKR080W	YKR080W	S288C_ctrl_hmg124h_8.JPG.dat	1200.3	29.7	1.0	0.01	#NUM!	2.4E-05
YKR082W	YKR082W	S288C_ctrl_hmg124h_8.JPG.dat	961.0	69.9	0.8	0.06	#NUM!	1.1E-04
YKR084C	YKR084C	S288C_ctrl_hmg124h_8.JPG.dat	1120.3	45.6	1.0	0.04	#NUM!	2.1E-05
YKR087C	YKR087C	S288C_ctrl_hmg124h_8.JPG.dat	1160.8	56.9	1.0	0.05	#NUM!	2.9E-05
YKR088C	YKR088C	S288C_ctrl_hmg124h_8.JPG.dat	1124.5	16.9	1.0	0.01	#NUM!	7.8E-07
YKR089C	YKR089C	S288C_ctrl_hmg124h_8.JPG.dat	1145.5	18.8	1.0	0.01	#NUM!	4.8E-07
YKR090W	YKR090W	S288C_ctrl_hmg124h_8.JPG.dat	1199.0	30.9	1.0	0.03	#NUM!	5.2E-06
YKR091W	YKR091W	S288C_ctrl_hmg124h_8.JPG.dat	894.5	592.5	1.0	0.01	#NUM!	6.8E-05
YKR092C	YKR092C	S288C_ctrl_hmg124h_8.JPG.dat	1087.3	33.6	0.9	0.03	#NUM!	8.2E-06
YKR093W	YKR093W	S288C_ctrl_hmg124h_8.JPG.dat	1112.3	16.8	1.0	0.02	#NUM!	4.5E-06
YKR094C	YKR094C	S288C_ctrl_hmg124h_8.JPG.dat	1034.8	81.6	0.9	0.07	#NUM!	1.4E-04
YKR095W	YKR095W	S288C_ctrl_hmg124h_8.JPG.dat	1142.5	29.7	1.0	0.00	#NUM!	6.0E-06
YKR096W	YKR096W	S288C_ctrl_hmg124h_8.JPG.dat	1132.5	12.6	1.0	0.01	#NUM!	1.6E-07
YKR097W	YKR097W	S288C_ctrl_hmg124h_8.JPG.dat	1130.8	10.6	1.0	0.01	#NUM!	2.3E-07
YKR098C	YKR098C	S288C_ctrl_hmg124h_8.JPG.dat	1136.3	19.1	1.0	0.02	#NUM!	2.1E-06
YKR099W	YKR099W	S288C_ctrl_hmg124h_8.JPG.dat	1049.3	27.1	1.0	0.02	#NUM!	3.8E-06
YKR100C	YKR100C	S288C_ctrl_hmg124h_8.JPG.dat	1137.0	9.6	1.0	0.01	#NUM!	3.4E-07
YKR101W	YKR101W	S288C_ctrl_hmg124h_8.JPG.dat	1124.0	21.8	1.0	0.02	#NUM!	1.5E-06
YKR102W	YKR102W	S288C_ctrl_hmg124h_8.JPG.dat	1195.5	42.0	1.0	0.01	#NUM!	3.3E-05
YKR103W	YKR103W	S288C_ctrl_hmg124h_8.JPG.dat	1185.8	31.1	1.0	0.03	#NUM!	5.0E-06
YKR105C	YKR105C	S288C_ctrl_hmg124h_8.JPG.dat	614.5	709.8	0.5	0.60	#NUM!	1.8E-01
YKR106W	YKR106W	S288C_ctrl_hmg124h_8.JPG.dat	1196.3	22.5	1.0	0.01	#NUM!	4.4E-07
YLL001W	YLL001W	S288C_ctrl_hmg124h_8.JPG.dat	1178.5	9.7	1.0	0.01	#NUM!	1.2E-07
YLL002W	YLL002W	S288C_ctrl_hmg124h_8.JPG.dat	924.3	11.8	0.8	0.00	#NUM!	5.5E-08
YLL005C	YLL005C	S288C_ctrl_hmg124h_8.JPG.dat	1103.5	44.8	1.0	0.03	#NUM!	1.0E-05
YLL006W	YLL006W	S288C_ctrl_hmg124h_8.JPG.dat	1131.8	12.0	1.0	0.02	#NUM!	1.2E-06
YLL007C	YLL007C	S288C_ctrl_hmg124h_8.JPG.dat	1032.8	19.6	0.9	0.02	#NUM!	5.2E-06
YLL009C	YLL009C	S288C_ctrl_hmg124h_8.JPG.dat	1132.8	23.1	1.0	0.02	#NUM!	1.1E-06
YLL010C	YLL010C	S288C_ctrl_hmg124h_8.JPG.dat	1170.5	24.9	1.0	0.02	#NUM!	1.2E-06
YLL012W	YLL012W	S288C_ctrl_hmg124h_8.JPG.dat	1200.0	26.4	1.0	0.01	#NUM!	2.1E-05
YLL013C	YLL013C	S288C_ctrl_hmg124h_8.JPG.dat	1149.3	20.9	1.0	0.02	#NUM!	1.9E-06
YLL014W	YLL014W	S288C_ctrl_hmg124h_8.JPG.dat	1186.5	61.4	1.0	0.01	#NUM!	4.2E-05
YLL015W	YLL015W	S288C_ctrl_hmg124h_8.JPG.dat	1218.5	11.1	1.0	0.00	#NUM!	3.3E-06
YLL016W	YLL016W	S288C_ctrl_hmg124h_8.JPG.dat	1203.0	20.4	1.0	0.01	#NUM!	6.4E-07
YLL017W	YLL017W	S288C_ctrl_hmg124h_8.JPG.dat	1193.3	30.9	1.0	0.02	#NUM!	2.5E-06
YLL019C	YLL019C	S288C_ctrl_hmg124h_8.JPG.dat	1102.0	46.4	1.0	0.04	#NUM!	2.2E-05
YLL020C	YLL020C	S288C_ctrl_hmg124h_8.JPG.dat	1141.8	24.4	1.0	0.01	#NUM!	1.8E-05
YLL021W	YLL021W	S288C_ctrl_hmg124h_8.JPG.dat	1207.0	16.2	1.1	0.01	#NUM!	6.3E-07
YLL023C	YLL023C	S288C_ctrl_hmg124h_8.JPG.dat	1155.0	22.0	1.0	0.01	#NUM!	1.2E-05
YLL024C	YLL024C	S288C_ctrl_hmg124h_8.JPG.dat	1141.0	4.2	1.0	0.00	#NUM!	2.0E-08
YLL025W	YLL025W	S288C_ctrl_hmg124h_8.JPG.dat	1169.3	18.0	1.0	0.01	#NUM!	1.1E-05
YLL026W	YLL026W	S288C_ctrl_hmg124h_8.JPG.dat	1172.5	19.2	1.0	0.00	#NUM!	2.6E-06
YLL028W	YLL028W	S288C_ctrl_hmg124h_8.JPG.dat	1257.5	61.7	1.0	0.03	#NUM!	7.6E-06
YLL029W	YLL029W	S288C_ctrl_hmg124h_8.JPG.dat	1200.5	15.7	1.0	0.01	#NUM!	1.5E-07
YLL032C	YLL032C	S288C_ctrl_hmg124h_8.JPG.dat	1299.5	44.6	1.0	0.03	#NUM!	6.2E-06
YLL038C	YLL038C	S288C_ctrl_hmg124h_8.JPG.dat	1196.3	64.7	1.0	0.05	#NUM!	2.6E-05
YLL039C	YLL039C	S288C_ctrl_hmg124h_8.JPG.dat	1206.5	7.2	1.0	0.02	#NUM!	2.3E-06
YLL040C	YLL040C	S288C_ctrl_hmg124h_8.JPG.dat	1183.3	26.4	1.0	0.01	#NUM!	7.4E-07
YLL041C	YLL041C	S288C_ctrl_hmg124h_8.JPG.dat	1170.3	61.0	1.0	0.04	#NUM!	2.6E-05
YLL042C	YLL042C	S288C_ctrl_hmg124h_8.JPG.dat	1190.8	8.5	1.0	0.02	#NUM!	2.1E-06
YLL043W	YLL043W	S288C_ctrl_hmg124h_8.JPG.dat	1249.5	37.1	1.0	0.03	#NUM!	5.3E-06
YLL044W	YLL044W	S288C_ctrl_hmg124h_8.JPG.dat	1100.0	117.7	1.0	0.02	#NUM!	1.2E-04
YLL045C	YLL045C	S288C_ctrl_hmg124h_8.JPG.dat	1141.0	15.2	1.0	0.00	#NUM!	1.9E-08
YOR202W	YOR202W	S288C_ctrl_hmg124h_8.JPG.dat	1200.6	137.9	1.0	0.09	#NUM!	1.1E-293
1	1	S288C_ctrl_hmg124h_14.JPG.dat	1675.8	130.9	1.1	0.05	#NUM!	3.2E-05
2	2	S288C_ctrl_hmg124h_14.JPG.dat	1413.8	200.4	1.0	0.12	#NUM!	5.1E-04
3	3	S288C_ctrl_hmg124h_14.JPG.dat	1365.8	148.1	1.0	0.09	#NUM!	1.9E-04
4	4	S288C_ctrl_hmg124h_14.JPG.dat	1427.8	116.2	1.0	0.07	#NUM!	7.6E-05
YAL024C	YAL024C	S288C_ctrl_hmg124h_14.JPG.dat	1354.3	26.8	1.0	0.02	#NUM!	1.6E-06
YAL046C	YAL046C	S288C_ctrl_hmg124h_14.JPG.dat	1344.0	29.4	1.0	0.00	#NUM!	7.7E-06

YAL048C	YAL048C	S288C_ctrl_hmg124h_14.JPG.dat	1444.3	16.4	1.0	0.00	#NUM!	1.1E-06
YBL103C	YBL103C	S288C_ctrl_hmg124h_14.JPG.dat	1282.5	70.8	1.0	0.04	#NUM!	1.6E-05
YBR020W	YBR020W	S288C_ctrl_hmg124h_14.JPG.dat	1393.3	38.3	1.0	0.03	#NUM!	6.3E-06
YBR061C	YBR061C	S288C_ctrl_hmg124h_14.JPG.dat	1343.8	34.4	1.0	0.01	#NUM!	1.7E-05
YBR075W	YBR075W	S288C_ctrl_hmg124h_14.JPG.dat	1371.5	52.1	1.0	0.04	#NUM!	1.3E-05
YBR078W	YBR078W	S288C_ctrl_hmg124h_14.JPG.dat	1645.0	39.8	1.1	0.03	#NUM!	4.6E-06
YBR082C	YBR082C	S288C_ctrl_hmg124h_14.JPG.dat	1294.3	33.4	1.0	0.02	#NUM!	2.9E-06
YBR085W	YBR085W	S288C_ctrl_hmg124h_14.JPG.dat	1253.8	24.3	1.0	0.02	#NUM!	1.7E-06
YBR086C	YBR086C	S288C_ctrl_hmg124h_14.JPG.dat	1258.3	29.2	1.0	0.02	#NUM!	3.4E-06
YBR095C	YBR095C	S288C_ctrl_hmg124h_14.JPG.dat	1283.0	30.8	1.0	0.03	#NUM!	4.6E-06
YBR115C	YBR115C	S288C_ctrl_hmg124h_14.JPG.dat	0.0	0.0	0.0	0.00	#NUM!	
YBR118W	YBR118W	S288C_ctrl_hmg124h_14.JPG.dat	1330.3	38.1	1.0	0.01	#NUM!	2.3E-05
YBR165W	YBR165W	S288C_ctrl_hmg124h_14.JPG.dat	1384.0	43.0	1.0	0.03	#NUM!	5.0E-06
YBR299W	YBR299W	S288C_ctrl_hmg124h_14.JPG.dat	1310.3	35.0	1.0	0.03	#NUM!	5.1E-06
YCR107W	YCR107W	S288C_ctrl_hmg124h_14.JPG.dat	1274.0	24.2	1.0	0.02	#NUM!	1.5E-06
YDL074C	YDL074C	S288C_ctrl_hmg124h_14.JPG.dat	1218.5	39.3	1.0	0.03	#NUM!	9.7E-06
YDR026C	YDR026C	S288C_ctrl_hmg124h_14.JPG.dat	1358.5	25.4	1.0	0.00	#NUM!	1.4E-07
YDR029W	YDR029W	S288C_ctrl_hmg124h_14.JPG.dat	1282.0	48.3	1.0	0.04	#NUM!	1.8E-05
YDR030C	YDR030C	S288C_ctrl_hmg124h_14.JPG.dat	1379.8	28.6	1.0	0.02	#NUM!	1.0E-06
YDR031W	YDR031W	S288C_ctrl_hmg124h_14.JPG.dat	1302.0	28.9	1.0	0.03	#NUM!	4.9E-06
YDR032C	YDR032C	S288C_ctrl_hmg124h_14.JPG.dat	1344.8	55.3	1.0	0.01	#NUM!	1.9E-05
YDR033W	YDR033W	S288C_ctrl_hmg124h_14.JPG.dat	1390.8	36.3	1.0	0.03	#NUM!	4.4E-06
YDR034C	YDR034C	S288C_ctrl_hmg124h_14.JPG.dat	308.8	617.5	0.0	0.00	#NUM!	
YDR035W	YDR035W	S288C_ctrl_hmg124h_14.JPG.dat	1331.8	44.0	1.0	0.04	#NUM!	1.2E-05
YDR036C	YDR036C	S288C_ctrl_hmg124h_14.JPG.dat	1323.3	8.0	1.0	0.01	#NUM!	4.0E-08
YDR042C	YDR042C	S288C_ctrl_hmg124h_14.JPG.dat	1398.3	31.5	1.0	0.02	#NUM!	2.4E-06
YDR043C	YDR043C	S288C_ctrl_hmg124h_14.JPG.dat	1329.3	40.0	1.0	0.01	#NUM!	8.5E-06
YDR046C	YDR046C	S288C_ctrl_hmg124h_14.JPG.dat	1338.5	67.0	1.1	0.00	#NUM!	4.7E-06
YDR242W	YDR242W	S288C_ctrl_hmg124h_14.JPG.dat	1315.0	9.6	1.0	0.01	#NUM!	1.2E-07
YDR269C	YDR269C	S288C_ctrl_hmg124h_14.JPG.dat	1296.8	23.5	1.0	0.02	#NUM!	1.5E-06
YDR271C	YDR271C	S288C_ctrl_hmg124h_14.JPG.dat	1318.5	6.6	1.0	0.00	#NUM!	3.2E-08
YDR290W	YDR290W	S288C_ctrl_hmg124h_14.JPG.dat	1384.3	37.2	1.0	0.02	#NUM!	4.6E-06
YDR326C	YDR326C	S288C_ctrl_hmg124h_14.JPG.dat	1424.5	21.0	1.0	0.01	#NUM!	5.0E-07
YDR444W	YDR444W	S288C_ctrl_hmg124h_14.JPG.dat	1364.5	42.4	1.0	0.02	#NUM!	1.1E-06
YDR461W	YDR461W	S288C_ctrl_hmg124h_14.JPG.dat	402.5	805.0	0.0	0.00	#NUM!	
YDR477W	YDR477W	S288C_ctrl_hmg124h_14.JPG.dat	1376.3	23.2	1.0	0.00	#NUM!	5.9E-06
YDR483W	YDR483W	S288C_ctrl_hmg124h_14.JPG.dat	1372.5	34.1	1.0	0.01	#NUM!	6.4E-07
YDR493W	YDR493W	S288C_ctrl_hmg124h_14.JPG.dat	1344.8	26.4	1.0	0.01	#NUM!	2.3E-07
YDR502C	YDR502C	S288C_ctrl_hmg124h_14.JPG.dat	1359.5	41.8	1.0	0.03	#NUM!	5.1E-06
YDR515W	YDR515W	S288C_ctrl_hmg124h_14.JPG.dat	1399.8	44.0	1.1	0.01	#NUM!	3.1E-05
YER089C	YER089C	S288C_ctrl_hmg124h_14.JPG.dat	1323.5	112.2	1.0	0.07	#NUM!	1.1E-04
YFL001W	YFL001W	S288C_ctrl_hmg124h_14.JPG.dat	1175.3	12.8	0.8	0.00	#NUM!	4.0E-06
YFL003C	YFL003C	S288C_ctrl_hmg124h_14.JPG.dat	1601.3	63.5	1.1	0.01	#NUM!	5.9E-05
YFL004W	YFL004W	S288C_ctrl_hmg124h_14.JPG.dat	1427.0	19.7	1.0	0.01	#NUM!	3.9E-07
YFL007W	YFL007W	S288C_ctrl_hmg124h_14.JPG.dat	1421.5	44.7	1.0	0.01	#NUM!	2.9E-05
YFL010C	YFL010C	S288C_ctrl_hmg124h_14.JPG.dat	1444.8	16.8	1.0	0.01	#NUM!	6.3E-07
YFL010W-/	YFL010W-A	S288C_ctrl_hmg124h_14.JPG.dat	1471.5	83.1	1.0	0.05	#NUM!	4.0E-05
YFL012W	YFL012W	S288C_ctrl_hmg124h_14.JPG.dat	1458.5	71.8	1.0	0.05	#NUM!	3.6E-05
YFL013C	YFL013C	S288C_ctrl_hmg124h_14.JPG.dat	1301.3	29.8	0.9	0.02	#NUM!	5.5E-06
YFL033C	YFL033C	S288C_ctrl_hmg124h_14.JPG.dat	1397.8	83.8	1.0	0.07	#NUM!	9.5E-05
YFL063W	YFL063W	S288C_ctrl_hmg124h_14.JPG.dat	1462.8	55.0	1.0	0.02	#NUM!	2.8E-06
YGL029W	YGL029W	S288C_ctrl_hmg124h_14.JPG.dat	1034.3	690.4	1.0	0.03	#NUM!	2.7E-04
YGL037C	YGL037C	S288C_ctrl_hmg124h_14.JPG.dat	1419.0	61.2	1.0	0.05	#NUM!	2.5E-05
YGL042C	YGL042C	S288C_ctrl_hmg124h_14.JPG.dat	1415.8	79.0	1.0	0.06	#NUM!	4.8E-05
YGL049C	YGL049C	S288C_ctrl_hmg124h_14.JPG.dat	1315.5	54.6	0.9	0.03	#NUM!	8.5E-06
YGL063W	YGL063W	S288C_ctrl_hmg124h_14.JPG.dat	1254.0	61.6	1.0	0.05	#NUM!	3.6E-05
YGL071W	YGL071W	S288C_ctrl_hmg124h_14.JPG.dat	1339.0	65.4	1.0	0.05	#NUM!	2.7E-05
YGL094C	YGL094C	S288C_ctrl_hmg124h_14.JPG.dat	1448.0	50.7	1.0	0.04	#NUM!	1.1E-05
YGL096W	YGL096W	S288C_ctrl_hmg124h_14.JPG.dat	1359.3	36.7	1.0	0.03	#NUM!	5.1E-06
YGR055W	YGR055W	S288C_ctrl_hmg124h_14.JPG.dat	1429.0	11.2	1.0	0.01	#NUM!	5.4E-08
YGR061C	YGR061C	S288C_ctrl_hmg124h_14.JPG.dat	1499.0	36.7	1.1	0.02	#NUM!	3.5E-06
YGR067C	YGR067C	S288C_ctrl_hmg124h_14.JPG.dat	1478.3	13.0	1.0	0.01	#NUM!	2.4E-07
YGR068C	YGR068C	S288C_ctrl_hmg124h_14.JPG.dat	1514.0	41.8	1.1	0.01	#NUM!	2.3E-05
YGR122C-/	YGR122C-A	S288C_ctrl_hmg124h_14.JPG.dat	1175.0	47.4	0.9	0.00	#NUM!	4.4E-07
YGR122W	YGR122W	S288C_ctrl_hmg124h_14.JPG.dat	1453.3	56.4	1.0	0.04	#NUM!	2.3E-05
YGR254W	YGR254W	S288C_ctrl_hmg124h_14.JPG.dat	1272.8	59.7	1.0	0.04	#NUM!	2.4E-05
YGR255C	YGR255C	S288C_ctrl_hmg124h_14.JPG.dat	1345.0	61.9	1.0	0.04	#NUM!	2.4E-05
YGR271W	YGR271W	S288C_ctrl_hmg124h_14.JPG.dat	1189.8	38.5	1.0	0.03	#NUM!	7.4E-06
YGR272C	YGR272C	S288C_ctrl_hmg124h_14.JPG.dat	1296.8	40.3	1.0	0.02	#NUM!	3.6E-06
YGR273C	YGR273C	S288C_ctrl_hmg124h_14.JPG.dat	1373.8	36.8	1.0	0.03	#NUM!	4.3E-06

YGR276C	YGR276C	S288C_ctrl_hmg124h_14.JPG.dat	1386.8	8.3	1.0	0.01	#NUM!	4.1E-08
YGR289C	YGR289C	S288C_ctrl_hmg124h_14.JPG.dat	1370.3	34.9	1.0	0.00	#NUM!	8.9E-08
YGR291C	YGR291C	S288C_ctrl_hmg124h_14.JPG.dat	1485.8	29.5	1.1	0.02	#NUM!	1.5E-06
YGR292W	YGR292W	S288C_ctrl_hmg124h_14.JPG.dat	1386.8	18.8	1.0	0.01	#NUM!	6.6E-07
YHR008C	YHR008C	S288C_ctrl_hmg124h_14.JPG.dat	1339.0	66.8	1.0	0.05	#NUM!	3.7E-05
YIL085C	YIL085C	S288C_ctrl_hmg124h_14.JPG.dat	1344.0	26.3	1.0	0.01	#NUM!	1.2E-05
YIL094C	YIL094C	S288C_ctrl_hmg124h_14.JPG.dat	688.8	796.9	0.5	0.58	#NUM!	1.8E-01
YIL102C	YIL102C	S288C_ctrl_hmg124h_14.JPG.dat	1346.5	26.1	1.0	0.01	#NUM!	6.8E-07
YIL111W	YIL111W	S288C_ctrl_hmg124h_14.JPG.dat	1358.0	37.4	1.0	0.02	#NUM!	2.9E-06
YIL122W	YIL122W	S288C_ctrl_hmg124h_14.JPG.dat	1298.3	55.1	1.0	0.04	#NUM!	1.4E-05
YIL125W	YIL125W	S288C_ctrl_hmg124h_14.JPG.dat	350.8	701.5	0.0	0.00	#NUM!	
YIL131C	YIL131C	S288C_ctrl_hmg124h_14.JPG.dat	1291.5	20.3	1.0	0.02	#NUM!	2.0E-06
YIL136W	YIL136W	S288C_ctrl_hmg124h_14.JPG.dat	1318.0	31.7	1.0	0.02	#NUM!	2.6E-06
YIL151C	YIL151C	S288C_ctrl_hmg124h_14.JPG.dat	1352.8	15.2	1.0	0.01	#NUM!	1.7E-07
YIL158W	YIL158W	S288C_ctrl_hmg124h_14.JPG.dat	1346.8	28.8	1.0	0.01	#NUM!	9.3E-06
YIR004W	YIR004W	S288C_ctrl_hmg124h_14.JPG.dat	1356.5	13.5	1.0	0.01	#NUM!	2.1E-07
YJL029C	YJL029C	S288C_ctrl_hmg124h_14.JPG.dat	1163.8	153.5	0.8	0.01	#NUM!	6.4E-05
YJL079C	YJL079C	S288C_ctrl_hmg124h_14.JPG.dat	1379.0	9.3	1.0	0.01	#NUM!	5.7E-08
YJL082W	YJL082W	S288C_ctrl_hmg124h_14.JPG.dat	1345.3	29.4	1.0	0.01	#NUM!	2.2E-05
YJL092W	YJL092W	S288C_ctrl_hmg124h_14.JPG.dat	1464.5	68.6	0.9	0.01	#NUM!	3.2E-05
YJL103C	YJL103C	S288C_ctrl_hmg124h_14.JPG.dat	1294.3	57.7	1.0	0.04	#NUM!	2.2E-05
YJL106W	YJL106W	S288C_ctrl_hmg124h_14.JPG.dat	1499.5	61.1	1.0	0.04	#NUM!	2.0E-05
YJL107C	YJL107C	S288C_ctrl_hmg124h_14.JPG.dat	1574.5	69.5	1.0	0.03	#NUM!	5.2E-06
YJL117W	YJL117W	S288C_ctrl_hmg124h_14.JPG.dat	1475.8	41.2	1.0	0.02	#NUM!	4.5E-06
YJL119C	YJL119C	S288C_ctrl_hmg124h_14.JPG.dat	1538.0	59.4	1.0	0.02	#NUM!	3.9E-06
YJL120W	YJL120W	S288C_ctrl_hmg124h_14.JPG.dat	1447.3	93.1	1.0	0.04	#NUM!	2.5E-05
YJL177W	YJL177W	S288C_ctrl_hmg124h_14.JPG.dat	1355.0	38.3	1.0	0.03	#NUM!	9.6E-06
YJR034W	YJR034W	S288C_ctrl_hmg124h_14.JPG.dat	1463.8	95.1	1.0	0.04	#NUM!	2.3E-05
YJR039W	YJR039W	S288C_ctrl_hmg124h_14.JPG.dat	1402.3	19.5	1.1	0.01	#NUM!	6.8E-07
YJR044C	YJR044C	S288C_ctrl_hmg124h_14.JPG.dat	1297.0	20.8	1.0	0.01	#NUM!	6.3E-07
YJR066W	YJR066W	S288C_ctrl_hmg124h_14.JPG.dat	1217.3	22.5	0.9	0.02	#NUM!	1.9E-06
YKL087C	YKL087C	S288C_ctrl_hmg124h_14.JPG.dat	1319.5	18.4	1.0	0.01	#NUM!	2.7E-07
YKR104W	YKR104W	S288C_ctrl_hmg124h_14.JPG.dat	1397.0	28.3	1.0	0.02	#NUM!	9.9E-07
YLR227C	YLR227C	S288C_ctrl_hmg124h_14.JPG.dat	1384.3	23.6	1.0	0.02	#NUM!	9.1E-07
YLR238W	YLR238W	S288C_ctrl_hmg124h_14.JPG.dat	1306.8	39.3	1.0	0.03	#NUM!	4.9E-06
YLR308W	YLR308W	S288C_ctrl_hmg124h_14.JPG.dat	1382.3	41.9	1.0	0.01	#NUM!	6.6E-07
YLR455W	YLR455W	S288C_ctrl_hmg124h_14.JPG.dat	1347.3	58.8	1.0	0.04	#NUM!	2.0E-05
YML050W	YML050W	S288C_ctrl_hmg124h_14.JPG.dat	1351.0	96.5	1.0	0.06	#NUM!	7.0E-05
YML111W	YML111W	S288C_ctrl_hmg124h_14.JPG.dat	1417.8	26.1	1.1	0.02	#NUM!	1.3E-06
YML112W	YML112W	S288C_ctrl_hmg124h_14.JPG.dat	1300.5	50.3	1.0	0.04	#NUM!	1.3E-05
YML129C	YML129C	S288C_ctrl_hmg124h_14.JPG.dat	694.3	802.5	0.5	0.63	#NUM!	1.8E-01
YMR074C	YMR074C	S288C_ctrl_hmg124h_14.JPG.dat	1206.3	37.9	0.9	0.03	#NUM!	7.6E-06
YMR118C	YMR118C	S288C_ctrl_hmg124h_14.JPG.dat	1312.0	23.2	1.0	0.02	#NUM!	4.0E-06
YMR119W	YMR119W	S288C_ctrl_hmg124h_14.JPG.dat	1341.0	26.1	1.0	0.02	#NUM!	1.3E-06
YMR165C	YMR165C	S288C_ctrl_hmg124h_14.JPG.dat	1305.5	24.8	1.0	0.02	#NUM!	1.7E-06
YMR194C-.	YMR194C-A	S288C_ctrl_hmg124h_14.JPG.dat	1350.8	5.7	1.0	0.00	#NUM!	3.1E-07
YNL011C	YNL011C	S288C_ctrl_hmg124h_14.JPG.dat	1320.0	109.3	1.0	0.03	#NUM!	2.2E-04
YNL014W	YNL014W	S288C_ctrl_hmg124h_14.JPG.dat	1389.3	30.9	1.0	0.02	#NUM!	2.0E-06
YNL042W	YNL042W	S288C_ctrl_hmg124h_14.JPG.dat	1421.8	26.2	1.0	0.02	#NUM!	2.2E-06
YNL047C	YNL047C	S288C_ctrl_hmg124h_14.JPG.dat	1366.8	11.7	1.0	0.00	#NUM!	1.9E-06
YNL053W	YNL053W	S288C_ctrl_hmg124h_14.JPG.dat	1252.0	19.5	0.9	0.01	#NUM!	1.0E-05
YNL086W	YNL086W	S288C_ctrl_hmg124h_14.JPG.dat	1315.0	10.6	1.0	0.00	#NUM!	3.4E-07
YNL096C	YNL096C	S288C_ctrl_hmg124h_14.JPG.dat	1199.8	15.6	0.9	0.01	#NUM!	7.8E-07
YNL109W	YNL109W	S288C_ctrl_hmg124h_14.JPG.dat	1294.8	27.8	1.0	0.01	#NUM!	1.3E-05
YNL111C	YNL111C	S288C_ctrl_hmg124h_14.JPG.dat	746.0	861.4	0.5	0.63	#NUM!	1.8E-01
YNL147W	YNL147W	S288C_ctrl_hmg124h_14.JPG.dat	1244.8	42.8	0.9	0.03	#NUM!	8.5E-06
YNL180C	YNL180C	S288C_ctrl_hmg124h_14.JPG.dat	1398.8	3.6	1.0	0.00	#NUM!	9.6E-09
YNL284C	YNL284C	S288C_ctrl_hmg124h_14.JPG.dat	1373.5	19.7	1.0	0.02	#NUM!	1.5E-06
YNR033W	YNR033W	S288C_ctrl_hmg124h_14.JPG.dat	1374.8	23.0	1.0	0.02	#NUM!	1.5E-06
YNR041C	YNR041C	S288C_ctrl_hmg124h_14.JPG.dat	1481.3	25.9	1.0	0.01	#NUM!	4.1E-05
YNR044W	YNR044W	S288C_ctrl_hmg124h_14.JPG.dat	1260.3	16.0	1.0	0.00	#NUM!	1.2E-06
YOL073C	YOL073C	S288C_ctrl_hmg124h_14.JPG.dat	1270.3	18.3	1.0	0.02	#NUM!	1.3E-06
YOL086C	YOL086C	S288C_ctrl_hmg124h_14.JPG.dat	1430.5	48.7	1.0	0.03	#NUM!	9.5E-06
YOL153C	YOL153C	S288C_ctrl_hmg124h_14.JPG.dat	1224.3	23.9	1.0	0.02	#NUM!	2.6E-06
YOR054C	YOR054C	S288C_ctrl_hmg124h_14.JPG.dat	1279.5	21.0	0.9	0.02	#NUM!	2.5E-06
YOR125C	YOR125C	S288C_ctrl_hmg124h_14.JPG.dat	1254.8	29.3	1.0	0.03	#NUM!	4.8E-06
YOR128C	YOR128C	S288C_ctrl_hmg124h_14.JPG.dat	1539.0	43.2	1.1	0.03	#NUM!	5.1E-06
YOR155C	YOR155C	S288C_ctrl_hmg124h_14.JPG.dat	1326.0	13.8	1.0	0.00	#NUM!	6.1E-06
YOR158W	YOR158W	S288C_ctrl_hmg124h_14.JPG.dat	1313.5	58.7	1.0	0.03	#NUM!	6.4E-06
YOR179C	YOR179C	S288C_ctrl_hmg124h_14.JPG.dat	1200.5	24.7	0.9	0.02	#NUM!	1.9E-06

YOR180C	YOR180C	S288C_ctrl_hmg124h_14.JPG.dat	1350.5	30.4	1.0	0.02	#NUM!	4.1E-06
YOR202W	YOR202W	S288C_ctrl_hmg124h_14.JPG.dat	1452.6	177.7	1.0	0.08	#NUM!	4.05164326
YOR220W	YOR220W	S288C_ctrl_hmg124h_14.JPG.dat	1409.5	20.6	1.0	0.00	#NUM!	5.0E-06
YOR240W	YOR240W	S288C_ctrl_hmg124h_14.JPG.dat	1377.3	15.7	1.0	0.01	#NUM!	3.2E-07
YOR248W	YOR248W	S288C_ctrl_hmg124h_14.JPG.dat	1404.8	29.2	1.0	0.02	#NUM!	1.9E-06
YOR300W	YOR300W	S288C_ctrl_hmg124h_14.JPG.dat	1531.5	60.2	1.1	0.04	#NUM!	1.5E-05
YOR306C	YOR306C	S288C_ctrl_hmg124h_14.JPG.dat	1367.8	36.7	1.0	0.03	#NUM!	4.8E-06
YOR309C	YOR309C	S288C_ctrl_hmg124h_14.JPG.dat	1277.0	58.2	1.0	0.05	#NUM!	3.1E-05
YOR325W	YOR325W	S288C_ctrl_hmg124h_14.JPG.dat	1226.8	56.2	1.0	0.01	#NUM!	3.6E-05
YOR333C	YOR333C	S288C_ctrl_hmg124h_14.JPG.dat	1197.8	28.0	1.0	0.02	#NUM!	3.1E-06
YOR345C	YOR345C	S288C_ctrl_hmg124h_14.JPG.dat	1263.3	22.8	1.0	0.02	#NUM!	1.7E-06
YOR366W	YOR366W	S288C_ctrl_hmg124h_14.JPG.dat	1295.3	15.7	1.0	0.01	#NUM!	4.5E-07
YOR379C	YOR379C	S288C_ctrl_hmg124h_14.JPG.dat	1315.0	15.6	1.0	0.01	#NUM!	3.1E-07
YPL015C	YPL015C	S288C_ctrl_hmg124h_14.JPG.dat	1386.8	38.0	1.0	0.02	#NUM!	3.2E-06
YPL035C	YPL035C	S288C_ctrl_hmg124h_14.JPG.dat	1338.0	61.6	1.0	0.01	#NUM!	6.7E-05
YPL072W	YPL072W	S288C_ctrl_hmg124h_14.JPG.dat	1271.3	23.4	1.0	0.00	#NUM!	2.0E-06
YPL158C	YPL158C	S288C_ctrl_hmg124h_14.JPG.dat	1225.3	123.9	0.8	0.03	#NUM!	4.4E-04
YPL261C	YPL261C	S288C_ctrl_hmg124h_14.JPG.dat	1263.0	26.2	1.0	0.02	#NUM!	2.6E-06
YPL262W	YPL262W	S288C_ctrl_hmg124h_14.JPG.dat	1262.8	57.8	0.9	0.04	#NUM!	1.8E-05
YPL263C	YPL263C	S288C_ctrl_hmg124h_14.JPG.dat	1391.3	36.7	1.0	0.00	#NUM!	6.9E-06
YPL264C	YPL264C	S288C_ctrl_hmg124h_14.JPG.dat	1415.3	23.6	1.0	0.02	#NUM!	1.5E-06
YPL265W	YPL265W	S288C_ctrl_hmg124h_14.JPG.dat	1393.3	36.5	1.0	0.03	#NUM!	5.5E-06
YPL267W	YPL267W	S288C_ctrl_hmg124h_14.JPG.dat	1513.0	22.1	1.1	0.02	#NUM!	9.8E-07
YPL269W	YPL269W	S288C_ctrl_hmg124h_14.JPG.dat	1446.3	27.5	1.0	0.02	#NUM!	2.5E-06
YPL270W	YPL270W	S288C_ctrl_hmg124h_14.JPG.dat	1422.3	20.1	1.0	0.02	#NUM!	1.1E-06
YPL272C	YPL272C	S288C_ctrl_hmg124h_14.JPG.dat	1425.5	56.9	1.0	0.04	#NUM!	1.7E-05
YPL273W	YPL273W	S288C_ctrl_hmg124h_14.JPG.dat	1324.5	56.5	0.9	0.04	#NUM!	1.7E-05
YPL274W	YPL274W	S288C_ctrl_hmg124h_14.JPG.dat	1351.3	36.6	1.0	0.03	#NUM!	7.6E-06
YPR001W	YPR001W	S288C_ctrl_hmg124h_14.JPG.dat	1286.3	71.1	1.0	0.05	#NUM!	3.2E-05
YPR002W	YPR002W	S288C_ctrl_hmg124h_14.JPG.dat	1426.8	35.1	1.0	0.02	#NUM!	2.0E-06
YPR003C	YPR003C	S288C_ctrl_hmg124h_14.JPG.dat	1409.5	19.7	1.0	0.01	#NUM!	9.1E-07
YPR004C	YPR004C	S288C_ctrl_hmg124h_14.JPG.dat	1412.0	25.7	1.0	0.02	#NUM!	1.5E-06
YPR005C	YPR005C	S288C_ctrl_hmg124h_14.JPG.dat	1383.0	22.0	1.0	0.01	#NUM!	6.6E-07
YPR006C	YPR006C	S288C_ctrl_hmg124h_14.JPG.dat	1389.8	17.4	1.0	0.01	#NUM!	4.6E-07
YPR007C	YPR007C	S288C_ctrl_hmg124h_14.JPG.dat	1370.8	43.1	1.0	0.03	#NUM!	7.9E-06
YPR008W	YPR008W	S288C_ctrl_hmg124h_14.JPG.dat	1380.0	17.8	1.0	0.01	#NUM!	2.4E-07
YPR009W	YPR009W	S288C_ctrl_hmg124h_14.JPG.dat	1382.5	40.0	1.0	0.03	#NUM!	9.0E-06
YPR011C	YPR011C	S288C_ctrl_hmg124h_14.JPG.dat	1358.5	21.0	1.0	0.01	#NUM!	7.0E-07
YPR012W	YPR012W	S288C_ctrl_hmg124h_14.JPG.dat	1365.5	18.2	1.0	0.02	#NUM!	1.5E-06
YPR013C	YPR013C	S288C_ctrl_hmg124h_14.JPG.dat	1437.0	26.2	1.1	0.04	#NUM!	1.0E-05
YPR014C	YPR014C	S288C_ctrl_hmg124h_14.JPG.dat	1302.8	13.5	1.0	0.01	#NUM!	6.7E-07
YPR015C	YPR015C	S288C_ctrl_hmg124h_14.JPG.dat	1391.0	19.0	1.0	0.02	#NUM!	1.2E-06
YPR017C	YPR017C	S288C_ctrl_hmg124h_14.JPG.dat	1410.0	17.0	1.0	0.02	#NUM!	1.0E-06
YPR018W	YPR018W	S288C_ctrl_hmg124h_14.JPG.dat	1307.5	48.3	1.0	0.03	#NUM!	1.0E-05
YPR020W	YPR020W	S288C_ctrl_hmg124h_14.JPG.dat	1258.0	9.3	1.0	0.01	#NUM!	3.4E-07
YPR021C	YPR021C	S288C_ctrl_hmg124h_14.JPG.dat	1307.5	87.9	1.0	0.01	#NUM!	3.8E-05
YPR022C	YPR022C	S288C_ctrl_hmg124h_14.JPG.dat	1212.3	53.2	0.9	0.00	#NUM!	9.2E-06
YPR023C	YPR023C	S288C_ctrl_hmg124h_14.JPG.dat	1303.8	42.5	1.0	0.03	#NUM!	9.7E-06
YPR024W	YPR024W	S288C_ctrl_hmg124h_14.JPG.dat	853.0	655.7	0.6	0.50	#NUM!	8.1E-02
YPR026W	YPR026W	S288C_ctrl_hmg124h_14.JPG.dat	1343.0	11.5	1.0	0.00	#NUM!	3.0E-06
YPR027C	YPR027C	S288C_ctrl_hmg124h_14.JPG.dat	1356.5	24.6	1.0	0.02	#NUM!	1.4E-06
YPR028W	YPR028W	S288C_ctrl_hmg124h_14.JPG.dat	1335.8	44.3	1.0	0.03	#NUM!	5.4E-06
YPR029C	YPR029C	S288C_ctrl_hmg124h_14.JPG.dat	1419.0	38.2	1.1	0.01	#NUM!	1.2E-05
YPR030W	YPR030W	S288C_ctrl_hmg124h_14.JPG.dat	1397.0	26.6	1.0	0.02	#NUM!	1.9E-06
YPR031W	YPR031W	S288C_ctrl_hmg124h_14.JPG.dat	1354.3	28.0	1.0	0.01	#NUM!	1.8E-05
YPR032W	YPR032W	S288C_ctrl_hmg124h_14.JPG.dat	1242.8	24.8	1.0	0.02	#NUM!	1.2E-06
YPR037C	YPR037C	S288C_ctrl_hmg124h_14.JPG.dat	1268.8	30.7	1.0	0.02	#NUM!	2.8E-06
YPR038W	YPR038W	S288C_ctrl_hmg124h_14.JPG.dat	1323.5	27.4	1.0	0.02	#NUM!	1.5E-06
YPR039W	YPR039W	S288C_ctrl_hmg124h_14.JPG.dat	1328.5	34.1	1.0	0.03	#NUM!	7.2E-06
YPR040W	YPR040W	S288C_ctrl_hmg124h_14.JPG.dat	1259.8	31.4	1.0	0.02	#NUM!	3.0E-06
YPR042C	YPR042C	S288C_ctrl_hmg124h_14.JPG.dat	1246.0	14.3	1.0	0.01	#NUM!	1.8E-07
YPR043W	YPR043W	S288C_ctrl_hmg124h_14.JPG.dat	1108.3	30.0	0.8	0.02	#NUM!	5.7E-06
YPR044C	YPR044C	S288C_ctrl_hmg124h_14.JPG.dat	1312.5	166.9	1.0	0.04	#NUM!	5.7E-04
YPR045C	YPR045C	S288C_ctrl_hmg124h_14.JPG.dat	1322.0	51.3	1.0	0.03	#NUM!	8.1E-06
YPR046W	YPR046W	S288C_ctrl_hmg124h_14.JPG.dat	1525.5	80.4	1.1	0.00	#NUM!	4.4E-06
YPR050C	YPR050C	S288C_ctrl_hmg124h_14.JPG.dat	1417.5	42.2	1.0	0.03	#NUM!	5.8E-06
YPR051W	YPR051W	S288C_ctrl_hmg124h_14.JPG.dat	1379.0	22.7	1.0	0.01	#NUM!	2.5E-07
YPR052C	YPR052C	S288C_ctrl_hmg124h_14.JPG.dat	1320.5	16.9	1.0	0.01	#NUM!	1.2E-05
YPR053C	YPR053C	S288C_ctrl_hmg124h_14.JPG.dat	1222.8	24.1	1.0	0.01	#NUM!	5.2E-07
YPR054W	YPR054W	S288C_ctrl_hmg124h_14.JPG.dat	1248.8	24.0	1.0	0.02	#NUM!	1.8E-06

YPR057W	YPR057W	S288C_ctrl_hmg124h_14.JPG.dat	972.3	200.3	0.8	0.16	#NUM!	2.4E-03
YPR058W	YPR058W	S288C_ctrl_hmg124h_14.JPG.dat	1286.3	42.5	1.0	0.03	#NUM!	5.9E-06
YPR059C	YPR059C	S288C_ctrl_hmg124h_14.JPG.dat	1309.8	38.6	1.0	0.03	#NUM!	9.6E-06
YPR060C	YPR060C	S288C_ctrl_hmg124h_14.JPG.dat	1245.0	37.2	0.9	0.00	#NUM!	3.1E-06
YPR061C	YPR061C	S288C_ctrl_hmg124h_14.JPG.dat	1279.3	79.2	1.0	0.05	#NUM!	2.6E-05
YPR062W	YPR062W	S288C_ctrl_hmg124h_14.JPG.dat	1553.0	22.2	1.1	0.01	#NUM!	6.7E-07
YPR063C	YPR063C	S288C_ctrl_hmg124h_14.JPG.dat	1360.8	20.2	1.0	0.01	#NUM!	6.0E-07
YPR064W	YPR064W	S288C_ctrl_hmg124h_14.JPG.dat	1408.8	46.1	1.0	0.04	#NUM!	1.2E-05
YPR065W	YPR065W	S288C_ctrl_hmg124h_14.JPG.dat	1382.3	37.7	1.0	0.01	#NUM!	2.0E-05
YPR066W	YPR066W	S288C_ctrl_hmg124h_14.JPG.dat	1214.3	51.3	0.9	0.04	#NUM!	2.3E-05
YPR068C	YPR068C	S288C_ctrl_hmg124h_14.JPG.dat	1275.3	38.3	1.0	0.02	#NUM!	3.5E-06
YPR069C	YPR069C	S288C_ctrl_hmg124h_14.JPG.dat	1150.3	83.7	0.9	0.06	#NUM!	1.1E-04
YPR070W	YPR070W	S288C_ctrl_hmg124h_14.JPG.dat	1119.5	55.1	0.9	0.01	#NUM!	8.0E-05
YPR071W	YPR071W	S288C_ctrl_hmg124h_14.JPG.dat	1359.5	33.4	1.0	0.03	#NUM!	5.1E-06
YPR073C	YPR073C	S288C_ctrl_hmg124h_14.JPG.dat	1345.0	46.3	1.0	0.04	#NUM!	1.3E-05
YPR074C	YPR074C	S288C_ctrl_hmg124h_14.JPG.dat	1308.5	116.3	1.0	0.08	#NUM!	1.1E-04
YPR075C	YPR075C	S288C_ctrl_hmg124h_14.JPG.dat	1489.5	68.3	1.0	0.05	#NUM!	3.2E-05
YPR076W	YPR076W	S288C_ctrl_hmg124h_14.JPG.dat	1571.8	45.5	1.0	0.02	#NUM!	2.1E-06
YPR077C	YPR077C	S288C_ctrl_hmg124h_14.JPG.dat	1643.5	76.3	1.1	0.03	#NUM!	5.2E-06
YPR078C	YPR078C	S288C_ctrl_hmg124h_14.JPG.dat	1578.5	62.5	1.0	0.00	#NUM!	1.4E-06
YPR079W	YPR079W	S288C_ctrl_hmg124h_14.JPG.dat	1513.0	99.0	1.0	0.04	#NUM!	2.2E-05
YPR083W	YPR083W	S288C_ctrl_hmg124h_14.JPG.dat	1345.3	21.8	1.0	0.01	#NUM!	1.0E-07
YPR084W	YPR084W	S288C_ctrl_hmg124h_14.JPG.dat	1520.0	103.4	1.0	0.05	#NUM!	3.1E-05
YPR089W	YPR089W	S288C_ctrl_hmg124h_14.JPG.dat	1485.8	52.4	1.0	0.02	#NUM!	3.6E-06
YPR090W	YPR090W	S288C_ctrl_hmg124h_14.JPG.dat	1437.8	43.0	1.0	0.02	#NUM!	3.9E-06
YPR091C	YPR091C	S288C_ctrl_hmg124h_14.JPG.dat	1416.0	29.9	1.0	0.02	#NUM!	1.2E-06
YPR092W	YPR092W	S288C_ctrl_hmg124h_14.JPG.dat	1473.0	25.4	1.0	0.00	#NUM!	1.8E-06
YPR093C	YPR093C	S288C_ctrl_hmg124h_14.JPG.dat	1360.8	20.2	1.0	0.02	#NUM!	4.6E-06
YPR095C	YPR095C	S288C_ctrl_hmg124h_14.JPG.dat	1356.8	44.5	1.0	0.04	#NUM!	1.7E-05
YPR096C	YPR096C	S288C_ctrl_hmg124h_14.JPG.dat	1318.8	34.8	1.0	0.01	#NUM!	1.8E-05
YPR097W	YPR097W	S288C_ctrl_hmg124h_14.JPG.dat	1300.5	44.7	1.0	0.04	#NUM!	1.3E-05
YPR098C	YPR098C	S288C_ctrl_hmg124h_14.JPG.dat	1364.3	38.6	1.0	0.03	#NUM!	6.6E-06
YPR106W	YPR106W	S288C_ctrl_hmg124h_14.JPG.dat	1345.3	11.1	1.0	0.01	#NUM!	8.7E-08
YPR109W	YPR109W	S288C_ctrl_hmg124h_14.JPG.dat	1471.0	12.5	1.0	0.01	#NUM!	1.6E-07
YPR111W	YPR111W	S288C_ctrl_hmg124h_14.JPG.dat	1411.5	28.6	1.0	0.02	#NUM!	2.7E-06
YPR114W	YPR114W	S288C_ctrl_hmg124h_14.JPG.dat	1462.0	38.0	1.0	0.03	#NUM!	4.2E-06
YPR115W	YPR115W	S288C_ctrl_hmg124h_14.JPG.dat	1405.5	27.7	1.0	0.02	#NUM!	2.2E-06
YPR117W	YPR117W	S288C_ctrl_hmg124h_14.JPG.dat	1475.3	40.5	1.0	0.03	#NUM!	4.4E-06
YPR118W	YPR118W	S288C_ctrl_hmg124h_14.JPG.dat	1355.8	33.8	1.0	0.02	#NUM!	1.6E-06
YPR119W	YPR119W	S288C_ctrl_hmg124h_14.JPG.dat	1409.0	11.2	1.0	0.01	#NUM!	4.1E-07
YPR120C	YPR120C	S288C_ctrl_hmg124h_14.JPG.dat	1377.0	25.8	1.0	0.01	#NUM!	7.7E-07
YPR121W	YPR121W	S288C_ctrl_hmg124h_14.JPG.dat	1190.5	37.0	0.9	0.03	#NUM!	6.1E-06
YPR122W	YPR122W	S288C_ctrl_hmg124h_14.JPG.dat	1339.3	34.7	1.0	0.02	#NUM!	3.8E-06
YPR123C	YPR123C	S288C_ctrl_hmg124h_14.JPG.dat	1381.3	78.5	1.0	0.05	#NUM!	3.7E-05
YPR125W	YPR125W	S288C_ctrl_hmg124h_14.JPG.dat	1409.3	18.0	1.0	0.01	#NUM!	6.1E-07
YPR126C	YPR126C	S288C_ctrl_hmg124h_14.JPG.dat	1380.8	8.4	1.0	0.01	#NUM!	3.7E-08
YPR127W	YPR127W	S288C_ctrl_hmg124h_14.JPG.dat	1350.3	16.6	1.0	0.01	#NUM!	6.3E-07
YPR128C	YPR128C	S288C_ctrl_hmg124h_14.JPG.dat	1372.0	34.4	1.0	0.02	#NUM!	3.1E-06
YPR129W	YPR129W	S288C_ctrl_hmg124h_14.JPG.dat	1328.8	25.8	1.0	0.02	#NUM!	1.7E-06
YPR130C	YPR130C	S288C_ctrl_hmg124h_14.JPG.dat	1400.3	19.9	1.0	0.02	#NUM!	1.2E-06
YPR132W	YPR132W	S288C_ctrl_hmg124h_14.JPG.dat	1309.8	86.9	0.9	0.06	#NUM!	8.6E-05
YPR134W	YPR134W	S288C_ctrl_hmg124h_14.JPG.dat	1069.3	415.7	0.8	0.30	#NUM!	1.4E-02
YPR135W	YPR135W	S288C_ctrl_hmg124h_14.JPG.dat	1144.8	46.2	0.9	0.03	#NUM!	9.7E-06
YPR138C	YPR138C	S288C_ctrl_hmg124h_14.JPG.dat	1394.8	4.8	1.0	0.00	#NUM!	1.3E-06
YPR140W	YPR140W	S288C_ctrl_hmg124h_14.JPG.dat	1417.8	25.4	1.0	0.02	#NUM!	1.7E-06
YPR141C	YPR141C	S288C_ctrl_hmg124h_14.JPG.dat	1115.3	26.7	0.8	0.01	#NUM!	1.5E-05
YPR145W	YPR145W	S288C_ctrl_hmg124h_14.JPG.dat	1290.8	15.4	1.0	0.01	#NUM!	2.5E-07
YPR146C	YPR146C	S288C_ctrl_hmg124h_14.JPG.dat	1332.0	9.9	1.0	0.01	#NUM!	5.1E-07
YPR147C	YPR147C	S288C_ctrl_hmg124h_14.JPG.dat	1351.8	24.1	1.0	0.02	#NUM!	1.6E-06
YPR148C	YPR148C	S288C_ctrl_hmg124h_14.JPG.dat	1291.3	7.6	1.0	0.01	#NUM!	1.7E-07
YPR149W	YPR149W	S288C_ctrl_hmg124h_14.JPG.dat	1356.8	18.3	1.0	0.01	#NUM!	6.0E-07
YPR150W	YPR150W	S288C_ctrl_hmg124h_14.JPG.dat	1332.0	22.7	1.0	0.02	#NUM!	1.2E-06
YPR151C	YPR151C	S288C_ctrl_hmg124h_14.JPG.dat	1379.0	65.3	1.0	0.05	#NUM!	4.1E-05
YPR152C	YPR152C	S288C_ctrl_hmg124h_14.JPG.dat	1354.5	11.1	1.0	0.01	#NUM!	2.7E-07
YPR153W	YPR153W	S288C_ctrl_hmg124h_14.JPG.dat	1314.0	49.6	1.0	0.03	#NUM!	1.2E-05
YPR154W	YPR154W	S288C_ctrl_hmg124h_14.JPG.dat	1391.0	21.2	1.0	0.02	#NUM!	1.0E-06
YPR155C	YPR155C	S288C_ctrl_hmg124h_14.JPG.dat	1345.8	21.3	1.0	0.02	#NUM!	1.1E-06
YPR156C	YPR156C	S288C_ctrl_hmg124h_14.JPG.dat	1355.5	41.9	1.0	0.01	#NUM!	3.1E-05
YPR157W	YPR157W	S288C_ctrl_hmg124h_14.JPG.dat	1225.5	14.7	1.0	0.01	#NUM!	2.6E-07
YPR158W	YPR158W	S288C_ctrl_hmg124h_14.JPG.dat	1299.5	57.4	1.0	0.04	#NUM!	1.5E-05

YPR160W	YPR160W	S288C_ctrl_hmg124h_14.JPG.dat	1269.0	34.9	1.0	0.03	#NUM!	5.6E-06
YPR164W	YPR164W	S288C_ctrl_hmg124h_14.JPG.dat	1193.8	31.9	0.9	0.02	#NUM!	4.1E-06
YPR167C	YPR167C	S288C_ctrl_hmg124h_14.JPG.dat	1334.5	39.6	1.0	0.01	#NUM!	2.5E-05
YPR170C	YPR170C	S288C_ctrl_hmg124h_14.JPG.dat	1350.3	31.1	1.0	0.03	#NUM!	4.5E-06
YPR171W	YPR171W	S288C_ctrl_hmg124h_14.JPG.dat	1346.8	21.3	1.0	0.01	#NUM!	4.5E-07
YPR172W	YPR172W	S288C_ctrl_hmg124h_14.JPG.dat	1442.8	8.6	1.1	0.01	#NUM!	9.4E-08
YPR173C	YPR173C	S288C_ctrl_hmg124h_14.JPG.dat	691.5	422.8	0.4	0.12	#NUM!	3.3E-02
YPR174C	YPR174C	S288C_ctrl_hmg124h_14.JPG.dat	1332.8	13.3	1.0	0.01	#NUM!	9.1E-08
YPR179C	YPR179C	S288C_ctrl_hmg124h_14.JPG.dat	1308.0	28.5	1.0	0.02	#NUM!	2.7E-06
YPR184W	YPR184W	S288C_ctrl_hmg124h_14.JPG.dat	1287.5	25.9	1.0	0.02	#NUM!	1.4E-06
YPR185W	YPR185W	S288C_ctrl_hmg124h_14.JPG.dat	1222.3	17.7	1.0	0.01	#NUM!	5.2E-07
YPR188C	YPR188C	S288C_ctrl_hmg124h_14.JPG.dat	1235.3	43.0	1.0	0.03	#NUM!	1.1E-05
YPR189W	YPR189W	S288C_ctrl_hmg124h_14.JPG.dat	1216.8	11.4	1.0	0.01	#NUM!	7.0E-07
YPR191W	YPR191W	S288C_ctrl_hmg124h_14.JPG.dat	1270.8	131.8	1.0	0.09	#NUM!	2.5E-04
YPR192W	YPR192W	S288C_ctrl_hmg124h_14.JPG.dat	1374.0	24.5	1.0	0.01	#NUM!	9.7E-06
YPR193C	YPR193C	S288C_ctrl_hmg124h_14.JPG.dat	1361.8	38.6	1.0	0.03	#NUM!	6.4E-06
YPR194C	YPR194C	S288C_ctrl_hmg124h_14.JPG.dat	1494.5	32.5	1.1	0.00	#NUM!	6.3E-06
YPR195C	YPR195C	S288C_ctrl_hmg124h_14.JPG.dat	1398.8	23.8	1.0	0.02	#NUM!	1.5E-06
YPR196W	YPR196W	S288C_ctrl_hmg124h_14.JPG.dat	1436.8	11.1	1.0	0.01	#NUM!	9.8E-08
YPR197C	YPR197C	S288C_ctrl_hmg124h_14.JPG.dat	1008.8	672.9	1.0	0.02	#NUM!	1.2E-04
YPR198W	YPR198W	S288C_ctrl_hmg124h_14.JPG.dat	1323.3	15.4	1.0	0.01	#NUM!	1.3E-07
YPR199C	YPR199C	S288C_ctrl_hmg124h_14.JPG.dat	1282.5	39.5	1.0	0.00	#NUM!	6.6E-06
YPR200C	YPR200C	S288C_ctrl_hmg124h_14.JPG.dat	1284.0	28.3	1.0	0.01	#NUM!	9.1E-06
YPR201W	YPR201W	S288C_ctrl_hmg124h_14.JPG.dat	1310.0	36.8	1.0	0.03	#NUM!	6.9E-06
1	1	S288C_ctrl_hmg124h_9.JPG.dat	332.3	664.5	0.0	0.00	#NUM!	
2	2	S288C_ctrl_hmg124h_9.JPG.dat	1143.8	89.2	1.0	0.08	#NUM!	1.4E-04
3	3	S288C_ctrl_hmg124h_9.JPG.dat	1168.0	202.8	1.0	0.18	#NUM!	1.5E-03
4	4	S288C_ctrl_hmg124h_9.JPG.dat	1072.8	46.0	1.0	0.01	#NUM!	2.6E-05
YLL046C	YLL046C	S288C_ctrl_hmg124h_9.JPG.dat	1231.8	39.6	1.0	0.00	#NUM!	2.6E-06
YLL047W	YLL047W	S288C_ctrl_hmg124h_9.JPG.dat	1194.8	31.6	1.0	0.01	#NUM!	1.7E-07
YLL048C	YLL048C	S288C_ctrl_hmg124h_9.JPG.dat	1184.5	36.2	1.0	0.02	#NUM!	1.1E-06
YLL049W	YLL049W	S288C_ctrl_hmg124h_9.JPG.dat	1169.8	42.3	1.0	0.03	#NUM!	6.1E-06
YLL051C	YLL051C	S288C_ctrl_hmg124h_9.JPG.dat	1156.3	30.1	1.0	0.01	#NUM!	3.9E-07
YLL052C	YLL052C	S288C_ctrl_hmg124h_9.JPG.dat	1171.0	28.5	1.0	0.02	#NUM!	1.4E-06
YLL053C	YLL053C	S288C_ctrl_hmg124h_9.JPG.dat	1169.5	22.9	1.0	0.03	#NUM!	6.9E-06
YLL054C	YLL054C	S288C_ctrl_hmg124h_9.JPG.dat	1171.5	21.6	1.0	0.01	#NUM!	2.8E-07
YLL055W	YLL055W	S288C_ctrl_hmg124h_9.JPG.dat	1107.0	20.3	1.0	0.00	#NUM!	1.2E-07
YLL056C	YLL056C	S288C_ctrl_hmg124h_9.JPG.dat	1101.8	29.5	1.0	0.02	#NUM!	1.4E-06
YLL057C	YLL057C	S288C_ctrl_hmg124h_9.JPG.dat	1209.8	38.9	1.0	0.01	#NUM!	5.0E-05
YLL058W	YLL058W	S288C_ctrl_hmg124h_9.JPG.dat	1193.3	32.4	1.0	0.02	#NUM!	3.1E-06
YLL059C	YLL059C	S288C_ctrl_hmg124h_9.JPG.dat	1178.5	25.4	1.0	0.02	#NUM!	2.6E-06
YLL060C	YLL060C	S288C_ctrl_hmg124h_9.JPG.dat	1180.0	39.7	1.0	0.03	#NUM!	8.2E-06
YLL061W	YLL061W	S288C_ctrl_hmg124h_9.JPG.dat	1142.5	10.2	1.0	0.01	#NUM!	6.7E-08
YLL062C	YLL062C	S288C_ctrl_hmg124h_9.JPG.dat	1087.8	15.4	1.0	0.01	#NUM!	1.4E-05
YLL063C	YLL063C	S288C_ctrl_hmg124h_9.JPG.dat	1096.8	19.2	1.0	0.02	#NUM!	2.3E-06
YLR001C	YLR001C	S288C_ctrl_hmg124h_9.JPG.dat	1140.0	23.5	1.0	0.02	#NUM!	1.7E-06
YLR003C	YLR003C	S288C_ctrl_hmg124h_9.JPG.dat	1091.0	10.7	1.0	0.01	#NUM!	7.9E-08
YLR004C	YLR004C	S288C_ctrl_hmg124h_9.JPG.dat	1114.8	19.6	1.0	0.00	#NUM!	7.5E-06
YLR006C	YLR006C	S288C_ctrl_hmg124h_9.JPG.dat	1116.3	23.0	1.0	0.02	#NUM!	3.4E-06
YLR011W	YLR011W	S288C_ctrl_hmg124h_9.JPG.dat	1115.8	59.4	1.0	0.06	#NUM!	5.6E-05
YLR012C	YLR012C	S288C_ctrl_hmg124h_9.JPG.dat	1155.3	91.4	1.0	0.07	#NUM!	1.1E-04
YLR013W	YLR013W	S288C_ctrl_hmg124h_9.JPG.dat	1131.0	27.9	1.0	0.02	#NUM!	4.5E-06
YLR014C	YLR014C	S288C_ctrl_hmg124h_9.JPG.dat	1191.3	14.3	1.0	0.00	#NUM!	1.3E-06
YLR015W	YLR015W	S288C_ctrl_hmg124h_9.JPG.dat	1115.3	15.3	1.0	0.02	#NUM!	1.6E-06
YLR016C	YLR016C	S288C_ctrl_hmg124h_9.JPG.dat	1130.8	15.5	1.0	0.01	#NUM!	2.6E-07
YLR017W	YLR017W	S288C_ctrl_hmg124h_9.JPG.dat	1094.5	24.6	1.0	0.02	#NUM!	3.6E-06
YLR018C	YLR018C	S288C_ctrl_hmg124h_9.JPG.dat	1115.8	21.3	1.0	0.01	#NUM!	8.4E-07
YLR019W	YLR019W	S288C_ctrl_hmg124h_9.JPG.dat	1103.3	23.9	1.0	0.02	#NUM!	2.3E-06
YLR020C	YLR020C	S288C_ctrl_hmg124h_9.JPG.dat	1103.5	7.2	1.0	0.00	#NUM!	1.5E-08
YLR021W	YLR021W	S288C_ctrl_hmg124h_9.JPG.dat	1118.5	26.3	1.0	0.03	#NUM!	4.6E-06
YLR023C	YLR023C	S288C_ctrl_hmg124h_9.JPG.dat	1117.5	19.7	1.0	0.02	#NUM!	1.4E-06
YLR024C	YLR024C	S288C_ctrl_hmg124h_9.JPG.dat	1117.0	76.0	1.0	0.07	#NUM!	9.8E-05
YLR028C	YLR028C	S288C_ctrl_hmg124h_9.JPG.dat	1200.5	37.7	1.0	0.03	#NUM!	1.1E-05
YLR030W	YLR030W	S288C_ctrl_hmg124h_9.JPG.dat	1191.0	30.6	1.0	0.03	#NUM!	5.6E-06
YLR031W	YLR031W	S288C_ctrl_hmg124h_9.JPG.dat	1117.3	47.6	1.0	0.04	#NUM!	1.7E-05
YLR032W	YLR032W	S288C_ctrl_hmg124h_9.JPG.dat	1150.5	18.7	1.0	0.02	#NUM!	1.3E-06
YLR034C	YLR034C	S288C_ctrl_hmg124h_9.JPG.dat	1127.0	16.7	1.0	0.01	#NUM!	6.1E-07
YLR035C	YLR035C	S288C_ctrl_hmg124h_9.JPG.dat	1126.8	37.1	1.0	0.01	#NUM!	3.7E-05
YLR036C	YLR036C	S288C_ctrl_hmg124h_9.JPG.dat	1130.3	32.3	1.0	0.03	#NUM!	8.3E-06
YLR037C	YLR037C	S288C_ctrl_hmg124h_9.JPG.dat	1111.5	28.6	1.0	0.01	#NUM!	1.5E-05

YLR038C	YLR038C	S288C_ctrl_hmg124h_9.JPG.dat	953.5	81.4	0.9	0.07	#NUM!	1.8E-04
YLR039C	YLR039C	S288C_ctrl_hmg124h_9.JPG.dat	1116.3	30.3	1.0	0.02	#NUM!	3.4E-06
YLR040C	YLR040C	S288C_ctrl_hmg124h_9.JPG.dat	1201.3	31.7	1.0	0.03	#NUM!	7.5E-06
YLR041W	YLR041W	S288C_ctrl_hmg124h_9.JPG.dat	1176.5	13.6	1.0	0.01	#NUM!	5.2E-07
YLR042C	YLR042C	S288C_ctrl_hmg124h_9.JPG.dat	1202.0	19.4	1.0	0.00	#NUM!	3.9E-06
YLR043C	YLR043C	S288C_ctrl_hmg124h_9.JPG.dat	1149.5	26.9	1.0	0.01	#NUM!	3.5E-05
YLR044C	YLR044C	S288C_ctrl_hmg124h_9.JPG.dat	1094.8	36.4	1.0	0.03	#NUM!	7.4E-06
YLR046C	YLR046C	S288C_ctrl_hmg124h_9.JPG.dat	1126.3	25.2	1.0	0.03	#NUM!	4.8E-06
YLR047C	YLR047C	S288C_ctrl_hmg124h_9.JPG.dat	1154.3	18.6	1.0	0.01	#NUM!	8.7E-07
YLR048W	YLR048W	S288C_ctrl_hmg124h_9.JPG.dat	1121.5	30.1	1.0	0.00	#NUM!	1.1E-06
YLR049C	YLR049C	S288C_ctrl_hmg124h_9.JPG.dat	1077.5	12.3	1.0	0.01	#NUM!	5.7E-07
YLR050C	YLR050C	S288C_ctrl_hmg124h_9.JPG.dat	1007.3	100.0	1.0	0.03	#NUM!	3.3E-04
YLR053C	YLR053C	S288C_ctrl_hmg124h_9.JPG.dat	1091.0	27.6	1.0	0.03	#NUM!	5.5E-06
YLR054C	YLR054C	S288C_ctrl_hmg124h_9.JPG.dat	1201.3	28.6	1.0	0.00	#NUM!	2.9E-06
YLR055C	YLR055C	S288C_ctrl_hmg124h_9.JPG.dat	1107.5	63.3	0.9	0.06	#NUM!	5.8E-05
YLR056W	YLR056W	S288C_ctrl_hmg124h_9.JPG.dat	1163.8	26.2	1.0	0.02	#NUM!	2.5E-06
YLR057W	YLR057W	S288C_ctrl_hmg124h_9.JPG.dat	1158.5	18.0	1.0	0.01	#NUM!	5.5E-07
YLR058C	YLR058C	S288C_ctrl_hmg124h_9.JPG.dat	1208.8	20.2	1.0	0.02	#NUM!	1.1E-06
YLR059C	YLR059C	S288C_ctrl_hmg124h_9.JPG.dat	1177.0	37.9	1.0	0.03	#NUM!	8.2E-06
YLR061W	YLR061W	S288C_ctrl_hmg124h_9.JPG.dat	1189.3	15.8	1.0	0.02	#NUM!	9.3E-07
YLR062C	YLR062C	S288C_ctrl_hmg124h_9.JPG.dat	1119.5	62.2	1.0	0.06	#NUM!	5.9E-05
YLR063W	YLR063W	S288C_ctrl_hmg124h_9.JPG.dat	1100.5	28.5	1.0	0.03	#NUM!	5.6E-06
YLR064W	YLR064W	S288C_ctrl_hmg124h_9.JPG.dat	1076.5	30.3	1.0	0.03	#NUM!	6.6E-06
YLR065C	YLR065C	S288C_ctrl_hmg124h_9.JPG.dat	841.5	561.7	1.0	0.03	#NUM!	3.0E-04
YLR070C	YLR070C	S288C_ctrl_hmg124h_9.JPG.dat	1291.5	89.9	1.0	0.02	#NUM!	1.3E-04
YLR072W	YLR072W	S288C_ctrl_hmg124h_9.JPG.dat	1328.8	55.7	1.0	0.03	#NUM!	1.1E-05
YLR073C	YLR073C	S288C_ctrl_hmg124h_9.JPG.dat	1296.5	71.5	1.0	0.02	#NUM!	2.4E-06
YLR077W	YLR077W	S288C_ctrl_hmg124h_9.JPG.dat	1257.5	41.9	1.0	0.02	#NUM!	2.8E-06
YLR079W	YLR079W	S288C_ctrl_hmg124h_9.JPG.dat	1307.5	37.3	1.0	0.01	#NUM!	2.6E-07
YLR080W	YLR080W	S288C_ctrl_hmg124h_9.JPG.dat	1285.5	47.9	1.0	0.01	#NUM!	6.8E-07
YLR081W	YLR081W	S288C_ctrl_hmg124h_9.JPG.dat	1262.5	69.5	1.0	0.03	#NUM!	7.2E-06
YLR082C	YLR082C	S288C_ctrl_hmg124h_9.JPG.dat	1262.8	31.2	1.0	0.01	#NUM!	5.3E-07
YLR083C	YLR083C	S288C_ctrl_hmg124h_9.JPG.dat	1166.3	42.2	1.0	0.02	#NUM!	3.4E-06
YLR084C	YLR084C	S288C_ctrl_hmg124h_9.JPG.dat	1161.5	32.6	1.0	0.01	#NUM!	7.1E-07
YLR085C	YLR085C	S288C_ctrl_hmg124h_9.JPG.dat	1140.8	27.2	1.0	0.01	#NUM!	7.2E-07
YLR087C	YLR087C	S288C_ctrl_hmg124h_9.JPG.dat	1138.8	24.9	1.0	0.02	#NUM!	4.1E-06
YLR089C	YLR089C	S288C_ctrl_hmg124h_9.JPG.dat	1108.8	29.3	0.9	0.01	#NUM!	1.5E-07
YLR090W	YLR090W	S288C_ctrl_hmg124h_9.JPG.dat	1187.0	41.0	1.0	0.02	#NUM!	2.5E-06
YLR091W	YLR091W	S288C_ctrl_hmg124h_9.JPG.dat	1204.3	60.9	1.0	0.01	#NUM!	7.5E-05
YLR092W	YLR092W	S288C_ctrl_hmg124h_9.JPG.dat	1235.0	39.3	1.1	0.02	#NUM!	3.3E-06
YLR093C	YLR093C	S288C_ctrl_hmg124h_9.JPG.dat	1134.0	10.7	1.0	0.02	#NUM!	4.3E-06
YLR094C	YLR094C	S288C_ctrl_hmg124h_9.JPG.dat	1144.8	13.3	1.0	0.01	#NUM!	5.1E-07
YLR095C	YLR095C	S288C_ctrl_hmg124h_9.JPG.dat	1132.8	18.4	1.0	0.00	#NUM!	2.9E-06
YLR096W	YLR096W	S288C_ctrl_hmg124h_9.JPG.dat	1140.3	25.7	1.0	0.01	#NUM!	6.5E-07
YLR097C	YLR097C	S288C_ctrl_hmg124h_9.JPG.dat	1106.5	18.1	1.0	0.02	#NUM!	1.3E-06
YLR098C	YLR098C	S288C_ctrl_hmg124h_9.JPG.dat	1136.3	15.9	1.0	0.01	#NUM!	9.9E-06
YLR099C	YLR099C	S288C_ctrl_hmg124h_9.JPG.dat	1115.0	46.2	1.0	0.03	#NUM!	6.9E-06
YLR102C	YLR102C	S288C_ctrl_hmg124h_9.JPG.dat	1182.5	31.2	1.0	0.03	#NUM!	7.2E-06
YLR104W	YLR104W	S288C_ctrl_hmg124h_9.JPG.dat	1218.0	31.8	1.0	0.01	#NUM!	2.8E-05
YLR107W	YLR107W	S288C_ctrl_hmg124h_9.JPG.dat	1189.8	20.6	1.0	0.02	#NUM!	1.8E-06
YLR108C	YLR108C	S288C_ctrl_hmg124h_9.JPG.dat	1118.0	23.5	1.0	0.02	#NUM!	1.5E-06
YLR109W	YLR109W	S288C_ctrl_hmg124h_9.JPG.dat	1095.8	13.1	1.0	0.01	#NUM!	1.5E-07
YLR110C	YLR110C	S288C_ctrl_hmg124h_9.JPG.dat	1086.3	18.6	1.0	0.02	#NUM!	2.4E-06
YLR111W	YLR111W	S288C_ctrl_hmg124h_9.JPG.dat	1082.5	22.5	1.0	0.01	#NUM!	1.8E-05
YLR112W	YLR112W	S288C_ctrl_hmg124h_9.JPG.dat	1105.8	26.6	1.0	0.02	#NUM!	4.1E-06
YLR113W	YLR113W	S288C_ctrl_hmg124h_9.JPG.dat	1122.0	19.4	1.0	0.01	#NUM!	8.6E-06
YLR114C	YLR114C	S288C_ctrl_hmg124h_9.JPG.dat	1103.5	9.3	1.0	0.00	#NUM!	7.6E-08
YLR118C	YLR118C	S288C_ctrl_hmg124h_9.JPG.dat	1108.3	68.3	1.0	0.07	#NUM!	9.0E-05
YLR119W	YLR119W	S288C_ctrl_hmg124h_9.JPG.dat	1343.5	29.3	1.1	0.02	#NUM!	2.0E-06
YLR120C	YLR120C	S288C_ctrl_hmg124h_9.JPG.dat	1184.5	29.7	1.0	0.00	#NUM!	6.4E-06
YLR121C	YLR121C	S288C_ctrl_hmg124h_9.JPG.dat	1163.8	19.6	1.0	0.01	#NUM!	4.9E-07
YLR122C	YLR122C	S288C_ctrl_hmg124h_9.JPG.dat	1130.3	43.2	1.0	0.04	#NUM!	2.2E-05
YLR123C	YLR123C	S288C_ctrl_hmg124h_9.JPG.dat	1099.3	23.6	1.0	0.00	#NUM!	7.8E-06
YLR124W	YLR124W	S288C_ctrl_hmg124h_9.JPG.dat	1094.3	14.8	1.0	0.01	#NUM!	8.9E-07
YLR125W	YLR125W	S288C_ctrl_hmg124h_9.JPG.dat	1116.8	7.8	1.0	0.01	#NUM!	2.8E-07
YLR126C	YLR126C	S288C_ctrl_hmg124h_9.JPG.dat	1061.3	18.5	1.0	0.02	#NUM!	1.5E-06
YLR128W	YLR128W	S288C_ctrl_hmg124h_9.JPG.dat	1142.8	12.5	1.0	0.00	#NUM!	2.0E-06
YLR130C	YLR130C	S288C_ctrl_hmg124h_9.JPG.dat	1133.3	26.8	1.0	0.03	#NUM!	6.2E-06
YLR131C	YLR131C	S288C_ctrl_hmg124h_9.JPG.dat	1205.3	65.6	1.1	0.08	#NUM!	9.9E-05
YLR133W	YLR133W	S288C_ctrl_hmg124h_9.JPG.dat	1187.3	9.2	1.0	0.00	#NUM!	7.5E-09

YLR134W	YLR134W	S288C_ctrl_hmg124h_9.JPG.dat	1222.0	38.5	1.0	0.03	#NUM!	4.9E-06
YLR135W	YLR135W	S288C_ctrl_hmg124h_9.JPG.dat	1122.3	43.6	1.0	0.00	#NUM!	7.9E-06
YLR136C	YLR136C	S288C_ctrl_hmg124h_9.JPG.dat	1144.0	14.8	1.0	0.02	#NUM!	1.4E-06
YLR137W	YLR137W	S288C_ctrl_hmg124h_9.JPG.dat	1163.8	22.6	1.0	0.02	#NUM!	1.4E-06
YLR138W	YLR138W	S288C_ctrl_hmg124h_9.JPG.dat	1113.8	10.4	1.0	0.01	#NUM!	3.4E-07
YLR142W	YLR142W	S288C_ctrl_hmg124h_9.JPG.dat	1120.3	10.6	1.0	0.00	#NUM!	1.2E-06
YLR143W	YLR143W	S288C_ctrl_hmg124h_9.JPG.dat	1056.0	41.0	1.0	0.04	#NUM!	2.3E-05
YLR144C	YLR144C	S288C_ctrl_hmg124h_9.JPG.dat	1137.0	23.5	1.0	0.00	#NUM!	2.2E-06
YLR146C	YLR146C	S288C_ctrl_hmg124h_9.JPG.dat	1104.0	54.8	1.0	0.04	#NUM!	1.9E-05
YLR149C	YLR149C	S288C_ctrl_hmg124h_9.JPG.dat	1162.3	59.2	1.0	0.05	#NUM!	3.6E-05
YLR150W	YLR150W	S288C_ctrl_hmg124h_9.JPG.dat	1228.3	52.7	1.0	0.04	#NUM!	2.0E-05
YLR151C	YLR151C	S288C_ctrl_hmg124h_9.JPG.dat	1171.3	4.9	1.0	0.01	#NUM!	6.0E-08
YLR152C	YLR152C	S288C_ctrl_hmg124h_9.JPG.dat	1213.3	29.7	1.0	0.03	#NUM!	7.0E-06
YLR154C	YLR154C	S288C_ctrl_hmg124h_9.JPG.dat	1169.3	28.1	1.0	0.03	#NUM!	5.2E-06
YLR164W	YLR164W	S288C_ctrl_hmg124h_9.JPG.dat	1106.3	9.2	1.0	0.01	#NUM!	4.6E-07
YLR165C	YLR165C	S288C_ctrl_hmg124h_9.JPG.dat	1134.8	34.3	1.0	0.01	#NUM!	9.5E-06
YLR168C	YLR168C	S288C_ctrl_hmg124h_9.JPG.dat	1113.5	51.0	1.0	0.04	#NUM!	2.0E-05
YLR169W	YLR169W	S288C_ctrl_hmg124h_9.JPG.dat	1065.8	18.3	1.0	0.02	#NUM!	1.6E-06
YLR170C	YLR170C	S288C_ctrl_hmg124h_9.JPG.dat	1034.3	30.4	0.9	0.01	#NUM!	2.6E-05
YLR171W	YLR171W	S288C_ctrl_hmg124h_9.JPG.dat	1088.3	37.7	1.0	0.01	#NUM!	3.2E-05
YLR172C	YLR172C	S288C_ctrl_hmg124h_9.JPG.dat	1209.3	37.3	1.0	0.05	#NUM!	3.0E-05
YLR173W	YLR173W	S288C_ctrl_hmg124h_9.JPG.dat	1196.3	23.6	1.0	0.02	#NUM!	2.6E-06
YLR174W	YLR174W	S288C_ctrl_hmg124h_9.JPG.dat	1159.5	26.4	1.0	0.00	#NUM!	3.2E-06
YLR176C	YLR176C	S288C_ctrl_hmg124h_9.JPG.dat	1151.5	53.0	1.0	0.04	#NUM!	2.4E-05
YLR177W	YLR177W	S288C_ctrl_hmg124h_9.JPG.dat	1173.3	52.3	1.0	0.04	#NUM!	2.2E-05
YLR178C	YLR178C	S288C_ctrl_hmg124h_9.JPG.dat	1153.8	32.3	1.0	0.03	#NUM!	4.5E-06
YLR179C	YLR179C	S288C_ctrl_hmg124h_9.JPG.dat	1167.0	50.3	1.0	0.01	#NUM!	4.7E-05
YLR180W	YLR180W	S288C_ctrl_hmg124h_9.JPG.dat	1083.3	6.5	1.0	0.01	#NUM!	1.6E-07
YLR181C	YLR181C	S288C_ctrl_hmg124h_9.JPG.dat	1139.3	34.0	1.0	0.03	#NUM!	7.7E-06
YLR182W	YLR182W	S288C_ctrl_hmg124h_9.JPG.dat	1057.5	41.5	1.0	0.04	#NUM!	1.4E-05
YLR183C	YLR183C	S288C_ctrl_hmg124h_9.JPG.dat	1140.3	19.4	1.0	0.02	#NUM!	1.3E-06
YLR184W	YLR184W	S288C_ctrl_hmg124h_9.JPG.dat	1189.0	153.3	0.9	0.13	#NUM!	7.5E-04
YLR185W	YLR185W	S288C_ctrl_hmg124h_9.JPG.dat	949.8	169.4	0.7	0.11	#NUM!	9.4E-04
YLR187W	YLR187W	S288C_ctrl_hmg124h_9.JPG.dat	1256.5	64.9	1.0	0.02	#NUM!	1.1E-06
YLR188W	YLR188W	S288C_ctrl_hmg124h_9.JPG.dat	1271.5	70.0	1.0	0.04	#NUM!	2.3E-05
YLR189C	YLR189C	S288C_ctrl_hmg124h_9.JPG.dat	1272.0	58.2	1.0	0.03	#NUM!	6.2E-06
YLR190W	YLR190W	S288C_ctrl_hmg124h_9.JPG.dat	1256.0	41.7	1.0	0.00	#NUM!	1.9E-07
YLR191W	YLR191W	S288C_ctrl_hmg124h_9.JPG.dat	1262.8	44.9	1.0	0.03	#NUM!	9.0E-06
YLR192C	YLR192C	S288C_ctrl_hmg124h_9.JPG.dat	1213.0	37.0	1.0	0.01	#NUM!	4.3E-08
YLR193C	YLR193C	S288C_ctrl_hmg124h_9.JPG.dat	1208.3	36.7	1.0	0.00	#NUM!	6.7E-07
YLR194C	YLR194C	S288C_ctrl_hmg124h_9.JPG.dat	1145.8	48.7	1.0	0.02	#NUM!	2.2E-06
YLR199C	YLR199C	S288C_ctrl_hmg124h_9.JPG.dat	1216.8	44.3	1.0	0.05	#NUM!	2.7E-05
YLR200W	YLR200W	S288C_ctrl_hmg124h_9.JPG.dat	1051.8	25.0	0.9	0.02	#NUM!	3.9E-06
YLR205C	YLR205C	S288C_ctrl_hmg124h_9.JPG.dat	1214.8	25.6	1.0	0.00	#NUM!	6.0E-06
YLR206W	YLR206W	S288C_ctrl_hmg124h_9.JPG.dat	1265.3	17.2	1.0	0.01	#NUM!	5.5E-07
YLR207W	YLR207W	S288C_ctrl_hmg124h_9.JPG.dat	1270.8	7.3	1.1	0.00	#NUM!	7.9E-09
YLR209C	YLR209C	S288C_ctrl_hmg124h_9.JPG.dat	1206.0	19.9	1.0	0.02	#NUM!	1.1E-06
YLR210W	YLR210W	S288C_ctrl_hmg124h_9.JPG.dat	1178.0	38.8	1.0	0.03	#NUM!	1.1E-05
YLR211C	YLR211C	S288C_ctrl_hmg124h_9.JPG.dat	1164.8	82.3	1.0	0.02	#NUM!	1.4E-04
YLR213C	YLR213C	S288C_ctrl_hmg124h_9.JPG.dat	1179.5	117.0	1.0	0.10	#NUM!	2.5E-04
YLR214W	YLR214W	S288C_ctrl_hmg124h_9.JPG.dat	1093.8	60.0	1.0	0.05	#NUM!	3.6E-05
YLR216C	YLR216C	S288C_ctrl_hmg124h_9.JPG.dat	1129.3	52.8	1.0	0.04	#NUM!	2.5E-05
YLR217W	YLR217W	S288C_ctrl_hmg124h_9.JPG.dat	1078.3	97.9	0.9	0.07	#NUM!	1.3E-04
YLR218C	YLR218C	S288C_ctrl_hmg124h_9.JPG.dat	258.8	517.5	0.0	0.00	#NUM!	
YLR219W	YLR219W	S288C_ctrl_hmg124h_9.JPG.dat	1165.3	13.9	1.0	0.01	#NUM!	2.1E-07
YLR220W	YLR220W	S288C_ctrl_hmg124h_9.JPG.dat	1154.8	39.9	1.0	0.03	#NUM!	1.0E-05
YLR221C	YLR221C	S288C_ctrl_hmg124h_9.JPG.dat	1115.3	34.8	1.0	0.03	#NUM!	1.2E-05
YLR224W	YLR224W	S288C_ctrl_hmg124h_9.JPG.dat	1067.5	13.0	1.0	0.02	#NUM!	1.2E-06
YLR225C	YLR225C	S288C_ctrl_hmg124h_9.JPG.dat	1060.8	18.9	1.0	0.00	#NUM!	8.4E-06
YLR228C	YLR228C	S288C_ctrl_hmg124h_9.JPG.dat	1125.8	7.1	1.0	0.01	#NUM!	1.6E-07
YLR231C	YLR231C	S288C_ctrl_hmg124h_9.JPG.dat	1122.3	26.1	1.0	0.03	#NUM!	4.3E-06
YLR232W	YLR232W	S288C_ctrl_hmg124h_9.JPG.dat	1090.3	15.2	1.0	0.01	#NUM!	3.6E-07
YLR233C	YLR233C	S288C_ctrl_hmg124h_9.JPG.dat	1116.3	4.6	1.0	0.01	#NUM!	1.8E-07
YLR234W	YLR234W	S288C_ctrl_hmg124h_9.JPG.dat	1000.5	26.3	0.9	0.02	#NUM!	5.4E-06
YLR235C	YLR235C	S288C_ctrl_hmg124h_9.JPG.dat	1123.5	50.1	1.0	0.01	#NUM!	4.0E-05
YLR236C	YLR236C	S288C_ctrl_hmg124h_9.JPG.dat	1178.3	34.5	1.0	0.02	#NUM!	4.0E-06
YLR237W	YLR237W	S288C_ctrl_hmg124h_9.JPG.dat	1153.0	47.6	1.0	0.01	#NUM!	4.5E-05
YLR239C	YLR239C	S288C_ctrl_hmg124h_9.JPG.dat	1174.3	24.7	1.0	0.02	#NUM!	1.5E-06
YLR241W	YLR241W	S288C_ctrl_hmg124h_9.JPG.dat	1099.0	53.3	1.0	0.01	#NUM!	4.9E-05
YLR242C	YLR242C	S288C_ctrl_hmg124h_9.JPG.dat	1100.3	38.0	1.0	0.01	#NUM!	3.3E-05

YLR246W	YLR246W	S288C_ctrl_hmg124h_9.JPG.dat	1103.3	20.8	1.0	0.02	#NUM!	2.2E-06
YLR247C	YLR247C	S288C_ctrl_hmg124h_9.JPG.dat	1075.8	55.7	1.0	0.05	#NUM!	3.1E-05
YLR248W	YLR248W	S288C_ctrl_hmg124h_9.JPG.dat	1094.5	5.4	1.0	0.01	#NUM!	1.8E-07
YLR250W	YLR250W	S288C_ctrl_hmg124h_9.JPG.dat	1115.0	50.1	1.0	0.01	#NUM!	4.4E-05
YLR251W	YLR251W	S288C_ctrl_hmg124h_9.JPG.dat	1128.8	61.5	1.0	0.06	#NUM!	6.8E-05
YLR252W	YLR252W	S288C_ctrl_hmg124h_9.JPG.dat	1191.8	22.7	1.0	0.02	#NUM!	1.0E-06
YLR253W	YLR253W	S288C_ctrl_hmg124h_9.JPG.dat	1180.8	26.6	1.0	0.02	#NUM!	4.0E-06
YLR254C	YLR254C	S288C_ctrl_hmg124h_9.JPG.dat	1180.0	19.7	1.0	0.01	#NUM!	5.2E-07
YLR255C	YLR255C	S288C_ctrl_hmg124h_9.JPG.dat	1138.5	29.7	1.0	0.02	#NUM!	3.8E-06
YLR257W	YLR257W	S288C_ctrl_hmg124h_9.JPG.dat	1126.5	7.9	1.0	0.01	#NUM!	8.7E-07
YLR258W	YLR258W	S288C_ctrl_hmg124h_9.JPG.dat	1135.0	4.0	1.0	0.01	#NUM!	7.9E-08
YLR261C	YLR261C	S288C_ctrl_hmg124h_9.JPG.dat	1131.5	20.1	1.0	0.02	#NUM!	3.2E-06
YLR262C	YLR262C	S288C_ctrl_hmg124h_9.JPG.dat	959.0	33.0	0.9	0.03	#NUM!	9.1E-06
YLR262C-A	YLR262C-A	S288C_ctrl_hmg124h_9.JPG.dat	1095.8	30.3	1.0	0.01	#NUM!	2.5E-05
YLR263W	YLR263W	S288C_ctrl_hmg124h_9.JPG.dat	1140.5	26.6	1.0	0.03	#NUM!	5.5E-06
YLR264W	YLR264W	S288C_ctrl_hmg124h_9.JPG.dat	298.3	596.5	0.0	0.00	#NUM!	
YLR265C	YLR265C	S288C_ctrl_hmg124h_9.JPG.dat	1186.5	48.2	1.0	0.04	#NUM!	1.3E-05
YLR266C	YLR266C	S288C_ctrl_hmg124h_9.JPG.dat	1174.5	12.2	1.0	0.01	#NUM!	1.5E-07
YLR267W	YLR267W	S288C_ctrl_hmg124h_9.JPG.dat	1174.8	18.0	1.0	0.01	#NUM!	7.0E-07
YLR268W	YLR268W	S288C_ctrl_hmg124h_9.JPG.dat	1147.0	17.1	1.0	0.01	#NUM!	4.5E-07
YLR269C	YLR269C	S288C_ctrl_hmg124h_9.JPG.dat	1190.3	52.0	1.0	0.05	#NUM!	2.6E-05
YLR271W	YLR271W	S288C_ctrl_hmg124h_9.JPG.dat	1147.0	25.2	1.0	0.00	#NUM!	6.6E-06
YLR273C	YLR273C	S288C_ctrl_hmg124h_9.JPG.dat	1132.5	16.5	1.0	0.02	#NUM!	9.6E-07
YLR278C	YLR278C	S288C_ctrl_hmg124h_9.JPG.dat	1062.8	31.4	1.0	0.01	#NUM!	3.8E-05
YLR279W	YLR279W	S288C_ctrl_hmg124h_9.JPG.dat	1115.8	11.8	1.0	0.00	#NUM!	5.9E-06
YLR280C	YLR280C	S288C_ctrl_hmg124h_9.JPG.dat	1086.3	29.8	1.0	0.03	#NUM!	6.7E-06
YLR281C	YLR281C	S288C_ctrl_hmg124h_9.JPG.dat	1096.0	35.5	1.0	0.01	#NUM!	1.9E-05
YLR282C	YLR282C	S288C_ctrl_hmg124h_9.JPG.dat	1189.0	22.9	1.0	0.02	#NUM!	2.1E-06
YLR283W	YLR283W	S288C_ctrl_hmg124h_9.JPG.dat	1190.5	29.5	1.0	0.02	#NUM!	3.3E-06
YLR284C	YLR284C	S288C_ctrl_hmg124h_9.JPG.dat	1185.8	17.3	1.0	0.01	#NUM!	8.4E-07
YLR285W	YLR285W	S288C_ctrl_hmg124h_9.JPG.dat	1134.0	22.5	1.0	0.02	#NUM!	2.4E-06
YLR286C	YLR286C	S288C_ctrl_hmg124h_9.JPG.dat	1173.8	60.0	1.0	0.05	#NUM!	3.3E-05
YLR287C	YLR287C	S288C_ctrl_hmg124h_9.JPG.dat	1144.5	18.1	1.0	0.01	#NUM!	7.8E-07
YLR287C-A	YLR287C-A	S288C_ctrl_hmg124h_9.JPG.dat	1190.3	8.1	1.0	0.00	#NUM!	3.7E-07
YLR289W	YLR289W	S288C_ctrl_hmg124h_9.JPG.dat	1063.8	19.0	1.0	0.00	#NUM!	1.4E-06
YLR290C	YLR290C	S288C_ctrl_hmg124h_9.JPG.dat	1039.5	22.4	1.0	0.00	#NUM!	2.9E-06
YLR292C	YLR292C	S288C_ctrl_hmg124h_9.JPG.dat	1121.5	39.4	1.0	0.04	#NUM!	1.2E-05
YLR294C	YLR294C	S288C_ctrl_hmg124h_9.JPG.dat	1088.5	47.5	1.0	0.04	#NUM!	2.1E-05
YLR296W	YLR296W	S288C_ctrl_hmg124h_9.JPG.dat	1233.0	51.9	1.0	0.04	#NUM!	1.6E-05
YLR297W	YLR297W	S288C_ctrl_hmg124h_9.JPG.dat	1217.3	15.9	1.0	0.01	#NUM!	1.1E-05
YLR299W	YLR299W	S288C_ctrl_hmg124h_9.JPG.dat	1199.8	47.6	1.0	0.03	#NUM!	9.5E-06
YLR300W	YLR300W	S288C_ctrl_hmg124h_9.JPG.dat	1204.8	27.6	1.0	0.01	#NUM!	8.8E-06
YLR303W	YLR303W	S288C_ctrl_hmg124h_9.JPG.dat	1173.0	45.8	1.0	0.01	#NUM!	4.0E-05
YLR306W	YLR306W	S288C_ctrl_hmg124h_9.JPG.dat	1186.8	32.4	1.0	0.03	#NUM!	4.7E-06
YLR307W	YLR307W	S288C_ctrl_hmg124h_9.JPG.dat	1191.5	10.1	1.0	0.01	#NUM!	1.3E-07
YLR309C	YLR309C	S288C_ctrl_hmg124h_9.JPG.dat	1153.5	39.3	1.0	0.03	#NUM!	6.3E-06
YLR311C	YLR311C	S288C_ctrl_hmg124h_9.JPG.dat	1109.8	13.1	1.0	0.01	#NUM!	4.5E-07
YLR312C	YLR312C	S288C_ctrl_hmg124h_9.JPG.dat	1127.0	33.6	1.0	0.03	#NUM!	8.6E-06
YLR313C	YLR313C	S288C_ctrl_hmg124h_9.JPG.dat	1129.8	30.7	1.0	0.02	#NUM!	3.3E-06
YLR315W	YLR315W	S288C_ctrl_hmg124h_9.JPG.dat	1032.0	32.6	0.9	0.02	#NUM!	1.5E-06
YLR318W	YLR318W	S288C_ctrl_hmg124h_9.JPG.dat	1256.5	52.5	1.0	0.05	#NUM!	2.4E-05
YLR319C	YLR319C	S288C_ctrl_hmg124h_9.JPG.dat	1278.5	62.8	1.0	0.06	#NUM!	4.3E-05
YLR320W	YLR320W	S288C_ctrl_hmg124h_9.JPG.dat	1255.0	37.4	1.0	0.04	#NUM!	1.3E-05
YLR324W	YLR324W	S288C_ctrl_hmg124h_9.JPG.dat	1197.5	25.2	1.0	0.02	#NUM!	2.4E-06
YLR325C	YLR325C	S288C_ctrl_hmg124h_9.JPG.dat	1182.8	23.4	1.0	0.02	#NUM!	2.7E-06
YLR326W	YLR326W	S288C_ctrl_hmg124h_9.JPG.dat	1166.3	43.7	1.0	0.04	#NUM!	1.4E-05
YLR327C	YLR327C	S288C_ctrl_hmg124h_9.JPG.dat	1171.8	64.0	1.0	0.06	#NUM!	4.7E-05
YLR328W	YLR328W	S288C_ctrl_hmg124h_9.JPG.dat	1056.0	87.3	0.9	0.07	#NUM!	1.3E-04
YLR329W	YLR329W	S288C_ctrl_hmg124h_9.JPG.dat	1088.0	39.0	0.9	0.03	#NUM!	1.1E-05
YLR330W	YLR330W	S288C_ctrl_hmg124h_9.JPG.dat	1198.8	38.6	1.0	0.03	#NUM!	7.2E-06
YLR332W	YLR332W	S288C_ctrl_hmg124h_9.JPG.dat	1200.5	54.5	1.1	0.04	#NUM!	1.5E-05
YLR333C	YLR333C	S288C_ctrl_hmg124h_9.JPG.dat	1147.3	26.0	1.0	0.02	#NUM!	3.7E-06
YLR334C	YLR334C	S288C_ctrl_hmg124h_9.JPG.dat	1152.5	26.8	1.0	0.02	#NUM!	2.0E-06
YLR335W	YLR335W	S288C_ctrl_hmg124h_9.JPG.dat	1149.8	44.2	1.0	0.01	#NUM!	6.9E-05
YLR337C	YLR337C	S288C_ctrl_hmg124h_9.JPG.dat	1048.3	134.7	0.9	0.12	#NUM!	6.0E-04
YLR338W	YLR338W	S288C_ctrl_hmg124h_9.JPG.dat	857.3	174.5	0.8	0.16	#NUM!	2.3E-03
YLR341W	YLR341W	S288C_ctrl_hmg124h_9.JPG.dat	1099.8	12.2	1.0	0.00	#NUM!	1.1E-06
YLR342W	YLR342W	S288C_ctrl_hmg124h_9.JPG.dat	1075.5	88.6	1.0	0.08	#NUM!	1.7E-04
YLR343W	YLR343W	S288C_ctrl_hmg124h_9.JPG.dat	1095.5	10.4	1.0	0.01	#NUM!	2.5E-07
YLR344W	YLR344W	S288C_ctrl_hmg124h_9.JPG.dat	1079.3	15.3	1.0	0.00	#NUM!	4.5E-06

YLR345W	YLR345W	S288C_ctrl_hmg124h_9.JPG.dat	1069.3	89.9	1.0	0.03	#NUM!	3.6E-04
YLR346C	YLR346C	S288C_ctrl_hmg124h_9.JPG.dat	1064.3	116.6	1.0	0.12	#NUM!	5.2E-04
YLR348C	YLR348C	S288C_ctrl_hmg124h_9.JPG.dat	1165.3	57.2	1.0	0.02	#NUM!	9.8E-05
YLR349W	YLR349W	S288C_ctrl_hmg124h_9.JPG.dat	1164.8	12.6	1.0	0.00	#NUM!	6.3E-06
YLR350W	YLR350W	S288C_ctrl_hmg124h_9.JPG.dat	1141.3	44.0	1.0	0.03	#NUM!	7.8E-06
YLR351C	YLR351C	S288C_ctrl_hmg124h_9.JPG.dat	1091.8	16.5	1.0	0.02	#NUM!	1.6E-06
YLR352W	YLR352W	S288C_ctrl_hmg124h_9.JPG.dat	1089.0	19.8	1.0	0.00	#NUM!	8.2E-06
YLR353W	YLR353W	S288C_ctrl_hmg124h_9.JPG.dat	1110.0	4.1	1.0	0.01	#NUM!	9.1E-08
YLR354C	YLR354C	S288C_ctrl_hmg124h_9.JPG.dat	1076.0	26.3	1.0	0.02	#NUM!	3.1E-06
YLR356W	YLR356W	S288C_ctrl_hmg124h_9.JPG.dat	1091.8	22.5	1.0	0.02	#NUM!	1.0E-06
YLR357W	YLR357W	S288C_ctrl_hmg124h_9.JPG.dat	809.0	226.1	0.6	0.06	#NUM!	2.8E-03
YLR360W	YLR360W	S288C_ctrl_hmg124h_9.JPG.dat	1019.8	44.8	0.9	0.04	#NUM!	2.2E-05
YLR361C	YLR361C	S288C_ctrl_hmg124h_9.JPG.dat	1158.8	74.7	1.0	0.07	#NUM!	7.3E-05
YLR362W	YLR362W	S288C_ctrl_hmg124h_9.JPG.dat	0.0	0.0	0.0	0.00	#NUM!	
YLR363C	YLR363C	S288C_ctrl_hmg124h_9.JPG.dat	1206.3	57.1	1.0	0.04	#NUM!	2.1E-05
YLR364W	YLR364W	S288C_ctrl_hmg124h_9.JPG.dat	1154.3	22.2	1.0	0.02	#NUM!	1.5E-06
YLR365W	YLR365W	S288C_ctrl_hmg124h_9.JPG.dat	1132.0	31.3	1.0	0.02	#NUM!	2.7E-06
YLR366W	YLR366W	S288C_ctrl_hmg124h_9.JPG.dat	1088.8	26.9	1.0	0.02	#NUM!	1.2E-06
YLR367W	YLR367W	S288C_ctrl_hmg124h_9.JPG.dat	1031.8	17.6	0.9	0.01	#NUM!	2.6E-07
YLR368W	YLR368W	S288C_ctrl_hmg124h_9.JPG.dat	1079.5	23.6	1.0	0.01	#NUM!	8.5E-07
YLR370C	YLR370C	S288C_ctrl_hmg124h_9.JPG.dat	1134.3	15.0	1.0	0.01	#NUM!	2.0E-07
YLR371W	YLR371W	S288C_ctrl_hmg124h_9.JPG.dat	338.0	175.4	0.3	0.16	#NUM!	3.1E-02
YLR372W	YLR372W	S288C_ctrl_hmg124h_9.JPG.dat	1123.5	22.9	1.0	0.00	#NUM!	8.3E-06
YLR373C	YLR373C	S288C_ctrl_hmg124h_9.JPG.dat	985.3	57.7	0.8	0.02	#NUM!	2.7E-04
YLR374C	YLR374C	S288C_ctrl_hmg124h_9.JPG.dat	1197.5	29.8	1.0	0.02	#NUM!	2.6E-06
YLR375W	YLR375W	S288C_ctrl_hmg124h_9.JPG.dat	1159.8	9.0	1.0	0.01	#NUM!	4.9E-08
YLR376C	YLR376C	S288C_ctrl_hmg124h_9.JPG.dat	1172.8	10.4	1.0	0.01	#NUM!	7.8E-08
YLR377C	YLR377C	S288C_ctrl_hmg124h_9.JPG.dat	1157.8	22.5	1.0	0.02	#NUM!	1.4E-06
YLR380W	YLR380W	S288C_ctrl_hmg124h_9.JPG.dat	1168.5	41.4	1.0	0.01	#NUM!	4.4E-05
YLR381W	YLR381W	S288C_ctrl_hmg124h_9.JPG.dat	1103.8	16.1	1.0	0.01	#NUM!	3.1E-07
YLR384C	YLR384C	S288C_ctrl_hmg124h_9.JPG.dat	1120.3	20.3	1.0	0.01	#NUM!	8.1E-07
YLR385C	YLR385C	S288C_ctrl_hmg124h_9.JPG.dat	1105.3	11.0	1.0	0.00	#NUM!	3.5E-06
YLR386W	YLR386W	S288C_ctrl_hmg124h_9.JPG.dat	1200.8	28.1	1.1	0.02	#NUM!	2.9E-06
YLR387C	YLR387C	S288C_ctrl_hmg124h_9.JPG.dat	1091.3	52.6	1.0	0.01	#NUM!	1.8E-05
YLR388W	YLR388W	S288C_ctrl_hmg124h_9.JPG.dat	991.8	96.6	0.9	0.08	#NUM!	2.1E-04
YLR389C	YLR389C	S288C_ctrl_hmg124h_9.JPG.dat	1214.5	62.6	1.0	0.05	#NUM!	3.6E-05
YLR390W	YLR390W	S288C_ctrl_hmg124h_9.JPG.dat	1206.5	21.1	1.0	0.02	#NUM!	1.3E-06
YLR390W-/	YLR390W-A	S288C_ctrl_hmg124h_9.JPG.dat	1178.8	8.5	1.0	0.00	#NUM!	7.1E-07
YLR391W	YLR391W	S288C_ctrl_hmg124h_9.JPG.dat	1173.5	24.5	1.0	0.02	#NUM!	2.3E-06
YLR392C	YLR392C	S288C_ctrl_hmg124h_9.JPG.dat	1162.0	8.3	1.0	0.01	#NUM!	9.4E-08
YLR393W	YLR393W	S288C_ctrl_hmg124h_9.JPG.dat	1138.0	19.3	1.0	0.02	#NUM!	1.7E-06
YLR394W	YLR394W	S288C_ctrl_hmg124h_9.JPG.dat	1152.0	9.8	1.0	0.00	#NUM!	4.7E-06
YLR395C	YLR395C	S288C_ctrl_hmg124h_9.JPG.dat	1079.3	5.4	1.0	0.00	#NUM!	3.5E-08
YLR398C	YLR398C	S288C_ctrl_hmg124h_9.JPG.dat	1065.8	101.8	0.9	0.03	#NUM!	3.7E-04
YLR400W	YLR400W	S288C_ctrl_hmg124h_9.JPG.dat	1103.5	29.0	1.0	0.03	#NUM!	5.3E-06
YLR401C	YLR401C	S288C_ctrl_hmg124h_9.JPG.dat	1274.5	55.7	1.1	0.05	#NUM!	3.0E-05
YLR402W	YLR402W	S288C_ctrl_hmg124h_9.JPG.dat	907.5	58.0	0.7	0.05	#NUM!	7.1E-05
YLR404W	YLR404W	S288C_ctrl_hmg124h_9.JPG.dat	1203.5	30.6	1.0	0.03	#NUM!	4.6E-06
YLR405W	YLR405W	S288C_ctrl_hmg124h_9.JPG.dat	1193.3	38.9	1.0	0.03	#NUM!	1.1E-05
YLR406C	YLR406C	S288C_ctrl_hmg124h_9.JPG.dat	1188.8	7.8	1.0	0.00	#NUM!	3.6E-06
YLR407W	YLR407W	S288C_ctrl_hmg124h_9.JPG.dat	1218.3	20.5	1.0	0.02	#NUM!	1.3E-06
YLR408C	YLR408C	S288C_ctrl_hmg124h_9.JPG.dat	1180.0	56.8	1.0	0.02	#NUM!	9.4E-05
YLR410W	YLR410W	S288C_ctrl_hmg124h_9.JPG.dat	1167.0	28.4	1.0	0.02	#NUM!	2.8E-06
YLR412W	YLR412W	S288C_ctrl_hmg124h_9.JPG.dat	1058.0	13.9	0.9	0.00	#NUM!	3.1E-07
YLR413W	YLR413W	S288C_ctrl_hmg124h_9.JPG.dat	1092.5	20.5	1.0	0.02	#NUM!	2.6E-06
YLR414C	YLR414C	S288C_ctrl_hmg124h_9.JPG.dat	1165.8	25.5	1.0	0.02	#NUM!	3.0E-06
YOR202W	YOR202W	S288C_ctrl_hmg124h_9.JPG.dat	1221.0	201.2	1.0	0.09	#NUM!	4.0E-292
1	1	S288C_ctrl_hmg124h_7.JPG.dat	1346.8	209.9	1.1	0.13	#NUM!	5.0E-04
2	2	S288C_ctrl_hmg124h_7.JPG.dat	1128.0	74.3	0.9	0.01	#NUM!	5.3E-05
3	3	S288C_ctrl_hmg124h_7.JPG.dat	1110.5	46.6	1.0	0.03	#NUM!	9.7E-06
4	4	S288C_ctrl_hmg124h_7.JPG.dat	1108.5	22.6	1.0	0.01	#NUM!	2.3E-05
YIL060W	YIL060W	S288C_ctrl_hmg124h_7.JPG.dat	1146.8	96.6	1.0	0.07	#NUM!	1.2E-04
YIL064W	YIL064W	S288C_ctrl_hmg124h_7.JPG.dat	1215.8	81.0	1.0	0.07	#NUM!	7.9E-05
YIL065C	YIL065C	S288C_ctrl_hmg124h_7.JPG.dat	1143.8	25.8	1.0	0.02	#NUM!	1.5E-06
YIL066C	YIL066C	S288C_ctrl_hmg124h_7.JPG.dat	1257.0	43.7	1.1	0.04	#NUM!	1.6E-05
YIL067C	YIL067C	S288C_ctrl_hmg124h_7.JPG.dat	1180.5	37.1	1.0	0.03	#NUM!	6.2E-06
YIL070C	YIL070C	S288C_ctrl_hmg124h_7.JPG.dat	1184.5	16.6	1.0	0.01	#NUM!	1.3E-07
YIL071C	YIL071C	S288C_ctrl_hmg124h_7.JPG.dat	1205.5	43.6	1.1	0.04	#NUM!	1.7E-05
YIL072W	YIL072W	S288C_ctrl_hmg124h_7.JPG.dat	1168.0	16.4	1.0	0.02	#NUM!	3.4E-06
YIL073C	YIL073C	S288C_ctrl_hmg124h_7.JPG.dat	1124.8	28.1	1.0	0.03	#NUM!	1.2E-05

YIL074C	YIL074C	S288C_ctrl_hmg124h_7.JPG.dat	1147.5	42.7	1.0	0.03	#NUM!	8.2E-06
YIL076W	YIL076W	S288C_ctrl_hmg124h_7.JPG.dat	1164.3	31.1	1.0	0.03	#NUM!	5.5E-06
YIL077C	YIL077C	S288C_ctrl_hmg124h_7.JPG.dat	1176.0	31.5	1.0	0.03	#NUM!	4.8E-06
YIL079C	YIL079C	S288C_ctrl_hmg124h_7.JPG.dat	1211.0	90.7	1.0	0.07	#NUM!	1.0E-04
YIL084C	YIL084C	S288C_ctrl_hmg124h_7.JPG.dat	1204.0	40.8	1.0	0.03	#NUM!	1.1E-05
YIL086C	YIL086C	S288C_ctrl_hmg124h_7.JPG.dat	1199.8	62.9	1.0	0.01	#NUM!	5.1E-05
YIL087C	YIL087C	S288C_ctrl_hmg124h_7.JPG.dat	1185.5	38.9	1.0	0.02	#NUM!	3.3E-06
YIL088C	YIL088C	S288C_ctrl_hmg124h_7.JPG.dat	1119.8	40.3	1.0	0.01	#NUM!	1.1E-05
YIL089W	YIL089W	S288C_ctrl_hmg124h_7.JPG.dat	1130.8	23.1	1.0	0.01	#NUM!	7.5E-07
YIL090W	YIL090W	S288C_ctrl_hmg124h_7.JPG.dat	0.0	0.0	0.0	0.00	#NUM!	
YIL092W	YIL092W	S288C_ctrl_hmg124h_7.JPG.dat	1168.0	27.3	1.0	0.03	#NUM!	4.8E-06
YIL093C	YIL093C	S288C_ctrl_hmg124h_7.JPG.dat	1117.0	26.8	1.0	0.01	#NUM!	5.5E-07
YIL095W	YIL095W	S288C_ctrl_hmg124h_7.JPG.dat	1169.0	43.4	1.0	0.06	#NUM!	5.0E-05
YIL096C	YIL096C	S288C_ctrl_hmg124h_7.JPG.dat	1274.5	41.7	1.1	0.03	#NUM!	4.2E-06
YIL097W	YIL097W	S288C_ctrl_hmg124h_7.JPG.dat	1131.5	34.1	0.9	0.03	#NUM!	6.7E-06
YIL098C	YIL098C	S288C_ctrl_hmg124h_7.JPG.dat	1182.8	32.7	1.0	0.03	#NUM!	5.5E-06
YIL099W	YIL099W	S288C_ctrl_hmg124h_7.JPG.dat	1197.5	49.3	1.0	0.04	#NUM!	1.3E-05
YIL100W	YIL100W	S288C_ctrl_hmg124h_7.JPG.dat	1204.5	22.1	1.0	0.02	#NUM!	1.2E-06
YIL101C	YIL101C	S288C_ctrl_hmg124h_7.JPG.dat	1091.8	22.1	1.0	0.02	#NUM!	4.0E-06
YIL103W	YIL103W	S288C_ctrl_hmg124h_7.JPG.dat	1055.5	16.1	1.0	0.01	#NUM!	1.0E-06
YIL105C	YIL105C	S288C_ctrl_hmg124h_7.JPG.dat	1192.0	32.6	1.1	0.03	#NUM!	5.0E-06
YIL107C	YIL107C	S288C_ctrl_hmg124h_7.JPG.dat	1143.3	33.5	1.0	0.00	#NUM!	2.8E-06
YIL108W	YIL108W	S288C_ctrl_hmg124h_7.JPG.dat	1186.5	25.0	1.0	0.02	#NUM!	2.9E-06
YIL110W	YIL110W	S288C_ctrl_hmg124h_7.JPG.dat	729.0	250.8	0.6	0.22	#NUM!	1.0E-02
YIL112W	YIL112W	S288C_ctrl_hmg124h_7.JPG.dat	1207.0	55.5	1.0	0.04	#NUM!	1.9E-05
YIL113W	YIL113W	S288C_ctrl_hmg124h_7.JPG.dat	1257.0	21.7	1.0	0.02	#NUM!	1.5E-06
YIL114C	YIL114C	S288C_ctrl_hmg124h_7.JPG.dat	1249.0	32.1	1.0	0.03	#NUM!	4.2E-06
YIL116W	YIL116W	S288C_ctrl_hmg124h_7.JPG.dat	0.0	0.0	0.0	0.00	#NUM!	
YIL117C	YIL117C	S288C_ctrl_hmg124h_7.JPG.dat	1254.0	43.0	1.0	0.01	#NUM!	2.3E-05
YIL119C	YIL119C	S288C_ctrl_hmg124h_7.JPG.dat	1188.5	35.5	1.0	0.03	#NUM!	5.5E-06
YIL120W	YIL120W	S288C_ctrl_hmg124h_7.JPG.dat	1096.5	65.2	1.0	0.06	#NUM!	5.2E-05
YIL123W	YIL123W	S288C_ctrl_hmg124h_7.JPG.dat	1129.5	25.9	1.0	0.02	#NUM!	3.2E-06
YIL124W	YIL124W	S288C_ctrl_hmg124h_7.JPG.dat	1108.3	27.3	1.0	0.03	#NUM!	7.8E-06
YIL128W	YIL128W	S288C_ctrl_hmg124h_7.JPG.dat	607.0	103.8	0.5	0.01	#NUM!	2.3E-04
YIL130W	YIL130W	S288C_ctrl_hmg124h_7.JPG.dat	1125.5	34.8	1.0	0.03	#NUM!	4.7E-06
YIL132C	YIL132C	S288C_ctrl_hmg124h_7.JPG.dat	1188.0	78.7	1.0	0.06	#NUM!	7.2E-05
YIL133C	YIL133C	S288C_ctrl_hmg124h_7.JPG.dat	1101.8	91.5	0.9	0.08	#NUM!	1.7E-04
YIL134W	YIL134W	S288C_ctrl_hmg124h_7.JPG.dat	1197.8	30.9	1.0	0.02	#NUM!	4.0E-06
YIL135C	YIL135C	S288C_ctrl_hmg124h_7.JPG.dat	1281.5	53.0	1.0	0.04	#NUM!	1.7E-05
YIL137C	YIL137C	S288C_ctrl_hmg124h_7.JPG.dat	1183.5	31.3	1.0	0.01	#NUM!	1.8E-05
YIL138C	YIL138C	S288C_ctrl_hmg124h_7.JPG.dat	1183.3	49.9	1.0	0.05	#NUM!	2.8E-05
YIL139C	YIL139C	S288C_ctrl_hmg124h_7.JPG.dat	1109.5	72.4	1.0	0.06	#NUM!	7.3E-05
YIL140W	YIL140W	S288C_ctrl_hmg124h_7.JPG.dat	1098.0	54.0	1.0	0.05	#NUM!	3.5E-05
YIL141W	YIL141W	S288C_ctrl_hmg124h_7.JPG.dat	1181.8	31.5	1.1	0.03	#NUM!	3.4E-06
YIL145C	YIL145C	S288C_ctrl_hmg124h_7.JPG.dat	1094.5	74.1	1.0	0.07	#NUM!	8.3E-05
YIL146C	YIL146C	S288C_ctrl_hmg124h_7.JPG.dat	1098.3	50.2	1.0	0.01	#NUM!	3.5E-05
YIL148W	YIL148W	S288C_ctrl_hmg124h_7.JPG.dat	854.8	337.3	0.9	0.04	#NUM!	8.6E-04
YIL149C	YIL149C	S288C_ctrl_hmg124h_7.JPG.dat	1193.8	60.6	1.0	0.05	#NUM!	3.5E-05
YIL152W	YIL152W	S288C_ctrl_hmg124h_7.JPG.dat	1210.8	32.6	1.0	0.03	#NUM!	5.0E-06
YIL153W	YIL153W	S288C_ctrl_hmg124h_7.JPG.dat	1067.5	123.1	0.9	0.04	#NUM!	7.0E-04
YIL154C	YIL154C	S288C_ctrl_hmg124h_7.JPG.dat	975.0	20.1	0.8	0.02	#NUM!	3.2E-06
YIL155C	YIL155C	S288C_ctrl_hmg124h_7.JPG.dat	1031.8	48.0	0.9	0.04	#NUM!	2.7E-05
YIL156W	YIL156W	S288C_ctrl_hmg124h_7.JPG.dat	1138.3	30.7	1.1	0.01	#NUM!	2.4E-05
YIL157C	YIL157C	S288C_ctrl_hmg124h_7.JPG.dat	1101.0	51.2	1.0	0.05	#NUM!	2.9E-05
YIL159W	YIL159W	S288C_ctrl_hmg124h_7.JPG.dat	1104.5	65.7	1.0	0.06	#NUM!	5.3E-05
YIL160C	YIL160C	S288C_ctrl_hmg124h_7.JPG.dat	1105.3	64.7	1.0	0.06	#NUM!	6.3E-05
YIL161W	YIL161W	S288C_ctrl_hmg124h_7.JPG.dat	1076.5	40.7	1.0	0.01	#NUM!	3.6E-05
YIL162W	YIL162W	S288C_ctrl_hmg124h_7.JPG.dat	1223.0	59.3	1.0	0.05	#NUM!	3.8E-05
YIL163C	YIL163C	S288C_ctrl_hmg124h_7.JPG.dat	1219.5	75.7	1.0	0.06	#NUM!	6.6E-05
YIL164C	YIL164C	S288C_ctrl_hmg124h_7.JPG.dat	1210.3	56.5	1.0	0.05	#NUM!	3.3E-05
YIL165C	YIL165C	S288C_ctrl_hmg124h_7.JPG.dat	1221.0	55.0	1.0	0.05	#NUM!	3.9E-05
YIL166C	YIL166C	S288C_ctrl_hmg124h_7.JPG.dat	1234.0	65.8	1.1	0.02	#NUM!	8.4E-05
YIL167W	YIL167W	S288C_ctrl_hmg124h_7.JPG.dat	1183.8	43.0	1.0	0.01	#NUM!	1.5E-05
YIL168W	YIL168W	S288C_ctrl_hmg124h_7.JPG.dat	1122.0	94.6	1.0	0.08	#NUM!	1.4E-04
YIL170W	YIL170W	S288C_ctrl_hmg124h_7.JPG.dat	1157.3	35.2	1.0	0.03	#NUM!	6.9E-06
YIL173W	YIL173W	S288C_ctrl_hmg124h_7.JPG.dat	1073.3	71.8	1.0	0.06	#NUM!	6.0E-05
YIR001C	YIR001C	S288C_ctrl_hmg124h_7.JPG.dat	1058.8	158.3	1.0	0.03	#NUM!	2.8E-04
YIR002C	YIR002C	S288C_ctrl_hmg124h_7.JPG.dat	1021.8	47.3	0.9	0.01	#NUM!	5.9E-05
YIR003W	YIR003W	S288C_ctrl_hmg124h_7.JPG.dat	1235.8	41.2	1.0	0.03	#NUM!	8.4E-06
YIR005W	YIR005W	S288C_ctrl_hmg124h_7.JPG.dat	1060.5	30.1	0.9	0.01	#NUM!	1.1E-06

YIR007W	YIR007W	S288C_ctrl_hmg124h_7.JPG.dat	1222.8	36.4	1.0	0.02	#NUM!	3.8E-06
YIR009W	YIR009W	S288C_ctrl_hmg124h_7.JPG.dat	1254.3	47.7	1.0	0.04	#NUM!	1.6E-05
YIR013C	YIR013C	S288C_ctrl_hmg124h_7.JPG.dat	1178.8	54.6	1.0	0.01	#NUM!	1.6E-05
YIR014W	YIR014W	S288C_ctrl_hmg124h_7.JPG.dat	1188.3	21.5	1.0	0.00	#NUM!	1.1E-06
YIR016W	YIR016W	S288C_ctrl_hmg124h_7.JPG.dat	1158.0	22.6	1.0	0.01	#NUM!	4.7E-07
YIR017C	YIR017C	S288C_ctrl_hmg124h_7.JPG.dat	1185.3	40.4	1.0	0.03	#NUM!	6.7E-06
YIR018W	YIR018W	S288C_ctrl_hmg124h_7.JPG.dat	1170.3	27.1	1.0	0.02	#NUM!	3.5E-06
YIR019C	YIR019C	S288C_ctrl_hmg124h_7.JPG.dat	1120.5	77.6	1.0	0.00	#NUM!	2.1E-06
YIR020C	YIR020C	S288C_ctrl_hmg124h_7.JPG.dat	1183.3	100.0	1.1	0.00	#NUM!	6.3E-07
YIR020W-E	YIR020W-B	S288C_ctrl_hmg124h_7.JPG.dat	1214.5	41.3	1.0	0.02	#NUM!	2.3E-06
YIR023W	YIR023W	S288C_ctrl_hmg124h_7.JPG.dat	1158.3	34.8	1.0	0.01	#NUM!	9.6E-06
YIR024C	YIR024C	S288C_ctrl_hmg124h_7.JPG.dat	1146.0	31.7	1.0	0.03	#NUM!	5.4E-06
YIR025W	YIR025W	S288C_ctrl_hmg124h_7.JPG.dat	1192.3	78.1	1.0	0.06	#NUM!	7.3E-05
YIR027C	YIR027C	S288C_ctrl_hmg124h_7.JPG.dat	1130.0	120.0	1.0	0.04	#NUM!	5.0E-04
YIR028W	YIR028W	S288C_ctrl_hmg124h_7.JPG.dat	1133.5	48.8	1.0	0.03	#NUM!	1.2E-05
YIR029W	YIR029W	S288C_ctrl_hmg124h_7.JPG.dat	1158.8	20.3	1.0	0.01	#NUM!	4.4E-07
YIR030C	YIR030C	S288C_ctrl_hmg124h_7.JPG.dat	1163.3	48.4	1.0	0.04	#NUM!	1.4E-05
YIR031C	YIR031C	S288C_ctrl_hmg124h_7.JPG.dat	1209.8	56.4	1.0	0.05	#NUM!	2.7E-05
YIR032C	YIR032C	S288C_ctrl_hmg124h_7.JPG.dat	1158.3	16.3	1.0	0.02	#NUM!	8.9E-07
YIR033W	YIR033W	S288C_ctrl_hmg124h_7.JPG.dat	1115.3	33.9	1.0	0.02	#NUM!	2.3E-06
YIR034C	YIR034C	S288C_ctrl_hmg124h_7.JPG.dat	0.0	0.0	0.0	0.00	#NUM!	
YIR035C	YIR035C	S288C_ctrl_hmg124h_7.JPG.dat	1234.0	23.1	1.0	0.02	#NUM!	3.2E-06
YIR036C	YIR036C	S288C_ctrl_hmg124h_7.JPG.dat	1278.0	10.9	1.1	0.01	#NUM!	6.4E-08
YIR037W	YIR037W	S288C_ctrl_hmg124h_7.JPG.dat	1228.0	87.0	1.0	0.07	#NUM!	8.0E-05
YIR038C	YIR038C	S288C_ctrl_hmg124h_7.JPG.dat	1196.5	10.8	1.0	0.01	#NUM!	5.4E-07
YIR039C	YIR039C	S288C_ctrl_hmg124h_7.JPG.dat	1162.0	68.0	1.0	0.02	#NUM!	7.5E-05
YIR042C	YIR042C	S288C_ctrl_hmg124h_7.JPG.dat	1062.0	60.0	1.0	0.05	#NUM!	4.4E-05
YIR043C	YIR043C	S288C_ctrl_hmg124h_7.JPG.dat	1161.5	23.1	1.0	0.02	#NUM!	1.3E-06
YIR044C	YIR044C	S288C_ctrl_hmg124h_7.JPG.dat	1083.3	67.2	1.0	0.06	#NUM!	6.5E-05
YJL004C	YJL004C	S288C_ctrl_hmg124h_7.JPG.dat	892.5	139.0	0.8	0.13	#NUM!	1.1E-03
YJL007C	YJL007C	S288C_ctrl_hmg124h_7.JPG.dat	1176.8	40.5	1.0	0.04	#NUM!	1.2E-05
YJL012C	YJL012C	S288C_ctrl_hmg124h_7.JPG.dat	1238.0	31.4	1.0	0.03	#NUM!	7.2E-06
YJL013C	YJL013C	S288C_ctrl_hmg124h_7.JPG.dat	1183.5	53.6	1.0	0.04	#NUM!	2.6E-05
YJL016W	YJL016W	S288C_ctrl_hmg124h_7.JPG.dat	1257.8	14.8	1.0	0.01	#NUM!	3.3E-07
YJL017W	YJL017W	S288C_ctrl_hmg124h_7.JPG.dat	1292.3	16.7	1.0	0.01	#NUM!	4.2E-07
YJL020C	YJL020C	S288C_ctrl_hmg124h_7.JPG.dat	1206.0	46.1	1.0	0.01	#NUM!	4.5E-05
YJL021C	YJL021C	S288C_ctrl_hmg124h_7.JPG.dat	1169.8	40.8	1.0	0.04	#NUM!	1.5E-05
YJL022W	YJL022W	S288C_ctrl_hmg124h_7.JPG.dat	1151.5	28.5	1.0	0.02	#NUM!	3.0E-06
YJL023C	YJL023C	S288C_ctrl_hmg124h_7.JPG.dat	1126.5	38.1	1.0	0.03	#NUM!	8.5E-06
YJL024C	YJL024C	S288C_ctrl_hmg124h_7.JPG.dat	1070.3	23.1	1.0	0.02	#NUM!	2.0E-06
YJL027C	YJL027C	S288C_ctrl_hmg124h_7.JPG.dat	1091.0	92.0	1.0	0.08	#NUM!	1.3E-04
YJL028W	YJL028W	S288C_ctrl_hmg124h_7.JPG.dat	901.3	599.1	1.0	0.03	#NUM!	3.6E-04
YJL030W	YJL030W	S288C_ctrl_hmg124h_7.JPG.dat	1244.8	32.2	1.0	0.03	#NUM!	4.7E-06
YJL036W	YJL036W	S288C_ctrl_hmg124h_7.JPG.dat	1146.5	18.7	1.0	0.02	#NUM!	1.4E-06
YJL037W	YJL037W	S288C_ctrl_hmg124h_7.JPG.dat	1252.8	42.0	1.0	0.03	#NUM!	1.1E-05
YJL038C	YJL038C	S288C_ctrl_hmg124h_7.JPG.dat	1255.3	42.0	1.0	0.00	#NUM!	7.7E-06
YJL042W	YJL042W	S288C_ctrl_hmg124h_7.JPG.dat	1235.0	55.2	1.0	0.01	#NUM!	3.9E-05
YJL043W	YJL043W	S288C_ctrl_hmg124h_7.JPG.dat	1210.3	25.4	1.0	0.02	#NUM!	2.1E-06
YJL044C	YJL044C	S288C_ctrl_hmg124h_7.JPG.dat	1105.5	25.0	1.0	0.00	#NUM!	6.0E-06
YJL045W	YJL045W	S288C_ctrl_hmg124h_7.JPG.dat	1097.5	29.7	1.0	0.01	#NUM!	2.0E-05
YJL046W	YJL046W	S288C_ctrl_hmg124h_7.JPG.dat	789.0	66.6	0.7	0.06	#NUM!	1.5E-04
YJL047C	YJL047C	S288C_ctrl_hmg124h_7.JPG.dat	1008.3	60.0	0.9	0.06	#NUM!	5.7E-05
YJL048C	YJL048C	S288C_ctrl_hmg124h_7.JPG.dat	1155.3	29.5	1.0	0.02	#NUM!	3.9E-06
YJL049W	YJL049W	S288C_ctrl_hmg124h_7.JPG.dat	1178.0	23.9	1.0	0.02	#NUM!	3.9E-06
YJL051W	YJL051W	S288C_ctrl_hmg124h_7.JPG.dat	1306.5	43.7	1.1	0.00	#NUM!	6.8E-06
YJL053W	YJL053W	S288C_ctrl_hmg124h_7.JPG.dat	1079.5	66.4	0.9	0.06	#NUM!	6.3E-05
YJL055W	YJL055W	S288C_ctrl_hmg124h_7.JPG.dat	1209.8	10.8	1.0	0.01	#NUM!	1.5E-07
YJL057C	YJL057C	S288C_ctrl_hmg124h_7.JPG.dat	1258.5	27.2	1.0	0.01	#NUM!	1.7E-05
YJL058C	YJL058C	S288C_ctrl_hmg124h_7.JPG.dat	1189.8	53.4	1.1	0.01	#NUM!	3.9E-05
YJL059W	YJL059W	S288C_ctrl_hmg124h_7.JPG.dat	1133.0	54.7	1.0	0.01	#NUM!	5.1E-05
YJL060W	YJL060W	S288C_ctrl_hmg124h_7.JPG.dat	1020.8	30.8	1.0	0.03	#NUM!	6.3E-06
YJL062W	YJL062W	S288C_ctrl_hmg124h_7.JPG.dat	132.8	265.5	0.0	0.00	#NUM!	
YJL064W	YJL064W	S288C_ctrl_hmg124h_7.JPG.dat	1027.0	16.3	1.0	0.02	#NUM!	1.7E-06
YJL065C	YJL065C	S288C_ctrl_hmg124h_7.JPG.dat	1078.0	39.6	1.0	0.03	#NUM!	6.0E-06
YJL066C	YJL066C	S288C_ctrl_hmg124h_7.JPG.dat	1197.8	42.2	1.0	0.01	#NUM!	5.4E-05
YJL067W	YJL067W	S288C_ctrl_hmg124h_7.JPG.dat	1280.0	41.8	1.1	0.01	#NUM!	2.3E-05
YJL068C	YJL068C	S288C_ctrl_hmg124h_7.JPG.dat	1279.3	58.8	1.0	0.05	#NUM!	3.7E-05
YJL070C	YJL070C	S288C_ctrl_hmg124h_7.JPG.dat	1257.0	50.1	1.0	0.05	#NUM!	2.5E-05
YJL071W	YJL071W	S288C_ctrl_hmg124h_7.JPG.dat	299.3	598.5	0.0	0.00	#NUM!	
YJL073W	YJL073W	S288C_ctrl_hmg124h_7.JPG.dat	1206.3	29.8	1.0	0.02	#NUM!	3.8E-06

YJL077C	YJL077C	S288C_ctrl_hmg124h_7.JPG.dat	1176.0	32.3	1.0	0.03	#NUM!	4.2E-06
YJL078C	YJL078C	S288C_ctrl_hmg124h_7.JPG.dat	1099.0	88.4	1.0	0.02	#NUM!	1.8E-04
YJL083W	YJL083W	S288C_ctrl_hmg124h_7.JPG.dat	1132.8	66.5	1.0	0.02	#NUM!	1.0E-04
YJL084C	YJL084C	S288C_ctrl_hmg124h_7.JPG.dat	1068.5	38.3	1.0	0.04	#NUM!	1.3E-05
YJL088W	YJL088W	S288C_ctrl_hmg124h_7.JPG.dat	598.0	691.2	0.5	0.58	#NUM!	1.8E-01
YJL089W	YJL089W	S288C_ctrl_hmg124h_7.JPG.dat	1292.3	26.6	1.0	0.04	#NUM!	1.6E-05
YJL093C	YJL093C	S288C_ctrl_hmg124h_7.JPG.dat	1235.5	34.8	1.0	0.03	#NUM!	1.0E-05
YJL094C	YJL094C	S288C_ctrl_hmg124h_7.JPG.dat	1246.0	53.2	1.0	0.05	#NUM!	2.6E-05
YJL095W	YJL095W	S288C_ctrl_hmg124h_7.JPG.dat	1256.5	96.0	1.0	0.02	#NUM!	1.4E-04
YJL098W	YJL098W	S288C_ctrl_hmg124h_7.JPG.dat	1261.5	58.6	1.0	0.05	#NUM!	2.3E-05
YJL099W	YJL099W	S288C_ctrl_hmg124h_7.JPG.dat	1292.0	29.7	1.1	0.03	#NUM!	4.8E-06
YJL100W	YJL100W	S288C_ctrl_hmg124h_7.JPG.dat	1179.3	16.7	1.0	0.02	#NUM!	3.5E-06
YJL101C	YJL101C	S288C_ctrl_hmg124h_7.JPG.dat	1166.3	57.5	1.0	0.05	#NUM!	3.6E-05
YJL105W	YJL105W	S288C_ctrl_hmg124h_7.JPG.dat	1186.5	61.3	1.0	0.05	#NUM!	3.4E-05
YJL108C	YJL108C	S288C_ctrl_hmg124h_7.JPG.dat	1045.0	294.2	0.9	0.23	#NUM!	4.9E-03
YJL110C	YJL110C	S288C_ctrl_hmg124h_7.JPG.dat	1324.3	86.4	1.1	0.07	#NUM!	7.8E-05
YJL112W	YJL112W	S288C_ctrl_hmg124h_7.JPG.dat	1143.8	60.6	1.0	0.05	#NUM!	2.9E-05
YJL115W	YJL115W	S288C_ctrl_hmg124h_7.JPG.dat	743.5	84.4	0.6	0.03	#NUM!	6.5E-04
YJL116C	YJL116C	S288C_ctrl_hmg124h_7.JPG.dat	1177.3	38.2	1.0	0.03	#NUM!	9.0E-06
YJL121C	YJL121C	S288C_ctrl_hmg124h_7.JPG.dat	1031.0	60.8	0.9	0.05	#NUM!	5.7E-05
YJL122W	YJL122W	S288C_ctrl_hmg124h_7.JPG.dat	1149.3	23.9	1.0	0.02	#NUM!	2.1E-06
YJL123C	YJL123C	S288C_ctrl_hmg124h_7.JPG.dat	1071.0	39.9	1.0	0.03	#NUM!	1.3E-05
YJL124C	YJL124C	S288C_ctrl_hmg124h_7.JPG.dat	980.8	30.3	0.9	0.03	#NUM!	8.1E-06
YJL126W	YJL126W	S288C_ctrl_hmg124h_7.JPG.dat	1207.3	32.3	1.1	0.03	#NUM!	5.5E-06
YJL128C	YJL128C	S288C_ctrl_hmg124h_7.JPG.dat	1125.5	25.2	1.0	0.02	#NUM!	2.0E-06
YJL130C	YJL130C	S288C_ctrl_hmg124h_7.JPG.dat	1099.3	26.1	1.0	0.02	#NUM!	3.9E-06
YJL131C	YJL131C	S288C_ctrl_hmg124h_7.JPG.dat	1181.3	28.5	1.1	0.04	#NUM!	1.5E-05
YJL132W	YJL132W	S288C_ctrl_hmg124h_7.JPG.dat	1253.8	70.8	1.0	0.05	#NUM!	2.9E-05
YJL133W	YJL133W	S288C_ctrl_hmg124h_7.JPG.dat	1232.5	59.8	1.0	0.05	#NUM!	2.3E-05
YJL134W	YJL134W	S288C_ctrl_hmg124h_7.JPG.dat	1181.0	69.2	1.0	0.05	#NUM!	4.4E-05
YJL135W	YJL135W	S288C_ctrl_hmg124h_7.JPG.dat	1226.5	15.8	1.0	0.02	#NUM!	9.0E-07
YJL136C	YJL136C	S288C_ctrl_hmg124h_7.JPG.dat	910.0	47.7	0.8	0.04	#NUM!	3.4E-05
YJL137C	YJL137C	S288C_ctrl_hmg124h_7.JPG.dat	1155.3	8.0	1.0	0.01	#NUM!	1.5E-07
YJL138C	YJL138C	S288C_ctrl_hmg124h_7.JPG.dat	1000.5	57.7	0.9	0.05	#NUM!	4.1E-05
YJL139C	YJL139C	S288C_ctrl_hmg124h_7.JPG.dat	810.0	547.7	0.9	0.11	#NUM!	4.2E-03
YJL141C	YJL141C	S288C_ctrl_hmg124h_7.JPG.dat	1072.8	86.0	0.9	0.08	#NUM!	1.4E-04
YJL142C	YJL142C	S288C_ctrl_hmg124h_7.JPG.dat	1191.5	15.4	1.0	0.01	#NUM!	3.3E-07
YJL144W	YJL144W	S288C_ctrl_hmg124h_7.JPG.dat	1209.0	29.5	1.1	0.03	#NUM!	6.4E-06
YJL145W	YJL145W	S288C_ctrl_hmg124h_7.JPG.dat	1230.8	17.7	1.0	0.01	#NUM!	2.0E-07
YJL146W	YJL146W	S288C_ctrl_hmg124h_7.JPG.dat	1224.3	38.8	1.0	0.03	#NUM!	1.1E-05
YJL147C	YJL147C	S288C_ctrl_hmg124h_7.JPG.dat	1163.8	47.5	0.9	0.04	#NUM!	1.8E-05
YJL148W	YJL148W	S288C_ctrl_hmg124h_7.JPG.dat	1180.5	101.7	1.0	0.08	#NUM!	1.6E-04
YJL149W	YJL149W	S288C_ctrl_hmg124h_7.JPG.dat	1169.3	21.6	1.0	0.02	#NUM!	2.2E-06
YJL150W	YJL150W	S288C_ctrl_hmg124h_7.JPG.dat	1156.5	41.3	1.0	0.01	#NUM!	6.5E-05
YJL151C	YJL151C	S288C_ctrl_hmg124h_7.JPG.dat	1087.3	77.6	1.0	0.01	#NUM!	2.5E-05
YJL152W	YJL152W	S288C_ctrl_hmg124h_7.JPG.dat	1118.8	38.2	1.0	0.01	#NUM!	1.5E-05
YJL153C	YJL153C	S288C_ctrl_hmg124h_7.JPG.dat	1032.3	101.2	0.9	0.09	#NUM!	2.8E-04
YJL154C	YJL154C	S288C_ctrl_hmg124h_7.JPG.dat	1263.0	31.3	1.1	0.03	#NUM!	8.6E-06
YJL155C	YJL155C	S288C_ctrl_hmg124h_7.JPG.dat	1162.5	30.3	1.0	0.03	#NUM!	4.3E-06
YJL157C	YJL157C	S288C_ctrl_hmg124h_7.JPG.dat	1174.0	59.9	1.0	0.04	#NUM!	2.5E-05
YJL158C	YJL158C	S288C_ctrl_hmg124h_7.JPG.dat	1278.3	58.6	1.1	0.04	#NUM!	1.3E-05
YJL159W	YJL159W	S288C_ctrl_hmg124h_7.JPG.dat	1300.8	49.0	1.1	0.00	#NUM!	5.3E-06
YJL160C	YJL160C	S288C_ctrl_hmg124h_7.JPG.dat	619.8	717.5	0.5	0.59	#NUM!	1.8E-01
YJL161W	YJL161W	S288C_ctrl_hmg124h_7.JPG.dat	1237.3	17.7	1.0	0.00	#NUM!	1.6E-06
YJL162C	YJL162C	S288C_ctrl_hmg124h_7.JPG.dat	1138.8	67.3	1.0	0.07	#NUM!	8.0E-05
YJL163C	YJL163C	S288C_ctrl_hmg124h_7.JPG.dat	1075.0	23.2	1.0	0.03	#NUM!	8.8E-06
YJL164C	YJL164C	S288C_ctrl_hmg124h_7.JPG.dat	1043.8	116.8	0.9	0.10	#NUM!	3.7E-04
YJL165C	YJL165C	S288C_ctrl_hmg124h_7.JPG.dat	846.0	140.1	0.8	0.13	#NUM!	1.3E-03
YJL168C	YJL168C	S288C_ctrl_hmg124h_7.JPG.dat	975.5	34.7	0.9	0.01	#NUM!	3.5E-05
YJL169W	YJL169W	S288C_ctrl_hmg124h_7.JPG.dat	954.5	110.4	0.8	0.02	#NUM!	2.2E-04
YJL170C	YJL170C	S288C_ctrl_hmg124h_7.JPG.dat	1244.8	53.8	1.0	0.04	#NUM!	1.4E-05
YJL171C	YJL171C	S288C_ctrl_hmg124h_7.JPG.dat	1186.3	58.8	1.0	0.05	#NUM!	3.3E-05
YJL172W	YJL172W	S288C_ctrl_hmg124h_7.JPG.dat	1112.3	51.6	0.9	0.04	#NUM!	2.8E-05
YJL175W	YJL175W	S288C_ctrl_hmg124h_7.JPG.dat	731.3	105.4	0.6	0.02	#NUM!	4.4E-04
YJL176C	YJL176C	S288C_ctrl_hmg124h_7.JPG.dat	1250.0	109.1	1.1	0.01	#NUM!	5.5E-05
YJL178C	YJL178C	S288C_ctrl_hmg124h_7.JPG.dat	1125.3	85.0	0.9	0.02	#NUM!	1.5E-04
YJL181W	YJL181W	S288C_ctrl_hmg124h_7.JPG.dat	1076.8	52.9	1.0	0.05	#NUM!	3.0E-05
YJL182C	YJL182C	S288C_ctrl_hmg124h_7.JPG.dat	1117.5	98.7	1.0	0.09	#NUM!	1.9E-04
YJL183W	YJL183W	S288C_ctrl_hmg124h_7.JPG.dat	1132.8	76.7	1.0	0.07	#NUM!	9.7E-05
YJL185C	YJL185C	S288C_ctrl_hmg124h_7.JPG.dat	1010.5	212.2	0.9	0.19	#NUM!	2.4E-03

YJL186W	YJL186W	S288C_ctrl_hmg124h_7.JPG.dat	1129.8	52.4	1.0	0.04	#NUM!	1.7E-05
YJL187C	YJL187C	S288C_ctrl_hmg124h_7.JPG.dat	991.8	663.1	1.1	0.06	#NUM!	9.4E-04
YJL188C	YJL188C	S288C_ctrl_hmg124h_7.JPG.dat	1167.0	28.6	1.0	0.03	#NUM!	1.2E-05
YJL190C	YJL190C	S288C_ctrl_hmg124h_7.JPG.dat	1188.0	43.9	1.0	0.04	#NUM!	2.2E-05
YJL191W	YJL191W	S288C_ctrl_hmg124h_7.JPG.dat	1155.3	52.2	1.0	0.04	#NUM!	2.4E-05
YJL192C	YJL192C	S288C_ctrl_hmg124h_7.JPG.dat	1194.0	98.1	1.1	0.01	#NUM!	6.0E-05
YJL193W	YJL193W	S288C_ctrl_hmg124h_7.JPG.dat	1146.0	73.2	1.0	0.06	#NUM!	5.7E-05
YJL196C	YJL196C	S288C_ctrl_hmg124h_7.JPG.dat	1141.5	49.1	1.0	0.04	#NUM!	1.1E-05
YJL197W	YJL197W	S288C_ctrl_hmg124h_7.JPG.dat	1055.0	35.4	1.0	0.03	#NUM!	6.8E-06
YJL198W	YJL198W	S288C_ctrl_hmg124h_7.JPG.dat	988.0	293.8	1.1	0.02	#NUM!	1.4E-04
YJL199C	YJL199C	S288C_ctrl_hmg124h_7.JPG.dat	873.5	587.2	1.1	0.08	#NUM!	1.7E-03
YJL201W	YJL201W	S288C_ctrl_hmg124h_7.JPG.dat	994.8	216.9	1.0	0.07	#NUM!	1.7E-03
YJL204C	YJL204C	S288C_ctrl_hmg124h_7.JPG.dat	590.3	579.2	0.5	0.46	#NUM!	1.4E-01
YJL206C	YJL206C	S288C_ctrl_hmg124h_7.JPG.dat	1272.3	15.9	1.0	0.02	#NUM!	1.3E-06
YJL206C-A	YJL206C-A	S288C_ctrl_hmg124h_7.JPG.dat	1227.5	50.3	1.0	0.03	#NUM!	9.0E-06
YJL207C	YJL207C	S288C_ctrl_hmg124h_7.JPG.dat	1247.8	23.2	1.0	0.02	#NUM!	1.2E-06
YJL208C	YJL208C	S288C_ctrl_hmg124h_7.JPG.dat	1277.8	50.8	1.0	0.04	#NUM!	1.7E-05
YJL210W	YJL210W	S288C_ctrl_hmg124h_7.JPG.dat	1158.3	61.6	1.0	0.00	#NUM!	6.4E-06
YJL211C	YJL211C	S288C_ctrl_hmg124h_7.JPG.dat	1125.0	9.8	1.0	0.00	#NUM!	6.0E-06
YJL212C	YJL212C	S288C_ctrl_hmg124h_7.JPG.dat	1172.5	78.2	1.0	0.01	#NUM!	6.1E-05
YJL213W	YJL213W	S288C_ctrl_hmg124h_7.JPG.dat	1185.0	21.8	1.0	0.02	#NUM!	1.4E-06
YJL214W	YJL214W	S288C_ctrl_hmg124h_7.JPG.dat	1161.0	47.8	1.0	0.04	#NUM!	1.4E-05
YJL215C	YJL215C	S288C_ctrl_hmg124h_7.JPG.dat	1231.8	89.9	1.0	0.06	#NUM!	5.4E-05
YJL216C	YJL216C	S288C_ctrl_hmg124h_7.JPG.dat	1205.3	39.7	1.0	0.01	#NUM!	3.8E-05
YJL217W	YJL217W	S288C_ctrl_hmg124h_7.JPG.dat	1202.5	36.8	1.0	0.03	#NUM!	5.6E-06
YJL218W	YJL218W	S288C_ctrl_hmg124h_7.JPG.dat	1155.0	22.0	1.0	0.02	#NUM!	2.0E-06
YJR001W	YJR001W	S288C_ctrl_hmg124h_7.JPG.dat	1150.3	35.0	1.0	0.03	#NUM!	7.1E-06
YJR003C	YJR003C	S288C_ctrl_hmg124h_7.JPG.dat	1154.8	15.0	1.0	0.00	#NUM!	7.4E-06
YJR005W	YJR005W	S288C_ctrl_hmg124h_7.JPG.dat	1134.3	37.2	1.0	0.04	#NUM!	1.4E-05
YJR008W	YJR008W	S288C_ctrl_hmg124h_7.JPG.dat	1149.8	39.2	1.1	0.01	#NUM!	2.9E-05
YJR009C	YJR009C	S288C_ctrl_hmg124h_7.JPG.dat	1167.5	25.2	1.0	0.02	#NUM!	2.8E-06
YJR010C-A	YJR010C-A	S288C_ctrl_hmg124h_7.JPG.dat	1143.8	25.6	1.0	0.02	#NUM!	2.4E-06
YJR010W	YJR010W	S288C_ctrl_hmg124h_7.JPG.dat	1130.8	11.9	1.0	0.01	#NUM!	6.9E-07
YJR011C	YJR011C	S288C_ctrl_hmg124h_7.JPG.dat	1064.8	70.2	0.9	0.06	#NUM!	7.8E-05
YJR014W	YJR014W	S288C_ctrl_hmg124h_7.JPG.dat	1146.8	28.1	1.0	0.03	#NUM!	6.7E-06
YJR015W	YJR015W	S288C_ctrl_hmg124h_7.JPG.dat	1192.3	24.3	1.0	0.02	#NUM!	3.3E-06
YJR019C	YJR019C	S288C_ctrl_hmg124h_7.JPG.dat	1215.3	15.8	1.0	0.00	#NUM!	6.7E-06
YJR020W	YJR020W	S288C_ctrl_hmg124h_7.JPG.dat	1179.8	25.9	1.0	0.02	#NUM!	3.2E-06
YJR021C	YJR021C	S288C_ctrl_hmg124h_7.JPG.dat	1205.5	17.2	1.0	0.01	#NUM!	3.3E-07
YJR024C	YJR024C	S288C_ctrl_hmg124h_7.JPG.dat	1148.0	60.6	1.0	0.05	#NUM!	3.2E-05
YJR025C	YJR025C	S288C_ctrl_hmg124h_7.JPG.dat	1060.8	49.9	0.9	0.05	#NUM!	4.2E-05
YJR026W	YJR026W	S288C_ctrl_hmg124h_7.JPG.dat	1143.0	44.1	1.0	0.04	#NUM!	2.1E-05
YJR030C	YJR030C	S288C_ctrl_hmg124h_7.JPG.dat	1143.8	60.8	1.0	0.01	#NUM!	3.9E-05
YJR031C	YJR031C	S288C_ctrl_hmg124h_7.JPG.dat	1141.5	54.3	1.0	0.05	#NUM!	2.7E-05
YJR032W	YJR032W	S288C_ctrl_hmg124h_7.JPG.dat	267.5	535.0	0.0	0.00	#NUM!	
YJR033C	YJR033C	S288C_ctrl_hmg124h_7.JPG.dat	1263.5	31.0	1.0	0.02	#NUM!	3.2E-06
YJR035W	YJR035W	S288C_ctrl_hmg124h_7.JPG.dat	1207.5	28.0	1.0	0.03	#NUM!	4.5E-06
YJR036C	YJR036C	S288C_ctrl_hmg124h_7.JPG.dat	1266.0	25.5	1.0	0.02	#NUM!	2.0E-06
YJR037W	YJR037W	S288C_ctrl_hmg124h_7.JPG.dat	1275.0	71.6	1.0	0.06	#NUM!	5.0E-05
YJR038C	YJR038C	S288C_ctrl_hmg124h_7.JPG.dat	1207.0	26.8	1.0	0.02	#NUM!	2.7E-06
YJR040W	YJR040W	S288C_ctrl_hmg124h_7.JPG.dat	1206.3	85.6	1.1	0.02	#NUM!	1.0E-04
YJR043C	YJR043C	S288C_ctrl_hmg124h_7.JPG.dat	1137.5	28.9	1.0	0.03	#NUM!	6.9E-06
YJR047C	YJR047C	S288C_ctrl_hmg124h_7.JPG.dat	1140.0	41.1	1.0	0.04	#NUM!	2.0E-05
YJR048W	YJR048W	S288C_ctrl_hmg124h_7.JPG.dat	1110.5	32.0	1.0	0.00	#NUM!	8.3E-06
YJR049C	YJR049C	S288C_ctrl_hmg124h_7.JPG.dat	904.8	603.8	1.1	0.02	#NUM!	1.4E-04
YJR050W	YJR050W	S288C_ctrl_hmg124h_7.JPG.dat	1168.0	99.2	1.0	0.08	#NUM!	1.4E-04
YJR051W	YJR051W	S288C_ctrl_hmg124h_7.JPG.dat	1254.3	17.6	1.0	0.02	#NUM!	2.0E-06
YJR052W	YJR052W	S288C_ctrl_hmg124h_7.JPG.dat	1225.8	40.7	1.0	0.03	#NUM!	5.5E-06
YJR053W	YJR053W	S288C_ctrl_hmg124h_7.JPG.dat	1179.3	40.9	1.0	0.00	#NUM!	2.2E-06
YJR054W	YJR054W	S288C_ctrl_hmg124h_7.JPG.dat	1232.3	65.6	1.0	0.05	#NUM!	4.1E-05
YJR058C	YJR058C	S288C_ctrl_hmg124h_7.JPG.dat	1207.0	95.5	1.0	0.07	#NUM!	8.8E-05
YJR059W	YJR059W	S288C_ctrl_hmg124h_7.JPG.dat	1138.5	89.7	1.0	0.07	#NUM!	8.9E-05
YJR060W	YJR060W	S288C_ctrl_hmg124h_7.JPG.dat	1088.0	67.7	1.0	0.07	#NUM!	8.8E-05
YJR061W	YJR061W	S288C_ctrl_hmg124h_7.JPG.dat	1136.5	59.9	1.0	0.05	#NUM!	2.5E-05
YJR062C	YJR062C	S288C_ctrl_hmg124h_7.JPG.dat	1112.5	26.3	1.0	0.02	#NUM!	1.1E-06
YJR069C	YJR069C	S288C_ctrl_hmg124h_7.JPG.dat	1087.3	52.1	1.0	0.06	#NUM!	4.5E-05
YJR070C	YJR070C	S288C_ctrl_hmg124h_7.JPG.dat	981.3	213.7	1.0	0.02	#NUM!	2.1E-04
YJR073C	YJR073C	S288C_ctrl_hmg124h_7.JPG.dat	581.5	673.8	0.5	0.55	#NUM!	1.8E-01
YJR074W	YJR074W	S288C_ctrl_hmg124h_7.JPG.dat	925.3	6.4	0.8	0.01	#NUM!	3.2E-07
YJR075W	YJR075W	S288C_ctrl_hmg124h_7.JPG.dat	1349.5	57.0	1.1	0.05	#NUM!	2.2E-05

YJR077C	YJR077C	S288C_ctrl_hmg124h_7.JPG.dat	1166.0	67.6	1.0	0.05	#NUM!	4.8E-05
YJR078W	YJR078W	S288C_ctrl_hmg124h_7.JPG.dat	1211.0	40.1	1.0	0.03	#NUM!	9.7E-06
YJR079W	YJR079W	S288C_ctrl_hmg124h_7.JPG.dat	1180.8	72.4	1.0	0.07	#NUM!	8.1E-05
YJR080C	YJR080C	S288C_ctrl_hmg124h_7.JPG.dat	1059.5	67.1	1.0	0.06	#NUM!	7.6E-05
YJR082C	YJR082C	S288C_ctrl_hmg124h_7.JPG.dat	1044.3	57.0	1.0	0.05	#NUM!	4.1E-05
YJR083C	YJR083C	S288C_ctrl_hmg124h_7.JPG.dat	1149.3	67.6	1.1	0.02	#NUM!	8.4E-05
YJR084W	YJR084W	S288C_ctrl_hmg124h_7.JPG.dat	491.0	583.7	0.5	0.54	#NUM!	1.9E-01
YJR087W	YJR087W	S288C_ctrl_hmg124h_7.JPG.dat	939.3	263.5	1.0	0.03	#NUM!	2.5E-04
YJR088C	YJR088C	S288C_ctrl_hmg124h_7.JPG.dat	1135.3	73.0	0.9	0.06	#NUM!	8.3E-05
YJR090C	YJR090C	S288C_ctrl_hmg124h_7.JPG.dat	305.5	611.0	0.0	0.00	#NUM!	
YJR091C	YJR091C	S288C_ctrl_hmg124h_7.JPG.dat	1121.8	127.3	1.0	0.04	#NUM!	5.9E-04
YJR092W	YJR092W	S288C_ctrl_hmg124h_7.JPG.dat	1186.5	22.5	1.0	0.01	#NUM!	9.2E-06
YJR094C	YJR094C	S288C_ctrl_hmg124h_7.JPG.dat	1102.8	128.6	0.9	0.11	#NUM!	4.1E-04
YJR094W-A	YJR094W-A	S288C_ctrl_hmg124h_7.JPG.dat	1189.8	37.4	1.0	0.02	#NUM!	3.7E-06
YJR095W	YJR095W	S288C_ctrl_hmg124h_7.JPG.dat	982.0	133.8	0.9	0.12	#NUM!	6.2E-04
YJR096W	YJR096W	S288C_ctrl_hmg124h_7.JPG.dat	1016.8	62.7	0.9	0.05	#NUM!	4.9E-05
YJR097W	YJR097W	S288C_ctrl_hmg124h_7.JPG.dat	1051.0	89.4	0.9	0.03	#NUM!	4.5E-04
YJR098C	YJR098C	S288C_ctrl_hmg124h_7.JPG.dat	1143.5	31.2	1.1	0.00	#NUM!	3.5E-06
YJR099W	YJR099W	S288C_ctrl_hmg124h_7.JPG.dat	748.5	146.6	0.7	0.14	#NUM!	2.4E-03
YOR202W	YOR202W	S288C_ctrl_hmg124h_7.JPG.dat	1242.1	139.8	1.0	0.07	#NUM!	2.30762351
1	1	S288C_ctrl_hmg124h_12.JPG.dat	1430.5	197.6	1.1	0.12	#NUM!	3.4E-04
2	2	S288C_ctrl_hmg124h_12.JPG.dat	1180.8	48.1	1.0	0.03	#NUM!	8.4E-06
3	3	S288C_ctrl_hmg124h_12.JPG.dat	1184.5	75.7	1.0	0.02	#NUM!	1.9E-04
4	4	S288C_ctrl_hmg124h_12.JPG.dat	1102.8	45.2	0.9	0.05	#NUM!	3.1E-05
YNR019W	YNR019W	S288C_ctrl_hmg124h_12.JPG.dat	1317.3	47.1	1.0	0.03	#NUM!	9.7E-06
YNR020C	YNR020C	S288C_ctrl_hmg124h_12.JPG.dat	1280.3	16.3	1.0	0.01	#NUM!	8.1E-07
YNR021W	YNR021W	S288C_ctrl_hmg124h_12.JPG.dat	1234.5	20.0	1.0	0.02	#NUM!	1.3E-06
YNR022C	YNR022C	S288C_ctrl_hmg124h_12.JPG.dat	1212.3	28.5	1.0	0.00	#NUM!	4.2E-06
YNR024W	YNR024W	S288C_ctrl_hmg124h_12.JPG.dat	1204.5	15.2	1.0	0.01	#NUM!	5.8E-07
YNR025C	YNR025C	S288C_ctrl_hmg124h_12.JPG.dat	1172.8	33.6	1.0	0.01	#NUM!	3.3E-05
YNR027W	YNR027W	S288C_ctrl_hmg124h_12.JPG.dat	1192.8	28.1	1.0	0.02	#NUM!	1.4E-06
YNR028W	YNR028W	S288C_ctrl_hmg124h_12.JPG.dat	1216.3	29.8	1.0	0.02	#NUM!	2.7E-06
YNR029C	YNR029C	S288C_ctrl_hmg124h_12.JPG.dat	1167.0	43.8	0.9	0.03	#NUM!	1.4E-05
YNR030W	YNR030W	S288C_ctrl_hmg124h_12.JPG.dat	1265.5	41.6	1.0	0.03	#NUM!	7.3E-06
YNR031C	YNR031C	S288C_ctrl_hmg124h_12.JPG.dat	1289.0	21.0	1.1	0.02	#NUM!	1.3E-06
YNR032C-A	YNR032C-A	S288C_ctrl_hmg124h_12.JPG.dat	1303.3	30.1	1.0	0.01	#NUM!	2.5E-05
YNR032W	YNR032W	S288C_ctrl_hmg124h_12.JPG.dat	1336.8	40.7	1.0	0.04	#NUM!	1.1E-05
YNR034W	YNR034W	S288C_ctrl_hmg124h_12.JPG.dat	1307.3	49.4	1.0	0.04	#NUM!	1.3E-05
YNR039C	YNR039C	S288C_ctrl_hmg124h_12.JPG.dat	1179.8	19.5	1.0	0.02	#NUM!	1.2E-06
YNR040W	YNR040W	S288C_ctrl_hmg124h_12.JPG.dat	1160.3	15.9	1.0	0.01	#NUM!	4.5E-07
YNR042W	YNR042W	S288C_ctrl_hmg124h_12.JPG.dat	1106.3	36.7	1.0	0.03	#NUM!	1.0E-05
YNR045W	YNR045W	S288C_ctrl_hmg124h_12.JPG.dat	1152.0	8.8	1.0	0.01	#NUM!	2.3E-07
YNR047W	YNR047W	S288C_ctrl_hmg124h_12.JPG.dat	1201.3	67.2	1.0	0.01	#NUM!	6.8E-05
YNR048W	YNR048W	S288C_ctrl_hmg124h_12.JPG.dat	1301.5	22.8	1.0	0.02	#NUM!	2.0E-06
YNR049C	YNR049C	S288C_ctrl_hmg124h_12.JPG.dat	1150.0	204.5	1.0	0.03	#NUM!	2.7E-04
YNR050C	YNR050C	S288C_ctrl_hmg124h_12.JPG.dat	0.0	0.0	0.0	0.00	#NUM!	
YNR051C	YNR051C	S288C_ctrl_hmg124h_12.JPG.dat	1153.8	16.5	0.9	0.01	#NUM!	4.8E-07
YNR055C	YNR055C	S288C_ctrl_hmg124h_12.JPG.dat	1252.3	23.8	1.0	0.02	#NUM!	1.5E-06
YNR056C	YNR056C	S288C_ctrl_hmg124h_12.JPG.dat	1213.0	21.8	1.0	0.02	#NUM!	3.2E-06
YNR057C	YNR057C	S288C_ctrl_hmg124h_12.JPG.dat	1179.5	11.5	1.0	0.01	#NUM!	6.8E-08
YNR058W	YNR058W	S288C_ctrl_hmg124h_12.JPG.dat	1168.5	29.9	1.0	0.03	#NUM!	5.7E-06
YNR059W	YNR059W	S288C_ctrl_hmg124h_12.JPG.dat	1058.0	26.4	0.9	0.01	#NUM!	1.4E-05
YNR060W	YNR060W	S288C_ctrl_hmg124h_12.JPG.dat	1105.3	22.5	1.0	0.01	#NUM!	3.6E-05
YNR061C	YNR061C	S288C_ctrl_hmg124h_12.JPG.dat	1173.0	44.9	1.0	0.01	#NUM!	2.0E-05
YNR062C	YNR062C	S288C_ctrl_hmg124h_12.JPG.dat	1230.8	24.8	1.0	0.03	#NUM!	4.9E-06
YNR063W	YNR063W	S288C_ctrl_hmg124h_12.JPG.dat	1299.3	16.0	1.0	0.02	#NUM!	8.8E-07
YNR064C	YNR064C	S288C_ctrl_hmg124h_12.JPG.dat	1266.0	66.3	1.0	0.01	#NUM!	5.5E-05
YNR065C	YNR065C	S288C_ctrl_hmg124h_12.JPG.dat	1287.0	21.6	1.0	0.01	#NUM!	1.8E-05
YNR066C	YNR066C	S288C_ctrl_hmg124h_12.JPG.dat	1237.3	34.2	1.0	0.03	#NUM!	5.1E-06
YNR067C	YNR067C	S288C_ctrl_hmg124h_12.JPG.dat	1170.0	28.0	1.0	0.01	#NUM!	2.1E-05
YNR069C	YNR069C	S288C_ctrl_hmg124h_12.JPG.dat	1075.8	28.6	1.0	0.02	#NUM!	2.6E-06
YNR071C	YNR071C	S288C_ctrl_hmg124h_12.JPG.dat	1108.8	44.8	1.0	0.04	#NUM!	1.4E-05
YNR072W	YNR072W	S288C_ctrl_hmg124h_12.JPG.dat	1089.5	16.7	1.0	0.01	#NUM!	9.3E-06
YNR073C	YNR073C	S288C_ctrl_hmg124h_12.JPG.dat	1107.3	3.0	1.0	0.01	#NUM!	5.2E-08
YNR074C	YNR074C	S288C_ctrl_hmg124h_12.JPG.dat	1122.8	18.7	1.0	0.01	#NUM!	1.8E-07
YNR075W	YNR075W	S288C_ctrl_hmg124h_12.JPG.dat	1212.0	28.5	1.0	0.03	#NUM!	8.6E-06
YOL001W	YOL001W	S288C_ctrl_hmg124h_12.JPG.dat	715.3	516.0	0.6	0.41	#NUM!	6.9E-02
YOL002C	YOL002C	S288C_ctrl_hmg124h_12.JPG.dat	1210.5	57.2	1.0	0.02	#NUM!	3.0E-06
YOL003C	YOL003C	S288C_ctrl_hmg124h_12.JPG.dat	1324.8	32.3	1.0	0.03	#NUM!	4.8E-06
YOL004W	YOL004W	S288C_ctrl_hmg124h_12.JPG.dat	13.0	26.0	0.0	0.00	#NUM!	

YOL006C	YOL006C	S288C_ctrl_hmg124h_12.JPG.dat	1133.3	38.1	0.9	0.03	#NUM!	6.2E-06
YOL007C	YOL007C	S288C_ctrl_hmg124h_12.JPG.dat	1152.8	14.5	1.0	0.02	#NUM!	1.4E-06
YOL008W	YOL008W	S288C_ctrl_hmg124h_12.JPG.dat	1050.0	19.1	0.9	0.01	#NUM!	9.3E-07
YOL009C	YOL009C	S288C_ctrl_hmg124h_12.JPG.dat	1110.5	15.9	1.0	0.02	#NUM!	1.8E-06
YOL011W	YOL011W	S288C_ctrl_hmg124h_12.JPG.dat	1160.5	18.7	1.0	0.00	#NUM!	1.9E-06
YOL012C	YOL012C	S288C_ctrl_hmg124h_12.JPG.dat	975.8	34.4	0.8	0.03	#NUM!	9.9E-06
YOL013C	YOL013C	S288C_ctrl_hmg124h_12.JPG.dat	1371.0	14.4	1.1	0.01	#NUM!	5.0E-08
YOL013W-	YOL013W-A	S288C_ctrl_hmg124h_12.JPG.dat	1131.8	198.9	0.9	0.16	#NUM!	1.5E-03
YOL014W	YOL014W	S288C_ctrl_hmg124h_12.JPG.dat	1172.5	89.6	1.0	0.05	#NUM!	3.9E-05
YOL015W	YOL015W	S288C_ctrl_hmg124h_12.JPG.dat	1319.0	62.1	1.0	0.04	#NUM!	2.2E-05
YOL016C	YOL016C	S288C_ctrl_hmg124h_12.JPG.dat	1314.8	47.2	1.0	0.04	#NUM!	1.1E-05
YOL017W	YOL017W	S288C_ctrl_hmg124h_12.JPG.dat	1229.0	16.5	1.0	0.02	#NUM!	1.8E-06
YOL018C	YOL018C	S288C_ctrl_hmg124h_12.JPG.dat	1190.0	52.7	1.0	0.03	#NUM!	7.0E-06
YOL019W	YOL019W	S288C_ctrl_hmg124h_12.JPG.dat	1192.8	22.4	1.0	0.01	#NUM!	5.7E-07
YOL020W	YOL020W	S288C_ctrl_hmg124h_12.JPG.dat	1155.8	37.4	1.0	0.02	#NUM!	2.1E-06
YOL024W	YOL024W	S288C_ctrl_hmg124h_12.JPG.dat	1220.0	29.7	1.0	0.01	#NUM!	1.5E-05
YOL025W	YOL025W	S288C_ctrl_hmg124h_12.JPG.dat	1274.3	26.1	1.0	0.01	#NUM!	4.5E-07
YOL027C	YOL027C	S288C_ctrl_hmg124h_12.JPG.dat	1272.5	32.0	1.0	0.02	#NUM!	1.9E-06
YOL028C	YOL028C	S288C_ctrl_hmg124h_12.JPG.dat	1350.5	43.9	1.1	0.03	#NUM!	5.2E-06
YOL029C	YOL029C	S288C_ctrl_hmg124h_12.JPG.dat	818.0	553.8	0.9	0.12	#NUM!	5.7E-03
YOL030W	YOL030W	S288C_ctrl_hmg124h_12.JPG.dat	1194.0	142.7	0.9	0.12	#NUM!	6.1E-04
YOL031C	YOL031C	S288C_ctrl_hmg124h_12.JPG.dat	1313.0	20.4	1.0	0.01	#NUM!	8.4E-07
YOL032W	YOL032W	S288C_ctrl_hmg124h_12.JPG.dat	1362.5	30.0	1.0	0.02	#NUM!	9.4E-07
YOL035C	YOL035C	S288C_ctrl_hmg124h_12.JPG.dat	1350.8	43.9	1.0	0.02	#NUM!	1.8E-06
YOL036W	YOL036W	S288C_ctrl_hmg124h_12.JPG.dat	1345.0	68.6	1.0	0.03	#NUM!	8.0E-06
YOL037C	YOL037C	S288C_ctrl_hmg124h_12.JPG.dat	1321.8	39.4	1.0	0.02	#NUM!	1.1E-06
YOL039W	YOL039W	S288C_ctrl_hmg124h_12.JPG.dat	1343.0	33.7	1.0	0.02	#NUM!	2.3E-06
YOL041C	YOL041C	S288C_ctrl_hmg124h_12.JPG.dat	1347.5	32.4	1.0	0.01	#NUM!	1.0E-05
YOL042W	YOL042W	S288C_ctrl_hmg124h_12.JPG.dat	1323.0	11.7	1.0	0.01	#NUM!	4.9E-07
YOL043C	YOL043C	S288C_ctrl_hmg124h_12.JPG.dat	1292.8	39.4	1.0	0.02	#NUM!	4.4E-06
YOL044W	YOL044W	S288C_ctrl_hmg124h_12.JPG.dat	1139.3	74.0	0.9	0.07	#NUM!	1.2E-04
YOL045W	YOL045W	S288C_ctrl_hmg124h_12.JPG.dat	1216.3	75.6	1.0	0.05	#NUM!	4.3E-05
YOL046C	YOL046C	S288C_ctrl_hmg124h_12.JPG.dat	1308.8	44.3	1.0	0.04	#NUM!	1.3E-05
YOL047C	YOL047C	S288C_ctrl_hmg124h_12.JPG.dat	1303.3	9.1	1.0	0.01	#NUM!	1.4E-07
YOL048C	YOL048C	S288C_ctrl_hmg124h_12.JPG.dat	1305.3	21.9	1.0	0.02	#NUM!	3.7E-06
YOL049W	YOL049W	S288C_ctrl_hmg124h_12.JPG.dat	915.0	48.3	0.8	0.04	#NUM!	3.8E-05
YOL050C	YOL050C	S288C_ctrl_hmg124h_12.JPG.dat	1177.8	35.6	1.0	0.03	#NUM!	5.6E-06
YOL052C	YOL052C	S288C_ctrl_hmg124h_12.JPG.dat	645.8	273.5	0.4	0.00	#NUM!	2.4E-05
YOL053C-A	YOL053C-A	S288C_ctrl_hmg124h_12.JPG.dat	1221.3	35.3	1.0	0.03	#NUM!	5.2E-06
YOL053W	YOL053W	S288C_ctrl_hmg124h_12.JPG.dat	1294.5	13.7	1.0	0.01	#NUM!	2.5E-07
YOL054W	YOL054W	S288C_ctrl_hmg124h_12.JPG.dat	1270.5	24.6	1.0	0.02	#NUM!	2.2E-06
YOL055C	YOL055C	S288C_ctrl_hmg124h_12.JPG.dat	1305.3	36.9	1.0	0.03	#NUM!	4.9E-06
YOL056W	YOL056W	S288C_ctrl_hmg124h_12.JPG.dat	1273.5	44.7	1.0	0.03	#NUM!	4.3E-06
YOL057W	YOL057W	S288C_ctrl_hmg124h_12.JPG.dat	1336.0	13.9	1.0	0.01	#NUM!	1.3E-07
YOL058W	YOL058W	S288C_ctrl_hmg124h_12.JPG.dat	0.0	0.0	0.0	0.00	#NUM!	
YOL059W	YOL059W	S288C_ctrl_hmg124h_12.JPG.dat	1212.3	39.6	1.0	0.03	#NUM!	7.1E-06
YOL060C	YOL060C	S288C_ctrl_hmg124h_12.JPG.dat	1126.0	25.6	1.0	0.02	#NUM!	2.6E-06
YOL061W	YOL061W	S288C_ctrl_hmg124h_12.JPG.dat	1121.5	43.4	1.0	0.04	#NUM!	1.6E-05
YOL062C	YOL062C	S288C_ctrl_hmg124h_12.JPG.dat	1125.5	14.7	1.0	0.01	#NUM!	7.7E-07
YOL063C	YOL063C	S288C_ctrl_hmg124h_12.JPG.dat	1101.3	78.7	1.0	0.07	#NUM!	1.1E-04
YOL064C	YOL064C	S288C_ctrl_hmg124h_12.JPG.dat	1075.8	8.3	0.9	0.01	#NUM!	1.2E-07
YOL065C	YOL065C	S288C_ctrl_hmg124h_12.JPG.dat	1284.5	13.1	1.0	0.01	#NUM!	6.6E-07
YOL067C	YOL067C	S288C_ctrl_hmg124h_12.JPG.dat	1283.3	41.3	1.0	0.04	#NUM!	1.2E-05
YOL068C	YOL068C	S288C_ctrl_hmg124h_12.JPG.dat	1268.0	84.2	1.0	0.06	#NUM!	5.6E-05
YOL070C	YOL070C	S288C_ctrl_hmg124h_12.JPG.dat	1368.5	14.9	1.1	0.01	#NUM!	5.7E-07
YOL071W	YOL071W	S288C_ctrl_hmg124h_12.JPG.dat	1201.8	16.3	1.0	0.00	#NUM!	1.8E-06
YOL075C	YOL075C	S288C_ctrl_hmg124h_12.JPG.dat	1190.5	23.2	1.0	0.01	#NUM!	2.9E-07
YOL079W	YOL079W	S288C_ctrl_hmg124h_12.JPG.dat	1138.8	9.7	1.0	0.01	#NUM!	7.9E-07
YOL080C	YOL080C	S288C_ctrl_hmg124h_12.JPG.dat	1093.5	26.1	1.0	0.03	#NUM!	6.7E-06
YOL081W	YOL081W	S288C_ctrl_hmg124h_12.JPG.dat	1055.8	17.9	1.0	0.02	#NUM!	1.1E-06
YOL082W	YOL082W	S288C_ctrl_hmg124h_12.JPG.dat	1117.3	24.6	1.0	0.02	#NUM!	1.5E-06
YOL083W	YOL083W	S288C_ctrl_hmg124h_12.JPG.dat	1239.3	37.1	1.0	0.02	#NUM!	3.7E-06
YOL084W	YOL084W	S288C_ctrl_hmg124h_12.JPG.dat	1252.5	30.2	1.0	0.02	#NUM!	1.7E-06
YOL085C	YOL085C	S288C_ctrl_hmg124h_12.JPG.dat	1295.8	23.0	1.0	0.02	#NUM!	2.3E-06
YOL087C	YOL087C	S288C_ctrl_hmg124h_12.JPG.dat	1239.8	65.0	1.0	0.05	#NUM!	4.5E-05
YOL088C	YOL088C	S288C_ctrl_hmg124h_12.JPG.dat	1261.8	27.4	1.0	0.01	#NUM!	1.3E-05
YOL089C	YOL089C	S288C_ctrl_hmg124h_12.JPG.dat	1203.5	26.0	1.0	0.01	#NUM!	2.9E-05
YOL090W	YOL090W	S288C_ctrl_hmg124h_12.JPG.dat	1041.8	14.4	0.9	0.02	#NUM!	2.8E-06
YOL091W	YOL091W	S288C_ctrl_hmg124h_12.JPG.dat	1074.5	28.8	1.0	0.03	#NUM!	4.8E-06
YOL092W	YOL092W	S288C_ctrl_hmg124h_12.JPG.dat	1145.3	20.9	1.1	0.02	#NUM!	8.3E-07

YOL093W	YOL093W	S288C_ctrl_hmg124h_12.JPG.dat	1064.8	31.7	1.0	0.03	#NUM!	6.8E-06
YOL095C	YOL095C	S288C_ctrl_hmg124h_12.JPG.dat	1090.3	34.1	1.0	0.03	#NUM!	8.6E-06
YOL098C	YOL098C	S288C_ctrl_hmg124h_12.JPG.dat	1192.0	8.8	1.0	0.01	#NUM!	3.6E-07
YOL099C	YOL099C	S288C_ctrl_hmg124h_12.JPG.dat	1262.8	33.7	1.0	0.03	#NUM!	5.8E-06
YOL101C	YOL101C	S288C_ctrl_hmg124h_12.JPG.dat	1314.0	43.3	1.1	0.03	#NUM!	6.6E-06
YOL103W	YOL103W	S288C_ctrl_hmg124h_12.JPG.dat	1286.3	65.5	1.0	0.01	#NUM!	4.1E-05
YOL104C	YOL104C	S288C_ctrl_hmg124h_12.JPG.dat	1329.0	22.9	1.0	0.02	#NUM!	2.7E-06
YOL105C	YOL105C	S288C_ctrl_hmg124h_12.JPG.dat	1248.5	25.4	1.0	0.02	#NUM!	1.8E-06
YOL106W	YOL106W	S288C_ctrl_hmg124h_12.JPG.dat	1134.8	29.2	1.0	0.03	#NUM!	8.1E-06
YOL107W	YOL107W	S288C_ctrl_hmg124h_12.JPG.dat	850.3	567.8	1.0	0.03	#NUM!	3.1E-04
YOL108C	YOL108C	S288C_ctrl_hmg124h_12.JPG.dat	1083.0	21.3	1.0	0.01	#NUM!	6.9E-07
YOL109W	YOL109W	S288C_ctrl_hmg124h_12.JPG.dat	1087.5	21.9	1.0	0.02	#NUM!	4.0E-06
YOL110W	YOL110W	S288C_ctrl_hmg124h_12.JPG.dat	1171.0	20.9	1.0	0.01	#NUM!	5.5E-07
YOL111C	YOL111C	S288C_ctrl_hmg124h_12.JPG.dat	1260.3	25.0	1.0	0.03	#NUM!	5.4E-06
YOL112W	YOL112W	S288C_ctrl_hmg124h_12.JPG.dat	1146.5	308.4	1.0	0.02	#NUM!	1.2E-04
YOL113W	YOL113W	S288C_ctrl_hmg124h_12.JPG.dat	1250.8	48.9	1.0	0.04	#NUM!	1.6E-05
YOL114C	YOL114C	S288C_ctrl_hmg124h_12.JPG.dat	1309.3	72.0	1.0	0.07	#NUM!	7.4E-05
YOL115W	YOL115W	S288C_ctrl_hmg124h_12.JPG.dat	0.0	0.0	0.0	0.00	#NUM!	
YOL116W	YOL116W	S288C_ctrl_hmg124h_12.JPG.dat	1306.5	12.8	1.0	0.01	#NUM!	4.1E-07
YOL117W	YOL117W	S288C_ctrl_hmg124h_12.JPG.dat	1226.8	19.1	1.0	0.01	#NUM!	4.3E-07
YOL118C	YOL118C	S288C_ctrl_hmg124h_12.JPG.dat	1180.3	21.7	1.0	0.01	#NUM!	7.6E-07
YOL119C	YOL119C	S288C_ctrl_hmg124h_12.JPG.dat	1168.3	29.1	1.0	0.01	#NUM!	5.4E-07
YOL121C	YOL121C	S288C_ctrl_hmg124h_12.JPG.dat	1164.3	17.2	1.0	0.02	#NUM!	1.3E-06
YOL122C	YOL122C	S288C_ctrl_hmg124h_12.JPG.dat	1223.8	10.6	1.0	0.01	#NUM!	9.0E-07
YOL124C	YOL124C	S288C_ctrl_hmg124h_12.JPG.dat	1263.5	13.1	1.0	0.02	#NUM!	1.1E-06
YOL126C	YOL126C	S288C_ctrl_hmg124h_12.JPG.dat	1326.8	16.3	1.0	0.00	#NUM!	4.4E-06
YOL128C	YOL128C	S288C_ctrl_hmg124h_12.JPG.dat	1155.0	366.2	1.1	0.03	#NUM!	2.7E-04
YOL129W	YOL129W	S288C_ctrl_hmg124h_12.JPG.dat	1236.8	47.2	0.9	0.04	#NUM!	1.7E-05
YOL131W	YOL131W	S288C_ctrl_hmg124h_12.JPG.dat	1298.5	22.5	1.0	0.03	#NUM!	7.9E-06
YOL132W	YOL132W	S288C_ctrl_hmg124h_12.JPG.dat	1329.0	17.0	1.0	0.01	#NUM!	1.2E-07
YOL136C	YOL136C	S288C_ctrl_hmg124h_12.JPG.dat	1308.8	51.2	1.0	0.01	#NUM!	4.4E-05
YOL137W	YOL137W	S288C_ctrl_hmg124h_12.JPG.dat	1316.5	40.1	1.0	0.02	#NUM!	3.1E-06
YOL138C	YOL138C	S288C_ctrl_hmg124h_12.JPG.dat	1345.3	31.5	1.0	0.01	#NUM!	8.0E-08
YOL141W	YOL141W	S288C_ctrl_hmg124h_12.JPG.dat	1335.3	53.8	1.0	0.02	#NUM!	3.6E-06
YOL147C	YOL147C	S288C_ctrl_hmg124h_12.JPG.dat	1349.3	40.5	1.0	0.02	#NUM!	2.6E-06
YOL150C	YOL150C	S288C_ctrl_hmg124h_12.JPG.dat	1311.0	42.2	1.0	0.04	#NUM!	1.8E-05
YOL151W	YOL151W	S288C_ctrl_hmg124h_12.JPG.dat	1293.5	32.6	1.0	0.02	#NUM!	4.2E-06
YOL152W	YOL152W	S288C_ctrl_hmg124h_12.JPG.dat	1140.0	163.6	0.9	0.12	#NUM!	7.9E-04
YOL155C	YOL155C	S288C_ctrl_hmg124h_12.JPG.dat	1310.8	103.6	1.0	0.09	#NUM!	1.9E-04
YOL158C	YOL158C	S288C_ctrl_hmg124h_12.JPG.dat	1317.5	34.4	1.0	0.05	#NUM!	2.9E-05
YOL159C	YOL159C	S288C_ctrl_hmg124h_12.JPG.dat	1348.3	42.5	1.0	0.01	#NUM!	1.1E-07
YOL160W	YOL160W	S288C_ctrl_hmg124h_12.JPG.dat	1266.3	111.7	1.0	0.03	#NUM!	3.4E-04
YOL162W	YOL162W	S288C_ctrl_hmg124h_12.JPG.dat	1293.3	39.0	1.0	0.00	#NUM!	3.1E-06
YOL163W	YOL163W	S288C_ctrl_hmg124h_12.JPG.dat	1309.0	39.1	1.0	0.02	#NUM!	3.4E-06
YOR001W	YOR001W	S288C_ctrl_hmg124h_12.JPG.dat	1159.0	46.8	0.9	0.00	#NUM!	4.8E-06
YOR002W	YOR002W	S288C_ctrl_hmg124h_12.JPG.dat	1368.0	68.2	1.0	0.01	#NUM!	5.1E-05
YOR003W	YOR003W	S288C_ctrl_hmg124h_12.JPG.dat	1351.8	40.5	1.0	0.01	#NUM!	7.9E-06
YOR005C	YOR005C	S288C_ctrl_hmg124h_12.JPG.dat	1338.3	68.4	1.0	0.03	#NUM!	8.2E-06
YOR006C	YOR006C	S288C_ctrl_hmg124h_12.JPG.dat	814.8	324.3	0.5	0.05	#NUM!	3.3E-03
YOR007C	YOR007C	S288C_ctrl_hmg124h_12.JPG.dat	1321.5	18.0	1.0	0.00	#NUM!	5.8E-07
YOR008C	YOR008C	S288C_ctrl_hmg124h_12.JPG.dat	1234.0	49.6	1.0	0.04	#NUM!	2.1E-05
YOR008C-/	YOR008C-A	S288C_ctrl_hmg124h_12.JPG.dat	1321.5	35.0	1.0	0.02	#NUM!	2.6E-06
YOR009W	YOR009W	S288C_ctrl_hmg124h_12.JPG.dat	1275.0	13.7	1.1	0.00	#NUM!	1.7E-08
YOR010C	YOR010C	S288C_ctrl_hmg124h_12.JPG.dat	1097.8	85.1	1.0	0.02	#NUM!	1.8E-04
YOR011W	YOR011W	S288C_ctrl_hmg124h_12.JPG.dat	1186.0	25.7	1.0	0.02	#NUM!	2.0E-06
YOR012W	YOR012W	S288C_ctrl_hmg124h_12.JPG.dat	1191.0	13.0	1.0	0.01	#NUM!	5.5E-08
YOR013W	YOR013W	S288C_ctrl_hmg124h_12.JPG.dat	1178.8	14.1	1.0	0.02	#NUM!	1.2E-06
YOR014W	YOR014W	S288C_ctrl_hmg124h_12.JPG.dat	1097.8	91.8	0.9	0.01	#NUM!	5.6E-05
YOR015W	YOR015W	S288C_ctrl_hmg124h_12.JPG.dat	1137.3	241.4	1.0	0.04	#NUM!	4.4E-04
YOR016C	YOR016C	S288C_ctrl_hmg124h_12.JPG.dat	1320.3	36.2	1.1	0.02	#NUM!	3.0E-06
YOR017W	YOR017W	S288C_ctrl_hmg124h_12.JPG.dat	1276.5	18.4	1.0	0.01	#NUM!	1.4E-05
YOR018W	YOR018W	S288C_ctrl_hmg124h_12.JPG.dat	1375.3	56.6	1.1	0.05	#NUM!	2.1E-05
YOR019W	YOR019W	S288C_ctrl_hmg124h_12.JPG.dat	1231.8	40.0	1.0	0.03	#NUM!	4.4E-06
YOR021C	YOR021C	S288C_ctrl_hmg124h_12.JPG.dat	1054.3	64.5	0.9	0.05	#NUM!	6.0E-05
YOR022C	YOR022C	S288C_ctrl_hmg124h_12.JPG.dat	1130.5	26.2	1.0	0.01	#NUM!	1.6E-05
YOR023C	YOR023C	S288C_ctrl_hmg124h_12.JPG.dat	1144.8	11.5	1.0	0.00	#NUM!	5.7E-07
YOR024W	YOR024W	S288C_ctrl_hmg124h_12.JPG.dat	1001.8	60.4	0.9	0.05	#NUM!	5.1E-05
YOR025W	YOR025W	S288C_ctrl_hmg124h_12.JPG.dat	1058.0	145.4	0.9	0.11	#NUM!	5.5E-04
YOR026W	YOR026W	S288C_ctrl_hmg124h_12.JPG.dat	959.0	134.8	0.7	0.04	#NUM!	8.8E-04
YOR027W	YOR027W	S288C_ctrl_hmg124h_12.JPG.dat	1254.5	42.0	1.0	0.01	#NUM!	3.5E-05

YOR028C	YOR028C	S288C_ctrl_hmg124h_12.JPG.dat	1265.0	9.8	1.0	0.03	#NUM!	4.8E-06
YOR029W	YOR029W	S288C_ctrl_hmg124h_12.JPG.dat	1307.0	38.7	1.0	0.03	#NUM!	6.6E-06
YOR030W	YOR030W	S288C_ctrl_hmg124h_12.JPG.dat	1167.3	20.8	1.0	0.02	#NUM!	1.3E-06
YOR031W	YOR031W	S288C_ctrl_hmg124h_12.JPG.dat	1204.3	30.2	1.0	0.03	#NUM!	4.2E-06
YOR032C	YOR032C	S288C_ctrl_hmg124h_12.JPG.dat	1048.0	48.7	1.0	0.00	#NUM!	8.4E-06
YOR033C	YOR033C	S288C_ctrl_hmg124h_12.JPG.dat	296.0	592.0	0.0	0.00	#NUM!	
YOR034C	YOR034C	S288C_ctrl_hmg124h_12.JPG.dat	1084.8	38.6	1.0	0.03	#NUM!	1.1E-05
YOR035C	YOR035C	S288C_ctrl_hmg124h_12.JPG.dat	850.8	40.9	0.8	0.04	#NUM!	2.9E-05
YOR037W	YOR037W	S288C_ctrl_hmg124h_12.JPG.dat	1052.5	26.3	0.9	0.02	#NUM!	2.5E-06
YOR038C	YOR038C	S288C_ctrl_hmg124h_12.JPG.dat	794.0	227.7	0.7	0.19	#NUM!	6.1E-03
YOR039W	YOR039W	S288C_ctrl_hmg124h_12.JPG.dat	1082.0	34.8	0.9	0.00	#NUM!	6.3E-07
YOR040W	YOR040W	S288C_ctrl_hmg124h_12.JPG.dat	1297.5	57.1	1.1	0.04	#NUM!	1.8E-05
YOR041C	YOR041C	S288C_ctrl_hmg124h_12.JPG.dat	1277.5	32.2	1.0	0.02	#NUM!	3.0E-06
YOR042W	YOR042W	S288C_ctrl_hmg124h_12.JPG.dat	1323.3	48.0	1.1	0.03	#NUM!	8.8E-06
YOR043W	YOR043W	S288C_ctrl_hmg124h_12.JPG.dat	1053.3	95.9	0.9	0.08	#NUM!	1.8E-04
YOR044W	YOR044W	S288C_ctrl_hmg124h_12.JPG.dat	1141.3	9.1	1.0	0.02	#NUM!	1.0E-06
YOR045W	YOR045W	S288C_ctrl_hmg124h_12.JPG.dat	1067.5	14.3	1.0	0.01	#NUM!	4.1E-07
YOR047C	YOR047C	S288C_ctrl_hmg124h_12.JPG.dat	1086.8	13.0	1.0	0.01	#NUM!	5.0E-07
YOR049C	YOR049C	S288C_ctrl_hmg124h_12.JPG.dat	1123.8	34.8	1.0	0.02	#NUM!	3.4E-06
YOR050C	YOR050C	S288C_ctrl_hmg124h_12.JPG.dat	1200.0	36.1	1.0	0.00	#NUM!	4.6E-06
YOR051C	YOR051C	S288C_ctrl_hmg124h_12.JPG.dat	1263.8	31.4	1.0	0.01	#NUM!	1.9E-05
YOR052C	YOR052C	S288C_ctrl_hmg124h_12.JPG.dat	1323.5	20.6	1.0	0.01	#NUM!	4.7E-07
YOR053W	YOR053W	S288C_ctrl_hmg124h_12.JPG.dat	1214.5	84.3	1.0	0.04	#NUM!	2.2E-05
YOR055W	YOR055W	S288C_ctrl_hmg124h_12.JPG.dat	1272.8	4.9	1.0	0.00	#NUM!	1.3E-06
YOR058C	YOR058C	S288C_ctrl_hmg124h_12.JPG.dat	1180.5	192.1	0.9	0.16	#NUM!	1.2E-03
YOR059C	YOR059C	S288C_ctrl_hmg124h_12.JPG.dat	1207.8	13.2	1.0	0.01	#NUM!	4.9E-07
YOR061W	YOR061W	S288C_ctrl_hmg124h_12.JPG.dat	1025.8	19.2	0.9	0.01	#NUM!	1.7E-05
YOR062C	YOR062C	S288C_ctrl_hmg124h_12.JPG.dat	1058.5	92.5	1.0	0.03	#NUM!	2.5E-04
YOR064C	YOR064C	S288C_ctrl_hmg124h_12.JPG.dat	1105.8	18.0	1.0	0.01	#NUM!	8.1E-07
YOR065W	YOR065W	S288C_ctrl_hmg124h_12.JPG.dat	919.3	613.1	1.0	0.02	#NUM!	1.1E-04
YOR066W	YOR066W	S288C_ctrl_hmg124h_12.JPG.dat	1257.5	22.3	1.0	0.01	#NUM!	6.1E-07
YOR067C	YOR067C	S288C_ctrl_hmg124h_12.JPG.dat	1300.0	6.4	1.0	0.00	#NUM!	3.2E-06
YOR068C	YOR068C	S288C_ctrl_hmg124h_12.JPG.dat	914.3	609.7	1.0	0.01	#NUM!	3.5E-05
YOR069W	YOR069W	S288C_ctrl_hmg124h_12.JPG.dat	1114.3	175.0	0.9	0.12	#NUM!	6.5E-04
YOR070C	YOR070C	S288C_ctrl_hmg124h_12.JPG.dat	1251.5	22.9	1.0	0.02	#NUM!	3.1E-06
YOR071C	YOR071C	S288C_ctrl_hmg124h_12.JPG.dat	1294.0	13.7	1.0	0.01	#NUM!	3.6E-07
YOR072W	YOR072W	S288C_ctrl_hmg124h_12.JPG.dat	1328.8	28.3	1.0	0.02	#NUM!	2.2E-06
YOR076C	YOR076C	S288C_ctrl_hmg124h_12.JPG.dat	1212.0	21.3	1.0	0.01	#NUM!	1.1E-07
YOR078W	YOR078W	S288C_ctrl_hmg124h_12.JPG.dat	844.5	489.6	0.7	0.40	#NUM!	4.1E-02
YOR079C	YOR079C	S288C_ctrl_hmg124h_12.JPG.dat	1232.3	30.4	1.0	0.01	#NUM!	6.0E-07
YOR080W	YOR080W	S288C_ctrl_hmg124h_12.JPG.dat	1265.3	21.0	1.0	0.01	#NUM!	7.7E-07
YOR081C	YOR081C	S288C_ctrl_hmg124h_12.JPG.dat	1292.8	8.6	1.0	0.01	#NUM!	3.7E-08
YOR082C	YOR082C	S288C_ctrl_hmg124h_12.JPG.dat	1296.8	30.0	1.0	0.02	#NUM!	3.1E-06
YOR083W	YOR083W	S288C_ctrl_hmg124h_12.JPG.dat	661.0	763.6	0.5	0.60	#NUM!	1.8E-01
YOR084W	YOR084W	S288C_ctrl_hmg124h_12.JPG.dat	1119.0	79.9	0.9	0.05	#NUM!	3.7E-05
YOR085W	YOR085W	S288C_ctrl_hmg124h_12.JPG.dat	1162.0	78.6	0.8	0.00	#NUM!	2.9E-06
YOR086C	YOR086C	S288C_ctrl_hmg124h_12.JPG.dat	1338.5	29.4	1.0	0.03	#NUM!	8.5E-06
YOR087W	YOR087W	S288C_ctrl_hmg124h_12.JPG.dat	1330.3	24.7	1.0	0.01	#NUM!	8.3E-08
YOR088W	YOR088W	S288C_ctrl_hmg124h_12.JPG.dat	1362.5	73.3	1.0	0.04	#NUM!	1.7E-05
YOR089C	YOR089C	S288C_ctrl_hmg124h_12.JPG.dat	1260.5	61.3	1.0	0.02	#NUM!	2.5E-06
YOR090C	YOR090C	S288C_ctrl_hmg124h_12.JPG.dat	1340.8	52.0	1.0	0.02	#NUM!	1.0E-06
YOR091W	YOR091W	S288C_ctrl_hmg124h_12.JPG.dat	1333.8	52.6	1.0	0.02	#NUM!	2.6E-06
YOR092W	YOR092W	S288C_ctrl_hmg124h_12.JPG.dat	1344.5	73.1	1.0	0.03	#NUM!	7.6E-06
YOR093C	YOR093C	S288C_ctrl_hmg124h_12.JPG.dat	1283.0	49.5	1.0	0.03	#NUM!	4.5E-06
YOR094W	YOR094W	S288C_ctrl_hmg124h_12.JPG.dat	1333.5	59.6	1.0	0.03	#NUM!	9.5E-06
YOR097C	YOR097C	S288C_ctrl_hmg124h_12.JPG.dat	1289.5	68.9	1.0	0.03	#NUM!	8.6E-06
YOR099W	YOR099W	S288C_ctrl_hmg124h_12.JPG.dat	1213.3	61.9	1.0	0.05	#NUM!	2.9E-05
YOR100C	YOR100C	S288C_ctrl_hmg124h_12.JPG.dat	1304.3	13.5	1.0	0.00	#NUM!	4.4E-07
YOR101W	YOR101W	S288C_ctrl_hmg124h_12.JPG.dat	1320.5	34.2	1.0	0.03	#NUM!	6.6E-06
YOR104W	YOR104W	S288C_ctrl_hmg124h_12.JPG.dat	1232.0	8.0	1.0	0.01	#NUM!	2.5E-07
YOR105W	YOR105W	S288C_ctrl_hmg124h_12.JPG.dat	1189.0	25.7	1.0	0.01	#NUM!	2.0E-05
YOR106W	YOR106W	S288C_ctrl_hmg124h_12.JPG.dat	920.5	37.5	0.8	0.00	#NUM!	7.8E-06
YOR107W	YOR107W	S288C_ctrl_hmg124h_12.JPG.dat	1196.0	13.9	1.0	0.01	#NUM!	9.3E-08
YOR108W	YOR108W	S288C_ctrl_hmg124h_12.JPG.dat	1201.0	5.7	1.0	0.01	#NUM!	3.9E-07
YOR109W	YOR109W	S288C_ctrl_hmg124h_12.JPG.dat	1272.8	12.1	1.0	0.01	#NUM!	3.5E-07
YOR111W	YOR111W	S288C_ctrl_hmg124h_12.JPG.dat	1179.3	66.3	0.9	0.01	#NUM!	2.3E-05
YOR112W	YOR112W	S288C_ctrl_hmg124h_12.JPG.dat	1321.8	10.9	1.0	0.01	#NUM!	1.2E-07
YOR113W	YOR113W	S288C_ctrl_hmg124h_12.JPG.dat	1279.8	44.1	1.0	0.03	#NUM!	8.1E-06
YOR114W	YOR114W	S288C_ctrl_hmg124h_12.JPG.dat	1308.5	39.7	1.0	0.03	#NUM!	7.4E-06
YOR115C	YOR115C	S288C_ctrl_hmg124h_12.JPG.dat	1255.8	15.9	1.0	0.01	#NUM!	4.3E-07

YOR118W	YOR118W	S288C_ctrl_hmg124h_12.JPG.dat	1168.8	16.7	1.0	0.01	#NUM!	1.6E-07
YOR120W	YOR120W	S288C_ctrl_hmg124h_12.JPG.dat	1185.5	20.9	1.0	0.02	#NUM!	1.4E-06
YOR121C	YOR121C	S288C_ctrl_hmg124h_12.JPG.dat	1100.0	45.3	1.0	0.01	#NUM!	5.3E-05
YOR123C	YOR123C	S288C_ctrl_hmg124h_12.JPG.dat	976.8	28.5	0.9	0.02	#NUM!	5.5E-06
YOR124C	YOR124C	S288C_ctrl_hmg124h_12.JPG.dat	1109.0	36.5	1.0	0.03	#NUM!	6.2E-06
YOR126C	YOR126C	S288C_ctrl_hmg124h_12.JPG.dat	1252.5	9.3	1.0	0.00	#NUM!	1.4E-07
YOR127W	YOR127W	S288C_ctrl_hmg124h_12.JPG.dat	1339.3	30.1	1.1	0.01	#NUM!	1.1E-05
YOR129C	YOR129C	S288C_ctrl_hmg124h_12.JPG.dat	1248.5	40.0	1.0	0.03	#NUM!	8.5E-06
YOR131C	YOR131C	S288C_ctrl_hmg124h_12.JPG.dat	1281.5	74.6	1.0	0.05	#NUM!	4.0E-05
YOR132W	YOR132W	S288C_ctrl_hmg124h_12.JPG.dat	1103.0	233.5	1.0	0.01	#NUM!	6.8E-05
YOR133W	YOR133W	S288C_ctrl_hmg124h_12.JPG.dat	1183.5	17.8	1.0	0.02	#NUM!	1.7E-06
YOR134W	YOR134W	S288C_ctrl_hmg124h_12.JPG.dat	1164.8	44.5	1.0	0.03	#NUM!	8.6E-06
YOR135C	YOR135C	S288C_ctrl_hmg124h_12.JPG.dat	1099.8	21.9	1.0	0.01	#NUM!	1.3E-05
YOR136W	YOR136W	S288C_ctrl_hmg124h_12.JPG.dat	1128.8	41.5	1.1	0.01	#NUM!	5.2E-05
YOR137C	YOR137C	S288C_ctrl_hmg124h_12.JPG.dat	1090.5	19.3	1.0	0.02	#NUM!	1.8E-06
YOR138C	YOR138C	S288C_ctrl_hmg124h_12.JPG.dat	1130.5	2.6	1.0	0.00	#NUM!	2.1E-08
YOR139C	YOR139C	S288C_ctrl_hmg124h_12.JPG.dat	1213.0	15.4	1.0	0.01	#NUM!	6.3E-07
YOR140W	YOR140W	S288C_ctrl_hmg124h_12.JPG.dat	1190.3	63.8	1.0	0.05	#NUM!	4.3E-05
YOR141C	YOR141C	S288C_ctrl_hmg124h_12.JPG.dat	540.0	650.3	0.4	0.52	#NUM!	2.0E-01
YOR142W	YOR142W	S288C_ctrl_hmg124h_12.JPG.dat	1268.0	19.1	1.0	0.02	#NUM!	3.8E-06
YOR144C	YOR144C	S288C_ctrl_hmg124h_12.JPG.dat	1278.5	36.7	1.0	0.02	#NUM!	3.9E-06
YOR152C	YOR152C	S288C_ctrl_hmg124h_12.JPG.dat	1246.8	40.0	1.0	0.02	#NUM!	3.8E-06
YOR153W	YOR153W	S288C_ctrl_hmg124h_12.JPG.dat	1146.5	18.0	1.0	0.00	#NUM!	6.1E-06
YOR154W	YOR154W	S288C_ctrl_hmg124h_12.JPG.dat	1075.8	12.1	1.0	0.00	#NUM!	2.2E-06
YOR156C	YOR156C	S288C_ctrl_hmg124h_12.JPG.dat	1094.3	18.2	1.0	0.02	#NUM!	2.9E-06
YOR161C	YOR161C	S288C_ctrl_hmg124h_12.JPG.dat	1112.5	11.3	1.0	0.01	#NUM!	4.2E-07
YOR162C	YOR162C	S288C_ctrl_hmg124h_12.JPG.dat	1157.8	18.8	1.0	0.01	#NUM!	3.7E-08
YOR163W	YOR163W	S288C_ctrl_hmg124h_12.JPG.dat	1232.0	23.4	1.0	0.01	#NUM!	2.6E-07
YOR164C	YOR164C	S288C_ctrl_hmg124h_12.JPG.dat	1291.0	44.5	1.0	0.01	#NUM!	2.9E-05
YOR165W	YOR165W	S288C_ctrl_hmg124h_12.JPG.dat	1286.0	32.7	1.0	0.03	#NUM!	5.3E-06
YOR166C	YOR166C	S288C_ctrl_hmg124h_12.JPG.dat	1353.0	28.0	1.1	0.01	#NUM!	2.3E-05
YOR167C	YOR167C	S288C_ctrl_hmg124h_12.JPG.dat	1241.8	35.7	1.0	0.03	#NUM!	5.4E-06
YOR170W	YOR170W	S288C_ctrl_hmg124h_12.JPG.dat	1242.3	37.2	1.0	0.03	#NUM!	7.0E-06
YOR171C	YOR171C	S288C_ctrl_hmg124h_12.JPG.dat	1222.3	34.2	1.0	0.03	#NUM!	6.4E-06
YOR172W	YOR172W	S288C_ctrl_hmg124h_12.JPG.dat	1137.5	61.8	1.0	0.06	#NUM!	6.2E-05
YOR173W	YOR173W	S288C_ctrl_hmg124h_12.JPG.dat	1117.0	18.1	1.0	0.02	#NUM!	1.2E-06
YOR175C	YOR175C	S288C_ctrl_hmg124h_12.JPG.dat	1186.3	38.7	1.0	0.03	#NUM!	8.5E-06
YOR177C	YOR177C	S288C_ctrl_hmg124h_12.JPG.dat	1200.0	25.9	1.0	0.01	#NUM!	7.8E-07
YOR178C	YOR178C	S288C_ctrl_hmg124h_12.JPG.dat	1217.3	22.7	1.0	0.02	#NUM!	1.6E-06
YOR182C	YOR182C	S288C_ctrl_hmg124h_12.JPG.dat	1148.5	121.3	1.0	0.03	#NUM!	3.0E-04
YOR183W	YOR183W	S288C_ctrl_hmg124h_12.JPG.dat	1181.8	93.4	0.9	0.08	#NUM!	1.4E-04
YOR184W	YOR184W	S288C_ctrl_hmg124h_12.JPG.dat	1312.8	23.4	1.0	0.03	#NUM!	4.7E-06
YOR185C	YOR185C	S288C_ctrl_hmg124h_12.JPG.dat	1324.0	37.6	1.0	0.03	#NUM!	7.9E-06
YOR186W	YOR186W	S288C_ctrl_hmg124h_12.JPG.dat	1271.5	20.0	1.0	0.01	#NUM!	4.8E-07
YOR188W	YOR188W	S288C_ctrl_hmg124h_12.JPG.dat	1285.3	34.5	1.0	0.02	#NUM!	1.5E-06
YOR189W	YOR189W	S288C_ctrl_hmg124h_12.JPG.dat	1248.8	21.0	1.0	0.01	#NUM!	2.5E-07
YOR190W	YOR190W	S288C_ctrl_hmg124h_12.JPG.dat	1229.8	44.5	1.0	0.01	#NUM!	2.3E-05
YOR191W	YOR191W	S288C_ctrl_hmg124h_12.JPG.dat	1179.3	69.6	1.0	0.01	#NUM!	7.2E-05
YOR192C	YOR192C	S288C_ctrl_hmg124h_12.JPG.dat	1231.8	18.6	1.0	0.02	#NUM!	1.5E-06
YOR193W	YOR193W	S288C_ctrl_hmg124h_12.JPG.dat	1332.5	20.2	1.0	0.02	#NUM!	1.1E-06
YOR195W	YOR195W	S288C_ctrl_hmg124h_12.JPG.dat	1343.3	26.1	1.0	0.02	#NUM!	3.1E-06
YOR196C	YOR196C	S288C_ctrl_hmg124h_12.JPG.dat	1098.3	116.4	0.9	0.10	#NUM!	3.7E-04
YOR202W	YOR202W	S288C_ctrl_hmg124h_12.JPG.dat	1304.5	147.4	1.0	0.08	#NUM!	3.3123882[illegible]
1	1	S288C_ctrl_hmg124h_13.JPG.dat	1395.0	167.7	1.0	0.14	#NUM!	6.2E-04
2	2	S288C_ctrl_hmg124h_13.JPG.dat	1204.8	118.6	0.9	0.09	#NUM!	2.2E-04
3	3	S288C_ctrl_hmg124h_13.JPG.dat	1183.5	86.9	1.0	0.07	#NUM!	1.0E-04
4	4	S288C_ctrl_hmg124h_13.JPG.dat	1184.0	65.5	1.0	0.06	#NUM!	5.0E-05
YOR197W	YOR197W	S288C_ctrl_hmg124h_13.JPG.dat	1303.8	20.0	1.0	0.00	#NUM!	3.7E-06
YOR202W	YOR202W	S288C_ctrl_hmg124h_13.JPG.dat	1363.8	122.7	1.0	0.08	#NUM!	2.1672444[illegible]
YOR208W	YOR208W	S288C_ctrl_hmg124h_13.JPG.dat	1354.0	23.0	1.0	0.01	#NUM!	8.3E-07
YOR209C	YOR209C	S288C_ctrl_hmg124h_13.JPG.dat	1232.5	18.7	0.9	0.01	#NUM!	2.8E-07
YOR212W	YOR212W	S288C_ctrl_hmg124h_13.JPG.dat	334.5	669.0	0.0	0.00	#NUM!	
YOR213C	YOR213C	S288C_ctrl_hmg124h_13.JPG.dat	1292.5	10.7	1.0	0.01	#NUM!	6.4E-07
YOR214C	YOR214C	S288C_ctrl_hmg124h_13.JPG.dat	1280.8	23.9	1.0	0.01	#NUM!	1.6E-05
YOR215C	YOR215C	S288C_ctrl_hmg124h_13.JPG.dat	1311.0	24.3	1.0	0.02	#NUM!	1.6E-06
YOR216C	YOR216C	S288C_ctrl_hmg124h_13.JPG.dat	1346.3	19.0	1.0	0.02	#NUM!	1.4E-06
YOR219C	YOR219C	S288C_ctrl_hmg124h_13.JPG.dat	1292.0	6.5	1.0	0.01	#NUM!	1.1E-07
YOR221C	YOR221C	S288C_ctrl_hmg124h_13.JPG.dat	1291.5	46.5	1.0	0.03	#NUM!	8.4E-06
YOR222W	YOR222W	S288C_ctrl_hmg124h_13.JPG.dat	1368.3	22.7	1.0	0.02	#NUM!	1.1E-06
YOR223W	YOR223W	S288C_ctrl_hmg124h_13.JPG.dat	1401.0	42.0	1.0	0.03	#NUM!	7.4E-06

YOR225W	YOR225W	S288C_ctrl_hmg124h_13.JPG.dat	1284.3	29.9	1.0	0.02	#NUM!	2.9E-06
YOR226C	YOR226C	S288C_ctrl_hmg124h_13.JPG.dat	1268.5	32.5	1.0	0.02	#NUM!	1.7E-06
YOR227W	YOR227W	S288C_ctrl_hmg124h_13.JPG.dat	1243.0	33.6	1.0	0.03	#NUM!	6.8E-06
YOR228C	YOR228C	S288C_ctrl_hmg124h_13.JPG.dat	1254.0	25.1	1.0	0.02	#NUM!	8.8E-07
YOR229W	YOR229W	S288C_ctrl_hmg124h_13.JPG.dat	1251.0	34.4	1.0	0.00	#NUM!	7.3E-06
YOR230W	YOR230W	S288C_ctrl_hmg124h_13.JPG.dat	1276.8	8.3	1.0	0.01	#NUM!	2.6E-07
YOR231W	YOR231W	S288C_ctrl_hmg124h_13.JPG.dat	1279.0	26.2	1.0	0.01	#NUM!	1.0E-05
YOR233W	YOR233W	S288C_ctrl_hmg124h_13.JPG.dat	1277.5	31.2	1.0	0.02	#NUM!	3.8E-06
YOR234C	YOR234C	S288C_ctrl_hmg124h_13.JPG.dat	1201.0	53.1	1.0	0.04	#NUM!	1.8E-05
YOR235W	YOR235W	S288C_ctrl_hmg124h_13.JPG.dat	1130.3	44.9	0.8	0.01	#NUM!	6.0E-05
YOR237W	YOR237W	S288C_ctrl_hmg124h_13.JPG.dat	1394.5	26.6	1.1	0.02	#NUM!	1.1E-06
YOR238W	YOR238W	S288C_ctrl_hmg124h_13.JPG.dat	1321.0	13.6	1.0	0.01	#NUM!	4.0E-07
YOR239W	YOR239W	S288C_ctrl_hmg124h_13.JPG.dat	1241.0	51.7	1.0	0.04	#NUM!	1.3E-05
YOR242C	YOR242C	S288C_ctrl_hmg124h_13.JPG.dat	1131.0	28.4	1.0	0.02	#NUM!	3.6E-06
YOR243C	YOR243C	S288C_ctrl_hmg124h_13.JPG.dat	1110.5	34.1	0.9	0.00	#NUM!	2.7E-06
YOR245C	YOR245C	S288C_ctrl_hmg124h_13.JPG.dat	1195.8	17.6	1.0	0.01	#NUM!	4.5E-07
YOR246C	YOR246C	S288C_ctrl_hmg124h_13.JPG.dat	1223.0	19.0	1.0	0.01	#NUM!	3.8E-07
YOR247W	YOR247W	S288C_ctrl_hmg124h_13.JPG.dat	1238.3	38.1	1.0	0.01	#NUM!	2.1E-05
YOR251C	YOR251C	S288C_ctrl_hmg124h_13.JPG.dat	1330.8	25.4	1.0	0.01	#NUM!	1.2E-05
YOR252W	YOR252W	S288C_ctrl_hmg124h_13.JPG.dat	1301.5	72.9	1.0	0.05	#NUM!	3.8E-05
YOR253W	YOR253W	S288C_ctrl_hmg124h_13.JPG.dat	1316.0	17.8	1.0	0.01	#NUM!	2.3E-07
YOR255W	YOR255W	S288C_ctrl_hmg124h_13.JPG.dat	1322.3	23.7	1.0	0.02	#NUM!	2.7E-06
YOR263C	YOR263C	S288C_ctrl_hmg124h_13.JPG.dat	1256.0	21.5	1.0	0.01	#NUM!	1.4E-05
YOR264W	YOR264W	S288C_ctrl_hmg124h_13.JPG.dat	1206.0	23.9	1.0	0.01	#NUM!	5.4E-07
YOR265W	YOR265W	S288C_ctrl_hmg124h_13.JPG.dat	1155.8	31.4	1.0	0.03	#NUM!	6.2E-06
YOR266W	YOR266W	S288C_ctrl_hmg124h_13.JPG.dat	1156.3	18.0	1.0	0.02	#NUM!	1.1E-06
YOR267C	YOR267C	S288C_ctrl_hmg124h_13.JPG.dat	1109.3	15.4	1.0	0.01	#NUM!	3.5E-07
YOR268C	YOR268C	S288C_ctrl_hmg124h_13.JPG.dat	1123.8	48.0	0.9	0.01	#NUM!	2.9E-05
YOR269W	YOR269W	S288C_ctrl_hmg124h_13.JPG.dat	1212.0	36.3	1.0	0.03	#NUM!	5.4E-06
YOR270C	YOR270C	S288C_ctrl_hmg124h_13.JPG.dat	1311.8	13.4	1.0	0.01	#NUM!	3.8E-07
YOR271C	YOR271C	S288C_ctrl_hmg124h_13.JPG.dat	1268.0	78.8	1.0	0.06	#NUM!	7.1E-05
YOR273C	YOR273C	S288C_ctrl_hmg124h_13.JPG.dat	1328.0	23.0	1.0	0.02	#NUM!	1.4E-06
YOR274W	YOR274W	S288C_ctrl_hmg124h_13.JPG.dat	1317.5	5.1	1.0	0.01	#NUM!	1.7E-07
YOR275C	YOR275C	S288C_ctrl_hmg124h_13.JPG.dat	1152.3	28.9	0.9	0.03	#NUM!	9.0E-06
YOR276W	YOR276W	S288C_ctrl_hmg124h_13.JPG.dat	1184.3	22.6	1.0	0.02	#NUM!	3.7E-06
YOR277C	YOR277C	S288C_ctrl_hmg124h_13.JPG.dat	1193.5	15.4	1.0	0.00	#NUM!	6.8E-06
YOR279C	YOR279C	S288C_ctrl_hmg124h_13.JPG.dat	1143.5	6.9	1.0	0.01	#NUM!	3.7E-07
YOR280C	YOR280C	S288C_ctrl_hmg124h_13.JPG.dat	1185.3	5.2	1.0	0.01	#NUM!	1.5E-07
YOR283W	YOR283W	S288C_ctrl_hmg124h_13.JPG.dat	1227.8	15.6	1.0	0.01	#NUM!	6.6E-07
YOR284W	YOR284W	S288C_ctrl_hmg124h_13.JPG.dat	1241.5	27.8	1.0	0.02	#NUM!	2.3E-06
YOR285W	YOR285W	S288C_ctrl_hmg124h_13.JPG.dat	1284.5	16.7	1.0	0.02	#NUM!	2.2E-06
YOR286W	YOR286W	S288C_ctrl_hmg124h_13.JPG.dat	1270.3	93.7	1.0	0.03	#NUM!	2.4E-04
YOR288C	YOR288C	S288C_ctrl_hmg124h_13.JPG.dat	1383.0	22.3	1.1	0.02	#NUM!	1.1E-06
YOR289W	YOR289W	S288C_ctrl_hmg124h_13.JPG.dat	1298.3	13.8	1.0	0.00	#NUM!	1.7E-06
YOR291W	YOR291W	S288C_ctrl_hmg124h_13.JPG.dat	1336.5	36.7	1.0	0.03	#NUM!	4.1E-06
YOR292C	YOR292C	S288C_ctrl_hmg124h_13.JPG.dat	1256.0	24.1	1.0	0.01	#NUM!	3.5E-07
YOR293W	YOR293W	S288C_ctrl_hmg124h_13.JPG.dat	1240.0	11.4	1.0	0.00	#NUM!	1.5E-06
YOR296W	YOR296W	S288C_ctrl_hmg124h_13.JPG.dat	1175.5	22.7	1.0	0.01	#NUM!	5.2E-07
YOR297C	YOR297C	S288C_ctrl_hmg124h_13.JPG.dat	1198.3	21.0	1.0	0.00	#NUM!	1.3E-06
YOR298C-/	YOR298C-A	S288C_ctrl_hmg124h_13.JPG.dat	1192.5	22.1	1.0	0.02	#NUM!	1.1E-06
YOR298W	YOR298W	S288C_ctrl_hmg124h_13.JPG.dat	1234.3	40.4	1.0	0.00	#NUM!	7.4E-06
YOR299W	YOR299W	S288C_ctrl_hmg124h_13.JPG.dat	1252.3	13.5	1.0	0.00	#NUM!	2.6E-06
YOR300W	YOR300W	S288C_ctrl_hmg124h_13.JPG.dat	1301.8	35.8	1.0	0.01	#NUM!	2.3E-05
YOR301W	YOR301W	S288C_ctrl_hmg124h_13.JPG.dat	1261.5	51.1	0.9	0.05	#NUM!	6.0E-05
YOR302W	YOR302W	S288C_ctrl_hmg124h_13.JPG.dat	0.0	0.0	0.0	0.00	#NUM!	
YOR303W	YOR303W	S288C_ctrl_hmg124h_13.JPG.dat	1339.3	23.0	1.0	0.01	#NUM!	2.3E-07
YOR304C-/	YOR304C-A	S288C_ctrl_hmg124h_13.JPG.dat	1411.3	58.8	1.0	0.02	#NUM!	2.8E-06
YOR304W	YOR304W	S288C_ctrl_hmg124h_13.JPG.dat	1307.0	49.9	1.0	0.00	#NUM!	6.0E-07
YOR306C	YOR306C	S288C_ctrl_hmg124h_13.JPG.dat	1365.5	70.2	1.0	0.03	#NUM!	6.3E-06
YOR307C	YOR307C	S288C_ctrl_hmg124h_13.JPG.dat	1379.3	65.8	1.0	0.03	#NUM!	4.1E-06
YOR308C	YOR308C	S288C_ctrl_hmg124h_13.JPG.dat	1324.5	37.3	1.0	0.02	#NUM!	1.2E-06
YOR309C	YOR309C	S288C_ctrl_hmg124h_13.JPG.dat	1307.8	31.6	1.0	0.01	#NUM!	2.8E-07
YOR311C	YOR311C	S288C_ctrl_hmg124h_13.JPG.dat	1265.5	30.0	1.0	0.02	#NUM!	3.6E-06
YOR312C	YOR312C	S288C_ctrl_hmg124h_13.JPG.dat	1168.5	42.4	0.9	0.02	#NUM!	5.8E-06
YOR313C	YOR313C	S288C_ctrl_hmg124h_13.JPG.dat	1298.8	20.4	1.0	0.02	#NUM!	1.6E-06
YOR314W	YOR314W	S288C_ctrl_hmg124h_13.JPG.dat	1314.0	48.2	1.0	0.03	#NUM!	1.1E-05
YOR315W	YOR315W	S288C_ctrl_hmg124h_13.JPG.dat	1329.3	13.0	1.0	0.01	#NUM!	3.9E-07
YOR316C	YOR316C	S288C_ctrl_hmg124h_13.JPG.dat	1304.8	13.6	1.0	0.02	#NUM!	9.7E-07
YOR317W	YOR317W	S288C_ctrl_hmg124h_13.JPG.dat	1262.8	26.9	1.0	0.02	#NUM!	1.9E-06
YOR318C	YOR318C	S288C_ctrl_hmg124h_13.JPG.dat	1301.3	24.3	1.0	0.02	#NUM!	3.9E-06

YOR320C	YOR320C	S288C_ctrl_hmg124h_13.JPG.dat	1220.0	22.1	1.0	0.02	#NUM!	1.3E-06
YOR321W	YOR321W	S288C_ctrl_hmg124h_13.JPG.dat	1300.8	22.2	1.0	0.02	#NUM!	2.2E-06
YOR322C	YOR322C	S288C_ctrl_hmg124h_13.JPG.dat	1250.3	25.0	1.0	0.02	#NUM!	2.8E-06
YOR324C	YOR324C	S288C_ctrl_hmg124h_13.JPG.dat	1294.5	25.1	1.0	0.00	#NUM!	2.9E-06
YOR327C	YOR327C	S288C_ctrl_hmg124h_13.JPG.dat	1280.3	23.8	1.0	0.02	#NUM!	1.8E-06
YOR328W	YOR328W	S288C_ctrl_hmg124h_13.JPG.dat	1378.3	17.0	1.0	0.01	#NUM!	4.0E-07
YOR334W	YOR334W	S288C_ctrl_hmg124h_13.JPG.dat	1366.8	22.2	1.0	0.01	#NUM!	7.2E-07
YOR337W	YOR337W	S288C_ctrl_hmg124h_13.JPG.dat	1343.5	54.9	1.0	0.04	#NUM!	2.0E-05
YOR338W	YOR338W	S288C_ctrl_hmg124h_13.JPG.dat	1303.5	13.3	1.0	0.01	#NUM!	7.4E-08
YOR339C	YOR339C	S288C_ctrl_hmg124h_13.JPG.dat	1176.3	29.7	0.9	0.02	#NUM!	2.1E-06
YOR342C	YOR342C	S288C_ctrl_hmg124h_13.JPG.dat	1235.0	33.6	1.0	0.02	#NUM!	2.5E-06
YOR343C	YOR343C	S288C_ctrl_hmg124h_13.JPG.dat	1174.5	15.8	1.0	0.02	#NUM!	1.8E-06
YOR344C	YOR344C	S288C_ctrl_hmg124h_13.JPG.dat	1240.0	6.4	1.0	0.01	#NUM!	4.4E-07
YOR346W	YOR346W	S288C_ctrl_hmg124h_13.JPG.dat	1294.8	18.6	1.0	0.01	#NUM!	4.8E-07
YOR347C	YOR347C	S288C_ctrl_hmg124h_13.JPG.dat	1288.8	19.2	1.0	0.01	#NUM!	7.4E-07
YOR348C	YOR348C	S288C_ctrl_hmg124h_13.JPG.dat	1367.3	8.6	1.1	0.01	#NUM!	5.1E-07
YOR349W	YOR349W	S288C_ctrl_hmg124h_13.JPG.dat	1375.3	25.7	1.0	0.01	#NUM!	8.7E-06
YOR350C	YOR350C	S288C_ctrl_hmg124h_13.JPG.dat	727.3	61.1	0.5	0.02	#NUM!	2.9E-04
YOR351C	YOR351C	S288C_ctrl_hmg124h_13.JPG.dat	1333.0	33.3	1.0	0.03	#NUM!	9.1E-06
YOR352W	YOR352W	S288C_ctrl_hmg124h_13.JPG.dat	1274.3	30.1	1.0	0.02	#NUM!	9.0E-07
YOR354C	YOR354C	S288C_ctrl_hmg124h_13.JPG.dat	1137.5	5.0	1.0	0.01	#NUM!	3.6E-07
YOR355W	YOR355W	S288C_ctrl_hmg124h_13.JPG.dat	1092.8	15.4	0.9	0.02	#NUM!	3.2E-06
YOR356W	YOR356W	S288C_ctrl_hmg124h_13.JPG.dat	1177.0	28.8	1.0	0.02	#NUM!	1.1E-06
YOR357C	YOR357C	S288C_ctrl_hmg124h_13.JPG.dat	1180.5	17.3	1.0	0.02	#NUM!	4.6E-06
YOR358W	YOR358W	S288C_ctrl_hmg124h_13.JPG.dat	1237.0	60.6	1.0	0.04	#NUM!	1.8E-05
YOR359W	YOR359W	S288C_ctrl_hmg124h_13.JPG.dat	1274.3	24.6	1.0	0.03	#NUM!	4.8E-06
YOR360C	YOR360C	S288C_ctrl_hmg124h_13.JPG.dat	857.0	616.6	0.7	0.48	#NUM!	7.0E-02
YOR363C	YOR363C	S288C_ctrl_hmg124h_13.JPG.dat	1364.5	38.1	1.0	0.02	#NUM!	3.8E-06
YOR364W	YOR364W	S288C_ctrl_hmg124h_13.JPG.dat	1316.8	31.3	1.0	0.02	#NUM!	3.1E-06
YOR365C	YOR365C	S288C_ctrl_hmg124h_13.JPG.dat	1219.0	19.5	1.0	0.00	#NUM!	8.0E-06
YOR367W	YOR367W	S288C_ctrl_hmg124h_13.JPG.dat	1143.8	21.4	0.9	0.02	#NUM!	2.7E-06
YOR368W	YOR368W	S288C_ctrl_hmg124h_13.JPG.dat	1120.0	35.6	1.0	0.03	#NUM!	8.2E-06
YOR371C	YOR371C	S288C_ctrl_hmg124h_13.JPG.dat	1162.3	14.2	1.0	0.00	#NUM!	5.7E-06
YOR374W	YOR374W	S288C_ctrl_hmg124h_13.JPG.dat	1169.0	7.5	1.0	0.00	#NUM!	4.3E-08
YOR375C	YOR375C	S288C_ctrl_hmg124h_13.JPG.dat	1200.3	18.2	1.0	0.01	#NUM!	4.6E-07
YOR376W	YOR376W	S288C_ctrl_hmg124h_13.JPG.dat	1247.5	14.4	1.0	0.01	#NUM!	9.2E-08
YOR377W	YOR377W	S288C_ctrl_hmg124h_13.JPG.dat	1264.0	28.2	1.0	0.02	#NUM!	3.1E-06
YOR378W	YOR378W	S288C_ctrl_hmg124h_13.JPG.dat	1330.3	19.5	1.0	0.01	#NUM!	6.4E-07
YOR380W	YOR380W	S288C_ctrl_hmg124h_13.JPG.dat	1375.5	51.1	1.0	0.03	#NUM!	1.0E-05
YOR381W	YOR381W	S288C_ctrl_hmg124h_13.JPG.dat	1327.5	26.3	1.0	0.02	#NUM!	2.6E-06
YOR382W	YOR382W	S288C_ctrl_hmg124h_13.JPG.dat	1255.8	9.6	1.0	0.00	#NUM!	2.5E-08
YOR383C	YOR383C	S288C_ctrl_hmg124h_13.JPG.dat	1229.0	18.2	1.0	0.01	#NUM!	2.6E-05
YOR384W	YOR384W	S288C_ctrl_hmg124h_13.JPG.dat	1162.8	30.1	1.0	0.02	#NUM!	3.4E-06
YOR385W	YOR385W	S288C_ctrl_hmg124h_13.JPG.dat	1165.8	13.8	1.0	0.01	#NUM!	8.3E-07
YOR386W	YOR386W	S288C_ctrl_hmg124h_13.JPG.dat	1149.0	29.2	1.0	0.02	#NUM!	3.1E-06
YPL001W	YPL001W	S288C_ctrl_hmg124h_13.JPG.dat	1135.0	43.5	1.0	0.04	#NUM!	1.7E-05
YPL003W	YPL003W	S288C_ctrl_hmg124h_13.JPG.dat	1241.5	13.5	1.0	0.01	#NUM!	1.7E-07
YPL004C	YPL004C	S288C_ctrl_hmg124h_13.JPG.dat	1241.8	46.1	1.0	0.01	#NUM!	1.9E-05
YPL008W	YPL008W	S288C_ctrl_hmg124h_13.JPG.dat	1304.0	23.6	1.0	0.02	#NUM!	2.1E-06
YPL009C	YPL009C	S288C_ctrl_hmg124h_13.JPG.dat	1324.5	43.8	1.0	0.04	#NUM!	1.2E-05
YPL014W	YPL014W	S288C_ctrl_hmg124h_13.JPG.dat	1317.3	9.0	1.0	0.00	#NUM!	9.9E-09
YPL017C	YPL017C	S288C_ctrl_hmg124h_13.JPG.dat	1245.8	23.8	1.0	0.00	#NUM!	8.7E-06
YPL018W	YPL018W	S288C_ctrl_hmg124h_13.JPG.dat	1297.8	20.5	1.0	0.01	#NUM!	8.0E-06
YPL019C	YPL019C	S288C_ctrl_hmg124h_13.JPG.dat	1201.5	12.2	1.0	0.01	#NUM!	1.4E-07
YPL021W	YPL021W	S288C_ctrl_hmg124h_13.JPG.dat	1199.3	20.4	1.0	0.00	#NUM!	5.6E-06
YPL022W	YPL022W	S288C_ctrl_hmg124h_13.JPG.dat	1167.0	34.4	1.0	0.02	#NUM!	2.6E-06
YPL023C	YPL023C	S288C_ctrl_hmg124h_13.JPG.dat	1178.0	41.2	1.0	0.02	#NUM!	4.6E-06
YPL024W	YPL024W	S288C_ctrl_hmg124h_13.JPG.dat	1039.0	29.4	0.8	0.02	#NUM!	2.7E-06
YPL025C	YPL025C	S288C_ctrl_hmg124h_13.JPG.dat	1292.3	21.8	1.0	0.02	#NUM!	1.9E-06
YPL026C	YPL026C	S288C_ctrl_hmg124h_13.JPG.dat	1266.0	19.9	1.0	0.02	#NUM!	2.0E-06
YPL027W	YPL027W	S288C_ctrl_hmg124h_13.JPG.dat	1242.3	104.5	0.9	0.08	#NUM!	1.9E-04
YPL030W	YPL030W	S288C_ctrl_hmg124h_13.JPG.dat	1327.8	89.5	1.0	0.08	#NUM!	1.7E-04
YPL032C	YPL032C	S288C_ctrl_hmg124h_13.JPG.dat	1432.3	27.4	1.0	0.01	#NUM!	2.0E-05
YPL033C	YPL033C	S288C_ctrl_hmg124h_13.JPG.dat	1403.5	49.2	1.0	0.01	#NUM!	6.1E-07
YPL034W	YPL034W	S288C_ctrl_hmg124h_13.JPG.dat	1347.3	18.1	1.0	0.01	#NUM!	5.7E-07
YPL036W	YPL036W	S288C_ctrl_hmg124h_13.JPG.dat	1341.0	65.8	1.0	0.01	#NUM!	4.5E-05
YPL037C	YPL037C	S288C_ctrl_hmg124h_13.JPG.dat	1322.0	38.1	1.0	0.02	#NUM!	4.1E-06
YPL038W	YPL038W	S288C_ctrl_hmg124h_13.JPG.dat	1352.0	25.5	1.0	0.01	#NUM!	5.1E-07
YPL039W	YPL039W	S288C_ctrl_hmg124h_13.JPG.dat	1328.5	51.4	1.0	0.03	#NUM!	8.4E-06
YPL041C	YPL041C	S288C_ctrl_hmg124h_13.JPG.dat	1316.3	44.2	1.0	0.01	#NUM!	1.6E-05

YPL046C	YPL046C	S288C_ctrl_hmg124h_13.JPG.dat	1305.5	28.4	1.0	0.01	#NUM!	2.4E-07
YPL047W	YPL047W	S288C_ctrl_hmg124h_13.JPG.dat	1328.8	20.1	1.0	0.01	#NUM!	2.4E-05
YPL048W	YPL048W	S288C_ctrl_hmg124h_13.JPG.dat	1403.3	106.9	1.0	0.06	#NUM!	5.3E-05
YPL051W	YPL051W	S288C_ctrl_hmg124h_13.JPG.dat	1410.3	43.4	1.0	0.02	#NUM!	1.4E-06
YPL052W	YPL052W	S288C_ctrl_hmg124h_13.JPG.dat	1398.8	35.2	1.0	0.02	#NUM!	1.9E-06
YPL053C	YPL053C	S288C_ctrl_hmg124h_13.JPG.dat	1375.8	17.2	1.0	0.03	#NUM!	7.8E-06
YPL054W	YPL054W	S288C_ctrl_hmg124h_13.JPG.dat	1315.3	29.5	1.0	0.02	#NUM!	3.1E-06
YPL055C	YPL055C	S288C_ctrl_hmg124h_13.JPG.dat	1330.3	44.3	1.0	0.02	#NUM!	9.3E-07
YPL056C	YPL056C	S288C_ctrl_hmg124h_13.JPG.dat	1338.8	29.4	1.0	0.01	#NUM!	2.4E-07
YPL057C	YPL057C	S288C_ctrl_hmg124h_13.JPG.dat	1291.8	56.0	1.0	0.04	#NUM!	2.1E-05
YPL058C	YPL058C	S288C_ctrl_hmg124h_13.JPG.dat	1265.0	45.4	1.0	0.00	#NUM!	2.6E-06
YPL060W	YPL060W	S288C_ctrl_hmg124h_13.JPG.dat	1187.3	108.2	0.9	0.02	#NUM!	1.4E-04
YPL061W	YPL061W	S288C_ctrl_hmg124h_13.JPG.dat	1236.5	54.2	1.0	0.05	#NUM!	3.0E-05
YPL062W	YPL062W	S288C_ctrl_hmg124h_13.JPG.dat	1351.3	42.6	1.0	0.04	#NUM!	1.6E-05
YPL064C	YPL064C	S288C_ctrl_hmg124h_13.JPG.dat	1284.5	25.3	1.0	0.01	#NUM!	3.2E-07
YPL066W	YPL066W	S288C_ctrl_hmg124h_13.JPG.dat	1217.0	18.7	1.0	0.03	#NUM!	5.2E-06
YPL067C	YPL067C	S288C_ctrl_hmg124h_13.JPG.dat	1231.5	61.7	1.0	0.04	#NUM!	1.5E-05
YPL068C	YPL068C	S288C_ctrl_hmg124h_13.JPG.dat	1213.5	40.9	1.0	0.01	#NUM!	1.3E-05
YPL069C	YPL069C	S288C_ctrl_hmg124h_13.JPG.dat	1205.5	33.8	1.0	0.02	#NUM!	1.5E-06
YPL070W	YPL070W	S288C_ctrl_hmg124h_13.JPG.dat	1260.8	34.5	1.0	0.03	#NUM!	8.3E-06
YPL071C	YPL071C	S288C_ctrl_hmg124h_13.JPG.dat	1334.0	23.6	1.1	0.01	#NUM!	5.4E-07
YPL073C	YPL073C	S288C_ctrl_hmg124h_13.JPG.dat	1261.8	74.0	1.0	0.02	#NUM!	9.4E-05
YPL074W	YPL074W	S288C_ctrl_hmg124h_13.JPG.dat	1251.8	36.0	1.0	0.03	#NUM!	4.7E-06
YPL079W	YPL079W	S288C_ctrl_hmg124h_13.JPG.dat	1193.0	29.0	0.9	0.02	#NUM!	3.1E-06
YPL080C	YPL080C	S288C_ctrl_hmg124h_13.JPG.dat	1197.0	17.8	0.9	0.02	#NUM!	1.6E-06
YPL081W	YPL081W	S288C_ctrl_hmg124h_13.JPG.dat	1269.5	29.0	1.0	0.02	#NUM!	2.4E-06
YPL086C	YPL086C	S288C_ctrl_hmg124h_13.JPG.dat	1075.0	114.3	0.8	0.01	#NUM!	4.3E-05
YPL087W	YPL087W	S288C_ctrl_hmg124h_13.JPG.dat	1146.3	22.5	1.0	0.01	#NUM!	1.0E-06
YPL088W	YPL088W	S288C_ctrl_hmg124h_13.JPG.dat	1147.8	11.9	1.0	0.01	#NUM!	4.6E-07
YPL089C	YPL089C	S288C_ctrl_hmg124h_13.JPG.dat	1167.3	14.9	1.0	0.02	#NUM!	1.6E-06
YPL090C	YPL090C	S288C_ctrl_hmg124h_13.JPG.dat	1181.8	74.7	1.0	0.06	#NUM!	6.1E-05
YPL091W	YPL091W	S288C_ctrl_hmg124h_13.JPG.dat	1286.0	23.6	1.0	0.02	#NUM!	1.0E-06
YPL092W	YPL092W	S288C_ctrl_hmg124h_13.JPG.dat	1328.8	19.5	1.0	0.02	#NUM!	8.9E-07
YPL095C	YPL095C	S288C_ctrl_hmg124h_13.JPG.dat	1242.8	43.3	1.0	0.04	#NUM!	2.0E-05
YPL096W	YPL096W	S288C_ctrl_hmg124h_13.JPG.dat	1412.5	24.0	1.1	0.02	#NUM!	2.3E-06
YPL098C	YPL098C	S288C_ctrl_hmg124h_13.JPG.dat	1207.8	9.0	0.9	0.01	#NUM!	2.2E-07
YPL099C	YPL099C	S288C_ctrl_hmg124h_13.JPG.dat	1297.8	17.9	1.0	0.01	#NUM!	7.6E-07
YPL100W	YPL100W	S288C_ctrl_hmg124h_13.JPG.dat	1165.5	35.1	1.0	0.01	#NUM!	2.8E-05
YPL101W	YPL101W	S288C_ctrl_hmg124h_13.JPG.dat	1180.8	32.2	1.0	0.03	#NUM!	5.0E-06
YPL102C	YPL102C	S288C_ctrl_hmg124h_13.JPG.dat	1170.0	47.7	1.0	0.01	#NUM!	2.4E-05
YPL103C	YPL103C	S288C_ctrl_hmg124h_13.JPG.dat	1131.3	20.7	1.0	0.01	#NUM!	7.8E-07
YPL105C	YPL105C	S288C_ctrl_hmg124h_13.JPG.dat	1215.5	9.3	1.0	0.01	#NUM!	5.8E-07
YPL106C	YPL106C	S288C_ctrl_hmg124h_13.JPG.dat	1247.0	26.4	1.0	0.00	#NUM!	1.1E-06
YPL107W	YPL107W	S288C_ctrl_hmg124h_13.JPG.dat	1300.5	53.4	1.0	0.04	#NUM!	1.7E-05
YPL108W	YPL108W	S288C_ctrl_hmg124h_13.JPG.dat	1278.0	53.7	1.0	0.04	#NUM!	2.4E-05
YPL109C	YPL109C	S288C_ctrl_hmg124h_13.JPG.dat	1377.8	58.8	1.0	0.04	#NUM!	1.6E-05
YPL110C	YPL110C	S288C_ctrl_hmg124h_13.JPG.dat	1290.3	13.8	1.0	0.01	#NUM!	7.0E-08
YPL111W	YPL111W	S288C_ctrl_hmg124h_13.JPG.dat	1241.3	45.6	1.0	0.03	#NUM!	7.9E-06
YPL112C	YPL112C	S288C_ctrl_hmg124h_13.JPG.dat	1160.5	14.8	1.0	0.00	#NUM!	8.6E-07
YPL113C	YPL113C	S288C_ctrl_hmg124h_13.JPG.dat	1185.5	24.7	1.0	0.02	#NUM!	3.6E-06
YPL114W	YPL114W	S288C_ctrl_hmg124h_13.JPG.dat	1136.5	31.7	1.0	0.03	#NUM!	6.9E-06
YPL115C	YPL115C	S288C_ctrl_hmg124h_13.JPG.dat	1127.8	16.0	1.0	0.02	#NUM!	2.4E-06
YPL116W	YPL116W	S288C_ctrl_hmg124h_13.JPG.dat	1184.3	13.0	1.0	0.00	#NUM!	2.0E-06
YPL119C	YPL119C	S288C_ctrl_hmg124h_13.JPG.dat	1248.8	23.1	1.0	0.02	#NUM!	1.2E-06
YPL120W	YPL120W	S288C_ctrl_hmg124h_13.JPG.dat	1208.3	48.4	1.0	0.01	#NUM!	1.5E-05
YPL121C	YPL121C	S288C_ctrl_hmg124h_13.JPG.dat	1347.5	24.6	1.0	0.00	#NUM!	2.8E-07
YPL123C	YPL123C	S288C_ctrl_hmg124h_13.JPG.dat	1364.5	35.4	1.0	0.02	#NUM!	3.6E-06
YPL125W	YPL125W	S288C_ctrl_hmg124h_13.JPG.dat	1282.8	20.1	1.0	0.01	#NUM!	5.5E-07
YPL127C	YPL127C	S288C_ctrl_hmg124h_13.JPG.dat	1208.3	11.3	1.0	0.01	#NUM!	1.6E-07
YPL130W	YPL130W	S288C_ctrl_hmg124h_13.JPG.dat	1230.5	11.9	1.0	0.01	#NUM!	5.3E-07
YPL133C	YPL133C	S288C_ctrl_hmg124h_13.JPG.dat	1156.5	23.6	1.0	0.02	#NUM!	1.4E-06
YPL134C	YPL134C	S288C_ctrl_hmg124h_13.JPG.dat	1148.0	12.3	1.0	0.01	#NUM!	4.7E-07
YPL135W	YPL135W	S288C_ctrl_hmg124h_13.JPG.dat	1155.8	37.8	1.0	0.03	#NUM!	7.2E-06
YPL136W	YPL136W	S288C_ctrl_hmg124h_13.JPG.dat	1221.3	16.6	1.0	0.01	#NUM!	3.1E-07
YPL137C	YPL137C	S288C_ctrl_hmg124h_13.JPG.dat	1206.8	18.3	1.0	0.01	#NUM!	6.5E-07
YPL138C	YPL138C	S288C_ctrl_hmg124h_13.JPG.dat	1230.8	17.6	1.0	0.02	#NUM!	2.0E-06
YPL139C	YPL139C	S288C_ctrl_hmg124h_13.JPG.dat	1318.0	36.9	1.0	0.00	#NUM!	7.4E-06
YPL140C	YPL140C	S288C_ctrl_hmg124h_13.JPG.dat	1268.8	95.7	1.0	0.08	#NUM!	1.5E-04
YPL141C	YPL141C	S288C_ctrl_hmg124h_13.JPG.dat	1373.0	55.0	1.0	0.03	#NUM!	1.0E-05
YPL144W	YPL144W	S288C_ctrl_hmg124h_13.JPG.dat	1343.0	34.4	1.0	0.03	#NUM!	4.2E-06

YPL145C	YPL145C	S288C_ctrl_hmg124h_13.JPG.dat	1296.0	17.9	1.0	0.01	#NUM!	6.6E-07
YPL147W	YPL147W	S288C_ctrl_hmg124h_13.JPG.dat	1268.5	19.7	1.0	0.01	#NUM!	5.2E-07
YPL149W	YPL149W	S288C_ctrl_hmg124h_13.JPG.dat	1259.3	41.5	1.0	0.02	#NUM!	2.6E-06
YPL150W	YPL150W	S288C_ctrl_hmg124h_13.JPG.dat	1225.0	21.4	1.0	0.00	#NUM!	1.4E-06
YPL152W	YPL152W	S288C_ctrl_hmg124h_13.JPG.dat	1277.8	26.7	1.0	0.00	#NUM!	2.9E-06
YPL154C	YPL154C	S288C_ctrl_hmg124h_13.JPG.dat	1247.0	20.1	1.0	0.01	#NUM!	1.4E-05
YPL155C	YPL155C	S288C_ctrl_hmg124h_13.JPG.dat	1288.0	6.3	1.0	0.01	#NUM!	1.9E-07
YPL156C	YPL156C	S288C_ctrl_hmg124h_13.JPG.dat	1277.3	14.2	1.0	0.01	#NUM!	2.2E-07
YPL157W	YPL157W	S288C_ctrl_hmg124h_13.JPG.dat	1124.3	75.5	0.8	0.01	#NUM!	5.9E-05
YPL159C	YPL159C	S288C_ctrl_hmg124h_13.JPG.dat	1297.5	120.1	0.9	0.07	#NUM!	1.1E-04
YPL161C	YPL161C	S288C_ctrl_hmg124h_13.JPG.dat	1360.3	72.6	1.0	0.03	#NUM!	9.8E-06
YPL162C	YPL162C	S288C_ctrl_hmg124h_13.JPG.dat	1356.8	59.8	1.0	0.03	#NUM!	1.2E-05
YPL163C	YPL163C	S288C_ctrl_hmg124h_13.JPG.dat	1398.3	33.5	1.0	0.01	#NUM!	4.5E-07
YPL164C	YPL164C	S288C_ctrl_hmg124h_13.JPG.dat	1275.5	42.1	1.0	0.01	#NUM!	3.5E-07
YPL165C	YPL165C	S288C_ctrl_hmg124h_13.JPG.dat	1343.0	55.0	1.0	0.03	#NUM!	4.4E-06
YPL166W	YPL166W	S288C_ctrl_hmg124h_13.JPG.dat	1316.0	34.2	1.0	0.01	#NUM!	8.7E-08
YPL167C	YPL167C	S288C_ctrl_hmg124h_13.JPG.dat	1369.5	48.3	1.0	0.03	#NUM!	6.3E-06
YPL168W	YPL168W	S288C_ctrl_hmg124h_13.JPG.dat	1326.5	45.0	1.0	0.04	#NUM!	2.0E-05
YPL170W	YPL170W	S288C_ctrl_hmg124h_13.JPG.dat	1306.3	15.7	1.0	0.02	#NUM!	1.6E-06
YPL171C	YPL171C	S288C_ctrl_hmg124h_13.JPG.dat	1292.0	38.9	1.0	0.03	#NUM!	8.0E-06
YPL172C	YPL172C	S288C_ctrl_hmg124h_13.JPG.dat	1029.5	403.0	0.8	0.31	#NUM!	1.5E-02
YPL174C	YPL174C	S288C_ctrl_hmg124h_13.JPG.dat	680.0	36.4	0.5	0.03	#NUM!	4.6E-05
YPL176C	YPL176C	S288C_ctrl_hmg124h_13.JPG.dat	1258.3	23.0	1.0	0.01	#NUM!	9.7E-06
YPL177C	YPL177C	S288C_ctrl_hmg124h_13.JPG.dat	1268.0	41.6	1.0	0.04	#NUM!	1.2E-05
YPL178W	YPL178W	S288C_ctrl_hmg124h_13.JPG.dat	717.3	34.8	0.6	0.03	#NUM!	3.3E-05
YPL179W	YPL179W	S288C_ctrl_hmg124h_13.JPG.dat	1201.0	16.9	1.0	0.02	#NUM!	2.9E-06
YPL180W	YPL180W	S288C_ctrl_hmg124h_13.JPG.dat	1294.8	7.3	1.0	0.02	#NUM!	9.8E-07
YPL181W	YPL181W	S288C_ctrl_hmg124h_13.JPG.dat	1289.5	56.1	1.0	0.04	#NUM!	1.3E-05
YPL182C	YPL182C	S288C_ctrl_hmg124h_13.JPG.dat	1205.8	18.1	0.9	0.00	#NUM!	6.4E-07
YPL183C	YPL183C	S288C_ctrl_hmg124h_13.JPG.dat	1212.0	29.5	1.0	0.03	#NUM!	4.9E-06
YPL183W-/	YPL183W-A	S288C_ctrl_hmg124h_13.JPG.dat	728.5	45.8	0.6	0.04	#NUM!	7.0E-05
YPL184C	YPL184C	S288C_ctrl_hmg124h_13.JPG.dat	1300.5	22.8	1.0	0.02	#NUM!	1.9E-06
YPL185W	YPL185W	S288C_ctrl_hmg124h_13.JPG.dat	1343.3	24.2	1.0	0.02	#NUM!	2.8E-06
YPL186C	YPL186C	S288C_ctrl_hmg124h_13.JPG.dat	1240.5	30.6	1.0	0.02	#NUM!	1.2E-06
YPL187W	YPL187W	S288C_ctrl_hmg124h_13.JPG.dat	1225.5	19.5	1.0	0.02	#NUM!	1.9E-06
YPL189W	YPL189W	S288C_ctrl_hmg124h_13.JPG.dat	1211.3	56.9	1.0	0.01	#NUM!	2.3E-05
YPL191C	YPL191C	S288C_ctrl_hmg124h_13.JPG.dat	1200.8	17.8	1.0	0.01	#NUM!	6.8E-07
YPL192C	YPL192C	S288C_ctrl_hmg124h_13.JPG.dat	1250.8	31.1	1.0	0.02	#NUM!	1.3E-06
YPL194W	YPL194W	S288C_ctrl_hmg124h_13.JPG.dat	1271.3	11.8	1.0	0.01	#NUM!	1.0E-07
YPL195W	YPL195W	S288C_ctrl_hmg124h_13.JPG.dat	1280.8	27.0	1.0	0.00	#NUM!	6.6E-06
YPL196W	YPL196W	S288C_ctrl_hmg124h_13.JPG.dat	1292.8	30.1	1.0	0.02	#NUM!	2.5E-06
YPL197C	YPL197C	S288C_ctrl_hmg124h_13.JPG.dat	1344.0	40.3	1.0	0.00	#NUM!	6.9E-06
YPL198W	YPL198W	S288C_ctrl_hmg124h_13.JPG.dat	1327.5	38.2	1.0	0.03	#NUM!	5.8E-06
YPL199C	YPL199C	S288C_ctrl_hmg124h_13.JPG.dat	1319.8	20.0	1.0	0.01	#NUM!	3.3E-07
YPL200W	YPL200W	S288C_ctrl_hmg124h_13.JPG.dat	1232.8	36.0	1.0	0.02	#NUM!	3.9E-06
YPL201C	YPL201C	S288C_ctrl_hmg124h_13.JPG.dat	1187.0	25.1	1.0	0.02	#NUM!	3.2E-06
YPL202C	YPL202C	S288C_ctrl_hmg124h_13.JPG.dat	1227.0	23.5	1.1	0.02	#NUM!	2.6E-06
YPL203W	YPL203W	S288C_ctrl_hmg124h_13.JPG.dat	1188.5	15.2	1.0	0.01	#NUM!	4.9E-07
YPL205C	YPL205C	S288C_ctrl_hmg124h_13.JPG.dat	1142.8	134.2	1.0	0.03	#NUM!	2.1E-04
YPL206C	YPL206C	S288C_ctrl_hmg124h_13.JPG.dat	1278.5	17.5	1.0	0.01	#NUM!	3.7E-07
YPL207W	YPL207W	S288C_ctrl_hmg124h_13.JPG.dat	1216.8	39.8	1.0	0.03	#NUM!	8.1E-06
YPL208W	YPL208W	S288C_ctrl_hmg124h_13.JPG.dat	1314.5	24.6	1.0	0.02	#NUM!	1.3E-06
YPL212C	YPL212C	S288C_ctrl_hmg124h_13.JPG.dat	1341.0	103.9	1.0	0.08	#NUM!	1.2E-04
YPL213W	YPL213W	S288C_ctrl_hmg124h_13.JPG.dat	1285.0	48.9	1.0	0.04	#NUM!	1.3E-05
YPL214C	YPL214C	S288C_ctrl_hmg124h_13.JPG.dat	1262.0	9.4	1.0	0.01	#NUM!	1.9E-07
YPL216W	YPL216W	S288C_ctrl_hmg124h_13.JPG.dat	1222.5	8.7	1.0	0.01	#NUM!	1.9E-07
YPL219W	YPL219W	S288C_ctrl_hmg124h_13.JPG.dat	1166.8	19.2	1.0	0.00	#NUM!	6.0E-06
YPL220W	YPL220W	S288C_ctrl_hmg124h_13.JPG.dat	1168.8	13.5	1.0	0.01	#NUM!	5.5E-07
YPL221W	YPL221W	S288C_ctrl_hmg124h_13.JPG.dat	1140.3	43.8	1.0	0.04	#NUM!	1.8E-05
YPL222W	YPL222W	S288C_ctrl_hmg124h_13.JPG.dat	1209.5	26.5	1.0	0.03	#NUM!	5.3E-06
YPL223C	YPL223C	S288C_ctrl_hmg124h_13.JPG.dat	1177.3	23.9	1.0	0.00	#NUM!	5.6E-06
YPL224C	YPL224C	S288C_ctrl_hmg124h_13.JPG.dat	1249.3	36.2	1.0	0.03	#NUM!	7.3E-06
YPL225W	YPL225W	S288C_ctrl_hmg124h_13.JPG.dat	1256.8	41.2	1.0	0.03	#NUM!	9.7E-06
YPL226W	YPL226W	S288C_ctrl_hmg124h_13.JPG.dat	1316.0	29.2	1.0	0.02	#NUM!	3.2E-06
YPL227C	YPL227C	S288C_ctrl_hmg124h_13.JPG.dat	1302.3	12.6	1.0	0.01	#NUM!	1.4E-07
YPL229W	YPL229W	S288C_ctrl_hmg124h_13.JPG.dat	1288.3	22.3	1.0	0.00	#NUM!	6.6E-07
YPL230W	YPL230W	S288C_ctrl_hmg124h_13.JPG.dat	1256.3	18.9	1.0	0.00	#NUM!	1.1E-06
YPL232W	YPL232W	S288C_ctrl_hmg124h_13.JPG.dat	1206.3	47.6	1.0	0.01	#NUM!	2.0E-05
YPL236C	YPL236C	S288C_ctrl_hmg124h_13.JPG.dat	1111.0	16.1	0.9	0.01	#NUM!	6.2E-07
YPL239W	YPL239W	S288C_ctrl_hmg124h_13.JPG.dat	1181.0	22.7	1.0	0.02	#NUM!	9.7E-07

YPL240C	YPL240C	S288C_ctrl_hmg124h_13.JPG.dat	1228.0	6.6	1.0	0.01	#NUM!	2.7E-07
YPL241C	YPL241C	S288C_ctrl_hmg124h_13.JPG.dat	1212.5	41.4	1.0	0.00	#NUM!	4.9E-06
YPL244C	YPL244C	S288C_ctrl_hmg124h_13.JPG.dat	1280.0	16.3	1.0	0.01	#NUM!	3.8E-07
YPL245W	YPL245W	S288C_ctrl_hmg124h_13.JPG.dat	1281.3	34.4	1.0	0.03	#NUM!	4.6E-06
YPL246C	YPL246C	S288C_ctrl_hmg124h_13.JPG.dat	1348.0	39.8	1.0	0.03	#NUM!	1.1E-05
YPL247C	YPL247C	S288C_ctrl_hmg124h_13.JPG.dat	1390.8	19.7	1.0	0.01	#NUM!	1.7E-07
YPL248C	YPL248C	S288C_ctrl_hmg124h_13.JPG.dat	1329.5	47.7	1.0	0.03	#NUM!	6.0E-06
YPL249C	YPL249C	S288C_ctrl_hmg124h_13.JPG.dat	1283.0	59.4	1.0	0.01	#NUM!	7.8E-05
YPL250C	YPL250C	S288C_ctrl_hmg124h_13.JPG.dat	1240.5	43.1	1.0	0.02	#NUM!	3.8E-06
YPL253C	YPL253C	S288C_ctrl_hmg124h_13.JPG.dat	1256.3	14.1	1.0	0.00	#NUM!	2.7E-08
YPL256C	YPL256C	S288C_ctrl_hmg124h_13.JPG.dat	1262.0	60.5	1.0	0.01	#NUM!	8.1E-06
YPL257W	YPL257W	S288C_ctrl_hmg124h_13.JPG.dat	1271.5	20.6	1.0	0.01	#NUM!	1.0E-05
YPL258C	YPL258C	S288C_ctrl_hmg124h_13.JPG.dat	1316.0	19.3	1.0	0.02	#NUM!	1.6E-06
YPL259C	YPL259C	S288C_ctrl_hmg124h_13.JPG.dat	1243.5	19.1	1.0	0.00	#NUM!	8.1E-06
YPL260W	YPL260W	S288C_ctrl_hmg124h_13.JPG.dat	1270.5	34.5	1.0	0.03	#NUM!	7.8E-06
1	1	S288C_ctrl_hmg124h_3.JPG.dat	1516.5	163.8	1.0	0.09	#NUM!	2.0E-04
2	2	S288C_ctrl_hmg124h_3.JPG.dat	1284.5	71.2	0.9	0.02	#NUM!	1.2E-04
3	3	S288C_ctrl_hmg124h_3.JPG.dat	1373.0	110.9	1.0	0.07	#NUM!	9.6E-05
4	4	S288C_ctrl_hmg124h_3.JPG.dat	1352.0	100.9	1.0	0.07	#NUM!	7.3E-05
YDL185W	YDL185W	S288C_ctrl_hmg124h_3.JPG.dat	1439.3	50.0	1.0	0.03	#NUM!	7.2E-06
YDL186W	YDL186W	S288C_ctrl_hmg124h_3.JPG.dat	1366.8	59.1	1.0	0.01	#NUM!	4.1E-05
YDL187C	YDL187C	S288C_ctrl_hmg124h_3.JPG.dat	1423.0	28.5	1.0	0.02	#NUM!	1.7E-06
YDL188C	YDL188C	S288C_ctrl_hmg124h_3.JPG.dat	1390.3	41.6	1.0	0.02	#NUM!	3.7E-06
YDL189W	YDL189W	S288C_ctrl_hmg124h_3.JPG.dat	1375.5	17.4	1.0	0.02	#NUM!	2.7E-06
YDL190C	YDL190C	S288C_ctrl_hmg124h_3.JPG.dat	1358.0	24.6	1.0	0.01	#NUM!	1.1E-05
YDL191W	YDL191W	S288C_ctrl_hmg124h_3.JPG.dat	1224.8	96.8	0.9	0.08	#NUM!	1.6E-04
YDL192W	YDL192W	S288C_ctrl_hmg124h_3.JPG.dat	1256.5	6.5	0.9	0.00	#NUM!	1.8E-07
YDL194W	YDL194W	S288C_ctrl_hmg124h_3.JPG.dat	1339.0	46.2	1.0	0.03	#NUM!	8.0E-06
YDL197C	YDL197C	S288C_ctrl_hmg124h_3.JPG.dat	1294.5	29.5	1.0	0.01	#NUM!	2.8E-05
YDL199C	YDL199C	S288C_ctrl_hmg124h_3.JPG.dat	1439.0	92.0	1.1	0.07	#NUM!	6.8E-05
YDL200C	YDL200C	S288C_ctrl_hmg124h_3.JPG.dat	1353.3	39.6	0.9	0.01	#NUM!	4.3E-05
YDL201W	YDL201W	S288C_ctrl_hmg124h_3.JPG.dat	1363.3	33.6	1.0	0.03	#NUM!	4.8E-06
YDL203C	YDL203C	S288C_ctrl_hmg124h_3.JPG.dat	1355.5	23.3	1.0	0.02	#NUM!	1.5E-06
YDL204W	YDL204W	S288C_ctrl_hmg124h_3.JPG.dat	1296.8	16.1	1.0	0.01	#NUM!	9.8E-07
YDL206W	YDL206W	S288C_ctrl_hmg124h_3.JPG.dat	1316.0	45.5	1.0	0.03	#NUM!	1.1E-05
YDL210W	YDL210W	S288C_ctrl_hmg124h_3.JPG.dat	1279.5	57.0	1.0	0.01	#NUM!	3.6E-05
YDL211C	YDL211C	S288C_ctrl_hmg124h_3.JPG.dat	1273.3	34.6	1.0	0.02	#NUM!	3.2E-06
YDL213C	YDL213C	S288C_ctrl_hmg124h_3.JPG.dat	1288.3	29.4	1.0	0.02	#NUM!	3.6E-06
YDL214C	YDL214C	S288C_ctrl_hmg124h_3.JPG.dat	1413.0	36.3	1.1	0.03	#NUM!	5.1E-06
YDL215C	YDL215C	S288C_ctrl_hmg124h_3.JPG.dat	1344.3	48.6	1.0	0.01	#NUM!	2.6E-05
YDL216C	YDL216C	S288C_ctrl_hmg124h_3.JPG.dat	1383.5	89.0	1.0	0.01	#NUM!	3.8E-05
YDL218W	YDL218W	S288C_ctrl_hmg124h_3.JPG.dat	1483.8	58.3	1.0	0.04	#NUM!	1.5E-05
YDL219W	YDL219W	S288C_ctrl_hmg124h_3.JPG.dat	1380.5	38.2	1.0	0.03	#NUM!	1.0E-05
YDL222C	YDL222C	S288C_ctrl_hmg124h_3.JPG.dat	1360.0	29.8	1.0	0.01	#NUM!	1.0E-05
YDL223C	YDL223C	S288C_ctrl_hmg124h_3.JPG.dat	1311.8	22.2	1.0	0.01	#NUM!	3.8E-07
YDL224C	YDL224C	S288C_ctrl_hmg124h_3.JPG.dat	1260.3	6.5	1.0	0.01	#NUM!	1.7E-07
YDL226C	YDL226C	S288C_ctrl_hmg124h_3.JPG.dat	1271.5	46.4	1.0	0.00	#NUM!	2.2E-06
YDL227C	YDL227C	S288C_ctrl_hmg124h_3.JPG.dat	1203.8	56.6	1.0	0.05	#NUM!	3.1E-05
YDL229W	YDL229W	S288C_ctrl_hmg124h_3.JPG.dat	1277.0	19.4	1.0	0.01	#NUM!	2.5E-07
YDL230W	YDL230W	S288C_ctrl_hmg124h_3.JPG.dat	1374.0	35.7	1.0	0.02	#NUM!	2.8E-06
YDL231C	YDL231C	S288C_ctrl_hmg124h_3.JPG.dat	1341.3	40.8	1.0	0.03	#NUM!	1.0E-05
YDL232W	YDL232W	S288C_ctrl_hmg124h_3.JPG.dat	1004.8	581.5	0.7	0.42	#NUM!	4.1E-02
YDL233W	YDL233W	S288C_ctrl_hmg124h_3.JPG.dat	1423.0	30.3	1.0	0.00	#NUM!	4.7E-07
YDL234C	YDL234C	S288C_ctrl_hmg124h_3.JPG.dat	1394.3	42.5	1.0	0.03	#NUM!	5.2E-06
YDL236W	YDL236W	S288C_ctrl_hmg124h_3.JPG.dat	1321.0	24.3	1.0	0.02	#NUM!	2.9E-06
YDL237W	YDL237W	S288C_ctrl_hmg124h_3.JPG.dat	1216.8	13.3	1.0	0.01	#NUM!	3.2E-07
YDL238C	YDL238C	S288C_ctrl_hmg124h_3.JPG.dat	1265.0	28.3	1.0	0.02	#NUM!	1.5E-06
YDL239C	YDL239C	S288C_ctrl_hmg124h_3.JPG.dat	1224.8	45.7	1.0	0.03	#NUM!	9.2E-06
YDL240W	YDL240W	S288C_ctrl_hmg124h_3.JPG.dat	1174.3	25.7	1.0	0.02	#NUM!	3.9E-06
YDL241W	YDL241W	S288C_ctrl_hmg124h_3.JPG.dat	1209.3	35.4	1.0	0.03	#NUM!	5.4E-06
YDL242W	YDL242W	S288C_ctrl_hmg124h_3.JPG.dat	1282.5	43.5	1.0	0.04	#NUM!	1.2E-05
YDL243C	YDL243C	S288C_ctrl_hmg124h_3.JPG.dat	1324.5	22.7	1.0	0.02	#NUM!	2.5E-06
YDR001C	YDR001C	S288C_ctrl_hmg124h_3.JPG.dat	1367.3	68.7	1.0	0.05	#NUM!	3.0E-05
YDR003W	YDR003W	S288C_ctrl_hmg124h_3.JPG.dat	1407.8	20.7	1.0	0.01	#NUM!	4.1E-07
YDR004W	YDR004W	S288C_ctrl_hmg124h_3.JPG.dat	1236.5	65.6	0.9	0.05	#NUM!	4.6E-05
YDR005C	YDR005C	S288C_ctrl_hmg124h_3.JPG.dat	1418.5	17.0	1.1	0.01	#NUM!	1.6E-07
YDR006C	YDR006C	S288C_ctrl_hmg124h_3.JPG.dat	1229.0	30.6	1.0	0.02	#NUM!	2.7E-06
YDR007W	YDR007W	S288C_ctrl_hmg124h_3.JPG.dat	1202.0	38.8	1.0	0.01	#NUM!	2.2E-05
YDR008C	YDR008C	S288C_ctrl_hmg124h_3.JPG.dat	1276.8	73.7	1.0	0.05	#NUM!	4.2E-05
YDR009W	YDR009W	S288C_ctrl_hmg124h_3.JPG.dat	1211.5	19.6	1.0	0.02	#NUM!	1.5E-06

YDR010C	YDR010C	S288C_ctrl_hmg124h_3.JPG.dat	1256.8	31.3	1.0	0.01	#NUM!	2.8E-05
YDR011W	YDR011W	S288C_ctrl_hmg124h_3.JPG.dat	1323.8	15.9	1.0	0.01	#NUM!	4.2E-07
YDR014W	YDR014W	S288C_ctrl_hmg124h_3.JPG.dat	1334.3	32.2	1.0	0.03	#NUM!	4.8E-06
YDR015C	YDR015C	S288C_ctrl_hmg124h_3.JPG.dat	1397.3	48.2	1.0	0.03	#NUM!	9.8E-06
YDR018C	YDR018C	S288C_ctrl_hmg124h_3.JPG.dat	1459.5	32.7	1.0	0.02	#NUM!	2.2E-06
YDR019C	YDR019C	S288C_ctrl_hmg124h_3.JPG.dat	1384.5	30.2	1.0	0.02	#NUM!	1.9E-06
YDR020C	YDR020C	S288C_ctrl_hmg124h_3.JPG.dat	1447.3	52.0	1.0	0.04	#NUM!	1.5E-05
YDR022C	YDR022C	S288C_ctrl_hmg124h_3.JPG.dat	1330.5	22.3	1.0	0.01	#NUM!	2.9E-07
YDR024W	YDR024W	S288C_ctrl_hmg124h_3.JPG.dat	1279.8	27.2	1.0	0.02	#NUM!	4.3E-06
YDR025W	YDR025W	S288C_ctrl_hmg124h_3.JPG.dat	1269.5	26.8	1.0	0.02	#NUM!	1.3E-06
YDR048C	YDR048C	S288C_ctrl_hmg124h_3.JPG.dat	1278.3	52.9	1.0	0.04	#NUM!	1.6E-05
YDR049W	YDR049W	S288C_ctrl_hmg124h_3.JPG.dat	1320.8	33.2	1.0	0.03	#NUM!	5.6E-06
YDR051C	YDR051C	S288C_ctrl_hmg124h_3.JPG.dat	1384.3	47.6	1.0	0.03	#NUM!	9.3E-06
YDR055W	YDR055W	S288C_ctrl_hmg124h_3.JPG.dat	1322.8	37.1	1.0	0.01	#NUM!	1.2E-05
YDR056C	YDR056C	S288C_ctrl_hmg124h_3.JPG.dat	1360.8	91.6	1.0	0.07	#NUM!	7.7E-05
YDR057W	YDR057W	S288C_ctrl_hmg124h_3.JPG.dat	1569.3	34.8	1.0	0.03	#NUM!	1.0E-05
YDR058C	YDR058C	S288C_ctrl_hmg124h_3.JPG.dat	1485.0	68.7	1.0	0.03	#NUM!	8.7E-06
YDR059C	YDR059C	S288C_ctrl_hmg124h_3.JPG.dat	1652.0	35.1	1.1	0.02	#NUM!	8.8E-07
YDR061W	YDR061W	S288C_ctrl_hmg124h_3.JPG.dat	1534.0	33.3	1.0	0.02	#NUM!	2.1E-06
YDR063W	YDR063W	S288C_ctrl_hmg124h_3.JPG.dat	1522.5	84.8	1.1	0.05	#NUM!	2.1E-05
YDR066C	YDR066C	S288C_ctrl_hmg124h_3.JPG.dat	1439.0	99.4	1.0	0.05	#NUM!	4.1E-05
YDR067C	YDR067C	S288C_ctrl_hmg124h_3.JPG.dat	1422.3	63.1	1.0	0.01	#NUM!	1.1E-05
YDR068W	YDR068W	S288C_ctrl_hmg124h_3.JPG.dat	1454.0	32.1	1.0	0.02	#NUM!	3.1E-06
YDR070C	YDR070C	S288C_ctrl_hmg124h_3.JPG.dat	1443.5	33.5	1.0	0.04	#NUM!	1.3E-05
YDR071C	YDR071C	S288C_ctrl_hmg124h_3.JPG.dat	1467.3	25.8	1.0	0.00	#NUM!	2.7E-06
YDR072C	YDR072C	S288C_ctrl_hmg124h_3.JPG.dat	1398.3	81.7	0.9	0.00	#NUM!	2.5E-06
YDR073W	YDR073W	S288C_ctrl_hmg124h_3.JPG.dat	1401.0	84.6	1.0	0.05	#NUM!	3.9E-05
YDR074W	YDR074W	S288C_ctrl_hmg124h_3.JPG.dat	1069.0	22.7	0.8	0.01	#NUM!	1.7E-06
YDR075W	YDR075W	S288C_ctrl_hmg124h_3.JPG.dat	1423.3	20.8	1.0	0.02	#NUM!	1.3E-06
YDR076W	YDR076W	S288C_ctrl_hmg124h_3.JPG.dat	1208.8	36.8	0.9	0.03	#NUM!	1.2E-05
YDR077W	YDR077W	S288C_ctrl_hmg124h_3.JPG.dat	1296.5	3.3	1.0	0.01	#NUM!	1.7E-07
YDR080W	YDR080W	S288C_ctrl_hmg124h_3.JPG.dat	1145.8	105.5	0.9	0.07	#NUM!	1.7E-04
YDR083W	YDR083W	S288C_ctrl_hmg124h_3.JPG.dat	1097.3	131.5	0.8	0.02	#NUM!	2.2E-04
YDR084C	YDR084C	S288C_ctrl_hmg124h_3.JPG.dat	1314.5	10.8	1.0	0.01	#NUM!	2.4E-07
YDR085C	YDR085C	S288C_ctrl_hmg124h_3.JPG.dat	1368.8	35.1	1.0	0.02	#NUM!	2.0E-06
YDR089W	YDR089W	S288C_ctrl_hmg124h_3.JPG.dat	1351.5	42.8	1.0	0.01	#NUM!	3.1E-05
YDR090C	YDR090C	S288C_ctrl_hmg124h_3.JPG.dat	1298.8	52.6	1.0	0.04	#NUM!	1.7E-05
YDR092W	YDR092W	S288C_ctrl_hmg124h_3.JPG.dat	1397.5	15.3	1.0	0.00	#NUM!	2.6E-08
YDR093W	YDR093W	S288C_ctrl_hmg124h_3.JPG.dat	1413.3	33.3	1.0	0.02	#NUM!	2.6E-06
YDR094W	YDR094W	S288C_ctrl_hmg124h_3.JPG.dat	1374.0	29.9	1.0	0.00	#NUM!	7.9E-06
YDR095C	YDR095C	S288C_ctrl_hmg124h_3.JPG.dat	1349.0	26.9	1.0	0.02	#NUM!	2.8E-06
YDR096W	YDR096W	S288C_ctrl_hmg124h_3.JPG.dat	1401.0	20.6	1.1	0.01	#NUM!	1.0E-05
YDR097C	YDR097C	S288C_ctrl_hmg124h_3.JPG.dat	1275.5	10.7	1.0	0.01	#NUM!	2.1E-07
YDR098C	YDR098C	S288C_ctrl_hmg124h_3.JPG.dat	1296.3	54.6	1.0	0.04	#NUM!	2.3E-05
YDR099W	YDR099W	S288C_ctrl_hmg124h_3.JPG.dat	1284.5	24.2	1.0	0.01	#NUM!	1.8E-05
YDR100W	YDR100W	S288C_ctrl_hmg124h_3.JPG.dat	1350.8	16.0	1.0	0.01	#NUM!	4.7E-07
YDR101C	YDR101C	S288C_ctrl_hmg124h_3.JPG.dat	1204.8	36.4	0.9	0.03	#NUM!	7.3E-06
YDR102C	YDR102C	S288C_ctrl_hmg124h_3.JPG.dat	1350.0	57.4	1.0	0.01	#NUM!	1.1E-05
YDR103W	YDR103W	S288C_ctrl_hmg124h_3.JPG.dat	0.0	0.0	0.0	0.00	#NUM!	
YDR104C	YDR104C	S288C_ctrl_hmg124h_3.JPG.dat	1541.0	27.1	1.1	0.02	#NUM!	7.7E-07
YDR105C	YDR105C	S288C_ctrl_hmg124h_3.JPG.dat	1413.8	25.0	1.0	0.02	#NUM!	2.1E-06
YDR107C	YDR107C	S288C_ctrl_hmg124h_3.JPG.dat	1380.5	5.7	1.0	0.01	#NUM!	1.3E-07
YDR108W	YDR108W	S288C_ctrl_hmg124h_3.JPG.dat	1225.3	41.7	0.9	0.03	#NUM!	7.7E-06
YDR109C	YDR109C	S288C_ctrl_hmg124h_3.JPG.dat	1297.3	33.0	1.0	0.00	#NUM!	2.1E-06
YDR110W	YDR110W	S288C_ctrl_hmg124h_3.JPG.dat	1185.8	13.6	1.0	0.01	#NUM!	5.1E-07
YDR111C	YDR111C	S288C_ctrl_hmg124h_3.JPG.dat	1278.8	39.0	1.0	0.03	#NUM!	8.2E-06
YDR112W	YDR112W	S288C_ctrl_hmg124h_3.JPG.dat	1269.0	43.9	1.0	0.03	#NUM!	5.0E-06
YDR116C	YDR116C	S288C_ctrl_hmg124h_3.JPG.dat	1300.8	37.3	1.0	0.03	#NUM!	6.2E-06
YDR117C	YDR117C	S288C_ctrl_hmg124h_3.JPG.dat	1365.0	57.2	1.0	0.04	#NUM!	1.3E-05
YDR119W	YDR119W	S288C_ctrl_hmg124h_3.JPG.dat	1383.0	6.4	1.0	0.01	#NUM!	3.4E-07
YDR120C	YDR120C	S288C_ctrl_hmg124h_3.JPG.dat	1448.5	40.6	1.0	0.02	#NUM!	3.4E-06
YDR121W	YDR121W	S288C_ctrl_hmg124h_3.JPG.dat	1272.3	39.3	1.0	0.03	#NUM!	8.4E-06
YDR122W	YDR122W	S288C_ctrl_hmg124h_3.JPG.dat	1312.3	24.0	1.0	0.02	#NUM!	3.6E-06
YDR123C	YDR123C	S288C_ctrl_hmg124h_3.JPG.dat	1005.8	19.3	0.8	0.01	#NUM!	1.5E-06
YDR124W	YDR124W	S288C_ctrl_hmg124h_3.JPG.dat	1240.5	39.1	1.0	0.03	#NUM!	4.7E-06
YDR125C	YDR125C	S288C_ctrl_hmg124h_3.JPG.dat	1162.0	26.1	1.0	0.02	#NUM!	1.4E-06
YDR126W	YDR126W	S288C_ctrl_hmg124h_3.JPG.dat	1206.3	51.5	1.0	0.04	#NUM!	2.2E-05
YDR127W	YDR127W	S288C_ctrl_hmg124h_3.JPG.dat	1135.8	27.3	0.9	0.02	#NUM!	4.9E-06
YDR128W	YDR128W	S288C_ctrl_hmg124h_3.JPG.dat	1226.5	20.0	1.0	0.01	#NUM!	5.1E-07
YDR130C	YDR130C	S288C_ctrl_hmg124h_3.JPG.dat	1342.5	22.8	1.0	0.02	#NUM!	3.8E-06

YDR131C	YDR131C	S288C_ctrl_hmg124h_3.JPG.dat	1408.5	23.4	1.0	0.02	#NUM!	2.2E-06
YDR132C	YDR132C	S288C_ctrl_hmg124h_3.JPG.dat	1464.5	61.3	1.1	0.01	#NUM!	2.0E-05
YDR133C	YDR133C	S288C_ctrl_hmg124h_3.JPG.dat	1381.8	22.1	1.0	0.02	#NUM!	1.2E-06
YDR134C	YDR134C	S288C_ctrl_hmg124h_3.JPG.dat	1341.8	68.7	1.0	0.05	#NUM!	3.3E-05
YDR135C	YDR135C	S288C_ctrl_hmg124h_3.JPG.dat	1218.3	29.7	1.0	0.03	#NUM!	6.7E-06
YDR139C	YDR139C	S288C_ctrl_hmg124h_3.JPG.dat	1311.3	42.0	1.1	0.04	#NUM!	1.0E-05
YDR142C	YDR142C	S288C_ctrl_hmg124h_3.JPG.dat	1225.8	45.9	1.0	0.04	#NUM!	1.5E-05
YDR143C	YDR143C	S288C_ctrl_hmg124h_3.JPG.dat	1292.3	28.0	1.0	0.02	#NUM!	2.0E-06
YDR144C	YDR144C	S288C_ctrl_hmg124h_3.JPG.dat	1213.0	18.8	1.0	0.01	#NUM!	1.3E-05
YDR146C	YDR146C	S288C_ctrl_hmg124h_3.JPG.dat	1249.5	10.7	1.0	0.01	#NUM!	1.8E-07
YDR147W	YDR147W	S288C_ctrl_hmg124h_3.JPG.dat	1309.8	67.4	1.0	0.05	#NUM!	4.2E-05
YDR148C	YDR148C	S288C_ctrl_hmg124h_3.JPG.dat	1432.8	41.8	1.0	0.03	#NUM!	6.3E-06
YDR149C	YDR149C	S288C_ctrl_hmg124h_3.JPG.dat	1317.3	28.9	0.9	0.02	#NUM!	3.2E-06
YDR150W	YDR150W	S288C_ctrl_hmg124h_3.JPG.dat	1338.3	35.0	1.0	0.03	#NUM!	5.0E-06
YDR151C	YDR151C	S288C_ctrl_hmg124h_3.JPG.dat	1439.0	54.6	1.1	0.03	#NUM!	9.2E-06
YDR152W	YDR152W	S288C_ctrl_hmg124h_3.JPG.dat	1273.0	22.7	1.0	0.02	#NUM!	2.3E-06
YDR153C	YDR153C	S288C_ctrl_hmg124h_3.JPG.dat	1269.5	23.5	1.0	0.02	#NUM!	3.2E-06
YDR154C	YDR154C	S288C_ctrl_hmg124h_3.JPG.dat	1321.5	22.5	1.0	0.00	#NUM!	6.2E-06
YDR155C	YDR155C	S288C_ctrl_hmg124h_3.JPG.dat	1345.8	11.1	1.0	0.01	#NUM!	7.0E-07
YDR156W	YDR156W	S288C_ctrl_hmg124h_3.JPG.dat	1220.0	70.5	0.9	0.05	#NUM!	4.5E-05
YDR157W	YDR157W	S288C_ctrl_hmg124h_3.JPG.dat	1378.0	33.9	1.1	0.03	#NUM!	5.1E-06
YDR158W	YDR158W	S288C_ctrl_hmg124h_3.JPG.dat	1309.3	39.8	1.0	0.03	#NUM!	4.7E-06
YDR159W	YDR159W	S288C_ctrl_hmg124h_3.JPG.dat	322.3	372.1	0.2	0.24	#NUM!	1.8E-01
YDR161W	YDR161W	S288C_ctrl_hmg124h_3.JPG.dat	1455.8	48.1	0.9	0.01	#NUM!	9.8E-06
YDR162C	YDR162C	S288C_ctrl_hmg124h_3.JPG.dat	1438.3	128.9	1.0	0.08	#NUM!	1.8E-04
YDR163W	YDR163W	S288C_ctrl_hmg124h_3.JPG.dat	1453.3	82.2	1.0	0.07	#NUM!	1.1E-04
YDR165W	YDR165W	S288C_ctrl_hmg124h_3.JPG.dat	1438.0	45.6	1.0	0.01	#NUM!	7.8E-07
YDR169C	YDR169C	S288C_ctrl_hmg124h_3.JPG.dat	1482.3	48.4	1.0	0.01	#NUM!	6.1E-07
YDR171W	YDR171W	S288C_ctrl_hmg124h_3.JPG.dat	1461.3	47.0	1.0	0.03	#NUM!	8.2E-06
YDR173C	YDR173C	S288C_ctrl_hmg124h_3.JPG.dat	1489.0	54.7	1.0	0.03	#NUM!	8.0E-06
YDR174W	YDR174W	S288C_ctrl_hmg124h_3.JPG.dat	1377.3	44.4	1.0	0.00	#NUM!	5.0E-06
YDR178W	YDR178W	S288C_ctrl_hmg124h_3.JPG.dat	1350.3	66.6	1.0	0.02	#NUM!	1.2E-04
YDR179C	YDR179C	S288C_ctrl_hmg124h_3.JPG.dat	1411.0	43.3	1.0	0.04	#NUM!	1.5E-05
YDR179W-	YDR179W-A	S288C_ctrl_hmg124h_3.JPG.dat	1252.8	55.0	0.8	0.06	#NUM!	1.0E-04
YDR181C	YDR181C	S288C_ctrl_hmg124h_3.JPG.dat	1462.8	76.7	1.0	0.01	#NUM!	6.1E-05
YDR183W	YDR183W	S288C_ctrl_hmg124h_3.JPG.dat	1458.5	135.5	1.0	0.01	#NUM!	4.2E-05
YDR184C	YDR184C	S288C_ctrl_hmg124h_3.JPG.dat	1503.5	43.1	1.0	0.02	#NUM!	2.4E-06
YDR185C	YDR185C	S288C_ctrl_hmg124h_3.JPG.dat	1389.8	40.8	1.0	0.03	#NUM!	1.2E-05
YDR186C	YDR186C	S288C_ctrl_hmg124h_3.JPG.dat	1362.8	107.0	0.9	0.02	#NUM!	1.3E-04
YDR191W	YDR191W	S288C_ctrl_hmg124h_3.JPG.dat	1411.8	130.1	1.0	0.06	#NUM!	4.4E-05
YDR192C	YDR192C	S288C_ctrl_hmg124h_3.JPG.dat	1456.3	87.5	1.0	0.02	#NUM!	3.4E-06
YDR193W	YDR193W	S288C_ctrl_hmg124h_3.JPG.dat	1537.0	67.6	1.1	0.05	#NUM!	2.7E-05
YDR198C	YDR198C	S288C_ctrl_hmg124h_3.JPG.dat	1410.8	32.4	1.0	0.02	#NUM!	3.7E-06
YDR199W	YDR199W	S288C_ctrl_hmg124h_3.JPG.dat	1472.5	109.6	1.0	0.07	#NUM!	8.5E-05
YDR202C	YDR202C	S288C_ctrl_hmg124h_3.JPG.dat	1419.0	33.0	1.0	0.02	#NUM!	1.9E-06
YDR203W	YDR203W	S288C_ctrl_hmg124h_3.JPG.dat	1417.0	42.1	1.0	0.04	#NUM!	1.5E-05
YDR205W	YDR205W	S288C_ctrl_hmg124h_3.JPG.dat	1332.5	14.7	1.0	0.00	#NUM!	3.7E-09
YDR206W	YDR206W	S288C_ctrl_hmg124h_3.JPG.dat	1371.5	79.4	1.0	0.05	#NUM!	3.5E-05
YDR207C	YDR207C	S288C_ctrl_hmg124h_3.JPG.dat	1030.5	153.5	0.8	0.12	#NUM!	8.7E-04
YDR209C	YDR209C	S288C_ctrl_hmg124h_3.JPG.dat	1267.5	25.1	1.0	0.01	#NUM!	3.1E-05
YDR210W	YDR210W	S288C_ctrl_hmg124h_3.JPG.dat	1365.0	46.8	1.1	0.03	#NUM!	6.2E-06
YDR213W	YDR213W	S288C_ctrl_hmg124h_3.JPG.dat	1330.8	48.2	1.0	0.04	#NUM!	1.3E-05
YDR214W	YDR214W	S288C_ctrl_hmg124h_3.JPG.dat	1333.3	8.7	1.0	0.01	#NUM!	6.8E-07
YDR215C	YDR215C	S288C_ctrl_hmg124h_3.JPG.dat	1338.3	61.9	1.0	0.04	#NUM!	1.7E-05
YDR216W	YDR216W	S288C_ctrl_hmg124h_3.JPG.dat	1365.8	38.7	1.0	0.02	#NUM!	3.1E-06
YDR217C	YDR217C	S288C_ctrl_hmg124h_3.JPG.dat	1381.3	39.9	1.0	0.02	#NUM!	2.9E-06
YDR218C	YDR218C	S288C_ctrl_hmg124h_3.JPG.dat	1398.3	45.3	1.0	0.03	#NUM!	9.9E-06
YDR219C	YDR219C	S288C_ctrl_hmg124h_3.JPG.dat	1266.8	13.8	0.9	0.00	#NUM!	8.4E-06
YDR220C	YDR220C	S288C_ctrl_hmg124h_3.JPG.dat	1339.3	47.5	1.0	0.01	#NUM!	3.9E-05
YDR221W	YDR221W	S288C_ctrl_hmg124h_3.JPG.dat	1290.8	34.3	1.0	0.01	#NUM!	2.8E-05
YDR222W	YDR222W	S288C_ctrl_hmg124h_3.JPG.dat	1229.0	63.1	1.0	0.05	#NUM!	3.0E-05
YDR223W	YDR223W	S288C_ctrl_hmg124h_3.JPG.dat	1226.5	28.1	1.0	0.02	#NUM!	3.8E-06
YDR225W	YDR225W	S288C_ctrl_hmg124h_3.JPG.dat	1087.8	91.1	0.8	0.01	#NUM!	1.1E-04
YDR227W	YDR227W	S288C_ctrl_hmg124h_3.JPG.dat	0.0	0.0	0.0	0.00	#NUM!	
YDR229W	YDR229W	S288C_ctrl_hmg124h_3.JPG.dat	1322.0	37.7	1.0	0.02	#NUM!	2.5E-06
YDR233C	YDR233C	S288C_ctrl_hmg124h_3.JPG.dat	1423.5	111.7	1.0	0.08	#NUM!	1.2E-04
YDR234W	YDR234W	S288C_ctrl_hmg124h_3.JPG.dat	9.5	19.0	0.0	0.00	#NUM!	
YDR239C	YDR239C	S288C_ctrl_hmg124h_3.JPG.dat	1395.5	13.9	1.0	0.00	#NUM!	6.1E-07
YDR241W	YDR241W	S288C_ctrl_hmg124h_3.JPG.dat	1187.0	86.1	0.9	0.07	#NUM!	1.1E-04
YDR244W	YDR244W	S288C_ctrl_hmg124h_3.JPG.dat	1171.5	27.3	0.9	0.02	#NUM!	3.2E-06

YDR245W	YDR245W	S288C_ctrl_hmg124h_3.JPG.dat	1187.8	81.4	1.0	0.06	#NUM!	7.9E-05
YDR247W	YDR247W	S288C_ctrl_hmg124h_3.JPG.dat	1307.5	49.2	1.1	0.01	#NUM!	5.2E-05
YDR248C	YDR248C	S288C_ctrl_hmg124h_3.JPG.dat	1238.5	33.8	1.0	0.03	#NUM!	7.0E-06
YDR249C	YDR249C	S288C_ctrl_hmg124h_3.JPG.dat	1321.5	45.8	1.1	0.04	#NUM!	1.3E-05
YDR250C	YDR250C	S288C_ctrl_hmg124h_3.JPG.dat	1269.8	26.9	1.0	0.02	#NUM!	3.2E-06
YDR251W	YDR251W	S288C_ctrl_hmg124h_3.JPG.dat	1395.8	23.5	1.1	0.02	#NUM!	9.7E-07
YDR252W	YDR252W	S288C_ctrl_hmg124h_3.JPG.dat	1289.8	71.8	0.9	0.01	#NUM!	6.1E-05
YDR253C	YDR253C	S288C_ctrl_hmg124h_3.JPG.dat	1432.8	44.1	1.0	0.03	#NUM!	8.0E-06
YDR254W	YDR254W	S288C_ctrl_hmg124h_3.JPG.dat	1412.3	39.6	1.0	0.01	#NUM!	1.7E-05
YDR255C	YDR255C	S288C_ctrl_hmg124h_3.JPG.dat	1384.0	8.6	1.0	0.01	#NUM!	5.3E-07
YDR256C	YDR256C	S288C_ctrl_hmg124h_3.JPG.dat	1382.8	45.7	1.1	0.03	#NUM!	3.8E-06
YDR257C	YDR257C	S288C_ctrl_hmg124h_3.JPG.dat	1236.8	63.3	1.0	0.05	#NUM!	4.3E-05
YDR258C	YDR258C	S288C_ctrl_hmg124h_3.JPG.dat	1219.5	38.8	1.0	0.02	#NUM!	4.0E-06
YDR259C	YDR259C	S288C_ctrl_hmg124h_3.JPG.dat	1277.0	61.6	1.0	0.05	#NUM!	2.9E-05
YDR260C	YDR260C	S288C_ctrl_hmg124h_3.JPG.dat	1321.5	31.0	1.0	0.02	#NUM!	1.5E-06
YDR261C	YDR261C	S288C_ctrl_hmg124h_3.JPG.dat	1343.0	41.8	1.0	0.03	#NUM!	6.7E-06
YDR262W	YDR262W	S288C_ctrl_hmg124h_3.JPG.dat	1323.0	19.4	1.0	0.00	#NUM!	5.4E-06
YDR263C	YDR263C	S288C_ctrl_hmg124h_3.JPG.dat	1400.8	62.6	1.0	0.05	#NUM!	2.7E-05
YDR265W	YDR265W	S288C_ctrl_hmg124h_3.JPG.dat	1331.3	5.9	0.9	0.01	#NUM!	4.4E-07
YDR266C	YDR266C	S288C_ctrl_hmg124h_3.JPG.dat	1404.0	29.8	1.0	0.02	#NUM!	1.2E-06
YDR270W	YDR270W	S288C_ctrl_hmg124h_3.JPG.dat	1430.5	12.7	1.0	0.01	#NUM!	3.8E-07
YDR272W	YDR272W	S288C_ctrl_hmg124h_3.JPG.dat	1255.8	34.8	1.0	0.01	#NUM!	1.9E-05
YDR273W	YDR273W	S288C_ctrl_hmg124h_3.JPG.dat	1232.8	31.8	1.0	0.03	#NUM!	5.5E-06
YDR274C	YDR274C	S288C_ctrl_hmg124h_3.JPG.dat	1310.0	46.7	1.0	0.04	#NUM!	1.2E-05
YDR275W	YDR275W	S288C_ctrl_hmg124h_3.JPG.dat	1226.0	24.4	1.0	0.02	#NUM!	1.3E-06
YDR276C	YDR276C	S288C_ctrl_hmg124h_3.JPG.dat	1267.3	26.4	1.0	0.02	#NUM!	2.1E-06
YDR277C	YDR277C	S288C_ctrl_hmg124h_3.JPG.dat	1344.0	22.1	1.0	0.01	#NUM!	5.7E-07
YDR278C	YDR278C	S288C_ctrl_hmg124h_3.JPG.dat	1315.0	27.8	1.0	0.02	#NUM!	1.3E-06
YDR279W	YDR279W	S288C_ctrl_hmg124h_3.JPG.dat	1384.5	45.1	1.0	0.01	#NUM!	2.3E-05
YDR281C	YDR281C	S288C_ctrl_hmg124h_3.JPG.dat	1508.8	64.3	1.0	0.04	#NUM!	1.3E-05
YDR282C	YDR282C	S288C_ctrl_hmg124h_3.JPG.dat	1444.3	35.6	1.0	0.01	#NUM!	9.3E-07
YDR284C	YDR284C	S288C_ctrl_hmg124h_3.JPG.dat	1494.3	45.7	1.0	0.03	#NUM!	4.1E-06
YDR285W	YDR285W	S288C_ctrl_hmg124h_3.JPG.dat	1438.0	72.3	1.0	0.04	#NUM!	1.9E-05
YDR286C	YDR286C	S288C_ctrl_hmg124h_3.JPG.dat	1318.8	21.1	1.0	0.01	#NUM!	1.2E-05
YDR287W	YDR287W	S288C_ctrl_hmg124h_3.JPG.dat	1279.5	15.7	0.9	0.01	#NUM!	7.0E-07
YDR289C	YDR289C	S288C_ctrl_hmg124h_3.JPG.dat	1242.3	31.3	0.9	0.04	#NUM!	1.9E-05
YDR291W	YDR291W	S288C_ctrl_hmg124h_3.JPG.dat	1396.8	31.6	1.0	0.02	#NUM!	1.5E-06
YDR293C	YDR293C	S288C_ctrl_hmg124h_3.JPG.dat	1318.0	45.5	0.9	0.00	#NUM!	1.6E-06
YDR294C	YDR294C	S288C_ctrl_hmg124h_3.JPG.dat	1419.8	42.0	1.0	0.02	#NUM!	1.1E-06
YDR297W	YDR297W	S288C_ctrl_hmg124h_3.JPG.dat	1473.0	22.0	1.0	0.02	#NUM!	2.8E-06
YDR304C	YDR304C	S288C_ctrl_hmg124h_3.JPG.dat	1352.5	48.7	0.9	0.05	#NUM!	5.1E-05
YDR305C	YDR305C	S288C_ctrl_hmg124h_3.JPG.dat	1397.3	33.7	0.9	0.06	#NUM!	5.9E-05
YDR306C	YDR306C	S288C_ctrl_hmg124h_3.JPG.dat	1482.3	40.9	1.0	0.04	#NUM!	1.7E-05
YDR307W	YDR307W	S288C_ctrl_hmg124h_3.JPG.dat	1463.8	33.5	1.0	0.04	#NUM!	1.7E-05
YDR309C	YDR309C	S288C_ctrl_hmg124h_3.JPG.dat	1423.0	92.0	1.0	0.01	#NUM!	8.5E-06
YDR310C	YDR310C	S288C_ctrl_hmg124h_3.JPG.dat	1419.0	58.9	1.0	0.01	#NUM!	3.7E-08
YDR312W	YDR312W	S288C_ctrl_hmg124h_3.JPG.dat	1466.5	55.5	1.1	0.00	#NUM!	6.6E-07
YDR313C	YDR313C	S288C_ctrl_hmg124h_3.JPG.dat	1526.3	132.1	1.1	0.05	#NUM!	3.3E-05
YDR314C	YDR314C	S288C_ctrl_hmg124h_3.JPG.dat	1424.0	31.5	1.0	0.02	#NUM!	2.2E-06
YDR315C	YDR315C	S288C_ctrl_hmg124h_3.JPG.dat	1203.3	95.1	0.9	0.06	#NUM!	1.1E-04
YDR316W	YDR316W	S288C_ctrl_hmg124h_3.JPG.dat	1375.3	50.0	1.0	0.02	#NUM!	1.8E-06
YDR317W	YDR317W	S288C_ctrl_hmg124h_3.JPG.dat	1349.8	62.8	1.0	0.04	#NUM!	1.6E-05
YDR318W	YDR318W	S288C_ctrl_hmg124h_3.JPG.dat	1378.5	72.9	1.0	0.05	#NUM!	3.2E-05
YDR319C	YDR319C	S288C_ctrl_hmg124h_3.JPG.dat	1323.3	47.6	1.0	0.04	#NUM!	2.3E-05
YDR320C	YDR320C	S288C_ctrl_hmg124h_3.JPG.dat	1279.8	41.7	1.0	0.03	#NUM!	7.0E-06
YDR321W	YDR321W	S288C_ctrl_hmg124h_3.JPG.dat	1272.3	9.3	1.0	0.02	#NUM!	1.1E-06
YDR329C	YDR329C	S288C_ctrl_hmg124h_3.JPG.dat	1216.0	37.7	0.9	0.04	#NUM!	2.0E-05
YDR330W	YDR330W	S288C_ctrl_hmg124h_3.JPG.dat	1311.8	25.5	1.0	0.02	#NUM!	1.3E-06
YDR332W	YDR332W	S288C_ctrl_hmg124h_3.JPG.dat	347.3	694.5	0.0	0.00	#NUM!	
YDR333C	YDR333C	S288C_ctrl_hmg124h_3.JPG.dat	1374.8	18.2	1.0	0.03	#NUM!	3.8E-06
YDR334W	YDR334W	S288C_ctrl_hmg124h_3.JPG.dat	1230.0	37.1	0.9	0.03	#NUM!	1.2E-05
YDR335W	YDR335W	S288C_ctrl_hmg124h_3.JPG.dat	1408.3	19.7	1.1	0.01	#NUM!	1.2E-07
YDR336W	YDR336W	S288C_ctrl_hmg124h_3.JPG.dat	1440.3	112.1	1.0	0.08	#NUM!	1.4E-04
YDR338C	YDR338C	S288C_ctrl_hmg124h_3.JPG.dat	1378.3	109.2	1.0	0.02	#NUM!	1.5E-04
YDR340W	YDR340W	S288C_ctrl_hmg124h_3.JPG.dat	1327.8	4.3	1.0	0.01	#NUM!	1.8E-07
YDR344C	YDR344C	S288C_ctrl_hmg124h_3.JPG.dat	1333.5	46.7	1.0	0.04	#NUM!	1.3E-05
YDR345C	YDR345C	S288C_ctrl_hmg124h_3.JPG.dat	1285.5	17.9	1.0	0.00	#NUM!	3.7E-06
YDR346C	YDR346C	S288C_ctrl_hmg124h_3.JPG.dat	1262.5	33.7	1.0	0.01	#NUM!	2.3E-05
YDR348C	YDR348C	S288C_ctrl_hmg124h_3.JPG.dat	1204.3	27.4	1.0	0.02	#NUM!	4.5E-06
YDR349C	YDR349C	S288C_ctrl_hmg124h_3.JPG.dat	1245.0	20.1	1.0	0.00	#NUM!	2.5E-06

YDR351W	YDR351W	S288C_ctrl_hmg124h_3.JPG.dat	1368.8	75.2	1.1	0.06	#NUM!	4.4E-05
YDR352W	YDR352W	S288C_ctrl_hmg124h_3.JPG.dat	1310.5	40.6	1.0	0.01	#NUM!	2.4E-05
YDR354W	YDR354W	S288C_ctrl_hmg124h_3.JPG.dat	1341.8	48.1	1.0	0.03	#NUM!	7.0E-06
YDR357C	YDR357C	S288C_ctrl_hmg124h_3.JPG.dat	1483.8	55.3	1.1	0.04	#NUM!	1.2E-05
YDR358W	YDR358W	S288C_ctrl_hmg124h_3.JPG.dat	1449.8	55.6	1.0	0.04	#NUM!	1.1E-05
YDR359C	YDR359C	S288C_ctrl_hmg124h_3.JPG.dat	1238.0	322.3	0.9	0.24	#NUM!	4.4E-03
YDR360W	YDR360W	S288C_ctrl_hmg124h_3.JPG.dat	1209.0	28.9	0.9	0.02	#NUM!	3.8E-06
YDR363W	YDR363W	S288C_ctrl_hmg124h_3.JPG.dat	1108.5	31.8	0.9	0.02	#NUM!	4.8E-06
YDR363W-	YDR363W-A	S288C_ctrl_hmg124h_3.JPG.dat	1203.8	18.9	1.0	0.02	#NUM!	1.1E-06
YDR368W	YDR368W	S288C_ctrl_hmg124h_3.JPG.dat	1171.0	38.3	1.0	0.03	#NUM!	9.9E-06
YDR369C	YDR369C	S288C_ctrl_hmg124h_3.JPG.dat	1034.3	123.4	0.8	0.03	#NUM!	4.6E-04
YDR370C	YDR370C	S288C_ctrl_hmg124h_3.JPG.dat	1250.3	12.1	1.0	0.01	#NUM!	2.2E-07
YDR371W	YDR371W	S288C_ctrl_hmg124h_3.JPG.dat	1333.3	22.0	1.1	0.02	#NUM!	1.4E-06
YDR372C	YDR372C	S288C_ctrl_hmg124h_3.JPG.dat	1298.8	13.4	1.0	0.00	#NUM!	4.6E-06
YDR374C	YDR374C	S288C_ctrl_hmg124h_3.JPG.dat	1457.0	76.1	1.0	0.05	#NUM!	2.7E-05
YDR375C	YDR375C	S288C_ctrl_hmg124h_3.JPG.dat	365.8	731.5	0.0	0.00	#NUM!	
YDR378C	YDR378C	S288C_ctrl_hmg124h_3.JPG.dat	1067.3	102.4	0.8	0.03	#NUM!	3.4E-04
YDR379W	YDR379W	S288C_ctrl_hmg124h_3.JPG.dat	1324.3	30.3	1.0	0.02	#NUM!	2.4E-06
YDR380W	YDR380W	S288C_ctrl_hmg124h_3.JPG.dat	1290.5	33.1	1.0	0.01	#NUM!	1.6E-05
YDR382W	YDR382W	S288C_ctrl_hmg124h_3.JPG.dat	959.5	162.0	0.8	0.13	#NUM!	1.3E-03
YDR383C	YDR383C	S288C_ctrl_hmg124h_3.JPG.dat	1171.3	17.1	1.0	0.00	#NUM!	1.2E-06
YDR384C	YDR384C	S288C_ctrl_hmg124h_3.JPG.dat	1210.8	15.0	1.0	0.01	#NUM!	4.9E-07
YDR385W	YDR385W	S288C_ctrl_hmg124h_3.JPG.dat	1205.5	24.2	0.9	0.02	#NUM!	1.4E-06
YDR386W	YDR386W	S288C_ctrl_hmg124h_3.JPG.dat	1237.8	19.5	1.0	0.01	#NUM!	5.0E-07
YDR387C	YDR387C	S288C_ctrl_hmg124h_3.JPG.dat	1380.0	44.0	1.0	0.04	#NUM!	1.2E-05
YDR388W	YDR388W	S288C_ctrl_hmg124h_3.JPG.dat	1474.0	22.2	1.0	0.02	#NUM!	8.2E-07
YDR389W	YDR389W	S288C_ctrl_hmg124h_3.JPG.dat	1371.8	15.6	1.0	0.01	#NUM!	1.1E-07
YDR391C	YDR391C	S288C_ctrl_hmg124h_3.JPG.dat	1385.5	23.3	1.0	0.02	#NUM!	9.7E-07
YDR392W	YDR392W	S288C_ctrl_hmg124h_3.JPG.dat	1184.0	158.9	0.9	0.12	#NUM!	6.2E-04
YDR393W	YDR393W	S288C_ctrl_hmg124h_3.JPG.dat	1075.3	63.5	0.9	0.01	#NUM!	2.3E-05
YDR395W	YDR395W	S288C_ctrl_hmg124h_3.JPG.dat	1249.3	20.7	1.0	0.02	#NUM!	1.2E-06
YDR399W	YDR399W	S288C_ctrl_hmg124h_3.JPG.dat	1222.8	29.0	1.0	0.01	#NUM!	2.0E-05
YDR400W	YDR400W	S288C_ctrl_hmg124h_3.JPG.dat	1311.8	60.2	1.0	0.01	#NUM!	2.7E-05
YDR401W	YDR401W	S288C_ctrl_hmg124h_3.JPG.dat	1309.0	40.5	1.0	0.04	#NUM!	1.5E-05
YDR402C	YDR402C	S288C_ctrl_hmg124h_3.JPG.dat	1332.3	14.3	1.0	0.01	#NUM!	1.3E-07
YDR403W	YDR403W	S288C_ctrl_hmg124h_3.JPG.dat	1402.5	54.4	1.1	0.04	#NUM!	1.2E-05
YDR406W	YDR406W	S288C_ctrl_hmg124h_3.JPG.dat	1527.5	23.5	1.0	0.03	#NUM!	4.2E-06
YDR408C	YDR408C	S288C_ctrl_hmg124h_3.JPG.dat	1448.0	44.4	1.0	0.02	#NUM!	1.3E-06
YDR409W	YDR409W	S288C_ctrl_hmg124h_3.JPG.dat	1471.8	71.8	1.0	0.01	#NUM!	6.8E-05
YDR410C	YDR410C	S288C_ctrl_hmg124h_3.JPG.dat	0.0	0.0	0.0	0.00	#NUM!	
YDR411C	YDR411C	S288C_ctrl_hmg124h_3.JPG.dat	1364.8	34.4	1.0	0.02	#NUM!	3.3E-06
YDR414C	YDR414C	S288C_ctrl_hmg124h_3.JPG.dat	1423.3	47.5	1.0	0.02	#NUM!	1.0E-06
YDR415C	YDR415C	S288C_ctrl_hmg124h_3.JPG.dat	1302.3	59.1	1.0	0.04	#NUM!	2.2E-05
YDR419W	YDR419W	S288C_ctrl_hmg124h_3.JPG.dat	1356.8	48.0	1.0	0.03	#NUM!	5.9E-06
YDR420W	YDR420W	S288C_ctrl_hmg124h_3.JPG.dat	1404.3	37.9	1.0	0.02	#NUM!	1.7E-06
YDR421W	YDR421W	S288C_ctrl_hmg124h_3.JPG.dat	1371.8	44.6	1.0	0.01	#NUM!	2.1E-05
YDR422C	YDR422C	S288C_ctrl_hmg124h_3.JPG.dat	1389.0	20.2	1.0	0.01	#NUM!	6.7E-07
YOR202W	YOR202W	S288C_ctrl_hmg124h_3.JPG.dat	1448.7	150.1	1.0	0.05	#NUM!	0.0E+00
1	1	S288C_ctrl_hmg124h_4.JPG.dat	1649.8	143.8	1.1	0.06	#NUM!	3.4E-05
2	2	S288C_ctrl_hmg124h_4.JPG.dat	1400.5	107.7	1.0	0.05	#NUM!	4.8E-05
3	3	S288C_ctrl_hmg124h_4.JPG.dat	1393.0	67.2	1.0	0.05	#NUM!	2.6E-05
4	4	S288C_ctrl_hmg124h_4.JPG.dat	1406.3	120.6	1.0	0.09	#NUM!	1.8E-04
YDR423C	YDR423C	S288C_ctrl_hmg124h_4.JPG.dat	1471.3	45.4	1.0	0.03	#NUM!	8.4E-06
YDR424C	YDR424C	S288C_ctrl_hmg124h_4.JPG.dat	1379.8	37.9	1.0	0.03	#NUM!	8.2E-06
YDR425W	YDR425W	S288C_ctrl_hmg124h_4.JPG.dat	1369.3	26.5	1.0	0.00	#NUM!	1.9E-06
YDR426C	YDR426C	S288C_ctrl_hmg124h_4.JPG.dat	1314.0	30.0	0.9	0.01	#NUM!	3.1E-05
YDR428C	YDR428C	S288C_ctrl_hmg124h_4.JPG.dat	1382.0	19.0	1.0	0.00	#NUM!	6.1E-07
YDR430C	YDR430C	S288C_ctrl_hmg124h_4.JPG.dat	1340.3	22.1	1.0	0.01	#NUM!	2.6E-07
YDR431W	YDR431W	S288C_ctrl_hmg124h_4.JPG.dat	1369.3	47.4	1.0	0.03	#NUM!	7.4E-06
YDR435C	YDR435C	S288C_ctrl_hmg124h_4.JPG.dat	1433.5	30.6	1.0	0.00	#NUM!	5.7E-06
YDR436W	YDR436W	S288C_ctrl_hmg124h_4.JPG.dat	1416.8	48.4	1.0	0.02	#NUM!	3.7E-06
YDR438W	YDR438W	S288C_ctrl_hmg124h_4.JPG.dat	1369.5	23.7	1.0	0.02	#NUM!	4.3E-06
YDR439W	YDR439W	S288C_ctrl_hmg124h_4.JPG.dat	1284.8	162.6	0.9	0.13	#NUM!	7.3E-04
YDR440W	YDR440W	S288C_ctrl_hmg124h_4.JPG.dat	1511.8	19.2	1.1	0.02	#NUM!	1.2E-06
YDR441C	YDR441C	S288C_ctrl_hmg124h_4.JPG.dat	1402.3	78.9	1.0	0.06	#NUM!	4.8E-05
YDR445C	YDR445C	S288C_ctrl_hmg124h_4.JPG.dat	1402.0	26.8	1.0	0.00	#NUM!	6.0E-06
YDR446W	YDR446W	S288C_ctrl_hmg124h_4.JPG.dat	1395.0	50.6	1.0	0.04	#NUM!	1.2E-05
YDR447C	YDR447C	S288C_ctrl_hmg124h_4.JPG.dat	1194.0	29.2	0.9	0.02	#NUM!	4.8E-06
YDR451C	YDR451C	S288C_ctrl_hmg124h_4.JPG.dat	1322.8	80.1	1.0	0.06	#NUM!	6.7E-05
YDR452W	YDR452W	S288C_ctrl_hmg124h_4.JPG.dat	1325.3	28.6	1.0	0.02	#NUM!	2.2E-06

YDR453C	YDR453C	S288C_ctrl_hmg124h_4.JPG.dat	1350.3	15.5	1.0	0.01	#NUM!	4.8E-07
YDR455C	YDR455C	S288C_ctrl_hmg124h_4.JPG.dat	1156.8	75.4	0.9	0.06	#NUM!	9.2E-05
YDR458C	YDR458C	S288C_ctrl_hmg124h_4.JPG.dat	1442.8	46.3	1.0	0.03	#NUM!	5.4E-06
YDR459C	YDR459C	S288C_ctrl_hmg124h_4.JPG.dat	1459.5	89.6	1.1	0.07	#NUM!	7.1E-05
YDR463W	YDR463W	S288C_ctrl_hmg124h_4.JPG.dat	1343.0	29.9	1.0	0.01	#NUM!	1.5E-05
YDR465C	YDR465C	S288C_ctrl_hmg124h_4.JPG.dat	1396.0	33.7	1.0	0.02	#NUM!	2.8E-06
YDR466W	YDR466W	S288C_ctrl_hmg124h_4.JPG.dat	1478.0	37.0	1.1	0.03	#NUM!	6.7E-06
YDR467C	YDR467C	S288C_ctrl_hmg124h_4.JPG.dat	1394.3	14.5	1.0	0.02	#NUM!	1.2E-06
YDR469W	YDR469W	S288C_ctrl_hmg124h_4.JPG.dat	1267.3	83.6	1.0	0.06	#NUM!	7.0E-05
YDR471W	YDR471W	S288C_ctrl_hmg124h_4.JPG.dat	1177.5	60.0	0.9	0.05	#NUM!	4.6E-05
YDR474C	YDR474C	S288C_ctrl_hmg124h_4.JPG.dat	1280.5	27.7	1.0	0.01	#NUM!	1.4E-05
YDR475C	YDR475C	S288C_ctrl_hmg124h_4.JPG.dat	1293.3	22.8	1.0	0.02	#NUM!	1.1E-06
YDR476C	YDR476C	S288C_ctrl_hmg124h_4.JPG.dat	1350.3	37.7	1.0	0.02	#NUM!	4.0E-06
YDR479C	YDR479C	S288C_ctrl_hmg124h_4.JPG.dat	1401.3	30.0	1.0	0.02	#NUM!	2.5E-06
YDR480W	YDR480W	S288C_ctrl_hmg124h_4.JPG.dat	1429.5	63.7	1.0	0.04	#NUM!	1.1E-05
YDR481C	YDR481C	S288C_ctrl_hmg124h_4.JPG.dat	1465.5	33.8	1.0	0.00	#NUM!	7.4E-06
YDR482C	YDR482C	S288C_ctrl_hmg124h_4.JPG.dat	1401.8	21.9	1.0	0.02	#NUM!	1.8E-06
YDR483W	YDR483W	S288C_ctrl_hmg124h_4.JPG.dat	1355.5	33.9	1.0	0.03	#NUM!	6.6E-06
YDR485C	YDR485C	S288C_ctrl_hmg124h_4.JPG.dat	1208.5	33.9	0.9	0.03	#NUM!	8.0E-06
YDR486C	YDR486C	S288C_ctrl_hmg124h_4.JPG.dat	1357.8	29.4	1.0	0.01	#NUM!	1.7E-05
YDR488C	YDR488C	S288C_ctrl_hmg124h_4.JPG.dat	1341.8	22.0	1.0	0.01	#NUM!	8.1E-06
YDR490C	YDR490C	S288C_ctrl_hmg124h_4.JPG.dat	1290.5	109.0	1.0	0.08	#NUM!	1.7E-04
YDR491C	YDR491C	S288C_ctrl_hmg124h_4.JPG.dat	1365.3	29.7	1.0	0.02	#NUM!	3.0E-06
YDR492W	YDR492W	S288C_ctrl_hmg124h_4.JPG.dat	1456.3	48.3	1.1	0.04	#NUM!	9.8E-06
YDR494W	YDR494W	S288C_ctrl_hmg124h_4.JPG.dat	1348.0	153.7	1.0	0.11	#NUM!	3.8E-04
YDR496C	YDR496C	S288C_ctrl_hmg124h_4.JPG.dat	1174.5	93.9	0.9	0.08	#NUM!	1.9E-04
YDR497C	YDR497C	S288C_ctrl_hmg124h_4.JPG.dat	1482.8	47.9	1.0	0.03	#NUM!	9.0E-06
YDR500C	YDR500C	S288C_ctrl_hmg124h_4.JPG.dat	1245.0	176.1	0.8	0.02	#NUM!	2.0E-04
YDR501W	YDR501W	S288C_ctrl_hmg124h_4.JPG.dat	1377.8	21.4	1.0	0.02	#NUM!	1.1E-06
YDR503C	YDR503C	S288C_ctrl_hmg124h_4.JPG.dat	1336.5	36.6	1.0	0.03	#NUM!	7.2E-06
YDR504C	YDR504C	S288C_ctrl_hmg124h_4.JPG.dat	1328.8	23.7	1.0	0.00	#NUM!	7.8E-06
YDR505C	YDR505C	S288C_ctrl_hmg124h_4.JPG.dat	1324.0	38.8	1.0	0.03	#NUM!	6.2E-06
YDR506C	YDR506C	S288C_ctrl_hmg124h_4.JPG.dat	1288.8	21.5	1.0	0.02	#NUM!	1.3E-06
YDR508C	YDR508C	S288C_ctrl_hmg124h_4.JPG.dat	1246.0	28.0	1.0	0.02	#NUM!	2.8E-06
YDR509W	YDR509W	S288C_ctrl_hmg124h_4.JPG.dat	1241.3	31.3	0.9	0.02	#NUM!	1.6E-06
YDR511W	YDR511W	S288C_ctrl_hmg124h_4.JPG.dat	1368.0	15.8	1.0	0.02	#NUM!	2.0E-06
YDR512C	YDR512C	S288C_ctrl_hmg124h_4.JPG.dat	1216.3	284.5	0.9	0.20	#NUM!	3.0E-03
YDR513W	YDR513W	S288C_ctrl_hmg124h_4.JPG.dat	1397.0	31.0	1.0	0.02	#NUM!	3.4E-06
YDR514C	YDR514C	S288C_ctrl_hmg124h_4.JPG.dat	1401.0	13.5	1.0	0.01	#NUM!	1.5E-07
YDR516C	YDR516C	S288C_ctrl_hmg124h_4.JPG.dat	1445.8	31.6	1.0	0.02	#NUM!	3.8E-06
YDR517W	YDR517W	S288C_ctrl_hmg124h_4.JPG.dat	1389.3	24.5	1.0	0.00	#NUM!	3.4E-06
YDR519W	YDR519W	S288C_ctrl_hmg124h_4.JPG.dat	1331.3	22.3	1.0	0.01	#NUM!	1.6E-05
YDR520C	YDR520C	S288C_ctrl_hmg124h_4.JPG.dat	1320.0	5.9	1.0	0.01	#NUM!	3.7E-08
YDR522C	YDR522C	S288C_ctrl_hmg124h_4.JPG.dat	1300.8	33.4	1.0	0.03	#NUM!	5.3E-06
YDR524C	YDR524C	S288C_ctrl_hmg124h_4.JPG.dat	1335.3	27.8	1.0	0.02	#NUM!	2.4E-06
YDR525W	YDR525W	S288C_ctrl_hmg124h_4.JPG.dat	1336.0	21.7	1.0	0.02	#NUM!	2.2E-06
YDR525W-	YDR525W-A	S288C_ctrl_hmg124h_4.JPG.dat	1280.5	64.7	0.9	0.05	#NUM!	4.5E-05
YDR528W	YDR528W	S288C_ctrl_hmg124h_4.JPG.dat	1368.3	101.5	1.1	0.10	#NUM!	2.3E-04
YDR530C	YDR530C	S288C_ctrl_hmg124h_4.JPG.dat	1568.0	70.3	1.0	0.04	#NUM!	2.1E-05
YDR532C	YDR532C	S288C_ctrl_hmg124h_4.JPG.dat	1546.3	90.9	1.0	0.06	#NUM!	4.8E-05
YDR533C	YDR533C	S288C_ctrl_hmg124h_4.JPG.dat	1533.3	47.2	1.0	0.03	#NUM!	5.4E-06
YDR534C	YDR534C	S288C_ctrl_hmg124h_4.JPG.dat	1518.3	23.3	1.0	0.02	#NUM!	1.2E-06
YDR535C	YDR535C	S288C_ctrl_hmg124h_4.JPG.dat	1482.8	28.5	1.0	0.02	#NUM!	1.0E-06
YDR536W	YDR536W	S288C_ctrl_hmg124h_4.JPG.dat	1442.3	23.1	1.0	0.02	#NUM!	1.3E-06
YDR537C	YDR537C	S288C_ctrl_hmg124h_4.JPG.dat	1359.0	31.5	0.9	0.02	#NUM!	3.2E-06
YDR538W	YDR538W	S288C_ctrl_hmg124h_4.JPG.dat	1398.5	48.8	1.0	0.04	#NUM!	1.7E-05
YDR539W	YDR539W	S288C_ctrl_hmg124h_4.JPG.dat	1440.8	33.8	1.0	0.01	#NUM!	8.3E-06
YDR540C	YDR540C	S288C_ctrl_hmg124h_4.JPG.dat	1451.5	39.3	1.0	0.05	#NUM!	3.3E-05
YDR541C	YDR541C	S288C_ctrl_hmg124h_4.JPG.dat	677.0	795.1	0.6	0.74	#NUM!	1.8E-01
YEL001C	YEL001C	S288C_ctrl_hmg124h_4.JPG.dat	1394.5	31.9	1.0	0.02	#NUM!	3.3E-06
YEL003W	YEL003W	S288C_ctrl_hmg124h_4.JPG.dat	1322.8	72.2	1.0	0.05	#NUM!	3.5E-05
YEL004W	YEL004W	S288C_ctrl_hmg124h_4.JPG.dat	1309.5	92.7	0.9	0.06	#NUM!	8.9E-05
YEL005C	YEL005C	S288C_ctrl_hmg124h_4.JPG.dat	1370.8	8.7	1.0	0.01	#NUM!	1.3E-07
YEL006W	YEL006W	S288C_ctrl_hmg124h_4.JPG.dat	1410.5	24.6	1.0	0.00	#NUM!	5.3E-07
YEL007W	YEL007W	S288C_ctrl_hmg124h_4.JPG.dat	1342.3	52.8	1.0	0.04	#NUM!	1.5E-05
YEL008W	YEL008W	S288C_ctrl_hmg124h_4.JPG.dat	1371.5	40.1	1.0	0.03	#NUM!	8.1E-06
YEL010W	YEL010W	S288C_ctrl_hmg124h_4.JPG.dat	1405.8	88.3	1.0	0.06	#NUM!	4.4E-05
YEL011W	YEL011W	S288C_ctrl_hmg124h_4.JPG.dat	1385.8	34.4	1.0	0.02	#NUM!	2.5E-06
YEL012W	YEL012W	S288C_ctrl_hmg124h_4.JPG.dat	1384.5	20.2	1.0	0.02	#NUM!	1.0E-06
YEL013W	YEL013W	S288C_ctrl_hmg124h_4.JPG.dat	822.3	251.0	0.6	0.18	#NUM!	7.7E-03

YEL014C	YEL014C	S288C_ctrl_hmg124h_4.JPG.dat	1326.5	105.6	0.9	0.03	#NUM!	2.8E-04
YEL015W	YEL015W	S288C_ctrl_hmg124h_4.JPG.dat	1415.0	62.2	1.0	0.04	#NUM!	2.2E-05
YEL016C	YEL016C	S288C_ctrl_hmg124h_4.JPG.dat	1438.8	44.8	1.0	0.01	#NUM!	4.6E-05
YEL017C-A	YEL017C-A	S288C_ctrl_hmg124h_4.JPG.dat	1388.8	25.2	1.0	0.01	#NUM!	7.1E-07
YEL017W	YEL017W	S288C_ctrl_hmg124h_4.JPG.dat	1371.8	15.0	1.0	0.01	#NUM!	2.0E-07
YEL020C	YEL020C	S288C_ctrl_hmg124h_4.JPG.dat	1341.8	43.7	1.0	0.03	#NUM!	8.1E-06
YEL023C	YEL023C	S288C_ctrl_hmg124h_4.JPG.dat	1355.8	18.2	1.0	0.01	#NUM!	3.9E-07
YEL025C	YEL025C	S288C_ctrl_hmg124h_4.JPG.dat	1282.0	19.1	1.0	0.01	#NUM!	6.3E-07
YEL028W	YEL028W	S288C_ctrl_hmg124h_4.JPG.dat	1390.5	7.6	1.0	0.01	#NUM!	7.8E-08
YEL030W	YEL030W	S288C_ctrl_hmg124h_4.JPG.dat	1385.8	33.1	1.0	0.03	#NUM!	4.5E-06
YEL031W	YEL031W	S288C_ctrl_hmg124h_4.JPG.dat	11.0	22.0	0.0	0.00	#NUM!	
YEL033W	YEL033W	S288C_ctrl_hmg124h_4.JPG.dat	1341.0	81.3	0.9	0.06	#NUM!	6.0E-05
YEL037C	YEL037C	S288C_ctrl_hmg124h_4.JPG.dat	1404.8	40.2	1.0	0.01	#NUM!	1.2E-05
YEL038W	YEL038W	S288C_ctrl_hmg124h_4.JPG.dat	1364.0	20.2	1.0	0.01	#NUM!	7.3E-07
YEL039C	YEL039C	S288C_ctrl_hmg124h_4.JPG.dat	1387.8	28.8	1.0	0.02	#NUM!	1.6E-06
YEL040W	YEL040W	S288C_ctrl_hmg124h_4.JPG.dat	1333.8	19.8	1.0	0.02	#NUM!	1.9E-06
YEL041W	YEL041W	S288C_ctrl_hmg124h_4.JPG.dat	1279.3	23.9	1.0	0.02	#NUM!	1.6E-06
YEL042W	YEL042W	S288C_ctrl_hmg124h_4.JPG.dat	1211.5	108.6	0.9	0.09	#NUM!	2.2E-04
YEL043W	YEL043W	S288C_ctrl_hmg124h_4.JPG.dat	1240.5	61.4	1.0	0.05	#NUM!	3.7E-05
YEL047C	YEL047C	S288C_ctrl_hmg124h_4.JPG.dat	1345.3	23.6	1.0	0.02	#NUM!	1.2E-06
YEL048C	YEL048C	S288C_ctrl_hmg124h_4.JPG.dat	1349.8	27.9	1.0	0.02	#NUM!	3.0E-06
YEL049W	YEL049W	S288C_ctrl_hmg124h_4.JPG.dat	1302.0	160.2	0.9	0.11	#NUM!	4.9E-04
YEL052W	YEL052W	S288C_ctrl_hmg124h_4.JPG.dat	1477.5	59.8	1.0	0.04	#NUM!	1.5E-05
YEL053C	YEL053C	S288C_ctrl_hmg124h_4.JPG.dat	1473.3	71.2	1.0	0.01	#NUM!	9.6E-06
YEL056W	YEL056W	S288C_ctrl_hmg124h_4.JPG.dat	1387.0	55.5	1.0	0.04	#NUM!	1.3E-05
YEL057C	YEL057C	S288C_ctrl_hmg124h_4.JPG.dat	1014.3	676.3	1.0	0.02	#NUM!	1.2E-04
YEL059W	YEL059W	S288C_ctrl_hmg124h_4.JPG.dat	897.0	600.5	0.9	0.05	#NUM!	1.0E-03
YEL060C	YEL060C	S288C_ctrl_hmg124h_4.JPG.dat	455.8	683.7	0.1	0.17	#NUM!	4.2E-01
YEL061C	YEL061C	S288C_ctrl_hmg124h_4.JPG.dat	432.5	865.0	0.0	0.00	#NUM!	
YEL062W	YEL062W	S288C_ctrl_hmg124h_4.JPG.dat	583.0	674.1	0.4	0.51	#NUM!	1.8E-01
YEL063C	YEL063C	S288C_ctrl_hmg124h_4.JPG.dat	0.0	0.0	0.0	0.00	#NUM!	
YEL064C	YEL064C	S288C_ctrl_hmg124h_4.JPG.dat	0.0	0.0	0.0	0.00	#NUM!	
YEL065W	YEL065W	S288C_ctrl_hmg124h_4.JPG.dat	1137.0	758.8	1.1	0.03	#NUM!	2.0E-04
YEL066W	YEL066W	S288C_ctrl_hmg124h_4.JPG.dat	1443.0	22.1	1.0	0.01	#NUM!	6.7E-07
YEL067C	YEL067C	S288C_ctrl_hmg124h_4.JPG.dat	1497.8	64.4	1.1	0.04	#NUM!	1.7E-05
YEL068C	YEL068C	S288C_ctrl_hmg124h_4.JPG.dat	1389.5	36.9	1.0	0.02	#NUM!	3.9E-06
YEL071W	YEL071W	S288C_ctrl_hmg124h_4.JPG.dat	1378.5	21.4	1.0	0.02	#NUM!	2.0E-06
YER001W	YER001W	S288C_ctrl_hmg124h_4.JPG.dat	1300.5	40.2	1.0	0.03	#NUM!	6.9E-06
YER002W	YER002W	S288C_ctrl_hmg124h_4.JPG.dat	1307.0	32.0	1.0	0.01	#NUM!	2.4E-05
YER004W	YER004W	S288C_ctrl_hmg124h_4.JPG.dat	1285.8	12.5	1.0	0.00	#NUM!	7.7E-08
YER005W	YER005W	S288C_ctrl_hmg124h_4.JPG.dat	1376.5	31.0	1.1	0.02	#NUM!	2.5E-06
YER007C-A	YER007C-A	S288C_ctrl_hmg124h_4.JPG.dat	1278.5	17.3	1.0	0.01	#NUM!	9.1E-07
YER007W	YER007W	S288C_ctrl_hmg124h_4.JPG.dat	1369.0	15.5	1.0	0.00	#NUM!	7.1E-06
YER010C	YER010C	S288C_ctrl_hmg124h_4.JPG.dat	1378.3	20.8	1.0	0.01	#NUM!	5.2E-07
YER011W	YER011W	S288C_ctrl_hmg124h_4.JPG.dat	1488.8	50.3	1.0	0.04	#NUM!	1.4E-05
YER016W	YER016W	S288C_ctrl_hmg124h_4.JPG.dat	1342.8	35.8	0.9	0.01	#NUM!	2.3E-05
YER019C-A	YER019C-A	S288C_ctrl_hmg124h_4.JPG.dat	1407.5	27.2	1.0	0.02	#NUM!	2.0E-06
YER019W	YER019W	S288C_ctrl_hmg124h_4.JPG.dat	1010.8	174.9	0.8	0.05	#NUM!	1.3E-03
YER020W	YER020W	S288C_ctrl_hmg124h_4.JPG.dat	1256.0	54.3	0.9	0.04	#NUM!	2.6E-05
YER024W	YER024W	S288C_ctrl_hmg124h_4.JPG.dat	1335.0	10.6	1.0	0.01	#NUM!	6.5E-08
YER027C	YER027C	S288C_ctrl_hmg124h_4.JPG.dat	1360.5	36.5	1.0	0.03	#NUM!	4.7E-06
YER028C	YER028C	S288C_ctrl_hmg124h_4.JPG.dat	1339.0	16.0	1.0	0.01	#NUM!	6.7E-07
YER030W	YER030W	S288C_ctrl_hmg124h_4.JPG.dat	1299.8	27.5	1.0	0.02	#NUM!	3.4E-06
YER031C	YER031C	S288C_ctrl_hmg124h_4.JPG.dat	1383.8	36.6	1.0	0.01	#NUM!	9.1E-06
YER032W	YER032W	S288C_ctrl_hmg124h_4.JPG.dat	1427.5	19.4	1.0	0.03	#NUM!	5.5E-06
YER033C	YER033C	S288C_ctrl_hmg124h_4.JPG.dat	1580.0	76.1	1.0	0.02	#NUM!	8.8E-05
YER034W	YER034W	S288C_ctrl_hmg124h_4.JPG.dat	1583.5	64.8	1.0	0.04	#NUM!	1.3E-05
YER035W	YER035W	S288C_ctrl_hmg124h_4.JPG.dat	1533.5	64.5	1.0	0.01	#NUM!	3.2E-05
YER038W-A	YER038W-A	S288C_ctrl_hmg124h_4.JPG.dat	1524.3	6.7	1.0	0.01	#NUM!	6.6E-08
YER039C	YER039C	S288C_ctrl_hmg124h_4.JPG.dat	1463.5	12.1	1.0	0.01	#NUM!	1.8E-07
YER039C-A	YER039C-A	S288C_ctrl_hmg124h_4.JPG.dat	1409.3	24.3	1.0	0.02	#NUM!	1.5E-06
YER041W	YER041W	S288C_ctrl_hmg124h_4.JPG.dat	1508.5	58.6	1.0	0.04	#NUM!	1.7E-05
YER042W	YER042W	S288C_ctrl_hmg124h_4.JPG.dat	1402.0	23.3	1.0	0.02	#NUM!	2.4E-06
YER044C-A	YER044C-A	S288C_ctrl_hmg124h_4.JPG.dat	1417.3	32.1	1.0	0.03	#NUM!	6.2E-06
YER045C	YER045C	S288C_ctrl_hmg124h_4.JPG.dat	1535.0	43.2	1.0	0.03	#NUM!	7.0E-06
YER046W	YER046W	S288C_ctrl_hmg124h_4.JPG.dat	1461.0	26.2	1.2	0.08	#NUM!	8.1E-05
YER046W-A	YER046W-A	S288C_ctrl_hmg124h_4.JPG.dat	1442.0	82.1	1.0	0.06	#NUM!	4.8E-05
YER047C	YER047C	S288C_ctrl_hmg124h_4.JPG.dat	1371.8	20.0	1.0	0.02	#NUM!	2.4E-06
YER048C	YER048C	S288C_ctrl_hmg124h_4.JPG.dat	1464.8	90.4	1.0	0.05	#NUM!	3.4E-05
YER049W	YER049W	S288C_ctrl_hmg124h_4.JPG.dat	1421.0	44.1	1.0	0.01	#NUM!	7.4E-05

YER051W	YER051W	S288C_ctrl_hmg124h_4.JPG.dat	1424.3	46.8	1.0	0.04	#NUM!	1.5E-05
YER052C	YER052C	S288C_ctrl_hmg124h_4.JPG.dat	1400.3	67.1	1.0	0.04	#NUM!	1.7E-05
YER053C	YER053C	S288C_ctrl_hmg124h_4.JPG.dat	1476.3	59.8	1.0	0.03	#NUM!	7.2E-06
YER054C	YER054C	S288C_ctrl_hmg124h_4.JPG.dat	1467.3	75.3	1.0	0.05	#NUM!	3.8E-05
YER055C	YER055C	S288C_ctrl_hmg124h_4.JPG.dat	366.0	732.0	0.0	0.00	#NUM!	
YER056C	YER056C	S288C_ctrl_hmg124h_4.JPG.dat	1413.8	129.4	1.0	0.07	#NUM!	1.2E-04
YER056C-A	YER056C-A	S288C_ctrl_hmg124h_4.JPG.dat	1290.5	104.9	0.9	0.03	#NUM!	3.0E-04
YER057C	YER057C	S288C_ctrl_hmg124h_4.JPG.dat	1399.3	46.2	1.0	0.03	#NUM!	1.2E-05
YER059W	YER059W	S288C_ctrl_hmg124h_4.JPG.dat	1407.3	31.8	1.0	0.02	#NUM!	3.8E-06
YER060W	YER060W	S288C_ctrl_hmg124h_4.JPG.dat	1423.3	28.3	1.0	0.02	#NUM!	3.1E-06
YER060W-.	YER060W-A	S288C_ctrl_hmg124h_4.JPG.dat	1359.5	86.5	1.0	0.06	#NUM!	6.6E-05
YER061C	YER061C	S288C_ctrl_hmg124h_4.JPG.dat	1157.5	116.3	0.8	0.00	#NUM!	3.7E-07
YER062C	YER062C	S288C_ctrl_hmg124h_4.JPG.dat	1367.0	49.8	1.0	0.04	#NUM!	1.3E-05
YER063W	YER063W	S288C_ctrl_hmg124h_4.JPG.dat	1395.0	17.5	1.0	0.01	#NUM!	4.2E-07
YER064C	YER064C	S288C_ctrl_hmg124h_4.JPG.dat	1353.5	16.4	1.0	0.01	#NUM!	4.7E-07
YER065C	YER065C	S288C_ctrl_hmg124h_4.JPG.dat	1356.0	43.4	1.0	0.00	#NUM!	4.4E-06
YER066C-A	YER066C-A	S288C_ctrl_hmg124h_4.JPG.dat	1348.0	59.1	1.0	0.05	#NUM!	2.9E-05
YER066W	YER066W	S288C_ctrl_hmg124h_4.JPG.dat	1362.3	46.2	1.0	0.05	#NUM!	2.7E-05
YER067C-A	YER067C-A	S288C_ctrl_hmg124h_4.JPG.dat	1393.5	65.9	1.0	0.04	#NUM!	2.3E-05
YER067W	YER067W	S288C_ctrl_hmg124h_4.JPG.dat	1402.8	34.9	1.0	0.00	#NUM!	2.0E-06
YER069W	YER069W	S288C_ctrl_hmg124h_4.JPG.dat	333.8	667.5	0.0	0.00	#NUM!	
YER071C	YER071C	S288C_ctrl_hmg124h_4.JPG.dat	1348.3	29.6	1.0	0.02	#NUM!	1.5E-06
YER072W	YER072W	S288C_ctrl_hmg124h_4.JPG.dat	1331.3	49.8	1.0	0.04	#NUM!	1.3E-05
YER073W	YER073W	S288C_ctrl_hmg124h_4.JPG.dat	1282.3	63.7	1.0	0.00	#NUM!	4.8E-06
YER074W	YER074W	S288C_ctrl_hmg124h_4.JPG.dat	1310.0	40.0	1.0	0.03	#NUM!	6.0E-06
YER075C	YER075C	S288C_ctrl_hmg124h_4.JPG.dat	1328.5	45.4	1.0	0.01	#NUM!	4.2E-05
YER077C	YER077C	S288C_ctrl_hmg124h_4.JPG.dat	1269.5	10.2	1.0	0.01	#NUM!	8.8E-08
YER078C	YER078C	S288C_ctrl_hmg124h_4.JPG.dat	1263.8	38.4	0.9	0.03	#NUM!	7.7E-06
YER079W	YER079W	S288C_ctrl_hmg124h_4.JPG.dat	1418.3	49.8	1.0	0.00	#NUM!	4.2E-07
YER080W	YER080W	S288C_ctrl_hmg124h_4.JPG.dat	1445.8	17.2	1.0	0.01	#NUM!	1.7E-05
YER081W	YER081W	S288C_ctrl_hmg124h_4.JPG.dat	1404.3	39.1	1.0	0.03	#NUM!	4.5E-06
YER083C	YER083C	S288C_ctrl_hmg124h_4.JPG.dat	744.8	127.7	0.5	0.09	#NUM!	1.4E-03
YER084W	YER084W	S288C_ctrl_hmg124h_4.JPG.dat	1353.3	35.7	1.0	0.03	#NUM!	4.5E-06
YER085C	YER085C	S288C_ctrl_hmg124h_4.JPG.dat	1324.0	33.3	1.0	0.02	#NUM!	3.7E-06
YER087C-A	YER087C-A	S288C_ctrl_hmg124h_4.JPG.dat	1326.5	69.3	1.0	0.04	#NUM!	1.8E-05
YER088C	YER088C	S288C_ctrl_hmg124h_4.JPG.dat	1324.8	36.3	1.0	0.03	#NUM!	5.5E-06
YER090W	YER090W	S288C_ctrl_hmg124h_4.JPG.dat	1252.8	87.9	1.0	0.07	#NUM!	8.6E-05
YER091C	YER091C	S288C_ctrl_hmg124h_4.JPG.dat	1291.0	65.2	1.0	0.04	#NUM!	1.6E-05
YER091C-A	YER091C-A	S288C_ctrl_hmg124h_4.JPG.dat	1391.8	14.0	1.0	0.01	#NUM!	6.8E-08
YER092W	YER092W	S288C_ctrl_hmg124h_4.JPG.dat	1019.3	145.3	0.7	0.09	#NUM!	5.5E-04
YER093C-A	YER093C-A	S288C_ctrl_hmg124h_4.JPG.dat	1435.5	55.0	1.0	0.04	#NUM!	2.1E-05
YER095W	YER095W	S288C_ctrl_hmg124h_4.JPG.dat	1245.8	186.3	0.8	0.01	#NUM!	3.2E-05
YER096W	YER096W	S288C_ctrl_hmg124h_4.JPG.dat	1375.8	17.1	1.0	0.00	#NUM!	7.2E-06
YER097W	YER097W	S288C_ctrl_hmg124h_4.JPG.dat	1325.8	27.8	1.0	0.02	#NUM!	2.6E-06
YER098W	YER098W	S288C_ctrl_hmg124h_4.JPG.dat	1313.3	25.3	1.0	0.02	#NUM!	2.2E-06
YER101C	YER101C	S288C_ctrl_hmg124h_4.JPG.dat	1337.8	55.7	1.0	0.04	#NUM!	1.6E-05
YER106W	YER106W	S288C_ctrl_hmg124h_4.JPG.dat	1321.8	51.5	1.0	0.01	#NUM!	1.5E-05
YER108C	YER108C	S288C_ctrl_hmg124h_4.JPG.dat	1343.0	38.1	1.0	0.03	#NUM!	4.4E-06
YER109C	YER109C	S288C_ctrl_hmg124h_4.JPG.dat	1375.8	54.7	1.0	0.04	#NUM!	1.2E-05
YER111C	YER111C	S288C_ctrl_hmg124h_4.JPG.dat	1387.0	17.0	1.0	0.01	#NUM!	3.0E-07
YER113C	YER113C	S288C_ctrl_hmg124h_4.JPG.dat	1363.3	64.5	1.0	0.04	#NUM!	1.3E-05
YER114C	YER114C	S288C_ctrl_hmg124h_4.JPG.dat	1456.3	37.1	1.0	0.02	#NUM!	2.5E-06
YER115C	YER115C	S288C_ctrl_hmg124h_4.JPG.dat	1418.3	30.5	1.0	0.02	#NUM!	3.5E-06
YER116C	YER116C	S288C_ctrl_hmg124h_4.JPG.dat	1089.0	107.5	0.8	0.03	#NUM!	4.4E-04
YER117W	YER117W	S288C_ctrl_hmg124h_4.JPG.dat	1308.3	51.8	1.0	0.04	#NUM!	1.3E-05
YER118C	YER118C	S288C_ctrl_hmg124h_4.JPG.dat	1279.5	37.9	1.0	0.03	#NUM!	6.2E-06
YER119C	YER119C	S288C_ctrl_hmg124h_4.JPG.dat	1320.5	31.0	1.0	0.01	#NUM!	1.1E-05
YER119C-A	YER119C-A	S288C_ctrl_hmg124h_4.JPG.dat	1325.8	46.3	1.0	0.03	#NUM!	6.7E-06
YER120W	YER120W	S288C_ctrl_hmg124h_4.JPG.dat	1289.3	18.0	1.0	0.00	#NUM!	2.9E-06
YER121W	YER121W	S288C_ctrl_hmg124h_4.JPG.dat	1329.0	17.4	1.0	0.01	#NUM!	1.3E-05
YER123W	YER123W	S288C_ctrl_hmg124h_4.JPG.dat	1221.0	137.8	0.9	0.10	#NUM!	4.1E-04
YER124C	YER124C	S288C_ctrl_hmg124h_4.JPG.dat	1402.3	29.7	1.0	0.03	#NUM!	5.7E-06
YER128W	YER128W	S288C_ctrl_hmg124h_4.JPG.dat	1468.5	66.9	1.0	0.03	#NUM!	6.8E-06
YER129W	YER129W	S288C_ctrl_hmg124h_4.JPG.dat	1410.8	19.4	1.0	0.00	#NUM!	1.5E-08
YER130C	YER130C	S288C_ctrl_hmg124h_4.JPG.dat	1479.8	26.2	1.0	0.01	#NUM!	2.0E-07
YER131W	YER131W	S288C_ctrl_hmg124h_4.JPG.dat	1250.3	43.7	0.9	0.02	#NUM!	1.5E-06
YER132C	YER132C	S288C_ctrl_hmg124h_4.JPG.dat	1362.8	36.7	1.0	0.01	#NUM!	4.4E-07
YER134C	YER134C	S288C_ctrl_hmg124h_4.JPG.dat	1362.3	26.7	1.0	0.02	#NUM!	1.3E-06
YER135C	YER135C	S288C_ctrl_hmg124h_4.JPG.dat	1366.0	21.1	1.0	0.00	#NUM!	1.7E-06
YER137C	YER137C	S288C_ctrl_hmg124h_4.JPG.dat	1382.0	23.6	1.0	0.01	#NUM!	8.7E-06

YER139C	YER139C	S288C_ctrl_hmg124h_4.JPG.dat	1306.3	12.1	1.0	0.01	#NUM!	2.3E-05
YER140W	YER140W	S288C_ctrl_hmg124h_4.JPG.dat	1434.3	34.1	1.0	0.02	#NUM!	2.6E-06
YER142C	YER142C	S288C_ctrl_hmg124h_4.JPG.dat	1452.3	99.1	1.3	0.09	#NUM!	9.8E-05
YER143W	YER143W	S288C_ctrl_hmg124h_4.JPG.dat	1427.0	44.3	1.0	0.01	#NUM!	1.1E-05
YER144C	YER144C	S288C_ctrl_hmg124h_4.JPG.dat	1399.5	34.9	1.0	0.01	#NUM!	1.6E-05
YER145C	YER145C	S288C_ctrl_hmg124h_4.JPG.dat	1406.3	77.0	1.0	0.04	#NUM!	2.0E-05
YER149C	YER149C	S288C_ctrl_hmg124h_4.JPG.dat	1447.5	60.2	1.0	0.04	#NUM!	1.3E-05
YER150W	YER150W	S288C_ctrl_hmg124h_4.JPG.dat	1464.0	28.0	1.0	0.04	#NUM!	1.3E-05
YER151C	YER151C	S288C_ctrl_hmg124h_4.JPG.dat	1254.8	153.9	0.8	0.02	#NUM!	1.3E-04
YER152C	YER152C	S288C_ctrl_hmg124h_4.JPG.dat	1465.3	67.6	1.0	0.03	#NUM!	9.7E-06
YER153C	YER153C	S288C_ctrl_hmg124h_4.JPG.dat	729.3	510.7	0.7	0.13	#NUM!	1.1E-02
YER155C	YER155C	S288C_ctrl_hmg124h_4.JPG.dat	1359.5	76.6	1.0	0.04	#NUM!	1.7E-05
YER156C	YER156C	S288C_ctrl_hmg124h_4.JPG.dat	1327.3	38.7	0.9	0.02	#NUM!	3.2E-06
YER158C	YER158C	S288C_ctrl_hmg124h_4.JPG.dat	1461.0	43.5	1.0	0.03	#NUM!	6.7E-06
YER161C	YER161C	S288C_ctrl_hmg124h_4.JPG.dat	1219.0	40.8	0.9	0.03	#NUM!	1.1E-05
YER162C	YER162C	S288C_ctrl_hmg124h_4.JPG.dat	1100.0	741.5	1.1	0.11	#NUM!	3.6E-03
YER163C	YER163C	S288C_ctrl_hmg124h_4.JPG.dat	1408.8	45.5	1.0	0.03	#NUM!	1.0E-05
YER164W	YER164W	S288C_ctrl_hmg124h_4.JPG.dat	1271.5	17.0	0.9	0.00	#NUM!	1.4E-06
YER166W	YER166W	S288C_ctrl_hmg124h_4.JPG.dat	1412.5	13.0	1.0	0.01	#NUM!	2.0E-07
YER167W	YER167W	S288C_ctrl_hmg124h_4.JPG.dat	1254.0	79.5	1.0	0.01	#NUM!	6.4E-05
YER170W	YER170W	S288C_ctrl_hmg124h_4.JPG.dat	1330.8	52.8	1.0	0.04	#NUM!	1.4E-05
YER173W	YER173W	S288C_ctrl_hmg124h_4.JPG.dat	1340.0	31.6	1.0	0.00	#NUM!	8.2E-06
YER174C	YER174C	S288C_ctrl_hmg124h_4.JPG.dat	1376.3	37.9	1.0	0.03	#NUM!	4.9E-06
YER175C	YER175C	S288C_ctrl_hmg124h_4.JPG.dat	1417.5	47.2	1.0	0.04	#NUM!	1.2E-05
YER176W	YER176W	S288C_ctrl_hmg124h_4.JPG.dat	1418.8	89.3	1.0	0.06	#NUM!	6.3E-05
YER177W	YER177W	S288C_ctrl_hmg124h_4.JPG.dat	1128.8	32.6	0.8	0.01	#NUM!	3.6E-05
YER178W	YER178W	S288C_ctrl_hmg124h_4.JPG.dat	1288.8	85.6	0.9	0.06	#NUM!	8.8E-05
YER179W	YER179W	S288C_ctrl_hmg124h_4.JPG.dat	1418.3	40.8	1.0	0.01	#NUM!	1.1E-05
YER180C	YER180C	S288C_ctrl_hmg124h_4.JPG.dat	1440.8	51.9	1.1	0.03	#NUM!	8.1E-06
YER181C	YER181C	S288C_ctrl_hmg124h_4.JPG.dat	1372.3	46.8	1.0	0.04	#NUM!	1.6E-05
YER182W	YER182W	S288C_ctrl_hmg124h_4.JPG.dat	1296.0	88.4	1.0	0.06	#NUM!	7.6E-05
YER183C	YER183C	S288C_ctrl_hmg124h_4.JPG.dat	1284.5	34.1	1.0	0.03	#NUM!	5.0E-06
YER184C	YER184C	S288C_ctrl_hmg124h_4.JPG.dat	1312.8	28.2	1.0	0.02	#NUM!	2.6E-06
YER185W	YER185W	S288C_ctrl_hmg124h_4.JPG.dat	1260.5	70.4	1.0	0.05	#NUM!	4.2E-05
YER186C	YER186C	S288C_ctrl_hmg124h_4.JPG.dat	1370.3	39.7	1.0	0.03	#NUM!	8.4E-06
YER187W	YER187W	S288C_ctrl_hmg124h_4.JPG.dat	1453.3	82.2	1.0	0.01	#NUM!	4.8E-05
YER188W	YER188W	S288C_ctrl_hmg124h_4.JPG.dat	1442.8	29.3	1.0	0.02	#NUM!	2.0E-06
YFL006W	YFL006W	S288C_ctrl_hmg124h_4.JPG.dat	1383.3	51.5	1.0	0.01	#NUM!	1.0E-05
YFL011W	YFL011W	S288C_ctrl_hmg124h_4.JPG.dat	1426.0	25.8	1.0	0.02	#NUM!	1.3E-06
YFL013W-/	YFL013W-A	S288C_ctrl_hmg124h_4.JPG.dat	1296.8	44.9	1.0	0.03	#NUM!	1.3E-05
YFL014W	YFL014W	S288C_ctrl_hmg124h_4.JPG.dat	1273.5	49.1	1.0	0.04	#NUM!	2.4E-05
YFL015C	YFL015C	S288C_ctrl_hmg124h_4.JPG.dat	1334.5	41.5	1.0	0.03	#NUM!	7.6E-06
YFL018C	YFL018C	S288C_ctrl_hmg124h_4.JPG.dat	1245.8	29.1	1.0	0.02	#NUM!	3.4E-06
YFL019C	YFL019C	S288C_ctrl_hmg124h_4.JPG.dat	1324.8	42.8	1.0	0.02	#NUM!	3.5E-06
YFL020C	YFL020C	S288C_ctrl_hmg124h_4.JPG.dat	1356.0	69.4	1.0	0.04	#NUM!	2.1E-05
YFL021W	YFL021W	S288C_ctrl_hmg124h_4.JPG.dat	1351.8	75.5	1.0	0.05	#NUM!	2.8E-05
YFL023W	YFL023W	S288C_ctrl_hmg124h_4.JPG.dat	1127.8	269.4	0.8	0.19	#NUM!	3.7E-03
YFL025C	YFL025C	S288C_ctrl_hmg124h_4.JPG.dat	1529.0	44.6	1.0	0.03	#NUM!	9.3E-06
YFL026W	YFL026W	S288C_ctrl_hmg124h_4.JPG.dat	0.0	0.0	0.0	0.00	#NUM!	
YFL027C	YFL027C	S288C_ctrl_hmg124h_4.JPG.dat	1416.5	47.7	1.0	0.03	#NUM!	8.6E-06
YFL028C	YFL028C	S288C_ctrl_hmg124h_4.JPG.dat	1354.5	24.8	1.0	0.01	#NUM!	2.1E-05
YFL030W	YFL030W	S288C_ctrl_hmg124h_4.JPG.dat	1310.3	30.3	1.0	0.02	#NUM!	3.1E-06
YFL031W	YFL031W	S288C_ctrl_hmg124h_4.JPG.dat	1297.0	40.8	1.0	0.01	#NUM!	4.8E-05
YFL032W	YFL032W	S288C_ctrl_hmg124h_4.JPG.dat	1210.3	84.9	0.9	0.06	#NUM!	8.8E-05
YFL034C-A	YFL034C-A	S288C_ctrl_hmg124h_4.JPG.dat	1332.5	23.6	1.0	0.02	#NUM!	1.4E-06
YFL034W	YFL034W	S288C_ctrl_hmg124h_4.JPG.dat	1385.3	42.0	1.0	0.03	#NUM!	6.4E-06
YFL035C-B	YFL035C-B	S288C_ctrl_hmg124h_4.JPG.dat	1391.3	14.7	1.0	0.01	#NUM!	6.3E-07
YFL036W	YFL036W	S288C_ctrl_hmg124h_4.JPG.dat	513.3	655.8	0.1	0.12	#NUM!	1.9E-01
YFL040W	YFL040W	S288C_ctrl_hmg124h_4.JPG.dat	1541.5	34.0	1.1	0.01	#NUM!	2.9E-05
YFL041W	YFL041W	S288C_ctrl_hmg124h_4.JPG.dat	1438.5	26.6	1.0	0.02	#NUM!	2.7E-06
YFL042C	YFL042C	S288C_ctrl_hmg124h_4.JPG.dat	1400.3	34.2	1.0	0.02	#NUM!	1.8E-06
YFL043C	YFL043C	S288C_ctrl_hmg124h_4.JPG.dat	1415.0	48.1	1.0	0.01	#NUM!	9.9E-06
YFL044C	YFL044C	S288C_ctrl_hmg124h_4.JPG.dat	1335.0	74.3	1.0	0.06	#NUM!	6.5E-05
YFL046W	YFL046W	S288C_ctrl_hmg124h_4.JPG.dat	1315.3	29.7	1.0	0.02	#NUM!	3.3E-06
YFL047W	YFL047W	S288C_ctrl_hmg124h_4.JPG.dat	1284.5	53.8	1.0	0.01	#NUM!	2.2E-05
YFL048C	YFL048C	S288C_ctrl_hmg124h_4.JPG.dat	1315.8	26.9	1.0	0.02	#NUM!	1.5E-06
YFL049W	YFL049W	S288C_ctrl_hmg124h_4.JPG.dat	1274.8	14.6	1.0	0.01	#NUM!	9.6E-07
YFL050C	YFL050C	S288C_ctrl_hmg124h_4.JPG.dat	1350.0	31.5	1.0	0.03	#NUM!	4.5E-06
YFL051C	YFL051C	S288C_ctrl_hmg124h_4.JPG.dat	1395.3	58.4	1.0	0.04	#NUM!	1.6E-05
YFL052W	YFL052W	S288C_ctrl_hmg124h_4.JPG.dat	1561.3	47.8	1.1	0.02	#NUM!	2.9E-06

YFL053W	YFL053W	S288C_ctrl_hmg124h_4.JPG.dat	1465.5	48.0	1.0	0.03	#NUM!		1.1E-05
YFL054C	YFL054C	S288C_ctrl_hmg124h_4.JPG.dat	1462.3	15.1	1.0	0.01	#NUM!		4.1E-07
YFL055W	YFL055W	S288C_ctrl_hmg124h_4.JPG.dat	1470.5	32.9	1.0	0.02	#NUM!		2.0E-06
YFL056C	YFL056C	S288C_ctrl_hmg124h_4.JPG.dat	1407.0	17.8	1.0	0.01	#NUM!		4.0E-07
YFR006W	YFR006W	S288C_ctrl_hmg124h_4.JPG.dat	1356.5	68.5	1.0	0.03	#NUM!		1.0E-05
YFR007W	YFR007W	S288C_ctrl_hmg124h_4.JPG.dat	1343.0	38.8	1.0	0.02	#NUM!		1.1E-06
YFR008W	YFR008W	S288C_ctrl_hmg124h_4.JPG.dat	1341.8	33.4	1.0	0.03	#NUM!		5.8E-06
YFR009W	YFR009W	S288C_ctrl_hmg124h_4.JPG.dat	1333.0	63.3	1.0	0.03	#NUM!		7.5E-06
YFR010W	YFR010W	S288C_ctrl_hmg124h_4.JPG.dat	878.8	494.1	0.6	0.35	#NUM!		3.7E-02
YFR011C	YFR011C	S288C_ctrl_hmg124h_4.JPG.dat	1368.3	64.1	1.0	0.07	#NUM!		7.6E-05
YOR202W	YOR202W	S288C_ctrl_hmg124h_4.JPG.dat	1412.0	316.4	1.0	0.21	#NUM!		1.8E-183
1	1	UWOPS87_Ator08_hmg124h_1.JPG.dat	995.8	66.9	1.1	0.03	0.066	0.029	5.3E-06
2	2	UWOPS87_Ator08_hmg124h_1.JPG.dat	861.0	80.7	1.0	0.08	0.006	0.075	1.3E-04
3	3	UWOPS87_Ator08_hmg124h_1.JPG.dat	824.3	92.0	1.0	0.09	0.015	0.085	1.9E-04
4	4	UWOPS87_Ator08_hmg124h_1.JPG.dat	930.3	60.0	1.1	0.03	0.054	0.030	2.6E-04
YAL002W	YAL002W	UWOPS87_Ator08_hmg124h_1.JPG.dat	799.5	42.6	1.0	0.07	-0.068	0.066	7.2E-05
YAL004W	YAL004W	UWOPS87_Ator08_hmg124h_1.JPG.dat	840.5	40.3	1.0	0.01	-0.069	0.008	2.2E-05
YAL005C	YAL005C	UWOPS87_Ator08_hmg124h_1.JPG.dat	906.5	43.7	1.1	0.01	0.081	0.010	2.7E-05
YAL007C	YAL007C	UWOPS87_Ator08_hmg124h_1.JPG.dat	950.3	55.1	1.0	0.02	-0.050	0.015	6.9E-05
YAL008W	YAL008W	UWOPS87_Ator08_hmg124h_1.JPG.dat	888.5	37.6	1.0	0.04	0.042	0.041	1.7E-05
YAL010C	YAL010C	UWOPS87_Ator08_hmg124h_1.JPG.dat	770.8	42.9	0.9	0.01	-0.075	0.005	1.1E-05
YAL011W	YAL011W	UWOPS87_Ator08_hmg124h_1.JPG.dat	843.8	83.6	1.0	0.12	-0.073	0.116	4.0E-04
YAL013W	YAL013W	UWOPS87_Ator08_hmg124h_1.JPG.dat	784.0	51.3	0.9	0.07	-0.007	0.067	1.0E-04
YAL014C	YAL014C	UWOPS87_Ator08_hmg124h_1.JPG.dat	830.8	31.6	1.0	0.04	-0.082	0.036	1.3E-05
YAL015C	YAL015C	UWOPS87_Ator08_hmg124h_1.JPG.dat	812.8	17.7	1.0	0.01	0.045	0.006	1.1E-05
YAL017W	YAL017W	UWOPS87_Ator08_hmg124h_1.JPG.dat	767.8	50.9	0.9	0.05	0.001	0.054	5.3E-05
YAL018C	YAL018C	UWOPS87_Ator08_hmg124h_1.JPG.dat	783.0	59.7	1.0	0.08	0.010	0.078	1.2E-04
YAL019W	YAL019W	UWOPS87_Ator08_hmg124h_1.JPG.dat	710.0	29.0	0.9	0.03	-0.201	0.031	1.2E-05
YAL020C	YAL020C	UWOPS87_Ator08_hmg124h_1.JPG.dat	791.8	34.2	1.0	0.04	-0.202	0.044	2.4E-05
YAL022C	YAL022C	UWOPS87_Ator08_hmg124h_1.JPG.dat	837.5	58.7	1.0	0.07	-0.042	0.071	9.9E-05
YAL023C	YAL023C	UWOPS87_Ator08_hmg124h_1.JPG.dat	836.3	44.9	1.0	0.04	0.088	0.044	2.3E-05
YAL026C	YAL026C	UWOPS87_Ator08_hmg124h_1.JPG.dat	760.5	45.8	1.0	0.01	-0.069	0.012	5.2E-05
YAL027W	YAL027W	UWOPS87_Ator08_hmg124h_1.JPG.dat	916.8	31.8	1.1	0.06	0.026	0.058	3.7E-05
YAL028W	YAL028W	UWOPS87_Ator08_hmg124h_1.JPG.dat	852.5	32.9	1.0	0.04	-0.055	0.037	1.3E-05
YAL029C	YAL029C	UWOPS87_Ator08_hmg124h_1.JPG.dat	813.3	28.4	1.0	0.03	-0.008	0.035	1.2E-05
YAL030W	YAL030W	UWOPS87_Ator08_hmg124h_1.JPG.dat	854.3	22.3	1.0	0.03	-0.019	0.031	7.4E-06
YAL031C	YAL031C	UWOPS87_Ator08_hmg124h_1.JPG.dat	752.3	117.9	1.0	0.03	0.099	0.035	3.8E-04
YAL034C	YAL034C	UWOPS87_Ator08_hmg124h_1.JPG.dat	811.5	86.3	1.1	0.11	0.027	0.106	2.8E-04
YAL036C	YAL036C	UWOPS87_Ator08_hmg124h_1.JPG.dat	778.5	39.3	1.0	0.04	0.075	0.044	2.4E-05
YAL037W	YAL037W	UWOPS87_Ator08_hmg124h_1.JPG.dat	757.8	25.7	1.0	0.03	-0.014	0.032	9.8E-06
YAL040C	YAL040C	UWOPS87_Ator08_hmg124h_1.JPG.dat	839.5	24.8	1.0	0.03	-0.024	0.025	4.3E-06
YAL042W	YAL042W	UWOPS87_Ator08_hmg124h_1.JPG.dat	802.5	46.2	1.0	0.05	-0.019	0.046	3.0E-05
YAL043C-A	YAL043C-A	UWOPS87_Ator08_hmg124h_1.JPG.dat	942.0	178.1	1.1	0.02	0.061	0.023	1.7E-04
YAL045C	YAL045C	UWOPS87_Ator08_hmg124h_1.JPG.dat	834.5	19.5	1.0	0.03	0.087	0.034	1.0E-05
YAL049C	YAL049C	UWOPS87_Ator08_hmg124h_1.JPG.dat	851.0	66.4	1.0	0.02	0.009	0.024	2.0E-04
YAL051W	YAL051W	UWOPS87_Ator08_hmg124h_1.JPG.dat	857.5	29.5	1.0	0.04	0.005	0.040	1.7E-05
YAL053W	YAL053W	UWOPS87_Ator08_hmg124h_1.JPG.dat	782.5	70.6	0.9	0.03	-0.159	0.031	3.8E-04
YAL054C	YAL054C	UWOPS87_Ator08_hmg124h_1.JPG.dat	825.3	15.1	1.1	0.02	0.052	0.019	1.6E-06
YAL055W	YAL055W	UWOPS87_Ator08_hmg124h_1.JPG.dat	783.5	15.5	1.0	0.02	0.032	0.019	2.0E-06
YAL056W	YAL056W	UWOPS87_Ator08_hmg124h_1.JPG.dat	811.8	28.8	1.0	0.01	-0.186	0.010	3.7E-05
YAL058C-A	YAL058C-A	UWOPS87_Ator08_hmg124h_1.JPG.dat	795.0	21.2	1.0	0.03	0.026	0.028	6.0E-06
YAL058W	YAL058W	UWOPS87_Ator08_hmg124h_1.JPG.dat	862.5	42.0	1.0	0.01	0.045	0.006	1.2E-05
YAL059W	YAL059W	UWOPS87_Ator08_hmg124h_1.JPG.dat	921.0	49.5	1.1	0.02	0.103	0.023	1.4E-04
YAL060W	YAL060W	UWOPS87_Ator08_hmg124h_1.JPG.dat	836.3	74.1	1.0	0.02	0.043	0.018	9.4E-05
YAL061W	YAL061W	UWOPS87_Ator08_hmg124h_1.JPG.dat	946.3	59.2	1.1	0.07	0.075	0.072	7.6E-05
YAL062W	YAL062W	UWOPS87_Ator08_hmg124h_1.JPG.dat	932.8	19.2	1.1	0.02	0.002	0.023	2.7E-06
YAL064C-A	YAL064C-A	UWOPS87_Ator08_hmg124h_1.JPG.dat	908.5	8.1	1.1	0.01	0.031	0.012	4.2E-07
YAL065C	YAL065C	UWOPS87_Ator08_hmg124h_1.JPG.dat	670.8	84.1	0.8	0.10	0.074	0.103	5.3E-04
YAL066W	YAL066W	UWOPS87_Ator08_hmg124h_1.JPG.dat	774.3	18.8	1.0	0.03	0.007	0.030	7.5E-06
YAL067C	YAL067C	UWOPS87_Ator08_hmg124h_1.JPG.dat	776.0	26.0	1.0	0.04	-0.155	0.039	1.6E-05
YAL068C	YAL068C	UWOPS87_Ator08_hmg124h_1.JPG.dat	762.5	25.7	1.0	0.01	0.042	0.007	1.8E-05
YAR002C-A	YAR002C-A	UWOPS87_Ator08_hmg124h_1.JPG.dat	973.5	59.6	1.2	0.08	0.148	0.081	8.2E-05
YAR002W	YAR002W	UWOPS87_Ator08_hmg124h_1.JPG.dat	764.8	153.4	1.0	0.05	0.038	0.052	9.2E-04
YAR003W	YAR003W	UWOPS87_Ator08_hmg124h_1.JPG.dat	799.5	61.5	1.0	0.06	-0.077	0.064	8.1E-05
YAR014C	YAR014C	UWOPS87_Ator08_hmg124h_1.JPG.dat	803.0	49.0	1.0	0.07	-0.041	0.066	8.0E-05
YAR015W	YAR015W	UWOPS87_Ator08_hmg124h_1.JPG.dat	800.8	41.1	1.0	0.04	0.004	0.036	1.4E-05
YAR018C	YAR018C	UWOPS87_Ator08_hmg124h_1.JPG.dat	823.0	88.4	1.0	0.10	0.125	0.100	3.1E-04
YAR020C	YAR020C	UWOPS87_Ator08_hmg124h_1.JPG.dat	862.8	20.5	1.0	0.03	-0.037	0.031	7.8E-06
YAR023C	YAR023C	UWOPS87_Ator08_hmg124h_1.JPG.dat	830.8	54.1	1.0	0.07	-0.103	0.066	7.3E-05
YAR027W	YAR027W	UWOPS87_Ator08_hmg124h_1.JPG.dat	809.3	73.3	1.1	0.08	0.087	0.079	1.2E-04

YAR028W	YAR028W	UWOPS87_Ator08_hmg124h_1.JPG.dat	615.3	183.6	0.9	0.06	0.076	0.065	1.6E-03
YAR029W	YAR029W	UWOPS87_Ator08_hmg124h_1.JPG.dat	840.0	53.1	1.1	0.06	0.006	0.058	4.5E-05
YAR030C	YAR030C	UWOPS87_Ator08_hmg124h_1.JPG.dat	817.3	45.3	1.0	0.05	0.046	0.052	3.5E-05
YAR031W	YAR031W	UWOPS87_Ator08_hmg124h_1.JPG.dat	801.5	90.6	1.0	0.11	-0.066	0.105	3.5E-04
YAR035W	YAR035W	UWOPS87_Ator08_hmg124h_1.JPG.dat	794.3	46.8	1.0	0.06	0.081	0.064	7.7E-05
YAR037W	YAR037W	UWOPS87_Ator08_hmg124h_1.JPG.dat	789.8	38.2	1.0	0.06	-0.083	0.056	4.6E-05
YAR040C	YAR040C	UWOPS87_Ator08_hmg124h_1.JPG.dat	702.0	93.3	0.9	0.12	0.040	0.122	7.2E-04
YAR042W	YAR042W	UWOPS87_Ator08_hmg124h_1.JPG.dat	838.3	28.6	1.0	0.04	-0.028	0.038	1.4E-05
YAR043C	YAR043C	UWOPS87_Ator08_hmg124h_1.JPG.dat	488.5	103.0	0.6	0.13	0.050	0.125	2.3E-03
YAR044W	YAR044W	UWOPS87_Ator08_hmg124h_1.JPG.dat	610.0	106.9	0.8	0.14	0.050	0.141	1.6E-03
YAR047C	YAR047C	UWOPS87_Ator08_hmg124h_1.JPG.dat	754.5	51.5	1.0	0.02	0.237	0.019	1.2E-04
YAR050W	YAR050W	UWOPS87_Ator08_hmg124h_1.JPG.dat	834.3	49.5	1.0	0.06	0.045	0.056	4.5E-05
YBL001C	YBL001C	UWOPS87_Ator08_hmg124h_1.JPG.dat	847.5	17.1	1.0	0.04	-0.025	0.038	1.5E-05
YBL003C	YBL003C	UWOPS87_Ator08_hmg124h_1.JPG.dat	827.3	73.0	1.0	0.07	0.085	0.071	1.0E-04
YBL005W	YBL005W	UWOPS87_Ator08_hmg124h_1.JPG.dat	733.5	204.2	0.8	0.24	-0.362	0.235	5.8E-03
YBL007C	YBL007C	UWOPS87_Ator08_hmg124h_1.JPG.dat	875.3	68.3	1.0	0.02	0.067	0.024	1.7E-04
YBL008W	YBL008W	UWOPS87_Ator08_hmg124h_1.JPG.dat	900.5	39.9	1.1	0.04	-0.040	0.044	1.9E-05
YBL009W	YBL009W	UWOPS87_Ator08_hmg124h_1.JPG.dat	858.0	72.4	1.0	0.07	0.045	0.067	8.2E-05
YBL010C	YBL010C	UWOPS87_Ator08_hmg124h_1.JPG.dat	905.0	63.6	1.0	0.09	-0.304	0.090	1.7E-04
YBL011W	YBL011W	UWOPS87_Ator08_hmg124h_1.JPG.dat	809.8	97.2	1.0	0.10	-0.156	0.101	3.2E-04
YBL013W	YBL013W	UWOPS87_Ator08_hmg124h_1.JPG.dat	497.8	126.4	0.6	0.15	-0.156	0.146	3.6E-03
YBL015W	YBL015W	UWOPS87_Ator08_hmg124h_1.JPG.dat	766.5	40.9	1.0	0.01	-0.021	0.011	3.6E-05
YBL016W	YBL016W	UWOPS87_Ator08_hmg124h_1.JPG.dat	711.3	100.8	1.0	0.13	-0.029	0.127	6.4E-04
YBL017C	YBL017C	UWOPS87_Ator08_hmg124h_1.JPG.dat	814.0	45.2	1.0	0.05	0.109	0.048	2.9E-05
YBL019W	YBL019W	UWOPS87_Ator08_hmg124h_1.JPG.dat	870.3	18.9	1.0	0.03	0.140	0.026	4.5E-06
YBL021C	YBL021C	UWOPS87_Ator08_hmg124h_1.JPG.dat	902.5	23.1	1.1	0.03	-0.003	0.028	4.9E-06
YBL024W	YBL024W	UWOPS87_Ator08_hmg124h_1.JPG.dat	883.5	53.8	1.0	0.06	0.122	0.061	6.2E-05
YBL025W	YBL025W	UWOPS87_Ator08_hmg124h_1.JPG.dat	808.3	49.8	1.0	0.06	-0.004	0.057	5.4E-05
YBL027W	YBL027W	UWOPS87_Ator08_hmg124h_1.JPG.dat	800.5	40.3	1.0	0.05	0.050	0.046	2.6E-05
YBL028C	YBL028C	UWOPS87_Ator08_hmg124h_1.JPG.dat	853.0	29.1	1.0	0.01	-0.086	0.013	5.3E-05
YBL029W	YBL029W	UWOPS87_Ator08_hmg124h_1.JPG.dat	834.0	36.9	1.0	0.04	-0.090	0.040	1.9E-05
YBL031W	YBL031W	UWOPS87_Ator08_hmg124h_1.JPG.dat	781.0	59.6	0.9	0.06	0.021	0.064	8.6E-05
YBL032W	YBL032W	UWOPS87_Ator08_hmg124h_1.JPG.dat	899.0	17.3	1.1	0.02	-0.090	0.024	3.0E-06
YBL036C	YBL036C	UWOPS87_Ator08_hmg124h_1.JPG.dat	744.5	36.3	1.0	0.04	0.033	0.036	1.3E-05
YBL037W	YBL037W	UWOPS87_Ator08_hmg124h_1.JPG.dat	772.0	29.0	1.0	0.04	0.017	0.037	1.4E-05
YBL039C	YBL039C	UWOPS87_Ator08_hmg124h_1.JPG.dat	810.3	40.1	1.0	0.05	0.118	0.051	3.6E-05
YBL042C	YBL042C	UWOPS87_Ator08_hmg124h_1.JPG.dat	816.0	45.9	1.0	0.05	0.028	0.047	3.0E-05
YBL043W	YBL043W	UWOPS87_Ator08_hmg124h_1.JPG.dat	896.8	82.3	1.1	0.10	0.009	0.099	2.2E-04
YBL046W	YBL046W	UWOPS87_Ator08_hmg124h_1.JPG.dat	853.0	31.3	1.1	0.04	0.055	0.036	1.1E-05
YBL047C	YBL047C	UWOPS87_Ator08_hmg124h_1.JPG.dat	706.3	57.1	0.9	0.07	-0.058	0.071	1.3E-04
YBL048W	YBL048W	UWOPS87_Ator08_hmg124h_1.JPG.dat	875.8	22.9	1.0	0.03	-0.037	0.025	4.0E-06
YBL049W	YBL049W	UWOPS87_Ator08_hmg124h_1.JPG.dat	917.5	49.9	1.1	0.06	0.081	0.059	4.3E-05
YBL051C	YBL051C	UWOPS87_Ator08_hmg124h_1.JPG.dat	736.3	20.9	0.9	0.03	-0.136	0.028	8.2E-06
YBL052C	YBL052C	UWOPS87_Ator08_hmg124h_1.JPG.dat	718.8	47.3	0.9	0.06	0.035	0.062	9.4E-05
YBL053W	YBL053W	UWOPS87_Ator08_hmg124h_1.JPG.dat	771.0	96.3	1.0	0.12	-0.234	0.123	4.5E-04
YBL054W	YBL054W	UWOPS87_Ator08_hmg124h_1.JPG.dat	742.8	38.9	1.0	0.05	-0.137	0.046	3.2E-05
YBL055C	YBL055C	UWOPS87_Ator08_hmg124h_1.JPG.dat	836.5	23.3	1.0	0.03	-0.043	0.028	5.4E-06
YBL056W	YBL056W	UWOPS87_Ator08_hmg124h_1.JPG.dat	749.5	41.0	0.9	0.05	-0.058	0.048	3.8E-05
YBL057C	YBL057C	UWOPS87_Ator08_hmg124h_1.JPG.dat	849.8	43.1	1.0	0.05	0.021	0.052	3.8E-05
YBL059W	YBL059W	UWOPS87_Ator08_hmg124h_1.JPG.dat	834.5	41.9	1.0	0.04	0.020	0.045	2.4E-05
YBL060W	YBL060W	UWOPS87_Ator08_hmg124h_1.JPG.dat	815.0	58.4	1.1	0.01	0.017	0.008	2.1E-05
YBL061C	YBL061C	UWOPS87_Ator08_hmg124h_1.JPG.dat	831.5	8.3	1.0	0.01	0.027	0.009	2.4E-07
YBL062W	YBL062W	UWOPS87_Ator08_hmg124h_1.JPG.dat	816.0	43.6	1.0	0.01	-0.037	0.014	6.9E-05
YBL063W	YBL063W	UWOPS87_Ator08_hmg124h_1.JPG.dat	929.3	45.6	1.1	0.04	0.169	0.043	1.5E-05
YBL064C	YBL064C	UWOPS87_Ator08_hmg124h_1.JPG.dat	796.8	47.0	1.0	0.07	0.007	0.070	1.0E-04
YBL065W	YBL065W	UWOPS87_Ator08_hmg124h_1.JPG.dat	749.3	11.6	1.0	0.00	0.037	0.005	7.3E-06
YBL066C	YBL066C	UWOPS87_Ator08_hmg124h_1.JPG.dat	828.5	48.2	1.0	0.06	0.057	0.060	5.3E-05
YBL067C	YBL067C	UWOPS87_Ator08_hmg124h_1.JPG.dat	738.8	27.5	0.9	0.01	0.017	0.007	2.2E-05
YBL068W	YBL068W	UWOPS87_Ator08_hmg124h_1.JPG.dat	765.3	8.7	0.9	0.01	-0.086	0.006	1.6E-05
YBL069W	YBL069W	UWOPS87_Ator08_hmg124h_1.JPG.dat	850.5	13.7	1.0	0.02	0.050	0.018	1.7E-06
YBL070C	YBL070C	UWOPS87_Ator08_hmg124h_1.JPG.dat	827.5	75.2	1.0	0.09	-0.124	0.090	2.1E-04
YBL071C	YBL071C	UWOPS87_Ator08_hmg124h_1.JPG.dat	763.5	34.6	0.9	0.04	-0.035	0.044	2.9E-05
YBL072C	YBL072C	UWOPS87_Ator08_hmg124h_1.JPG.dat	882.0	38.4	1.0	0.01	-0.036	0.008	2.2E-05
YBL075C	YBL075C	UWOPS87_Ator08_hmg124h_1.JPG.dat	882.8	53.0	1.0	0.06	-0.034	0.057	4.8E-05
YBL078C	YBL078C	UWOPS87_Ator08_hmg124h_1.JPG.dat	810.3	45.9	1.0	0.06	-0.037	0.058	5.9E-05
YBL079W	YBL079W	UWOPS87_Ator08_hmg124h_1.JPG.dat	757.8	33.3	0.9	0.04	-0.071	0.044	2.8E-05
YBL081W	YBL081W	UWOPS87_Ator08_hmg124h_1.JPG.dat	776.8	105.6	1.0	0.15	0.050	0.150	8.2E-04
YBL082C	YBL082C	UWOPS87_Ator08_hmg124h_1.JPG.dat	803.3	27.8	1.0	0.03	-0.079	0.034	1.1E-05
YBL083C	YBL083C	UWOPS87_Ator08_hmg124h_1.JPG.dat	860.5	44.7	1.1	0.02	0.012	0.019	1.1E-04
YBL085W	YBL085W	UWOPS87_Ator08_hmg124h_1.JPG.dat	798.3	33.2	1.0	0.04	0.061	0.043	2.4E-05

YBL086C	YBL086C	UWOPS87_Ator08_hmg124h_1.JPG.dat	767.5	55.8	0.9	0.07	-0.097	0.071	1.3E-04
YBL087C	YBL087C	UWOPS87_Ator08_hmg124h_1.JPG.dat	849.5	19.5	1.0	0.01	0.168	0.007	1.6E-05
YBL088C	YBL088C	UWOPS87_Ator08_hmg124h_1.JPG.dat	821.0	87.2	1.0	0.11	-0.038	0.110	3.4E-04
YBL089W	YBL089W	UWOPS87_Ator08_hmg124h_1.JPG.dat	937.8	27.0	1.1	0.03	0.082	0.033	7.7E-06
YBL091C	YBL091C	UWOPS87_Ator08_hmg124h_1.JPG.dat	827.0	37.0	1.0	0.04	0.004	0.040	2.0E-05
YBL091C-A	YBL091C-A	UWOPS87_Ator08_hmg124h_1.JPG.dat	781.8	97.4	1.0	0.02	-0.059	0.023	1.7E-04
YBL094C	YBL094C	UWOPS87_Ator08_hmg124h_1.JPG.dat	793.0	42.8	1.0	0.06	0.140	0.061	6.4E-05
YBL095W	YBL095W	UWOPS87_Ator08_hmg124h_1.JPG.dat	800.3	27.3	1.1	0.03	0.127	0.027	4.2E-06
YBL096C	YBL096C	UWOPS87_Ator08_hmg124h_1.JPG.dat	781.0	51.7	1.0	0.07	-0.096	0.068	8.5E-05
YBL098W	YBL098W	UWOPS87_Ator08_hmg124h_1.JPG.dat	739.8	41.3	0.9	0.01	0.153	0.012	5.1E-05
YBL100C	YBL100C	UWOPS87_Ator08_hmg124h_1.JPG.dat	861.0	26.2	1.1	0.04	-0.061	0.042	1.6E-05
YBL101C	YBL101C	UWOPS87_Ator08_hmg124h_1.JPG.dat	823.8	27.6	1.0	0.04	-0.123	0.037	1.4E-05
YBL102W	YBL102W	UWOPS87_Ator08_hmg124h_1.JPG.dat	728.0	14.4	0.9	0.02	0.035	0.018	2.0E-06
YBL103C	YBL103C	UWOPS87_Ator08_hmg124h_1.JPG.dat	762.0	53.6	1.0	0.06	0.003	0.065	7.7E-05
YBL104C	YBL104C	UWOPS87_Ator08_hmg124h_1.JPG.dat	818.8	42.4	1.0	0.05	-0.056	0.051	3.8E-05
YBL106C	YBL106C	UWOPS87_Ator08_hmg124h_1.JPG.dat	826.5	22.8	1.0	0.03	0.024	0.026	4.9E-06
YBL107C	YBL107C	UWOPS87_Ator08_hmg124h_1.JPG.dat	777.0	73.4	1.0	0.10	-0.013	0.098	2.7E-04
YBR001C	YBR001C	UWOPS87_Ator08_hmg124h_1.JPG.dat	813.3	70.1	1.0	0.08	0.015	0.081	1.3E-04
YBR005W	YBR005W	UWOPS87_Ator08_hmg124h_1.JPG.dat	792.0	34.7	1.0	0.00	-0.023	0.004	4.0E-06
YBR006W	YBR006W	UWOPS87_Ator08_hmg124h_1.JPG.dat	800.5	36.7	1.0	0.03	-0.181	0.034	1.2E-05
YBR007C	YBR007C	UWOPS87_Ator08_hmg124h_1.JPG.dat	836.0	81.7	1.0	0.10	0.035	0.098	2.7E-04
YBR008C	YBR008C	UWOPS87_Ator08_hmg124h_1.JPG.dat	895.0	12.1	1.0	0.01	0.227	0.014	6.7E-07
YBR009C	YBR009C	UWOPS87_Ator08_hmg124h_1.JPG.dat	905.3	23.1	1.0	0.01	0.001	0.013	5.6E-05
YBR010W	YBR010W	UWOPS87_Ator08_hmg124h_1.JPG.dat	836.0	40.8	1.0	0.05	-0.133	0.046	2.9E-05
YBR012C	YBR012C	UWOPS87_Ator08_hmg124h_1.JPG.dat	798.0	56.0	1.0	0.07	0.124	0.067	9.3E-05
YBR013C	YBR013C	UWOPS87_Ator08_hmg124h_1.JPG.dat	891.0	28.9	1.0	0.01	0.105	0.014	7.5E-07
YBR014C	YBR014C	UWOPS87_Ator08_hmg124h_1.JPG.dat	839.0	56.4	1.0	0.06	-0.123	0.064	7.8E-05
YBR015C	YBR015C	UWOPS87_Ator08_hmg124h_1.JPG.dat	874.8	63.6	1.1	0.01	0.023	0.012	4.0E-05
YBR016W	YBR016W	UWOPS87_Ator08_hmg124h_1.JPG.dat	851.0	36.2	1.1	0.03	0.038	0.028	5.3E-06
YBR018C	YBR018C	UWOPS87_Ator08_hmg124h_1.JPG.dat	765.3	30.5	0.9	0.02	-0.037	0.015	8.6E-05
YBR019C	YBR019C	UWOPS87_Ator08_hmg124h_1.JPG.dat	903.8	15.8	1.1	0.01	0.139	0.006	1.2E-05
YBR020W	YBR020W	UWOPS87_Ator08_hmg124h_1.JPG.dat	875.8	18.5	1.0	0.03	0.046	0.031	7.2E-06
YBR022W	YBR022W	UWOPS87_Ator08_hmg124h_1.JPG.dat	922.0	52.0	1.0	0.05	0.149	0.047	2.7E-05
YBR023C	YBR023C	UWOPS87_Ator08_hmg124h_1.JPG.dat	839.3	44.8	0.9	0.04	-0.205	0.039	2.0E-05
YBR024W	YBR024W	UWOPS87_Ator08_hmg124h_1.JPG.dat	860.3	35.4	1.0	0.03	-0.005	0.026	4.7E-06
YBR025C	YBR025C	UWOPS87_Ator08_hmg124h_1.JPG.dat	855.8	58.0	1.0	0.07	0.025	0.072	9.9E-05
YBR026C	YBR026C	UWOPS87_Ator08_hmg124h_1.JPG.dat	759.0	50.5	0.9	0.06	-0.097	0.058	8.1E-05
YBR027C	YBR027C	UWOPS87_Ator08_hmg124h_1.JPG.dat	813.8	38.3	0.9	0.04	-0.026	0.037	1.6E-05
YBR028C	YBR028C	UWOPS87_Ator08_hmg124h_1.JPG.dat	860.5	51.1	1.0	0.01	0.015	0.010	3.4E-05
YBR030W	YBR030W	UWOPS87_Ator08_hmg124h_1.JPG.dat	743.0	30.3	0.9	0.04	-0.129	0.045	3.7E-05
YBR031W	YBR031W	UWOPS87_Ator08_hmg124h_1.JPG.dat	755.8	15.3	1.0	0.03	0.188	0.028	6.0E-06
YBR032W	YBR032W	UWOPS87_Ator08_hmg124h_1.JPG.dat	826.3	66.4	1.0	0.09	0.076	0.089	1.7E-04
YBR033W	YBR033W	UWOPS87_Ator08_hmg124h_1.JPG.dat	814.5	32.8	1.0	0.03	0.102	0.030	7.0E-06
YBR034C	YBR034C	UWOPS87_Ator08_hmg124h_1.JPG.dat	772.8	32.2	0.9	0.05	-0.142	0.047	3.8E-05
YBR036C	YBR036C	UWOPS87_Ator08_hmg124h_1.JPG.dat	715.3	21.5	0.9	0.03	-0.268	0.026	8.0E-06
YBR040W	YBR040W	UWOPS87_Ator08_hmg124h_1.JPG.dat	745.5	88.5	0.9	0.10	-0.091	0.100	3.3E-04
YBR041W	YBR041W	UWOPS87_Ator08_hmg124h_1.JPG.dat	743.5	42.1	0.9	0.06	-0.132	0.057	6.5E-05
YBR042C	YBR042C	UWOPS87_Ator08_hmg124h_1.JPG.dat	833.5	22.0	1.0	0.01	-0.020	0.006	1.0E-05
YBR043C	YBR043C	UWOPS87_Ator08_hmg124h_1.JPG.dat	842.8	22.5	1.0	0.03	0.067	0.034	1.0E-05
YBR044C	YBR044C	UWOPS87_Ator08_hmg124h_1.JPG.dat	866.0	29.3	1.1	0.03	0.057	0.028	5.0E-06
YBR045C	YBR045C	UWOPS87_Ator08_hmg124h_1.JPG.dat	794.5	56.1	1.0	0.06	0.067	0.057	5.1E-05
YBR046C	YBR046C	UWOPS87_Ator08_hmg124h_1.JPG.dat	766.5	40.0	1.0	0.05	-0.019	0.049	3.2E-05
YBR047W	YBR047W	UWOPS87_Ator08_hmg124h_1.JPG.dat	825.8	48.7	1.1	0.02	-0.162	0.017	9.0E-05
YBR048W	YBR048W	UWOPS87_Ator08_hmg124h_1.JPG.dat	709.8	24.0	0.9	0.03	0.052	0.028	9.0E-06
YBR050C	YBR050C	UWOPS87_Ator08_hmg124h_1.JPG.dat	824.8	52.4	1.0	0.05	-0.040	0.055	4.9E-05
YBR051W	YBR051W	UWOPS87_Ator08_hmg124h_1.JPG.dat	885.5	9.0	1.1	0.02	0.014	0.020	1.9E-06
YBR052C	YBR052C	UWOPS87_Ator08_hmg124h_1.JPG.dat	719.8	40.4	0.9	0.05	-0.084	0.046	3.9E-05
YBR053C	YBR053C	UWOPS87_Ator08_hmg124h_1.JPG.dat	850.3	25.9	1.0	0.02	0.030	0.021	2.5E-06
YBR054W	YBR054W	UWOPS87_Ator08_hmg124h_1.JPG.dat	796.3	21.6	0.9	0.03	-0.078	0.026	5.5E-06
YBR056W	YBR056W	UWOPS87_Ator08_hmg124h_1.JPG.dat	823.5	30.7	1.0	0.04	-0.032	0.038	1.6E-05
YBR057C	YBR057C	UWOPS87_Ator08_hmg124h_1.JPG.dat	886.8	41.7	1.1	0.01	0.066	0.008	1.9E-05
YBR058C	YBR058C	UWOPS87_Ator08_hmg124h_1.JPG.dat	857.8	79.7	1.1	0.10	-0.083	0.101	2.2E-04
YBR059C	YBR059C	UWOPS87_Ator08_hmg124h_1.JPG.dat	657.5	20.8	0.9	0.02	0.009	0.023	5.0E-06
YBR061C	YBR061C	UWOPS87_Ator08_hmg124h_1.JPG.dat	770.3	55.1	1.0	0.07	0.008	0.065	8.2E-05
YBR062C	YBR062C	UWOPS87_Ator08_hmg124h_1.JPG.dat	809.8	22.8	1.0	0.01	-0.007	0.007	1.4E-05
YBR063C	YBR063C	UWOPS87_Ator08_hmg124h_1.JPG.dat	844.8	18.6	1.0	0.02	0.001	0.018	1.6E-06
YBR064W	YBR064W	UWOPS87_Ator08_hmg124h_1.JPG.dat	882.3	36.7	1.1	0.04	0.069	0.040	1.5E-05
YBR065C	YBR065C	UWOPS87_Ator08_hmg124h_1.JPG.dat	730.5	94.4	0.9	0.12	-0.165	0.120	6.5E-04
YBR066C	YBR066C	UWOPS87_Ator08_hmg124h_1.JPG.dat	968.0	160.8	1.2	0.18	0.016	0.183	1.1E-03
YBR067C	YBR067C	UWOPS87_Ator08_hmg124h_1.JPG.dat	864.8	23.3	1.0	0.01	-0.026	0.006	1.1E-05

YBR068C	YBR068C	UWOPS87_Ator08_hmg124h_1.JPG.dat	834.3	44.5	1.0	0.05	0.042	0.049	3.2E-05
YBR069C	YBR069C	UWOPS87_Ator08_hmg124h_1.JPG.dat	768.5	16.9	1.0	0.02	-0.091	0.022	3.6E-06
YBR071W	YBR071W	UWOPS87_Ator08_hmg124h_1.JPG.dat	784.5	51.0	1.0	0.07	0.081	0.072	9.9E-05
YBR072W	YBR072W	UWOPS87_Ator08_hmg124h_1.JPG.dat	785.3	43.1	1.0	0.05	0.046	0.050	3.4E-05
YBR073W	YBR073W	UWOPS87_Ator08_hmg124h_1.JPG.dat	803.0	31.9	1.0	0.01	0.008	0.011	4.1E-05
YBR074W	YBR074W	UWOPS87_Ator08_hmg124h_1.JPG.dat	751.3	35.2	0.9	0.01	-0.188	0.014	7.6E-05
YBR075W	YBR075W	UWOPS87_Ator08_hmg124h_1.JPG.dat	813.8	57.3	1.0	0.06	-0.062	0.062	7.4E-05
YBR076W	YBR076W	UWOPS87_Ator08_hmg124h_1.JPG.dat	906.3	100.1	1.1	0.11	0.025	0.110	3.0E-04
YBR077C	YBR077C	UWOPS87_Ator08_hmg124h_1.JPG.dat	912.8	38.0	1.1	0.05	-0.066	0.047	2.2E-05
YBR082C	YBR082C	UWOPS87_Ator08_hmg124h_1.JPG.dat	616.8	412.2	1.0	0.04	0.035	0.042	6.3E-04
YBR083W	YBR083W	UWOPS87_Ator08_hmg124h_1.JPG.dat	848.5	18.4	1.0	0.02	0.009	0.022	3.2E-06
YBR084C-A	YBR084C-A	UWOPS87_Ator08_hmg124h_1.JPG.dat	822.0	50.6	1.0	0.06	0.222	0.064	7.9E-05
YBR084W	YBR084W	UWOPS87_Ator08_hmg124h_1.JPG.dat	836.3	25.1	1.0	0.01	-0.036	0.010	2.9E-05
YBR090C	YBR090C	UWOPS87_Ator08_hmg124h_1.JPG.dat	752.5	25.5	1.0	0.02	-0.004	0.021	2.9E-06
YBR090C-A	YBR090C-A	UWOPS87_Ator08_hmg124h_1.JPG.dat	851.5	50.4	1.1	0.01	-0.049	0.015	5.6E-05
YBR092C	YBR092C	UWOPS87_Ator08_hmg124h_1.JPG.dat	842.3	38.3	1.0	0.04	0.054	0.041	1.7E-05
YBR093C	YBR093C	UWOPS87_Ator08_hmg124h_1.JPG.dat	659.0	28.7	0.8	0.05	-0.001	0.047	5.1E-05
YBR094W	YBR094W	UWOPS87_Ator08_hmg124h_1.JPG.dat	861.8	36.7	1.0	0.03	0.094	0.030	6.8E-06
YBR095C	YBR095C	UWOPS87_Ator08_hmg124h_1.JPG.dat	1108.8	55.6	1.3	0.07	0.248	0.074	4.8E-05
YBR098W	YBR098W	UWOPS87_Ator08_hmg124h_1.JPG.dat	834.3	28.4	1.0	0.03	0.089	0.032	7.8E-06
YBR099C	YBR099C	UWOPS87_Ator08_hmg124h_1.JPG.dat	762.0	42.0	1.0	0.01	-0.019	0.014	6.8E-05
YBR100W	YBR100W	UWOPS87_Ator08_hmg124h_1.JPG.dat	841.5	33.0	1.0	0.05	0.047	0.046	2.7E-05
YBR101C	YBR101C	UWOPS87_Ator08_hmg124h_1.JPG.dat	751.8	27.6	0.9	0.03	-0.080	0.026	6.4E-06
YBR103W	YBR103W	UWOPS87_Ator08_hmg124h_1.JPG.dat	649.5	37.0	0.8	0.01	-0.273	0.007	2.6E-05
YBR104W	YBR104W	UWOPS87_Ator08_hmg124h_1.JPG.dat	803.5	101.4	1.1	0.04	0.183	0.035	3.3E-04
YBR105C	YBR105C	UWOPS87_Ator08_hmg124h_1.JPG.dat	885.0	70.7	1.0	0.02	0.071	0.022	1.5E-04
YBR106W	YBR106W	UWOPS87_Ator08_hmg124h_1.JPG.dat	761.3	132.9	0.9	0.16	0.124	0.159	1.5E-03
YBR107C	YBR107C	UWOPS87_Ator08_hmg124h_1.JPG.dat	792.0	26.9	0.9	0.03	0.025	0.033	1.2E-05
YBR108W	YBR108W	UWOPS87_Ator08_hmg124h_1.JPG.dat	738.3	178.0	0.8	0.20	-0.140	0.203	3.8E-03
YBR111C	YBR111C	UWOPS87_Ator08_hmg124h_1.JPG.dat	885.3	31.2	1.0	0.01	-0.048	0.012	4.8E-05
YBR113W	YBR113W	UWOPS87_Ator08_hmg124h_1.JPG.dat	698.8	97.4	0.9	0.03	-0.166	0.029	3.5E-04
YBR114W	YBR114W	UWOPS87_Ator08_hmg124h_1.JPG.dat	802.0	57.6	0.9	0.01	-0.022	0.012	5.5E-05
YBR115C	YBR115C	UWOPS87_Ator08_hmg124h_1.JPG.dat	0.0	0.0	0.0	0.00	0.000	0.000	
YBR116C	YBR116C	UWOPS87_Ator08_hmg124h_1.JPG.dat	889.3	29.7	1.0	0.04	-0.043	0.036	1.2E-05
YBR119W	YBR119W	UWOPS87_Ator08_hmg124h_1.JPG.dat	862.8	11.5	1.0	0.01	0.163	0.013	5.5E-07
YBR121C	YBR121C	UWOPS87_Ator08_hmg124h_1.JPG.dat	795.8	44.1	1.0	0.07	-0.018	0.071	9.6E-05
YBR122C	YBR122C	UWOPS87_Ator08_hmg124h_1.JPG.dat	789.8	51.8	1.0	0.07	-0.076	0.071	9.2E-05
YBR125C	YBR125C	UWOPS87_Ator08_hmg124h_1.JPG.dat	843.5	34.8	1.0	0.05	0.124	0.048	2.8E-05
YBR126C	YBR126C	UWOPS87_Ator08_hmg124h_1.JPG.dat	832.8	51.3	0.9	0.01	-0.175	0.014	7.1E-05
YBR128C	YBR128C	UWOPS87_Ator08_hmg124h_1.JPG.dat	929.8	24.3	1.1	0.02	-0.031	0.016	1.0E-06
YBR129C	YBR129C	UWOPS87_Ator08_hmg124h_1.JPG.dat	970.8	21.5	1.1	0.00	0.081	0.002	9.9E-07
YBR130C	YBR130C	UWOPS87_Ator08_hmg124h_1.JPG.dat	812.5	8.2	0.9	0.00	-0.074	0.001	1.7E-07
YBR131W	YBR131W	UWOPS87_Ator08_hmg124h_1.JPG.dat	873.8	39.1	1.1	0.04	0.019	0.036	1.1E-05
YBR132C	YBR132C	UWOPS87_Ator08_hmg124h_1.JPG.dat	911.8	58.9	1.0	0.06	0.071	0.064	6.6E-05
YBR134W	YBR134W	UWOPS87_Ator08_hmg124h_1.JPG.dat	952.0	56.8	1.1	0.02	0.012	0.021	1.2E-04
YBR137W	YBR137W	UWOPS87_Ator08_hmg124h_1.JPG.dat	880.3	54.3	1.0	0.05	-0.102	0.053	3.8E-05
YBR138C	YBR138C	UWOPS87_Ator08_hmg124h_1.JPG.dat	829.8	59.7	1.0	0.07	-0.030	0.068	9.8E-05
YBR139W	YBR139W	UWOPS87_Ator08_hmg124h_1.JPG.dat	693.8	53.9	1.0	0.01	0.037	0.013	6.3E-05
YBR141C	YBR141C	UWOPS87_Ator08_hmg124h_1.JPG.dat	825.5	27.3	1.1	0.04	-0.020	0.035	1.0E-05
YBR144C	YBR144C	UWOPS87_Ator08_hmg124h_1.JPG.dat	818.5	22.3	1.0	0.01	-0.081	0.013	5.5E-05
YBR145W	YBR145W	UWOPS87_Ator08_hmg124h_1.JPG.dat	843.3	23.4	1.0	0.02	0.023	0.023	3.3E-06
YBR146W	YBR146W	UWOPS87_Ator08_hmg124h_1.JPG.dat	834.0	37.7	1.0	0.01	-0.101	0.012	5.4E-05
YBR147W	YBR147W	UWOPS87_Ator08_hmg124h_1.JPG.dat	849.3	35.5	1.0	0.05	0.020	0.053	3.6E-05
YBR148W	YBR148W	UWOPS87_Ator08_hmg124h_1.JPG.dat	800.8	7.5	1.0	0.01	0.033	0.008	1.5E-07
YBR149W	YBR149W	UWOPS87_Ator08_hmg124h_1.JPG.dat	933.5	46.2	1.1	0.06	0.037	0.055	3.4E-05
YBR150C	YBR150C	UWOPS87_Ator08_hmg124h_1.JPG.dat	840.0	42.5	1.0	0.05	0.053	0.055	4.4E-05
YBR151W	YBR151W	UWOPS87_Ator08_hmg124h_1.JPG.dat	826.0	35.1	1.0	0.04	0.071	0.042	1.9E-05
YBR156C	YBR156C	UWOPS87_Ator08_hmg124h_1.JPG.dat	801.5	59.1	1.0	0.07	0.086	0.066	8.4E-05
YBR157C	YBR157C	UWOPS87_Ator08_hmg124h_1.JPG.dat	746.8	33.5	1.0	0.04	-0.007	0.036	1.3E-05
YBR158W	YBR158W	UWOPS87_Ator08_hmg124h_1.JPG.dat	788.0	26.8	1.0	0.01	0.023	0.011	3.8E-05
YBR159W	YBR159W	UWOPS87_Ator08_hmg124h_1.JPG.dat	811.5	41.3	1.0	0.05	-0.017	0.050	3.5E-05
YBR161W	YBR161W	UWOPS87_Ator08_hmg124h_1.JPG.dat	844.3	18.0	1.0	0.01	-0.115	0.007	1.6E-05
YBR162C	YBR162C	UWOPS87_Ator08_hmg124h_1.JPG.dat	897.0	53.3	1.0	0.06	-0.008	0.061	5.5E-05
YBR162W-	YBR162W-A	UWOPS87_Ator08_hmg124h_1.JPG.dat	865.0	51.2	1.0	0.06	0.041	0.063	6.1E-05
YBR164C	YBR164C	UWOPS87_Ator08_hmg124h_1.JPG.dat	713.5	12.2	0.9	0.01	-0.087	0.014	1.2E-06
YBR165W	YBR165W	UWOPS87_Ator08_hmg124h_1.JPG.dat	849.0	20.4	1.0	0.02	0.104	0.025	4.4E-06
YBR166C	YBR166C	UWOPS87_Ator08_hmg124h_1.JPG.dat	836.3	45.9	1.0	0.01	0.124	0.007	1.7E-05
YBR168W	YBR168W	UWOPS87_Ator08_hmg124h_1.JPG.dat	876.3	33.6	1.1	0.04	0.187	0.035	1.0E-05
YBR169C	YBR169C	UWOPS87_Ator08_hmg124h_1.JPG.dat	847.5	53.2	1.0	0.07	0.006	0.069	8.4E-05
YBR170C	YBR170C	UWOPS87_Ator08_hmg124h_1.JPG.dat	753.5	46.4	1.0	0.01	-0.017	0.008	2.2E-05

YBR171W	YBR171W	UWOPS87_Ator08_hmg124h_1.JPG.dat	821.8	17.1	1.0	0.01	0.140	0.006	1.0E-05
YBR172C	YBR172C	UWOPS87_Ator08_hmg124h_1.JPG.dat	822.8	32.1	1.0	0.04	-0.002	0.043	2.0E-05
YBR174C	YBR174C	UWOPS87_Ator08_hmg124h_1.JPG.dat	752.8	50.4	0.9	0.06	-0.081	0.062	7.9E-05
YBR175W	YBR175W	UWOPS87_Ator08_hmg124h_1.JPG.dat	818.3	8.5	1.0	0.01	0.064	0.010	2.7E-07
YBR176W	YBR176W	UWOPS87_Ator08_hmg124h_1.JPG.dat	818.8	30.2	1.0	0.01	-0.081	0.013	5.5E-05
YBR177C	YBR177C	UWOPS87_Ator08_hmg124h_1.JPG.dat	860.8	54.6	1.1	0.07	0.049	0.065	6.3E-05
YBR178W	YBR178W	UWOPS87_Ator08_hmg124h_1.JPG.dat	868.0	70.5	1.0	0.02	0.003	0.023	1.6E-04
YBR180W	YBR180W	UWOPS87_Ator08_hmg124h_1.JPG.dat	850.0	54.5	1.0	0.06	0.012	0.064	7.3E-05
YBR181C	YBR181C	UWOPS87_Ator08_hmg124h_1.JPG.dat	758.0	26.9	0.9	0.03	0.108	0.031	9.9E-06
YBR182C	YBR182C	UWOPS87_Ator08_hmg124h_1.JPG.dat	868.8	61.3	1.1	0.07	-0.027	0.069	7.1E-05
YBR183W	YBR183W	UWOPS87_Ator08_hmg124h_1.JPG.dat	735.3	15.0	1.0	0.02	-0.049	0.020	2.4E-06
YBR184W	YBR184W	UWOPS87_Ator08_hmg124h_1.JPG.dat	815.3	29.3	1.0	0.04	0.078	0.044	2.2E-05
YBR185C	YBR185C	UWOPS87_Ator08_hmg124h_1.JPG.dat	865.3	62.7	1.1	0.08	-0.002	0.079	1.2E-04
YBR186W	YBR186W	UWOPS87_Ator08_hmg124h_1.JPG.dat	807.5	26.1	1.0	0.04	0.007	0.044	2.4E-05
YBR187W	YBR187W	UWOPS87_Ator08_hmg124h_1.JPG.dat	764.3	177.3	1.0	0.06	-0.018	0.058	1.2E-03
YBR188C	YBR188C	UWOPS87_Ator08_hmg124h_1.JPG.dat	816.5	64.2	1.0	0.08	0.006	0.076	1.3E-04
YBR189W	YBR189W	UWOPS87_Ator08_hmg124h_1.JPG.dat	491.3	373.7	0.6	0.45	-0.050	0.454	7.7E-02
YBR194W	YBR194W	UWOPS87_Ator08_hmg124h_1.JPG.dat	948.5	42.5	1.1	0.00	0.155	0.003	2.8E-06
YBR195C	YBR195C	UWOPS87_Ator08_hmg124h_1.JPG.dat	925.3	30.4	1.1	0.03	0.081	0.029	5.7E-06
YBR197C	YBR197C	UWOPS87_Ator08_hmg124h_1.JPG.dat	960.0	25.5	1.2	0.04	0.091	0.039	1.0E-05
YBR199W	YBR199W	UWOPS87_Ator08_hmg124h_1.JPG.dat	784.0	34.1	1.0	0.04	0.009	0.040	1.9E-05
YBR200W	YBR200W	UWOPS87_Ator08_hmg124h_1.JPG.dat	0.0	0.0	0.0	0.00	0.000	0.000	
YBR201W	YBR201W	UWOPS87_Ator08_hmg124h_1.JPG.dat	801.3	36.5	1.0	0.05	-0.109	0.046	2.6E-05
YBR203W	YBR203W	UWOPS87_Ator08_hmg124h_1.JPG.dat	811.3	79.7	1.0	0.02	-0.019	0.016	8.0E-05
YBR204C	YBR204C	UWOPS87_Ator08_hmg124h_1.JPG.dat	836.8	18.9	1.0	0.04	-0.073	0.038	1.5E-05
YBR205W	YBR205W	UWOPS87_Ator08_hmg124h_1.JPG.dat	860.5	26.1	1.0	0.04	0.015	0.039	1.6E-05
YBR206W	YBR206W	UWOPS87_Ator08_hmg124h_1.JPG.dat	785.8	28.9	1.0	0.04	0.001	0.038	1.7E-05
YBR207W	YBR207W	UWOPS87_Ator08_hmg124h_1.JPG.dat	760.3	44.1	1.0	0.06	-0.102	0.064	8.1E-05
YBR208C	YBR208C	UWOPS87_Ator08_hmg124h_1.JPG.dat	879.8	40.4	1.0	0.01	-0.018	0.008	2.3E-05
YBR209W	YBR209W	UWOPS87_Ator08_hmg124h_1.JPG.dat	919.3	37.9	1.1	0.04	0.015	0.042	1.6E-05
YBR210W	YBR210W	UWOPS87_Ator08_hmg124h_1.JPG.dat	799.8	64.7	1.0	0.03	0.026	0.025	2.1E-04
YBR212W	YBR212W	UWOPS87_Ator08_hmg124h_1.JPG.dat	849.3	35.0	1.0	0.00	-0.023	0.004	5.2E-06
YBR213W	YBR213W	UWOPS87_Ator08_hmg124h_1.JPG.dat	769.5	51.6	1.0	0.05	0.089	0.054	4.9E-05
YBR214W	YBR214W	UWOPS87_Ator08_hmg124h_1.JPG.dat	829.8	64.1	1.0	0.08	-0.207	0.077	1.3E-04
YBR215W	YBR215W	UWOPS87_Ator08_hmg124h_1.JPG.dat	968.0	10.3	1.1	0.01	0.054	0.008	1.2E-07
YBR216C	YBR216C	UWOPS87_Ator08_hmg124h_1.JPG.dat	901.0	256.5	1.0	0.30	0.235	0.297	6.0E-03
YBR217W	YBR217W	UWOPS87_Ator08_hmg124h_1.JPG.dat	808.0	73.1	0.9	0.08	-0.153	0.084	2.2E-04
YBR218C	YBR218C	UWOPS87_Ator08_hmg124h_1.JPG.dat	883.3	23.2	1.0	0.03	0.131	0.031	7.8E-06
YBR219C	YBR219C	UWOPS87_Ator08_hmg124h_1.JPG.dat	823.5	4.7	1.0	0.01	-0.078	0.007	8.6E-08
YBR220C	YBR220C	UWOPS87_Ator08_hmg124h_1.JPG.dat	912.5	31.1	1.0	0.03	-0.007	0.033	9.5E-06
YBR221C	YBR221C	UWOPS87_Ator08_hmg124h_1.JPG.dat	964.8	65.2	1.1	0.07	-0.026	0.072	7.9E-05
YBR222C	YBR222C	UWOPS87_Ator08_hmg124h_1.JPG.dat	850.8	34.2	1.0	0.04	-0.058	0.039	1.5E-05
YBR223C	YBR223C	UWOPS87_Ator08_hmg124h_1.JPG.dat	865.5	43.0	1.0	0.05	-0.061	0.046	2.3E-05
YOR202W	YOR202W	UWOPS87_Ator08_hmg124h_1.JPG.dat	842.3	93.2	1.0	0.08	0.006	0.080	1.3E-304
1	1	UWOPS87_Ator08_hmg124h_10.JPG.dat	1015.8	127.4	1.2	0.07	0.031	0.072	6.6E-05
2	2	UWOPS87_Ator08_hmg124h_10.JPG.dat	822.0	52.1	1.0	0.01	-0.113	0.006	1.3E-05
3	3	UWOPS87_Ator08_hmg124h_10.JPG.dat	918.8	120.4	1.1	0.13	0.128	0.130	4.2E-04
4	4	UWOPS87_Ator08_hmg124h_10.JPG.dat	811.3	105.7	1.0	0.12	0.140	0.116	3.8E-04
YLR415C	YLR415C	UWOPS87_Ator08_hmg124h_10.JPG.dat	611.3	74.1	1.0	0.11	0.074	0.112	4.4E-04
YLR416C	YLR416C	UWOPS87_Ator08_hmg124h_10.JPG.dat	661.5	23.7	1.0	0.01	0.052	0.010	3.7E-05
YLR418C	YLR418C	UWOPS87_Ator08_hmg124h_10.JPG.dat	504.3	49.0	0.7	0.05	-0.091	0.053	1.1E-04
YLR420W	YLR420W	UWOPS87_Ator08_hmg124h_10.JPG.dat	707.0	72.1	1.0	0.08	0.016	0.080	1.6E-04
YLR421C	YLR421C	UWOPS87_Ator08_hmg124h_10.JPG.dat	770.5	73.8	1.0	0.07	0.071	0.067	8.6E-05
YLR422W	YLR422W	UWOPS87_Ator08_hmg124h_10.JPG.dat	835.3	58.9	1.1	0.07	-0.177	0.065	6.1E-05
YLR423C	YLR423C	UWOPS87_Ator08_hmg124h_10.JPG.dat	723.5	67.1	0.9	0.08	-0.024	0.082	2.0E-04
YLR425W	YLR425W	UWOPS87_Ator08_hmg124h_10.JPG.dat	667.0	17.4	0.9	0.03	-0.188	0.034	1.6E-05
YLR426W	YLR426W	UWOPS87_Ator08_hmg124h_10.JPG.dat	817.3	46.4	1.1	0.07	0.013	0.070	7.7E-05
YLR427W	YLR427W	UWOPS87_Ator08_hmg124h_10.JPG.dat	764.8	21.5	1.0	0.04	-0.163	0.040	1.8E-05
YLR428C	YLR428C	UWOPS87_Ator08_hmg124h_10.JPG.dat	805.0	58.5	1.0	0.09	0.119	0.088	1.7E-04
YLR429W	YLR429W	UWOPS87_Ator08_hmg124h_10.JPG.dat	571.3	21.9	1.0	0.03	0.015	0.035	1.3E-05
YLR431C	YLR431C	UWOPS87_Ator08_hmg124h_10.JPG.dat	676.8	34.0	1.1	0.04	0.089	0.044	1.9E-05
YLR432W	YLR432W	UWOPS87_Ator08_hmg124h_10.JPG.dat	727.0	32.2	1.1	0.05	-0.085	0.049	2.6E-05
YLR433C	YLR433C	UWOPS87_Ator08_hmg124h_10.JPG.dat	770.0	78.2	1.1	0.10	0.026	0.105	2.2E-04
YLR434C	YLR434C	UWOPS87_Ator08_hmg124h_10.JPG.dat	649.5	14.0	0.9	0.01	-0.126	0.007	1.7E-05
YLR435W	YLR435W	UWOPS87_Ator08_hmg124h_10.JPG.dat	539.5	16.7	0.8	0.03	-0.256	0.026	1.1E-05
YLR436C	YLR436C	UWOPS87_Ator08_hmg124h_10.JPG.dat	791.3	69.2	1.0	0.09	-0.009	0.094	2.0E-04
YLR437C	YLR437C	UWOPS87_Ator08_hmg124h_10.JPG.dat	795.0	72.8	1.1	0.03	-0.026	0.029	2.2E-04
YLR438W	YLR438W	UWOPS87_Ator08_hmg124h_10.JPG.dat	712.3	39.2	1.0	0.05	-0.003	0.049	3.5E-05
YLR441C	YLR441C	UWOPS87_Ator08_hmg124h_10.JPG.dat	644.3	78.0	1.0	0.04	0.069	0.043	6.6E-04
YLR442C	YLR442C	UWOPS87_Ator08_hmg124h_10.JPG.dat	610.3	82.8	0.9	0.11	0.152	0.114	6.4E-04

YLR443W	YLR443W	UWOPS87_Ator08_hmg124h_10.JPG.dat 643.5	21.2	1.0	0.03	-0.008	0.031	7.6E-06
YLR444C	YLR444C	UWOPS87_Ator08_hmg124h_10.JPG.dat 656.0	27.9	1.0	0.05	-0.097	0.051	3.7E-05
YLR445W	YLR445W	UWOPS87_Ator08_hmg124h_10.JPG.dat 765.0	73.9	1.1	0.11	0.130	0.109	2.6E-04
YLR446W	YLR446W	UWOPS87_Ator08_hmg124h_10.JPG.dat 692.5	24.3	1.0	0.01	0.125	0.015	7.4E-05
YLR448W	YLR448W	UWOPS87_Ator08_hmg124h_10.JPG.dat 621.5	170.3	1.0	0.07	0.108	0.069	1.7E-03
YLR449W	YLR449W	UWOPS87_Ator08_hmg124h_10.JPG.dat 537.3	2.2	0.7	0.01	-0.161	0.005	1.2E-07
YLR450W	YLR450W	UWOPS87_Ator08_hmg124h_10.JPG.dat 29.8	59.5	0.0	0.00	0.000	0.000	
YLR451W	YLR451W	UWOPS87_Ator08_hmg124h_10.JPG.dat 726.8	83.0	1.0	0.11	-0.074	0.110	3.9E-04
YLR452C	YLR452C	UWOPS87_Ator08_hmg124h_10.JPG.dat 777.5	21.3	1.0	0.01	-0.060	0.008	2.1E-05
YLR453C	YLR453C	UWOPS87_Ator08_hmg124h_10.JPG.dat 747.5	73.6	1.0	0.03	0.004	0.030	3.1E-04
YLR454W	YLR454W	UWOPS87_Ator08_hmg124h_10.JPG.dat 638.0	35.8	0.9	0.02	0.054	0.021	1.8E-04
YLR455W	YLR455W	UWOPS87_Ator08_hmg124h_10.JPG.dat 644.3	54.5	0.9	0.02	-0.039	0.024	2.4E-04
YLR456W	YLR456W	UWOPS87_Ator08_hmg124h_10.JPG.dat 604.0	14.9	0.8	0.01	-0.333	0.011	5.6E-05
YLR460C	YLR460C	UWOPS87_Ator08_hmg124h_10.JPG.dat 685.0	460.1	1.2	0.08	0.184	0.082	1.6E-03
YLR461W	YLR461W	UWOPS87_Ator08_hmg124h_10.JPG.dat 840.8	62.0	1.1	0.09	-0.030	0.088	1.5E-04
YML001W	YML001W	UWOPS87_Ator08_hmg124h_10.JPG.dat 908.5	161.3	1.1	0.20	0.206	0.198	1.5E-03
YML002W	YML002W	UWOPS87_Ator08_hmg124h_10.JPG.dat 858.3	55.9	1.1	0.07	0.026	0.074	9.7E-05
YML003W	YML003W	UWOPS87_Ator08_hmg124h_10.JPG.dat 815.5	45.8	1.0	0.05	-0.063	0.051	3.9E-05
YML004C	YML004C	UWOPS87_Ator08_hmg124h_10.JPG.dat 803.0	46.4	1.0	0.06	0.076	0.059	5.3E-05
YML005W	YML005W	UWOPS87_Ator08_hmg124h_10.JPG.dat 772.3	34.0	1.0	0.04	-0.085	0.045	2.6E-05
YML006C	YML006C	UWOPS87_Ator08_hmg124h_10.JPG.dat 784.0	76.9	1.0	0.10	0.103	0.101	2.6E-04
YML007W	YML007W	UWOPS87_Ator08_hmg124h_10.JPG.dat 722.0	33.2	0.9	0.01	0.055	0.011	4.4E-05
YML008C	YML008C	UWOPS87_Ator08_hmg124h_10.JPG.dat 759.8	79.7	1.1	0.10	0.094	0.098	2.0E-04
YML009c	YML009c	UWOPS87_Ator08_hmg124h_10.JPG.dat 742.5	39.8	1.0	0.07	0.091	0.069	9.7E-05
YML010C-I	YML010C-B	UWOPS87_Ator08_hmg124h_10.JPG.dat 792.3	9.0	1.0	0.01	-0.086	0.005	9.6E-06
YML011C	YML011C	UWOPS87_Ator08_hmg124h_10.JPG.dat 827.8	96.1	1.0	0.03	-0.008	0.034	4.3E-04
YML012W	YML012W	UWOPS87_Ator08_hmg124h_10.JPG.dat 1010.8	50.1	1.2	0.07	0.146	0.074	6.6E-05
YML013C-/	YML013C-A	UWOPS87_Ator08_hmg124h_10.JPG.dat 881.5	100.4	1.1	0.12	0.101	0.121	4.1E-04
YML013W	YML013W	UWOPS87_Ator08_hmg124h_10.JPG.dat 797.5	25.6	1.0	0.03	-0.057	0.033	1.2E-05
YML016C	YML016C	UWOPS87_Ator08_hmg124h_10.JPG.dat 758.0	58.5	1.0	0.08	-0.024	0.084	1.8E-04
YML017W	YML017W	UWOPS87_Ator08_hmg124h_10.JPG.dat 830.8	66.4	1.1	0.10	0.020	0.097	2.1E-04
YML018C	YML018C	UWOPS87_Ator08_hmg124h_10.JPG.dat 771.0	42.3	1.0	0.05	-0.067	0.054	4.2E-05
YML019W	YML019W	UWOPS87_Ator08_hmg124h_10.JPG.dat 795.3	40.0	1.0	0.04	0.040	0.041	1.7E-05
YML020W	YML020W	UWOPS87_Ator08_hmg124h_10.JPG.dat 753.0	160.0	1.1	0.25	0.097	0.250	3.0E-03
YML021C	YML021C	UWOPS87_Ator08_hmg124h_10.JPG.dat 665.5	84.1	1.0	0.03	0.020	0.033	3.9E-04
YML022W	YML022W	UWOPS87_Ator08_hmg124h_10.JPG.dat 784.3	122.5	0.9	0.05	0.002	0.049	9.3E-04
YML026C	YML026C	UWOPS87_Ator08_hmg124h_10.JPG.dat 797.8	108.8	1.0	0.14	-0.042	0.136	6.6E-04
YML027W	YML027W	UWOPS87_Ator08_hmg124h_10.JPG.dat 818.5	55.0	1.0	0.08	-0.086	0.077	1.2E-04
YML028W	YML028W	UWOPS87_Ator08_hmg124h_10.JPG.dat 846.5	70.7	1.1	0.09	0.109	0.086	1.3E-04
YML029W	YML029W	UWOPS87_Ator08_hmg124h_10.JPG.dat 836.8	123.5	1.0	0.02	0.073	0.018	1.0E-04
YML030W	YML030W	UWOPS87_Ator08_hmg124h_10.JPG.dat 765.8	31.2	1.1	0.05	0.002	0.052	3.3E-05
YML032C	YML032C	UWOPS87_Ator08_hmg124h_10.JPG.dat 479.5	65.1	0.7	0.03	0.089	0.034	7.8E-04
YML033W	YML033W	UWOPS87_Ator08_hmg124h_10.JPG.dat 504.8	339.6	0.9	0.07	-0.039	0.074	2.1E-03
YML034W	YML034W	UWOPS87_Ator08_hmg124h_10.JPG.dat 730.8	13.5	1.0	0.01	0.046	0.009	2.4E-05
YML035C	YML035C	UWOPS87_Ator08_hmg124h_10.JPG.dat 649.8	186.4	0.9	0.25	0.126	0.245	5.9E-03
YML035C-/	YML035C-A	UWOPS87_Ator08_hmg124h_10.JPG.dat 831.3	65.4	1.1	0.04	0.146	0.036	3.7E-04
YML036W	YML036W	UWOPS87_Ator08_hmg124h_10.JPG.dat 716.3	148.8	0.9	0.19	0.088	0.186	2.6E-03
YML037C	YML037C	UWOPS87_Ator08_hmg124h_10.JPG.dat 830.3	32.9	1.0	0.03	-0.110	0.032	8.5E-06
YML038C	YML038C	UWOPS87_Ator08_hmg124h_10.JPG.dat 946.8	70.7	1.1	0.03	-0.080	0.029	2.3E-04
YML041C	YML041C	UWOPS87_Ator08_hmg124h_10.JPG.dat 832.0	152.8	1.1	0.20	-0.072	0.204	1.8E-03
YML042W	YML042W	UWOPS87_Ator08_hmg124h_10.JPG.dat 836.0	61.1	1.1	0.08	0.069	0.081	1.1E-04
YML047C	YML047C	UWOPS87_Ator08_hmg124h_10.JPG.dat 749.5	90.1	1.0	0.12	0.139	0.123	5.1E-04
YML048W	YML048W	UWOPS87_Ator08_hmg124h_10.JPG.dat 752.0	54.6	1.0	0.07	0.062	0.068	8.7E-05
YML048W-	YML048W-A	UWOPS87_Ator08_hmg124h_10.JPG.dat 536.0	358.2	0.9	0.03	-0.103	0.033	4.2E-04
YML051W	YML051W	UWOPS87_Ator08_hmg124h_10.JPG.dat 806.0	50.4	1.0	0.06	0.006	0.064	6.5E-05
YML052W	YML052W	UWOPS87_Ator08_hmg124h_10.JPG.dat 758.3	28.8	1.2	0.01	0.091	0.015	5.2E-05
YML053C	YML053C	UWOPS87_Ator08_hmg124h_10.JPG.dat 762.5	83.8	1.2	0.04	-0.035	0.040	3.7E-04
YML054C	YML054C	UWOPS87_Ator08_hmg124h_10.JPG.dat 573.3	87.4	0.9	0.03	0.025	0.027	3.4E-04
YML055W	YML055W	UWOPS87_Ator08_hmg124h_10.JPG.dat 752.3	88.1	1.1	0.13	-0.015	0.129	5.0E-04
YML056C	YML056C	UWOPS87_Ator08_hmg124h_10.JPG.dat 1035.0	324.1	1.2	0.04	-0.008	0.043	4.7E-04
YML057W	YML057W	UWOPS87_Ator08_hmg124h_10.JPG.dat 679.5	74.2	0.9	0.09	-0.081	0.087	2.7E-04
YML058C-/	YML058C-A	UWOPS87_Ator08_hmg124h_10.JPG.dat 822.3	90.3	1.1	0.02	-0.008	0.022	1.4E-04
YML058W	YML058W	UWOPS87_Ator08_hmg124h_10.JPG.dat 791.0	39.0	1.0	0.04	0.002	0.045	2.3E-05
YML059C	YML059C	UWOPS87_Ator08_hmg124h_10.JPG.dat 762.8	52.7	1.0	0.08	0.015	0.077	1.3E-04
YML060W	YML060W	UWOPS87_Ator08_hmg124h_10.JPG.dat 614.3	411.9	1.1	0.08	0.014	0.081	1.9E-03
YML062C	YML062C	UWOPS87_Ator08_hmg124h_10.JPG.dat 791.5	41.2	1.0	0.00	0.024	0.005	6.9E-06
YML063W	YML063W	UWOPS87_Ator08_hmg124h_10.JPG.dat 613.5	25.3	1.0	0.04	-0.033	0.042	1.9E-05
YML066C	YML066C	UWOPS87_Ator08_hmg124h_10.JPG.dat 526.5	236.8	0.9	0.39	0.086	0.391	2.1E-02
YML067C	YML067C	UWOPS87_Ator08_hmg124h_10.JPG.dat 554.0	295.1	0.8	0.45	-0.184	0.446	3.3E-02
YML068W	YML068W	UWOPS87_Ator08_hmg124h_10.JPG.dat 400.5	324.8	0.6	0.49	-0.221	0.491	9.2E-02

YML070W	YML070W	UWOPS87_Ator08_hmg124h_10.JPG.dat	641.3	229.3	1.1	0.08	0.031	0.079	1.7E-03
YML071C	YML071C	UWOPS87_Ator08_hmg124h_10.JPG.dat	531.3	356.8	1.0	0.08	-0.086	0.081	2.2E-03
YML072C	YML072C	UWOPS87_Ator08_hmg124h_10.JPG.dat	317.8	335.2	0.4	0.45	0.018	0.454	1.5E-01
YML074C	YML074C	UWOPS87_Ator08_hmg124h_10.JPG.dat	581.3	396.9	1.0	0.13	0.089	0.133	5.3E-03
YML075C	YML075C	UWOPS87_Ator08_hmg124h_10.JPG.dat	234.0	315.3	0.3	0.43	0.026	0.430	2.3E-01
YML076C	YML076C	UWOPS87_Ator08_hmg124h_10.JPG.dat	84.3	168.5	0.0	0.00	0.000	0.000	
YML081C-/	YML081C-A	UWOPS87_Ator08_hmg124h_10.JPG.dat	481.8	372.8	0.7	0.52	-0.111	0.523	8.2E-02
YML090W	YML090W	UWOPS87_Ator08_hmg124h_10.JPG.dat	671.3	30.3	1.1	0.01	0.049	0.009	2.4E-05
YML094W	YML094W	UWOPS87_Ator08_hmg124h_10.JPG.dat	348.8	343.9	0.3	0.14	-0.253	0.142	7.5E-02
YML095C	YML095C	UWOPS87_Ator08_hmg124h_10.JPG.dat	651.0	64.1	1.0	0.09	-0.076	0.093	2.6E-04
YML096W	YML096W	UWOPS87_Ator08_hmg124h_10.JPG.dat	620.8	29.8	0.9	0.04	-0.123	0.045	3.4E-05
YML097C	YML097C	UWOPS87_Ator08_hmg124h_10.JPG.dat	713.3	36.1	1.0	0.06	-0.132	0.056	4.7E-05
YML099C	YML099C	UWOPS87_Ator08_hmg124h_10.JPG.dat	875.0	89.2	1.3	0.04	0.269	0.037	3.0E-04
YML100W	YML100W	UWOPS87_Ator08_hmg124h_10.JPG.dat	765.8	41.2	1.0	0.04	-0.060	0.045	2.4E-05
YML100W-	YML100W-A	UWOPS87_Ator08_hmg124h_10.JPG.dat	824.8	87.1	1.1	0.12	0.090	0.118	3.4E-04
YML101C	YML101C	UWOPS87_Ator08_hmg124h_10.JPG.dat	754.0	38.1	1.0	0.05	-0.004	0.050	3.3E-05
YML102C-/	YML102C-A	UWOPS87_Ator08_hmg124h_10.JPG.dat	360.3	499.9	0.2	0.14	-0.299	0.144	2.0E-01
YML102W	YML102W	UWOPS87_Ator08_hmg124h_10.JPG.dat	839.5	60.8	1.1	0.01	0.086	0.006	1.1E-05
YML103C	YML103C	UWOPS87_Ator08_hmg124h_10.JPG.dat	725.5	8.1	1.1	0.02	0.039	0.018	1.3E-06
YML104C	YML104C	UWOPS87_Ator08_hmg124h_10.JPG.dat	737.8	28.4	1.1	0.04	0.198	0.041	1.7E-05
YML106W	YML106W	UWOPS87_Ator08_hmg124h_10.JPG.dat	751.5	35.9	1.0	0.01	-0.030	0.011	3.5E-05
YML107C	YML107C	UWOPS87_Ator08_hmg124h_10.JPG.dat	756.5	366.7	1.2	0.16	0.178	0.157	5.5E-03
YML108W	YML108W	UWOPS87_Ator08_hmg124h_10.JPG.dat	822.0	126.5	1.0	0.16	0.041	0.157	9.7E-04
YML109W	YML109W	UWOPS87_Ator08_hmg124h_10.JPG.dat	691.3	85.1	0.8	0.02	-0.274	0.023	2.7E-04
YML113W	YML113W	UWOPS87_Ator08_hmg124h_10.JPG.dat	634.8	387.7	1.0	0.16	0.011	0.156	8.1E-03
YML116W	YML116W	UWOPS87_Ator08_hmg124h_10.JPG.dat	759.3	40.7	1.0	0.01	-0.064	0.015	7.6E-05
YML117W	YML117W	UWOPS87_Ator08_hmg124h_10.JPG.dat	839.8	113.4	1.1	0.14	0.069	0.144	6.7E-04
YML117W-	YML117W-A	UWOPS87_Ator08_hmg124h_10.JPG.dat	836.8	53.0	1.1	0.06	-0.021	0.061	4.8E-05
YML118W	YML118W	UWOPS87_Ator08_hmg124h_10.JPG.dat	741.0	33.0	1.0	0.04	0.041	0.044	2.6E-05
YML119W	YML119W	UWOPS87_Ator08_hmg124h_10.JPG.dat	733.8	42.2	1.0	0.05	-0.030	0.053	3.7E-05
YML120C	YML120C	UWOPS87_Ator08_hmg124h_10.JPG.dat	639.8	128.6	0.9	0.18	-0.167	0.180	2.3E-03
YML121W	YML121W	UWOPS87_Ator08_hmg124h_10.JPG.dat	804.3	42.3	1.0	0.02	0.015	0.019	1.2E-04
YML122C	YML122C	UWOPS87_Ator08_hmg124h_10.JPG.dat	882.0	115.8	1.1	0.15	0.101	0.148	6.6E-04
YML123C	YML123C	UWOPS87_Ator08_hmg124h_10.JPG.dat	894.8	72.9	1.1	0.08	0.095	0.078	1.1E-04
YML124C	YML124C	UWOPS87_Ator08_hmg124h_10.JPG.dat	700.5	22.8	0.8	0.04	-0.137	0.039	2.8E-05
YML128C	YML128C	UWOPS87_Ator08_hmg124h_10.JPG.dat	948.5	48.9	1.1	0.06	0.099	0.062	4.4E-05
YML131W	YML131W	UWOPS87_Ator08_hmg124h_10.JPG.dat	763.5	114.6	0.9	0.02	-0.116	0.024	2.4E-04
YMR002W	YMR002W	UWOPS87_Ator08_hmg124h_10.JPG.dat	713.3	16.7	0.9	0.02	-0.074	0.017	1.8E-06
YMR003W	YMR003W	UWOPS87_Ator08_hmg124h_10.JPG.dat	795.3	58.5	1.0	0.08	-0.011	0.083	1.4E-04
YMR004W	YMR004W	UWOPS87_Ator08_hmg124h_10.JPG.dat	721.0	42.0	0.9	0.06	-0.093	0.061	7.3E-05
YMR006C	YMR006C	UWOPS87_Ator08_hmg124h_10.JPG.dat	700.0	54.0	1.0	0.01	-0.114	0.005	9.9E-06
YMR007W	YMR007W	UWOPS87_Ator08_hmg124h_10.JPG.dat	797.0	115.7	1.1	0.18	-0.002	0.176	1.0E-03
YMR008C	YMR008C	UWOPS87_Ator08_hmg124h_10.JPG.dat	836.8	110.7	1.1	0.13	0.001	0.133	4.8E-04
YMR009W	YMR009W	UWOPS87_Ator08_hmg124h_10.JPG.dat	834.5	110.0	1.1	0.14	0.198	0.144	6.4E-04
YMR010W	YMR010W	UWOPS87_Ator08_hmg124h_10.JPG.dat	536.0	71.4	0.7	0.09	-0.140	0.091	6.7E-04
YMR011W	YMR011W	UWOPS87_Ator08_hmg124h_10.JPG.dat	902.3	114.0	1.1	0.13	0.078	0.132	4.3E-04
YMR012W	YMR012W	UWOPS87_Ator08_hmg124h_10.JPG.dat	772.0	56.6	1.0	0.07	0.094	0.072	1.0E-04
YMR015C	YMR015C	UWOPS87_Ator08_hmg124h_10.JPG.dat	731.5	113.7	1.0	0.16	-0.025	0.161	1.1E-03
YMR016C	YMR016C	UWOPS87_Ator08_hmg124h_10.JPG.dat	752.8	42.8	1.0	0.06	-0.032	0.056	4.3E-05
YMR017W	YMR017W	UWOPS87_Ator08_hmg124h_10.JPG.dat	643.0	17.7	0.9	0.03	-0.120	0.027	7.6E-06
YMR018W	YMR018W	UWOPS87_Ator08_hmg124h_10.JPG.dat	700.3	25.1	1.0	0.04	-0.038	0.042	2.2E-05
YMR019W	YMR019W	UWOPS87_Ator08_hmg124h_10.JPG.dat	785.3	103.2	1.0	0.15	0.037	0.146	7.6E-04
YMR020W	YMR020W	UWOPS87_Ator08_hmg124h_10.JPG.dat	629.5	37.9	0.8	0.06	-0.141	0.056	9.0E-05
YMR021C	YMR021C	UWOPS87_Ator08_hmg124h_10.JPG.dat	810.8	80.7	1.0	0.08	-0.054	0.085	1.7E-04
YMR022W	YMR022W	UWOPS87_Ator08_hmg124h_10.JPG.dat	829.0	11.7	1.0	0.01	0.170	0.013	6.0E-07
YMR023C	YMR023C	UWOPS87_Ator08_hmg124h_10.JPG.dat	841.8	40.4	1.0	0.05	-0.097	0.047	2.7E-05
YMR025W	YMR025W	UWOPS87_Ator08_hmg124h_10.JPG.dat	887.0	47.6	1.1	0.08	0.206	0.076	8.5E-05
YMR026C	YMR026C	UWOPS87_Ator08_hmg124h_10.JPG.dat	823.3	116.6	1.1	0.15	0.017	0.155	7.9E-04
YMR027W	YMR027W	UWOPS87_Ator08_hmg124h_10.JPG.dat	731.5	25.2	1.0	0.03	-0.018	0.033	1.0E-05
YMR029C	YMR029C	UWOPS87_Ator08_hmg124h_10.JPG.dat	788.5	94.7	1.1	0.14	-0.041	0.136	5.9E-04
YMR030W	YMR030W	UWOPS87_Ator08_hmg124h_10.JPG.dat	765.5	65.9	1.0	0.10	0.000	0.096	2.2E-04
YMR031C	YMR031C	UWOPS87_Ator08_hmg124h_10.JPG.dat	733.8	93.4	0.9	0.11	0.004	0.112	4.5E-04
YMR031W	YMR031W-A	UWOPS87_Ator08_hmg124h_10.JPG.dat	607.8	20.5	0.9	0.04	-0.014	0.039	2.3E-05
YMR034C	YMR034C	UWOPS87_Ator08_hmg124h_10.JPG.dat	693.5	28.8	1.0	0.08	-0.055	0.077	1.3E-04
YMR035W	YMR035W	UWOPS87_Ator08_hmg124h_10.JPG.dat	717.0	42.0	1.0	0.04	-0.053	0.041	2.0E-05
YMR036C	YMR036C	UWOPS87_Ator08_hmg124h_10.JPG.dat	828.8	42.5	1.1	0.02	0.005	0.025	1.9E-04
YMR037C	YMR037C	UWOPS87_Ator08_hmg124h_10.JPG.dat	813.8	72.1	1.0	0.06	0.021	0.062	6.5E-05
YMR038C	YMR038C	UWOPS87_Ator08_hmg124h_10.JPG.dat	944.0	101.9	1.2	0.13	0.338	0.134	4.0E-04
YMR039C	YMR039C	UWOPS87_Ator08_hmg124h_10.JPG.dat	970.0	39.3	1.2	0.09	0.242	0.092	1.2E-04
YMR040W	YMR040W	UWOPS87_Ator08_hmg124h_10.JPG.dat	848.3	47.3	1.1	0.03	0.004	0.025	3.4E-06

YMR041C	YMR041C	UWOPS87_Ator08_hmg124h_10.JPG.dat	791.5	68.8	1.0	0.08	-0.092	0.081	1.4E-04
YMR042W	YMR042W	UWOPS87_Ator08_hmg124h_10.JPG.dat	852.5	71.7	1.1	0.05	0.076	0.047	2.2E-05
YMR044W	YMR044W	UWOPS87_Ator08_hmg124h_10.JPG.dat	867.3	20.8	1.1	0.04	-0.021	0.044	1.8E-05
YMR048W	YMR048W	UWOPS87_Ator08_hmg124h_10.JPG.dat	637.5	41.4	1.1	0.06	0.043	0.064	6.3E-05
YMR052C-.	YMR052C-A	UWOPS87_Ator08_hmg124h_10.JPG.dat	737.3	60.8	1.1	0.09	0.029	0.094	1.5E-04
YMR052W	YMR052W	UWOPS87_Ator08_hmg124h_10.JPG.dat	795.0	133.7	1.3	0.07	0.129	0.065	9.0E-04
YMR053C	YMR053C	UWOPS87_Ator08_hmg124h_10.JPG.dat	784.3	22.0	1.1	0.03	0.022	0.032	6.2E-06
YMR054W	YMR054W	UWOPS87_Ator08_hmg124h_10.JPG.dat	792.8	75.2	1.1	0.10	0.000	0.103	2.4E-04
YMR055C	YMR055C	UWOPS87_Ator08_hmg124h_10.JPG.dat	727.3	64.7	1.0	0.09	-0.051	0.088	1.9E-04
YMR056C	YMR056C	UWOPS87_Ator08_hmg124h_10.JPG.dat	816.0	65.5	1.0	0.02	-0.066	0.022	1.6E-04
YMR057C	YMR057C	UWOPS87_Ator08_hmg124h_10.JPG.dat	831.5	51.0	1.1	0.08	0.067	0.079	1.0E-04
YMR058W	YMR058W	UWOPS87_Ator08_hmg124h_10.JPG.dat	780.5	49.4	1.0	0.07	0.030	0.065	6.7E-05
YMR060C	YMR060C	UWOPS87_Ator08_hmg124h_10.JPG.dat	810.5	121.6	1.1	0.16	0.092	0.158	8.0E-04
YMR063W	YMR063W	UWOPS87_Ator08_hmg124h_10.JPG.dat	768.3	8.4	1.0	0.02	-0.024	0.020	1.9E-06
YMR065W	YMR065W	UWOPS87_Ator08_hmg124h_10.JPG.dat	633.0	26.0	1.0	0.03	-0.042	0.033	9.0E-06
YMR067C	YMR067C	UWOPS87_Ator08_hmg124h_10.JPG.dat	690.5	99.7	1.0	0.15	0.158	0.154	8.8E-04
YMR068W	YMR068W	UWOPS87_Ator08_hmg124h_10.JPG.dat	655.5	62.8	0.9	0.08	-0.045	0.085	2.0E-04
YMR070W	YMR070W	UWOPS87_Ator08_hmg124h_10.JPG.dat	815.8	93.1	1.1	0.13	0.112	0.131	4.1E-04
YMR073C	YMR073C	UWOPS87_Ator08_hmg124h_10.JPG.dat	823.0	37.3	1.1	0.05	0.100	0.045	1.8E-05
YMR075C-.	YMR075C-A	UWOPS87_Ator08_hmg124h_10.JPG.dat	710.5	40.4	1.0	0.01	0.060	0.015	7.4E-05
YMR075W	YMR075W	UWOPS87_Ator08_hmg124h_10.JPG.dat	986.8	308.4	1.1	0.10	0.019	0.102	3.1E-03
YMR078C	YMR078C	UWOPS87_Ator08_hmg124h_10.JPG.dat	690.3	329.3	0.9	0.43	0.141	0.434	2.5E-02
YMR080C	YMR080C	UWOPS87_Ator08_hmg124h_10.JPG.dat	920.0	50.8	1.2	0.07	0.114	0.073	6.2E-05
YMR085W	YMR085W	UWOPS87_Ator08_hmg124h_10.JPG.dat	771.0	39.6	1.0	0.05	0.000	0.049	2.9E-05
YMR086C-.	YMR086C-A	UWOPS87_Ator08_hmg124h_10.JPG.dat	755.5	91.7	1.0	0.12	-0.011	0.117	4.1E-04
YMR086W	YMR086W	UWOPS87_Ator08_hmg124h_10.JPG.dat	682.8	8.4	1.0	0.01	0.171	0.011	3.2E-07
YMR087W	YMR087W	UWOPS87_Ator08_hmg124h_10.JPG.dat	750.0	20.9	1.1	0.03	0.025	0.029	5.8E-06
YMR088C	YMR088C	UWOPS87_Ator08_hmg124h_10.JPG.dat	847.8	58.1	1.1	0.08	0.143	0.081	1.0E-04
YMR092C	YMR092C	UWOPS87_Ator08_hmg124h_10.JPG.dat	792.0	103.3	1.0	0.13	0.072	0.133	5.9E-04
YMR095C	YMR095C	UWOPS87_Ator08_hmg124h_10.JPG.dat	725.5	15.8	0.9	0.02	-0.113	0.020	2.8E-06
YMR096W	YMR096W	UWOPS87_Ator08_hmg124h_10.JPG.dat	892.3	52.7	1.1	0.01	0.184	0.010	2.4E-05
YMR099C	YMR099C	UWOPS87_Ator08_hmg124h_10.JPG.dat	866.3	86.2	1.0	0.11	0.040	0.113	3.4E-04
YMR100W	YMR100W	UWOPS87_Ator08_hmg124h_10.JPG.dat	911.3	30.3	1.1	0.04	0.190	0.036	8.3E-06
YMR101C	YMR101C	UWOPS87_Ator08_hmg124h_10.JPG.dat	798.3	61.0	1.0	0.08	-0.008	0.078	1.3E-04
YMR102C	YMR102C	UWOPS87_Ator08_hmg124h_10.JPG.dat	804.5	31.9	1.1	0.05	-0.077	0.046	2.4E-05
YMR103C	YMR103C	UWOPS87_Ator08_hmg124h_10.JPG.dat	782.0	36.5	1.0	0.02	-0.173	0.019	1.2E-04
YMR104C	YMR104C	UWOPS87_Ator08_hmg124h_10.JPG.dat	772.8	65.0	1.2	0.10	-0.018	0.097	1.6E-04
YMR105C	YMR105C	UWOPS87_Ator08_hmg124h_10.JPG.dat	822.0	130.7	1.2	0.20	0.111	0.198	1.3E-03
YMR106C	YMR106C	UWOPS87_Ator08_hmg124h_10.JPG.dat	750.0	28.4	1.0	0.03	-0.056	0.033	9.9E-06
YMR107W	YMR107W	UWOPS87_Ator08_hmg124h_10.JPG.dat	820.0	43.8	1.1	0.07	0.040	0.071	8.2E-05
YMR109W	YMR109W	UWOPS87_Ator08_hmg124h_10.JPG.dat	811.0	101.4	1.0	0.12	0.070	0.119	4.4E-04
YMR110C	YMR110C	UWOPS87_Ator08_hmg124h_10.JPG.dat	880.8	80.3	1.1	0.10	0.070	0.096	1.7E-04
YMR111C	YMR111C	UWOPS87_Ator08_hmg124h_10.JPG.dat	810.8	72.6	1.0	0.08	-0.005	0.082	1.4E-04
YMR114C	YMR114C	UWOPS87_Ator08_hmg124h_10.JPG.dat	810.0	77.0	1.1	0.10	0.139	0.104	2.4E-04
YMR115W	YMR115W	UWOPS87_Ator08_hmg124h_10.JPG.dat	781.8	76.1	1.0	0.03	-0.034	0.033	3.7E-04
YMR116C	YMR116C	UWOPS87_Ator08_hmg124h_10.JPG.dat	696.0	52.0	1.0	0.01	0.176	0.010	3.3E-05
YMR119W	YMR119W	UWOPS87_Ator08_hmg124h_10.JPG.dat	703.8	30.6	1.0	0.04	-0.031	0.040	1.9E-05
YMR119W	YMR119W-A	UWOPS87_Ator08_hmg124h_10.JPG.dat	583.0	42.0	0.8	0.07	-0.050	0.069	1.5E-04
YMR120C	YMR120C	UWOPS87_Ator08_hmg124h_10.JPG.dat	716.0	69.5	1.0	0.09	0.002	0.086	2.0E-04
YMR121C	YMR121C	UWOPS87_Ator08_hmg124h_10.JPG.dat	768.3	106.6	1.0	0.13	-0.011	0.129	6.5E-04
YMR122C	YMR122C	UWOPS87_Ator08_hmg124h_10.JPG.dat	721.0	65.9	0.9	0.08	-0.076	0.075	1.7E-04
YMR123W	YMR123W	UWOPS87_Ator08_hmg124h_10.JPG.dat	157.0	314.0	0.0	0.00	0.000	0.000	
YMR124W	YMR124W	UWOPS87_Ator08_hmg124h_10.JPG.dat	760.3	38.5	0.9	0.04	-0.134	0.042	2.6E-05
YMR126C	YMR126C	UWOPS87_Ator08_hmg124h_10.JPG.dat	763.0	112.5	0.9	0.05	-0.150	0.045	9.0E-04
YMR127C	YMR127C	UWOPS87_Ator08_hmg124h_10.JPG.dat	698.0	42.0	0.9	0.01	-0.177	0.014	8.3E-05
YMR129W	YMR129W	UWOPS87_Ator08_hmg124h_10.JPG.dat	795.8	56.0	1.0	0.01	-0.006	0.011	3.8E-05
YMR130W	YMR130W	UWOPS87_Ator08_hmg124h_10.JPG.dat	782.0	16.5	1.0	0.03	-0.066	0.025	3.7E-06
YMR132C	YMR132C	UWOPS87_Ator08_hmg124h_10.JPG.dat	784.5	31.5	1.0	0.04	0.067	0.039	1.5E-05
YMR133W	YMR133W	UWOPS87_Ator08_hmg124h_10.JPG.dat	709.5	31.4	1.0	0.05	-0.100	0.048	3.2E-05
YMR135C	YMR135C	UWOPS87_Ator08_hmg124h_10.JPG.dat	895.5	87.1	1.2	0.02	0.065	0.016	5.7E-05
YMR135W	YMR135W-A	UWOPS87_Ator08_hmg124h_10.JPG.dat	865.8	30.5	1.1	0.04	-0.040	0.043	1.9E-05
YMR136W	YMR136W	UWOPS87_Ator08_hmg124h_10.JPG.dat	789.5	48.6	1.0	0.07	-0.136	0.075	1.3E-04
YMR137C	YMR137C	UWOPS87_Ator08_hmg124h_10.JPG.dat	761.8	116.2	0.9	0.12	0.030	0.124	6.8E-04
YMR138W	YMR138W	UWOPS87_Ator08_hmg124h_10.JPG.dat	682.0	41.8	0.9	0.08	-0.129	0.076	1.9E-04
YMR139W	YMR139W	UWOPS87_Ator08_hmg124h_10.JPG.dat	774.0	61.3	1.0	0.05	0.069	0.050	3.7E-05
YMR140W	YMR140W	UWOPS87_Ator08_hmg124h_10.JPG.dat	773.3	70.7	1.0	0.11	0.011	0.106	3.0E-04
YMR141C	YMR141C	UWOPS87_Ator08_hmg124h_10.JPG.dat	756.8	18.9	1.0	0.03	0.015	0.028	6.3E-06
YMR143W	YMR143W	UWOPS87_Ator08_hmg124h_10.JPG.dat	784.3	13.5	1.0	0.02	-0.001	0.023	2.9E-06
YMR144W	YMR144W	UWOPS87_Ator08_hmg124h_10.JPG.dat	761.0	43.3	1.0	0.05	0.060	0.046	2.7E-05
YMR145C	YMR145C	UWOPS87_Ator08_hmg124h_10.JPG.dat	503.3	63.0	0.7	0.10	-0.078	0.101	7.3E-04

YMR147W	YMR147W	UWOPS87_Ator08_hmg124h_10.JPG.dat	694.8	59.7	1.0	0.08	0.011	0.081	1.4E-04
YMR148W	YMR148W	UWOPS87_Ator08_hmg124h_10.JPG.dat	813.8	19.8	1.1	0.05	0.057	0.049	2.2E-05
YMR152W	YMR152W	UWOPS87_Ator08_hmg124h_10.JPG.dat	671.0	99.7	1.0	0.04	0.013	0.036	4.8E-04
YMR153C-.	YMR153C-A	UWOPS87_Ator08_hmg124h_10.JPG.dat	638.5	53.1	0.8	0.07	-0.107	0.067	1.6E-04
YMR153W	YMR153W	UWOPS87_Ator08_hmg124h_10.JPG.dat	559.0	138.9	0.7	0.20	-0.131	0.202	6.2E-03
YMR154C	YMR154C	UWOPS87_Ator08_hmg124h_10.JPG.dat	726.8	34.3	0.9	0.02	0.014	0.020	3.0E-06
YMR155W	YMR155W	UWOPS87_Ator08_hmg124h_10.JPG.dat	745.0	60.0	1.0	0.05	-0.145	0.053	4.7E-05
YMR156C	YMR156C	UWOPS87_Ator08_hmg124h_10.JPG.dat	765.5	81.7	1.0	0.03	-0.053	0.030	2.8E-04
YMR157C	YMR157C	UWOPS87_Ator08_hmg124h_10.JPG.dat	781.3	41.3	1.0	0.03	-0.038	0.035	1.1E-05
YMR158C-	YMR158C-B	UWOPS87_Ator08_hmg124h_10.JPG.dat	905.0	91.7	1.1	0.10	-0.016	0.098	1.7E-04
YMR158W	YMR158W-A	UWOPS87_Ator08_hmg124h_10.JPG.dat	532.8	71.4	0.9	0.11	-0.083	0.114	6.3E-04
YMR159C	YMR159C	UWOPS87_Ator08_hmg124h_10.JPG.dat	601.5	15.8	1.0	0.02	0.098	0.016	1.2E-06
YMR160W	YMR160W	UWOPS87_Ator08_hmg124h_10.JPG.dat	631.3	151.0	0.9	0.23	-0.080	0.228	3.7E-03
YMR161W	YMR161W	UWOPS87_Ator08_hmg124h_10.JPG.dat	586.8	53.9	0.9	0.01	0.002	0.006	1.6E-05
YMR162C	YMR162C	UWOPS87_Ator08_hmg124h_10.JPG.dat	590.8	47.5	0.8	0.02	0.105	0.015	1.2E-04
YMR163C	YMR163C	UWOPS87_Ator08_hmg124h_10.JPG.dat	661.5	38.4	0.9	0.05	0.073	0.055	6.1E-05
YMR164C	YMR164C	UWOPS87_Ator08_hmg124h_10.JPG.dat	766.3	37.0	1.0	0.04	-0.046	0.044	2.2E-05
YMR166C	YMR166C	UWOPS87_Ator08_hmg124h_10.JPG.dat	698.5	56.7	0.9	0.08	0.173	0.078	1.6E-04
YMR167W	YMR167W	UWOPS87_Ator08_hmg124h_10.JPG.dat	842.0	85.3	1.1	0.10	0.097	0.104	2.2E-04
YMR169c	YMR169c	UWOPS87_Ator08_hmg124h_10.JPG.dat	661.5	88.1	0.9	0.11	0.081	0.115	5.5E-04
YMR170C	YMR170C	UWOPS87_Ator08_hmg124h_10.JPG.dat	700.0	112.9	0.9	0.15	-0.065	0.149	1.1E-03
YMR171C	YMR171C	UWOPS87_Ator08_hmg124h_10.JPG.dat	637.3	77.0	1.0	0.12	-0.002	0.119	4.7E-04
YMR172C-.	YMR172C-A	UWOPS87_Ator08_hmg124h_10.JPG.dat	629.3	30.5	1.0	0.01	-0.085	0.013	6.4E-05
YMR172W	YMR172W	UWOPS87_Ator08_hmg124h_10.JPG.dat	717.0	44.3	1.0	0.05	0.037	0.051	3.3E-05
YMR173W	YMR173W	UWOPS87_Ator08_hmg124h_10.JPG.dat	650.3	18.8	0.9	0.03	-0.086	0.033	1.2E-05
YMR173W	YMR173W-A	UWOPS87_Ator08_hmg124h_10.JPG.dat	673.0	19.6	0.9	0.03	-0.128	0.033	1.3E-05
YMR174C	YMR174C	UWOPS87_Ator08_hmg124h_10.JPG.dat	707.8	63.6	1.0	0.08	-0.049	0.084	1.9E-04
YMR175w	YMR175w	UWOPS87_Ator08_hmg124h_10.JPG.dat	743.0	40.8	1.0	0.06	-0.056	0.064	8.0E-05
YMR176W	YMR176W	UWOPS87_Ator08_hmg124h_10.JPG.dat	824.8	35.2	1.1	0.05	0.073	0.053	3.4E-05
YMR177W	YMR177W	UWOPS87_Ator08_hmg124h_10.JPG.dat	750.0	76.7	1.0	0.10	0.106	0.099	2.8E-04
YMR178W	YMR178W	UWOPS87_Ator08_hmg124h_10.JPG.dat	740.0	81.6	1.0	0.12	0.063	0.116	4.3E-04
YMR179W	YMR179W	UWOPS87_Ator08_hmg124h_10.JPG.dat	758.8	38.4	1.0	0.05	-0.123	0.047	2.8E-05
YMR180C	YMR180C	UWOPS87_Ator08_hmg124h_10.JPG.dat	602.5	71.6	0.8	0.04	-0.089	0.035	6.0E-04
YMR181C	YMR181C	UWOPS87_Ator08_hmg124h_10.JPG.dat	628.5	54.8	0.9	0.08	-0.209	0.077	1.6E-04
YMR182C	YMR182C	UWOPS87_Ator08_hmg124h_10.JPG.dat	805.8	80.1	1.1	0.10	0.023	0.103	2.3E-04
YMR183C	YMR183C	UWOPS87_Ator08_hmg124h_10.JPG.dat	643.3	49.2	0.9	0.07	-0.166	0.066	1.2E-04
YMR186W	YMR186W	UWOPS87_Ator08_hmg124h_10.JPG.dat	758.5	53.2	0.9	0.00	-0.060	0.004	4.9E-06
YMR187C	YMR187C	UWOPS87_Ator08_hmg124h_10.JPG.dat	867.8	109.9	1.1	0.13	0.120	0.135	5.2E-04
YMR188C	YMR188C	UWOPS87_Ator08_hmg124h_10.JPG.dat	835.8	101.7	1.0	0.12	-0.023	0.122	4.6E-04
YMR189W	YMR189W	UWOPS87_Ator08_hmg124h_10.JPG.dat	739.8	58.2	0.9	0.06	-0.073	0.064	9.2E-05
YMR190C	YMR190C	UWOPS87_Ator08_hmg124h_10.JPG.dat	794.0	12.9	1.0	0.02	-0.041	0.024	3.6E-06
YMR191W	YMR191W	UWOPS87_Ator08_hmg124h_10.JPG.dat	755.5	66.5	1.0	0.10	0.016	0.097	2.6E-04
YMR192W	YMR192W	UWOPS87_Ator08_hmg124h_10.JPG.dat	741.0	16.3	1.0	0.03	0.030	0.027	6.1E-06
YMR193C-.	YMR193C-A	UWOPS87_Ator08_hmg124h_10.JPG.dat	624.3	32.3	0.9	0.04	0.144	0.041	2.2E-05
YMR194W	YMR194W	UWOPS87_Ator08_hmg124h_10.JPG.dat	683.3	29.2	1.0	0.06	0.070	0.060	5.7E-05
YMR195W	YMR195W	UWOPS87_Ator08_hmg124h_10.JPG.dat	716.5	37.9	1.0	0.01	-0.075	0.011	4.0E-05
YMR196W	YMR196W	UWOPS87_Ator08_hmg124h_10.JPG.dat	727.3	21.9	1.0	0.04	-0.151	0.041	2.0E-05
YMR198W	YMR198W	UWOPS87_Ator08_hmg124h_10.JPG.dat	878.8	89.9	1.1	0.11	0.075	0.114	2.9E-04
YMR199W	YMR199W	UWOPS87_Ator08_hmg124h_10.JPG.dat	783.5	42.9	1.0	0.04	-0.053	0.045	2.6E-05
YMR201C	YMR201C	UWOPS87_Ator08_hmg124h_10.JPG.dat	774.0	46.1	1.0	0.06	-0.051	0.059	6.1E-05
YMR202W	YMR202W	UWOPS87_Ator08_hmg124h_10.JPG.dat	816.8	97.8	1.1	0.12	0.014	0.122	4.0E-04
YMR204C	YMR204C	UWOPS87_Ator08_hmg124h_10.JPG.dat	738.8	52.6	1.0	0.07	-0.036	0.069	9.4E-05
YMR205C	YMR205C	UWOPS87_Ator08_hmg124h_10.JPG.dat	614.8	42.7	0.8	0.06	-0.007	0.063	1.2E-04
YMR206W	YMR206W	UWOPS87_Ator08_hmg124h_10.JPG.dat	700.5	53.1	0.9	0.03	0.021	0.027	2.9E-04
YMR207C	YMR207C	UWOPS87_Ator08_hmg124h_10.JPG.dat	783.3	75.5	1.1	0.11	0.049	0.108	2.4E-04
YMR209C	YMR209C	UWOPS87_Ator08_hmg124h_10.JPG.dat	724.0	64.6	1.0	0.08	-0.002	0.080	1.4E-04
YMR210W	YMR210W	UWOPS87_Ator08_hmg124h_10.JPG.dat	765.5	74.4	1.0	0.10	0.008	0.105	3.3E-04
YMR214W	YMR214W	UWOPS87_Ator08_hmg124h_10.JPG.dat	1007.0	63.8	1.2	0.03	0.240	0.027	1.6E-04
YMR215W	YMR215W	UWOPS87_Ator08_hmg124h_10.JPG.dat	923.8	87.3	1.1	0.11	0.077	0.113	2.8E-04
YMR216C	YMR216C	UWOPS87_Ator08_hmg124h_10.JPG.dat	1024.3	33.8	1.2	0.04	0.130	0.038	8.1E-06
YMR219W	YMR219W	UWOPS87_Ator08_hmg124h_10.JPG.dat	886.0	32.2	1.1	0.04	0.070	0.044	1.9E-05
YMR221C	YMR221C	UWOPS87_Ator08_hmg124h_10.JPG.dat	720.5	73.7	0.9	0.10	-0.020	0.096	3.0E-04
YMR222C	YMR222C	UWOPS87_Ator08_hmg124h_10.JPG.dat	837.8	40.8	1.1	0.05	0.079	0.052	2.9E-05
YMR223W	YMR223W	UWOPS87_Ator08_hmg124h_10.JPG.dat	843.8	31.7	1.1	0.05	0.142	0.046	1.9E-05
YMR224C	YMR224C	UWOPS87_Ator08_hmg124h_10.JPG.dat	718.8	58.3	1.0	0.09	-0.021	0.085	1.9E-04
YMR225C	YMR225C	UWOPS87_Ator08_hmg124h_10.JPG.dat	733.3	77.3	1.0	0.11	0.055	0.106	3.2E-04
YMR226C	YMR226C	UWOPS87_Ator08_hmg124h_10.JPG.dat	818.3	56.9	1.1	0.01	-0.005	0.010	2.6E-05
YMR230W	YMR230W	UWOPS87_Ator08_hmg124h_10.JPG.dat	704.8	91.7	0.9	0.03	0.149	0.032	4.1E-04
YMR232W	YMR232W	UWOPS87_Ator08_hmg124h_10.JPG.dat	857.0	68.4	1.1	0.07	0.049	0.069	7.4E-05
YMR233W	YMR233W	UWOPS87_Ator08_hmg124h_10.JPG.dat	820.5	113.3	1.0	0.13	0.160	0.129	6.0E-04

YMR234W	YMR234W	UWOPS87_Ator08_hmg124h_10.JPG.dat	901.3	57.3	1.1	0.08	-0.062	0.077	9.1E-05
YMR237W	YMR237W	UWOPS87_Ator08_hmg124h_10.JPG.dat	795.0	56.9	1.0	0.03	-0.054	0.028	2.9E-04
YMR238W	YMR238W	UWOPS87_Ator08_hmg124h_10.JPG.dat	817.0	41.9	1.1	0.04	-0.035	0.044	1.8E-05
YMR241W	YMR241W	UWOPS87_Ator08_hmg124h_10.JPG.dat	747.0	41.0	1.0	0.04	0.024	0.041	2.0E-05
YMR243C	YMR243C	UWOPS87_Ator08_hmg124h_10.JPG.dat	490.3	340.0	0.9	0.16	0.293	0.162	1.1E-02
YMR244C-.	YMR244C-A	UWOPS87_Ator08_hmg124h_10.JPG.dat	778.8	32.3	1.0	0.00	0.103	0.004	5.5E-06
YOR202W	YOR202W	UWOPS87_Ator08_hmg124h_10.JPG.dat	762.5	192.9	1.0	0.21	-0.035	0.213	1.4E-183
1	1	UWOPS87_Ator08_hmg124h_11.JPG.dat	994.3	91.5	1.0	0.02	0.065	0.021	1.5E-04
2	2	UWOPS87_Ator08_hmg124h_11.JPG.dat	835.5	106.6	1.0	0.09	0.046	0.087	1.7E-04
3	3	UWOPS87_Ator08_hmg124h_11.JPG.dat	814.8	85.5	1.0	0.06	-0.104	0.060	6.2E-05
4	4	UWOPS87_Ator08_hmg124h_11.JPG.dat	761.0	109.9	1.0	0.10	0.004	0.099	3.1E-04
YJR109C	YJR109C	UWOPS87_Ator08_hmg124h_11.JPG.dat	759.8	132.3	0.9	0.15	-0.161	0.150	1.4E-03
YMR244W	YMR244W	UWOPS87_Ator08_hmg124h_11.JPG.dat	731.0	45.3	1.0	0.06	0.026	0.060	6.0E-05
YMR245W	YMR245W	UWOPS87_Ator08_hmg124h_11.JPG.dat	814.3	7.6	1.1	0.01	0.023	0.008	1.2E-07
YMR246W	YMR246W	UWOPS87_Ator08_hmg124h_11.JPG.dat	833.3	123.6	1.1	0.16	0.138	0.162	9.3E-04
YMR247C	YMR247C	UWOPS87_Ator08_hmg124h_11.JPG.dat	908.8	62.6	1.1	0.07	0.044	0.074	7.9E-05
YMR250W	YMR250W	UWOPS87_Ator08_hmg124h_11.JPG.dat	844.3	77.2	1.1	0.09	0.201	0.085	1.4E-04
YMR251W	YMR251W	UWOPS87_Ator08_hmg124h_11.JPG.dat	785.0	27.5	0.9	0.03	-0.116	0.028	7.1E-06
YMR251W	YMR251W-A	UWOPS87_Ator08_hmg124h_11.JPG.dat	814.3	42.8	1.0	0.04	0.043	0.044	2.6E-05
YMR252C	YMR252C	UWOPS87_Ator08_hmg124h_11.JPG.dat	793.0	26.3	1.0	0.03	-0.082	0.030	8.1E-06
YMR253C	YMR253C	UWOPS87_Ator08_hmg124h_11.JPG.dat	772.3	50.5	0.9	0.06	-0.085	0.059	7.2E-05
YMR254C	YMR254C	UWOPS87_Ator08_hmg124h_11.JPG.dat	797.0	68.5	1.0	0.08	-0.013	0.083	1.5E-04
YMR255W	YMR255W	UWOPS87_Ator08_hmg124h_11.JPG.dat	659.5	40.6	0.9	0.05	0.005	0.053	5.8E-05
YMR256C	YMR256C	UWOPS87_Ator08_hmg124h_11.JPG.dat	243.8	243.3	0.3	0.33	-0.026	0.329	1.4E-01
YMR258C	YMR258C	UWOPS87_Ator08_hmg124h_11.JPG.dat	690.0	26.7	0.9	0.01	-0.212	0.011	5.0E-05
YMR259C	YMR259C	UWOPS87_Ator08_hmg124h_11.JPG.dat	785.0	29.7	1.0	0.04	0.082	0.039	1.8E-05
YMR261C	YMR261C	UWOPS87_Ator08_hmg124h_11.JPG.dat	842.5	68.0	1.0	0.08	-0.050	0.078	1.4E-04
YMR262W	YMR262W	UWOPS87_Ator08_hmg124h_11.JPG.dat	750.5	17.9	0.9	0.02	-0.109	0.021	3.5E-06
YMR263W	YMR263W	UWOPS87_Ator08_hmg124h_11.JPG.dat	1088.3	14.4	1.2	0.02	0.094	0.021	1.3E-06
YMR264W	YMR264W	UWOPS87_Ator08_hmg124h_11.JPG.dat	898.0	66.6	1.0	0.07	0.022	0.074	1.1E-04
YMR265C	YMR265C	UWOPS87_Ator08_hmg124h_11.JPG.dat	931.8	59.0	1.0	0.01	0.078	0.007	1.4E-05
YMR266W	YMR266W	UWOPS87_Ator08_hmg124h_11.JPG.dat	866.0	19.3	1.0	0.01	0.002	0.006	1.2E-05
YMR269W	YMR269W	UWOPS87_Ator08_hmg124h_11.JPG.dat	825.3	58.1	1.0	0.01	0.049	0.012	4.7E-05
YMR271C	YMR271C	UWOPS87_Ator08_hmg124h_11.JPG.dat	751.0	54.9	1.1	0.02	0.090	0.015	6.9E-05
YMR272C	YMR272C	UWOPS87_Ator08_hmg124h_11.JPG.dat	764.3	13.2	1.0	0.01	0.046	0.011	3.5E-07
YMR273C	YMR273C	UWOPS87_Ator08_hmg124h_11.JPG.dat	801.5	69.6	1.0	0.09	0.015	0.090	1.9E-04
YMR274C	YMR274C	UWOPS87_Ator08_hmg124h_11.JPG.dat	747.8	33.9	0.9	0.04	-0.132	0.039	2.4E-05
YMR275C	YMR275C	UWOPS87_Ator08_hmg124h_11.JPG.dat	897.0	50.0	1.1	0.02	0.128	0.018	9.9E-05
YMR276W	YMR276W	UWOPS87_Ator08_hmg124h_11.JPG.dat	832.3	48.5	1.0	0.06	0.043	0.060	6.4E-05
YMR278W	YMR278W	UWOPS87_Ator08_hmg124h_11.JPG.dat	837.5	36.9	1.0	0.04	-0.101	0.040	2.0E-05
YMR279C	YMR279C	UWOPS87_Ator08_hmg124h_11.JPG.dat	878.3	70.7	1.0	0.02	0.115	0.019	1.1E-04
YMR280C	YMR280C	UWOPS87_Ator08_hmg124h_11.JPG.dat	600.5	388.9	0.9	0.03	-0.149	0.030	3.4E-04
YMR282C	YMR282C	UWOPS87_Ator08_hmg124h_11.JPG.dat	209.0	250.2	0.2	0.29	-0.243	0.290	1.9E-01
YMR283C	YMR283C	UWOPS87_Ator08_hmg124h_11.JPG.dat	771.5	51.9	1.0	0.02	0.125	0.023	1.7E-04
YMR284W	YMR284W	UWOPS87_Ator08_hmg124h_11.JPG.dat	763.3	61.0	1.1	0.08	0.010	0.076	1.0E-04
YMR285C	YMR285C	UWOPS87_Ator08_hmg124h_11.JPG.dat	769.8	51.3	1.0	0.05	0.064	0.048	3.0E-05
YMR289W	YMR289W	UWOPS87_Ator08_hmg124h_11.JPG.dat	852.8	54.5	1.0	0.05	-0.065	0.051	3.4E-05
YMR291W	YMR291W	UWOPS87_Ator08_hmg124h_11.JPG.dat	868.5	64.3	1.0	0.06	0.090	0.060	6.1E-05
YMR292W	YMR292W	UWOPS87_Ator08_hmg124h_11.JPG.dat	957.5	75.2	1.1	0.09	0.108	0.091	1.7E-04
YMR294W	YMR294W	UWOPS87_Ator08_hmg124h_11.JPG.dat	868.5	26.6	1.0	0.04	0.055	0.038	1.5E-05
YMR294W	YMR294W-A	UWOPS87_Ator08_hmg124h_11.JPG.dat	959.3	40.0	1.1	0.02	0.043	0.017	8.1E-05
YMR295C	YMR295C	UWOPS87_Ator08_hmg124h_11.JPG.dat	835.5	70.9	0.9	0.01	-0.109	0.013	7.0E-05
YMR297W	YMR297W	UWOPS87_Ator08_hmg124h_11.JPG.dat	838.8	28.7	1.0	0.03	-0.011	0.034	1.2E-05
YMR299C	YMR299C	UWOPS87_Ator08_hmg124h_11.JPG.dat	292.8	245.1	0.3	0.29	-0.097	0.285	9.6E-02
YMR300C	YMR300C	UWOPS87_Ator08_hmg124h_11.JPG.dat	736.5	81.1	0.9	0.10	0.046	0.098	3.0E-04
YMR302C	YMR302C	UWOPS87_Ator08_hmg124h_11.JPG.dat	702.5	10.6	1.0	0.01	0.076	0.006	1.1E-05
YMR303C	YMR303C	UWOPS87_Ator08_hmg124h_11.JPG.dat	743.5	70.1	1.0	0.08	-0.031	0.077	1.4E-04
YMR304C-.	YMR304C-A	UWOPS87_Ator08_hmg124h_11.JPG.dat	815.0	19.4	1.0	0.03	-0.052	0.031	8.8E-06
YMR304W	YMR304W	UWOPS87_Ator08_hmg124h_11.JPG.dat	961.0	60.6	1.1	0.06	-0.038	0.064	5.4E-05
YMR305C	YMR305C	UWOPS87_Ator08_hmg124h_11.JPG.dat	891.0	83.1	1.0	0.10	0.049	0.102	2.9E-04
YMR306C-.	YMR306C-A	UWOPS87_Ator08_hmg124h_11.JPG.dat	876.8	25.4	1.0	0.00	-0.103	0.003	3.5E-06
YMR306W	YMR306W	UWOPS87_Ator08_hmg124h_11.JPG.dat	860.0	44.7	1.0	0.05	0.023	0.051	3.9E-05
YMR307W	YMR307W	UWOPS87_Ator08_hmg124h_11.JPG.dat	865.8	10.0	1.0	0.02	-0.002	0.019	1.9E-06
YMR310C	YMR310C	UWOPS87_Ator08_hmg124h_11.JPG.dat	850.0	28.4	1.0	0.01	0.012	0.007	1.9E-05
YMR311C	YMR311C	UWOPS87_Ator08_hmg124h_11.JPG.dat	845.0	51.4	1.0	0.06	-0.033	0.060	5.8E-05
YMR312W	YMR312W	UWOPS87_Ator08_hmg124h_11.JPG.dat	737.5	50.6	1.0	0.01	0.028	0.012	4.7E-05
YMR313C	YMR313C	UWOPS87_Ator08_hmg124h_11.JPG.dat	765.8	34.7	1.1	0.05	0.079	0.053	3.4E-05
YMR315W	YMR315W	UWOPS87_Ator08_hmg124h_11.JPG.dat	771.3	25.0	1.0	0.03	-0.058	0.034	1.2E-05
YMR316C-.	YMR316C-A	UWOPS87_Ator08_hmg124h_11.JPG.dat	914.3	44.8	1.1	0.01	-0.077	0.011	3.3E-05
YMR316C-	YMR316C-B	UWOPS87_Ator08_hmg124h_11.JPG.dat	998.3	69.2	1.1	0.07	0.174	0.073	7.6E-05

YMR316W	YMR316W	UWOPS87_Ator08_hmg124h_11.JPG.dat	913.0	93.4	1.0	0.11	0.039	0.113	3.7E-04
YMR317W	YMR317W	UWOPS87_Ator08_hmg124h_11.JPG.dat	903.3	30.0	1.0	0.04	-0.025	0.042	1.8E-05
YMR318C	YMR318C	UWOPS87_Ator08_hmg124h_11.JPG.dat	924.3	1.7	1.0	0.01	-0.089	0.011	3.4E-07
YMR319C	YMR319C	UWOPS87_Ator08_hmg124h_11.JPG.dat	911.8	11.2	1.0	0.02	0.009	0.023	3.0E-06
YMR320W	YMR320W	UWOPS87_Ator08_hmg124h_11.JPG.dat	906.0	35.1	1.1	0.04	0.123	0.038	1.3E-05
YMR322C	YMR322C	UWOPS87_Ator08_hmg124h_11.JPG.dat	866.3	28.0	1.0	0.01	0.016	0.008	2.1E-05
YMR326C	YMR326C	UWOPS87_Ator08_hmg124h_11.JPG.dat	759.8	35.4	1.0	0.05	0.028	0.047	2.8E-05
YNL001W	YNL001W	UWOPS87_Ator08_hmg124h_11.JPG.dat	862.3	37.7	1.0	0.03	0.098	0.033	1.0E-05
YNL003C	YNL003C	UWOPS87_Ator08_hmg124h_11.JPG.dat	738.3	93.0	0.9	0.11	0.190	0.106	4.1E-04
YNL004W	YNL004W	UWOPS87_Ator08_hmg124h_11.JPG.dat	718.3	54.7	0.9	0.01	0.008	0.009	3.3E-05
YNL008C	YNL008C	UWOPS87_Ator08_hmg124h_11.JPG.dat	886.3	62.7	1.1	0.02	-0.060	0.022	1.3E-04
YNL009W	YNL009W	UWOPS87_Ator08_hmg124h_11.JPG.dat	736.8	54.6	0.9	0.07	-0.032	0.068	1.2E-04
YNL010W	YNL010W	UWOPS87_Ator08_hmg124h_11.JPG.dat	851.0	14.9	1.1	0.02	0.106	0.017	1.0E-06
YNL012W	YNL012W	UWOPS87_Ator08_hmg124h_11.JPG.dat	895.5	68.0	1.1	0.08	0.117	0.083	1.2E-04
YNL013C	YNL013C	UWOPS87_Ator08_hmg124h_11.JPG.dat	911.5	55.8	1.1	0.02	0.031	0.020	1.1E-04
YNL015W	YNL015W	UWOPS87_Ator08_hmg124h_11.JPG.dat	801.5	37.8	1.0	0.04	0.029	0.044	2.3E-05
YNL016W	YNL016W	UWOPS87_Ator08_hmg124h_11.JPG.dat	728.3	32.6	0.9	0.04	-0.053	0.042	2.5E-05
YNL020C	YNL020C	UWOPS87_Ator08_hmg124h_11.JPG.dat	688.3	39.9	0.9	0.05	-0.073	0.052	4.7E-05
YNL021W	YNL021W	UWOPS87_Ator08_hmg124h_11.JPG.dat	715.0	22.2	1.0	0.02	-0.055	0.020	2.3E-06
YNL022C	YNL022C	UWOPS87_Ator08_hmg124h_11.JPG.dat	634.5	40.1	0.9	0.01	-0.072	0.009	3.0E-05
YNL023C	YNL023C	UWOPS87_Ator08_hmg124h_11.JPG.dat	794.8	32.9	1.1	0.04	0.087	0.045	2.0E-05
YNL024C	YNL024C	UWOPS87_Ator08_hmg124h_11.JPG.dat	719.5	16.3	0.9	0.02	-0.023	0.022	3.7E-06
YNL027W	YNL027W	UWOPS87_Ator08_hmg124h_11.JPG.dat	958.8	129.1	1.2	0.15	0.174	0.150	5.5E-04
YNL028W	YNL028W	UWOPS87_Ator08_hmg124h_11.JPG.dat	778.0	70.1	1.0	0.08	0.020	0.076	1.3E-04
YNL029C	YNL029C	UWOPS87_Ator08_hmg124h_11.JPG.dat	837.0	24.2	1.0	0.04	0.036	0.037	1.3E-05
YNL030W	YNL030W	UWOPS87_Ator08_hmg124h_11.JPG.dat	934.5	32.2	1.1	0.04	-0.062	0.045	1.8E-05
YNL031C	YNL031C	UWOPS87_Ator08_hmg124h_11.JPG.dat	860.5	38.2	1.0	0.04	-0.109	0.044	2.2E-05
YNL032W	YNL032W	UWOPS87_Ator08_hmg124h_11.JPG.dat	818.0	18.3	1.0	0.03	0.028	0.029	7.7E-06
YNL034W	YNL034W	UWOPS87_Ator08_hmg124h_11.JPG.dat	834.8	98.7	1.0	0.12	-0.054	0.121	4.7E-04
YNL035C	YNL035C	UWOPS87_Ator08_hmg124h_11.JPG.dat	888.8	52.3	1.1	0.07	0.035	0.066	5.5E-05
YNL037C	YNL037C	UWOPS87_Ator08_hmg124h_11.JPG.dat	739.5	48.8	1.0	0.07	-0.063	0.067	8.3E-05
YNL040W	YNL040W	UWOPS87_Ator08_hmg124h_11.JPG.dat	776.5	31.4	1.0	0.04	0.095	0.041	1.8E-05
YNL041C	YNL041C	UWOPS87_Ator08_hmg124h_11.JPG.dat	911.0	124.0	1.1	0.16	0.102	0.165	8.1E-04
YNL043C	YNL043C	UWOPS87_Ator08_hmg124h_11.JPG.dat	974.8	52.7	1.1	0.07	0.020	0.069	6.2E-05
YNL044W	YNL044W	UWOPS87_Ator08_hmg124h_11.JPG.dat	866.5	61.1	1.0	0.07	-0.103	0.073	1.0E-04
YNL045W	YNL045W	UWOPS87_Ator08_hmg124h_11.JPG.dat	814.0	21.6	0.9	0.03	0.015	0.033	1.1E-05
YNL046W	YNL046W	UWOPS87_Ator08_hmg124h_11.JPG.dat	897.8	31.9	1.0	0.01	0.018	0.013	5.9E-05
YNL049C	YNL049C	UWOPS87_Ator08_hmg124h_11.JPG.dat	968.8	64.8	1.1	0.03	0.069	0.027	1.9E-04
YNL050C	YNL050C	UWOPS87_Ator08_hmg124h_11.JPG.dat	899.5	103.3	1.0	0.12	0.033	0.116	4.2E-04
YNL051W	YNL051W	UWOPS87_Ator08_hmg124h_11.JPG.dat	855.3	37.8	1.0	0.04	-0.199	0.043	2.2E-05
YNL052W	YNL052W	UWOPS87_Ator08_hmg124h_11.JPG.dat	235.8	375.3	0.1	0.11	-0.377	0.112	4.2E-01
YNL054W	YNL054W	UWOPS87_Ator08_hmg124h_11.JPG.dat	739.8	28.5	1.0	0.02	0.118	0.016	7.7E-05
YNL056W	YNL056W	UWOPS87_Ator08_hmg124h_11.JPG.dat	802.0	92.4	1.0	0.12	-0.113	0.115	3.8E-04
YNL057W	YNL057W	UWOPS87_Ator08_hmg124h_11.JPG.dat	897.5	153.0	1.1	0.18	0.087	0.184	1.3E-03
YNL058C	YNL058C	UWOPS87_Ator08_hmg124h_11.JPG.dat	855.5	67.8	1.0	0.08	-0.010	0.082	1.6E-04
YNL063W	YNL063W	UWOPS87_Ator08_hmg124h_11.JPG.dat	873.0	66.1	1.0	0.08	-0.022	0.077	1.2E-04
YNL064C	YNL064C	UWOPS87_Ator08_hmg124h_11.JPG.dat	948.8	28.4	1.1	0.01	0.000	0.007	1.4E-05
YNL065W	YNL065W	UWOPS87_Ator08_hmg124h_11.JPG.dat	859.5	14.3	1.0	0.02	-0.086	0.020	2.2E-06
YNL066W	YNL066W	UWOPS87_Ator08_hmg124h_11.JPG.dat	931.5	122.7	1.1	0.14	0.021	0.139	6.1E-04
YNL067W	YNL067W	UWOPS87_Ator08_hmg124h_11.JPG.dat	956.5	54.2	1.2	0.08	0.218	0.079	8.7E-05
YNL068C	YNL068C	UWOPS87_Ator08_hmg124h_11.JPG.dat	900.8	20.0	1.2	0.03	0.014	0.026	3.2E-06
YNL069C	YNL069C	UWOPS87_Ator08_hmg124h_11.JPG.dat	754.8	28.4	1.0	0.03	-0.133	0.034	1.1E-05
YNL070W	YNL070W	UWOPS87_Ator08_hmg124h_11.JPG.dat	783.0	61.2	1.0	0.09	0.001	0.086	1.9E-04
YNL071W	YNL071W	UWOPS87_Ator08_hmg124h_11.JPG.dat	961.0	58.6	1.1	0.06	-0.177	0.063	5.1E-05
YNL072W	YNL072W	UWOPS87_Ator08_hmg124h_11.JPG.dat	1022.3	117.7	1.1	0.12	0.014	0.115	2.9E-04
YNL074C	YNL074C	UWOPS87_Ator08_hmg124h_11.JPG.dat	899.8	32.5	1.0	0.05	-0.019	0.046	2.6E-05
YNL076W	YNL076W	UWOPS87_Ator08_hmg124h_11.JPG.dat	1003.3	49.1	1.1	0.06	0.041	0.060	4.2E-05
YNL077W	YNL077W	UWOPS87_Ator08_hmg124h_11.JPG.dat	906.8	15.8	1.0	0.01	0.055	0.013	6.0E-07
YNL078W	YNL078W	UWOPS87_Ator08_hmg124h_11.JPG.dat	856.5	36.1	1.0	0.03	-0.059	0.035	1.3E-05
YNL079C	YNL079C	UWOPS87_Ator08_hmg124h_11.JPG.dat	582.3	135.5	0.7	0.16	-0.228	0.158	3.5E-03
YNL080C	YNL080C	UWOPS87_Ator08_hmg124h_11.JPG.dat	686.5	19.3	0.8	0.03	-0.317	0.031	1.5E-05
YNL081C	YNL081C	UWOPS87_Ator08_hmg124h_11.JPG.dat	869.5	46.6	1.1	0.07	0.054	0.070	6.6E-05
YNL082W	YNL082W	UWOPS87_Ator08_hmg124h_11.JPG.dat	785.0	54.6	1.1	0.07	0.022	0.074	9.4E-05
YNL083W	YNL083W	UWOPS87_Ator08_hmg124h_11.JPG.dat	913.8	125.0	1.1	0.17	0.216	0.166	8.7E-04
YNL085W	YNL085W	UWOPS87_Ator08_hmg124h_11.JPG.dat	902.8	79.0	1.0	0.09	0.162	0.087	1.6E-04
YNL087W	YNL087W	UWOPS87_Ator08_hmg124h_11.JPG.dat	946.3	87.5	1.0	0.09	-0.041	0.088	1.6E-04
YNL089C	YNL089C	UWOPS87_Ator08_hmg124h_11.JPG.dat	855.0	43.0	1.0	0.05	-0.141	0.050	3.6E-05
YNL090W	YNL090W	UWOPS87_Ator08_hmg124h_11.JPG.dat	848.5	36.6	1.0	0.04	-0.003	0.041	2.0E-05
YNL091W	YNL091W	UWOPS87_Ator08_hmg124h_11.JPG.dat	850.0	56.9	1.0	0.06	0.034	0.060	6.6E-05
YNL092W	YNL092W	UWOPS87_Ator08_hmg124h_11.JPG.dat	870.0	22.1	1.0	0.02	0.004	0.025	4.2E-06

YNL093W	YNL093W	UWOPS87_Ator08_hmg124h_11.JPG.dat 928.5	54.9	1.1	0.07	0.006	0.066	6.4E-05
YNL094W	YNL094W	UWOPS87_Ator08_hmg124h_11.JPG.dat 697.0	91.6	0.9	0.12	0.115	0.115	6.4E-04
YNL095C	YNL095C	UWOPS87_Ator08_hmg124h_11.JPG.dat 740.3	14.5	1.0	0.02	-0.098	0.016	1.1E-06
YNL097C	YNL097C	UWOPS87_Ator08_hmg124h_11.JPG.dat 863.5	42.1	1.1	0.07	0.067	0.069	6.7E-05
YNL098C	YNL098C	UWOPS87_Ator08_hmg124h_11.JPG.dat 824.3	38.0	1.0	0.04	-0.105	0.042	2.1E-05
YNL099C	YNL099C	UWOPS87_Ator08_hmg124h_11.JPG.dat 785.5	157.2	0.9	0.18	0.001	0.179	2.1E-03
YNL100W	YNL100W	UWOPS87_Ator08_hmg124h_11.JPG.dat 880.5	59.4	1.0	0.07	-0.059	0.067	9.3E-05
YNL101W	YNL101W	UWOPS87_Ator08_hmg124h_11.JPG.dat 960.5	18.5	1.1	0.03	0.037	0.032	7.1E-06
YNL104C	YNL104C	UWOPS87_Ator08_hmg124h_11.JPG.dat 864.3	28.2	1.0	0.03	-0.189	0.028	6.6E-06
YNL105W	YNL105W	UWOPS87_Ator08_hmg124h_11.JPG.dat 876.3	22.8	1.0	0.02	-0.092	0.021	2.6E-06
YNL106C	YNL106C	UWOPS87_Ator08_hmg124h_11.JPG.dat 939.5	59.9	1.1	0.07	0.182	0.066	6.5E-05
YNL107W	YNL107W	UWOPS87_Ator08_hmg124h_11.JPG.dat 805.3	20.0	0.9	0.03	0.026	0.026	5.8E-06
YNL108C	YNL108C	UWOPS87_Ator08_hmg124h_11.JPG.dat 741.3	14.3	0.9	0.03	0.110	0.030	9.6E-06
YNL115C	YNL115C	UWOPS87_Ator08_hmg124h_11.JPG.dat 720.5	64.9	0.9	0.07	-0.041	0.074	1.3E-04
YNL116W	YNL116W	UWOPS87_Ator08_hmg124h_11.JPG.dat 794.3	43.7	1.0	0.05	0.059	0.047	2.9E-05
YNL117W	YNL117W	UWOPS87_Ator08_hmg124h_11.JPG.dat 848.8	10.7	1.1	0.02	-0.098	0.021	2.2E-06
YNL119W	YNL119W	UWOPS87_Ator08_hmg124h_11.JPG.dat 759.8	45.0	1.0	0.06	0.025	0.057	6.0E-05
YNL120C	YNL120C	UWOPS87_Ator08_hmg124h_11.JPG.dat 768.8	30.1	0.9	0.04	-0.055	0.036	1.6E-05
YNL121C	YNL121C	UWOPS87_Ator08_hmg124h_11.JPG.dat 866.0	49.0	1.1	0.06	0.053	0.057	4.3E-05
YNL122C	YNL122C	UWOPS87_Ator08_hmg124h_11.JPG.dat 829.5	20.5	1.0	0.03	0.106	0.031	8.1E-06
YNL123W	YNL123W	UWOPS87_Ator08_hmg124h_11.JPG.dat 806.8	41.7	1.0	0.01	0.032	0.010	3.4E-05
YNL125C	YNL125C	UWOPS87_Ator08_hmg124h_11.JPG.dat 970.0	35.1	1.2	0.04	0.150	0.037	8.6E-06
YNL127W	YNL127W	UWOPS87_Ator08_hmg124h_11.JPG.dat 781.3	109.0	1.0	0.13	-0.099	0.134	6.9E-04
YNL128W	YNL128W	UWOPS87_Ator08_hmg124h_11.JPG.dat 737.8	34.9	1.0	0.04	-0.055	0.038	1.6E-05
YNL129W	YNL129W	UWOPS87_Ator08_hmg124h_11.JPG.dat 771.0	55.4	1.0	0.08	-0.024	0.078	1.2E-04
YNL130C	YNL130C	UWOPS87_Ator08_hmg124h_11.JPG.dat 735.3	64.1	1.0	0.09	-0.066	0.092	2.0E-04
YNL134C	YNL134C	UWOPS87_Ator08_hmg124h_11.JPG.dat 705.0	30.1	1.0	0.04	-0.090	0.039	1.8E-05
YNL135C	YNL135C	UWOPS87_Ator08_hmg124h_11.JPG.dat 746.3	52.2	1.0	0.08	-0.001	0.076	1.2E-04
YNL136W	YNL136W	UWOPS87_Ator08_hmg124h_11.JPG.dat 739.0	38.6	1.0	0.05	0.012	0.048	3.4E-05
YNL140C	YNL140C	UWOPS87_Ator08_hmg124h_11.JPG.dat 787.8	61.5	1.1	0.01	0.062	0.014	5.9E-05
YNL141W	YNL141W	UWOPS87_Ator08_hmg124h_11.JPG.dat 833.8	20.9	1.0	0.03	0.074	0.029	5.9E-06
YNL142W	YNL142W	UWOPS87_Ator08_hmg124h_11.JPG.dat 804.8	50.6	1.0	0.06	0.026	0.063	6.7E-05
YNL143C	YNL143C	UWOPS87_Ator08_hmg124h_11.JPG.dat 804.5	75.8	1.0	0.10	0.042	0.096	2.3E-04
YNL144C	YNL144C	UWOPS87_Ator08_hmg124h_11.JPG.dat 790.0	35.1	1.0	0.05	0.075	0.047	3.1E-05
YNL145W	YNL145W	UWOPS87_Ator08_hmg124h_11.JPG.dat 705.5	61.8	0.9	0.07	-0.319	0.073	1.4E-04
YNL146W	YNL146W	UWOPS87_Ator08_hmg124h_11.JPG.dat 702.3	88.4	0.9	0.11	-0.089	0.114	4.8E-04
YNL153C	YNL153C	UWOPS87_Ator08_hmg124h_11.JPG.dat 763.0	56.3	1.0	0.07	0.165	0.073	9.3E-05
YNL154C	YNL154C	UWOPS87_Ator08_hmg124h_11.JPG.dat 805.5	142.5	1.1	0.19	0.105	0.192	1.5E-03
YNL155W	YNL155W	UWOPS87_Ator08_hmg124h_11.JPG.dat 726.0	73.1	0.9	0.08	-0.141	0.081	1.8E-04
YNL156C	YNL156C	UWOPS87_Ator08_hmg124h_11.JPG.dat 874.0	103.1	1.1	0.13	0.221	0.128	4.7E-04
YNL157W	YNL157W	UWOPS87_Ator08_hmg124h_11.JPG.dat 753.5	34.1	0.9	0.04	-0.139	0.040	2.3E-05
YNL159C	YNL159C	UWOPS87_Ator08_hmg124h_11.JPG.dat 838.3	102.9	1.0	0.12	0.034	0.118	4.7E-04
YNL162W	YNL162W	UWOPS87_Ator08_hmg124h_11.JPG.dat 758.3	14.5	0.9	0.01	-0.093	0.013	7.9E-07
YNL164C	YNL164C	UWOPS87_Ator08_hmg124h_11.JPG.dat 783.3	58.4	0.9	0.06	-0.027	0.061	7.9E-05
YNL165W	YNL165W	UWOPS87_Ator08_hmg124h_11.JPG.dat 895.8	69.4	1.0	0.03	-0.147	0.028	2.5E-04
YNL166C	YNL166C	UWOPS87_Ator08_hmg124h_11.JPG.dat 774.8	30.9	1.0	0.01	0.051	0.007	2.0E-05
YNL167C	YNL167C	UWOPS87_Ator08_hmg124h_11.JPG.dat 653.5	58.1	0.9	0.02	0.004	0.017	1.3E-04
YNL168C	YNL168C	UWOPS87_Ator08_hmg124h_11.JPG.dat 761.5	84.5	1.0	0.02	-0.232	0.019	1.2E-04
YNL169C	YNL169C	UWOPS87_Ator08_hmg124h_11.JPG.dat 727.3	68.7	1.0	0.08	0.048	0.083	1.8E-04
YNL170W	YNL170W	UWOPS87_Ator08_hmg124h_11.JPG.dat 742.0	74.6	0.9	0.10	-0.024	0.101	3.7E-04
YNL171C	YNL171C	UWOPS87_Ator08_hmg124h_11.JPG.dat 747.0	119.5	1.0	0.01	-0.048	0.010	4.0E-05
YNL173C	YNL173C	UWOPS87_Ator08_hmg124h_11.JPG.dat 817.0	36.7	1.0	0.04	0.166	0.041	2.1E-05
YNL175C	YNL175C	UWOPS87_Ator08_hmg124h_11.JPG.dat 854.3	24.2	1.0	0.03	0.212	0.033	1.1E-05
YNL176C	YNL176C	UWOPS87_Ator08_hmg124h_11.JPG.dat 875.0	25.2	1.0	0.04	0.165	0.035	1.2E-05
YNL179C	YNL179C	UWOPS87_Ator08_hmg124h_11.JPG.dat 936.8	37.5	1.1	0.04	0.057	0.040	1.3E-05
YNL183C	YNL183C	UWOPS87_Ator08_hmg124h_11.JPG.dat 942.3	46.3	1.1	0.02	0.027	0.016	7.4E-05
YNL187W	YNL187W	UWOPS87_Ator08_hmg124h_11.JPG.dat 799.5	78.8	1.0	0.00	0.022	0.005	7.3E-06
YNL190W	YNL190W	UWOPS87_Ator08_hmg124h_11.JPG.dat 736.5	62.2	1.0	0.08	-0.015	0.079	1.2E-04
YNL191W	YNL191W	UWOPS87_Ator08_hmg124h_11.JPG.dat 818.0	22.8	1.1	0.04	0.000	0.039	1.3E-05
YNL192W	YNL192W	UWOPS87_Ator08_hmg124h_11.JPG.dat 730.8	39.5	0.9	0.02	0.001	0.016	1.0E-04
YNL193W	YNL193W	UWOPS87_Ator08_hmg124h_11.JPG.dat 890.0	34.5	1.0	0.05	-0.262	0.046	2.4E-05
YNL194C	YNL194C	UWOPS87_Ator08_hmg124h_11.JPG.dat 773.8	78.3	0.9	0.02	-0.042	0.018	1.2E-04
YNL195C	YNL195C	UWOPS87_Ator08_hmg124h_11.JPG.dat 893.5	30.7	1.0	0.03	0.128	0.033	8.6E-06
YNL196C	YNL196C	UWOPS87_Ator08_hmg124h_11.JPG.dat 876.3	57.0	1.0	0.06	0.110	0.065	7.6E-05
YNL197C	YNL197C	UWOPS87_Ator08_hmg124h_11.JPG.dat 808.8	92.0	1.0	0.04	0.027	0.038	4.9E-04
YNL198C	YNL198C	UWOPS87_Ator08_hmg124h_11.JPG.dat 644.5	27.6	0.8	0.03	-0.129	0.032	2.1E-05
YNL199C	YNL199C	UWOPS87_Ator08_hmg124h_11.JPG.dat 909.5	44.4	1.1	0.05	-0.159	0.046	2.2E-05
YNL200C	YNL200C	UWOPS87_Ator08_hmg124h_11.JPG.dat 724.3	20.2	0.9	0.03	-0.068	0.033	1.2E-05
YNL201C	YNL201C	UWOPS87_Ator08_hmg124h_11.JPG.dat 761.8	16.7	1.1	0.03	0.091	0.035	9.8E-06
YNL202W	YNL202W	UWOPS87_Ator08_hmg124h_11.JPG.dat 755.8	21.1	1.0	0.04	0.059	0.042	2.0E-05

YNL203C	YNL203C	UWOPS87_Ator08_hmg124h_11.JPG.dat	747.0	79.0	0.9	0.03	-0.094	0.026	3.0E-04
YNL204C	YNL204C	UWOPS87_Ator08_hmg124h_11.JPG.dat	858.8	33.7	1.0	0.05	0.018	0.049	3.1E-05
YNL205C	YNL205C	UWOPS87_Ator08_hmg124h_11.JPG.dat	756.0	80.2	0.9	0.10	0.035	0.100	4.0E-04
YNL206C	YNL206C	UWOPS87_Ator08_hmg124h_11.JPG.dat	839.0	59.5	1.0	0.08	-0.053	0.081	1.4E-04
YNL208W	YNL208W	UWOPS87_Ator08_hmg124h_11.JPG.dat	814.8	17.2	0.9	0.01	0.029	0.011	4.4E-07
YNL211C	YNL211C	UWOPS87_Ator08_hmg124h_11.JPG.dat	913.0	40.8	1.1	0.01	-0.112	0.014	5.8E-05
YNL212W	YNL212W	UWOPS87_Ator08_hmg124h_11.JPG.dat	771.3	74.1	0.9	0.09	0.020	0.087	2.3E-04
YNL214W	YNL214W	UWOPS87_Ator08_hmg124h_11.JPG.dat	930.8	89.0	1.1	0.10	0.024	0.098	1.8E-04
YNL215W	YNL215W	UWOPS87_Ator08_hmg124h_11.JPG.dat	775.0	64.3	1.1	0.02	0.078	0.020	1.1E-04
YNL217W	YNL217W	UWOPS87_Ator08_hmg124h_11.JPG.dat	679.8	30.4	0.9	0.00	0.021	0.005	8.7E-06
YNL218W	YNL218W	UWOPS87_Ator08_hmg124h_11.JPG.dat	712.5	32.7	0.9	0.05	-0.099	0.047	3.6E-05
YNL219C	YNL219C	UWOPS87_Ator08_hmg124h_11.JPG.dat	1036.0	35.2	1.3	0.06	-0.007	0.057	2.7E-05
YNL223W	YNL223W	UWOPS87_Ator08_hmg124h_11.JPG.dat	889.5	61.5	1.0	0.08	0.012	0.081	1.4E-04
YNL224C	YNL224C	UWOPS87_Ator08_hmg124h_11.JPG.dat	820.3	46.5	0.9	0.06	0.049	0.058	6.5E-05
YNL226W	YNL226W	UWOPS87_Ator08_hmg124h_11.JPG.dat	887.5	26.4	1.0	0.02	-0.097	0.025	3.7E-06
YNL227C	YNL227C	UWOPS87_Ator08_hmg124h_11.JPG.dat	882.0	30.2	1.0	0.01	0.090	0.007	1.7E-05
YNL228W	YNL228W	UWOPS87_Ator08_hmg124h_11.JPG.dat	843.0	8.5	1.0	0.02	-0.179	0.018	1.9E-06
YNL229C	YNL229C	UWOPS87_Ator08_hmg124h_11.JPG.dat	843.8	52.5	1.0	0.07	0.144	0.066	8.0E-05
YNL230C	YNL230C	UWOPS87_Ator08_hmg124h_11.JPG.dat	804.5	103.6	1.0	0.11	0.046	0.114	4.4E-04
YNL231C	YNL231C	UWOPS87_Ator08_hmg124h_11.JPG.dat	751.5	23.0	1.0	0.04	-0.092	0.037	1.3E-05
YNL233W	YNL233W	UWOPS87_Ator08_hmg124h_11.JPG.dat	685.5	47.9	1.0	0.07	-0.099	0.073	1.2E-04
YNL234W	YNL234W	UWOPS87_Ator08_hmg124h_11.JPG.dat	722.8	24.8	0.9	0.03	0.003	0.035	1.5E-05
YNL235C	YNL235C	UWOPS87_Ator08_hmg124h_11.JPG.dat	836.0	65.9	1.0	0.03	0.161	0.030	3.1E-04
YNL236W	YNL236W	UWOPS87_Ator08_hmg124h_11.JPG.dat	781.8	39.6	0.9	0.04	-0.057	0.041	2.3E-05
YNL237W	YNL237W	UWOPS87_Ator08_hmg124h_11.JPG.dat	916.5	73.6	1.1	0.03	-0.005	0.031	2.6E-04
YNL238W	YNL238W	UWOPS87_Ator08_hmg124h_11.JPG.dat	608.0	50.4	0.7	0.06	-0.396	0.062	1.7E-04
YNL239W	YNL239W	UWOPS87_Ator08_hmg124h_11.JPG.dat	925.5	65.4	1.1	0.08	0.012	0.078	1.0E-04
YNL241C	YNL241C	UWOPS87_Ator08_hmg124h_11.JPG.dat	887.5	32.9	1.1	0.04	0.142	0.037	1.2E-05
YNL242W	YNL242W	UWOPS87_Ator08_hmg124h_11.JPG.dat	821.0	90.1	1.0	0.11	-0.183	0.109	3.5E-04
YNL246W	YNL246W	UWOPS87_Ator08_hmg124h_11.JPG.dat	763.0	48.9	1.0	0.07	0.129	0.067	9.4E-05
YNL249C	YNL249C	UWOPS87_Ator08_hmg124h_11.JPG.dat	755.5	84.9	1.0	0.11	-0.042	0.112	3.5E-04
YNL253W	YNL253W	UWOPS87_Ator08_hmg124h_11.JPG.dat	633.0	25.5	0.9	0.03	-0.094	0.030	1.0E-05
YNL254C	YNL254C	UWOPS87_Ator08_hmg124h_11.JPG.dat	689.3	41.9	1.0	0.02	-0.067	0.015	8.1E-05
YNL255C	YNL255C	UWOPS87_Ator08_hmg124h_11.JPG.dat	712.0	50.7	1.0	0.01	-0.015	0.010	3.5E-05
YNL257C	YNL257C	UWOPS87_Ator08_hmg124h_11.JPG.dat	711.8	22.6	1.0	0.03	-0.066	0.031	9.0E-06
YNL259C	YNL259C	UWOPS87_Ator08_hmg124h_11.JPG.dat	795.0	48.0	1.1	0.01	-0.047	0.005	7.5E-06
YNL265C	YNL265C	UWOPS87_Ator08_hmg124h_11.JPG.dat	805.8	52.8	1.1	0.06	0.105	0.064	6.0E-05
YNL266W	YNL266W	UWOPS87_Ator08_hmg124h_11.JPG.dat	819.3	26.4	1.1	0.04	0.079	0.044	2.0E-05
YNL268W	YNL268W	UWOPS87_Ator08_hmg124h_11.JPG.dat	902.0	38.0	1.1	0.05	0.171	0.047	2.0E-05
YNL270C	YNL270C	UWOPS87_Ator08_hmg124h_11.JPG.dat	839.8	41.4	1.0	0.05	0.020	0.054	3.7E-05
YNL271C	YNL271C	UWOPS87_Ator08_hmg124h_11.JPG.dat	791.5	45.9	0.9	0.01	0.005	0.012	5.6E-05
YNL273W	YNL273W	UWOPS87_Ator08_hmg124h_11.JPG.dat	851.8	49.4	1.1	0.06	0.101	0.059	4.8E-05
YNL274C	YNL274C	UWOPS87_Ator08_hmg124h_11.JPG.dat	723.8	56.4	0.9	0.07	-0.061	0.067	1.1E-04
YNL275W	YNL275W	UWOPS87_Ator08_hmg124h_11.JPG.dat	703.5	18.5	1.0	0.03	0.135	0.026	5.4E-06
YNL277W	YNL277W	UWOPS87_Ator08_hmg124h_11.JPG.dat	773.5	37.1	1.0	0.05	0.122	0.052	3.3E-05
YNL278W	YNL278W	UWOPS87_Ator08_hmg124h_11.JPG.dat	772.3	35.7	1.0	0.01	-0.033	0.011	3.9E-05
YNL279W	YNL279W	UWOPS87_Ator08_hmg124h_11.JPG.dat	829.5	39.4	1.0	0.04	0.019	0.042	1.9E-05
YNL280C	YNL280C	UWOPS87_Ator08_hmg124h_11.JPG.dat	864.0	40.5	1.1	0.05	0.123	0.055	3.9E-05
YNL281W	YNL281W	UWOPS87_Ator08_hmg124h_11.JPG.dat	864.8	17.0	1.0	0.02	-0.037	0.023	2.9E-06
YNL283C	YNL283C	UWOPS87_Ator08_hmg124h_11.JPG.dat	914.3	29.6	1.1	0.04	-0.092	0.042	1.6E-05
YNL285W	YNL285W	UWOPS87_Ator08_hmg124h_11.JPG.dat	916.8	50.3	1.1	0.05	-0.082	0.054	3.5E-05
YNL286W	YNL286W	UWOPS87_Ator08_hmg124h_11.JPG.dat	822.8	74.2	1.0	0.03	0.046	0.025	2.2E-04
YNL288W	YNL288W	UWOPS87_Ator08_hmg124h_11.JPG.dat	984.5	55.4	1.2	0.08	0.014	0.084	9.7E-05
YNL289W	YNL289W	UWOPS87_Ator08_hmg124h_11.JPG.dat	828.8	45.8	1.1	0.06	0.046	0.062	5.5E-05
YNL291C	YNL291C	UWOPS87_Ator08_hmg124h_11.JPG.dat	656.0	121.4	0.8	0.02	-0.243	0.021	2.1E-04
YNL292W	YNL292W	UWOPS87_Ator08_hmg124h_11.JPG.dat	741.0	29.2	1.0	0.04	-0.004	0.040	1.8E-05
YNL293W	YNL293W	UWOPS87_Ator08_hmg124h_11.JPG.dat	762.5	31.1	1.0	0.05	-0.050	0.046	2.9E-05
YNL294C	YNL294C	UWOPS87_Ator08_hmg124h_11.JPG.dat	916.3	25.5	1.1	0.04	-0.169	0.036	1.0E-05
YNL295W	YNL295W	UWOPS87_Ator08_hmg124h_11.JPG.dat	905.8	20.7	1.1	0.03	-0.015	0.028	4.9E-06
YNL296W	YNL296W	UWOPS87_Ator08_hmg124h_11.JPG.dat	686.8	30.7	0.8	0.04	-0.223	0.038	2.9E-05
YNL297C	YNL297C	UWOPS87_Ator08_hmg124h_11.JPG.dat	914.3	13.1	1.1	0.01	0.072	0.015	7.6E-07
YNL298W	YNL298W	UWOPS87_Ator08_hmg124h_11.JPG.dat	777.3	68.4	0.9	0.07	-0.139	0.073	1.5E-04
YNL299W	YNL299W	UWOPS87_Ator08_hmg124h_11.JPG.dat	879.5	65.8	1.0	0.01	0.031	0.010	3.8E-05
YNL300W	YNL300W	UWOPS87_Ator08_hmg124h_11.JPG.dat	863.8	41.9	1.0	0.05	0.062	0.054	3.9E-05
YNL301C	YNL301C	UWOPS87_Ator08_hmg124h_11.JPG.dat	759.3	42.6	1.0	0.07	0.029	0.067	8.5E-05
YNL302C	YNL302C	UWOPS87_Ator08_hmg124h_11.JPG.dat	521.8	161.4	0.7	0.22	0.105	0.222	7.9E-03
YNL303W	YNL303W	UWOPS87_Ator08_hmg124h_11.JPG.dat	813.8	31.1	1.0	0.04	0.073	0.043	2.0E-05
YNL304W	YNL304W	UWOPS87_Ator08_hmg124h_11.JPG.dat	830.0	42.0	1.0	0.06	0.055	0.058	5.4E-05
YNL305C	YNL305C	UWOPS87_Ator08_hmg124h_11.JPG.dat	883.3	37.9	1.0	0.04	0.099	0.043	2.3E-05
YNL307C	YNL307C	UWOPS87_Ator08_hmg124h_11.JPG.dat	814.0	46.2	0.9	0.06	-0.178	0.059	7.0E-05

YNL309W	YNL309W	UWOPS87_Ator08_hmg124h_11.JPG.dat	889.3	40.1	1.0	0.05	-0.024	0.049	3.0E-05
YNL311C	YNL311C	UWOPS87_Ator08_hmg124h_11.JPG.dat	797.5	5.9	0.9	0.01	-0.120	0.007	1.5E-07
YNL314W	YNL314W	UWOPS87_Ator08_hmg124h_11.JPG.dat	925.0	33.6	1.1	0.04	0.006	0.040	1.5E-05
YNL315C	YNL315C	UWOPS87_Ator08_hmg124h_11.JPG.dat	920.5	53.2	1.1	0.06	-0.143	0.061	5.3E-05
YNL316C	YNL316C	UWOPS87_Ator08_hmg124h_11.JPG.dat	0.0	0.0	0.0	0.00	0.000	0.000	
YNL318C	YNL318C	UWOPS87_Ator08_hmg124h_11.JPG.dat	789.5	66.2	1.0	0.09	-0.003	0.089	1.8E-04
YNL319W	YNL319W	UWOPS87_Ator08_hmg124h_11.JPG.dat	723.0	20.1	1.0	0.02	0.045	0.020	2.1E-06
YNL320W	YNL320W	UWOPS87_Ator08_hmg124h_11.JPG.dat	850.8	86.4	1.1	0.12	-0.111	0.116	3.5E-04
YNL321W	YNL321W	UWOPS87_Ator08_hmg124h_11.JPG.dat	892.8	51.9	1.1	0.07	0.007	0.072	8.7E-05
YNL322C	YNL322C	UWOPS87_Ator08_hmg124h_11.JPG.dat	877.8	33.3	1.0	0.03	0.047	0.033	1.0E-05
YNL323W	YNL323W	UWOPS87_Ator08_hmg124h_11.JPG.dat	673.8	31.2	0.8	0.04	-0.108	0.040	3.5E-05
YNL324W	YNL324W	UWOPS87_Ator08_hmg124h_11.JPG.dat	823.5	17.6	1.0	0.01	0.099	0.010	3.3E-07
YNL325C	YNL325C	UWOPS87_Ator08_hmg124h_11.JPG.dat	820.3	15.1	1.0	0.01	-0.114	0.011	4.0E-07
YNL326C	YNL326C	UWOPS87_Ator08_hmg124h_11.JPG.dat	854.5	21.0	1.0	0.03	-0.124	0.030	7.2E-06
YNL327W	YNL327W	UWOPS87_Ator08_hmg124h_11.JPG.dat	989.3	27.3	1.2	0.03	0.004	0.034	6.4E-06
YNL328C	YNL328C	UWOPS87_Ator08_hmg124h_11.JPG.dat	937.0	103.6	1.2	0.12	0.233	0.117	2.6E-04
YNL329C	YNL329C	UWOPS87_Ator08_hmg124h_11.JPG.dat	738.5	71.1	1.0	0.08	-0.007	0.084	1.7E-04
YNL330C	YNL330C	UWOPS87_Ator08_hmg124h_11.JPG.dat	732.8	21.0	1.0	0.01	0.142	0.007	1.8E-05
YNL332W	YNL332W	UWOPS87_Ator08_hmg124h_11.JPG.dat	835.5	35.7	1.0	0.01	-0.065	0.013	4.7E-05
YNL333W	YNL333W	UWOPS87_Ator08_hmg124h_11.JPG.dat	927.0	74.6	1.1	0.09	-0.001	0.088	1.5E-04
YNL334C	YNL334C	UWOPS87_Ator08_hmg124h_11.JPG.dat	896.8	14.9	1.0	0.00	-0.065	0.004	6.4E-06
YNL335W	YNL335W	UWOPS87_Ator08_hmg124h_11.JPG.dat	878.5	67.2	1.0	0.07	0.020	0.073	1.1E-04
YNL336W	YNL336W	UWOPS87_Ator08_hmg124h_11.JPG.dat	653.0	436.0	1.0	0.02	-0.066	0.022	1.7E-04
YNL338W	YNL338W	UWOPS87_Ator08_hmg124h_11.JPG.dat	860.5	45.3	1.0	0.02	0.065	0.021	1.4E-04
YNL339C	YNL339C	UWOPS87_Ator08_hmg124h_11.JPG.dat	618.5	37.8	0.8	0.04	-0.092	0.043	3.7E-05
YNR001C	YNR001C	UWOPS87_Ator08_hmg124h_11.JPG.dat	873.8	63.9	1.0	0.06	0.022	0.063	6.3E-05
YNR002C	YNR002C	UWOPS87_Ator08_hmg124h_11.JPG.dat	846.3	48.2	1.1	0.01	-0.020	0.012	3.8E-05
YNR004W	YNR004W	UWOPS87_Ator08_hmg124h_11.JPG.dat	794.5	43.7	1.1	0.06	0.125	0.055	3.8E-05
YNR005C	YNR005C	UWOPS87_Ator08_hmg124h_11.JPG.dat	861.8	21.0	1.1	0.02	0.031	0.022	2.2E-06
YNR006W	YNR006W	UWOPS87_Ator08_hmg124h_11.JPG.dat	804.8	16.2	1.0	0.02	-0.131	0.022	2.7E-06
YNR007C	YNR007C	UWOPS87_Ator08_hmg124h_11.JPG.dat	916.5	99.2	1.1	0.12	0.128	0.119	3.3E-04
YNR008W	YNR008W	UWOPS87_Ator08_hmg124h_11.JPG.dat	903.8	18.4	1.1	0.02	-0.134	0.017	1.2E-06
YNR009W	YNR009W	UWOPS87_Ator08_hmg124h_11.JPG.dat	841.3	62.5	1.0	0.07	0.152	0.072	1.0E-04
YNR010W	YNR010W	UWOPS87_Ator08_hmg124h_11.JPG.dat	915.3	56.3	1.1	0.06	0.239	0.062	5.0E-05
YNR012W	YNR012W	UWOPS87_Ator08_hmg124h_11.JPG.dat	909.0	20.2	1.1	0.03	-0.075	0.026	4.2E-06
YNR013C	YNR013C	UWOPS87_Ator08_hmg124h_11.JPG.dat	785.3	30.3	0.9	0.03	0.172	0.028	7.4E-06
YNR014W	YNR014W	UWOPS87_Ator08_hmg124h_11.JPG.dat	837.5	117.8	1.0	0.14	-0.107	0.140	7.0E-04
YNR015W	YNR015W	UWOPS87_Ator08_hmg124h_11.JPG.dat	780.5	159.9	1.0	0.20	0.039	0.201	2.1E-03
YNR018W	YNR018W	UWOPS87_Ator08_hmg124h_11.JPG.dat	633.0	47.3	0.9	0.02	-0.082	0.022	2.0E-04
YOR202W	YOR202W	UWOPS87_Ator08_hmg124h_11.JPG.dat	849.8	107.6	1.0	0.08	-0.003	0.076	6.6424802
1	1	UWOPS87_Ator08_hmg124h_12.JPG.dat	1065.3	125.6	1.0	0.00	-0.119	0.004	6.6E-06
2	2	UWOPS87_Ator08_hmg124h_12.JPG.dat	883.0	125.8	0.9	0.09	0.004	0.093	2.7E-04
3	3	UWOPS87_Ator08_hmg124h_12.JPG.dat	879.5	84.7	1.0	0.01	0.063	0.015	7.3E-05
4	4	UWOPS87_Ator08_hmg124h_12.JPG.dat	927.8	107.9	1.1	0.07	0.098	0.068	7.4E-05
YNR019W	YNR019W	UWOPS87_Ator08_hmg124h_12.JPG.dat	640.0	45.7	0.8	0.02	-0.184	0.025	3.0E-04
YNR020C	YNR020C	UWOPS87_Ator08_hmg124h_12.JPG.dat	0.0	0.0	0.0	0.00	0.000	0.000	
YNR021W	YNR021W	UWOPS87_Ator08_hmg124h_12.JPG.dat	0.0	0.0	0.0	0.00	0.000	0.000	
YNR022C	YNR022C	UWOPS87_Ator08_hmg124h_12.JPG.dat	693.8	49.0	0.9	0.02	0.022	0.016	1.1E-04
YNR024W	YNR024W	UWOPS87_Ator08_hmg124h_12.JPG.dat	617.8	55.7	0.9	0.08	-0.029	0.081	2.3E-04
YNR025C	YNR025C	UWOPS87_Ator08_hmg124h_12.JPG.dat	709.5	69.9	1.0	0.09	-0.072	0.095	2.5E-04
YNR027W	YNR027W	UWOPS87_Ator08_hmg124h_12.JPG.dat	719.5	70.3	1.0	0.09	0.132	0.092	2.4E-04
YNR028W	YNR028W	UWOPS87_Ator08_hmg124h_12.JPG.dat	829.3	54.0	1.1	0.07	0.049	0.074	9.0E-05
YNR029C	YNR029C	UWOPS87_Ator08_hmg124h_12.JPG.dat	789.3	65.7	1.0	0.08	0.066	0.080	1.5E-04
YNR030W	YNR030W	UWOPS87_Ator08_hmg124h_12.JPG.dat	971.5	102.1	1.2	0.12	0.013	0.120	2.8E-04
YNR031C	YNR031C	UWOPS87_Ator08_hmg124h_12.JPG.dat	772.5	112.0	1.0	0.14	-0.017	0.136	7.4E-04
YNR032C-/	YNR032C-A	UWOPS87_Ator08_hmg124h_12.JPG.dat	788.5	52.8	1.1	0.07	0.068	0.067	6.7E-05
YNR032W	YNR032W	UWOPS87_Ator08_hmg124h_12.JPG.dat	797.0	37.1	1.1	0.04	-0.099	0.036	9.7E-06
YNR034W	YNR034W	UWOPS87_Ator08_hmg124h_12.JPG.dat	819.5	82.8	1.1	0.10	0.041	0.100	2.0E-04
YNR039C	YNR039C	UWOPS87_Ator08_hmg124h_12.JPG.dat	708.8	62.8	0.9	0.07	0.198	0.075	1.5E-04
YNR040W	YNR040W	UWOPS87_Ator08_hmg124h_12.JPG.dat	758.0	40.7	1.0	0.05	0.088	0.051	3.4E-05
YNR042W	YNR042W	UWOPS87_Ator08_hmg124h_12.JPG.dat	740.8	43.1	1.0	0.06	0.013	0.059	5.5E-05
YNR045W	YNR045W	UWOPS87_Ator08_hmg124h_12.JPG.dat	812.0	72.8	1.1	0.09	-0.049	0.093	1.8E-04
YNR047W	YNR047W	UWOPS87_Ator08_hmg124h_12.JPG.dat	748.3	26.2	0.9	0.03	-0.151	0.030	8.8E-06
YNR048W	YNR048W	UWOPS87_Ator08_hmg124h_12.JPG.dat	891.8	17.5	1.1	0.02	-0.130	0.019	1.4E-06
YNR049C	YNR049C	UWOPS87_Ator08_hmg124h_12.JPG.dat	795.8	64.6	1.0	0.08	0.023	0.078	1.4E-04
YNR050C	YNR050C	UWOPS87_Ator08_hmg124h_12.JPG.dat	178.8	232.7	0.2	0.29	0.226	0.294	2.2E-01
YNR051C	YNR051C	UWOPS87_Ator08_hmg124h_12.JPG.dat	635.8	39.8	0.9	0.07	0.083	0.065	1.1E-04
YNR055C	YNR055C	UWOPS87_Ator08_hmg124h_12.JPG.dat	751.3	90.2	1.0	0.12	-0.001	0.117	4.7E-04
YNR056C	YNR056C	UWOPS87_Ator08_hmg124h_12.JPG.dat	739.5	21.9	0.9	0.02	0.025	0.024	4.5E-06
YNR057C	YNR057C	UWOPS87_Ator08_hmg124h_12.JPG.dat	811.8	31.4	1.0	0.01	-0.057	0.010	3.3E-05

YNR058W	YNR058W	UWOPS87_Ator08_hmg124h_12.JPG.dat	744.5	54.1	1.0	0.07	0.069	0.071	1.0E-04
YNR059W	YNR059W	UWOPS87_Ator08_hmg124h_12.JPG.dat	797.0	34.4	1.1	0.05	-0.099	0.048	2.6E-05
YNR060W	YNR060W	UWOPS87_Ator08_hmg124h_12.JPG.dat	612.3	347.4	1.0	0.08	0.128	0.079	2.0E-03
YNR061C	YNR061C	UWOPS87_Ator08_hmg124h_12.JPG.dat	853.8	26.2	1.1	0.01	-0.006	0.006	1.1E-05
YNR062C	YNR062C	UWOPS87_Ator08_hmg124h_12.JPG.dat	884.8	82.5	1.1	0.11	0.044	0.105	2.6E-04
YNR063W	YNR063W	UWOPS87_Ator08_hmg124h_12.JPG.dat	808.8	15.3	1.0	0.02	-0.059	0.018	1.7E-06
YNR064C	YNR064C	UWOPS87_Ator08_hmg124h_12.JPG.dat	748.3	26.9	1.0	0.04	-0.007	0.039	1.8E-05
YNR065C	YNR065C	UWOPS87_Ator08_hmg124h_12.JPG.dat	774.3	81.6	1.0	0.04	-0.087	0.038	4.7E-04
YNR066C	YNR066C	UWOPS87_Ator08_hmg124h_12.JPG.dat	895.8	33.9	1.1	0.05	0.025	0.051	2.4E-05
YNR067C	YNR067C	UWOPS87_Ator08_hmg124h_12.JPG.dat	792.0	81.8	1.0	0.09	-0.130	0.095	2.6E-04
YNR069C	YNR069C	UWOPS87_Ator08_hmg124h_12.JPG.dat	818.3	18.9	1.0	0.01	0.047	0.015	8.8E-07
YNR071C	YNR071C	UWOPS87_Ator08_hmg124h_12.JPG.dat	769.5	39.3	1.0	0.04	-0.136	0.041	2.1E-05
YNR072W	YNR072W	UWOPS87_Ator08_hmg124h_12.JPG.dat	812.3	40.5	1.0	0.04	0.146	0.044	2.3E-05
YNR073C	YNR073C	UWOPS87_Ator08_hmg124h_12.JPG.dat	781.8	21.7	1.0	0.03	-0.067	0.030	7.8E-06
YNR074C	YNR074C	UWOPS87_Ator08_hmg124h_12.JPG.dat	819.3	72.0	1.0	0.08	-0.018	0.081	1.5E-04
YNR075W	YNR075W	UWOPS87_Ator08_hmg124h_12.JPG.dat	835.5	61.1	1.0	0.07	0.082	0.075	1.2E-04
YOL001W	YOL001W	UWOPS87_Ator08_hmg124h_12.JPG.dat	888.8	28.4	1.1	0.04	0.179	0.038	1.1E-05
YOL002C	YOL002C	UWOPS87_Ator08_hmg124h_12.JPG.dat	797.3	41.2	1.0	0.05	-0.070	0.050	3.0E-05
YOL003C	YOL003C	UWOPS87_Ator08_hmg124h_12.JPG.dat	801.0	103.7	1.1	0.15	-0.066	0.147	6.0E-04
YOL004W	YOL004W	UWOPS87_Ator08_hmg124h_12.JPG.dat	880.8	71.4	1.1	0.09	0.136	0.090	1.4E-04
YOL006C	YOL006C	UWOPS87_Ator08_hmg124h_12.JPG.dat	896.5	40.7	1.1	0.04	-0.024	0.040	1.4E-05
YOL007C	YOL007C	UWOPS87_Ator08_hmg124h_12.JPG.dat	830.3	34.6	1.0	0.04	-0.074	0.037	1.5E-05
YOL008W	YOL008W	UWOPS87_Ator08_hmg124h_12.JPG.dat	840.0	233.6	1.2	0.03	-0.100	0.027	1.7E-04
YOL009C	YOL009C	UWOPS87_Ator08_hmg124h_12.JPG.dat	814.5	29.6	1.0	0.04	0.114	0.038	1.4E-05
YOL011W	YOL011W	UWOPS87_Ator08_hmg124h_12.JPG.dat	739.0	29.4	0.9	0.00	0.002	0.005	8.9E-06
YOL012C	YOL012C	UWOPS87_Ator08_hmg124h_12.JPG.dat	606.0	357.5	0.7	0.44	-0.216	0.437	4.3E-02
YOL013C	YOL013C	UWOPS87_Ator08_hmg124h_12.JPG.dat	810.3	76.1	1.0	0.10	-0.196	0.097	2.7E-04
YOL013W-	YOL013W-A	UWOPS87_Ator08_hmg124h_12.JPG.dat	763.5	117.5	1.0	0.15	-0.093	0.148	1.0E-03
YOL014W	YOL014W	UWOPS87_Ator08_hmg124h_12.JPG.dat	763.3	24.9	1.0	0.00	0.009	0.002	1.4E-06
YOL015W	YOL015W	UWOPS87_Ator08_hmg124h_12.JPG.dat	753.5	59.4	1.1	0.08	-0.069	0.083	1.4E-04
YOL016C	YOL016C	UWOPS87_Ator08_hmg124h_12.JPG.dat	596.5	59.2	0.8	0.07	-0.055	0.073	2.5E-04
YOL017W	YOL017W	UWOPS87_Ator08_hmg124h_12.JPG.dat	814.5	50.8	1.0	0.07	0.043	0.066	8.3E-05
YOL018C	YOL018C	UWOPS87_Ator08_hmg124h_12.JPG.dat	894.5	26.7	1.0	0.03	0.011	0.030	6.2E-06
YOL019W	YOL019W	UWOPS87_Ator08_hmg124h_12.JPG.dat	831.0	35.9	1.0	0.05	0.072	0.045	2.5E-05
YOL020W	YOL020W	UWOPS87_Ator08_hmg124h_12.JPG.dat	727.3	74.1	0.9	0.09	0.019	0.090	2.9E-04
YOL024W	YOL024W	UWOPS87_Ator08_hmg124h_12.JPG.dat	709.8	36.7	0.9	0.04	0.056	0.044	3.6E-05
YOL025W	YOL025W	UWOPS87_Ator08_hmg124h_12.JPG.dat	793.5	51.8	1.0	0.06	-0.046	0.060	6.6E-05
YOL027C	YOL027C	UWOPS87_Ator08_hmg124h_12.JPG.dat	261.5	297.1	0.3	0.36	0.061	0.356	1.8E-01
YOL028C	YOL028C	UWOPS87_Ator08_hmg124h_12.JPG.dat	791.3	31.6	1.0	0.04	0.000	0.038	1.6E-05
YOL029C	YOL029C	UWOPS87_Ator08_hmg124h_12.JPG.dat	755.0	19.4	1.0	0.03	0.051	0.027	6.0E-06
YOL030W	YOL030W	UWOPS87_Ator08_hmg124h_12.JPG.dat	707.3	34.8	0.9	0.01	0.175	0.013	6.6E-05
YOL031C	YOL031C	UWOPS87_Ator08_hmg124h_12.JPG.dat	838.0	68.9	1.0	0.08	0.004	0.084	1.6E-04
YOL032W	YOL032W	UWOPS87_Ator08_hmg124h_12.JPG.dat	817.0	31.1	1.0	0.03	0.000	0.027	6.5E-06
YOL035C	YOL035C	UWOPS87_Ator08_hmg124h_12.JPG.dat	811.5	81.2	0.9	0.09	-0.060	0.090	2.5E-04
YOL036W	YOL036W	UWOPS87_Ator08_hmg124h_12.JPG.dat	871.8	95.4	1.0	0.04	0.110	0.036	4.5E-04
YOL037C	YOL037C	UWOPS87_Ator08_hmg124h_12.JPG.dat	851.0	105.4	1.0	0.12	-0.105	0.116	4.3E-04
YOL039W	YOL039W	UWOPS87_Ator08_hmg124h_12.JPG.dat	902.3	92.6	1.1	0.11	0.187	0.107	2.8E-04
YOL041C	YOL041C	UWOPS87_Ator08_hmg124h_12.JPG.dat	791.8	74.3	0.9	0.08	0.037	0.081	1.7E-04
YOL042W	YOL042W	UWOPS87_Ator08_hmg124h_12.JPG.dat	802.5	46.4	0.9	0.06	0.094	0.058	6.4E-05
YOL043C	YOL043C	UWOPS87_Ator08_hmg124h_12.JPG.dat	839.8	29.3	1.0	0.01	-0.063	0.005	9.2E-06
YOL044W	YOL044W	UWOPS87_Ator08_hmg124h_12.JPG.dat	764.8	33.7	0.9	0.04	-0.009	0.042	2.5E-05
YOL045W	YOL045W	UWOPS87_Ator08_hmg124h_12.JPG.dat	742.5	101.6	0.9	0.03	0.008	0.027	2.8E-04
YOL046C	YOL046C	UWOPS87_Ator08_hmg124h_12.JPG.dat	774.8	55.1	1.1	0.09	-0.115	0.090	1.6E-04
YOL047C	YOL047C	UWOPS87_Ator08_hmg124h_12.JPG.dat	0.0	0.0	0.0	0.00	0.000	0.000	
YOL048C	YOL048C	UWOPS87_Ator08_hmg124h_12.JPG.dat	95.8	112.2	0.1	0.16	0.133	0.156	1.9E-01
YOL049W	YOL049W	UWOPS87_Ator08_hmg124h_12.JPG.dat	730.3	30.0	1.0	0.01	-0.193	0.011	3.9E-05
YOL050C	YOL050C	UWOPS87_Ator08_hmg124h_12.JPG.dat	806.3	41.7	1.1	0.05	0.086	0.051	2.6E-05
YOL052C	YOL052C	UWOPS87_Ator08_hmg124h_12.JPG.dat	760.5	47.1	1.0	0.07	-0.095	0.066	7.4E-05
YOL053C-A	YOL053C-A	UWOPS87_Ator08_hmg124h_12.JPG.dat	644.3	335.9	1.1	0.10	-0.001	0.099	2.9E-03
YOL053W	YOL053W	UWOPS87_Ator08_hmg124h_12.JPG.dat	897.3	18.9	1.1	0.00	0.064	0.001	3.1E-07
YOL054W	YOL054W	UWOPS87_Ator08_hmg124h_12.JPG.dat	747.3	58.8	0.9	0.02	0.012	0.022	1.9E-04
YOL055C	YOL055C	UWOPS87_Ator08_hmg124h_12.JPG.dat	883.0	42.7	1.1	0.06	-0.073	0.057	3.9E-05
YOL056W	YOL056W	UWOPS87_Ator08_hmg124h_12.JPG.dat	665.0	63.3	0.9	0.09	-0.028	0.089	2.7E-04
YOL057W	YOL057W	UWOPS87_Ator08_hmg124h_12.JPG.dat	828.0	160.3	1.1	0.22	0.207	0.215	1.8E-03
YOL058W	YOL058W	UWOPS87_Ator08_hmg124h_12.JPG.dat	99.8	199.5	0.0	0.00	0.000	0.000	
YOL059W	YOL059W	UWOPS87_Ator08_hmg124h_12.JPG.dat	851.8	41.1	1.1	0.06	-0.081	0.062	4.4E-05
YOL060C	YOL060C	UWOPS87_Ator08_hmg124h_12.JPG.dat	778.3	61.3	1.0	0.08	-0.136	0.084	1.5E-04
YOL061W	YOL061W	UWOPS87_Ator08_hmg124h_12.JPG.dat	748.0	37.3	1.0	0.05	0.128	0.052	3.8E-05
YOL062C	YOL062C	UWOPS87_Ator08_hmg124h_12.JPG.dat	701.5	111.1	0.9	0.04	-0.025	0.042	7.9E-04
YOL063C	YOL063C	UWOPS87_Ator08_hmg124h_12.JPG.dat	749.8	58.2	1.0	0.08	-0.069	0.083	1.8E-04

YOL064C	YOL064C	UWOPS87_Ator08_hmg124h_12.JPG.dat	493.0	309.7	0.8	0.03	-0.018	0.030	4.6E-04
YOL065C	YOL065C	UWOPS87_Ator08_hmg124h_12.JPG.dat	730.8	24.6	0.9	0.03	0.121	0.031	1.1E-05
YOL067C	YOL067C	UWOPS87_Ator08_hmg124h_12.JPG.dat	834.0	76.1	1.0	0.09	0.024	0.094	2.1E-04
YOL068C	YOL068C	UWOPS87_Ator08_hmg124h_12.JPG.dat	801.0	36.7	1.1	0.01	-0.191	0.013	4.4E-05
YOL070C	YOL070C	UWOPS87_Ator08_hmg124h_12.JPG.dat	550.8	57.4	0.8	0.07	-0.146	0.070	2.2E-04
YOL071W	YOL071W	UWOPS87_Ator08_hmg124h_12.JPG.dat	835.8	17.2	1.1	0.02	0.163	0.017	1.2E-06
YOL075C	YOL075C	UWOPS87_Ator08_hmg124h_12.JPG.dat	775.8	109.9	1.0	0.14	-0.246	0.138	8.0E-04
YOL079W	YOL079W	UWOPS87_Ator08_hmg124h_12.JPG.dat	770.8	76.2	1.0	0.10	-0.008	0.100	2.7E-04
YOL080C	YOL080C	UWOPS87_Ator08_hmg124h_12.JPG.dat	736.0	80.7	1.0	0.03	-0.133	0.034	3.6E-04
YOL081W	YOL081W	UWOPS87_Ator08_hmg124h_12.JPG.dat	815.5	50.1	1.1	0.06	-0.081	0.064	6.2E-05
YOL082W	YOL082W	UWOPS87_Ator08_hmg124h_12.JPG.dat	761.0	56.9	1.0	0.07	0.067	0.069	1.1E-04
YOL083W	YOL083W	UWOPS87_Ator08_hmg124h_12.JPG.dat	799.5	16.4	1.0	0.02	0.075	0.016	1.2E-06
YOL084W	YOL084W	UWOPS87_Ator08_hmg124h_12.JPG.dat	856.3	39.8	1.1	0.05	-0.090	0.052	3.1E-05
YOL085C	YOL085C	UWOPS87_Ator08_hmg124h_12.JPG.dat	801.3	63.8	1.1	0.02	-0.038	0.021	1.3E-04
YOL087C	YOL087C	UWOPS87_Ator08_hmg124h_12.JPG.dat	675.3	57.7	0.9	0.08	-0.097	0.077	1.6E-04
YOL088C	YOL088C	UWOPS87_Ator08_hmg124h_12.JPG.dat	816.5	97.3	1.1	0.12	0.142	0.115	3.2E-04
YOL089C	YOL089C	UWOPS87_Ator08_hmg124h_12.JPG.dat	685.8	63.4	0.9	0.09	-0.167	0.086	2.7E-04
YOL090W	YOL090W	UWOPS87_Ator08_hmg124h_12.JPG.dat	802.3	25.6	1.0	0.03	-0.075	0.034	1.2E-05
YOL091W	YOL091W	UWOPS87_Ator08_hmg124h_12.JPG.dat	772.0	33.3	1.0	0.03	0.137	0.031	8.9E-06
YOL092W	YOL092W	UWOPS87_Ator08_hmg124h_12.JPG.dat	838.8	57.9	1.1	0.07	0.003	0.068	7.4E-05
YOL093W	YOL093W	UWOPS87_Ator08_hmg124h_12.JPG.dat	763.8	96.7	0.9	0.13	0.066	0.129	6.9E-04
YOL095C	YOL095C	UWOPS87_Ator08_hmg124h_12.JPG.dat	882.3	55.9	1.1	0.06	-0.022	0.065	6.1E-05
YOL098C	YOL098C	UWOPS87_Ator08_hmg124h_12.JPG.dat	855.3	80.0	1.0	0.09	-0.023	0.093	2.0E-04
YOL099C	YOL099C	UWOPS87_Ator08_hmg124h_12.JPG.dat	814.0	61.6	1.0	0.03	-0.119	0.026	2.3E-04
YOL101C	YOL101C	UWOPS87_Ator08_hmg124h_12.JPG.dat	680.5	20.8	0.9	0.03	-0.004	0.033	1.6E-05
YOL103W	YOL103W	UWOPS87_Ator08_hmg124h_12.JPG.dat	594.3	47.2	0.8	0.07	-0.014	0.068	1.5E-04
YOL104C	YOL104C	UWOPS87_Ator08_hmg124h_12.JPG.dat	737.3	53.7	1.0	0.06	0.059	0.057	5.0E-05
YOL105C	YOL105C	UWOPS87_Ator08_hmg124h_12.JPG.dat	706.5	66.9	0.9	0.09	-0.186	0.085	2.5E-04
YOL106W	YOL106W	UWOPS87_Ator08_hmg124h_12.JPG.dat	830.0	89.9	1.0	0.02	0.034	0.024	1.8E-04
YOL107W	YOL107W	UWOPS87_Ator08_hmg124h_12.JPG.dat	737.3	39.7	0.9	0.04	0.019	0.041	2.7E-05
YOL108C	YOL108C	UWOPS87_Ator08_hmg124h_12.JPG.dat	841.0	27.1	1.0	0.03	-0.009	0.031	7.6E-06
YOL109W	YOL109W	UWOPS87_Ator08_hmg124h_12.JPG.dat	867.0	26.5	1.0	0.01	0.042	0.011	4.0E-05
YOL110W	YOL110W	UWOPS87_Ator08_hmg124h_12.JPG.dat	802.8	20.0	1.0	0.02	-0.017	0.024	4.1E-06
YOL111C	YOL111C	UWOPS87_Ator08_hmg124h_12.JPG.dat	923.3	87.8	1.1	0.11	-0.003	0.112	2.7E-04
YOL112W	YOL112W	UWOPS87_Ator08_hmg124h_12.JPG.dat	750.8	72.3	0.9	0.10	0.049	0.096	2.9E-04
YOL113W	YOL113W	UWOPS87_Ator08_hmg124h_12.JPG.dat	611.8	419.5	1.0	0.15	-0.015	0.146	6.5E-03
YOL114C	YOL114C	UWOPS87_Ator08_hmg124h_12.JPG.dat	783.3	16.6	1.1	0.02	-0.213	0.024	2.9E-06
YOL115W	YOL115W	UWOPS87_Ator08_hmg124h_12.JPG.dat	691.3	50.8	0.9	0.07	-0.088	0.073	1.3E-04
YOL116W	YOL116W	UWOPS87_Ator08_hmg124h_12.JPG.dat	819.5	58.5	1.0	0.08	-0.056	0.082	1.5E-04
YOL117W	YOL117W	UWOPS87_Ator08_hmg124h_12.JPG.dat	882.3	43.3	1.0	0.05	-0.002	0.048	2.8E-05
YOL118C	YOL118C	UWOPS87_Ator08_hmg124h_12.JPG.dat	893.8	31.6	1.1	0.01	0.049	0.010	2.9E-05
YOL119C	YOL119C	UWOPS87_Ator08_hmg124h_12.JPG.dat	837.0	17.1	1.0	0.02	-0.036	0.020	2.1E-06
YOL121C	YOL121C	UWOPS87_Ator08_hmg124h_12.JPG.dat	854.3	56.7	1.0	0.07	0.006	0.066	7.2E-05
YOL122C	YOL122C	UWOPS87_Ator08_hmg124h_12.JPG.dat	951.8	43.2	1.2	0.02	0.031	0.016	6.6E-05
YOL124C	YOL124C	UWOPS87_Ator08_hmg124h_12.JPG.dat	923.5	78.9	1.1	0.09	-0.012	0.093	1.5E-04
YOL126C	YOL126C	UWOPS87_Ator08_hmg124h_12.JPG.dat	65.0	130.0	0.0	0.00	0.000	0.000	
YOL128C	YOL128C	UWOPS87_Ator08_hmg124h_12.JPG.dat	972.3	29.7	1.2	0.04	-0.004	0.038	8.4E-06
YOL129W	YOL129W	UWOPS87_Ator08_hmg124h_12.JPG.dat	824.5	34.9	1.1	0.05	0.199	0.046	2.1E-05
YOL131W	YOL131W	UWOPS87_Ator08_hmg124h_12.JPG.dat	832.3	26.0	1.1	0.01	-0.140	0.010	3.0E-05
YOL132W	YOL132W	UWOPS87_Ator08_hmg124h_12.JPG.dat	899.0	35.1	1.1	0.01	0.024	0.010	2.8E-05
YOL136C	YOL136C	UWOPS87_Ator08_hmg124h_12.JPG.dat	884.3	33.2	1.0	0.04	-0.052	0.039	1.5E-05
YOL137W	YOL137W	UWOPS87_Ator08_hmg124h_12.JPG.dat	886.3	52.0	1.0	0.06	-0.012	0.062	5.9E-05
YOL138C	YOL138C	UWOPS87_Ator08_hmg124h_12.JPG.dat	906.0	39.2	1.1	0.01	-0.018	0.011	3.5E-05
YOL141W	YOL141W	UWOPS87_Ator08_hmg124h_12.JPG.dat	977.5	30.8	1.1	0.03	0.008	0.028	4.0E-06
YOL147C	YOL147C	UWOPS87_Ator08_hmg124h_12.JPG.dat	867.0	16.4	1.0	0.01	-0.129	0.014	7.5E-07
YOL150C	YOL150C	UWOPS87_Ator08_hmg124h_12.JPG.dat	917.3	47.5	1.1	0.01	0.049	0.009	2.3E-05
YOL151W	YOL151W	UWOPS87_Ator08_hmg124h_12.JPG.dat	658.0	30.8	0.8	0.04	-0.060	0.039	3.3E-05
YOL152W	YOL152W	UWOPS87_Ator08_hmg124h_12.JPG.dat	846.0	39.0	1.0	0.05	0.007	0.054	4.2E-05
YOL155C	YOL155C	UWOPS87_Ator08_hmg124h_12.JPG.dat	809.5	26.2	1.0	0.04	-0.069	0.035	1.1E-05
YOL158C	YOL158C	UWOPS87_Ator08_hmg124h_12.JPG.dat	800.0	86.1	1.1	0.02	-0.023	0.021	1.2E-04
YOL159C	YOL159C	UWOPS87_Ator08_hmg124h_12.JPG.dat	283.5	361.2	0.4	0.49	-0.036	0.486	2.1E-01
YOL160W	YOL160W	UWOPS87_Ator08_hmg124h_12.JPG.dat	842.5	30.9	1.1	0.05	0.012	0.050	2.6E-05
YOL162W	YOL162W	UWOPS87_Ator08_hmg124h_12.JPG.dat	770.0	46.3	1.0	0.06	-0.174	0.062	5.9E-05
YOL163W	YOL163W	UWOPS87_Ator08_hmg124h_12.JPG.dat	754.5	109.0	1.0	0.14	-0.047	0.142	7.7E-04
YOR001W	YOR001W	UWOPS87_Ator08_hmg124h_12.JPG.dat	842.5	50.8	1.1	0.06	0.077	0.065	5.8E-05
YOR002W	YOR002W	UWOPS87_Ator08_hmg124h_12.JPG.dat	846.3	94.7	1.1	0.03	0.143	0.025	1.7E-04
YOR003W	YOR003W	UWOPS87_Ator08_hmg124h_12.JPG.dat	869.3	52.7	1.0	0.01	-0.053	0.008	1.8E-05
YOR005C	YOR005C	UWOPS87_Ator08_hmg124h_12.JPG.dat	745.5	95.1	1.0	0.01	0.070	0.015	7.9E-05
YOR006C	YOR006C	UWOPS87_Ator08_hmg124h_12.JPG.dat	697.8	44.4	0.9	0.06	-0.197	0.057	8.2E-05
YOR007C	YOR007C	UWOPS87_Ator08_hmg124h_12.JPG.dat	865.3	26.3	1.2	0.03	0.205	0.027	2.9E-06

YOR008C	YOR008C	UWOPS87_Ator08_hmg124h_12.JPG.dat	405.0	144.2	0.6	0.21	-0.476	0.206	1.1E-02
YOR008C-/	YOR008C-A	UWOPS87_Ator08_hmg124h_12.JPG.dat	120.5	241.0	0.0	0.00	0.000	0.000	
YOR009W	YOR009W	UWOPS87_Ator08_hmg124h_12.JPG.dat	800.8	60.1	1.1	0.02	0.189	0.018	8.9E-05
YOR010C	YOR010C	UWOPS87_Ator08_hmg124h_12.JPG.dat	707.8	98.0	1.0	0.14	0.025	0.137	7.0E-04
YOR011W	YOR011W	UWOPS87_Ator08_hmg124h_12.JPG.dat	607.8	69.8	0.9	0.10	0.013	0.098	4.0E-04
YOR012W	YOR012W	UWOPS87_Ator08_hmg124h_12.JPG.dat	628.8	43.1	0.9	0.06	-0.326	0.060	9.2E-05
YOR013W	YOR013W	UWOPS87_Ator08_hmg124h_12.JPG.dat	781.5	53.4	1.0	0.02	0.056	0.018	1.1E-04
YOR014W	YOR014W	UWOPS87_Ator08_hmg124h_12.JPG.dat	831.8	70.5	1.1	0.09	-0.053	0.091	1.8E-04
YOR015W	YOR015W	UWOPS87_Ator08_hmg124h_12.JPG.dat	625.3	423.9	1.1	0.12	0.079	0.119	4.3E-03
YOR016C	YOR016C	UWOPS87_Ator08_hmg124h_12.JPG.dat	751.3	82.7	1.0	0.11	0.117	0.108	3.7E-04
YOR017W	YOR017W	UWOPS87_Ator08_hmg124h_12.JPG.dat	739.8	41.1	1.0	0.05	0.099	0.045	2.4E-05
YOR018W	YOR018W	UWOPS87_Ator08_hmg124h_12.JPG.dat	831.3	61.1	1.1	0.02	0.003	0.021	1.3E-04
YOR019W	YOR019W	UWOPS87_Ator08_hmg124h_12.JPG.dat	847.8	49.9	1.1	0.07	0.038	0.067	6.2E-05
YOR021C	YOR021C	UWOPS87_Ator08_hmg124h_12.JPG.dat	335.8	403.6	0.4	0.52	-0.027	0.517	2.0E-01
YOR022C	YOR022C	UWOPS87_Ator08_hmg124h_12.JPG.dat	712.0	18.1	1.0	0.03	-0.009	0.026	5.8E-06
YOR023C	YOR023C	UWOPS87_Ator08_hmg124h_12.JPG.dat	693.8	22.8	1.0	0.01	0.023	0.010	4.0E-05
YOR024W	YOR024W	UWOPS87_Ator08_hmg124h_12.JPG.dat	724.8	55.7	1.0	0.07	-0.118	0.074	1.3E-04
YOR025W	YOR025W	UWOPS87_Ator08_hmg124h_12.JPG.dat	906.8	53.2	1.1	0.06	0.032	0.061	4.4E-05
YOR026W	YOR026W	UWOPS87_Ator08_hmg124h_12.JPG.dat	200.3	234.9	0.2	0.28	-0.100	0.282	1.9E-01
YOR027W	YOR027W	UWOPS87_Ator08_hmg124h_12.JPG.dat	815.5	37.8	1.0	0.04	0.032	0.043	2.2E-05
YOR028C	YOR028C	UWOPS87_Ator08_hmg124h_12.JPG.dat	808.5	43.2	1.0	0.05	0.068	0.054	3.9E-05
YOR029W	YOR029W	UWOPS87_Ator08_hmg124h_12.JPG.dat	630.5	58.0	0.9	0.09	0.069	0.087	2.4E-04
YOR030W	YOR030W	UWOPS87_Ator08_hmg124h_12.JPG.dat	721.8	38.4	0.9	0.02	0.023	0.019	1.3E-04
YOR031W	YOR031W	UWOPS87_Ator08_hmg124h_12.JPG.dat	814.0	37.4	1.1	0.00	-0.008	0.005	6.7E-06
YOR032C	YOR032C	UWOPS87_Ator08_hmg124h_12.JPG.dat	796.5	106.9	1.0	0.14	-0.067	0.135	6.2E-04
YOR033C	YOR033C	UWOPS87_Ator08_hmg124h_12.JPG.dat	747.8	39.5	1.0	0.05	-0.044	0.049	3.1E-05
YOR034C	YOR034C	UWOPS87_Ator08_hmg124h_12.JPG.dat	832.8	71.1	1.1	0.09	-0.058	0.090	1.5E-04
YOR035C	YOR035C	UWOPS87_Ator08_hmg124h_12.JPG.dat	262.5	68.0	0.3	0.09	-0.225	0.089	4.5E-03
YOR037W	YOR037W	UWOPS87_Ator08_hmg124h_12.JPG.dat	833.3	16.3	1.1	0.02	-0.117	0.022	2.6E-06
YOR038C	YOR038C	UWOPS87_Ator08_hmg124h_12.JPG.dat	854.0	38.9	1.1	0.05	-0.039	0.046	2.2E-05
YOR039W	YOR039W	UWOPS87_Ator08_hmg124h_12.JPG.dat	808.5	25.2	1.0	0.03	-0.089	0.034	9.6E-06
YOR040W	YOR040W	UWOPS87_Ator08_hmg124h_12.JPG.dat	710.3	20.7	1.0	0.03	0.068	0.027	6.1E-06
YOR041C	YOR041C	UWOPS87_Ator08_hmg124h_12.JPG.dat	736.5	81.8	1.1	0.12	0.118	0.118	3.9E-04
YOR042W	YOR042W	UWOPS87_Ator08_hmg124h_12.JPG.dat	836.5	59.3	1.1	0.08	0.135	0.085	1.3E-04
YOR043W	YOR043W	UWOPS87_Ator08_hmg124h_12.JPG.dat	569.5	45.3	0.7	0.06	0.023	0.057	1.4E-04
YOR044W	YOR044W	UWOPS87_Ator08_hmg124h_12.JPG.dat	843.5	35.1	1.0	0.04	0.016	0.042	1.8E-05
YOR045W	YOR045W	UWOPS87_Ator08_hmg124h_12.JPG.dat	819.5	39.9	1.0	0.02	0.007	0.016	7.8E-05
YOR047C	YOR047C	UWOPS87_Ator08_hmg124h_12.JPG.dat	824.0	21.5	1.0	0.03	0.019	0.029	5.8E-06
YOR049C	YOR049C	UWOPS87_Ator08_hmg124h_12.JPG.dat	809.3	28.8	1.0	0.04	-0.119	0.036	1.2E-05
YOR050C	YOR050C	UWOPS87_Ator08_hmg124h_12.JPG.dat	787.5	23.4	1.0	0.03	0.024	0.027	5.8E-06
YOR051C	YOR051C	UWOPS87_Ator08_hmg124h_12.JPG.dat	883.8	58.0	1.1	0.08	-0.068	0.077	9.8E-05
YOR052C	YOR052C	UWOPS87_Ator08_hmg124h_12.JPG.dat	707.8	37.7	0.9	0.05	0.030	0.047	3.9E-05
YOR053W	YOR053W	UWOPS87_Ator08_hmg124h_12.JPG.dat	738.8	45.7	1.0	0.06	-0.065	0.063	7.2E-05
YOR055W	YOR055W	UWOPS87_Ator08_hmg124h_12.JPG.dat	709.8	45.9	1.0	0.06	0.061	0.065	7.1E-05
YOR058C	YOR058C	UWOPS87_Ator08_hmg124h_12.JPG.dat	648.3	90.2	0.8	0.02	0.118	0.023	2.9E-04
YOR059C	YOR059C	UWOPS87_Ator08_hmg124h_12.JPG.dat	778.3	105.5	0.9	0.14	-0.032	0.135	7.9E-04
YOR061W	YOR061W	UWOPS87_Ator08_hmg124h_12.JPG.dat	671.3	118.6	0.8	0.14	-0.094	0.136	1.3E-03
YOR062C	YOR062C	UWOPS87_Ator08_hmg124h_12.JPG.dat	664.0	45.3	0.8	0.06	-0.027	0.062	1.2E-04
YOR064C	YOR064C	UWOPS87_Ator08_hmg124h_12.JPG.dat	833.5	30.1	1.0	0.04	0.032	0.036	1.1E-05
YOR065W	YOR065W	UWOPS87_Ator08_hmg124h_12.JPG.dat	814.3	83.2	1.0	0.10	-0.063	0.096	2.4E-04
YOR066W	YOR066W	UWOPS87_Ator08_hmg124h_12.JPG.dat	804.5	62.9	1.0	0.08	0.008	0.081	1.5E-04
YOR067C	YOR067C	UWOPS87_Ator08_hmg124h_12.JPG.dat	933.5	43.0	1.1	0.05	0.147	0.047	1.9E-05
YOR068C	YOR068C	UWOPS87_Ator08_hmg124h_12.JPG.dat	681.3	384.2	1.1	0.04	-0.054	0.039	4.3E-04
YOR069W	YOR069W	UWOPS87_Ator08_hmg124h_12.JPG.dat	866.3	34.2	1.1	0.04	0.054	0.038	1.1E-05
YOR070C	YOR070C	UWOPS87_Ator08_hmg124h_12.JPG.dat	786.5	61.3	1.0	0.02	-0.016	0.024	1.8E-04
YOR071C	YOR071C	UWOPS87_Ator08_hmg124h_12.JPG.dat	655.8	79.4	0.8	0.10	-0.136	0.098	4.7E-04
YOR072W	YOR072W	UWOPS87_Ator08_hmg124h_12.JPG.dat	808.0	33.8	1.0	0.03	0.129	0.034	1.2E-05
YOR076C	YOR076C	UWOPS87_Ator08_hmg124h_12.JPG.dat	908.3	51.1	1.1	0.02	0.071	0.019	1.0E-04
YOR078W	YOR078W	UWOPS87_Ator08_hmg124h_12.JPG.dat	858.3	21.5	1.0	0.03	0.063	0.032	8.3E-06
YOR079C	YOR079C	UWOPS87_Ator08_hmg124h_12.JPG.dat	923.0	10.6	1.1	0.02	0.004	0.019	1.3E-06
YOR080W	YOR080W	UWOPS87_Ator08_hmg124h_12.JPG.dat	1000.5	82.7	1.2	0.03	0.018	0.028	1.9E-04
YOR081C	YOR081C	UWOPS87_Ator08_hmg124h_12.JPG.dat	704.0	84.1	0.8	0.10	0.002	0.096	3.9E-04
YOR082C	YOR082C	UWOPS87_Ator08_hmg124h_12.JPG.dat	730.5	42.2	0.9	0.04	-0.028	0.039	2.5E-05
YOR083W	YOR083W	UWOPS87_Ator08_hmg124h_12.JPG.dat	861.5	11.1	1.1	0.00	-0.035	0.002	8.6E-07
YOR084W	YOR084W	UWOPS87_Ator08_hmg124h_12.JPG.dat	781.3	49.1	0.9	0.01	-0.008	0.008	2.3E-05
YOR085W	YOR085W	UWOPS87_Ator08_hmg124h_12.JPG.dat	867.3	18.4	1.1	0.03	0.063	0.027	4.1E-06
YOR086C	YOR086C	UWOPS87_Ator08_hmg124h_12.JPG.dat	700.0	24.8	0.9	0.03	0.029	0.029	8.8E-06
YOR087W	YOR087W	UWOPS87_Ator08_hmg124h_12.JPG.dat	544.3	364.1	1.0	0.07	0.177	0.066	1.5E-03
YOR088W	YOR088W	UWOPS87_Ator08_hmg124h_12.JPG.dat	343.5	386.3	0.5	0.53	-0.203	0.525	1.7E-01
YOR089C	YOR089C	UWOPS87_Ator08_hmg124h_12.JPG.dat	794.3	55.3	1.1	0.02	0.022	0.024	1.6E-04

YOR090C	YOR090C	UWOPS87_Ator08_hmg124h_12.JPG.dat 789.8	13.5	1.0	0.01	-0.076	0.006	1.0E-05
YOR091W	YOR091W	UWOPS87_Ator08_hmg124h_12.JPG.dat 799.8	42.9	1.0	0.05	0.097	0.052	3.3E-05
YOR092W	YOR092W	UWOPS87_Ator08_hmg124h_12.JPG.dat 800.0	93.7	1.0	0.12	0.126	0.124	5.0E-04
YOR093C	YOR093C	UWOPS87_Ator08_hmg124h_12.JPG.dat 665.5	27.7	0.8	0.03	-0.071	0.030	1.3E-05
YOR094W	YOR094W	UWOPS87_Ator08_hmg124h_12.JPG.dat 626.8	92.8	0.8	0.12	-0.126	0.116	8.8E-04
YOR097C	YOR097C	UWOPS87_Ator08_hmg124h_12.JPG.dat 837.8	37.1	1.0	0.05	0.023	0.048	3.0E-05
YOR099W	YOR099W	UWOPS87_Ator08_hmg124h_12.JPG.dat 695.8	19.6	1.0	0.01	-0.007	0.006	1.3E-05
YOR100C	YOR100C	UWOPS87_Ator08_hmg124h_12.JPG.dat 717.8	35.8	1.0	0.05	-0.027	0.052	3.6E-05
YOR101W	YOR101W	UWOPS87_Ator08_hmg124h_12.JPG.dat 739.0	56.2	1.1	0.08	-0.083	0.084	1.3E-04
YOR104W	YOR104W	UWOPS87_Ator08_hmg124h_12.JPG.dat 820.3	38.0	1.2	0.05	-0.106	0.053	2.6E-05
YOR105W	YOR105W	UWOPS87_Ator08_hmg124h_12.JPG.dat 739.8	48.1	1.0	0.07	0.081	0.067	7.7E-05
YOR106W	YOR106W	UWOPS87_Ator08_hmg124h_12.JPG.dat 611.5	42.1	0.9	0.06	-0.127	0.059	9.0E-05
YOR107W	YOR107W	UWOPS87_Ator08_hmg124h_12.JPG.dat 607.0	36.7	0.8	0.05	-0.065	0.050	5.9E-05
YOR108W	YOR108W	UWOPS87_Ator08_hmg124h_12.JPG.dat 758.3	123.4	1.1	0.05	0.097	0.052	7.5E-04
YOR109W	YOR109W	UWOPS87_Ator08_hmg124h_12.JPG.dat 730.8	16.0	1.0	0.00	-0.180	0.005	8.6E-06
YOR111W	YOR111W	UWOPS87_Ator08_hmg124h_12.JPG.dat 741.8	146.8	1.0	0.19	0.040	0.189	2.0E-03
YOR112W	YOR112W	UWOPS87_Ator08_hmg124h_12.JPG.dat 880.8	44.5	1.1	0.06	0.172	0.058	3.8E-05
YOR113W	YOR113W	UWOPS87_Ator08_hmg124h_12.JPG.dat 721.0	43.4	1.0	0.05	-0.162	0.049	3.4E-05
YOR114W	YOR114W	UWOPS87_Ator08_hmg124h_12.JPG.dat 779.5	65.0	1.0	0.02	-0.002	0.020	1.3E-04
YOR115C	YOR115C	UWOPS87_Ator08_hmg124h_12.JPG.dat 593.5	33.5	0.8	0.05	-0.178	0.049	6.9E-05
YOR118W	YOR118W	UWOPS87_Ator08_hmg124h_12.JPG.dat 750.8	38.6	1.0	0.05	-0.134	0.050	3.9E-05
YOR120W	YOR120W	UWOPS87_Ator08_hmg124h_12.JPG.dat 784.0	52.4	1.0	0.08	0.129	0.079	1.2E-04
YOR121C	YOR121C	UWOPS87_Ator08_hmg124h_12.JPG.dat 595.5	409.6	1.1	0.16	0.002	0.164	7.8E-03
YOR123C	YOR123C	UWOPS87_Ator08_hmg124h_12.JPG.dat 595.8	43.9	0.8	0.06	-0.199	0.062	1.3E-04
YOR124C	YOR124C	UWOPS87_Ator08_hmg124h_12.JPG.dat 839.0	24.2	1.1	0.03	0.115	0.026	3.8E-06
YOR126C	YOR126C	UWOPS87_Ator08_hmg124h_12.JPG.dat 814.3	31.2	1.0	0.03	0.054	0.031	8.3E-06
YOR127W	YOR127W	UWOPS87_Ator08_hmg124h_12.JPG.dat 883.8	98.8	1.1	0.12	0.053	0.115	3.1E-04
YOR129C	YOR129C	UWOPS87_Ator08_hmg124h_12.JPG.dat 756.0	105.8	1.0	0.04	-0.034	0.044	6.3E-04
YOR131C	YOR131C	UWOPS87_Ator08_hmg124h_12.JPG.dat 717.3	70.2	1.0	0.03	0.118	0.032	3.7E-04
YOR132W	YOR132W	UWOPS87_Ator08_hmg124h_12.JPG.dat 723.8	45.2	1.0	0.05	0.020	0.048	2.9E-05
YOR133W	YOR133W	UWOPS87_Ator08_hmg124h_12.JPG.dat 789.8	94.9	1.0	0.12	-0.021	0.118	4.0E-04
YOR134W	YOR134W	UWOPS87_Ator08_hmg124h_12.JPG.dat 700.8	53.6	0.9	0.07	0.111	0.068	1.2E-04
YOR135C	YOR135C	UWOPS87_Ator08_hmg124h_12.JPG.dat 562.5	55.9	0.7	0.07	-0.268	0.074	2.7E-04
YOR136W	YOR136W	UWOPS87_Ator08_hmg124h_12.JPG.dat 756.5	24.3	1.0	0.04	-0.050	0.037	1.3E-05
YOR137C	YOR137C	UWOPS87_Ator08_hmg124h_12.JPG.dat 828.8	45.1	1.1	0.01	0.004	0.012	4.0E-05
YOR138C	YOR138C	UWOPS87_Ator08_hmg124h_12.JPG.dat 840.5	23.1	1.1	0.01	0.037	0.009	2.2E-05
YOR139C	YOR139C	UWOPS87_Ator08_hmg124h_12.JPG.dat 678.8	82.7	0.9	0.11	-0.027	0.109	5.5E-04
YOR140W	YOR140W	UWOPS87_Ator08_hmg124h_12.JPG.dat 706.0	21.9	0.9	0.03	-0.104	0.029	8.6E-06
YOR141C	YOR141C	UWOPS87_Ator08_hmg124h_12.JPG.dat 724.8	14.7	1.0	0.03	0.064	0.027	6.2E-06
YOR142W	YOR142W	UWOPS87_Ator08_hmg124h_12.JPG.dat 644.3	53.2	0.9	0.08	-0.002	0.078	1.7E-04
YOR144C	YOR144C	UWOPS87_Ator08_hmg124h_12.JPG.dat 691.8	96.2	0.9	0.01	-0.230	0.014	8.0E-05
YOR152C	YOR152C	UWOPS87_Ator08_hmg124h_12.JPG.dat 785.0	55.6	1.0	0.07	-0.116	0.069	9.3E-05
YOR153W	YOR153W	UWOPS87_Ator08_hmg124h_12.JPG.dat 901.8	31.5	1.1	0.04	0.209	0.039	1.2E-05
YOR154W	YOR154W	UWOPS87_Ator08_hmg124h_12.JPG.dat 806.0	90.2	1.0	0.10	0.047	0.103	2.9E-04
YOR156C	YOR156C	UWOPS87_Ator08_hmg124h_12.JPG.dat 795.0	36.4	1.0	0.04	-0.050	0.043	2.2E-05
YOR161C	YOR161C	UWOPS87_Ator08_hmg124h_12.JPG.dat 838.5	43.6	1.0	0.02	-0.053	0.018	1.0E-04
YOR162C	YOR162C	UWOPS87_Ator08_hmg124h_12.JPG.dat 780.0	25.6	1.0	0.04	-0.018	0.036	1.4E-05
YOR163W	YOR163W	UWOPS87_Ator08_hmg124h_12.JPG.dat 752.8	24.9	0.9	0.03	0.067	0.032	1.1E-05
YOR164C	YOR164C	UWOPS87_Ator08_hmg124h_12.JPG.dat 838.0	37.1	1.1	0.04	-0.072	0.043	1.8E-05
YOR165W	YOR165W	UWOPS87_Ator08_hmg124h_12.JPG.dat 766.8	43.0	1.0	0.05	0.031	0.050	3.5E-05
YOR166C	YOR166C	UWOPS87_Ator08_hmg124h_12.JPG.dat 711.8	23.2	1.0	0.04	-0.096	0.040	1.6E-05
YOR167C	YOR167C	UWOPS87_Ator08_hmg124h_12.JPG.dat 662.3	92.5	0.9	0.14	0.181	0.135	8.8E-04
YOR170W	YOR170W	UWOPS87_Ator08_hmg124h_12.JPG.dat 872.3	38.3	1.1	0.01	-0.058	0.010	3.1E-05
YOR171C	YOR171C	UWOPS87_Ator08_hmg124h_12.JPG.dat 792.8	54.5	0.9	0.07	-0.153	0.071	1.2E-04
YOR172W	YOR172W	UWOPS87_Ator08_hmg124h_12.JPG.dat 800.5	44.8	1.0	0.06	-0.067	0.062	7.1E-05
YOR173W	YOR173W	UWOPS87_Ator08_hmg124h_12.JPG.dat 843.8	3.9	1.0	0.01	0.000	0.011	2.9E-07
YOR175C	YOR175C	UWOPS87_Ator08_hmg124h_12.JPG.dat 843.5	29.3	1.0	0.04	-0.037	0.041	1.8E-05
YOR177C	YOR177C	UWOPS87_Ator08_hmg124h_12.JPG.dat 830.3	52.9	1.0	0.06	-0.065	0.063	6.6E-05
YOR178C	YOR178C	UWOPS87_Ator08_hmg124h_12.JPG.dat 898.3	51.5	1.1	0.06	-0.034	0.065	5.5E-05
YOR182C	YOR182C	UWOPS87_Ator08_hmg124h_12.JPG.dat 820.8	39.3	1.0	0.00	0.087	0.004	4.4E-06
YOR183W	YOR183W	UWOPS87_Ator08_hmg124h_12.JPG.dat 675.3	24.0	0.9	0.02	0.056	0.023	5.5E-06
YOR184W	YOR184W	UWOPS87_Ator08_hmg124h_12.JPG.dat 716.0	32.2	1.0	0.04	-0.121	0.037	1.5E-05
YOR185C	YOR185C	UWOPS87_Ator08_hmg124h_12.JPG.dat 753.8	94.0	1.0	0.13	-0.075	0.128	5.8E-04
YOR186W	YOR186W	UWOPS87_Ator08_hmg124h_12.JPG.dat 868.0	142.5	1.1	0.17	0.113	0.172	1.1E-03
YOR188W	YOR188W	UWOPS87_Ator08_hmg124h_12.JPG.dat 885.8	175.2	1.0	0.20	0.116	0.199	1.9E-03
YOR189W	YOR189W	UWOPS87_Ator08_hmg124h_12.JPG.dat 971.5	78.5	1.2	0.02	0.187	0.018	7.2E-05
YOR190W	YOR190W	UWOPS87_Ator08_hmg124h_12.JPG.dat 826.5	41.7	1.0	0.01	-0.029	0.009	2.5E-05
YOR191W	YOR191W	UWOPS87_Ator08_hmg124h_12.JPG.dat 850.8	100.0	1.0	0.11	0.045	0.110	3.6E-04
YOR192C	YOR192C	UWOPS87_Ator08_hmg124h_12.JPG.dat 811.8	50.4	1.0	0.06	0.038	0.059	6.3E-05
YOR193W	YOR193W	UWOPS87_Ator08_hmg124h_12.JPG.dat 823.3	45.0	1.0	0.05	0.026	0.049	3.3E-05

YOR195W	YOR195W	UWOPS87_Ator08_hmg124h_12.JPG.dat	838.5	40.4	1.0	0.06	-0.050	0.064	6.6E-05
YOR196C	YOR196C	UWOPS87_Ator08_hmg124h_12.JPG.dat	821.0	24.0	1.0	0.01	0.010	0.013	6.0E-05
YOR202W	YOR202W	UWOPS87_Ator08_hmg124h_12.JPG.dat	881.2	125.5	1.0	0.09	-0.004	0.087	9.9E-290
1	1	UWOPS87_Ator08_hmg124h_13.JPG.dat	1142.3	174.4	1.1	0.08	0.067	0.080	1.2E-04
2	2	UWOPS87_Ator08_hmg124h_13.JPG.dat	772.0	144.0	0.8	0.11	-0.249	0.113	7.1E-04
3	3	UWOPS87_Ator08_hmg124h_13.JPG.dat	887.3	117.6	1.0	0.08	0.007	0.081	1.6E-04
4	4	UWOPS87_Ator08_hmg124h_13.JPG.dat	979.0	147.8	1.1	0.11	0.088	0.109	2.8E-04
YOR197W	YOR197W	UWOPS87_Ator08_hmg124h_13.JPG.dat	795.3	62.2	1.0	0.06	-0.004	0.065	7.3E-05
YOR202W	YOR202W	UWOPS87_Ator08_hmg124h_13.JPG.dat	924.3	116.2	1.0	0.07	0.001	0.069	0.0E+00
YOR208W	YOR208W	UWOPS87_Ator08_hmg124h_13.JPG.dat	866.0	30.1	1.0	0.03	0.059	0.033	8.3E-06
YOR209C	YOR209C	UWOPS87_Ator08_hmg124h_13.JPG.dat	820.8	50.2	1.0	0.01	0.001	0.015	8.1E-05
YOR212W	YOR212W	UWOPS87_Ator08_hmg124h_13.JPG.dat	482.3	332.7	0.8	0.12	-0.070	0.117	7.6E-03
YOR213C	YOR213C	UWOPS87_Ator08_hmg124h_13.JPG.dat	760.3	21.2	0.9	0.01	-0.029	0.008	2.5E-05
YOR214C	YOR214C	UWOPS87_Ator08_hmg124h_13.JPG.dat	914.8	37.2	1.1	0.04	0.121	0.036	9.0E-06
YOR215C	YOR215C	UWOPS87_Ator08_hmg124h_13.JPG.dat	849.5	45.8	1.0	0.07	-0.038	0.065	8.0E-05
YOR216C	YOR216C	UWOPS87_Ator08_hmg124h_13.JPG.dat	970.5	34.5	1.1	0.04	0.004	0.042	1.6E-05
YOR219C	YOR219C	UWOPS87_Ator08_hmg124h_13.JPG.dat	886.0	32.8	1.1	0.00	0.059	0.002	1.7E-06
YOR221C	YOR221C	UWOPS87_Ator08_hmg124h_13.JPG.dat	801.3	66.7	1.0	0.09	0.022	0.089	2.0E-04
YOR222W	YOR222W	UWOPS87_Ator08_hmg124h_13.JPG.dat	814.5	19.2	1.0	0.04	0.000	0.043	2.0E-05
YOR223W	YOR223W	UWOPS87_Ator08_hmg124h_13.JPG.dat	819.8	103.9	1.0	0.12	0.010	0.116	4.3E-04
YOR225W	YOR225W	UWOPS87_Ator08_hmg124h_13.JPG.dat	924.5	77.0	1.1	0.11	0.026	0.112	3.1E-04
YOR226C	YOR226C	UWOPS87_Ator08_hmg124h_13.JPG.dat	879.5	45.6	1.0	0.03	-0.020	0.033	9.8E-06
YOR227W	YOR227W	UWOPS87_Ator08_hmg124h_13.JPG.dat	844.0	79.3	1.0	0.08	-0.066	0.076	1.3E-04
YOR228C	YOR228C	UWOPS87_Ator08_hmg124h_13.JPG.dat	831.0	41.5	1.0	0.06	-0.018	0.056	5.2E-05
YOR229W	YOR229W	UWOPS87_Ator08_hmg124h_13.JPG.dat	787.5	43.3	1.0	0.05	0.052	0.047	3.3E-05
YOR230W	YOR230W	UWOPS87_Ator08_hmg124h_13.JPG.dat	901.5	19.2	1.0	0.01	0.039	0.006	9.6E-06
YOR231W	YOR231W	UWOPS87_Ator08_hmg124h_13.JPG.dat	928.5	22.1	1.0	0.01	-0.075	0.011	3.7E-05
YOR233W	YOR233W	UWOPS87_Ator08_hmg124h_13.JPG.dat	860.5	80.6	0.9	0.02	0.024	0.017	1.0E-04
YOR234C	YOR234C	UWOPS87_Ator08_hmg124h_13.JPG.dat	721.0	67.8	0.9	0.08	0.023	0.085	2.5E-04
YOR235W	YOR235W	UWOPS87_Ator08_hmg124h_13.JPG.dat	750.8	39.8	1.0	0.04	0.081	0.040	1.9E-05
YOR237W	YOR237W	UWOPS87_Ator08_hmg124h_13.JPG.dat	713.5	51.3	0.9	0.05	-0.214	0.055	6.3E-05
YOR238W	YOR238W	UWOPS87_Ator08_hmg124h_13.JPG.dat	902.8	22.1	1.1	0.03	0.046	0.033	7.8E-06
YOR239W	YOR239W	UWOPS87_Ator08_hmg124h_13.JPG.dat	767.5	42.8	0.9	0.06	-0.092	0.057	6.4E-05
YOR242C	YOR242C	UWOPS87_Ator08_hmg124h_13.JPG.dat	823.0	59.1	1.0	0.07	0.005	0.069	9.1E-05
YOR243C	YOR243C	UWOPS87_Ator08_hmg124h_13.JPG.dat	864.5	67.0	1.0	0.01	0.089	0.011	3.7E-05
YOR245C	YOR245C	UWOPS87_Ator08_hmg124h_13.JPG.dat	758.8	28.1	1.0	0.04	-0.205	0.044	2.7E-05
YOR246C	YOR246C	UWOPS87_Ator08_hmg124h_13.JPG.dat	806.0	147.7	1.0	0.18	-0.053	0.184	1.8E-03
YOR247W	YOR247W	UWOPS87_Ator08_hmg124h_13.JPG.dat	786.0	90.2	0.9	0.11	-0.042	0.106	4.1E-04
YOR251C	YOR251C	UWOPS87_Ator08_hmg124h_13.JPG.dat	947.8	41.5	1.1	0.06	0.127	0.060	4.0E-05
YOR252W	YOR252W	UWOPS87_Ator08_hmg124h_13.JPG.dat	819.3	13.9	1.0	0.02	-0.024	0.018	1.6E-06
YOR253W	YOR253W	UWOPS87_Ator08_hmg124h_13.JPG.dat	844.8	102.0	1.0	0.13	0.058	0.134	5.7E-04
YOR255W	YOR255W	UWOPS87_Ator08_hmg124h_13.JPG.dat	831.5	92.9	1.0	0.11	-0.029	0.106	3.5E-04
YOR263C	YOR263C	UWOPS87_Ator08_hmg124h_13.JPG.dat	862.5	51.2	1.0	0.06	0.005	0.063	6.9E-05
YOR264W	YOR264W	UWOPS87_Ator08_hmg124h_13.JPG.dat	892.8	86.9	1.1	0.09	0.076	0.095	1.9E-04
YOR265W	YOR265W	UWOPS87_Ator08_hmg124h_13.JPG.dat	809.0	57.8	1.0	0.07	-0.054	0.067	9.2E-05
YOR266W	YOR266W	UWOPS87_Ator08_hmg124h_13.JPG.dat	906.0	25.5	1.1	0.04	0.051	0.037	1.1E-05
YOR267C	YOR267C	UWOPS87_Ator08_hmg124h_13.JPG.dat	879.5	43.2	1.1	0.05	-0.038	0.050	2.8E-05
YOR268C	YOR268C	UWOPS87_Ator08_hmg124h_13.JPG.dat	800.3	64.5	0.9	0.08	0.013	0.076	1.5E-04
YOR269W	YOR269W	UWOPS87_Ator08_hmg124h_13.JPG.dat	678.3	455.4	1.0	0.08	-0.140	0.080	2.0E-03
YOR270C	YOR270C	UWOPS87_Ator08_hmg124h_13.JPG.dat	481.5	328.2	0.7	0.10	0.019	0.097	5.6E-03
YOR271C	YOR271C	UWOPS87_Ator08_hmg124h_13.JPG.dat	850.0	37.3	1.0	0.05	0.041	0.051	3.2E-05
YOR273C	YOR273C	UWOPS87_Ator08_hmg124h_13.JPG.dat	869.0	54.1	1.1	0.05	0.108	0.050	2.8E-05
YOR274W	YOR274W	UWOPS87_Ator08_hmg124h_13.JPG.dat	853.3	44.4	1.0	0.05	0.010	0.055	4.3E-05
YOR275C	YOR275C	UWOPS87_Ator08_hmg124h_13.JPG.dat	820.0	36.7	1.0	0.01	0.028	0.009	3.0E-05
YOR276W	YOR276W	UWOPS87_Ator08_hmg124h_13.JPG.dat	631.8	44.1	0.8	0.05	-0.126	0.054	9.6E-05
YOR277C	YOR277C	UWOPS87_Ator08_hmg124h_13.JPG.dat	838.8	23.7	1.0	0.03	0.044	0.031	8.2E-06
YOR279C	YOR279C	UWOPS87_Ator08_hmg124h_13.JPG.dat	944.5	79.5	1.1	0.01	0.038	0.009	2.6E-05
YOR280C	YOR280C	UWOPS87_Ator08_hmg124h_13.JPG.dat	861.8	64.9	1.0	0.08	0.089	0.079	1.2E-04
YOR283W	YOR283W	UWOPS87_Ator08_hmg124h_13.JPG.dat	885.8	36.9	1.1	0.02	0.031	0.016	7.5E-05
YOR284W	YOR284W	UWOPS87_Ator08_hmg124h_13.JPG.dat	883.3	72.0	1.0	0.03	-0.054	0.029	3.0E-04
YOR285W	YOR285W	UWOPS87_Ator08_hmg124h_13.JPG.dat	852.8	72.7	1.0	0.09	0.013	0.092	2.1E-04
YOR286W	YOR286W	UWOPS87_Ator08_hmg124h_13.JPG.dat	796.0	24.9	1.0	0.03	0.026	0.027	6.1E-06
YOR288C	YOR288C	UWOPS87_Ator08_hmg124h_13.JPG.dat	841.0	53.3	1.0	0.00	-0.016	0.004	4.5E-06
YOR289W	YOR289W	UWOPS87_Ator08_hmg124h_13.JPG.dat	767.8	68.0	0.9	0.09	-0.037	0.085	2.0E-04
YOR291W	YOR291W	UWOPS87_Ator08_hmg124h_13.JPG.dat	836.0	15.2	1.0	0.01	0.076	0.012	5.0E-07
YOR292C	YOR292C	UWOPS87_Ator08_hmg124h_13.JPG.dat	867.3	40.2	1.0	0.04	0.066	0.037	1.3E-05
YOR293W	YOR293W	UWOPS87_Ator08_hmg124h_13.JPG.dat	712.0	33.4	0.9	0.01	-0.058	0.012	6.7E-05
YOR296W	YOR296W	UWOPS87_Ator08_hmg124h_13.JPG.dat	605.0	399.9	1.0	0.04	-0.124	0.036	4.8E-04
YOR297C	YOR297C	UWOPS87_Ator08_hmg124h_13.JPG.dat	787.0	18.7	1.0	0.03	0.062	0.025	4.8E-06
YOR298C-/	YOR298C-A	UWOPS87_Ator08_hmg124h_13.JPG.dat	873.3	37.7	1.1	0.05	0.067	0.046	2.2E-05

YOR298W	YOR298W	UWOPS87_Ator08_hmg124h_13.JPG.dat	889.3	46.3	1.1	0.05	-0.023	0.053	3.4E-05
YOR299W	YOR299W	UWOPS87_Ator08_hmg124h_13.JPG.dat	963.0	57.9	1.2	0.07	0.203	0.070	6.1E-05
YOR300W	YOR300W	UWOPS87_Ator08_hmg124h_13.JPG.dat	751.0	28.9	0.9	0.04	0.083	0.038	1.8E-05
YOR301W	YOR301W	UWOPS87_Ator08_hmg124h_13.JPG.dat	853.8	64.7	1.0	0.07	-0.027	0.069	8.9E-05
YOR302W	YOR302W	UWOPS87_Ator08_hmg124h_13.JPG.dat	818.0	98.4	1.0	0.12	-0.061	0.124	5.9E-04
YOR303W	YOR303W	UWOPS87_Ator08_hmg124h_13.JPG.dat	900.5	24.6	1.0	0.03	0.033	0.029	6.2E-06
YOR304C-/	YOR304C-A	UWOPS87_Ator08_hmg124h_13.JPG.dat	920.8	35.8	1.0	0.03	0.113	0.031	7.0E-06
YOR304W	YOR304W	UWOPS87_Ator08_hmg124h_13.JPG.dat	837.5	26.5	0.9	0.04	-0.015	0.037	1.7E-05
YOR306C	YOR306C	UWOPS87_Ator08_hmg124h_13.JPG.dat	905.8	8.0	1.0	0.01	0.048	0.013	5.0E-07
YOR307C	YOR307C	UWOPS87_Ator08_hmg124h_13.JPG.dat	828.8	80.0	1.0	0.09	-0.100	0.094	2.3E-04
YOR308C	YOR308C	UWOPS87_Ator08_hmg124h_13.JPG.dat	811.3	83.1	1.0	0.09	0.013	0.087	2.0E-04
YOR309C	YOR309C	UWOPS87_Ator08_hmg124h_13.JPG.dat	811.0	40.7	1.0	0.04	0.013	0.040	1.9E-05
YOR311C	YOR311C	UWOPS87_Ator08_hmg124h_13.JPG.dat	748.0	90.2	0.9	0.11	-0.082	0.115	5.8E-04
YOR312C	YOR312C	UWOPS87_Ator08_hmg124h_13.JPG.dat	949.8	102.4	1.1	0.12	0.362	0.123	3.4E-04
YOR313C	YOR313C	UWOPS87_Ator08_hmg124h_13.JPG.dat	749.3	21.9	1.0	0.02	0.009	0.021	2.8E-06
YOR314W	YOR314W	UWOPS87_Ator08_hmg124h_13.JPG.dat	823.5	38.6	1.0	0.05	-0.019	0.047	2.6E-05
YOR315W	YOR315W	UWOPS87_Ator08_hmg124h_13.JPG.dat	766.5	16.0	0.9	0.02	-0.127	0.021	3.2E-06
YOR316C	YOR316C	UWOPS87_Ator08_hmg124h_13.JPG.dat	854.8	61.8	1.0	0.01	0.023	0.010	3.3E-05
YOR317W	YOR317W	UWOPS87_Ator08_hmg124h_13.JPG.dat	826.3	21.4	1.0	0.02	-0.063	0.023	3.7E-06
YOR318C	YOR318C	UWOPS87_Ator08_hmg124h_13.JPG.dat	878.5	38.1	1.0	0.04	0.046	0.044	2.1E-05
YOR320C	YOR320C	UWOPS87_Ator08_hmg124h_13.JPG.dat	716.0	107.5	0.9	0.13	-0.189	0.126	8.1E-04
YOR321W	YOR321W	UWOPS87_Ator08_hmg124h_13.JPG.dat	788.3	51.1	0.9	0.07	-0.087	0.071	1.2E-04
YOR322C	YOR322C	UWOPS87_Ator08_hmg124h_13.JPG.dat	925.5	66.4	1.0	0.02	0.007	0.023	1.7E-04
YOR324C	YOR324C	UWOPS87_Ator08_hmg124h_13.JPG.dat	1010.8	36.0	1.1	0.04	0.159	0.041	1.3E-05
YOR327C	YOR327C	UWOPS87_Ator08_hmg124h_13.JPG.dat	787.0	26.3	1.0	0.01	0.003	0.007	1.8E-05
YOR328W	YOR328W	UWOPS87_Ator08_hmg124h_13.JPG.dat	757.5	32.5	1.0	0.00	0.007	0.003	2.3E-06
YOR334W	YOR334W	UWOPS87_Ator08_hmg124h_13.JPG.dat	794.0	127.2	1.0	0.14	-0.042	0.138	7.7E-04
YOR337W	YOR337W	UWOPS87_Ator08_hmg124h_13.JPG.dat	797.5	19.1	1.0	0.01	-0.031	0.013	7.7E-07
YOR338W	YOR338W	UWOPS87_Ator08_hmg124h_13.JPG.dat	884.0	74.6	1.0	0.10	0.008	0.101	2.6E-04
YOR339C	YOR339C	UWOPS87_Ator08_hmg124h_13.JPG.dat	876.3	34.2	1.0	0.05	-0.009	0.046	2.5E-05
YOR342C	YOR342C	UWOPS87_Ator08_hmg124h_13.JPG.dat	806.3	89.6	1.0	0.03	0.061	0.033	3.8E-04
YOR343C	YOR343C	UWOPS87_Ator08_hmg124h_13.JPG.dat	878.5	58.9	1.0	0.02	0.000	0.021	1.4E-04
YOR344C	YOR344C	UWOPS87_Ator08_hmg124h_13.JPG.dat	776.8	18.4	0.9	0.04	-0.046	0.040	2.3E-05
YOR346W	YOR346W	UWOPS87_Ator08_hmg124h_13.JPG.dat	928.5	17.4	1.0	0.01	0.049	0.008	2.0E-05
YOR347C	YOR347C	UWOPS87_Ator08_hmg124h_13.JPG.dat	908.5	67.8	1.0	0.07	0.032	0.071	9.4E-05
YOR348C	YOR348C	UWOPS87_Ator08_hmg124h_13.JPG.dat	867.0	19.4	1.0	0.00	0.048	0.002	7.1E-07
YOR349W	YOR349W	UWOPS87_Ator08_hmg124h_13.JPG.dat	716.8	53.3	1.0	0.02	0.054	0.018	9.9E-05
YOR350C	YOR350C	UWOPS87_Ator08_hmg124h_13.JPG.dat	668.8	80.7	0.8	0.11	-0.152	0.105	5.2E-04
YOR351C	YOR351C	UWOPS87_Ator08_hmg124h_13.JPG.dat	832.3	56.3	1.0	0.02	-0.061	0.018	1.0E-04
YOR352W	YOR352W	UWOPS87_Ator08_hmg124h_13.JPG.dat	849.8	21.7	1.0	0.01	0.003	0.008	2.3E-05
YOR354C	YOR354C	UWOPS87_Ator08_hmg124h_13.JPG.dat	796.0	24.9	1.0	0.03	-0.016	0.028	7.0E-06
YOR355W	YOR355W	UWOPS87_Ator08_hmg124h_13.JPG.dat	758.5	91.1	0.9	0.11	0.002	0.107	4.4E-04
YOR356W	YOR356W	UWOPS87_Ator08_hmg124h_13.JPG.dat	931.3	39.7	1.2	0.04	0.113	0.040	1.1E-05
YOR357C	YOR357C	UWOPS87_Ator08_hmg124h_13.JPG.dat	751.5	30.2	1.0	0.01	-0.035	0.010	3.4E-05
YOR358W	YOR358W	UWOPS87_Ator08_hmg124h_13.JPG.dat	863.0	44.1	1.0	0.01	0.042	0.013	5.2E-05
YOR359W	YOR359W	UWOPS87_Ator08_hmg124h_13.JPG.dat	963.5	19.5	1.1	0.02	0.019	0.018	1.1E-06
YOR360C	YOR360C	UWOPS87_Ator08_hmg124h_13.JPG.dat	571.5	390.1	1.0	0.12	-0.146	0.121	5.3E-03
YOR363C	YOR363C	UWOPS87_Ator08_hmg124h_13.JPG.dat	764.5	37.2	1.0	0.05	-0.104	0.047	2.9E-05
YOR364W	YOR364W	UWOPS87_Ator08_hmg124h_13.JPG.dat	906.5	61.6	1.1	0.07	0.035	0.074	9.0E-05
YOR365C	YOR365C	UWOPS87_Ator08_hmg124h_13.JPG.dat	910.3	39.0	1.1	0.04	0.092	0.041	1.7E-05
YOR367W	YOR367W	UWOPS87_Ator08_hmg124h_13.JPG.dat	834.5	56.5	1.0	0.07	0.026	0.073	1.1E-04
YOR368W	YOR368W	UWOPS87_Ator08_hmg124h_13.JPG.dat	778.5	32.4	0.9	0.03	-0.041	0.032	1.1E-05
YOR371C	YOR371C	UWOPS87_Ator08_hmg124h_13.JPG.dat	852.0	28.7	1.0	0.01	-0.116	0.006	1.3E-05
YOR374W	YOR374W	UWOPS87_Ator08_hmg124h_13.JPG.dat	763.5	55.0	1.0	0.03	0.009	0.026	2.4E-04
YOR375C	YOR375C	UWOPS87_Ator08_hmg124h_13.JPG.dat	797.8	27.0	0.9	0.01	0.082	0.007	2.1E-05
YOR376W	YOR376W	UWOPS87_Ator08_hmg124h_13.JPG.dat	805.3	48.1	0.9	0.05	-0.078	0.050	4.3E-05
YOR377W	YOR377W	UWOPS87_Ator08_hmg124h_13.JPG.dat	922.0	19.0	1.1	0.03	0.027	0.029	5.9E-06
YOR378W	YOR378W	UWOPS87_Ator08_hmg124h_13.JPG.dat	771.3	26.7	0.9	0.04	0.005	0.037	1.7E-05
YOR380W	YOR380W	UWOPS87_Ator08_hmg124h_13.JPG.dat	784.3	34.0	1.0	0.01	0.037	0.009	2.4E-05
YOR381W	YOR381W	UWOPS87_Ator08_hmg124h_13.JPG.dat	872.8	89.2	1.0	0.10	-0.075	0.105	2.8E-04
YOR382W	YOR382W	UWOPS87_Ator08_hmg124h_13.JPG.dat	869.5	44.9	1.0	0.06	0.066	0.055	4.3E-05
YOR383C	YOR383C	UWOPS87_Ator08_hmg124h_13.JPG.dat	760.5	55.4	0.9	0.07	-0.155	0.069	1.2E-04
YOR384W	YOR384W	UWOPS87_Ator08_hmg124h_13.JPG.dat	807.8	17.8	1.0	0.01	-0.168	0.008	2.5E-05
YOR385W	YOR385W	UWOPS87_Ator08_hmg124h_13.JPG.dat	877.5	40.0	1.0	0.05	0.002	0.046	2.4E-05
YOR386W	YOR386W	UWOPS87_Ator08_hmg124h_13.JPG.dat	782.5	27.9	0.9	0.04	0.008	0.036	1.5E-05
YPL001W	YPL001W	UWOPS87_Ator08_hmg124h_13.JPG.dat	627.5	428.1	1.0	0.13	0.014	0.128	5.4E-03
YPL003W	YPL003W	UWOPS87_Ator08_hmg124h_13.JPG.dat	759.0	60.0	0.9	0.07	-0.019	0.068	1.2E-04
YPL004C	YPL004C	UWOPS87_Ator08_hmg124h_13.JPG.dat	933.8	55.8	1.1	0.07	0.015	0.065	6.2E-05
YPL008W	YPL008W	UWOPS87_Ator08_hmg124h_13.JPG.dat	848.0	22.9	1.0	0.03	-0.079	0.028	5.4E-06
YPL009C	YPL009C	UWOPS87_Ator08_hmg124h_13.JPG.dat	772.8	35.6	1.0	0.05	-0.140	0.047	2.8E-05

YPL014W	YPL014W	UWOPS87_Ator08_hmg124h_13.JPG.dat 754.5	20.4	0.9	0.01	0.014	0.006	1.3E-05
YPL017C	YPL017C	UWOPS87_Ator08_hmg124h_13.JPG.dat 795.0	28.5	1.0	0.04	-0.146	0.038	1.8E-05
YPL018W	YPL018W	UWOPS87_Ator08_hmg124h_13.JPG.dat 819.8	14.6	1.0	0.01	-0.063	0.007	1.6E-05
YPL019C	YPL019C	UWOPS87_Ator08_hmg124h_13.JPG.dat 881.5	43.1	1.0	0.06	-0.002	0.059	4.9E-05
YPL021W	YPL021W	UWOPS87_Ator08_hmg124h_13.JPG.dat 891.3	18.6	1.0	0.02	-0.025	0.020	1.9E-06
YPL022W	YPL022W	UWOPS87_Ator08_hmg124h_13.JPG.dat 834.0	18.4	1.0	0.02	0.094	0.022	2.8E-06
YPL023C	YPL023C	UWOPS87_Ator08_hmg124h_13.JPG.dat 884.0	30.1	1.1	0.01	0.086	0.009	2.1E-05
YPL024W	YPL024W	UWOPS87_Ator08_hmg124h_13.JPG.dat 426.0	368.8	0.5	0.45	-0.240	0.449	1.0E-01
YPL025C	YPL025C	UWOPS87_Ator08_hmg124h_13.JPG.dat 869.0	56.3	1.0	0.07	0.051	0.072	9.1E-05
YPL026C	YPL026C	UWOPS87_Ator08_hmg124h_13.JPG.dat 838.8	80.8	1.0	0.09	-0.085	0.093	2.0E-04
YPL027W	YPL027W	UWOPS87_Ator08_hmg124h_13.JPG.dat 767.3	64.6	0.9	0.08	-0.004	0.077	1.5E-04
YPL030W	YPL030W	UWOPS87_Ator08_hmg124h_13.JPG.dat 867.0	68.2	1.0	0.08	-0.006	0.079	1.3E-04
YPL032C	YPL032C	UWOPS87_Ator08_hmg124h_13.JPG.dat 881.5	145.1	1.0	0.16	-0.097	0.163	1.1E-03
YPL033C	YPL033C	UWOPS87_Ator08_hmg124h_13.JPG.dat 1007.3	56.9	1.1	0.05	0.100	0.053	2.7E-05
YPL034W	YPL034W	UWOPS87_Ator08_hmg124h_13.JPG.dat 976.0	56.1	1.1	0.06	-0.029	0.058	4.1E-05
YPL036W	YPL036W	UWOPS87_Ator08_hmg124h_13.JPG.dat 946.3	157.5	1.1	0.17	0.090	0.173	1.2E-03
YPL037C	YPL037C	UWOPS87_Ator08_hmg124h_13.JPG.dat 853.5	38.9	1.0	0.05	0.006	0.047	2.8E-05
YPL038W	YPL038W	UWOPS87_Ator08_hmg124h_13.JPG.dat 864.3	42.1	1.0	0.05	0.006	0.053	3.7E-05
YPL039W	YPL039W	UWOPS87_Ator08_hmg124h_13.JPG.dat 808.8	10.7	1.0	0.00	-0.064	0.001	5.7E-07
YPL041C	YPL041C	UWOPS87_Ator08_hmg124h_13.JPG.dat 794.8	16.9	1.0	0.01	-0.019	0.014	9.2E-07
YPL046C	YPL046C	UWOPS87_Ator08_hmg124h_13.JPG.dat 786.5	19.1	0.9	0.02	-0.014	0.021	3.3E-06
YPL047W	YPL047W	UWOPS87_Ator08_hmg124h_13.JPG.dat 789.0	18.1	1.0	0.05	-0.004	0.047	3.0E-05
YPL048W	YPL048W	UWOPS87_Ator08_hmg124h_13.JPG.dat 828.8	23.6	1.0	0.04	0.123	0.036	1.2E-05
YPL051W	YPL051W	UWOPS87_Ator08_hmg124h_13.JPG.dat 822.8	27.2	1.0	0.04	-0.099	0.045	2.5E-05
YPL052W	YPL052W	UWOPS87_Ator08_hmg124h_13.JPG.dat 851.8	23.9	1.0	0.05	-0.010	0.049	3.1E-05
YPL053C	YPL053C	UWOPS87_Ator08_hmg124h_13.JPG.dat 893.5	57.8	1.1	0.06	-0.005	0.061	5.2E-05
YPL054W	YPL054W	UWOPS87_Ator08_hmg124h_13.JPG.dat 874.5	49.6	1.0	0.03	-0.061	0.030	6.8E-06
YPL055C	YPL055C	UWOPS87_Ator08_hmg124h_13.JPG.dat 922.5	90.2	1.1	0.09	0.053	0.089	1.4E-04
YPL056C	YPL056C	UWOPS87_Ator08_hmg124h_13.JPG.dat 830.3	40.7	1.0	0.04	-0.064	0.038	1.7E-05
YPL057C	YPL057C	UWOPS87_Ator08_hmg124h_13.JPG.dat 826.5	42.3	0.9	0.01	-0.088	0.009	3.5E-05
YPL058C	YPL058C	UWOPS87_Ator08_hmg124h_13.JPG.dat 916.0	50.5	1.0	0.07	0.014	0.070	8.1E-05
YPL060W	YPL060W	UWOPS87_Ator08_hmg124h_13.JPG.dat 761.8	45.5	0.9	0.02	-0.256	0.024	2.5E-04
YPL061W	YPL061W	UWOPS87_Ator08_hmg124h_13.JPG.dat 719.3	75.8	0.9	0.09	0.003	0.087	2.2E-04
YPL062W	YPL062W	UWOPS87_Ator08_hmg124h_13.JPG.dat 781.3	46.2	1.0	0.02	0.037	0.023	1.6E-04
YPL064C	YPL064C	UWOPS87_Ator08_hmg124h_13.JPG.dat 851.3	106.6	1.0	0.13	0.070	0.132	5.6E-04
YPL066W	YPL066W	UWOPS87_Ator08_hmg124h_13.JPG.dat 757.8	116.2	0.9	0.14	-0.051	0.142	1.0E-03
YPL067C	YPL067C	UWOPS87_Ator08_hmg124h_13.JPG.dat 852.8	40.3	1.0	0.01	-0.176	0.013	5.3E-05
YPL068C	YPL068C	UWOPS87_Ator08_hmg124h_13.JPG.dat 825.5	39.9	1.0	0.02	-0.006	0.015	8.2E-05
YPL069C	YPL069C	UWOPS87_Ator08_hmg124h_13.JPG.dat 676.3	39.6	0.8	0.05	-0.252	0.050	5.7E-05
YPL070W	YPL070W	UWOPS87_Ator08_hmg124h_13.JPG.dat 860.3	36.4	1.0	0.04	-0.026	0.036	1.2E-05
YPL071C	YPL071C	UWOPS87_Ator08_hmg124h_13.JPG.dat 904.5	43.4	1.0	0.04	0.065	0.043	2.0E-05
YPL073C	YPL073C	UWOPS87_Ator08_hmg124h_13.JPG.dat 808.8	133.7	1.0	0.04	-0.012	0.041	5.2E-04
YPL074W	YPL074W	UWOPS87_Ator08_hmg124h_13.JPG.dat 810.8	21.9	1.0	0.03	-0.049	0.033	9.3E-06
YPL079W	YPL079W	UWOPS87_Ator08_hmg124h_13.JPG.dat 816.8	48.2	1.1	0.06	0.026	0.064	5.8E-05
YPL080C	YPL080C	UWOPS87_Ator08_hmg124h_13.JPG.dat 790.3	62.0	1.0	0.08	0.193	0.081	1.5E-04
YPL081W	YPL081W	UWOPS87_Ator08_hmg124h_13.JPG.dat 752.8	25.9	0.9	0.03	-0.011	0.033	1.4E-05
YPL086C	YPL086C	UWOPS87_Ator08_hmg124h_13.JPG.dat 910.5	44.5	1.1	0.01	0.101	0.011	3.6E-05
YPL087W	YPL087W	UWOPS87_Ator08_hmg124h_13.JPG.dat 861.8	90.6	1.0	0.11	0.100	0.109	3.2E-04
YPL088W	YPL088W	UWOPS87_Ator08_hmg124h_13.JPG.dat 816.8	49.6	1.0	0.06	0.059	0.065	7.7E-05
YPL089C	YPL089C	UWOPS87_Ator08_hmg124h_13.JPG.dat 763.5	33.3	0.9	0.01	-0.163	0.013	6.0E-05
YPL090C	YPL090C	UWOPS87_Ator08_hmg124h_13.JPG.dat 714.5	178.0	0.9	0.21	0.034	0.211	3.9E-03
YPL091W	YPL091W	UWOPS87_Ator08_hmg124h_13.JPG.dat 876.3	24.1	1.0	0.03	-0.050	0.031	7.9E-06
YPL092W	YPL092W	UWOPS87_Ator08_hmg124h_13.JPG.dat 766.5	33.8	0.9	0.01	-0.130	0.008	2.3E-05
YPL095C	YPL095C	UWOPS87_Ator08_hmg124h_13.JPG.dat 819.5	10.3	1.0	0.00	0.007	0.005	6.9E-06
YPL096W	YPL096W	UWOPS87_Ator08_hmg124h_13.JPG.dat 821.3	64.1	1.0	0.09	-0.023	0.085	1.5E-04
YPL098C	YPL098C	UWOPS87_Ator08_hmg124h_13.JPG.dat 722.5	108.2	0.8	0.03	-0.193	0.034	5.8E-04
YPL099C	YPL099C	UWOPS87_Ator08_hmg124h_13.JPG.dat 906.8	78.6	1.1	0.09	-0.030	0.090	1.7E-04
YPL100W	YPL100W	UWOPS87_Ator08_hmg124h_13.JPG.dat 909.0	44.3	1.1	0.05	0.040	0.054	3.4E-05
YPL101W	YPL101W	UWOPS87_Ator08_hmg124h_13.JPG.dat 796.0	23.3	1.0	0.03	-0.102	0.032	1.0E-05
YPL102C	YPL102C	UWOPS87_Ator08_hmg124h_13.JPG.dat 730.0	165.4	1.0	0.05	-0.039	0.050	8.9E-04
YPL103C	YPL103C	UWOPS87_Ator08_hmg124h_13.JPG.dat 782.0	51.5	1.0	0.06	0.071	0.061	7.0E-05
YPL105C	YPL105C	UWOPS87_Ator08_hmg124h_13.JPG.dat 855.5	29.3	1.0	0.01	-0.051	0.009	3.0E-05
YPL106C	YPL106C	UWOPS87_Ator08_hmg124h_13.JPG.dat 845.8	35.0	1.0	0.04	0.037	0.041	2.1E-05
YPL107W	YPL107W	UWOPS87_Ator08_hmg124h_13.JPG.dat 899.5	41.6	1.1	0.02	-0.024	0.016	7.6E-05
YPL108W	YPL108W	UWOPS87_Ator08_hmg124h_13.JPG.dat 791.8	17.4	1.0	0.00	0.042	0.004	4.2E-06
YPL109C	YPL109C	UWOPS87_Ator08_hmg124h_13.JPG.dat 702.8	46.2	0.9	0.05	0.020	0.054	6.1E-05
YPL110C	YPL110C	UWOPS87_Ator08_hmg124h_13.JPG.dat 904.3	47.9	1.1	0.06	-0.015	0.059	4.4E-05
YPL111W	YPL111W	UWOPS87_Ator08_hmg124h_13.JPG.dat 463.5	327.2	0.6	0.39	-0.137	0.392	6.6E-02
YPL112C	YPL112C	UWOPS87_Ator08_hmg124h_13.JPG.dat 778.8	59.2	1.0	0.07	-0.035	0.072	1.1E-04
YPL113C	YPL113C	UWOPS87_Ator08_hmg124h_13.JPG.dat 817.0	29.9	1.0	0.03	0.015	0.033	9.4E-06

YPL114W	YPL114W	UWOPS87_Ator08_hmg124h_13.JPG.dat 740.8	29.8	0.9	0.04	-0.127	0.039	2.2E-05
YPL115C	YPL115C	UWOPS87_Ator08_hmg124h_13.JPG.dat 805.5	22.4	1.0	0.03	0.091	0.029	6.7E-06
YPL116W	YPL116W	UWOPS87_Ator08_hmg124h_13.JPG.dat 900.5	47.0	1.1	0.06	0.035	0.056	3.8E-05
YPL119C	YPL119C	UWOPS87_Ator08_hmg124h_13.JPG.dat 862.3	32.9	1.0	0.03	0.002	0.034	1.1E-05
YPL120W	YPL120W	UWOPS87_Ator08_hmg124h_13.JPG.dat 914.5	87.6	1.0	0.03	-0.045	0.031	2.9E-04
YPL121C	YPL121C	UWOPS87_Ator08_hmg124h_13.JPG.dat 782.3	52.2	1.0	0.06	0.001	0.062	7.1E-05
YPL123C	YPL123C	UWOPS87_Ator08_hmg124h_13.JPG.dat 851.3	95.2	1.1	0.10	0.001	0.104	2.6E-04
YPL125W	YPL125W	UWOPS87_Ator08_hmg124h_13.JPG.dat 828.3	57.9	1.0	0.06	0.026	0.060	5.7E-05
YPL127C	YPL127C	UWOPS87_Ator08_hmg124h_13.JPG.dat 813.0	20.8	1.0	0.03	-0.064	0.026	5.3E-06
YPL130W	YPL130W	UWOPS87_Ator08_hmg124h_13.JPG.dat 817.8	20.1	1.0	0.03	-0.013	0.030	7.4E-06
YPL133C	YPL133C	UWOPS87_Ator08_hmg124h_13.JPG.dat 891.8	34.1	1.1	0.04	-0.012	0.035	9.9E-06
YPL134C	YPL134C	UWOPS87_Ator08_hmg124h_13.JPG.dat 902.8	51.6	1.1	0.05	0.033	0.052	3.1E-05
YPL135W	YPL135W	UWOPS87_Ator08_hmg124h_13.JPG.dat 817.0	20.5	1.0	0.03	-0.116	0.026	4.6E-06
YPL136W	YPL136W	UWOPS87_Ator08_hmg124h_13.JPG.dat 775.5	24.0	0.9	0.01	0.042	0.007	2.0E-05
YPL137C	YPL137C	UWOPS87_Ator08_hmg124h_13.JPG.dat 870.0	91.8	1.0	0.11	0.054	0.110	3.4E-04
YPL138C	YPL138C	UWOPS87_Ator08_hmg124h_13.JPG.dat 790.3	18.4	0.9	0.02	-0.066	0.023	4.1E-06
YPL139C	YPL139C	UWOPS87_Ator08_hmg124h_13.JPG.dat 904.8	50.2	1.1	0.07	0.000	0.073	7.5E-05
YPL140C	YPL140C	UWOPS87_Ator08_hmg124h_13.JPG.dat 809.5	6.8	1.0	0.01	-0.118	0.010	2.5E-07
YPL141C	YPL141C	UWOPS87_Ator08_hmg124h_13.JPG.dat 936.3	64.9	1.1	0.08	0.080	0.081	1.0E-04
YPL144W	YPL144W	UWOPS87_Ator08_hmg124h_13.JPG.dat 837.3	191.9	1.0	0.22	-0.006	0.224	3.2E-03
YPL145C	YPL145C	UWOPS87_Ator08_hmg124h_13.JPG.dat 850.3	27.5	1.0	0.03	-0.036	0.034	1.1E-05
YPL147W	YPL147W	UWOPS87_Ator08_hmg124h_13.JPG.dat 896.3	35.5	1.0	0.05	0.002	0.045	2.3E-05
YPL149W	YPL149W	UWOPS87_Ator08_hmg124h_13.JPG.dat 931.5	37.3	1.1	0.04	0.076	0.037	1.1E-05
YPL150W	YPL150W	UWOPS87_Ator08_hmg124h_13.JPG.dat 835.3	65.9	1.0	0.08	0.097	0.084	1.5E-04
YPL152W	YPL152W	UWOPS87_Ator08_hmg124h_13.JPG.dat 832.8	69.4	1.0	0.02	-0.057	0.022	1.7E-04
YPL154C	YPL154C	UWOPS87_Ator08_hmg124h_13.JPG.dat 833.5	23.9	1.0	0.01	0.001	0.010	3.4E-05
YPL155C	YPL155C	UWOPS87_Ator08_hmg124h_13.JPG.dat 816.8	93.8	1.0	0.11	0.015	0.114	4.3E-04
YPL156C	YPL156C	UWOPS87_Ator08_hmg124h_13.JPG.dat 708.0	47.9	0.9	0.06	0.011	0.060	9.3E-05
YPL157W	YPL157W	UWOPS87_Ator08_hmg124h_13.JPG.dat 732.3	95.6	0.9	0.09	0.077	0.093	2.7E-04
YPL159C	YPL159C	UWOPS87_Ator08_hmg124h_13.JPG.dat 832.3	28.3	1.0	0.06	0.101	0.055	4.4E-05
YPL161C	YPL161C	UWOPS87_Ator08_hmg124h_13.JPG.dat 816.8	37.2	1.0	0.02	0.223	0.018	1.8E-06
YPL162C	YPL162C	UWOPS87_Ator08_hmg124h_13.JPG.dat 866.3	57.6	1.0	0.06	0.075	0.058	4.8E-05
YPL163C	YPL163C	UWOPS87_Ator08_hmg124h_13.JPG.dat 810.5	50.1	0.9	0.01	0.081	0.012	5.0E-05
YPL164C	YPL164C	UWOPS87_Ator08_hmg124h_13.JPG.dat 840.3	28.5	1.0	0.00	0.062	0.002	1.1E-06
YPL165C	YPL165C	UWOPS87_Ator08_hmg124h_13.JPG.dat 831.0	62.4	1.0	0.07	-0.031	0.071	9.4E-05
YPL166W	YPL166W	UWOPS87_Ator08_hmg124h_13.JPG.dat 876.5	76.7	1.0	0.06	0.067	0.063	6.0E-05
YPL167C	YPL167C	UWOPS87_Ator08_hmg124h_13.JPG.dat 889.8	29.0	1.0	0.03	-0.017	0.034	1.0E-05
YPL168W	YPL168W	UWOPS87_Ator08_hmg124h_13.JPG.dat 879.5	82.6	1.0	0.09	-0.030	0.087	1.9E-04
YPL170W	YPL170W	UWOPS87_Ator08_hmg124h_13.JPG.dat 848.3	92.4	1.0	0.08	0.024	0.084	1.6E-04
YPL171C	YPL171C	UWOPS87_Ator08_hmg124h_13.JPG.dat 719.3	70.3	1.0	0.10	0.009	0.097	2.7E-04
YPL172C	YPL172C	UWOPS87_Ator08_hmg124h_13.JPG.dat 813.3	39.7	1.0	0.05	0.007	0.049	2.9E-05
YPL174C	YPL174C	UWOPS87_Ator08_hmg124h_13.JPG.dat 799.3	176.7	1.0	0.21	-0.002	0.208	2.5E-03
YPL176C	YPL176C	UWOPS87_Ator08_hmg124h_13.JPG.dat 911.3	79.8	1.1	0.10	0.071	0.098	1.9E-04
YPL177C	YPL177C	UWOPS87_Ator08_hmg124h_13.JPG.dat 875.0	81.3	1.0	0.10	0.009	0.095	2.0E-04
YPL178W	YPL178W	UWOPS87_Ator08_hmg124h_13.JPG.dat 904.0	21.3	1.1	0.03	0.069	0.031	6.5E-06
YPL179W	YPL179W	UWOPS87_Ator08_hmg124h_13.JPG.dat 792.8	31.0	1.0	0.03	0.076	0.031	8.4E-06
YPL180W	YPL180W	UWOPS87_Ator08_hmg124h_13.JPG.dat 818.8	33.6	1.0	0.02	-0.046	0.015	7.9E-05
YPL181W	YPL181W	UWOPS87_Ator08_hmg124h_13.JPG.dat 865.0	18.9	1.0	0.01	0.035	0.014	7.8E-07
YPL182C	YPL182C	UWOPS87_Ator08_hmg124h_13.JPG.dat 770.0	38.3	0.9	0.01	-0.053	0.015	8.5E-05
YPL183C	YPL183C	UWOPS87_Ator08_hmg124h_13.JPG.dat 726.8	69.2	0.9	0.03	0.052	0.028	3.0E-04
YPL183W-/	YPL183W-A	UWOPS87_Ator08_hmg124h_13.JPG.dat 727.3	55.7	1.0	0.08	-0.074	0.077	1.3E-04
YPL184C	YPL184C	UWOPS87_Ator08_hmg124h_13.JPG.dat 772.5	129.3	1.0	0.17	0.013	0.165	1.3E-03
YPL185W	YPL185W	UWOPS87_Ator08_hmg124h_13.JPG.dat 763.8	39.5	1.0	0.01	-0.017	0.008	2.3E-05
YPL186C	YPL186C	UWOPS87_Ator08_hmg124h_13.JPG.dat 817.3	20.8	1.0	0.03	-0.032	0.026	5.4E-06
YPL187W	YPL187W	UWOPS87_Ator08_hmg124h_13.JPG.dat 944.0	17.0	1.1	0.02	0.105	0.024	2.5E-06
YPL189W	YPL189W	UWOPS87_Ator08_hmg124h_13.JPG.dat 865.3	60.6	1.0	0.03	-0.015	0.026	2.3E-04
YPL191C	YPL191C	UWOPS87_Ator08_hmg124h_13.JPG.dat 779.8	52.3	1.0	0.02	-0.010	0.025	2.0E-04
YPL192C	YPL192C	UWOPS87_Ator08_hmg124h_13.JPG.dat 829.0	12.8	1.0	0.01	0.044	0.008	1.9E-05
YPL194W	YPL194W	UWOPS87_Ator08_hmg124h_13.JPG.dat 812.3	46.3	1.0	0.05	0.024	0.050	3.8E-05
YPL195W	YPL195W	UWOPS87_Ator08_hmg124h_13.JPG.dat 725.0	87.4	0.9	0.02	-0.111	0.022	2.0E-04
YPL196W	YPL196W	UWOPS87_Ator08_hmg124h_13.JPG.dat 831.5	36.8	1.0	0.04	-0.107	0.043	2.0E-05
YPL197C	YPL197C	UWOPS87_Ator08_hmg124h_13.JPG.dat 710.5	61.5	0.9	0.08	-0.005	0.082	1.8E-04
YPL198W	YPL198W	UWOPS87_Ator08_hmg124h_13.JPG.dat 820.5	106.7	1.0	0.13	-0.025	0.127	5.6E-04
YPL199C	YPL199C	UWOPS87_Ator08_hmg124h_13.JPG.dat 936.0	52.0	1.1	0.01	0.138	0.007	1.4E-05
YPL200W	YPL200W	UWOPS87_Ator08_hmg124h_13.JPG.dat 893.0	78.1	1.0	0.09	-0.012	0.094	2.0E-04
YPL201C	YPL201C	UWOPS87_Ator08_hmg124h_13.JPG.dat 801.8	25.4	1.0	0.03	0.036	0.031	9.2E-06
YPL202C	YPL202C	UWOPS87_Ator08_hmg124h_13.JPG.dat 912.3	75.1	1.1	0.09	0.000	0.089	1.5E-04
YPL203W	YPL203W	UWOPS87_Ator08_hmg124h_13.JPG.dat 843.3	21.2	1.0	0.03	-0.065	0.027	5.2E-06
YPL205C	YPL205C	UWOPS87_Ator08_hmg124h_13.JPG.dat 814.8	18.4	1.0	0.01	-0.013	0.014	7.8E-07
YPL206C	YPL206C	UWOPS87_Ator08_hmg124h_13.JPG.dat 846.3	31.1	1.0	0.04	-0.026	0.040	1.8E-05

YPL207W	YPL207W	UWOPS87_Ator08_hmg124h_13.JPG.dat	793.0	15.1	0.9	0.02	-0.004	0.018	2.1E-06
YPL208W	YPL208W	UWOPS87_Ator08_hmg124h_13.JPG.dat	854.8	30.9	1.0	0.04	0.010	0.040	1.6E-05
YPL212C	YPL212C	UWOPS87_Ator08_hmg124h_13.JPG.dat	769.8	30.2	1.0	0.04	0.121	0.041	1.7E-05
YPL213W	YPL213W	UWOPS87_Ator08_hmg124h_13.JPG.dat	916.0	53.4	1.1	0.07	0.083	0.068	6.4E-05
YPL214C	YPL214C	UWOPS87_Ator08_hmg124h_13.JPG.dat	960.5	68.2	1.1	0.08	0.104	0.079	9.0E-05
YPL216W	YPL216W	UWOPS87_Ator08_hmg124h_13.JPG.dat	781.8	38.4	1.0	0.04	-0.121	0.043	2.6E-05
YPL219W	YPL219W	UWOPS87_Ator08_hmg124h_13.JPG.dat	787.8	46.8	1.0	0.06	-0.038	0.061	6.8E-05
YPL220W	YPL220W	UWOPS87_Ator08_hmg124h_13.JPG.dat	774.5	19.4	0.9	0.02	0.017	0.024	4.6E-06
YPL221W	YPL221W	UWOPS87_Ator08_hmg124h_13.JPG.dat	794.5	78.3	1.0	0.10	0.001	0.100	2.8E-04
YPL222W	YPL222W	UWOPS87_Ator08_hmg124h_13.JPG.dat	864.5	20.1	1.1	0.03	0.017	0.029	5.5E-06
YPL223C	YPL223C	UWOPS87_Ator08_hmg124h_13.JPG.dat	931.5	61.0	1.1	0.07	0.021	0.070	6.9E-05
YPL224C	YPL224C	UWOPS87_Ator08_hmg124h_13.JPG.dat	867.3	34.1	1.0	0.04	0.023	0.043	2.1E-05
YPL225W	YPL225W	UWOPS87_Ator08_hmg124h_13.JPG.dat	760.8	43.1	0.9	0.05	0.071	0.049	4.0E-05
YPL226W	YPL226W	UWOPS87_Ator08_hmg124h_13.JPG.dat	706.0	32.8	0.9	0.01	-0.008	0.011	5.1E-05
YPL227C	YPL227C	UWOPS87_Ator08_hmg124h_13.JPG.dat	919.3	95.4	1.1	0.12	0.025	0.116	3.0E-04
YPL229W	YPL229W	UWOPS87_Ator08_hmg124h_13.JPG.dat	641.3	335.8	1.0	0.05	-0.037	0.054	1.0E-03
YPL230W	YPL230W	UWOPS87_Ator08_hmg124h_13.JPG.dat	801.0	32.6	1.0	0.01	-0.042	0.011	4.7E-05
YPL232W	YPL232W	UWOPS87_Ator08_hmg124h_13.JPG.dat	852.3	26.7	1.0	0.04	0.013	0.036	1.2E-05
YPL236C	YPL236C	UWOPS87_Ator08_hmg124h_13.JPG.dat	894.0	135.4	1.1	0.15	0.067	0.155	8.6E-04
YPL239W	YPL239W	UWOPS87_Ator08_hmg124h_13.JPG.dat	782.3	54.3	1.0	0.06	-0.086	0.060	7.0E-05
YPL240C	YPL240C	UWOPS87_Ator08_hmg124h_13.JPG.dat	894.0	38.3	1.1	0.04	0.094	0.042	1.6E-05
YPL241C	YPL241C	UWOPS87_Ator08_hmg124h_13.JPG.dat	816.8	37.6	1.0	0.06	0.024	0.055	5.0E-05
YPL244C	YPL244C	UWOPS87_Ator08_hmg124h_13.JPG.dat	884.8	32.3	1.0	0.04	0.106	0.044	2.0E-05
YPL245W	YPL245W	UWOPS87_Ator08_hmg124h_13.JPG.dat	795.5	59.5	1.0	0.08	0.005	0.085	1.8E-04
YPL246C	YPL246C	UWOPS87_Ator08_hmg124h_13.JPG.dat	765.8	23.0	1.0	0.03	-0.075	0.029	7.5E-06
YPL247C	YPL247C	UWOPS87_Ator08_hmg124h_13.JPG.dat	797.8	60.2	1.0	0.02	-0.030	0.024	2.0E-04
YPL248C	YPL248C	UWOPS87_Ator08_hmg124h_13.JPG.dat	887.8	83.2	1.0	0.09	0.010	0.089	1.7E-04
YPL249C	YPL249C	UWOPS87_Ator08_hmg124h_13.JPG.dat	941.3	72.9	1.1	0.09	0.085	0.087	1.4E-04
YPL250C	YPL250C	UWOPS87_Ator08_hmg124h_13.JPG.dat	865.8	28.0	1.0	0.03	0.037	0.032	8.7E-06
YPL253C	YPL253C	UWOPS87_Ator08_hmg124h_13.JPG.dat	791.0	86.6	0.9	0.10	-0.051	0.101	3.7E-04
YPL256C	YPL256C	UWOPS87_Ator08_hmg124h_13.JPG.dat	836.0	6.6	1.0	0.01	0.091	0.015	8.9E-07
YPL257W	YPL257W	UWOPS87_Ator08_hmg124h_13.JPG.dat	806.8	42.4	1.0	0.05	-0.109	0.046	2.8E-05
YPL258C	YPL258C	UWOPS87_Ator08_hmg124h_13.JPG.dat	806.5	83.4	1.0	0.02	0.077	0.022	1.5E-04
YPL259C	YPL259C	UWOPS87_Ator08_hmg124h_13.JPG.dat	723.5	65.9	0.9	0.07	-0.121	0.073	1.6E-04
YPL260W	YPL260W	UWOPS87_Ator08_hmg124h_13.JPG.dat	967.3	84.9	1.2	0.03	0.227	0.027	1.6E-04
1	1	UWOPS87_Ator08_hmg124h_14.JPG.dat	1368.8	297.7	#NUM!		#NUM!		
2	2	UWOPS87_Ator08_hmg124h_14.JPG.dat	939.0	139.6	1.0	0.12	0.120	0.124	4.9E-04
3	3	UWOPS87_Ator08_hmg124h_14.JPG.dat	979.8	180.5	1.1	0.04	0.056	0.040	4.2E-04
4	4	UWOPS87_Ator08_hmg124h_14.JPG.dat	934.8	67.3	0.9	0.01	-0.008	0.013	6.3E-05
YAL024C	YAL024C	UWOPS87_Ator08_hmg124h_14.JPG.dat	695.5	86.6	0.8	0.10	-0.135	0.100	4.9E-04
YAL046C	YAL046C	UWOPS87_Ator08_hmg124h_14.JPG.dat	827.5	20.5	1.0	0.00	-0.101	0.004	5.7E-06
YAL048C	YAL048C	UWOPS87_Ator08_hmg124h_14.JPG.dat	830.0	23.9	1.0	0.01	-0.096	0.006	1.3E-05
YBL103C	YBL103C	UWOPS87_Ator08_hmg124h_14.JPG.dat	769.8	10.6	0.9	0.01	-0.017	0.010	4.5E-07
YBR020W	YBR020W	UWOPS87_Ator08_hmg124h_14.JPG.dat	894.8	48.0	1.1	0.05	-0.042	0.051	2.6E-05
YBR061C	YBR061C	UWOPS87_Ator08_hmg124h_14.JPG.dat	659.5	442.1	1.1	0.05	-0.073	0.045	6.1E-04
YBR075W	YBR075W	UWOPS87_Ator08_hmg124h_14.JPG.dat	775.5	62.8	0.9	0.06	-0.020	0.059	6.5E-05
YBR078W	YBR078W	UWOPS87_Ator08_hmg124h_14.JPG.dat	1026.5	26.7	1.0	0.01	-0.014	0.012	4.6E-05
YBR082C	YBR082C	UWOPS87_Ator08_hmg124h_14.JPG.dat	802.0	34.4	0.9	0.05	0.090	0.049	3.9E-05
YBR085W	YBR085W	UWOPS87_Ator08_hmg124h_14.JPG.dat	685.0	457.7	1.1	0.03	-0.085	0.028	2.2E-04
YBR086C	YBR086C	UWOPS87_Ator08_hmg124h_14.JPG.dat	896.0	42.8	1.1	0.06	0.109	0.065	5.6E-05
YBR095C	YBR095C	UWOPS87_Ator08_hmg124h_14.JPG.dat	789.3	36.2	1.0	0.05	-0.032	0.048	3.2E-05
YBR115C	YBR115C	UWOPS87_Ator08_hmg124h_14.JPG.dat	661.0	44.3	0.8	0.01	-0.232	0.007	2.9E-05
YBR118W	YBR118W	UWOPS87_Ator08_hmg124h_14.JPG.dat	986.3	47.7	1.2	0.05	0.142	0.053	2.5E-05
YBR165W	YBR165W	UWOPS87_Ator08_hmg124h_14.JPG.dat	915.0	49.5	1.1	0.05	0.032	0.049	2.6E-05
YBR299W	YBR299W	UWOPS87_Ator08_hmg124h_14.JPG.dat	846.8	13.8	1.0	0.02	-0.020	0.020	2.7E-06
YCR107W	YCR107W	UWOPS87_Ator08_hmg124h_14.JPG.dat	887.3	71.1	1.0	0.08	0.035	0.076	1.1E-04
YDL074C	YDL074C	UWOPS87_Ator08_hmg124h_14.JPG.dat	860.0	23.1	1.0	0.03	-0.112	0.029	6.5E-06
YDR026C	YDR026C	UWOPS87_Ator08_hmg124h_14.JPG.dat	730.3	51.9	0.9	0.07	-0.083	0.066	1.0E-04
YDR029W	YDR029W	UWOPS87_Ator08_hmg124h_14.JPG.dat	781.5	20.4	1.0	0.01	-0.070	0.007	2.0E-05
YDR030C	YDR030C	UWOPS87_Ator08_hmg124h_14.JPG.dat	908.5	157.7	1.0	0.04	-0.203	0.039	4.7E-04
YDR031W	YDR031W	UWOPS87_Ator08_hmg124h_14.JPG.dat	752.5	51.7	0.9	0.06	0.043	0.065	8.8E-05
YDR032C	YDR032C	UWOPS87_Ator08_hmg124h_14.JPG.dat	845.3	76.9	1.0	0.09	0.011	0.086	1.8E-04
YDR033W	YDR033W	UWOPS87_Ator08_hmg124h_14.JPG.dat	822.5	92.1	1.0	0.11	0.040	0.110	3.7E-04
YDR034C	YDR034C	UWOPS87_Ator08_hmg124h_14.JPG.dat	708.5	121.9	1.0	0.04	-0.034	0.036	4.5E-04
YDR035W	YDR035W	UWOPS87_Ator08_hmg124h_14.JPG.dat	733.3	11.1	0.9	0.00	-0.061	0.000	4.1E-08
YDR036C	YDR036C	UWOPS87_Ator08_hmg124h_14.JPG.dat	850.8	29.9	1.0	0.04	-0.028	0.037	1.4E-05
YDR042C	YDR042C	UWOPS87_Ator08_hmg124h_14.JPG.dat	762.0	46.0	0.9	0.01	0.092	0.014	7.2E-05
YDR043C	YDR043C	UWOPS87_Ator08_hmg124h_14.JPG.dat	914.8	41.3	1.1	0.02	-0.071	0.016	7.1E-05
YDR046C	YDR046C	UWOPS87_Ator08_hmg124h_14.JPG.dat	850.8	69.3	1.0	0.08	0.020	0.082	1.3E-04
YDR242W	YDR242W	UWOPS87_Ator08_hmg124h_14.JPG.dat	871.8	43.6	1.0	0.06	-0.011	0.060	5.2E-05

YDR269C	YDR269C	UWOPS87_Ator08_hmg124h_14.JPG.dat	888.8	42.7	1.1	0.02	-0.040	0.018	8.3E-05
YDR271C	YDR271C	UWOPS87_Ator08_hmg124h_14.JPG.dat	848.5	79.6	1.0	0.09	-0.152	0.087	1.9E-04
YDR290W	YDR290W	UWOPS87_Ator08_hmg124h_14.JPG.dat	866.8	32.6	1.0	0.01	-0.160	0.011	3.4E-05
YDR326C	YDR326C	UWOPS87_Ator08_hmg124h_14.JPG.dat	846.3	15.8	1.0	0.02	0.033	0.021	2.4E-06
YDR444W	YDR444W	UWOPS87_Ator08_hmg124h_14.JPG.dat	855.5	50.2	1.0	0.06	0.101	0.060	5.4E-05
YDR461W	YDR461W	UWOPS87_Ator08_hmg124h_14.JPG.dat	832.0	95.8	1.0	0.12	0.083	0.116	3.7E-04
YDR477W	YDR477W	UWOPS87_Ator08_hmg124h_14.JPG.dat	824.5	15.5	1.0	0.02	-0.036	0.015	9.2E-07
YDR483W	YDR483W	UWOPS87_Ator08_hmg124h_14.JPG.dat	771.0	66.0	1.0	0.01	0.172	0.008	1.9E-05
YDR493W	YDR493W	UWOPS87_Ator08_hmg124h_14.JPG.dat	670.0	61.8	0.9	0.08	-0.028	0.079	2.0E-04
YDR502C	YDR502C	UWOPS87_Ator08_hmg124h_14.JPG.dat	829.3	83.6	1.0	0.11	0.091	0.105	3.0E-04
YDR515W	YDR515W	UWOPS87_Ator08_hmg124h_14.JPG.dat	798.5	50.5	1.0	0.06	0.006	0.063	7.2E-05
YER089C	YER089C	UWOPS87_Ator08_hmg124h_14.JPG.dat	936.8	63.8	1.1	0.01	0.019	0.011	3.3E-05
YFL001W	YFL001W	UWOPS87_Ator08_hmg124h_14.JPG.dat	936.8	73.7	1.1	0.06	-0.116	0.065	6.2E-05
YFL003C	YFL003C	UWOPS87_Ator08_hmg124h_14.JPG.dat	910.0	55.0	1.0	0.06	-0.079	0.059	5.1E-05
YFL004W	YFL004W	UWOPS87_Ator08_hmg124h_14.JPG.dat	739.0	86.9	0.8	0.09	0.015	0.092	3.6E-04
YFL007W	YFL007W	UWOPS87_Ator08_hmg124h_14.JPG.dat	856.3	70.8	1.0	0.07	-0.027	0.067	8.9E-05
YFL010C	YFL010C	UWOPS87_Ator08_hmg124h_14.JPG.dat	894.0	25.5	1.0	0.02	0.129	0.018	1.6E-06
YFL010W-/	YFL010W-A	UWOPS87_Ator08_hmg124h_14.JPG.dat	932.0	28.1	1.0	0.02	-0.047	0.021	2.5E-06
YFL012W	YFL012W	UWOPS87_Ator08_hmg124h_14.JPG.dat	946.3	31.0	1.0	0.02	-0.127	0.024	3.8E-06
YFL013C	YFL013C	UWOPS87_Ator08_hmg124h_14.JPG.dat	896.3	54.0	1.0	0.01	0.178	0.012	4.9E-05
YFL033C	YFL033C	UWOPS87_Ator08_hmg124h_14.JPG.dat	889.8	70.9	1.1	0.01	-0.272	0.011	3.4E-05
YFL063W	YFL063W	UWOPS87_Ator08_hmg124h_14.JPG.dat	788.3	52.0	1.0	0.06	-0.269	0.060	6.6E-05
YGL029W	YGL029W	UWOPS87_Ator08_hmg124h_14.JPG.dat	831.5	16.6	1.0	0.02	0.111	0.019	1.8E-06
YGL037C	YGL037C	UWOPS87_Ator08_hmg124h_14.JPG.dat	851.5	13.5	1.0	0.01	-0.075	0.006	1.1E-05
YGL042C	YGL042C	UWOPS87_Ator08_hmg124h_14.JPG.dat	895.0	27.8	1.0	0.04	-0.009	0.042	2.2E-05
YGL049C	YGL049C	UWOPS87_Ator08_hmg124h_14.JPG.dat	969.0	60.9	1.1	0.08	0.033	0.077	8.7E-05
YGL063W	YGL063W	UWOPS87_Ator08_hmg124h_14.JPG.dat	863.0	33.5	0.9	0.04	-0.029	0.040	2.0E-05
YGL071W	YGL071W	UWOPS87_Ator08_hmg124h_14.JPG.dat	944.8	19.5	1.0	0.04	0.061	0.043	1.9E-05
YGL094C	YGL094C	UWOPS87_Ator08_hmg124h_14.JPG.dat	898.3	35.0	1.0	0.05	-0.050	0.053	3.7E-05
YGL096W	YGL096W	UWOPS87_Ator08_hmg124h_14.JPG.dat	789.0	124.4	0.9	0.13	-0.021	0.132	8.2E-04
YGR055W	YGR055W	UWOPS87_Ator08_hmg124h_14.JPG.dat	905.8	69.6	1.0	0.07	0.019	0.073	9.6E-05
YGR061C	YGR061C	UWOPS87_Ator08_hmg124h_14.JPG.dat	902.8	104.5	1.0	0.10	0.087	0.104	3.0E-04
YGR067C	YGR067C	UWOPS87_Ator08_hmg124h_14.JPG.dat	902.0	30.3	1.0	0.05	-0.125	0.048	2.9E-05
YGR068C	YGR068C	UWOPS87_Ator08_hmg124h_14.JPG.dat	846.3	73.0	1.0	0.07	-0.156	0.066	9.2E-05
YGR122C-/	YGR122C-A	UWOPS87_Ator08_hmg124h_14.JPG.dat	884.0	36.4	1.0	0.06	-0.065	0.063	7.3E-05
YGR122W	YGR122W	UWOPS87_Ator08_hmg124h_14.JPG.dat	907.0	31.1	1.0	0.03	0.047	0.027	5.0E-06
YGR254W	YGR254W	UWOPS87_Ator08_hmg124h_14.JPG.dat	880.0	47.4	1.0	0.07	0.041	0.070	1.0E-04
YGR255C	YGR255C	UWOPS87_Ator08_hmg124h_14.JPG.dat	923.8	44.3	1.0	0.04	-0.100	0.038	1.4E-05
YGR271W	YGR271W	UWOPS87_Ator08_hmg124h_14.JPG.dat	817.3	53.2	1.0	0.05	0.077	0.048	3.2E-05
YGR272C	YGR272C	UWOPS87_Ator08_hmg124h_14.JPG.dat	998.0	29.4	1.2	0.05	-0.032	0.047	1.7E-05
YGR273C	YGR273C	UWOPS87_Ator08_hmg124h_14.JPG.dat	836.3	43.4	1.0	0.02	0.017	0.016	7.8E-05
YGR276C	YGR276C	UWOPS87_Ator08_hmg124h_14.JPG.dat	884.5	55.7	1.1	0.07	0.169	0.067	6.8E-05
YGR289C	YGR289C	UWOPS87_Ator08_hmg124h_14.JPG.dat	848.3	38.5	1.0	0.06	-0.120	0.060	5.6E-05
YGR291C	YGR291C	UWOPS87_Ator08_hmg124h_14.JPG.dat	812.8	46.3	1.0	0.05	0.020	0.047	3.1E-05
YGR292W	YGR292W	UWOPS87_Ator08_hmg124h_14.JPG.dat	848.0	57.3	1.0	0.02	0.133	0.018	1.0E-04
YHR008C	YHR008C	UWOPS87_Ator08_hmg124h_14.JPG.dat	459.0	73.0	0.5	0.03	-0.180	0.025	7.8E-04
YIL085C	YIL085C	UWOPS87_Ator08_hmg124h_14.JPG.dat	722.0	22.0	0.9	0.02	-0.094	0.021	3.6E-06
YIL094C	YIL094C	UWOPS87_Ator08_hmg124h_14.JPG.dat	800.0	63.1	1.0	0.08	0.050	0.076	1.2E-04
YIL102C	YIL102C	UWOPS87_Ator08_hmg124h_14.JPG.dat	745.0	26.4	0.9	0.04	-0.324	0.038	1.9E-05
YIL111W	YIL111W	UWOPS87_Ator08_hmg124h_14.JPG.dat	850.3	330.2	0.9	0.05	-0.026	0.052	1.2E-03
YIL122W	YIL122W	UWOPS87_Ator08_hmg124h_14.JPG.dat	909.0	50.1	1.1	0.06	0.053	0.063	4.6E-05
YIL125W	YIL125W	UWOPS87_Ator08_hmg124h_14.JPG.dat	806.5	79.8	1.0	0.09	-0.021	0.089	2.2E-04
YIL131C	YIL131C	UWOPS87_Ator08_hmg124h_14.JPG.dat	809.3	52.5	1.0	0.06	0.064	0.064	7.0E-05
YIL136W	YIL136W	UWOPS87_Ator08_hmg124h_14.JPG.dat	850.5	22.2	1.1	0.03	0.042	0.031	6.8E-06
YIL151C	YIL151C	UWOPS87_Ator08_hmg124h_14.JPG.dat	810.0	33.9	1.0	0.05	-0.063	0.054	4.6E-05
YIL158W	YIL158W	UWOPS87_Ator08_hmg124h_14.JPG.dat	818.3	9.4	1.0	0.01	-0.022	0.013	6.3E-07
YIR004W	YIR004W	UWOPS87_Ator08_hmg124h_14.JPG.dat	874.0	48.5	1.1	0.02	0.109	0.020	1.2E-04
YJL029C	YJL029C	UWOPS87_Ator08_hmg124h_14.JPG.dat	839.3	121.1	1.1	0.15	0.038	0.152	8.1E-04
YJL079C	YJL079C	UWOPS87_Ator08_hmg124h_14.JPG.dat	13.5	27.0	0.0	0.00	0.000	0.000	
YJL082W	YJL082W	UWOPS87_Ator08_hmg124h_14.JPG.dat	802.8	65.5	1.0	0.06	-0.025	0.061	7.3E-05
YJL092W	YJL092W	UWOPS87_Ator08_hmg124h_14.JPG.dat	797.5	19.6	1.0	0.04	-0.129	0.040	2.0E-05
YJL103C	YJL103C	UWOPS87_Ator08_hmg124h_14.JPG.dat	790.3	28.2	1.0	0.01	-0.061	0.012	4.6E-05
YJL106W	YJL106W	UWOPS87_Ator08_hmg124h_14.JPG.dat	878.8	83.2	1.1	0.08	-0.066	0.081	1.2E-04
YJL107C	YJL107C	UWOPS87_Ator08_hmg124h_14.JPG.dat	870.3	88.5	1.1	0.02	0.110	0.017	8.4E-05
YJL117W	YJL117W	UWOPS87_Ator08_hmg124h_14.JPG.dat	878.8	93.0	1.0	0.08	0.038	0.084	1.5E-04
YJL119C	YJL119C	UWOPS87_Ator08_hmg124h_14.JPG.dat	890.3	54.4	1.0	0.06	-0.094	0.064	6.2E-05
YJL120W	YJL120W	UWOPS87_Ator08_hmg124h_14.JPG.dat	664.3	57.1	0.8	0.05	-0.055	0.047	5.8E-05
YJL177W	YJL177W	UWOPS87_Ator08_hmg124h_14.JPG.dat	850.0	10.9	0.9	0.03	0.182	0.033	1.2E-05
YJR034W	YJR034W	UWOPS87_Ator08_hmg124h_14.JPG.dat	884.0	33.5	1.1	0.04	0.006	0.040	1.5E-05
YJR039W	YJR039W	UWOPS87_Ator08_hmg124h_14.JPG.dat	736.8	54.2	0.9	0.00	0.062	0.002	1.8E-06

YJR044C	YJR044C	UWOPS87_Ator08_hmg124h_14.JPG.dat 872.3	37.0	1.1	0.03	-0.191	0.029	5.7E-06
YJR066W	YJR066W	UWOPS87_Ator08_hmg124h_14.JPG.dat 888.5	26.5	1.0	0.00	-0.130	0.000	6.5E-08
YKL087C	YKL087C	UWOPS87_Ator08_hmg124h_14.JPG.dat 865.0	40.7	1.0	0.03	-0.071	0.034	1.0E-05
YKR104W	YKR104W	UWOPS87_Ator08_hmg124h_14.JPG.dat 882.0	42.8	1.0	0.02	0.125	0.016	7.8E-05
YLR227C	YLR227C	UWOPS87_Ator08_hmg124h_14.JPG.dat 864.8	48.5	1.1	0.06	0.074	0.059	4.4E-05
YLR238W	YLR238W	UWOPS87_Ator08_hmg124h_14.JPG.dat 941.8	34.6	1.2	0.04	0.111	0.041	1.2E-05
YLR308W	YLR308W	UWOPS87_Ator08_hmg124h_14.JPG.dat 787.0	81.3	1.0	0.03	0.040	0.030	3.0E-04
YLR455W	YLR455W	UWOPS87_Ator08_hmg124h_14.JPG.dat 901.3	42.7	1.1	0.05	-0.135	0.045	2.2E-05
YML050W	YML050W	UWOPS87_Ator08_hmg124h_14.JPG.dat 808.5	121.3	0.9	0.05	-0.131	0.051	1.1E-03
YML111W	YML111W	UWOPS87_Ator08_hmg124h_14.JPG.dat 826.8	35.0	1.0	0.07	0.108	0.066	8.8E-05
YML112W	YML112W	UWOPS87_Ator08_hmg124h_14.JPG.dat 962.3	44.4	1.1	0.03	0.027	0.035	7.6E-06
YML129C	YML129C	UWOPS87_Ator08_hmg124h_14.JPG.dat 487.5	283.3	0.6	0.33	-0.059	0.329	4.1E-02
YMR074C	YMR074C	UWOPS87_Ator08_hmg124h_14.JPG.dat 815.5	42.0	1.0	0.05	0.097	0.048	2.9E-05
YMR118C	YMR118C	UWOPS87_Ator08_hmg124h_14.JPG.dat 840.8	72.4	1.0	0.08	-0.023	0.076	1.2E-04
YMR119W	YMR119W	UWOPS87_Ator08_hmg124h_14.JPG.dat 849.5	31.2	1.0	0.03	-0.034	0.033	1.0E-05
YMR165C	YMR165C	UWOPS87_Ator08_hmg124h_14.JPG.dat 194.0	234.0	0.2	0.28	-0.166	0.281	2.0E-01
YMR194C-.	YMR194C-A	UWOPS87_Ator08_hmg124h_14.JPG.dat 770.0	120.0	0.9	0.01	-0.109	0.008	3.0E-05
YNL011C	YNL011C	UWOPS87_Ator08_hmg124h_14.JPG.dat 784.3	58.5	1.0	0.07	-0.091	0.071	1.0E-04
YNL014W	YNL014W	UWOPS87_Ator08_hmg124h_14.JPG.dat 785.5	51.3	1.0	0.07	0.161	0.069	9.4E-05
YNL042W	YNL042W	UWOPS87_Ator08_hmg124h_14.JPG.dat 818.0	46.2	1.0	0.07	-0.055	0.072	1.1E-04
YNL047C	YNL047C	UWOPS87_Ator08_hmg124h_14.JPG.dat 853.8	46.2	1.1	0.06	0.051	0.060	5.0E-05
YNL053W	YNL053W	UWOPS87_Ator08_hmg124h_14.JPG.dat 881.0	44.3	1.1	0.06	0.178	0.058	4.2E-05
YNL086W	YNL086W	UWOPS87_Ator08_hmg124h_14.JPG.dat 720.0	25.8	0.9	0.03	-0.093	0.034	1.5E-05
YNL096C	YNL096C	UWOPS87_Ator08_hmg124h_14.JPG.dat 845.3	101.7	1.0	0.11	0.108	0.112	4.1E-04
YNL109W	YNL109W	UWOPS87_Ator08_hmg124h_14.JPG.dat 823.3	84.4	1.0	0.09	0.112	0.094	2.6E-04
YNL111C	YNL111C	UWOPS87_Ator08_hmg124h_14.JPG.dat 828.5	58.1	1.0	0.01	0.034	0.007	1.4E-05
YNL147W	YNL147W	UWOPS87_Ator08_hmg124h_14.JPG.dat 712.8	111.6	0.9	0.12	0.089	0.125	7.5E-04
YNL180C	YNL180C	UWOPS87_Ator08_hmg124h_14.JPG.dat 853.3	90.0	1.0	0.01	0.076	0.014	6.4E-05
YNL284C	YNL284C	UWOPS87_Ator08_hmg124h_14.JPG.dat 786.3	17.1	1.0	0.02	-0.182	0.021	2.8E-06
YNR033W	YNR033W	UWOPS87_Ator08_hmg124h_14.JPG.dat 841.3	18.3	1.0	0.02	-0.028	0.023	3.5E-06
YNR041C	YNR041C	UWOPS87_Ator08_hmg124h_14.JPG.dat 771.5	59.3	1.0	0.03	-0.038	0.026	2.4E-04
YNR044W	YNR044W	UWOPS87_Ator08_hmg124h_14.JPG.dat 424.8	498.3	0.5	0.58	-0.039	0.583	1.9E-01
YOL073C	YOL073C	UWOPS87_Ator08_hmg124h_14.JPG.dat 772.3	25.0	0.9	0.01	-0.050	0.009	3.3E-05
YOL086C	YOL086C	UWOPS87_Ator08_hmg124h_14.JPG.dat 777.5	82.4	1.0	0.10	0.114	0.099	2.8E-04
YOL153C	YOL153C	UWOPS87_Ator08_hmg124h_14.JPG.dat 672.0	22.6	0.8	0.03	0.033	0.033	1.6E-05
YOR054C	YOR054C	UWOPS87_Ator08_hmg124h_14.JPG.dat 795.3	70.1	0.9	0.01	-0.111	0.013	6.6E-05
YOR125C	YOR125C	UWOPS87_Ator08_hmg124h_14.JPG.dat 833.5	23.4	1.0	0.02	-0.020	0.021	2.5E-06
YOR128C	YOR128C	UWOPS87_Ator08_hmg124h_14.JPG.dat 830.0	77.9	1.0	0.03	-0.036	0.028	2.7E-04
YOR155C	YOR155C	UWOPS87_Ator08_hmg124h_14.JPG.dat 932.0	125.6	1.1	0.15	-0.105	0.148	7.1E-04
YOR158W	YOR158W	UWOPS87_Ator08_hmg124h_14.JPG.dat 835.8	57.8	1.0	0.07	-0.069	0.069	9.7E-05
YOR179C	YOR179C	UWOPS87_Ator08_hmg124h_14.JPG.dat 834.5	71.6	1.0	0.08	0.014	0.077	1.3E-04
YOR180C	YOR180C	UWOPS87_Ator08_hmg124h_14.JPG.dat 746.3	68.1	0.9	0.08	-0.102	0.077	1.9E-04
YOR202W	YOR202W	UWOPS87_Ator08_hmg124h_14.JPG.dat 948.6	143.5	1.0	0.08	-0.003	0.076	1.3833838(
YOR220W	YOR220W	UWOPS87_Ator08_hmg124h_14.JPG.dat 958.0	31.9	1.2	0.03	-0.134	0.029	4.4E-06
YOR240W	YOR240W	UWOPS87_Ator08_hmg124h_14.JPG.dat 882.0	40.1	1.1	0.05	0.205	0.051	2.8E-05
YOR248W	YOR248W	UWOPS87_Ator08_hmg124h_14.JPG.dat 798.5	50.4	0.9	0.02	0.074	0.020	1.6E-04
YOR300W	YOR300W	UWOPS87_Ator08_hmg124h_14.JPG.dat 909.5	94.9	1.0	0.03	0.097	0.026	2.1E-04
YOR306C	YOR306C	UWOPS87_Ator08_hmg124h_14.JPG.dat 931.8	54.6	1.1	0.02	0.146	0.019	1.0E-04
YOR309C	YOR309C	UWOPS87_Ator08_hmg124h_14.JPG.dat 763.8	22.2	0.9	0.01	-0.016	0.013	9.1E-07
YOR325W	YOR325W	UWOPS87_Ator08_hmg124h_14.JPG.dat 909.5	14.9	1.0	0.02	0.016	0.015	8.9E-07
YOR333C	YOR333C	UWOPS87_Ator08_hmg124h_14.JPG.dat 775.5	61.7	0.9	0.07	-0.102	0.073	1.5E-04
YOR345C	YOR345C	UWOPS87_Ator08_hmg124h_14.JPG.dat 773.0	92.5	0.9	0.11	-0.281	0.108	4.3E-04
YOR366W	YOR366W	UWOPS87_Ator08_hmg124h_14.JPG.dat 814.5	50.9	1.0	0.05	0.053	0.049	3.5E-05
YOR379C	YOR379C	UWOPS87_Ator08_hmg124h_14.JPG.dat 924.3	11.0	1.1	0.01	-0.066	0.008	1.7E-05
YPL015C	YPL015C	UWOPS87_Ator08_hmg124h_14.JPG.dat 810.8	32.1	1.0	0.01	-0.221	0.015	7.1E-05
YPL035C	YPL035C	UWOPS87_Ator08_hmg124h_14.JPG.dat 796.8	12.0	1.0	0.00	-0.061	0.002	1.2E-06
YPL072W	YPL072W	UWOPS87_Ator08_hmg124h_14.JPG.dat 234.0	270.9	0.3	0.34	-0.068	0.338	1.8E-01
YPL158C	YPL158C	UWOPS87_Ator08_hmg124h_14.JPG.dat 737.5	9.4	0.9	0.01	-0.122	0.013	8.3E-07
YPL261C	YPL261C	UWOPS87_Ator08_hmg124h_14.JPG.dat 979.0	48.1	1.1	0.02	0.130	0.017	8.0E-05
YPL262W	YPL262W	UWOPS87_Ator08_hmg124h_14.JPG.dat 809.3	47.0	0.9	0.06	-0.130	0.058	6.2E-05
YPL263C	YPL263C	UWOPS87_Ator08_hmg124h_14.JPG.dat 733.3	61.6	0.9	0.02	0.005	0.022	2.1E-04
YPL264C	YPL264C	UWOPS87_Ator08_hmg124h_14.JPG.dat 777.0	81.6	0.9	0.09	-0.124	0.091	2.6E-04
YPL265W	YPL265W	UWOPS87_Ator08_hmg124h_14.JPG.dat 891.3	66.2	1.1	0.08	0.016	0.079	1.1E-04
YPL267W	YPL267W	UWOPS87_Ator08_hmg124h_14.JPG.dat 880.3	42.5	1.0	0.05	0.048	0.050	3.0E-05
YPL269W	YPL269W	UWOPS87_Ator08_hmg124h_14.JPG.dat 851.8	28.5	1.0	0.03	-0.098	0.035	1.1E-05
YPL270W	YPL270W	UWOPS87_Ator08_hmg124h_14.JPG.dat 853.3	28.4	1.0	0.03	-0.127	0.027	5.3E-06
YPL272C	YPL272C	UWOPS87_Ator08_hmg124h_14.JPG.dat 907.5	9.7	1.0	0.01	-0.077	0.014	6.9E-07
YPL273W	YPL273W	UWOPS87_Ator08_hmg124h_14.JPG.dat 932.0	43.6	1.1	0.06	-0.021	0.055	3.7E-05
YPL274W	YPL274W	UWOPS87_Ator08_hmg124h_14.JPG.dat 401.8	473.2	0.5	0.56	-0.023	0.560	1.9E-01
YPR001W	YPR001W	UWOPS87_Ator08_hmg124h_14.JPG.dat 897.8	32.5	1.1	0.04	0.123	0.042	1.5E-05

YPR002W	YPR002W	UWOPS87_Ator08_hmg124h_14.JPG.dat	858.3	84.3	1.1	0.01	-0.010	0.007	1.3E-05
YPR003C	YPR003C	UWOPS87_Ator08_hmg124h_14.JPG.dat	743.8	65.1	1.0	0.02	0.009	0.024	1.9E-04
YPR004C	YPR004C	UWOPS87_Ator08_hmg124h_14.JPG.dat	763.3	41.6	1.0	0.01	-0.181	0.013	5.5E-05
YPR005C	YPR005C	UWOPS87_Ator08_hmg124h_14.JPG.dat	772.8	53.3	1.0	0.07	0.092	0.065	8.3E-05
YPR006C	YPR006C	UWOPS87_Ator08_hmg124h_14.JPG.dat	739.3	64.9	0.9	0.09	0.063	0.088	2.4E-04
YPR007C	YPR007C	UWOPS87_Ator08_hmg124h_14.JPG.dat	798.0	58.5	1.0	0.08	-0.066	0.075	1.2E-04
YPR008W	YPR008W	UWOPS87_Ator08_hmg124h_14.JPG.dat	632.5	34.5	0.8	0.04	-0.092	0.043	4.2E-05
YPR009W	YPR009W	UWOPS87_Ator08_hmg124h_14.JPG.dat	885.3	145.3	1.0	0.03	-0.111	0.033	3.8E-04
YPR011C	YPR011C	UWOPS87_Ator08_hmg124h_14.JPG.dat	809.0	42.8	1.0	0.05	-0.184	0.053	4.1E-05
YPR012W	YPR012W	UWOPS87_Ator08_hmg124h_14.JPG.dat	700.5	76.6	0.9	0.09	-0.039	0.092	3.5E-04
YPR013C	YPR013C	UWOPS87_Ator08_hmg124h_14.JPG.dat	665.3	59.4	0.8	0.01	0.033	0.011	5.7E-05
YPR014C	YPR014C	UWOPS87_Ator08_hmg124h_14.JPG.dat	1003.5	95.4	1.2	0.12	0.029	0.119	2.5E-04
YPR015C	YPR015C	UWOPS87_Ator08_hmg124h_14.JPG.dat	849.5	40.6	1.0	0.05	-0.190	0.050	3.2E-05
YPR017C	YPR017C	UWOPS87_Ator08_hmg124h_14.JPG.dat	691.0	31.7	0.9	0.01	-0.094	0.014	8.4E-05
YPR018W	YPR018W	UWOPS87_Ator08_hmg124h_14.JPG.dat	890.5	37.5	1.1	0.01	0.257	0.011	3.2E-05
YPR020W	YPR020W	UWOPS87_Ator08_hmg124h_14.JPG.dat	665.8	62.8	0.8	0.08	-0.104	0.078	2.3E-04
YPR021C	YPR021C	UWOPS87_Ator08_hmg124h_14.JPG.dat	622.5	38.7	0.8	0.05	-0.003	0.052	8.2E-05
YPR022C	YPR022C	UWOPS87_Ator08_hmg124h_14.JPG.dat	768.0	89.8	1.0	0.04	0.131	0.039	4.9E-04
YPR023C	YPR023C	UWOPS87_Ator08_hmg124h_14.JPG.dat	705.8	99.0	0.9	0.12	0.064	0.122	7.4E-04
YPR024W	YPR024W	UWOPS87_Ator08_hmg124h_14.JPG.dat	853.8	25.7	1.1	0.03	-0.005	0.027	4.7E-06
YPR026W	YPR026W	UWOPS87_Ator08_hmg124h_14.JPG.dat	891.5	34.3	1.0	0.04	-0.077	0.045	2.2E-05
YPR027C	YPR027C	UWOPS87_Ator08_hmg124h_14.JPG.dat	911.0	41.6	1.1	0.05	-0.031	0.047	2.3E-05
YPR028W	YPR028W	UWOPS87_Ator08_hmg124h_14.JPG.dat	818.8	46.4	1.0	0.02	0.091	0.020	1.4E-04
YPR029C	YPR029C	UWOPS87_Ator08_hmg124h_14.JPG.dat	639.5	68.4	0.7	0.03	-0.172	0.026	4.3E-04
YPR030W	YPR030W	UWOPS87_Ator08_hmg124h_14.JPG.dat	828.0	16.9	1.0	0.01	-0.006	0.010	2.6E-07
YPR031W	YPR031W	UWOPS87_Ator08_hmg124h_14.JPG.dat	769.3	40.4	0.9	0.04	-0.013	0.037	1.8E-05
YPR032W	YPR032W	UWOPS87_Ator08_hmg124h_14.JPG.dat	946.0	33.8	1.1	0.01	0.048	0.010	2.7E-05
YPR037C	YPR037C	UWOPS87_Ator08_hmg124h_14.JPG.dat	852.3	42.8	1.0	0.06	-0.022	0.059	5.3E-05
YPR038W	YPR038W	UWOPS87_Ator08_hmg124h_14.JPG.dat	713.5	81.8	0.9	0.11	0.032	0.110	5.5E-04
YPR039W	YPR039W	UWOPS87_Ator08_hmg124h_14.JPG.dat	856.3	29.1	1.1	0.01	0.121	0.011	3.5E-05
YPR040W	YPR040W	UWOPS87_Ator08_hmg124h_14.JPG.dat	669.8	368.3	1.0	0.10	-0.032	0.101	3.2E-03
YPR042C	YPR042C	UWOPS87_Ator08_hmg124h_14.JPG.dat	775.8	62.4	0.9	0.08	0.036	0.076	1.4E-04
YPR043W	YPR043W	UWOPS87_Ator08_hmg124h_14.JPG.dat	947.3	71.3	1.1	0.07	0.200	0.073	8.5E-05
YPR044C	YPR044C	UWOPS87_Ator08_hmg124h_14.JPG.dat	856.0	15.2	1.0	0.02	-0.046	0.016	1.1E-06
YPR045C	YPR045C	UWOPS87_Ator08_hmg124h_14.JPG.dat	876.3	202.0	1.0	0.22	0.037	0.220	2.9E-03
YPR046W	YPR046W	UWOPS87_Ator08_hmg124h_14.JPG.dat	754.0	33.0	0.9	0.04	0.027	0.044	2.7E-05
YPR050C	YPR050C	UWOPS87_Ator08_hmg124h_14.JPG.dat	774.5	56.9	0.9	0.06	-0.116	0.064	8.6E-05
YPR051W	YPR051W	UWOPS87_Ator08_hmg124h_14.JPG.dat	710.5	32.9	0.9	0.05	-0.143	0.046	4.3E-05
YPR052C	YPR052C	UWOPS87_Ator08_hmg124h_14.JPG.dat	895.8	31.0	1.1	0.01	0.108	0.006	1.1E-05
YPR053C	YPR053C	UWOPS87_Ator08_hmg124h_14.JPG.dat	442.8	507.1	0.5	0.61	-0.088	0.607	1.8E-01
YPR054W	YPR054W	UWOPS87_Ator08_hmg124h_14.JPG.dat	422.0	327.8	0.5	0.40	-0.348	0.402	8.3E-02
YPR057W	YPR057W	UWOPS87_Ator08_hmg124h_14.JPG.dat	764.3	87.8	0.9	0.10	-0.145	0.103	3.6E-04
YPR058W	YPR058W	UWOPS87_Ator08_hmg124h_14.JPG.dat	812.0	120.2	1.0	0.15	0.050	0.149	8.8E-04
YPR059C	YPR059C	UWOPS87_Ator08_hmg124h_14.JPG.dat	850.8	62.9	1.0	0.08	-0.191	0.076	1.2E-04
YPR060C	YPR060C	UWOPS87_Ator08_hmg124h_14.JPG.dat	914.5	196.7	1.1	0.24	0.127	0.235	2.6E-03
YPR061C	YPR061C	UWOPS87_Ator08_hmg124h_14.JPG.dat	922.8	36.6	1.1	0.04	0.081	0.041	1.5E-05
YPR062W	YPR062W	UWOPS87_Ator08_hmg124h_14.JPG.dat	735.0	40.0	0.9	0.05	0.046	0.048	3.9E-05
YPR063C	YPR063C	UWOPS87_Ator08_hmg124h_14.JPG.dat	778.5	39.7	1.0	0.02	0.027	0.016	9.4E-05
YPR064W	YPR064W	UWOPS87_Ator08_hmg124h_14.JPG.dat	883.0	42.8	1.1	0.07	0.260	0.068	7.0E-05
YPR065W	YPR065W	UWOPS87_Ator08_hmg124h_14.JPG.dat	1149.5	49.2	1.3	0.08	0.258	0.081	6.0E-05
YPR066W	YPR066W	UWOPS87_Ator08_hmg124h_14.JPG.dat	860.3	31.9	1.0	0.04	0.138	0.039	1.5E-05
YPR068C	YPR068C	UWOPS87_Ator08_hmg124h_14.JPG.dat	858.3	38.0	1.1	0.02	0.004	0.015	6.6E-05
YPR069C	YPR069C	UWOPS87_Ator08_hmg124h_14.JPG.dat	829.0	20.7	1.0	0.02	-0.104	0.020	2.1E-06
YPR070W	YPR070W	UWOPS87_Ator08_hmg124h_14.JPG.dat	727.8	46.9	0.9	0.07	-0.095	0.070	1.2E-04
YPR071W	YPR071W	UWOPS87_Ator08_hmg124h_14.JPG.dat	249.5	355.0	0.3	0.42	0.065	0.420	2.6E-01
YPR073C	YPR073C	UWOPS87_Ator08_hmg124h_14.JPG.dat	866.8	37.9	1.1	0.06	0.034	0.055	3.9E-05
YPR074C	YPR074C	UWOPS87_Ator08_hmg124h_14.JPG.dat	756.8	117.3	0.8	0.03	-0.108	0.026	3.3E-04
YPR075C	YPR075C	UWOPS87_Ator08_hmg124h_14.JPG.dat	836.3	48.3	1.0	0.08	0.013	0.077	1.3E-04
YPR076W	YPR076W	UWOPS87_Ator08_hmg124h_14.JPG.dat	823.5	48.8	1.0	0.05	-0.030	0.051	4.0E-05
YPR077C	YPR077C	UWOPS87_Ator08_hmg124h_14.JPG.dat	787.5	52.7	0.9	0.05	0.024	0.052	4.7E-05
YPR078C	YPR078C	UWOPS87_Ator08_hmg124h_14.JPG.dat	900.5	63.4	1.1	0.03	-0.101	0.030	2.6E-04
YPR079W	YPR079W	UWOPS87_Ator08_hmg124h_14.JPG.dat	876.8	122.2	1.0	0.12	0.072	0.118	4.1E-04
YPR083W	YPR083W	UWOPS87_Ator08_hmg124h_14.JPG.dat	765.0	61.5	1.0	0.07	0.127	0.074	1.2E-04
YPR084W	YPR084W	UWOPS87_Ator08_hmg124h_14.JPG.dat	818.0	31.7	1.0	0.06	0.070	0.061	6.8E-05
YPR089W	YPR089W	UWOPS87_Ator08_hmg124h_14.JPG.dat	918.3	45.9	1.1	0.06	0.054	0.063	5.1E-05
YPR090W	YPR090W	UWOPS87_Ator08_hmg124h_14.JPG.dat	756.0	54.4	0.9	0.07	-0.007	0.069	1.1E-04
YPR091C	YPR091C	UWOPS87_Ator08_hmg124h_14.JPG.dat	853.3	31.8	1.1	0.05	0.088	0.053	3.2E-05
YPR092W	YPR092W	UWOPS87_Ator08_hmg124h_14.JPG.dat	884.0	62.1	1.0	0.06	-0.010	0.057	4.6E-05
YPR093C	YPR093C	UWOPS87_Ator08_hmg124h_14.JPG.dat	806.3	75.0	0.9	0.07	-0.042	0.070	1.1E-04
YPR095C	YPR095C	UWOPS87_Ator08_hmg124h_14.JPG.dat	860.8	64.7	1.0	0.05	-0.080	0.052	4.2E-05

YPR096C	YPR096C	UWOPS87_Ator08_hmg124h_14.JPG.dat	839.3	50.8	1.0	0.02	0.019	0.017	9.3E-05
YPR097W	YPR097W	UWOPS87_Ator08_hmg124h_14.JPG.dat	796.5	33.7	0.9	0.03	0.050	0.033	1.2E-05
YPR098C	YPR098C	UWOPS87_Ator08_hmg124h_14.JPG.dat	760.0	84.3	1.0	0.03	0.101	0.027	2.7E-04
YPR106W	YPR106W	UWOPS87_Ator08_hmg124h_14.JPG.dat	768.8	46.0	0.9	0.06	0.064	0.059	7.1E-05
YPR109W	YPR109W	UWOPS87_Ator08_hmg124h_14.JPG.dat	843.8	14.3	1.0	0.02	0.201	0.020	2.2E-06
YPR111W	YPR111W	UWOPS87_Ator08_hmg124h_14.JPG.dat	825.3	47.9	1.0	0.02	-0.189	0.019	1.2E-04
YPR114W	YPR114W	UWOPS87_Ator08_hmg124h_14.JPG.dat	821.8	27.9	1.0	0.01	0.093	0.010	3.3E-05
YPR115W	YPR115W	UWOPS87_Ator08_hmg124h_14.JPG.dat	871.0	20.7	1.0	0.00	-0.165	0.001	5.7E-07
YPR117W	YPR117W	UWOPS87_Ator08_hmg124h_14.JPG.dat	906.5	33.3	1.0	0.03	0.078	0.026	4.5E-06
YPR118W	YPR118W	UWOPS87_Ator08_hmg124h_14.JPG.dat	826.8	55.1	1.0	0.07	-0.019	0.071	9.6E-05
YPR119W	YPR119W	UWOPS87_Ator08_hmg124h_14.JPG.dat	817.5	69.2	0.9	0.07	0.021	0.067	1.0E-04
YPR120C	YPR120C	UWOPS87_Ator08_hmg124h_14.JPG.dat	868.5	43.1	1.0	0.05	-0.241	0.047	2.7E-05
YPR121W	YPR121W	UWOPS87_Ator08_hmg124h_14.JPG.dat	699.8	48.2	0.9	0.06	-0.026	0.063	9.8E-05
YPR122W	YPR122W	UWOPS87_Ator08_hmg124h_14.JPG.dat	847.0	67.0	1.0	0.07	-0.034	0.072	9.0E-05
YPR123C	YPR123C	UWOPS87_Ator08_hmg124h_14.JPG.dat	835.0	33.2	1.0	0.03	0.123	0.033	8.8E-06
YPR125W	YPR125W	UWOPS87_Ator08_hmg124h_14.JPG.dat	811.3	94.9	1.0	0.02	0.026	0.024	2.0E-04
YPR126C	YPR126C	UWOPS87_Ator08_hmg124h_14.JPG.dat	848.3	59.3	1.1	0.08	-0.026	0.077	9.9E-05
YPR127W	YPR127W	UWOPS87_Ator08_hmg124h_14.JPG.dat	942.8	58.8	1.2	0.08	0.103	0.083	9.4E-05
YPR128C	YPR128C	UWOPS87_Ator08_hmg124h_14.JPG.dat	878.3	31.0	1.1	0.04	-0.025	0.039	1.2E-05
YPR129W	YPR129W	UWOPS87_Ator08_hmg124h_14.JPG.dat	796.5	37.5	1.0	0.05	-0.068	0.052	3.9E-05
YPR130C	YPR130C	UWOPS87_Ator08_hmg124h_14.JPG.dat	789.8	88.8	1.0	0.12	-0.024	0.120	5.3E-04
YPR132W	YPR132W	UWOPS87_Ator08_hmg124h_14.JPG.dat	824.5	41.7	1.0	0.05	0.025	0.050	3.6E-05
YPR134W	YPR134W	UWOPS87_Ator08_hmg124h_14.JPG.dat	898.8	35.9	1.1	0.05	0.003	0.046	2.1E-05
YPR135W	YPR135W	UWOPS87_Ator08_hmg124h_14.JPG.dat	760.5	13.3	0.9	0.02	0.025	0.020	2.7E-06
YPR138C	YPR138C	UWOPS87_Ator08_hmg124h_14.JPG.dat	818.3	71.2	1.0	0.09	-0.072	0.092	2.1E-04
YPR140W	YPR140W	UWOPS87_Ator08_hmg124h_14.JPG.dat	753.5	49.8	0.9	0.06	-0.240	0.055	6.0E-05
YPR141C	YPR141C	UWOPS87_Ator08_hmg124h_14.JPG.dat	812.5	58.4	1.0	0.07	0.109	0.073	1.1E-04
YPR145W	YPR145W	UWOPS87_Ator08_hmg124h_14.JPG.dat	741.0	22.2	0.9	0.03	-0.102	0.030	9.1E-06
YPR146C	YPR146C	UWOPS87_Ator08_hmg124h_14.JPG.dat	676.8	469.0	1.1	0.20	0.030	0.196	1.0E-02
YPR147C	YPR147C	UWOPS87_Ator08_hmg124h_14.JPG.dat	829.0	27.4	1.0	0.04	-0.051	0.036	1.1E-05
YPR148C	YPR148C	UWOPS87_Ator08_hmg124h_14.JPG.dat	803.3	37.4	1.0	0.05	0.026	0.045	2.6E-05
YPR149W	YPR149W	UWOPS87_Ator08_hmg124h_14.JPG.dat	875.5	23.7	1.0	0.03	0.102	0.032	7.9E-06
YPR150W	YPR150W	UWOPS87_Ator08_hmg124h_14.JPG.dat	968.8	28.7	1.1	0.03	-0.011	0.033	6.9E-06
YPR151C	YPR151C	UWOPS87_Ator08_hmg124h_14.JPG.dat	1033.8	108.3	1.2	0.14	-0.058	0.139	3.9E-04
YPR152C	YPR152C	UWOPS87_Ator08_hmg124h_14.JPG.dat	871.3	38.2	1.0	0.01	-0.085	0.014	5.8E-05
YPR153W	YPR153W	UWOPS87_Ator08_hmg124h_14.JPG.dat	1098.8	152.7	1.3	0.03	0.101	0.025	1.4E-04
YPR154W	YPR154W	UWOPS87_Ator08_hmg124h_14.JPG.dat	860.3	127.9	1.1	0.15	-0.140	0.154	8.6E-04
YPR155C	YPR155C	UWOPS87_Ator08_hmg124h_14.JPG.dat	842.0	35.7	1.0	0.01	-0.015	0.012	5.1E-05
YPR156C	YPR156C	UWOPS87_Ator08_hmg124h_14.JPG.dat	842.5	43.7	1.0	0.06	-0.033	0.056	4.9E-05
YPR157W	YPR157W	UWOPS87_Ator08_hmg124h_14.JPG.dat	861.8	48.6	1.0	0.05	-0.015	0.053	3.8E-05
YPR158W	YPR158W	UWOPS87_Ator08_hmg124h_14.JPG.dat	913.3	10.5	1.1	0.03	0.089	0.026	3.7E-06
YPR160W	YPR160W	UWOPS87_Ator08_hmg124h_14.JPG.dat	547.3	372.0	0.9	0.12	-0.298	0.116	5.4E-03
YPR164W	YPR164W	UWOPS87_Ator08_hmg124h_14.JPG.dat	933.8	81.0	1.1	0.11	0.051	0.109	2.5E-04
YPR167C	YPR167C	UWOPS87_Ator08_hmg124h_14.JPG.dat	774.8	34.1	0.9	0.03	0.052	0.034	1.4E-05
YPR170C	YPR170C	UWOPS87_Ator08_hmg124h_14.JPG.dat	822.8	67.0	0.9	0.02	0.027	0.017	1.2E-04
YPR171W	YPR171W	UWOPS87_Ator08_hmg124h_14.JPG.dat	913.8	85.1	1.0	0.10	0.041	0.101	2.5E-04
YPR172W	YPR172W	UWOPS87_Ator08_hmg124h_14.JPG.dat	801.0	47.7	1.0	0.06	0.065	0.064	7.1E-05
YPR173C	YPR173C	UWOPS87_Ator08_hmg124h_14.JPG.dat	504.0	233.4	0.6	0.29	-0.255	0.293	2.3E-02
YPR174C	YPR174C	UWOPS87_Ator08_hmg124h_14.JPG.dat	841.0	79.6	1.1	0.01	0.021	0.014	5.4E-05
YPR179C	YPR179C	UWOPS87_Ator08_hmg124h_14.JPG.dat	926.0	42.2	1.1	0.05	0.155	0.046	2.0E-05
YPR184W	YPR184W	UWOPS87_Ator08_hmg124h_14.JPG.dat	875.0	27.8	1.0	0.03	-0.017	0.032	8.0E-06
YPR185W	YPR185W	UWOPS87_Ator08_hmg124h_14.JPG.dat	335.8	395.9	0.4	0.48	-0.146	0.480	1.9E-01
YPR188C	YPR188C	UWOPS87_Ator08_hmg124h_14.JPG.dat	846.5	43.9	1.0	0.05	0.114	0.049	2.8E-05
YPR189W	YPR189W	UWOPS87_Ator08_hmg124h_14.JPG.dat	855.0	51.0	1.0	0.06	0.060	0.058	4.8E-05
YPR191W	YPR191W	UWOPS87_Ator08_hmg124h_14.JPG.dat	793.3	77.1	0.9	0.09	-0.199	0.091	2.4E-04
YPR192W	YPR192W	UWOPS87_Ator08_hmg124h_14.JPG.dat	863.0	89.9	1.1	0.04	0.027	0.039	4.4E-04
YPR193C	YPR193C	UWOPS87_Ator08_hmg124h_14.JPG.dat	827.5	62.7	1.0	0.07	-0.052	0.072	1.1E-04
YPR194C	YPR194C	UWOPS87_Ator08_hmg124h_14.JPG.dat	721.5	70.2	0.9	0.02	-0.068	0.021	2.0E-04
YPR195C	YPR195C	UWOPS87_Ator08_hmg124h_14.JPG.dat	776.8	45.1	1.0	0.01	0.090	0.014	6.8E-05
YPR196W	YPR196W	UWOPS87_Ator08_hmg124h_14.JPG.dat	848.5	80.3	1.0	0.09	-0.078	0.094	2.1E-04
YPR197C	YPR197C	UWOPS87_Ator08_hmg124h_14.JPG.dat	798.3	40.6	0.9	0.05	-0.040	0.054	5.1E-05
YPR198W	YPR198W	UWOPS87_Ator08_hmg124h_14.JPG.dat	895.8	47.9	1.1	0.05	-0.093	0.054	3.5E-05
YPR199C	YPR199C	UWOPS87_Ator08_hmg124h_14.JPG.dat	886.0	72.5	1.1	0.08	-0.037	0.081	1.2E-04
YPR200C	YPR200C	UWOPS87_Ator08_hmg124h_14.JPG.dat	835.0	68.2	1.0	0.09	0.095	0.088	1.7E-04
YPR201W	YPR201W	UWOPS87_Ator08_hmg124h_14.JPG.dat	928.5	29.0	1.2	0.06	-0.041	0.058	3.5E-05
1	1	UWOPS87_Ator08_hmg124h_2.JPG.dat	960.3	99.4	1.1	0.05	0.050	0.054	3.8E-05
2	2	UWOPS87_Ator08_hmg124h_2.JPG.dat	832.5	99.1	1.0	0.08	-0.035	0.077	1.2E-04
3	3	UWOPS87_Ator08_hmg124h_2.JPG.dat	791.3	84.7	1.0	0.07	-0.012	0.068	1.0E-04
4	4	UWOPS87_Ator08_hmg124h_2.JPG.dat	790.8	85.6	1.0	0.07	0.019	0.069	1.0E-04
YBR224W	YBR224W	UWOPS87_Ator08_hmg124h_2.JPG.dat	809.0	23.5	1.1	0.05	-0.002	0.047	2.0E-05

YBR225W	YBR225W	UWOPS87_Ator08_hmg124h_2.JPG.dat	819.5	36.4	1.1	0.05	0.148	0.051	2.7E-05
YBR226C	YBR226C	UWOPS87_Ator08_hmg124h_2.JPG.dat	912.0	59.9	1.2	0.07	0.068	0.071	5.8E-05
YBR227C	YBR227C	UWOPS87_Ator08_hmg124h_2.JPG.dat	864.3	61.0	1.1	0.07	0.100	0.070	7.4E-05
YBR228W	YBR228W	UWOPS87_Ator08_hmg124h_2.JPG.dat	871.0	41.3	1.0	0.05	-0.047	0.049	2.8E-05
YBR229C	YBR229C	UWOPS87_Ator08_hmg124h_2.JPG.dat	867.5	25.2	1.0	0.01	-0.073	0.008	2.1E-05
YBR230C	YBR230C	UWOPS87_Ator08_hmg124h_2.JPG.dat	820.5	77.4	1.0	0.10	0.123	0.098	2.7E-04
YBR231C	YBR231C	UWOPS87_Ator08_hmg124h_2.JPG.dat	760.3	20.9	0.9	0.01	0.019	0.005	1.1E-05
YBR232C	YBR232C	UWOPS87_Ator08_hmg124h_2.JPG.dat	831.3	29.3	1.0	0.03	-0.050	0.029	6.2E-06
YBR233W	YBR233W	UWOPS87_Ator08_hmg124h_2.JPG.dat	730.8	92.6	0.9	0.11	-0.016	0.113	5.3E-04
YBR235W	YBR235W	UWOPS87_Ator08_hmg124h_2.JPG.dat	718.8	19.7	0.9	0.03	-0.018	0.034	1.3E-05
YBR238C	YBR238C	UWOPS87_Ator08_hmg124h_2.JPG.dat	659.0	12.6	1.0	0.02	-0.002	0.021	3.0E-06
YBR239C	YBR239C	UWOPS87_Ator08_hmg124h_2.JPG.dat	714.0	20.5	1.0	0.03	-0.019	0.034	1.0E-05
YBR240C	YBR240C	UWOPS87_Ator08_hmg124h_2.JPG.dat	721.5	35.4	1.0	0.04	0.042	0.040	1.8E-05
YBR241C	YBR241C	UWOPS87_Ator08_hmg124h_2.JPG.dat	844.8	102.5	1.1	0.12	0.083	0.123	3.9E-04
YBR242W	YBR242W	UWOPS87_Ator08_hmg124h_2.JPG.dat	883.5	62.5	1.1	0.09	0.103	0.087	1.4E-04
YBR244W	YBR244W	UWOPS87_Ator08_hmg124h_2.JPG.dat	765.3	57.3	0.9	0.07	0.121	0.071	1.1E-04
YBR245C	YBR245C	UWOPS87_Ator08_hmg124h_2.JPG.dat	762.0	57.5	1.0	0.07	-0.153	0.075	1.3E-04
YBR246W	YBR246W	UWOPS87_Ator08_hmg124h_2.JPG.dat	680.3	42.5	0.9	0.05	-0.207	0.053	5.7E-05
YBR248C	YBR248C	UWOPS87_Ator08_hmg124h_2.JPG.dat	856.0	52.0	1.1	0.03	0.008	0.025	1.6E-04
YBR249C	YBR249C	UWOPS87_Ator08_hmg124h_2.JPG.dat	866.5	83.9	1.1	0.11	-0.041	0.107	2.3E-04
YBR250W	YBR250W	UWOPS87_Ator08_hmg124h_2.JPG.dat	717.5	16.8	1.0	0.02	-0.018	0.025	4.0E-06
YBR255W	YBR255W	UWOPS87_Ator08_hmg124h_2.JPG.dat	885.5	60.9	1.3	0.09	0.207	0.089	9.7E-05
YBR258C	YBR258C	UWOPS87_Ator08_hmg124h_2.JPG.dat	778.0	38.7	1.1	0.06	0.047	0.062	5.2E-05
YBR259W	YBR259W	UWOPS87_Ator08_hmg124h_2.JPG.dat	869.3	47.3	1.2	0.06	-0.075	0.059	3.6E-05
YBR260C	YBR260C	UWOPS87_Ator08_hmg124h_2.JPG.dat	813.0	18.1	1.0	0.03	-0.177	0.030	7.2E-06
YBR261C	YBR261C	UWOPS87_Ator08_hmg124h_2.JPG.dat	863.8	34.3	1.1	0.00	0.050	0.005	6.7E-06
YBR262C	YBR262C	UWOPS87_Ator08_hmg124h_2.JPG.dat	831.0	30.4	1.0	0.04	0.037	0.039	1.6E-05
YBR263W	YBR263W	UWOPS87_Ator08_hmg124h_2.JPG.dat	832.5	49.9	1.0	0.06	0.099	0.061	6.1E-05
YBR264C	YBR264C	UWOPS87_Ator08_hmg124h_2.JPG.dat	811.5	91.2	1.0	0.11	0.036	0.110	3.3E-04
YBR266C	YBR266C	UWOPS87_Ator08_hmg124h_2.JPG.dat	0.0	0.0	0.0	0.00	0.000	0.000	
YBR267W	YBR267W	UWOPS87_Ator08_hmg124h_2.JPG.dat	0.0	0.0	0.0	0.00	0.000	0.000	
YBR269C	YBR269C	UWOPS87_Ator08_hmg124h_2.JPG.dat	645.8	44.1	0.9	0.06	0.013	0.057	7.2E-05
YBR270C	YBR270C	UWOPS87_Ator08_hmg124h_2.JPG.dat	685.8	28.9	1.0	0.00	-0.095	0.002	1.5E-06
YBR271W	YBR271W	UWOPS87_Ator08_hmg124h_2.JPG.dat	715.5	68.9	1.0	0.10	0.022	0.096	2.5E-04
YBR272C	YBR272C	UWOPS87_Ator08_hmg124h_2.JPG.dat	761.3	52.0	1.0	0.07	-0.064	0.072	1.0E-04
YBR273C	YBR273C	UWOPS87_Ator08_hmg124h_2.JPG.dat	881.5	27.8	1.1	0.03	0.038	0.031	6.4E-06
YBR274W	YBR274W	UWOPS87_Ator08_hmg124h_2.JPG.dat	823.8	67.7	1.0	0.08	0.140	0.077	1.3E-04
YBR275C	YBR275C	UWOPS87_Ator08_hmg124h_2.JPG.dat	792.8	37.9	1.0	0.05	0.042	0.046	3.1E-05
YBR276C	YBR276C	UWOPS87_Ator08_hmg124h_2.JPG.dat	841.0	85.0	1.0	0.10	0.080	0.100	2.6E-04
YBR277C	YBR277C	UWOPS87_Ator08_hmg124h_2.JPG.dat	850.5	56.7	1.1	0.08	-0.049	0.076	9.9E-05
YBR278W	YBR278W	UWOPS87_Ator08_hmg124h_2.JPG.dat	744.8	34.2	0.9	0.04	-0.113	0.044	2.9E-05
YBR280C	YBR280C	UWOPS87_Ator08_hmg124h_2.JPG.dat	751.0	59.5	1.0	0.08	-0.038	0.083	1.7E-04
YBR281C	YBR281C	UWOPS87_Ator08_hmg124h_2.JPG.dat	735.3	14.5	1.0	0.02	0.071	0.020	2.2E-06
YBR283C	YBR283C	UWOPS87_Ator08_hmg124h_2.JPG.dat	724.3	50.1	1.0	0.03	0.027	0.027	2.5E-04
YBR284W	YBR284W	UWOPS87_Ator08_hmg124h_2.JPG.dat	699.3	47.7	1.0	0.06	-0.069	0.061	6.7E-05
YBR285W	YBR285W	UWOPS87_Ator08_hmg124h_2.JPG.dat	742.8	40.6	1.0	0.04	0.037	0.044	2.5E-05
YBR286W	YBR286W	UWOPS87_Ator08_hmg124h_2.JPG.dat	817.8	61.0	1.0	0.07	-0.028	0.071	9.6E-05
YBR287W	YBR287W	UWOPS87_Ator08_hmg124h_2.JPG.dat	827.0	26.3	1.0	0.01	-0.088	0.008	2.2E-05
YBR288C	YBR288C	UWOPS87_Ator08_hmg124h_2.JPG.dat	755.0	20.8	0.9	0.02	-0.138	0.019	2.3E-06
YBR289W	YBR289W	UWOPS87_Ator08_hmg124h_2.JPG.dat	827.0	28.5	1.0	0.01	0.068	0.005	9.3E-06
YBR290W	YBR290W	UWOPS87_Ator08_hmg124h_2.JPG.dat	783.0	68.6	1.0	0.08	-0.087	0.082	1.6E-04
YBR291C	YBR291C	UWOPS87_Ator08_hmg124h_2.JPG.dat	874.8	54.3	1.1	0.07	0.127	0.070	7.0E-05
YBR292C	YBR292C	UWOPS87_Ator08_hmg124h_2.JPG.dat	792.0	42.3	1.0	0.05	0.017	0.048	2.8E-05
YBR293W	YBR293W	UWOPS87_Ator08_hmg124h_2.JPG.dat	905.5	114.7	1.2	0.15	0.229	0.153	5.1E-04
YBR294W	YBR294W	UWOPS87_Ator08_hmg124h_2.JPG.dat	715.5	18.1	1.0	0.02	-0.003	0.023	3.2E-06
YBR295W	YBR295W	UWOPS87_Ator08_hmg124h_2.JPG.dat	687.8	11.3	0.9	0.02	-0.135	0.016	1.2E-06
YBR296C	YBR296C	UWOPS87_Ator08_hmg124h_2.JPG.dat	755.5	30.3	1.0	0.05	-0.036	0.052	4.0E-05
YBR297W	YBR297W	UWOPS87_Ator08_hmg124h_2.JPG.dat	825.5	40.9	1.0	0.04	0.070	0.044	2.1E-05
YBR298C	YBR298C	UWOPS87_Ator08_hmg124h_2.JPG.dat	900.5	36.9	1.1	0.05	0.112	0.048	2.6E-05
YBR300C	YBR300C	UWOPS87_Ator08_hmg124h_2.JPG.dat	912.8	72.4	1.1	0.02	0.064	0.021	1.1E-04
YBR301W	YBR301W	UWOPS87_Ator08_hmg124h_2.JPG.dat	821.3	26.5	1.0	0.03	0.036	0.029	6.8E-06
YCL001W	YCL001W	UWOPS87_Ator08_hmg124h_2.JPG.dat	734.3	74.6	0.9	0.09	0.079	0.094	2.8E-04
YCL001W-/	YCL001W-A	UWOPS87_Ator08_hmg124h_2.JPG.dat	776.0	40.1	1.0	0.05	0.041	0.049	3.4E-05
YCL002C	YCL002C	UWOPS87_Ator08_hmg124h_2.JPG.dat	773.5	74.7	1.0	0.09	-0.010	0.091	2.2E-04
YCL006C	YCL006C	UWOPS87_Ator08_hmg124h_2.JPG.dat	834.8	54.0	1.1	0.07	-0.004	0.074	8.0E-05
YCL009C	YCL009C	UWOPS87_Ator08_hmg124h_2.JPG.dat	737.5	20.5	1.0	0.02	0.091	0.020	2.6E-06
YCL010C	YCL010C	UWOPS87_Ator08_hmg124h_2.JPG.dat	177.3	354.5	0.0	0.00	0.000	0.000	
YCL011C	YCL011C	UWOPS87_Ator08_hmg124h_2.JPG.dat	561.3	375.4	1.0	0.07	-0.202	0.069	1.6E-03
YCL012W	YCL012W	UWOPS87_Ator08_hmg124h_2.JPG.dat	394.8	456.0	0.5	0.57	0.008	0.570	1.8E-01
YCL013W	YCL013W	UWOPS87_Ator08_hmg124h_2.JPG.dat	724.3	904.6	0.8	0.97	0.194	0.967	2.0E-01

YCL014W	YCL014W	UWOPS87_Ator08_hmg124h_2.JPG.dat	455.8	537.3	0.6	0.65	0.064	0.650	1.9E-01
YCL016C	YCL016C	UWOPS87_Ator08_hmg124h_2.JPG.dat	777.8	519.7	1.3	0.03	0.181	0.034	2.5E-04
YCL022C	YCL022C	UWOPS87_Ator08_hmg124h_2.JPG.dat	0.0	0.0	0.0	0.00	0.000	0.000	
YCL023C	YCL023C	UWOPS87_Ator08_hmg124h_2.JPG.dat	801.3	52.9	1.0	0.02	0.068	0.018	1.0E-04
YCL024W	YCL024W	UWOPS87_Ator08_hmg124h_2.JPG.dat	213.3	426.5	0.0	0.00	0.000	0.000	
YCL025C	YCL025C	UWOPS87_Ator08_hmg124h_2.JPG.dat	335.8	387.7	0.4	0.50	0.051	0.502	1.8E-01
YCL026C	YCL026C	UWOPS87_Ator08_hmg124h_2.JPG.dat	341.8	394.7	0.5	0.54	0.057	0.537	1.8E-01
YCL026C-A	YCL026C-A	UWOPS87_Ator08_hmg124h_2.JPG.dat	561.8	376.1	1.0	0.04	-0.124	0.045	6.4E-04
YCL027W	YCL027W	UWOPS87_Ator08_hmg124h_2.JPG.dat	802.0	39.0	1.1	0.04	-0.129	0.042	1.8E-05
YCL028W	YCL028W	UWOPS87_Ator08_hmg124h_2.JPG.dat	511.0	349.2	0.9	0.12	-0.090	0.118	6.0E-03
YCL029C	YCL029C	UWOPS87_Ator08_hmg124h_2.JPG.dat	796.5	42.0	1.0	0.05	-0.022	0.048	3.2E-05
YCL030C	YCL030C	UWOPS87_Ator08_hmg124h_2.JPG.dat	0.0	0.0	0.0	0.00	0.000	0.000	
YCL032W	YCL032W	UWOPS87_Ator08_hmg124h_2.JPG.dat	851.8	102.1	1.0	0.13	-0.135	0.129	5.7E-04
YCL033C	YCL033C	UWOPS87_Ator08_hmg124h_2.JPG.dat	807.8	56.4	0.9	0.01	0.034	0.010	3.4E-05
YCL034W	YCL034W	UWOPS87_Ator08_hmg124h_2.JPG.dat	711.8	76.5	0.9	0.08	-0.073	0.084	2.6E-04
YCL035C	YCL035C	UWOPS87_Ator08_hmg124h_2.JPG.dat	735.0	53.9	0.9	0.07	0.055	0.069	1.2E-04
YCL036W	YCL036W	UWOPS87_Ator08_hmg124h_2.JPG.dat	778.0	40.6	1.0	0.03	-0.017	0.035	1.2E-05
YCL037C	YCL037C	UWOPS87_Ator08_hmg124h_2.JPG.dat	658.8	29.5	1.0	0.03	0.087	0.034	1.3E-05
YCL038C	YCL038C	UWOPS87_Ator08_hmg124h_2.JPG.dat	676.3	39.0	1.0	0.06	0.039	0.060	6.2E-05
YCL039W	YCL039W	UWOPS87_Ator08_hmg124h_2.JPG.dat	803.8	38.4	1.1	0.06	0.055	0.058	3.8E-05
YCL040W	YCL040W	UWOPS87_Ator08_hmg124h_2.JPG.dat	763.3	9.1	1.0	0.02	0.021	0.021	2.5E-06
YCL042W	YCL042W	UWOPS87_Ator08_hmg124h_2.JPG.dat	804.3	25.1	1.0	0.01	-0.021	0.013	5.2E-05
YCL044C	YCL044C	UWOPS87_Ator08_hmg124h_2.JPG.dat	832.8	27.0	1.0	0.04	-0.214	0.039	1.5E-05
YCL045C	YCL045C	UWOPS87_Ator08_hmg124h_2.JPG.dat	714.0	16.5	0.9	0.02	0.070	0.024	5.4E-06
YCL046W	YCL046W	UWOPS87_Ator08_hmg124h_2.JPG.dat	747.5	111.1	1.0	0.13	-0.217	0.134	7.3E-04
YCL047C	YCL047C	UWOPS87_Ator08_hmg124h_2.JPG.dat	740.3	50.0	0.9	0.02	0.043	0.023	2.0E-04
YCL048W	YCL048W	UWOPS87_Ator08_hmg124h_2.JPG.dat	814.0	127.5	1.1	0.17	0.130	0.165	1.0E-03
YCL049C	YCL049C	UWOPS87_Ator08_hmg124h_2.JPG.dat	515.3	344.9	0.9	0.05	0.193	0.050	9.7E-04
YCL050C	YCL050C	UWOPS87_Ator08_hmg124h_2.JPG.dat	734.5	23.6	1.0	0.04	-0.020	0.039	1.5E-05
YCL051W	YCL051W	UWOPS87_Ator08_hmg124h_2.JPG.dat	693.3	7.2	1.0	0.01	0.020	0.011	4.1E-07
YCL055W	YCL055W	UWOPS87_Ator08_hmg124h_2.JPG.dat	629.5	40.1	0.9	0.06	0.028	0.058	8.8E-05
YCL056C	YCL056C	UWOPS87_Ator08_hmg124h_2.JPG.dat	766.3	37.9	1.0	0.06	-0.090	0.059	6.0E-05
YCL057W	YCL057W	UWOPS87_Ator08_hmg124h_2.JPG.dat	780.5	38.5	1.0	0.04	-0.031	0.042	2.4E-05
YCL060C	YCL060C	UWOPS87_Ator08_hmg124h_2.JPG.dat	803.5	67.7	1.0	0.08	0.004	0.082	1.7E-04
YCL061C	YCL061C	UWOPS87_Ator08_hmg124h_2.JPG.dat	860.0	13.2	1.0	0.02	-0.169	0.020	2.1E-06
YCL062W	YCL062W	UWOPS87_Ator08_hmg124h_2.JPG.dat	768.0	53.7	1.0	0.06	0.117	0.063	8.0E-05
YCL063W	YCL063W	UWOPS87_Ator08_hmg124h_2.JPG.dat	805.0	25.0	1.0	0.03	0.073	0.034	1.0E-05
YCL064C	YCL064C	UWOPS87_Ator08_hmg124h_2.JPG.dat	753.5	32.9	1.0	0.04	-0.028	0.043	2.5E-05
YCL069W	YCL069W	UWOPS87_Ator08_hmg124h_2.JPG.dat	771.3	17.8	1.0	0.01	-0.011	0.013	6.2E-07
YCL074W	YCL074W	UWOPS87_Ator08_hmg124h_2.JPG.dat	720.8	51.1	1.0	0.07	0.024	0.070	8.6E-05
YCL075W	YCL075W	UWOPS87_Ator08_hmg124h_2.JPG.dat	768.0	80.8	1.1	0.12	0.092	0.117	3.4E-04
YCL076W	YCL076W	UWOPS87_Ator08_hmg124h_2.JPG.dat	765.3	24.3	1.0	0.01	0.072	0.011	3.6E-05
YCR001W	YCR001W	UWOPS87_Ator08_hmg124h_2.JPG.dat	860.3	77.1	1.1	0.09	0.016	0.090	1.6E-04
YCR005C	YCR005C	UWOPS87_Ator08_hmg124h_2.JPG.dat	862.0	17.9	1.0	0.03	-0.131	0.030	6.4E-06
YCR006C	YCR006C	UWOPS87_Ator08_hmg124h_2.JPG.dat	799.8	55.3	1.0	0.06	0.138	0.060	6.7E-05
YCR007C	YCR007C	UWOPS87_Ator08_hmg124h_2.JPG.dat	851.8	43.7	1.0	0.05	-0.010	0.053	3.7E-05
YCR008W	YCR008W	UWOPS87_Ator08_hmg124h_2.JPG.dat	799.8	54.3	1.0	0.06	0.019	0.062	7.0E-05
YCR009C	YCR009C	UWOPS87_Ator08_hmg124h_2.JPG.dat	852.0	45.0	1.0	0.05	-0.148	0.050	3.1E-05
YCR010C	YCR010C	UWOPS87_Ator08_hmg124h_2.JPG.dat	761.5	73.1	1.0	0.09	0.029	0.089	2.1E-04
YCR011C	YCR011C	UWOPS87_Ator08_hmg124h_2.JPG.dat	685.8	55.7	0.9	0.07	0.012	0.073	1.4E-04
YCR014C	YCR014C	UWOPS87_Ator08_hmg124h_2.JPG.dat	741.3	9.2	1.1	0.02	0.029	0.017	1.1E-06
YCR015C	YCR015C	UWOPS87_Ator08_hmg124h_2.JPG.dat	698.0	29.9	1.0	0.04	-0.080	0.042	2.1E-05
YCR016W	YCR016W	UWOPS87_Ator08_hmg124h_2.JPG.dat	714.5	32.4	1.0	0.04	0.037	0.036	1.4E-05
YCR017C	YCR017C	UWOPS87_Ator08_hmg124h_2.JPG.dat	849.8	30.8	1.1	0.00	0.068	0.002	1.3E-06
YCR019W	YCR019W	UWOPS87_Ator08_hmg124h_2.JPG.dat	787.0	30.5	0.9	0.02	0.004	0.016	9.8E-05
YCR020C	YCR020C	UWOPS87_Ator08_hmg124h_2.JPG.dat	835.8	20.6	1.0	0.03	0.040	0.029	6.7E-06
YCR020C-A	YCR020C-A	UWOPS87_Ator08_hmg124h_2.JPG.dat	762.5	17.6	0.9	0.02	-0.022	0.020	2.8E-06
YCR021C	YCR021C	UWOPS87_Ator08_hmg124h_2.JPG.dat	821.3	45.1	1.0	0.07	0.097	0.067	7.8E-05
YCR022C	YCR022C	UWOPS87_Ator08_hmg124h_2.JPG.dat	871.8	31.7	1.1	0.04	-0.052	0.037	1.1E-05
YCR023C	YCR023C	UWOPS87_Ator08_hmg124h_2.JPG.dat	797.5	116.1	0.9	0.02	-0.140	0.022	1.9E-04
YCR024C-A	YCR024C-A	UWOPS87_Ator08_hmg124h_2.JPG.dat	782.3	26.6	1.0	0.04	-0.332	0.043	1.9E-05
YCR025C	YCR025C	UWOPS87_Ator08_hmg124h_2.JPG.dat	678.0	15.9	0.9	0.02	-0.010	0.022	3.3E-06
YCR026C	YCR026C	UWOPS87_Ator08_hmg124h_2.JPG.dat	774.0	32.8	1.1	0.05	-0.065	0.049	2.5E-05
YCR027C	YCR027C	UWOPS87_Ator08_hmg124h_2.JPG.dat	731.3	50.3	1.0	0.07	0.024	0.071	1.0E-04
YCR028C-A	YCR028C-A	UWOPS87_Ator08_hmg124h_2.JPG.dat	915.3	45.1	1.2	0.06	-0.070	0.065	4.7E-05
YCR030C	YCR030C	UWOPS87_Ator08_hmg124h_2.JPG.dat	908.8	16.7	1.1	0.00	-0.007	0.001	3.4E-07
YCR031C	YCR031C	UWOPS87_Ator08_hmg124h_2.JPG.dat	747.5	20.2	0.9	0.03	0.199	0.028	8.4E-06
YCR032W	YCR032W	UWOPS87_Ator08_hmg124h_2.JPG.dat	810.8	97.6	1.0	0.04	-0.034	0.036	4.1E-04
YCR033W	YCR033W	UWOPS87_Ator08_hmg124h_2.JPG.dat	832.3	554.2	0.7	0.09	-0.142	0.090	5.6E-03
YCR034W	YCR034W	UWOPS87_Ator08_hmg124h_2.JPG.dat	607.8	19.1	0.8	0.01	-0.359	0.010	5.7E-05

YCR036W	YCR036W	UWOPS87_Ator08_hmg124h_2.JPG.dat	0.0	0.0	0.0	0.00	0.000	0.000	
YCR037C	YCR037C	UWOPS87_Ator08_hmg124h_2.JPG.dat	830.5	146.3	1.1	0.20	0.041	0.196	1.6E-03
YCR043C	YCR043C	UWOPS87_Ator08_hmg124h_2.JPG.dat	803.0	37.7	1.0	0.03	0.043	0.028	5.3E-06
YCR044C	YCR044C	UWOPS87_Ator08_hmg124h_2.JPG.dat	729.0	22.1	1.0	0.03	-0.005	0.029	7.8E-06
YCR045C	YCR045C	UWOPS87_Ator08_hmg124h_2.JPG.dat	714.5	22.6	0.9	0.02	-0.079	0.020	2.6E-06
YCR048W	YCR048W	UWOPS87_Ator08_hmg124h_2.JPG.dat	767.3	12.7	1.0	0.03	0.006	0.028	6.2E-06
YCR049C	YCR049C	UWOPS87_Ator08_hmg124h_2.JPG.dat	773.3	51.4	1.0	0.05	0.099	0.049	3.6E-05
YCR050C	YCR050C	UWOPS87_Ator08_hmg124h_2.JPG.dat	857.3	123.7	1.0	0.02	0.074	0.017	1.0E-04
YCR051W	YCR051W	UWOPS87_Ator08_hmg124h_2.JPG.dat	873.5	44.9	1.0	0.02	0.067	0.021	1.4E-04
YCR053W	YCR053W	UWOPS87_Ator08_hmg124h_2.JPG.dat	81.3	162.5	0.0	0.00	0.000	0.000	
YCR059C	YCR059C	UWOPS87_Ator08_hmg124h_2.JPG.dat	855.3	121.3	1.1	0.15	-0.013	0.147	7.2E-04
YCR060W	YCR060W	UWOPS87_Ator08_hmg124h_2.JPG.dat	922.8	37.1	1.1	0.06	0.003	0.060	4.0E-05
YCR061W	YCR061W	UWOPS87_Ator08_hmg124h_2.JPG.dat	912.5	80.8	1.1	0.09	0.093	0.093	1.5E-04
YCR062W	YCR062W	UWOPS87_Ator08_hmg124h_2.JPG.dat	725.5	17.8	1.0	0.02	0.017	0.017	1.3E-06
YCR063W	YCR063W	UWOPS87_Ator08_hmg124h_2.JPG.dat	869.8	57.8	1.2	0.09	0.035	0.091	1.3E-04
YCR065W	YCR065W	UWOPS87_Ator08_hmg124h_2.JPG.dat	783.5	53.4	1.0	0.06	0.046	0.065	6.8E-05
YCR067C	YCR067C	UWOPS87_Ator08_hmg124h_2.JPG.dat	729.0	44.5	0.9	0.06	0.003	0.058	6.8E-05
YCR068W	YCR068W	UWOPS87_Ator08_hmg124h_2.JPG.dat	794.8	20.5	1.0	0.03	0.024	0.033	1.1E-05
YCR069W	YCR069W	UWOPS87_Ator08_hmg124h_2.JPG.dat	948.0	82.7	1.1	0.11	0.123	0.113	2.6E-04
YCR071C	YCR071C	UWOPS87_Ator08_hmg124h_2.JPG.dat	1030.3	87.8	1.2	0.09	0.125	0.094	1.2E-04
YCR073C	YCR073C	UWOPS87_Ator08_hmg124h_2.JPG.dat	919.3	53.8	1.1	0.07	-0.038	0.070	6.5E-05
YCR073W-	YCR073W-A	UWOPS87_Ator08_hmg124h_2.JPG.dat	897.5	50.3	1.1	0.06	0.058	0.061	4.7E-05
YCR075C	YCR075C	UWOPS87_Ator08_hmg124h_2.JPG.dat	815.3	73.7	1.0	0.09	-0.060	0.089	1.9E-04
YCR076C	YCR076C	UWOPS87_Ator08_hmg124h_2.JPG.dat	800.5	47.4	1.1	0.06	-0.180	0.058	4.5E-05
YCR077C	YCR077C	UWOPS87_Ator08_hmg124h_2.JPG.dat	534.8	31.8	0.7	0.04	-0.117	0.043	5.2E-05
YCR079W	YCR079W	UWOPS87_Ator08_hmg124h_2.JPG.dat	733.8	130.2	1.0	0.17	0.058	0.168	1.3E-03
YCR081W	YCR081W	UWOPS87_Ator08_hmg124h_2.JPG.dat	476.3	78.8	0.6	0.02	-0.032	0.017	2.8E-04
YCR082W	YCR082W	UWOPS87_Ator08_hmg124h_2.JPG.dat	781.3	33.2	1.0	0.04	0.073	0.043	2.3E-05
YCR083W	YCR083W	UWOPS87_Ator08_hmg124h_2.JPG.dat	871.8	23.1	1.0	0.03	-0.126	0.028	5.2E-06
YCR085W	YCR085W	UWOPS87_Ator08_hmg124h_2.JPG.dat	781.0	19.2	0.9	0.02	0.076	0.021	3.1E-06
YCR086W	YCR086W	UWOPS87_Ator08_hmg124h_2.JPG.dat	0.0	0.0	0.0	0.00	0.000	0.000	
YCR087C-A	YCR087C-A	UWOPS87_Ator08_hmg124h_2.JPG.dat	777.0	110.2	1.0	0.14	0.079	0.142	8.2E-04
YCR087W	YCR087W	UWOPS87_Ator08_hmg124h_2.JPG.dat	764.0	35.4	1.0	0.04	0.037	0.038	1.8E-05
YCR088W	YCR088W	UWOPS87_Ator08_hmg124h_2.JPG.dat	779.5	65.0	1.0	0.08	-0.020	0.077	1.4E-04
YCR089W	YCR089W	UWOPS87_Ator08_hmg124h_2.JPG.dat	777.0	20.6	1.0	0.02	0.038	0.022	2.6E-06
YCR090C	YCR090C	UWOPS87_Ator08_hmg124h_2.JPG.dat	739.3	32.3	1.0	0.02	0.024	0.018	9.4E-05
YCR091W	YCR091W	UWOPS87_Ator08_hmg124h_2.JPG.dat	751.8	16.6	1.0	0.03	0.031	0.028	5.9E-06
YCR092C	YCR092C	UWOPS87_Ator08_hmg124h_2.JPG.dat	728.8	33.7	0.9	0.04	-0.022	0.036	1.5E-05
YCR095C	YCR095C	UWOPS87_Ator08_hmg124h_2.JPG.dat	760.5	23.1	0.9	0.04	-0.031	0.035	1.5E-05
YCR098C	YCR098C	UWOPS87_Ator08_hmg124h_2.JPG.dat	832.3	27.5	1.0	0.01	-0.199	0.008	2.2E-05
YCR099C	YCR099C	UWOPS87_Ator08_hmg124h_2.JPG.dat	914.3	45.3	1.0	0.01	0.159	0.009	2.7E-05
YCR100C	YCR100C	UWOPS87_Ator08_hmg124h_2.JPG.dat	871.3	42.7	1.0	0.06	0.047	0.060	5.1E-05
YCR101C	YCR101C	UWOPS87_Ator08_hmg124h_2.JPG.dat	837.0	76.3	1.0	0.04	0.066	0.037	4.6E-04
YCR102C	YCR102C	UWOPS87_Ator08_hmg124h_2.JPG.dat	931.5	66.5	1.2	0.08	0.033	0.079	8.6E-05
YCR102W-	YCR102W-A	UWOPS87_Ator08_hmg124h_2.JPG.dat	795.5	12.2	1.0	0.02	-0.033	0.019	1.9E-06
YCR105W	YCR105W	UWOPS87_Ator08_hmg124h_2.JPG.dat	734.8	21.0	1.0	0.03	0.050	0.029	6.9E-06
YCR106W	YCR106W	UWOPS87_Ator08_hmg124h_2.JPG.dat	696.0	14.3	1.0	0.03	-0.060	0.029	6.5E-06
YDL001W	YDL001W	UWOPS87_Ator08_hmg124h_2.JPG.dat	657.8	51.8	0.9	0.08	-0.057	0.077	1.6E-04
YDL002C	YDL002C	UWOPS87_Ator08_hmg124h_2.JPG.dat	743.5	85.2	1.0	0.13	0.091	0.125	5.3E-04
YDL006W	YDL006W	UWOPS87_Ator08_hmg124h_2.JPG.dat	574.5	424.1	0.7	0.54	0.109	0.537	7.3E-02
YDL010W	YDL010W	UWOPS87_Ator08_hmg124h_2.JPG.dat	834.8	10.4	1.0	0.01	0.017	0.010	2.7E-07
YDL011C	YDL011C	UWOPS87_Ator08_hmg124h_2.JPG.dat	845.0	88.2	1.0	0.11	-0.173	0.111	3.2E-04
YDL012C	YDL012C	UWOPS87_Ator08_hmg124h_2.JPG.dat	740.3	54.8	0.9	0.02	-0.152	0.022	1.7E-04
YDL013W	YDL013W	UWOPS87_Ator08_hmg124h_2.JPG.dat	724.8	32.6	1.0	0.01	0.048	0.012	5.0E-05
YDL018C	YDL018C	UWOPS87_Ator08_hmg124h_2.JPG.dat	663.0	34.5	0.9	0.05	0.031	0.047	4.5E-05
YDL019C	YDL019C	UWOPS87_Ator08_hmg124h_2.JPG.dat	777.3	35.1	1.0	0.06	-0.018	0.056	4.3E-05
YDL020C	YDL020C	UWOPS87_Ator08_hmg124h_2.JPG.dat	653.5	115.4	0.9	0.16	0.084	0.163	1.5E-03
YDL023C	YDL023C	UWOPS87_Ator08_hmg124h_2.JPG.dat	708.0	24.1	1.0	0.01	-0.190	0.009	2.9E-05
YDL024C	YDL024C	UWOPS87_Ator08_hmg124h_2.JPG.dat	694.3	80.8	1.0	0.12	0.008	0.120	5.1E-04
YDL025C	YDL025C	UWOPS87_Ator08_hmg124h_2.JPG.dat	690.8	62.7	0.9	0.07	0.046	0.070	1.2E-04
YDL026W	YDL026W	UWOPS87_Ator08_hmg124h_2.JPG.dat	797.8	17.4	1.0	0.02	-0.075	0.023	3.3E-06
YDL027C	YDL027C	UWOPS87_Ator08_hmg124h_2.JPG.dat	820.5	27.3	1.0	0.03	0.118	0.034	1.1E-05
YDL033C	YDL033C	UWOPS87_Ator08_hmg124h_2.JPG.dat	780.8	32.9	1.0	0.04	-0.037	0.041	2.1E-05
YDL034W	YDL034W	UWOPS87_Ator08_hmg124h_2.JPG.dat	774.8	17.9	1.0	0.03	0.041	0.030	8.2E-06
YDL035C	YDL035C	UWOPS87_Ator08_hmg124h_2.JPG.dat	833.5	46.3	1.1	0.02	-0.144	0.018	9.2E-05
YDL036C	YDL036C	UWOPS87_Ator08_hmg124h_2.JPG.dat	776.5	85.9	1.0	0.10	0.036	0.098	2.7E-04
YDL037C	YDL037C	UWOPS87_Ator08_hmg124h_2.JPG.dat	798.5	101.0	1.0	0.13	0.059	0.132	5.5E-04
YDL038C	YDL038C	UWOPS87_Ator08_hmg124h_2.JPG.dat	755.5	79.2	1.0	0.02	-0.155	0.019	1.2E-04
YDL039C	YDL039C	UWOPS87_Ator08_hmg124h_2.JPG.dat	706.3	26.4	1.0	0.04	0.059	0.036	1.4E-05
YDL040C	YDL040C	UWOPS87_Ator08_hmg124h_2.JPG.dat	733.5	22.1	1.0	0.03	0.051	0.026	4.9E-06

YDL041W	YDL041W	UWOPS87_Ator08_hmg124h_2.JPG.dat	610.5	412.2	1.1	0.10	0.007	0.104	3.2E-03
YDL042C	YDL042C	UWOPS87_Ator08_hmg124h_2.JPG.dat	0.0	0.0	0.0	0.00	0.000	0.000	
YDL046W	YDL046W	UWOPS87_Ator08_hmg124h_2.JPG.dat	909.8	9.6	1.1	0.01	0.057	0.013	4.6E-07
YDL048C	YDL048C	UWOPS87_Ator08_hmg124h_2.JPG.dat	807.5	15.5	1.0	0.02	-0.183	0.018	1.9E-06
YDL050C	YDL050C	UWOPS87_Ator08_hmg124h_2.JPG.dat	868.8	44.5	1.0	0.05	0.067	0.050	3.3E-05
YDL051W	YDL051W	UWOPS87_Ator08_hmg124h_2.JPG.dat	819.0	60.5	1.0	0.07	0.182	0.069	9.7E-05
YDL052C	YDL052C	UWOPS87_Ator08_hmg124h_2.JPG.dat	817.5	42.8	1.0	0.05	0.060	0.053	4.0E-05
YDL053C	YDL053C	UWOPS87_Ator08_hmg124h_2.JPG.dat	747.0	59.5	0.9	0.07	-0.039	0.072	1.2E-04
YDL054C	YDL054C	UWOPS87_Ator08_hmg124h_2.JPG.dat	716.8	34.7	0.9	0.05	0.083	0.046	3.2E-05
YDL056W	YDL056W	UWOPS87_Ator08_hmg124h_2.JPG.dat	719.8	47.7	1.0	0.05	0.012	0.049	3.2E-05
YDL059C	YDL059C	UWOPS87_Ator08_hmg124h_2.JPG.dat	729.3	51.7	1.0	0.00	-0.125	0.002	1.1E-06
YDL061C	YDL061C	UWOPS87_Ator08_hmg124h_2.JPG.dat	695.8	21.2	0.9	0.02	-0.065	0.024	4.7E-06
YDL062W	YDL062W	UWOPS87_Ator08_hmg124h_2.JPG.dat	741.8	15.9	0.9	0.03	0.092	0.029	8.3E-06
YDL065C	YDL065C	UWOPS87_Ator08_hmg124h_2.JPG.dat	819.0	28.2	1.0	0.03	-0.007	0.026	4.7E-06
YDL066W	YDL066W	UWOPS87_Ator08_hmg124h_2.JPG.dat	853.5	23.4	1.0	0.02	-0.170	0.023	3.0E-06
YDL070W	YDL070W	UWOPS87_Ator08_hmg124h_2.JPG.dat	820.0	18.7	1.0	0.01	-0.084	0.007	1.5E-05
YDL071C	YDL071C	UWOPS87_Ator08_hmg124h_2.JPG.dat	786.5	113.1	0.9	0.02	-0.041	0.017	1.1E-04
YDL074C	YDL074C	UWOPS87_Ator08_hmg124h_2.JPG.dat	689.5	67.1	0.9	0.09	-0.190	0.087	2.7E-04
YDL076C	YDL076C	UWOPS87_Ator08_hmg124h_2.JPG.dat	919.3	36.3	1.2	0.04	0.076	0.037	8.8E-06
YDL077C	YDL077C	UWOPS87_Ator08_hmg124h_2.JPG.dat	0.0	0.0	0.0	0.00	0.000	0.000	
YDL078C	YDL078C	UWOPS87_Ator08_hmg124h_2.JPG.dat	720.3	25.8	1.0	0.03	-0.195	0.026	5.3E-06
YDL079C	YDL079C	UWOPS87_Ator08_hmg124h_2.JPG.dat	696.8	14.3	0.9	0.02	-0.075	0.018	1.8E-06
YDL080C	YDL080C	UWOPS87_Ator08_hmg124h_2.JPG.dat	733.8	62.8	1.0	0.08	0.029	0.083	1.7E-04
YDL081C	YDL081C	UWOPS87_Ator08_hmg124h_2.JPG.dat	707.5	43.8	0.9	0.01	0.213	0.006	1.6E-05
YDL082W	YDL082W	UWOPS87_Ator08_hmg124h_2.JPG.dat	746.0	9.8	0.9	0.00	0.247	0.002	1.5E-06
YDL083C	YDL083C	UWOPS87_Ator08_hmg124h_2.JPG.dat	852.8	23.9	1.0	0.04	0.012	0.036	1.1E-05
YDL085W	YDL085W	UWOPS87_Ator08_hmg124h_2.JPG.dat	854.3	66.2	1.0	0.07	0.137	0.074	1.1E-04
YDL086W	YDL086W	UWOPS87_Ator08_hmg124h_2.JPG.dat	780.0	72.4	0.9	0.03	-0.066	0.029	3.4E-04
YDL088C	YDL088C	UWOPS87_Ator08_hmg124h_2.JPG.dat	822.5	43.0	1.0	0.04	-0.060	0.037	1.4E-05
YDL089W	YDL089W	UWOPS87_Ator08_hmg124h_2.JPG.dat	815.5	30.7	1.0	0.05	-0.038	0.049	3.2E-05
YDL090C	YDL090C	UWOPS87_Ator08_hmg124h_2.JPG.dat	0.0	0.0	0.0	0.00	0.000	0.000	
YDL091C	YDL091C	UWOPS87_Ator08_hmg124h_2.JPG.dat	678.8	36.3	0.9	0.05	-0.039	0.048	3.7E-05
YDL093W	YDL093W	UWOPS87_Ator08_hmg124h_2.JPG.dat	762.5	21.8	1.1	0.03	-0.011	0.030	6.5E-06
YDL094C	YDL094C	UWOPS87_Ator08_hmg124h_2.JPG.dat	705.3	20.7	0.9	0.03	-0.042	0.030	8.7E-06
YDL095W	YDL095W	UWOPS87_Ator08_hmg124h_2.JPG.dat	1040.8	36.1	1.3	0.04	0.064	0.040	7.6E-06
YDL096C	YDL096C	UWOPS87_Ator08_hmg124h_2.JPG.dat	951.3	47.7	1.2	0.05	0.039	0.048	1.9E-05
YDL099W	YDL099W	UWOPS87_Ator08_hmg124h_2.JPG.dat	854.8	14.4	1.0	0.02	-0.070	0.016	1.0E-06
YDL100C	YDL100C	UWOPS87_Ator08_hmg124h_2.JPG.dat	867.3	38.5	1.0	0.05	-0.065	0.050	3.1E-05
YDL104C	YDL104C	UWOPS87_Ator08_hmg124h_2.JPG.dat	986.3	58.9	1.2	0.06	-0.024	0.060	3.3E-05
YDL106C	YDL106C	UWOPS87_Ator08_hmg124h_2.JPG.dat	937.8	43.9	1.2	0.06	-0.035	0.059	3.7E-05
YDL109C	YDL109C	UWOPS87_Ator08_hmg124h_2.JPG.dat	786.3	70.2	1.0	0.09	0.118	0.089	2.0E-04
YDL110C	YDL110C	UWOPS87_Ator08_hmg124h_2.JPG.dat	776.5	16.5	1.0	0.02	-0.055	0.017	1.3E-06
YDL112W	YDL112W	UWOPS87_Ator08_hmg124h_2.JPG.dat	761.3	60.3	1.0	0.09	-0.022	0.087	1.6E-04
YDL113C	YDL113C	UWOPS87_Ator08_hmg124h_2.JPG.dat	689.3	13.7	1.0	0.02	-0.011	0.022	3.2E-06
YDL114W	YDL114W	UWOPS87_Ator08_hmg124h_2.JPG.dat	813.3	52.8	1.1	0.07	-0.018	0.069	7.3E-05
YDL117W	YDL117W	UWOPS87_Ator08_hmg124h_2.JPG.dat	596.5	41.9	0.8	0.02	-0.099	0.015	1.3E-04
YDL118W	YDL118W	UWOPS87_Ator08_hmg124h_2.JPG.dat	843.0	44.0	1.0	0.02	0.016	0.015	7.2E-05
YDL119C	YDL119C	UWOPS87_Ator08_hmg124h_2.JPG.dat	869.3	33.7	1.0	0.01	0.037	0.008	2.1E-05
YDL121C	YDL121C	UWOPS87_Ator08_hmg124h_2.JPG.dat	839.8	65.9	1.0	0.08	0.008	0.078	1.3E-04
YDL122W	YDL122W	UWOPS87_Ator08_hmg124h_2.JPG.dat	851.8	24.8	1.0	0.02	-0.033	0.021	2.3E-06
YDL123W	YDL123W	UWOPS87_Ator08_hmg124h_2.JPG.dat	775.0	60.3	1.0	0.07	-0.097	0.068	1.0E-04
YDL124W	YDL124W	UWOPS87_Ator08_hmg124h_2.JPG.dat	772.5	38.6	1.0	0.05	-0.008	0.049	3.5E-05
YDL125C	YDL125C	UWOPS87_Ator08_hmg124h_2.JPG.dat	832.8	59.1	1.1	0.03	-0.020	0.029	2.2E-04
YDL127W	YDL127W	UWOPS87_Ator08_hmg124h_2.JPG.dat	691.3	23.2	1.0	0.03	0.005	0.026	4.8E-06
YDL128W	YDL128W	UWOPS87_Ator08_hmg124h_2.JPG.dat	733.5	24.7	1.1	0.04	-0.035	0.041	1.6E-05
YDL129W	YDL129W	UWOPS87_Ator08_hmg124h_2.JPG.dat	782.3	37.1	1.0	0.01	-0.012	0.012	4.3E-05
YDL130W	YDL130W	UWOPS87_Ator08_hmg124h_2.JPG.dat	784.3	25.7	1.0	0.03	0.104	0.025	4.3E-06
YDL130W-,	YDL130W-A	UWOPS87_Ator08_hmg124h_2.JPG.dat	796.0	57.6	1.0	0.08	0.102	0.081	1.5E-04
YDL131W	YDL131W	UWOPS87_Ator08_hmg124h_2.JPG.dat	861.3	53.4	1.1	0.06	-0.066	0.056	4.1E-05
YDL133C-A	YDL133C-A	UWOPS87_Ator08_hmg124h_2.JPG.dat	848.8	27.2	1.0	0.03	0.033	0.031	7.5E-06
YDL133W	YDL133W	UWOPS87_Ator08_hmg124h_2.JPG.dat	920.5	265.0	1.0	0.04	0.004	0.042	5.9E-04
YDL134C	YDL134C	UWOPS87_Ator08_hmg124h_2.JPG.dat	886.5	50.4	1.1	0.06	0.045	0.061	4.5E-05
YDL134C-A	YDL134C-A	UWOPS87_Ator08_hmg124h_2.JPG.dat	850.3	106.2	1.1	0.13	-0.091	0.134	4.7E-04
YDL135C	YDL135C	UWOPS87_Ator08_hmg124h_2.JPG.dat	712.3	8.9	1.0	0.01	-0.028	0.011	3.5E-07
YDL136W	YDL136W	UWOPS87_Ator08_hmg124h_2.JPG.dat	681.8	20.0	1.0	0.02	0.178	0.019	1.9E-06
YDL137W	YDL137W	UWOPS87_Ator08_hmg124h_2.JPG.dat	694.5	28.8	1.0	0.05	0.073	0.051	3.7E-05
YDL138W	YDL138W	UWOPS87_Ator08_hmg124h_2.JPG.dat	724.3	37.9	1.0	0.05	-0.156	0.047	2.9E-05
YDL142C	YDL142C	UWOPS87_Ator08_hmg124h_2.JPG.dat	820.3	26.4	1.0	0.05	0.071	0.048	2.7E-05
YDL144C	YDL144C	UWOPS87_Ator08_hmg124h_2.JPG.dat	823.5	38.0	1.0	0.04	0.008	0.036	1.2E-05
YDL146W	YDL146W	UWOPS87_Ator08_hmg124h_2.JPG.dat	828.3	31.2	1.0	0.03	-0.071	0.031	7.5E-06

YDL149W	YDL149W	UWOPS87_Ator08_hmg124h_2.JPG.dat	859.0	34.9	1.0	0.02	0.017	0.016	8.0E-05
YDL154W	YDL154W	UWOPS87_Ator08_hmg124h_2.JPG.dat	810.3	38.2	1.0	0.05	-0.054	0.048	3.0E-05
YDL155W	YDL155W	UWOPS87_Ator08_hmg124h_2.JPG.dat	853.3	80.8	1.1	0.09	0.242	0.092	1.8E-04
YDL156W	YDL156W	UWOPS87_Ator08_hmg124h_2.JPG.dat	793.3	71.8	1.0	0.09	-0.107	0.094	2.1E-04
YDL157C	YDL157C	UWOPS87_Ator08_hmg124h_2.JPG.dat	765.0	81.0	1.0	0.10	0.099	0.098	2.3E-04
YDL159W	YDL159W	UWOPS87_Ator08_hmg124h_2.JPG.dat	0.0	0.0	0.0	0.00	0.000	0.000	
YDL160C	YDL160C	UWOPS87_Ator08_hmg124h_2.JPG.dat	813.3	89.4	1.1	0.12	0.070	0.123	3.6E-04
YDL161W	YDL161W	UWOPS87_Ator08_hmg124h_2.JPG.dat	779.5	50.5	1.0	0.01	0.045	0.010	3.2E-05
YDL162C	YDL162C	UWOPS87_Ator08_hmg124h_2.JPG.dat	754.8	31.4	0.9	0.05	-0.129	0.047	3.5E-05
YDL167C	YDL167C	UWOPS87_Ator08_hmg124h_2.JPG.dat	951.3	39.1	1.1	0.05	-0.044	0.051	2.5E-05
YDL168W	YDL168W	UWOPS87_Ator08_hmg124h_2.JPG.dat	852.0	34.6	1.0	0.04	-0.094	0.037	1.3E-05
YDL169C	YDL169C	UWOPS87_Ator08_hmg124h_2.JPG.dat	862.0	17.8	1.0	0.02	-0.084	0.024	3.7E-06
YDL170W	YDL170W	UWOPS87_Ator08_hmg124h_2.JPG.dat	834.5	10.8	1.0	0.02	-0.091	0.024	3.6E-06
YDL171C	YDL171C	UWOPS87_Ator08_hmg124h_2.JPG.dat	848.5	104.5	1.0	0.13	0.079	0.127	5.1E-04
YDL172C	YDL172C	UWOPS87_Ator08_hmg124h_2.JPG.dat	807.5	89.5	1.0	0.11	0.025	0.110	3.5E-04
YDL173W	YDL173W	UWOPS87_Ator08_hmg124h_2.JPG.dat	626.8	77.5	0.8	0.03	-0.019	0.034	6.6E-04
YDL174C	YDL174C	UWOPS87_Ator08_hmg124h_2.JPG.dat	719.0	6.9	1.0	0.03	-0.131	0.030	7.6E-06
YDL175C	YDL175C	UWOPS87_Ator08_hmg124h_2.JPG.dat	661.5	26.6	0.9	0.04	-0.056	0.044	3.0E-05
YDL176W	YDL176W	UWOPS87_Ator08_hmg124h_2.JPG.dat	786.8	9.9	1.1	0.03	-0.034	0.031	6.7E-06
YDL177C	YDL177C	UWOPS87_Ator08_hmg124h_2.JPG.dat	811.5	30.8	1.1	0.05	0.015	0.045	2.2E-05
YDL178W	YDL178W	UWOPS87_Ator08_hmg124h_2.JPG.dat	781.8	47.5	1.0	0.01	0.141	0.014	6.2E-05
YDL179W	YDL179W	UWOPS87_Ator08_hmg124h_2.JPG.dat	903.5	78.3	1.1	0.08	0.008	0.083	1.2E-04
YDL180W	YDL180W	UWOPS87_Ator08_hmg124h_2.JPG.dat	823.8	96.9	0.9	0.01	-0.016	0.010	3.8E-05
YDL181W	YDL181W	UWOPS87_Ator08_hmg124h_2.JPG.dat	873.8	45.9	1.1	0.04	0.152	0.039	1.2E-05
YDL182W	YDL182W	UWOPS87_Ator08_hmg124h_2.JPG.dat	838.8	46.1	1.1	0.05	0.173	0.052	3.3E-05
YDL183C	YDL183C	UWOPS87_Ator08_hmg124h_2.JPG.dat	814.8	49.6	1.0	0.08	0.147	0.078	1.1E-04
YDL184C	YDL184C	UWOPS87_Ator08_hmg124h_2.JPG.dat	783.3	82.9	1.0	0.09	0.009	0.094	2.2E-04
YOR202W	YOR202W	UWOPS87_Ator08_hmg124h_2.JPG.dat	842.5	96.3	1.0	0.07	-0.006	0.067	0.0E+00
1	1	UWOPS87_Ator08_hmg124h_3.JPG.dat	0.0	0.0	0.0	0.00	0.000	0.000	
2	2	UWOPS87_Ator08_hmg124h_3.JPG.dat	902.3	127.3	1.1	0.08	-0.203	0.083	1.8E-03
3	3	UWOPS87_Ator08_hmg124h_3.JPG.dat	817.5	92.9	1.0	0.10	-0.122	0.102	3.0E-04
4	4	UWOPS87_Ator08_hmg124h_3.JPG.dat	865.5	71.3	1.0	0.07	-0.036	0.071	9.3E-05
YDL185W	YDL185W	UWOPS87_Ator08_hmg124h_3.JPG.dat	751.0	39.2	0.9	0.06	-0.054	0.061	7.6E-05
YDL186W	YDL186W	UWOPS87_Ator08_hmg124h_3.JPG.dat	781.3	20.9	1.0	0.01	0.047	0.011	4.6E-05
YDL187C	YDL187C	UWOPS87_Ator08_hmg124h_3.JPG.dat	806.5	133.7	0.9	0.01	0.005	0.014	8.7E-05
YDL188C	YDL188C	UWOPS87_Ator08_hmg124h_3.JPG.dat	778.5	19.7	0.9	0.03	0.057	0.028	7.6E-06
YDL189W	YDL189W	UWOPS87_Ator08_hmg124h_3.JPG.dat	842.5	55.1	1.0	0.07	-0.167	0.066	7.9E-05
YDL190C	YDL190C	UWOPS87_Ator08_hmg124h_3.JPG.dat	870.8	37.6	1.0	0.01	0.039	0.009	2.4E-05
YDL191W	YDL191W	UWOPS87_Ator08_hmg124h_3.JPG.dat	842.0	62.2	1.0	0.07	-0.098	0.071	1.1E-04
YDL192W	YDL192W	UWOPS87_Ator08_hmg124h_3.JPG.dat	940.0	73.8	1.1	0.08	0.075	0.077	9.6E-05
YDL194W	YDL194W	UWOPS87_Ator08_hmg124h_3.JPG.dat	913.0	69.0	1.1	0.08	-0.101	0.077	1.0E-04
YDL197C	YDL197C	UWOPS87_Ator08_hmg124h_3.JPG.dat	934.0	59.1	1.1	0.07	0.129	0.069	7.0E-05
YDL199C	YDL199C	UWOPS87_Ator08_hmg124h_3.JPG.dat	828.0	65.9	1.1	0.02	0.068	0.022	1.4E-04
YDL200C	YDL200C	UWOPS87_Ator08_hmg124h_3.JPG.dat	773.8	37.5	0.9	0.05	0.055	0.045	3.0E-05
YDL201W	YDL201W	UWOPS87_Ator08_hmg124h_3.JPG.dat	910.0	71.5	1.0	0.02	-0.044	0.023	1.5E-04
YDL203C	YDL203C	UWOPS87_Ator08_hmg124h_3.JPG.dat	844.8	111.3	1.0	0.13	0.047	0.133	7.1E-04
YDL204W	YDL204W	UWOPS87_Ator08_hmg124h_3.JPG.dat	952.8	31.6	1.1	0.00	-0.074	0.004	4.8E-06
YDL206W	YDL206W	UWOPS87_Ator08_hmg124h_3.JPG.dat	903.8	53.0	1.0	0.01	-0.078	0.013	5.4E-05
YDL210W	YDL210W	UWOPS87_Ator08_hmg124h_3.JPG.dat	899.8	37.5	1.0	0.00	-0.076	0.004	5.1E-06
YDL211C	YDL211C	UWOPS87_Ator08_hmg124h_3.JPG.dat	961.3	36.0	1.1	0.05	0.040	0.046	2.3E-05
YDL213C	YDL213C	UWOPS87_Ator08_hmg124h_3.JPG.dat	878.0	16.7	1.0	0.02	0.073	0.019	2.0E-06
YDL214C	YDL214C	UWOPS87_Ator08_hmg124h_3.JPG.dat	931.0	68.3	1.1	0.07	-0.121	0.070	7.7E-05
YDL215C	YDL215C	UWOPS87_Ator08_hmg124h_3.JPG.dat	995.8	55.2	1.1	0.06	-0.092	0.063	4.6E-05
YDL216C	YDL216C	UWOPS87_Ator08_hmg124h_3.JPG.dat	778.8	19.9	0.9	0.03	-0.001	0.030	8.7E-06
YDL218W	YDL218W	UWOPS87_Ator08_hmg124h_3.JPG.dat	786.5	85.0	1.0	0.11	0.062	0.105	3.3E-04
YDL219W	YDL219W	UWOPS87_Ator08_hmg124h_3.JPG.dat	835.0	50.2	1.0	0.02	0.052	0.018	1.0E-04
YDL222C	YDL222C	UWOPS87_Ator08_hmg124h_3.JPG.dat	919.5	59.4	1.1	0.07	-0.039	0.070	7.7E-05
YDL223C	YDL223C	UWOPS87_Ator08_hmg124h_3.JPG.dat	799.0	71.4	0.9	0.08	0.098	0.077	1.6E-04
YDL224C	YDL224C	UWOPS87_Ator08_hmg124h_3.JPG.dat	930.8	74.7	1.1	0.08	0.038	0.084	1.4E-04
YDL226C	YDL226C	UWOPS87_Ator08_hmg124h_3.JPG.dat	778.0	11.5	0.9	0.01	-0.022	0.013	7.9E-07
YDL227C	YDL227C	UWOPS87_Ator08_hmg124h_3.JPG.dat	919.3	39.3	1.0	0.04	0.029	0.043	1.9E-05
YDL229W	YDL229W	UWOPS87_Ator08_hmg124h_3.JPG.dat	853.8	19.3	1.0	0.01	-0.089	0.008	2.1E-05
YDL230W	YDL230W	UWOPS87_Ator08_hmg124h_3.JPG.dat	978.8	98.7	1.2	0.12	0.036	0.116	2.7E-04
YDL231C	YDL231C	UWOPS87_Ator08_hmg124h_3.JPG.dat	897.8	134.8	1.1	0.16	-0.030	0.158	8.9E-04
YDL232W	YDL232W	UWOPS87_Ator08_hmg124h_3.JPG.dat	0.0	0.0	0.0	0.00	0.000	0.000	
YDL233W	YDL233W	UWOPS87_Ator08_hmg124h_3.JPG.dat	939.0	38.5	1.1	0.04	-0.081	0.041	1.4E-05
YDL234C	YDL234C	UWOPS87_Ator08_hmg124h_3.JPG.dat	861.3	55.1	1.0	0.06	0.014	0.065	7.2E-05
YDL236W	YDL236W	UWOPS87_Ator08_hmg124h_3.JPG.dat	964.5	65.8	1.1	0.08	-0.085	0.084	1.3E-04
YDL237W	YDL237W	UWOPS87_Ator08_hmg124h_3.JPG.dat	913.3	32.8	1.0	0.04	0.007	0.038	1.5E-05
YDL238C	YDL238C	UWOPS87_Ator08_hmg124h_3.JPG.dat	1003.3	39.1	1.1	0.05	-0.152	0.049	2.4E-05

YDL239C	YDL239C	UWOPS87_Ator08_hmg124h_3.JPG.dat	798.3	34.7	0.9	0.04	0.018	0.036	1.7E-05
YDL240W	YDL240W	UWOPS87_Ator08_hmg124h_3.JPG.dat	922.8	32.8	1.0	0.01	0.153	0.008	1.9E-05
YDL241W	YDL241W	UWOPS87_Ator08_hmg124h_3.JPG.dat	851.5	86.4	1.0	0.10	-0.001	0.095	2.7E-04
YDL242W	YDL242W	UWOPS87_Ator08_hmg124h_3.JPG.dat	933.5	53.0	1.1	0.06	0.044	0.062	5.3E-05
YDL243C	YDL243C	UWOPS87_Ator08_hmg124h_3.JPG.dat	895.8	95.0	1.0	0.11	-0.026	0.113	3.7E-04
YDR001C	YDR001C	UWOPS87_Ator08_hmg124h_3.JPG.dat	826.8	44.0	1.0	0.05	0.043	0.046	2.7E-05
YDR003W	YDR003W	UWOPS87_Ator08_hmg124h_3.JPG.dat	911.5	85.8	1.1	0.03	0.059	0.032	3.0E-04
YDR004W	YDR004W	UWOPS87_Ator08_hmg124h_3.JPG.dat	885.0	80.0	1.0	0.01	-0.130	0.009	2.4E-05
YDR005C	YDR005C	UWOPS87_Ator08_hmg124h_3.JPG.dat	757.5	511.3	1.2	0.11	0.010	0.115	3.2E-03
YDR006C	YDR006C	UWOPS87_Ator08_hmg124h_3.JPG.dat	960.0	62.9	1.1	0.03	-0.077	0.027	2.0E-04
YDR007W	YDR007W	UWOPS87_Ator08_hmg124h_3.JPG.dat	928.3	78.5	1.0	0.08	-0.004	0.085	1.6E-04
YDR008C	YDR008C	UWOPS87_Ator08_hmg124h_3.JPG.dat	839.3	78.9	1.0	0.09	-0.009	0.086	2.0E-04
YDR009W	YDR009W	UWOPS87_Ator08_hmg124h_3.JPG.dat	903.0	98.4	1.0	0.12	-0.030	0.116	4.0E-04
YDR010C	YDR010C	UWOPS87_Ator08_hmg124h_3.JPG.dat	901.5	75.7	1.0	0.08	0.010	0.080	1.2E-04
YDR011W	YDR011W	UWOPS87_Ator08_hmg124h_3.JPG.dat	912.3	65.1	1.1	0.08	0.001	0.079	1.1E-04
YDR014W	YDR014W	UWOPS87_Ator08_hmg124h_3.JPG.dat	829.5	42.8	1.0	0.01	-0.030	0.011	4.4E-05
YDR015C	YDR015C	UWOPS87_Ator08_hmg124h_3.JPG.dat	790.0	34.9	1.0	0.05	0.021	0.047	3.2E-05
YDR018C	YDR018C	UWOPS87_Ator08_hmg124h_3.JPG.dat	803.8	78.3	0.9	0.09	0.006	0.093	2.6E-04
YDR019C	YDR019C	UWOPS87_Ator08_hmg124h_3.JPG.dat	787.0	26.7	0.9	0.03	-0.089	0.028	7.1E-06
YDR020C	YDR020C	UWOPS87_Ator08_hmg124h_3.JPG.dat	794.8	67.8	1.0	0.02	0.068	0.017	1.0E-04
YDR022C	YDR022C	UWOPS87_Ator08_hmg124h_3.JPG.dat	831.8	18.6	0.9	0.02	-0.090	0.021	3.2E-06
YDR024W	YDR024W	UWOPS87_Ator08_hmg124h_3.JPG.dat	843.8	37.6	0.9	0.04	0.020	0.039	1.9E-05
YDR025W	YDR025W	UWOPS87_Ator08_hmg124h_3.JPG.dat	933.0	14.6	1.1	0.00	-0.030	0.005	7.0E-06
YDR048C	YDR048C	UWOPS87_Ator08_hmg124h_3.JPG.dat	817.5	81.6	0.9	0.09	-0.018	0.091	2.6E-04
YDR049W	YDR049W	UWOPS87_Ator08_hmg124h_3.JPG.dat	811.3	45.4	0.9	0.05	-0.013	0.053	4.9E-05
YDR051C	YDR051C	UWOPS87_Ator08_hmg124h_3.JPG.dat	985.3	93.7	1.2	0.11	-0.024	0.114	2.6E-04
YDR055W	YDR055W	UWOPS87_Ator08_hmg124h_3.JPG.dat	930.0	113.8	1.1	0.13	0.127	0.134	5.4E-04
YDR056C	YDR056C	UWOPS87_Ator08_hmg124h_3.JPG.dat	831.0	140.5	1.0	0.17	-0.014	0.170	1.3E-03
YDR057W	YDR057W	UWOPS87_Ator08_hmg124h_3.JPG.dat	908.8	31.7	1.1	0.01	0.006	0.005	6.6E-06
YDR058C	YDR058C	UWOPS87_Ator08_hmg124h_3.JPG.dat	870.5	32.5	1.1	0.04	-0.084	0.037	1.1E-05
YDR059C	YDR059C	UWOPS87_Ator08_hmg124h_3.JPG.dat	785.0	46.3	1.0	0.05	-0.043	0.054	5.0E-05
YDR061W	YDR061W	UWOPS87_Ator08_hmg124h_3.JPG.dat	788.5	52.9	0.9	0.03	-0.034	0.026	2.6E-04
YDR063W	YDR063W	UWOPS87_Ator08_hmg124h_3.JPG.dat	732.8	20.0	0.9	0.03	0.066	0.029	9.0E-06
YDR066C	YDR066C	UWOPS87_Ator08_hmg124h_3.JPG.dat	731.8	62.2	0.9	0.07	0.038	0.071	1.2E-04
YDR067C	YDR067C	UWOPS87_Ator08_hmg124h_3.JPG.dat	768.8	50.7	1.0	0.06	0.019	0.063	7.9E-05
YDR068W	YDR068W	UWOPS87_Ator08_hmg124h_3.JPG.dat	714.5	50.0	0.9	0.06	-0.051	0.064	1.0E-04
YDR070C	YDR070C	UWOPS87_Ator08_hmg124h_3.JPG.dat	726.0	23.2	0.9	0.01	-0.018	0.009	3.0E-05
YDR071C	YDR071C	UWOPS87_Ator08_hmg124h_3.JPG.dat	526.5	377.1	0.6	0.46	-0.532	0.464	6.8E-02
YDR072C	YDR072C	UWOPS87_Ator08_hmg124h_3.JPG.dat	698.8	56.5	0.9	0.01	0.015	0.005	1.0E-05
YDR073W	YDR073W	UWOPS87_Ator08_hmg124h_3.JPG.dat	810.3	55.3	1.0	0.08	-0.134	0.080	1.3E-04
YDR074W	YDR074W	UWOPS87_Ator08_hmg124h_3.JPG.dat	865.5	73.6	1.0	0.02	-0.288	0.022	1.6E-04
YDR075W	YDR075W	UWOPS87_Ator08_hmg124h_3.JPG.dat	912.0	32.4	1.1	0.03	0.143	0.034	8.2E-06
YDR076W	YDR076W	UWOPS87_Ator08_hmg124h_3.JPG.dat	811.8	36.5	1.0	0.04	-0.032	0.043	2.4E-05
YDR077W	YDR077W	UWOPS87_Ator08_hmg124h_3.JPG.dat	940.8	15.9	1.1	0.02	-0.089	0.020	1.7E-06
YDR080W	YDR080W	UWOPS87_Ator08_hmg124h_3.JPG.dat	913.0	23.7	1.1	0.03	0.067	0.025	3.7E-06
YDR083W	YDR083W	UWOPS87_Ator08_hmg124h_3.JPG.dat	817.0	45.3	1.0	0.05	0.185	0.045	2.9E-05
YDR084C	YDR084C	UWOPS87_Ator08_hmg124h_3.JPG.dat	849.3	14.9	1.0	0.01	-0.004	0.007	1.5E-05
YDR085C	YDR085C	UWOPS87_Ator08_hmg124h_3.JPG.dat	797.5	97.8	0.9	0.11	0.036	0.107	4.2E-04
YDR089W	YDR089W	UWOPS87_Ator08_hmg124h_3.JPG.dat	780.3	46.0	0.9	0.05	-0.041	0.049	4.1E-05
YDR090C	YDR090C	UWOPS87_Ator08_hmg124h_3.JPG.dat	859.8	57.3	1.0	0.07	0.000	0.067	8.1E-05
YDR092W	YDR092W	UWOPS87_Ator08_hmg124h_3.JPG.dat	896.5	90.1	1.1	0.10	0.027	0.102	2.1E-04
YDR093W	YDR093W	UWOPS87_Ator08_hmg124h_3.JPG.dat	660.0	167.2	0.9	0.06	-0.056	0.064	1.7E-03
YDR094W	YDR094W	UWOPS87_Ator08_hmg124h_3.JPG.dat	844.0	58.8	0.9	0.01	0.021	0.015	8.2E-05
YDR095C	YDR095C	UWOPS87_Ator08_hmg124h_3.JPG.dat	782.0	79.6	0.9	0.09	-0.067	0.091	2.9E-04
YDR096W	YDR096W	UWOPS87_Ator08_hmg124h_3.JPG.dat	1011.3	70.3	1.1	0.08	-0.171	0.076	8.7E-05
YDR097C	YDR097C	UWOPS87_Ator08_hmg124h_3.JPG.dat	1023.8	23.3	1.1	0.00	-0.105	0.004	5.3E-06
YDR098C	YDR098C	UWOPS87_Ator08_hmg124h_3.JPG.dat	885.8	28.0	1.0	0.00	-0.013	0.003	2.4E-06
YDR099W	YDR099W	UWOPS87_Ator08_hmg124h_3.JPG.dat	799.0	28.7	0.9	0.04	-0.067	0.037	2.0E-05
YDR100W	YDR100W	UWOPS87_Ator08_hmg124h_3.JPG.dat	782.5	43.3	0.9	0.05	-0.049	0.046	3.8E-05
YDR101C	YDR101C	UWOPS87_Ator08_hmg124h_3.JPG.dat	823.8	53.9	1.0	0.06	0.250	0.062	7.1E-05
YDR102C	YDR102C	UWOPS87_Ator08_hmg124h_3.JPG.dat	779.5	56.2	0.9	0.06	-0.028	0.065	9.9E-05
YDR103W	YDR103W	UWOPS87_Ator08_hmg124h_3.JPG.dat	0.0	0.0	0.0	0.00	0.000	0.000	
YDR104C	YDR104C	UWOPS87_Ator08_hmg124h_3.JPG.dat	810.0	75.9	1.0	0.01	-0.005	0.009	3.1E-05
YDR105C	YDR105C	UWOPS87_Ator08_hmg124h_3.JPG.dat	802.3	32.6	1.0	0.01	0.005	0.011	4.2E-05
YDR107C	YDR107C	UWOPS87_Ator08_hmg124h_3.JPG.dat	872.8	117.6	1.0	0.14	-0.028	0.139	6.6E-04
YDR108W	YDR108W	UWOPS87_Ator08_hmg124h_3.JPG.dat	873.5	88.1	1.0	0.02	-0.057	0.022	1.8E-04
YDR109C	YDR109C	UWOPS87_Ator08_hmg124h_3.JPG.dat	857.3	53.9	1.0	0.06	-0.003	0.061	6.9E-05
YDR110W	YDR110W	UWOPS87_Ator08_hmg124h_3.JPG.dat	898.3	30.7	1.0	0.03	0.008	0.033	9.0E-06
YDR111C	YDR111C	UWOPS87_Ator08_hmg124h_3.JPG.dat	851.0	62.2	1.0	0.07	0.040	0.068	9.2E-05
YDR112W	YDR112W	UWOPS87_Ator08_hmg124h_3.JPG.dat	836.8	55.9	1.0	0.07	-0.053	0.066	8.3E-05

YDR116C	YDR116C	UWOPS87_Ator08_hmg124h_3.JPG.dat	842.8	141.3	1.0	0.17	0.046	0.173	1.3E-03
YDR117C	YDR117C	UWOPS87_Ator08_hmg124h_3.JPG.dat	800.5	40.7	0.9	0.01	-0.091	0.013	6.6E-05
YDR119W	YDR119W	UWOPS87_Ator08_hmg124h_3.JPG.dat	830.0	56.8	1.0	0.06	0.096	0.064	7.1E-05
YDR120C	YDR120C	UWOPS87_Ator08_hmg124h_3.JPG.dat	851.0	63.1	1.0	0.07	-0.035	0.071	9.1E-05
YDR121W	YDR121W	UWOPS87_Ator08_hmg124h_3.JPG.dat	775.0	84.1	0.9	0.10	-0.173	0.100	4.0E-04
YDR122W	YDR122W	UWOPS87_Ator08_hmg124h_3.JPG.dat	922.8	96.2	1.0	0.10	0.047	0.102	2.7E-04
YDR123C	YDR123C	UWOPS87_Ator08_hmg124h_3.JPG.dat	0.0	0.0	0.0	0.00	0.000	0.000	
YDR124W	YDR124W	UWOPS87_Ator08_hmg124h_3.JPG.dat	960.5	100.1	1.1	0.04	-0.045	0.040	4.4E-04
YDR125C	YDR125C	UWOPS87_Ator08_hmg124h_3.JPG.dat	884.8	28.7	1.0	0.01	-0.028	0.011	4.3E-05
YDR126W	YDR126W	UWOPS87_Ator08_hmg124h_3.JPG.dat	868.3	10.7	1.0	0.00	-0.085	0.003	3.7E-06
YDR127W	YDR127W	UWOPS87_Ator08_hmg124h_3.JPG.dat	0.0	0.0	0.0	0.00	0.000	0.000	
YDR128W	YDR128W	UWOPS87_Ator08_hmg124h_3.JPG.dat	863.8	50.2	1.0	0.01	0.006	0.005	8.8E-06
YDR130C	YDR130C	UWOPS87_Ator08_hmg124h_3.JPG.dat	995.8	218.4	1.0	0.07	-0.023	0.074	1.7E-03
YDR131C	YDR131C	UWOPS87_Ator08_hmg124h_3.JPG.dat	791.0	54.9	1.0	0.07	-0.001	0.071	1.1E-04
YDR132C	YDR132C	UWOPS87_Ator08_hmg124h_3.JPG.dat	967.5	54.2	1.2	0.07	-0.116	0.073	6.7E-05
YDR133C	YDR133C	UWOPS87_Ator08_hmg124h_3.JPG.dat	776.0	31.9	0.9	0.04	0.094	0.037	1.9E-05
YDR134C	YDR134C	UWOPS87_Ator08_hmg124h_3.JPG.dat	841.8	72.1	1.0	0.08	0.205	0.085	1.9E-04
YDR135C	YDR135C	UWOPS87_Ator08_hmg124h_3.JPG.dat	847.0	86.8	0.9	0.09	0.055	0.094	2.9E-04
YDR139C	YDR139C	UWOPS87_Ator08_hmg124h_3.JPG.dat	935.3	58.1	1.0	0.06	0.045	0.063	6.1E-05
YDR142C	YDR142C	UWOPS87_Ator08_hmg124h_3.JPG.dat	935.3	60.0	1.1	0.08	-0.052	0.080	1.2E-04
YDR143C	YDR143C	UWOPS87_Ator08_hmg124h_3.JPG.dat	668.3	82.5	0.7	0.04	-0.017	0.039	9.7E-04
YDR144C	YDR144C	UWOPS87_Ator08_hmg124h_3.JPG.dat	947.3	44.4	1.1	0.04	0.060	0.041	1.4E-05
YDR146C	YDR146C	UWOPS87_Ator08_hmg124h_3.JPG.dat	724.0	45.0	0.9	0.04	-0.226	0.043	3.5E-05
YDR147W	YDR147W	UWOPS87_Ator08_hmg124h_3.JPG.dat	796.3	29.1	0.9	0.01	0.070	0.014	7.3E-05
YDR148C	YDR148C	UWOPS87_Ator08_hmg124h_3.JPG.dat	897.0	68.9	1.1	0.08	-0.042	0.084	1.3E-04
YDR149C	YDR149C	UWOPS87_Ator08_hmg124h_3.JPG.dat	39.3	78.5	0.0	0.00	0.000	0.000	
YDR150W	YDR150W	UWOPS87_Ator08_hmg124h_3.JPG.dat	579.0	735.3	0.3	0.23	-0.099	0.233	2.0E-01
YDR151C	YDR151C	UWOPS87_Ator08_hmg124h_3.JPG.dat	893.8	74.2	1.0	0.09	0.064	0.086	1.6E-04
YDR152W	YDR152W	UWOPS87_Ator08_hmg124h_3.JPG.dat	949.8	37.1	1.0	0.04	0.044	0.038	1.3E-05
YDR153C	YDR153C	UWOPS87_Ator08_hmg124h_3.JPG.dat	676.3	79.4	0.8	0.02	-0.117	0.021	2.3E-04
YDR154C	YDR154C	UWOPS87_Ator08_hmg124h_3.JPG.dat	937.0	34.9	1.1	0.03	-0.073	0.034	8.9E-06
YDR155C	YDR155C	UWOPS87_Ator08_hmg124h_3.JPG.dat	1033.8	46.5	1.2	0.06	0.043	0.055	2.8E-05
YDR156W	YDR156W	UWOPS87_Ator08_hmg124h_3.JPG.dat	767.3	22.3	0.9	0.01	-0.053	0.008	2.6E-05
YDR157W	YDR157W	UWOPS87_Ator08_hmg124h_3.JPG.dat	1018.8	40.4	1.2	0.05	0.177	0.046	1.5E-05
YDR158W	YDR158W	UWOPS87_Ator08_hmg124h_3.JPG.dat	740.5	494.1	1.1	0.02	-0.054	0.023	1.4E-04
YDR159W	YDR159W	UWOPS87_Ator08_hmg124h_3.JPG.dat	386.3	460.7	0.5	0.56	0.059	0.561	1.9E-01
YDR161W	YDR161W	UWOPS87_Ator08_hmg124h_3.JPG.dat	766.5	55.9	0.9	0.07	0.020	0.073	1.3E-04
YDR162C	YDR162C	UWOPS87_Ator08_hmg124h_3.JPG.dat	910.3	48.8	1.1	0.06	0.046	0.059	4.3E-05
YDR163W	YDR163W	UWOPS87_Ator08_hmg124h_3.JPG.dat	854.3	33.0	1.0	0.04	0.058	0.040	1.6E-05
YDR165W	YDR165W	UWOPS87_Ator08_hmg124h_3.JPG.dat	850.3	18.0	1.0	0.02	0.046	0.024	3.4E-06
YDR169C	YDR169C	UWOPS87_Ator08_hmg124h_3.JPG.dat	778.5	51.6	1.0	0.06	0.001	0.065	7.9E-05
YDR171W	YDR171W	UWOPS87_Ator08_hmg124h_3.JPG.dat	794.3	60.1	1.0	0.08	0.027	0.081	1.4E-04
YDR173C	YDR173C	UWOPS87_Ator08_hmg124h_3.JPG.dat	853.5	76.9	1.1	0.10	-0.078	0.095	2.0E-04
YDR174W	YDR174W	UWOPS87_Ator08_hmg124h_3.JPG.dat	883.0	50.0	1.1	0.06	0.006	0.061	4.7E-05
YDR178W	YDR178W	UWOPS87_Ator08_hmg124h_3.JPG.dat	847.5	28.2	1.1	0.04	-0.046	0.042	1.7E-05
YDR179C	YDR179C	UWOPS87_Ator08_hmg124h_3.JPG.dat	840.8	561.0	1.4	0.04	0.088	0.039	2.7E-04
YDR179W-	YDR179W-A	UWOPS87_Ator08_hmg124h_3.JPG.dat	828.5	32.9	1.0	0.04	-0.070	0.038	1.6E-05
YDR181C	YDR181C	UWOPS87_Ator08_hmg124h_3.JPG.dat	784.5	9.0	0.9	0.02	-0.105	0.020	2.8E-06
YDR183W	YDR183W	UWOPS87_Ator08_hmg124h_3.JPG.dat	817.8	33.7	1.0	0.06	0.030	0.057	5.9E-05
YDR184C	YDR184C	UWOPS87_Ator08_hmg124h_3.JPG.dat	809.5	67.1	0.9	0.03	0.124	0.028	3.0E-04
YDR185C	YDR185C	UWOPS87_Ator08_hmg124h_3.JPG.dat	724.8	48.3	0.9	0.07	0.004	0.074	1.7E-04
YDR186C	YDR186C	UWOPS87_Ator08_hmg124h_3.JPG.dat	831.5	39.7	1.0	0.03	-0.024	0.029	6.8E-06
YDR191W	YDR191W	UWOPS87_Ator08_hmg124h_3.JPG.dat	985.5	74.4	1.1	0.02	-0.017	0.024	1.5E-04
YDR192C	YDR192C	UWOPS87_Ator08_hmg124h_3.JPG.dat	901.5	25.3	1.0	0.03	-0.028	0.028	5.9E-06
YDR193W	YDR193W	UWOPS87_Ator08_hmg124h_3.JPG.dat	879.5	60.7	1.0	0.05	0.021	0.054	4.3E-05
YDR198C	YDR198C	UWOPS87_Ator08_hmg124h_3.JPG.dat	850.8	83.7	1.0	0.02	-0.074	0.024	1.9E-04
YDR199W	YDR199W	UWOPS87_Ator08_hmg124h_3.JPG.dat	848.8	63.8	1.2	0.01	0.183	0.013	4.0E-05
YDR202C	YDR202C	UWOPS87_Ator08_hmg124h_3.JPG.dat	815.3	32.6	1.0	0.03	0.086	0.031	7.5E-06
YDR203W	YDR203W	UWOPS87_Ator08_hmg124h_3.JPG.dat	913.5	82.0	1.1	0.09	0.026	0.088	1.4E-04
YDR205W	YDR205W	UWOPS87_Ator08_hmg124h_3.JPG.dat	864.0	51.9	1.0	0.07	-0.097	0.067	7.7E-05
YDR206W	YDR206W	UWOPS87_Ator08_hmg124h_3.JPG.dat	911.8	63.1	1.1	0.09	0.060	0.085	1.3E-04
YDR207C	YDR207C	UWOPS87_Ator08_hmg124h_3.JPG.dat	338.3	390.7	0.4	0.45	0.135	0.454	1.8E-01
YDR209C	YDR209C	UWOPS87_Ator08_hmg124h_3.JPG.dat	823.0	40.3	1.0	0.04	-0.017	0.042	2.3E-05
YDR210W	YDR210W	UWOPS87_Ator08_hmg124h_3.JPG.dat	882.5	67.5	1.0	0.03	-0.024	0.028	2.9E-04
YDR213W	YDR213W	UWOPS87_Ator08_hmg124h_3.JPG.dat	904.3	109.3	1.1	0.12	-0.063	0.123	4.3E-04
YDR214W	YDR214W	UWOPS87_Ator08_hmg124h_3.JPG.dat	809.8	54.2	1.0	0.06	-0.044	0.058	6.0E-05
YDR215C	YDR215C	UWOPS87_Ator08_hmg124h_3.JPG.dat	840.3	63.7	1.0	0.08	0.077	0.077	1.3E-04
YDR216W	YDR216W	UWOPS87_Ator08_hmg124h_3.JPG.dat	689.3	61.8	0.9	0.08	0.036	0.075	1.9E-04
YDR217C	YDR217C	UWOPS87_Ator08_hmg124h_3.JPG.dat	775.0	25.8	1.0	0.01	-0.159	0.012	5.2E-05
YDR218C	YDR218C	UWOPS87_Ator08_hmg124h_3.JPG.dat	867.8	52.9	1.1	0.06	0.033	0.059	4.7E-05

YDR219C	YDR219C	UWOPS87_Ator08_hmg124h_3.JPG.dat	903.5	70.7	1.0	0.02	0.035	0.024	1.9E-04
YDR220C	YDR220C	UWOPS87_Ator08_hmg124h_3.JPG.dat	832.8	45.1	1.0	0.06	-0.079	0.056	5.5E-05
YDR221W	YDR221W	UWOPS87_Ator08_hmg124h_3.JPG.dat	788.0	53.0	0.9	0.06	0.151	0.060	8.1E-05
YDR222W	YDR222W	UWOPS87_Ator08_hmg124h_3.JPG.dat	870.3	29.1	1.0	0.01	-0.220	0.005	8.5E-06
YDR223W	YDR223W	UWOPS87_Ator08_hmg124h_3.JPG.dat	910.5	19.8	1.0	0.02	-0.057	0.023	2.8E-06
YDR225W	YDR225W	UWOPS87_Ator08_hmg124h_3.JPG.dat	896.3	54.4	1.0	0.06	0.145	0.060	5.0E-05
YDR227W	YDR227W	UWOPS87_Ator08_hmg124h_3.JPG.dat	0.0	0.0	0.0	0.00	0.000	0.000	
YDR229W	YDR229W	UWOPS87_Ator08_hmg124h_3.JPG.dat	883.8	57.1	1.1	0.07	-0.089	0.065	6.5E-05
YDR233C	YDR233C	UWOPS87_Ator08_hmg124h_3.JPG.dat	796.5	7.3	1.0	0.00	-0.036	0.001	9.2E-08
YDR234W	YDR234W	UWOPS87_Ator08_hmg124h_3.JPG.dat	774.5	30.4	1.0	0.04	0.098	0.041	2.1E-05
YDR239C	YDR239C	UWOPS87_Ator08_hmg124h_3.JPG.dat	696.8	46.1	0.8	0.06	0.029	0.060	9.5E-05
YDR241W	YDR241W	UWOPS87_Ator08_hmg124h_3.JPG.dat	843.0	60.1	1.0	0.08	-0.171	0.077	1.3E-04
YDR244W	YDR244W	UWOPS87_Ator08_hmg124h_3.JPG.dat	952.0	31.7	1.1	0.04	-0.142	0.044	1.8E-05
YDR245W	YDR245W	UWOPS87_Ator08_hmg124h_3.JPG.dat	940.3	88.5	1.1	0.10	0.011	0.101	2.4E-04
YDR247W	YDR247W	UWOPS87_Ator08_hmg124h_3.JPG.dat	882.5	27.1	1.0	0.03	-0.089	0.026	4.6E-06
YDR248C	YDR248C	UWOPS87_Ator08_hmg124h_3.JPG.dat	913.3	17.6	1.0	0.03	-0.019	0.026	4.2E-06
YDR249C	YDR249C	UWOPS87_Ator08_hmg124h_3.JPG.dat	896.0	63.0	1.1	0.02	0.001	0.023	1.6E-04
YDR250C	YDR250C	UWOPS87_Ator08_hmg124h_3.JPG.dat	842.8	95.5	1.0	0.11	0.009	0.105	3.3E-04
YDR251W	YDR251W	UWOPS87_Ator08_hmg124h_3.JPG.dat	742.8	118.0	0.9	0.14	-0.025	0.136	1.0E-03
YDR252W	YDR252W	UWOPS87_Ator08_hmg124h_3.JPG.dat	724.3	19.9	0.9	0.01	-0.011	0.013	8.9E-07
YDR253C	YDR253C	UWOPS87_Ator08_hmg124h_3.JPG.dat	852.5	39.4	1.0	0.06	0.022	0.057	4.4E-05
YDR254W	YDR254W	UWOPS87_Ator08_hmg124h_3.JPG.dat	852.8	68.9	1.0	0.09	-0.104	0.093	2.0E-04
YDR255C	YDR255C	UWOPS87_Ator08_hmg124h_3.JPG.dat	866.3	20.0	1.0	0.02	0.014	0.021	2.6E-06
YDR256C	YDR256C	UWOPS87_Ator08_hmg124h_3.JPG.dat	931.5	18.5	1.1	0.03	-0.103	0.033	8.5E-06
YDR257C	YDR257C	UWOPS87_Ator08_hmg124h_3.JPG.dat	873.8	69.4	1.0	0.08	0.095	0.080	1.5E-04
YDR258C	YDR258C	UWOPS87_Ator08_hmg124h_3.JPG.dat	884.5	16.2	1.0	0.01	0.010	0.009	1.8E-07
YDR259C	YDR259C	UWOPS87_Ator08_hmg124h_3.JPG.dat	751.3	29.9	0.9	0.03	-0.111	0.030	1.2E-05
YDR260C	YDR260C	UWOPS87_Ator08_hmg124h_3.JPG.dat	813.0	73.1	0.9	0.09	-0.198	0.089	2.3E-04
YDR261C	YDR261C	UWOPS87_Ator08_hmg124h_3.JPG.dat	776.8	46.8	0.9	0.04	-0.152	0.042	2.7E-05
YDR262W	YDR262W	UWOPS87_Ator08_hmg124h_3.JPG.dat	891.8	91.8	1.0	0.10	0.132	0.102	2.5E-04
YDR263C	YDR263C	UWOPS87_Ator08_hmg124h_3.JPG.dat	835.3	69.2	1.0	0.07	-0.015	0.074	1.0E-04
YDR265W	YDR265W	UWOPS87_Ator08_hmg124h_3.JPG.dat	781.8	61.7	0.9	0.07	-0.004	0.069	1.1E-04
YDR266C	YDR266C	UWOPS87_Ator08_hmg124h_3.JPG.dat	808.0	39.5	1.0	0.03	-0.019	0.033	1.1E-05
YDR270W	YDR270W	UWOPS87_Ator08_hmg124h_3.JPG.dat	940.3	12.8	1.1	0.02	-0.373	0.015	7.4E-07
YDR272W	YDR272W	UWOPS87_Ator08_hmg124h_3.JPG.dat	871.5	47.1	1.0	0.00	-0.016	0.004	6.3E-06
YDR273W	YDR273W	UWOPS87_Ator08_hmg124h_3.JPG.dat	944.5	28.2	1.0	0.02	-0.041	0.021	2.3E-06
YDR274C	YDR274C	UWOPS87_Ator08_hmg124h_3.JPG.dat	844.3	35.5	1.0	0.05	-0.124	0.049	3.6E-05
YDR275W	YDR275W	UWOPS87_Ator08_hmg124h_3.JPG.dat	937.8	34.1	1.1	0.04	-0.076	0.041	1.6E-05
YDR276C	YDR276C	UWOPS87_Ator08_hmg124h_3.JPG.dat	855.0	33.4	1.0	0.04	-0.082	0.044	2.3E-05
YDR277C	YDR277C	UWOPS87_Ator08_hmg124h_3.JPG.dat	911.3	16.6	1.1	0.03	0.081	0.035	9.5E-06
YDR278C	YDR278C	UWOPS87_Ator08_hmg124h_3.JPG.dat	848.8	66.5	1.0	0.08	-0.095	0.081	1.5E-04
YDR279W	YDR279W	UWOPS87_Ator08_hmg124h_3.JPG.dat	928.3	10.4	1.1	0.01	0.058	0.006	8.5E-06
YDR281C	YDR281C	UWOPS87_Ator08_hmg124h_3.JPG.dat	793.8	32.5	1.0	0.04	0.034	0.035	1.3E-05
YDR282C	YDR282C	UWOPS87_Ator08_hmg124h_3.JPG.dat	783.8	77.2	1.0	0.09	0.021	0.092	2.4E-04
YDR284C	YDR284C	UWOPS87_Ator08_hmg124h_3.JPG.dat	815.5	57.6	1.0	0.07	-0.012	0.067	8.6E-05
YDR285W	YDR285W	UWOPS87_Ator08_hmg124h_3.JPG.dat	800.5	83.8	1.0	0.03	0.139	0.034	3.8E-04
YDR286C	YDR286C	UWOPS87_Ator08_hmg124h_3.JPG.dat	803.3	47.5	1.0	0.02	-0.077	0.015	8.2E-05
YDR287W	YDR287W	UWOPS87_Ator08_hmg124h_3.JPG.dat	927.3	75.9	1.1	0.09	-0.018	0.087	1.2E-04
YDR289C	YDR289C	UWOPS87_Ator08_hmg124h_3.JPG.dat	780.5	79.5	0.9	0.03	-0.043	0.030	3.7E-04
YDR291W	YDR291W	UWOPS87_Ator08_hmg124h_3.JPG.dat	871.5	77.8	1.1	0.09	0.006	0.091	1.7E-04
YDR293C	YDR293C	UWOPS87_Ator08_hmg124h_3.JPG.dat	628.5	39.8	0.8	0.05	-0.337	0.053	8.8E-05
YDR294C	YDR294C	UWOPS87_Ator08_hmg124h_3.JPG.dat	802.5	18.2	1.0	0.03	0.052	0.030	8.2E-06
YDR297W	YDR297W	UWOPS87_Ator08_hmg124h_3.JPG.dat	806.3	34.2	1.0	0.04	-0.103	0.044	2.4E-05
YDR304C	YDR304C	UWOPS87_Ator08_hmg124h_3.JPG.dat	757.8	31.4	0.9	0.03	-0.143	0.032	1.3E-05
YDR305C	YDR305C	UWOPS87_Ator08_hmg124h_3.JPG.dat	790.0	67.7	1.0	0.01	-0.011	0.014	7.4E-05
YDR306C	YDR306C	UWOPS87_Ator08_hmg124h_3.JPG.dat	914.3	67.5	1.1	0.06	0.063	0.064	6.3E-05
YDR307W	YDR307W	UWOPS87_Ator08_hmg124h_3.JPG.dat	918.3	111.7	1.1	0.13	-0.009	0.126	4.2E-04
YDR309C	YDR309C	UWOPS87_Ator08_hmg124h_3.JPG.dat	841.3	48.3	1.0	0.05	-0.006	0.054	4.6E-05
YDR310C	YDR310C	UWOPS87_Ator08_hmg124h_3.JPG.dat	869.0	36.4	1.0	0.01	0.026	0.010	2.9E-05
YDR312W	YDR312W	UWOPS87_Ator08_hmg124h_3.JPG.dat	895.8	101.2	1.0	0.10	-0.017	0.096	2.2E-04
YDR313C	YDR313C	UWOPS87_Ator08_hmg124h_3.JPG.dat	934.0	25.4	1.1	0.03	-0.082	0.034	9.2E-06
YDR314C	YDR314C	UWOPS87_Ator08_hmg124h_3.JPG.dat	926.5	48.2	1.1	0.04	0.102	0.042	1.8E-05
YDR315C	YDR315C	UWOPS87_Ator08_hmg124h_3.JPG.dat	904.8	30.1	1.0	0.04	0.268	0.039	1.4E-05
YDR316W	YDR316W	UWOPS87_Ator08_hmg124h_3.JPG.dat	937.3	69.7	1.1	0.06	-0.124	0.056	3.8E-05
YDR317W	YDR317W	UWOPS87_Ator08_hmg124h_3.JPG.dat	697.8	27.1	0.9	0.02	-0.065	0.024	5.3E-06
YDR318W	YDR318W	UWOPS87_Ator08_hmg124h_3.JPG.dat	792.8	67.3	1.0	0.04	-0.023	0.036	3.9E-04
YDR319C	YDR319C	UWOPS87_Ator08_hmg124h_3.JPG.dat	934.0	42.4	1.1	0.04	-0.041	0.044	1.7E-05
YDR320C	YDR320C	UWOPS87_Ator08_hmg124h_3.JPG.dat	591.0	63.3	0.7	0.07	-0.204	0.069	2.6E-04
YDR321W	YDR321W	UWOPS87_Ator08_hmg124h_3.JPG.dat	917.8	104.2	1.1	0.11	0.058	0.110	3.1E-04
YDR329C	YDR329C	UWOPS87_Ator08_hmg124h_3.JPG.dat	923.5	60.4	1.1	0.06	-0.093	0.062	5.3E-05

YDR330W	YDR330W	UWOPS87_Ator08_hmg124h_3.JPG.dat	884.5	101.7	1.0	0.10	-0.025	0.104	2.9E-04
YDR332W	YDR332W	UWOPS87_Ator08_hmg124h_3.JPG.dat	949.3	75.6	1.1	0.08	0.065	0.080	1.0E-04
YDR333C	YDR333C	UWOPS87_Ator08_hmg124h_3.JPG.dat	857.5	72.1	1.0	0.09	0.124	0.092	2.1E-04
YDR334W	YDR334W	UWOPS87_Ator08_hmg124h_3.JPG.dat	688.3	65.8	0.8	0.02	-0.159	0.021	2.4E-04
YDR335W	YDR335W	UWOPS87_Ator08_hmg124h_3.JPG.dat	908.5	88.7	1.1	0.10	-0.070	0.105	2.5E-04
YDR336W	YDR336W	UWOPS87_Ator08_hmg124h_3.JPG.dat	805.0	35.4	1.0	0.04	0.048	0.043	2.0E-05
YDR338C	YDR338C	UWOPS87_Ator08_hmg124h_3.JPG.dat	807.3	106.6	1.0	0.13	-0.008	0.128	5.5E-04
YDR340W	YDR340W	UWOPS87_Ator08_hmg124h_3.JPG.dat	766.3	28.2	0.9	0.03	-0.012	0.034	1.4E-05
YDR344C	YDR344C	UWOPS87_Ator08_hmg124h_3.JPG.dat	964.8	44.8	1.2	0.02	-0.175	0.018	8.4E-05
YDR345C	YDR345C	UWOPS87_Ator08_hmg124h_3.JPG.dat	832.5	46.2	0.9	0.05	0.058	0.049	4.0E-05
YDR346C	YDR346C	UWOPS87_Ator08_hmg124h_3.JPG.dat	843.5	83.6	1.0	0.09	0.071	0.090	2.2E-04
YDR348C	YDR348C	UWOPS87_Ator08_hmg124h_3.JPG.dat	882.5	8.4	1.0	0.01	0.083	0.007	8.9E-08
YDR349C	YDR349C	UWOPS87_Ator08_hmg124h_3.JPG.dat	896.5	39.7	1.1	0.01	0.001	0.009	2.4E-05
YDR351W	YDR351W	UWOPS87_Ator08_hmg124h_3.JPG.dat	829.3	50.3	1.0	0.06	0.023	0.055	4.9E-05
YDR352W	YDR352W	UWOPS87_Ator08_hmg124h_3.JPG.dat	833.3	27.2	1.0	0.04	-0.186	0.038	1.5E-05
YDR354W	YDR354W	UWOPS87_Ator08_hmg124h_3.JPG.dat	723.0	56.8	0.8	0.02	-0.033	0.016	1.3E-04
YDR357C	YDR357C	UWOPS87_Ator08_hmg124h_3.JPG.dat	864.3	80.9	1.1	0.10	0.042	0.100	2.2E-04
YDR358W	YDR358W	UWOPS87_Ator08_hmg124h_3.JPG.dat	799.3	65.6	1.0	0.03	0.052	0.028	2.8E-04
YDR359C	YDR359C	UWOPS87_Ator08_hmg124h_3.JPG.dat	880.8	300.7	1.0	0.36	0.212	0.359	1.0E-02
YDR360W	YDR360W	UWOPS87_Ator08_hmg124h_3.JPG.dat	742.0	82.3	0.9	0.09	-0.111	0.094	3.7E-04
YDR363W	YDR363W	UWOPS87_Ator08_hmg124h_3.JPG.dat	950.3	28.8	1.0	0.01	-0.027	0.007	1.7E-05
YDR363W-	YDR363W-A	UWOPS87_Ator08_hmg124h_3.JPG.dat	785.8	109.8	0.9	0.12	0.026	0.116	6.0E-04
YDR368W	YDR368W	UWOPS87_Ator08_hmg124h_3.JPG.dat	787.3	52.2	0.9	0.00	0.010	0.002	1.3E-06
YDR369C	YDR369C	UWOPS87_Ator08_hmg124h_3.JPG.dat	822.0	35.5	0.9	0.04	0.044	0.040	2.1E-05
YDR370C	YDR370C	UWOPS87_Ator08_hmg124h_3.JPG.dat	941.5	36.1	1.1	0.01	-0.103	0.013	4.8E-05
YDR371W	YDR371W	UWOPS87_Ator08_hmg124h_3.JPG.dat	863.3	63.8	1.0	0.07	-0.056	0.070	8.6E-05
YDR372C	YDR372C	UWOPS87_Ator08_hmg124h_3.JPG.dat	995.0	33.8	1.2	0.05	-0.057	0.048	1.8E-05
YDR374C	YDR374C	UWOPS87_Ator08_hmg124h_3.JPG.dat	821.0	35.5	1.0	0.03	0.020	0.029	6.6E-06
YDR375C	YDR375C	UWOPS87_Ator08_hmg124h_3.JPG.dat	0.0	0.0	0.0	0.00	0.000	0.000	
YDR378C	YDR378C	UWOPS87_Ator08_hmg124h_3.JPG.dat	0.0	0.0	0.0	0.00	0.000	0.000	
YDR379W	YDR379W	UWOPS87_Ator08_hmg124h_3.JPG.dat	911.3	53.8	1.0	0.06	-0.197	0.062	5.8E-05
YDR380W	YDR380W	UWOPS87_Ator08_hmg124h_3.JPG.dat	846.5	102.8	0.9	0.10	0.074	0.103	3.7E-04
YDR382W	YDR382W	UWOPS87_Ator08_hmg124h_3.JPG.dat	0.0	0.0	0.0	0.00	0.000	0.000	
YDR383C	YDR383C	UWOPS87_Ator08_hmg124h_3.JPG.dat	894.5	97.8	1.0	0.11	0.033	0.112	3.5E-04
YDR384C	YDR384C	UWOPS87_Ator08_hmg124h_3.JPG.dat	891.3	14.1	1.0	0.03	0.042	0.026	4.7E-06
YDR385W	YDR385W	UWOPS87_Ator08_hmg124h_3.JPG.dat	912.8	38.8	1.1	0.05	0.136	0.048	2.6E-05
YDR386W	YDR386W	UWOPS87_Ator08_hmg124h_3.JPG.dat	853.0	47.3	1.0	0.05	-0.014	0.048	2.9E-05
YDR387C	YDR387C	UWOPS87_Ator08_hmg124h_3.JPG.dat	841.3	62.2	1.0	0.08	0.067	0.084	1.6E-04
YDR388W	YDR388W	UWOPS87_Ator08_hmg124h_3.JPG.dat	905.3	18.4	1.1	0.03	0.056	0.029	5.0E-06
YDR389W	YDR389W	UWOPS87_Ator08_hmg124h_3.JPG.dat	834.3	149.8	0.9	0.04	0.017	0.036	5.1E-04
YDR391C	YDR391C	UWOPS87_Ator08_hmg124h_3.JPG.dat	836.5	64.7	1.0	0.07	0.003	0.072	1.1E-04
YDR392W	YDR392W	UWOPS87_Ator08_hmg124h_3.JPG.dat	0.0	0.0	0.0	0.00	0.000	0.000	
YDR393W	YDR393W	UWOPS87_Ator08_hmg124h_3.JPG.dat	1009.3	29.4	1.1	0.02	0.038	0.018	1.2E-06
YDR395W	YDR395W	UWOPS87_Ator08_hmg124h_3.JPG.dat	1046.5	54.2	1.2	0.05	0.101	0.050	2.2E-05
YDR399W	YDR399W	UWOPS87_Ator08_hmg124h_3.JPG.dat	813.5	36.4	1.0	0.02	0.018	0.018	1.2E-04
YDR400W	YDR400W	UWOPS87_Ator08_hmg124h_3.JPG.dat	886.8	31.7	1.0	0.00	-0.022	0.004	4.7E-06
YDR401W	YDR401W	UWOPS87_Ator08_hmg124h_3.JPG.dat	812.0	33.9	1.0	0.01	0.019	0.007	1.6E-05
YDR402C	YDR402C	UWOPS87_Ator08_hmg124h_3.JPG.dat	694.5	45.4	0.8	0.05	-0.015	0.053	7.3E-05
YDR403W	YDR403W	UWOPS87_Ator08_hmg124h_3.JPG.dat	796.5	92.0	0.9	0.10	-0.054	0.105	3.9E-04
YDR406W	YDR406W	UWOPS87_Ator08_hmg124h_3.JPG.dat	811.5	51.8	1.0	0.06	-0.066	0.062	6.5E-05
YDR408C	YDR408C	UWOPS87_Ator08_hmg124h_3.JPG.dat	864.8	100.9	1.1	0.12	0.039	0.122	4.0E-04
YDR409W	YDR409W	UWOPS87_Ator08_hmg124h_3.JPG.dat	972.8	35.8	1.2	0.04	-0.178	0.039	1.0E-05
YDR410C	YDR410C	UWOPS87_Ator08_hmg124h_3.JPG.dat	0.0	0.0	0.0	0.00	0.000	0.000	
YDR411C	YDR411C	UWOPS87_Ator08_hmg124h_3.JPG.dat	889.5	39.0	1.0	0.04	0.011	0.043	1.9E-05
YDR414C	YDR414C	UWOPS87_Ator08_hmg124h_3.JPG.dat	1143.5	114.1	1.4	0.16	0.039	0.159	4.3E-04
YDR415C	YDR415C	UWOPS87_Ator08_hmg124h_3.JPG.dat	775.8	76.0	0.9	0.09	0.025	0.088	2.3E-04
YDR419W	YDR419W	UWOPS87_Ator08_hmg124h_3.JPG.dat	850.8	69.6	1.0	0.08	0.092	0.082	1.4E-04
YDR420W	YDR420W	UWOPS87_Ator08_hmg124h_3.JPG.dat	804.8	86.0	1.0	0.11	0.050	0.112	4.0E-04
YDR421W	YDR421W	UWOPS87_Ator08_hmg124h_3.JPG.dat	775.8	39.4	1.0	0.05	-0.023	0.049	3.5E-05
YDR422C	YDR422C	UWOPS87_Ator08_hmg124h_3.JPG.dat	931.0	79.4	1.1	0.09	0.127	0.087	1.3E-04
YOR202W	YOR202W	UWOPS87_Ator08_hmg124h_3.JPG.dat	878.7	122.6	1.0	0.09	0.000	0.092	8.4E-288
1	1	UWOPS87_Ator08_hmg124h_4.JPG.dat	1094.3	151.4	1.1	0.06	0.128	0.061	4.3E-05
2	2	UWOPS87_Ator08_hmg124h_4.JPG.dat	881.8	91.6	0.9	0.03	-0.015	0.032	4.0E-04
3	3	UWOPS87_Ator08_hmg124h_4.JPG.dat	770.0	109.0	0.9	0.11	0.032	0.113	5.5E-04
4	4	UWOPS87_Ator08_hmg124h_4.JPG.dat	948.8	57.9	1.0	0.05	0.001	0.045	2.2E-05
YDR423C	YDR423C	UWOPS87_Ator08_hmg124h_4.JPG.dat	731.3	26.4	1.0	0.05	-0.030	0.053	4.6E-05
YDR424C	YDR424C	UWOPS87_Ator08_hmg124h_4.JPG.dat	690.5	29.1	0.9	0.02	-0.019	0.015	9.9E-05
YDR425W	YDR425W	UWOPS87_Ator08_hmg124h_4.JPG.dat	855.5	29.4	1.0	0.03	-0.060	0.032	8.2E-06
YDR426C	YDR426C	UWOPS87_Ator08_hmg124h_4.JPG.dat	855.3	83.5	1.1	0.02	-0.009	0.020	1.1E-04
YDR428C	YDR428C	UWOPS87_Ator08_hmg124h_4.JPG.dat	803.3	30.7	1.0	0.04	0.104	0.044	2.4E-05

YDR430C	YDR430C	UWOPS87_Ator08_hmg124h_4.JPG.dat	860.0	22.2	1.0	0.03	0.031	0.035	1.1E-05
YDR431W	YDR431W	UWOPS87_Ator08_hmg124h_4.JPG.dat	944.8	30.6	1.1	0.02	0.026	0.020	1.8E-06
YDR435C	YDR435C	UWOPS87_Ator08_hmg124h_4.JPG.dat	955.3	48.3	1.1	0.07	0.130	0.074	8.4E-05
YDR436W	YDR436W	UWOPS87_Ator08_hmg124h_4.JPG.dat	930.3	42.9	1.1	0.05	0.019	0.051	3.0E-05
YDR438W	YDR438W	UWOPS87_Ator08_hmg124h_4.JPG.dat	846.8	45.8	1.0	0.02	-0.042	0.021	1.5E-04
YDR439W	YDR439W	UWOPS87_Ator08_hmg124h_4.JPG.dat	843.8	64.2	1.0	0.01	-0.029	0.012	4.3E-05
YDR440W	YDR440W	UWOPS87_Ator08_hmg124h_4.JPG.dat	935.3	43.0	1.2	0.02	-0.009	0.019	8.4E-05
YDR441C	YDR441C	UWOPS87_Ator08_hmg124h_4.JPG.dat	837.0	53.8	1.0	0.06	0.068	0.062	6.0E-05
YDR445C	YDR445C	UWOPS87_Ator08_hmg124h_4.JPG.dat	963.8	26.6	1.2	0.03	-0.044	0.034	6.8E-06
YDR446W	YDR446W	UWOPS87_Ator08_hmg124h_4.JPG.dat	813.8	13.7	1.0	0.02	0.011	0.023	3.4E-06
YDR447C	YDR447C	UWOPS87_Ator08_hmg124h_4.JPG.dat	750.5	55.7	0.9	0.08	0.067	0.077	1.5E-04
YDR451C	YDR451C	UWOPS87_Ator08_hmg124h_4.JPG.dat	806.0	67.3	0.9	0.03	-0.050	0.030	3.5E-04
YDR452W	YDR452W	UWOPS87_Ator08_hmg124h_4.JPG.dat	762.8	13.9	0.9	0.02	-0.038	0.018	2.2E-06
YDR453C	YDR453C	UWOPS87_Ator08_hmg124h_4.JPG.dat	924.8	64.2	1.1	0.09	-0.016	0.086	1.3E-04
YDR455C	YDR455C	UWOPS87_Ator08_hmg124h_4.JPG.dat	821.0	63.1	0.9	0.08	-0.051	0.079	1.6E-04
YDR458C	YDR458C	UWOPS87_Ator08_hmg124h_4.JPG.dat	859.3	160.3	1.0	0.18	0.001	0.182	1.7E-03
YDR459C	YDR459C	UWOPS87_Ator08_hmg124h_4.JPG.dat	792.8	80.0	0.9	0.02	-0.016	0.021	1.8E-04
YDR463W	YDR463W	UWOPS87_Ator08_hmg124h_4.JPG.dat	702.0	53.2	0.9	0.07	-0.062	0.070	1.3E-04
YDR465C	YDR465C	UWOPS87_Ator08_hmg124h_4.JPG.dat	812.0	31.2	1.0	0.04	0.001	0.036	1.3E-05
YDR466W	YDR466W	UWOPS87_Ator08_hmg124h_4.JPG.dat	866.3	10.6	1.1	0.01	0.043	0.012	4.1E-07
YDR467C	YDR467C	UWOPS87_Ator08_hmg124h_4.JPG.dat	701.0	42.4	0.9	0.02	-0.043	0.020	1.7E-04
YDR469W	YDR469W	UWOPS87_Ator08_hmg124h_4.JPG.dat	702.0	22.1	0.9	0.03	-0.084	0.025	5.7E-06
YDR471W	YDR471W	UWOPS87_Ator08_hmg124h_4.JPG.dat	817.0	58.6	1.0	0.07	-0.117	0.073	9.7E-05
YDR474C	YDR474C	UWOPS87_Ator08_hmg124h_4.JPG.dat	811.5	46.8	1.0	0.05	-0.036	0.050	3.4E-05
YDR475C	YDR475C	UWOPS87_Ator08_hmg124h_4.JPG.dat	767.8	71.6	0.9	0.09	-0.009	0.091	2.4E-04
YDR476C	YDR476C	UWOPS87_Ator08_hmg124h_4.JPG.dat	858.0	52.7	1.0	0.06	-0.065	0.057	5.1E-05
YDR479C	YDR479C	UWOPS87_Ator08_hmg124h_4.JPG.dat	933.0	54.2	1.1	0.06	0.075	0.062	5.3E-05
YDR480W	YDR480W	UWOPS87_Ator08_hmg124h_4.JPG.dat	711.3	35.3	0.9	0.04	-0.032	0.037	2.3E-05
YDR481C	YDR481C	UWOPS87_Ator08_hmg124h_4.JPG.dat	775.3	43.6	1.0	0.05	-0.005	0.054	5.1E-05
YDR482C	YDR482C	UWOPS87_Ator08_hmg124h_4.JPG.dat	927.3	7.6	1.1	0.01	0.002	0.011	2.5E-07
YDR483W	YDR483W	UWOPS87_Ator08_hmg124h_4.JPG.dat	870.3	51.1	1.0	0.06	-0.020	0.060	5.3E-05
YDR485C	YDR485C	UWOPS87_Ator08_hmg124h_4.JPG.dat	626.0	13.6	0.8	0.00	-0.107	0.004	8.9E-06
YDR486C	YDR486C	UWOPS87_Ator08_hmg124h_4.JPG.dat	0.0	0.0	0.0	0.00	0.000	0.000	
YDR488C	YDR488C	UWOPS87_Ator08_hmg124h_4.JPG.dat	726.8	61.4	0.9	0.08	0.026	0.078	1.7E-04
YDR490C	YDR490C	UWOPS87_Ator08_hmg124h_4.JPG.dat	781.3	42.8	1.0	0.06	-0.065	0.055	5.3E-05
YDR491C	YDR491C	UWOPS87_Ator08_hmg124h_4.JPG.dat	816.5	16.3	1.0	0.02	-0.020	0.021	2.5E-06
YDR492W	YDR492W	UWOPS87_Ator08_hmg124h_4.JPG.dat	892.3	55.9	1.0	0.06	-0.069	0.055	4.3E-05
YDR494W	YDR494W	UWOPS87_Ator08_hmg124h_4.JPG.dat	776.0	54.0	0.9	0.06	0.045	0.062	9.3E-05
YDR496C	YDR496C	UWOPS87_Ator08_hmg124h_4.JPG.dat	840.3	37.7	1.0	0.07	0.105	0.065	7.5E-05
YDR497C	YDR497C	UWOPS87_Ator08_hmg124h_4.JPG.dat	948.5	64.9	1.1	0.08	0.109	0.077	8.8E-05
YDR500C	YDR500C	UWOPS87_Ator08_hmg124h_4.JPG.dat	930.8	55.6	1.1	0.06	-0.018	0.062	5.6E-05
YDR501W	YDR501W	UWOPS87_Ator08_hmg124h_4.JPG.dat	846.5	51.5	1.0	0.05	-0.023	0.052	3.9E-05
YDR503C	YDR503C	UWOPS87_Ator08_hmg124h_4.JPG.dat	805.8	44.9	1.0	0.02	0.057	0.022	1.6E-04
YDR504C	YDR504C	UWOPS87_Ator08_hmg124h_4.JPG.dat	715.3	21.2	0.9	0.02	-0.093	0.022	3.8E-06
YDR505C	YDR505C	UWOPS87_Ator08_hmg124h_4.JPG.dat	822.3	13.0	1.0	0.02	0.026	0.018	1.5E-06
YDR506C	YDR506C	UWOPS87_Ator08_hmg124h_4.JPG.dat	798.3	22.8	1.0	0.03	-0.023	0.032	9.9E-06
YDR508C	YDR508C	UWOPS87_Ator08_hmg124h_4.JPG.dat	769.0	22.4	0.9	0.04	-0.098	0.036	1.6E-05
YDR509W	YDR509W	UWOPS87_Ator08_hmg124h_4.JPG.dat	751.8	39.0	0.9	0.05	-0.007	0.047	4.7E-05
YDR511W	YDR511W	UWOPS87_Ator08_hmg124h_4.JPG.dat	894.8	85.6	1.0	0.10	-0.052	0.097	2.5E-04
YDR512C	YDR512C	UWOPS87_Ator08_hmg124h_4.JPG.dat	893.8	39.1	1.1	0.06	0.079	0.057	4.3E-05
YDR513W	YDR513W	UWOPS87_Ator08_hmg124h_4.JPG.dat	764.5	56.3	0.9	0.07	-0.130	0.072	1.3E-04
YDR514C	YDR514C	UWOPS87_Ator08_hmg124h_4.JPG.dat	916.3	7.8	1.1	0.01	-0.092	0.008	1.3E-07
YDR516C	YDR516C	UWOPS87_Ator08_hmg124h_4.JPG.dat	828.3	42.1	1.0	0.06	0.002	0.055	5.0E-05
YDR517W	YDR517W	UWOPS87_Ator08_hmg124h_4.JPG.dat	775.5	58.1	1.0	0.03	0.026	0.026	2.4E-04
YDR519W	YDR519W	UWOPS87_Ator08_hmg124h_4.JPG.dat	938.3	84.6	1.1	0.04	-0.079	0.038	4.0E-04
YDR520C	YDR520C	UWOPS87_Ator08_hmg124h_4.JPG.dat	752.5	26.1	0.9	0.03	0.070	0.032	1.1E-05
YDR522C	YDR522C	UWOPS87_Ator08_hmg124h_4.JPG.dat	757.5	30.2	0.9	0.04	-0.005	0.039	2.2E-05
YDR524C	YDR524C	UWOPS87_Ator08_hmg124h_4.JPG.dat	839.8	59.3	1.0	0.09	0.079	0.088	1.7E-04
YDR525W	YDR525W	UWOPS87_Ator08_hmg124h_4.JPG.dat	953.8	74.3	1.2	0.01	0.087	0.013	4.3E-05
YDR525W-	YDR525W-A	UWOPS87_Ator08_hmg124h_4.JPG.dat	867.8	276.1	0.9	0.09	-0.103	0.091	3.8E-03
YDR528W	YDR528W	UWOPS87_Ator08_hmg124h_4.JPG.dat	795.0	13.8	1.0	0.03	0.009	0.027	6.0E-06
YDR530C	YDR530C	UWOPS87_Ator08_hmg124h_4.JPG.dat	666.8	26.0	0.8	0.03	0.023	0.029	1.3E-05
YDR532C	YDR532C	UWOPS87_Ator08_hmg124h_4.JPG.dat	649.5	434.8	1.0	0.08	0.119	0.080	2.0E-03
YDR533C	YDR533C	UWOPS87_Ator08_hmg124h_4.JPG.dat	831.5	59.7	1.0	0.06	0.002	0.064	7.2E-05
YDR534C	YDR534C	UWOPS87_Ator08_hmg124h_4.JPG.dat	909.8	42.8	1.1	0.03	0.056	0.033	7.2E-06
YDR535C	YDR535C	UWOPS87_Ator08_hmg124h_4.JPG.dat	832.3	31.4	1.0	0.03	0.102	0.029	5.8E-06
YDR536W	YDR536W	UWOPS87_Ator08_hmg124h_4.JPG.dat	815.3	102.1	1.0	0.11	0.005	0.109	3.5E-04
YDR537C	YDR537C	UWOPS87_Ator08_hmg124h_4.JPG.dat	764.8	52.5	0.9	0.04	-0.028	0.043	2.6E-05
YDR538W	YDR538W	UWOPS87_Ator08_hmg124h_4.JPG.dat	752.3	16.1	1.0	0.04	-0.038	0.037	1.5E-05
YDR539W	YDR539W	UWOPS87_Ator08_hmg124h_4.JPG.dat	740.5	69.1	0.9	0.08	-0.051	0.080	1.8E-04

YDR540C	YDR540C	UWOPS87_Ator08_hmg124h_4.JPG.dat	646.8	442.6	1.1	0.14	0.069	0.142	5.8E-03
YDR541C	YDR541C	UWOPS87_Ator08_hmg124h_4.JPG.dat	739.0	31.2	0.9	0.02	-0.011	0.016	1.5E-06
YEL001C	YEL001C	UWOPS87_Ator08_hmg124h_4.JPG.dat	686.3	32.5	0.9	0.04	-0.152	0.041	2.6E-05
YEL003W	YEL003W	UWOPS87_Ator08_hmg124h_4.JPG.dat	765.5	38.1	1.0	0.05	-0.142	0.054	4.5E-05
YEL004W	YEL004W	UWOPS87_Ator08_hmg124h_4.JPG.dat	778.0	41.4	1.0	0.04	0.095	0.043	2.3E-05
YEL005C	YEL005C	UWOPS87_Ator08_hmg124h_4.JPG.dat	731.8	18.3	0.9	0.03	0.001	0.025	5.9E-06
YEL006W	YEL006W	UWOPS87_Ator08_hmg124h_4.JPG.dat	956.5	7.6	1.2	0.01	0.042	0.010	2.0E-07
YEL007W	YEL007W	UWOPS87_Ator08_hmg124h_4.JPG.dat	884.8	31.7	1.1	0.03	-0.011	0.035	9.7E-06
YEL008W	YEL008W	UWOPS87_Ator08_hmg124h_4.JPG.dat	876.0	100.3	1.0	0.11	-0.054	0.110	3.4E-04
YEL010W	YEL010W	UWOPS87_Ator08_hmg124h_4.JPG.dat	847.3	37.9	1.0	0.04	-0.019	0.041	2.2E-05
YEL011W	YEL011W	UWOPS87_Ator08_hmg124h_4.JPG.dat	812.8	38.7	0.9	0.01	-0.057	0.008	2.3E-05
YEL012W	YEL012W	UWOPS87_Ator08_hmg124h_4.JPG.dat	754.0	508.1	1.2	0.12	0.287	0.118	3.5E-03
YEL013W	YEL013W	UWOPS87_Ator08_hmg124h_4.JPG.dat	908.3	33.8	1.0	0.01	0.232	0.008	1.8E-05
YEL014C	YEL014C	UWOPS87_Ator08_hmg124h_4.JPG.dat	586.8	392.6	1.0	0.05	0.043	0.051	7.9E-04
YEL015W	YEL015W	UWOPS87_Ator08_hmg124h_4.JPG.dat	782.3	51.6	1.0	0.06	0.086	0.055	4.6E-05
YEL016C	YEL016C	UWOPS87_Ator08_hmg124h_4.JPG.dat	761.3	30.3	1.0	0.01	0.014	0.013	6.1E-05
YEL017C-A	YEL017C-A	UWOPS87_Ator08_hmg124h_4.JPG.dat	907.8	58.1	1.1	0.08	0.018	0.075	8.5E-05
YEL017W	YEL017W	UWOPS87_Ator08_hmg124h_4.JPG.dat	894.3	27.7	1.1	0.04	0.000	0.041	1.5E-05
YEL020C	YEL020C	UWOPS87_Ator08_hmg124h_4.JPG.dat	874.3	36.8	1.0	0.02	0.010	0.020	1.2E-04
YEL023C	YEL023C	UWOPS87_Ator08_hmg124h_4.JPG.dat	760.0	41.5	0.9	0.01	-0.083	0.008	3.0E-05
YEL025C	YEL025C	UWOPS87_Ator08_hmg124h_4.JPG.dat	908.8	226.3	0.9	0.05	-0.127	0.047	8.6E-04
YEL028W	YEL028W	UWOPS87_Ator08_hmg124h_4.JPG.dat	790.3	66.7	1.0	0.07	0.106	0.067	9.3E-05
YEL030W	YEL030W	UWOPS87_Ator08_hmg124h_4.JPG.dat	792.8	48.8	0.9	0.06	-0.048	0.056	6.5E-05
YEL031W	YEL031W	UWOPS87_Ator08_hmg124h_4.JPG.dat	687.3	185.6	0.8	0.21	0.043	0.211	5.0E-03
YEL033W	YEL033W	UWOPS87_Ator08_hmg124h_4.JPG.dat	739.0	17.8	1.0	0.02	0.241	0.018	1.8E-06
YEL037C	YEL037C	UWOPS87_Ator08_hmg124h_4.JPG.dat	800.5	37.5	1.0	0.06	0.002	0.056	4.7E-05
YEL038W	YEL038W	UWOPS87_Ator08_hmg124h_4.JPG.dat	952.5	32.8	1.2	0.01	0.064	0.011	2.9E-05
YEL039C	YEL039C	UWOPS87_Ator08_hmg124h_4.JPG.dat	805.5	16.3	1.0	0.02	-0.004	0.018	1.6E-06
YEL040W	YEL040W	UWOPS87_Ator08_hmg124h_4.JPG.dat	757.0	21.7	1.0	0.03	0.041	0.030	8.4E-06
YEL041W	YEL041W	UWOPS87_Ator08_hmg124h_4.JPG.dat	783.8	46.2	1.0	0.06	-0.009	0.062	6.3E-05
YEL042W	YEL042W	UWOPS87_Ator08_hmg124h_4.JPG.dat	891.5	19.1	1.1	0.01	0.167	0.005	7.5E-06
YEL043W	YEL043W	UWOPS87_Ator08_hmg124h_4.JPG.dat	970.0	56.8	1.2	0.07	0.142	0.070	5.7E-05
YEL047C	YEL047C	UWOPS87_Ator08_hmg124h_4.JPG.dat	788.5	54.3	1.0	0.02	-0.016	0.016	9.0E-05
YEL048C	YEL048C	UWOPS87_Ator08_hmg124h_4.JPG.dat	828.8	75.4	1.0	0.09	0.061	0.088	2.0E-04
YEL049W	YEL049W	UWOPS87_Ator08_hmg124h_4.JPG.dat	895.5	61.2	1.0	0.07	-0.120	0.068	7.7E-05
YEL052W	YEL052W	UWOPS87_Ator08_hmg124h_4.JPG.dat	929.3	56.3	1.2	0.06	0.012	0.061	4.0E-05
YEL053C	YEL053C	UWOPS87_Ator08_hmg124h_4.JPG.dat	758.0	33.8	0.9	0.04	-0.139	0.037	1.8E-05
YEL056W	YEL056W	UWOPS87_Ator08_hmg124h_4.JPG.dat	836.3	30.5	1.0	0.04	0.058	0.036	1.3E-05
YEL057C	YEL057C	UWOPS87_Ator08_hmg124h_4.JPG.dat	840.3	20.9	1.0	0.01	-0.136	0.012	4.8E-05
YEL059W	YEL059W	UWOPS87_Ator08_hmg124h_4.JPG.dat	759.5	17.7	1.0	0.03	0.006	0.027	5.9E-06
YEL060C	YEL060C	UWOPS87_Ator08_hmg124h_4.JPG.dat	818.0	30.2	1.0	0.01	0.021	0.012	4.2E-05
YEL061C	YEL061C	UWOPS87_Ator08_hmg124h_4.JPG.dat	856.8	43.7	1.1	0.06	0.069	0.056	3.9E-05
YEL062W	YEL062W	UWOPS87_Ator08_hmg124h_4.JPG.dat	466.0	543.7	0.6	0.66	0.213	0.656	1.8E-01
YEL063C	YEL063C	UWOPS87_Ator08_hmg124h_4.JPG.dat	0.0	0.0	0.0	0.00	0.000	0.000	
YEL064C	YEL064C	UWOPS87_Ator08_hmg124h_4.JPG.dat	917.0	77.6	1.1	0.09	0.036	0.089	1.6E-04
YEL065W	YEL065W	UWOPS87_Ator08_hmg124h_4.JPG.dat	813.8	49.3	0.9	0.05	-0.057	0.054	5.3E-05
YEL066W	YEL066W	UWOPS87_Ator08_hmg124h_4.JPG.dat	886.0	29.8	1.1	0.01	0.072	0.009	2.2E-05
YEL067C	YEL067C	UWOPS87_Ator08_hmg124h_4.JPG.dat	932.0	32.0	1.1	0.05	-0.062	0.046	2.0E-05
YEL068C	YEL068C	UWOPS87_Ator08_hmg124h_4.JPG.dat	860.3	64.8	1.0	0.08	0.060	0.077	1.3E-04
YEL071W	YEL071W	UWOPS87_Ator08_hmg124h_4.JPG.dat	806.3	27.5	1.0	0.01	-0.005	0.006	1.1E-05
YER001W	YER001W	UWOPS87_Ator08_hmg124h_4.JPG.dat	770.3	21.9	1.0	0.03	0.001	0.028	6.9E-06
YER002W	YER002W	UWOPS87_Ator08_hmg124h_4.JPG.dat	821.5	49.8	1.0	0.06	0.074	0.056	4.5E-05
YER004W	YER004W	UWOPS87_Ator08_hmg124h_4.JPG.dat	808.0	40.0	1.0	0.01	0.014	0.010	3.6E-05
YER005W	YER005W	UWOPS87_Ator08_hmg124h_4.JPG.dat	1007.0	51.7	1.2	0.06	0.088	0.059	3.4E-05
YER007C-A	YER007C-A	UWOPS87_Ator08_hmg124h_4.JPG.dat	760.3	63.5	1.0	0.02	-0.046	0.015	8.7E-05
YER007W	YER007W	UWOPS87_Ator08_hmg124h_4.JPG.dat	990.8	48.6	1.1	0.05	-0.012	0.053	2.9E-05
YER010C	YER010C	UWOPS87_Ator08_hmg124h_4.JPG.dat	1004.3	95.9	1.1	0.11	0.030	0.114	2.8E-04
YER011W	YER011W	UWOPS87_Ator08_hmg124h_4.JPG.dat	797.8	62.6	1.0	0.08	0.194	0.077	1.3E-04
YER016W	YER016W	UWOPS87_Ator08_hmg124h_4.JPG.dat	821.0	30.6	1.0	0.04	0.005	0.041	2.0E-05
YER019C-A	YER019C-A	UWOPS87_Ator08_hmg124h_4.JPG.dat	853.5	106.2	1.0	0.12	0.060	0.120	4.7E-04
YER019W	YER019W	UWOPS87_Ator08_hmg124h_4.JPG.dat	925.8	123.7	1.1	0.15	0.030	0.148	6.3E-04
YER020W	YER020W	UWOPS87_Ator08_hmg124h_4.JPG.dat	935.0	37.4	1.1	0.05	0.042	0.046	1.9E-05
YER024W	YER024W	UWOPS87_Ator08_hmg124h_4.JPG.dat	842.8	75.8	1.0	0.09	-0.042	0.090	1.8E-04
YER027C	YER027C	UWOPS87_Ator08_hmg124h_4.JPG.dat	884.8	19.3	1.1	0.01	0.010	0.006	1.2E-05
YER028C	YER028C	UWOPS87_Ator08_hmg124h_4.JPG.dat	881.8	42.9	1.1	0.05	-0.036	0.048	2.7E-05
YER030W	YER030W	UWOPS87_Ator08_hmg124h_4.JPG.dat	717.8	50.6	0.9	0.02	0.001	0.018	1.2E-04
YER031C	YER031C	UWOPS87_Ator08_hmg124h_4.JPG.dat	673.3	96.0	0.8	0.11	0.082	0.113	7.8E-04
YER032W	YER032W	UWOPS87_Ator08_hmg124h_4.JPG.dat	926.3	38.4	1.1	0.05	0.019	0.047	2.3E-05
YER033C	YER033C	UWOPS87_Ator08_hmg124h_4.JPG.dat	927.3	31.3	1.1	0.03	-0.039	0.032	5.9E-06
YER034W	YER034W	UWOPS87_Ator08_hmg124h_4.JPG.dat	790.3	44.2	1.0	0.05	0.152	0.050	3.9E-05

YER035W	YER035W	UWOPS87_Ator08_hmg124h_4.JPG.dat	760.0	36.6	0.9	0.04	-0.104	0.044	2.9E-05
YER038W-.	YER038W-A	UWOPS87_Ator08_hmg124h_4.JPG.dat	879.0	26.7	1.1	0.01	0.011	0.013	4.4E-05
YER039C	YER039C	UWOPS87_Ator08_hmg124h_4.JPG.dat	856.3	36.5	1.1	0.01	0.022	0.007	1.3E-05
YER039C-A	YER039C-A	UWOPS87_Ator08_hmg124h_4.JPG.dat	840.8	62.9	1.0	0.06	0.037	0.058	4.6E-05
YER041W	YER041W	UWOPS87_Ator08_hmg124h_4.JPG.dat	928.8	86.5	1.1	0.08	0.080	0.081	9.8E-05
YER042W	YER042W	UWOPS87_Ator08_hmg124h_4.JPG.dat	727.0	41.8	0.9	0.02	-0.031	0.018	1.3E-04
YER044C-A	YER044C-A	UWOPS87_Ator08_hmg124h_4.JPG.dat	817.0	55.0	1.1	0.09	-0.108	0.088	1.5E-04
YER045C	YER045C	UWOPS87_Ator08_hmg124h_4.JPG.dat	754.8	46.3	0.9	0.05	-0.088	0.045	3.1E-05
YER046W	YER046W	UWOPS87_Ator08_hmg124h_4.JPG.dat	826.5	106.9	0.9	0.04	0.000	0.038	5.7E-04
YER046W-.	YER046W-A	UWOPS87_Ator08_hmg124h_4.JPG.dat	690.0	17.4	0.8	0.01	0.003	0.007	2.2E-05
YER047C	YER047C	UWOPS87_Ator08_hmg124h_4.JPG.dat	812.5	75.7	1.0	0.09	0.027	0.085	1.8E-04
YER048C	YER048C	UWOPS87_Ator08_hmg124h_4.JPG.dat	814.8	25.1	1.0	0.00	0.006	0.001	1.6E-07
YER049W	YER049W	UWOPS87_Ator08_hmg124h_4.JPG.dat	811.0	88.9	1.0	0.12	0.042	0.121	5.1E-04
YER051W	YER051W	UWOPS87_Ator08_hmg124h_4.JPG.dat	748.0	37.8	0.9	0.04	-0.065	0.043	2.9E-05
YER052C	YER052C	UWOPS87_Ator08_hmg124h_4.JPG.dat	892.5	63.5	1.0	0.06	0.019	0.057	4.5E-05
YER053C	YER053C	UWOPS87_Ator08_hmg124h_4.JPG.dat	986.3	41.9	1.1	0.06	0.096	0.065	5.5E-05
YER054C	YER054C	UWOPS87_Ator08_hmg124h_4.JPG.dat	1030.3	20.1	1.2	0.04	0.047	0.041	1.3E-05
YER055C	YER055C	UWOPS87_Ator08_hmg124h_4.JPG.dat	987.8	79.9	1.1	0.10	0.000	0.096	1.8E-04
YER056C	YER056C	UWOPS87_Ator08_hmg124h_4.JPG.dat	571.3	385.7	0.9	0.08	-0.139	0.078	2.7E-03
YER056C-A	YER056C-A	UWOPS87_Ator08_hmg124h_4.JPG.dat	873.5	172.0	1.0	0.18	-0.129	0.181	1.5E-03
YER057C	YER057C	UWOPS87_Ator08_hmg124h_4.JPG.dat	732.3	102.1	0.9	0.04	-0.022	0.042	8.1E-04
YER059W	YER059W	UWOPS87_Ator08_hmg124h_4.JPG.dat	883.8	76.9	1.0	0.02	-0.117	0.017	9.7E-05
YER060W	YER060W	UWOPS87_Ator08_hmg124h_4.JPG.dat	865.8	93.2	1.0	0.03	-0.060	0.035	3.7E-04
YER060W-.	YER060W-A	UWOPS87_Ator08_hmg124h_4.JPG.dat	901.3	12.8	1.0	0.01	-0.009	0.015	8.1E-07
YER061C	YER061C	UWOPS87_Ator08_hmg124h_4.JPG.dat	0.0	0.0	0.0	0.00	0.000	0.000	
YER062C	YER062C	UWOPS87_Ator08_hmg124h_4.JPG.dat	834.0	79.4	0.9	0.01	0.027	0.008	2.3E-05
YER063W	YER063W	UWOPS87_Ator08_hmg124h_4.JPG.dat	919.8	60.7	1.0	0.06	-0.028	0.061	6.1E-05
YER064C	YER064C	UWOPS87_Ator08_hmg124h_4.JPG.dat	779.5	72.7	0.9	0.09	-0.004	0.086	2.7E-04
YER065C	YER065C	UWOPS87_Ator08_hmg124h_4.JPG.dat	1020.5	27.0	1.1	0.03	-0.014	0.034	7.8E-06
YER066C-A	YER066C-A	UWOPS87_Ator08_hmg124h_4.JPG.dat	945.0	95.7	1.0	0.10	-0.050	0.104	2.8E-04
YER066W	YER066W	UWOPS87_Ator08_hmg124h_4.JPG.dat	862.0	41.4	0.9	0.01	-0.169	0.012	5.0E-05
YER067C-A	YER067C-A	UWOPS87_Ator08_hmg124h_4.JPG.dat	917.3	50.8	1.1	0.06	0.005	0.062	4.8E-05
YER067W	YER067W	UWOPS87_Ator08_hmg124h_4.JPG.dat	873.5	68.8	1.0	0.08	0.087	0.080	1.4E-04
YER069W	YER069W	UWOPS87_Ator08_hmg124h_4.JPG.dat	0.0	0.0	0.0	0.00	0.000	0.000	
YER071C	YER071C	UWOPS87_Ator08_hmg124h_4.JPG.dat	900.5	32.8	1.0	0.03	-0.045	0.032	8.0E-06
YER072W	YER072W	UWOPS87_Ator08_hmg124h_4.JPG.dat	915.8	13.9	1.1	0.01	0.139	0.015	6.9E-07
YER073W	YER073W	UWOPS87_Ator08_hmg124h_4.JPG.dat	859.0	31.2	1.0	0.03	-0.040	0.034	1.1E-05
YER074W	YER074W	UWOPS87_Ator08_hmg124h_4.JPG.dat	891.8	42.8	1.0	0.04	-0.011	0.045	2.5E-05
YER075C	YER075C	UWOPS87_Ator08_hmg124h_4.JPG.dat	905.3	32.1	1.0	0.05	-0.043	0.045	2.4E-05
YER077C	YER077C	UWOPS87_Ator08_hmg124h_4.JPG.dat	980.3	30.5	1.1	0.03	0.040	0.029	5.7E-06
YER078C	YER078C	UWOPS87_Ator08_hmg124h_4.JPG.dat	995.3	49.5	1.1	0.05	0.030	0.052	3.2E-05
YER079W	YER079W	UWOPS87_Ator08_hmg124h_4.JPG.dat	927.5	30.4	1.0	0.02	0.016	0.019	1.8E-06
YER080W	YER080W	UWOPS87_Ator08_hmg124h_4.JPG.dat	876.5	104.2	1.0	0.12	0.062	0.120	4.5E-04
YER081W	YER081W	UWOPS87_Ator08_hmg124h_4.JPG.dat	922.0	36.2	1.0	0.04	-0.055	0.040	1.7E-05
YER083C	YER083C	UWOPS87_Ator08_hmg124h_4.JPG.dat	1010.8	27.0	1.1	0.02	0.191	0.022	2.0E-06
YER084W	YER084W	UWOPS87_Ator08_hmg124h_4.JPG.dat	851.3	13.3	1.0	0.02	-0.108	0.018	1.5E-06
YER085C	YER085C	UWOPS87_Ator08_hmg124h_4.JPG.dat	787.0	36.3	1.0	0.01	-0.020	0.008	2.2E-05
YER087C-A	YER087C-A	UWOPS87_Ator08_hmg124h_4.JPG.dat	900.0	29.7	1.1	0.04	-0.052	0.041	1.6E-05
YER088C	YER088C	UWOPS87_Ator08_hmg124h_4.JPG.dat	887.0	11.7	1.0	0.01	-0.013	0.013	5.3E-07
YER090W	YER090W	UWOPS87_Ator08_hmg124h_4.JPG.dat	969.5	19.9	1.1	0.01	0.005	0.006	8.7E-06
YER091C	YER091C	UWOPS87_Ator08_hmg124h_4.JPG.dat	961.3	49.8	1.1	0.02	0.100	0.017	8.2E-05
YER091C-A	YER091C-A	UWOPS87_Ator08_hmg124h_4.JPG.dat	950.5	53.3	1.0	0.01	-0.071	0.013	6.0E-05
YER092W	YER092W	UWOPS87_Ator08_hmg124h_4.JPG.dat	892.3	89.5	1.0	0.09	0.095	0.090	2.0E-04
YER093C-A	YER093C-A	UWOPS87_Ator08_hmg124h_4.JPG.dat	912.8	15.9	1.0	0.02	-0.086	0.023	2.9E-06
YER095W	YER095W	UWOPS87_Ator08_hmg124h_4.JPG.dat	874.3	67.9	1.0	0.02	0.177	0.024	2.0E-04
YER096W	YER096W	UWOPS87_Ator08_hmg124h_4.JPG.dat	944.8	17.0	1.1	0.02	0.001	0.017	1.2E-06
YER097W	YER097W	UWOPS87_Ator08_hmg124h_4.JPG.dat	830.8	25.4	1.0	0.02	-0.070	0.024	3.6E-06
YER098W	YER098W	UWOPS87_Ator08_hmg124h_4.JPG.dat	831.0	7.1	1.0	0.01	-0.063	0.013	6.1E-07
YER101C	YER101C	UWOPS87_Ator08_hmg124h_4.JPG.dat	859.5	33.6	1.0	0.04	-0.030	0.043	1.9E-05
YER106W	YER106W	UWOPS87_Ator08_hmg124h_4.JPG.dat	950.8	59.3	1.1	0.07	0.067	0.074	8.4E-05
YER108C	YER108C	UWOPS87_Ator08_hmg124h_4.JPG.dat	917.3	18.1	1.1	0.00	0.003	0.003	3.4E-06
YER109C	YER109C	UWOPS87_Ator08_hmg124h_4.JPG.dat	1002.0	30.6	1.1	0.04	0.024	0.038	1.2E-05
YER111C	YER111C	UWOPS87_Ator08_hmg124h_4.JPG.dat	1002.8	78.1	1.1	0.09	0.057	0.087	1.4E-04
YER113C	YER113C	UWOPS87_Ator08_hmg124h_4.JPG.dat	958.8	75.1	1.0	0.01	0.023	0.006	9.9E-06
YER114C	YER114C	UWOPS87_Ator08_hmg124h_4.JPG.dat	900.0	77.6	1.1	0.02	0.147	0.022	1.3E-04
YER115C	YER115C	UWOPS87_Ator08_hmg124h_4.JPG.dat	906.3	56.3	1.0	0.06	-0.065	0.061	6.1E-05
YER116C	YER116C	UWOPS87_Ator08_hmg124h_4.JPG.dat	875.3	29.2	1.0	0.03	0.012	0.028	6.3E-06
YER117W	YER117W	UWOPS87_Ator08_hmg124h_4.JPG.dat	912.8	24.3	1.1	0.04	-0.145	0.037	1.1E-05
YER118C	YER118C	UWOPS87_Ator08_hmg124h_4.JPG.dat	813.8	45.3	1.0	0.06	-0.023	0.056	4.9E-05
YER119C	YER119C	UWOPS87_Ator08_hmg124h_4.JPG.dat	898.8	15.8	1.1	0.01	-0.057	0.014	6.4E-07

YER119C-A	YER119C-A	UWOPS87_Ator08_hmg124h_4.JPG.dat	920.8	72.4	1.1	0.08	0.071	0.080	1.1E-04
YER120W	YER120W	UWOPS87_Ator08_hmg124h_4.JPG.dat	906.5	16.7	1.1	0.02	-0.017	0.019	1.6E-06
YER121W	YER121W	UWOPS87_Ator08_hmg124h_4.JPG.dat	865.3	78.2	1.0	0.01	-0.011	0.011	3.6E-05
YER123W	YER123W	UWOPS87_Ator08_hmg124h_4.JPG.dat	980.0	23.8	1.1	0.03	0.098	0.027	4.3E-06
YER124C	YER124C	UWOPS87_Ator08_hmg124h_4.JPG.dat	906.5	58.7	1.1	0.07	-0.124	0.069	7.7E-05
YER128W	YER128W	UWOPS87_Ator08_hmg124h_4.JPG.dat	944.3	19.0	1.1	0.02	-0.202	0.025	3.4E-06
YER129W	YER129W	UWOPS87_Ator08_hmg124h_4.JPG.dat	891.8	41.7	1.0	0.01	-0.068	0.005	1.0E-05
YER130C	YER130C	UWOPS87_Ator08_hmg124h_4.JPG.dat	847.3	85.2	0.9	0.03	-0.061	0.031	3.8E-04
YER131W	YER131W	UWOPS87_Ator08_hmg124h_4.JPG.dat	820.0	18.9	1.0	0.00	0.094	0.003	4.3E-06
YER132C	YER132C	UWOPS87_Ator08_hmg124h_4.JPG.dat	915.0	17.0	1.1	0.02	-0.008	0.016	8.7E-07
YER134C	YER134C	UWOPS87_Ator08_hmg124h_4.JPG.dat	880.0	70.1	1.0	0.08	0.029	0.080	1.4E-04
YER135C	YER135C	UWOPS87_Ator08_hmg124h_4.JPG.dat	886.3	81.2	1.0	0.09	-0.033	0.094	2.1E-04
YER137C	YER137C	UWOPS87_Ator08_hmg124h_4.JPG.dat	782.0	18.0	0.9	0.03	-0.073	0.034	1.4E-05
YER139C	YER139C	UWOPS87_Ator08_hmg124h_4.JPG.dat	1004.0	26.0	1.1	0.04	-0.014	0.037	9.6E-06
YER140W	YER140W	UWOPS87_Ator08_hmg124h_4.JPG.dat	990.0	22.6	1.1	0.03	-0.001	0.026	3.6E-06
YER142C	YER142C	UWOPS87_Ator08_hmg124h_4.JPG.dat	611.8	413.5	0.9	0.08	0.049	0.080	2.4E-03
YER143W	YER143W	UWOPS87_Ator08_hmg124h_4.JPG.dat	768.8	34.8	1.0	0.05	-0.141	0.049	3.7E-05
YER144C	YER144C	UWOPS87_Ator08_hmg124h_4.JPG.dat	746.5	13.7	0.9	0.02	-0.113	0.021	3.3E-06
YER145C	YER145C	UWOPS87_Ator08_hmg124h_4.JPG.dat	878.0	56.0	1.1	0.01	0.080	0.012	4.0E-05
YER149C	YER149C	UWOPS87_Ator08_hmg124h_4.JPG.dat	868.8	99.9	1.1	0.13	0.030	0.133	5.3E-04
YER150W	YER150W	UWOPS87_Ator08_hmg124h_4.JPG.dat	997.8	37.7	1.2	0.04	0.094	0.038	8.5E-06
YER151C	YER151C	UWOPS87_Ator08_hmg124h_4.JPG.dat	879.0	33.4	1.1	0.01	0.072	0.008	1.8E-05
YER152C	YER152C	UWOPS87_Ator08_hmg124h_4.JPG.dat	944.8	47.4	1.1	0.05	0.051	0.047	2.2E-05
YER153C	YER153C	UWOPS87_Ator08_hmg124h_4.JPG.dat	905.8	709.4	1.0	0.77	-0.076	0.771	8.3E-02
YER155C	YER155C	UWOPS87_Ator08_hmg124h_4.JPG.dat	857.5	13.4	1.0	0.01	0.204	0.011	3.5E-07
YER156C	YER156C	UWOPS87_Ator08_hmg124h_4.JPG.dat	760.8	43.4	0.9	0.06	0.015	0.058	8.2E-05
YER158C	YER158C	UWOPS87_Ator08_hmg124h_4.JPG.dat	927.3	79.5	1.0	0.08	0.021	0.076	1.1E-04
YER161C	YER161C	UWOPS87_Ator08_hmg124h_4.JPG.dat	763.3	39.4	1.0	0.04	0.106	0.042	2.3E-05
YER162C	YER162C	UWOPS87_Ator08_hmg124h_4.JPG.dat	863.8	71.2	1.1	0.09	0.020	0.090	1.7E-04
YER163C	YER163C	UWOPS87_Ator08_hmg124h_4.JPG.dat	831.0	47.9	1.0	0.05	0.046	0.050	3.5E-05
YER164W	YER164W	UWOPS87_Ator08_hmg124h_4.JPG.dat	789.0	14.1	0.9	0.02	0.072	0.017	1.7E-06
YER166W	YER166W	UWOPS87_Ator08_hmg124h_4.JPG.dat	813.0	41.8	0.9	0.05	0.054	0.051	4.4E-05
YER167W	YER167W	UWOPS87_Ator08_hmg124h_4.JPG.dat	1057.5	20.9	1.2	0.03	0.121	0.028	3.5E-06
YER170W	YER170W	UWOPS87_Ator08_hmg124h_4.JPG.dat	905.5	10.8	1.0	0.01	-0.028	0.012	5.1E-07
YER173W	YER173W	UWOPS87_Ator08_hmg124h_4.JPG.dat	894.0	47.2	1.0	0.05	0.068	0.049	3.6E-05
YER174C	YER174C	UWOPS87_Ator08_hmg124h_4.JPG.dat	863.5	65.3	1.0	0.07	-0.014	0.070	1.0E-04
YER175C	YER175C	UWOPS87_Ator08_hmg124h_4.JPG.dat	1005.0	24.2	1.1	0.02	0.076	0.024	2.9E-06
YER176W	YER176W	UWOPS87_Ator08_hmg124h_4.JPG.dat	937.8	116.7	1.1	0.05	-0.060	0.045	5.8E-04
YER177W	YER177W	UWOPS87_Ator08_hmg124h_4.JPG.dat	822.8	17.3	1.0	0.02	0.017	0.018	1.5E-06
YER178W	YER178W	UWOPS87_Ator08_hmg124h_4.JPG.dat	863.8	63.7	1.0	0.08	0.093	0.077	1.2E-04
YER179W	YER179W	UWOPS87_Ator08_hmg124h_4.JPG.dat	778.3	63.8	0.9	0.08	0.020	0.076	1.7E-04
YER180C	YER180C	UWOPS87_Ator08_hmg124h_4.JPG.dat	919.3	35.3	1.1	0.04	0.010	0.043	1.9E-05
YER181C	YER181C	UWOPS87_Ator08_hmg124h_4.JPG.dat	859.5	17.6	1.0	0.03	-0.022	0.028	6.0E-06
YER182W	YER182W	UWOPS87_Ator08_hmg124h_4.JPG.dat	795.5	33.1	0.9	0.04	-0.066	0.040	2.2E-05
YER183C	YER183C	UWOPS87_Ator08_hmg124h_4.JPG.dat	876.8	32.3	1.0	0.01	-0.002	0.011	4.3E-05
YER184C	YER184C	UWOPS87_Ator08_hmg124h_4.JPG.dat	829.5	20.9	0.9	0.03	-0.109	0.028	7.8E-06
YER185W	YER185W	UWOPS87_Ator08_hmg124h_4.JPG.dat	877.0	38.2	1.0	0.05	0.038	0.048	3.1E-05
YER186C	YER186C	UWOPS87_Ator08_hmg124h_4.JPG.dat	838.0	67.8	0.9	0.07	-0.051	0.072	1.4E-04
YER187W	YER187W	UWOPS87_Ator08_hmg124h_4.JPG.dat	944.5	39.6	1.0	0.05	0.000	0.046	2.6E-05
YER188W	YER188W	UWOPS87_Ator08_hmg124h_4.JPG.dat	843.8	55.4	1.0	0.06	-0.051	0.057	5.2E-05
YFL006W	YFL006W	UWOPS87_Ator08_hmg124h_4.JPG.dat	943.3	20.6	1.1	0.01	0.053	0.008	1.8E-05
YFL011W	YFL011W	UWOPS87_Ator08_hmg124h_4.JPG.dat	860.3	30.4	0.9	0.01	-0.102	0.011	4.4E-05
YFL013W-A	YFL013W-A	UWOPS87_Ator08_hmg124h_4.JPG.dat	872.0	15.8	1.0	0.01	0.003	0.006	1.1E-05
YFL014W	YFL014W	UWOPS87_Ator08_hmg124h_4.JPG.dat	757.0	29.7	0.9	0.03	-0.046	0.032	1.3E-05
YFL015C	YFL015C	UWOPS87_Ator08_hmg124h_4.JPG.dat	932.0	44.4	1.1	0.06	0.048	0.058	3.8E-05
YFL018C	YFL018C	UWOPS87_Ator08_hmg124h_4.JPG.dat	595.8	405.7	0.9	0.11	0.920	0.111	4.8E-03
YFL019C	YFL019C	UWOPS87_Ator08_hmg124h_4.JPG.dat	772.5	48.0	0.9	0.05	0.004	0.051	5.8E-05
YFL020C	YFL020C	UWOPS87_Ator08_hmg124h_4.JPG.dat	796.8	39.3	0.9	0.01	-0.037	0.012	6.3E-05
YFL021W	YFL021W	UWOPS87_Ator08_hmg124h_4.JPG.dat	876.3	65.7	0.9	0.07	-0.003	0.068	1.0E-04
YFL023W	YFL023W	UWOPS87_Ator08_hmg124h_4.JPG.dat	1016.3	102.9	1.1	0.11	0.314	0.112	3.0E-04
YFL025C	YFL025C	UWOPS87_Ator08_hmg124h_4.JPG.dat	843.0	58.5	1.0	0.07	0.139	0.074	1.1E-04
YFL026W	YFL026W	UWOPS87_Ator08_hmg124h_4.JPG.dat	788.5	81.8	0.9	0.09	0.116	0.088	2.7E-04
YFL027C	YFL027C	UWOPS87_Ator08_hmg124h_4.JPG.dat	866.5	28.5	1.0	0.03	0.013	0.029	7.1E-06
YFL028C	YFL028C	UWOPS87_Ator08_hmg124h_4.JPG.dat	781.5	41.2	0.9	0.05	0.003	0.055	5.8E-05
YFL030W	YFL030W	UWOPS87_Ator08_hmg124h_4.JPG.dat	832.8	21.6	1.0	0.02	-0.027	0.018	1.5E-06
YFL031W	YFL031W	UWOPS87_Ator08_hmg124h_4.JPG.dat	706.5	38.1	0.9	0.05	-0.074	0.052	6.2E-05
YFL032W	YFL032W	UWOPS87_Ator08_hmg124h_4.JPG.dat	340.0	246.8	0.4	0.29	-0.006	0.293	7.0E-02
YFL034C-A	YFL034C-A	UWOPS87_Ator08_hmg124h_4.JPG.dat	861.8	36.7	1.0	0.04	0.028	0.038	1.7E-05
YFL034W	YFL034W	UWOPS87_Ator08_hmg124h_4.JPG.dat	829.3	134.7	0.9	0.04	0.002	0.038	6.0E-04
YFL035C-B	YFL035C-B	UWOPS87_Ator08_hmg124h_4.JPG.dat	955.3	26.9	1.0	0.03	-0.001	0.032	8.0E-06

YFL036W	YFL036W	UWOPS87_Ator08_hmg124h_4.JPG.dat	922.0	33.5	1.0	0.01	-0.017	0.014	7.3E-05
YFL040W	YFL040W	UWOPS87_Ator08_hmg124h_4.JPG.dat	844.5	62.3	1.0	0.06	0.019	0.064	7.0E-05
YFL041W	YFL041W	UWOPS87_Ator08_hmg124h_4.JPG.dat	871.5	36.3	1.0	0.05	0.021	0.045	2.6E-05
YFL042C	YFL042C	UWOPS87_Ator08_hmg124h_4.JPG.dat	873.8	21.4	1.0	0.02	-0.044	0.023	3.4E-06
YFL043C	YFL043C	UWOPS87_Ator08_hmg124h_4.JPG.dat	770.0	38.3	0.9	0.04	0.052	0.043	3.0E-05
YFL044C	YFL044C	UWOPS87_Ator08_hmg124h_4.JPG.dat	787.5	58.5	1.0	0.01	0.068	0.012	5.3E-05
YFL046W	YFL046W	UWOPS87_Ator08_hmg124h_4.JPG.dat	676.5	16.3	0.8	0.01	-0.079	0.006	1.9E-05
YFL047W	YFL047W	UWOPS87_Ator08_hmg124h_4.JPG.dat	819.5	40.2	1.0	0.05	0.076	0.052	4.2E-05
YFL048C	YFL048C	UWOPS87_Ator08_hmg124h_4.JPG.dat	917.5	34.2	1.1	0.04	0.000	0.041	1.6E-05
YFL049W	YFL049W	UWOPS87_Ator08_hmg124h_4.JPG.dat	824.3	52.3	1.0	0.06	-0.039	0.062	6.8E-05
YFL050C	YFL050C	UWOPS87_Ator08_hmg124h_4.JPG.dat	904.0	95.4	1.0	0.11	0.065	0.108	3.3E-04
YFL051C	YFL051C	UWOPS87_Ator08_hmg124h_4.JPG.dat	825.0	59.5	0.9	0.07	-0.037	0.069	1.2E-04
YFL052W	YFL052W	UWOPS87_Ator08_hmg124h_4.JPG.dat	753.0	23.0	0.9	0.03	0.097	0.027	8.4E-06
YFL053W	YFL053W	UWOPS87_Ator08_hmg124h_4.JPG.dat	640.5	428.6	1.0	0.05	0.062	0.046	7.7E-04
YFL054C	YFL054C	UWOPS87_Ator08_hmg124h_4.JPG.dat	657.5	438.6	1.0	0.02	-0.063	0.021	1.5E-04
YFL055W	YFL055W	UWOPS87_Ator08_hmg124h_4.JPG.dat	898.0	48.2	1.0	0.06	-0.020	0.055	4.3E-05
YFL056C	YFL056C	UWOPS87_Ator08_hmg124h_4.JPG.dat	842.5	66.3	1.0	0.07	-0.033	0.074	1.3E-04
YFR006W	YFR006W	UWOPS87_Ator08_hmg124h_4.JPG.dat	902.0	87.2	1.0	0.10	0.071	0.098	2.3E-04
YFR007W	YFR007W	UWOPS87_Ator08_hmg124h_4.JPG.dat	827.3	55.7	1.0	0.06	-0.080	0.064	8.4E-05
YFR008W	YFR008W	UWOPS87_Ator08_hmg124h_4.JPG.dat	1007.8	26.1	1.2	0.03	0.103	0.031	5.1E-06
YFR009W	YFR009W	UWOPS87_Ator08_hmg124h_4.JPG.dat	598.5	399.3	1.0	0.01	0.102	0.015	7.8E-05
YFR010W	YFR010W	UWOPS87_Ator08_hmg124h_4.JPG.dat	792.3	532.0	1.2	0.09	0.277	0.089	1.8E-03
YFR011C	YFR011C	UWOPS87_Ator08_hmg124h_4.JPG.dat	919.5	69.3	1.0	0.01	0.046	0.013	6.3E-05
YOR202W	YOR202W	UWOPS87_Ator08_hmg124h_4.JPG.dat	888.5	114.5	1.0	0.08	0.002	0.085	4.1E-294
1	1	UWOPS87_Ator08_hmg124h_5.JPG.dat	783.3	136.9	1.0	0.14	0.036	0.138	7.6E-04
2	2	UWOPS87_Ator08_hmg124h_5.JPG.dat	730.5	106.4	0.9	0.12	-0.035	0.119	5.5E-04
3	3	UWOPS87_Ator08_hmg124h_5.JPG.dat	746.8	22.0	1.0	0.03	0.000	0.029	7.2E-06
4	4	UWOPS87_Ator08_hmg124h_5.JPG.dat	702.3	55.0	1.0	0.01	0.015	0.010	3.6E-05
YFR012W	YFR012W	UWOPS87_Ator08_hmg124h_5.JPG.dat	825.0	44.7	1.1	0.06	-0.048	0.058	4.2E-05
YFR013W	YFR013W	UWOPS87_Ator08_hmg124h_5.JPG.dat	711.8	56.0	1.0	0.02	0.155	0.015	8.4E-05
YFR014C	YFR014C	UWOPS87_Ator08_hmg124h_5.JPG.dat	777.8	66.7	1.0	0.09	0.067	0.087	1.6E-04
YFR015C	YFR015C	UWOPS87_Ator08_hmg124h_5.JPG.dat	643.8	37.2	0.9	0.01	-0.169	0.013	7.7E-05
YFR016C	YFR016C	UWOPS87_Ator08_hmg124h_5.JPG.dat	766.3	70.4	1.0	0.10	0.012	0.098	2.3E-04
YFR017C	YFR017C	UWOPS87_Ator08_hmg124h_5.JPG.dat	790.5	92.6	1.0	0.11	0.021	0.113	3.4E-04
YFR018C	YFR018C	UWOPS87_Ator08_hmg124h_5.JPG.dat	887.0	54.6	1.1	0.06	-0.013	0.060	4.2E-05
YFR019W	YFR019W	UWOPS87_Ator08_hmg124h_5.JPG.dat	696.8	68.7	0.9	0.09	-0.054	0.086	2.5E-04
YFR020W	YFR020W	UWOPS87_Ator08_hmg124h_5.JPG.dat	744.5	68.5	1.0	0.09	-0.109	0.093	2.5E-04
YFR021W	YFR021W	UWOPS87_Ator08_hmg124h_5.JPG.dat	778.5	35.1	1.0	0.04	-0.001	0.038	1.6E-05
YFR022W	YFR022W	UWOPS87_Ator08_hmg124h_5.JPG.dat	923.5	104.5	1.2	0.14	0.142	0.143	4.6E-04
YFR023W	YFR023W	UWOPS87_Ator08_hmg124h_5.JPG.dat	753.3	61.7	1.0	0.07	0.020	0.075	1.3E-04
YFR024C	YFR024C	UWOPS87_Ator08_hmg124h_5.JPG.dat	756.0	96.7	0.9	0.11	-0.005	0.113	4.7E-04
YFR024C-A	YFR024C-A	UWOPS87_Ator08_hmg124h_5.JPG.dat	759.5	93.6	0.9	0.12	-0.024	0.124	6.1E-04
YFR025C	YFR025C	UWOPS87_Ator08_hmg124h_5.JPG.dat	0.0	0.0	0.0	0.00	0.000	0.000	
YFR026C	YFR026C	UWOPS87_Ator08_hmg124h_5.JPG.dat	884.8	94.0	1.2	0.02	0.089	0.023	1.3E-04
YFR030W	YFR030W	UWOPS87_Ator08_hmg124h_5.JPG.dat	0.0	0.0	0.0	0.00	0.000	0.000	
YFR031C-A	YFR031C-A	UWOPS87_Ator08_hmg124h_5.JPG.dat	777.5	79.5	0.9	0.10	0.036	0.100	3.5E-04
YFR032C	YFR032C	UWOPS87_Ator08_hmg124h_5.JPG.dat	709.0	31.0	0.9	0.01	-0.089	0.007	2.4E-05
YFR032C-A	YFR032C-A	UWOPS87_Ator08_hmg124h_5.JPG.dat	902.0	32.4	1.1	0.03	0.115	0.030	5.6E-06
YFR033C	YFR033C	UWOPS87_Ator08_hmg124h_5.JPG.dat	748.3	60.7	0.9	0.08	-0.027	0.078	1.7E-04
YFR034C	YFR034C	UWOPS87_Ator08_hmg124h_5.JPG.dat	952.3	123.5	1.2	0.16	0.090	0.155	5.5E-04
YFR035C	YFR035C	UWOPS87_Ator08_hmg124h_5.JPG.dat	727.0	51.0	1.0	0.08	0.021	0.078	1.4E-04
YFR038W	YFR038W	UWOPS87_Ator08_hmg124h_5.JPG.dat	842.3	37.9	1.1	0.05	-0.128	0.051	2.9E-05
YFR039C	YFR039C	UWOPS87_Ator08_hmg124h_5.JPG.dat	844.8	73.3	1.0	0.03	-0.086	0.030	3.0E-04
YFR040W	YFR040W	UWOPS87_Ator08_hmg124h_5.JPG.dat	956.0	24.2	1.1	0.03	0.232	0.033	6.3E-06
YFR041C	YFR041C	UWOPS87_Ator08_hmg124h_5.JPG.dat	771.8	48.1	1.0	0.06	-0.053	0.057	5.7E-05
YFR043C	YFR043C	UWOPS87_Ator08_hmg124h_5.JPG.dat	0.0	0.0	0.0	0.00	0.000	0.000	
YFR044C	YFR044C	UWOPS87_Ator08_hmg124h_5.JPG.dat	848.3	27.2	1.0	0.00	-0.006	0.001	3.9E-07
YFR045W	YFR045W	UWOPS87_Ator08_hmg124h_5.JPG.dat	0.0	0.0	0.0	0.00	0.000	0.000	
YFR046C	YFR046C	UWOPS87_Ator08_hmg124h_5.JPG.dat	726.5	54.8	0.9	0.06	-0.003	0.061	8.1E-05
YFR047C	YFR047C	UWOPS87_Ator08_hmg124h_5.JPG.dat	648.0	54.1	0.9	0.07	-0.046	0.072	1.6E-04
YFR048W	YFR048W	UWOPS87_Ator08_hmg124h_5.JPG.dat	536.0	40.7	0.7	0.06	-0.154	0.057	1.2E-04
YFR049W	YFR049W	UWOPS87_Ator08_hmg124h_5.JPG.dat	875.5	47.0	1.2	0.06	-0.006	0.064	4.5E-05
YFR053C	YFR053C	UWOPS87_Ator08_hmg124h_5.JPG.dat	746.5	143.0	0.9	0.17	-0.052	0.172	1.6E-03
YFR054C	YFR054C	UWOPS87_Ator08_hmg124h_5.JPG.dat	774.8	93.2	1.0	0.12	-0.036	0.120	5.3E-04
YFR055W	YFR055W	UWOPS87_Ator08_hmg124h_5.JPG.dat	764.8	47.7	0.9	0.04	-0.049	0.042	2.7E-05
YFR056C	YFR056C	UWOPS87_Ator08_hmg124h_5.JPG.dat	814.0	136.7	1.0	0.16	0.049	0.159	1.1E-03
YFR057W	YFR057W	UWOPS87_Ator08_hmg124h_5.JPG.dat	860.8	68.1	1.1	0.07	0.017	0.068	7.5E-05
YGL002W	YGL002W	UWOPS87_Ator08_hmg124h_5.JPG.dat	990.8	50.1	1.1	0.03	0.032	0.026	1.8E-04
YGL004C	YGL004C	UWOPS87_Ator08_hmg124h_5.JPG.dat	806.5	37.9	1.0	0.05	0.043	0.047	2.9E-05
YGL005C	YGL005C	UWOPS87_Ator08_hmg124h_5.JPG.dat	841.0	24.4	1.1	0.01	-0.007	0.012	3.7E-07

YGL006W	YGL006W	UWOPS87_Ator08_hmg124h_5.JPG.dat	720.8	72.9	0.9	0.09	-0.038	0.091	2.4E-04
YGL007W	YGL007W	UWOPS87_Ator08_hmg124h_5.JPG.dat	625.5	308.2	0.7	0.05	-0.098	0.049	1.9E-03
YGL009C	YGL009C	UWOPS87_Ator08_hmg124h_5.JPG.dat	0.0	0.0	0.0	0.00	0.000	0.000	
YGL010W	YGL010W	UWOPS87_Ator08_hmg124h_5.JPG.dat	817.8	17.2	1.0	0.02	0.011	0.022	2.6E-06
YGL013C	YGL013C	UWOPS87_Ator08_hmg124h_5.JPG.dat	731.5	51.4	0.9	0.06	-0.073	0.064	9.2E-05
YGL014W	YGL014W	UWOPS87_Ator08_hmg124h_5.JPG.dat	932.0	42.5	1.1	0.06	0.082	0.057	3.7E-05
YGL015C	YGL015C	UWOPS87_Ator08_hmg124h_5.JPG.dat	769.5	55.6	1.0	0.07	0.036	0.070	1.0E-04
YGL016W	YGL016W	UWOPS87_Ator08_hmg124h_5.JPG.dat	686.5	95.2	0.9	0.01	0.021	0.014	8.1E-05
YGL017W	YGL017W	UWOPS87_Ator08_hmg124h_5.JPG.dat	890.0	43.1	1.1	0.06	-0.002	0.056	3.8E-05
YGL019W	YGL019W	UWOPS87_Ator08_hmg124h_5.JPG.dat	934.0	64.8	1.2	0.08	0.126	0.084	1.0E-04
YGL020C	YGL020C	UWOPS87_Ator08_hmg124h_5.JPG.dat	782.8	86.0	1.0	0.11	0.019	0.114	3.8E-04
YGL021W	YGL021W	UWOPS87_Ator08_hmg124h_5.JPG.dat	823.8	18.7	1.1	0.03	0.028	0.027	4.2E-06
YGL023C	YGL023C	UWOPS87_Ator08_hmg124h_5.JPG.dat	761.0	72.6	1.0	0.03	-0.019	0.027	2.6E-04
YGL024W	YGL024W	UWOPS87_Ator08_hmg124h_5.JPG.dat	846.5	32.0	1.1	0.03	0.105	0.026	3.8E-06
YGL026C	YGL026C	UWOPS87_Ator08_hmg124h_5.JPG.dat	0.0	0.0	0.0	0.00	0.000	0.000	
YGL027C	YGL027C	UWOPS87_Ator08_hmg124h_5.JPG.dat	829.3	14.6	1.0	0.02	-0.049	0.019	1.7E-06
YGL028C	YGL028C	UWOPS87_Ator08_hmg124h_5.JPG.dat	835.8	49.5	1.0	0.02	0.091	0.019	1.2E-04
YGL031C	YGL031C	UWOPS87_Ator08_hmg124h_5.JPG.dat	907.0	66.3	1.1	0.08	-0.008	0.077	9.1E-05
YGL032C	YGL032C	UWOPS87_Ator08_hmg124h_5.JPG.dat	943.8	51.9	1.2	0.01	0.103	0.012	3.2E-05
YGL034C	YGL034C	UWOPS87_Ator08_hmg124h_5.JPG.dat	804.5	72.2	1.0	0.09	-0.089	0.088	1.9E-04
YGL035C	YGL035C	UWOPS87_Ator08_hmg124h_5.JPG.dat	815.3	121.0	1.0	0.15	0.078	0.149	8.1E-04
YGL036W	YGL036W	UWOPS87_Ator08_hmg124h_5.JPG.dat	730.0	38.9	1.0	0.06	-0.037	0.064	8.4E-05
YGL039W	YGL039W	UWOPS87_Ator08_hmg124h_5.JPG.dat	701.3	20.6	0.9	0.03	-0.074	0.028	8.0E-06
YGL041C	YGL041C	UWOPS87_Ator08_hmg124h_5.JPG.dat	900.0	46.1	1.1	0.07	0.104	0.073	7.3E-05
YGL043W	YGL043W	UWOPS87_Ator08_hmg124h_5.JPG.dat	723.0	107.1	1.0	0.03	0.099	0.026	2.2E-04
YGL045W	YGL045W	UWOPS87_Ator08_hmg124h_5.JPG.dat	829.3	56.3	1.1	0.10	0.090	0.100	2.3E-04
YGL046W	YGL046W	UWOPS87_Ator08_hmg124h_5.JPG.dat	863.8	40.3	1.1	0.08	0.020	0.083	1.1E-04
YGL050W	YGL050W	UWOPS87_Ator08_hmg124h_5.JPG.dat	799.8	77.9	1.0	0.02	0.054	0.021	1.4E-04
YGL051W	YGL051W	UWOPS87_Ator08_hmg124h_5.JPG.dat	766.3	135.9	0.9	0.05	0.010	0.051	9.9E-04
YGL053W	YGL053W	UWOPS87_Ator08_hmg124h_5.JPG.dat	797.3	43.3	1.1	0.05	-0.018	0.047	2.3E-05
YGL054C	YGL054C	UWOPS87_Ator08_hmg124h_5.JPG.dat	696.0	62.1	0.9	0.09	-0.007	0.086	2.2E-04
YGL056C	YGL056C	UWOPS87_Ator08_hmg124h_5.JPG.dat	739.8	112.0	1.0	0.14	-0.027	0.144	7.8E-04
YGL057C	YGL057C	UWOPS87_Ator08_hmg124h_5.JPG.dat	738.8	125.0	1.0	0.16	0.057	0.160	1.0E-03
YGL058W	YGL058W	UWOPS87_Ator08_hmg124h_5.JPG.dat	671.8	151.2	0.9	0.17	-0.073	0.171	1.9E-03
YGL059W	YGL059W	UWOPS87_Ator08_hmg124h_5.JPG.dat	706.0	83.8	0.9	0.11	-0.040	0.109	4.9E-04
YGL060W	YGL060W	UWOPS87_Ator08_hmg124h_5.JPG.dat	785.8	20.1	1.1	0.04	-0.044	0.036	1.0E-05
YGL062W	YGL062W	UWOPS87_Ator08_hmg124h_5.JPG.dat	762.0	60.8	1.0	0.08	0.109	0.080	1.4E-04
YGL066W	YGL066W	UWOPS87_Ator08_hmg124h_5.JPG.dat	702.0	72.2	0.9	0.10	-0.093	0.099	3.3E-04
YGL067W	YGL067W	UWOPS87_Ator08_hmg124h_5.JPG.dat	740.5	83.4	1.0	0.10	-0.020	0.103	3.3E-04
YGL077C	YGL077C	UWOPS87_Ator08_hmg124h_5.JPG.dat	768.3	41.8	1.0	0.06	-0.068	0.064	6.4E-05
YGL078C	YGL078C	UWOPS87_Ator08_hmg124h_5.JPG.dat	731.0	78.1	1.0	0.09	0.116	0.094	2.4E-04
YGL079W	YGL079W	UWOPS87_Ator08_hmg124h_5.JPG.dat	793.3	10.7	1.0	0.01	0.078	0.013	5.1E-07
YGL080W	YGL080W	UWOPS87_Ator08_hmg124h_5.JPG.dat	794.0	48.6	1.0	0.06	0.051	0.062	6.1E-05
YGL081W	YGL081W	UWOPS87_Ator08_hmg124h_5.JPG.dat	813.3	32.9	1.0	0.01	-0.108	0.008	1.7E-05
YGL082W	YGL082W	UWOPS87_Ator08_hmg124h_5.JPG.dat	769.3	19.0	1.0	0.01	-0.120	0.011	4.0E-05
YGL083W	YGL083W	UWOPS87_Ator08_hmg124h_5.JPG.dat	846.0	26.8	1.1	0.01	0.072	0.007	1.4E-05
YGL084C	YGL084C	UWOPS87_Ator08_hmg124h_5.JPG.dat	749.3	68.8	1.0	0.10	0.042	0.103	3.0E-04
YGL085W	YGL085W	UWOPS87_Ator08_hmg124h_5.JPG.dat	823.8	24.0	1.0	0.03	-0.058	0.029	6.2E-06
YGL086W	YGL086W	UWOPS87_Ator08_hmg124h_5.JPG.dat	820.5	28.4	1.0	0.01	-0.006	0.006	1.4E-05
YGL087C	YGL087C	UWOPS87_Ator08_hmg124h_5.JPG.dat	927.3	42.0	1.1	0.02	-0.008	0.017	7.5E-05
YGL089C	YGL089C	UWOPS87_Ator08_hmg124h_5.JPG.dat	998.5	64.6	1.2	0.06	0.153	0.061	3.4E-05
YGL090W	YGL090W	UWOPS87_Ator08_hmg124h_5.JPG.dat	834.3	55.2	1.0	0.06	-0.011	0.055	4.3E-05
YGL101W	YGL101W	UWOPS87_Ator08_hmg124h_5.JPG.dat	825.5	50.4	1.0	0.06	0.001	0.063	7.0E-05
YGL104C	YGL104C	UWOPS87_Ator08_hmg124h_5.JPG.dat	870.8	23.9	1.0	0.01	0.041	0.008	1.9E-05
YGL105W	YGL105W	UWOPS87_Ator08_hmg124h_5.JPG.dat	0.0	0.0	0.0	0.00	0.000	0.000	
YGL108C	YGL108C	UWOPS87_Ator08_hmg124h_5.JPG.dat	821.0	43.7	1.0	0.05	-0.029	0.050	3.2E-05
YGL109W	YGL109W	UWOPS87_Ator08_hmg124h_5.JPG.dat	767.8	40.6	1.0	0.01	-0.100	0.015	7.7E-05
YGL110C	YGL110C	UWOPS87_Ator08_hmg124h_5.JPG.dat	698.5	54.4	1.0	0.07	0.076	0.065	8.2E-05
YGL114W	YGL114W	UWOPS87_Ator08_hmg124h_5.JPG.dat	865.8	36.7	1.1	0.05	0.090	0.051	2.4E-05
YGL115W	YGL115W	UWOPS87_Ator08_hmg124h_5.JPG.dat	856.8	624.6	1.1	0.77	0.395	0.770	6.8E-02
YGL117W	YGL117W	UWOPS87_Ator08_hmg124h_5.JPG.dat	884.8	36.1	1.1	0.03	0.046	0.033	7.6E-06
YGL118C	YGL118C	UWOPS87_Ator08_hmg124h_5.JPG.dat	893.8	68.0	1.1	0.10	0.007	0.096	1.8E-04
YGL121C	YGL121C	UWOPS87_Ator08_hmg124h_5.JPG.dat	896.0	69.0	1.1	0.03	0.051	0.026	1.7E-04
YGL124C	YGL124C	UWOPS87_Ator08_hmg124h_5.JPG.dat	844.3	24.4	1.0	0.03	-0.059	0.034	9.2E-06
YGL125W	YGL125W	UWOPS87_Ator08_hmg124h_5.JPG.dat	813.5	50.4	1.0	0.01	-0.138	0.011	4.2E-05
YGL126W	YGL126W	UWOPS87_Ator08_hmg124h_5.JPG.dat	895.5	63.9	1.1	0.08	0.025	0.083	1.2E-04
YGL127C	YGL127C	UWOPS87_Ator08_hmg124h_5.JPG.dat	356.8	413.0	0.5	0.54	0.161	0.544	1.8E-01
YGL131C	YGL131C	UWOPS87_Ator08_hmg124h_5.JPG.dat	711.0	40.7	0.9	0.06	0.020	0.056	5.7E-05
YGL132W	YGL132W	UWOPS87_Ator08_hmg124h_5.JPG.dat	747.8	46.9	1.0	0.03	-0.032	0.027	2.4E-04
YGL133W	YGL133W	UWOPS87_Ator08_hmg124h_5.JPG.dat	679.0	36.3	0.9	0.03	-0.103	0.028	8.5E-06

YGL136C	YGL136C	UWOPS87_Ator08_hmg124h_5.JPG.dat	806.3	47.9	1.0	0.05	-0.009	0.049	3.2E-05
YGL138C	YGL138C	UWOPS87_Ator08_hmg124h_5.JPG.dat	837.8	47.2	1.0	0.04	-0.048	0.040	1.8E-05
YGL139W	YGL139W	UWOPS87_Ator08_hmg124h_5.JPG.dat	854.8	79.6	1.0	0.09	-0.045	0.087	1.6E-04
YGL140C	YGL140C	UWOPS87_Ator08_hmg124h_5.JPG.dat	895.8	96.5	1.1	0.03	0.060	0.028	2.1E-04
YGL141W	YGL141W	UWOPS87_Ator08_hmg124h_5.JPG.dat	772.0	107.1	0.9	0.15	0.054	0.150	1.1E-03
YGL144C	YGL144C	UWOPS87_Ator08_hmg124h_5.JPG.dat	730.0	47.1	0.9	0.08	0.007	0.078	1.9E-04
YGL146C	YGL146C	UWOPS87_Ator08_hmg124h_5.JPG.dat	893.5	12.0	1.1	0.02	-0.020	0.019	1.4E-06
YGL147C	YGL147C	UWOPS87_Ator08_hmg124h_5.JPG.dat	872.0	66.9	1.1	0.08	0.227	0.081	9.9E-05
YGL148W	YGL148W	UWOPS87_Ator08_hmg124h_5.JPG.dat	672.8	90.6	0.9	0.10	-0.099	0.103	4.3E-04
YGL149W	YGL149W	UWOPS87_Ator08_hmg124h_5.JPG.dat	667.3	24.8	0.9	0.04	-0.057	0.036	1.5E-05
YGL151W	YGL151W	UWOPS87_Ator08_hmg124h_5.JPG.dat	655.0	104.5	0.8	0.02	-0.017	0.024	3.0E-04
YGL152C	YGL152C	UWOPS87_Ator08_hmg124h_5.JPG.dat	840.0	47.4	1.1	0.06	-0.042	0.059	4.9E-05
YGL153W	YGL153W	UWOPS87_Ator08_hmg124h_5.JPG.dat	827.5	45.7	1.0	0.05	0.009	0.049	3.3E-05
YGL154C	YGL154C	UWOPS87_Ator08_hmg124h_5.JPG.dat	817.0	27.5	1.0	0.04	0.075	0.041	1.8E-05
YGL156W	YGL156W	UWOPS87_Ator08_hmg124h_5.JPG.dat	894.0	109.4	1.1	0.13	0.072	0.131	4.6E-04
YGL157W	YGL157W	UWOPS87_Ator08_hmg124h_5.JPG.dat	821.5	51.3	1.0	0.07	-0.081	0.069	8.5E-05
YGL158W	YGL158W	UWOPS87_Ator08_hmg124h_5.JPG.dat	698.8	113.5	0.9	0.14	-0.088	0.137	1.0E-03
YGL159W	YGL159W	UWOPS87_Ator08_hmg124h_5.JPG.dat	732.0	41.5	0.9	0.05	-0.072	0.049	4.2E-05
YGL160W	YGL160W	UWOPS87_Ator08_hmg124h_5.JPG.dat	761.0	45.6	1.0	0.00	0.016	0.004	5.3E-06
YGL161C	YGL161C	UWOPS87_Ator08_hmg124h_5.JPG.dat	693.0	61.4	0.9	0.08	-0.063	0.078	1.8E-04
YGL162W	YGL162W	UWOPS87_Ator08_hmg124h_5.JPG.dat	715.8	42.6	1.0	0.01	-0.006	0.010	3.3E-05
YGL163C	YGL163C	UWOPS87_Ator08_hmg124h_5.JPG.dat	803.0	39.0	1.0	0.01	-0.002	0.015	7.4E-05
YGL164C	YGL164C	UWOPS87_Ator08_hmg124h_5.JPG.dat	917.0	48.3	1.1	0.05	-0.022	0.050	2.5E-05
YGL165C	YGL165C	UWOPS87_Ator08_hmg124h_5.JPG.dat	749.3	69.9	0.9	0.07	-0.079	0.068	1.2E-04
YGL166W	YGL166W	UWOPS87_Ator08_hmg124h_5.JPG.dat	723.8	34.3	0.9	0.02	-0.009	0.019	1.4E-04
YGL167C	YGL167C	UWOPS87_Ator08_hmg124h_5.JPG.dat	746.0	69.7	0.9	0.09	-0.087	0.090	2.6E-04
YGL168W	YGL168W	UWOPS87_Ator08_hmg124h_5.JPG.dat	853.5	66.6	1.1	0.02	0.150	0.022	1.3E-04
YGL170C	YGL170C	UWOPS87_Ator08_hmg124h_5.JPG.dat	775.8	36.1	1.0	0.00	-0.018	0.003	2.9E-06
YGL173C	YGL173C	UWOPS87_Ator08_hmg124h_5.JPG.dat	800.8	42.9	1.0	0.07	0.001	0.066	7.6E-05
YGL174W	YGL174W	UWOPS87_Ator08_hmg124h_5.JPG.dat	0.0	0.0	0.0	0.00	0.000	0.000	
YGL175C	YGL175C	UWOPS87_Ator08_hmg124h_5.JPG.dat	601.3	402.2	1.0	0.05	-0.013	0.049	7.9E-04
YGL176C	YGL176C	UWOPS87_Ator08_hmg124h_5.JPG.dat	670.3	26.6	0.9	0.05	-0.075	0.048	3.9E-05
YGL177W	YGL177W	UWOPS87_Ator08_hmg124h_5.JPG.dat	821.8	99.8	1.1	0.03	0.083	0.027	2.0E-04
YGL179C	YGL179C	UWOPS87_Ator08_hmg124h_5.JPG.dat	853.8	67.7	1.1	0.12	0.141	0.118	3.4E-04
YGL180W	YGL180W	UWOPS87_Ator08_hmg124h_5.JPG.dat	894.3	29.8	1.1	0.05	-0.072	0.054	3.0E-05
YGL181W	YGL181W	UWOPS87_Ator08_hmg124h_5.JPG.dat	915.3	38.0	1.2	0.03	0.092	0.031	4.8E-06
YGL194C	YGL194C	UWOPS87_Ator08_hmg124h_5.JPG.dat	736.5	23.4	1.0	0.03	-0.150	0.032	9.8E-06
YGL195W	YGL195W	UWOPS87_Ator08_hmg124h_5.JPG.dat	659.5	51.2	0.9	0.07	-0.109	0.070	1.3E-04
YGL196W	YGL196W	UWOPS87_Ator08_hmg124h_5.JPG.dat	825.5	130.1	1.1	0.19	0.111	0.190	1.3E-03
YGL197W	YGL197W	UWOPS87_Ator08_hmg124h_5.JPG.dat	769.5	66.0	1.0	0.06	0.087	0.062	5.9E-05
YGL198W	YGL198W	UWOPS87_Ator08_hmg124h_5.JPG.dat	506.3	343.2	0.9	0.07	-0.027	0.074	2.1E-03
YGL199C	YGL199C	UWOPS87_Ator08_hmg124h_5.JPG.dat	675.0	44.7	0.9	0.05	0.043	0.049	5.1E-05
YGL202W	YGL202W	UWOPS87_Ator08_hmg124h_5.JPG.dat	801.3	85.9	1.0	0.12	0.029	0.116	4.0E-04
YGL203C	YGL203C	UWOPS87_Ator08_hmg124h_5.JPG.dat	781.5	18.6	1.0	0.02	0.044	0.019	1.9E-06
YGL205W	YGL205W	UWOPS87_Ator08_hmg124h_5.JPG.dat	870.3	53.9	1.1	0.08	-0.033	0.075	8.0E-05
YGL208W	YGL208W	UWOPS87_Ator08_hmg124h_5.JPG.dat	852.3	125.0	1.1	0.16	-0.008	0.159	8.4E-04
YGL209W	YGL209W	UWOPS87_Ator08_hmg124h_5.JPG.dat	740.8	54.6	1.0	0.08	-0.080	0.075	1.2E-04
YGL210W	YGL210W	UWOPS87_Ator08_hmg124h_5.JPG.dat	665.3	82.2	0.9	0.10	-0.095	0.099	3.9E-04
YGL211W	YGL211W	UWOPS87_Ator08_hmg124h_5.JPG.dat	860.3	52.6	1.1	0.08	0.086	0.079	1.0E-04
YGL212W	YGL212W	UWOPS87_Ator08_hmg124h_5.JPG.dat	762.3	36.5	0.9	0.01	0.000	0.012	5.6E-05
YGL213C	YGL213C	UWOPS87_Ator08_hmg124h_5.JPG.dat	826.5	64.3	1.0	0.02	-0.056	0.016	8.4E-05
YGL214W	YGL214W	UWOPS87_Ator08_hmg124h_5.JPG.dat	880.5	34.5	1.1	0.00	0.067	0.001	5.4E-07
YGL215W	YGL215W	UWOPS87_Ator08_hmg124h_5.JPG.dat	851.3	81.5	1.1	0.11	0.184	0.115	3.3E-04
YGL216W	YGL216W	UWOPS87_Ator08_hmg124h_5.JPG.dat	737.0	37.3	1.0	0.01	0.034	0.007	1.8E-05
YGL217C	YGL217C	UWOPS87_Ator08_hmg124h_5.JPG.dat	799.0	12.8	1.0	0.02	0.007	0.024	3.8E-06
YGL218W	YGL218W	UWOPS87_Ator08_hmg124h_5.JPG.dat	755.5	24.9	1.0	0.04	0.085	0.035	1.3E-05
YGL219C	YGL219C	UWOPS87_Ator08_hmg124h_5.JPG.dat	612.5	408.8	1.0	0.02	0.338	0.023	1.7E-04
YGL221C	YGL221C	UWOPS87_Ator08_hmg124h_5.JPG.dat	824.5	47.3	1.1	0.05	0.054	0.055	3.6E-05
YGL222C	YGL222C	UWOPS87_Ator08_hmg124h_5.JPG.dat	689.3	92.6	0.9	0.11	-0.132	0.107	4.9E-04
YGL224C	YGL224C	UWOPS87_Ator08_hmg124h_5.JPG.dat	854.0	51.4	1.0	0.06	0.029	0.057	4.5E-05
YGL226C-A	YGL226C-A	UWOPS87_Ator08_hmg124h_5.JPG.dat	798.8	110.1	1.0	0.02	0.100	0.024	1.7E-04
YGL226W	YGL226W	UWOPS87_Ator08_hmg124h_5.JPG.dat	816.5	42.9	1.0	0.05	-0.027	0.051	3.5E-05
YGL227W	YGL227W	UWOPS87_Ator08_hmg124h_5.JPG.dat	863.8	56.5	1.1	0.08	0.013	0.076	9.6E-05
YGL228W	YGL228W	UWOPS87_Ator08_hmg124h_5.JPG.dat	743.3	95.9	1.0	0.13	0.090	0.129	6.6E-04
YGL229C	YGL229C	UWOPS87_Ator08_hmg124h_5.JPG.dat	766.5	65.0	1.0	0.09	-0.152	0.094	2.2E-04
YGL230C	YGL230C	UWOPS87_Ator08_hmg124h_5.JPG.dat	722.5	70.0	0.9	0.01	-0.137	0.013	7.2E-05
YGL231C	YGL231C	UWOPS87_Ator08_hmg124h_5.JPG.dat	757.3	58.5	1.0	0.06	-0.097	0.064	8.3E-05
YGL232W	YGL232W	UWOPS87_Ator08_hmg124h_5.JPG.dat	996.8	29.0	1.2	0.05	0.077	0.052	2.3E-05
YGL234W	YGL234W	UWOPS87_Ator08_hmg124h_5.JPG.dat	754.8	70.7	0.9	0.10	-0.076	0.099	3.1E-04
YGL235W	YGL235W	UWOPS87_Ator08_hmg124h_5.JPG.dat	920.8	40.2	1.1	0.05	0.036	0.047	1.9E-05

YGL236C	YGL236C	UWOPS87_Ator08_hmg124h_5.JPG.dat	871.0	26.7	1.0	0.03	-0.048	0.033	9.0E-06
YGL237C	YGL237C	UWOPS87_Ator08_hmg124h_5.JPG.dat	900.3	44.1	1.1	0.05	-0.065	0.046	2.1E-05
YGL241W	YGL241W	UWOPS87_Ator08_hmg124h_5.JPG.dat	794.8	43.8	1.0	0.05	0.000	0.046	2.7E-05
YGL242C	YGL242C	UWOPS87_Ator08_hmg124h_5.JPG.dat	842.5	23.0	1.1	0.01	-0.056	0.007	1.3E-05
YGL243W	YGL243W	UWOPS87_Ator08_hmg124h_5.JPG.dat	888.0	54.1	1.2	0.06	-0.030	0.059	3.3E-05
YGL244W	YGL244W	UWOPS87_Ator08_hmg124h_5.JPG.dat	662.8	49.3	0.9	0.08	0.073	0.076	1.5E-04
YGL248W	YGL248W	UWOPS87_Ator08_hmg124h_5.JPG.dat	790.0	49.4	1.0	0.06	0.025	0.058	4.7E-05
YGL249W	YGL249W	UWOPS87_Ator08_hmg124h_5.JPG.dat	773.8	82.8	1.0	0.10	0.058	0.102	2.9E-04
YGL250W	YGL250W	UWOPS87_Ator08_hmg124h_5.JPG.dat	818.8	20.3	1.0	0.02	-0.087	0.021	2.4E-06
YGL251C	YGL251C	UWOPS87_Ator08_hmg124h_5.JPG.dat	732.3	61.0	0.9	0.09	0.026	0.088	2.2E-04
YGL252C	YGL252C	UWOPS87_Ator08_hmg124h_5.JPG.dat	732.8	54.2	0.9	0.05	-0.069	0.048	3.8E-05
YGL253W	YGL253W	UWOPS87_Ator08_hmg124h_5.JPG.dat	795.0	23.4	1.0	0.05	0.033	0.050	3.6E-05
YGL254W	YGL254W	UWOPS87_Ator08_hmg124h_5.JPG.dat	871.0	98.2	1.1	0.11	0.022	0.113	3.0E-04
YGL255W	YGL255W	UWOPS87_Ator08_hmg124h_5.JPG.dat	686.3	96.4	1.0	0.04	-0.138	0.042	6.3E-04
YGL256W	YGL256W	UWOPS87_Ator08_hmg124h_5.JPG.dat	811.5	65.4	1.1	0.08	0.097	0.075	8.6E-05
YGL257C	YGL257C	UWOPS87_Ator08_hmg124h_5.JPG.dat	721.3	99.6	1.0	0.15	0.057	0.151	8.2E-04
YGL258W	YGL258W	UWOPS87_Ator08_hmg124h_5.JPG.dat	758.3	40.0	1.0	0.05	-0.210	0.047	2.6E-05
YGL259W	YGL259W	UWOPS87_Ator08_hmg124h_5.JPG.dat	847.8	50.2	1.1	0.07	-0.035	0.071	7.5E-05
YGL260W	YGL260W	UWOPS87_Ator08_hmg124h_5.JPG.dat	886.8	103.4	1.1	0.13	0.062	0.132	4.6E-04
YGL261C	YGL261C	UWOPS87_Ator08_hmg124h_5.JPG.dat	883.0	113.4	1.1	0.13	0.070	0.132	5.3E-04
YGL262W	YGL262W	UWOPS87_Ator08_hmg124h_5.JPG.dat	843.5	49.0	1.1	0.06	0.028	0.063	5.7E-05
YGL263W	YGL263W	UWOPS87_Ator08_hmg124h_5.JPG.dat	828.3	43.1	1.0	0.06	0.103	0.055	4.3E-05
YGR001C	YGR001C	UWOPS87_Ator08_hmg124h_5.JPG.dat	802.3	24.3	1.0	0.03	0.004	0.028	6.7E-06
YGR003W	YGR003W	UWOPS87_Ator08_hmg124h_5.JPG.dat	822.5	54.8	1.0	0.07	0.015	0.072	9.4E-05
YGR004W	YGR004W	UWOPS87_Ator08_hmg124h_5.JPG.dat	782.5	56.4	1.0	0.07	-0.039	0.067	7.8E-05
YGR007W	YGR007W	UWOPS87_Ator08_hmg124h_5.JPG.dat	650.8	67.0	0.9	0.09	-0.077	0.093	3.3E-04
YGR008C	YGR008C	UWOPS87_Ator08_hmg124h_5.JPG.dat	695.8	50.1	1.0	0.08	-0.046	0.081	1.7E-04
YGR010W	YGR010W	UWOPS87_Ator08_hmg124h_5.JPG.dat	701.3	66.9	0.9	0.08	-0.048	0.077	1.5E-04
YGR011W	YGR011W	UWOPS87_Ator08_hmg124h_5.JPG.dat	895.3	87.9	1.1	0.12	0.017	0.116	3.0E-04
YGR012W	YGR012W	UWOPS87_Ator08_hmg124h_5.JPG.dat	829.0	10.4	1.0	0.02	-0.002	0.022	2.8E-06
YGR014W	YGR014W	UWOPS87_Ator08_hmg124h_5.JPG.dat	938.8	99.0	1.2	0.04	0.065	0.036	3.2E-04
YGR015C	YGR015C	UWOPS87_Ator08_hmg124h_5.JPG.dat	787.3	18.4	1.0	0.02	0.044	0.017	1.6E-06
YGR016W	YGR016W	UWOPS87_Ator08_hmg124h_5.JPG.dat	831.5	39.2	1.0	0.06	0.032	0.055	4.3E-05
YGR017W	YGR017W	UWOPS87_Ator08_hmg124h_5.JPG.dat	841.0	96.4	1.0	0.12	-0.061	0.115	3.8E-04
YGR018C	YGR018C	UWOPS87_Ator08_hmg124h_5.JPG.dat	782.5	97.3	1.0	0.12	0.024	0.124	5.4E-04
YGR019W	YGR019W	UWOPS87_Ator08_hmg124h_5.JPG.dat	816.8	69.3	1.1	0.08	-0.015	0.083	1.3E-04
YGR021W	YGR021W	UWOPS87_Ator08_hmg124h_5.JPG.dat	597.3	408.9	1.0	0.16	0.017	0.157	7.4E-03
YGR022C	YGR022C	UWOPS87_Ator08_hmg124h_5.JPG.dat	848.8	95.6	1.1	0.13	0.009	0.125	4.2E-04
YGR023W	YGR023W	UWOPS87_Ator08_hmg124h_5.JPG.dat	747.5	63.2	1.0	0.09	-0.030	0.085	1.8E-04
YGR025W	YGR025W	UWOPS87_Ator08_hmg124h_5.JPG.dat	789.8	119.1	1.0	0.15	0.110	0.149	8.9E-04
YGR026W	YGR026W	UWOPS87_Ator08_hmg124h_5.JPG.dat	837.5	45.9	1.1	0.06	0.027	0.058	4.5E-05
YGR027C	YGR027C	UWOPS87_Ator08_hmg124h_5.JPG.dat	793.5	22.2	1.0	0.03	-0.068	0.029	7.9E-06
YGR028W	YGR028W	UWOPS87_Ator08_hmg124h_5.JPG.dat	852.5	40.1	1.1	0.02	-0.039	0.019	1.1E-04
YGR031W	YGR031W	UWOPS87_Ator08_hmg124h_5.JPG.dat	747.0	30.6	1.0	0.04	0.068	0.040	1.9E-05
YGR032W	YGR032W	UWOPS87_Ator08_hmg124h_5.JPG.dat	747.3	26.1	1.0	0.03	-0.016	0.032	1.0E-05
YGR033C	YGR033C	UWOPS87_Ator08_hmg124h_5.JPG.dat	818.0	36.0	1.1	0.05	0.039	0.045	2.0E-05
YGR034W	YGR034W	UWOPS87_Ator08_hmg124h_5.JPG.dat	789.5	53.6	1.1	0.08	-0.014	0.077	1.0E-04
YGR035C	YGR035C	UWOPS87_Ator08_hmg124h_5.JPG.dat	669.5	447.1	1.2	0.04	0.011	0.040	3.8E-04
YGR037C	YGR037C	UWOPS87_Ator08_hmg124h_5.JPG.dat	626.0	97.6	0.8	0.12	0.016	0.119	9.0E-04
YGR038W	YGR038W	UWOPS87_Ator08_hmg124h_5.JPG.dat	828.0	74.4	1.1	0.08	0.076	0.080	1.1E-04
YGR039W	YGR039W	UWOPS87_Ator08_hmg124h_5.JPG.dat	872.5	58.4	1.1	0.02	0.036	0.023	1.6E-04
YGR040W	YGR040W	UWOPS87_Ator08_hmg124h_5.JPG.dat	786.0	30.0	1.0	0.00	0.036	0.004	5.0E-06
YGR041W	YGR041W	UWOPS87_Ator08_hmg124h_5.JPG.dat	699.8	76.4	0.9	0.11	0.029	0.110	5.0E-04
YGR042W	YGR042W	UWOPS87_Ator08_hmg124h_5.JPG.dat	680.0	43.6	0.9	0.01	-0.057	0.012	5.5E-05
YGR043C	YGR043C	UWOPS87_Ator08_hmg124h_5.JPG.dat	639.3	64.5	0.9	0.03	-0.104	0.028	3.4E-04
YGR044C	YGR044C	UWOPS87_Ator08_hmg124h_5.JPG.dat	713.0	19.1	0.9	0.00	-0.010	0.003	3.1E-06
YGR045C	YGR045C	UWOPS87_Ator08_hmg124h_5.JPG.dat	809.5	115.8	1.0	0.04	-0.041	0.043	6.5E-04
YGR049W	YGR049W	UWOPS87_Ator08_hmg124h_5.JPG.dat	950.0	80.0	1.2	0.11	0.087	0.107	2.0E-04
YGR050C	YGR050C	UWOPS87_Ator08_hmg124h_5.JPG.dat	685.5	76.8	0.9	0.09	-0.045	0.089	2.9E-04
YGR051C	YGR051C	UWOPS87_Ator08_hmg124h_5.JPG.dat	966.8	123.0	1.2	0.14	0.021	0.136	4.2E-04
YGR052W	YGR052W	UWOPS87_Ator08_hmg124h_5.JPG.dat	791.5	37.2	1.1	0.01	0.128	0.010	2.8E-05
YGR053C	YGR053C	UWOPS87_Ator08_hmg124h_5.JPG.dat	763.0	33.5	1.0	0.05	-0.015	0.052	4.1E-05
YGR054W	YGR054W	UWOPS87_Ator08_hmg124h_5.JPG.dat	896.3	36.2	1.1	0.04	0.041	0.038	1.0E-05
YGR058W	YGR058W	UWOPS87_Ator08_hmg124h_5.JPG.dat	807.3	112.3	1.1	0.03	-0.034	0.030	2.6E-04
YGR059W	YGR059W	UWOPS87_Ator08_hmg124h_5.JPG.dat	665.5	75.2	0.9	0.09	-0.127	0.089	3.1E-04
YGR066C	YGR066C	UWOPS87_Ator08_hmg124h_5.JPG.dat	835.8	106.6	1.1	0.13	-0.006	0.129	4.8E-04
YGR069W	YGR069W	UWOPS87_Ator08_hmg124h_5.JPG.dat	757.8	65.0	1.0	0.08	-0.046	0.081	1.7E-04
YGR070W	YGR070W	UWOPS87_Ator08_hmg124h_5.JPG.dat	786.8	46.8	1.0	0.05	-0.111	0.050	3.7E-05
YGR071C	YGR071C	UWOPS87_Ator08_hmg124h_5.JPG.dat	910.3	87.4	1.1	0.02	-0.072	0.023	1.6E-04
YGR072W	YGR072W	UWOPS87_Ator08_hmg124h_5.JPG.dat	820.8	133.9	1.0	0.17	0.047	0.170	1.2E-03

YGR077C	YGR077C	UWOPS87_Ator08_hmg124h_5.JPG.dat	805.3	39.6	1.0	0.04	-0.002	0.043	2.2E-05
YGR078C	YGR078C	UWOPS87_Ator08_hmg124h_5.JPG.dat	686.0	18.5	0.9	0.01	-0.026	0.008	2.6E-05
YGR079W	YGR079W	UWOPS87_Ator08_hmg124h_5.JPG.dat	752.0	27.1	1.0	0.03	0.070	0.034	1.2E-05
YGR080W	YGR080W	UWOPS87_Ator08_hmg124h_5.JPG.dat	595.5	91.5	0.8	0.03	-0.007	0.033	5.7E-04
YGR081C	YGR081C	UWOPS87_Ator08_hmg124h_5.JPG.dat	713.0	106.8	0.9	0.04	0.024	0.036	4.9E-04
YGR084C	YGR084C	UWOPS87_Ator08_hmg124h_5.JPG.dat	716.3	46.0	0.9	0.06	-0.040	0.060	8.4E-05
YGR085C	YGR085C	UWOPS87_Ator08_hmg124h_5.JPG.dat	792.3	36.8	1.0	0.04	0.056	0.035	1.3E-05
YGR086C	YGR086C	UWOPS87_Ator08_hmg124h_5.JPG.dat	844.0	34.1	1.0	0.06	0.038	0.060	5.2E-05
YGR087C	YGR087C	UWOPS87_Ator08_hmg124h_5.JPG.dat	865.5	48.0	1.0	0.05	-0.027	0.046	2.4E-05
YGR088W	YGR088W	UWOPS87_Ator08_hmg124h_5.JPG.dat	789.8	57.0	1.0	0.03	0.074	0.030	3.0E-04
YGR089W	YGR089W	UWOPS87_Ator08_hmg124h_5.JPG.dat	733.5	53.2	0.9	0.07	0.005	0.067	9.9E-05
YGR092W	YGR092W	UWOPS87_Ator08_hmg124h_5.JPG.dat	538.5	369.3	0.9	0.13	0.114	0.127	6.3E-03
YGR093W	YGR093W	UWOPS87_Ator08_hmg124h_5.JPG.dat	689.8	76.3	1.0	0.03	-0.013	0.033	3.9E-04
YGR096W	YGR096W	UWOPS87_Ator08_hmg124h_5.JPG.dat	703.0	39.3	1.0	0.04	0.032	0.041	2.1E-05
YGR097W	YGR097W	UWOPS87_Ator08_hmg124h_5.JPG.dat	803.8	54.6	1.0	0.08	-0.010	0.083	1.3E-04
YGR100W	YGR100W	UWOPS87_Ator08_hmg124h_5.JPG.dat	793.3	26.7	1.0	0.05	-0.011	0.053	4.1E-05
YGR101W	YGR101W	UWOPS87_Ator08_hmg124h_5.JPG.dat	804.3	41.8	1.0	0.08	-0.069	0.078	1.2E-04
YGR105W	YGR105W	UWOPS87_Ator08_hmg124h_5.JPG.dat	815.3	140.2	1.0	0.15	-0.088	0.155	9.2E-04
YGR106C	YGR106C	UWOPS87_Ator08_hmg124h_5.JPG.dat	724.3	41.4	0.9	0.07	-0.021	0.072	1.3E-04
YGR107W	YGR107W	UWOPS87_Ator08_hmg124h_5.JPG.dat	864.3	29.7	1.1	0.04	0.060	0.042	1.6E-05
YGR108W	YGR108W	UWOPS87_Ator08_hmg124h_5.JPG.dat	811.8	54.2	1.0	0.03	0.018	0.029	2.8E-04
YGR109C	YGR109C	UWOPS87_Ator08_hmg124h_5.JPG.dat	725.3	27.5	1.0	0.06	-0.032	0.058	5.7E-05
YGR110W	YGR110W	UWOPS87_Ator08_hmg124h_5.JPG.dat	825.5	30.3	1.2	0.02	0.096	0.016	6.8E-05
YGR111W	YGR111W	UWOPS87_Ator08_hmg124h_5.JPG.dat	727.8	11.5	1.0	0.03	0.017	0.027	4.7E-06
YGR117C	YGR117C	UWOPS87_Ator08_hmg124h_5.JPG.dat	721.5	52.4	1.0	0.07	-0.057	0.071	1.1E-04
YGR118W	YGR118W	UWOPS87_Ator08_hmg124h_5.JPG.dat	627.3	102.5	0.8	0.13	0.058	0.130	1.2E-03
YGR121C	YGR121C	UWOPS87_Ator08_hmg124h_5.JPG.dat	920.0	22.7	1.1	0.03	0.020	0.033	7.2E-06
YGR123C	YGR123C	UWOPS87_Ator08_hmg124h_5.JPG.dat	782.3	23.9	1.0	0.03	0.022	0.028	7.0E-06
YGR124W	YGR124W	UWOPS87_Ator08_hmg124h_5.JPG.dat	832.0	80.5	1.0	0.02	-0.041	0.018	1.2E-04
YGR125W	YGR125W	UWOPS87_Ator08_hmg124h_5.JPG.dat	918.5	75.9	1.1	0.09	0.014	0.092	1.5E-04
YGR126W	YGR126W	UWOPS87_Ator08_hmg124h_5.JPG.dat	752.3	98.3	0.9	0.13	0.002	0.128	6.9E-04
YGR127W	YGR127W	UWOPS87_Ator08_hmg124h_5.JPG.dat	745.0	60.0	1.0	0.02	0.054	0.018	1.1E-04
YGR129W	YGR129W	UWOPS87_Ator08_hmg124h_5.JPG.dat	717.3	56.4	0.9	0.03	-0.038	0.025	2.5E-04
YGR130C	YGR130C	UWOPS87_Ator08_hmg124h_5.JPG.dat	843.8	42.4	1.1	0.05	0.156	0.051	2.6E-05
YGR131W	YGR131W	UWOPS87_Ator08_hmg124h_5.JPG.dat	687.3	59.8	1.0	0.07	0.006	0.073	1.2E-04
YGR132C	YGR132C	UWOPS87_Ator08_hmg124h_5.JPG.dat	851.8	64.6	1.1	0.07	-0.049	0.069	7.1E-05
YGR133W	YGR133W	UWOPS87_Ator08_hmg124h_5.JPG.dat	812.0	50.7	1.0	0.07	0.053	0.070	9.4E-05
YGR134W	YGR134W	UWOPS87_Ator08_hmg124h_5.JPG.dat	735.3	46.4	0.9	0.04	-0.028	0.045	3.6E-05
YGR135W	YGR135W	UWOPS87_Ator08_hmg124h_5.JPG.dat	792.3	116.6	1.0	0.13	-0.024	0.130	6.7E-04
YGR136W	YGR136W	UWOPS87_Ator08_hmg124h_5.JPG.dat	855.5	14.6	1.0	0.02	0.053	0.024	3.3E-06
YGR137W	YGR137W	UWOPS87_Ator08_hmg124h_5.JPG.dat	0.0	0.0	0.0	0.00	0.000	0.000	
YGR138C	YGR138C	UWOPS87_Ator08_hmg124h_5.JPG.dat	665.0	17.9	0.8	0.02	-0.095	0.020	3.8E-06
YGR139W	YGR139W	UWOPS87_Ator08_hmg124h_5.JPG.dat	776.3	46.2	1.0	0.05	-0.019	0.053	4.5E-05
YGR141W	YGR141W	UWOPS87_Ator08_hmg124h_5.JPG.dat	351.3	405.8	0.5	0.54	-0.007	0.537	1.8E-01
YGR142W	YGR142W	UWOPS87_Ator08_hmg124h_5.JPG.dat	874.0	55.0	1.1	0.07	-0.021	0.067	6.0E-05
YGR143W	YGR143W	UWOPS87_Ator08_hmg124h_5.JPG.dat	666.3	69.9	0.9	0.03	-0.111	0.027	3.3E-04
YGR144W	YGR144W	UWOPS87_Ator08_hmg124h_5.JPG.dat	871.3	65.2	1.1	0.09	-0.120	0.091	1.5E-04
YGR146C	YGR146C	UWOPS87_Ator08_hmg124h_5.JPG.dat	755.0	38.1	0.9	0.05	-0.023	0.047	3.4E-05
YGR148C	YGR148C	UWOPS87_Ator08_hmg124h_5.JPG.dat	865.3	56.3	1.1	0.06	0.131	0.064	5.9E-05
YGR149W	YGR149W	UWOPS87_Ator08_hmg124h_5.JPG.dat	757.5	40.1	1.0	0.01	0.000	0.011	4.0E-05
YGR151C	YGR151C	UWOPS87_Ator08_hmg124h_5.JPG.dat	869.5	90.6	1.2	0.04	0.148	0.040	3.8E-04
YGR152C	YGR152C	UWOPS87_Ator08_hmg124h_5.JPG.dat	704.8	49.7	0.9	0.06	-0.004	0.061	7.9E-05
YGR153W	YGR153W	UWOPS87_Ator08_hmg124h_5.JPG.dat	761.8	49.6	1.0	0.07	0.045	0.065	7.4E-05
YGR154C	YGR154C	UWOPS87_Ator08_hmg124h_5.JPG.dat	721.8	51.6	1.0	0.07	-0.043	0.067	9.5E-05
YGR157W	YGR157W	UWOPS87_Ator08_hmg124h_5.JPG.dat	0.0	0.0	0.0	0.00	0.000	0.000	
YGR161C	YGR161C	UWOPS87_Ator08_hmg124h_5.JPG.dat	893.5	142.5	1.2	0.04	0.122	0.038	3.3E-04
YOR202W	YOR202W	UWOPS87_Ator08_hmg124h_5.JPG.dat	776.9	125.9	1.0	0.12	0.002	0.123	1.9E-247
1	1	UWOPS87_Ator08_hmg124h_6.JPG.dat	933.5	66.4	1.1	0.01	0.040	0.010	2.8E-05
2	2	UWOPS87_Ator08_hmg124h_6.JPG.dat	833.8	54.9	1.0	0.00	-0.010	0.003	3.4E-06
3	3	UWOPS87_Ator08_hmg124h_6.JPG.dat	767.8	101.0	0.9	0.11	0.136	0.111	5.0E-04
4	4	UWOPS87_Ator08_hmg124h_6.JPG.dat	763.0	125.5	0.9	0.12	0.035	0.120	6.9E-04
YGR163W	YGR163W	UWOPS87_Ator08_hmg124h_6.JPG.dat	771.5	23.2	1.0	0.04	0.099	0.037	1.3E-05
YGR164W	YGR164W	UWOPS87_Ator08_hmg124h_6.JPG.dat	741.0	39.1	0.9	0.02	-0.119	0.018	1.2E-04
YGR166W	YGR166W	UWOPS87_Ator08_hmg124h_6.JPG.dat	647.0	46.9	0.8	0.06	-0.054	0.058	1.0E-04
YGR168C	YGR168C	UWOPS87_Ator08_hmg124h_6.JPG.dat	824.0	50.3	1.0	0.06	0.062	0.061	5.7E-05
YGR169C	YGR169C	UWOPS87_Ator08_hmg124h_6.JPG.dat	767.5	42.5	1.0	0.05	-0.033	0.054	4.5E-05
YGR170W	YGR170W	UWOPS87_Ator08_hmg124h_6.JPG.dat	794.8	26.1	1.0	0.00	-0.010	0.004	4.2E-06
YGR173W	YGR173W	UWOPS87_Ator08_hmg124h_6.JPG.dat	798.0	12.1	1.0	0.00	0.087	0.001	4.8E-07
YGR174C	YGR174C	UWOPS87_Ator08_hmg124h_6.JPG.dat	902.0	47.8	1.0	0.02	0.011	0.017	9.3E-05
YGR176W	YGR176W	UWOPS87_Ator08_hmg124h_6.JPG.dat	890.5	43.3	1.0	0.05	0.096	0.055	4.2E-05

YGR177C	YGR177C	UWOPS87_Ator08_hmg124h_6.JPG.dat	879.5	9.3	1.0	0.01	-0.015	0.010	2.4E-07
YGR178C	YGR178C	UWOPS87_Ator08_hmg124h_6.JPG.dat	907.8	45.4	1.1	0.06	0.011	0.056	3.3E-05
YGR181W	YGR181W	UWOPS87_Ator08_hmg124h_6.JPG.dat	836.5	49.7	1.1	0.06	0.021	0.062	4.9E-05
YGR182C	YGR182C	UWOPS87_Ator08_hmg124h_6.JPG.dat	844.8	57.0	1.1	0.06	-0.081	0.060	4.9E-05
YGR183C	YGR183C	UWOPS87_Ator08_hmg124h_6.JPG.dat	918.3	99.1	1.1	0.12	-0.106	0.120	3.3E-04
YGR184C	YGR184C	UWOPS87_Ator08_hmg124h_6.JPG.dat	0.0	0.0	0.0	0.00	0.000	0.000	
YGR187C	YGR187C	UWOPS87_Ator08_hmg124h_6.JPG.dat	819.3	56.4	1.0	0.07	0.117	0.067	7.3E-05
YGR188C	YGR188C	UWOPS87_Ator08_hmg124h_6.JPG.dat	735.3	44.8	0.9	0.06	-0.043	0.056	6.0E-05
YGR189C	YGR189C	UWOPS87_Ator08_hmg124h_6.JPG.dat	803.8	34.7	1.0	0.01	0.062	0.009	2.7E-05
YGR192C	YGR192C	UWOPS87_Ator08_hmg124h_6.JPG.dat	805.3	20.8	0.9	0.00	-0.130	0.004	6.2E-06
YGR193C	YGR193C	UWOPS87_Ator08_hmg124h_6.JPG.dat	977.3	54.7	1.1	0.07	0.050	0.072	7.4E-05
YGR194C	YGR194C	UWOPS87_Ator08_hmg124h_6.JPG.dat	843.3	171.3	1.0	0.19	-0.129	0.189	2.0E-03
YGR196C	YGR196C	UWOPS87_Ator08_hmg124h_6.JPG.dat	742.5	69.4	0.9	0.07	0.047	0.070	1.1E-04
YGR197C	YGR197C	UWOPS87_Ator08_hmg124h_6.JPG.dat	824.8	82.6	1.1	0.11	-0.037	0.107	2.5E-04
YGR199W	YGR199W	UWOPS87_Ator08_hmg124h_6.JPG.dat	869.3	15.8	1.1	0.03	0.058	0.025	3.2E-06
YGR200C	YGR200C	UWOPS87_Ator08_hmg124h_6.JPG.dat	826.8	45.5	1.0	0.06	-0.047	0.057	4.7E-05
YGR201C	YGR201C	UWOPS87_Ator08_hmg124h_6.JPG.dat	778.5	70.0	1.0	0.08	-0.044	0.081	1.7E-04
YGR202C	YGR202C	UWOPS87_Ator08_hmg124h_6.JPG.dat	745.8	45.2	1.0	0.05	-0.083	0.055	5.2E-05
YGR203W	YGR203W	UWOPS87_Ator08_hmg124h_6.JPG.dat	745.3	12.7	1.0	0.01	0.005	0.007	1.8E-05
YGR205W	YGR205W	UWOPS87_Ator08_hmg124h_6.JPG.dat	854.3	46.5	1.0	0.02	-0.088	0.016	8.5E-05
YGR206W	YGR206W	UWOPS87_Ator08_hmg124h_6.JPG.dat	961.5	50.1	1.1	0.05	0.154	0.054	3.3E-05
YGR207C	YGR207C	UWOPS87_Ator08_hmg124h_6.JPG.dat	896.5	49.6	1.0	0.05	-0.047	0.053	4.0E-05
YGR208W	YGR208W	UWOPS87_Ator08_hmg124h_6.JPG.dat	873.0	72.1	1.0	0.09	0.003	0.088	1.8E-04
YGR209C	YGR209C	UWOPS87_Ator08_hmg124h_6.JPG.dat	763.3	41.7	1.0	0.05	-0.132	0.052	4.1E-05
YGR210C	YGR210C	UWOPS87_Ator08_hmg124h_6.JPG.dat	534.0	358.0	1.0	0.06	-0.214	0.062	1.3E-03
YGR212W	YGR212W	UWOPS87_Ator08_hmg124h_6.JPG.dat	661.0	9.1	0.9	0.02	-0.134	0.015	1.4E-06
YGR213C	YGR213C	UWOPS87_Ator08_hmg124h_6.JPG.dat	745.3	75.4	1.0	0.09	0.051	0.094	2.6E-04
YGR214W	YGR214W	UWOPS87_Ator08_hmg124h_6.JPG.dat	721.0	26.3	0.9	0.01	0.098	0.009	3.7E-05
YGR217W	YGR217W	UWOPS87_Ator08_hmg124h_6.JPG.dat	703.8	36.7	0.9	0.05	-0.151	0.049	4.2E-05
YGR221C	YGR221C	UWOPS87_Ator08_hmg124h_6.JPG.dat	883.0	15.9	1.1	0.02	0.024	0.019	1.3E-06
YGR223C	YGR223C	UWOPS87_Ator08_hmg124h_6.JPG.dat	840.0	32.2	1.0	0.01	-0.072	0.013	5.6E-05
YGR224W	YGR224W	UWOPS87_Ator08_hmg124h_6.JPG.dat	891.5	47.6	1.0	0.02	-0.105	0.018	1.0E-04
YGR225W	YGR225W	UWOPS87_Ator08_hmg124h_6.JPG.dat	920.5	38.5	1.1	0.05	-0.007	0.048	2.6E-05
YGR226C	YGR226C	UWOPS87_Ator08_hmg124h_6.JPG.dat	940.5	58.4	1.1	0.06	0.128	0.065	5.6E-05
YGR227W	YGR227W	UWOPS87_Ator08_hmg124h_6.JPG.dat	831.8	60.7	1.1	0.06	0.135	0.060	4.8E-05
YGR228W	YGR228W	UWOPS87_Ator08_hmg124h_6.JPG.dat	755.0	9.0	1.1	0.00	0.123	0.002	1.7E-06
YGR229C	YGR229C	UWOPS87_Ator08_hmg124h_6.JPG.dat	0.0	0.0	0.0	0.00	0.000	0.000	
YGR230W	YGR230W	UWOPS87_Ator08_hmg124h_6.JPG.dat	861.3	69.5	1.1	0.09	0.023	0.093	1.6E-04
YGR231C	YGR231C	UWOPS87_Ator08_hmg124h_6.JPG.dat	889.8	40.3	1.1	0.05	-0.083	0.051	2.4E-05
YGR232W	YGR232W	UWOPS87_Ator08_hmg124h_6.JPG.dat	833.3	38.5	1.0	0.05	-0.050	0.049	2.9E-05
YGR233C	YGR233C	UWOPS87_Ator08_hmg124h_6.JPG.dat	783.8	10.9	1.0	0.01	0.105	0.012	5.4E-07
YGR234W	YGR234W	UWOPS87_Ator08_hmg124h_6.JPG.dat	778.5	24.1	1.0	0.01	0.004	0.011	4.3E-05
YGR235C	YGR235C	UWOPS87_Ator08_hmg124h_6.JPG.dat	906.3	46.6	1.1	0.05	-0.098	0.049	2.7E-05
YGR236C	YGR236C	UWOPS87_Ator08_hmg124h_6.JPG.dat	847.3	108.0	1.0	0.13	-0.130	0.125	5.7E-04
YGR237C	YGR237C	UWOPS87_Ator08_hmg124h_6.JPG.dat	833.5	130.5	1.0	0.15	-0.121	0.154	1.0E-03
YGR238C	YGR238C	UWOPS87_Ator08_hmg124h_6.JPG.dat	725.3	46.6	1.0	0.01	-0.003	0.013	6.3E-05
YGR239C	YGR239C	UWOPS87_Ator08_hmg124h_6.JPG.dat	787.0	18.8	1.1	0.04	-0.161	0.037	1.0E-05
YGR241C	YGR241C	UWOPS87_Ator08_hmg124h_6.JPG.dat	611.3	410.4	1.1	0.08	0.101	0.082	1.8E-03
YGR242W	YGR242W	UWOPS87_Ator08_hmg124h_6.JPG.dat	903.3	88.5	1.2	0.11	0.042	0.112	2.4E-04
YGR243W	YGR243W	UWOPS87_Ator08_hmg124h_6.JPG.dat	897.3	67.8	1.2	0.03	0.130	0.026	1.7E-04
YGR244C	YGR244C	UWOPS87_Ator08_hmg124h_6.JPG.dat	812.3	66.6	1.0	0.08	0.004	0.080	1.2E-04
YGR247W	YGR247W	UWOPS87_Ator08_hmg124h_6.JPG.dat	757.5	50.5	0.9	0.06	0.121	0.061	7.5E-05
YGR248W	YGR248W	UWOPS87_Ator08_hmg124h_6.JPG.dat	836.8	40.8	1.0	0.05	-0.019	0.047	2.8E-05
YGR249W	YGR249W	UWOPS87_Ator08_hmg124h_6.JPG.dat	841.3	47.5	1.0	0.01	-0.042	0.008	2.1E-05
YGR250C	YGR250C	UWOPS87_Ator08_hmg124h_6.JPG.dat	873.8	39.9	1.0	0.04	-0.103	0.041	1.8E-05
YGR256W	YGR256W	UWOPS87_Ator08_hmg124h_6.JPG.dat	794.5	47.1	1.0	0.02	0.054	0.020	1.4E-04
YGR259C	YGR259C	UWOPS87_Ator08_hmg124h_6.JPG.dat	823.0	145.2	1.1	0.19	0.094	0.194	1.6E-03
YGR260W	YGR260W	UWOPS87_Ator08_hmg124h_6.JPG.dat	735.5	19.0	1.0	0.03	0.023	0.027	4.9E-06
YGR261C	YGR261C	UWOPS87_Ator08_hmg124h_6.JPG.dat	662.0	11.0	1.0	0.00	-0.084	0.004	5.9E-06
YGR263C	YGR263C	UWOPS87_Ator08_hmg124h_6.JPG.dat	720.3	37.7	1.0	0.05	-0.045	0.053	3.9E-05
YGR266W	YGR266W	UWOPS87_Ator08_hmg124h_6.JPG.dat	721.0	54.3	1.0	0.03	-0.050	0.030	2.8E-04
YGR268C	YGR268C	UWOPS87_Ator08_hmg124h_6.JPG.dat	707.0	14.7	1.0	0.01	-0.012	0.011	4.5E-07
YGR269W	YGR269W	UWOPS87_Ator08_hmg124h_6.JPG.dat	775.5	75.0	1.0	0.09	-0.047	0.091	1.9E-04
YGR270W	YGR270W	UWOPS87_Ator08_hmg124h_6.JPG.dat	875.8	53.0	1.1	0.02	-0.009	0.024	1.6E-04
YGR275W	YGR275W	UWOPS87_Ator08_hmg124h_6.JPG.dat	885.3	99.5	1.1	0.12	0.147	0.116	3.0E-04
YGR279C	YGR279C	UWOPS87_Ator08_hmg124h_6.JPG.dat	740.5	40.1	0.9	0.04	0.053	0.042	2.5E-05
YGR281W	YGR281W	UWOPS87_Ator08_hmg124h_6.JPG.dat	523.8	20.4	0.7	0.02	-0.395	0.020	7.5E-06
YGR282C	YGR282C	UWOPS87_Ator08_hmg124h_6.JPG.dat	658.8	26.4	0.9	0.03	-0.158	0.028	8.7E-06
YGR283C	YGR283C	UWOPS87_Ator08_hmg124h_6.JPG.dat	783.8	97.2	1.0	0.13	0.092	0.128	5.0E-04
YGR284C	YGR284C	UWOPS87_Ator08_hmg124h_6.JPG.dat	870.0	81.1	1.1	0.10	-0.016	0.102	2.0E-04

YGR286C	YGR286C	UWOPS87_Ator08_hmg124h_6.JPG.dat	828.8	24.0	1.0	0.03	-0.104	0.031	7.1E-06
YGR287C	YGR287C	UWOPS87_Ator08_hmg124h_6.JPG.dat	747.0	26.5	0.9	0.01	0.077	0.006	1.4E-05
YGR288W	YGR288W	UWOPS87_Ator08_hmg124h_6.JPG.dat	795.0	48.6	1.0	0.06	-0.028	0.059	5.5E-05
YGR290W	YGR290W	UWOPS87_Ator08_hmg124h_6.JPG.dat	758.5	17.6	1.0	0.02	-0.027	0.021	2.9E-06
YGR295C	YGR295C	UWOPS87_Ator08_hmg124h_6.JPG.dat	863.5	28.7	1.1	0.04	0.069	0.040	1.4E-05
YHL002W	YHL002W	UWOPS87_Ator08_hmg124h_6.JPG.dat	830.8	49.6	1.0	0.06	-0.170	0.062	6.3E-05
YHL003C	YHL003C	UWOPS87_Ator08_hmg124h_6.JPG.dat	834.3	64.4	1.0	0.08	-0.024	0.080	1.5E-04
YHL005C	YHL005C	UWOPS87_Ator08_hmg124h_6.JPG.dat	964.5	36.6	1.1	0.04	0.139	0.042	1.5E-05
YHL006C	YHL006C	UWOPS87_Ator08_hmg124h_6.JPG.dat	822.5	104.7	1.0	0.11	-0.104	0.114	4.4E-04
YHL007C	YHL007C	UWOPS87_Ator08_hmg124h_6.JPG.dat	0.0	0.0	0.0	0.00	0.000	0.000	
YHL008C	YHL008C	UWOPS87_Ator08_hmg124h_6.JPG.dat	835.5	59.7	1.1	0.07	-0.019	0.067	6.8E-05
YHL009C	YHL009C	UWOPS87_Ator08_hmg124h_6.JPG.dat	855.3	22.5	1.1	0.04	0.040	0.036	1.1E-05
YHL010C	YHL010C	UWOPS87_Ator08_hmg124h_6.JPG.dat	824.0	54.5	1.0	0.06	-0.051	0.062	6.7E-05
YHL012W	YHL012W	UWOPS87_Ator08_hmg124h_6.JPG.dat	757.3	34.0	1.0	0.01	-0.149	0.010	3.3E-05
YHL013C	YHL013C	UWOPS87_Ator08_hmg124h_6.JPG.dat	739.8	72.1	0.9	0.08	0.034	0.083	1.9E-04
YHL014C	YHL014C	UWOPS87_Ator08_hmg124h_6.JPG.dat	836.8	17.1	1.0	0.03	-0.011	0.031	7.2E-06
YHL016C	YHL016C	UWOPS87_Ator08_hmg124h_6.JPG.dat	764.3	14.7	0.9	0.02	0.073	0.017	1.7E-06
YHL017W	YHL017W	UWOPS87_Ator08_hmg124h_6.JPG.dat	837.0	70.6	1.0	0.09	0.002	0.086	1.9E-04
YHL019C	YHL019C	UWOPS87_Ator08_hmg124h_6.JPG.dat	887.8	85.2	1.0	0.09	0.054	0.093	2.2E-04
YHL020C	YHL020C	UWOPS87_Ator08_hmg124h_6.JPG.dat	815.0	74.1	1.0	0.08	0.138	0.083	1.7E-04
YHL021C	YHL021C	UWOPS87_Ator08_hmg124h_6.JPG.dat	720.8	15.0	1.0	0.02	0.038	0.022	3.1E-06
YHL022C	YHL022C	UWOPS87_Ator08_hmg124h_6.JPG.dat	703.8	22.7	0.9	0.03	0.081	0.032	1.2E-05
YHL023C	YHL023C	UWOPS87_Ator08_hmg124h_6.JPG.dat	821.8	65.4	1.0	0.03	0.187	0.031	3.4E-04
YHL024W	YHL024W	UWOPS87_Ator08_hmg124h_6.JPG.dat	785.0	100.8	1.0	0.13	-0.081	0.128	6.5E-04
YHL025W	YHL025W	UWOPS87_Ator08_hmg124h_6.JPG.dat	763.0	88.5	0.9	0.02	-0.047	0.022	1.9E-04
YHL026C	YHL026C	UWOPS87_Ator08_hmg124h_6.JPG.dat	812.8	21.6	1.0	0.02	0.022	0.022	2.7E-06
YHL027W	YHL027W	UWOPS87_Ator08_hmg124h_6.JPG.dat	804.5	59.3	1.0	0.07	0.045	0.068	8.5E-05
YHL028W	YHL028W	UWOPS87_Ator08_hmg124h_6.JPG.dat	850.3	91.9	1.0	0.11	0.053	0.107	3.2E-04
YHL029C	YHL029C	UWOPS87_Ator08_hmg124h_6.JPG.dat	906.8	82.7	1.0	0.09	0.145	0.089	1.7E-04
YHL030W	YHL030W	UWOPS87_Ator08_hmg124h_6.JPG.dat	941.0	77.9	1.1	0.08	0.037	0.084	1.4E-04
YHL031C	YHL031C	UWOPS87_Ator08_hmg124h_6.JPG.dat	731.5	53.7	0.9	0.06	-0.119	0.063	9.9E-05
YHL032C	YHL032C	UWOPS87_Ator08_hmg124h_6.JPG.dat	690.0	14.6	1.0	0.02	0.055	0.022	3.1E-06
YHL033C	YHL033C	UWOPS87_Ator08_hmg124h_6.JPG.dat	793.5	157.0	1.1	0.22	0.235	0.215	2.1E-03
YHL034C	YHL034C	UWOPS87_Ator08_hmg124h_6.JPG.dat	784.0	11.6	1.0	0.02	0.010	0.015	9.7E-07
YHL035C	YHL035C	UWOPS87_Ator08_hmg124h_6.JPG.dat	746.5	65.3	1.0	0.03	0.153	0.031	3.4E-04
YHL036W	YHL036W	UWOPS87_Ator08_hmg124h_6.JPG.dat	835.3	30.6	1.1	0.04	0.009	0.041	1.6E-05
YHL037C	YHL037C	UWOPS87_Ator08_hmg124h_6.JPG.dat	858.5	87.7	1.1	0.11	0.008	0.115	3.1E-04
YHL039W	YHL039W	UWOPS87_Ator08_hmg124h_6.JPG.dat	795.0	18.9	1.0	0.02	0.043	0.021	2.5E-06
YHL040C	YHL040C	UWOPS87_Ator08_hmg124h_6.JPG.dat	771.0	83.0	0.9	0.09	0.084	0.095	3.0E-04
YHL041W	YHL041W	UWOPS87_Ator08_hmg124h_6.JPG.dat	913.0	83.6	1.1	0.03	0.070	0.028	2.2E-04
YHL042W	YHL042W	UWOPS87_Ator08_hmg124h_6.JPG.dat	791.5	80.5	0.9	0.09	-0.080	0.091	2.8E-04
YHL043W	YHL043W	UWOPS87_Ator08_hmg124h_6.JPG.dat	810.3	39.0	1.0	0.06	-0.060	0.058	5.4E-05
YHL044W	YHL044W	UWOPS87_Ator08_hmg124h_6.JPG.dat	542.0	361.7	1.0	0.03	0.015	0.033	3.4E-04
YHL045W	YHL045W	UWOPS87_Ator08_hmg124h_6.JPG.dat	672.5	20.4	0.9	0.03	0.056	0.028	6.9E-06
YHL046C	YHL046C	UWOPS87_Ator08_hmg124h_6.JPG.dat	719.0	38.9	0.9	0.05	-0.085	0.048	3.6E-05
YHL047C	YHL047C	UWOPS87_Ator08_hmg124h_6.JPG.dat	769.8	92.5	1.0	0.11	-0.079	0.113	4.3E-04
YHR001W-	YHR001W-A	UWOPS87_Ator08_hmg124h_6.JPG.dat	723.8	24.0	0.9	0.03	0.082	0.033	1.3E-05
YHR003C	YHR003C	UWOPS87_Ator08_hmg124h_6.JPG.dat	795.5	15.5	1.0	0.02	-0.034	0.017	1.2E-06
YHR004C	YHR004C	UWOPS87_Ator08_hmg124h_6.JPG.dat	773.5	5.7	1.0	0.01	0.010	0.010	2.9E-07
YHR005C	YHR005C	UWOPS87_Ator08_hmg124h_6.JPG.dat	799.0	50.2	1.0	0.06	0.043	0.056	5.2E-05
YHR006W	YHR006W	UWOPS87_Ator08_hmg124h_6.JPG.dat	881.0	49.8	1.0	0.02	-0.168	0.020	1.3E-04
YHR009C	YHR009C	UWOPS87_Ator08_hmg124h_6.JPG.dat	844.3	98.1	1.0	0.12	0.109	0.116	4.5E-04
YHR012W	YHR012W	UWOPS87_Ator08_hmg124h_6.JPG.dat	777.3	161.8	1.0	0.20	0.147	0.195	2.3E-03
YHR014W	YHR014W	UWOPS87_Ator08_hmg124h_6.JPG.dat	693.8	35.0	1.0	0.04	0.086	0.044	2.6E-05
YHR015W	YHR015W	UWOPS87_Ator08_hmg124h_6.JPG.dat	511.0	340.9	0.9	0.01	0.088	0.012	5.1E-05
YHR016C	YHR016C	UWOPS87_Ator08_hmg124h_6.JPG.dat	751.3	46.3	1.0	0.07	-0.146	0.067	8.5E-05
YHR017W	YHR017W	UWOPS87_Ator08_hmg124h_6.JPG.dat	782.3	40.1	1.0	0.05	0.117	0.054	4.6E-05
YHR018C	YHR018C	UWOPS87_Ator08_hmg124h_6.JPG.dat	722.5	45.4	0.9	0.06	0.083	0.059	7.1E-05
YHR021C	YHR021C	UWOPS87_Ator08_hmg124h_6.JPG.dat	870.0	71.5	1.1	0.08	0.064	0.082	1.2E-04
YHR021W-	YHR021W-A	UWOPS87_Ator08_hmg124h_6.JPG.dat	824.5	8.7	1.0	0.00	-0.103	0.002	1.1E-06
YHR022C	YHR022C	UWOPS87_Ator08_hmg124h_6.JPG.dat	618.0	416.9	1.0	0.08	0.069	0.081	2.2E-03
YHR025W	YHR025W	UWOPS87_Ator08_hmg124h_6.JPG.dat	784.5	48.5	0.9	0.05	-0.002	0.052	4.9E-05
YHR028C	YHR028C	UWOPS87_Ator08_hmg124h_6.JPG.dat	815.8	82.7	0.9	0.09	0.019	0.093	2.6E-04
YHR029C	YHR029C	UWOPS87_Ator08_hmg124h_6.JPG.dat	904.8	70.9	1.1	0.08	0.068	0.075	8.7E-05
YHR030C	YHR030C	UWOPS87_Ator08_hmg124h_6.JPG.dat	298.0	22.1	0.4	0.03	-0.704	0.030	1.1E-04
YHR031C	YHR031C	UWOPS87_Ator08_hmg124h_6.JPG.dat	698.5	12.0	1.0	0.02	0.126	0.022	2.9E-06
YHR032W	YHR032W	UWOPS87_Ator08_hmg124h_6.JPG.dat	738.5	29.8	1.0	0.04	0.100	0.040	1.5E-05
YHR033W	YHR033W	UWOPS87_Ator08_hmg124h_6.JPG.dat	735.3	48.3	1.0	0.08	0.091	0.076	1.1E-04
YHR034C	YHR034C	UWOPS87_Ator08_hmg124h_6.JPG.dat	677.8	25.7	0.9	0.01	-0.057	0.012	5.8E-05
YHR035W	YHR035W	UWOPS87_Ator08_hmg124h_6.JPG.dat	705.8	97.5	0.9	0.13	0.042	0.128	6.7E-04

YHR037W	YHR037W	UWOPS87_Ator08_hmg124h_6.JPG.dat	802.0	47.6	1.0	0.07	0.148	0.069	8.0E-05
YHR039C	YHR039C	UWOPS87_Ator08_hmg124h_6.JPG.dat	830.3	58.0	1.1	0.07	-0.008	0.072	8.5E-05
YHR041C	YHR041C	UWOPS87_Ator08_hmg124h_6.JPG.dat	741.3	50.2	0.9	0.06	0.045	0.064	8.5E-05
YHR043C	YHR043C	UWOPS87_Ator08_hmg124h_6.JPG.dat	838.3	96.9	1.0	0.04	0.078	0.038	5.0E-04
YHR044C	YHR044C	UWOPS87_Ator08_hmg124h_6.JPG.dat	825.5	81.2	1.1	0.10	0.092	0.099	2.1E-04
YHR045W	YHR045W	UWOPS87_Ator08_hmg124h_6.JPG.dat	712.5	71.5	0.9	0.02	0.038	0.015	8.5E-05
YHR046C	YHR046C	UWOPS87_Ator08_hmg124h_6.JPG.dat	801.5	37.7	1.0	0.01	0.080	0.013	5.6E-05
YHR047C	YHR047C	UWOPS87_Ator08_hmg124h_6.JPG.dat	807.0	47.1	1.0	0.02	-0.030	0.016	8.1E-05
YHR048W	YHR048W	UWOPS87_Ator08_hmg124h_6.JPG.dat	798.3	34.7	1.0	0.02	0.022	0.025	4.3E-06
YHR049C-/	YHR049C-A	UWOPS87_Ator08_hmg124h_6.JPG.dat	770.3	19.1	1.0	0.03	0.137	0.034	1.2E-05
YHR049W	YHR049W	UWOPS87_Ator08_hmg124h_6.JPG.dat	791.3	42.0	1.0	0.06	-0.132	0.055	5.0E-05
YHR050W	YHR050W	UWOPS87_Ator08_hmg124h_6.JPG.dat	884.8	39.5	1.1	0.04	-0.178	0.041	1.6E-05
YHR057C	YHR057C	UWOPS87_Ator08_hmg124h_6.JPG.dat	943.8	42.6	1.1	0.01	0.189	0.008	1.8E-05
YHR059W	YHR059W	UWOPS87_Ator08_hmg124h_6.JPG.dat	825.0	45.1	1.0	0.02	-0.062	0.022	1.8E-04
YHR061C	YHR061C	UWOPS87_Ator08_hmg124h_6.JPG.dat	816.0	102.3	1.0	0.04	-0.205	0.038	5.1E-04
YHR066W	YHR066W	UWOPS87_Ator08_hmg124h_6.JPG.dat	869.3	38.7	1.1	0.03	0.108	0.026	4.0E-06
YHR067W	YHR067W	UWOPS87_Ator08_hmg124h_6.JPG.dat	664.8	58.3	0.9	0.08	-0.019	0.081	2.4E-04
YHR073W	YHR073W	UWOPS87_Ator08_hmg124h_6.JPG.dat	619.5	32.1	0.7	0.02	-0.088	0.017	1.7E-04
YHR075C	YHR075C	UWOPS87_Ator08_hmg124h_6.JPG.dat	860.5	45.0	1.0	0.04	0.063	0.040	1.6E-05
YHR076W	YHR076W	UWOPS87_Ator08_hmg124h_6.JPG.dat	885.3	38.6	1.1	0.04	-0.063	0.040	1.5E-05
YHR077C	YHR077C	UWOPS87_Ator08_hmg124h_6.JPG.dat	920.3	33.0	1.1	0.05	0.110	0.053	2.7E-05
YHR078W	YHR078W	UWOPS87_Ator08_hmg124h_6.JPG.dat	902.5	14.6	1.1	0.01	-0.038	0.013	4.1E-07
YHR079C	YHR079C	UWOPS87_Ator08_hmg124h_6.JPG.dat	602.0	167.8	0.6	0.07	-0.355	0.066	3.8E-03
YHR079C-E	YHR079C-B	UWOPS87_Ator08_hmg124h_6.JPG.dat	883.5	41.6	1.0	0.02	-0.011	0.019	1.3E-04
YHR080C	YHR080C	UWOPS87_Ator08_hmg124h_6.JPG.dat	926.0	107.9	1.0	0.01	-0.036	0.005	1.1E-05
YHR081W	YHR081W	UWOPS87_Ator08_hmg124h_6.JPG.dat	840.0	55.0	0.9	0.06	0.061	0.060	7.3E-05
YHR082C	YHR082C	UWOPS87_Ator08_hmg124h_6.JPG.dat	928.8	78.5	1.1	0.08	0.055	0.078	9.3E-05
YHR086W	YHR086W	UWOPS87_Ator08_hmg124h_6.JPG.dat	805.8	39.6	1.1	0.05	0.038	0.045	2.2E-05
YHR087W	YHR087W	UWOPS87_Ator08_hmg124h_6.JPG.dat	861.0	114.8	1.1	0.14	0.146	0.140	6.0E-04
YHR092C	YHR092C	UWOPS87_Ator08_hmg124h_6.JPG.dat	780.8	21.2	0.9	0.03	0.049	0.027	6.3E-06
YHR093W	YHR093W	UWOPS87_Ator08_hmg124h_6.JPG.dat	876.0	103.5	1.1	0.04	0.088	0.037	3.6E-04
YHR094C	YHR094C	UWOPS87_Ator08_hmg124h_6.JPG.dat	786.5	31.9	1.0	0.04	-0.049	0.037	1.4E-05
YHR095W	YHR095W	UWOPS87_Ator08_hmg124h_6.JPG.dat	800.0	58.9	1.0	0.08	0.059	0.078	1.3E-04
YHR096C	YHR096C	UWOPS87_Ator08_hmg124h_6.JPG.dat	788.8	62.3	0.9	0.02	-0.120	0.020	1.6E-04
YHR097C	YHR097C	UWOPS87_Ator08_hmg124h_6.JPG.dat	769.8	7.5	0.9	0.01	-0.119	0.008	2.2E-07
YHR103W	YHR103W	UWOPS87_Ator08_hmg124h_6.JPG.dat	949.3	76.0	1.1	0.08	0.062	0.085	1.4E-04
YHR104W	YHR104W	UWOPS87_Ator08_hmg124h_6.JPG.dat	885.3	29.6	1.0	0.01	0.003	0.008	2.3E-05
YHR105W	YHR105W	UWOPS87_Ator08_hmg124h_6.JPG.dat	719.8	26.3	0.9	0.03	-0.112	0.029	8.9E-06
YHR106W	YHR106W	UWOPS87_Ator08_hmg124h_6.JPG.dat	679.5	52.3	0.9	0.02	-0.095	0.016	1.0E-04
YHR108W	YHR108W	UWOPS87_Ator08_hmg124h_6.JPG.dat	800.3	105.3	1.1	0.03	-0.015	0.028	2.2E-04
YHR109W	YHR109W	UWOPS87_Ator08_hmg124h_6.JPG.dat	741.3	38.9	0.9	0.05	0.041	0.052	4.6E-05
YHR110W	YHR110W	UWOPS87_Ator08_hmg124h_6.JPG.dat	839.8	22.9	1.0	0.03	-0.025	0.030	6.3E-06
YHR111W	YHR111W	UWOPS87_Ator08_hmg124h_6.JPG.dat	759.8	28.2	1.0	0.01	0.037	0.010	3.7E-05
YHR112C	YHR112C	UWOPS87_Ator08_hmg124h_6.JPG.dat	775.0	55.1	1.0	0.02	0.066	0.017	1.1E-04
YHR113W	YHR113W	UWOPS87_Ator08_hmg124h_6.JPG.dat	849.8	37.5	1.1	0.01	0.061	0.011	3.4E-05
YHR114W	YHR114W	UWOPS87_Ator08_hmg124h_6.JPG.dat	883.5	79.4	1.0	0.09	0.054	0.090	1.9E-04
YHR115C	YHR115C	UWOPS87_Ator08_hmg124h_6.JPG.dat	875.5	53.8	1.0	0.06	-0.059	0.063	7.0E-05
YHR116W	YHR116W	UWOPS87_Ator08_hmg124h_6.JPG.dat	874.3	54.4	1.0	0.06	-0.142	0.058	5.0E-05
YHR117W	YHR117W	UWOPS87_Ator08_hmg124h_6.JPG.dat	683.8	26.8	0.9	0.03	-0.015	0.030	1.1E-05
YHR121W	YHR121W	UWOPS87_Ator08_hmg124h_6.JPG.dat	706.0	24.6	1.0	0.01	0.068	0.012	4.5E-05
YHR123W	YHR123W	UWOPS87_Ator08_hmg124h_6.JPG.dat	801.3	27.2	1.1	0.03	0.035	0.029	5.3E-06
YHR124W	YHR124W	UWOPS87_Ator08_hmg124h_6.JPG.dat	740.5	79.5	0.9	0.10	0.016	0.095	2.8E-04
YHR125W	YHR125W	UWOPS87_Ator08_hmg124h_6.JPG.dat	697.8	34.0	0.9	0.05	0.163	0.046	4.0E-05
YHR126C	YHR126C	UWOPS87_Ator08_hmg124h_6.JPG.dat	819.5	34.8	1.1	0.06	0.012	0.055	4.0E-05
YHR127W	YHR127W	UWOPS87_Ator08_hmg124h_6.JPG.dat	749.5	45.6	0.9	0.05	0.058	0.054	5.1E-05
YHR129C	YHR129C	UWOPS87_Ator08_hmg124h_6.JPG.dat	861.8	45.9	1.1	0.05	0.133	0.048	2.5E-05
YHR130C	YHR130C	UWOPS87_Ator08_hmg124h_6.JPG.dat	845.3	46.5	1.0	0.06	-0.078	0.060	6.4E-05
YHR131C	YHR131C	UWOPS87_Ator08_hmg124h_6.JPG.dat	836.0	26.9	1.0	0.01	0.013	0.007	2.0E-05
YHR132C	YHR132C	UWOPS87_Ator08_hmg124h_6.JPG.dat	841.5	115.1	1.0	0.13	0.037	0.130	6.2E-04
YHR132W-	YHR132W-A	UWOPS87_Ator08_hmg124h_6.JPG.dat	772.8	91.4	1.0	0.04	-0.070	0.038	5.3E-04
YHR133C	YHR133C	UWOPS87_Ator08_hmg124h_6.JPG.dat	613.0	37.2	0.8	0.06	-0.085	0.057	8.6E-05
YHR134W	YHR134W	UWOPS87_Ator08_hmg124h_6.JPG.dat	557.3	373.4	1.0	0.07	-0.025	0.067	1.5E-03
YHR135C	YHR135C	UWOPS87_Ator08_hmg124h_6.JPG.dat	681.5	95.9	0.8	0.03	0.041	0.029	4.4E-04
YHR136C	YHR136C	UWOPS87_Ator08_hmg124h_6.JPG.dat	839.3	47.3	1.0	0.01	0.023	0.008	2.4E-05
YHR137W	YHR137W	UWOPS87_Ator08_hmg124h_6.JPG.dat	817.0	33.0	1.0	0.01	0.039	0.014	6.5E-05
YHR138C	YHR138C	UWOPS87_Ator08_hmg124h_6.JPG.dat	877.5	7.1	1.1	0.01	-0.056	0.013	5.2E-07
YHR139C	YHR139C	UWOPS87_Ator08_hmg124h_6.JPG.dat	793.3	38.9	0.9	0.05	-0.025	0.054	5.2E-05
YHR139C-/	YHR139C-A	UWOPS87_Ator08_hmg124h_6.JPG.dat	837.8	86.0	1.0	0.10	-0.037	0.100	3.2E-04
YHR140W	YHR140W	UWOPS87_Ator08_hmg124h_6.JPG.dat	942.3	63.3	1.1	0.06	0.031	0.058	4.6E-05
YHR142W	YHR142W	UWOPS87_Ator08_hmg124h_6.JPG.dat	912.8	45.7	1.0	0.04	-0.190	0.045	2.2E-05

YHR143W	YHR143W	UWOPS87_Ator08_hmg124h_6.JPG.dat	768.3	32.9	1.0	0.03	-0.044	0.025	4.9E-06
YHR146W	YHR146W	UWOPS87_Ator08_hmg124h_6.JPG.dat	714.3	15.1	0.9	0.01	-0.067	0.010	3.4E-05
YHR150W	YHR150W	UWOPS87_Ator08_hmg124h_6.JPG.dat	511.3	342.9	0.9	0.07	0.102	0.067	1.8E-03
YHR151C	YHR151C	UWOPS87_Ator08_hmg124h_6.JPG.dat	965.0	65.0	1.3	0.09	0.018	0.088	9.7E-05
YHR152W	YHR152W	UWOPS87_Ator08_hmg124h_6.JPG.dat	759.0	30.5	1.0	0.04	-0.033	0.038	1.7E-05
YHR153C	YHR153C	UWOPS87_Ator08_hmg124h_6.JPG.dat	724.3	77.5	0.9	0.10	0.093	0.097	3.2E-04
YHR154W	YHR154W	UWOPS87_Ator08_hmg124h_6.JPG.dat	834.8	4.9	1.0	0.01	-0.105	0.013	5.3E-07
YHR155W	YHR155W	UWOPS87_Ator08_hmg124h_6.JPG.dat	896.8	22.1	1.1	0.03	-0.001	0.029	5.4E-06
YHR156C	YHR156C	UWOPS87_Ator08_hmg124h_6.JPG.dat	916.8	40.5	1.1	0.05	0.080	0.050	2.8E-05
YHR157W	YHR157W	UWOPS87_Ator08_hmg124h_6.JPG.dat	884.5	48.4	1.0	0.01	0.025	0.011	4.4E-05
YHR158C	YHR158C	UWOPS87_Ator08_hmg124h_6.JPG.dat	910.3	38.6	1.1	0.04	0.103	0.044	2.0E-05
YHR159W	YHR159W	UWOPS87_Ator08_hmg124h_6.JPG.dat	819.8	82.3	1.0	0.08	0.097	0.084	1.5E-04
YHR160C	YHR160C	UWOPS87_Ator08_hmg124h_6.JPG.dat	781.3	30.4	1.0	0.05	0.080	0.048	3.1E-05
YHR161C	YHR161C	UWOPS87_Ator08_hmg124h_6.JPG.dat	792.3	11.4	1.0	0.03	0.076	0.030	7.2E-06
YHR162W	YHR162W	UWOPS87_Ator08_hmg124h_6.JPG.dat	825.5	81.6	1.0	0.08	0.186	0.083	1.4E-04
YHR163W	YHR163W	UWOPS87_Ator08_hmg124h_6.JPG.dat	835.5	27.2	1.0	0.05	0.046	0.053	3.7E-05
YHR167W	YHR167W	UWOPS87_Ator08_hmg124h_6.JPG.dat	836.3	22.2	1.1	0.03	-0.156	0.029	5.6E-06
YHR171W	YHR171W	UWOPS87_Ator08_hmg124h_6.JPG.dat	802.3	39.2	1.0	0.03	0.007	0.033	1.0E-05
YHR176W	YHR176W	UWOPS87_Ator08_hmg124h_6.JPG.dat	849.0	26.1	1.0	0.01	-0.146	0.010	2.2E-07
YHR178W	YHR178W	UWOPS87_Ator08_hmg124h_6.JPG.dat	801.0	58.8	0.9	0.06	0.225	0.055	5.5E-05
YHR179W	YHR179W	UWOPS87_Ator08_hmg124h_6.JPG.dat	956.8	116.2	1.1	0.11	0.066	0.110	2.7E-04
YHR180W	YHR180W	UWOPS87_Ator08_hmg124h_6.JPG.dat	913.0	70.2	1.0	0.10	-0.131	0.098	2.4E-04
YHR181W	YHR181W	UWOPS87_Ator08_hmg124h_6.JPG.dat	843.3	257.6	1.0	0.28	-0.060	0.279	6.2E-03
YHR182W	YHR182W	UWOPS87_Ator08_hmg124h_6.JPG.dat	801.0	45.8	1.0	0.06	-0.050	0.060	5.4E-05
YHR184W	YHR184W	UWOPS87_Ator08_hmg124h_6.JPG.dat	788.5	45.0	1.0	0.06	-0.073	0.063	7.2E-05
YHR185C	YHR185C	UWOPS87_Ator08_hmg124h_6.JPG.dat	751.3	17.5	0.9	0.02	0.035	0.023	4.4E-06
YHR189W	YHR189W	UWOPS87_Ator08_hmg124h_6.JPG.dat	901.3	22.9	1.1	0.02	-0.120	0.023	2.6E-06
YHR191C	YHR191C	UWOPS87_Ator08_hmg124h_6.JPG.dat	836.8	25.3	1.0	0.02	0.090	0.021	2.3E-06
YHR193C	YHR193C	UWOPS87_Ator08_hmg124h_6.JPG.dat	785.5	30.4	1.0	0.03	-0.038	0.032	9.7E-06
YHR194W	YHR194W	UWOPS87_Ator08_hmg124h_6.JPG.dat	820.5	49.5	1.0	0.05	0.000	0.055	4.6E-05
YHR195W	YHR195W	UWOPS87_Ator08_hmg124h_6.JPG.dat	864.8	59.8	1.0	0.06	0.015	0.062	6.3E-05
YHR198C	YHR198C	UWOPS87_Ator08_hmg124h_6.JPG.dat	842.5	62.4	1.0	0.07	-0.096	0.073	1.2E-04
YHR199C	YHR199C	UWOPS87_Ator08_hmg124h_6.JPG.dat	906.5	30.3	1.0	0.01	0.004	0.014	6.5E-05
YHR200W	YHR200W	UWOPS87_Ator08_hmg124h_6.JPG.dat	1020.5	113.6	1.2	0.14	0.099	0.138	4.4E-04
YHR202W	YHR202W	UWOPS87_Ator08_hmg124h_6.JPG.dat	827.5	27.3	1.1	0.04	-0.031	0.040	1.3E-05
YHR203C	YHR203C	UWOPS87_Ator08_hmg124h_6.JPG.dat	750.3	19.8	1.0	0.03	0.170	0.026	5.5E-06
YHR204W	YHR204W	UWOPS87_Ator08_hmg124h_6.JPG.dat	902.0	45.0	1.1	0.06	0.044	0.056	3.6E-05
YHR206W	YHR206W	UWOPS87_Ator08_hmg124h_6.JPG.dat	864.8	47.0	1.0	0.06	0.197	0.057	4.6E-05
YHR207C	YHR207C	UWOPS87_Ator08_hmg124h_6.JPG.dat	867.3	27.4	1.1	0.03	0.006	0.028	4.6E-06
YHR209W	YHR209W	UWOPS87_Ator08_hmg124h_6.JPG.dat	749.8	82.5	0.9	0.11	-0.152	0.106	3.9E-04
YHR210C	YHR210C	UWOPS87_Ator08_hmg124h_6.JPG.dat	801.0	46.7	1.0	0.02	0.062	0.017	9.9E-05
YIL001W	YIL001W	UWOPS87_Ator08_hmg124h_6.JPG.dat	853.0	19.8	1.0	0.01	-0.103	0.012	4.6E-07
YIL002C	YIL002C	UWOPS87_Ator08_hmg124h_6.JPG.dat	896.8	131.5	1.0	0.15	0.070	0.145	7.7E-04
YIL005W	YIL005W	UWOPS87_Ator08_hmg124h_6.JPG.dat	915.3	40.4	1.0	0.04	-0.055	0.041	1.8E-05
YIL006W	YIL006W	UWOPS87_Ator08_hmg124h_6.JPG.dat	780.0	59.5	0.9	0.07	-0.169	0.070	1.2E-04
YIL007C	YIL007C	UWOPS87_Ator08_hmg124h_6.JPG.dat	701.8	12.6	1.0	0.00	0.093	0.001	5.4E-07
YIL008W	YIL008W	UWOPS87_Ator08_hmg124h_6.JPG.dat	878.3	115.1	1.1	0.03	-0.112	0.028	2.1E-04
YIL009C-A	YIL009C-A	UWOPS87_Ator08_hmg124h_6.JPG.dat	0.0	0.0	0.0	0.00	0.000	0.000	
YIL010W	YIL010W	UWOPS87_Ator08_hmg124h_6.JPG.dat	853.8	35.2	1.1	0.01	-0.016	0.009	2.2E-05
YIL011W	YIL011W	UWOPS87_Ator08_hmg124h_6.JPG.dat	800.8	49.2	1.1	0.02	0.042	0.019	1.1E-04
YIL012W	YIL012W	UWOPS87_Ator08_hmg124h_6.JPG.dat	773.5	49.2	1.0	0.06	0.162	0.059	5.8E-05
YIL013C	YIL013C	UWOPS87_Ator08_hmg124h_6.JPG.dat	815.5	13.5	1.0	0.02	-0.042	0.023	3.2E-06
YIL014W	YIL014W	UWOPS87_Ator08_hmg124h_6.JPG.dat	860.3	18.9	1.0	0.03	0.036	0.026	4.2E-06
YIL015C-A	YIL015C-A	UWOPS87_Ator08_hmg124h_6.JPG.dat	837.3	27.9	1.0	0.01	-0.025	0.007	1.7E-05
YIL015W	YIL015W	UWOPS87_Ator08_hmg124h_6.JPG.dat	918.3	54.7	1.0	0.06	0.005	0.065	6.5E-05
YIL016W	YIL016W	UWOPS87_Ator08_hmg124h_6.JPG.dat	872.0	68.5	1.0	0.03	-0.054	0.026	2.2E-04
YIL017C	YIL017C	UWOPS87_Ator08_hmg124h_6.JPG.dat	704.8	10.2	1.0	0.00	-0.224	0.003	4.0E-06
YIL020C	YIL020C	UWOPS87_Ator08_hmg124h_6.JPG.dat	767.5	52.0	1.1	0.02	0.023	0.024	1.6E-04
YIL023C	YIL023C	UWOPS87_Ator08_hmg124h_6.JPG.dat	812.3	104.7	1.1	0.13	-0.006	0.126	4.7E-04
YIL024C	YIL024C	UWOPS87_Ator08_hmg124h_6.JPG.dat	847.8	14.5	1.0	0.00	-0.082	0.003	2.5E-06
YIL025C	YIL025C	UWOPS87_Ator08_hmg124h_6.JPG.dat	838.5	19.6	1.1	0.02	-0.065	0.017	1.2E-06
YIL027C	YIL027C	UWOPS87_Ator08_hmg124h_6.JPG.dat	692.8	32.1	0.9	0.04	0.000	0.037	2.1E-05
YIL028W	YIL028W	UWOPS87_Ator08_hmg124h_6.JPG.dat	849.3	16.9	1.1	0.03	-0.002	0.026	4.2E-06
YIL029C	YIL029C	UWOPS87_Ator08_hmg124h_6.JPG.dat	792.3	91.4	1.0	0.11	-0.045	0.109	4.1E-04
YIL030C	YIL030C	UWOPS87_Ator08_hmg124h_6.JPG.dat	903.5	30.3	1.0	0.03	0.081	0.033	8.4E-06
YIL032C	YIL032C	UWOPS87_Ator08_hmg124h_6.JPG.dat	815.0	62.4	0.9	0.07	-0.023	0.072	1.3E-04
YIL034C	YIL034C	UWOPS87_Ator08_hmg124h_6.JPG.dat	761.0	178.2	0.9	0.22	-0.077	0.215	3.2E-03
YIL035C	YIL035C	UWOPS87_Ator08_hmg124h_6.JPG.dat	676.0	68.9	0.9	0.09	0.052	0.087	2.3E-04
YIL036W	YIL036W	UWOPS87_Ator08_hmg124h_6.JPG.dat	752.5	50.9	1.0	0.07	-0.113	0.069	8.7E-05
YIL037C	YIL037C	UWOPS87_Ator08_hmg124h_6.JPG.dat	876.3	47.2	1.1	0.01	-0.053	0.013	4.1E-05

YIL038C	YIL038C	UWOPS87_Ator08_hmg124h_6.JPG.dat	890.8	24.7	1.1	0.04	0.001	0.042	1.6E-05
YIL039W	YIL039W	UWOPS87_Ator08_hmg124h_6.JPG.dat	772.0	38.5	1.0	0.04	0.116	0.037	1.7E-05
YIL040W	YIL040W	UWOPS87_Ator08_hmg124h_6.JPG.dat	834.5	13.7	1.0	0.01	0.010	0.006	1.2E-05
YIL041W	YIL041W	UWOPS87_Ator08_hmg124h_6.JPG.dat	769.5	85.8	0.9	0.10	0.038	0.102	3.7E-04
YIL042C	YIL042C	UWOPS87_Ator08_hmg124h_6.JPG.dat	882.5	55.2	1.0	0.06	-0.011	0.057	4.7E-05
YIL043C	YIL043C	UWOPS87_Ator08_hmg124h_6.JPG.dat	845.5	56.5	1.0	0.01	0.012	0.008	2.4E-05
YIL044C	YIL044C	UWOPS87_Ator08_hmg124h_6.JPG.dat	964.5	81.3	1.1	0.08	0.116	0.081	1.2E-04
YIL045W	YIL045W	UWOPS87_Ator08_hmg124h_6.JPG.dat	869.0	62.6	1.0	0.08	0.172	0.082	1.4E-04
YIL047C	YIL047C	UWOPS87_Ator08_hmg124h_6.JPG.dat	739.0	10.8	1.0	0.01	0.053	0.009	2.3E-07
YIL049W	YIL049W	UWOPS87_Ator08_hmg124h_6.JPG.dat	778.0	44.5	1.0	0.07	-0.149	0.073	9.2E-05
YIL050W	YIL050W	UWOPS87_Ator08_hmg124h_6.JPG.dat	499.3	333.1	0.9	0.02	0.107	0.016	1.1E-04
YIL052C	YIL052C	UWOPS87_Ator08_hmg124h_6.JPG.dat	794.3	50.0	1.0	0.06	-0.003	0.063	6.6E-05
YIL053W	YIL053W	UWOPS87_Ator08_hmg124h_6.JPG.dat	772.0	95.7	1.0	0.12	-0.037	0.118	4.7E-04
YIL054W	YIL054W	UWOPS87_Ator08_hmg124h_6.JPG.dat	872.8	63.9	1.1	0.01	-0.039	0.008	1.5E-05
YIL055C	YIL055C	UWOPS87_Ator08_hmg124h_6.JPG.dat	794.3	37.3	1.0	0.04	-0.095	0.041	2.1E-05
YIL056W	YIL056W	UWOPS87_Ator08_hmg124h_6.JPG.dat	828.0	36.8	1.0	0.05	0.006	0.052	4.0E-05
YIL057C	YIL057C	UWOPS87_Ator08_hmg124h_6.JPG.dat	905.3	79.1	1.1	0.09	0.059	0.093	1.9E-04
YIL058W	YIL058W	UWOPS87_Ator08_hmg124h_6.JPG.dat	938.8	50.3	1.1	0.06	-0.072	0.055	3.7E-05
YIL059C	YIL059C	UWOPS87_Ator08_hmg124h_6.JPG.dat	765.0	64.2	0.9	0.07	0.053	0.075	1.5E-04
YOR202W	YOR202W	UWOPS87_Ator08_hmg124h_6.JPG.dat	816.8	128.8	1.0	0.13	-0.010	0.129	3.2E-241
1	1	UWOPS87_Ator08_hmg124h_7.JPG.dat	943.8	113.2	1.1	0.06	0.063	0.064	5.0E-05
2	2	UWOPS87_Ator08_hmg124h_7.JPG.dat	813.3	16.4	1.0	0.03	-0.113	0.027	5.6E-06
3	3	UWOPS87_Ator08_hmg124h_7.JPG.dat	791.8	118.6	1.0	0.13	0.051	0.125	6.2E-04
4	4	UWOPS87_Ator08_hmg124h_7.JPG.dat	747.0	89.2	0.9	0.09	-0.038	0.092	2.5E-04
YIL060W	YIL060W	UWOPS87_Ator08_hmg124h_7.JPG.dat	648.5	51.3	0.9	0.06	0.089	0.064	1.1E-04
YIL064W	YIL064W	UWOPS87_Ator08_hmg124h_7.JPG.dat	731.3	28.0	0.9	0.04	0.047	0.038	1.8E-05
YIL065C	YIL065C	UWOPS87_Ator08_hmg124h_7.JPG.dat	554.8	370.1	0.9	0.02	-0.021	0.021	1.7E-04
YIL066C	YIL066C	UWOPS87_Ator08_hmg124h_7.JPG.dat	790.5	87.2	0.9	0.10	0.051	0.099	3.1E-04
YIL067C	YIL067C	UWOPS87_Ator08_hmg124h_7.JPG.dat	866.8	30.1	1.0	0.03	0.100	0.034	9.8E-06
YIL070C	YIL070C	UWOPS87_Ator08_hmg124h_7.JPG.dat	851.3	19.1	1.0	0.02	-0.105	0.020	2.2E-06
YIL071C	YIL071C	UWOPS87_Ator08_hmg124h_7.JPG.dat	769.3	8.1	0.9	0.01	0.024	0.009	2.9E-07
YIL072W	YIL072W	UWOPS87_Ator08_hmg124h_7.JPG.dat	799.0	46.4	1.0	0.06	-0.014	0.057	5.9E-05
YIL073C	YIL073C	UWOPS87_Ator08_hmg124h_7.JPG.dat	754.0	135.4	0.9	0.15	-0.043	0.150	1.4E-03
YIL074C	YIL074C	UWOPS87_Ator08_hmg124h_7.JPG.dat	792.3	62.4	0.9	0.07	0.072	0.068	1.0E-04
YIL076W	YIL076W	UWOPS87_Ator08_hmg124h_7.JPG.dat	860.8	67.5	1.1	0.10	0.109	0.103	2.3E-04
YIL077C	YIL077C	UWOPS87_Ator08_hmg124h_7.JPG.dat	720.8	28.5	1.0	0.04	0.093	0.042	2.3E-05
YIL079C	YIL079C	UWOPS87_Ator08_hmg124h_7.JPG.dat	725.3	31.7	0.9	0.04	-0.062	0.040	2.3E-05
YIL084C	YIL084C	UWOPS87_Ator08_hmg124h_7.JPG.dat	0.0	0.0	0.0	0.00	0.000	0.000	
YIL086C	YIL086C	UWOPS87_Ator08_hmg124h_7.JPG.dat	860.5	69.3	1.0	0.08	-0.023	0.077	1.3E-04
YIL087C	YIL087C	UWOPS87_Ator08_hmg124h_7.JPG.dat	873.0	17.2	1.0	0.02	-0.033	0.024	3.9E-06
YIL088C	YIL088C	UWOPS87_Ator08_hmg124h_7.JPG.dat	838.5	30.8	1.0	0.01	0.055	0.014	6.8E-05
YIL089W	YIL089W	UWOPS87_Ator08_hmg124h_7.JPG.dat	776.0	48.1	0.9	0.06	0.065	0.058	6.9E-05
YIL090W	YIL090W	UWOPS87_Ator08_hmg124h_7.JPG.dat	614.8	420.0	0.9	0.13	0.044	0.128	6.0E-03
YIL092W	YIL092W	UWOPS87_Ator08_hmg124h_7.JPG.dat	829.3	56.1	1.0	0.01	-0.047	0.007	1.7E-05
YIL093C	YIL093C	UWOPS87_Ator08_hmg124h_7.JPG.dat	782.5	155.2	0.9	0.18	0.131	0.178	2.0E-03
YIL095W	YIL095W	UWOPS87_Ator08_hmg124h_7.JPG.dat	747.8	39.6	0.9	0.05	0.015	0.052	4.6E-05
YIL096C	YIL096C	UWOPS87_Ator08_hmg124h_7.JPG.dat	683.0	22.9	0.9	0.03	0.021	0.027	6.7E-06
YIL097W	YIL097W	UWOPS87_Ator08_hmg124h_7.JPG.dat	785.8	45.6	1.0	0.02	-0.107	0.017	1.1E-04
YIL098C	YIL098C	UWOPS87_Ator08_hmg124h_7.JPG.dat	824.3	11.7	1.0	0.00	-0.012	0.001	1.4E-07
YIL099W	YIL099W	UWOPS87_Ator08_hmg124h_7.JPG.dat	855.3	81.7	1.0	0.10	-0.049	0.103	3.0E-04
YIL100W	YIL100W	UWOPS87_Ator08_hmg124h_7.JPG.dat	898.8	11.1	1.0	0.01	-0.009	0.006	1.3E-05
YIL101C	YIL101C	UWOPS87_Ator08_hmg124h_7.JPG.dat	894.0	52.3	1.0	0.05	0.053	0.053	3.7E-05
YIL103W	YIL103W	UWOPS87_Ator08_hmg124h_7.JPG.dat	723.0	65.0	0.8	0.07	-0.125	0.074	1.8E-04
YIL105C	YIL105C	UWOPS87_Ator08_hmg124h_7.JPG.dat	793.8	60.8	0.9	0.07	-0.033	0.067	1.1E-04
YIL107C	YIL107C	UWOPS87_Ator08_hmg124h_7.JPG.dat	906.5	96.5	1.0	0.11	0.063	0.110	3.3E-04
YIL108W	YIL108W	UWOPS87_Ator08_hmg124h_7.JPG.dat	934.5	63.3	1.1	0.08	0.005	0.079	9.5E-05
YIL110W	YIL110W	UWOPS87_Ator08_hmg124h_7.JPG.dat	771.8	73.7	1.0	0.07	0.165	0.070	9.2E-05
YIL112W	YIL112W	UWOPS87_Ator08_hmg124h_7.JPG.dat	671.3	32.6	0.9	0.03	-0.099	0.030	9.8E-06
YIL113W	YIL113W	UWOPS87_Ator08_hmg124h_7.JPG.dat	700.0	22.2	0.9	0.01	-0.047	0.009	3.1E-05
YIL114C	YIL114C	UWOPS87_Ator08_hmg124h_7.JPG.dat	827.0	32.1	1.0	0.04	0.034	0.038	1.5E-05
YIL116W	YIL116W	UWOPS87_Ator08_hmg124h_7.JPG.dat	808.3	50.5	1.0	0.07	0.047	0.067	8.5E-05
YIL117C	YIL117C	UWOPS87_Ator08_hmg124h_7.JPG.dat	879.0	57.1	1.0	0.00	-0.009	0.004	4.2E-06
YIL119C	YIL119C	UWOPS87_Ator08_hmg124h_7.JPG.dat	812.3	41.1	0.9	0.02	-0.120	0.018	1.2E-04
YIL120W	YIL120W	UWOPS87_Ator08_hmg124h_7.JPG.dat	861.3	51.4	1.1	0.02	0.075	0.022	1.4E-04
YIL123W	YIL123W	UWOPS87_Ator08_hmg124h_7.JPG.dat	890.0	48.8	1.0	0.05	0.116	0.051	3.2E-05
YIL124W	YIL124W	UWOPS87_Ator08_hmg124h_7.JPG.dat	896.3	44.4	1.0	0.06	-0.092	0.055	4.0E-05
YIL128W	YIL128W	UWOPS87_Ator08_hmg124h_7.JPG.dat	925.8	63.9	1.2	0.08	0.121	0.075	7.7E-05
YIL130W	YIL130W	UWOPS87_Ator08_hmg124h_7.JPG.dat	711.5	70.4	1.0	0.09	0.026	0.087	2.1E-04
YIL132C	YIL132C	UWOPS87_Ator08_hmg124h_7.JPG.dat	735.3	27.9	1.0	0.05	0.111	0.051	4.3E-05
YIL133C	YIL133C	UWOPS87_Ator08_hmg124h_7.JPG.dat	835.5	44.4	1.0	0.06	0.110	0.063	6.5E-05

YIL134W	YIL134W	UWOPS87_Ator08_hmg124h_7.JPG.dat	931.5	34.9	1.1	0.04	-0.087	0.042	1.5E-05
YIL135C	YIL135C	UWOPS87_Ator08_hmg124h_7.JPG.dat	877.0	34.4	1.0	0.04	-0.088	0.036	1.1E-05
YIL137C	YIL137C	UWOPS87_Ator08_hmg124h_7.JPG.dat	852.3	42.3	1.0	0.03	0.112	0.030	7.5E-06
YIL138C	YIL138C	UWOPS87_Ator08_hmg124h_7.JPG.dat	902.8	19.4	1.0	0.03	0.000	0.034	9.6E-06
YIL139C	YIL139C	UWOPS87_Ator08_hmg124h_7.JPG.dat	944.0	15.0	1.1	0.03	-0.064	0.031	6.6E-06
YIL140W	YIL140W	UWOPS87_Ator08_hmg124h_7.JPG.dat	881.5	63.3	1.0	0.07	-0.037	0.068	8.7E-05
YIL141W	YIL141W	UWOPS87_Ator08_hmg124h_7.JPG.dat	868.3	36.6	1.0	0.05	-0.070	0.046	2.9E-05
YIL145C	YIL145C	UWOPS87_Ator08_hmg124h_7.JPG.dat	742.0	133.4	1.0	0.04	0.086	0.044	7.0E-04
YIL146C	YIL146C	UWOPS87_Ator08_hmg124h_7.JPG.dat	903.0	59.3	1.1	0.10	0.014	0.096	1.7E-04
YIL148W	YIL148W	UWOPS87_Ator08_hmg124h_7.JPG.dat	721.8	19.6	0.9	0.04	0.078	0.036	1.5E-05
YIL149C	YIL149C	UWOPS87_Ator08_hmg124h_7.JPG.dat	873.0	36.8	1.0	0.01	0.057	0.015	6.7E-05
YIL152W	YIL152W	UWOPS87_Ator08_hmg124h_7.JPG.dat	854.3	69.9	1.0	0.08	0.049	0.082	1.4E-04
YIL153W	YIL153W	UWOPS87_Ator08_hmg124h_7.JPG.dat	898.8	83.3	1.1	0.10	0.079	0.096	1.8E-04
YIL154C	YIL154C	UWOPS87_Ator08_hmg124h_7.JPG.dat	836.3	28.2	1.0	0.04	0.006	0.043	2.2E-05
YIL155C	YIL155C	UWOPS87_Ator08_hmg124h_7.JPG.dat	842.8	12.1	1.0	0.01	-0.079	0.013	6.1E-07
YIL156W	YIL156W	UWOPS87_Ator08_hmg124h_7.JPG.dat	897.8	21.3	1.1	0.02	-0.039	0.024	3.2E-06
YIL157C	YIL157C	UWOPS87_Ator08_hmg124h_7.JPG.dat	870.3	80.3	1.0	0.09	-0.022	0.094	2.1E-04
YIL159W	YIL159W	UWOPS87_Ator08_hmg124h_7.JPG.dat	961.5	38.1	1.1	0.03	0.116	0.032	6.4E-06
YIL160C	YIL160C	UWOPS87_Ator08_hmg124h_7.JPG.dat	811.5	74.6	0.9	0.02	-0.169	0.022	1.9E-04
YIL161W	YIL161W	UWOPS87_Ator08_hmg124h_7.JPG.dat	771.0	41.6	1.0	0.05	-0.073	0.049	3.6E-05
YIL162W	YIL162W	UWOPS87_Ator08_hmg124h_7.JPG.dat	717.8	40.2	0.9	0.06	-0.037	0.060	8.4E-05
YIL163C	YIL163C	UWOPS87_Ator08_hmg124h_7.JPG.dat	791.3	53.7	1.0	0.08	-0.159	0.079	1.5E-04
YIL164C	YIL164C	UWOPS87_Ator08_hmg124h_7.JPG.dat	927.3	40.2	1.1	0.05	-0.003	0.052	2.8E-05
YIL165C	YIL165C	UWOPS87_Ator08_hmg124h_7.JPG.dat	843.3	30.3	1.0	0.01	-0.015	0.006	1.0E-05
YIL166C	YIL166C	UWOPS87_Ator08_hmg124h_7.JPG.dat	783.3	71.9	1.0	0.09	-0.143	0.092	2.4E-04
YIL167W	YIL167W	UWOPS87_Ator08_hmg124h_7.JPG.dat	799.3	38.7	1.0	0.04	0.121	0.042	2.3E-05
YIL168W	YIL168W	UWOPS87_Ator08_hmg124h_7.JPG.dat	911.5	46.6	1.1	0.02	0.081	0.019	1.0E-04
YIL170W	YIL170W	UWOPS87_Ator08_hmg124h_7.JPG.dat	957.8	99.5	1.1	0.11	0.206	0.111	2.5E-04
YIL173W	YIL173W	UWOPS87_Ator08_hmg124h_7.JPG.dat	899.3	20.5	1.1	0.00	-0.006	0.004	4.6E-06
YIR001C	YIR001C	UWOPS87_Ator08_hmg124h_7.JPG.dat	869.0	36.8	1.0	0.04	-0.016	0.041	1.7E-05
YIR002C	YIR002C	UWOPS87_Ator08_hmg124h_7.JPG.dat	742.5	79.7	0.9	0.02	-0.068	0.015	1.0E-04
YIR003W	YIR003W	UWOPS87_Ator08_hmg124h_7.JPG.dat	861.8	38.0	1.2	0.04	-0.003	0.044	1.5E-05
YIR005W	YIR005W	UWOPS87_Ator08_hmg124h_7.JPG.dat	839.5	13.6	1.1	0.02	-0.118	0.016	9.6E-07
YIR007W	YIR007W	UWOPS87_Ator08_hmg124h_7.JPG.dat	716.8	14.6	0.9	0.02	-0.105	0.020	2.6E-06
YIR009W	YIR009W	UWOPS87_Ator08_hmg124h_7.JPG.dat	806.8	4.4	1.0	0.01	0.033	0.009	2.0E-07
YIR013C	YIR013C	UWOPS87_Ator08_hmg124h_7.JPG.dat	890.3	24.6	1.1	0.02	-0.073	0.024	3.3E-06
YIR014W	YIR014W	UWOPS87_Ator08_hmg124h_7.JPG.dat	867.8	15.7	1.0	0.01	-0.105	0.013	5.8E-07
YIR016W	YIR016W	UWOPS87_Ator08_hmg124h_7.JPG.dat	864.5	33.6	1.0	0.04	-0.002	0.045	2.2E-05
YIR017C	YIR017C	UWOPS87_Ator08_hmg124h_7.JPG.dat	890.5	16.4	1.1	0.03	-0.014	0.027	4.6E-06
YIR018W	YIR018W	UWOPS87_Ator08_hmg124h_7.JPG.dat	915.5	15.5	1.1	0.02	0.014	0.022	2.4E-06
YIR019C	YIR019C	UWOPS87_Ator08_hmg124h_7.JPG.dat	924.8	1.9	1.1	0.01	0.036	0.011	2.5E-07
YIR020C	YIR020C	UWOPS87_Ator08_hmg124h_7.JPG.dat	891.3	44.2	1.1	0.05	-0.068	0.050	2.7E-05
YIR020W-E	YIR020W-B	UWOPS87_Ator08_hmg124h_7.JPG.dat	875.3	166.3	1.1	0.04	-0.149	0.042	4.9E-04
YIR023W	YIR023W	UWOPS87_Ator08_hmg124h_7.JPG.dat	848.5	36.7	1.1	0.04	0.082	0.038	1.2E-05
YIR024C	YIR024C	UWOPS87_Ator08_hmg124h_7.JPG.dat	854.5	58.3	1.1	0.07	-0.049	0.070	7.6E-05
YIR025W	YIR025W	UWOPS87_Ator08_hmg124h_7.JPG.dat	875.0	27.6	1.0	0.03	-0.015	0.031	8.2E-06
YIR027C	YIR027C	UWOPS87_Ator08_hmg124h_7.JPG.dat	904.3	15.0	1.0	0.02	0.084	0.019	1.7E-06
YIR028W	YIR028W	UWOPS87_Ator08_hmg124h_7.JPG.dat	885.3	11.1	1.0	0.02	0.035	0.016	1.2E-06
YIR029W	YIR029W	UWOPS87_Ator08_hmg124h_7.JPG.dat	902.0	6.2	1.0	0.01	-0.072	0.014	7.1E-07
YIR030C	YIR030C	UWOPS87_Ator08_hmg124h_7.JPG.dat	887.3	15.3	1.0	0.00	0.011	0.004	5.7E-06
YIR031C	YIR031C	UWOPS87_Ator08_hmg124h_7.JPG.dat	899.5	21.4	1.0	0.03	-0.077	0.030	7.1E-06
YIR032C	YIR032C	UWOPS87_Ator08_hmg124h_7.JPG.dat	970.3	24.2	1.1	0.03	0.014	0.031	5.6E-06
YIR033W	YIR033W	UWOPS87_Ator08_hmg124h_7.JPG.dat	877.3	50.2	1.0	0.05	-0.198	0.055	4.0E-05
YIR034C	YIR034C	UWOPS87_Ator08_hmg124h_7.JPG.dat	691.5	35.8	1.0	0.04	0.115	0.044	2.4E-05
YIR035C	YIR035C	UWOPS87_Ator08_hmg124h_7.JPG.dat	855.0	28.0	1.1	0.02	-0.042	0.025	3.3E-06
YIR036C	YIR036C	UWOPS87_Ator08_hmg124h_7.JPG.dat	854.8	32.8	1.1	0.04	-0.143	0.037	1.1E-05
YIR037W	YIR037W	UWOPS87_Ator08_hmg124h_7.JPG.dat	895.5	72.6	1.0	0.07	0.037	0.072	9.3E-05
YIR038C	YIR038C	UWOPS87_Ator08_hmg124h_7.JPG.dat	823.8	55.0	1.0	0.07	-0.008	0.066	8.9E-05
YIR039C	YIR039C	UWOPS87_Ator08_hmg124h_7.JPG.dat	915.3	17.6	1.0	0.03	0.076	0.032	8.0E-06
YIR042C	YIR042C	UWOPS87_Ator08_hmg124h_7.JPG.dat	866.0	13.4	1.0	0.01	-0.041	0.009	2.7E-05
YIR043C	YIR043C	UWOPS87_Ator08_hmg124h_7.JPG.dat	929.5	23.3	1.1	0.00	0.040	0.003	3.5E-06
YIR044C	YIR044C	UWOPS87_Ator08_hmg124h_7.JPG.dat	907.3	62.2	1.0	0.07	-0.121	0.071	9.5E-05
YJL004C	YJL004C	UWOPS87_Ator08_hmg124h_7.JPG.dat	0.0	0.0	0.0	0.00	0.000	0.000	
YJL007C	YJL007C	UWOPS87_Ator08_hmg124h_7.JPG.dat	851.0	86.4	1.0	0.10	0.003	0.097	2.2E-04
YJL012C	YJL012C	UWOPS87_Ator08_hmg124h_7.JPG.dat	731.8	40.7	1.1	0.05	0.005	0.047	2.5E-05
YJL013C	YJL013C	UWOPS87_Ator08_hmg124h_7.JPG.dat	803.3	46.3	1.0	0.05	0.052	0.048	2.8E-05
YJL016W	YJL016W	UWOPS87_Ator08_hmg124h_7.JPG.dat	769.3	67.0	1.0	0.08	0.043	0.081	1.5E-04
YJL017W	YJL017W	UWOPS87_Ator08_hmg124h_7.JPG.dat	774.8	48.9	0.9	0.06	0.033	0.063	8.6E-05
YJL020C	YJL020C	UWOPS87_Ator08_hmg124h_7.JPG.dat	844.8	50.2	1.1	0.01	0.001	0.006	1.2E-05
YJL021C	YJL021C	UWOPS87_Ator08_hmg124h_7.JPG.dat	888.3	87.0	1.0	0.09	0.018	0.094	2.0E-04

YJL022W	YJL022W	UWOPS87_Ator08_hmg124h_7.JPG.dat	868.3	38.0	1.0	0.05	0.025	0.046	2.5E-05
YJL023C	YJL023C	UWOPS87_Ator08_hmg124h_7.JPG.dat	857.0	21.9	1.0	0.03	-0.074	0.033	9.6E-06
YJL024C	YJL024C	UWOPS87_Ator08_hmg124h_7.JPG.dat	935.0	60.6	1.1	0.07	-0.035	0.068	6.8E-05
YJL027C	YJL027C	UWOPS87_Ator08_hmg124h_7.JPG.dat	801.5	26.4	1.0	0.02	-0.097	0.024	4.0E-06
YJL028W	YJL028W	UWOPS87_Ator08_hmg124h_7.JPG.dat	788.0	63.8	1.0	0.08	0.025	0.076	1.2E-04
YJL030W	YJL030W	UWOPS87_Ator08_hmg124h_7.JPG.dat	741.5	74.3	1.0	0.10	0.096	0.105	3.2E-04
YJL036W	YJL036W	UWOPS87_Ator08_hmg124h_7.JPG.dat	750.3	58.5	0.9	0.07	-0.301	0.069	1.2E-04
YJL037W	YJL037W	UWOPS87_Ator08_hmg124h_7.JPG.dat	756.5	55.6	1.0	0.02	0.085	0.022	1.7E-04
YJL038C	YJL038C	UWOPS87_Ator08_hmg124h_7.JPG.dat	870.0	56.1	1.0	0.05	0.017	0.046	2.7E-05
YJL042W	YJL042W	UWOPS87_Ator08_hmg124h_7.JPG.dat	845.3	109.9	1.0	0.13	0.018	0.128	5.5E-04
YJL043W	YJL043W	UWOPS87_Ator08_hmg124h_7.JPG.dat	870.5	53.2	1.0	0.05	0.062	0.046	2.7E-05
YJL044C	YJL044C	UWOPS87_Ator08_hmg124h_7.JPG.dat	890.5	100.3	1.0	0.01	-0.057	0.013	5.8E-05
YJL045W	YJL045W	UWOPS87_Ator08_hmg124h_7.JPG.dat	802.0	88.1	0.9	0.09	-0.025	0.087	2.4E-04
YJL046W	YJL046W	UWOPS87_Ator08_hmg124h_7.JPG.dat	0.0	0.0	0.0	0.00	0.000	0.000	
YJL047C	YJL047C	UWOPS87_Ator08_hmg124h_7.JPG.dat	959.3	73.0	1.1	0.08	-0.039	0.078	9.2E-05
YJL048C	YJL048C	UWOPS87_Ator08_hmg124h_7.JPG.dat	768.3	115.2	0.9	0.14	0.024	0.138	9.3E-04
YJL049W	YJL049W	UWOPS87_Ator08_hmg124h_7.JPG.dat	740.5	60.1	1.0	0.09	0.105	0.090	2.0E-04
YJL051W	YJL051W	UWOPS87_Ator08_hmg124h_7.JPG.dat	772.3	27.3	0.9	0.04	-0.135	0.045	2.9E-05
YJL053W	YJL053W	UWOPS87_Ator08_hmg124h_7.JPG.dat	583.0	29.0	0.7	0.04	-0.134	0.038	3.8E-05
YJL055W	YJL055W	UWOPS87_Ator08_hmg124h_7.JPG.dat	739.0	51.9	0.9	0.06	0.002	0.064	1.1E-04
YJL057C	YJL057C	UWOPS87_Ator08_hmg124h_7.JPG.dat	889.8	59.5	1.1	0.02	-0.045	0.021	1.4E-04
YJL058C	YJL058C	UWOPS87_Ator08_hmg124h_7.JPG.dat	823.8	18.7	1.0	0.02	-0.034	0.024	4.0E-06
YJL059W	YJL059W	UWOPS87_Ator08_hmg124h_7.JPG.dat	838.8	65.4	1.0	0.03	0.028	0.026	2.2E-04
YJL060W	YJL060W	UWOPS87_Ator08_hmg124h_7.JPG.dat	899.5	32.3	1.0	0.01	-0.095	0.014	6.5E-05
YJL062W	YJL062W	UWOPS87_Ator08_hmg124h_7.JPG.dat	965.0	83.0	1.1	0.09	0.216	0.091	1.5E-04
YJL064W	YJL064W	UWOPS87_Ator08_hmg124h_7.JPG.dat	546.8	365.5	0.9	0.04	0.035	0.043	8.2E-04
YJL065C	YJL065C	UWOPS87_Ator08_hmg124h_7.JPG.dat	815.0	59.3	1.0	0.07	-0.118	0.065	8.3E-05
YJL066C	YJL066C	UWOPS87_Ator08_hmg124h_7.JPG.dat	751.0	68.5	0.9	0.03	-0.036	0.029	3.3E-04
YJL067W	YJL067W	UWOPS87_Ator08_hmg124h_7.JPG.dat	886.0	36.7	1.1	0.06	-0.040	0.061	5.4E-05
YJL068C	YJL068C	UWOPS87_Ator08_hmg124h_7.JPG.dat	767.0	52.3	1.0	0.06	0.124	0.057	5.8E-05
YJL070C	YJL070C	UWOPS87_Ator08_hmg124h_7.JPG.dat	767.3	70.9	0.9	0.03	-0.163	0.032	4.3E-04
YJL071W	YJL071W	UWOPS87_Ator08_hmg124h_7.JPG.dat	0.0	0.0	0.0	0.00	0.000	0.000	
YJL073W	YJL073W	UWOPS87_Ator08_hmg124h_7.JPG.dat	940.5	29.3	1.1	0.03	-0.180	0.034	7.6E-06
YJL077C	YJL077C	UWOPS87_Ator08_hmg124h_7.JPG.dat	824.3	89.3	1.0	0.10	-0.121	0.102	3.1E-04
YJL078C	YJL078C	UWOPS87_Ator08_hmg124h_7.JPG.dat	837.5	48.8	1.0	0.05	0.007	0.052	4.0E-05
YJL083W	YJL083W	UWOPS87_Ator08_hmg124h_7.JPG.dat	852.3	127.4	1.0	0.14	0.085	0.141	7.7E-04
YJL084C	YJL084C	UWOPS87_Ator08_hmg124h_7.JPG.dat	721.0	50.4	0.9	0.02	0.127	0.021	1.9E-04
YJL088W	YJL088W	UWOPS87_Ator08_hmg124h_7.JPG.dat	979.5	41.7	1.2	0.02	0.026	0.015	5.6E-05
YJL089W	YJL089W	UWOPS87_Ator08_hmg124h_7.JPG.dat	653.5	33.6	0.9	0.05	-0.072	0.047	4.3E-05
YJL093C	YJL093C	UWOPS87_Ator08_hmg124h_7.JPG.dat	845.8	31.4	1.1	0.01	-0.039	0.012	4.0E-05
YJL094C	YJL094C	UWOPS87_Ator08_hmg124h_7.JPG.dat	847.0	30.8	1.1	0.05	0.056	0.055	3.8E-05
YJL095W	YJL095W	UWOPS87_Ator08_hmg124h_7.JPG.dat	642.0	616.7	0.4	0.17	-0.691	0.169	5.1E-02
YJL098W	YJL098W	UWOPS87_Ator08_hmg124h_7.JPG.dat	775.5	39.4	0.9	0.04	-0.172	0.040	2.1E-05
YJL099W	YJL099W	UWOPS87_Ator08_hmg124h_7.JPG.dat	842.5	86.9	1.0	0.11	0.111	0.108	3.3E-04
YJL100W	YJL100W	UWOPS87_Ator08_hmg124h_7.JPG.dat	792.8	61.9	0.9	0.02	0.043	0.022	1.8E-04
YJL101C	YJL101C	UWOPS87_Ator08_hmg124h_7.JPG.dat	778.0	24.0	1.0	0.03	0.080	0.031	9.2E-06
YJL105W	YJL105W	UWOPS87_Ator08_hmg124h_7.JPG.dat	855.8	89.1	1.0	0.02	0.052	0.019	1.2E-04
YJL108C	YJL108C	UWOPS87_Ator08_hmg124h_7.JPG.dat	764.0	156.2	0.8	0.07	-0.199	0.068	2.1E-03
YJL110C	YJL110C	UWOPS87_Ator08_hmg124h_7.JPG.dat	757.5	105.6	1.0	0.14	-0.028	0.144	9.1E-04
YJL112W	YJL112W	UWOPS87_Ator08_hmg124h_7.JPG.dat	805.5	18.2	1.1	0.00	0.010	0.001	2.1E-07
YJL115W	YJL115W	UWOPS87_Ator08_hmg124h_7.JPG.dat	736.8	69.7	1.0	0.11	0.070	0.107	3.5E-04
YJL116C	YJL116C	UWOPS87_Ator08_hmg124h_7.JPG.dat	843.8	46.1	1.0	0.01	-0.134	0.009	2.5E-05
YJL121C	YJL121C	UWOPS87_Ator08_hmg124h_7.JPG.dat	0.0	0.0	0.0	0.00	0.000	0.000	
YJL122W	YJL122W	UWOPS87_Ator08_hmg124h_7.JPG.dat	893.0	40.9	1.1	0.02	0.100	0.020	1.1E-04
YJL123C	YJL123C	UWOPS87_Ator08_hmg124h_7.JPG.dat	894.8	50.5	1.1	0.06	0.001	0.062	5.1E-05
YJL124C	YJL124C	UWOPS87_Ator08_hmg124h_7.JPG.dat	686.0	16.1	0.9	0.02	0.055	0.016	1.7E-06
YJL126W	YJL126W	UWOPS87_Ator08_hmg124h_7.JPG.dat	950.3	19.7	1.2	0.02	-0.016	0.022	1.7E-06
YJL128C	YJL128C	UWOPS87_Ator08_hmg124h_7.JPG.dat	778.5	126.9	0.8	0.04	-0.022	0.036	6.0E-04
YJL130C	YJL130C	UWOPS87_Ator08_hmg124h_7.JPG.dat	0.0	0.0	0.0	0.00	0.000	0.000	
YJL131C	YJL131C	UWOPS87_Ator08_hmg124h_7.JPG.dat	751.0	81.0	1.0	0.10	0.036	0.097	2.6E-04
YJL132W	YJL132W	UWOPS87_Ator08_hmg124h_7.JPG.dat	699.3	45.7	1.0	0.02	0.018	0.021	1.5E-04
YJL133W	YJL133W	UWOPS87_Ator08_hmg124h_7.JPG.dat	744.8	47.0	1.0	0.06	0.049	0.064	7.1E-05
YJL134W	YJL134W	UWOPS87_Ator08_hmg124h_7.JPG.dat	830.8	61.1	1.1	0.07	-0.030	0.074	9.7E-05
YJL135W	YJL135W	UWOPS87_Ator08_hmg124h_7.JPG.dat	812.5	62.4	1.0	0.08	-0.011	0.080	1.4E-04
YJL136C	YJL136C	UWOPS87_Ator08_hmg124h_7.JPG.dat	706.3	71.6	0.9	0.03	0.032	0.027	2.9E-04
YJL137C	YJL137C	UWOPS87_Ator08_hmg124h_7.JPG.dat	848.8	28.5	1.0	0.04	0.027	0.040	1.5E-05
YJL138C	YJL138C	UWOPS87_Ator08_hmg124h_7.JPG.dat	741.0	51.8	0.9	0.06	0.023	0.064	9.0E-05
YJL139C	YJL139C	UWOPS87_Ator08_hmg124h_7.JPG.dat	861.5	29.8	1.1	0.03	-0.061	0.033	8.0E-06
YJL141C	YJL141C	UWOPS87_Ator08_hmg124h_7.JPG.dat	945.3	68.1	1.1	0.08	-0.030	0.080	9.8E-05
YJL142C	YJL142C	UWOPS87_Ator08_hmg124h_7.JPG.dat	849.8	46.6	1.1	0.06	-0.121	0.061	5.0E-05

YJL144W	YJL144W	UWOPS87_Ator08_hmg124h_7.JPG.dat	834.0	62.5	1.1	0.01	-0.010	0.008	1.8E-05
YJL145W	YJL145W	UWOPS87_Ator08_hmg124h_7.JPG.dat	782.8	33.8	1.1	0.04	0.047	0.043	1.6E-05
YJL146W	YJL146W	UWOPS87_Ator08_hmg124h_7.JPG.dat	767.8	37.6	1.0	0.05	-0.032	0.046	2.7E-05
YJL147C	YJL147C	UWOPS87_Ator08_hmg124h_7.JPG.dat	771.5	83.7	1.0	0.09	0.048	0.092	2.4E-04
YJL148W	YJL148W	UWOPS87_Ator08_hmg124h_7.JPG.dat	821.5	57.2	1.0	0.08	0.044	0.077	1.2E-04
YJL149W	YJL149W	UWOPS87_Ator08_hmg124h_7.JPG.dat	849.8	31.0	1.0	0.04	0.042	0.039	1.5E-05
YJL150W	YJL150W	UWOPS87_Ator08_hmg124h_7.JPG.dat	892.0	48.9	1.1	0.06	-0.002	0.056	3.8E-05
YJL151C	YJL151C	UWOPS87_Ator08_hmg124h_7.JPG.dat	860.5	56.4	1.0	0.00	-0.018	0.002	7.9E-07
YJL152W	YJL152W	UWOPS87_Ator08_hmg124h_7.JPG.dat	757.8	31.1	0.9	0.01	-0.031	0.006	1.4E-05
YJL153C	YJL153C	UWOPS87_Ator08_hmg124h_7.JPG.dat	886.3	79.9	1.1	0.11	0.005	0.107	2.9E-04
YJL154C	YJL154C	UWOPS87_Ator08_hmg124h_7.JPG.dat	793.8	65.3	1.0	0.01	0.006	0.015	6.6E-05
YJL155C	YJL155C	UWOPS87_Ator08_hmg124h_7.JPG.dat	684.8	44.6	0.9	0.01	-0.022	0.012	6.0E-05
YJL157C	YJL157C	UWOPS87_Ator08_hmg124h_7.JPG.dat	743.8	22.6	1.0	0.02	-0.174	0.025	3.7E-06
YJL158C	YJL158C	UWOPS87_Ator08_hmg124h_7.JPG.dat	845.0	39.5	1.1	0.06	0.038	0.059	4.2E-05
YJL159W	YJL159W	UWOPS87_Ator08_hmg124h_7.JPG.dat	713.8	22.6	0.9	0.03	-0.063	0.026	6.7E-06
YJL160C	YJL160C	UWOPS87_Ator08_hmg124h_7.JPG.dat	725.8	48.6	0.9	0.06	0.066	0.056	6.3E-05
YJL161W	YJL161W	UWOPS87_Ator08_hmg124h_7.JPG.dat	752.8	41.6	1.0	0.02	0.144	0.017	1.1E-04
YJL162C	YJL162C	UWOPS87_Ator08_hmg124h_7.JPG.dat	779.8	14.6	1.0	0.02	0.002	0.018	1.9E-06
YJL163C	YJL163C	UWOPS87_Ator08_hmg124h_7.JPG.dat	820.3	52.5	1.0	0.06	0.042	0.061	6.0E-05
YJL164C	YJL164C	UWOPS87_Ator08_hmg124h_7.JPG.dat	912.0	16.9	1.1	0.02	0.055	0.024	2.7E-06
YJL165C	YJL165C	UWOPS87_Ator08_hmg124h_7.JPG.dat	810.3	55.7	1.0	0.06	0.059	0.058	5.5E-05
YJL168C	YJL168C	UWOPS87_Ator08_hmg124h_7.JPG.dat	644.3	71.7	0.8	0.03	-0.292	0.032	5.4E-04
YJL169W	YJL169W	UWOPS87_Ator08_hmg124h_7.JPG.dat	557.5	17.9	0.8	0.04	-0.176	0.036	3.0E-05
YJL170C	YJL170C	UWOPS87_Ator08_hmg124h_7.JPG.dat	823.3	31.2	1.1	0.03	-0.152	0.034	8.8E-06
YJL171C	YJL171C	UWOPS87_Ator08_hmg124h_7.JPG.dat	860.5	40.6	1.1	0.04	-0.138	0.042	1.7E-05
YJL172W	YJL172W	UWOPS87_Ator08_hmg124h_7.JPG.dat	891.5	64.4	1.0	0.02	-0.019	0.017	8.9E-05
YJL175W	YJL175W	UWOPS87_Ator08_hmg124h_7.JPG.dat	767.3	51.0	0.9	0.07	-0.096	0.067	1.0E-04
YJL176C	YJL176C	UWOPS87_Ator08_hmg124h_7.JPG.dat	818.0	5.9	1.0	0.01	-0.045	0.010	2.8E-07
YJL178C	YJL178C	UWOPS87_Ator08_hmg124h_7.JPG.dat	836.5	35.8	1.0	0.04	0.045	0.043	2.3E-05
YJL181W	YJL181W	UWOPS87_Ator08_hmg124h_7.JPG.dat	929.5	307.3	0.9	0.04	-0.025	0.044	7.7E-04
YJL182C	YJL182C	UWOPS87_Ator08_hmg124h_7.JPG.dat	803.8	15.2	0.9	0.02	-0.018	0.018	1.9E-06
YJL183W	YJL183W	UWOPS87_Ator08_hmg124h_7.JPG.dat	814.3	62.6	0.9	0.08	-0.027	0.076	1.5E-04
YJL185C	YJL185C	UWOPS87_Ator08_hmg124h_7.JPG.dat	862.3	24.6	1.0	0.03	0.134	0.030	6.8E-06
YJL186W	YJL186W	UWOPS87_Ator08_hmg124h_7.JPG.dat	803.5	83.7	1.0	0.12	0.067	0.124	4.9E-04
YJL187C	YJL187C	UWOPS87_Ator08_hmg124h_7.JPG.dat	864.5	15.4	1.1	0.03	-0.148	0.032	6.4E-06
YJL188C	YJL188C	UWOPS87_Ator08_hmg124h_7.JPG.dat	829.3	30.5	1.0	0.03	-0.025	0.026	4.8E-06
YJL190C	YJL190C	UWOPS87_Ator08_hmg124h_7.JPG.dat	784.8	51.3	0.9	0.06	-0.098	0.063	8.1E-05
YJL191W	YJL191W	UWOPS87_Ator08_hmg124h_7.JPG.dat	820.0	32.7	1.0	0.04	-0.099	0.044	2.4E-05
YJL192C	YJL192C	UWOPS87_Ator08_hmg124h_7.JPG.dat	819.0	31.4	1.0	0.04	-0.022	0.038	1.6E-05
YJL193W	YJL193W	UWOPS87_Ator08_hmg124h_7.JPG.dat	731.3	101.9	0.9	0.02	0.027	0.021	1.7E-04
YJL196C	YJL196C	UWOPS87_Ator08_hmg124h_7.JPG.dat	859.5	7.0	1.0	0.01	0.074	0.006	4.7E-08
YJL197W	YJL197W	UWOPS87_Ator08_hmg124h_7.JPG.dat	748.0	33.8	0.9	0.04	0.057	0.040	2.5E-05
YJL198W	YJL198W	UWOPS87_Ator08_hmg124h_7.JPG.dat	851.8	54.9	1.0	0.06	0.069	0.061	6.2E-05
YJL199C	YJL199C	UWOPS87_Ator08_hmg124h_7.JPG.dat	896.5	27.0	1.1	0.01	-0.016	0.008	1.9E-05
YJL201W	YJL201W	UWOPS87_Ator08_hmg124h_7.JPG.dat	779.0	45.4	1.0	0.06	-0.099	0.056	5.2E-05
YJL204C	YJL204C	UWOPS87_Ator08_hmg124h_7.JPG.dat	654.3	83.9	0.9	0.12	-0.027	0.115	6.1E-04
YJL206C	YJL206C	UWOPS87_Ator08_hmg124h_7.JPG.dat	778.0	54.1	1.0	0.06	0.017	0.060	6.0E-05
YJL206C-A	YJL206C-A	UWOPS87_Ator08_hmg124h_7.JPG.dat	777.0	39.2	1.0	0.02	0.045	0.016	8.4E-05
YJL207C	YJL207C	UWOPS87_Ator08_hmg124h_7.JPG.dat	837.0	25.7	1.0	0.03	-0.077	0.026	4.7E-06
YJL208C	YJL208C	UWOPS87_Ator08_hmg124h_7.JPG.dat	768.0	95.7	1.0	0.04	0.078	0.042	5.9E-04
YJL210W	YJL210W	UWOPS87_Ator08_hmg124h_7.JPG.dat	867.5	8.5	1.0	0.01	0.047	0.012	3.9E-07
YJL211C	YJL211C	UWOPS87_Ator08_hmg124h_7.JPG.dat	871.3	42.0	1.0	0.01	-0.124	0.014	6.3E-05
YJL212C	YJL212C	UWOPS87_Ator08_hmg124h_7.JPG.dat	860.5	76.4	1.1	0.09	0.212	0.091	1.7E-04
YJL213W	YJL213W	UWOPS87_Ator08_hmg124h_7.JPG.dat	938.5	45.1	1.1	0.05	0.103	0.049	2.3E-05
YJL214W	YJL214W	UWOPS87_Ator08_hmg124h_7.JPG.dat	851.5	85.7	1.0	0.10	-0.017	0.103	2.6E-04
YJL215C	YJL215C	UWOPS87_Ator08_hmg124h_7.JPG.dat	780.8	89.6	1.0	0.03	0.080	0.032	3.4E-04
YJL216C	YJL216C	UWOPS87_Ator08_hmg124h_7.JPG.dat	736.3	69.2	1.1	0.11	0.076	0.106	2.7E-04
YJL217W	YJL217W	UWOPS87_Ator08_hmg124h_7.JPG.dat	712.0	25.1	1.0	0.01	0.091	0.007	1.6E-05
YJL218W	YJL218W	UWOPS87_Ator08_hmg124h_7.JPG.dat	701.8	43.6	0.9	0.05	0.052	0.052	4.7E-05
YJR001W	YJR001W	UWOPS87_Ator08_hmg124h_7.JPG.dat	956.0	24.7	1.2	0.02	-0.042	0.015	6.4E-07
YJR003C	YJR003C	UWOPS87_Ator08_hmg124h_7.JPG.dat	766.3	33.9	0.9	0.03	-0.032	0.029	8.3E-06
YJR005W	YJR005W	UWOPS87_Ator08_hmg124h_7.JPG.dat	863.0	41.2	1.0	0.02	-0.145	0.018	9.8E-05
YJR008W	YJR008W	UWOPS87_Ator08_hmg124h_7.JPG.dat	758.8	34.7	0.9	0.01	0.044	0.012	5.7E-05
YJR009C	YJR009C	UWOPS87_Ator08_hmg124h_7.JPG.dat	743.5	44.5	0.9	0.04	-0.040	0.043	2.9E-05
YJR010C-A	YJR010C-A	UWOPS87_Ator08_hmg124h_7.JPG.dat	793.3	63.9	0.9	0.06	-0.105	0.063	8.3E-05
YJR010W	YJR010W	UWOPS87_Ator08_hmg124h_7.JPG.dat	849.3	51.3	1.0	0.05	0.033	0.051	3.3E-05
YJR011C	YJR011C	UWOPS87_Ator08_hmg124h_7.JPG.dat	822.3	38.2	1.0	0.04	0.083	0.037	1.3E-05
YJR014W	YJR014W	UWOPS87_Ator08_hmg124h_7.JPG.dat	649.0	37.8	1.0	0.05	0.103	0.048	3.3E-05
YJR015W	YJR015W	UWOPS87_Ator08_hmg124h_7.JPG.dat	640.0	32.7	0.9	0.05	0.042	0.046	4.1E-05
YJR019C	YJR019C	UWOPS87_Ator08_hmg124h_7.JPG.dat	758.3	37.1	1.0	0.04	-0.092	0.043	2.0E-05

YJR020W	YJR020W	UWOPS87_Ator08_hmg124h_7.JPG.dat	876.8	62.2	1.1	0.08	-0.033	0.080	1.2E-04
YJR021C	YJR021C	UWOPS87_Ator08_hmg124h_7.JPG.dat	766.5	58.5	1.0	0.07	-0.011	0.071	1.1E-04
YJR024C	YJR024C	UWOPS87_Ator08_hmg124h_7.JPG.dat	830.5	26.8	1.0	0.03	-0.083	0.025	4.5E-06
YJR025C	YJR025C	UWOPS87_Ator08_hmg124h_7.JPG.dat	781.8	32.1	1.0	0.04	0.065	0.045	2.7E-05
YJR026W	YJR026W	UWOPS87_Ator08_hmg124h_7.JPG.dat	860.5	56.3	1.1	0.06	0.079	0.063	5.7E-05
YJR030C	YJR030C	UWOPS87_Ator08_hmg124h_7.JPG.dat	762.5	47.3	0.9	0.05	0.024	0.049	4.5E-05
YJR031C	YJR031C	UWOPS87_Ator08_hmg124h_7.JPG.dat	783.5	134.8	0.9	0.04	-0.163	0.039	6.3E-04
YJR032W	YJR032W	UWOPS87_Ator08_hmg124h_7.JPG.dat	0.0	0.0	0.0	0.00	0.000	0.000	
YJR033C	YJR033C	UWOPS87_Ator08_hmg124h_7.JPG.dat	612.5	23.4	0.9	0.04	-0.132	0.039	2.1E-05
YJR035W	YJR035W	UWOPS87_Ator08_hmg124h_7.JPG.dat	827.0	33.2	1.1	0.03	0.005	0.032	7.0E-06
YJR036C	YJR036C	UWOPS87_Ator08_hmg124h_7.JPG.dat	878.0	54.6	1.1	0.07	0.081	0.073	7.1E-05
YJR037W	YJR037W	UWOPS87_Ator08_hmg124h_7.JPG.dat	619.5	326.8	0.8	0.40	-0.234	0.402	3.3E-02
YJR038C	YJR038C	UWOPS87_Ator08_hmg124h_7.JPG.dat	700.3	10.2	0.9	0.01	-0.111	0.009	2.6E-07
YJR040W	YJR040W	UWOPS87_Ator08_hmg124h_7.JPG.dat	776.5	54.6	0.9	0.07	-0.072	0.066	9.9E-05
YJR043C	YJR043C	UWOPS87_Ator08_hmg124h_7.JPG.dat	774.5	32.6	0.9	0.01	-0.098	0.006	1.3E-05
YJR047C	YJR047C	UWOPS87_Ator08_hmg124h_7.JPG.dat	908.8	26.4	1.1	0.03	-0.008	0.026	3.7E-06
YJR048W	YJR048W	UWOPS87_Ator08_hmg124h_7.JPG.dat	867.8	12.3	1.0	0.01	-0.025	0.014	7.5E-07
YJR049C	YJR049C	UWOPS87_Ator08_hmg124h_7.JPG.dat	788.5	60.2	1.0	0.03	0.153	0.026	2.2E-04
YJR050W	YJR050W	UWOPS87_Ator08_hmg124h_7.JPG.dat	695.8	71.8	0.9	0.03	0.238	0.032	3.7E-04
YJR051W	YJR051W	UWOPS87_Ator08_hmg124h_7.JPG.dat	704.5	60.8	1.0	0.08	-0.174	0.080	1.3E-04
YJR052W	YJR052W	UWOPS87_Ator08_hmg124h_7.JPG.dat	800.5	31.0	1.0	0.03	-0.093	0.033	8.6E-06
YJR053W	YJR053W	UWOPS87_Ator08_hmg124h_7.JPG.dat	733.5	26.1	1.0	0.04	-0.026	0.039	1.7E-05
YJR054W	YJR054W	UWOPS87_Ator08_hmg124h_7.JPG.dat	868.8	53.6	1.1	0.07	0.087	0.072	8.1E-05
YJR058C	YJR058C	UWOPS87_Ator08_hmg124h_7.JPG.dat	807.0	25.2	1.0	0.03	-0.018	0.033	9.1E-06
YJR059W	YJR059W	UWOPS87_Ator08_hmg124h_7.JPG.dat	876.3	72.7	1.1	0.09	0.048	0.086	1.4E-04
YJR060W	YJR060W	UWOPS87_Ator08_hmg124h_7.JPG.dat	737.8	19.1	0.9	0.03	-0.135	0.026	6.4E-06
YJR061W	YJR061W	UWOPS87_Ator08_hmg124h_7.JPG.dat	886.3	81.6	1.1	0.09	0.089	0.095	1.9E-04
YJR062C	YJR062C	UWOPS87_Ator08_hmg124h_7.JPG.dat	875.3	76.2	1.0	0.09	0.074	0.088	1.6E-04
YJR069C	YJR069C	UWOPS87_Ator08_hmg124h_7.JPG.dat	750.8	39.6	0.9	0.00	-0.085	0.003	3.9E-06
YJR070C	YJR070C	UWOPS87_Ator08_hmg124h_7.JPG.dat	829.3	44.9	1.1	0.07	-0.024	0.066	6.3E-05
YJR073C	YJR073C	UWOPS87_Ator08_hmg124h_7.JPG.dat	836.0	51.6	1.1	0.07	0.007	0.068	5.8E-05
YJR074W	YJR074W	UWOPS87_Ator08_hmg124h_7.JPG.dat	830.8	45.6	1.0	0.01	0.075	0.009	2.5E-05
YJR075W	YJR075W	UWOPS87_Ator08_hmg124h_7.JPG.dat	986.3	55.2	1.2	0.07	0.147	0.065	4.1E-05
YJR077C	YJR077C	UWOPS87_Ator08_hmg124h_7.JPG.dat	0.0	0.0	0.0	0.00	0.000	0.000	
YJR078W	YJR078W	UWOPS87_Ator08_hmg124h_7.JPG.dat	771.5	25.2	0.9	0.03	0.140	0.030	8.9E-06
YJR079W	YJR079W	UWOPS87_Ator08_hmg124h_7.JPG.dat	877.3	21.9	1.0	0.03	0.044	0.028	5.3E-06
YJR080C	YJR080C	UWOPS87_Ator08_hmg124h_7.JPG.dat	0.0	0.0	0.0	0.00	0.000	0.000	
YJR082C	YJR082C	UWOPS87_Ator08_hmg124h_7.JPG.dat	857.3	24.6	1.0	0.03	-0.065	0.029	6.7E-06
YJR083C	YJR083C	UWOPS87_Ator08_hmg124h_7.JPG.dat	709.8	240.0	0.9	0.07	-0.110	0.068	1.7E-03
YJR084W	YJR084W	UWOPS87_Ator08_hmg124h_7.JPG.dat	800.8	38.3	1.0	0.04	0.077	0.044	2.7E-05
YJR087W	YJR087W	UWOPS87_Ator08_hmg124h_7.JPG.dat	859.3	35.2	1.0	0.04	-0.307	0.041	1.8E-05
YJR088C	YJR088C	UWOPS87_Ator08_hmg124h_7.JPG.dat	721.3	92.5	0.9	0.12	-0.062	0.121	5.7E-04
YJR090C	YJR090C	UWOPS87_Ator08_hmg124h_7.JPG.dat	190.0	42.8	0.2	0.05	-0.070	0.053	3.1E-03
YJR091C	YJR091C	UWOPS87_Ator08_hmg124h_7.JPG.dat	823.0	26.5	1.0	0.03	0.015	0.031	7.5E-06
YJR092W	YJR092W	UWOPS87_Ator08_hmg124h_7.JPG.dat	859.8	47.9	1.0	0.06	0.074	0.059	5.3E-05
YJR094C	YJR094C	UWOPS87_Ator08_hmg124h_7.JPG.dat	852.8	19.6	1.0	0.02	0.018	0.023	3.0E-06
YJR094W-/	YJR094W-A	UWOPS87_Ator08_hmg124h_7.JPG.dat	889.5	19.7	1.1	0.03	0.140	0.026	4.1E-06
YJR095W	YJR095W	UWOPS87_Ator08_hmg124h_7.JPG.dat	883.5	53.8	1.0	0.06	-0.054	0.065	6.5E-05
YJR096W	YJR096W	UWOPS87_Ator08_hmg124h_7.JPG.dat	787.3	30.1	0.9	0.04	0.006	0.038	1.9E-05
YJR097W	YJR097W	UWOPS87_Ator08_hmg124h_7.JPG.dat	905.3	41.5	1.0	0.05	-0.103	0.050	3.1E-05
YJR098C	YJR098C	UWOPS87_Ator08_hmg124h_7.JPG.dat	877.5	23.2	1.1	0.03	-0.338	0.031	7.0E-06
YJR099W	YJR099W	UWOPS87_Ator08_hmg124h_7.JPG.dat	825.8	46.1	1.0	0.02	0.171	0.020	1.3E-04
YOR202W	YOR202W	UWOPS87_Ator08_hmg124h_7.JPG.dat	802.6	116.6	1.0	0.09	0.012	0.093	1.8E-283
1	1	UWOPS87_Ator08_hmg124h_8.JPG.dat	978.5	124.9	1.1	0.09	#NUM!		1.4E-04
2	2	UWOPS87_Ator08_hmg124h_8.JPG.dat	763.3	110.6	1.0	0.11	-0.192	0.111	4.1E-04
3	3	UWOPS87_Ator08_hmg124h_8.JPG.dat	780.5	79.3	1.0	0.06	-0.019	0.064	7.6E-05
4	4	UWOPS87_Ator08_hmg124h_8.JPG.dat	768.5	83.1	1.0	0.07	0.031	0.074	1.1E-04
YJR100C	YJR100C	UWOPS87_Ator08_hmg124h_8.JPG.dat	665.0	15.1	0.9	0.03	-0.008	0.030	9.1E-06
YJR103W	YJR103W	UWOPS87_Ator08_hmg124h_8.JPG.dat	171.8	343.5	0.0	0.00	0.000	0.000	
YJR106W	YJR106W	UWOPS87_Ator08_hmg124h_8.JPG.dat	758.3	32.3	1.0	0.05	0.015	0.052	3.7E-05
YJR107W	YJR107W	UWOPS87_Ator08_hmg124h_8.JPG.dat	803.8	43.2	1.0	0.05	-0.006	0.052	3.9E-05
YJR108W	YJR108W	UWOPS87_Ator08_hmg124h_8.JPG.dat	828.8	11.5	1.0	0.02	-0.073	0.017	1.3E-06
YJR109C	YJR109C	UWOPS87_Ator08_hmg124h_8.JPG.dat	831.3	116.7	0.9	0.03	-0.068	0.032	3.9E-04
YJR110W	YJR110W	UWOPS87_Ator08_hmg124h_8.JPG.dat	781.5	15.6	0.9	0.01	-0.039	0.011	4.7E-07
YJR111C	YJR111C	UWOPS87_Ator08_hmg124h_8.JPG.dat	918.8	53.5	1.1	0.07	0.119	0.067	6.6E-05
YJR115W	YJR115W	UWOPS87_Ator08_hmg124h_8.JPG.dat	882.8	55.1	1.0	0.06	0.012	0.064	6.5E-05
YJR116W	YJR116W	UWOPS87_Ator08_hmg124h_8.JPG.dat	742.5	61.0	0.9	0.07	-0.067	0.072	1.3E-04
YJR117W	YJR117W	UWOPS87_Ator08_hmg124h_8.JPG.dat	744.3	37.0	1.0	0.05	-0.069	0.046	2.7E-05
YJR118C	YJR118C	UWOPS87_Ator08_hmg124h_8.JPG.dat	656.0	29.2	1.0	0.05	0.130	0.052	4.3E-05
YJR119C	YJR119C	UWOPS87_Ator08_hmg124h_8.JPG.dat	717.3	20.2	1.0	0.02	0.006	0.022	2.7E-06

YJR120W	YJR120W	UWOPS87_Ator08_hmg124h_8.JPG.dat	0.0	0.0	0.0	0.00	0.000	0.000	
YJR121W	YJR121W	UWOPS87_Ator08_hmg124h_8.JPG.dat	816.3	80.8	1.0	0.11	-0.030	0.112	3.5E-04
YJR124C	YJR124C	UWOPS87_Ator08_hmg124h_8.JPG.dat	792.3	33.0	1.0	0.05	0.019	0.049	3.4E-05
YJR125C	YJR125C	UWOPS87_Ator08_hmg124h_8.JPG.dat	805.0	45.4	1.0	0.05	-0.037	0.047	3.0E-05
YJR126C	YJR126C	UWOPS87_Ator08_hmg124h_8.JPG.dat	806.3	36.1	1.0	0.04	0.072	0.039	1.8E-05
YJR127C	YJR127C	UWOPS87_Ator08_hmg124h_8.JPG.dat	754.0	15.3	0.9	0.01	-0.025	0.009	3.0E-07
YJR128W	YJR128W	UWOPS87_Ator08_hmg124h_8.JPG.dat	734.3	36.9	0.8	0.01	-0.242	0.007	2.3E-05
YJR129C	YJR129C	UWOPS87_Ator08_hmg124h_8.JPG.dat	942.8	27.3	1.2	0.02	0.107	0.025	2.5E-06
YJR130C	YJR130C	UWOPS87_Ator08_hmg124h_8.JPG.dat	693.5	60.8	1.0	0.09	-0.035	0.094	2.4E-04
YJR131W	YJR131W	UWOPS87_Ator08_hmg124h_8.JPG.dat	749.0	150.6	1.1	0.22	-0.084	0.222	2.3E-03
YJR133W	YJR133W	UWOPS87_Ator08_hmg124h_8.JPG.dat	921.0	93.0	1.2	0.12	0.104	0.119	2.5E-04
YJR134C	YJR134C	UWOPS87_Ator08_hmg124h_8.JPG.dat	831.0	76.7	1.1	0.10	0.044	0.099	2.2E-04
YJR135C	YJR135C	UWOPS87_Ator08_hmg124h_8.JPG.dat	834.0	72.1	1.0	0.08	0.037	0.083	1.4E-04
YJR137C	YJR137C	UWOPS87_Ator08_hmg124h_8.JPG.dat	804.3	36.7	1.0	0.04	0.017	0.040	1.8E-05
YJR139C	YJR139C	UWOPS87_Ator08_hmg124h_8.JPG.dat	799.0	38.5	1.0	0.04	0.180	0.045	2.7E-05
YJR140C	YJR140C	UWOPS87_Ator08_hmg124h_8.JPG.dat	673.3	449.0	1.1	0.02	0.147	0.018	9.5E-05
YJR142W	YJR142W	UWOPS87_Ator08_hmg124h_8.JPG.dat	871.0	13.0	1.0	0.01	-0.016	0.005	4.3E-08
YJR145C	YJR145C	UWOPS87_Ator08_hmg124h_8.JPG.dat	832.5	75.8	1.0	0.02	0.102	0.017	1.0E-04
YJR146W	YJR146W	UWOPS87_Ator08_hmg124h_8.JPG.dat	868.3	52.2	1.1	0.02	0.050	0.023	1.4E-04
YJR147W	YJR147W	UWOPS87_Ator08_hmg124h_8.JPG.dat	729.3	28.6	1.0	0.04	-0.016	0.039	1.6E-05
YJR148W	YJR148W	UWOPS87_Ator08_hmg124h_8.JPG.dat	644.5	19.5	0.9	0.02	-0.103	0.020	2.8E-06
YJR149W	YJR149W	UWOPS87_Ator08_hmg124h_8.JPG.dat	750.5	36.9	1.0	0.04	-0.051	0.041	2.1E-05
YJR150C	YJR150C	UWOPS87_Ator08_hmg124h_8.JPG.dat	892.8	39.7	1.1	0.02	0.011	0.022	1.4E-04
YJR152W	YJR152W	UWOPS87_Ator08_hmg124h_8.JPG.dat	772.0	70.2	0.9	0.08	-0.026	0.082	1.8E-04
YJR153W	YJR153W	UWOPS87_Ator08_hmg124h_8.JPG.dat	841.5	46.6	1.0	0.05	-0.063	0.052	3.8E-05
YJR154W	YJR154W	UWOPS87_Ator08_hmg124h_8.JPG.dat	214.8	429.5	0.0	0.00	0.000	0.000	
YKL001C	YKL001C	UWOPS87_Ator08_hmg124h_8.JPG.dat	0.0	0.0	0.0	0.00	0.000	0.000	
YKL005C	YKL005C	UWOPS87_Ator08_hmg124h_8.JPG.dat	908.5	51.2	1.0	0.06	-0.022	0.061	5.6E-05
YKL006W	YKL006W	UWOPS87_Ator08_hmg124h_8.JPG.dat	0.0	0.0	0.0	0.00	0.000	0.000	
YKL007W	YKL007W	UWOPS87_Ator08_hmg124h_8.JPG.dat	854.5	67.8	1.0	0.08	0.072	0.076	1.1E-04
YKL008C	YKL008C	UWOPS87_Ator08_hmg124h_8.JPG.dat	724.5	22.7	1.0	0.01	-0.091	0.006	1.3E-05
YKL009W	YKL009W	UWOPS87_Ator08_hmg124h_8.JPG.dat	669.5	29.5	0.9	0.03	0.031	0.033	1.2E-05
YKL010C	YKL010C	UWOPS87_Ator08_hmg124h_8.JPG.dat	808.5	55.9	1.0	0.07	0.024	0.070	8.6E-05
YKL015W	YKL015W	UWOPS87_Ator08_hmg124h_8.JPG.dat	821.5	93.4	1.0	0.12	-0.006	0.117	4.2E-04
YKL017C	YKL017C	UWOPS87_Ator08_hmg124h_8.JPG.dat	913.8	50.0	1.1	0.06	0.044	0.064	5.5E-05
YKL020C	YKL020C	UWOPS87_Ator08_hmg124h_8.JPG.dat	837.0	61.6	1.0	0.07	0.025	0.069	8.9E-05
YKL023W	YKL023W	UWOPS87_Ator08_hmg124h_8.JPG.dat	850.3	18.5	1.0	0.02	-0.060	0.022	2.9E-06
YKL025C	YKL025C	UWOPS87_Ator08_hmg124h_8.JPG.dat	798.0	67.8	0.9	0.08	-0.154	0.085	2.0E-04
YKL026C	YKL026C	UWOPS87_Ator08_hmg124h_8.JPG.dat	896.5	87.1	1.0	0.01	0.009	0.007	1.5E-05
YKL027W	YKL027W	UWOPS87_Ator08_hmg124h_8.JPG.dat	937.8	22.7	1.1	0.01	0.099	0.006	1.1E-05
YKL029C	YKL029C	UWOPS87_Ator08_hmg124h_8.JPG.dat	0.0	0.0	0.0	0.00	0.000	0.000	
YKL030W	YKL030W	UWOPS87_Ator08_hmg124h_8.JPG.dat	805.3	106.3	1.0	0.03	-0.076	0.035	4.0E-04
YKL031W	YKL031W	UWOPS87_Ator08_hmg124h_8.JPG.dat	893.5	91.8	1.2	0.13	0.000	0.132	3.3E-04
YKL032C	YKL032C	UWOPS87_Ator08_hmg124h_8.JPG.dat	911.0	66.0	1.2	0.08	0.071	0.078	8.5E-05
YKL033W-/	YKL033W-A	UWOPS87_Ator08_hmg124h_8.JPG.dat	579.8	391.0	1.0	0.09	-0.004	0.091	2.9E-03
YKL034W	YKL034W	UWOPS87_Ator08_hmg124h_8.JPG.dat	841.3	11.8	1.0	0.02	0.009	0.019	1.8E-06
YKL037W	YKL037W	UWOPS87_Ator08_hmg124h_8.JPG.dat	784.0	20.1	1.0	0.03	-0.023	0.030	8.1E-06
YKL038W	YKL038W	UWOPS87_Ator08_hmg124h_8.JPG.dat	775.3	122.6	1.0	0.04	0.005	0.044	6.1E-04
YKL039W	YKL039W	UWOPS87_Ator08_hmg124h_8.JPG.dat	807.0	27.9	1.0	0.03	-0.058	0.035	1.3E-05
YKL040C	YKL040C	UWOPS87_Ator08_hmg124h_8.JPG.dat	901.5	13.8	1.1	0.00	-0.010	0.005	6.4E-06
YKL041W	YKL041W	UWOPS87_Ator08_hmg124h_8.JPG.dat	935.0	43.3	1.1	0.01	0.059	0.007	1.1E-05
YKL043W	YKL043W	UWOPS87_Ator08_hmg124h_8.JPG.dat	933.5	50.7	1.2	0.02	0.129	0.024	1.4E-04
YKL044W	YKL044W	UWOPS87_Ator08_hmg124h_8.JPG.dat	701.8	12.3	0.9	0.02	-0.016	0.022	3.6E-06
YKL046C	YKL046C	UWOPS87_Ator08_hmg124h_8.JPG.dat	785.5	15.6	1.1	0.03	0.026	0.028	5.1E-06
YKL047W	YKL047W	UWOPS87_Ator08_hmg124h_8.JPG.dat	756.0	113.7	1.0	0.15	0.012	0.151	9.2E-04
YKL048C	YKL048C	UWOPS87_Ator08_hmg124h_8.JPG.dat	756.0	37.3	1.0	0.05	-0.023	0.048	3.0E-05
YKL050C	YKL050C	UWOPS87_Ator08_hmg124h_8.JPG.dat	819.3	71.6	1.1	0.10	-0.024	0.096	2.0E-04
YKL051W	YKL051W	UWOPS87_Ator08_hmg124h_8.JPG.dat	743.0	62.9	1.0	0.08	-0.083	0.080	1.6E-04
YKL053C-A	YKL053C-A	UWOPS87_Ator08_hmg124h_8.JPG.dat	876.3	31.2	1.1	0.04	0.025	0.039	1.2E-05
YKL053W	YKL053W	UWOPS87_Ator08_hmg124h_8.JPG.dat	783.0	45.2	1.0	0.06	-0.013	0.055	4.8E-05
YKL055C	YKL055C	UWOPS87_Ator08_hmg124h_8.JPG.dat	869.3	66.7	1.1	0.08	-0.008	0.075	8.8E-05
YKL056C	YKL056C	UWOPS87_Ator08_hmg124h_8.JPG.dat	816.3	30.9	1.0	0.04	0.071	0.036	1.2E-05
YKL061W	YKL061W	UWOPS87_Ator08_hmg124h_8.JPG.dat	758.3	42.8	1.0	0.06	0.039	0.057	5.3E-05
YKL062W	YKL062W	UWOPS87_Ator08_hmg124h_8.JPG.dat	765.0	63.6	1.0	0.03	-0.005	0.027	2.4E-04
YKL063C	YKL063C	UWOPS87_Ator08_hmg124h_8.JPG.dat	709.3	42.7	1.0	0.02	0.118	0.022	1.4E-04
YKL064W	YKL064W	UWOPS87_Ator08_hmg124h_8.JPG.dat	716.3	21.8	1.0	0.03	-0.001	0.028	6.2E-06
YKL065C	YKL065C	UWOPS87_Ator08_hmg124h_8.JPG.dat	0.0	0.0	0.0	0.00	0.000	0.000	
YKL066W	YKL066W	UWOPS87_Ator08_hmg124h_8.JPG.dat	866.8	39.6	1.1	0.04	0.041	0.042	1.5E-05
YKL067W	YKL067W	UWOPS87_Ator08_hmg124h_8.JPG.dat	826.0	44.8	1.0	0.06	0.037	0.056	4.8E-05
YKL068W	YKL068W	UWOPS87_Ator08_hmg124h_8.JPG.dat	819.0	13.7	1.0	0.01	0.044	0.005	9.7E-06

YKL069W	YKL069W	UWOPS87_Ator08_hmg124h_8.JPG.dat	769.8	101.1	1.0	0.04	0.017	0.041	5.8E-04
YKL070W	YKL070W	UWOPS87_Ator08_hmg124h_8.JPG.dat	873.5	38.3	1.0	0.04	-0.022	0.042	1.9E-05
YKL071W	YKL071W	UWOPS87_Ator08_hmg124h_8.JPG.dat	983.3	53.2	1.1	0.01	0.025	0.014	5.5E-05
YKL072W	YKL072W	UWOPS87_Ator08_hmg124h_8.JPG.dat	830.0	16.3	1.0	0.01	-0.056	0.013	6.1E-07
YKL073W	YKL073W	UWOPS87_Ator08_hmg124h_8.JPG.dat	797.8	72.4	1.0	0.08	-0.023	0.084	1.5E-04
YKL074C	YKL074C	UWOPS87_Ator08_hmg124h_8.JPG.dat	500.5	335.3	1.0	0.05	0.038	0.050	8.4E-04
YKL075C	YKL075C	UWOPS87_Ator08_hmg124h_8.JPG.dat	752.8	26.8	1.1	0.05	0.068	0.051	2.9E-05
YKL076C	YKL076C	UWOPS87_Ator08_hmg124h_8.JPG.dat	381.5	440.8	0.5	0.61	0.036	0.607	1.8E-01
YKL077W	YKL077W	UWOPS87_Ator08_hmg124h_8.JPG.dat	738.5	55.6	0.9	0.02	-0.029	0.021	1.8E-04
YKL079W	YKL079W	UWOPS87_Ator08_hmg124h_8.JPG.dat	817.5	28.5	1.0	0.01	-0.047	0.007	1.5E-05
YKL081W	YKL081W	UWOPS87_Ator08_hmg124h_8.JPG.dat	815.8	26.8	1.0	0.03	0.022	0.031	7.7E-06
YKL084W	YKL084W	UWOPS87_Ator08_hmg124h_8.JPG.dat	846.5	44.0	1.0	0.05	0.035	0.047	2.5E-05
YKL085W	YKL085W	UWOPS87_Ator08_hmg124h_8.JPG.dat	850.3	43.1	1.0	0.01	-0.007	0.014	6.9E-05
YKL086W	YKL086W	UWOPS87_Ator08_hmg124h_8.JPG.dat	919.5	21.3	1.1	0.01	0.023	0.008	1.9E-05
YKL090W	YKL090W	UWOPS87_Ator08_hmg124h_8.JPG.dat	809.5	89.2	1.0	0.10	-0.066	0.102	3.0E-04
YKL091C	YKL091C	UWOPS87_Ator08_hmg124h_8.JPG.dat	781.3	91.1	1.0	0.12	0.014	0.120	4.3E-04
YKL092C	YKL092C	UWOPS87_Ator08_hmg124h_8.JPG.dat	741.5	12.1	1.1	0.02	0.109	0.015	8.2E-07
YKL093W	YKL093W	UWOPS87_Ator08_hmg124h_8.JPG.dat	675.0	40.1	0.9	0.05	-0.016	0.051	4.6E-05
YKL094W	YKL094W	UWOPS87_Ator08_hmg124h_8.JPG.dat	696.5	42.3	0.9	0.05	0.029	0.053	5.3E-05
YKL096W	YKL096W	UWOPS87_Ator08_hmg124h_8.JPG.dat	837.3	6.6	1.0	0.01	-0.061	0.009	1.6E-07
YKL096W-/	YKL096W-A	UWOPS87_Ator08_hmg124h_8.JPG.dat	856.0	39.4	1.0	0.02	-0.036	0.017	8.9E-05
YKL097C	YKL097C	UWOPS87_Ator08_hmg124h_8.JPG.dat	799.3	14.1	1.0	0.00	-0.048	0.003	3.3E-06
YKL098W	YKL098W	UWOPS87_Ator08_hmg124h_8.JPG.dat	1075.8	32.2	1.3	0.05	0.241	0.045	1.2E-05
YKL100C	YKL100C	UWOPS87_Ator08_hmg124h_8.JPG.dat	798.0	31.3	1.0	0.01	0.022	0.013	6.2E-05
YKL101W	YKL101W	UWOPS87_Ator08_hmg124h_8.JPG.dat	841.3	97.2	1.0	0.11	-0.079	0.112	4.4E-04
YKL102C	YKL102C	UWOPS87_Ator08_hmg124h_8.JPG.dat	811.5	51.4	1.0	0.05	-0.013	0.049	3.6E-05
YKL103C	YKL103C	UWOPS87_Ator08_hmg124h_8.JPG.dat	849.3	226.8	0.9	0.07	-0.024	0.072	1.9E-03
YKL105C	YKL105C	UWOPS87_Ator08_hmg124h_8.JPG.dat	710.8	19.0	1.0	0.03	-0.034	0.032	8.8E-06
YKL106W	YKL106W	UWOPS87_Ator08_hmg124h_8.JPG.dat	218.5	437.0	0.0	0.00	0.000	0.000	
YKL107W	YKL107W	UWOPS87_Ator08_hmg124h_8.JPG.dat	735.5	24.4	0.9	0.03	-0.031	0.034	1.3E-05
YKL109W	YKL109W	UWOPS87_Ator08_hmg124h_8.JPG.dat	675.0	452.4	1.1	0.07	-0.055	0.073	1.5E-03
YKL110C	YKL110C	UWOPS87_Ator08_hmg124h_8.JPG.dat	831.3	54.2	1.0	0.02	0.022	0.018	1.2E-04
YKL113C	YKL113C	UWOPS87_Ator08_hmg124h_8.JPG.dat	915.3	51.3	1.1	0.02	0.119	0.020	1.2E-04
YKL114C	YKL114C	UWOPS87_Ator08_hmg124h_8.JPG.dat	856.5	56.8	1.0	0.06	0.040	0.064	6.6E-05
YKL115C	YKL115C	UWOPS87_Ator08_hmg124h_8.JPG.dat	844.3	55.0	1.0	0.07	0.059	0.072	1.1E-04
YKL116C	YKL116C	UWOPS87_Ator08_hmg124h_8.JPG.dat	937.8	58.7	1.0	0.06	-0.009	0.063	5.9E-05
YKL117W	YKL117W	UWOPS87_Ator08_hmg124h_8.JPG.dat	877.0	88.4	1.0	0.09	0.025	0.090	1.9E-04
YKL120W	YKL120W	UWOPS87_Ator08_hmg124h_8.JPG.dat	737.5	38.3	0.9	0.05	-0.054	0.054	5.5E-05
YKL121W	YKL121W	UWOPS87_Ator08_hmg124h_8.JPG.dat	675.3	35.2	0.9	0.01	-0.017	0.007	1.8E-05
YKL123W	YKL123W	UWOPS87_Ator08_hmg124h_8.JPG.dat	724.3	45.2	1.0	0.04	0.027	0.045	2.8E-05
YKL124W	YKL124W	UWOPS87_Ator08_hmg124h_8.JPG.dat	744.3	88.3	0.9	0.11	-0.087	0.115	5.0E-04
YKL127W	YKL127W	UWOPS87_Ator08_hmg124h_8.JPG.dat	905.0	12.4	1.1	0.00	0.035	0.002	6.8E-07
YKL128C	YKL128C	UWOPS87_Ator08_hmg124h_8.JPG.dat	861.5	27.7	1.0	0.03	-0.008	0.035	1.1E-05
YKL129C	YKL129C	UWOPS87_Ator08_hmg124h_8.JPG.dat	831.3	28.3	1.0	0.04	-0.041	0.036	1.2E-05
YKL130C	YKL130C	UWOPS87_Ator08_hmg124h_8.JPG.dat	0.0	0.0	0.0	0.00	0.000	0.000	
YKL131W	YKL131W	UWOPS87_Ator08_hmg124h_8.JPG.dat	901.0	55.3	1.0	0.02	-0.017	0.016	7.9E-05
YKL132C	YKL132C	UWOPS87_Ator08_hmg124h_8.JPG.dat	800.0	38.5	0.9	0.04	-0.020	0.045	3.4E-05
YKL133C	YKL133C	UWOPS87_Ator08_hmg124h_8.JPG.dat	882.3	49.8	1.1	0.01	-0.005	0.014	6.1E-05
YKL136W	YKL136W	UWOPS87_Ator08_hmg124h_8.JPG.dat	804.3	162.0	1.0	0.20	-0.035	0.199	2.0E-03
YKL137W	YKL137W	UWOPS87_Ator08_hmg124h_8.JPG.dat	734.5	18.2	1.0	0.03	0.107	0.031	6.9E-06
YKL139W	YKL139W	UWOPS87_Ator08_hmg124h_8.JPG.dat	747.3	36.4	1.0	0.03	0.060	0.033	1.0E-05
YKL140W	YKL140W	UWOPS87_Ator08_hmg124h_8.JPG.dat	905.8	21.4	1.2	0.02	0.074	0.021	1.7E-06
YKL142W	YKL142W	UWOPS87_Ator08_hmg124h_8.JPG.dat	786.5	42.7	1.0	0.06	-0.046	0.056	5.2E-05
YKL146W	YKL146W	UWOPS87_Ator08_hmg124h_8.JPG.dat	776.0	61.9	0.9	0.07	-0.052	0.073	1.2E-04
YKL147C	YKL147C	UWOPS87_Ator08_hmg124h_8.JPG.dat	750.3	58.9	0.9	0.01	-0.072	0.012	6.2E-05
YKL148C	YKL148C	UWOPS87_Ator08_hmg124h_8.JPG.dat	840.0	21.6	1.0	0.01	0.017	0.008	1.9E-05
YKL149C	YKL149C	UWOPS87_Ator08_hmg124h_8.JPG.dat	851.8	50.2	1.0	0.01	-0.037	0.009	3.1E-05
YKL150W	YKL150W	UWOPS87_Ator08_hmg124h_8.JPG.dat	809.5	62.4	0.9	0.07	-0.045	0.068	1.0E-04
YKL151C	YKL151C	UWOPS87_Ator08_hmg124h_8.JPG.dat	719.0	64.3	0.9	0.08	-0.125	0.085	2.4E-04
YKL156W	YKL156W	UWOPS87_Ator08_hmg124h_8.JPG.dat	765.0	21.9	1.0	0.03	-0.004	0.033	1.0E-05
YKL157W	YKL157W	UWOPS87_Ator08_hmg124h_8.JPG.dat	695.5	14.5	1.0	0.01	-0.049	0.009	2.9E-05
YKL158W	YKL158W	UWOPS87_Ator08_hmg124h_8.JPG.dat	702.5	25.3	0.9	0.04	-0.040	0.038	1.8E-05
YKL159C	YKL159C	UWOPS87_Ator08_hmg124h_8.JPG.dat	719.0	26.3	1.0	0.04	-0.071	0.039	1.8E-05
YKL160W	YKL160W	UWOPS87_Ator08_hmg124h_8.JPG.dat	801.3	53.5	1.0	0.07	0.117	0.074	9.8E-05
YKL161C	YKL161C	UWOPS87_Ator08_hmg124h_8.JPG.dat	771.5	29.6	1.0	0.04	-0.009	0.039	1.6E-05
YKL162C	YKL162C	UWOPS87_Ator08_hmg124h_8.JPG.dat	712.5	41.7	0.9	0.06	0.017	0.057	6.6E-05
YKL163W	YKL163W	UWOPS87_Ator08_hmg124h_8.JPG.dat	725.8	18.9	0.9	0.02	0.003	0.025	5.5E-06
YKL164C	YKL164C	UWOPS87_Ator08_hmg124h_8.JPG.dat	827.8	34.1	1.0	0.04	-0.009	0.039	1.5E-05
YKL166C	YKL166C	UWOPS87_Ator08_hmg124h_8.JPG.dat	760.0	31.7	0.9	0.04	-0.035	0.036	1.6E-05
YKL167C	YKL167C	UWOPS87_Ator08_hmg124h_8.JPG.dat	758.3	59.9	0.9	0.02	-0.094	0.020	1.5E-04

YKL168C	YKL168C	UWOPS87_Ator08_hmg124h_8.JPG.dat	651.0	443.1	1.2	0.15	0.141	0.152	5.8E-03
YKL171W	YKL171W	UWOPS87_Ator08_hmg124h_8.JPG.dat	763.5	37.4	1.0	0.02	0.031	0.019	1.1E-04
YKL174C	YKL174C	UWOPS87_Ator08_hmg124h_8.JPG.dat	698.3	24.1	1.0	0.03	-0.060	0.034	1.3E-05
YKL175W	YKL175W	UWOPS87_Ator08_hmg124h_8.JPG.dat	694.8	20.1	0.9	0.00	0.003	0.003	4.5E-06
YKL176C	YKL176C	UWOPS87_Ator08_hmg124h_8.JPG.dat	726.5	30.8	0.9	0.04	-0.002	0.045	3.1E-05
YKL177W	YKL177W	UWOPS87_Ator08_hmg124h_8.JPG.dat	845.5	45.1	1.1	0.05	0.038	0.054	3.7E-05
YKL178C	YKL178C	UWOPS87_Ator08_hmg124h_8.JPG.dat	826.5	49.5	1.0	0.07	0.026	0.066	7.4E-05
YKL179C	YKL179C	UWOPS87_Ator08_hmg124h_8.JPG.dat	892.8	20.5	1.1	0.03	-0.056	0.026	4.0E-06
YKL183W	YKL183W	UWOPS87_Ator08_hmg124h_8.JPG.dat	691.5	65.0	0.8	0.08	-0.070	0.076	2.1E-04
YKL184W	YKL184W	UWOPS87_Ator08_hmg124h_8.JPG.dat	713.3	112.1	0.8	0.04	-0.035	0.040	8.4E-04
YKL185W	YKL185W	UWOPS87_Ator08_hmg124h_8.JPG.dat	811.3	114.9	1.0	0.14	0.004	0.142	7.3E-04
YKL187C	YKL187C	UWOPS87_Ator08_hmg124h_8.JPG.dat	732.8	34.4	1.0	0.04	-0.126	0.037	1.4E-05
YKL188C	YKL188C	UWOPS87_Ator08_hmg124h_8.JPG.dat	683.8	43.3	1.0	0.06	0.034	0.059	5.5E-05
YKL190W	YKL190W	UWOPS87_Ator08_hmg124h_8.JPG.dat	343.0	231.7	0.6	0.06	-0.353	0.063	3.2E-03
YKL191W	YKL191W	UWOPS87_Ator08_hmg124h_8.JPG.dat	651.0	103.3	0.8	0.04	-0.227	0.044	9.6E-04
YKL197C	YKL197C	UWOPS87_Ator08_hmg124h_8.JPG.dat	910.0	32.5	1.1	0.05	0.063	0.046	1.7E-05
YKL198C	YKL198C	UWOPS87_Ator08_hmg124h_8.JPG.dat	598.5	369.8	1.0	0.05	-0.067	0.048	8.0E-04
YKL199C	YKL199C	UWOPS87_Ator08_hmg124h_8.JPG.dat	773.3	17.9	1.0	0.02	-0.009	0.024	4.3E-06
YKL200C	YKL200C	UWOPS87_Ator08_hmg124h_8.JPG.dat	884.8	25.1	1.1	0.03	-0.030	0.027	4.5E-06
YKL201C	YKL201C	UWOPS87_Ator08_hmg124h_8.JPG.dat	813.5	48.3	1.0	0.06	0.007	0.058	5.8E-05
YKL202W	YKL202W	UWOPS87_Ator08_hmg124h_8.JPG.dat	764.5	66.6	0.9	0.08	-0.039	0.077	1.7E-04
YKL204W	YKL204W	UWOPS87_Ator08_hmg124h_8.JPG.dat	701.0	128.4	0.9	0.16	-0.159	0.163	1.7E-03
YKL205W	YKL205W	UWOPS87_Ator08_hmg124h_8.JPG.dat	750.5	28.0	1.0	0.01	0.081	0.010	2.9E-05
YKL206C	YKL206C	UWOPS87_Ator08_hmg124h_8.JPG.dat	792.8	51.9	1.2	0.02	0.057	0.016	6.1E-05
YKL207W	YKL207W	UWOPS87_Ator08_hmg124h_8.JPG.dat	603.0	16.1	0.8	0.02	-0.252	0.019	3.5E-06
YKL208W	YKL208W	UWOPS87_Ator08_hmg124h_8.JPG.dat	1014.0	63.2	1.3	0.08	0.252	0.076	5.0E-05
YKL211C	YKL211C	UWOPS87_Ator08_hmg124h_8.JPG.dat	861.0	42.4	1.1	0.06	0.028	0.058	4.2E-05
YKL212W	YKL212W	UWOPS87_Ator08_hmg124h_8.JPG.dat	818.3	21.7	1.0	0.02	0.051	0.021	2.5E-06
YKL213C	YKL213C	UWOPS87_Ator08_hmg124h_8.JPG.dat	833.8	39.6	1.1	0.01	0.060	0.014	5.8E-05
YKL214C	YKL214C	UWOPS87_Ator08_hmg124h_8.JPG.dat	764.8	12.7	0.9	0.01	0.009	0.010	3.5E-07
YKL215C	YKL215C	UWOPS87_Ator08_hmg124h_8.JPG.dat	662.8	31.9	0.8	0.03	-0.082	0.030	1.6E-05
YKL216W	YKL216W	UWOPS87_Ator08_hmg124h_8.JPG.dat	876.3	93.2	1.0	0.10	-0.022	0.102	2.7E-04
YKL217W	YKL217W	UWOPS87_Ator08_hmg124h_8.JPG.dat	774.5	101.1	0.9	0.02	-0.085	0.018	1.4E-04
YKL218C	YKL218C	UWOPS87_Ator08_hmg124h_8.JPG.dat	689.3	33.2	1.0	0.04	0.024	0.043	2.4E-05
YKL220C	YKL220C	UWOPS87_Ator08_hmg124h_8.JPG.dat	701.0	23.8	1.0	0.04	-0.057	0.038	1.6E-05
YKL221W	YKL221W	UWOPS87_Ator08_hmg124h_8.JPG.dat	822.8	79.9	1.1	0.03	0.084	0.031	2.6E-04
YKL222C	YKL222C	UWOPS87_Ator08_hmg124h_8.JPG.dat	855.8	57.5	1.1	0.07	0.071	0.071	7.8E-05
YKR001C	YKR001C	UWOPS87_Ator08_hmg124h_8.JPG.dat	815.8	50.7	1.0	0.06	-0.029	0.059	5.8E-05
YKR003W	YKR003W	UWOPS87_Ator08_hmg124h_8.JPG.dat	780.5	6.9	0.9	0.01	-0.020	0.008	1.5E-07
YKR005C	YKR005C	UWOPS87_Ator08_hmg124h_8.JPG.dat	860.3	34.5	1.0	0.04	0.009	0.041	1.8E-05
YKR007W	YKR007W	UWOPS87_Ator08_hmg124h_8.JPG.dat	826.5	27.9	1.0	0.00	-0.064	0.002	2.0E-06
YKR009C	YKR009C	UWOPS87_Ator08_hmg124h_8.JPG.dat	875.0	39.6	1.0	0.04	-0.024	0.035	1.2E-05
YKR010C	YKR010C	UWOPS87_Ator08_hmg124h_8.JPG.dat	872.3	27.8	1.0	0.00	0.050	0.003	2.8E-06
YKR011C	YKR011C	UWOPS87_Ator08_hmg124h_8.JPG.dat	925.3	31.2	1.1	0.05	0.034	0.046	1.9E-05
YKR012C	YKR012C	UWOPS87_Ator08_hmg124h_8.JPG.dat	746.5	43.8	1.0	0.07	0.084	0.069	8.7E-05
YKR013W	YKR013W	UWOPS87_Ator08_hmg124h_8.JPG.dat	694.8	36.4	1.0	0.01	-0.089	0.015	7.7E-05
YKR014C	YKR014C	UWOPS87_Ator08_hmg124h_8.JPG.dat	867.3	42.7	1.1	0.00	0.066	0.001	1.0E-07
YKR015C	YKR015C	UWOPS87_Ator08_hmg124h_8.JPG.dat	773.8	29.4	1.0	0.04	-0.051	0.043	2.4E-05
YKR016W	YKR016W	UWOPS87_Ator08_hmg124h_8.JPG.dat	667.5	39.3	0.8	0.01	-0.155	0.007	2.5E-05
YKR017C	YKR017C	UWOPS87_Ator08_hmg124h_8.JPG.dat	784.8	52.4	1.0	0.06	0.031	0.060	6.6E-05
YKR018C	YKR018C	UWOPS87_Ator08_hmg124h_8.JPG.dat	803.5	30.2	1.0	0.04	-0.031	0.040	1.9E-05
YKR019C	YKR019C	UWOPS87_Ator08_hmg124h_8.JPG.dat	899.0	47.5	1.1	0.05	0.041	0.048	2.4E-05
YKR020W	YKR020W	UWOPS87_Ator08_hmg124h_8.JPG.dat	212.0	424.0	0.0	0.00	0.000	0.000	
YKR021W	YKR021W	UWOPS87_Ator08_hmg124h_8.JPG.dat	931.3	67.3	1.1	0.07	-0.011	0.069	7.3E-05
YKR023W	YKR023W	UWOPS87_Ator08_hmg124h_8.JPG.dat	766.3	30.0	0.9	0.04	-0.058	0.039	1.9E-05
YKR024C	YKR024C	UWOPS87_Ator08_hmg124h_8.JPG.dat	788.8	66.7	1.1	0.08	0.169	0.080	1.2E-04
YKR026C	YKR026C	UWOPS87_Ator08_hmg124h_8.JPG.dat	689.3	45.4	1.0	0.06	-0.006	0.060	6.7E-05
YKR027W	YKR027W	UWOPS87_Ator08_hmg124h_8.JPG.dat	866.3	33.9	1.1	0.03	0.073	0.032	6.8E-06
YKR028W	YKR028W	UWOPS87_Ator08_hmg124h_8.JPG.dat	602.0	52.3	0.8	0.06	-0.149	0.064	1.7E-04
YKR029C	YKR029C	UWOPS87_Ator08_hmg124h_8.JPG.dat	737.0	13.9	0.9	0.02	-0.149	0.018	2.1E-06
YKR030W	YKR030W	UWOPS87_Ator08_hmg124h_8.JPG.dat	872.8	10.3	1.1	0.02	0.001	0.024	3.2E-06
YKR031C	YKR031C	UWOPS87_Ator08_hmg124h_8.JPG.dat	793.0	40.6	1.0	0.06	0.006	0.057	5.6E-05
YKR032W	YKR032W	UWOPS87_Ator08_hmg124h_8.JPG.dat	766.0	185.3	1.0	0.01	-0.020	0.014	6.6E-05
YKR033C	YKR033C	UWOPS87_Ator08_hmg124h_8.JPG.dat	796.5	211.3	0.9	0.25	0.003	0.248	4.9E-03
YKR034W	YKR034W	UWOPS87_Ator08_hmg124h_8.JPG.dat	806.5	27.7	0.9	0.03	-0.044	0.030	8.9E-06
YKR035C	YKR035C	UWOPS87_Ator08_hmg124h_8.JPG.dat	839.5	35.8	1.1	0.05	0.083	0.051	3.1E-05
YKR035W-	YKR035W-A	UWOPS87_Ator08_hmg124h_8.JPG.dat	748.8	48.4	1.0	0.07	0.079	0.067	8.0E-05
YKR036C	YKR036C	UWOPS87_Ator08_hmg124h_8.JPG.dat	664.3	21.5	0.9	0.03	-0.052	0.031	1.1E-05
YKR039W	YKR039W	UWOPS87_Ator08_hmg124h_8.JPG.dat	731.5	71.9	0.9	0.09	-0.024	0.093	2.7E-04
YKR040C	YKR040C	UWOPS87_Ator08_hmg124h_8.JPG.dat	811.8	47.4	1.0	0.06	-0.008	0.064	6.6E-05

YKR041W	YKR041W	UWOPS87_Ator08_hmg124h_8.JPG.dat	748.0	14.4	0.9	0.01	-0.026	0.013	8.1E-07
YKR042W	YKR042W	UWOPS87_Ator08_hmg124h_8.JPG.dat	793.5	30.3	1.0	0.03	-0.052	0.034	1.1E-05
YKR043C	YKR043C	UWOPS87_Ator08_hmg124h_8.JPG.dat	819.0	49.9	1.0	0.06	-0.038	0.058	5.4E-05
YKR044W	YKR044W	UWOPS87_Ator08_hmg124h_8.JPG.dat	858.5	34.8	1.0	0.05	0.038	0.052	3.4E-05
YKR045C	YKR045C	UWOPS87_Ator08_hmg124h_8.JPG.dat	914.0	60.0	1.1	0.07	-0.009	0.072	7.8E-05
YKR046C	YKR046C	UWOPS87_Ator08_hmg124h_8.JPG.dat	808.0	110.4	1.0	0.13	-0.063	0.133	6.8E-04
YKR047W	YKR047W	UWOPS87_Ator08_hmg124h_8.JPG.dat	702.0	34.3	0.9	0.04	-0.027	0.040	2.4E-05
YKR048C	YKR048C	UWOPS87_Ator08_hmg124h_8.JPG.dat	708.5	31.1	0.9	0.04	-0.026	0.041	2.2E-05
YKR049C	YKR049C	UWOPS87_Ator08_hmg124h_8.JPG.dat	737.5	13.0	1.0	0.01	-0.004	0.012	4.1E-07
YKR050W	YKR050W	UWOPS87_Ator08_hmg124h_8.JPG.dat	712.0	32.9	1.0	0.04	0.035	0.044	2.4E-05
YKR051W	YKR051W	UWOPS87_Ator08_hmg124h_8.JPG.dat	812.3	29.6	1.1	0.01	-0.046	0.008	1.8E-05
YKR052C	YKR052C	UWOPS87_Ator08_hmg124h_8.JPG.dat	795.3	64.0	1.0	0.07	0.089	0.075	1.1E-04
YKR053C	YKR053C	UWOPS87_Ator08_hmg124h_8.JPG.dat	879.0	47.5	1.1	0.06	0.018	0.057	3.8E-05
YKR054C	YKR054C	UWOPS87_Ator08_hmg124h_8.JPG.dat	646.8	114.9	0.8	0.14	0.366	0.141	1.4E-03
YKR055W	YKR055W	UWOPS87_Ator08_hmg124h_8.JPG.dat	917.8	27.5	1.1	0.04	0.047	0.036	9.3E-06
YKR056W	YKR056W	UWOPS87_Ator08_hmg124h_8.JPG.dat	866.5	57.4	1.0	0.07	0.015	0.072	9.3E-05
YKR057W	YKR057W	UWOPS87_Ator08_hmg124h_8.JPG.dat	666.8	37.8	0.8	0.05	0.060	0.045	5.2E-05
YKR058W	YKR058W	UWOPS87_Ator08_hmg124h_8.JPG.dat	873.3	102.6	1.1	0.11	-0.016	0.115	3.4E-04
YKR059W	YKR059W	UWOPS87_Ator08_hmg124h_8.JPG.dat	673.8	95.1	0.9	0.12	0.049	0.120	7.0E-04
YKR060W	YKR060W	UWOPS87_Ator08_hmg124h_8.JPG.dat	669.8	26.4	1.0	0.04	-0.026	0.043	2.2E-05
YKR061W	YKR061W	UWOPS87_Ator08_hmg124h_8.JPG.dat	689.8	28.2	1.0	0.04	0.041	0.041	2.0E-05
YKR064W	YKR064W	UWOPS87_Ator08_hmg124h_8.JPG.dat	0.0	0.0	0.0	0.00	0.000	0.000	
YKR065C	YKR065C	UWOPS87_Ator08_hmg124h_8.JPG.dat	743.5	20.3	1.0	0.02	-0.032	0.016	1.2E-06
YKR066C	YKR066C	UWOPS87_Ator08_hmg124h_8.JPG.dat	857.8	42.7	1.1	0.01	0.022	0.005	7.7E-06
YKR067W	YKR067W	UWOPS87_Ator08_hmg124h_8.JPG.dat	819.8	21.1	1.0	0.03	-0.008	0.025	4.2E-06
YKR069W	YKR069W	UWOPS87_Ator08_hmg124h_8.JPG.dat	805.8	9.2	1.0	0.02	0.005	0.015	9.5E-07
YKR070W	YKR070W	UWOPS87_Ator08_hmg124h_8.JPG.dat	785.3	29.1	0.9	0.03	-0.086	0.033	1.2E-05
YKR072C	YKR072C	UWOPS87_Ator08_hmg124h_8.JPG.dat	929.3	18.8	1.1	0.02	0.094	0.021	2.1E-06
YKR074W	YKR074W	UWOPS87_Ator08_hmg124h_8.JPG.dat	946.5	118.8	1.2	0.14	0.232	0.144	5.2E-04
YKR076W	YKR076W	UWOPS87_Ator08_hmg124h_8.JPG.dat	788.8	105.8	1.0	0.16	-0.027	0.157	9.3E-04
YKR077W	YKR077W	UWOPS87_Ator08_hmg124h_8.JPG.dat	683.0	35.6	1.0	0.05	0.017	0.054	4.3E-05
YKR078W	YKR078W	UWOPS87_Ator08_hmg124h_8.JPG.dat	731.0	2.6	1.0	0.02	0.065	0.019	1.6E-06
YKR080W	YKR080W	UWOPS87_Ator08_hmg124h_8.JPG.dat	833.5	166.9	1.0	0.04	0.033	0.037	4.3E-04
YKR082W	YKR082W	UWOPS87_Ator08_hmg124h_8.JPG.dat	745.0	111.1	0.9	0.15	0.184	0.150	1.1E-03
YKR084C	YKR084C	UWOPS87_Ator08_hmg124h_8.JPG.dat	715.5	27.3	0.9	0.03	0.026	0.035	1.7E-05
YKR087C	YKR087C	UWOPS87_Ator08_hmg124h_8.JPG.dat	832.8	38.0	1.0	0.04	-0.026	0.044	2.1E-05
YKR088C	YKR088C	UWOPS87_Ator08_hmg124h_8.JPG.dat	897.8	25.5	1.1	0.03	0.052	0.026	3.5E-06
YKR089C	YKR089C	UWOPS87_Ator08_hmg124h_8.JPG.dat	884.3	43.0	1.1	0.05	-0.007	0.050	2.9E-05
YKR090W	YKR090W	UWOPS87_Ator08_hmg124h_8.JPG.dat	918.0	34.5	1.1	0.05	-0.008	0.046	2.2E-05
YKR091W	YKR091W	UWOPS87_Ator08_hmg124h_8.JPG.dat	858.8	96.0	1.0	0.10	0.052	0.102	2.6E-04
YKR092C	YKR092C	UWOPS87_Ator08_hmg124h_8.JPG.dat	705.3	58.8	0.9	0.06	-0.014	0.056	6.0E-05
YKR093W	YKR093W	UWOPS87_Ator08_hmg124h_8.JPG.dat	637.0	20.7	0.9	0.01	-0.020	0.005	1.1E-05
YKR094C	YKR094C	UWOPS87_Ator08_hmg124h_8.JPG.dat	817.3	49.5	1.1	0.05	0.020	0.047	2.2E-05
YKR095W	YKR095W	UWOPS87_Ator08_hmg124h_8.JPG.dat	707.3	95.5	0.9	0.12	-0.068	0.124	6.9E-04
YKR096W	YKR096W	UWOPS87_Ator08_hmg124h_8.JPG.dat	755.5	32.6	0.9	0.03	-0.031	0.032	1.2E-05
YKR097W	YKR097W	UWOPS87_Ator08_hmg124h_8.JPG.dat	861.5	59.4	1.1	0.03	-0.071	0.027	2.1E-04
YKR098C	YKR098C	UWOPS87_Ator08_hmg124h_8.JPG.dat	847.8	49.4	1.0	0.06	-0.070	0.061	6.0E-05
YKR099W	YKR099W	UWOPS87_Ator08_hmg124h_8.JPG.dat	764.3	55.2	0.9	0.02	-0.079	0.021	1.8E-04
YKR100C	YKR100C	UWOPS87_Ator08_hmg124h_8.JPG.dat	888.3	65.6	1.0	0.07	-0.043	0.075	1.1E-04
YKR101W	YKR101W	UWOPS87_Ator08_hmg124h_8.JPG.dat	913.3	57.2	1.0	0.07	-0.003	0.066	7.6E-05
YKR102W	YKR102W	UWOPS87_Ator08_hmg124h_8.JPG.dat	898.5	62.7	1.1	0.02	0.064	0.016	7.2E-05
YKR103W	YKR103W	UWOPS87_Ator08_hmg124h_8.JPG.dat	854.5	146.1	1.1	0.17	0.057	0.166	9.8E-04
YKR105C	YKR105C	UWOPS87_Ator08_hmg124h_8.JPG.dat	719.0	30.8	1.0	0.05	-0.003	0.045	2.3E-05
YKR106W	YKR106W	UWOPS87_Ator08_hmg124h_8.JPG.dat	748.5	46.5	1.0	0.07	-0.125	0.069	8.4E-05
YLL001W	YLL001W	UWOPS87_Ator08_hmg124h_8.JPG.dat	746.5	40.8	1.0	0.05	0.074	0.048	3.5E-05
YLL002W	YLL002W	UWOPS87_Ator08_hmg124h_8.JPG.dat	380.3	439.1	0.5	0.54	0.126	0.538	1.8E-01
YLL005C	YLL005C	UWOPS87_Ator08_hmg124h_8.JPG.dat	878.5	23.7	1.1	0.03	0.033	0.033	8.5E-06
YLL006W	YLL006W	UWOPS87_Ator08_hmg124h_8.JPG.dat	790.8	49.7	1.0	0.06	-0.044	0.059	6.2E-05
YLL007C	YLL007C	UWOPS87_Ator08_hmg124h_8.JPG.dat	789.5	22.4	1.0	0.01	-0.026	0.009	2.6E-05
YLL009C	YLL009C	UWOPS87_Ator08_hmg124h_8.JPG.dat	851.0	30.5	1.0	0.03	0.038	0.033	1.0E-05
YLL010C	YLL010C	UWOPS87_Ator08_hmg124h_8.JPG.dat	884.3	11.7	1.0	0.01	0.003	0.012	4.8E-07
YLL012W	YLL012W	UWOPS87_Ator08_hmg124h_8.JPG.dat	832.8	33.4	1.0	0.05	-0.015	0.049	3.3E-05
YLL013C	YLL013C	UWOPS87_Ator08_hmg124h_8.JPG.dat	908.3	50.7	1.2	0.01	0.135	0.014	4.9E-05
YLL014W	YLL014W	UWOPS87_Ator08_hmg124h_8.JPG.dat	652.3	56.9	0.9	0.08	-0.095	0.083	1.9E-04
YLL015W	YLL015W	UWOPS87_Ator08_hmg124h_8.JPG.dat	770.5	47.7	1.0	0.06	0.018	0.062	6.1E-05
YLL016W	YLL016W	UWOPS87_Ator08_hmg124h_8.JPG.dat	900.5	28.9	1.2	0.01	0.082	0.012	3.6E-05
YLL017W	YLL017W	UWOPS87_Ator08_hmg124h_8.JPG.dat	843.5	36.4	1.0	0.01	0.054	0.009	2.2E-05
YLL019C	YLL019C	UWOPS87_Ator08_hmg124h_8.JPG.dat	807.8	34.8	1.0	0.04	-0.003	0.040	1.9E-05
YLL020C	YLL020C	UWOPS87_Ator08_hmg124h_8.JPG.dat	631.8	421.4	1.0	0.02	0.047	0.016	8.4E-05
YLL021W	YLL021W	UWOPS87_Ator08_hmg124h_8.JPG.dat	790.3	47.1	1.0	0.05	-0.037	0.048	3.5E-05

YLL023C	YLL023C	UWOPS87_Ator08_hmg124h_8.JPG.dat	851.8	17.2	1.0	0.02	-0.003	0.018	1.6E-06
YLL024C	YLL024C	UWOPS87_Ator08_hmg124h_8.JPG.dat	900.8	56.3	1.0	0.03	0.032	0.025	2.1E-04
YLL025W	YLL025W	UWOPS87_Ator08_hmg124h_8.JPG.dat	769.8	65.4	0.9	0.09	-0.126	0.092	2.6E-04
YLL026W	YLL026W	UWOPS87_Ator08_hmg124h_8.JPG.dat	768.3	41.9	1.0	0.02	-0.033	0.016	9.7E-05
YLL028W	YLL028W	UWOPS87_Ator08_hmg124h_8.JPG.dat	703.0	34.8	1.0	0.05	-0.005	0.049	3.6E-05
YLL029W	YLL029W	UWOPS87_Ator08_hmg124h_8.JPG.dat	816.8	53.8	1.1	0.08	0.006	0.084	1.3E-04
YLL032C	YLL032C	UWOPS87_Ator08_hmg124h_8.JPG.dat	714.3	48.5	0.9	0.07	-0.084	0.065	9.9E-05
YLL038C	YLL038C	UWOPS87_Ator08_hmg124h_8.JPG.dat	863.8	51.7	1.1	0.05	0.036	0.053	3.4E-05
YLL039C	YLL039C	UWOPS87_Ator08_hmg124h_8.JPG.dat	824.0	41.3	1.0	0.05	0.021	0.055	4.2E-05
YLL040C	YLL040C	UWOPS87_Ator08_hmg124h_8.JPG.dat	910.3	36.0	1.1	0.05	0.042	0.054	3.1E-05
YLL041C	YLL041C	UWOPS87_Ator08_hmg124h_8.JPG.dat	798.5	52.6	1.0	0.06	-0.101	0.061	6.7E-05
YLL042C	YLL042C	UWOPS87_Ator08_hmg124h_8.JPG.dat	909.0	30.9	1.1	0.04	0.050	0.040	1.4E-05
YLL043W	YLL043W	UWOPS87_Ator08_hmg124h_8.JPG.dat	1090.8	43.5	1.3	0.05	0.153	0.054	2.0E-05
YLL044W	YLL044W	UWOPS87_Ator08_hmg124h_8.JPG.dat	837.5	37.4	1.0	0.04	0.102	0.044	2.1E-05
YLL045C	YLL045C	UWOPS87_Ator08_hmg124h_8.JPG.dat	809.5	29.9	1.1	0.01	0.236	0.009	2.7E-05
YOR202W	YOR202W	UWOPS87_Ator08_hmg124h_8.JPG.dat	835.1	116.4	1.0	0.08	0.006	0.083	3.4E-297
1	1	UWOPS87_Ator08_hmg124h_9.JPG.dat	1029.5	65.2	1.1	0.03	#NUM!		4.9E-06
2	2	UWOPS87_Ator08_hmg124h_9.JPG.dat	823.3	80.9	0.9	0.06	-0.249	0.060	6.9E-05
3	3	UWOPS87_Ator08_hmg124h_9.JPG.dat	793.8	85.5	1.0	0.06	-0.027	0.064	8.1E-05
4	4	UWOPS87_Ator08_hmg124h_9.JPG.dat	788.8	146.0	0.9	0.13	0.029	0.134	7.7E-04
YLL046C	YLL046C	UWOPS87_Ator08_hmg124h_9.JPG.dat	756.5	42.5	0.9	0.05	-0.022	0.053	4.9E-05
YLL047W	YLL047W	UWOPS87_Ator08_hmg124h_9.JPG.dat	772.3	51.4	0.9	0.07	-0.132	0.066	9.4E-05
YLL048C	YLL048C	UWOPS87_Ator08_hmg124h_9.JPG.dat	895.3	36.9	1.0	0.05	-0.027	0.049	2.8E-05
YLL049W	YLL049W	UWOPS87_Ator08_hmg124h_9.JPG.dat	927.0	446.7	1.0	0.51	0.029	0.505	2.5E-02
YLL051C	YLL051C	UWOPS87_Ator08_hmg124h_9.JPG.dat	882.3	45.5	1.0	0.01	0.017	0.013	5.8E-05
YLL052C	YLL052C	UWOPS87_Ator08_hmg124h_9.JPG.dat	932.8	73.4	1.1	0.08	-0.062	0.081	1.3E-04
YLL053C	YLL053C	UWOPS87_Ator08_hmg124h_9.JPG.dat	946.3	90.2	1.0	0.03	0.054	0.031	3.3E-04
YLL054C	YLL054C	UWOPS87_Ator08_hmg124h_9.JPG.dat	953.5	40.8	1.1	0.04	0.055	0.043	1.8E-05
YLL055W	YLL055W	UWOPS87_Ator08_hmg124h_9.JPG.dat	940.5	33.1	1.1	0.04	0.043	0.038	1.2E-05
YLL056C	YLL056C	UWOPS87_Ator08_hmg124h_9.JPG.dat	802.3	64.8	1.0	0.03	0.068	0.027	2.4E-04
YLL057C	YLL057C	UWOPS87_Ator08_hmg124h_9.JPG.dat	747.3	34.7	1.0	0.04	-0.047	0.045	2.5E-05
YLL058W	YLL058W	UWOPS87_Ator08_hmg124h_9.JPG.dat	886.3	57.4	1.1	0.09	-0.033	0.090	1.5E-04
YLL059C	YLL059C	UWOPS87_Ator08_hmg124h_9.JPG.dat	792.8	47.4	1.0	0.06	0.084	0.061	6.8E-05
YLL060C	YLL060C	UWOPS87_Ator08_hmg124h_9.JPG.dat	837.5	72.2	1.0	0.08	0.021	0.084	1.7E-04
YLL061W	YLL061W	UWOPS87_Ator08_hmg124h_9.JPG.dat	883.8	27.0	1.0	0.03	-0.017	0.033	9.6E-06
YLL062C	YLL062C	UWOPS87_Ator08_hmg124h_9.JPG.dat	876.5	29.6	1.0	0.03	-0.102	0.034	1.1E-05
YLL063C	YLL063C	UWOPS87_Ator08_hmg124h_9.JPG.dat	937.3	18.4	1.1	0.00	0.030	0.005	6.4E-06
YLR001C	YLR001C	UWOPS87_Ator08_hmg124h_9.JPG.dat	855.8	32.5	0.9	0.04	-0.099	0.040	2.0E-05
YLR003C	YLR003C	UWOPS87_Ator08_hmg124h_9.JPG.dat	917.0	26.2	1.0	0.02	-0.163	0.022	2.6E-06
YLR004C	YLR004C	UWOPS87_Ator08_hmg124h_9.JPG.dat	783.3	20.0	0.9	0.02	-0.049	0.024	4.9E-06
YLR006C	YLR006C	UWOPS87_Ator08_hmg124h_9.JPG.dat	811.3	43.3	1.0	0.01	-0.080	0.009	2.7E-05
YLR011W	YLR011W	UWOPS87_Ator08_hmg124h_9.JPG.dat	686.5	30.0	0.9	0.01	-0.094	0.012	5.8E-05
YLR012C	YLR012C	UWOPS87_Ator08_hmg124h_9.JPG.dat	762.0	83.2	1.0	0.03	0.146	0.028	2.7E-04
YLR013W	YLR013W	UWOPS87_Ator08_hmg124h_9.JPG.dat	761.3	77.4	0.9	0.10	-0.084	0.096	3.1E-04
YLR014C	YLR014C	UWOPS87_Ator08_hmg124h_9.JPG.dat	981.0	107.5	1.2	0.12	0.056	0.121	3.1E-04
YLR015W	YLR015W	UWOPS87_Ator08_hmg124h_9.JPG.dat	828.3	15.5	1.0	0.03	-0.166	0.026	5.1E-06
YLR016C	YLR016C	UWOPS87_Ator08_hmg124h_9.JPG.dat	822.8	57.1	1.0	0.07	0.057	0.072	1.1E-04
YLR017W	YLR017W	UWOPS87_Ator08_hmg124h_9.JPG.dat	817.0	56.9	0.9	0.01	-0.006	0.015	8.5E-05
YLR018C	YLR018C	UWOPS87_Ator08_hmg124h_9.JPG.dat	799.0	65.6	0.9	0.07	-0.028	0.071	1.3E-04
YLR019W	YLR019W	UWOPS87_Ator08_hmg124h_9.JPG.dat	806.0	61.6	0.9	0.02	-0.073	0.021	1.7E-04
YLR020C	YLR020C	UWOPS87_Ator08_hmg124h_9.JPG.dat	855.0	55.9	1.0	0.08	-0.083	0.075	1.2E-04
YLR021W	YLR021W	UWOPS87_Ator08_hmg124h_9.JPG.dat	1091.3	98.8	1.3	0.12	0.173	0.118	2.0E-04
YLR023C	YLR023C	UWOPS87_Ator08_hmg124h_9.JPG.dat	821.0	44.4	1.1	0.05	-0.103	0.050	2.9E-05
YLR024C	YLR024C	UWOPS87_Ator08_hmg124h_9.JPG.dat	992.8	29.3	1.1	0.01	0.177	0.012	3.6E-05
YLR028C	YLR028C	UWOPS87_Ator08_hmg124h_9.JPG.dat	907.5	65.8	1.1	0.08	-0.064	0.078	1.1E-04
YLR030W	YLR030W	UWOPS87_Ator08_hmg124h_9.JPG.dat	869.3	71.7	1.0	0.08	0.058	0.081	1.4E-04
YLR031W	YLR031W	UWOPS87_Ator08_hmg124h_9.JPG.dat	767.5	64.3	0.9	0.07	0.025	0.071	1.4E-04
YLR032W	YLR032W	UWOPS87_Ator08_hmg124h_9.JPG.dat	843.3	21.1	1.0	0.02	-0.034	0.022	3.3E-06
YLR034C	YLR034C	UWOPS87_Ator08_hmg124h_9.JPG.dat	914.0	74.4	1.1	0.08	0.030	0.082	1.3E-04
YLR035C	YLR035C	UWOPS87_Ator08_hmg124h_9.JPG.dat	796.0	20.0	0.9	0.01	-0.051	0.008	2.3E-05
YLR036C	YLR036C	UWOPS87_Ator08_hmg124h_9.JPG.dat	766.3	9.4	0.9	0.01	-0.034	0.007	1.3E-07
YLR037C	YLR037C	UWOPS87_Ator08_hmg124h_9.JPG.dat	893.3	23.6	1.0	0.02	-0.087	0.024	3.2E-06
YLR038C	YLR038C	UWOPS87_Ator08_hmg124h_9.JPG.dat	961.8	23.3	1.2	0.01	-0.089	0.010	2.5E-05
YLR039C	YLR039C	UWOPS87_Ator08_hmg124h_9.JPG.dat	459.3	181.6	0.6	0.23	-0.352	0.233	1.5E-02
YLR040C	YLR040C	UWOPS87_Ator08_hmg124h_9.JPG.dat	951.8	63.9	1.1	0.02	0.171	0.022	1.3E-04
YLR041W	YLR041W	UWOPS87_Ator08_hmg124h_9.JPG.dat	798.3	38.9	0.9	0.05	-0.068	0.049	4.4E-05
YLR042C	YLR042C	UWOPS87_Ator08_hmg124h_9.JPG.dat	885.8	33.2	1.0	0.01	0.108	0.011	3.5E-05
YLR043C	YLR043C	UWOPS87_Ator08_hmg124h_9.JPG.dat	835.3	37.4	0.9	0.04	-0.082	0.039	2.0E-05
YLR044C	YLR044C	UWOPS87_Ator08_hmg124h_9.JPG.dat	1064.0	10.4	1.2	0.01	0.165	0.006	3.3E-08
YLR046C	YLR046C	UWOPS87_Ator08_hmg124h_9.JPG.dat	861.3	50.0	1.0	0.01	0.024	0.014	7.7E-05

YLR047C	YLR047C	UWOPS87_Ator08_hmg124h_9.JPG.dat	849.5	66.7	1.0	0.02	0.004	0.017	9.3E-05
YLR048W	YLR048W	UWOPS87_Ator08_hmg124h_9.JPG.dat	949.0	42.7	1.1	0.06	-0.013	0.056	3.7E-05
YLR049C	YLR049C	UWOPS87_Ator08_hmg124h_9.JPG.dat	958.5	35.4	1.1	0.05	0.180	0.051	2.8E-05
YLR050C	YLR050C	UWOPS87_Ator08_hmg124h_9.JPG.dat	901.5	83.0	1.1	0.09	0.191	0.092	1.9E-04
YLR053C	YLR053C	UWOPS87_Ator08_hmg124h_9.JPG.dat	740.0	86.9	0.9	0.11	-0.032	0.105	3.7E-04
YLR054C	YLR054C	UWOPS87_Ator08_hmg124h_9.JPG.dat	881.3	21.7	1.0	0.02	0.035	0.023	3.6E-06
YLR055C	YLR055C	UWOPS87_Ator08_hmg124h_9.JPG.dat	1061.3	96.0	1.2	0.12	0.091	0.116	2.5E-04
YLR056W	YLR056W	UWOPS87_Ator08_hmg124h_9.JPG.dat	858.3	37.9	1.0	0.05	0.009	0.049	3.7E-05
YLR057W	YLR057W	UWOPS87_Ator08_hmg124h_9.JPG.dat	863.3	48.4	1.0	0.02	-0.014	0.019	1.2E-04
YLR058C	YLR058C	UWOPS87_Ator08_hmg124h_9.JPG.dat	851.5	28.8	1.0	0.04	0.025	0.038	1.5E-05
YLR059C	YLR059C	UWOPS87_Ator08_hmg124h_9.JPG.dat	803.0	42.0	0.9	0.05	0.111	0.051	4.5E-05
YLR061W	YLR061W	UWOPS87_Ator08_hmg124h_9.JPG.dat	931.5	16.0	1.1	0.01	-0.031	0.008	1.9E-05
YLR062C	YLR062C	UWOPS87_Ator08_hmg124h_9.JPG.dat	869.8	23.9	1.0	0.01	0.096	0.011	3.9E-05
YLR063W	YLR063W	UWOPS87_Ator08_hmg124h_9.JPG.dat	817.8	121.5	1.0	0.04	-0.158	0.043	6.4E-04
YLR064W	YLR064W	UWOPS87_Ator08_hmg124h_9.JPG.dat	897.3	47.0	1.0	0.05	0.019	0.054	3.9E-05
YLR065C	YLR065C	UWOPS87_Ator08_hmg124h_9.JPG.dat	791.8	88.2	1.0	0.10	0.239	0.099	2.8E-04
YLR070C	YLR070C	UWOPS87_Ator08_hmg124h_9.JPG.dat	805.0	17.8	0.9	0.03	-0.034	0.025	5.3E-06
YLR072W	YLR072W	UWOPS87_Ator08_hmg124h_9.JPG.dat	891.3	30.6	1.0	0.01	0.030	0.010	3.3E-05
YLR073C	YLR073C	UWOPS87_Ator08_hmg124h_9.JPG.dat	866.3	46.1	1.0	0.05	0.087	0.055	4.2E-05
YLR077W	YLR077W	UWOPS87_Ator08_hmg124h_9.JPG.dat	803.5	45.4	1.0	0.01	0.010	0.015	7.6E-05
YLR079W	YLR079W	UWOPS87_Ator08_hmg124h_9.JPG.dat	817.5	49.0	1.0	0.01	0.018	0.011	4.4E-05
YLR080W	YLR080W	UWOPS87_Ator08_hmg124h_9.JPG.dat	780.3	85.4	1.0	0.11	-0.071	0.106	3.5E-04
YLR081W	YLR081W	UWOPS87_Ator08_hmg124h_9.JPG.dat	776.5	67.6	0.9	0.08	-0.077	0.076	1.5E-04
YLR082C	YLR082C	UWOPS87_Ator08_hmg124h_9.JPG.dat	891.5	53.1	1.1	0.06	-0.181	0.061	5.0E-05
YLR083C	YLR083C	UWOPS87_Ator08_hmg124h_9.JPG.dat	951.8	41.9	1.1	0.05	-0.042	0.052	2.7E-05
YLR084C	YLR084C	UWOPS87_Ator08_hmg124h_9.JPG.dat	802.8	60.1	1.0	0.07	0.066	0.072	1.2E-04
YLR085C	YLR085C	UWOPS87_Ator08_hmg124h_9.JPG.dat	674.3	84.0	0.9	0.11	0.005	0.108	5.4E-04
YLR087C	YLR087C	UWOPS87_Ator08_hmg124h_9.JPG.dat	719.0	41.3	0.9	0.05	0.096	0.047	3.4E-05
YLR089C	YLR089C	UWOPS87_Ator08_hmg124h_9.JPG.dat	785.5	42.7	1.0	0.05	-0.026	0.052	4.3E-05
YLR090W	YLR090W	UWOPS87_Ator08_hmg124h_9.JPG.dat	813.8	83.5	1.0	0.10	0.016	0.103	3.3E-04
YLR091W	YLR091W	UWOPS87_Ator08_hmg124h_9.JPG.dat	884.8	25.8	1.0	0.03	0.060	0.028	5.9E-06
YLR092W	YLR092W	UWOPS87_Ator08_hmg124h_9.JPG.dat	910.3	5.2	1.0	0.00	-0.131	0.003	3.1E-06
YLR093C	YLR093C	UWOPS87_Ator08_hmg124h_9.JPG.dat	830.3	50.5	0.9	0.02	-0.064	0.018	1.3E-04
YLR094C	YLR094C	UWOPS87_Ator08_hmg124h_9.JPG.dat	929.0	33.2	1.0	0.01	-0.006	0.012	4.3E-05
YLR095C	YLR095C	UWOPS87_Ator08_hmg124h_9.JPG.dat	821.0	57.5	0.9	0.02	-0.009	0.019	1.3E-04
YLR096W	YLR096W	UWOPS87_Ator08_hmg124h_9.JPG.dat	949.5	15.8	1.1	0.02	0.109	0.018	1.2E-06
YLR097C	YLR097C	UWOPS87_Ator08_hmg124h_9.JPG.dat	786.5	31.9	0.9	0.04	-0.128	0.044	2.7E-05
YLR098C	YLR098C	UWOPS87_Ator08_hmg124h_9.JPG.dat	981.8	88.1	1.2	0.02	0.026	0.022	1.2E-04
YLR099C	YLR099C	UWOPS87_Ator08_hmg124h_9.JPG.dat	784.8	67.8	1.0	0.09	-0.181	0.086	1.6E-04
YLR102C	YLR102C	UWOPS87_Ator08_hmg124h_9.JPG.dat	797.3	52.6	1.0	0.07	-0.067	0.070	1.1E-04
YLR104W	YLR104W	UWOPS87_Ator08_hmg124h_9.JPG.dat	907.3	75.7	1.1	0.09	0.151	0.087	1.4E-04
YLR107W	YLR107W	UWOPS87_Ator08_hmg124h_9.JPG.dat	809.0	50.2	0.9	0.06	0.314	0.061	7.8E-05
YLR108C	YLR108C	UWOPS87_Ator08_hmg124h_9.JPG.dat	812.5	79.4	0.9	0.09	0.084	0.092	2.5E-04
YLR109W	YLR109W	UWOPS87_Ator08_hmg124h_9.JPG.dat	874.8	49.2	1.0	0.06	-0.042	0.056	4.7E-05
YLR110C	YLR110C	UWOPS87_Ator08_hmg124h_9.JPG.dat	912.8	35.2	1.0	0.04	0.056	0.042	1.9E-05
YLR111W	YLR111W	UWOPS87_Ator08_hmg124h_9.JPG.dat	886.5	41.2	1.0	0.05	-0.051	0.046	2.8E-05
YLR112W	YLR112W	UWOPS87_Ator08_hmg124h_9.JPG.dat	831.0	67.3	1.0	0.08	0.036	0.079	1.5E-04
YLR113W	YLR113W	UWOPS87_Ator08_hmg124h_9.JPG.dat	862.3	58.2	1.1	0.06	0.092	0.061	5.3E-05
YLR114C	YLR114C	UWOPS87_Ator08_hmg124h_9.JPG.dat	792.8	98.8	1.0	0.11	0.041	0.112	3.8E-04
YLR118C	YLR118C	UWOPS87_Ator08_hmg124h_9.JPG.dat	810.8	164.9	1.2	0.07	0.151	0.067	1.1E-03
YLR119W	YLR119W	UWOPS87_Ator08_hmg124h_9.JPG.dat	951.3	48.7	1.1	0.01	-0.050	0.007	1.5E-05
YLR120C	YLR120C	UWOPS87_Ator08_hmg124h_9.JPG.dat	847.8	50.3	1.0	0.05	0.185	0.048	3.0E-05
YLR121C	YLR121C	UWOPS87_Ator08_hmg124h_9.JPG.dat	865.5	117.7	1.0	0.13	-0.036	0.127	5.5E-04
YLR122C	YLR122C	UWOPS87_Ator08_hmg124h_9.JPG.dat	854.0	40.5	1.0	0.04	0.070	0.041	1.8E-05
YLR123C	YLR123C	UWOPS87_Ator08_hmg124h_9.JPG.dat	901.3	43.8	1.0	0.04	0.058	0.041	1.7E-05
YLR124W	YLR124W	UWOPS87_Ator08_hmg124h_9.JPG.dat	866.8	24.6	1.0	0.02	0.046	0.020	2.3E-06
YLR125W	YLR125W	UWOPS87_Ator08_hmg124h_9.JPG.dat	891.5	25.8	1.0	0.04	0.010	0.040	1.7E-05
YLR126C	YLR126C	UWOPS87_Ator08_hmg124h_9.JPG.dat	839.3	36.7	1.0	0.03	0.016	0.031	8.5E-06
YLR128W	YLR128W	UWOPS87_Ator08_hmg124h_9.JPG.dat	754.0	37.5	0.9	0.05	-0.110	0.053	5.3E-05
YLR130C	YLR130C	UWOPS87_Ator08_hmg124h_9.JPG.dat	791.8	190.9	1.1	0.05	0.122	0.050	6.8E-04
YLR131C	YLR131C	UWOPS87_Ator08_hmg124h_9.JPG.dat	689.3	15.5	0.9	0.03	-0.248	0.029	1.1E-05
YLR133W	YLR133W	UWOPS87_Ator08_hmg124h_9.JPG.dat	781.0	118.5	1.0	0.03	0.100	0.031	3.4E-04
YLR134W	YLR134W	UWOPS87_Ator08_hmg124h_9.JPG.dat	984.8	59.6	1.2	0.01	-0.056	0.008	1.6E-05
YLR135W	YLR135W	UWOPS87_Ator08_hmg124h_9.JPG.dat	863.5	12.8	1.0	0.01	0.107	0.014	8.5E-07
YLR136C	YLR136C	UWOPS87_Ator08_hmg124h_9.JPG.dat	852.3	28.5	1.0	0.03	-0.215	0.032	9.4E-06
YLR137W	YLR137W	UWOPS87_Ator08_hmg124h_9.JPG.dat	814.8	63.1	0.9	0.01	-0.112	0.006	1.3E-05
YLR138W	YLR138W	UWOPS87_Ator08_hmg124h_9.JPG.dat	811.0	71.3	0.9	0.09	-0.163	0.086	2.1E-04
YLR142W	YLR142W	UWOPS87_Ator08_hmg124h_9.JPG.dat	919.5	19.3	1.1	0.03	0.052	0.029	5.5E-06
YLR143W	YLR143W	UWOPS87_Ator08_hmg124h_9.JPG.dat	804.5	40.1	1.0	0.05	-0.053	0.051	4.3E-05
YLR144C	YLR144C	UWOPS87_Ator08_hmg124h_9.JPG.dat	917.3	73.6	1.1	0.02	0.113	0.020	1.0E-04

YLR146C	YLR146C	UWOPS87_Ator08_hmg124h_9.JPG.dat	795.5	24.3	1.0	0.03	0.058	0.028	6.5E-06
YLR149C	YLR149C	UWOPS87_Ator08_hmg124h_9.JPG.dat	988.5	95.5	1.2	0.10	0.163	0.098	1.5E-04
YLR150W	YLR150W	UWOPS87_Ator08_hmg124h_9.JPG.dat	980.5	45.9	1.1	0.05	0.093	0.048	2.4E-05
YLR151C	YLR151C	UWOPS87_Ator08_hmg124h_9.JPG.dat	874.8	27.6	1.0	0.01	0.035	0.008	2.1E-05
YLR152C	YLR152C	UWOPS87_Ator08_hmg124h_9.JPG.dat	879.8	45.7	1.0	0.05	-0.156	0.046	2.9E-05
YLR154C	YLR154C	UWOPS87_Ator08_hmg124h_9.JPG.dat	870.5	26.0	1.0	0.03	-0.073	0.027	5.5E-06
YLR164W	YLR164W	UWOPS87_Ator08_hmg124h_9.JPG.dat	852.0	23.2	1.0	0.03	-0.012	0.028	6.4E-06
YLR165C	YLR165C	UWOPS87_Ator08_hmg124h_9.JPG.dat	824.8	36.3	0.9	0.04	0.026	0.041	2.2E-05
YLR168C	YLR168C	UWOPS87_Ator08_hmg124h_9.JPG.dat	893.8	14.1	1.0	0.02	-0.090	0.020	2.0E-06
YLR169W	YLR169W	UWOPS87_Ator08_hmg124h_9.JPG.dat	751.5	20.8	0.9	0.02	-0.181	0.019	2.8E-06
YLR170C	YLR170C	UWOPS87_Ator08_hmg124h_9.JPG.dat	763.0	49.0	0.9	0.02	-0.102	0.019	1.6E-04
YLR171W	YLR171W	UWOPS87_Ator08_hmg124h_9.JPG.dat	786.3	87.3	1.0	0.09	-0.136	0.090	2.3E-04
YLR172C	YLR172C	UWOPS87_Ator08_hmg124h_9.JPG.dat	872.3	60.4	1.1	0.02	0.152	0.021	1.2E-04
YLR173W	YLR173W	UWOPS87_Ator08_hmg124h_9.JPG.dat	912.3	57.7	1.0	0.06	0.048	0.063	6.6E-05
YLR174W	YLR174W	UWOPS87_Ator08_hmg124h_9.JPG.dat	970.0	38.7	1.1	0.04	-0.126	0.043	1.7E-05
YLR176C	YLR176C	UWOPS87_Ator08_hmg124h_9.JPG.dat	944.8	33.5	1.0	0.04	0.077	0.039	1.5E-05
YLR177W	YLR177W	UWOPS87_Ator08_hmg124h_9.JPG.dat	798.8	20.2	0.9	0.02	-0.024	0.021	3.1E-06
YLR178C	YLR178C	UWOPS87_Ator08_hmg124h_9.JPG.dat	883.0	15.2	1.0	0.02	0.006	0.019	1.6E-06
YLR179C	YLR179C	UWOPS87_Ator08_hmg124h_9.JPG.dat	916.3	42.8	1.0	0.02	-0.069	0.017	9.2E-05
YLR180W	YLR180W	UWOPS87_Ator08_hmg124h_9.JPG.dat	904.3	37.0	1.0	0.04	-0.007	0.037	1.3E-05
YLR181C	YLR181C	UWOPS87_Ator08_hmg124h_9.JPG.dat	1089.3	59.0	1.3	0.01	0.057	0.012	3.1E-05
YLR182W	YLR182W	UWOPS87_Ator08_hmg124h_9.JPG.dat	490.3	497.1	0.6	0.57	0.565	0.571	1.4E-01
YLR183C	YLR183C	UWOPS87_Ator08_hmg124h_9.JPG.dat	717.3	72.4	0.9	0.10	-0.096	0.098	4.0E-04
YLR184W	YLR184W	UWOPS87_Ator08_hmg124h_9.JPG.dat	697.0	89.2	0.9	0.11	0.257	0.112	5.8E-04
YLR185W	YLR185W	UWOPS87_Ator08_hmg124h_9.JPG.dat	808.5	27.9	0.9	0.03	0.355	0.034	1.3E-05
YLR187W	YLR187W	UWOPS87_Ator08_hmg124h_9.JPG.dat	854.5	40.6	1.0	0.05	0.033	0.047	2.8E-05
YLR188W	YLR188W	UWOPS87_Ator08_hmg124h_9.JPG.dat	886.5	63.3	1.0	0.08	-0.170	0.075	1.0E-04
YLR189C	YLR189C	UWOPS87_Ator08_hmg124h_9.JPG.dat	797.8	25.0	1.0	0.03	-0.004	0.026	5.2E-06
YLR190W	YLR190W	UWOPS87_Ator08_hmg124h_9.JPG.dat	772.5	24.3	1.0	0.03	0.014	0.028	6.6E-06
YLR191W	YLR191W	UWOPS87_Ator08_hmg124h_9.JPG.dat	988.0	108.9	1.2	0.13	0.034	0.127	3.1E-04
YLR192C	YLR192C	UWOPS87_Ator08_hmg124h_9.JPG.dat	831.0	47.5	1.0	0.06	-0.032	0.061	6.4E-05
YLR193C	YLR193C	UWOPS87_Ator08_hmg124h_9.JPG.dat	845.8	83.9	1.0	0.10	0.011	0.097	2.4E-04
YLR194C	YLR194C	UWOPS87_Ator08_hmg124h_9.JPG.dat	868.5	38.7	1.0	0.05	0.075	0.046	2.4E-05
YLR199C	YLR199C	UWOPS87_Ator08_hmg124h_9.JPG.dat	903.3	100.5	1.1	0.12	0.052	0.122	3.6E-04
YLR200W	YLR200W	UWOPS87_Ator08_hmg124h_9.JPG.dat	799.0	74.1	0.9	0.01	-0.021	0.013	7.4E-05
YLR205C	YLR205C	UWOPS87_Ator08_hmg124h_9.JPG.dat	840.5	17.9	1.0	0.03	-0.067	0.029	6.8E-06
YLR206W	YLR206W	UWOPS87_Ator08_hmg124h_9.JPG.dat	836.3	51.2	0.9	0.06	0.064	0.059	6.6E-05
YLR207W	YLR207W	UWOPS87_Ator08_hmg124h_9.JPG.dat	1011.5	82.4	1.1	0.03	0.057	0.027	2.1E-04
YLR209C	YLR209C	UWOPS87_Ator08_hmg124h_9.JPG.dat	900.3	38.3	1.0	0.01	-0.002	0.011	4.2E-05
YLR210W	YLR210W	UWOPS87_Ator08_hmg124h_9.JPG.dat	918.5	30.8	1.0	0.04	-0.134	0.036	1.3E-05
YLR211C	YLR211C	UWOPS87_Ator08_hmg124h_9.JPG.dat	1007.5	40.1	1.1	0.01	0.063	0.009	2.3E-05
YLR213C	YLR213C	UWOPS87_Ator08_hmg124h_9.JPG.dat	879.3	37.0	1.0	0.04	-0.252	0.035	1.4E-05
YLR214W	YLR214W	UWOPS87_Ator08_hmg124h_9.JPG.dat	977.0	22.1	1.1	0.02	0.091	0.017	1.2E-06
YLR216C	YLR216C	UWOPS87_Ator08_hmg124h_9.JPG.dat	781.3	114.7	0.8	0.05	-0.163	0.052	1.3E-03
YLR217W	YLR217W	UWOPS87_Ator08_hmg124h_9.JPG.dat	827.5	66.7	1.0	0.09	-0.117	0.093	1.9E-04
YLR218C	YLR218C	UWOPS87_Ator08_hmg124h_9.JPG.dat	820.3	35.5	1.0	0.03	-0.095	0.031	7.7E-06
YLR219W	YLR219W	UWOPS87_Ator08_hmg124h_9.JPG.dat	848.0	81.4	1.0	0.09	0.108	0.092	1.9E-04
YLR220W	YLR220W	UWOPS87_Ator08_hmg124h_9.JPG.dat	871.5	70.9	1.0	0.08	-0.108	0.076	1.2E-04
YLR221C	YLR221C	UWOPS87_Ator08_hmg124h_9.JPG.dat	905.3	23.8	1.0	0.03	0.113	0.025	4.1E-06
YLR224W	YLR224W	UWOPS87_Ator08_hmg124h_9.JPG.dat	846.0	30.1	1.0	0.01	-0.026	0.010	3.6E-05
YLR225C	YLR225C	UWOPS87_Ator08_hmg124h_9.JPG.dat	916.3	53.1	1.0	0.06	-0.054	0.056	4.3E-05
YLR228C	YLR228C	UWOPS87_Ator08_hmg124h_9.JPG.dat	905.3	37.4	1.0	0.04	-0.033	0.036	1.3E-05
YLR231C	YLR231C	UWOPS87_Ator08_hmg124h_9.JPG.dat	909.8	82.2	1.0	0.10	0.167	0.100	2.5E-04
YLR232W	YLR232W	UWOPS87_Ator08_hmg124h_9.JPG.dat	863.8	37.6	1.0	0.05	-0.168	0.045	2.6E-05
YLR233C	YLR233C	UWOPS87_Ator08_hmg124h_9.JPG.dat	201.5	238.3	0.2	0.29	0.249	0.294	1.9E-01
YLR234W	YLR234W	UWOPS87_Ator08_hmg124h_9.JPG.dat	678.8	43.3	0.9	0.05	-0.003	0.055	6.1E-05
YLR235C	YLR235C	UWOPS87_Ator08_hmg124h_9.JPG.dat	922.8	83.7	1.2	0.03	-0.017	0.026	1.7E-04
YLR236C	YLR236C	UWOPS87_Ator08_hmg124h_9.JPG.dat	931.5	121.7	1.1	0.13	0.182	0.134	4.9E-04
YLR237W	YLR237W	UWOPS87_Ator08_hmg124h_9.JPG.dat	808.3	28.7	0.9	0.03	-0.164	0.035	1.4E-05
YLR239C	YLR239C	UWOPS87_Ator08_hmg124h_9.JPG.dat	471.8	314.8	0.5	0.36	-0.086	0.357	5.8E-02
YLR241W	YLR241W	UWOPS87_Ator08_hmg124h_9.JPG.dat	845.3	71.3	0.9	0.01	-0.026	0.013	6.2E-05
YLR242C	YLR242C	UWOPS87_Ator08_hmg124h_9.JPG.dat	470.3	320.3	0.7	0.09	0.703	0.093	5.7E-03
YLR246W	YLR246W	UWOPS87_Ator08_hmg124h_9.JPG.dat	1021.8	31.4	1.1	0.04	0.057	0.037	9.7E-06
YLR247C	YLR247C	UWOPS87_Ator08_hmg124h_9.JPG.dat	869.3	17.7	1.0	0.02	0.101	0.022	3.1E-06
YLR248W	YLR248W	UWOPS87_Ator08_hmg124h_9.JPG.dat	874.8	19.6	1.0	0.03	0.039	0.031	8.5E-06
YLR250W	YLR250W	UWOPS87_Ator08_hmg124h_9.JPG.dat	826.5	89.1	1.0	0.10	-0.043	0.097	2.6E-04
YLR251W	YLR251W	UWOPS87_Ator08_hmg124h_9.JPG.dat	808.5	49.2	1.1	0.03	0.131	0.025	1.9E-04
YLR252W	YLR252W	UWOPS87_Ator08_hmg124h_9.JPG.dat	853.8	62.2	1.0	0.02	-0.151	0.017	1.0E-04
YLR253W	YLR253W	UWOPS87_Ator08_hmg124h_9.JPG.dat	844.8	99.9	1.0	0.02	0.006	0.023	1.6E-04
YLR254C	YLR254C	UWOPS87_Ator08_hmg124h_9.JPG.dat	812.5	36.1	0.9	0.04	-0.063	0.036	1.6E-05

YLR255C	YLR255C	UWOPS87_Ator08_hmg124h_9.JPG.dat	884.0	38.2	1.0	0.05	0.074	0.051	3.6E-05
YLR257W	YLR257W	UWOPS87_Ator08_hmg124h_9.JPG.dat	927.0	13.6	1.0	0.01	-0.004	0.008	1.1E-07
YLR258W	YLR258W	UWOPS87_Ator08_hmg124h_9.JPG.dat	889.8	39.8	1.0	0.04	0.011	0.040	1.8E-05
YLR261C	YLR261C	UWOPS87_Ator08_hmg124h_9.JPG.dat	894.8	46.3	1.0	0.05	-0.061	0.051	3.8E-05
YLR262C	YLR262C	UWOPS87_Ator08_hmg124h_9.JPG.dat	707.0	42.9	0.8	0.05	-0.070	0.053	7.6E-05
YLR262C-A	YLR262C-A	UWOPS87_Ator08_hmg124h_9.JPG.dat	889.8	44.1	1.0	0.06	0.106	0.056	4.7E-05
YLR263W	YLR263W	UWOPS87_Ator08_hmg124h_9.JPG.dat	953.3	138.1	1.1	0.15	0.179	0.149	6.8E-04
YLR264W	YLR264W	UWOPS87_Ator08_hmg124h_9.JPG.dat	797.3	214.3	1.0	0.27	0.444	0.267	5.1E-03
YLR265C	YLR265C	UWOPS87_Ator08_hmg124h_9.JPG.dat	854.8	65.9	1.0	0.06	-0.016	0.058	6.1E-05
YLR266C	YLR266C	UWOPS87_Ator08_hmg124h_9.JPG.dat	924.8	42.6	1.0	0.02	-0.004	0.017	9.1E-05
YLR267W	YLR267W	UWOPS87_Ator08_hmg124h_9.JPG.dat	959.8	72.1	1.1	0.07	0.112	0.070	7.5E-05
YLR268W	YLR268W	UWOPS87_Ator08_hmg124h_9.JPG.dat	939.0	18.9	1.0	0.02	-0.074	0.021	2.2E-06
YLR269C	YLR269C	UWOPS87_Ator08_hmg124h_9.JPG.dat	982.0	57.6	1.1	0.08	-0.030	0.076	9.1E-05
YLR271W	YLR271W	UWOPS87_Ator08_hmg124h_9.JPG.dat	928.3	7.7	1.0	0.02	-0.007	0.018	1.5E-06
YLR273C	YLR273C	UWOPS87_Ator08_hmg124h_9.JPG.dat	862.3	53.5	0.9	0.02	-0.048	0.018	1.3E-04
YLR278C	YLR278C	UWOPS87_Ator08_hmg124h_9.JPG.dat	811.3	17.1	0.9	0.02	-0.037	0.023	4.0E-06
YLR279W	YLR279W	UWOPS87_Ator08_hmg124h_9.JPG.dat	917.5	84.5	1.0	0.09	0.099	0.094	2.1E-04
YLR280C	YLR280C	UWOPS87_Ator08_hmg124h_9.JPG.dat	891.0	28.3	1.0	0.02	-0.025	0.023	3.1E-06
YLR281C	YLR281C	UWOPS87_Ator08_hmg124h_9.JPG.dat	812.5	105.2	1.0	0.05	0.047	0.046	7.5E-04
YLR282C	YLR282C	UWOPS87_Ator08_hmg124h_9.JPG.dat	861.8	21.9	1.0	0.01	-0.060	0.007	1.2E-07
YLR283W	YLR283W	UWOPS87_Ator08_hmg124h_9.JPG.dat	814.5	53.0	0.9	0.06	-0.084	0.059	7.6E-05
YLR284C	YLR284C	UWOPS87_Ator08_hmg124h_9.JPG.dat	884.3	88.4	1.0	0.09	-0.032	0.094	2.4E-04
YLR285W	YLR285W	UWOPS87_Ator08_hmg124h_9.JPG.dat	880.3	45.4	1.0	0.05	0.076	0.050	3.7E-05
YLR286C	YLR286C	UWOPS87_Ator08_hmg124h_9.JPG.dat	907.5	30.7	1.0	0.03	-0.071	0.034	1.0E-05
YLR287C	YLR287C	UWOPS87_Ator08_hmg124h_9.JPG.dat	898.8	31.1	1.0	0.03	-0.087	0.034	1.0E-05
YLR287C-A	YLR287C-A	UWOPS87_Ator08_hmg124h_9.JPG.dat	933.8	10.4	1.0	0.00	-0.100	0.001	1.8E-07
YLR289W	YLR289W	UWOPS87_Ator08_hmg124h_9.JPG.dat	933.3	32.5	1.1	0.04	-0.002	0.042	1.7E-05
YLR290C	YLR290C	UWOPS87_Ator08_hmg124h_9.JPG.dat	978.3	35.8	1.1	0.01	-0.100	0.015	6.3E-05
YLR292C	YLR292C	UWOPS87_Ator08_hmg124h_9.JPG.dat	970.8	60.0	1.1	0.07	0.186	0.066	5.7E-05
YLR294C	YLR294C	UWOPS87_Ator08_hmg124h_9.JPG.dat	821.8	50.3	1.0	0.06	0.065	0.057	4.8E-05
YLR296W	YLR296W	UWOPS87_Ator08_hmg124h_9.JPG.dat	904.5	59.4	1.0	0.08	-0.005	0.082	1.3E-04
YLR297W	YLR297W	UWOPS87_Ator08_hmg124h_9.JPG.dat	835.8	39.2	1.0	0.05	-0.038	0.049	3.5E-05
YLR299W	YLR299W	UWOPS87_Ator08_hmg124h_9.JPG.dat	914.0	69.8	1.1	0.08	0.018	0.082	1.3E-04
YLR300W	YLR300W	UWOPS87_Ator08_hmg124h_9.JPG.dat	915.8	40.9	1.1	0.04	0.012	0.043	1.9E-05
YLR303W	YLR303W	UWOPS87_Ator08_hmg124h_9.JPG.dat	814.5	44.0	1.0	0.05	-0.054	0.054	4.6E-05
YLR306W	YLR306W	UWOPS87_Ator08_hmg124h_9.JPG.dat	847.5	50.0	1.0	0.06	-0.010	0.063	6.4E-05
YLR307W	YLR307W	UWOPS87_Ator08_hmg124h_9.JPG.dat	857.0	72.4	1.0	0.02	0.008	0.018	1.0E-04
YLR309C	YLR309C	UWOPS87_Ator08_hmg124h_9.JPG.dat	891.3	24.4	1.0	0.03	0.096	0.026	4.4E-06
YLR311C	YLR311C	UWOPS87_Ator08_hmg124h_9.JPG.dat	822.0	16.7	0.9	0.02	0.016	0.022	3.7E-06
YLR312C	YLR312C	UWOPS87_Ator08_hmg124h_9.JPG.dat	782.3	27.1	0.9	0.01	-0.093	0.012	5.6E-05
YLR313C	YLR313C	UWOPS87_Ator08_hmg124h_9.JPG.dat	886.0	115.7	1.1	0.15	0.053	0.150	6.7E-04
YLR315W	YLR315W	UWOPS87_Ator08_hmg124h_9.JPG.dat	709.0	28.4	0.9	0.03	-0.076	0.032	1.4E-05
YLR318W	YLR318W	UWOPS87_Ator08_hmg124h_9.JPG.dat	838.3	63.7	1.0	0.07	-0.219	0.073	1.2E-04
YLR319C	YLR319C	UWOPS87_Ator08_hmg124h_9.JPG.dat	916.0	20.8	1.0	0.02	0.034	0.017	1.3E-06
YLR320W	YLR320W	UWOPS87_Ator08_hmg124h_9.JPG.dat	925.5	21.0	1.0	0.02	0.090	0.025	4.1E-06
YLR324W	YLR324W	UWOPS87_Ator08_hmg124h_9.JPG.dat	1021.3	69.9	1.1	0.06	0.235	0.065	5.1E-05
YLR325C	YLR325C	UWOPS87_Ator08_hmg124h_9.JPG.dat	950.5	32.4	1.0	0.03	-0.063	0.029	5.9E-06
YLR326W	YLR326W	UWOPS87_Ator08_hmg124h_9.JPG.dat	950.5	30.7	1.0	0.01	0.121	0.009	2.6E-05
YLR327C	YLR327C	UWOPS87_Ator08_hmg124h_9.JPG.dat	963.3	65.3	1.0	0.01	0.012	0.014	6.2E-05
YLR328W	YLR328W	UWOPS87_Ator08_hmg124h_9.JPG.dat	947.8	96.5	1.0	0.10	0.155	0.098	2.3E-04
YLR329W	YLR329W	UWOPS87_Ator08_hmg124h_9.JPG.dat	808.5	86.2	0.9	0.08	0.002	0.082	1.9E-04
YLR330W	YLR330W	UWOPS87_Ator08_hmg124h_9.JPG.dat	805.8	71.0	1.0	0.09	-0.109	0.091	2.2E-04
YLR332W	YLR332W	UWOPS87_Ator08_hmg124h_9.JPG.dat	600.0	48.3	0.8	0.05	-0.172	0.055	9.0E-05
YLR333C	YLR333C	UWOPS87_Ator08_hmg124h_9.JPG.dat	837.0	35.6	1.0	0.05	0.080	0.050	3.2E-05
YLR334C	YLR334C	UWOPS87_Ator08_hmg124h_9.JPG.dat	886.5	50.2	1.1	0.07	-0.132	0.067	7.0E-05
YLR335W	YLR335W	UWOPS87_Ator08_hmg124h_9.JPG.dat	912.0	31.8	1.0	0.04	-0.039	0.040	1.5E-05
YLR337C	YLR337C	UWOPS87_Ator08_hmg124h_9.JPG.dat	1198.8	452.6	1.4	0.52	0.151	0.515	1.3E-02
YLR338W	YLR338W	UWOPS87_Ator08_hmg124h_9.JPG.dat	331.5	402.9	0.4	0.46	0.377	0.458	2.0E-01
YLR341W	YLR341W	UWOPS87_Ator08_hmg124h_9.JPG.dat	728.8	98.1	0.8	0.11	-0.031	0.112	7.0E-04
YLR342W	YLR342W	UWOPS87_Ator08_hmg124h_9.JPG.dat	933.3	10.7	1.0	0.00	0.031	0.002	7.2E-07
YLR343W	YLR343W	UWOPS87_Ator08_hmg124h_9.JPG.dat	878.3	67.7	1.0	0.08	-0.001	0.079	1.3E-04
YLR344W	YLR344W	UWOPS87_Ator08_hmg124h_9.JPG.dat	820.8	47.0	1.0	0.07	-0.125	0.065	7.6E-05
YLR345W	YLR345W	UWOPS87_Ator08_hmg124h_9.JPG.dat	777.3	32.1	1.0	0.04	-0.018	0.044	2.4E-05
YLR346C	YLR346C	UWOPS87_Ator08_hmg124h_9.JPG.dat	741.0	32.9	1.0	0.01	0.061	0.013	5.9E-05
YLR348C	YLR348C	UWOPS87_Ator08_hmg124h_9.JPG.dat	843.0	38.6	1.0	0.04	0.020	0.042	2.1E-05
YLR349W	YLR349W	UWOPS87_Ator08_hmg124h_9.JPG.dat	846.0	58.0	1.0	0.06	-0.242	0.061	6.6E-05
YLR350W	YLR350W	UWOPS87_Ator08_hmg124h_9.JPG.dat	898.5	44.5	1.0	0.05	-0.150	0.054	4.1E-05
YLR351C	YLR351C	UWOPS87_Ator08_hmg124h_9.JPG.dat	892.0	32.2	1.0	0.04	0.057	0.036	1.2E-05
YLR352W	YLR352W	UWOPS87_Ator08_hmg124h_9.JPG.dat	863.8	24.6	1.0	0.01	-0.079	0.010	3.1E-05
YLR353W	YLR353W	UWOPS87_Ator08_hmg124h_9.JPG.dat	891.8	45.2	1.0	0.02	-0.057	0.018	1.0E-04

YLR354C	YLR354C	UWOPS87_Ator08_hmg124h_9.JPG.dat	943.5	28.4	1.0	0.04	0.002	0.036	1.2E-05
YLR356W	YLR356W	UWOPS87_Ator08_hmg124h_9.JPG.dat	963.3	34.9	1.1	0.04	0.050	0.044	1.7E-05
YLR357W	YLR357W	UWOPS87_Ator08_hmg124h_9.JPG.dat	484.3	402.0	0.6	0.48	0.481	0.478	9.4E-02
YLR360W	YLR360W	UWOPS87_Ator08_hmg124h_9.JPG.dat	680.8	34.2	0.9	0.01	-0.040	0.009	3.5E-05
YLR361C	YLR361C	UWOPS87_Ator08_hmg124h_9.JPG.dat	789.5	46.5	1.0	0.05	0.026	0.050	3.8E-05
YLR362W	YLR362W	UWOPS87_Ator08_hmg124h_9.JPG.dat	299.5	314.0	0.3	0.35	0.338	0.351	1.5E-01
YLR363C	YLR363C	UWOPS87_Ator08_hmg124h_9.JPG.dat	868.0	66.7	1.0	0.08	-0.038	0.085	1.7E-04
YLR364W	YLR364W	UWOPS87_Ator08_hmg124h_9.JPG.dat	950.5	25.7	1.1	0.00	-0.101	0.005	6.0E-06
YLR365W	YLR365W	UWOPS87_Ator08_hmg124h_9.JPG.dat	814.5	23.4	0.9	0.01	-0.060	0.007	1.8E-05
YLR366W	YLR366W	UWOPS87_Ator08_hmg124h_9.JPG.dat	924.8	65.9	1.0	0.07	0.001	0.071	8.8E-05
YLR367W	YLR367W	UWOPS87_Ator08_hmg124h_9.JPG.dat	894.8	41.5	1.0	0.05	0.015	0.049	3.3E-05
YLR368W	YLR368W	UWOPS87_Ator08_hmg124h_9.JPG.dat	964.3	35.4	1.1	0.05	0.053	0.045	2.0E-05
YLR370C	YLR370C	UWOPS87_Ator08_hmg124h_9.JPG.dat	866.3	92.1	1.1	0.02	-0.059	0.023	1.6E-04
YLR371W	YLR371W	UWOPS87_Ator08_hmg124h_9.JPG.dat	771.8	102.2	0.9	0.12	-0.193	0.120	6.4E-04
YLR372W	YLR372W	UWOPS87_Ator08_hmg124h_9.JPG.dat	854.3	100.1	1.0	0.11	0.052	0.108	3.2E-04
YLR373C	YLR373C	UWOPS87_Ator08_hmg124h_9.JPG.dat	1070.5	40.1	1.3	0.07	0.275	0.069	4.1E-05
YLR374C	YLR374C	UWOPS87_Ator08_hmg124h_9.JPG.dat	929.8	12.6	1.0	0.00	-0.111	0.002	1.2E-06
YLR375W	YLR375W	UWOPS87_Ator08_hmg124h_9.JPG.dat	874.5	48.7	1.0	0.00	0.011	0.004	5.2E-06
YLR376C	YLR376C	UWOPS87_Ator08_hmg124h_9.JPG.dat	868.0	54.8	1.0	0.05	0.062	0.052	4.3E-05
YLR377C	YLR377C	UWOPS87_Ator08_hmg124h_9.JPG.dat	974.3	34.8	1.1	0.05	-0.148	0.049	2.4E-05
YLR380W	YLR380W	UWOPS87_Ator08_hmg124h_9.JPG.dat	899.5	59.3	1.0	0.07	0.013	0.070	9.2E-05
YLR381W	YLR381W	UWOPS87_Ator08_hmg124h_9.JPG.dat	907.5	15.2	1.0	0.03	-0.053	0.027	5.1E-06
YLR384C	YLR384C	UWOPS87_Ator08_hmg124h_9.JPG.dat	917.5	24.9	1.0	0.03	0.189	0.035	1.0E-05
YLR385C	YLR385C	UWOPS87_Ator08_hmg124h_9.JPG.dat	757.0	26.7	0.9	0.04	-0.035	0.038	2.3E-05
YLR386W	YLR386W	UWOPS87_Ator08_hmg124h_9.JPG.dat	790.5	58.3	0.9	0.02	0.351	0.021	1.7E-04
YLR387C	YLR387C	UWOPS87_Ator08_hmg124h_9.JPG.dat	806.5	76.8	1.0	0.08	-0.110	0.085	1.8E-04
YLR388W	YLR388W	UWOPS87_Ator08_hmg124h_9.JPG.dat	746.8	53.3	0.9	0.06	0.191	0.056	6.8E-05
YLR389C	YLR389C	UWOPS87_Ator08_hmg124h_9.JPG.dat	982.3	71.1	1.1	0.08	0.033	0.080	1.2E-04
YLR390W	YLR390W	UWOPS87_Ator08_hmg124h_9.JPG.dat	879.0	80.0	1.0	0.09	0.014	0.087	1.9E-04
YLR390W-/	YLR390W-A	UWOPS87_Ator08_hmg124h_9.JPG.dat	933.3	28.0	1.0	0.03	-0.021	0.028	5.7E-06
YLR391W	YLR391W	UWOPS87_Ator08_hmg124h_9.JPG.dat	852.8	37.2	0.9	0.02	0.026	0.016	9.0E-05
YLR392C	YLR392C	UWOPS87_Ator08_hmg124h_9.JPG.dat	869.5	60.1	1.0	0.07	0.018	0.069	9.2E-05
YLR393W	YLR393W	UWOPS87_Ator08_hmg124h_9.JPG.dat	842.8	40.6	1.0	0.00	0.034	0.001	5.4E-07
YLR394W	YLR394W	UWOPS87_Ator08_hmg124h_9.JPG.dat	889.8	32.8	1.0	0.03	0.031	0.032	9.2E-06
YLR395C	YLR395C	UWOPS87_Ator08_hmg124h_9.JPG.dat	849.3	57.4	0.9	0.02	-0.027	0.016	9.8E-05
YLR398C	YLR398C	UWOPS87_Ator08_hmg124h_9.JPG.dat	997.0	67.4	1.1	0.07	0.131	0.074	7.5E-05
YLR400W	YLR400W	UWOPS87_Ator08_hmg124h_9.JPG.dat	802.5	65.8	1.0	0.08	0.024	0.077	1.4E-04
YLR401C	YLR401C	UWOPS87_Ator08_hmg124h_9.JPG.dat	888.3	67.8	1.1	0.09	0.122	0.093	1.7E-04
YLR402W	YLR402W	UWOPS87_Ator08_hmg124h_9.JPG.dat	922.3	42.5	1.0	0.04	0.230	0.041	1.6E-05
YLR404W	YLR404W	UWOPS87_Ator08_hmg124h_9.JPG.dat	819.8	16.4	0.9	0.02	0.093	0.016	1.4E-06
YLR405W	YLR405W	UWOPS87_Ator08_hmg124h_9.JPG.dat	938.8	43.2	1.1	0.05	0.042	0.050	2.7E-05
YLR406C	YLR406C	UWOPS87_Ator08_hmg124h_9.JPG.dat	813.8	26.3	1.0	0.03	-0.059	0.028	6.4E-06
YLR407W	YLR407W	UWOPS87_Ator08_hmg124h_9.JPG.dat	816.8	23.6	1.0	0.03	-0.021	0.029	6.9E-06
YLR408C	YLR408C	UWOPS87_Ator08_hmg124h_9.JPG.dat	772.8	24.4	0.9	0.03	-0.107	0.026	6.0E-06
YLR410W	YLR410W	UWOPS87_Ator08_hmg124h_9.JPG.dat	793.5	24.2	0.9	0.03	-0.035	0.028	7.6E-06
YLR412W	YLR412W	UWOPS87_Ator08_hmg124h_9.JPG.dat	856.3	73.8	1.0	0.09	-0.084	0.087	1.8E-04
YLR413W	YLR413W	UWOPS87_Ator08_hmg124h_9.JPG.dat	837.3	58.8	1.0	0.07	0.012	0.072	1.1E-04
YLR414C	YLR414C	UWOPS87_Ator08_hmg124h_9.JPG.dat	943.3	99.2	1.2	0.04	-0.035	0.037	3.2E-04
YOR202W	YOR202W	UWOPS87_Ator08_hmg124h_9.JPG.dat	913.6	122.0	1.0	0.09	-0.002	0.086	2.5E-293
1	1	UWOPS87_ctrl_hmg124h_1.JPG.dat	1537.3	269.4	1.0	0.11	#NUM!		3.4E-04
2	2	UWOPS87_ctrl_hmg124h_1.JPG.dat	1269.8	191.0	0.9	0.08	#NUM!		1.9E-04
3	3	UWOPS87_ctrl_hmg124h_1.JPG.dat	1283.3	202.5	0.9	0.09	#NUM!		2.4E-04
4	4	UWOPS87_ctrl_hmg124h_1.JPG.dat	1361.5	173.5	1.0	0.08	#NUM!		1.3E-04
YAL002W	YAL002W	UWOPS87_ctrl_hmg124h_1.JPG.dat	1156.3	63.1	1.1	0.07	#NUM!		7.8E-05
YAL004W	YAL004W	UWOPS87_ctrl_hmg124h_1.JPG.dat	1195.5	55.8	1.1	0.04	#NUM!		2.0E-05
YAL005C	YAL005C	UWOPS87_ctrl_hmg124h_1.JPG.dat	1206.0	74.4	1.0	0.07	#NUM!		8.2E-05
YAL007C	YAL007C	UWOPS87_ctrl_hmg124h_1.JPG.dat	1410.8	83.7	1.1	0.06	#NUM!		5.7E-05
YAL008W	YAL008W	UWOPS87_ctrl_hmg124h_1.JPG.dat	1312.3	71.3	1.0	0.06	#NUM!		5.1E-05
YAL010C	YAL010C	UWOPS87_ctrl_hmg124h_1.JPG.dat	1295.5	79.7	1.0	0.07	#NUM!		8.9E-05
YAL011W	YAL011W	UWOPS87_ctrl_hmg124h_1.JPG.dat	1419.8	62.3	1.1	0.05	#NUM!		2.3E-05
YAL013W	YAL013W	UWOPS87_ctrl_hmg124h_1.JPG.dat	1207.8	84.2	0.9	0.06	#NUM!		9.5E-05
YAL014C	YAL014C	UWOPS87_ctrl_hmg124h_1.JPG.dat	1381.8	55.6	1.1	0.05	#NUM!		2.4E-05
YAL015C	YAL015C	UWOPS87_ctrl_hmg124h_1.JPG.dat	1196.8	126.3	1.0	0.10	#NUM!		3.5E-04
YAL017W	YAL017W	UWOPS87_ctrl_hmg124h_1.JPG.dat	1063.0	46.3	0.9	0.05	#NUM!		3.3E-05
YAL018C	YAL018C	UWOPS87_ctrl_hmg124h_1.JPG.dat	1043.3	16.9	1.0	0.00	#NUM!		2.8E-07
YAL019W	YAL019W	UWOPS87_ctrl_hmg124h_1.JPG.dat	1182.0	140.6	1.1	0.12	#NUM!		3.6E-04
YAL020C	YAL020C	UWOPS87_ctrl_hmg124h_1.JPG.dat	1339.0	124.5	1.2	0.02	#NUM!		1.4E-04
YAL022C	YAL022C	UWOPS87_ctrl_hmg124h_1.JPG.dat	1312.8	136.0	1.0	0.10	#NUM!		2.8E-04
YAL023C	YAL023C	UWOPS87_ctrl_hmg124h_1.JPG.dat	1212.0	51.5	0.9	0.05	#NUM!		4.7E-05
YAL026C	YAL026C	UWOPS87_ctrl_hmg124h_1.JPG.dat	1361.8	94.2	1.1	0.02	#NUM!		1.7E-04

YAL027W	YAL027W	UWOPS87_ctrl_hmg124h_1.JPG.dat	1453.0	43.6	1.1	0.05	#NUM!	2.0E-05
YAL028W	YAL028W	UWOPS87_ctrl_hmg124h_1.JPG.dat	1366.5	81.8	1.1	0.01	#NUM!	6.2E-05
YAL029C	YAL029C	UWOPS87_ctrl_hmg124h_1.JPG.dat	1271.8	53.5	1.0	0.03	#NUM!	6.0E-06
YAL030W	YAL030W	UWOPS87_ctrl_hmg124h_1.JPG.dat	1323.0	119.4	1.1	0.11	#NUM!	2.5E-04
YAL031C	YAL031C	UWOPS87_ctrl_hmg124h_1.JPG.dat	1033.3	77.4	0.9	0.07	#NUM!	1.3E-04
YAL034C	YAL034C	UWOPS87_ctrl_hmg124h_1.JPG.dat	1055.3	49.3	1.0	0.02	#NUM!	9.0E-05
YAL036C	YAL036C	UWOPS87_ctrl_hmg124h_1.JPG.dat	1061.8	62.3	0.9	0.02	#NUM!	1.1E-04
YAL037W	YAL037W	UWOPS87_ctrl_hmg124h_1.JPG.dat	1197.3	77.6	1.0	0.07	#NUM!	8.6E-05
YAL040C	YAL040C	UWOPS87_ctrl_hmg124h_1.JPG.dat	1367.5	29.9	1.0	0.02	#NUM!	1.6E-06
YAL042W	YAL042W	UWOPS87_ctrl_hmg124h_1.JPG.dat	1347.8	66.7	1.0	0.05	#NUM!	3.9E-05
YAL043C-A	YAL043C-A	UWOPS87_ctrl_hmg124h_1.JPG.dat	1350.0	52.5	1.0	0.04	#NUM!	2.2E-05
YAL045C	YAL045C	UWOPS87_ctrl_hmg124h_1.JPG.dat	1228.8	23.9	0.9	0.02	#NUM!	2.3E-06
YAL049C	YAL049C	UWOPS87_ctrl_hmg124h_1.JPG.dat	1254.3	92.7	0.9	0.07	#NUM!	1.1E-04
YAL051W	YAL051W	UWOPS87_ctrl_hmg124h_1.JPG.dat	1334.3	118.2	1.0	0.10	#NUM!	2.3E-04
YAL053W	YAL053W	UWOPS87_ctrl_hmg124h_1.JPG.dat	1373.5	80.1	1.1	0.07	#NUM!	5.9E-05
YAL054C	YAL054C	UWOPS87_ctrl_hmg124h_1.JPG.dat	1159.0	54.9	1.0	0.04	#NUM!	2.1E-05
YAL055W	YAL055W	UWOPS87_ctrl_hmg124h_1.JPG.dat	1047.3	60.4	1.0	0.07	#NUM!	8.5E-05
YAL056W	YAL056W	UWOPS87_ctrl_hmg124h_1.JPG.dat	1362.5	44.5	1.2	0.03	#NUM!	4.3E-06
YAL058C-A	YAL058C-A	UWOPS87_ctrl_hmg124h_1.JPG.dat	1190.5	52.9	1.0	0.05	#NUM!	2.8E-05
YAL058W	YAL058W	UWOPS87_ctrl_hmg124h_1.JPG.dat	1332.8	25.9	1.0	0.02	#NUM!	1.9E-06
YAL059W	YAL059W	UWOPS87_ctrl_hmg124h_1.JPG.dat	1374.8	14.5	1.0	0.01	#NUM!	8.3E-06
YAL060W	YAL060W	UWOPS87_ctrl_hmg124h_1.JPG.dat	1323.8	120.0	1.0	0.08	#NUM!	1.7E-04
YAL061W	YAL061W	UWOPS87_ctrl_hmg124h_1.JPG.dat	1350.0	114.3	1.0	0.09	#NUM!	1.7E-04
YAL062W	YAL062W	UWOPS87_ctrl_hmg124h_1.JPG.dat	1402.0	28.6	1.1	0.02	#NUM!	2.1E-06
YAL064C-A	YAL064C-A	UWOPS87_ctrl_hmg124h_1.JPG.dat	1357.0	57.3	1.0	0.05	#NUM!	2.6E-05
YAL065C	YAL065C	UWOPS87_ctrl_hmg124h_1.JPG.dat	967.5	207.3	0.8	0.16	#NUM!	2.4E-03
YAL066W	YAL066W	UWOPS87_ctrl_hmg124h_1.JPG.dat	1118.5	34.8	1.0	0.03	#NUM!	8.9E-06
YAL067C	YAL067C	UWOPS87_ctrl_hmg124h_1.JPG.dat	1327.8	81.5	1.2	0.10	#NUM!	1.5E-04
YAL068C	YAL068C	UWOPS87_ctrl_hmg124h_1.JPG.dat	1135.8	81.3	0.9	0.02	#NUM!	2.1E-04
YAR002C-A	YAR002C-A	UWOPS87_ctrl_hmg124h_1.JPG.dat	1333.3	79.0	1.1	0.07	#NUM!	7.1E-05
YAR002W	YAR002W	UWOPS87_ctrl_hmg124h_1.JPG.dat	1307.0	92.4	1.0	0.07	#NUM!	1.1E-04
YAR003W	YAR003W	UWOPS87_ctrl_hmg124h_1.JPG.dat	1422.5	65.2	1.0	0.01	#NUM!	5.5E-05
YAR014C	YAR014C	UWOPS87_ctrl_hmg124h_1.JPG.dat	1399.3	69.4	1.0	0.06	#NUM!	4.6E-05
YAR015W	YAR015W	UWOPS87_ctrl_hmg124h_1.JPG.dat	1263.0	49.9	1.0	0.01	#NUM!	3.1E-05
YAR018C	YAR018C	UWOPS87_ctrl_hmg124h_1.JPG.dat	1101.3	114.3	0.9	0.08	#NUM!	2.5E-04
YAR020C	YAR020C	UWOPS87_ctrl_hmg124h_1.JPG.dat	1338.5	77.1	1.1	0.02	#NUM!	9.5E-05
YAR023C	YAR023C	UWOPS87_ctrl_hmg124h_1.JPG.dat	1373.8	77.7	1.1	0.01	#NUM!	3.0E-05
YAR027W	YAR027W	UWOPS87_ctrl_hmg124h_1.JPG.dat	1094.5	9.9	1.0	0.01	#NUM!	2.3E-07
YAR028W	YAR028W	UWOPS87_ctrl_hmg124h_1.JPG.dat	947.8	165.4	0.8	0.13	#NUM!	1.1E-03
YAR029W	YAR029W	UWOPS87_ctrl_hmg124h_1.JPG.dat	1246.0	66.1	1.1	0.05	#NUM!	2.7E-05
YAR030C	YAR030C	UWOPS87_ctrl_hmg124h_1.JPG.dat	1203.8	79.1	1.0	0.06	#NUM!	5.9E-05
YAR031W	YAR031W	UWOPS87_ctrl_hmg124h_1.JPG.dat	1323.8	75.8	1.0	0.08	#NUM!	1.2E-04
YAR035W	YAR035W	UWOPS87_ctrl_hmg124h_1.JPG.dat	1161.5	108.9	0.9	0.07	#NUM!	1.4E-04
YAR037W	YAR037W	UWOPS87_ctrl_hmg124h_1.JPG.dat	1373.8	107.8	1.1	0.02	#NUM!	1.6E-04
YAR040C	YAR040C	UWOPS87_ctrl_hmg124h_1.JPG.dat	1149.0	192.3	0.8	0.05	#NUM!	1.3E-03
YAR042W	YAR042W	UWOPS87_ctrl_hmg124h_1.JPG.dat	1319.5	77.4	1.0	0.06	#NUM!	4.9E-05
YAR043C	YAR043C	UWOPS87_ctrl_hmg124h_1.JPG.dat	703.3	28.1	0.6	0.02	#NUM!	6.4E-06
YAR044W	YAR044W	UWOPS87_ctrl_hmg124h_1.JPG.dat	927.8	237.8	0.8	0.20	#NUM!	4.9E-03
YAR047C	YAR047C	UWOPS87_ctrl_hmg124h_1.JPG.dat	833.3	132.1	0.7	0.12	#NUM!	1.0E-03
YAR050W	YAR050W	UWOPS87_ctrl_hmg124h_1.JPG.dat	1080.5	49.9	1.0	0.05	#NUM!	3.5E-05
YBL001C	YBL001C	UWOPS87_ctrl_hmg124h_1.JPG.dat	1151.0	39.2	1.0	0.05	#NUM!	2.4E-05
YBL003C	YBL003C	UWOPS87_ctrl_hmg124h_1.JPG.dat	1001.3	41.5	0.9	0.01	#NUM!	3.7E-05
YBL005W	YBL005W	UWOPS87_ctrl_hmg124h_1.JPG.dat	1412.0	126.7	1.2	0.11	#NUM!	2.4E-04
YBL007C	YBL007C	UWOPS87_ctrl_hmg124h_1.JPG.dat	1208.5	93.5	1.0	0.06	#NUM!	7.1E-05
YBL008W	YBL008W	UWOPS87_ctrl_hmg124h_1.JPG.dat	1301.5	43.0	1.1	0.01	#NUM!	1.8E-05
YBL009W	YBL009W	UWOPS87_ctrl_hmg124h_1.JPG.dat	1104.0	56.3	1.0	0.06	#NUM!	6.4E-05
YBL010C	YBL010C	UWOPS87_ctrl_hmg124h_1.JPG.dat	1483.8	202.8	1.4	0.01	#NUM!	9.8E-06
YBL011W	YBL011W	UWOPS87_ctrl_hmg124h_1.JPG.dat	1305.3	173.2	1.1	0.14	#NUM!	5.0E-04
YBL013W	YBL013W	UWOPS87_ctrl_hmg124h_1.JPG.dat	883.0	285.3	0.8	0.24	#NUM!	7.7E-03
YBL015W	YBL015W	UWOPS87_ctrl_hmg124h_1.JPG.dat	1052.3	159.4	1.0	0.14	#NUM!	8.2E-04
YBL016W	YBL016W	UWOPS87_ctrl_hmg124h_1.JPG.dat	1131.8	149.2	1.0	0.03	#NUM!	3.3E-04
YBL017C	YBL017C	UWOPS87_ctrl_hmg124h_1.JPG.dat	1010.3	39.8	0.9	0.04	#NUM!	2.5E-05
YBL019W	YBL019W	UWOPS87_ctrl_hmg124h_1.JPG.dat	1071.3	49.0	0.9	0.01	#NUM!	5.1E-05
YBL021C	YBL021C	UWOPS87_ctrl_hmg124h_1.JPG.dat	1312.0	41.7	1.1	0.03	#NUM!	4.9E-06
YBL024W	YBL024W	UWOPS87_ctrl_hmg124h_1.JPG.dat	1124.5	65.2	0.9	0.05	#NUM!	5.4E-05
YBL025W	YBL025W	UWOPS87_ctrl_hmg124h_1.JPG.dat	1214.0	25.9	1.0	0.01	#NUM!	1.3E-05
YBL027W	YBL027W	UWOPS87_ctrl_hmg124h_1.JPG.dat	1243.3	42.1	1.0	0.04	#NUM!	1.5E-05
YBL028C	YBL028C	UWOPS87_ctrl_hmg124h_1.JPG.dat	1442.3	26.8	1.1	0.02	#NUM!	1.8E-06
YBL029W	YBL029W	UWOPS87_ctrl_hmg124h_1.JPG.dat	1395.8	60.6	1.1	0.04	#NUM!	1.9E-05
YBL031W	YBL031W	UWOPS87_ctrl_hmg124h_1.JPG.dat	1138.3	62.6	0.9	0.05	#NUM!	4.8E-05

YBL032W	YBL032W	UWOPS87_ctrl_hmg124h_1.JPG.dat	1402.0	78.5	1.2	0.06	#NUM!	4.8E-05
YBL036C	YBL036C	UWOPS87_ctrl_hmg124h_1.JPG.dat	1018.8	25.4	1.0	0.01	#NUM!	9.7E-07
YBL037W	YBL037W	UWOPS87_ctrl_hmg124h_1.JPG.dat	1042.5	28.7	1.0	0.03	#NUM!	7.6E-06
YBL039C	YBL039C	UWOPS87_ctrl_hmg124h_1.JPG.dat	1001.8	82.5	0.9	0.06	#NUM!	1.0E-04
YBL042C	YBL042C	UWOPS87_ctrl_hmg124h_1.JPG.dat	1181.8	61.2	1.0	0.06	#NUM!	7.4E-05
YBL043W	YBL043W	UWOPS87_ctrl_hmg124h_1.JPG.dat	1323.3	118.3	1.0	0.00	#NUM!	1.5E-06
YBL046W	YBL046W	UWOPS87_ctrl_hmg124h_1.JPG.dat	1271.0	23.7	1.0	0.03	#NUM!	6.3E-06
YBL047C	YBL047C	UWOPS87_ctrl_hmg124h_1.JPG.dat	1267.5	69.7	1.0	0.05	#NUM!	4.9E-05
YBL048W	YBL048W	UWOPS87_ctrl_hmg124h_1.JPG.dat	1406.0	26.2	1.1	0.03	#NUM!	4.6E-06
YBL049W	YBL049W	UWOPS87_ctrl_hmg124h_1.JPG.dat	1328.8	55.7	1.0	0.04	#NUM!	1.2E-05
YBL051C	YBL051C	UWOPS87_ctrl_hmg124h_1.JPG.dat	1287.5	20.8	1.0	0.00	#NUM!	7.8E-07
YBL052C	YBL052C	UWOPS87_ctrl_hmg124h_1.JPG.dat	1041.8	94.9	0.8	0.00	#NUM!	6.7E-06
YBL053W	YBL053W	UWOPS87_ctrl_hmg124h_1.JPG.dat	1326.8	117.5	1.2	0.11	#NUM!	1.8E-04
YBL054W	YBL054W	UWOPS87_ctrl_hmg124h_1.JPG.dat	1211.0	136.7	1.1	0.12	#NUM!	3.7E-04
YBL055C	YBL055C	UWOPS87_ctrl_hmg124h_1.JPG.dat	1291.3	65.8	1.1	0.05	#NUM!	2.8E-05
YBL056W	YBL056W	UWOPS87_ctrl_hmg124h_1.JPG.dat	1268.8	54.0	1.0	0.04	#NUM!	1.3E-05
YBL057C	YBL057C	UWOPS87_ctrl_hmg124h_1.JPG.dat	1320.5	116.4	1.0	0.08	#NUM!	1.5E-04
YBL059W	YBL059W	UWOPS87_ctrl_hmg124h_1.JPG.dat	1310.3	36.3	1.0	0.01	#NUM!	1.8E-05
YBL060W	YBL060W	UWOPS87_ctrl_hmg124h_1.JPG.dat	1399.5	38.0	1.0	0.03	#NUM!	7.8E-06
YBL061C	YBL061C	UWOPS87_ctrl_hmg124h_1.JPG.dat	1253.0	82.6	0.9	0.06	#NUM!	8.6E-05
YBL062W	YBL062W	UWOPS87_ctrl_hmg124h_1.JPG.dat	1359.8	27.4	1.0	0.02	#NUM!	1.5E-06
YBL063W	YBL063W	UWOPS87_ctrl_hmg124h_1.JPG.dat	1249.5	50.1	1.0	0.01	#NUM!	2.8E-05
YBL064C	YBL064C	UWOPS87_ctrl_hmg124h_1.JPG.dat	1216.8	124.0	1.0	0.03	#NUM!	4.0E-04
YBL065W	YBL065W	UWOPS87_ctrl_hmg124h_1.JPG.dat	1016.0	19.7	0.9	0.02	#NUM!	2.2E-06
YBL066C	YBL066C	UWOPS87_ctrl_hmg124h_1.JPG.dat	1113.0	17.6	1.0	0.02	#NUM!	1.3E-06
YBL067C	YBL067C	UWOPS87_ctrl_hmg124h_1.JPG.dat	1053.3	47.1	0.9	0.03	#NUM!	1.2E-05
YBL068W	YBL068W	UWOPS87_ctrl_hmg124h_1.JPG.dat	1310.0	50.4	1.0	0.03	#NUM!	7.1E-06
YBL069W	YBL069W	UWOPS87_ctrl_hmg124h_1.JPG.dat	1270.5	28.1	0.9	0.02	#NUM!	4.1E-06
YBL070C	YBL070C	UWOPS87_ctrl_hmg124h_1.JPG.dat	1427.3	84.0	1.1	0.06	#NUM!	5.4E-05
YBL071C	YBL071C	UWOPS87_ctrl_hmg124h_1.JPG.dat	1313.8	112.6	1.0	0.09	#NUM!	2.2E-04
YBL072C	YBL072C	UWOPS87_ctrl_hmg124h_1.JPG.dat	1324.0	48.7	1.0	0.04	#NUM!	1.3E-05
YBL075C	YBL075C	UWOPS87_ctrl_hmg124h_1.JPG.dat	1382.5	29.9	1.0	0.02	#NUM!	2.8E-06
YBL078C	YBL078C	UWOPS87_ctrl_hmg124h_1.JPG.dat	1270.3	108.1	1.0	0.08	#NUM!	1.3E-04
YBL079W	YBL079W	UWOPS87_ctrl_hmg124h_1.JPG.dat	1216.3	115.5	1.0	0.11	#NUM!	3.7E-04
YBL081W	YBL081W	UWOPS87_ctrl_hmg124h_1.JPG.dat	1096.3	133.7	1.0	0.11	#NUM!	4.1E-04
YBL082C	YBL082C	UWOPS87_ctrl_hmg124h_1.JPG.dat	1288.8	115.2	1.1	0.11	#NUM!	2.9E-04
YBL083C	YBL083C	UWOPS87_ctrl_hmg124h_1.JPG.dat	1313.5	85.4	1.1	0.06	#NUM!	5.0E-05
YBL085W	YBL085W	UWOPS87_ctrl_hmg124h_1.JPG.dat	1182.5	77.7	0.9	0.07	#NUM!	1.3E-04
YBL086C	YBL086C	UWOPS87_ctrl_hmg124h_1.JPG.dat	1369.8	97.7	1.0	0.07	#NUM!	7.9E-05
YBL087C	YBL087C	UWOPS87_ctrl_hmg124h_1.JPG.dat	1111.8	15.5	0.9	0.01	#NUM!	9.5E-07
YBL088C	YBL088C	UWOPS87_ctrl_hmg124h_1.JPG.dat	1360.8	56.4	1.1	0.02	#NUM!	9.7E-05
YBL089W	YBL089W	UWOPS87_ctrl_hmg124h_1.JPG.dat	1294.8	39.4	1.0	0.03	#NUM!	6.0E-06
YBL091C	YBL091C	UWOPS87_ctrl_hmg124h_1.JPG.dat	1275.3	68.9	1.0	0.02	#NUM!	1.2E-04
YBL091C-A	YBL091C-A	UWOPS87_ctrl_hmg124h_1.JPG.dat	1282.5	147.3	1.1	0.04	#NUM!	4.8E-04
YBL094C	YBL094C	UWOPS87_ctrl_hmg124h_1.JPG.dat	1092.5	196.7	0.8	0.05	#NUM!	1.2E-03
YBL095W	YBL095W	UWOPS87_ctrl_hmg124h_1.JPG.dat	1176.8	202.3	0.9	0.05	#NUM!	1.1E-03
YBL096C	YBL096C	UWOPS87_ctrl_hmg124h_1.JPG.dat	1331.3	43.9	1.1	0.01	#NUM!	2.4E-05
YBL098W	YBL098W	UWOPS87_ctrl_hmg124h_1.JPG.dat	957.8	55.3	0.8	0.06	#NUM!	1.3E-04
YBL100C	YBL100C	UWOPS87_ctrl_hmg124h_1.JPG.dat	1403.0	115.4	1.1	0.08	#NUM!	8.6E-05
YBL101C	YBL101C	UWOPS87_ctrl_hmg124h_1.JPG.dat	1453.5	23.6	1.1	0.03	#NUM!	7.4E-06
YBL102W	YBL102W	UWOPS87_ctrl_hmg124h_1.JPG.dat	1129.8	63.7	0.9	0.01	#NUM!	8.4E-05
YBL103C	YBL103C	UWOPS87_ctrl_hmg124h_1.JPG.dat	1271.3	23.7	1.0	0.04	#NUM!	1.4E-05
YBL104C	YBL104C	UWOPS87_ctrl_hmg124h_1.JPG.dat	1316.8	53.3	1.0	0.04	#NUM!	1.1E-05
YBL106C	YBL106C	UWOPS87_ctrl_hmg124h_1.JPG.dat	1252.0	100.6	1.0	0.06	#NUM!	7.4E-05
YBL107C	YBL107C	UWOPS87_ctrl_hmg124h_1.JPG.dat	1190.0	127.3	1.0	0.11	#NUM!	4.0E-04
YBR001C	YBR001C	UWOPS87_ctrl_hmg124h_1.JPG.dat	1209.5	41.3	1.0	0.01	#NUM!	7.9E-06
YBR005W	YBR005W	UWOPS87_ctrl_hmg124h_1.JPG.dat	1140.8	87.0	1.0	0.06	#NUM!	6.8E-05
YBR006W	YBR006W	UWOPS87_ctrl_hmg124h_1.JPG.dat	1305.5	104.6	1.1	0.09	#NUM!	1.2E-04
YBR007C	YBR007C	UWOPS87_ctrl_hmg124h_1.JPG.dat	1056.8	30.1	0.9	0.03	#NUM!	1.0E-05
YBR008C	YBR008C	UWOPS87_ctrl_hmg124h_1.JPG.dat	968.5	35.1	0.8	0.01	#NUM!	2.3E-05
YBR009C	YBR009C	UWOPS87_ctrl_hmg124h_1.JPG.dat	1219.5	48.8	1.0	0.05	#NUM!	3.7E-05
YBR010W	YBR010W	UWOPS87_ctrl_hmg124h_1.JPG.dat	1256.5	172.3	1.1	0.15	#NUM!	6.8E-04
YBR012C	YBR012C	UWOPS87_ctrl_hmg124h_1.JPG.dat	1007.5	119.5	0.9	0.09	#NUM!	3.3E-04
YBR013C	YBR013C	UWOPS87_ctrl_hmg124h_1.JPG.dat	1052.8	44.6	0.9	0.05	#NUM!	4.3E-05
YBR014C	YBR014C	UWOPS87_ctrl_hmg124h_1.JPG.dat	1262.5	169.9	1.1	0.15	#NUM!	6.9E-04
YBR015C	YBR015C	UWOPS87_ctrl_hmg124h_1.JPG.dat	1212.8	181.6	1.1	0.15	#NUM!	7.2E-04
YBR016W	YBR016W	UWOPS87_ctrl_hmg124h_1.JPG.dat	1135.8	101.3	1.0	0.08	#NUM!	1.1E-04
YBR018C	YBR018C	UWOPS87_ctrl_hmg124h_1.JPG.dat	984.0	76.2	1.0	0.01	#NUM!	2.3E-05
YBR019C	YBR019C	UWOPS87_ctrl_hmg124h_1.JPG.dat	1050.0	31.6	0.9	0.02	#NUM!	2.6E-06
YBR020W	YBR020W	UWOPS87_ctrl_hmg124h_1.JPG.dat	1141.8	40.5	1.0	0.03	#NUM!	5.5E-06

YBR022W	YBR022W	UWOPS87_ctrl_hmg124h_1.JPG.dat	1086.0	67.7	0.9	0.05	#NUM!	5.8E-05
YBR023C	YBR023C	UWOPS87_ctrl_hmg124h_1.JPG.dat	1391.8	181.0	1.1	0.14	#NUM!	5.9E-04
YBR024W	YBR024W	UWOPS87_ctrl_hmg124h_1.JPG.dat	1260.8	65.9	1.0	0.02	#NUM!	1.2E-04
YBR025C	YBR025C	UWOPS87_ctrl_hmg124h_1.JPG.dat	1243.8	23.2	1.0	0.01	#NUM!	9.2E-06
YBR026C	YBR026C	UWOPS87_ctrl_hmg124h_1.JPG.dat	1230.8	24.0	1.0	0.02	#NUM!	4.3E-06
YBR027C	YBR027C	UWOPS87_ctrl_hmg124h_1.JPG.dat	1213.5	108.3	1.0	0.09	#NUM!	2.5E-04
YBR028C	YBR028C	UWOPS87_ctrl_hmg124h_1.JPG.dat	1171.0	23.7	1.0	0.02	#NUM!	1.2E-06
YBR030W	YBR030W	UWOPS87_ctrl_hmg124h_1.JPG.dat	1136.0	26.2	1.0	0.03	#NUM!	1.1E-05
YBR031W	YBR031W	UWOPS87_ctrl_hmg124h_1.JPG.dat	851.8	96.4	0.8	0.09	#NUM!	3.4E-04
YBR032W	YBR032W	UWOPS87_ctrl_hmg124h_1.JPG.dat	1020.5	37.9	1.0	0.01	#NUM!	3.3E-05
YBR033W	YBR033W	UWOPS87_ctrl_hmg124h_1.JPG.dat	1075.8	100.3	0.9	0.03	#NUM!	3.6E-04
YBR034C	YBR034C	UWOPS87_ctrl_hmg124h_1.JPG.dat	1326.0	78.0	1.0	0.06	#NUM!	4.3E-05
YBR036C	YBR036C	UWOPS87_ctrl_hmg124h_1.JPG.dat	1466.3	54.6	1.1	0.04	#NUM!	1.2E-05
YBR040W	YBR040W	UWOPS87_ctrl_hmg124h_1.JPG.dat	1340.3	80.6	1.0	0.06	#NUM!	6.1E-05
YBR041W	YBR041W	UWOPS87_ctrl_hmg124h_1.JPG.dat	1347.5	79.2	1.0	0.06	#NUM!	4.1E-05
YBR042C	YBR042C	UWOPS87_ctrl_hmg124h_1.JPG.dat	1324.0	20.7	1.0	0.00	#NUM!	7.3E-06
YBR043C	YBR043C	UWOPS87_ctrl_hmg124h_1.JPG.dat	1216.3	57.8	1.0	0.04	#NUM!	1.7E-05
YBR044C	YBR044C	UWOPS87_ctrl_hmg124h_1.JPG.dat	1247.8	100.0	1.0	0.08	#NUM!	1.5E-04
YBR045C	YBR045C	UWOPS87_ctrl_hmg124h_1.JPG.dat	1074.8	84.7	0.9	0.02	#NUM!	1.8E-04
YBR046C	YBR046C	UWOPS87_ctrl_hmg124h_1.JPG.dat	1047.3	55.3	1.0	0.05	#NUM!	2.5E-05
YBR047W	YBR047W	UWOPS87_ctrl_hmg124h_1.JPG.dat	1386.5	251.5	1.3	0.22	#NUM!	1.4E-03
YBR048W	YBR048W	UWOPS87_ctrl_hmg124h_1.JPG.dat	993.0	38.0	0.8	0.04	#NUM!	4.2E-05
YBR050C	YBR050C	UWOPS87_ctrl_hmg124h_1.JPG.dat	1297.3	53.5	1.0	0.01	#NUM!	6.3E-05
YBR051W	YBR051W	UWOPS87_ctrl_hmg124h_1.JPG.dat	1360.8	83.4	1.0	0.07	#NUM!	9.7E-05
YBR052C	YBR052C	UWOPS87_ctrl_hmg124h_1.JPG.dat	1325.5	58.3	1.0	0.01	#NUM!	1.7E-05
YBR053C	YBR053C	UWOPS87_ctrl_hmg124h_1.JPG.dat	1310.3	53.0	1.0	0.04	#NUM!	1.7E-05
YBR054W	YBR054W	UWOPS87_ctrl_hmg124h_1.JPG.dat	1336.3	63.0	1.0	0.05	#NUM!	2.8E-05
YBR056W	YBR056W	UWOPS87_ctrl_hmg124h_1.JPG.dat	1302.8	29.2	1.0	0.02	#NUM!	3.2E-06
YBR057C	YBR057C	UWOPS87_ctrl_hmg124h_1.JPG.dat	1296.0	106.0	1.0	0.09	#NUM!	1.5E-04
YBR058C	YBR058C	UWOPS87_ctrl_hmg124h_1.JPG.dat	1317.0	111.2	1.2	0.10	#NUM!	1.6E-04
YBR059C	YBR059C	UWOPS87_ctrl_hmg124h_1.JPG.dat	894.5	22.2	0.8	0.01	#NUM!	3.2E-05
YBR061C	YBR061C	UWOPS87_ctrl_hmg124h_1.JPG.dat	1089.0	94.7	1.0	0.08	#NUM!	1.6E-04
YBR062C	YBR062C	UWOPS87_ctrl_hmg124h_1.JPG.dat	1255.3	32.8	1.0	0.01	#NUM!	1.2E-05
YBR063C	YBR063C	UWOPS87_ctrl_hmg124h_1.JPG.dat	1342.0	78.5	1.0	0.05	#NUM!	3.7E-05
YBR064W	YBR064W	UWOPS87_ctrl_hmg124h_1.JPG.dat	1313.3	33.9	1.0	0.02	#NUM!	4.0E-06
YBR065C	YBR065C	UWOPS87_ctrl_hmg124h_1.JPG.dat	1384.8	59.0	1.1	0.01	#NUM!	1.2E-05
YBR066C	YBR066C	UWOPS87_ctrl_hmg124h_1.JPG.dat	1434.0	101.5	1.1	0.03	#NUM!	1.7E-04
YBR067C	YBR067C	UWOPS87_ctrl_hmg124h_1.JPG.dat	1339.0	27.8	1.0	0.02	#NUM!	3.7E-06
YBR068C	YBR068C	UWOPS87_ctrl_hmg124h_1.JPG.dat	1235.8	24.0	1.0	0.02	#NUM!	4.6E-06
YBR069C	YBR069C	UWOPS87_ctrl_hmg124h_1.JPG.dat	1308.5	97.2	1.1	0.07	#NUM!	9.2E-05
YBR071W	YBR071W	UWOPS87_ctrl_hmg124h_1.JPG.dat	1054.0	26.9	0.9	0.03	#NUM!	5.4E-06
YBR072W	YBR072W	UWOPS87_ctrl_hmg124h_1.JPG.dat	1043.0	112.4	1.0	0.09	#NUM!	2.4E-04
YBR073W	YBR073W	UWOPS87_ctrl_hmg124h_1.JPG.dat	1128.5	45.5	1.0	0.01	#NUM!	1.1E-05
YBR074W	YBR074W	UWOPS87_ctrl_hmg124h_1.JPG.dat	1315.5	77.1	1.1	0.06	#NUM!	5.3E-05
YBR075W	YBR075W	UWOPS87_ctrl_hmg124h_1.JPG.dat	1309.0	26.6	1.0	0.00	#NUM!	2.4E-06
YBR076W	YBR076W	UWOPS87_ctrl_hmg124h_1.JPG.dat	1353.5	144.8	1.0	0.10	#NUM!	2.4E-04
YBR077C	YBR077C	UWOPS87_ctrl_hmg124h_1.JPG.dat	1539.3	47.8	1.2	0.03	#NUM!	3.0E-06
YBR082C	YBR082C	UWOPS87_ctrl_hmg124h_1.JPG.dat	865.0	577.9	0.9	0.05	#NUM!	8.6E-04
YBR083W	YBR083W	UWOPS87_ctrl_hmg124h_1.JPG.dat	1262.5	77.8	1.0	0.03	#NUM!	2.3E-04
YBR084C-A	YBR084C-A	UWOPS87_ctrl_hmg124h_1.JPG.dat	939.0	10.9	0.7	0.02	#NUM!	2.6E-06
YBR084W	YBR084W	UWOPS87_ctrl_hmg124h_1.JPG.dat	1313.0	58.6	1.1	0.04	#NUM!	1.1E-05
YBR090C	YBR090C	UWOPS87_ctrl_hmg124h_1.JPG.dat	1048.5	35.8	1.0	0.01	#NUM!	2.1E-05
YBR090C-A	YBR090C-A	UWOPS87_ctrl_hmg124h_1.JPG.dat	1324.5	49.5	1.2	0.06	#NUM!	3.7E-05
YBR092C	YBR092C	UWOPS87_ctrl_hmg124h_1.JPG.dat	1110.5	81.8	1.0	0.02	#NUM!	1.8E-04
YBR093C	YBR093C	UWOPS87_ctrl_hmg124h_1.JPG.dat	1002.0	36.7	0.8	0.00	#NUM!	2.2E-06
YBR094W	YBR094W	UWOPS87_ctrl_hmg124h_1.JPG.dat	1206.0	41.1	0.9	0.04	#NUM!	2.0E-05
YBR095C	YBR095C	UWOPS87_ctrl_hmg124h_1.JPG.dat	1423.3	30.1	1.1	0.02	#NUM!	2.0E-06
YBR098W	YBR098W	UWOPS87_ctrl_hmg124h_1.JPG.dat	1236.8	43.7	0.9	0.03	#NUM!	9.6E-06
YBR099C	YBR099C	UWOPS87_ctrl_hmg124h_1.JPG.dat	1217.8	27.8	1.0	0.02	#NUM!	1.6E-06
YBR100W	YBR100W	UWOPS87_ctrl_hmg124h_1.JPG.dat	1201.0	23.8	1.0	0.02	#NUM!	1.3E-06
YBR101C	YBR101C	UWOPS87_ctrl_hmg124h_1.JPG.dat	1233.3	64.1	1.0	0.05	#NUM!	4.2E-05
YBR103W	YBR103W	UWOPS87_ctrl_hmg124h_1.JPG.dat	1280.0	113.5	1.1	0.09	#NUM!	1.8E-04
YBR104W	YBR104W	UWOPS87_ctrl_hmg124h_1.JPG.dat	1051.5	75.9	0.9	0.06	#NUM!	8.8E-05
YBR105C	YBR105C	UWOPS87_ctrl_hmg124h_1.JPG.dat	1163.8	25.4	1.0	0.02	#NUM!	2.8E-06
YBR106W	YBR106W	UWOPS87_ctrl_hmg124h_1.JPG.dat	957.5	47.7	0.8	0.04	#NUM!	4.0E-05
YBR107C	YBR107C	UWOPS87_ctrl_hmg124h_1.JPG.dat	1148.5	49.3	0.9	0.01	#NUM!	3.1E-05
YBR108W	YBR108W	UWOPS87_ctrl_hmg124h_1.JPG.dat	1265.5	153.7	1.0	0.11	#NUM!	3.8E-04
YBR111C	YBR111C	UWOPS87_ctrl_hmg124h_1.JPG.dat	1391.3	52.4	1.0	0.04	#NUM!	2.0E-05
YBR113W	YBR113W	UWOPS87_ctrl_hmg124h_1.JPG.dat	1423.8	106.5	1.1	0.07	#NUM!	7.2E-05
YBR114W	YBR114W	UWOPS87_ctrl_hmg124h_1.JPG.dat	1198.0	95.7	0.9	0.07	#NUM!	1.1E-04

YBR115C	YBR115C	UWOPS87_ctrl_hmg124h_1.JPG.dat	0.0	0.0	0.0	0.00	#NUM!		
YBR116C	YBR116C	UWOPS87_ctrl_hmg124h_1.JPG.dat	1414.8	111.5	1.1	0.09	#NUM!		1.6E-04
YBR119W	YBR119W	UWOPS87_ctrl_hmg124h_1.JPG.dat	1113.5	56.5	0.9	0.04	#NUM!		2.5E-05
YBR121C	YBR121C	UWOPS87_ctrl_hmg124h_1.JPG.dat	1221.5	127.3	1.0	0.10	#NUM!		2.7E-04
YBR122C	YBR122C	UWOPS87_ctrl_hmg124h_1.JPG.dat	1191.0	60.5	1.1	0.07	#NUM!		6.7E-05
YBR125C	YBR125C	UWOPS87_ctrl_hmg124h_1.JPG.dat	1010.5	48.0	0.9	0.06	#NUM!		6.7E-05
YBR126C	YBR126C	UWOPS87_ctrl_hmg124h_1.JPG.dat	1259.0	53.0	1.1	0.01	#NUM!		4.9E-05
YBR128C	YBR128C	UWOPS87_ctrl_hmg124h_1.JPG.dat	1316.8	68.7	1.1	0.06	#NUM!		4.9E-05
YBR129C	YBR129C	UWOPS87_ctrl_hmg124h_1.JPG.dat	1237.5	38.4	1.0	0.03	#NUM!		1.1E-05
YBR130C	YBR130C	UWOPS87_ctrl_hmg124h_1.JPG.dat	1229.0	30.2	1.0	0.02	#NUM!		3.0E-06
YBR131W	YBR131W	UWOPS87_ctrl_hmg124h_1.JPG.dat	1298.0	36.2	1.0	0.01	#NUM!		3.4E-05
YBR132C	YBR132C	UWOPS87_ctrl_hmg124h_1.JPG.dat	1254.3	55.1	1.0	0.00	#NUM!		3.1E-06
YBR134W	YBR134W	UWOPS87_ctrl_hmg124h_1.JPG.dat	1399.0	71.1	1.1	0.05	#NUM!		2.5E-05
YBR137W	YBR137W	UWOPS87_ctrl_hmg124h_1.JPG.dat	1322.0	140.0	1.1	0.02	#NUM!		1.4E-04
YBR138C	YBR138C	UWOPS87_ctrl_hmg124h_1.JPG.dat	1187.8	58.5	1.0	0.01	#NUM!		3.3E-05
YBR139W	YBR139W	UWOPS87_ctrl_hmg124h_1.JPG.dat	960.5	50.3	0.9	0.05	#NUM!		4.8E-05
YBR141C	YBR141C	UWOPS87_ctrl_hmg124h_1.JPG.dat	1168.0	101.1	1.1	0.10	#NUM!		2.1E-04
YBR144C	YBR144C	UWOPS87_ctrl_hmg124h_1.JPG.dat	1237.5	56.6	1.1	0.05	#NUM!		2.7E-05
YBR145W	YBR145W	UWOPS87_ctrl_hmg124h_1.JPG.dat	1209.0	105.1	1.0	0.08	#NUM!		1.5E-04
YBR146W	YBR146W	UWOPS87_ctrl_hmg124h_1.JPG.dat	1356.5	52.6	1.1	0.04	#NUM!		1.4E-05
YBR147W	YBR147W	UWOPS87_ctrl_hmg124h_1.JPG.dat	1291.5	92.2	1.0	0.07	#NUM!		7.3E-05
YBR148W	YBR148W	UWOPS87_ctrl_hmg124h_1.JPG.dat	1260.8	66.6	1.0	0.06	#NUM!		5.8E-05
YBR149W	YBR149W	UWOPS87_ctrl_hmg124h_1.JPG.dat	1351.3	73.8	1.1	0.01	#NUM!		1.4E-05
YBR150C	YBR150C	UWOPS87_ctrl_hmg124h_1.JPG.dat	1242.8	71.8	1.0	0.06	#NUM!		5.5E-05
YBR151W	YBR151W	UWOPS87_ctrl_hmg124h_1.JPG.dat	1179.5	77.6	0.9	0.02	#NUM!		1.1E-04
YBR156C	YBR156C	UWOPS87_ctrl_hmg124h_1.JPG.dat	1068.0	93.1	0.9	0.09	#NUM!		2.8E-04
YBR157C	YBR157C	UWOPS87_ctrl_hmg124h_1.JPG.dat	1085.0	78.8	1.0	0.00	#NUM!		7.3E-06
YBR158W	YBR158W	UWOPS87_ctrl_hmg124h_1.JPG.dat	1062.5	30.1	1.0	0.01	#NUM!		1.2E-05
YBR159W	YBR159W	UWOPS87_ctrl_hmg124h_1.JPG.dat	1179.3	100.8	1.0	0.09	#NUM!		2.2E-04
YBR161W	YBR161W	UWOPS87_ctrl_hmg124h_1.JPG.dat	1416.8	8.3	1.1	0.03	#NUM!		4.0E-06
YBR162C	YBR162C	UWOPS87_ctrl_hmg124h_1.JPG.dat	1363.8	115.0	1.1	0.03	#NUM!		3.3E-04
YBR162W-	YBR162W-A	UWOPS87_ctrl_hmg124h_1.JPG.dat	1306.0	61.9	1.0	0.06	#NUM!		4.8E-05
YBR164C	YBR164C	UWOPS87_ctrl_hmg124h_1.JPG.dat	1295.5	22.4	1.0	0.01	#NUM!		4.2E-07
YBR165W	YBR165W	UWOPS87_ctrl_hmg124h_1.JPG.dat	1161.3	35.5	0.9	0.02	#NUM!		3.5E-06
YBR166C	YBR166C	UWOPS87_ctrl_hmg124h_1.JPG.dat	1225.5	129.7	0.9	0.03	#NUM!		4.9E-04
YBR168W	YBR168W	UWOPS87_ctrl_hmg124h_1.JPG.dat	1054.5	106.7	0.8	0.09	#NUM!		3.3E-04
YBR169C	YBR169C	UWOPS87_ctrl_hmg124h_1.JPG.dat	1276.8	235.0	1.1	0.18	#NUM!		1.3E-03
YBR170C	YBR170C	UWOPS87_ctrl_hmg124h_1.JPG.dat	1069.0	30.6	1.0	0.01	#NUM!		1.8E-05
YBR171W	YBR171W	UWOPS87_ctrl_hmg124h_1.JPG.dat	997.5	9.5	0.9	0.01	#NUM!		1.1E-06
YBR172C	YBR172C	UWOPS87_ctrl_hmg124h_1.JPG.dat	1228.5	89.5	1.0	0.07	#NUM!		9.2E-05
YBR174C	YBR174C	UWOPS87_ctrl_hmg124h_1.JPG.dat	1281.0	17.1	1.0	0.03	#NUM!		5.8E-06
YBR175W	YBR175W	UWOPS87_ctrl_hmg124h_1.JPG.dat	1190.3	37.7	0.9	0.03	#NUM!		7.4E-06
YBR176W	YBR176W	UWOPS87_ctrl_hmg124h_1.JPG.dat	1341.5	61.9	1.0	0.05	#NUM!		2.7E-05
YBR177C	YBR177C	UWOPS87_ctrl_hmg124h_1.JPG.dat	1352.5	40.0	1.0	0.00	#NUM!		3.4E-06
YBR178W	YBR178W	UWOPS87_ctrl_hmg124h_1.JPG.dat	1353.8	18.2	1.0	0.02	#NUM!		1.3E-06
YBR180W	YBR180W	UWOPS87_ctrl_hmg124h_1.JPG.dat	1285.5	64.1	1.0	0.05	#NUM!		4.4E-05
YBR181C	YBR181C	UWOPS87_ctrl_hmg124h_1.JPG.dat	1016.3	44.2	0.8	0.03	#NUM!		2.0E-05
YBR182C	YBR182C	UWOPS87_ctrl_hmg124h_1.JPG.dat	1320.3	85.1	1.1	0.07	#NUM!		5.7E-05
YBR183W	YBR183W	UWOPS87_ctrl_hmg124h_1.JPG.dat	1080.3	22.4	1.0	0.01	#NUM!		5.6E-07
YBR184W	YBR184W	UWOPS87_ctrl_hmg124h_1.JPG.dat	1058.5	41.2	0.9	0.04	#NUM!		1.7E-05
YBR185C	YBR185C	UWOPS87_ctrl_hmg124h_1.JPG.dat	1301.5	92.7	1.1	0.02	#NUM!		1.1E-04
YBR186W	YBR186W	UWOPS87_ctrl_hmg124h_1.JPG.dat	1225.8	83.4	1.0	0.07	#NUM!		9.3E-05
YBR187W	YBR187W	UWOPS87_ctrl_hmg124h_1.JPG.dat	1329.3	91.8	1.0	0.08	#NUM!		1.3E-04
YBR188C	YBR188C	UWOPS87_ctrl_hmg124h_1.JPG.dat	1199.0	66.8	1.0	0.01	#NUM!		2.5E-05
YBR189W	YBR189W	UWOPS87_ctrl_hmg124h_1.JPG.dat	730.5	521.4	0.6	0.41	#NUM!		6.9E-02
YBR194W	YBR194W	UWOPS87_ctrl_hmg124h_1.JPG.dat	1194.3	13.4	0.9	0.00	#NUM!		3.9E-08
YBR195C	YBR195C	UWOPS87_ctrl_hmg124h_1.JPG.dat	1240.5	61.9	1.0	0.05	#NUM!		4.8E-05
YBR197C	YBR197C	UWOPS87_ctrl_hmg124h_1.JPG.dat	1317.8	37.5	1.1	0.03	#NUM!		9.3E-06
YBR199W	YBR199W	UWOPS87_ctrl_hmg124h_1.JPG.dat	1148.5	77.8	1.0	0.02	#NUM!		8.8E-05
YBR200W	YBR200W	UWOPS87_ctrl_hmg124h_1.JPG.dat	0.0	0.0	0.0	0.00	#NUM!		
YBR201W	YBR201W	UWOPS87_ctrl_hmg124h_1.JPG.dat	1305.8	68.1	1.1	0.06	#NUM!		4.5E-05
YBR203W	YBR203W	UWOPS87_ctrl_hmg124h_1.JPG.dat	1311.3	119.8	1.0	0.02	#NUM!		1.5E-04
YBR204C	YBR204C	UWOPS87_ctrl_hmg124h_1.JPG.dat	1346.8	103.0	1.1	0.08	#NUM!		1.0E-04
YBR205W	YBR205W	UWOPS87_ctrl_hmg124h_1.JPG.dat	1306.3	108.1	1.0	0.08	#NUM!		1.3E-04
YBR206W	YBR206W	UWOPS87_ctrl_hmg124h_1.JPG.dat	1223.5	47.7	1.0	0.04	#NUM!		2.0E-05
YBR207W	YBR207W	UWOPS87_ctrl_hmg124h_1.JPG.dat	1354.0	43.8	1.1	0.04	#NUM!		1.2E-05
YBR208C	YBR208C	UWOPS87_ctrl_hmg124h_1.JPG.dat	1281.3	103.4	1.0	0.08	#NUM!		1.6E-04
YBR209W	YBR209W	UWOPS87_ctrl_hmg124h_1.JPG.dat	1379.8	49.0	1.1	0.01	#NUM!		6.4E-05
YBR210W	YBR210W	UWOPS87_ctrl_hmg124h_1.JPG.dat	1198.8	44.8	1.0	0.03	#NUM!		7.3E-06
YBR212W	YBR212W	UWOPS87_ctrl_hmg124h_1.JPG.dat	1236.5	211.3	1.1	0.18	#NUM!		1.4E-03

YBR213W	YBR213W	UWOPS87_ctrl_hmg124h_1.JPG.dat	1075.0	114.7	0.9	0.09	#NUM!	2.7E-04
YBR214W	YBR214W	UWOPS87_ctrl_hmg124h_1.JPG.dat	1444.0	89.2	1.2	0.01	#NUM!	5.0E-05
YBR215W	YBR215W	UWOPS87_ctrl_hmg124h_1.JPG.dat	1319.8	35.1	1.1	0.03	#NUM!	4.5E-06
YBR216C	YBR216C	UWOPS87_ctrl_hmg124h_1.JPG.dat	1124.0	179.1	0.8	0.05	#NUM!	1.5E-03
YBR217W	YBR217W	UWOPS87_ctrl_hmg124h_1.JPG.dat	1430.8	27.1	1.1	0.03	#NUM!	5.4E-06
YBR218C	YBR218C	UWOPS87_ctrl_hmg124h_1.JPG.dat	1140.3	63.9	0.9	0.05	#NUM!	4.6E-05
YBR219C	YBR219C	UWOPS87_ctrl_hmg124h_1.JPG.dat	1390.0	66.7	1.1	0.05	#NUM!	3.7E-05
YBR220C	YBR220C	UWOPS87_ctrl_hmg124h_1.JPG.dat	1359.5	20.8	1.0	0.00	#NUM!	4.0E-07
YBR221C	YBR221C	UWOPS87_ctrl_hmg124h_1.JPG.dat	1461.8	49.1	1.1	0.01	#NUM!	3.6E-05
YBR222C	YBR222C	UWOPS87_ctrl_hmg124h_1.JPG.dat	1350.3	33.6	1.1	0.03	#NUM!	9.5E-06
YBR223C	YBR223C	UWOPS87_ctrl_hmg124h_1.JPG.dat	1344.0	70.8	1.1	0.06	#NUM!	4.4E-05
YOR202W	YOR202W	UWOPS87_ctrl_hmg124h_1.JPG.dat	1306.4	178.4	1.0	0.09	#NUM!	4.2E-288
1	1	UWOPS87_ctrl_hmg124h_10.JPG.dat	1643.0	295.5	1.1	0.02	#NUM!	6.9E-05
2	2	UWOPS87_ctrl_hmg124h_10.JPG.dat	1277.0	143.7	1.1	0.03	#NUM!	2.3E-04
3	3	UWOPS87_ctrl_hmg124h_10.JPG.dat	1249.8	69.0	1.0	0.03	#NUM!	2.4E-04
4	4	UWOPS87_ctrl_hmg124h_10.JPG.dat	1085.3	122.0	0.9	0.06	#NUM!	8.8E-05
YLR415C	YLR415C	UWOPS87_ctrl_hmg124h_10.JPG.dat	840.3	93.5	0.9	0.09	#NUM!	3.0E-04
YLR416C	YLR416C	UWOPS87_ctrl_hmg124h_10.JPG.dat	901.0	42.4	0.9	0.04	#NUM!	2.3E-05
YLR418C	YLR418C	UWOPS87_ctrl_hmg124h_10.JPG.dat	819.0	47.2	0.8	0.04	#NUM!	3.9E-05
YLR420W	YLR420W	UWOPS87_ctrl_hmg124h_10.JPG.dat	981.0	20.6	0.9	0.03	#NUM!	5.8E-06
YLR421C	YLR421C	UWOPS87_ctrl_hmg124h_10.JPG.dat	997.8	129.8	0.9	0.11	#NUM!	4.6E-04
YLR422W	YLR422W	UWOPS87_ctrl_hmg124h_10.JPG.dat	1342.3	42.7	1.2	0.04	#NUM!	8.4E-06
YLR423C	YLR423C	UWOPS87_ctrl_hmg124h_10.JPG.dat	1012.0	64.6	0.9	0.06	#NUM!	8.0E-05
YLR425W	YLR425W	UWOPS87_ctrl_hmg124h_10.JPG.dat	1149.3	21.6	1.1	0.02	#NUM!	1.9E-06
YLR426W	YLR426W	UWOPS87_ctrl_hmg124h_10.JPG.dat	1136.0	143.2	1.0	0.13	#NUM!	6.0E-04
YLR427W	YLR427W	UWOPS87_ctrl_hmg124h_10.JPG.dat	1258.0	61.6	1.2	0.05	#NUM!	2.0E-05
YLR428C	YLR428C	UWOPS87_ctrl_hmg124h_10.JPG.dat	972.0	58.5	0.9	0.06	#NUM!	7.0E-05
YLR429W	YLR429W	UWOPS87_ctrl_hmg124h_10.JPG.dat	902.3	66.0	1.0	0.08	#NUM!	1.7E-04
YLR431C	YLR431C	UWOPS87_ctrl_hmg124h_10.JPG.dat	965.0	38.0	1.0	0.00	#NUM!	4.4E-07
YLR432W	YLR432W	UWOPS87_ctrl_hmg124h_10.JPG.dat	1203.3	128.2	1.1	0.12	#NUM!	3.6E-04
YLR433C	YLR433C	UWOPS87_ctrl_hmg124h_10.JPG.dat	1161.5	131.2	1.1	0.13	#NUM!	5.1E-04
YLR434C	YLR434C	UWOPS87_ctrl_hmg124h_10.JPG.dat	1156.5	62.2	1.1	0.07	#NUM!	8.0E-05
YLR435W	YLR435W	UWOPS87_ctrl_hmg124h_10.JPG.dat	1131.8	22.5	1.0	0.02	#NUM!	2.0E-06
YLR436C	YLR436C	UWOPS87_ctrl_hmg124h_10.JPG.dat	1119.3	127.4	1.1	0.02	#NUM!	9.3E-05
YLR437C	YLR437C	UWOPS87_ctrl_hmg124h_10.JPG.dat	1207.8	133.0	1.1	0.03	#NUM!	1.9E-04
YLR438W	YLR438W	UWOPS87_ctrl_hmg124h_10.JPG.dat	1061.0	31.4	1.0	0.03	#NUM!	1.2E-05
YLR441C	YLR441C	UWOPS87_ctrl_hmg124h_10.JPG.dat	889.3	85.0	0.9	0.01	#NUM!	3.1E-05
YLR442C	YLR442C	UWOPS87_ctrl_hmg124h_10.JPG.dat	695.0	63.3	0.7	0.07	#NUM!	2.5E-04
YLR443W	YLR443W	UWOPS87_ctrl_hmg124h_10.JPG.dat	1065.0	39.4	1.1	0.04	#NUM!	1.8E-05
YLR444C	YLR444C	UWOPS87_ctrl_hmg124h_10.JPG.dat	1193.3	44.2	1.1	0.04	#NUM!	9.6E-06
YLR445W	YLR445W	UWOPS87_ctrl_hmg124h_10.JPG.dat	1103.5	33.7	1.0	0.03	#NUM!	8.8E-06
YLR446W	YLR446W	UWOPS87_ctrl_hmg124h_10.JPG.dat	1064.8	94.5	0.9	0.01	#NUM!	1.2E-05
YLR448W	YLR448W	UWOPS87_ctrl_hmg124h_10.JPG.dat	895.0	172.1	0.9	0.04	#NUM!	7.8E-04
YLR449W	YLR449W	UWOPS87_ctrl_hmg124h_10.JPG.dat	1051.8	15.8	0.9	0.02	#NUM!	3.0E-06
YLR450W	YLR450W	UWOPS87_ctrl_hmg124h_10.JPG.dat	0.0	0.0	0.0	0.00	#NUM!	
YLR451W	YLR451W	UWOPS87_ctrl_hmg124h_10.JPG.dat	1198.0	65.7	1.0	0.06	#NUM!	5.4E-05
YLR452C	YLR452C	UWOPS87_ctrl_hmg124h_10.JPG.dat	1192.5	54.9	1.1	0.01	#NUM!	7.5E-06
YLR453C	YLR453C	UWOPS87_ctrl_hmg124h_10.JPG.dat	1087.0	88.3	1.0	0.03	#NUM!	2.6E-04
YLR454W	YLR454W	UWOPS87_ctrl_hmg124h_10.JPG.dat	931.0	88.7	0.9	0.03	#NUM!	3.5E-04
YLR455W	YLR455W	UWOPS87_ctrl_hmg124h_10.JPG.dat	1019.3	69.2	1.0	0.01	#NUM!	3.5E-05
YLR456W	YLR456W	UWOPS87_ctrl_hmg124h_10.JPG.dat	1273.3	53.6	1.1	0.04	#NUM!	7.9E-06
YLR460C	YLR460C	UWOPS87_ctrl_hmg124h_10.JPG.dat	873.0	582.5	1.0	0.02	#NUM!	2.0E-04
YLR461W	YLR461W	UWOPS87_ctrl_hmg124h_10.JPG.dat	1237.3	70.8	1.1	0.02	#NUM!	1.2E-04
YML001W	YML001W	UWOPS87_ctrl_hmg124h_10.JPG.dat	1113.5	124.3	0.9	0.03	#NUM!	4.3E-04
YML002W	YML002W	UWOPS87_ctrl_hmg124h_10.JPG.dat	1232.3	55.4	1.0	0.05	#NUM!	3.4E-05
YML003W	YML003W	UWOPS87_ctrl_hmg124h_10.JPG.dat	1225.5	76.0	1.0	0.06	#NUM!	5.1E-05
YML004C	YML004C	UWOPS87_ctrl_hmg124h_10.JPG.dat	1090.3	48.8	0.9	0.04	#NUM!	1.6E-05
YML005W	YML005W	UWOPS87_ctrl_hmg124h_10.JPG.dat	1192.0	77.1	1.1	0.07	#NUM!	9.7E-05
YML006C	YML006C	UWOPS87_ctrl_hmg124h_10.JPG.dat	949.0	59.0	0.9	0.01	#NUM!	6.8E-05
YML007W	YML007W	UWOPS87_ctrl_hmg124h_10.JPG.dat	873.8	21.0	0.9	0.01	#NUM!	1.3E-05
YML008C	YML008C	UWOPS87_ctrl_hmg124h_10.JPG.dat	1020.3	77.6	1.0	0.07	#NUM!	1.2E-04
YML009c	YML009c	UWOPS87_ctrl_hmg124h_10.JPG.dat	984.0	52.3	0.9	0.05	#NUM!	6.1E-05
YML010C-I	YML010C-B	UWOPS87_ctrl_hmg124h_10.JPG.dat	1237.8	5.1	1.1	0.00	#NUM!	6.9E-09
YML011C	YML011C	UWOPS87_ctrl_hmg124h_10.JPG.dat	1096.5	50.1	0.9	0.05	#NUM!	3.4E-05
YML012W	YML012W	UWOPS87_ctrl_hmg124h_10.JPG.dat	1210.8	20.6	1.0	0.03	#NUM!	7.0E-06
YML013C-/	YML013C-A	UWOPS87_ctrl_hmg124h_10.JPG.dat	1152.5	83.5	1.0	0.07	#NUM!	1.1E-04
YML013W	YML013W	UWOPS87_ctrl_hmg124h_10.JPG.dat	1193.0	39.2	1.0	0.04	#NUM!	1.8E-05
YML016C	YML016C	UWOPS87_ctrl_hmg124h_10.JPG.dat	1157.5	56.5	1.0	0.05	#NUM!	3.7E-05
YML017W	YML017W	UWOPS87_ctrl_hmg124h_10.JPG.dat	1176.8	41.3	1.0	0.03	#NUM!	1.0E-05
YML018C	YML018C	UWOPS87_ctrl_hmg124h_10.JPG.dat	1127.0	54.9	1.1	0.05	#NUM!	3.3E-05

YML019W	YML019W	UWOPS87_ctrl_hmg124h_10.JPG.dat	1004.5	30.8	1.0	0.04	#NUM!	1.4E-05
YML020W	YML020W	UWOPS87_ctrl_hmg124h_10.JPG.dat	1031.3	71.9	1.0	0.07	#NUM!	9.8E-05
YML021C	YML021C	UWOPS87_ctrl_hmg124h_10.JPG.dat	996.8	60.6	0.9	0.05	#NUM!	3.5E-05
YML022W	YML022W	UWOPS87_ctrl_hmg124h_10.JPG.dat	1001.3	121.6	0.9	0.11	#NUM!	5.2E-04
YML026C	YML026C	UWOPS87_ctrl_hmg124h_10.JPG.dat	1177.5	49.4	1.0	0.04	#NUM!	1.4E-05
YML027W	YML027W	UWOPS87_ctrl_hmg124h_10.JPG.dat	1266.8	58.8	1.1	0.06	#NUM!	3.9E-05
YML028W	YML028W	UWOPS87_ctrl_hmg124h_10.JPG.dat	1108.5	44.5	1.0	0.05	#NUM!	3.5E-05
YML029W	YML029W	UWOPS87_ctrl_hmg124h_10.JPG.dat	1066.5	100.8	1.0	0.10	#NUM!	3.1E-04
YML030W	YML030W	UWOPS87_ctrl_hmg124h_10.JPG.dat	1138.8	143.1	1.0	0.12	#NUM!	3.9E-04
YML032C	YML032C	UWOPS87_ctrl_hmg124h_10.JPG.dat	647.3	75.3	0.6	0.06	#NUM!	2.9E-04
YML033W	YML033W	UWOPS87_ctrl_hmg124h_10.JPG.dat	807.3	367.0	1.0	0.01	#NUM!	6.3E-05
YML034W	YML034W	UWOPS87_ctrl_hmg124h_10.JPG.dat	1013.8	74.9	1.0	0.01	#NUM!	7.0E-05
YML035C	YML035C	UWOPS87_ctrl_hmg124h_10.JPG.dat	757.3	110.4	0.7	0.11	#NUM!	8.1E-04
YML035C-/	YML035C-A	UWOPS87_ctrl_hmg124h_10.JPG.dat	948.8	92.4	0.9	0.03	#NUM!	4.1E-04
YML036W	YML036W	UWOPS87_ctrl_hmg124h_10.JPG.dat	906.8	117.1	0.8	0.01	#NUM!	6.8E-05
YML037C	YML037C	UWOPS87_ctrl_hmg124h_10.JPG.dat	1244.8	43.0	1.1	0.04	#NUM!	1.2E-05
YML038C	YML038C	UWOPS87_ctrl_hmg124h_10.JPG.dat	1287.3	91.6	1.2	0.08	#NUM!	7.7E-05
YML041C	YML041C	UWOPS87_ctrl_hmg124h_10.JPG.dat	1206.3	90.6	1.1	0.02	#NUM!	1.5E-04
YML042W	YML042W	UWOPS87_ctrl_hmg124h_10.JPG.dat	1082.0	62.2	1.0	0.07	#NUM!	7.0E-05
YML047C	YML047C	UWOPS87_ctrl_hmg124h_10.JPG.dat	915.3	53.9	0.9	0.05	#NUM!	6.0E-05
YML048W	YML048W	UWOPS87_ctrl_hmg124h_10.JPG.dat	913.5	137.4	0.9	0.04	#NUM!	5.5E-04
YML048W-	YML048W-A	UWOPS87_ctrl_hmg124h_10.JPG.dat	835.5	558.1	1.1	0.04	#NUM!	3.7E-04
YML051W	YML051W	UWOPS87_ctrl_hmg124h_10.JPG.dat	1108.3	70.1	1.0	0.06	#NUM!	4.5E-05
YML052W	YML052W	UWOPS87_ctrl_hmg124h_10.JPG.dat	1031.3	26.6	1.1	0.01	#NUM!	6.2E-07
YML053C	YML053C	UWOPS87_ctrl_hmg124h_10.JPG.dat	1144.0	149.4	1.3	0.05	#NUM!	4.5E-04
YML054C	YML054C	UWOPS87_ctrl_hmg124h_10.JPG.dat	883.5	97.6	0.8	0.03	#NUM!	4.5E-04
YML055W	YML055W	UWOPS87_ctrl_hmg124h_10.JPG.dat	1092.3	145.6	1.1	0.14	#NUM!	6.3E-04
YML056C	YML056C	UWOPS87_ctrl_hmg124h_10.JPG.dat	1210.0	179.5	1.2	0.17	#NUM!	7.9E-04
YML057W	YML057W	UWOPS87_ctrl_hmg124h_10.JPG.dat	1041.5	175.3	1.0	0.16	#NUM!	1.4E-03
YML058C-/	YML058C-A	UWOPS87_ctrl_hmg124h_10.JPG.dat	1170.3	180.1	1.1	0.17	#NUM!	9.7E-04
YML058W	YML058W	UWOPS87_ctrl_hmg124h_10.JPG.dat	1109.3	104.2	1.0	0.09	#NUM!	2.2E-04
YML059C	YML059C	UWOPS87_ctrl_hmg124h_10.JPG.dat	1106.0	146.5	1.0	0.13	#NUM!	6.3E-04
YML060W	YML060W	UWOPS87_ctrl_hmg124h_10.JPG.dat	810.0	549.6	1.0	0.12	#NUM!	4.8E-03
YML062C	YML062C	UWOPS87_ctrl_hmg124h_10.JPG.dat	1037.8	57.0	1.0	0.05	#NUM!	3.4E-05
YML063W	YML063W	UWOPS87_ctrl_hmg124h_10.JPG.dat	975.5	135.6	1.0	0.13	#NUM!	5.5E-04
YML066C	YML066C	UWOPS87_ctrl_hmg124h_10.JPG.dat	801.0	205.7	0.8	0.20	#NUM!	3.8E-03
YML067C	YML067C	UWOPS87_ctrl_hmg124h_10.JPG.dat	932.3	316.6	1.1	0.07	#NUM!	1.7E-03
YML068W	YML068W	UWOPS87_ctrl_hmg124h_10.JPG.dat	751.3	559.5	0.7	0.52	#NUM!	7.4E-02
YML070W	YML070W	UWOPS87_ctrl_hmg124h_10.JPG.dat	981.3	300.8	1.0	0.06	#NUM!	9.3E-04
YML071C	YML071C	UWOPS87_ctrl_hmg124h_10.JPG.dat	929.3	617.4	1.1	0.03	#NUM!	2.7E-04
YML072C	YML072C	UWOPS87_ctrl_hmg124h_10.JPG.dat	513.5	477.0	0.5	0.42	#NUM!	1.2E-01
YML074C	YML074C	UWOPS87_ctrl_hmg124h_10.JPG.dat	877.8	620.5	0.8	0.56	#NUM!	6.7E-02
YML075C	YML075C	UWOPS87_ctrl_hmg124h_10.JPG.dat	396.0	474.2	0.4	0.44	#NUM!	1.9E-01
YML076C	YML076C	UWOPS87_ctrl_hmg124h_10.JPG.dat	77.5	155.0	0.0	0.00	#NUM!	
YML081C-/	YML081C-A	UWOPS87_ctrl_hmg124h_10.JPG.dat	669.8	451.0	0.7	0.44	#NUM!	5.9E-02
YML090W	YML090W	UWOPS87_ctrl_hmg124h_10.JPG.dat	1059.0	45.6	1.0	0.05	#NUM!	2.3E-05
YML094W	YML094W	UWOPS87_ctrl_hmg124h_10.JPG.dat	773.0	353.0	0.6	0.08	#NUM!	6.5E-03
YML095C	YML095C	UWOPS87_ctrl_hmg124h_10.JPG.dat	1145.0	24.9	1.0	0.03	#NUM!	4.8E-06
YML096W	YML096W	UWOPS87_ctrl_hmg124h_10.JPG.dat	1167.3	33.2	1.0	0.03	#NUM!	4.7E-06
YML097C	YML097C	UWOPS87_ctrl_hmg124h_10.JPG.dat	1276.0	49.6	1.1	0.04	#NUM!	1.4E-05
YML099C	YML099C	UWOPS87_ctrl_hmg124h_10.JPG.dat	1162.0	43.3	1.0	0.03	#NUM!	9.6E-06
YML100W	YML100W	UWOPS87_ctrl_hmg124h_10.JPG.dat	1276.0	83.1	1.1	0.07	#NUM!	9.0E-05
YML100W-	YML100W-A	UWOPS87_ctrl_hmg124h_10.JPG.dat	1151.8	55.9	1.0	0.05	#NUM!	4.6E-05
YML101C	YML101C	UWOPS87_ctrl_hmg124h_10.JPG.dat	1109.8	73.4	1.0	0.02	#NUM!	1.2E-04
YML102C-/	YML102C-A	UWOPS87_ctrl_hmg124h_10.JPG.dat	512.0	446.2	0.5	0.41	#NUM!	1.0E-01
YML102W	YML102W	UWOPS87_ctrl_hmg124h_10.JPG.dat	1055.0	8.0	1.0	0.01	#NUM!	4.7E-07
YML103C	YML103C	UWOPS87_ctrl_hmg124h_10.JPG.dat	1052.5	63.3	1.0	0.06	#NUM!	5.0E-05
YML104C	YML104C	UWOPS87_ctrl_hmg124h_10.JPG.dat	972.5	76.8	0.9	0.02	#NUM!	2.5E-04
YML106W	YML106W	UWOPS87_ctrl_hmg124h_10.JPG.dat	1206.0	11.8	1.1	0.02	#NUM!	2.6E-06
YML107C	YML107C	UWOPS87_ctrl_hmg124h_10.JPG.dat	876.0	558.1	1.0	0.09	#NUM!	2.9E-03
YML108W	YML108W	UWOPS87_ctrl_hmg124h_10.JPG.dat	1141.0	46.4	1.0	0.05	#NUM!	3.3E-05
YML109W	YML109W	UWOPS87_ctrl_hmg124h_10.JPG.dat	1256.8	77.5	1.1	0.01	#NUM!	1.6E-05
YML113W	YML113W	UWOPS87_ctrl_hmg124h_10.JPG.dat	1122.0	98.2	1.0	0.01	#NUM!	1.1E-05
YML116W	YML116W	UWOPS87_ctrl_hmg124h_10.JPG.dat	1200.5	48.8	1.0	0.05	#NUM!	2.8E-05
YML117W	YML117W	UWOPS87_ctrl_hmg124h_10.JPG.dat	1142.8	14.8	1.0	0.02	#NUM!	3.8E-06
YML117W-	YML117W-A	UWOPS87_ctrl_hmg124h_10.JPG.dat	1177.8	21.7	1.1	0.00	#NUM!	3.1E-06
YML118W	YML118W	UWOPS87_ctrl_hmg124h_10.JPG.dat	936.8	37.9	0.9	0.03	#NUM!	5.9E-06
YML119W	YML119W	UWOPS87_ctrl_hmg124h_10.JPG.dat	1110.3	21.1	1.1	0.02	#NUM!	1.7E-06
YML120C	YML120C	UWOPS87_ctrl_hmg124h_10.JPG.dat	1122.0	69.6	1.0	0.06	#NUM!	5.9E-05
YML121W	YML121W	UWOPS87_ctrl_hmg124h_10.JPG.dat	1113.0	35.2	1.0	0.04	#NUM!	1.5E-05

YML122C	YML122C	UWOPS87_ctrl_hmg124h_10.JPG.dat	1118.8	102.1	1.0	0.09	#NUM!	2.1E-04
YML123C	YML123C	UWOPS87_ctrl_hmg124h_10.JPG.dat	1110.8	81.0	1.0	0.07	#NUM!	1.1E-04
YML124C	YML124C	UWOPS87_ctrl_hmg124h_10.JPG.dat	1192.8	61.6	1.0	0.05	#NUM!	4.9E-05
YML128C	YML128C	UWOPS87_ctrl_hmg124h_10.JPG.dat	1257.0	25.1	1.0	0.02	#NUM!	1.6E-06
YML131W	YML131W	UWOPS87_ctrl_hmg124h_10.JPG.dat	1138.5	116.3	1.0	0.10	#NUM!	2.9E-04
YMR002W	YMR002W	UWOPS87_ctrl_hmg124h_10.JPG.dat	1118.5	86.6	1.0	0.08	#NUM!	1.4E-04
YMR003W	YMR003W	UWOPS87_ctrl_hmg124h_10.JPG.dat	1117.8	50.2	1.1	0.06	#NUM!	4.2E-05
YMR004W	YMR004W	UWOPS87_ctrl_hmg124h_10.JPG.dat	1039.8	38.2	1.0	0.03	#NUM!	6.1E-06
YMR006C	YMR006C	UWOPS87_ctrl_hmg124h_10.JPG.dat	1119.0	87.2	1.1	0.08	#NUM!	1.2E-04
YMR007W	YMR007W	UWOPS87_ctrl_hmg124h_10.JPG.dat	1167.3	32.9	1.1	0.04	#NUM!	1.3E-05
YMR008C	YMR008C	UWOPS87_ctrl_hmg124h_10.JPG.dat	1137.8	81.2	1.1	0.02	#NUM!	1.4E-04
YMR009W	YMR009W	UWOPS87_ctrl_hmg124h_10.JPG.dat	981.0	74.0	0.9	0.02	#NUM!	2.5E-04
YMR010W	YMR010W	UWOPS87_ctrl_hmg124h_10.JPG.dat	899.3	55.0	0.8	0.04	#NUM!	4.6E-05
YMR011W	YMR011W	UWOPS87_ctrl_hmg124h_10.JPG.dat	1258.0	80.8	1.1	0.06	#NUM!	5.4E-05
YMR012W	YMR012W	UWOPS87_ctrl_hmg124h_10.JPG.dat	1035.3	55.6	0.9	0.05	#NUM!	3.7E-05
YMR015C	YMR015C	UWOPS87_ctrl_hmg124h_10.JPG.dat	1105.3	159.9	1.0	0.14	#NUM!	8.0E-04
YMR016C	YMR016C	UWOPS87_ctrl_hmg124h_10.JPG.dat	1131.0	60.3	1.1	0.02	#NUM!	1.5E-04
YMR017W	YMR017W	UWOPS87_ctrl_hmg124h_10.JPG.dat	1057.5	47.9	1.0	0.06	#NUM!	5.7E-05
YMR018W	YMR018W	UWOPS87_ctrl_hmg124h_10.JPG.dat	989.3	66.9	1.0	0.06	#NUM!	6.2E-05
YMR019W	YMR019W	UWOPS87_ctrl_hmg124h_10.JPG.dat	1029.5	29.6	1.0	0.03	#NUM!	6.6E-06
YMR020W	YMR020W	UWOPS87_ctrl_hmg124h_10.JPG.dat	968.8	144.5	0.9	0.14	#NUM!	8.9E-04
YMR021C	YMR021C	UWOPS87_ctrl_hmg124h_10.JPG.dat	1114.5	96.9	1.0	0.09	#NUM!	1.9E-04
YMR022W	YMR022W	UWOPS87_ctrl_hmg124h_10.JPG.dat	914.0	52.2	0.9	0.05	#NUM!	5.1E-05
YMR023C	YMR023C	UWOPS87_ctrl_hmg124h_10.JPG.dat	1196.5	48.8	1.1	0.01	#NUM!	6.0E-05
YMR025W	YMR025W	UWOPS87_ctrl_hmg124h_10.JPG.dat	1085.8	90.7	0.9	0.02	#NUM!	1.1E-04
YMR026C	YMR026C	UWOPS87_ctrl_hmg124h_10.JPG.dat	1130.5	47.7	1.1	0.05	#NUM!	2.4E-05
YMR027W	YMR027W	UWOPS87_ctrl_hmg124h_10.JPG.dat	1052.8	68.4	1.0	0.07	#NUM!	8.7E-05
YMR029C	YMR029C	UWOPS87_ctrl_hmg124h_10.JPG.dat	1167.3	70.5	1.1	0.07	#NUM!	6.7E-05
YMR030W	YMR030W	UWOPS87_ctrl_hmg124h_10.JPG.dat	1066.5	35.0	1.0	0.04	#NUM!	1.4E-05
YMR031C	YMR031C	UWOPS87_ctrl_hmg124h_10.JPG.dat	990.5	18.9	0.9	0.01	#NUM!	3.4E-07
YMR031W	YMR031W-A	UWOPS87_ctrl_hmg124h_10.JPG.dat	875.0	52.8	0.9	0.04	#NUM!	2.7E-05
YMR034C	YMR034C	UWOPS87_ctrl_hmg124h_10.JPG.dat	1031.5	27.5	1.0	0.01	#NUM!	3.3E-07
YMR035W	YMR035W	UWOPS87_ctrl_hmg124h_10.JPG.dat	1027.3	56.8	1.0	0.02	#NUM!	9.4E-05
YMR036C	YMR036C	UWOPS87_ctrl_hmg124h_10.JPG.dat	1088.5	50.8	1.1	0.04	#NUM!	1.4E-05
YMR037C	YMR037C	UWOPS87_ctrl_hmg124h_10.JPG.dat	1059.0	79.6	1.0	0.02	#NUM!	9.4E-05
YMR038C	YMR038C	UWOPS87_ctrl_hmg124h_10.JPG.dat	922.8	148.7	0.9	0.15	#NUM!	1.2E-03
YMR039C	YMR039C	UWOPS87_ctrl_hmg124h_10.JPG.dat	1018.3	54.5	1.0	0.02	#NUM!	1.0E-04
YMR040W	YMR040W	UWOPS87_ctrl_hmg124h_10.JPG.dat	1140.3	58.2	1.1	0.02	#NUM!	1.1E-04
YMR041C	YMR041C	UWOPS87_ctrl_hmg124h_10.JPG.dat	1223.3	62.0	1.1	0.07	#NUM!	7.5E-05
YMR042W	YMR042W	UWOPS87_ctrl_hmg124h_10.JPG.dat	1132.0	78.3	1.0	0.06	#NUM!	5.5E-05
YMR044W	YMR044W	UWOPS87_ctrl_hmg124h_10.JPG.dat	1267.0	102.0	1.1	0.01	#NUM!	5.9E-05
YMR048W	YMR048W	UWOPS87_ctrl_hmg124h_10.JPG.dat	980.8	62.0	1.0	0.06	#NUM!	5.4E-05
YMR052C-.	YMR052C-A	UWOPS87_ctrl_hmg124h_10.JPG.dat	1139.8	44.7	1.1	0.05	#NUM!	2.9E-05
YMR052W	YMR052W	UWOPS87_ctrl_hmg124h_10.JPG.dat	1177.8	86.4	1.1	0.02	#NUM!	8.1E-05
YMR053C	YMR053C	UWOPS87_ctrl_hmg124h_10.JPG.dat	1186.5	58.2	1.1	0.06	#NUM!	4.1E-05
YMR054W	YMR054W	UWOPS87_ctrl_hmg124h_10.JPG.dat	1198.0	36.4	1.1	0.03	#NUM!	6.0E-06
YMR055C	YMR055C	UWOPS87_ctrl_hmg124h_10.JPG.dat	1194.8	35.2	1.0	0.02	#NUM!	2.9E-06
YMR056C	YMR056C	UWOPS87_ctrl_hmg124h_10.JPG.dat	1236.8	57.8	1.1	0.05	#NUM!	3.3E-05
YMR057C	YMR057C	UWOPS87_ctrl_hmg124h_10.JPG.dat	1182.5	62.2	1.0	0.06	#NUM!	5.4E-05
YMR058W	YMR058W	UWOPS87_ctrl_hmg124h_10.JPG.dat	1153.0	30.9	1.0	0.02	#NUM!	4.1E-06
YMR060C	YMR060C	UWOPS87_ctrl_hmg124h_10.JPG.dat	1106.0	81.4	1.0	0.08	#NUM!	1.3E-04
YMR063W	YMR063W	UWOPS87_ctrl_hmg124h_10.JPG.dat	1144.0	40.9	1.1	0.03	#NUM!	5.9E-06
YMR065W	YMR065W	UWOPS87_ctrl_hmg124h_10.JPG.dat	1066.3	68.3	1.1	0.07	#NUM!	9.2E-05
YMR067C	YMR067C	UWOPS87_ctrl_hmg124h_10.JPG.dat	928.5	34.5	0.9	0.04	#NUM!	2.2E-05
YMR068W	YMR068W	UWOPS87_ctrl_hmg124h_10.JPG.dat	1072.3	119.2	1.0	0.11	#NUM!	4.2E-04
YMR070W	YMR070W	UWOPS87_ctrl_hmg124h_10.JPG.dat	1160.5	266.6	1.0	0.24	#NUM!	3.6E-03
YMR073C	YMR073C	UWOPS87_ctrl_hmg124h_10.JPG.dat	1162.0	20.1	1.0	0.01	#NUM!	8.8E-07
YMR075C-.	YMR075C-A	UWOPS87_ctrl_hmg124h_10.JPG.dat	1077.8	4.3	0.9	0.01	#NUM!	6.5E-07
YMR075W	YMR075W	UWOPS87_ctrl_hmg124h_10.JPG.dat	1211.8	71.1	1.0	0.05	#NUM!	3.1E-05
YMR078C	YMR078C	UWOPS87_ctrl_hmg124h_10.JPG.dat	834.8	409.3	0.7	0.35	#NUM!	2.5E-02
YMR080C	YMR080C	UWOPS87_ctrl_hmg124h_10.JPG.dat	1210.5	47.8	1.1	0.01	#NUM!	2.4E-05
YMR085W	YMR085W	UWOPS87_ctrl_hmg124h_10.JPG.dat	1123.5	55.2	1.0	0.06	#NUM!	4.1E-05
YMR086C-.	YMR086C-A	UWOPS87_ctrl_hmg124h_10.JPG.dat	1050.0	132.0	1.0	0.13	#NUM!	5.5E-04
YMR086W	YMR086W	UWOPS87_ctrl_hmg124h_10.JPG.dat	897.8	52.6	0.9	0.05	#NUM!	5.2E-05
YMR087W	YMR087W	UWOPS87_ctrl_hmg124h_10.JPG.dat	1188.3	146.6	1.1	0.14	#NUM!	5.9E-04
YMR088C	YMR088C	UWOPS87_ctrl_hmg124h_10.JPG.dat	1157.0	10.3	1.0	0.01	#NUM!	1.9E-07
YMR092C	YMR092C	UWOPS87_ctrl_hmg124h_10.JPG.dat	1140.0	52.0	1.0	0.04	#NUM!	2.3E-05
YMR095C	YMR095C	UWOPS87_ctrl_hmg124h_10.JPG.dat	1186.8	50.4	1.0	0.04	#NUM!	2.3E-05
YMR096W	YMR096W	UWOPS87_ctrl_hmg124h_10.JPG.dat	1162.0	24.1	1.0	0.00	#NUM!	1.6E-06
YMR099C	YMR099C	UWOPS87_ctrl_hmg124h_10.JPG.dat	1172.0	32.1	1.0	0.01	#NUM!	1.8E-05

YMR100W	YMR100W	UWOPS87_ctrl_hmg124h_10.JPG.dat	1117.3	69.6	1.0	0.05	#NUM!		5.2E-05
YMR101C	YMR101C	UWOPS87_ctrl_hmg124h_10.JPG.dat	1159.3	41.9	1.0	0.05	#NUM!		3.2E-05
YMR102C	YMR102C	UWOPS87_ctrl_hmg124h_10.JPG.dat	1190.8	47.0	1.1	0.05	#NUM!		2.3E-05
YMR103C	YMR103C	UWOPS87_ctrl_hmg124h_10.JPG.dat	1210.3	28.5	1.2	0.04	#NUM!		8.2E-06
YMR104C	YMR104C	UWOPS87_ctrl_hmg124h_10.JPG.dat	1202.5	57.3	1.2	0.06	#NUM!		2.6E-05
YMR105C	YMR105C	UWOPS87_ctrl_hmg124h_10.JPG.dat	1114.3	53.5	1.0	0.04	#NUM!		1.9E-05
YMR106C	YMR106C	UWOPS87_ctrl_hmg124h_10.JPG.dat	1183.0	44.3	1.1	0.04	#NUM!		1.7E-05
YMR107W	YMR107W	UWOPS87_ctrl_hmg124h_10.JPG.dat	1157.8	49.6	1.0	0.04	#NUM!		2.2E-05
YMR109W	YMR109W	UWOPS87_ctrl_hmg124h_10.JPG.dat	1056.0	75.3	0.9	0.08	#NUM!		1.7E-04
YMR110C	YMR110C	UWOPS87_ctrl_hmg124h_10.JPG.dat	1237.5	68.9	1.1	0.06	#NUM!		4.0E-05
YMR111C	YMR111C	UWOPS87_ctrl_hmg124h_10.JPG.dat	1161.3	24.4	1.0	0.01	#NUM!		6.0E-07
YMR114C	YMR114C	UWOPS87_ctrl_hmg124h_10.JPG.dat	1057.8	78.2	0.9	0.07	#NUM!		9.1E-05
YMR115W	YMR115W	UWOPS87_ctrl_hmg124h_10.JPG.dat	1113.3	23.0	1.0	0.03	#NUM!		8.7E-06
YMR116C	YMR116C	UWOPS87_ctrl_hmg124h_10.JPG.dat	857.8	49.1	0.8	0.02	#NUM!		1.3E-04
YMR119W	YMR119W	UWOPS87_ctrl_hmg124h_10.JPG.dat	958.3	12.8	1.0	0.02	#NUM!		1.8E-06
YMR119W	YMR119W-A	UWOPS87_ctrl_hmg124h_10.JPG.dat	952.5	74.0	0.9	0.07	#NUM!		1.5E-04
YMR120C	YMR120C	UWOPS87_ctrl_hmg124h_10.JPG.dat	1050.3	44.8	1.0	0.04	#NUM!		1.4E-05
YMR121C	YMR121C	UWOPS87_ctrl_hmg124h_10.JPG.dat	1118.5	37.5	1.0	0.03	#NUM!		1.0E-05
YMR122C	YMR122C	UWOPS87_ctrl_hmg124h_10.JPG.dat	1118.0	104.1	1.0	0.08	#NUM!		1.8E-04
YMR123W	YMR123W	UWOPS87_ctrl_hmg124h_10.JPG.dat	251.8	503.5	0.0	0.00	#NUM!		
YMR124W	YMR124W	UWOPS87_ctrl_hmg124h_10.JPG.dat	1258.0	101.0	1.0	0.08	#NUM!		1.3E-04
YMR126C	YMR126C	UWOPS87_ctrl_hmg124h_10.JPG.dat	1203.8	98.9	1.0	0.08	#NUM!		1.3E-04
YMR127C	YMR127C	UWOPS87_ctrl_hmg124h_10.JPG.dat	1241.0	31.5	1.1	0.01	#NUM!		2.2E-05
YMR129W	YMR129W	UWOPS87_ctrl_hmg124h_10.JPG.dat	1151.0	86.0	1.0	0.06	#NUM!		6.9E-05
YMR130W	YMR130W	UWOPS87_ctrl_hmg124h_10.JPG.dat	1178.8	51.9	1.1	0.05	#NUM!		2.0E-05
YMR132C	YMR132C	UWOPS87_ctrl_hmg124h_10.JPG.dat	1004.3	38.7	1.0	0.05	#NUM!		3.3E-05
YMR133W	YMR133W	UWOPS87_ctrl_hmg124h_10.JPG.dat	1142.3	76.2	1.1	0.06	#NUM!		5.5E-05
YMR135C	YMR135C	UWOPS87_ctrl_hmg124h_10.JPG.dat	1235.8	54.2	1.1	0.05	#NUM!		1.9E-05
YMR135W	YMR135W-A	UWOPS87_ctrl_hmg124h_10.JPG.dat	1243.8	59.7	1.1	0.05	#NUM!		3.2E-05
YMR136W	YMR136W	UWOPS87_ctrl_hmg124h_10.JPG.dat	1263.5	52.4	1.1	0.04	#NUM!		1.2E-05
YMR137C	YMR137C	UWOPS87_ctrl_hmg124h_10.JPG.dat	1021.8	57.1	0.9	0.06	#NUM!		7.5E-05
YMR138W	YMR138W	UWOPS87_ctrl_hmg124h_10.JPG.dat	1142.3	47.3	1.0	0.04	#NUM!		1.9E-05
YMR139W	YMR139W	UWOPS87_ctrl_hmg124h_10.JPG.dat	1025.8	39.5	0.9	0.01	#NUM!		4.0E-05
YMR140W	YMR140W	UWOPS87_ctrl_hmg124h_10.JPG.dat	1163.8	51.9	1.0	0.01	#NUM!		1.5E-05
YMR141C	YMR141C	UWOPS87_ctrl_hmg124h_10.JPG.dat	1086.8	74.8	1.0	0.07	#NUM!		9.5E-05
YMR143W	YMR143W	UWOPS87_ctrl_hmg124h_10.JPG.dat	1130.5	91.3	1.1	0.08	#NUM!		1.0E-04
YMR144W	YMR144W	UWOPS87_ctrl_hmg124h_10.JPG.dat	996.8	41.5	0.9	0.05	#NUM!		4.7E-05
YMR145C	YMR145C	UWOPS87_ctrl_hmg124h_10.JPG.dat	778.3	48.2	0.8	0.04	#NUM!		3.9E-05
YMR147W	YMR147W	UWOPS87_ctrl_hmg124h_10.JPG.dat	1006.0	124.6	1.0	0.12	#NUM!		4.3E-04
YMR148W	YMR148W	UWOPS87_ctrl_hmg124h_10.JPG.dat	1072.5	71.1	1.1	0.09	#NUM!		1.7E-04
YMR152W	YMR152W	UWOPS87_ctrl_hmg124h_10.JPG.dat	918.8	70.4	0.9	0.02	#NUM!		2.2E-04
YMR153C-A	YMR153C-A	UWOPS87_ctrl_hmg124h_10.JPG.dat	941.0	33.0	0.9	0.03	#NUM!		9.9E-06
YMR153W	YMR153W	UWOPS87_ctrl_hmg124h_10.JPG.dat	863.5	75.5	0.8	0.07	#NUM!		1.7E-04
YMR154C	YMR154C	UWOPS87_ctrl_hmg124h_10.JPG.dat	942.8	43.4	0.9	0.04	#NUM!		3.0E-05
YMR155W	YMR155W	UWOPS87_ctrl_hmg124h_10.JPG.dat	1101.3	140.5	1.1	0.02	#NUM!		1.6E-04
YMR156C	YMR156C	UWOPS87_ctrl_hmg124h_10.JPG.dat	1137.8	218.4	1.1	0.20	#NUM!		1.7E-03
YMR157C	YMR157C	UWOPS87_ctrl_hmg124h_10.JPG.dat	1133.3	58.6	1.1	0.07	#NUM!		8.0E-05
YMR158C-	YMR158C-B	UWOPS87_ctrl_hmg124h_10.JPG.dat	1252.0	135.4	1.2	0.12	#NUM!		3.0E-04
YMR158W	YMR158W-A	UWOPS87_ctrl_hmg124h_10.JPG.dat	919.3	104.7	0.9	0.11	#NUM!		3.8E-04
YMR159C	YMR159C	UWOPS87_ctrl_hmg124h_10.JPG.dat	870.8	72.8	0.9	0.08	#NUM!		1.8E-04
YMR160W	YMR160W	UWOPS87_ctrl_hmg124h_10.JPG.dat	1022.3	206.3	1.0	0.20	#NUM!		2.4E-03
YMR161W	YMR161W	UWOPS87_ctrl_hmg124h_10.JPG.dat	940.3	155.3	0.9	0.15	#NUM!		1.3E-03
YMR162C	YMR162C	UWOPS87_ctrl_hmg124h_10.JPG.dat	828.3	147.7	0.7	0.04	#NUM!		8.9E-04
YMR163C	YMR163C	UWOPS87_ctrl_hmg124h_10.JPG.dat	958.8	75.6	0.8	0.07	#NUM!		1.9E-04
YMR164C	YMR164C	UWOPS87_ctrl_hmg124h_10.JPG.dat	1230.5	18.5	1.1	0.01	#NUM!		3.2E-07
YMR166C	YMR166C	UWOPS87_ctrl_hmg124h_10.JPG.dat	856.3	71.3	0.7	0.06	#NUM!		1.4E-04
YMR167W	YMR167W	UWOPS87_ctrl_hmg124h_10.JPG.dat	1143.5	153.9	1.0	0.13	#NUM!		6.4E-04
YMR169c	YMR169c	UWOPS87_ctrl_hmg124h_10.JPG.dat	909.3	149.5	0.8	0.13	#NUM!		1.1E-03
YMR170C	YMR170C	UWOPS87_ctrl_hmg124h_10.JPG.dat	1048.3	298.4	1.0	0.28	#NUM!		6.1E-03
YMR171C	YMR171C	UWOPS87_ctrl_hmg124h_10.JPG.dat	965.8	124.6	1.0	0.12	#NUM!		4.7E-04
YMR172C-A	YMR172C-A	UWOPS87_ctrl_hmg124h_10.JPG.dat	1092.8	31.0	1.0	0.01	#NUM!		1.9E-05
YMR172W	YMR172W	UWOPS87_ctrl_hmg124h_10.JPG.dat	1060.8	77.8	1.0	0.00	#NUM!		7.2E-06
YMR173W	YMR173W	UWOPS87_ctrl_hmg124h_10.JPG.dat	1126.5	89.3	1.0	0.07	#NUM!		1.1E-04
YMR173W	YMR173W-A	UWOPS87_ctrl_hmg124h_10.JPG.dat	1085.3	211.3	1.0	0.05	#NUM!		6.8E-04
YMR174C	YMR174C	UWOPS87_ctrl_hmg124h_10.JPG.dat	1099.5	179.3	1.0	0.03	#NUM!		2.3E-04
YMR175w	YMR175w	UWOPS87_ctrl_hmg124h_10.JPG.dat	1208.8	99.8	1.0	0.08	#NUM!		1.3E-04
YMR176W	YMR176W	UWOPS87_ctrl_hmg124h_10.JPG.dat	1156.5	40.7	1.0	0.04	#NUM!		1.6E-05
YMR177W	YMR177W	UWOPS87_ctrl_hmg124h_10.JPG.dat	1004.8	191.2	0.9	0.17	#NUM!		1.9E-03
YMR178W	YMR178W	UWOPS87_ctrl_hmg124h_10.JPG.dat	1002.8	134.2	0.9	0.12	#NUM!		5.3E-04
YMR179W	YMR179W	UWOPS87_ctrl_hmg124h_10.JPG.dat	1130.3	146.1	1.2	0.03	#NUM!		2.5E-04

YMR180C	YMR180C	UWOPS87_ctrl_hmg124h_10.JPG.dat	1068.0	205.5	0.9	0.06	#NUM!	1.3E-03
YMR181C	YMR181C	UWOPS87_ctrl_hmg124h_10.JPG.dat	1205.5	58.7	1.1	0.06	#NUM!	4.3E-05
YMR182C	YMR182C	UWOPS87_ctrl_hmg124h_10.JPG.dat	1229.8	21.7	1.1	0.03	#NUM!	4.2E-06
YMR183C	YMR183C	UWOPS87_ctrl_hmg124h_10.JPG.dat	1173.5	47.1	1.0	0.04	#NUM!	1.6E-05
YMR186W	YMR186W	UWOPS87_ctrl_hmg124h_10.JPG.dat	1153.5	107.9	1.0	0.09	#NUM!	2.3E-04
YMR187C	YMR187C	UWOPS87_ctrl_hmg124h_10.JPG.dat	1170.5	68.8	1.0	0.06	#NUM!	5.1E-05
YMR188C	YMR188C	UWOPS87_ctrl_hmg124h_10.JPG.dat	1247.8	73.5	1.0	0.06	#NUM!	5.3E-05
YMR189W	YMR189W	UWOPS87_ctrl_hmg124h_10.JPG.dat	1135.5	94.9	1.0	0.02	#NUM!	2.0E-04
YMR190C	YMR190C	UWOPS87_ctrl_hmg124h_10.JPG.dat	1195.0	31.6	1.0	0.01	#NUM!	2.4E-05
YMR191W	YMR191W	UWOPS87_ctrl_hmg124h_10.JPG.dat	1052.5	58.9	1.0	0.06	#NUM!	5.3E-05
YMR192W	YMR192W	UWOPS87_ctrl_hmg124h_10.JPG.dat	994.0	117.0	1.0	0.11	#NUM!	4.0E-04
YMR193C-.	YMR193C-A	UWOPS87_ctrl_hmg124h_10.JPG.dat	813.5	39.2	0.8	0.01	#NUM!	6.9E-05
YMR194W	YMR194W	UWOPS87_ctrl_hmg124h_10.JPG.dat	960.5	35.0	0.9	0.03	#NUM!	1.3E-05
YMR195W	YMR195W	UWOPS87_ctrl_hmg124h_10.JPG.dat	1171.0	64.7	1.1	0.06	#NUM!	6.0E-05
YMR196W	YMR196W	UWOPS87_ctrl_hmg124h_10.JPG.dat	1204.5	105.9	1.1	0.09	#NUM!	1.6E-04
YMR198W	YMR198W	UWOPS87_ctrl_hmg124h_10.JPG.dat	1147.5	61.6	1.0	0.06	#NUM!	4.4E-05
YMR199W	YMR199W	UWOPS87_ctrl_hmg124h_10.JPG.dat	1239.0	75.7	1.1	0.07	#NUM!	7.0E-05
YMR201C	YMR201C	UWOPS87_ctrl_hmg124h_10.JPG.dat	1194.5	75.3	1.0	0.06	#NUM!	6.7E-05
YMR202W	YMR202W	UWOPS87_ctrl_hmg124h_10.JPG.dat	1188.8	14.4	1.1	0.02	#NUM!	8.4E-07
YMR204C	YMR204C	UWOPS87_ctrl_hmg124h_10.JPG.dat	1089.3	109.3	1.0	0.03	#NUM!	3.1E-04
YMR205C	YMR205C	UWOPS87_ctrl_hmg124h_10.JPG.dat	869.0	59.5	0.8	0.05	#NUM!	7.5E-05
YMR206W	YMR206W	UWOPS87_ctrl_hmg124h_10.JPG.dat	859.5	126.7	0.9	0.13	#NUM!	8.2E-04
YMR207C	YMR207C	UWOPS87_ctrl_hmg124h_10.JPG.dat	1128.5	59.3	1.1	0.05	#NUM!	3.4E-05
YMR209C	YMR209C	UWOPS87_ctrl_hmg124h_10.JPG.dat	1083.0	51.3	1.0	0.05	#NUM!	3.5E-05
YMR210W	YMR210W	UWOPS87_ctrl_hmg124h_10.JPG.dat	1078.0	93.8	1.0	0.09	#NUM!	2.0E-04
YMR214W	YMR214W	UWOPS87_ctrl_hmg124h_10.JPG.dat	1115.3	56.6	1.0	0.05	#NUM!	3.6E-05
YMR215W	YMR215W	UWOPS87_ctrl_hmg124h_10.JPG.dat	1199.3	50.2	1.0	0.04	#NUM!	1.9E-05
YMR216C	YMR216C	UWOPS87_ctrl_hmg124h_10.JPG.dat	1352.0	47.8	1.1	0.04	#NUM!	1.3E-05
YMR219W	YMR219W	UWOPS87_ctrl_hmg124h_10.JPG.dat	1217.8	37.7	1.0	0.03	#NUM!	7.8E-06
YMR221C	YMR221C	UWOPS87_ctrl_hmg124h_10.JPG.dat	1173.0	147.3	0.9	0.04	#NUM!	6.7E-04
YMR222C	YMR222C	UWOPS87_ctrl_hmg124h_10.JPG.dat	1169.8	43.0	1.0	0.04	#NUM!	1.2E-05
YMR223W	YMR223W	UWOPS87_ctrl_hmg124h_10.JPG.dat	1052.3	41.0	1.0	0.04	#NUM!	2.5E-05
YMR224C	YMR224C	UWOPS87_ctrl_hmg124h_10.JPG.dat	996.8	28.6	1.0	0.02	#NUM!	3.7E-06
YMR225C	YMR225C	UWOPS87_ctrl_hmg124h_10.JPG.dat	990.5	38.5	0.9	0.04	#NUM!	1.5E-05
YMR226C	YMR226C	UWOPS87_ctrl_hmg124h_10.JPG.dat	1211.8	31.9	1.1	0.03	#NUM!	7.7E-06
YMR230W	YMR230W	UWOPS87_ctrl_hmg124h_10.JPG.dat	869.8	49.8	0.8	0.05	#NUM!	6.8E-05
YMR232W	YMR232W	UWOPS87_ctrl_hmg124h_10.JPG.dat	1126.0	44.4	1.0	0.04	#NUM!	1.5E-05
YMR233W	YMR233W	UWOPS87_ctrl_hmg124h_10.JPG.dat	939.5	120.4	0.8	0.10	#NUM!	5.5E-04
YMR234W	YMR234W	UWOPS87_ctrl_hmg124h_10.JPG.dat	1402.0	76.9	1.2	0.07	#NUM!	5.6E-05
YMR237W	YMR237W	UWOPS87_ctrl_hmg124h_10.JPG.dat	1164.0	109.3	1.0	0.10	#NUM!	2.9E-04
YMR238W	YMR238W	UWOPS87_ctrl_hmg124h_10.JPG.dat	1239.8	14.0	1.1	0.01	#NUM!	3.0E-07
YMR241W	YMR241W	UWOPS87_ctrl_hmg124h_10.JPG.dat	1063.3	70.6	1.0	0.07	#NUM!	8.5E-05
YMR243C	YMR243C	UWOPS87_ctrl_hmg124h_10.JPG.dat	543.8	389.2	0.5	0.37	#NUM!	6.8E-02
YMR244C-.	YMR244C-A	UWOPS87_ctrl_hmg124h_10.JPG.dat	969.3	48.2	0.9	0.01	#NUM!	1.9E-05
YOR202W	YOR202W	UWOPS87_ctrl_hmg124h_10.JPG.dat	1156.9	288.4	1.0	0.21	#NUM!	2.2E-189
1	1	UWOPS87_ctrl_hmg124h_11.JPG.dat	1368.0	227.6	0.9	0.11	#NUM!	4.1E-04
2	2	UWOPS87_ctrl_hmg124h_11.JPG.dat	1138.5	163.8	0.9	0.08	#NUM!	1.9E-04
3	3	UWOPS87_ctrl_hmg124h_11.JPG.dat	1334.3	148.1	1.1	0.06	#NUM!	4.5E-05
4	4	UWOPS87_ctrl_hmg124h_11.JPG.dat	1115.8	219.1	0.9	0.14	#NUM!	8.0E-04
YJR109C	YJR109C	UWOPS87_ctrl_hmg124h_11.JPG.dat	1260.8	62.3	1.0	0.06	#NUM!	6.6E-05
YMR244W	YMR244W	UWOPS87_ctrl_hmg124h_11.JPG.dat	944.0	66.2	1.0	0.02	#NUM!	9.8E-05
YMR245W	YMR245W	UWOPS87_ctrl_hmg124h_11.JPG.dat	1068.0	55.5	1.1	0.04	#NUM!	1.1E-05
YMR246W	YMR246W	UWOPS87_ctrl_hmg124h_11.JPG.dat	966.0	66.9	1.0	0.09	#NUM!	2.0E-04
YMR247C	YMR247C	UWOPS87_ctrl_hmg124h_11.JPG.dat	1022.8	117.7	1.1	0.04	#NUM!	4.9E-04
YMR250W	YMR250W	UWOPS87_ctrl_hmg124h_11.JPG.dat	920.3	73.5	0.9	0.07	#NUM!	1.2E-04
YMR251W	YMR251W	UWOPS87_ctrl_hmg124h_11.JPG.dat	1228.3	190.9	1.0	0.04	#NUM!	5.0E-04
YMR251W	YMR251W-A	UWOPS87_ctrl_hmg124h_11.JPG.dat	1037.3	50.1	0.9	0.04	#NUM!	2.4E-05
YMR252C	YMR252C	UWOPS87_ctrl_hmg124h_11.JPG.dat	1131.5	47.1	1.0	0.04	#NUM!	2.0E-05
YMR253C	YMR253C	UWOPS87_ctrl_hmg124h_11.JPG.dat	1151.3	77.5	1.0	0.07	#NUM!	8.1E-05
YMR254C	YMR254C	UWOPS87_ctrl_hmg124h_11.JPG.dat	1140.0	57.8	1.0	0.05	#NUM!	2.8E-05
YMR255W	YMR255W	UWOPS87_ctrl_hmg124h_11.JPG.dat	954.8	43.3	0.9	0.01	#NUM!	2.7E-05
YMR256C	YMR256C	UWOPS87_ctrl_hmg124h_11.JPG.dat	316.0	249.7	0.3	0.27	#NUM!	8.5E-02
YMR258C	YMR258C	UWOPS87_ctrl_hmg124h_11.JPG.dat	1044.5	164.2	1.1	0.17	#NUM!	1.1E-03
YMR259C	YMR259C	UWOPS87_ctrl_hmg124h_11.JPG.dat	941.3	99.7	0.9	0.02	#NUM!	2.4E-04
YMR261C	YMR261C	UWOPS87_ctrl_hmg124h_11.JPG.dat	1115.8	100.7	1.0	0.02	#NUM!	1.5E-04
YMR262W	YMR262W	UWOPS87_ctrl_hmg124h_11.JPG.dat	1107.5	50.1	1.0	0.06	#NUM!	6.6E-05
YMR263W	YMR263W	UWOPS87_ctrl_hmg124h_11.JPG.dat	1279.5	18.0	1.1	0.01	#NUM!	6.7E-06
YMR264W	YMR264W	UWOPS87_ctrl_hmg124h_11.JPG.dat	1158.3	111.2	1.0	0.11	#NUM!	3.4E-04
YMR265C	YMR265C	UWOPS87_ctrl_hmg124h_11.JPG.dat	1095.3	79.3	1.0	0.06	#NUM!	7.2E-05
YMR266W	YMR266W	UWOPS87_ctrl_hmg124h_11.JPG.dat	1105.5	132.7	0.9	0.11	#NUM!	4.2E-04

YMR269W	YMR269W	UWOPS87_ctrl_hmg124h_11.JPG.dat	1017.5	21.8	0.9	0.03	#NUM!	7.4E-06
YMR271C	YMR271C	UWOPS87_ctrl_hmg124h_11.JPG.dat	989.0	77.2	1.0	0.07	#NUM!	8.7E-05
YMR272C	YMR272C	UWOPS87_ctrl_hmg124h_11.JPG.dat	934.5	22.4	1.0	0.05	#NUM!	4.7E-05
YMR273C	YMR273C	UWOPS87_ctrl_hmg124h_11.JPG.dat	965.0	107.3	1.0	0.13	#NUM!	6.3E-04
YMR274C	YMR274C	UWOPS87_ctrl_hmg124h_11.JPG.dat	1062.8	121.3	1.0	0.09	#NUM!	2.1E-04
YMR275C	YMR275C	UWOPS87_ctrl_hmg124h_11.JPG.dat	1039.3	154.5	0.9	0.11	#NUM!	4.2E-04
YMR276W	YMR276W	UWOPS87_ctrl_hmg124h_11.JPG.dat	1089.3	25.3	0.9	0.05	#NUM!	4.2E-05
YMR278W	YMR278W	UWOPS87_ctrl_hmg124h_11.JPG.dat	1229.0	141.3	1.0	0.10	#NUM!	2.4E-04
YMR279C	YMR279C	UWOPS87_ctrl_hmg124h_11.JPG.dat	1130.5	115.4	0.9	0.06	#NUM!	9.1E-05
YMR280C	YMR280C	UWOPS87_ctrl_hmg124h_11.JPG.dat	993.0	563.9	1.1	0.02	#NUM!	1.3E-04
YMR282C	YMR282C	UWOPS87_ctrl_hmg124h_11.JPG.dat	608.3	709.5	0.5	0.62	#NUM!	1.8E-01
YMR283C	YMR283C	UWOPS87_ctrl_hmg124h_11.JPG.dat	971.8	50.4	0.9	0.02	#NUM!	1.3E-04
YMR284W	YMR284W	UWOPS87_ctrl_hmg124h_11.JPG.dat	1078.5	28.7	1.1	0.06	#NUM!	5.1E-05
YMR285C	YMR285C	UWOPS87_ctrl_hmg124h_11.JPG.dat	909.3	64.4	0.9	0.05	#NUM!	4.3E-05
YMR289W	YMR289W	UWOPS87_ctrl_hmg124h_11.JPG.dat	1139.8	87.8	1.1	0.09	#NUM!	1.4E-04
YMR291W	YMR291W	UWOPS87_ctrl_hmg124h_11.JPG.dat	990.0	84.7	0.9	0.06	#NUM!	8.6E-05
YMR292W	YMR292W	UWOPS87_ctrl_hmg124h_11.JPG.dat	1066.5	190.9	0.9	0.17	#NUM!	1.6E-03
YMR294W	YMR294W	UWOPS87_ctrl_hmg124h_11.JPG.dat	1179.0	73.2	0.9	0.01	#NUM!	1.1E-05
YMR294W	YMR294W-A	UWOPS87_ctrl_hmg124h_11.JPG.dat	1297.0	34.7	1.1	0.02	#NUM!	1.0E-06
YMR295C	YMR295C	UWOPS87_ctrl_hmg124h_11.JPG.dat	1255.5	106.1	1.0	0.08	#NUM!	1.5E-04
YMR297W	YMR297W	UWOPS87_ctrl_hmg124h_11.JPG.dat	1177.5	69.5	1.0	0.05	#NUM!	4.1E-05
YMR299C	YMR299C	UWOPS87_ctrl_hmg124h_11.JPG.dat	456.0	332.0	0.4	0.29	#NUM!	7.1E-02
YMR300C	YMR300C	UWOPS87_ctrl_hmg124h_11.JPG.dat	935.8	54.3	0.9	0.02	#NUM!	1.4E-04
YMR302C	YMR302C	UWOPS87_ctrl_hmg124h_11.JPG.dat	938.5	34.1	0.9	0.01	#NUM!	4.3E-05
YMR303C	YMR303C	UWOPS87_ctrl_hmg124h_11.JPG.dat	1008.0	115.2	1.0	0.10	#NUM!	3.0E-04
YMR304C-.	YMR304C-A	UWOPS87_ctrl_hmg124h_11.JPG.dat	1111.5	51.4	1.0	0.04	#NUM!	1.7E-05
YMR304W	YMR304W	UWOPS87_ctrl_hmg124h_11.JPG.dat	1272.0	31.8	1.1	0.01	#NUM!	4.6E-07
YMR305C	YMR305C	UWOPS87_ctrl_hmg124h_11.JPG.dat	1104.5	115.2	1.0	0.10	#NUM!	3.4E-04
YMR306C-.	YMR306C-A	UWOPS87_ctrl_hmg124h_11.JPG.dat	1344.5	65.7	1.1	0.01	#NUM!	3.5E-05
YMR306W	YMR306W	UWOPS87_ctrl_hmg124h_11.JPG.dat	1185.5	21.9	1.0	0.00	#NUM!	5.2E-06
YMR307W	YMR307W	UWOPS87_ctrl_hmg124h_11.JPG.dat	1210.5	30.5	1.0	0.03	#NUM!	4.6E-06
YMR310C	YMR310C	UWOPS87_ctrl_hmg124h_11.JPG.dat	1116.0	14.4	1.0	0.00	#NUM!	7.9E-06
YMR311C	YMR311C	UWOPS87_ctrl_hmg124h_11.JPG.dat	1175.3	53.9	1.0	0.06	#NUM!	4.7E-05
YMR312W	YMR312W	UWOPS87_ctrl_hmg124h_11.JPG.dat	1049.0	143.0	1.0	0.13	#NUM!	6.0E-04
YMR313C	YMR313C	UWOPS87_ctrl_hmg124h_11.JPG.dat	1054.0	32.3	1.0	0.03	#NUM!	7.3E-06
YMR315W	YMR315W	UWOPS87_ctrl_hmg124h_11.JPG.dat	1061.0	35.7	1.0	0.04	#NUM!	1.6E-05
YMR316C-.	YMR316C-A	UWOPS87_ctrl_hmg124h_11.JPG.dat	1242.8	153.4	1.1	0.14	#NUM!	4.6E-04
YMR316C-	YMR316C-B	UWOPS87_ctrl_hmg124h_11.JPG.dat	1087.0	37.4	1.0	0.01	#NUM!	2.0E-05
YMR316W	YMR316W	UWOPS87_ctrl_hmg124h_11.JPG.dat	1138.3	64.5	1.0	0.01	#NUM!	2.7E-05
YMR317W	YMR317W	UWOPS87_ctrl_hmg124h_11.JPG.dat	1246.5	35.8	1.1	0.04	#NUM!	1.1E-05
YMR318C	YMR318C	UWOPS87_ctrl_hmg124h_11.JPG.dat	1311.0	112.5	1.1	0.10	#NUM!	1.7E-04
YMR319C	YMR319C	UWOPS87_ctrl_hmg124h_11.JPG.dat	1161.3	24.2	1.0	0.02	#NUM!	1.7E-06
YMR320W	YMR320W	UWOPS87_ctrl_hmg124h_11.JPG.dat	993.3	40.8	0.9	0.04	#NUM!	2.3E-05
YMR322C	YMR322C	UWOPS87_ctrl_hmg124h_11.JPG.dat	1065.8	127.4	1.0	0.12	#NUM!	4.3E-04
YMR326C	YMR326C	UWOPS87_ctrl_hmg124h_11.JPG.dat	993.0	43.4	1.0	0.01	#NUM!	1.8E-05
YNL001W	YNL001W	UWOPS87_ctrl_hmg124h_11.JPG.dat	983.3	69.2	0.9	0.08	#NUM!	1.9E-04
YNL003C	YNL003C	UWOPS87_ctrl_hmg124h_11.JPG.dat	766.8	74.4	0.8	0.08	#NUM!	3.2E-04
YNL004W	YNL004W	UWOPS87_ctrl_hmg124h_11.JPG.dat	897.0	16.1	0.9	0.02	#NUM!	3.3E-06
YNL008C	YNL008C	UWOPS87_ctrl_hmg124h_11.JPG.dat	1249.3	9.8	1.2	0.02	#NUM!	1.4E-06
YNL009W	YNL009W	UWOPS87_ctrl_hmg124h_11.JPG.dat	963.8	60.6	0.9	0.07	#NUM!	1.0E-04
YNL010W	YNL010W	UWOPS87_ctrl_hmg124h_11.JPG.dat	1023.0	86.3	1.0	0.09	#NUM!	1.9E-04
YNL012W	YNL012W	UWOPS87_ctrl_hmg124h_11.JPG.dat	1051.8	66.5	1.0	0.02	#NUM!	1.3E-04
YNL013C	YNL013C	UWOPS87_ctrl_hmg124h_11.JPG.dat	1103.5	55.7	1.1	0.06	#NUM!	6.1E-05
YNL015W	YNL015W	UWOPS87_ctrl_hmg124h_11.JPG.dat	958.5	40.6	1.0	0.03	#NUM!	6.0E-06
YNL016W	YNL016W	UWOPS87_ctrl_hmg124h_11.JPG.dat	960.3	9.1	1.0	0.01	#NUM!	1.1E-07
YNL020C	YNL020C	UWOPS87_ctrl_hmg124h_11.JPG.dat	995.0	32.9	1.0	0.01	#NUM!	3.7E-05
YNL021W	YNL021W	UWOPS87_ctrl_hmg124h_11.JPG.dat	1060.5	62.5	1.0	0.02	#NUM!	9.6E-05
YNL022C	YNL022C	UWOPS87_ctrl_hmg124h_11.JPG.dat	950.5	62.9	1.0	0.06	#NUM!	6.5E-05
YNL023C	YNL023C	UWOPS87_ctrl_hmg124h_11.JPG.dat	1013.5	74.2	1.0	0.05	#NUM!	4.7E-05
YNL024C	YNL024C	UWOPS87_ctrl_hmg124h_11.JPG.dat	929.0	83.4	1.0	0.10	#NUM!	2.9E-04
YNL027W	YNL027W	UWOPS87_ctrl_hmg124h_11.JPG.dat	1031.8	65.0	1.0	0.02	#NUM!	1.8E-04
YNL028W	YNL028W	UWOPS87_ctrl_hmg124h_11.JPG.dat	1021.3	55.8	0.9	0.02	#NUM!	9.0E-05
YNL029C	YNL029C	UWOPS87_ctrl_hmg124h_11.JPG.dat	1048.3	110.7	1.0	0.10	#NUM!	3.2E-04
YNL030W	YNL030W	UWOPS87_ctrl_hmg124h_11.JPG.dat	1310.8	76.6	1.2	0.07	#NUM!	5.4E-05
YNL031C	YNL031C	UWOPS87_ctrl_hmg124h_11.JPG.dat	1276.5	58.6	1.1	0.05	#NUM!	2.5E-05
YNL032W	YNL032W	UWOPS87_ctrl_hmg124h_11.JPG.dat	1060.0	33.6	0.9	0.03	#NUM!	7.8E-06
YNL034W	YNL034W	UWOPS87_ctrl_hmg124h_11.JPG.dat	1207.0	113.4	1.0	0.09	#NUM!	2.0E-04
YNL035C	YNL035C	UWOPS87_ctrl_hmg124h_11.JPG.dat	1223.5	32.1	1.1	0.02	#NUM!	1.4E-06
YNL037C	YNL037C	UWOPS87_ctrl_hmg124h_11.JPG.dat	947.0	111.4	1.1	0.01	#NUM!	1.9E-05
YNL040W	YNL040W	UWOPS87_ctrl_hmg124h_11.JPG.dat	920.0	153.1	0.9	0.14	#NUM!	1.0E-03

YNL041C	YNL041C	UWOPS87_ctrl_hmg124h_11.JPG.dat	947.0	139.1	1.1	0.04	#NUM!	3.8E-04
YNL043C	YNL043C	UWOPS87_ctrl_hmg124h_11.JPG.dat	1200.3	60.0	1.1	0.07	#NUM!	6.0E-05
YNL044W	YNL044W	UWOPS87_ctrl_hmg124h_11.JPG.dat	1229.0	154.4	1.1	0.14	#NUM!	5.4E-04
YNL045W	YNL045W	UWOPS87_ctrl_hmg124h_11.JPG.dat	1050.8	75.4	0.9	0.07	#NUM!	1.2E-04
YNL046W	YNL046W	UWOPS87_ctrl_hmg124h_11.JPG.dat	1159.3	61.1	1.0	0.04	#NUM!	2.7E-05
YNL049C	YNL049C	UWOPS87_ctrl_hmg124h_11.JPG.dat	1256.0	49.6	1.1	0.04	#NUM!	1.9E-05
YNL050C	YNL050C	UWOPS87_ctrl_hmg124h_11.JPG.dat	1144.3	46.5	1.0	0.05	#NUM!	3.1E-05
YNL051W	YNL051W	UWOPS87_ctrl_hmg124h_11.JPG.dat	1361.5	18.1	1.2	0.03	#NUM!	6.1E-06
YNL052W	YNL052W	UWOPS87_ctrl_hmg124h_11.JPG.dat	575.3	683.2	0.5	0.63	#NUM!	1.9E-01
YNL054W	YNL054W	UWOPS87_ctrl_hmg124h_11.JPG.dat	874.8	115.0	0.9	0.09	#NUM!	2.7E-04
YNL056W	YNL056W	UWOPS87_ctrl_hmg124h_11.JPG.dat	1136.8	29.8	1.1	0.04	#NUM!	1.4E-05
YNL057W	YNL057W	UWOPS87_ctrl_hmg124h_11.JPG.dat	964.5	156.3	1.0	0.15	#NUM!	9.0E-04
YNL058C	YNL058C	UWOPS87_ctrl_hmg124h_11.JPG.dat	1075.5	69.0	1.0	0.01	#NUM!	1.0E-05
YNL063W	YNL063W	UWOPS87_ctrl_hmg124h_11.JPG.dat	1193.0	49.2	1.0	0.03	#NUM!	6.1E-06
YNL064C	YNL064C	UWOPS87_ctrl_hmg124h_11.JPG.dat	1286.0	121.2	1.1	0.07	#NUM!	6.9E-05
YNL065W	YNL065W	UWOPS87_ctrl_hmg124h_11.JPG.dat	1321.5	48.8	1.1	0.03	#NUM!	4.0E-06
YNL066W	YNL066W	UWOPS87_ctrl_hmg124h_11.JPG.dat	1256.8	142.5	1.1	0.11	#NUM!	2.8E-04
YNL067W	YNL067W	UWOPS87_ctrl_hmg124h_11.JPG.dat	1054.0	47.0	0.9	0.01	#NUM!	1.0E-06
YNL068C	YNL068C	UWOPS87_ctrl_hmg124h_11.JPG.dat	1259.8	61.1	1.2	0.02	#NUM!	8.2E-05
YNL069C	YNL069C	UWOPS87_ctrl_hmg124h_11.JPG.dat	1122.0	58.1	1.1	0.05	#NUM!	3.1E-05
YNL070W	YNL070W	UWOPS87_ctrl_hmg124h_11.JPG.dat	987.0	33.1	1.0	0.01	#NUM!	2.4E-05
YNL071W	YNL071W	UWOPS87_ctrl_hmg124h_11.JPG.dat	1324.8	57.8	1.3	0.05	#NUM!	2.2E-05
YNL072W	YNL072W	UWOPS87_ctrl_hmg124h_11.JPG.dat	1299.3	164.1	1.1	0.14	#NUM!	5.7E-04
YNL074C	YNL074C	UWOPS87_ctrl_hmg124h_11.JPG.dat	1231.3	41.1	1.0	0.02	#NUM!	1.1E-06
YNL076W	YNL076W	UWOPS87_ctrl_hmg124h_11.JPG.dat	1321.8	71.8	1.1	0.08	#NUM!	9.9E-05
YNL077W	YNL077W	UWOPS87_ctrl_hmg124h_11.JPG.dat	1287.8	102.9	1.0	0.02	#NUM!	1.4E-04
YNL078W	YNL078W	UWOPS87_ctrl_hmg124h_11.JPG.dat	1294.0	52.8	1.0	0.04	#NUM!	2.2E-05
YNL079C	YNL079C	UWOPS87_ctrl_hmg124h_11.JPG.dat	983.3	142.2	0.9	0.04	#NUM!	8.1E-04
YNL080C	YNL080C	UWOPS87_ctrl_hmg124h_11.JPG.dat	1257.5	64.2	1.1	0.02	#NUM!	8.1E-05
YNL081C	YNL081C	UWOPS87_ctrl_hmg124h_11.JPG.dat	1146.0	98.0	1.1	0.09	#NUM!	1.5E-04
YNL082W	YNL082W	UWOPS87_ctrl_hmg124h_11.JPG.dat	1043.5	84.6	1.0	0.08	#NUM!	1.4E-04
YNL083W	YNL083W	UWOPS87_ctrl_hmg124h_11.JPG.dat	1037.8	89.8	0.9	0.01	#NUM!	4.2E-05
YNL085W	YNL085W	UWOPS87_ctrl_hmg124h_11.JPG.dat	933.5	67.4	0.9	0.05	#NUM!	6.4E-05
YNL087W	YNL087W	UWOPS87_ctrl_hmg124h_11.JPG.dat	1275.8	72.7	1.1	0.06	#NUM!	4.4E-05
YNL089C	YNL089C	UWOPS87_ctrl_hmg124h_11.JPG.dat	1341.8	33.1	1.1	0.03	#NUM!	6.0E-06
YNL090W	YNL090W	UWOPS87_ctrl_hmg124h_11.JPG.dat	1194.5	58.8	1.0	0.05	#NUM!	4.2E-05
YNL091W	YNL091W	UWOPS87_ctrl_hmg124h_11.JPG.dat	1204.3	167.2	1.0	0.13	#NUM!	7.4E-04
YNL092W	YNL092W	UWOPS87_ctrl_hmg124h_11.JPG.dat	1196.8	50.3	1.0	0.05	#NUM!	3.3E-05
YNL093W	YNL093W	UWOPS87_ctrl_hmg124h_11.JPG.dat	1240.3	59.0	1.1	0.06	#NUM!	3.6E-05
YNL094W	YNL094W	UWOPS87_ctrl_hmg124h_11.JPG.dat	851.5	58.3	0.7	0.01	#NUM!	8.3E-05
YNL095C	YNL095C	UWOPS87_ctrl_hmg124h_11.JPG.dat	1161.8	64.9	1.1	0.07	#NUM!	6.2E-05
YNL097C	YNL097C	UWOPS87_ctrl_hmg124h_11.JPG.dat	1079.3	95.3	1.0	0.11	#NUM!	2.9E-04
YNL098C	YNL098C	UWOPS87_ctrl_hmg124h_11.JPG.dat	1187.8	59.8	1.1	0.04	#NUM!	1.7E-05
YNL099C	YNL099C	UWOPS87_ctrl_hmg124h_11.JPG.dat	986.8	55.9	0.9	0.01	#NUM!	7.9E-05
YNL100W	YNL100W	UWOPS87_ctrl_hmg124h_11.JPG.dat	1200.5	34.5	1.0	0.03	#NUM!	8.9E-06
YNL101W	YNL101W	UWOPS87_ctrl_hmg124h_11.JPG.dat	1231.3	59.0	1.0	0.05	#NUM!	3.0E-05
YNL104C	YNL104C	UWOPS87_ctrl_hmg124h_11.JPG.dat	1370.3	21.8	1.2	0.02	#NUM!	1.6E-06
YNL105W	YNL105W	UWOPS87_ctrl_hmg124h_11.JPG.dat	1305.0	65.2	1.1	0.02	#NUM!	8.7E-05
YNL106C	YNL106C	UWOPS87_ctrl_hmg124h_11.JPG.dat	976.5	52.6	0.9	0.04	#NUM!	2.4E-05
YNL107W	YNL107W	UWOPS87_ctrl_hmg124h_11.JPG.dat	984.5	66.1	0.9	0.06	#NUM!	6.7E-05
YNL108C	YNL108C	UWOPS87_ctrl_hmg124h_11.JPG.dat	903.3	70.3	0.8	0.03	#NUM!	3.7E-04
YNL115C	YNL115C	UWOPS87_ctrl_hmg124h_11.JPG.dat	1049.5	48.4	1.0	0.05	#NUM!	3.1E-05
YNL116W	YNL116W	UWOPS87_ctrl_hmg124h_11.JPG.dat	944.0	83.9	0.9	0.08	#NUM!	1.5E-04
YNL117W	YNL117W	UWOPS87_ctrl_hmg124h_11.JPG.dat	1225.5	83.7	1.2	0.09	#NUM!	1.5E-04
YNL119W	YNL119W	UWOPS87_ctrl_hmg124h_11.JPG.dat	918.5	103.3	0.9	0.10	#NUM!	3.7E-04
YNL120C	YNL120C	UWOPS87_ctrl_hmg124h_11.JPG.dat	1068.8	99.4	1.0	0.09	#NUM!	2.2E-04
YNL121C	YNL121C	UWOPS87_ctrl_hmg124h_11.JPG.dat	1106.8	42.1	1.0	0.02	#NUM!	1.1E-04
YNL122C	YNL122C	UWOPS87_ctrl_hmg124h_11.JPG.dat	950.8	87.2	0.9	0.08	#NUM!	2.1E-04
YNL123W	YNL123W	UWOPS87_ctrl_hmg124h_11.JPG.dat	1055.3	36.3	1.0	0.03	#NUM!	6.4E-06
YNL125C	YNL125C	UWOPS87_ctrl_hmg124h_11.JPG.dat	1050.8	54.7	1.0	0.07	#NUM!	7.7E-05
YNL127W	YNL127W	UWOPS87_ctrl_hmg124h_11.JPG.dat	1061.3	57.1	1.1	0.04	#NUM!	2.0E-05
YNL128W	YNL128W	UWOPS87_ctrl_hmg124h_11.JPG.dat	1009.8	36.1	1.0	0.04	#NUM!	2.2E-05
YNL129W	YNL129W	UWOPS87_ctrl_hmg124h_11.JPG.dat	1085.0	40.5	1.1	0.01	#NUM!	5.6E-05
YNL130C	YNL130C	UWOPS87_ctrl_hmg124h_11.JPG.dat	1042.3	59.7	1.1	0.02	#NUM!	6.6E-05
YNL134C	YNL134C	UWOPS87_ctrl_hmg124h_11.JPG.dat	1051.3	22.6	1.1	0.02	#NUM!	2.7E-06
YNL135C	YNL135C	UWOPS87_ctrl_hmg124h_11.JPG.dat	971.8	56.3	1.0	0.01	#NUM!	1.9E-05
YNL136W	YNL136W	UWOPS87_ctrl_hmg124h_11.JPG.dat	924.0	44.9	0.9	0.06	#NUM!	5.7E-05
YNL140C	YNL140C	UWOPS87_ctrl_hmg124h_11.JPG.dat	1054.5	54.9	1.0	0.06	#NUM!	6.5E-05
YNL141W	YNL141W	UWOPS87_ctrl_hmg124h_11.JPG.dat	978.0	46.8	1.0	0.05	#NUM!	3.9E-05
YNL142W	YNL142W	UWOPS87_ctrl_hmg124h_11.JPG.dat	1032.8	90.3	1.0	0.08	#NUM!	1.5E-04

YNL143C	YNL143C	UWOPS87_ctrl_hmg124h_11.JPG.dat	1018.8	56.8	1.0	0.06	#NUM!	5.4E-05
YNL144C	YNL144C	UWOPS87_ctrl_hmg124h_11.JPG.dat	975.8	52.8	0.9	0.04	#NUM!	2.3E-05
YNL145W	YNL145W	UWOPS87_ctrl_hmg124h_11.JPG.dat	1345.5	58.1	1.2	0.04	#NUM!	8.3E-06
YNL146W	YNL146W	UWOPS87_ctrl_hmg124h_11.JPG.dat	1086.0	149.3	1.0	0.13	#NUM!	5.5E-04
YNL153C	YNL153C	UWOPS87_ctrl_hmg124h_11.JPG.dat	821.5	134.7	0.9	0.14	#NUM!	1.1E-03
YNL154C	YNL154C	UWOPS87_ctrl_hmg124h_11.JPG.dat	981.0	223.7	1.0	0.23	#NUM!	2.9E-03
YNL155W	YNL155W	UWOPS87_ctrl_hmg124h_11.JPG.dat	988.3	160.9	1.1	0.05	#NUM!	8.2E-04
YNL156C	YNL156C	UWOPS87_ctrl_hmg124h_11.JPG.dat	863.8	141.6	0.9	0.15	#NUM!	1.4E-03
YNL157W	YNL157W	UWOPS87_ctrl_hmg124h_11.JPG.dat	1148.0	43.0	1.1	0.02	#NUM!	7.1E-05
YNL159C	YNL159C	UWOPS87_ctrl_hmg124h_11.JPG.dat	1025.0	41.9	0.9	0.02	#NUM!	3.0E-06
YNL162W	YNL162W	UWOPS87_ctrl_hmg124h_11.JPG.dat	1103.8	39.5	1.0	0.05	#NUM!	2.7E-05
YNL164C	YNL164C	UWOPS87_ctrl_hmg124h_11.JPG.dat	1048.3	75.1	1.0	0.07	#NUM!	9.1E-05
YNL165W	YNL165W	UWOPS87_ctrl_hmg124h_11.JPG.dat	1330.3	80.7	1.2	0.02	#NUM!	1.6E-04
YNL166C	YNL166C	UWOPS87_ctrl_hmg124h_11.JPG.dat	962.3	50.8	0.9	0.01	#NUM!	7.8E-05
YNL167C	YNL167C	UWOPS87_ctrl_hmg124h_11.JPG.dat	857.8	30.6	0.9	0.01	#NUM!	3.8E-05
YNL168C	YNL168C	UWOPS87_ctrl_hmg124h_11.JPG.dat	1098.0	23.4	1.2	0.04	#NUM!	9.8E-06
YNL169C	YNL169C	UWOPS87_ctrl_hmg124h_11.JPG.dat	834.3	65.3	0.9	0.08	#NUM!	2.0E-04
YNL170W	YNL170W	UWOPS87_ctrl_hmg124h_11.JPG.dat	843.0	152.3	0.9	0.05	#NUM!	9.0E-04
YNL171C	YNL171C	UWOPS87_ctrl_hmg124h_11.JPG.dat	1042.8	24.1	1.0	0.03	#NUM!	6.8E-06
YNL173C	YNL173C	UWOPS87_ctrl_hmg124h_11.JPG.dat	913.3	68.6	0.8	0.07	#NUM!	1.4E-04
YNL175C	YNL175C	UWOPS87_ctrl_hmg124h_11.JPG.dat	892.0	102.8	0.8	0.10	#NUM!	4.9E-04
YNL176C	YNL176C	UWOPS87_ctrl_hmg124h_11.JPG.dat	971.0	85.8	0.8	0.08	#NUM!	2.0E-04
YNL179C	YNL179C	UWOPS87_ctrl_hmg124h_11.JPG.dat	1196.8	135.6	1.1	0.12	#NUM!	4.4E-04
YNL183C	YNL183C	UWOPS87_ctrl_hmg124h_11.JPG.dat	1206.3	69.5	1.1	0.07	#NUM!	6.4E-05
YNL187W	YNL187W	UWOPS87_ctrl_hmg124h_11.JPG.dat	1014.5	86.0	1.0	0.07	#NUM!	9.9E-05
YNL190W	YNL190W	UWOPS87_ctrl_hmg124h_11.JPG.dat	1021.3	117.1	1.0	0.11	#NUM!	3.4E-04
YNL191W	YNL191W	UWOPS87_ctrl_hmg124h_11.JPG.dat	971.0	48.3	1.1	0.06	#NUM!	4.1E-05
YNL192W	YNL192W	UWOPS87_ctrl_hmg124h_11.JPG.dat	837.5	20.9	0.9	0.02	#NUM!	4.3E-06
YNL193W	YNL193W	UWOPS87_ctrl_hmg124h_11.JPG.dat	1315.5	58.3	1.3	0.05	#NUM!	1.4E-05
YNL194C	YNL194C	UWOPS87_ctrl_hmg124h_11.JPG.dat	1035.8	108.0	1.0	0.10	#NUM!	2.8E-04
YNL195C	YNL195C	UWOPS87_ctrl_hmg124h_11.JPG.dat	1021.0	73.2	0.9	0.07	#NUM!	1.0E-04
YNL196C	YNL196C	UWOPS87_ctrl_hmg124h_11.JPG.dat	1022.5	176.7	0.9	0.17	#NUM!	1.7E-03
YNL197C	YNL197C	UWOPS87_ctrl_hmg124h_11.JPG.dat	1085.3	81.5	0.9	0.07	#NUM!	1.3E-04
YNL198C	YNL198C	UWOPS87_ctrl_hmg124h_11.JPG.dat	939.8	79.0	0.9	0.02	#NUM!	1.4E-04
YNL199C	YNL199C	UWOPS87_ctrl_hmg124h_11.JPG.dat	1371.5	55.6	1.2	0.02	#NUM!	5.8E-05
YNL200C	YNL200C	UWOPS87_ctrl_hmg124h_11.JPG.dat	978.8	46.8	1.0	0.01	#NUM!	3.2E-05
YNL201C	YNL201C	UWOPS87_ctrl_hmg124h_11.JPG.dat	950.0	46.1	1.0	0.04	#NUM!	2.0E-05
YNL202W	YNL202W	UWOPS87_ctrl_hmg124h_11.JPG.dat	940.3	224.8	1.0	0.25	#NUM!	4.0E-03
YNL203C	YNL203C	UWOPS87_ctrl_hmg124h_11.JPG.dat	984.8	62.6	1.0	0.06	#NUM!	7.1E-05
YNL204C	YNL204C	UWOPS87_ctrl_hmg124h_11.JPG.dat	1021.3	144.7	1.0	0.14	#NUM!	7.4E-04
YNL205C	YNL205C	UWOPS87_ctrl_hmg124h_11.JPG.dat	908.8	18.3	0.8	0.02	#NUM!	2.5E-06
YNL206C	YNL206C	UWOPS87_ctrl_hmg124h_11.JPG.dat	1171.3	65.7	1.0	0.02	#NUM!	7.5E-05
YNL208W	YNL208W	UWOPS87_ctrl_hmg124h_11.JPG.dat	1055.0	50.3	0.9	0.04	#NUM!	3.0E-05
YNL211C	YNL211C	UWOPS87_ctrl_hmg124h_11.JPG.dat	1378.5	34.2	1.2	0.03	#NUM!	2.7E-06
YNL212W	YNL212W	UWOPS87_ctrl_hmg124h_11.JPG.dat	966.8	69.0	0.9	0.01	#NUM!	2.0E-05
YNL214W	YNL214W	UWOPS87_ctrl_hmg124h_11.JPG.dat	1263.0	83.2	1.1	0.03	#NUM!	2.7E-04
YNL215W	YNL215W	UWOPS87_ctrl_hmg124h_11.JPG.dat	1008.3	106.0	1.0	0.11	#NUM!	3.3E-04
YNL217W	YNL217W	UWOPS87_ctrl_hmg124h_11.JPG.dat	934.0	37.0	0.9	0.04	#NUM!	1.9E-05
YNL218W	YNL218W	UWOPS87_ctrl_hmg124h_11.JPG.dat	1001.0	67.9	1.0	0.07	#NUM!	7.9E-05
YNL219C	YNL219C	UWOPS87_ctrl_hmg124h_11.JPG.dat	1198.3	238.0	1.3	0.01	#NUM!	2.1E-05
YNL223W	YNL223W	UWOPS87_ctrl_hmg124h_11.JPG.dat	1078.3	133.8	1.0	0.11	#NUM!	3.5E-04
YNL224C	YNL224C	UWOPS87_ctrl_hmg124h_11.JPG.dat	1002.8	69.6	0.9	0.00	#NUM!	6.7E-06
YNL226W	YNL226W	UWOPS87_ctrl_hmg124h_11.JPG.dat	1325.5	62.3	1.1	0.07	#NUM!	5.8E-05
YNL227C	YNL227C	UWOPS87_ctrl_hmg124h_11.JPG.dat	1094.8	72.0	0.9	0.05	#NUM!	3.8E-05
YNL228W	YNL228W	UWOPS87_ctrl_hmg124h_11.JPG.dat	1276.5	77.8	1.1	0.02	#NUM!	8.3E-05
YNL229C	YNL229C	UWOPS87_ctrl_hmg124h_11.JPG.dat	887.3	83.9	0.8	0.09	#NUM!	3.9E-04
YNL230C	YNL230C	UWOPS87_ctrl_hmg124h_11.JPG.dat	999.8	116.7	0.9	0.10	#NUM!	3.1E-04
YNL231C	YNL231C	UWOPS87_ctrl_hmg124h_11.JPG.dat	1103.0	58.2	1.1	0.06	#NUM!	5.0E-05
YNL233W	YNL233W	UWOPS87_ctrl_hmg124h_11.JPG.dat	1066.8	45.7	1.1	0.05	#NUM!	2.8E-05
YNL234W	YNL234W	UWOPS87_ctrl_hmg124h_11.JPG.dat	946.0	46.8	0.9	0.05	#NUM!	4.3E-05
YNL235C	YNL235C	UWOPS87_ctrl_hmg124h_11.JPG.dat	893.5	25.3	0.8	0.03	#NUM!	1.1E-05
YNL236W	YNL236W	UWOPS87_ctrl_hmg124h_11.JPG.dat	1070.0	45.6	1.0	0.03	#NUM!	5.5E-06
YNL237W	YNL237W	UWOPS87_ctrl_hmg124h_11.JPG.dat	1198.3	127.6	1.1	0.11	#NUM!	3.0E-04
YNL238W	YNL238W	UWOPS87_ctrl_hmg124h_11.JPG.dat	1265.5	46.5	1.1	0.04	#NUM!	9.9E-06
YNL239W	YNL239W	UWOPS87_ctrl_hmg124h_11.JPG.dat	1154.0	164.8	1.0	0.14	#NUM!	7.4E-04
YNL241C	YNL241C	UWOPS87_ctrl_hmg124h_11.JPG.dat	1040.8	89.7	0.9	0.02	#NUM!	1.4E-04
YNL242W	YNL242W	UWOPS87_ctrl_hmg124h_11.JPG.dat	1228.8	61.5	1.2	0.02	#NUM!	9.5E-05
YNL246W	YNL246W	UWOPS87_ctrl_hmg124h_11.JPG.dat	863.5	78.8	0.8	0.08	#NUM!	2.5E-04
YNL249C	YNL249C	UWOPS87_ctrl_hmg124h_11.JPG.dat	1089.8	40.7	1.1	0.04	#NUM!	1.1E-05
YNL253W	YNL253W	UWOPS87_ctrl_hmg124h_11.JPG.dat	996.0	29.8	1.0	0.03	#NUM!	8.2E-06

YNL254C	YNL254C	UWOPS87_ctrl_hmg124h_11.JPG.dat	1050.8	36.6	1.1	0.04	#NUM!	1.0E-05
YNL255C	YNL255C	UWOPS87_ctrl_hmg124h_11.JPG.dat	1030.3	39.2	1.0	0.06	#NUM!	4.9E-05
YNL257C	YNL257C	UWOPS87_ctrl_hmg124h_11.JPG.dat	989.5	40.8	1.0	0.03	#NUM!	9.2E-06
YNL259C	YNL259C	UWOPS87_ctrl_hmg124h_11.JPG.dat	1131.8	122.8	1.1	0.10	#NUM!	2.0E-04
YNL265C	YNL265C	UWOPS87_ctrl_hmg124h_11.JPG.dat	961.3	36.8	0.9	0.04	#NUM!	1.7E-05
YNL266W	YNL266W	UWOPS87_ctrl_hmg124h_11.JPG.dat	991.8	16.2	1.0	0.02	#NUM!	4.3E-06
YNL268W	YNL268W	UWOPS87_ctrl_hmg124h_11.JPG.dat	1010.8	81.6	1.0	0.07	#NUM!	1.1E-04
YNL270C	YNL270C	UWOPS87_ctrl_hmg124h_11.JPG.dat	1061.3	68.1	1.0	0.07	#NUM!	9.8E-05
YNL271C	YNL271C	UWOPS87_ctrl_hmg124h_11.JPG.dat	1029.0	81.4	1.0	0.07	#NUM!	9.9E-05
YNL273W	YNL273W	UWOPS87_ctrl_hmg124h_11.JPG.dat	1103.3	71.1	1.0	0.02	#NUM!	1.2E-04
YNL274C	YNL274C	UWOPS87_ctrl_hmg124h_11.JPG.dat	1061.5	66.6	1.0	0.07	#NUM!	8.6E-05
YNL275W	YNL275W	UWOPS87_ctrl_hmg124h_11.JPG.dat	770.3	85.5	0.8	0.09	#NUM!	3.7E-04
YNL277W	YNL277W	UWOPS87_ctrl_hmg124h_11.JPG.dat	889.8	60.4	0.9	0.06	#NUM!	8.6E-05
YNL278W	YNL278W	UWOPS87_ctrl_hmg124h_11.JPG.dat	944.8	57.4	1.0	0.06	#NUM!	5.8E-05
YNL279W	YNL279W	UWOPS87_ctrl_hmg124h_11.JPG.dat	1021.5	129.5	1.0	0.11	#NUM!	4.0E-04
YNL280C	YNL280C	UWOPS87_ctrl_hmg124h_11.JPG.dat	974.0	77.5	0.9	0.08	#NUM!	1.4E-04
YNL281W	YNL281W	UWOPS87_ctrl_hmg124h_11.JPG.dat	1144.8	29.0	1.1	0.04	#NUM!	1.6E-05
YNL283C	YNL283C	UWOPS87_ctrl_hmg124h_11.JPG.dat	1315.5	43.8	1.2	0.04	#NUM!	1.2E-05
YNL285W	YNL285W	UWOPS87_ctrl_hmg124h_11.JPG.dat	1302.5	45.8	1.2	0.04	#NUM!	1.6E-05
YNL286W	YNL286W	UWOPS87_ctrl_hmg124h_11.JPG.dat	1081.3	147.5	1.0	0.13	#NUM!	6.7E-04
YNL288W	YNL288W	UWOPS87_ctrl_hmg124h_11.JPG.dat	1301.3	53.7	1.2	0.06	#NUM!	4.0E-05
YNL289W	YNL289W	UWOPS87_ctrl_hmg124h_11.JPG.dat	1110.5	67.2	1.0	0.02	#NUM!	1.0E-04
YNL291C	YNL291C	UWOPS87_ctrl_hmg124h_11.JPG.dat	972.5	65.0	1.1	0.06	#NUM!	5.2E-05
YNL292W	YNL292W	UWOPS87_ctrl_hmg124h_11.JPG.dat	951.5	37.0	1.0	0.03	#NUM!	1.1E-05
YNL293W	YNL293W	UWOPS87_ctrl_hmg124h_11.JPG.dat	955.5	48.9	1.0	0.06	#NUM!	5.2E-05
YNL294C	YNL294C	UWOPS87_ctrl_hmg124h_11.JPG.dat	1337.5	17.3	1.3	0.01	#NUM!	2.7E-07
YNL295W	YNL295W	UWOPS87_ctrl_hmg124h_11.JPG.dat	1202.8	42.6	1.1	0.04	#NUM!	1.7E-05
YNL296W	YNL296W	UWOPS87_ctrl_hmg124h_11.JPG.dat	1152.8	85.4	1.0	0.07	#NUM!	8.1E-05
YNL297C	YNL297C	UWOPS87_ctrl_hmg124h_11.JPG.dat	1173.0	43.0	1.0	0.04	#NUM!	2.1E-05
YNL298W	YNL298W	UWOPS87_ctrl_hmg124h_11.JPG.dat	1214.5	57.8	1.0	0.04	#NUM!	1.7E-05
YNL299W	YNL299W	UWOPS87_ctrl_hmg124h_11.JPG.dat	1102.0	43.8	0.9	0.01	#NUM!	2.7E-05
YNL300W	YNL300W	UWOPS87_ctrl_hmg124h_11.JPG.dat	1090.0	115.0	1.0	0.12	#NUM!	4.4E-04
YNL301C	YNL301C	UWOPS87_ctrl_hmg124h_11.JPG.dat	997.3	43.7	1.0	0.04	#NUM!	1.7E-05
YNL302C	YNL302C	UWOPS87_ctrl_hmg124h_11.JPG.dat	546.8	87.7	0.6	0.10	#NUM!	1.1E-03
YNL303W	YNL303W	UWOPS87_ctrl_hmg124h_11.JPG.dat	915.5	43.5	1.0	0.05	#NUM!	5.0E-05
YNL304W	YNL304W	UWOPS87_ctrl_hmg124h_11.JPG.dat	895.3	54.1	0.9	0.05	#NUM!	5.1E-05
YNL305C	YNL305C	UWOPS87_ctrl_hmg124h_11.JPG.dat	972.8	34.0	0.9	0.03	#NUM!	1.1E-05
YNL307C	YNL307C	UWOPS87_ctrl_hmg124h_11.JPG.dat	1234.0	44.7	1.1	0.04	#NUM!	1.6E-05
YNL309W	YNL309W	UWOPS87_ctrl_hmg124h_11.JPG.dat	1171.3	44.4	1.0	0.04	#NUM!	1.5E-05
YNL311C	YNL311C	UWOPS87_ctrl_hmg124h_11.JPG.dat	1192.3	40.3	1.0	0.01	#NUM!	3.9E-05
YNL314W	YNL314W	UWOPS87_ctrl_hmg124h_11.JPG.dat	1167.5	92.5	1.0	0.02	#NUM!	1.5E-04
YNL315C	YNL315C	UWOPS87_ctrl_hmg124h_11.JPG.dat	1339.5	25.5	1.2	0.03	#NUM!	4.9E-06
YNL316C	YNL316C	UWOPS87_ctrl_hmg124h_11.JPG.dat	0.0	0.0	0.0	0.00	#NUM!	
YNL318C	YNL318C	UWOPS87_ctrl_hmg124h_11.JPG.dat	1069.0	86.2	1.0	0.02	#NUM!	1.4E-04
YNL319W	YNL319W	UWOPS87_ctrl_hmg124h_11.JPG.dat	893.5	21.8	0.9	0.03	#NUM!	5.2E-06
YNL320W	YNL320W	UWOPS87_ctrl_hmg124h_11.JPG.dat	1220.0	122.3	1.2	0.11	#NUM!	2.1E-04
YNL321W	YNL321W	UWOPS87_ctrl_hmg124h_11.JPG.dat	1058.8	91.0	1.1	0.09	#NUM!	1.5E-04
YNL322C	YNL322C	UWOPS87_ctrl_hmg124h_11.JPG.dat	1066.3	71.9	1.0	0.07	#NUM!	8.8E-05
YNL323W	YNL323W	UWOPS87_ctrl_hmg124h_11.JPG.dat	1018.5	57.5	0.9	0.05	#NUM!	4.9E-05
YNL324W	YNL324W	UWOPS87_ctrl_hmg124h_11.JPG.dat	997.0	61.1	0.9	0.05	#NUM!	6.3E-05
YNL325C	YNL325C	UWOPS87_ctrl_hmg124h_11.JPG.dat	1284.3	41.5	1.1	0.04	#NUM!	1.1E-05
YNL326C	YNL326C	UWOPS87_ctrl_hmg124h_11.JPG.dat	1341.8	49.1	1.1	0.02	#NUM!	8.0E-05
YNL327W	YNL327W	UWOPS87_ctrl_hmg124h_11.JPG.dat	1311.0	52.5	1.2	0.05	#NUM!	2.5E-05
YNL328C	YNL328C	UWOPS87_ctrl_hmg124h_11.JPG.dat	1056.5	115.2	1.0	0.01	#NUM!	8.1E-05
YNL329C	YNL329C	UWOPS87_ctrl_hmg124h_11.JPG.dat	1031.3	30.9	1.0	0.01	#NUM!	2.5E-05
YNL330C	YNL330C	UWOPS87_ctrl_hmg124h_11.JPG.dat	845.8	38.7	0.9	0.04	#NUM!	2.2E-05
YNL332W	YNL332W	UWOPS87_ctrl_hmg124h_11.JPG.dat	1177.5	49.7	1.1	0.04	#NUM!	9.7E-06
YNL333W	YNL333W	UWOPS87_ctrl_hmg124h_11.JPG.dat	1122.0	29.7	1.1	0.03	#NUM!	4.5E-06
YNL334C	YNL334C	UWOPS87_ctrl_hmg124h_11.JPG.dat	1226.5	250.3	1.1	0.22	#NUM!	2.4E-03
YNL335W	YNL335W	UWOPS87_ctrl_hmg124h_11.JPG.dat	1145.0	22.3	1.0	0.02	#NUM!	1.3E-06
YNL336W	YNL336W	UWOPS87_ctrl_hmg124h_11.JPG.dat	943.5	633.9	1.1	0.07	#NUM!	1.5E-03
YNL338W	YNL338W	UWOPS87_ctrl_hmg124h_11.JPG.dat	1126.0	61.3	0.9	0.05	#NUM!	3.3E-05
YNL339C	YNL339C	UWOPS87_ctrl_hmg124h_11.JPG.dat	972.5	65.9	0.9	0.07	#NUM!	1.2E-04
YNR001C	YNR001C	UWOPS87_ctrl_hmg124h_11.JPG.dat	1083.3	78.6	1.0	0.08	#NUM!	1.5E-04
YNR002C	YNR002C	UWOPS87_ctrl_hmg124h_11.JPG.dat	1132.5	84.4	1.1	0.08	#NUM!	9.6E-05
YNR004W	YNR004W	UWOPS87_ctrl_hmg124h_11.JPG.dat	992.0	92.4	1.0	0.08	#NUM!	1.7E-04
YNR005C	YNR005C	UWOPS87_ctrl_hmg124h_11.JPG.dat	1139.3	56.5	1.1	0.01	#NUM!	1.8E-05
YNR006W	YNR006W	UWOPS87_ctrl_hmg124h_11.JPG.dat	1199.5	74.7	1.1	0.02	#NUM!	1.2E-04
YNR007C	YNR007C	UWOPS87_ctrl_hmg124h_11.JPG.dat	1027.3	62.4	1.0	0.06	#NUM!	6.0E-05
YNR008W	YNR008W	UWOPS87_ctrl_hmg124h_11.JPG.dat	1367.8	55.6	1.2	0.01	#NUM!	4.1E-05

YNR009W	YNR009W	UWOPS87_ctrl_hmg124h_11.JPG.dat	959.5	112.1	0.9	0.09	#NUM!	3.1E-04
YNR010W	YNR010W	UWOPS87_ctrl_hmg124h_11.JPG.dat	934.0	39.5	0.8	0.04	#NUM!	2.6E-05
YNR012W	YNR012W	UWOPS87_ctrl_hmg124h_11.JPG.dat	1296.8	22.4	1.1	0.02	#NUM!	7.2E-07
YNR013C	YNR013C	UWOPS87_ctrl_hmg124h_11.JPG.dat	856.8	110.5	0.8	0.02	#NUM!	2.3E-04
YNR014W	YNR014W	UWOPS87_ctrl_hmg124h_11.JPG.dat	1163.3	161.3	1.1	0.15	#NUM!	6.0E-04
YNR015W	YNR015W	UWOPS87_ctrl_hmg124h_11.JPG.dat	994.3	53.2	1.0	0.05	#NUM!	3.7E-05
YNR018W	YNR018W	UWOPS87_ctrl_hmg124h_11.JPG.dat	1017.5	39.5	1.0	0.01	#NUM!	1.8E-05
YOR202W	YOR202W	UWOPS87_ctrl_hmg124h_11.JPG.dat	1210.6	178.8	1.0	0.09	#NUM!	4.0E-283
1	1	UWOPS87_ctrl_hmg124h_12.JPG.dat	1538.0	221.7	1.1	0.08	#NUM!	1.1E-04
2	2	UWOPS87_ctrl_hmg124h_12.JPG.dat	1178.0	158.3	0.9	0.08	#NUM!	1.9E-04
3	3	UWOPS87_ctrl_hmg124h_12.JPG.dat	1139.5	144.9	0.9	0.07	#NUM!	1.0E-04
4	4	UWOPS87_ctrl_hmg124h_12.JPG.dat	1183.8	130.1	1.0	0.05	#NUM!	4.1E-05
YNR019W	YNR019W	UWOPS87_ctrl_hmg124h_12.JPG.dat	1031.5	99.4	1.0	0.11	#NUM!	3.4E-04
YNR020C	YNR020C	UWOPS87_ctrl_hmg124h_12.JPG.dat	0.0	0.0	0.0	0.00	#NUM!	
YNR021W	YNR021W	UWOPS87_ctrl_hmg124h_12.JPG.dat	0.0	0.0	0.0	0.00	#NUM!	
YNR022C	YNR022C	UWOPS87_ctrl_hmg124h_12.JPG.dat	938.5	53.2	0.9	0.06	#NUM!	9.3E-05
YNR024W	YNR024W	UWOPS87_ctrl_hmg124h_12.JPG.dat	953.5	74.2	0.9	0.07	#NUM!	1.4E-04
YNR025C	YNR025C	UWOPS87_ctrl_hmg124h_12.JPG.dat	1107.5	52.2	1.1	0.01	#NUM!	4.3E-05
YNR027W	YNR027W	UWOPS87_ctrl_hmg124h_12.JPG.dat	994.0	117.2	0.8	0.02	#NUM!	2.3E-04
YNR028W	YNR028W	UWOPS87_ctrl_hmg124h_12.JPG.dat	1107.8	82.2	1.0	0.09	#NUM!	1.8E-04
YNR029C	YNR029C	UWOPS87_ctrl_hmg124h_12.JPG.dat	995.5	57.9	0.9	0.01	#NUM!	2.8E-05
YNR030W	YNR030W	UWOPS87_ctrl_hmg124h_12.JPG.dat	1344.0	71.1	1.2	0.05	#NUM!	2.0E-05
YNR031C	YNR031C	UWOPS87_ctrl_hmg124h_12.JPG.dat	991.8	242.3	0.9	0.22	#NUM!	3.6E-03
YNR032C-/	YNR032C-A	UWOPS87_ctrl_hmg124h_12.JPG.dat	957.8	22.7	1.0	0.02	#NUM!	3.1E-06
YNR032W	YNR032W	UWOPS87_ctrl_hmg124h_12.JPG.dat	1183.3	28.3	1.2	0.02	#NUM!	6.7E-07
YNR034W	YNR034W	UWOPS87_ctrl_hmg124h_12.JPG.dat	1170.0	138.2	1.1	0.12	#NUM!	4.1E-04
YNR039C	YNR039C	UWOPS87_ctrl_hmg124h_12.JPG.dat	783.5	45.6	0.7	0.05	#NUM!	7.0E-05
YNR040W	YNR040W	UWOPS87_ctrl_hmg124h_12.JPG.dat	1102.8	151.4	1.0	0.03	#NUM!	3.8E-04
YNR042W	YNR042W	UWOPS87_ctrl_hmg124h_12.JPG.dat	1071.0	39.2	1.0	0.03	#NUM!	9.7E-06
YNR045W	YNR045W	UWOPS87_ctrl_hmg124h_12.JPG.dat	1231.3	79.0	1.1	0.07	#NUM!	6.5E-05
YNR047W	YNR047W	UWOPS87_ctrl_hmg124h_12.JPG.dat	1213.8	129.0	1.1	0.12	#NUM!	3.0E-04
YNR048W	YNR048W	UWOPS87_ctrl_hmg124h_12.JPG.dat	1333.8	49.4	1.2	0.00	#NUM!	1.6E-06
YNR049C	YNR049C	UWOPS87_ctrl_hmg124h_12.JPG.dat	1106.5	67.2	0.9	0.01	#NUM!	7.5E-05
YNR050C	YNR050C	UWOPS87_ctrl_hmg124h_12.JPG.dat	142.0	284.0	0.0	0.00	#NUM!	
YNR051C	YNR051C	UWOPS87_ctrl_hmg124h_12.JPG.dat	780.8	100.1	0.8	0.02	#NUM!	2.1E-04
YNR055C	YNR055C	UWOPS87_ctrl_hmg124h_12.JPG.dat	974.3	46.7	1.0	0.05	#NUM!	3.4E-05
YNR056C	YNR056C	UWOPS87_ctrl_hmg124h_12.JPG.dat	986.0	93.0	0.9	0.09	#NUM!	2.7E-04
YNR057C	YNR057C	UWOPS87_ctrl_hmg124h_12.JPG.dat	1144.8	114.8	1.1	0.02	#NUM!	8.0E-05
YNR058W	YNR058W	UWOPS87_ctrl_hmg124h_12.JPG.dat	997.0	31.2	0.9	0.04	#NUM!	1.9E-05
YNR059W	YNR059W	UWOPS87_ctrl_hmg124h_12.JPG.dat	1243.0	38.0	1.1	0.04	#NUM!	7.9E-06
YNR060W	YNR060W	UWOPS87_ctrl_hmg124h_12.JPG.dat	883.5	237.1	0.9	0.05	#NUM!	1.1E-03
YNR061C	YNR061C	UWOPS87_ctrl_hmg124h_12.JPG.dat	1164.8	72.9	1.1	0.07	#NUM!	6.8E-05
YNR062C	YNR062C	UWOPS87_ctrl_hmg124h_12.JPG.dat	1154.8	88.2	1.0	0.08	#NUM!	1.2E-04
YNR063W	YNR063W	UWOPS87_ctrl_hmg124h_12.JPG.dat	1222.8	45.3	1.1	0.03	#NUM!	6.0E-06
YNR064C	YNR064C	UWOPS87_ctrl_hmg124h_12.JPG.dat	1035.8	88.8	1.0	0.09	#NUM!	1.8E-04
YNR065C	YNR065C	UWOPS87_ctrl_hmg124h_12.JPG.dat	1064.8	72.4	1.1	0.00	#NUM!	1.1E-06
YNR066C	YNR066C	UWOPS87_ctrl_hmg124h_12.JPG.dat	1054.5	108.4	1.1	0.04	#NUM!	5.1E-04
YNR067C	YNR067C	UWOPS87_ctrl_hmg124h_12.JPG.dat	1205.3	74.2	1.1	0.02	#NUM!	1.0E-04
YNR069C	YNR069C	UWOPS87_ctrl_hmg124h_12.JPG.dat	1085.5	83.6	1.0	0.07	#NUM!	9.3E-05
YNR071C	YNR071C	UWOPS87_ctrl_hmg124h_12.JPG.dat	1265.8	30.6	1.1	0.04	#NUM!	1.4E-05
YNR072W	YNR072W	UWOPS87_ctrl_hmg124h_12.JPG.dat	990.0	92.4	0.9	0.07	#NUM!	1.6E-04
YNR073C	YNR073C	UWOPS87_ctrl_hmg124h_12.JPG.dat	1203.0	140.9	1.0	0.11	#NUM!	3.1E-04
YNR074C	YNR074C	UWOPS87_ctrl_hmg124h_12.JPG.dat	1113.3	103.9	1.0	0.08	#NUM!	1.7E-04
YNR075W	YNR075W	UWOPS87_ctrl_hmg124h_12.JPG.dat	1093.8	108.6	0.9	0.10	#NUM!	3.0E-04
YOL001W	YOL001W	UWOPS87_ctrl_hmg124h_12.JPG.dat	1106.3	105.2	1.0	0.09	#NUM!	2.4E-04
YOL002C	YOL002C	UWOPS87_ctrl_hmg124h_12.JPG.dat	1184.8	41.5	1.1	0.03	#NUM!	5.2E-06
YOL003C	YOL003C	UWOPS87_ctrl_hmg124h_12.JPG.dat	1041.5	137.4	1.2	0.16	#NUM!	6.9E-04
YOL004W	YOL004W	UWOPS87_ctrl_hmg124h_12.JPG.dat	956.8	57.9	1.0	0.04	#NUM!	1.6E-05
YOL006C	YOL006C	UWOPS87_ctrl_hmg124h_12.JPG.dat	1161.5	84.0	1.1	0.01	#NUM!	1.2E-05
YOL007C	YOL007C	UWOPS87_ctrl_hmg124h_12.JPG.dat	1143.0	72.4	1.1	0.07	#NUM!	9.1E-05
YOL008W	YOL008W	UWOPS87_ctrl_hmg124h_12.JPG.dat	1248.5	245.2	1.3	0.02	#NUM!	9.8E-05
YOL009C	YOL009C	UWOPS87_ctrl_hmg124h_12.JPG.dat	975.8	85.3	0.9	0.09	#NUM!	2.4E-04
YOL011W	YOL011W	UWOPS87_ctrl_hmg124h_12.JPG.dat	1037.5	39.6	0.9	0.03	#NUM!	6.9E-06
YOL012C	YOL012C	UWOPS87_ctrl_hmg124h_12.JPG.dat	794.3	446.9	1.0	0.01	#NUM!	5.1E-05
YOL013C	YOL013C	UWOPS87_ctrl_hmg124h_12.JPG.dat	1156.3	239.2	1.2	0.08	#NUM!	1.5E-03
YOL013W-	YOL013W-A	UWOPS87_ctrl_hmg124h_12.JPG.dat	1133.5	139.9	1.0	0.13	#NUM!	5.2E-04
YOL014W	YOL014W	UWOPS87_ctrl_hmg124h_12.JPG.dat	1007.0	35.7	1.0	0.05	#NUM!	4.3E-05
YOL015W	YOL015W	UWOPS87_ctrl_hmg124h_12.JPG.dat	1073.0	169.1	1.2	0.18	#NUM!	9.5E-04
YOL016C	YOL016C	UWOPS87_ctrl_hmg124h_12.JPG.dat	797.5	54.4	0.8	0.02	#NUM!	1.5E-04
YOL017W	YOL017W	UWOPS87_ctrl_hmg124h_12.JPG.dat	1078.0	165.9	0.9	0.15	#NUM!	1.0E-03

YOL018C	YOL018C	UWOPS87_ctrl_hmg124h_12.JPG.dat	1174.5	54.0	1.0	0.05	#NUM!		2.5E-05
YOL019W	YOL019W	UWOPS87_ctrl_hmg124h_12.JPG.dat	1063.3	85.3	0.9	0.07	#NUM!		1.3E-04
YOL020W	YOL020W	UWOPS87_ctrl_hmg124h_12.JPG.dat	969.0	149.1	0.9	0.13	#NUM!		9.9E-04
YOL024W	YOL024W	UWOPS87_ctrl_hmg124h_12.JPG.dat	918.3	44.7	0.8	0.01	#NUM!		1.1E-04
YOL025W	YOL025W	UWOPS87_ctrl_hmg124h_12.JPG.dat	1116.3	84.5	1.0	0.08	#NUM!		1.3E-04
YOL027C	YOL027C	UWOPS87_ctrl_hmg124h_12.JPG.dat	308.3	358.3	0.3	0.32	#NUM!		1.8E-01
YOL028C	YOL028C	UWOPS87_ctrl_hmg124h_12.JPG.dat	1094.5	140.4	1.0	0.13	#NUM!		6.1E-04
YOL029C	YOL029C	UWOPS87_ctrl_hmg124h_12.JPG.dat	986.5	18.8	0.9	0.01	#NUM!		9.0E-07
YOL030W	YOL030W	UWOPS87_ctrl_hmg124h_12.JPG.dat	680.0	211.7	0.7	0.22	#NUM!		7.2E-03
YOL031C	YOL031C	UWOPS87_ctrl_hmg124h_12.JPG.dat	972.5	84.8	1.0	0.10	#NUM!		2.7E-04
YOL032W	YOL032W	UWOPS87_ctrl_hmg124h_12.JPG.dat	1014.3	194.3	0.9	0.16	#NUM!		1.4E-03
YOL035C	YOL035C	UWOPS87_ctrl_hmg124h_12.JPG.dat	1042.3	47.8	1.0	0.02	#NUM!		4.1E-06
YOL036W	YOL036W	UWOPS87_ctrl_hmg124h_12.JPG.dat	935.3	76.7	0.9	0.08	#NUM!		2.4E-04
YOL037C	YOL037C	UWOPS87_ctrl_hmg124h_12.JPG.dat	1135.8	138.9	1.1	0.04	#NUM!		4.0E-04
YOL039W	YOL039W	UWOPS87_ctrl_hmg124h_12.JPG.dat	1032.3	149.8	0.9	0.12	#NUM!		7.1E-04
YOL041C	YOL041C	UWOPS87_ctrl_hmg124h_12.JPG.dat	994.3	34.6	0.9	0.04	#NUM!		2.4E-05
YOL042W	YOL042W	UWOPS87_ctrl_hmg124h_12.JPG.dat	941.8	52.4	0.8	0.04	#NUM!		2.9E-05
YOL043C	YOL043C	UWOPS87_ctrl_hmg124h_12.JPG.dat	1217.0	41.8	1.1	0.04	#NUM!		1.7E-05
YOL044W	YOL044W	UWOPS87_ctrl_hmg124h_12.JPG.dat	1012.8	27.9	0.9	0.03	#NUM!		1.2E-05
YOL045W	YOL045W	UWOPS87_ctrl_hmg124h_12.JPG.dat	930.3	112.2	0.9	0.09	#NUM!		2.7E-04
YOL046C	YOL046C	UWOPS87_ctrl_hmg124h_12.JPG.dat	1172.5	75.9	1.2	0.10	#NUM!		1.6E-04
YOL047C	YOL047C	UWOPS87_ctrl_hmg124h_12.JPG.dat	0.0	0.0	0.0	0.00	#NUM!		
YOL048C	YOL048C	UWOPS87_ctrl_hmg124h_12.JPG.dat	175.5	351.0	0.0	0.00	#NUM!		
YOL049W	YOL049W	UWOPS87_ctrl_hmg124h_12.JPG.dat	1308.0	58.2	1.2	0.06	#NUM!		3.4E-05
YOL050C	YOL050C	UWOPS87_ctrl_hmg124h_12.JPG.dat	1144.0	106.7	1.0	0.08	#NUM!		1.4E-04
YOL052C	YOL052C	UWOPS87_ctrl_hmg124h_12.JPG.dat	1231.0	140.1	1.1	0.14	#NUM!		5.0E-04
YOL053C-A	YOL053C-A	UWOPS87_ctrl_hmg124h_12.JPG.dat	874.0	595.5	1.1	0.12	#NUM!		4.3E-03
YOL053W	YOL053W	UWOPS87_ctrl_hmg124h_12.JPG.dat	1157.8	110.3	1.1	0.10	#NUM!		2.3E-04
YOL054W	YOL054W	UWOPS87_ctrl_hmg124h_12.JPG.dat	989.8	75.6	0.9	0.07	#NUM!		1.2E-04
YOL055C	YOL055C	UWOPS87_ctrl_hmg124h_12.JPG.dat	1243.3	84.5	1.2	0.07	#NUM!		6.1E-05
YOL056W	YOL056W	UWOPS87_ctrl_hmg124h_12.JPG.dat	891.3	136.6	0.9	0.15	#NUM!		1.2E-03
YOL057W	YOL057W	UWOPS87_ctrl_hmg124h_12.JPG.dat	929.5	82.5	1.0	0.09	#NUM!		2.3E-04
YOL058W	YOL058W	UWOPS87_ctrl_hmg124h_12.JPG.dat	223.8	447.5	0.0	0.00	#NUM!		
YOL059W	YOL059W	UWOPS87_ctrl_hmg124h_12.JPG.dat	1303.0	100.9	1.2	0.02	#NUM!		9.5E-05
YOL060C	YOL060C	UWOPS87_ctrl_hmg124h_12.JPG.dat	1276.0	47.8	1.2	0.01	#NUM!		1.4E-05
YOL061W	YOL061W	UWOPS87_ctrl_hmg124h_12.JPG.dat	974.5	48.1	0.9	0.05	#NUM!		4.5E-05
YOL062C	YOL062C	UWOPS87_ctrl_hmg124h_12.JPG.dat	976.8	77.2	0.9	0.07	#NUM!		1.6E-04
YOL063C	YOL063C	UWOPS87_ctrl_hmg124h_12.JPG.dat	1116.3	37.9	1.0	0.01	#NUM!		2.4E-05
YOL064C	YOL064C	UWOPS87_ctrl_hmg124h_12.JPG.dat	754.3	311.9	0.8	0.01	#NUM!		5.9E-05
YOL065C	YOL065C	UWOPS87_ctrl_hmg124h_12.JPG.dat	900.3	92.5	0.8	0.09	#NUM!		3.6E-04
YOL067C	YOL067C	UWOPS87_ctrl_hmg124h_12.JPG.dat	1133.3	123.9	1.0	0.03	#NUM!		2.4E-04
YOL068C	YOL068C	UWOPS87_ctrl_hmg124h_12.JPG.dat	1230.0	55.9	1.3	0.05	#NUM!		1.5E-05
YOL070C	YOL070C	UWOPS87_ctrl_hmg124h_12.JPG.dat	852.5	51.4	0.9	0.06	#NUM!		6.8E-05
YOL071W	YOL071W	UWOPS87_ctrl_hmg124h_12.JPG.dat	983.5	98.8	0.9	0.10	#NUM!		3.5E-04
YOL075C	YOL075C	UWOPS87_ctrl_hmg124h_12.JPG.dat	1286.8	178.8	1.2	0.02	#NUM!		6.7E-05
YOL079W	YOL079W	UWOPS87_ctrl_hmg124h_12.JPG.dat	1072.0	107.2	1.0	0.10	#NUM!		2.9E-04
YOL080C	YOL080C	UWOPS87_ctrl_hmg124h_12.JPG.dat	1223.3	109.1	1.1	0.03	#NUM!		1.7E-04
YOL081W	YOL081W	UWOPS87_ctrl_hmg124h_12.JPG.dat	1233.0	82.4	1.1	0.02	#NUM!		6.2E-05
YOL082W	YOL082W	UWOPS87_ctrl_hmg124h_12.JPG.dat	986.3	165.3	0.9	0.15	#NUM!		1.3E-03
YOL083W	YOL083W	UWOPS87_ctrl_hmg124h_12.JPG.dat	1055.8	88.6	0.9	0.01	#NUM!		4.9E-05
YOL084W	YOL084W	UWOPS87_ctrl_hmg124h_12.JPG.dat	1279.3	81.4	1.2	0.00	#NUM!		2.4E-06
YOL085C	YOL085C	UWOPS87_ctrl_hmg124h_12.JPG.dat	1152.8	105.8	1.1	0.11	#NUM!		2.8E-04
YOL087C	YOL087C	UWOPS87_ctrl_hmg124h_12.JPG.dat	940.3	127.4	1.0	0.13	#NUM!		6.1E-04
YOL088C	YOL088C	UWOPS87_ctrl_hmg124h_12.JPG.dat	1028.0	225.8	0.9	0.03	#NUM!		2.6E-04
YOL089C	YOL089C	UWOPS87_ctrl_hmg124h_12.JPG.dat	1118.5	190.8	1.0	0.19	#NUM!		1.9E-03
YOL090W	YOL090W	UWOPS87_ctrl_hmg124h_12.JPG.dat	1210.8	26.5	1.0	0.03	#NUM!		9.0E-06
YOL091W	YOL091W	UWOPS87_ctrl_hmg124h_12.JPG.dat	947.8	101.9	0.8	0.08	#NUM!		2.8E-04
YOL092W	YOL092W	UWOPS87_ctrl_hmg124h_12.JPG.dat	1223.0	81.4	1.0	0.06	#NUM!		4.1E-05
YOL093W	YOL093W	UWOPS87_ctrl_hmg124h_12.JPG.dat	955.5	155.0	0.8	0.14	#NUM!		1.4E-03
YOL095C	YOL095C	UWOPS87_ctrl_hmg124h_12.JPG.dat	1188.0	134.7	1.1	0.02	#NUM!		1.3E-04
YOL098C	YOL098C	UWOPS87_ctrl_hmg124h_12.JPG.dat	1169.0	179.3	1.0	0.15	#NUM!		8.6E-04
YOL099C	YOL099C	UWOPS87_ctrl_hmg124h_12.JPG.dat	1273.0	107.9	1.1	0.10	#NUM!		2.2E-04
YOL101C	YOL101C	UWOPS87_ctrl_hmg124h_12.JPG.dat	931.5	68.2	0.9	0.08	#NUM!		2.1E-04
YOL103W	YOL103W	UWOPS87_ctrl_hmg124h_12.JPG.dat	755.8	61.8	0.9	0.07	#NUM!		1.2E-04
YOL104C	YOL104C	UWOPS87_ctrl_hmg124h_12.JPG.dat	882.0	36.8	1.0	0.01	#NUM!		3.5E-05
YOL105C	YOL105C	UWOPS87_ctrl_hmg124h_12.JPG.dat	1104.5	148.3	1.1	0.14	#NUM!		6.8E-04
YOL106W	YOL106W	UWOPS87_ctrl_hmg124h_12.JPG.dat	1059.3	112.5	1.0	0.02	#NUM!		1.1E-04
YOL107W	YOL107W	UWOPS87_ctrl_hmg124h_12.JPG.dat	952.5	42.9	0.9	0.04	#NUM!		1.9E-05
YOL108C	YOL108C	UWOPS87_ctrl_hmg124h_12.JPG.dat	1161.5	27.4	1.1	0.03	#NUM!		5.7E-06
YOL109W	YOL109W	UWOPS87_ctrl_hmg124h_12.JPG.dat	1097.0	28.8	1.0	0.03	#NUM!		8.7E-06

YOL110W	YOL110W	UWOPS87_ctrl_hmg124h_12.JPG.dat	1050.8	158.2	1.0	0.14	#NUM!	8.5E-04
YOL111C	YOL111C	UWOPS87_ctrl_hmg124h_12.JPG.dat	1226.0	139.1	1.1	0.14	#NUM!	4.7E-04
YOL112W	YOL112W	UWOPS87_ctrl_hmg124h_12.JPG.dat	1065.0	194.2	0.9	0.05	#NUM!	1.1E-03
YOL113W	YOL113W	UWOPS87_ctrl_hmg124h_12.JPG.dat	798.0	541.7	1.1	0.10	#NUM!	3.2E-03
YOL114C	YOL114C	UWOPS87_ctrl_hmg124h_12.JPG.dat	1256.0	71.4	1.3	0.02	#NUM!	6.8E-05
YOL115W	YOL115W	UWOPS87_ctrl_hmg124h_12.JPG.dat	950.0	108.5	1.0	0.04	#NUM!	4.4E-04
YOL116W	YOL116W	UWOPS87_ctrl_hmg124h_12.JPG.dat	1186.8	31.1	1.1	0.01	#NUM!	6.7E-07
YOL117W	YOL117W	UWOPS87_ctrl_hmg124h_12.JPG.dat	1223.5	57.2	1.0	0.05	#NUM!	3.2E-05
YOL118C	YOL118C	UWOPS87_ctrl_hmg124h_12.JPG.dat	1160.3	36.7	1.0	0.03	#NUM!	8.3E-06
YOL119C	YOL119C	UWOPS87_ctrl_hmg124h_12.JPG.dat	1182.3	150.5	1.0	0.13	#NUM!	5.4E-04
YOL121C	YOL121C	UWOPS87_ctrl_hmg124h_12.JPG.dat	1185.0	96.8	1.0	0.09	#NUM!	1.7E-04
YOL122C	YOL122C	UWOPS87_ctrl_hmg124h_12.JPG.dat	1307.0	65.3	1.1	0.06	#NUM!	3.1E-05
YOL124C	YOL124C	UWOPS87_ctrl_hmg124h_12.JPG.dat	1272.0	69.1	1.1	0.06	#NUM!	5.0E-05
YOL126C	YOL126C	UWOPS87_ctrl_hmg124h_12.JPG.dat	51.8	103.5	0.0	0.00	#NUM!	
YOL128C	YOL128C	UWOPS87_ctrl_hmg124h_12.JPG.dat	1333.5	81.2	1.2	0.06	#NUM!	3.9E-05
YOL129W	YOL129W	UWOPS87_ctrl_hmg124h_12.JPG.dat	817.8	137.0	0.9	0.16	#NUM!	1.6E-03
YOL131W	YOL131W	UWOPS87_ctrl_hmg124h_12.JPG.dat	1122.0	91.0	1.2	0.03	#NUM!	1.6E-04
YOL132W	YOL132W	UWOPS87_ctrl_hmg124h_12.JPG.dat	1193.3	132.0	1.1	0.03	#NUM!	2.8E-04
YOL136C	YOL136C	UWOPS87_ctrl_hmg124h_12.JPG.dat	1151.3	59.4	1.1	0.06	#NUM!	5.0E-05
YOL137W	YOL137W	UWOPS87_ctrl_hmg124h_12.JPG.dat	1134.3	82.8	1.0	0.01	#NUM!	6.7E-05
YOL138C	YOL138C	UWOPS87_ctrl_hmg124h_12.JPG.dat	1198.0	51.7	1.1	0.00	#NUM!	4.9E-06
YOL141W	YOL141W	UWOPS87_ctrl_hmg124h_12.JPG.dat	1263.5	56.5	1.1	0.03	#NUM!	6.1E-06
YOL147C	YOL147C	UWOPS87_ctrl_hmg124h_12.JPG.dat	1232.3	84.9	1.1	0.02	#NUM!	1.5E-04
YOL150C	YOL150C	UWOPS87_ctrl_hmg124h_12.JPG.dat	1120.8	17.7	1.0	0.02	#NUM!	2.4E-06
YOL151W	YOL151W	UWOPS87_ctrl_hmg124h_12.JPG.dat	938.5	80.3	0.8	0.06	#NUM!	1.2E-04
YOL152W	YOL152W	UWOPS87_ctrl_hmg124h_12.JPG.dat	1078.0	17.9	1.0	0.03	#NUM!	8.4E-06
YOL155C	YOL155C	UWOPS87_ctrl_hmg124h_12.JPG.dat	1142.3	109.0	1.1	0.11	#NUM!	2.8E-04
YOL158C	YOL158C	UWOPS87_ctrl_hmg124h_12.JPG.dat	1092.5	123.7	1.1	0.02	#NUM!	1.3E-04
YOL159C	YOL159C	UWOPS87_ctrl_hmg124h_12.JPG.dat	548.8	652.6	0.5	0.60	#NUM!	1.9E-01
YOL160W	YOL160W	UWOPS87_ctrl_hmg124h_12.JPG.dat	1185.0	44.5	1.1	0.03	#NUM!	4.7E-06
YOL162W	YOL162W	UWOPS87_ctrl_hmg124h_12.JPG.dat	1313.0	113.8	1.2	0.13	#NUM!	3.1E-04
YOL163W	YOL163W	UWOPS87_ctrl_hmg124h_12.JPG.dat	1117.0	119.8	1.0	0.11	#NUM!	3.5E-04
YOR001W	YOR001W	UWOPS87_ctrl_hmg124h_12.JPG.dat	1122.5	126.8	1.0	0.10	#NUM!	3.1E-04
YOR002W	YOR002W	UWOPS87_ctrl_hmg124h_12.JPG.dat	1078.5	196.8	1.0	0.16	#NUM!	1.2E-03
YOR003W	YOR003W	UWOPS87_ctrl_hmg124h_12.JPG.dat	1231.8	125.2	1.1	0.10	#NUM!	2.1E-04
YOR005C	YOR005C	UWOPS87_ctrl_hmg124h_12.JPG.dat	1094.0	129.8	0.9	0.01	#NUM!	1.2E-05
YOR006C	YOR006C	UWOPS87_ctrl_hmg124h_12.JPG.dat	1126.5	91.2	1.0	0.10	#NUM!	2.7E-04
YOR007C	YOR007C	UWOPS87_ctrl_hmg124h_12.JPG.dat	970.5	17.4	1.0	0.03	#NUM!	5.7E-06
YOR008C	YOR008C	UWOPS87_ctrl_hmg124h_12.JPG.dat	1097.5	102.2	1.1	0.02	#NUM!	1.5E-04
YOR008C-/	YOR008C-A	UWOPS87_ctrl_hmg124h_12.JPG.dat	304.8	609.5	0.0	0.00	#NUM!	
YOR009W	YOR009W	UWOPS87_ctrl_hmg124h_12.JPG.dat	1077.8	151.0	0.9	0.04	#NUM!	7.1E-04
YOR010C	YOR010C	UWOPS87_ctrl_hmg124h_12.JPG.dat	1043.5	179.2	1.0	0.17	#NUM!	1.5E-03
YOR011W	YOR011W	UWOPS87_ctrl_hmg124h_12.JPG.dat	913.0	75.9	0.9	0.07	#NUM!	1.6E-04
YOR012W	YOR012W	UWOPS87_ctrl_hmg124h_12.JPG.dat	1316.0	79.2	1.2	0.07	#NUM!	5.8E-05
YOR013W	YOR013W	UWOPS87_ctrl_hmg124h_12.JPG.dat	1018.5	33.7	0.9	0.03	#NUM!	1.0E-05
YOR014W	YOR014W	UWOPS87_ctrl_hmg124h_12.JPG.dat	1234.3	102.3	1.1	0.09	#NUM!	1.6E-04
YOR015W	YOR015W	UWOPS87_ctrl_hmg124h_12.JPG.dat	830.3	563.8	1.0	0.11	#NUM!	4.2E-03
YOR016C	YOR016C	UWOPS87_ctrl_hmg124h_12.JPG.dat	1031.0	191.5	0.9	0.06	#NUM!	1.8E-03
YOR017W	YOR017W	UWOPS87_ctrl_hmg124h_12.JPG.dat	859.8	58.8	0.9	0.06	#NUM!	6.3E-05
YOR018W	YOR018W	UWOPS87_ctrl_hmg124h_12.JPG.dat	1117.3	119.3	1.1	0.03	#NUM!	3.1E-04
YOR019W	YOR019W	UWOPS87_ctrl_hmg124h_12.JPG.dat	1181.8	128.5	1.1	0.11	#NUM!	3.2E-04
YOR021C	YOR021C	UWOPS87_ctrl_hmg124h_12.JPG.dat	534.0	619.6	0.5	0.57	#NUM!	1.8E-01
YOR022C	YOR022C	UWOPS87_ctrl_hmg124h_12.JPG.dat	1048.3	55.9	1.0	0.05	#NUM!	2.9E-05
YOR023C	YOR023C	UWOPS87_ctrl_hmg124h_12.JPG.dat	1016.3	53.0	0.9	0.05	#NUM!	3.6E-05
YOR024W	YOR024W	UWOPS87_ctrl_hmg124h_12.JPG.dat	1199.8	71.7	1.1	0.07	#NUM!	6.3E-05
YOR025W	YOR025W	UWOPS87_ctrl_hmg124h_12.JPG.dat	1129.5	140.4	1.1	0.03	#NUM!	2.2E-04
YOR026W	YOR026W	UWOPS87_ctrl_hmg124h_12.JPG.dat	401.0	463.6	0.4	0.41	#NUM!	1.8E-01
YOR027W	YOR027W	UWOPS87_ctrl_hmg124h_12.JPG.dat	1081.3	125.3	0.9	0.10	#NUM!	3.6E-04
YOR028C	YOR028C	UWOPS87_ctrl_hmg124h_12.JPG.dat	1056.8	114.5	1.0	0.11	#NUM!	4.1E-04
YOR029W	YOR029W	UWOPS87_ctrl_hmg124h_12.JPG.dat	676.5	147.0	0.8	0.03	#NUM!	5.3E-04
YOR030W	YOR030W	UWOPS87_ctrl_hmg124h_12.JPG.dat	876.8	29.1	0.9	0.05	#NUM!	3.9E-05
YOR031W	YOR031W	UWOPS87_ctrl_hmg124h_12.JPG.dat	1112.8	91.7	1.1	0.01	#NUM!	3.3E-05
YOR032C	YOR032C	UWOPS87_ctrl_hmg124h_12.JPG.dat	1180.5	51.5	1.1	0.04	#NUM!	1.8E-05
YOR033C	YOR033C	UWOPS87_ctrl_hmg124h_12.JPG.dat	1165.3	84.8	1.0	0.03	#NUM!	2.6E-04
YOR034C	YOR034C	UWOPS87_ctrl_hmg124h_12.JPG.dat	1234.0	35.1	1.2	0.01	#NUM!	3.7E-05
YOR035C	YOR035C	UWOPS87_ctrl_hmg124h_12.JPG.dat	644.0	138.6	0.6	0.13	#NUM!	2.6E-03
YOR037W	YOR037W	UWOPS87_ctrl_hmg124h_12.JPG.dat	1259.5	85.7	1.2	0.07	#NUM!	7.0E-05
YOR038C	YOR038C	UWOPS87_ctrl_hmg124h_12.JPG.dat	1212.5	35.6	1.1	0.01	#NUM!	1.6E-05
YOR039W	YOR039W	UWOPS87_ctrl_hmg124h_12.JPG.dat	1280.0	45.6	1.1	0.01	#NUM!	5.1E-05
YOR040W	YOR040W	UWOPS87_ctrl_hmg124h_12.JPG.dat	922.5	27.1	0.9	0.03	#NUM!	1.3E-05

YOR041C	YOR041C	UWOPS87_ctrl_hmg124h_12.JPG.dat	851.0	153.2	1.0	0.17	#NUM!	1.5E-03
YOR042W	YOR042W	UWOPS87_ctrl_hmg124h_12.JPG.dat	938.8	96.5	1.0	0.12	#NUM!	4.5E-04
YOR043W	YOR043W	UWOPS87_ctrl_hmg124h_12.JPG.dat	721.0	80.6	0.7	0.08	#NUM!	4.6E-04
YOR044W	YOR044W	UWOPS87_ctrl_hmg124h_12.JPG.dat	1083.5	70.5	1.0	0.06	#NUM!	5.5E-05
YOR045W	YOR045W	UWOPS87_ctrl_hmg124h_12.JPG.dat	1095.3	38.5	1.0	0.03	#NUM!	5.4E-06
YOR047C	YOR047C	UWOPS87_ctrl_hmg124h_12.JPG.dat	1076.0	29.3	1.0	0.01	#NUM!	2.4E-05
YOR049C	YOR049C	UWOPS87_ctrl_hmg124h_12.JPG.dat	1251.8	31.1	1.1	0.03	#NUM!	7.9E-06
YOR050C	YOR050C	UWOPS87_ctrl_hmg124h_12.JPG.dat	1024.8	63.9	1.0	0.06	#NUM!	6.1E-05
YOR051C	YOR051C	UWOPS87_ctrl_hmg124h_12.JPG.dat	1237.5	61.4	1.1	0.06	#NUM!	5.2E-05
YOR052C	YOR052C	UWOPS87_ctrl_hmg124h_12.JPG.dat	972.8	141.8	0.9	0.12	#NUM!	6.9E-04
YOR053W	YOR053W	UWOPS87_ctrl_hmg124h_12.JPG.dat	1054.8	47.7	1.1	0.06	#NUM!	5.8E-05
YOR055W	YOR055W	UWOPS87_ctrl_hmg124h_12.JPG.dat	870.5	88.4	1.0	0.10	#NUM!	3.1E-04
YOR058C	YOR058C	UWOPS87_ctrl_hmg124h_12.JPG.dat	762.3	173.4	0.7	0.04	#NUM!	1.1E-03
YOR059C	YOR059C	UWOPS87_ctrl_hmg124h_12.JPG.dat	1105.5	49.9	1.0	0.05	#NUM!	3.1E-05
YOR061W	YOR061W	UWOPS87_ctrl_hmg124h_12.JPG.dat	997.8	55.6	0.9	0.04	#NUM!	3.4E-05
YOR062C	YOR062C	UWOPS87_ctrl_hmg124h_12.JPG.dat	927.8	71.0	0.8	0.05	#NUM!	6.8E-05
YOR064C	YOR064C	UWOPS87_ctrl_hmg124h_12.JPG.dat	1104.8	36.8	1.0	0.03	#NUM!	7.9E-06
YOR065W	YOR065W	UWOPS87_ctrl_hmg124h_12.JPG.dat	1254.8	78.6	1.1	0.07	#NUM!	7.2E-05
YOR066W	YOR066W	UWOPS87_ctrl_hmg124h_12.JPG.dat	1097.8	123.9	1.0	0.11	#NUM!	3.3E-04
YOR067C	YOR067C	UWOPS87_ctrl_hmg124h_12.JPG.dat	1114.8	162.3	1.0	0.15	#NUM!	8.1E-04
YOR068C	YOR068C	UWOPS87_ctrl_hmg124h_12.JPG.dat	984.5	663.3	1.2	0.10	#NUM!	2.4E-03
YOR069W	YOR069W	UWOPS87_ctrl_hmg124h_12.JPG.dat	1105.0	17.3	1.1	0.00	#NUM!	4.6E-06
YOR070C	YOR070C	UWOPS87_ctrl_hmg124h_12.JPG.dat	989.0	94.6	1.1	0.02	#NUM!	1.2E-04
YOR071C	YOR071C	UWOPS87_ctrl_hmg124h_12.JPG.dat	875.0	126.5	1.0	0.04	#NUM!	5.9E-04
YOR072W	YOR072W	UWOPS87_ctrl_hmg124h_12.JPG.dat	956.3	115.6	0.8	0.04	#NUM!	8.3E-04
YOR076C	YOR076C	UWOPS87_ctrl_hmg124h_12.JPG.dat	1068.0	45.8	1.0	0.04	#NUM!	2.2E-05
YOR078W	YOR078W	UWOPS87_ctrl_hmg124h_12.JPG.dat	1032.5	49.0	1.0	0.04	#NUM!	2.2E-05
YOR079C	YOR079C	UWOPS87_ctrl_hmg124h_12.JPG.dat	1232.0	75.9	1.1	0.03	#NUM!	2.0E-04
YOR080W	YOR080W	UWOPS87_ctrl_hmg124h_12.JPG.dat	1288.3	94.3	1.2	0.09	#NUM!	1.3E-04
YOR081C	YOR081C	UWOPS87_ctrl_hmg124h_12.JPG.dat	918.5	85.7	0.9	0.08	#NUM!	2.0E-04
YOR082C	YOR082C	UWOPS87_ctrl_hmg124h_12.JPG.dat	978.3	60.9	0.9	0.06	#NUM!	7.7E-05
YOR083W	YOR083W	UWOPS87_ctrl_hmg124h_12.JPG.dat	1162.3	70.0	1.1	0.07	#NUM!	7.8E-05
YOR084W	YOR084W	UWOPS87_ctrl_hmg124h_12.JPG.dat	987.0	28.4	1.0	0.03	#NUM!	5.3E-06
YOR085W	YOR085W	UWOPS87_ctrl_hmg124h_12.JPG.dat	1102.5	58.2	1.0	0.02	#NUM!	1.1E-04
YOR086C	YOR086C	UWOPS87_ctrl_hmg124h_12.JPG.dat	909.5	69.7	0.9	0.07	#NUM!	1.3E-04
YOR087W	YOR087W	UWOPS87_ctrl_hmg124h_12.JPG.dat	691.3	470.7	0.8	0.09	#NUM!	3.7E-03
YOR088W	YOR088W	UWOPS87_ctrl_hmg124h_12.JPG.dat	652.0	505.7	0.6	0.45	#NUM!	8.1E-02
YOR089C	YOR089C	UWOPS87_ctrl_hmg124h_12.JPG.dat	1175.0	90.7	1.1	0.07	#NUM!	8.6E-05
YOR090C	YOR090C	UWOPS87_ctrl_hmg124h_12.JPG.dat	1267.3	139.2	1.1	0.11	#NUM!	2.5E-04
YOR091W	YOR091W	UWOPS87_ctrl_hmg124h_12.JPG.dat	1078.8	120.4	1.0	0.10	#NUM!	2.6E-04
YOR092W	YOR092W	UWOPS87_ctrl_hmg124h_12.JPG.dat	979.3	60.5	0.9	0.06	#NUM!	8.1E-05
YOR093C	YOR093C	UWOPS87_ctrl_hmg124h_12.JPG.dat	1004.5	35.4	0.9	0.04	#NUM!	2.2E-05
YOR094W	YOR094W	UWOPS87_ctrl_hmg124h_12.JPG.dat	1039.5	72.5	0.9	0.05	#NUM!	3.9E-05
YOR097C	YOR097C	UWOPS87_ctrl_hmg124h_12.JPG.dat	1082.8	18.7	1.0	0.01	#NUM!	2.3E-07
YOR099W	YOR099W	UWOPS87_ctrl_hmg124h_12.JPG.dat	942.0	23.6	1.0	0.01	#NUM!	8.7E-07
YOR100C	YOR100C	UWOPS87_ctrl_hmg124h_12.JPG.dat	1031.8	149.1	1.0	0.14	#NUM!	7.2E-04
YOR101W	YOR101W	UWOPS87_ctrl_hmg124h_12.JPG.dat	1239.5	159.5	1.2	0.14	#NUM!	4.7E-04
YOR104W	YOR104W	UWOPS87_ctrl_hmg124h_12.JPG.dat	1383.5	10.1	1.3	0.01	#NUM!	5.7E-08
YOR105W	YOR105W	UWOPS87_ctrl_hmg124h_12.JPG.dat	1005.5	175.9	0.9	0.16	#NUM!	1.4E-03
YOR106W	YOR106W	UWOPS87_ctrl_hmg124h_12.JPG.dat	1080.5	52.0	1.0	0.05	#NUM!	3.0E-05
YOR107W	YOR107W	UWOPS87_ctrl_hmg124h_12.JPG.dat	991.0	81.0	0.9	0.07	#NUM!	1.5E-04
YOR108W	YOR108W	UWOPS87_ctrl_hmg124h_12.JPG.dat	1093.5	138.7	1.0	0.12	#NUM!	4.9E-04
YOR109W	YOR109W	UWOPS87_ctrl_hmg124h_12.JPG.dat	1260.3	61.4	1.1	0.05	#NUM!	2.9E-05
YOR111W	YOR111W	UWOPS87_ctrl_hmg124h_12.JPG.dat	1068.8	202.5	1.0	0.18	#NUM!	1.9E-03
YOR112W	YOR112W	UWOPS87_ctrl_hmg124h_12.JPG.dat	1023.5	144.7	1.0	0.14	#NUM!	8.2E-04
YOR113W	YOR113W	UWOPS87_ctrl_hmg124h_12.JPG.dat	1083.8	30.8	1.1	0.04	#NUM!	1.3E-05
YOR114W	YOR114W	UWOPS87_ctrl_hmg124h_12.JPG.dat	965.0	56.9	1.0	0.01	#NUM!	6.3E-05
YOR115C	YOR115C	UWOPS87_ctrl_hmg124h_12.JPG.dat	1037.5	16.4	1.0	0.03	#NUM!	5.4E-06
YOR118W	YOR118W	UWOPS87_ctrl_hmg124h_12.JPG.dat	1275.3	60.9	1.1	0.02	#NUM!	7.3E-05
YOR120W	YOR120W	UWOPS87_ctrl_hmg124h_12.JPG.dat	973.3	33.5	0.9	0.03	#NUM!	8.9E-06
YOR121C	YOR121C	UWOPS87_ctrl_hmg124h_12.JPG.dat	884.0	590.1	1.1	0.03	#NUM!	2.7E-04
YOR123C	YOR123C	UWOPS87_ctrl_hmg124h_12.JPG.dat	1111.8	102.0	1.0	0.10	#NUM!	2.5E-04
YOR124C	YOR124C	UWOPS87_ctrl_hmg124h_12.JPG.dat	1073.5	119.7	1.0	0.11	#NUM!	4.4E-04
YOR126C	YOR126C	UWOPS87_ctrl_hmg124h_12.JPG.dat	1044.3	76.5	0.9	0.06	#NUM!	9.2E-05
YOR127W	YOR127W	UWOPS87_ctrl_hmg124h_12.JPG.dat	1257.3	198.2	1.1	0.18	#NUM!	1.2E-03
YOR129C	YOR129C	UWOPS87_ctrl_hmg124h_12.JPG.dat	1098.0	149.7	1.0	0.13	#NUM!	5.6E-04
YOR131C	YOR131C	UWOPS87_ctrl_hmg124h_12.JPG.dat	800.0	99.5	0.9	0.12	#NUM!	7.2E-04
YOR132W	YOR132W	UWOPS87_ctrl_hmg124h_12.JPG.dat	918.8	34.8	1.0	0.03	#NUM!	6.4E-06
YOR133W	YOR133W	UWOPS87_ctrl_hmg124h_12.JPG.dat	1139.0	36.3	1.1	0.01	#NUM!	6.1E-05
YOR134W	YOR134W	UWOPS87_ctrl_hmg124h_12.JPG.dat	879.0	39.2	0.8	0.04	#NUM!	2.9E-05

YOR135C	YOR135C	UWOPS87_ctrl_hmg124h_12.JPG.dat	1089.8	73.9	1.0	0.07	#NUM!	7.5E-05
YOR136W	YOR136W	UWOPS87_ctrl_hmg124h_12.JPG.dat	1161.8	46.9	1.1	0.04	#NUM!	2.1E-05
YOR137C	YOR137C	UWOPS87_ctrl_hmg124h_12.JPG.dat	1139.3	38.8	1.0	0.04	#NUM!	1.7E-05
YOR138C	YOR138C	UWOPS87_ctrl_hmg124h_12.JPG.dat	1109.0	37.8	1.0	0.04	#NUM!	1.3E-05
YOR139C	YOR139C	UWOPS87_ctrl_hmg124h_12.JPG.dat	1000.5	197.1	0.9	0.19	#NUM!	2.3E-03
YOR140W	YOR140W	UWOPS87_ctrl_hmg124h_12.JPG.dat	1100.0	76.6	1.0	0.02	#NUM!	1.1E-04
YOR141C	YOR141C	UWOPS87_ctrl_hmg124h_12.JPG.dat	956.0	73.7	0.9	0.09	#NUM!	2.2E-04
YOR142W	YOR142W	UWOPS87_ctrl_hmg124h_12.JPG.dat	732.3	131.4	0.9	0.06	#NUM!	1.2E-03
YOR144C	YOR144C	UWOPS87_ctrl_hmg124h_12.JPG.dat	1031.3	12.0	1.1	0.03	#NUM!	3.3E-06
YOR152C	YOR152C	UWOPS87_ctrl_hmg124h_12.JPG.dat	1064.0	218.1	1.1	0.01	#NUM!	3.9E-05
YOR153W	YOR153W	UWOPS87_ctrl_hmg124h_12.JPG.dat	983.3	74.1	0.9	0.06	#NUM!	9.6E-05
YOR154W	YOR154W	UWOPS87_ctrl_hmg124h_12.JPG.dat	1055.3	121.8	1.0	0.11	#NUM!	3.7E-04
YOR156C	YOR156C	UWOPS87_ctrl_hmg124h_12.JPG.dat	1168.0	24.4	1.1	0.03	#NUM!	4.4E-06
YOR161C	YOR161C	UWOPS87_ctrl_hmg124h_12.JPG.dat	1170.0	56.2	1.1	0.05	#NUM!	2.1E-05
YOR162C	YOR162C	UWOPS87_ctrl_hmg124h_12.JPG.dat	1066.0	49.1	1.0	0.05	#NUM!	3.7E-05
YOR163W	YOR163W	UWOPS87_ctrl_hmg124h_12.JPG.dat	967.0	47.1	0.9	0.01	#NUM!	3.6E-05
YOR164C	YOR164C	UWOPS87_ctrl_hmg124h_12.JPG.dat	1198.8	109.9	1.1	0.01	#NUM!	1.9E-05
YOR165W	YOR165W	UWOPS87_ctrl_hmg124h_12.JPG.dat	984.8	30.0	1.0	0.02	#NUM!	2.5E-06
YOR166C	YOR166C	UWOPS87_ctrl_hmg124h_12.JPG.dat	991.0	88.2	1.1	0.09	#NUM!	1.5E-04
YOR167C	YOR167C	UWOPS87_ctrl_hmg124h_12.JPG.dat	705.3	48.2	0.7	0.06	#NUM!	1.5E-04
YOR170W	YOR170W	UWOPS87_ctrl_hmg124h_12.JPG.dat	1191.8	123.3	1.1	0.12	#NUM!	3.4E-04
YOR171C	YOR171C	UWOPS87_ctrl_hmg124h_12.JPG.dat	1261.3	16.8	1.1	0.01	#NUM!	1.4E-07
YOR172W	YOR172W	UWOPS87_ctrl_hmg124h_12.JPG.dat	1136.5	60.0	1.0	0.05	#NUM!	2.3E-05
YOR173W	YOR173W	UWOPS87_ctrl_hmg124h_12.JPG.dat	1188.5	85.7	1.0	0.09	#NUM!	1.5E-04
YOR175C	YOR175C	UWOPS87_ctrl_hmg124h_12.JPG.dat	1225.5	34.8	1.1	0.02	#NUM!	1.6E-06
YOR177C	YOR177C	UWOPS87_ctrl_hmg124h_12.JPG.dat	1193.0	103.3	1.1	0.09	#NUM!	1.9E-04
YOR178C	YOR178C	UWOPS87_ctrl_hmg124h_12.JPG.dat	1260.0	48.8	1.1	0.04	#NUM!	1.6E-05
YOR182C	YOR182C	UWOPS87_ctrl_hmg124h_12.JPG.dat	1104.3	108.1	1.0	0.04	#NUM!	5.6E-04
YOR183W	YOR183W	UWOPS87_ctrl_hmg124h_12.JPG.dat	846.8	30.6	0.8	0.02	#NUM!	3.4E-06
YOR184W	YOR184W	UWOPS87_ctrl_hmg124h_12.JPG.dat	961.3	58.1	1.1	0.02	#NUM!	1.4E-04
YOR185C	YOR185C	UWOPS87_ctrl_hmg124h_12.JPG.dat	952.0	172.3	1.0	0.19	#NUM!	1.8E-03
YOR186W	YOR186W	UWOPS87_ctrl_hmg124h_12.JPG.dat	1014.8	165.7	1.0	0.15	#NUM!	1.0E-03
YOR188W	YOR188W	UWOPS87_ctrl_hmg124h_12.JPG.dat	1022.5	238.2	0.9	0.22	#NUM!	3.4E-03
YOR189W	YOR189W	UWOPS87_ctrl_hmg124h_12.JPG.dat	1037.8	76.9	1.0	0.07	#NUM!	1.1E-04
YOR190W	YOR190W	UWOPS87_ctrl_hmg124h_12.JPG.dat	1134.0	163.7	1.0	0.15	#NUM!	8.0E-04
YOR191W	YOR191W	UWOPS87_ctrl_hmg124h_12.JPG.dat	1076.3	45.0	1.0	0.01	#NUM!	4.6E-05
YOR192C	YOR192C	UWOPS87_ctrl_hmg124h_12.JPG.dat	999.8	79.1	0.9	0.08	#NUM!	1.6E-04
YOR193W	YOR193W	UWOPS87_ctrl_hmg124h_12.JPG.dat	1050.8	64.0	1.0	0.05	#NUM!	4.5E-05
YOR195W	YOR195W	UWOPS87_ctrl_hmg124h_12.JPG.dat	1174.0	55.2	1.1	0.06	#NUM!	5.4E-05
YOR196C	YOR196C	UWOPS87_ctrl_hmg124h_12.JPG.dat	1019.0	64.4	1.0	0.08	#NUM!	1.2E-04
YOR202W	YOR202W	UWOPS87_ctrl_hmg124h_12.JPG.dat	1166.9	216.2	1.0	0.13	#NUM!	3.1E-246
1	1	UWOPS87_ctrl_hmg124h_13.JPG.dat	1619.5	223.6	1.0	0.09	#NUM!	1.8E-04
2	2	UWOPS87_ctrl_hmg124h_13.JPG.dat	1538.0	124.3	1.1	0.03	#NUM!	3.6E-06
3	3	UWOPS87_ctrl_hmg124h_13.JPG.dat	1295.8	157.8	0.9	0.06	#NUM!	8.3E-05
4	4	UWOPS87_ctrl_hmg124h_13.JPG.dat	1351.3	235.7	1.0	0.12	#NUM!	4.8E-04
YOR197W	YOR197W	UWOPS87_ctrl_hmg124h_13.JPG.dat	1133.3	59.2	1.0	0.05	#NUM!	2.7E-05
YOR202W	YOR202W	UWOPS87_ctrl_hmg124h_13.JPG.dat	1374.3	201.9	1.0	0.08	#NUM!	5.0349147
YOR208W	YOR208W	UWOPS87_ctrl_hmg124h_13.JPG.dat	1169.0	30.0	1.0	0.02	#NUM!	2.0E-06
YOR209C	YOR209C	UWOPS87_ctrl_hmg124h_13.JPG.dat	1210.0	156.3	1.0	0.11	#NUM!	3.5E-04
YOR212W	YOR212W	UWOPS87_ctrl_hmg124h_13.JPG.dat	766.0	474.5	0.8	0.09	#NUM!	3.9E-03
YOR213C	YOR213C	UWOPS87_ctrl_hmg124h_13.JPG.dat	1268.3	71.1	1.0	0.01	#NUM!	2.3E-05
YOR214C	YOR214C	UWOPS87_ctrl_hmg124h_13.JPG.dat	1374.0	76.9	1.0	0.02	#NUM!	1.1E-04
YOR215C	YOR215C	UWOPS87_ctrl_hmg124h_13.JPG.dat	1324.5	98.2	1.0	0.10	#NUM!	2.3E-04
YOR216C	YOR216C	UWOPS87_ctrl_hmg124h_13.JPG.dat	1425.0	40.4	1.1	0.02	#NUM!	1.2E-06
YOR219C	YOR219C	UWOPS87_ctrl_hmg124h_13.JPG.dat	1261.5	60.6	1.0	0.04	#NUM!	2.3E-05
YOR221C	YOR221C	UWOPS87_ctrl_hmg124h_13.JPG.dat	1171.3	40.8	1.0	0.04	#NUM!	1.5E-05
YOR222W	YOR222W	UWOPS87_ctrl_hmg124h_13.JPG.dat	1155.8	51.3	1.0	0.04	#NUM!	2.2E-05
YOR223W	YOR223W	UWOPS87_ctrl_hmg124h_13.JPG.dat	1171.8	86.0	1.0	0.08	#NUM!	1.3E-04
YOR225W	YOR225W	UWOPS87_ctrl_hmg124h_13.JPG.dat	1264.3	55.6	1.0	0.04	#NUM!	2.1E-05
YOR226C	YOR226C	UWOPS87_ctrl_hmg124h_13.JPG.dat	1298.0	92.7	1.0	0.07	#NUM!	9.1E-05
YOR227W	YOR227W	UWOPS87_ctrl_hmg124h_13.JPG.dat	1328.0	51.1	1.0	0.04	#NUM!	1.5E-05
YOR228C	YOR228C	UWOPS87_ctrl_hmg124h_13.JPG.dat	1299.3	29.2	1.0	0.01	#NUM!	1.9E-05
YOR229W	YOR229W	UWOPS87_ctrl_hmg124h_13.JPG.dat	1284.0	152.6	0.9	0.03	#NUM!	3.3E-04
YOR230W	YOR230W	UWOPS87_ctrl_hmg124h_13.JPG.dat	1322.5	49.7	1.0	0.04	#NUM!	1.9E-05
YOR231W	YOR231W	UWOPS87_ctrl_hmg124h_13.JPG.dat	1449.8	26.8	1.1	0.02	#NUM!	3.1E-06
YOR233W	YOR233W	UWOPS87_ctrl_hmg124h_13.JPG.dat	1198.0	76.2	0.9	0.07	#NUM!	9.3E-05
YOR234C	YOR234C	UWOPS87_ctrl_hmg124h_13.JPG.dat	1038.0	99.2	0.9	0.08	#NUM!	2.3E-04
YOR235W	YOR235W	UWOPS87_ctrl_hmg124h_13.JPG.dat	1007.0	32.3	0.9	0.01	#NUM!	9.7E-08
YOR237W	YOR237W	UWOPS87_ctrl_hmg124h_13.JPG.dat	1189.5	185.4	1.1	0.04	#NUM!	4.6E-04
YOR238W	YOR238W	UWOPS87_ctrl_hmg124h_13.JPG.dat	1242.0	134.5	1.0	0.11	#NUM!	3.5E-04

YOR239W	YOR239W	UWOPS87_ctrl_hmg124h_13.JPG.dat	1224.5	104.4	1.0	0.01	#NUM!	3.4E-05
YOR242C	YOR242C	UWOPS87_ctrl_hmg124h_13.JPG.dat	1235.8	177.2	1.0	0.14	#NUM!	7.6E-04
YOR243C	YOR243C	UWOPS87_ctrl_hmg124h_13.JPG.dat	1180.3	69.0	0.9	0.04	#NUM!	2.0E-05
YOR245C	YOR245C	UWOPS87_ctrl_hmg124h_13.JPG.dat	1504.0	62.7	1.2	0.04	#NUM!	8.8E-06
YOR246C	YOR246C	UWOPS87_ctrl_hmg124h_13.JPG.dat	1293.0	61.7	1.0	0.04	#NUM!	2.0E-05
YOR247W	YOR247W	UWOPS87_ctrl_hmg124h_13.JPG.dat	1235.0	45.4	1.0	0.03	#NUM!	7.4E-06
YOR251C	YOR251C	UWOPS87_ctrl_hmg124h_13.JPG.dat	1269.3	36.3	1.0	0.03	#NUM!	8.2E-06
YOR252W	YOR252W	UWOPS87_ctrl_hmg124h_13.JPG.dat	1235.3	73.5	1.1	0.06	#NUM!	4.1E-05
YOR253W	YOR253W	UWOPS87_ctrl_hmg124h_13.JPG.dat	1138.0	59.5	1.0	0.06	#NUM!	5.8E-05
YOR255W	YOR255W	UWOPS87_ctrl_hmg124h_13.JPG.dat	1221.0	56.3	1.0	0.05	#NUM!	3.1E-05
YOR263C	YOR263C	UWOPS87_ctrl_hmg124h_13.JPG.dat	1224.8	83.8	1.0	0.07	#NUM!	9.0E-05
YOR264W	YOR264W	UWOPS87_ctrl_hmg124h_13.JPG.dat	1283.8	115.9	1.0	0.09	#NUM!	2.0E-04
YOR265W	YOR265W	UWOPS87_ctrl_hmg124h_13.JPG.dat	1304.0	82.2	1.0	0.07	#NUM!	8.2E-05
YOR266W	YOR266W	UWOPS87_ctrl_hmg124h_13.JPG.dat	1321.8	100.3	1.0	0.08	#NUM!	1.3E-04
YOR267C	YOR267C	UWOPS87_ctrl_hmg124h_13.JPG.dat	1419.0	125.0	1.1	0.09	#NUM!	1.6E-04
YOR268C	YOR268C	UWOPS87_ctrl_hmg124h_13.JPG.dat	1153.3	39.3	0.9	0.03	#NUM!	8.1E-06
YOR269W	YOR269W	UWOPS87_ctrl_hmg124h_13.JPG.dat	1101.0	736.3	1.1	0.06	#NUM!	9.0E-04
YOR270C	YOR270C	UWOPS87_ctrl_hmg124h_13.JPG.dat	771.0	542.7	0.6	0.44	#NUM!	6.6E-02
YOR271C	YOR271C	UWOPS87_ctrl_hmg124h_13.JPG.dat	1171.5	95.0	1.0	0.08	#NUM!	1.6E-04
YOR273C	YOR273C	UWOPS87_ctrl_hmg124h_13.JPG.dat	1152.5	114.0	1.0	0.02	#NUM!	8.5E-05
YOR274W	YOR274W	UWOPS87_ctrl_hmg124h_13.JPG.dat	1223.5	87.3	1.0	0.07	#NUM!	7.4E-05
YOR275C	YOR275C	UWOPS87_ctrl_hmg124h_13.JPG.dat	1116.3	62.7	0.9	0.05	#NUM!	4.5E-05
YOR276W	YOR276W	UWOPS87_ctrl_hmg124h_13.JPG.dat	1132.5	233.6	0.9	0.18	#NUM!	2.0E-03
YOR277C	YOR277C	UWOPS87_ctrl_hmg124h_13.JPG.dat	1185.5	43.3	1.0	0.05	#NUM!	3.3E-05
YOR279C	YOR279C	UWOPS87_ctrl_hmg124h_13.JPG.dat	1268.0	32.3	1.0	0.02	#NUM!	9.9E-07
YOR280C	YOR280C	UWOPS87_ctrl_hmg124h_13.JPG.dat	1177.3	83.7	1.0	0.05	#NUM!	4.4E-05
YOR283W	YOR283W	UWOPS87_ctrl_hmg124h_13.JPG.dat	1217.8	81.7	1.0	0.05	#NUM!	3.8E-05
YOR284W	YOR284W	UWOPS87_ctrl_hmg124h_13.JPG.dat	1251.3	29.8	1.0	0.04	#NUM!	1.7E-05
YOR285W	YOR285W	UWOPS87_ctrl_hmg124h_13.JPG.dat	1203.3	81.7	1.0	0.02	#NUM!	2.1E-04
YOR286W	YOR286W	UWOPS87_ctrl_hmg124h_13.JPG.dat	1099.5	61.2	0.9	0.01	#NUM!	1.3E-05
YOR288C	YOR288C	UWOPS87_ctrl_hmg124h_13.JPG.dat	1236.8	57.7	1.0	0.06	#NUM!	6.5E-05
YOR289W	YOR289W	UWOPS87_ctrl_hmg124h_13.JPG.dat	1200.8	95.5	1.0	0.07	#NUM!	8.4E-05
YOR291W	YOR291W	UWOPS87_ctrl_hmg124h_13.JPG.dat	1121.3	68.6	0.9	0.07	#NUM!	1.2E-04
YOR292C	YOR292C	UWOPS87_ctrl_hmg124h_13.JPG.dat	1269.5	119.0	1.0	0.02	#NUM!	2.2E-04
YOR293W	YOR293W	UWOPS87_ctrl_hmg124h_13.JPG.dat	1198.8	89.8	0.9	0.03	#NUM!	3.2E-04
YOR296W	YOR296W	UWOPS87_ctrl_hmg124h_13.JPG.dat	961.3	647.4	1.1	0.08	#NUM!	1.7E-03
YOR297C	YOR297C	UWOPS87_ctrl_hmg124h_13.JPG.dat	1107.3	124.2	0.9	0.09	#NUM!	2.7E-04
YOR298C-/	YOR298C-A	UWOPS87_ctrl_hmg124h_13.JPG.dat	1161.0	32.4	1.0	0.00	#NUM!	6.0E-06
YOR298W	YOR298W	UWOPS87_ctrl_hmg124h_13.JPG.dat	1294.3	62.3	1.1	0.04	#NUM!	9.2E-06
YOR299W	YOR299W	UWOPS87_ctrl_hmg124h_13.JPG.dat	1102.3	94.1	0.9	0.06	#NUM!	8.5E-05
YOR300W	YOR300W	UWOPS87_ctrl_hmg124h_13.JPG.dat	970.5	20.0	0.9	0.03	#NUM!	1.2E-05
YOR301W	YOR301W	UWOPS87_ctrl_hmg124h_13.JPG.dat	1238.3	76.5	1.0	0.05	#NUM!	2.9E-05
YOR302W	YOR302W	UWOPS87_ctrl_hmg124h_13.JPG.dat	1227.8	111.1	1.0	0.08	#NUM!	1.4E-04
YOR303W	YOR303W	UWOPS87_ctrl_hmg124h_13.JPG.dat	1188.8	40.8	1.0	0.04	#NUM!	2.0E-05
YOR304C-/	YOR304C-A	UWOPS87_ctrl_hmg124h_13.JPG.dat	1158.0	118.1	1.0	0.09	#NUM!	2.4E-04
YOR304W	YOR304W	UWOPS87_ctrl_hmg124h_13.JPG.dat	1179.5	9.5	1.0	0.02	#NUM!	2.6E-06
YOR306C	YOR306C	UWOPS87_ctrl_hmg124h_13.JPG.dat	1152.0	45.0	1.0	0.04	#NUM!	1.6E-05
YOR307C	YOR307C	UWOPS87_ctrl_hmg124h_13.JPG.dat	1381.8	183.8	1.1	0.02	#NUM!	9.6E-05
YOR308C	YOR308C	UWOPS87_ctrl_hmg124h_13.JPG.dat	1057.5	63.0	1.0	0.02	#NUM!	9.8E-05
YOR309C	YOR309C	UWOPS87_ctrl_hmg124h_13.JPG.dat	1089.5	19.3	1.0	0.01	#NUM!	6.0E-07
YOR311C	YOR311C	UWOPS87_ctrl_hmg124h_13.JPG.dat	1065.3	132.3	1.0	0.12	#NUM!	5.5E-04
YOR312C	YOR312C	UWOPS87_ctrl_hmg124h_13.JPG.dat	996.0	272.2	0.8	0.03	#NUM!	5.0E-04
YOR313C	YOR313C	UWOPS87_ctrl_hmg124h_13.JPG.dat	1095.8	28.5	1.0	0.03	#NUM!	1.3E-05
YOR314W	YOR314W	UWOPS87_ctrl_hmg124h_13.JPG.dat	1186.8	46.4	1.0	0.03	#NUM!	9.4E-06
YOR315W	YOR315W	UWOPS87_ctrl_hmg124h_13.JPG.dat	1262.5	23.4	1.1	0.03	#NUM!	4.7E-06
YOR316C	YOR316C	UWOPS87_ctrl_hmg124h_13.JPG.dat	1174.0	60.9	1.0	0.03	#NUM!	8.9E-06
YOR317W	YOR317W	UWOPS87_ctrl_hmg124h_13.JPG.dat	1316.8	71.1	1.0	0.04	#NUM!	1.5E-05
YOR318C	YOR318C	UWOPS87_ctrl_hmg124h_13.JPG.dat	1267.3	75.4	1.0	0.06	#NUM!	6.5E-05
YOR320C	YOR320C	UWOPS87_ctrl_hmg124h_13.JPG.dat	1380.0	79.4	1.1	0.06	#NUM!	5.0E-05
YOR321W	YOR321W	UWOPS87_ctrl_hmg124h_13.JPG.dat	1336.0	45.1	1.0	0.03	#NUM!	6.8E-06
YOR322C	YOR322C	UWOPS87_ctrl_hmg124h_13.JPG.dat	1314.8	86.8	1.0	0.06	#NUM!	5.4E-05
YOR324C	YOR324C	UWOPS87_ctrl_hmg124h_13.JPG.dat	1279.3	53.9	1.0	0.04	#NUM!	1.6E-05
YOR327C	YOR327C	UWOPS87_ctrl_hmg124h_13.JPG.dat	1203.3	78.5	1.0	0.05	#NUM!	4.6E-05
YOR328W	YOR328W	UWOPS87_ctrl_hmg124h_13.JPG.dat	1115.3	42.9	1.0	0.04	#NUM!	1.4E-05
YOR334W	YOR334W	UWOPS87_ctrl_hmg124h_13.JPG.dat	1194.0	47.4	1.0	0.04	#NUM!	1.3E-05
YOR337W	YOR337W	UWOPS87_ctrl_hmg124h_13.JPG.dat	1193.8	28.4	1.0	0.02	#NUM!	1.3E-06
YOR338W	YOR338W	UWOPS87_ctrl_hmg124h_13.JPG.dat	1277.5	150.0	1.0	0.12	#NUM!	4.1E-04
YOR339C	YOR339C	UWOPS87_ctrl_hmg124h_13.JPG.dat	1300.0	76.9	1.0	0.06	#NUM!	5.7E-05
YOR342C	YOR342C	UWOPS87_ctrl_hmg124h_13.JPG.dat	1185.5	114.1	0.9	0.09	#NUM!	2.5E-04
YOR343C	YOR343C	UWOPS87_ctrl_hmg124h_13.JPG.dat	1366.0	66.4	1.0	0.04	#NUM!	1.9E-05

YOR344C	YOR344C	UWOPS87_ctrl_hmg124h_13.JPG.dat	1288.3	91.6	1.0	0.07	#NUM!	8.3E-05
YOR346W	YOR346W	UWOPS87_ctrl_hmg124h_13.JPG.dat	1318.3	30.8	1.0	0.01	#NUM!	1.2E-05
YOR347C	YOR347C	UWOPS87_ctrl_hmg124h_13.JPG.dat	1297.5	145.0	1.0	0.11	#NUM!	4.0E-04
YOR348C	YOR348C	UWOPS87_ctrl_hmg124h_13.JPG.dat	1212.3	37.9	1.0	0.01	#NUM!	1.4E-05
YOR349W	YOR349W	UWOPS87_ctrl_hmg124h_13.JPG.dat	1050.8	58.7	1.0	0.04	#NUM!	2.3E-05
YOR350C	YOR350C	UWOPS87_ctrl_hmg124h_13.JPG.dat	1120.0	142.1	1.0	0.13	#NUM!	6.3E-04
YOR351C	YOR351C	UWOPS87_ctrl_hmg124h_13.JPG.dat	1358.3	91.9	1.1	0.09	#NUM!	1.6E-04
YOR352W	YOR352W	UWOPS87_ctrl_hmg124h_13.JPG.dat	1254.8	66.1	1.0	0.07	#NUM!	9.8E-05
YOR354C	YOR354C	UWOPS87_ctrl_hmg124h_13.JPG.dat	1216.0	88.7	1.0	0.01	#NUM!	5.3E-05
YOR355W	YOR355W	UWOPS87_ctrl_hmg124h_13.JPG.dat	1155.0	188.9	0.9	0.13	#NUM!	7.3E-04
YOR356W	YOR356W	UWOPS87_ctrl_hmg124h_13.JPG.dat	1256.3	187.4	1.1	0.02	#NUM!	1.0E-04
YOR357C	YOR357C	UWOPS87_ctrl_hmg124h_13.JPG.dat	1226.5	77.9	1.0	0.07	#NUM!	1.0E-04
YOR358W	YOR358W	UWOPS87_ctrl_hmg124h_13.JPG.dat	1232.3	43.5	1.0	0.01	#NUM!	4.5E-07
YOR359W	YOR359W	UWOPS87_ctrl_hmg124h_13.JPG.dat	1421.0	30.5	1.1	0.05	#NUM!	2.8E-05
YOR360C	YOR360C	UWOPS87_ctrl_hmg124h_13.JPG.dat	1047.3	526.6	1.1	0.12	#NUM!	3.7E-03
YOR363C	YOR363C	UWOPS87_ctrl_hmg124h_13.JPG.dat	1252.8	61.5	1.1	0.05	#NUM!	3.3E-05
YOR364W	YOR364W	UWOPS87_ctrl_hmg124h_13.JPG.dat	1249.3	49.3	1.0	0.04	#NUM!	1.6E-05
YOR365C	YOR365C	UWOPS87_ctrl_hmg124h_13.JPG.dat	1214.5	132.4	1.0	0.10	#NUM!	3.1E-04
YOR367W	YOR367W	UWOPS87_ctrl_hmg124h_13.JPG.dat	1183.5	88.3	0.9	0.08	#NUM!	1.4E-04
YOR368W	YOR368W	UWOPS87_ctrl_hmg124h_13.JPG.dat	1234.0	47.6	1.0	0.01	#NUM!	5.1E-05
YOR371C	YOR371C	UWOPS87_ctrl_hmg124h_13.JPG.dat	1386.0	137.1	1.1	0.11	#NUM!	2.8E-04
YOR374W	YOR374W	UWOPS87_ctrl_hmg124h_13.JPG.dat	1182.3	135.1	0.9	0.11	#NUM!	4.2E-04
YOR375C	YOR375C	UWOPS87_ctrl_hmg124h_13.JPG.dat	1121.3	125.5	0.8	0.03	#NUM!	4.2E-04
YOR376W	YOR376W	UWOPS87_ctrl_hmg124h_13.JPG.dat	1286.5	151.1	1.0	0.11	#NUM!	3.8E-04
YOR377W	YOR377W	UWOPS87_ctrl_hmg124h_13.JPG.dat	1295.8	29.5	1.0	0.03	#NUM!	8.0E-06
YOR378W	YOR378W	UWOPS87_ctrl_hmg124h_13.JPG.dat	1109.0	72.0	0.9	0.07	#NUM!	1.2E-04
YOR380W	YOR380W	UWOPS87_ctrl_hmg124h_13.JPG.dat	1136.8	52.7	1.0	0.06	#NUM!	4.6E-05
YOR381W	YOR381W	UWOPS87_ctrl_hmg124h_13.JPG.dat	1276.8	132.6	1.1	0.04	#NUM!	4.9E-04
YOR382W	YOR382W	UWOPS87_ctrl_hmg124h_13.JPG.dat	1172.8	50.4	1.0	0.05	#NUM!	4.2E-05
YOR383C	YOR383C	UWOPS87_ctrl_hmg124h_13.JPG.dat	1279.5	156.0	1.0	0.12	#NUM!	4.4E-04
YOR384W	YOR384W	UWOPS87_ctrl_hmg124h_13.JPG.dat	1384.0	110.8	1.1	0.10	#NUM!	2.0E-04
YOR385W	YOR385W	UWOPS87_ctrl_hmg124h_13.JPG.dat	1270.0	39.8	1.0	0.03	#NUM!	7.5E-06
YOR386W	YOR386W	UWOPS87_ctrl_hmg124h_13.JPG.dat	1154.5	77.7	0.9	0.06	#NUM!	5.7E-05
YPL001W	YPL001W	UWOPS87_ctrl_hmg124h_13.JPG.dat	989.5	689.3	0.8	0.59	#NUM!	6.5E-02
YPL003W	YPL003W	UWOPS87_ctrl_hmg124h_13.JPG.dat	1080.5	77.1	0.9	0.05	#NUM!	4.3E-05
YPL004C	YPL004C	UWOPS87_ctrl_hmg124h_13.JPG.dat	1275.3	27.0	1.1	0.02	#NUM!	1.8E-06
YPL008W	YPL008W	UWOPS87_ctrl_hmg124h_13.JPG.dat	1274.3	41.5	1.1	0.04	#NUM!	1.8E-05
YPL009C	YPL009C	UWOPS87_ctrl_hmg124h_13.JPG.dat	1332.3	18.5	1.1	0.01	#NUM!	1.0E-07
YPL014W	YPL014W	UWOPS87_ctrl_hmg124h_13.JPG.dat	1125.3	82.2	0.9	0.07	#NUM!	1.1E-04
YPL017C	YPL017C	UWOPS87_ctrl_hmg124h_13.JPG.dat	1364.5	69.8	1.1	0.05	#NUM!	3.1E-05
YPL018W	YPL018W	UWOPS87_ctrl_hmg124h_13.JPG.dat	1289.8	86.3	1.0	0.06	#NUM!	4.8E-05
YPL019C	YPL019C	UWOPS87_ctrl_hmg124h_13.JPG.dat	1264.5	74.5	1.0	0.02	#NUM!	9.5E-05
YPL021W	YPL021W	UWOPS87_ctrl_hmg124h_13.JPG.dat	1286.5	48.3	1.1	0.02	#NUM!	2.6E-06
YPL022W	YPL022W	UWOPS87_ctrl_hmg124h_13.JPG.dat	1128.3	141.9	0.9	0.11	#NUM!	4.7E-04
YPL023C	YPL023C	UWOPS87_ctrl_hmg124h_13.JPG.dat	1178.0	47.9	1.0	0.04	#NUM!	1.4E-05
YPL024W	YPL024W	UWOPS87_ctrl_hmg124h_13.JPG.dat	788.0	604.7	0.7	0.52	#NUM!	8.1E-02
YPL025C	YPL025C	UWOPS87_ctrl_hmg124h_13.JPG.dat	1142.0	97.4	1.0	0.09	#NUM!	2.1E-04
YPL026C	YPL026C	UWOPS87_ctrl_hmg124h_13.JPG.dat	1269.8	38.4	1.1	0.01	#NUM!	7.4E-06
YPL027W	YPL027W	UWOPS87_ctrl_hmg124h_13.JPG.dat	1119.3	79.6	1.0	0.07	#NUM!	8.7E-05
YPL030W	YPL030W	UWOPS87_ctrl_hmg124h_13.JPG.dat	1266.0	116.7	1.0	0.09	#NUM!	1.7E-04
YPL032C	YPL032C	UWOPS87_ctrl_hmg124h_13.JPG.dat	1376.0	6.5	1.1	0.02	#NUM!	1.8E-06
YPL033C	YPL033C	UWOPS87_ctrl_hmg124h_13.JPG.dat	1288.8	140.0	1.1	0.10	#NUM!	2.4E-04
YPL034W	YPL034W	UWOPS87_ctrl_hmg124h_13.JPG.dat	1394.5	158.6	1.1	0.14	#NUM!	5.3E-04
YPL036W	YPL036W	UWOPS87_ctrl_hmg124h_13.JPG.dat	1131.3	36.3	1.0	0.03	#NUM!	1.3E-05
YPL037C	YPL037C	UWOPS87_ctrl_hmg124h_13.JPG.dat	1222.5	123.3	1.0	0.02	#NUM!	1.1E-04
YPL038W	YPL038W	UWOPS87_ctrl_hmg124h_13.JPG.dat	1190.3	62.1	1.0	0.07	#NUM!	8.2E-05
YPL039W	YPL039W	UWOPS87_ctrl_hmg124h_13.JPG.dat	1181.3	42.2	1.1	0.03	#NUM!	7.8E-06
YPL041C	YPL041C	UWOPS87_ctrl_hmg124h_13.JPG.dat	1086.3	18.7	1.0	0.02	#NUM!	1.5E-06
YPL046C	YPL046C	UWOPS87_ctrl_hmg124h_13.JPG.dat	1037.3	67.9	1.0	0.02	#NUM!	1.4E-04
YPL047W	YPL047W	UWOPS87_ctrl_hmg124h_13.JPG.dat	1174.0	61.0	1.0	0.01	#NUM!	7.6E-05
YPL048W	YPL048W	UWOPS87_ctrl_hmg124h_13.JPG.dat	1123.0	68.2	0.9	0.03	#NUM!	1.6E-05
YPL051W	YPL051W	UWOPS87_ctrl_hmg124h_13.JPG.dat	1385.8	76.5	1.1	0.08	#NUM!	1.0E-04
YPL052W	YPL052W	UWOPS87_ctrl_hmg124h_13.JPG.dat	1343.3	65.6	1.0	0.02	#NUM!	1.9E-06
YPL053C	YPL053C	UWOPS87_ctrl_hmg124h_13.JPG.dat	1428.0	107.9	1.1	0.06	#NUM!	3.9E-05
YPL054W	YPL054W	UWOPS87_ctrl_hmg124h_13.JPG.dat	1452.5	147.4	1.1	0.09	#NUM!	1.5E-04
YPL055C	YPL055C	UWOPS87_ctrl_hmg124h_13.JPG.dat	1464.3	89.2	1.1	0.09	#NUM!	1.6E-04
YPL056C	YPL056C	UWOPS87_ctrl_hmg124h_13.JPG.dat	1385.3	119.5	1.0	0.10	#NUM!	2.4E-04
YPL057C	YPL057C	UWOPS87_ctrl_hmg124h_13.JPG.dat	1329.0	55.0	1.0	0.04	#NUM!	1.8E-05
YPL058C	YPL058C	UWOPS87_ctrl_hmg124h_13.JPG.dat	1345.0	112.6	1.0	0.06	#NUM!	5.3E-05
YPL060W	YPL060W	UWOPS87_ctrl_hmg124h_13.JPG.dat	1370.8	142.4	1.1	0.14	#NUM!	5.3E-04

YPL061W	YPL061W	UWOPS87_ctrl_hmg124h_13.JPG.dat	1011.5	41.6	0.9	0.01	#NUM!	2.1E-05
YPL062W	YPL062W	UWOPS87_ctrl_hmg124h_13.JPG.dat	1131.8	60.0	1.0	0.06	#NUM!	5.2E-05
YPL064C	YPL064C	UWOPS87_ctrl_hmg124h_13.JPG.dat	1160.8	91.1	1.0	0.07	#NUM!	1.1E-04
YPL066W	YPL066W	UWOPS87_ctrl_hmg124h_13.JPG.dat	1185.0	75.0	1.0	0.07	#NUM!	8.6E-05
YPL067C	YPL067C	UWOPS87_ctrl_hmg124h_13.JPG.dat	1447.8	165.4	1.2	0.13	#NUM!	3.7E-04
YPL068C	YPL068C	UWOPS87_ctrl_hmg124h_13.JPG.dat	1281.0	69.4	1.0	0.01	#NUM!	4.4E-05
YPL069C	YPL069C	UWOPS87_ctrl_hmg124h_13.JPG.dat	1432.8	82.3	1.1	0.06	#NUM!	4.7E-05
YPL070W	YPL070W	UWOPS87_ctrl_hmg124h_13.JPG.dat	1344.5	47.5	1.0	0.04	#NUM!	1.8E-05
YPL071C	YPL071C	UWOPS87_ctrl_hmg124h_13.JPG.dat	1294.3	44.8	1.0	0.01	#NUM!	6.7E-05
YPL073C	YPL073C	UWOPS87_ctrl_hmg124h_13.JPG.dat	1195.5	207.7	1.0	0.04	#NUM!	4.6E-04
YPL074W	YPL074W	UWOPS87_ctrl_hmg124h_13.JPG.dat	1279.5	30.8	1.1	0.03	#NUM!	9.3E-06
YPL079W	YPL079W	UWOPS87_ctrl_hmg124h_13.JPG.dat	1186.8	101.4	1.0	0.02	#NUM!	1.1E-04
YPL080C	YPL080C	UWOPS87_ctrl_hmg124h_13.JPG.dat	924.8	68.6	0.8	0.06	#NUM!	8.9E-05
YPL081W	YPL081W	UWOPS87_ctrl_hmg124h_13.JPG.dat	1104.0	58.6	0.9	0.01	#NUM!	4.3E-05
YPL086C	YPL086C	UWOPS87_ctrl_hmg124h_13.JPG.dat	1226.5	90.0	1.0	0.02	#NUM!	8.8E-05
YPL087W	YPL087W	UWOPS87_ctrl_hmg124h_13.JPG.dat	1174.5	93.7	0.9	0.07	#NUM!	1.3E-04
YPL088W	YPL088W	UWOPS87_ctrl_hmg124h_13.JPG.dat	1182.0	31.0	0.9	0.01	#NUM!	1.9E-05
YPL089C	YPL089C	UWOPS87_ctrl_hmg124h_13.JPG.dat	1399.3	96.7	1.1	0.08	#NUM!	9.4E-05
YPL090C	YPL090C	UWOPS87_ctrl_hmg124h_13.JPG.dat	1055.8	322.0	0.8	0.25	#NUM!	7.1E-03
YPL091W	YPL091W	UWOPS87_ctrl_hmg124h_13.JPG.dat	1314.8	93.0	1.1	0.01	#NUM!	5.5E-05
YPL092W	YPL092W	UWOPS87_ctrl_hmg124h_13.JPG.dat	1341.8	49.3	1.1	0.01	#NUM!	5.7E-05
YPL095C	YPL095C	UWOPS87_ctrl_hmg124h_13.JPG.dat	1176.8	74.7	1.0	0.06	#NUM!	6.3E-05
YPL096W	YPL096W	UWOPS87_ctrl_hmg124h_13.JPG.dat	1178.5	50.3	1.1	0.01	#NUM!	4.3E-05
YPL098C	YPL098C	UWOPS87_ctrl_hmg124h_13.JPG.dat	1224.0	139.3	1.0	0.11	#NUM!	3.4E-04
YPL099C	YPL099C	UWOPS87_ctrl_hmg124h_13.JPG.dat	1308.3	119.9	1.1	0.09	#NUM!	1.6E-04
YPL100W	YPL100W	UWOPS87_ctrl_hmg124h_13.JPG.dat	1318.0	24.9	1.0	0.01	#NUM!	1.5E-07
YPL101W	YPL101W	UWOPS87_ctrl_hmg124h_13.JPG.dat	1310.8	117.7	1.0	0.08	#NUM!	1.4E-04
YPL102C	YPL102C	UWOPS87_ctrl_hmg124h_13.JPG.dat	1233.0	141.7	1.0	0.11	#NUM!	4.5E-04
YPL103C	YPL103C	UWOPS87_ctrl_hmg124h_13.JPG.dat	1143.5	65.6	0.9	0.06	#NUM!	8.9E-05
YPL105C	YPL105C	UWOPS87_ctrl_hmg124h_13.JPG.dat	1308.0	41.5	1.0	0.03	#NUM!	6.2E-06
YPL106C	YPL106C	UWOPS87_ctrl_hmg124h_13.JPG.dat	1157.5	61.3	0.9	0.01	#NUM!	5.5E-05
YPL107W	YPL107W	UWOPS87_ctrl_hmg124h_13.JPG.dat	1360.3	58.6	1.1	0.06	#NUM!	4.2E-05
YPL108W	YPL108W	UWOPS87_ctrl_hmg124h_13.JPG.dat	1104.0	65.3	0.9	0.06	#NUM!	5.5E-05
YPL109C	YPL109C	UWOPS87_ctrl_hmg124h_13.JPG.dat	989.8	56.8	0.9	0.04	#NUM!	3.5E-05
YPL110C	YPL110C	UWOPS87_ctrl_hmg124h_13.JPG.dat	1325.8	29.9	1.1	0.02	#NUM!	1.1E-06
YPL111W	YPL111W	UWOPS87_ctrl_hmg124h_13.JPG.dat	700.5	515.1	0.6	0.43	#NUM!	7.3E-02
YPL112C	YPL112C	UWOPS87_ctrl_hmg124h_13.JPG.dat	1255.3	50.2	1.0	0.03	#NUM!	8.0E-06
YPL113C	YPL113C	UWOPS87_ctrl_hmg124h_13.JPG.dat	1256.8	92.1	1.0	0.08	#NUM!	1.3E-04
YPL114W	YPL114W	UWOPS87_ctrl_hmg124h_13.JPG.dat	1290.8	143.0	1.0	0.11	#NUM!	3.1E-04
YPL115C	YPL115C	UWOPS87_ctrl_hmg124h_13.JPG.dat	1187.8	80.0	0.9	0.00	#NUM!	9.2E-06
YPL116W	YPL116W	UWOPS87_ctrl_hmg124h_13.JPG.dat	1265.0	78.8	1.0	0.07	#NUM!	6.8E-05
YPL119C	YPL119C	UWOPS87_ctrl_hmg124h_13.JPG.dat	1219.0	41.4	1.0	0.01	#NUM!	3.9E-05
YPL120W	YPL120W	UWOPS87_ctrl_hmg124h_13.JPG.dat	1318.3	105.4	1.1	0.09	#NUM!	1.4E-04
YPL121C	YPL121C	UWOPS87_ctrl_hmg124h_13.JPG.dat	1120.0	26.1	1.0	0.02	#NUM!	4.5E-06
YPL123C	YPL123C	UWOPS87_ctrl_hmg124h_13.JPG.dat	1232.5	77.7	1.0	0.08	#NUM!	1.1E-04
YPL125W	YPL125W	UWOPS87_ctrl_hmg124h_13.JPG.dat	1183.8	44.2	1.0	0.05	#NUM!	3.6E-05
YPL127C	YPL127C	UWOPS87_ctrl_hmg124h_13.JPG.dat	1278.0	41.9	1.0	0.02	#NUM!	1.4E-06
YPL130W	YPL130W	UWOPS87_ctrl_hmg124h_13.JPG.dat	1283.3	109.1	1.0	0.09	#NUM!	2.0E-04
YPL133C	YPL133C	UWOPS87_ctrl_hmg124h_13.JPG.dat	1303.0	183.3	1.0	0.14	#NUM!	6.0E-04
YPL134C	YPL134C	UWOPS87_ctrl_hmg124h_13.JPG.dat	1257.3	125.4	1.0	0.11	#NUM!	3.0E-04
YPL135W	YPL135W	UWOPS87_ctrl_hmg124h_13.JPG.dat	1365.3	73.3	1.1	0.07	#NUM!	6.4E-05
YPL136W	YPL136W	UWOPS87_ctrl_hmg124h_13.JPG.dat	1055.3	73.9	0.9	0.06	#NUM!	6.5E-05
YPL137C	YPL137C	UWOPS87_ctrl_hmg124h_13.JPG.dat	1185.3	135.0	1.0	0.11	#NUM!	3.7E-04
YPL138C	YPL138C	UWOPS87_ctrl_hmg124h_13.JPG.dat	1230.5	73.7	1.0	0.00	#NUM!	2.2E-06
YPL139C	YPL139C	UWOPS87_ctrl_hmg124h_13.JPG.dat	1258.0	55.0	1.1	0.01	#NUM!	1.6E-05
YPL140C	YPL140C	UWOPS87_ctrl_hmg124h_13.JPG.dat	1316.8	49.3	1.1	0.04	#NUM!	1.4E-05
YPL141C	YPL141C	UWOPS87_ctrl_hmg124h_13.JPG.dat	1278.3	54.7	1.0	0.06	#NUM!	4.5E-05
YPL144W	YPL144W	UWOPS87_ctrl_hmg124h_13.JPG.dat	1154.8	172.2	0.9	0.13	#NUM!	7.0E-04
YPL145C	YPL145C	UWOPS87_ctrl_hmg124h_13.JPG.dat	1272.8	96.9	1.0	0.09	#NUM!	1.6E-04
YPL147W	YPL147W	UWOPS87_ctrl_hmg124h_13.JPG.dat	1260.0	40.3	1.0	0.04	#NUM!	1.6E-05
YPL149W	YPL149W	UWOPS87_ctrl_hmg124h_13.JPG.dat	1201.5	62.0	1.0	0.04	#NUM!	2.1E-05
YPL150W	YPL150W	UWOPS87_ctrl_hmg124h_13.JPG.dat	1130.3	115.4	0.9	0.03	#NUM!	2.7E-04
YPL152W	YPL152W	UWOPS87_ctrl_hmg124h_13.JPG.dat	1170.3	35.8	1.0	0.02	#NUM!	2.3E-06
YPL154C	YPL154C	UWOPS87_ctrl_hmg124h_13.JPG.dat	1192.8	40.4	1.0	0.04	#NUM!	1.3E-05
YPL155C	YPL155C	UWOPS87_ctrl_hmg124h_13.JPG.dat	1096.5	44.7	1.0	0.03	#NUM!	1.2E-05
YPL156C	YPL156C	UWOPS87_ctrl_hmg124h_13.JPG.dat	967.0	26.5	0.9	0.03	#NUM!	1.7E-05
YPL157W	YPL157W	UWOPS87_ctrl_hmg124h_13.JPG.dat	1054.3	154.3	0.9	0.11	#NUM!	5.1E-04
YPL159C	YPL159C	UWOPS87_ctrl_hmg124h_13.JPG.dat	1143.8	24.6	0.9	0.05	#NUM!	4.9E-05
YPL161C	YPL161C	UWOPS87_ctrl_hmg124h_13.JPG.dat	990.5	111.8	0.8	0.08	#NUM!	2.5E-04
YPL162C	YPL162C	UWOPS87_ctrl_hmg124h_13.JPG.dat	1243.0	130.4	1.0	0.10	#NUM!	3.0E-04

YPL163C	YPL163C	UWOPS87_ctrl_hmg124h_13.JPG.dat	1180.5	106.5	0.9	0.00	#NUM!	8.7E-07
YPL164C	YPL164C	UWOPS87_ctrl_hmg124h_13.JPG.dat	1290.5	171.6	0.9	0.03	#NUM!	4.6E-04
YPL165C	YPL165C	UWOPS87_ctrl_hmg124h_13.JPG.dat	1378.5	175.3	1.0	0.15	#NUM!	8.9E-04
YPL166W	YPL166W	UWOPS87_ctrl_hmg124h_13.JPG.dat	1318.5	28.0	1.0	0.02	#NUM!	1.1E-06
YPL167C	YPL167C	UWOPS87_ctrl_hmg124h_13.JPG.dat	1399.5	104.3	1.0	0.05	#NUM!	3.8E-05
YPL168W	YPL168W	UWOPS87_ctrl_hmg124h_13.JPG.dat	1355.3	28.1	1.0	0.01	#NUM!	2.1E-07
YPL170W	YPL170W	UWOPS87_ctrl_hmg124h_13.JPG.dat	1250.5	85.5	1.0	0.07	#NUM!	1.1E-04
YPL171C	YPL171C	UWOPS87_ctrl_hmg124h_13.JPG.dat	1056.5	64.5	1.0	0.07	#NUM!	1.2E-04
YPL172C	YPL172C	UWOPS87_ctrl_hmg124h_13.JPG.dat	1165.0	34.3	1.0	0.03	#NUM!	4.3E-06
YPL174C	YPL174C	UWOPS87_ctrl_hmg124h_13.JPG.dat	1182.0	200.2	1.0	0.16	#NUM!	1.1E-03
YPL176C	YPL176C	UWOPS87_ctrl_hmg124h_13.JPG.dat	1255.3	54.5	1.0	0.05	#NUM!	3.6E-05
YPL177C	YPL177C	UWOPS87_ctrl_hmg124h_13.JPG.dat	1315.0	108.8	1.0	0.09	#NUM!	1.6E-04
YPL178W	YPL178W	UWOPS87_ctrl_hmg124h_13.JPG.dat	1272.0	62.9	1.0	0.05	#NUM!	2.6E-05
YPL179W	YPL179W	UWOPS87_ctrl_hmg124h_13.JPG.dat	1190.0	121.8	0.9	0.09	#NUM!	2.2E-04
YPL180W	YPL180W	UWOPS87_ctrl_hmg124h_13.JPG.dat	1312.0	72.1	1.0	0.05	#NUM!	4.2E-05
YPL181W	YPL181W	UWOPS87_ctrl_hmg124h_13.JPG.dat	1268.8	17.6	1.0	0.01	#NUM!	9.0E-07
YPL182C	YPL182C	UWOPS87_ctrl_hmg124h_13.JPG.dat	1234.3	25.3	1.0	0.03	#NUM!	7.5E-06
YPL183C	YPL183C	UWOPS87_ctrl_hmg124h_13.JPG.dat	1075.3	99.8	0.9	0.07	#NUM!	1.7E-04
YPL183W-A	YPL183W-A	UWOPS87_ctrl_hmg124h_13.JPG.dat	1140.5	31.3	1.1	0.03	#NUM!	5.4E-06
YPL184C	YPL184C	UWOPS87_ctrl_hmg124h_13.JPG.dat	1096.8	52.2	1.0	0.05	#NUM!	4.5E-05
YPL185W	YPL185W	UWOPS87_ctrl_hmg124h_13.JPG.dat	1191.8	72.5	1.0	0.01	#NUM!	4.2E-05
YPL186C	YPL186C	UWOPS87_ctrl_hmg124h_13.JPG.dat	1219.0	66.1	1.0	0.05	#NUM!	2.6E-05
YPL187W	YPL187W	UWOPS87_ctrl_hmg124h_13.JPG.dat	1274.8	39.3	1.0	0.03	#NUM!	8.0E-06
YPL189W	YPL189W	UWOPS87_ctrl_hmg124h_13.JPG.dat	1253.8	43.9	1.0	0.04	#NUM!	1.2E-05
YPL191C	YPL191C	UWOPS87_ctrl_hmg124h_13.JPG.dat	1295.3	45.5	1.0	0.03	#NUM!	7.5E-06
YPL192C	YPL192C	UWOPS87_ctrl_hmg124h_13.JPG.dat	1257.5	43.4	1.0	0.03	#NUM!	8.4E-06
YPL194W	YPL194W	UWOPS87_ctrl_hmg124h_13.JPG.dat	1194.0	47.1	0.9	0.03	#NUM!	1.3E-05
YPL195W	YPL195W	UWOPS87_ctrl_hmg124h_13.JPG.dat	1271.0	15.7	1.0	0.01	#NUM!	2.9E-07
YPL196W	YPL196W	UWOPS87_ctrl_hmg124h_13.JPG.dat	1376.3	95.0	1.1	0.06	#NUM!	4.8E-05
YPL197C	YPL197C	UWOPS87_ctrl_hmg124h_13.JPG.dat	1063.3	56.9	1.0	0.05	#NUM!	4.5E-05
YPL198W	YPL198W	UWOPS87_ctrl_hmg124h_13.JPG.dat	1217.5	105.6	1.0	0.10	#NUM!	2.1E-04
YPL199C	YPL199C	UWOPS87_ctrl_hmg124h_13.JPG.dat	1221.0	61.8	1.0	0.06	#NUM!	4.9E-05
YPL200W	YPL200W	UWOPS87_ctrl_hmg124h_13.JPG.dat	1274.5	116.0	1.1	0.02	#NUM!	1.5E-04
YPL201C	YPL201C	UWOPS87_ctrl_hmg124h_13.JPG.dat	1183.0	23.9	0.9	0.01	#NUM!	1.1E-06
YPL202C	YPL202C	UWOPS87_ctrl_hmg124h_13.JPG.dat	1372.0	38.5	1.1	0.03	#NUM!	6.2E-06
YPL203W	YPL203W	UWOPS87_ctrl_hmg124h_13.JPG.dat	1385.5	15.8	1.1	0.00	#NUM!	4.7E-06
YPL205C	YPL205C	UWOPS87_ctrl_hmg124h_13.JPG.dat	1241.8	22.4	1.0	0.01	#NUM!	9.0E-06
YPL206C	YPL206C	UWOPS87_ctrl_hmg124h_13.JPG.dat	1267.5	50.0	1.0	0.01	#NUM!	1.7E-05
YPL207W	YPL207W	UWOPS87_ctrl_hmg124h_13.JPG.dat	1191.8	62.8	0.9	0.00	#NUM!	6.3E-06
YPL208W	YPL208W	UWOPS87_ctrl_hmg124h_13.JPG.dat	1199.0	58.2	1.0	0.01	#NUM!	6.4E-05
YPL212C	YPL212C	UWOPS87_ctrl_hmg124h_13.JPG.dat	1052.5	45.4	0.9	0.02	#NUM!	1.1E-04
YPL213W	YPL213W	UWOPS87_ctrl_hmg124h_13.JPG.dat	1242.0	76.5	1.0	0.02	#NUM!	1.4E-04
YPL214C	YPL214C	UWOPS87_ctrl_hmg124h_13.JPG.dat	1268.0	124.6	1.0	0.10	#NUM!	2.2E-04
YPL216W	YPL216W	UWOPS87_ctrl_hmg124h_13.JPG.dat	1319.5	107.9	1.1	0.08	#NUM!	1.2E-04
YPL219W	YPL219W	UWOPS87_ctrl_hmg124h_13.JPG.dat	1336.8	97.9	1.0	0.01	#NUM!	4.9E-05
YPL220W	YPL220W	UWOPS87_ctrl_hmg124h_13.JPG.dat	1144.0	82.0	0.9	0.06	#NUM!	9.6E-05
YPL221W	YPL221W	UWOPS87_ctrl_hmg124h_13.JPG.dat	1212.5	54.1	1.0	0.05	#NUM!	4.0E-05
YPL222W	YPL222W	UWOPS87_ctrl_hmg124h_13.JPG.dat	1274.8	32.1	1.0	0.03	#NUM!	6.2E-06
YPL223C	YPL223C	UWOPS87_ctrl_hmg124h_13.JPG.dat	1328.3	69.1	1.1	0.05	#NUM!	2.1E-05
YPL224C	YPL224C	UWOPS87_ctrl_hmg124h_13.JPG.dat	1194.0	110.7	1.0	0.08	#NUM!	1.8E-04
YPL225W	YPL225W	UWOPS87_ctrl_hmg124h_13.JPG.dat	1034.3	102.0	0.9	0.09	#NUM!	2.5E-04
YPL226W	YPL226W	UWOPS87_ctrl_hmg124h_13.JPG.dat	1048.0	70.0	0.9	0.04	#NUM!	3.1E-05
YPL227C	YPL227C	UWOPS87_ctrl_hmg124h_13.JPG.dat	1327.8	63.8	1.1	0.07	#NUM!	6.0E-05
YPL229W	YPL229W	UWOPS87_ctrl_hmg124h_13.JPG.dat	927.0	615.3	1.0	0.06	#NUM!	1.2E-03
YPL230W	YPL230W	UWOPS87_ctrl_hmg124h_13.JPG.dat	1214.8	39.8	1.0	0.05	#NUM!	3.3E-05
YPL232W	YPL232W	UWOPS87_ctrl_hmg124h_13.JPG.dat	1273.8	109.3	1.0	0.08	#NUM!	1.2E-04
YPL236C	YPL236C	UWOPS87_ctrl_hmg124h_13.JPG.dat	1191.8	138.3	1.0	0.12	#NUM!	5.6E-04
YPL239W	YPL239W	UWOPS87_ctrl_hmg124h_13.JPG.dat	1262.3	97.7	1.0	0.07	#NUM!	8.0E-05
YPL240C	YPL240C	UWOPS87_ctrl_hmg124h_13.JPG.dat	1219.8	102.7	1.0	0.03	#NUM!	2.6E-04
YPL241C	YPL241C	UWOPS87_ctrl_hmg124h_13.JPG.dat	1136.5	62.9	1.0	0.06	#NUM!	5.6E-05
YPL244C	YPL244C	UWOPS87_ctrl_hmg124h_13.JPG.dat	1126.3	85.7	0.9	0.07	#NUM!	1.2E-04
YPL245W	YPL245W	UWOPS87_ctrl_hmg124h_13.JPG.dat	1115.3	158.7	1.0	0.12	#NUM!	6.0E-04
YPL246C	YPL246C	UWOPS87_ctrl_hmg124h_13.JPG.dat	1206.3	81.5	1.0	0.06	#NUM!	6.5E-05
YPL247C	YPL247C	UWOPS87_ctrl_hmg124h_13.JPG.dat	1256.0	39.1	1.0	0.03	#NUM!	4.5E-06
YPL248C	YPL248C	UWOPS87_ctrl_hmg124h_13.JPG.dat	1290.5	89.8	1.0	0.06	#NUM!	5.0E-05
YPL249C	YPL249C	UWOPS87_ctrl_hmg124h_13.JPG.dat	1326.3	151.2	1.0	0.01	#NUM!	5.1E-05
YPL250C	YPL250C	UWOPS87_ctrl_hmg124h_13.JPG.dat	1199.8	40.8	1.0	0.03	#NUM!	9.4E-06
YPL253C	YPL253C	UWOPS87_ctrl_hmg124h_13.JPG.dat	1161.5	95.7	1.0	0.08	#NUM!	1.6E-04
YPL256C	YPL256C	UWOPS87_ctrl_hmg124h_13.JPG.dat	1075.3	28.8	0.9	0.02	#NUM!	4.8E-06
YPL257W	YPL257W	UWOPS87_ctrl_hmg124h_13.JPG.dat	1236.3	39.9	1.1	0.01	#NUM!	1.7E-05

YPL258C	YPL258C	UWOPS87_ctrl_hmg124h_13.JPG.dat	1097.5	84.0	1.0	0.06	#NUM!	8.0E-05
YPL259C	YPL259C	UWOPS87_ctrl_hmg124h_13.JPG.dat	1127.5	102.6	1.0	0.08	#NUM!	1.2E-04
YPL260W	YPL260W	UWOPS87_ctrl_hmg124h_13.JPG.dat	1123.5	78.3	1.0	0.08	#NUM!	1.4E-04
1	1	UWOPS87_ctrl_hmg124h_14.JPG.dat	1412.5	100.7	1.1	0.09	#NUM!	1.5E-04
2	2	UWOPS87_ctrl_hmg124h_14.JPG.dat	1056.8	88.6	0.9	0.04	#NUM!	3.1E-05
3	3	UWOPS87_ctrl_hmg124h_14.JPG.dat	1192.0	216.3	1.0	0.17	#NUM!	1.2E-03
4	4	UWOPS87_ctrl_hmg124h_14.JPG.dat	1119.3	112.7	1.0	0.08	#NUM!	1.7E-04
YAL024C	YAL024C	UWOPS87_ctrl_hmg124h_14.JPG.dat	969.8	184.7	0.9	0.17	#NUM!	1.6E-03
YAL046C	YAL046C	UWOPS87_ctrl_hmg124h_14.JPG.dat	1234.8	14.8	1.1	0.01	#NUM!	3.9E-07
YAL048C	YAL048C	UWOPS87_ctrl_hmg124h_14.JPG.dat	1269.3	35.9	1.1	0.00	#NUM!	6.8E-06
YBL103C	YBL103C	UWOPS87_ctrl_hmg124h_14.JPG.dat	900.0	92.0	0.9	0.09	#NUM!	2.9E-04
YBR020W	YBR020W	UWOPS87_ctrl_hmg124h_14.JPG.dat	1199.8	153.8	1.1	0.13	#NUM!	4.6E-04
YBR061C	YBR061C	UWOPS87_ctrl_hmg124h_14.JPG.dat	993.3	667.2	1.2	0.07	#NUM!	1.1E-03
YBR075W	YBR075W	UWOPS87_ctrl_hmg124h_14.JPG.dat	1024.5	93.5	0.9	0.09	#NUM!	2.3E-04
YBR078W	YBR078W	UWOPS87_ctrl_hmg124h_14.JPG.dat	1297.3	119.1	1.1	0.10	#NUM!	2.4E-04
YBR082C	YBR082C	UWOPS87_ctrl_hmg124h_14.JPG.dat	996.3	98.2	0.8	0.03	#NUM!	5.5E-04
YBR085W	YBR085W	UWOPS87_ctrl_hmg124h_14.JPG.dat	1011.0	674.9	1.2	0.03	#NUM!	1.5E-04
YBR086C	YBR086C	UWOPS87_ctrl_hmg124h_14.JPG.dat	1099.0	45.8	1.0	0.05	#NUM!	4.7E-05
YBR095C	YBR095C	UWOPS87_ctrl_hmg124h_14.JPG.dat	1221.5	64.8	1.0	0.04	#NUM!	2.3E-05
YBR115C	YBR115C	UWOPS87_ctrl_hmg124h_14.JPG.dat	1054.3	78.8	1.0	0.08	#NUM!	1.1E-04
YBR118W	YBR118W	UWOPS87_ctrl_hmg124h_14.JPG.dat	1136.0	38.2	1.0	0.03	#NUM!	5.1E-06
YBR165W	YBR165W	UWOPS87_ctrl_hmg124h_14.JPG.dat	1123.5	82.6	1.1	0.08	#NUM!	1.3E-04
YBR299W	YBR299W	UWOPS87_ctrl_hmg124h_14.JPG.dat	1085.5	33.6	1.0	0.04	#NUM!	2.6E-05
YCR107W	YCR107W	UWOPS87_ctrl_hmg124h_14.JPG.dat	1135.0	83.1	1.0	0.07	#NUM!	8.2E-05
YDL074C	YDL074C	UWOPS87_ctrl_hmg124h_14.JPG.dat	1244.3	48.8	1.1	0.04	#NUM!	1.7E-05
YDR026C	YDR026C	UWOPS87_ctrl_hmg124h_14.JPG.dat	1127.3	97.4	1.0	0.08	#NUM!	1.5E-04
YDR029W	YDR029W	UWOPS87_ctrl_hmg124h_14.JPG.dat	1085.8	88.4	1.0	0.01	#NUM!	2.2E-05
YDR030C	YDR030C	UWOPS87_ctrl_hmg124h_14.JPG.dat	1418.8	97.5	1.2	0.09	#NUM!	1.1E-04
YDR031W	YDR031W	UWOPS87_ctrl_hmg124h_14.JPG.dat	1015.3	72.7	0.9	0.06	#NUM!	9.0E-05
YDR032C	YDR032C	UWOPS87_ctrl_hmg124h_14.JPG.dat	1151.3	177.0	1.0	0.14	#NUM!	9.3E-04
YDR033W	YDR033W	UWOPS87_ctrl_hmg124h_14.JPG.dat	1079.8	147.2	1.0	0.12	#NUM!	5.4E-04
YDR034C	YDR034C	UWOPS87_ctrl_hmg124h_14.JPG.dat	1148.8	185.5	1.0	0.16	#NUM!	1.0E-03
YDR035W	YDR035W	UWOPS87_ctrl_hmg124h_14.JPG.dat	1081.3	44.6	1.0	0.00	#NUM!	3.7E-06
YDR036C	YDR036C	UWOPS87_ctrl_hmg124h_14.JPG.dat	1228.8	104.7	1.0	0.03	#NUM!	3.3E-04
YDR042C	YDR042C	UWOPS87_ctrl_hmg124h_14.JPG.dat	886.5	42.2	0.8	0.01	#NUM!	7.8E-05
YDR043C	YDR043C	UWOPS87_ctrl_hmg124h_14.JPG.dat	1166.3	149.5	1.2	0.15	#NUM!	5.8E-04
YDR046C	YDR046C	UWOPS87_ctrl_hmg124h_14.JPG.dat	1095.8	106.5	1.0	0.10	#NUM!	2.6E-04
YDR242W	YDR242W	UWOPS87_ctrl_hmg124h_14.JPG.dat	1239.3	50.4	1.0	0.05	#NUM!	2.4E-05
YDR269C	YDR269C	UWOPS87_ctrl_hmg124h_14.JPG.dat	1212.0	63.0	1.2	0.01	#NUM!	1.1E-05
YDR271C	YDR271C	UWOPS87_ctrl_hmg124h_14.JPG.dat	1162.8	157.8	1.1	0.05	#NUM!	7.1E-04
YDR290W	YDR290W	UWOPS87_ctrl_hmg124h_14.JPG.dat	1215.5	46.5	1.2	0.04	#NUM!	8.0E-06
YDR326C	YDR326C	UWOPS87_ctrl_hmg124h_14.JPG.dat	1071.5	100.1	1.0	0.03	#NUM!	3.7E-04
YDR444W	YDR444W	UWOPS87_ctrl_hmg124h_14.JPG.dat	1049.3	92.2	0.9	0.02	#NUM!	1.7E-04
YDR461W	YDR461W	UWOPS87_ctrl_hmg124h_14.JPG.dat	930.0	34.0	1.0	0.03	#NUM!	1.2E-05
YDR477W	YDR477W	UWOPS87_ctrl_hmg124h_14.JPG.dat	1089.5	75.1	1.0	0.09	#NUM!	1.5E-04
YDR483W	YDR483W	UWOPS87_ctrl_hmg124h_14.JPG.dat	901.8	92.8	0.8	0.09	#NUM!	3.2E-04
YDR493W	YDR493W	UWOPS87_ctrl_hmg124h_14.JPG.dat	877.0	26.5	0.9	0.03	#NUM!	1.3E-05
YDR502C	YDR502C	UWOPS87_ctrl_hmg124h_14.JPG.dat	949.5	44.5	0.9	0.04	#NUM!	2.8E-05
YDR515W	YDR515W	UWOPS87_ctrl_hmg124h_14.JPG.dat	976.3	104.3	1.0	0.10	#NUM!	3.0E-04
YER089C	YER089C	UWOPS87_ctrl_hmg124h_14.JPG.dat	1125.5	89.1	1.1	0.10	#NUM!	2.0E-04
YFL001W	YFL001W	UWOPS87_ctrl_hmg124h_14.JPG.dat	1212.8	138.8	1.2	0.12	#NUM!	3.2E-04
YFL003C	YFL003C	UWOPS87_ctrl_hmg124h_14.JPG.dat	1241.5	80.3	1.1	0.07	#NUM!	6.5E-05
YFL004W	YFL004W	UWOPS87_ctrl_hmg124h_14.JPG.dat	865.3	122.7	0.8	0.11	#NUM!	7.8E-04
YFL007W	YFL007W	UWOPS87_ctrl_hmg124h_14.JPG.dat	1056.0	133.2	1.0	0.14	#NUM!	7.9E-04
YFL010C	YFL010C	UWOPS87_ctrl_hmg124h_14.JPG.dat	961.5	45.4	0.9	0.05	#NUM!	4.6E-05
YFL010W-/	YFL010W-A	UWOPS87_ctrl_hmg124h_14.JPG.dat	1193.0	118.7	1.0	0.10	#NUM!	2.4E-04
YFL012W	YFL012W	UWOPS87_ctrl_hmg124h_14.JPG.dat	1339.5	26.9	1.1	0.03	#NUM!	6.0E-06
YFL013C	YFL013C	UWOPS87_ctrl_hmg124h_14.JPG.dat	981.5	134.8	0.8	0.03	#NUM!	4.2E-04
YFL033C	YFL033C	UWOPS87_ctrl_hmg124h_14.JPG.dat	1281.8	110.1	1.4	0.03	#NUM!	1.2E-04
YFL063W	YFL063W	UWOPS87_ctrl_hmg124h_14.JPG.dat	1229.0	112.9	1.2	0.03	#NUM!	1.4E-04
YGL029W	YGL029W	UWOPS87_ctrl_hmg124h_14.JPG.dat	1007.0	83.7	0.9	0.10	#NUM!	3.3E-04
YGL037C	YGL037C	UWOPS87_ctrl_hmg124h_14.JPG.dat	1221.0	68.4	1.1	0.02	#NUM!	1.0E-04
YGL042C	YGL042C	UWOPS87_ctrl_hmg124h_14.JPG.dat	1123.3	90.6	1.0	0.09	#NUM!	2.2E-04
YGL049C	YGL049C	UWOPS87_ctrl_hmg124h_14.JPG.dat	1166.0	157.8	1.1	0.15	#NUM!	7.5E-04
YGL063W	YGL063W	UWOPS87_ctrl_hmg124h_14.JPG.dat	1060.0	29.9	1.0	0.01	#NUM!	2.8E-05
YGL071W	YGL071W	UWOPS87_ctrl_hmg124h_14.JPG.dat	1104.0	28.6	1.0	0.03	#NUM!	6.2E-06
YGL094C	YGL094C	UWOPS87_ctrl_hmg124h_14.JPG.dat	1319.5	56.5	1.1	0.07	#NUM!	6.2E-05
YGL096W	YGL096W	UWOPS87_ctrl_hmg124h_14.JPG.dat	1063.5	250.1	1.0	0.20	#NUM!	2.6E-03
YGR055W	YGR055W	UWOPS87_ctrl_hmg124h_14.JPG.dat	1108.8	151.1	1.1	0.14	#NUM!	5.9E-04
YGR061C	YGR061C	UWOPS87_ctrl_hmg124h_14.JPG.dat	1057.3	250.8	1.0	0.23	#NUM!	3.5E-03

YGR067C	YGR067C	UWOPS87_ctrl_hmg124h_14.JPG.dat	1202.5	135.6	1.1	0.13	#NUM!	4.2E-04
YGR068C	YGR068C	UWOPS87_ctrl_hmg124h_14.JPG.dat	1161.0	140.1	1.1	0.13	#NUM!	4.2E-04
YGR122C-A	YGR122C-A	UWOPS87_ctrl_hmg124h_14.JPG.dat	1170.3	45.4	1.1	0.03	#NUM!	9.8E-06
YGR122W	YGR122W	UWOPS87_ctrl_hmg124h_14.JPG.dat	1161.5	86.0	1.0	0.01	#NUM!	3.5E-05
YGR254W	YGR254W	UWOPS87_ctrl_hmg124h_14.JPG.dat	1030.5	30.9	0.9	0.03	#NUM!	1.3E-05
YGR255C	YGR255C	UWOPS87_ctrl_hmg124h_14.JPG.dat	1260.5	88.5	1.2	0.07	#NUM!	6.7E-05
YGR271W	YGR271W	UWOPS87_ctrl_hmg124h_14.JPG.dat	962.0	42.2	0.9	0.03	#NUM!	7.8E-06
YGR272C	YGR272C	UWOPS87_ctrl_hmg124h_14.JPG.dat	1299.3	62.9	1.2	0.06	#NUM!	4.0E-05
YGR273C	YGR273C	UWOPS87_ctrl_hmg124h_14.JPG.dat	1086.8	140.7	1.0	0.12	#NUM!	5.0E-04
YGR276C	YGR276C	UWOPS87_ctrl_hmg124h_14.JPG.dat	953.5	89.7	0.9	0.07	#NUM!	1.3E-04
YGR289C	YGR289C	UWOPS87_ctrl_hmg124h_14.JPG.dat	1324.0	28.4	1.1	0.02	#NUM!	2.2E-06
YGR291C	YGR291C	UWOPS87_ctrl_hmg124h_14.JPG.dat	1084.8	163.6	1.0	0.13	#NUM!	7.2E-04
YGR292W	YGR292W	UWOPS87_ctrl_hmg124h_14.JPG.dat	1000.3	40.0	0.9	0.02	#NUM!	2.3E-06
YHR008C	YHR008C	UWOPS87_ctrl_hmg124h_14.JPG.dat	690.3	120.1	0.7	0.12	#NUM!	1.5E-03
YIL085C	YIL085C	UWOPS87_ctrl_hmg124h_14.JPG.dat	1051.0	35.9	1.0	0.04	#NUM!	1.5E-05
YIL094C	YIL094C	UWOPS87_ctrl_hmg124h_14.JPG.dat	998.5	56.3	0.9	0.04	#NUM!	2.2E-05
YIL102C	YIL102C	UWOPS87_ctrl_hmg124h_14.JPG.dat	1311.3	58.5	1.3	0.07	#NUM!	4.6E-05
YIL111W	YIL111W	UWOPS87_ctrl_hmg124h_14.JPG.dat	1059.5	182.0	0.9	0.15	#NUM!	1.2E-03
YIL122W	YIL122W	UWOPS87_ctrl_hmg124h_14.JPG.dat	1219.5	74.5	1.1	0.06	#NUM!	5.9E-05
YIL125W	YIL125W	UWOPS87_ctrl_hmg124h_14.JPG.dat	1077.8	84.6	1.0	0.07	#NUM!	1.2E-04
YIL131C	YIL131C	UWOPS87_ctrl_hmg124h_14.JPG.dat	1041.8	71.5	1.0	0.08	#NUM!	1.7E-04
YIL136W	YIL136W	UWOPS87_ctrl_hmg124h_14.JPG.dat	1096.5	126.7	1.0	0.13	#NUM!	6.4E-04
YIL151C	YIL151C	UWOPS87_ctrl_hmg124h_14.JPG.dat	1229.5	30.1	1.0	0.01	#NUM!	1.5E-05
YIL158W	YIL158W	UWOPS87_ctrl_hmg124h_14.JPG.dat	1165.5	74.4	1.0	0.06	#NUM!	7.4E-05
YIR004W	YIR004W	UWOPS87_ctrl_hmg124h_14.JPG.dat	1065.0	152.2	1.0	0.14	#NUM!	8.5E-04
YJL029C	YJL029C	UWOPS87_ctrl_hmg124h_14.JPG.dat	1133.3	107.5	1.0	0.10	#NUM!	2.3E-04
YJL079C	YJL079C	UWOPS87_ctrl_hmg124h_14.JPG.dat	46.3	92.5	0.0	0.00	#NUM!	
YJL082W	YJL082W	UWOPS87_ctrl_hmg124h_14.JPG.dat	952.3	53.9	1.0	0.05	#NUM!	3.8E-05
YJL092W	YJL092W	UWOPS87_ctrl_hmg124h_14.JPG.dat	1079.0	35.3	1.1	0.05	#NUM!	2.1E-05
YJL103C	YJL103C	UWOPS87_ctrl_hmg124h_14.JPG.dat	1125.5	42.0	1.0	0.06	#NUM!	4.1E-05
YJL106W	YJL106W	UWOPS87_ctrl_hmg124h_14.JPG.dat	1158.5	128.6	1.1	0.11	#NUM!	2.7E-04
YJL107C	YJL107C	UWOPS87_ctrl_hmg124h_14.JPG.dat	1004.0	69.8	1.0	0.08	#NUM!	1.3E-04
YJL117W	YJL117W	UWOPS87_ctrl_hmg124h_14.JPG.dat	1015.5	55.3	1.0	0.03	#NUM!	1.1E-05
YJL119C	YJL119C	UWOPS87_ctrl_hmg124h_14.JPG.dat	1268.0	74.1	1.1	0.07	#NUM!	5.2E-05
YJL120W	YJL120W	UWOPS87_ctrl_hmg124h_14.JPG.dat	916.5	26.8	0.8	0.02	#NUM!	6.2E-06
YJL177W	YJL177W	UWOPS87_ctrl_hmg124h_14.JPG.dat	879.3	59.1	0.8	0.02	#NUM!	2.0E-04
YJR034W	YJR034W	UWOPS87_ctrl_hmg124h_14.JPG.dat	1136.8	75.1	1.1	0.02	#NUM!	9.7E-05
YJR039W	YJR039W	UWOPS87_ctrl_hmg124h_14.JPG.dat	902.0	50.4	0.8	0.04	#NUM!	2.7E-05
YJR044C	YJR044C	UWOPS87_ctrl_hmg124h_14.JPG.dat	1400.3	75.0	1.2	0.06	#NUM!	2.7E-05
YJR066W	YJR066W	UWOPS87_ctrl_hmg124h_14.JPG.dat	1337.0	74.8	1.2	0.08	#NUM!	7.9E-05
YKL087C	YKL087C	UWOPS87_ctrl_hmg124h_14.JPG.dat	1293.8	131.5	1.1	0.10	#NUM!	1.8E-04
YKR104W	YKR104W	UWOPS87_ctrl_hmg124h_14.JPG.dat	1015.5	65.2	0.9	0.07	#NUM!	9.4E-05
YLR227C	YLR227C	UWOPS87_ctrl_hmg124h_14.JPG.dat	1052.3	46.4	1.0	0.05	#NUM!	3.7E-05
YLR238W	YLR238W	UWOPS87_ctrl_hmg124h_14.JPG.dat	1187.0	26.2	1.1	0.02	#NUM!	1.4E-06
YLR308W	YLR308W	UWOPS87_ctrl_hmg124h_14.JPG.dat	1012.3	87.9	1.0	0.08	#NUM!	1.7E-04
YLR455W	YLR455W	UWOPS87_ctrl_hmg124h_14.JPG.dat	1247.3	98.1	1.2	0.10	#NUM!	1.4E-04
YML050W	YML050W	UWOPS87_ctrl_hmg124h_14.JPG.dat	1072.0	128.9	1.1	0.13	#NUM!	5.0E-04
YML111W	YML111W	UWOPS87_ctrl_hmg124h_14.JPG.dat	1052.5	97.3	0.9	0.04	#NUM!	5.7E-04
YML112W	YML112W	UWOPS87_ctrl_hmg124h_14.JPG.dat	1182.0	66.4	1.1	0.04	#NUM!	8.8E-06
YML129C	YML129C	UWOPS87_ctrl_hmg124h_14.JPG.dat	728.8	265.7	0.7	0.26	#NUM!	1.3E-02
YMR074C	YMR074C	UWOPS87_ctrl_hmg124h_14.JPG.dat	983.0	20.5	0.9	0.01	#NUM!	2.5E-05
YMR118C	YMR118C	UWOPS87_ctrl_hmg124h_14.JPG.dat	1109.3	199.5	1.0	0.02	#NUM!	1.4E-04
YMR119W	YMR119W	UWOPS87_ctrl_hmg124h_14.JPG.dat	1166.5	83.3	1.0	0.05	#NUM!	3.7E-05
YMR165C	YMR165C	UWOPS87_ctrl_hmg124h_14.JPG.dat	438.0	468.6	0.4	0.46	#NUM!	1.6E-01
YMR194C-A	YMR194C-A	UWOPS87_ctrl_hmg124h_14.JPG.dat	1048.0	74.6	1.0	0.07	#NUM!	1.0E-04
YNL011C	YNL011C	UWOPS87_ctrl_hmg124h_14.JPG.dat	1100.5	44.9	1.1	0.01	#NUM!	2.3E-05
YNL014W	YNL014W	UWOPS87_ctrl_hmg124h_14.JPG.dat	911.8	67.2	0.8	0.02	#NUM!	1.4E-04
YNL042W	YNL042W	UWOPS87_ctrl_hmg124h_14.JPG.dat	1132.3	50.6	1.0	0.02	#NUM!	7.2E-05
YNL047C	YNL047C	UWOPS87_ctrl_hmg124h_14.JPG.dat	1057.5	94.1	1.0	0.09	#NUM!	1.9E-04
YNL053W	YNL053W	UWOPS87_ctrl_hmg124h_14.JPG.dat	946.8	67.0	0.9	0.06	#NUM!	9.2E-05
YNL086W	YNL086W	UWOPS87_ctrl_hmg124h_14.JPG.dat	1121.3	38.6	1.0	0.04	#NUM!	1.4E-05
YNL096C	YNL096C	UWOPS87_ctrl_hmg124h_14.JPG.dat	996.0	193.6	0.9	0.18	#NUM!	1.8E-03
YNL109W	YNL109W	UWOPS87_ctrl_hmg124h_14.JPG.dat	934.5	133.1	0.8	0.03	#NUM!	5.4E-04
YNL111C	YNL111C	UWOPS87_ctrl_hmg124h_14.JPG.dat	1086.5	107.8	1.0	0.04	#NUM!	4.8E-04
YNL147W	YNL147W	UWOPS87_ctrl_hmg124h_14.JPG.dat	1017.8	325.8	0.8	0.02	#NUM!	2.2E-04
YNL180C	YNL180C	UWOPS87_ctrl_hmg124h_14.JPG.dat	1073.8	158.1	0.9	0.03	#NUM!	3.0E-04
YNL284C	YNL284C	UWOPS87_ctrl_hmg124h_14.JPG.dat	1258.5	35.2	1.1	0.06	#NUM!	3.9E-05
YNR033W	YNR033W	UWOPS87_ctrl_hmg124h_14.JPG.dat	1091.5	47.0	1.0	0.06	#NUM!	5.9E-05
YNR041C	YNR041C	UWOPS87_ctrl_hmg124h_14.JPG.dat	1001.8	180.9	1.0	0.15	#NUM!	1.1E-03
YNR044W	YNR044W	UWOPS87_ctrl_hmg124h_14.JPG.dat	641.5	740.8	0.5	0.62	#NUM!	1.8E-01

YOL073C	YOL073C	UWOPS87_ctrl_hmg124h_14.JPG.dat	1131.3	19.1	1.0	0.03	#NUM!	7.4E-06
YOL086C	YOL086C	UWOPS87_ctrl_hmg124h_14.JPG.dat	861.5	81.8	0.9	0.07	#NUM!	1.8E-04
YOL153C	YOL153C	UWOPS87_ctrl_hmg124h_14.JPG.dat	935.3	132.2	0.8	0.10	#NUM!	4.5E-04
YOR054C	YOR054C	UWOPS87_ctrl_hmg124h_14.JPG.dat	1189.8	57.2	1.0	0.02	#NUM!	9.2E-05
YOR125C	YOR125C	UWOPS87_ctrl_hmg124h_14.JPG.dat	1184.3	106.3	1.0	0.07	#NUM!	9.2E-05
YOR128C	YOR128C	UWOPS87_ctrl_hmg124h_14.JPG.dat	1054.3	76.2	1.0	0.02	#NUM!	1.4E-04
YOR155C	YOR155C	UWOPS87_ctrl_hmg124h_14.JPG.dat	1167.3	178.0	1.2	0.04	#NUM!	3.9E-04
YOR158W	YOR158W	UWOPS87_ctrl_hmg124h_14.JPG.dat	1100.3	56.9	1.1	0.05	#NUM!	2.7E-05
YOR179C	YOR179C	UWOPS87_ctrl_hmg124h_14.JPG.dat	995.8	97.9	1.0	0.01	#NUM!	6.6E-05
YOR180C	YOR180C	UWOPS87_ctrl_hmg124h_14.JPG.dat	1080.8	42.0	1.0	0.05	#NUM!	3.4E-05
YOR202W	YOR202W	UWOPS87_ctrl_hmg124h_14.JPG.dat	1178.8	162.7	1.0	0.10	#NUM!	1.3E-290
YOR220W	YOR220W	UWOPS87_ctrl_hmg124h_14.JPG.dat	1350.5	21.3	1.3	0.02	#NUM!	1.6E-06
YOR240W	YOR240W	UWOPS87_ctrl_hmg124h_14.JPG.dat	959.8	65.1	0.9	0.06	#NUM!	7.8E-05
YOR248W	YOR248W	UWOPS87_ctrl_hmg124h_14.JPG.dat	964.3	97.6	0.9	0.07	#NUM!	1.4E-04
YOR300W	YOR300W	UWOPS87_ctrl_hmg124h_14.JPG.dat	1086.5	248.0	1.0	0.25	#NUM!	3.6E-03
YOR306C	YOR306C	UWOPS87_ctrl_hmg124h_14.JPG.dat	1051.0	126.7	1.0	0.12	#NUM!	4.8E-04
YOR309C	YOR309C	UWOPS87_ctrl_hmg124h_14.JPG.dat	911.3	71.4	0.9	0.02	#NUM!	1.2E-04
YOR325W	YOR325W	UWOPS87_ctrl_hmg124h_14.JPG.dat	1177.5	28.2	1.0	0.01	#NUM!	1.4E-05
YOR333C	YOR333C	UWOPS87_ctrl_hmg124h_14.JPG.dat	1132.0	143.4	1.0	0.12	#NUM!	5.5E-04
YOR345C	YOR345C	UWOPS87_ctrl_hmg124h_14.JPG.dat	1312.8	63.2	1.2	0.01	#NUM!	4.0E-05
YOR366W	YOR366W	UWOPS87_ctrl_hmg124h_14.JPG.dat	989.3	53.9	0.9	0.02	#NUM!	1.3E-04
YOR379C	YOR379C	UWOPS87_ctrl_hmg124h_14.JPG.dat	1267.3	28.9	1.1	0.04	#NUM!	1.1E-05
YPL015C	YPL015C	UWOPS87_ctrl_hmg124h_14.JPG.dat	1283.5	120.0	1.2	0.12	#NUM!	2.5E-04
YPL035C	YPL035C	UWOPS87_ctrl_hmg124h_14.JPG.dat	1135.8	52.0	1.0	0.05	#NUM!	3.3E-05
YPL072W	YPL072W	UWOPS87_ctrl_hmg124h_14.JPG.dat	440.5	512.5	0.4	0.46	#NUM!	1.8E-01
YPL158C	YPL158C	UWOPS87_ctrl_hmg124h_14.JPG.dat	1136.3	44.0	1.0	0.04	#NUM!	1.7E-05
YPL261C	YPL261C	UWOPS87_ctrl_hmg124h_14.JPG.dat	1039.5	34.9	1.0	0.01	#NUM!	1.6E-05
YPL262W	YPL262W	UWOPS87_ctrl_hmg124h_14.JPG.dat	1208.3	80.9	1.1	0.02	#NUM!	1.6E-04
YPL263C	YPL263C	UWOPS87_ctrl_hmg124h_14.JPG.dat	883.3	45.7	0.9	0.04	#NUM!	3.1E-05
YPL264C	YPL264C	UWOPS87_ctrl_hmg124h_14.JPG.dat	1117.3	115.4	1.0	0.12	#NUM!	4.3E-04
YPL265W	YPL265W	UWOPS87_ctrl_hmg124h_14.JPG.dat	1178.8	114.0	1.1	0.10	#NUM!	2.3E-04
YPL267W	YPL267W	UWOPS87_ctrl_hmg124h_14.JPG.dat	1030.8	81.1	1.0	0.08	#NUM!	1.6E-04
YPL269W	YPL269W	UWOPS87_ctrl_hmg124h_14.JPG.dat	1266.3	54.3	1.1	0.05	#NUM!	2.3E-05
YPL270W	YPL270W	UWOPS87_ctrl_hmg124h_14.JPG.dat	1261.5	38.2	1.1	0.03	#NUM!	6.0E-06
YPL272C	YPL272C	UWOPS87_ctrl_hmg124h_14.JPG.dat	1240.8	167.6	1.1	0.02	#NUM!	8.9E-05
YPL273W	YPL273W	UWOPS87_ctrl_hmg124h_14.JPG.dat	1255.5	6.2	1.1	0.02	#NUM!	2.1E-06
YPL274W	YPL274W	UWOPS87_ctrl_hmg124h_14.JPG.dat	531.0	602.9	0.5	0.57	#NUM!	1.8E-01
YPR001W	YPR001W	UWOPS87_ctrl_hmg124h_14.JPG.dat	986.8	46.6	1.0	0.05	#NUM!	3.8E-05
YPR002W	YPR002W	UWOPS87_ctrl_hmg124h_14.JPG.dat	1165.5	79.2	1.1	0.08	#NUM!	1.0E-04
YPR003C	YPR003C	UWOPS87_ctrl_hmg124h_14.JPG.dat	965.0	63.3	1.0	0.06	#NUM!	5.0E-05
YPR004C	YPR004C	UWOPS87_ctrl_hmg124h_14.JPG.dat	1219.3	52.3	1.2	0.07	#NUM!	5.2E-05
YPR005C	YPR005C	UWOPS87_ctrl_hmg124h_14.JPG.dat	974.5	27.3	0.9	0.01	#NUM!	3.9E-05
YPR006C	YPR006C	UWOPS87_ctrl_hmg124h_14.JPG.dat	922.8	110.1	0.9	0.10	#NUM!	4.3E-04
YPR007C	YPR007C	UWOPS87_ctrl_hmg124h_14.JPG.dat	1170.5	97.3	1.0	0.09	#NUM!	1.9E-04
YPR008W	YPR008W	UWOPS87_ctrl_hmg124h_14.JPG.dat	957.0	25.8	0.9	0.03	#NUM!	8.4E-06
YPR009W	YPR009W	UWOPS87_ctrl_hmg124h_14.JPG.dat	1252.0	41.8	1.1	0.03	#NUM!	6.9E-06
YPR011C	YPR011C	UWOPS87_ctrl_hmg124h_14.JPG.dat	1281.3	26.7	1.2	0.03	#NUM!	4.8E-06
YPR012W	YPR012W	UWOPS87_ctrl_hmg124h_14.JPG.dat	1019.8	170.5	0.9	0.14	#NUM!	9.8E-04
YPR013C	YPR013C	UWOPS87_ctrl_hmg124h_14.JPG.dat	801.8	55.2	0.8	0.06	#NUM!	9.9E-05
YPR014C	YPR014C	UWOPS87_ctrl_hmg124h_14.JPG.dat	1262.8	99.8	1.2	0.09	#NUM!	1.3E-04
YPR015C	YPR015C	UWOPS87_ctrl_hmg124h_14.JPG.dat	1353.5	99.6	1.2	0.09	#NUM!	9.7E-05
YPR017C	YPR017C	UWOPS87_ctrl_hmg124h_14.JPG.dat	1042.3	72.5	1.0	0.06	#NUM!	7.1E-05
YPR018W	YPR018W	UWOPS87_ctrl_hmg124h_14.JPG.dat	917.5	12.9	0.8	0.02	#NUM!	2.3E-06
YPR020W	YPR020W	UWOPS87_ctrl_hmg124h_14.JPG.dat	1063.8	76.2	0.9	0.07	#NUM!	1.0E-04
YPR021C	YPR021C	UWOPS87_ctrl_hmg124h_14.JPG.dat	800.3	41.5	0.8	0.03	#NUM!	2.4E-05
YPR022C	YPR022C	UWOPS87_ctrl_hmg124h_14.JPG.dat	916.5	212.8	0.8	0.19	#NUM!	3.4E-03
YPR023C	YPR023C	UWOPS87_ctrl_hmg124h_14.JPG.dat	949.5	63.5	0.8	0.05	#NUM!	6.4E-05
YPR024W	YPR024W	UWOPS87_ctrl_hmg124h_14.JPG.dat	1237.0	151.1	1.1	0.12	#NUM!	3.7E-04
YPR026W	YPR026W	UWOPS87_ctrl_hmg124h_14.JPG.dat	1383.8	81.5	1.1	0.02	#NUM!	1.3E-04
YPR027C	YPR027C	UWOPS87_ctrl_hmg124h_14.JPG.dat	1233.3	38.9	1.1	0.02	#NUM!	9.3E-05
YPR028W	YPR028W	UWOPS87_ctrl_hmg124h_14.JPG.dat	904.8	27.3	0.9	0.01	#NUM!	2.0E-05
YPR029C	YPR029C	UWOPS87_ctrl_hmg124h_14.JPG.dat	957.3	123.9	0.9	0.12	#NUM!	5.7E-04
YPR030W	YPR030W	UWOPS87_ctrl_hmg124h_14.JPG.dat	1096.8	129.7	1.0	0.12	#NUM!	4.4E-04
YPR031W	YPR031W	UWOPS87_ctrl_hmg124h_14.JPG.dat	1022.8	189.2	1.0	0.19	#NUM!	2.0E-03
YPR032W	YPR032W	UWOPS87_ctrl_hmg124h_14.JPG.dat	1136.8	53.0	1.0	0.04	#NUM!	1.1E-05
YPR037C	YPR037C	UWOPS87_ctrl_hmg124h_14.JPG.dat	1179.5	93.3	1.0	0.03	#NUM!	2.8E-04
YPR038W	YPR038W	UWOPS87_ctrl_hmg124h_14.JPG.dat	915.3	89.5	0.9	0.09	#NUM!	3.1E-04
YPR039W	YPR039W	UWOPS87_ctrl_hmg124h_14.JPG.dat	1104.5	99.3	1.0	0.04	#NUM!	4.8E-04
YPR040W	YPR040W	UWOPS87_ctrl_hmg124h_14.JPG.dat	955.8	522.2	1.1	0.06	#NUM!	1.1E-03
YPR042C	YPR042C	UWOPS87_ctrl_hmg124h_14.JPG.dat	1011.5	95.2	0.9	0.09	#NUM!	2.4E-04

YPR043W	YPR043W	UWOPS87_ctrl_hmg124h_14.JPG.dat	1013.5	50.4	0.9	0.05	#NUM!	4.8E-05
YPR044C	YPR044C	UWOPS87_ctrl_hmg124h_14.JPG.dat	1104.3	47.4	1.0	0.05	#NUM!	2.2E-05
YPR045C	YPR045C	UWOPS87_ctrl_hmg124h_14.JPG.dat	959.5	58.5	1.0	0.06	#NUM!	6.1E-05
YPR046W	YPR046W	UWOPS87_ctrl_hmg124h_14.JPG.dat	946.3	50.3	0.9	0.05	#NUM!	4.7E-05
YPR050C	YPR050C	UWOPS87_ctrl_hmg124h_14.JPG.dat	1141.5	30.2	1.1	0.01	#NUM!	3.2E-05
YPR051W	YPR051W	UWOPS87_ctrl_hmg124h_14.JPG.dat	1041.8	105.7	1.0	0.10	#NUM!	2.6E-04
YPR052C	YPR052C	UWOPS87_ctrl_hmg124h_14.JPG.dat	1041.5	70.3	1.0	0.07	#NUM!	1.2E-04
YPR053C	YPR053C	UWOPS87_ctrl_hmg124h_14.JPG.dat	669.0	723.7	0.6	0.65	#NUM!	1.6E-01
YPR054W	YPR054W	UWOPS87_ctrl_hmg124h_14.JPG.dat	780.3	536.7	1.0	0.15	#NUM!	8.6E-03
YPR057W	YPR057W	UWOPS87_ctrl_hmg124h_14.JPG.dat	1269.0	30.7	1.1	0.03	#NUM!	6.1E-06
YPR058W	YPR058W	UWOPS87_ctrl_hmg124h_14.JPG.dat	1069.8	161.4	1.0	0.14	#NUM!	8.0E-04
YPR059C	YPR059C	UWOPS87_ctrl_hmg124h_14.JPG.dat	1272.8	119.3	1.2	0.02	#NUM!	6.1E-05
YPR060C	YPR060C	UWOPS87_ctrl_hmg124h_14.JPG.dat	991.8	95.8	1.0	0.10	#NUM!	2.8E-04
YPR061C	YPR061C	UWOPS87_ctrl_hmg124h_14.JPG.dat	1007.3	57.5	1.0	0.05	#NUM!	3.1E-05
YPR062W	YPR062W	UWOPS87_ctrl_hmg124h_14.JPG.dat	838.5	40.4	0.9	0.03	#NUM!	6.7E-06
YPR063C	YPR063C	UWOPS87_ctrl_hmg124h_14.JPG.dat	984.8	133.5	1.0	0.13	#NUM!	6.1E-04
YPR064W	YPR064W	UWOPS87_ctrl_hmg124h_14.JPG.dat	823.5	27.9	0.8	0.02	#NUM!	1.3E-04
YPR065W	YPR065W	UWOPS87_ctrl_hmg124h_14.JPG.dat	1174.8	71.2	1.1	0.01	#NUM!	3.9E-05
YPR066W	YPR066W	UWOPS87_ctrl_hmg124h_14.JPG.dat	940.8	37.0	0.9	0.01	#NUM!	1.2E-06
YPR068C	YPR068C	UWOPS87_ctrl_hmg124h_14.JPG.dat	1076.0	153.8	1.0	0.15	#NUM!	7.9E-04
YPR069C	YPR069C	UWOPS87_ctrl_hmg124h_14.JPG.dat	1224.8	30.3	1.1	0.03	#NUM!	3.2E-06
YPR070W	YPR070W	UWOPS87_ctrl_hmg124h_14.JPG.dat	978.8	113.3	1.0	0.12	#NUM!	4.5E-04
YPR071W	YPR071W	UWOPS87_ctrl_hmg124h_14.JPG.dat	430.5	589.5	0.4	0.57	#NUM!	2.4E-01
YPR073C	YPR073C	UWOPS87_ctrl_hmg124h_14.JPG.dat	988.0	44.2	1.0	0.07	#NUM!	7.2E-05
YPR074C	YPR074C	UWOPS87_ctrl_hmg124h_14.JPG.dat	955.3	108.2	0.9	0.02	#NUM!	1.5E-04
YPR075C	YPR075C	UWOPS87_ctrl_hmg124h_14.JPG.dat	980.3	36.3	1.0	0.04	#NUM!	2.3E-05
YPR076W	YPR076W	UWOPS87_ctrl_hmg124h_14.JPG.dat	1057.3	38.8	1.0	0.03	#NUM!	9.2E-06
YPR077C	YPR077C	UWOPS87_ctrl_hmg124h_14.JPG.dat	935.8	57.2	0.9	0.06	#NUM!	9.0E-05
YPR078C	YPR078C	UWOPS87_ctrl_hmg124h_14.JPG.dat	1274.3	34.6	1.2	0.04	#NUM!	1.1E-05
YPR079W	YPR079W	UWOPS87_ctrl_hmg124h_14.JPG.dat	1031.5	142.6	0.9	0.12	#NUM!	5.7E-04
YPR083W	YPR083W	UWOPS87_ctrl_hmg124h_14.JPG.dat	869.0	63.2	0.8	0.01	#NUM!	3.7E-05
YPR084W	YPR084W	UWOPS87_ctrl_hmg124h_14.JPG.dat	948.0	17.3	0.9	0.02	#NUM!	4.1E-06
YPR089W	YPR089W	UWOPS87_ctrl_hmg124h_14.JPG.dat	1160.8	102.1	1.1	0.08	#NUM!	1.4E-04
YPR090W	YPR090W	UWOPS87_ctrl_hmg124h_14.JPG.dat	929.5	42.6	0.9	0.04	#NUM!	2.4E-05
YPR091C	YPR091C	UWOPS87_ctrl_hmg124h_14.JPG.dat	994.5	59.4	1.0	0.06	#NUM!	5.6E-05
YPR092W	YPR092W	UWOPS87_ctrl_hmg124h_14.JPG.dat	1089.5	77.3	1.0	0.07	#NUM!	6.9E-05
YPR093C	YPR093C	UWOPS87_ctrl_hmg124h_14.JPG.dat	992.3	41.5	1.0	0.04	#NUM!	1.3E-05
YPR095C	YPR095C	UWOPS87_ctrl_hmg124h_14.JPG.dat	1103.5	66.3	1.0	0.02	#NUM!	1.3E-04
YPR096C	YPR096C	UWOPS87_ctrl_hmg124h_14.JPG.dat	1065.0	30.7	1.0	0.01	#NUM!	8.2E-06
YPR097W	YPR097W	UWOPS87_ctrl_hmg124h_14.JPG.dat	947.8	23.0	0.9	0.02	#NUM!	2.2E-06
YPR098C	YPR098C	UWOPS87_ctrl_hmg124h_14.JPG.dat	892.8	69.5	0.8	0.06	#NUM!	1.2E-04
YPR106W	YPR106W	UWOPS87_ctrl_hmg124h_14.JPG.dat	888.5	54.3	0.9	0.04	#NUM!	3.3E-05
YPR109W	YPR109W	UWOPS87_ctrl_hmg124h_14.JPG.dat	931.5	83.7	0.8	0.07	#NUM!	1.9E-04
YPR111W	YPR111W	UWOPS87_ctrl_hmg124h_14.JPG.dat	1224.3	225.1	1.2	0.06	#NUM!	7.4E-04
YPR114W	YPR114W	UWOPS87_ctrl_hmg124h_14.JPG.dat	918.8	51.7	0.9	0.07	#NUM!	1.2E-04
YPR115W	YPR115W	UWOPS87_ctrl_hmg124h_14.JPG.dat	1286.8	30.3	1.2	0.00	#NUM!	2.1E-06
YPR117W	YPR117W	UWOPS87_ctrl_hmg124h_14.JPG.dat	1182.0	109.4	1.0	0.02	#NUM!	9.7E-05
YPR118W	YPR118W	UWOPS87_ctrl_hmg124h_14.JPG.dat	1097.3	107.3	1.0	0.03	#NUM!	2.5E-04
YPR119W	YPR119W	UWOPS87_ctrl_hmg124h_14.JPG.dat	1080.5	129.1	0.9	0.11	#NUM!	4.6E-04
YPR120C	YPR120C	UWOPS87_ctrl_hmg124h_14.JPG.dat	1359.3	50.1	1.2	0.05	#NUM!	2.4E-05
YPR121W	YPR121W	UWOPS87_ctrl_hmg124h_14.JPG.dat	911.0	26.0	0.9	0.02	#NUM!	5.5E-06
YPR122W	YPR122W	UWOPS87_ctrl_hmg124h_14.JPG.dat	1130.0	153.3	1.1	0.14	#NUM!	5.3E-04
YPR123C	YPR123C	UWOPS87_ctrl_hmg124h_14.JPG.dat	928.5	20.2	0.9	0.03	#NUM!	6.5E-06
YPR125W	YPR125W	UWOPS87_ctrl_hmg124h_14.JPG.dat	973.5	84.9	0.9	0.03	#NUM!	2.6E-04
YPR126C	YPR126C	UWOPS87_ctrl_hmg124h_14.JPG.dat	1219.5	80.0	1.1	0.08	#NUM!	9.7E-05
YPR127W	YPR127W	UWOPS87_ctrl_hmg124h_14.JPG.dat	1234.3	118.2	1.1	0.04	#NUM!	5.0E-04
YPR128C	YPR128C	UWOPS87_ctrl_hmg124h_14.JPG.dat	1182.0	23.1	1.1	0.02	#NUM!	1.3E-06
YPR129W	YPR129W	UWOPS87_ctrl_hmg124h_14.JPG.dat	1129.5	97.5	1.1	0.09	#NUM!	1.6E-04
YPR130C	YPR130C	UWOPS87_ctrl_hmg124h_14.JPG.dat	1148.3	70.5	1.0	0.02	#NUM!	1.1E-04
YPR132W	YPR132W	UWOPS87_ctrl_hmg124h_14.JPG.dat	1034.5	128.1	0.9	0.03	#NUM!	3.8E-04
YPR134W	YPR134W	UWOPS87_ctrl_hmg124h_14.JPG.dat	1147.0	85.1	1.1	0.08	#NUM!	1.1E-04
YPR135W	YPR135W	UWOPS87_ctrl_hmg124h_14.JPG.dat	991.8	57.4	0.9	0.05	#NUM!	5.4E-05
YPR138C	YPR138C	UWOPS87_ctrl_hmg124h_14.JPG.dat	1201.3	158.2	1.1	0.15	#NUM!	6.3E-04
YPR140W	YPR140W	UWOPS87_ctrl_hmg124h_14.JPG.dat	1231.3	81.5	1.2	0.03	#NUM!	1.7E-04
YPR141C	YPR141C	UWOPS87_ctrl_hmg124h_14.JPG.dat	952.5	48.9	0.9	0.05	#NUM!	5.2E-05
YPR145W	YPR145W	UWOPS87_ctrl_hmg124h_14.JPG.dat	1197.8	52.2	1.0	0.04	#NUM!	2.0E-05
YPR146C	YPR146C	UWOPS87_ctrl_hmg124h_14.JPG.dat	987.8	669.6	1.1	0.12	#NUM!	3.8E-03
YPR147C	YPR147C	UWOPS87_ctrl_hmg124h_14.JPG.dat	1203.0	32.3	1.1	0.02	#NUM!	2.9E-06
YPR148C	YPR148C	UWOPS87_ctrl_hmg124h_14.JPG.dat	1106.8	82.3	1.0	0.01	#NUM!	4.6E-05
YPR149W	YPR149W	UWOPS87_ctrl_hmg124h_14.JPG.dat	1107.8	47.3	0.9	0.03	#NUM!	1.4E-05

YPR150W	YPR150W	UWOPS87_ctrl_hmg124h_14.JPG.dat	1373.3	75.1	1.1	0.07	#NUM!	6.3E-05
YPR151C	YPR151C	UWOPS87_ctrl_hmg124h_14.JPG.dat	1292.3	166.4	1.3	0.17	#NUM!	6.6E-04
YPR152C	YPR152C	UWOPS87_ctrl_hmg124h_14.JPG.dat	1191.0	208.5	1.1	0.19	#NUM!	1.4E-03
YPR153W	YPR153W	UWOPS87_ctrl_hmg124h_14.JPG.dat	1182.0	69.0	1.2	0.06	#NUM!	3.4E-05
YPR154W	YPR154W	UWOPS87_ctrl_hmg124h_14.JPG.dat	1254.0	95.7	1.2	0.09	#NUM!	1.1E-04
YPR155C	YPR155C	UWOPS87_ctrl_hmg124h_14.JPG.dat	1070.8	71.5	1.0	0.07	#NUM!	8.2E-05
YPR156C	YPR156C	UWOPS87_ctrl_hmg124h_14.JPG.dat	1039.5	91.1	1.0	0.08	#NUM!	1.4E-04
YPR157W	YPR157W	UWOPS87_ctrl_hmg124h_14.JPG.dat	1168.8	57.4	1.0	0.05	#NUM!	3.0E-05
YPR158W	YPR158W	UWOPS87_ctrl_hmg124h_14.JPG.dat	1139.5	77.5	1.0	0.07	#NUM!	7.2E-05
YPR160W	YPR160W	UWOPS87_ctrl_hmg124h_14.JPG.dat	968.3	645.7	1.2	0.01	#NUM!	9.0E-06
YPR164W	YPR164W	UWOPS87_ctrl_hmg124h_14.JPG.dat	1164.3	35.9	1.1	0.01	#NUM!	1.7E-05
YPR167C	YPR167C	UWOPS87_ctrl_hmg124h_14.JPG.dat	983.8	55.7	0.9	0.06	#NUM!	8.3E-05
YPR170C	YPR170C	UWOPS87_ctrl_hmg124h_14.JPG.dat	981.3	81.6	0.9	0.07	#NUM!	1.3E-04
YPR171W	YPR171W	UWOPS87_ctrl_hmg124h_14.JPG.dat	1045.5	96.0	1.0	0.10	#NUM!	2.5E-04
YPR172W	YPR172W	UWOPS87_ctrl_hmg124h_14.JPG.dat	945.0	31.0	1.0	0.03	#NUM!	7.2E-06
YPR173C	YPR173C	UWOPS87_ctrl_hmg124h_14.JPG.dat	980.5	221.4	0.9	0.20	#NUM!	2.7E-03
YPR174C	YPR174C	UWOPS87_ctrl_hmg124h_14.JPG.dat	1103.0	93.5	1.1	0.02	#NUM!	9.5E-05
YPR179C	YPR179C	UWOPS87_ctrl_hmg124h_14.JPG.dat	921.5	108.8	0.9	0.04	#NUM!	5.5E-04
YPR184W	YPR184W	UWOPS87_ctrl_hmg124h_14.JPG.dat	1189.5	93.9	1.0	0.08	#NUM!	1.5E-04
YPR185W	YPR185W	UWOPS87_ctrl_hmg124h_14.JPG.dat	637.3	590.8	0.6	0.52	#NUM!	1.2E-01
YPR188C	YPR188C	UWOPS87_ctrl_hmg124h_14.JPG.dat	1021.3	34.6	0.9	0.03	#NUM!	9.5E-06
YPR189W	YPR189W	UWOPS87_ctrl_hmg124h_14.JPG.dat	1069.3	50.8	1.0	0.01	#NUM!	2.4E-05
YPR191W	YPR191W	UWOPS87_ctrl_hmg124h_14.JPG.dat	1310.8	70.5	1.1	0.02	#NUM!	9.3E-05
YPR192W	YPR192W	UWOPS87_ctrl_hmg124h_14.JPG.dat	1086.3	191.8	1.0	0.18	#NUM!	1.6E-03
YPR193C	YPR193C	UWOPS87_ctrl_hmg124h_14.JPG.dat	1029.0	171.2	1.0	0.17	#NUM!	1.2E-03
YPR194C	YPR194C	UWOPS87_ctrl_hmg124h_14.JPG.dat	866.0	41.9	0.9	0.05	#NUM!	5.6E-05
YPR195C	YPR195C	UWOPS87_ctrl_hmg124h_14.JPG.dat	887.3	38.4	0.9	0.05	#NUM!	4.7E-05
YPR196W	YPR196W	UWOPS87_ctrl_hmg124h_14.JPG.dat	1127.8	60.1	1.1	0.08	#NUM!	8.8E-05
YPR197C	YPR197C	UWOPS87_ctrl_hmg124h_14.JPG.dat	997.8	93.4	1.0	0.09	#NUM!	1.9E-04
YPR198W	YPR198W	UWOPS87_ctrl_hmg124h_14.JPG.dat	1190.0	132.6	1.1	0.03	#NUM!	3.0E-04
YPR199C	YPR199C	UWOPS87_ctrl_hmg124h_14.JPG.dat	1155.8	99.8	1.1	0.08	#NUM!	9.9E-05
YPR200C	YPR200C	UWOPS87_ctrl_hmg124h_14.JPG.dat	981.0	98.7	1.0	0.09	#NUM!	2.3E-04
YPR201W	YPR201W	UWOPS87_ctrl_hmg124h_14.JPG.dat	1195.8	29.7	1.2	0.05	#NUM!	2.2E-05
1	1	UWOPS87_ctrl_hmg124h_2.JPG.dat	1604.8	233.6	1.0	0.07	#NUM!	9.9E-05
2	2	UWOPS87_ctrl_hmg124h_2.JPG.dat	1359.3	243.8	1.1	0.13	#NUM!	4.9E-04
3	3	UWOPS87_ctrl_hmg124h_2.JPG.dat	1281.8	144.9	1.0	0.06	#NUM!	7.1E-05
4	4	UWOPS87_ctrl_hmg124h_2.JPG.dat	1239.8	237.9	0.9	0.14	#NUM!	9.0E-04
YBR224W	YBR224W	UWOPS87_ctrl_hmg124h_2.JPG.dat	1215.3	77.4	1.1	0.03	#NUM!	2.0E-04
YBR225W	YBR225W	UWOPS87_ctrl_hmg124h_2.JPG.dat	1088.8	130.3	1.0	0.11	#NUM!	4.5E-04
YBR226C	YBR226C	UWOPS87_ctrl_hmg124h_2.JPG.dat	1219.5	105.3	1.1	0.10	#NUM!	1.8E-04
YBR227C	YBR227C	UWOPS87_ctrl_hmg124h_2.JPG.dat	1171.0	138.6	1.0	0.04	#NUM!	5.2E-04
YBR228W	YBR228W	UWOPS87_ctrl_hmg124h_2.JPG.dat	1255.8	51.6	1.1	0.05	#NUM!	2.2E-05
YBR229C	YBR229C	UWOPS87_ctrl_hmg124h_2.JPG.dat	1323.0	15.5	1.1	0.01	#NUM!	1.7E-07
YBR230C	YBR230C	UWOPS87_ctrl_hmg124h_2.JPG.dat	1145.0	235.2	0.9	0.03	#NUM!	5.1E-04
YBR231C	YBR231C	UWOPS87_ctrl_hmg124h_2.JPG.dat	1077.8	32.2	0.9	0.03	#NUM!	7.2E-06
YBR232C	YBR232C	UWOPS87_ctrl_hmg124h_2.JPG.dat	1212.5	123.0	1.1	0.10	#NUM!	2.5E-04
YBR233W	YBR233W	UWOPS87_ctrl_hmg124h_2.JPG.dat	1054.3	146.7	0.9	0.02	#NUM!	1.0E-04
YBR235W	YBR235W	UWOPS87_ctrl_hmg124h_2.JPG.dat	1217.5	169.8	1.0	0.01	#NUM!	4.0E-05
YBR238C	YBR238C	UWOPS87_ctrl_hmg124h_2.JPG.dat	1060.0	24.4	1.0	0.02	#NUM!	4.7E-06
YBR239C	YBR239C	UWOPS87_ctrl_hmg124h_2.JPG.dat	1147.0	28.9	1.0	0.02	#NUM!	3.6E-06
YBR240C	YBR240C	UWOPS87_ctrl_hmg124h_2.JPG.dat	1007.8	34.4	1.0	0.03	#NUM!	1.1E-05
YBR241C	YBR241C	UWOPS87_ctrl_hmg124h_2.JPG.dat	1096.0	110.8	1.0	0.10	#NUM!	2.6E-04
YBR242W	YBR242W	UWOPS87_ctrl_hmg124h_2.JPG.dat	1119.0	120.8	1.0	0.11	#NUM!	3.9E-04
YBR244W	YBR244W	UWOPS87_ctrl_hmg124h_2.JPG.dat	943.8	36.8	0.8	0.03	#NUM!	1.4E-05
YBR245C	YBR245C	UWOPS87_ctrl_hmg124h_2.JPG.dat	1274.3	82.4	1.1	0.07	#NUM!	6.4E-05
YBR246W	YBR246W	UWOPS87_ctrl_hmg124h_2.JPG.dat	1238.0	74.5	1.1	0.06	#NUM!	5.5E-05
YBR248C	YBR248C	UWOPS87_ctrl_hmg124h_2.JPG.dat	1280.5	26.8	1.1	0.03	#NUM!	4.5E-06
YBR249C	YBR249C	UWOPS87_ctrl_hmg124h_2.JPG.dat	1361.5	134.7	1.2	0.12	#NUM!	3.0E-04
YBR250W	YBR250W	UWOPS87_ctrl_hmg124h_2.JPG.dat	1171.3	71.0	1.0	0.07	#NUM!	1.0E-04
YBR255W	YBR255W	UWOPS87_ctrl_hmg124h_2.JPG.dat	1161.0	115.0	1.1	0.11	#NUM!	3.0E-04
YBR258C	YBR258C	UWOPS87_ctrl_hmg124h_2.JPG.dat	1105.5	24.5	1.0	0.01	#NUM!	1.1E-05
YBR259W	YBR259W	UWOPS87_ctrl_hmg124h_2.JPG.dat	1246.0	67.0	1.2	0.07	#NUM!	5.6E-05
YBR260C	YBR260C	UWOPS87_ctrl_hmg124h_2.JPG.dat	1232.3	83.8	1.2	0.08	#NUM!	8.7E-05
YBR261C	YBR261C	UWOPS87_ctrl_hmg124h_2.JPG.dat	1046.3	70.4	1.0	0.07	#NUM!	1.0E-04
YBR262C	YBR262C	UWOPS87_ctrl_hmg124h_2.JPG.dat	1084.5	59.6	1.0	0.01	#NUM!	6.6E-05
YBR263W	YBR263W	UWOPS87_ctrl_hmg124h_2.JPG.dat	1033.0	70.9	0.9	0.06	#NUM!	9.2E-05
YBR264C	YBR264C	UWOPS87_ctrl_hmg124h_2.JPG.dat	1089.8	61.3	1.0	0.05	#NUM!	3.1E-05
YBR266C	YBR266C	UWOPS87_ctrl_hmg124h_2.JPG.dat	0.0	0.0	0.0	0.00	#NUM!	
YBR267W	YBR267W	UWOPS87_ctrl_hmg124h_2.JPG.dat	0.0	0.0	0.0	0.00	#NUM!	
YBR269C	YBR269C	UWOPS87_ctrl_hmg124h_2.JPG.dat	961.3	42.2	0.9	0.03	#NUM!	1.8E-05

YBR270C	YBR270C	UWOPS87_ctrl_hmg124h_2.JPG.dat	1159.8	48.5	1.0	0.05	#NUM!		4.0E-05
YBR271W	YBR271W	UWOPS87_ctrl_hmg124h_2.JPG.dat	1053.5	72.5	1.0	0.06	#NUM!		5.8E-05
YBR272C	YBR272C	UWOPS87_ctrl_hmg124h_2.JPG.dat	1146.8	59.8	1.1	0.06	#NUM!		5.4E-05
YBR273C	YBR273C	UWOPS87_ctrl_hmg124h_2.JPG.dat	1168.3	35.3	1.1	0.01	#NUM!		4.3E-05
YBR274W	YBR274W	UWOPS87_ctrl_hmg124h_2.JPG.dat	1071.5	235.0	0.9	0.04	#NUM!		6.2E-04
YBR275C	YBR275C	UWOPS87_ctrl_hmg124h_2.JPG.dat	1098.0	273.3	0.9	0.23	#NUM!		4.0E-03
YBR276C	YBR276C	UWOPS87_ctrl_hmg124h_2.JPG.dat	1213.3	193.2	0.9	0.05	#NUM!		1.0E-03
YBR277C	YBR277C	UWOPS87_ctrl_hmg124h_2.JPG.dat	1293.5	35.3	1.1	0.05	#NUM!		1.9E-05
YBR278W	YBR278W	UWOPS87_ctrl_hmg124h_2.JPG.dat	1212.8	46.1	1.0	0.04	#NUM!		1.1E-05
YBR280C	YBR280C	UWOPS87_ctrl_hmg124h_2.JPG.dat	1214.3	80.9	1.0	0.06	#NUM!		5.8E-05
YBR281C	YBR281C	UWOPS87_ctrl_hmg124h_2.JPG.dat	1067.5	46.3	0.9	0.01	#NUM!		4.4E-05
YBR283C	YBR283C	UWOPS87_ctrl_hmg124h_2.JPG.dat	1096.8	94.1	1.0	0.08	#NUM!		1.3E-04
YBR284W	YBR284W	UWOPS87_ctrl_hmg124h_2.JPG.dat	1080.8	110.9	1.0	0.02	#NUM!		1.2E-04
YBR285W	YBR285W	UWOPS87_ctrl_hmg124h_2.JPG.dat	1004.0	51.8	0.9	0.04	#NUM!		2.1E-05
YBR286W	YBR286W	UWOPS87_ctrl_hmg124h_2.JPG.dat	1137.5	42.0	1.0	0.04	#NUM!		1.6E-05
YBR287W	YBR287W	UWOPS87_ctrl_hmg124h_2.JPG.dat	1255.3	36.3	1.1	0.01	#NUM!		1.7E-05
YBR288C	YBR288C	UWOPS87_ctrl_hmg124h_2.JPG.dat	1279.3	36.8	1.1	0.04	#NUM!		1.0E-05
YBR289W	YBR289W	UWOPS87_ctrl_hmg124h_2.JPG.dat	1148.3	56.1	1.0	0.05	#NUM!		3.5E-05
YBR290W	YBR290W	UWOPS87_ctrl_hmg124h_2.JPG.dat	1220.5	26.9	1.1	0.02	#NUM!		2.6E-06
YBR291C	YBR291C	UWOPS87_ctrl_hmg124h_2.JPG.dat	1126.0	125.1	1.0	0.11	#NUM!		3.7E-04
YBR292C	YBR292C	UWOPS87_ctrl_hmg124h_2.JPG.dat	1128.8	105.4	1.0	0.02	#NUM!		1.0E-04
YBR293W	YBR293W	UWOPS87_ctrl_hmg124h_2.JPG.dat	1174.8	177.8	1.0	0.14	#NUM!		7.5E-04
YBR294W	YBR294W	UWOPS87_ctrl_hmg124h_2.JPG.dat	1173.3	81.1	1.0	0.08	#NUM!		1.3E-04
YBR295W	YBR295W	UWOPS87_ctrl_hmg124h_2.JPG.dat	1234.5	40.2	1.1	0.03	#NUM!		8.4E-06
YBR296C	YBR296C	UWOPS87_ctrl_hmg124h_2.JPG.dat	1184.0	109.0	1.1	0.09	#NUM!		2.0E-04
YBR297W	YBR297W	UWOPS87_ctrl_hmg124h_2.JPG.dat	1076.8	59.2	1.0	0.05	#NUM!		5.1E-05
YBR298C	YBR298C	UWOPS87_ctrl_hmg124h_2.JPG.dat	1145.5	188.0	1.0	0.16	#NUM!		1.2E-03
YBR300C	YBR300C	UWOPS87_ctrl_hmg124h_2.JPG.dat	1323.3	184.1	1.0	0.14	#NUM!		6.5E-04
YBR301W	YBR301W	UWOPS87_ctrl_hmg124h_2.JPG.dat	1197.8	95.1	1.0	0.08	#NUM!		1.7E-04
YCL001W	YCL001W	UWOPS87_ctrl_hmg124h_2.JPG.dat	955.8	103.9	0.8	0.09	#NUM!		3.4E-04
YCL001W-/	YCL001W-A	UWOPS87_ctrl_hmg124h_2.JPG.dat	1081.5	89.1	1.0	0.08	#NUM!		1.8E-04
YCL002C	YCL002C	UWOPS87_ctrl_hmg124h_2.JPG.dat	1185.8	89.2	1.0	0.07	#NUM!		7.7E-05
YCL006C	YCL006C	UWOPS87_ctrl_hmg124h_2.JPG.dat	1253.3	106.5	1.1	0.03	#NUM!		2.2E-04
YCL009C	YCL009C	UWOPS87_ctrl_hmg124h_2.JPG.dat	1015.0	36.0	0.9	0.01	#NUM!		2.7E-05
YCL010C	YCL010C	UWOPS87_ctrl_hmg124h_2.JPG.dat	227.3	454.5	0.0	0.00	#NUM!		
YCL011C	YCL011C	UWOPS87_ctrl_hmg124h_2.JPG.dat	938.5	639.6	1.1	0.19	#NUM!		8.7E-03
YCL012W	YCL012W	UWOPS87_ctrl_hmg124h_2.JPG.dat	566.0	653.7	0.5	0.57	#NUM!		1.8E-01
YCL013W	YCL013W	UWOPS87_ctrl_hmg124h_2.JPG.dat	819.8	958.6	0.7	0.83	#NUM!		1.9E-01
YCL014W	YCL014W	UWOPS87_ctrl_hmg124h_2.JPG.dat	701.3	824.1	0.6	0.68	#NUM!		1.9E-01
YCL016C	YCL016C	UWOPS87_ctrl_hmg124h_2.JPG.dat	1051.3	735.6	1.2	0.18	#NUM!		7.5E-03
YCL022C	YCL022C	UWOPS87_ctrl_hmg124h_2.JPG.dat	0.0	0.0	0.0	0.00	#NUM!		
YCL023C	YCL023C	UWOPS87_ctrl_hmg124h_2.JPG.dat	1081.3	75.6	1.0	0.05	#NUM!		4.5E-05
YCL024W	YCL024W	UWOPS87_ctrl_hmg124h_2.JPG.dat	388.5	777.0	0.0	0.00	#NUM!		
YCL025C	YCL025C	UWOPS87_ctrl_hmg124h_2.JPG.dat	505.3	587.7	0.4	0.51	#NUM!		1.9E-01
YCL026C	YCL026C	UWOPS87_ctrl_hmg124h_2.JPG.dat	528.0	620.8	0.5	0.55	#NUM!		1.9E-01
YCL026C-A	YCL026C-A	UWOPS87_ctrl_hmg124h_2.JPG.dat	920.5	632.8	1.1	0.16	#NUM!		6.9E-03
YCL027W	YCL027W	UWOPS87_ctrl_hmg124h_2.JPG.dat	1336.5	51.5	1.2	0.03	#NUM!		7.0E-06
YCL028W	YCL028W	UWOPS87_ctrl_hmg124h_2.JPG.dat	789.8	553.2	1.0	0.19	#NUM!		1.2E-02
YCL029C	YCL029C	UWOPS87_ctrl_hmg124h_2.JPG.dat	1132.8	82.3	1.0	0.07	#NUM!		8.2E-05
YCL030C	YCL030C	UWOPS87_ctrl_hmg124h_2.JPG.dat	0.0	0.0	0.0	0.00	#NUM!		
YCL032W	YCL032W	UWOPS87_ctrl_hmg124h_2.JPG.dat	1279.0	267.0	1.1	0.23	#NUM!		2.7E-03
YCL033C	YCL033C	UWOPS87_ctrl_hmg124h_2.JPG.dat	1066.0	67.7	0.9	0.07	#NUM!		1.0E-04
YCL034W	YCL034W	UWOPS87_ctrl_hmg124h_2.JPG.dat	1070.8	137.1	0.9	0.12	#NUM!		5.9E-04
YCL035C	YCL035C	UWOPS87_ctrl_hmg124h_2.JPG.dat	1040.3	61.4	0.9	0.02	#NUM!		1.8E-04
YCL036W	YCL036W	UWOPS87_ctrl_hmg124h_2.JPG.dat	1224.8	122.1	1.0	0.10	#NUM!		2.6E-04
YCL037C	YCL037C	UWOPS87_ctrl_hmg124h_2.JPG.dat	947.3	120.8	0.9	0.11	#NUM!		5.2E-04
YCL038C	YCL038C	UWOPS87_ctrl_hmg124h_2.JPG.dat	1038.5	34.9	0.9	0.04	#NUM!		1.5E-05
YCL039W	YCL039W	UWOPS87_ctrl_hmg124h_2.JPG.dat	1168.0	66.2	1.1	0.05	#NUM!		3.2E-05
YCL040W	YCL040W	UWOPS87_ctrl_hmg124h_2.JPG.dat	1072.0	23.5	1.0	0.03	#NUM!		4.4E-06
YCL042W	YCL042W	UWOPS87_ctrl_hmg124h_2.JPG.dat	1111.0	69.9	1.0	0.07	#NUM!		9.7E-05
YCL044C	YCL044C	UWOPS87_ctrl_hmg124h_2.JPG.dat	1391.8	62.6	1.2	0.05	#NUM!		2.3E-05
YCL045C	YCL045C	UWOPS87_ctrl_hmg124h_2.JPG.dat	960.5	36.7	0.8	0.01	#NUM!		7.3E-05
YCL046W	YCL046W	UWOPS87_ctrl_hmg124h_2.JPG.dat	1331.8	168.7	1.2	0.15	#NUM!		5.5E-04
YCL047C	YCL047C	UWOPS87_ctrl_hmg124h_2.JPG.dat	1016.8	84.8	0.9	0.07	#NUM!		1.6E-04
YCL048W	YCL048W	UWOPS87_ctrl_hmg124h_2.JPG.dat	1023.8	170.2	0.9	0.14	#NUM!		1.1E-03
YCL049C	YCL049C	UWOPS87_ctrl_hmg124h_2.JPG.dat	726.5	524.5	0.6	0.44	#NUM!		7.0E-02
YCL050C	YCL050C	UWOPS87_ctrl_hmg124h_2.JPG.dat	1113.3	48.9	1.0	0.05	#NUM!		2.9E-05
YCL051W	YCL051W	UWOPS87_ctrl_hmg124h_2.JPG.dat	1040.8	27.4	1.0	0.03	#NUM!		5.7E-06
YCL055W	YCL055W	UWOPS87_ctrl_hmg124h_2.JPG.dat	872.5	61.9	0.8	0.06	#NUM!		1.2E-04
YCL056C	YCL056C	UWOPS87_ctrl_hmg124h_2.JPG.dat	1169.5	106.3	1.1	0.02	#NUM!		1.6E-04

YCL057W	YCL057W	UWOPS87_ctrl_hmg124h_2.JPG.dat	1021.3	23.1	1.0	0.02	#NUM!	4.1E-06
YCL060C	YCL060C	UWOPS87_ctrl_hmg124h_2.JPG.dat	1029.3	18.4	1.0	0.02	#NUM!	3.7E-06
YCL061C	YCL061C	UWOPS87_ctrl_hmg124h_2.JPG.dat	1349.3	8.5	1.2	0.01	#NUM!	4.2E-07
YCL062W	YCL062W	UWOPS87_ctrl_hmg124h_2.JPG.dat	943.3	129.4	0.9	0.11	#NUM!	6.2E-04
YCL063W	YCL063W	UWOPS87_ctrl_hmg124h_2.JPG.dat	1037.0	23.3	0.9	0.03	#NUM!	6.0E-06
YCL064C	YCL064C	UWOPS87_ctrl_hmg124h_2.JPG.dat	1107.5	21.3	1.0	0.02	#NUM!	2.1E-06
YCL069W	YCL069W	UWOPS87_ctrl_hmg124h_2.JPG.dat	1207.8	48.7	1.1	0.03	#NUM!	8.7E-06
YCL074W	YCL074W	UWOPS87_ctrl_hmg124h_2.JPG.dat	1098.0	59.4	1.0	0.05	#NUM!	2.5E-05
YCL075W	YCL075W	UWOPS87_ctrl_hmg124h_2.JPG.dat	1107.0	48.4	1.0	0.04	#NUM!	1.5E-05
YCL076W	YCL076W	UWOPS87_ctrl_hmg124h_2.JPG.dat	992.8	94.8	0.9	0.02	#NUM!	1.3E-04
YCR001W	YCR001W	UWOPS87_ctrl_hmg124h_2.JPG.dat	1173.0	129.7	1.1	0.11	#NUM!	2.7E-04
YCR005C	YCR005C	UWOPS87_ctrl_hmg124h_2.JPG.dat	1301.5	88.1	1.2	0.10	#NUM!	1.4E-04
YCR006C	YCR006C	UWOPS87_ctrl_hmg124h_2.JPG.dat	926.3	88.2	0.8	0.06	#NUM!	1.4E-04
YCR007C	YCR007C	UWOPS87_ctrl_hmg124h_2.JPG.dat	1221.3	115.8	1.0	0.10	#NUM!	2.8E-04
YCR008W	YCR008W	UWOPS87_ctrl_hmg124h_2.JPG.dat	1143.0	111.9	1.0	0.09	#NUM!	1.9E-04
YCR009C	YCR009C	UWOPS87_ctrl_hmg124h_2.JPG.dat	1386.8	14.5	1.2	0.03	#NUM!	3.1E-06
YCR010C	YCR010C	UWOPS87_ctrl_hmg124h_2.JPG.dat	1125.5	92.5	1.0	0.07	#NUM!	1.3E-04
YCR011C	YCR011C	UWOPS87_ctrl_hmg124h_2.JPG.dat	1096.0	77.5	0.9	0.07	#NUM!	1.3E-04
YCR014C	YCR014C	UWOPS87_ctrl_hmg124h_2.JPG.dat	1137.0	23.8	1.0	0.03	#NUM!	7.7E-06
YCR015C	YCR015C	UWOPS87_ctrl_hmg124h_2.JPG.dat	1196.0	96.4	1.1	0.09	#NUM!	1.4E-04
YCR016W	YCR016W	UWOPS87_ctrl_hmg124h_2.JPG.dat	1024.3	32.8	0.9	0.02	#NUM!	4.4E-06
YCR017C	YCR017C	UWOPS87_ctrl_hmg124h_2.JPG.dat	1126.3	31.5	1.0	0.03	#NUM!	4.9E-06
YCR019W	YCR019W	UWOPS87_ctrl_hmg124h_2.JPG.dat	1056.5	46.5	0.9	0.04	#NUM!	2.8E-05
YCR020C	YCR020C	UWOPS87_ctrl_hmg124h_2.JPG.dat	1173.0	93.9	1.0	0.07	#NUM!	1.1E-04
YCR020C-A	YCR020C-A	UWOPS87_ctrl_hmg124h_2.JPG.dat	1129.0	56.1	1.0	0.02	#NUM!	8.3E-05
YCR021C	YCR021C	UWOPS87_ctrl_hmg124h_2.JPG.dat	1079.5	89.8	0.9	0.08	#NUM!	1.7E-04
YCR022C	YCR022C	UWOPS87_ctrl_hmg124h_2.JPG.dat	1278.8	56.7	1.1	0.05	#NUM!	3.0E-05
YCR023C	YCR023C	UWOPS87_ctrl_hmg124h_2.JPG.dat	1215.5	110.3	1.1	0.09	#NUM!	1.9E-04
YCR024C-A	YCR024C-A	UWOPS87_ctrl_hmg124h_2.JPG.dat	1554.5	113.2	1.4	0.04	#NUM!	2.5E-04
YCR025C	YCR025C	UWOPS87_ctrl_hmg124h_2.JPG.dat	1127.5	61.0	1.0	0.06	#NUM!	5.7E-05
YCR026C	YCR026C	UWOPS87_ctrl_hmg124h_2.JPG.dat	1289.0	122.4	1.1	0.10	#NUM!	2.1E-04
YCR027C	YCR027C	UWOPS87_ctrl_hmg124h_2.JPG.dat	1095.3	72.8	1.0	0.06	#NUM!	6.1E-05
YCR028C-A	YCR028C-A	UWOPS87_ctrl_hmg124h_2.JPG.dat	1343.5	134.2	1.2	0.03	#NUM!	1.4E-04
YCR030C	YCR030C	UWOPS87_ctrl_hmg124h_2.JPG.dat	1324.3	47.4	1.1	0.02	#NUM!	8.9E-05
YCR031C	YCR031C	UWOPS87_ctrl_hmg124h_2.JPG.dat	876.3	70.0	0.7	0.06	#NUM!	1.4E-04
YCR032W	YCR032W	UWOPS87_ctrl_hmg124h_2.JPG.dat	1270.3	161.6	1.1	0.03	#NUM!	2.9E-04
YCR033W	YCR033W	UWOPS87_ctrl_hmg124h_2.JPG.dat	1137.0	323.0	0.8	0.08	#NUM!	2.8E-03
YCR034W	YCR034W	UWOPS87_ctrl_hmg124h_2.JPG.dat	1275.5	55.4	1.1	0.05	#NUM!	2.1E-05
YCR036W	YCR036W	UWOPS87_ctrl_hmg124h_2.JPG.dat	0.0	0.0	0.0	0.00	#NUM!	
YCR037C	YCR037C	UWOPS87_ctrl_hmg124h_2.JPG.dat	1256.5	237.0	1.0	0.19	#NUM!	1.6E-03
YCR043C	YCR043C	UWOPS87_ctrl_hmg124h_2.JPG.dat	1198.3	68.9	1.0	0.03	#NUM!	7.9E-06
YCR044C	YCR044C	UWOPS87_ctrl_hmg124h_2.JPG.dat	1190.8	88.2	1.0	0.02	#NUM!	1.7E-04
YCR045C	YCR045C	UWOPS87_ctrl_hmg124h_2.JPG.dat	1175.3	72.9	1.0	0.05	#NUM!	2.5E-05
YCR048W	YCR048W	UWOPS87_ctrl_hmg124h_2.JPG.dat	1108.0	45.7	1.0	0.02	#NUM!	1.7E-06
YCR049C	YCR049C	UWOPS87_ctrl_hmg124h_2.JPG.dat	1031.0	132.3	0.9	0.01	#NUM!	2.3E-05
YCR050C	YCR050C	UWOPS87_ctrl_hmg124h_2.JPG.dat	1143.5	196.9	0.9	0.03	#NUM!	4.3E-04
YCR051W	YCR051W	UWOPS87_ctrl_hmg124h_2.JPG.dat	1163.5	93.8	0.9	0.06	#NUM!	5.5E-05
YCR053W	YCR053W	UWOPS87_ctrl_hmg124h_2.JPG.dat	178.0	356.0	0.0	0.00	#NUM!	
YCR059C	YCR059C	UWOPS87_ctrl_hmg124h_2.JPG.dat	1222.8	157.9	1.1	0.13	#NUM!	5.0E-04
YCR060W	YCR060W	UWOPS87_ctrl_hmg124h_2.JPG.dat	1332.8	103.9	1.2	0.12	#NUM!	3.3E-04
YCR061W	YCR061W	UWOPS87_ctrl_hmg124h_2.JPG.dat	1280.3	165.2	1.1	0.10	#NUM!	2.5E-04
YCR062W	YCR062W	UWOPS87_ctrl_hmg124h_2.JPG.dat	1076.0	65.8	1.0	0.01	#NUM!	2.7E-05
YCR063W	YCR063W	UWOPS87_ctrl_hmg124h_2.JPG.dat	1274.8	93.7	1.1	0.11	#NUM!	2.4E-04
YCR065W	YCR065W	UWOPS87_ctrl_hmg124h_2.JPG.dat	1057.3	136.7	1.0	0.11	#NUM!	3.7E-04
YCR067C	YCR067C	UWOPS87_ctrl_hmg124h_2.JPG.dat	1017.0	24.1	0.9	0.05	#NUM!	3.3E-05
YCR068W	YCR068W	UWOPS87_ctrl_hmg124h_2.JPG.dat	1084.3	26.2	0.9	0.01	#NUM!	9.7E-06
YCR069W	YCR069W	UWOPS87_ctrl_hmg124h_2.JPG.dat	1200.3	71.9	1.0	0.09	#NUM!	1.8E-04
YCR071C	YCR071C	UWOPS87_ctrl_hmg124h_2.JPG.dat	1319.8	66.5	1.1	0.02	#NUM!	1.7E-04
YCR073C	YCR073C	UWOPS87_ctrl_hmg124h_2.JPG.dat	1337.3	158.1	1.2	0.16	#NUM!	7.1E-04
YCR073W-A	YCR073W-A	UWOPS87_ctrl_hmg124h_2.JPG.dat	1126.0	124.6	1.0	0.02	#NUM!	7.2E-05
YCR075C	YCR075C	UWOPS87_ctrl_hmg124h_2.JPG.dat	1199.8	289.7	1.0	0.23	#NUM!	3.3E-03
YCR076C	YCR076C	UWOPS87_ctrl_hmg124h_2.JPG.dat	1413.5	59.0	1.2	0.06	#NUM!	3.5E-05
YCR077C	YCR077C	UWOPS87_ctrl_hmg124h_2.JPG.dat	1003.8	24.8	0.9	0.01	#NUM!	7.0E-07
YCR079W	YCR079W	UWOPS87_ctrl_hmg124h_2.JPG.dat	1099.0	41.5	0.9	0.03	#NUM!	1.3E-05
YCR081W	YCR081W	UWOPS87_ctrl_hmg124h_2.JPG.dat	700.5	65.9	0.6	0.07	#NUM!	3.2E-04
YCR082W	YCR082W	UWOPS87_ctrl_hmg124h_2.JPG.dat	1032.5	11.0	0.9	0.02	#NUM!	2.6E-06
YCR083W	YCR083W	UWOPS87_ctrl_hmg124h_2.JPG.dat	1368.3	60.8	1.2	0.05	#NUM!	2.6E-05
YCR085W	YCR085W	UWOPS87_ctrl_hmg124h_2.JPG.dat	1038.0	24.5	0.9	0.03	#NUM!	1.5E-05
YCR086W	YCR086W	UWOPS87_ctrl_hmg124h_2.JPG.dat	0.0	0.0	0.0	0.00	#NUM!	
YCR087C-A	YCR087C-A	UWOPS87_ctrl_hmg124h_2.JPG.dat	1104.0	49.9	0.9	0.01	#NUM!	3.6E-05

YCR087W	YCR087W	UWOPS87_ctrl_hmg124h_2.JPG.dat	1089.3	17.2	0.9	0.02	#NUM!	5.2E-06
YCR088W	YCR088W	UWOPS87_ctrl_hmg124h_2.JPG.dat	1208.5	137.5	1.0	0.12	#NUM!	4.7E-04
YCR089W	YCR089W	UWOPS87_ctrl_hmg124h_2.JPG.dat	1240.3	94.2	1.0	0.06	#NUM!	5.8E-05
YCR090C	YCR090C	UWOPS87_ctrl_hmg124h_2.JPG.dat	1156.8	50.8	1.0	0.05	#NUM!	2.9E-05
YCR091W	YCR091W	UWOPS87_ctrl_hmg124h_2.JPG.dat	1138.5	41.9	1.0	0.01	#NUM!	3.6E-05
YCR092C	YCR092C	UWOPS87_ctrl_hmg124h_2.JPG.dat	1059.3	16.6	1.0	0.02	#NUM!	2.8E-06
YCR095C	YCR095C	UWOPS87_ctrl_hmg124h_2.JPG.dat	1065.0	27.4	1.0	0.03	#NUM!	1.2E-05
YCR098C	YCR098C	UWOPS87_ctrl_hmg124h_2.JPG.dat	1239.0	138.5	1.2	0.03	#NUM!	2.0E-04
YCR099C	YCR099C	UWOPS87_ctrl_hmg124h_2.JPG.dat	1140.5	192.1	0.9	0.03	#NUM!	3.9E-04
YCR100C	YCR100C	UWOPS87_ctrl_hmg124h_2.JPG.dat	1167.3	114.2	1.0	0.10	#NUM!	2.7E-04
YCR101C	YCR101C	UWOPS87_ctrl_hmg124h_2.JPG.dat	1160.5	125.4	0.9	0.04	#NUM!	6.0E-04
YCR102C	YCR102C	UWOPS87_ctrl_hmg124h_2.JPG.dat	1300.5	133.4	1.1	0.12	#NUM!	3.2E-04
YCR102W-	YCR102W-A	UWOPS87_ctrl_hmg124h_2.JPG.dat	1226.0	122.4	1.0	0.09	#NUM!	2.2E-04
YCR105W	YCR105W	UWOPS87_ctrl_hmg124h_2.JPG.dat	1069.3	45.9	0.9	0.05	#NUM!	5.5E-05
YCR106W	YCR106W	UWOPS87_ctrl_hmg124h_2.JPG.dat	1164.0	41.8	1.1	0.03	#NUM!	7.5E-06
YDL001W	YDL001W	UWOPS87_ctrl_hmg124h_2.JPG.dat	1009.3	106.9	1.0	0.02	#NUM!	1.2E-04
YDL002C	YDL002C	UWOPS87_ctrl_hmg124h_2.JPG.dat	949.5	39.8	0.9	0.03	#NUM!	1.5E-05
YDL006W	YDL006W	UWOPS87_ctrl_hmg124h_2.JPG.dat	559.8	400.3	0.5	0.39	#NUM!	6.9E-02
YDL010W	YDL010W	UWOPS87_ctrl_hmg124h_2.JPG.dat	1069.8	72.0	1.0	0.08	#NUM!	1.4E-04
YDL011C	YDL011C	UWOPS87_ctrl_hmg124h_2.JPG.dat	1350.8	210.0	1.2	0.18	#NUM!	9.6E-04
YDL012C	YDL012C	UWOPS87_ctrl_hmg124h_2.JPG.dat	1247.0	186.7	1.1	0.18	#NUM!	1.1E-03
YDL013W	YDL013W	UWOPS87_ctrl_hmg124h_2.JPG.dat	996.3	43.0	0.9	0.05	#NUM!	4.2E-05
YDL018C	YDL018C	UWOPS87_ctrl_hmg124h_2.JPG.dat	922.8	65.7	0.8	0.07	#NUM!	1.4E-04
YDL019C	YDL019C	UWOPS87_ctrl_hmg124h_2.JPG.dat	1207.0	80.2	1.0	0.06	#NUM!	6.0E-05
YDL020C	YDL020C	UWOPS87_ctrl_hmg124h_2.JPG.dat	941.5	305.7	0.8	0.26	#NUM!	7.8E-03
YDL023C	YDL023C	UWOPS87_ctrl_hmg124h_2.JPG.dat	1318.5	32.8	1.2	0.02	#NUM!	1.3E-06
YDL024C	YDL024C	UWOPS87_ctrl_hmg124h_2.JPG.dat	1096.5	164.2	1.0	0.15	#NUM!	9.9E-04
YDL025C	YDL025C	UWOPS87_ctrl_hmg124h_2.JPG.dat	956.8	79.7	0.9	0.08	#NUM!	1.9E-04
YDL026W	YDL026W	UWOPS87_ctrl_hmg124h_2.JPG.dat	1162.3	49.2	1.1	0.06	#NUM!	3.9E-05
YDL027C	YDL027C	UWOPS87_ctrl_hmg124h_2.JPG.dat	994.3	74.2	0.9	0.07	#NUM!	1.4E-04
YDL033C	YDL033C	UWOPS87_ctrl_hmg124h_2.JPG.dat	1189.0	93.0	1.0	0.07	#NUM!	9.2E-05
YDL034W	YDL034W	UWOPS87_ctrl_hmg124h_2.JPG.dat	1084.3	23.0	0.9	0.03	#NUM!	9.9E-06
YDL035C	YDL035C	UWOPS87_ctrl_hmg124h_2.JPG.dat	1447.5	57.1	1.2	0.01	#NUM!	1.8E-05
YDL036C	YDL036C	UWOPS87_ctrl_hmg124h_2.JPG.dat	1172.8	145.3	1.0	0.02	#NUM!	1.9E-04
YDL037C	YDL037C	UWOPS87_ctrl_hmg124h_2.JPG.dat	1150.8	92.3	1.0	0.07	#NUM!	1.1E-04
YDL038C	YDL038C	UWOPS87_ctrl_hmg124h_2.JPG.dat	1321.3	71.7	1.1	0.05	#NUM!	2.1E-05
YDL039C	YDL039C	UWOPS87_ctrl_hmg124h_2.JPG.dat	1074.8	35.4	0.9	0.01	#NUM!	3.9E-05
YDL040C	YDL040C	UWOPS87_ctrl_hmg124h_2.JPG.dat	1077.3	58.4	0.9	0.06	#NUM!	6.2E-05
YDL041W	YDL041W	UWOPS87_ctrl_hmg124h_2.JPG.dat	900.5	602.8	1.1	0.07	#NUM!	1.3E-03
YDL042C	YDL042C	UWOPS87_ctrl_hmg124h_2.JPG.dat	0.0	0.0	0.0	0.00	#NUM!	
YDL046W	YDL046W	UWOPS87_ctrl_hmg124h_2.JPG.dat	1148.0	69.6	1.0	0.01	#NUM!	3.1E-05
YDL048C	YDL048C	UWOPS87_ctrl_hmg124h_2.JPG.dat	1454.0	40.4	1.2	0.03	#NUM!	4.5E-06
YDL050C	YDL050C	UWOPS87_ctrl_hmg124h_2.JPG.dat	1211.3	128.0	1.0	0.03	#NUM!	4.0E-04
YDL051W	YDL051W	UWOPS87_ctrl_hmg124h_2.JPG.dat	1047.3	166.9	0.8	0.02	#NUM!	3.0E-04
YDL052C	YDL052C	UWOPS87_ctrl_hmg124h_2.JPG.dat	1099.3	43.8	1.0	0.04	#NUM!	1.8E-05
YDL053C	YDL053C	UWOPS87_ctrl_hmg124h_2.JPG.dat	1147.0	102.9	1.0	0.09	#NUM!	2.3E-04
YDL054C	YDL054C	UWOPS87_ctrl_hmg124h_2.JPG.dat	1033.8	110.2	0.9	0.09	#NUM!	3.1E-04
YDL056W	YDL056W	UWOPS87_ctrl_hmg124h_2.JPG.dat	1121.0	111.8	1.0	0.10	#NUM!	2.6E-04
YDL059C	YDL059C	UWOPS87_ctrl_hmg124h_2.JPG.dat	1253.3	44.0	1.1	0.07	#NUM!	8.3E-05
YDL061C	YDL061C	UWOPS87_ctrl_hmg124h_2.JPG.dat	1106.0	84.1	1.0	0.07	#NUM!	1.0E-04
YDL062W	YDL062W	UWOPS87_ctrl_hmg124h_2.JPG.dat	966.3	26.9	0.9	0.00	#NUM!	2.7E-08
YDL065C	YDL065C	UWOPS87_ctrl_hmg124h_2.JPG.dat	1144.8	79.0	1.0	0.02	#NUM!	1.1E-04
YDL066W	YDL066W	UWOPS87_ctrl_hmg124h_2.JPG.dat	1467.5	87.8	1.2	0.02	#NUM!	1.2E-04
YDL070W	YDL070W	UWOPS87_ctrl_hmg124h_2.JPG.dat	1371.0	148.6	1.1	0.03	#NUM!	3.5E-04
YDL071C	YDL071C	UWOPS87_ctrl_hmg124h_2.JPG.dat	1139.5	130.5	1.0	0.10	#NUM!	2.6E-04
YDL074C	YDL074C	UWOPS87_ctrl_hmg124h_2.JPG.dat	1210.8	81.1	1.1	0.04	#NUM!	1.4E-05
YDL076C	YDL076C	UWOPS87_ctrl_hmg124h_2.JPG.dat	1331.3	131.3	1.1	0.01	#NUM!	1.8E-05
YDL077C	YDL077C	UWOPS87_ctrl_hmg124h_2.JPG.dat	0.0	0.0	0.0	0.00	#NUM!	
YDL078C	YDL078C	UWOPS87_ctrl_hmg124h_2.JPG.dat	1307.3	111.8	1.2	0.07	#NUM!	6.7E-05
YDL079C	YDL079C	UWOPS87_ctrl_hmg124h_2.JPG.dat	1165.8	57.8	1.0	0.03	#NUM!	4.8E-06
YDL080C	YDL080C	UWOPS87_ctrl_hmg124h_2.JPG.dat	1018.3	114.7	0.9	0.10	#NUM!	3.3E-04
YDL081C	YDL081C	UWOPS87_ctrl_hmg124h_2.JPG.dat	723.0	50.5	0.7	0.02	#NUM!	2.4E-04
YDL082W	YDL082W	UWOPS87_ctrl_hmg124h_2.JPG.dat	767.8	58.7	0.7	0.03	#NUM!	3.0E-05
YDL083C	YDL083C	UWOPS87_ctrl_hmg124h_2.JPG.dat	1164.5	34.4	1.0	0.04	#NUM!	1.2E-05
YDL085W	YDL085W	UWOPS87_ctrl_hmg124h_2.JPG.dat	1022.3	40.8	0.9	0.02	#NUM!	3.2E-06
YDL086W	YDL086W	UWOPS87_ctrl_hmg124h_2.JPG.dat	1105.5	54.5	1.0	0.05	#NUM!	4.4E-05
YDL088C	YDL088C	UWOPS87_ctrl_hmg124h_2.JPG.dat	1160.8	98.1	1.0	0.02	#NUM!	1.8E-04
YDL089W	YDL089W	UWOPS87_ctrl_hmg124h_2.JPG.dat	1195.5	38.4	1.0	0.01	#NUM!	3.5E-07
YDL090C	YDL090C	UWOPS87_ctrl_hmg124h_2.JPG.dat	0.0	0.0	0.0	0.00	#NUM!	
YDL091C	YDL091C	UWOPS87_ctrl_hmg124h_2.JPG.dat	1161.0	192.6	1.0	0.16	#NUM!	1.1E-03

YDL093W	YDL093W	UWOPS87_ctrl_hmg124h_2.JPG.dat	1248.8	121.6	1.1	0.10	#NUM!	2.5E-04
YDL094C	YDL094C	UWOPS87_ctrl_hmg124h_2.JPG.dat	1136.8	39.0	1.0	0.04	#NUM!	1.7E-05
YDL095W	YDL095W	UWOPS87_ctrl_hmg124h_2.JPG.dat	1432.8	29.6	1.3	0.01	#NUM!	8.2E-06
YDL096C	YDL096C	UWOPS87_ctrl_hmg124h_2.JPG.dat	1280.0	53.4	1.1	0.01	#NUM!	2.1E-05
YDL099W	YDL099W	UWOPS87_ctrl_hmg124h_2.JPG.dat	1295.0	87.9	1.1	0.08	#NUM!	9.8E-05
YDL100C	YDL100C	UWOPS87_ctrl_hmg124h_2.JPG.dat	1344.3	100.1	1.1	0.08	#NUM!	9.3E-05
YDL104C	YDL104C	UWOPS87_ctrl_hmg124h_2.JPG.dat	1508.0	121.9	1.3	0.09	#NUM!	1.2E-04
YDL106C	YDL106C	UWOPS87_ctrl_hmg124h_2.JPG.dat	1428.0	44.9	1.2	0.03	#NUM!	5.4E-06
YDL109C	YDL109C	UWOPS87_ctrl_hmg124h_2.JPG.dat	1153.0	226.0	0.9	0.03	#NUM!	5.3E-04
YDL110C	YDL110C	UWOPS87_ctrl_hmg124h_2.JPG.dat	1322.0	102.8	1.0	0.09	#NUM!	1.7E-04
YDL112W	YDL112W	UWOPS87_ctrl_hmg124h_2.JPG.dat	1205.0	57.5	1.1	0.05	#NUM!	2.9E-05
YDL113C	YDL113C	UWOPS87_ctrl_hmg124h_2.JPG.dat	1137.3	65.7	1.0	0.01	#NUM!	7.1E-05
YDL114W	YDL114W	UWOPS87_ctrl_hmg124h_2.JPG.dat	1228.5	48.6	1.1	0.05	#NUM!	2.7E-05
YDL117W	YDL117W	UWOPS87_ctrl_hmg124h_2.JPG.dat	931.3	103.4	0.9	0.02	#NUM!	1.4E-04
YDL118W	YDL118W	UWOPS87_ctrl_hmg124h_2.JPG.dat	1106.3	79.1	1.0	0.07	#NUM!	8.8E-05
YDL119C	YDL119C	UWOPS87_ctrl_hmg124h_2.JPG.dat	1141.5	127.9	1.0	0.04	#NUM!	6.2E-04
YDL121C	YDL121C	UWOPS87_ctrl_hmg124h_2.JPG.dat	1188.3	255.1	1.0	0.20	#NUM!	2.2E-03
YDL122W	YDL122W	UWOPS87_ctrl_hmg124h_2.JPG.dat	1256.0	47.4	1.1	0.03	#NUM!	6.2E-06
YDL123W	YDL123W	UWOPS87_ctrl_hmg124h_2.JPG.dat	1185.0	76.2	1.0	0.02	#NUM!	1.3E-04
YDL124W	YDL124W	UWOPS87_ctrl_hmg124h_2.JPG.dat	1157.5	89.2	1.0	0.08	#NUM!	1.4E-04
YDL125C	YDL125C	UWOPS87_ctrl_hmg124h_2.JPG.dat	1380.8	57.2	1.1	0.03	#NUM!	5.4E-06
YDL127W	YDL127W	UWOPS87_ctrl_hmg124h_2.JPG.dat	1065.0	32.2	1.0	0.03	#NUM!	4.6E-06
YDL128W	YDL128W	UWOPS87_ctrl_hmg124h_2.JPG.dat	1192.0	24.2	1.1	0.00	#NUM!	1.9E-06
YDL129W	YDL129W	UWOPS87_ctrl_hmg124h_2.JPG.dat	1141.0	90.3	1.1	0.08	#NUM!	1.0E-04
YDL130W	YDL130W	UWOPS87_ctrl_hmg124h_2.JPG.dat	963.8	68.2	0.9	0.06	#NUM!	9.6E-05
YDL130W-,	YDL130W-A	UWOPS87_ctrl_hmg124h_2.JPG.dat	928.5	33.2	0.9	0.03	#NUM!	1.2E-05
YDL131W	YDL131W	UWOPS87_ctrl_hmg124h_2.JPG.dat	1228.3	125.8	1.1	0.13	#NUM!	4.1E-04
YDL133C-A	YDL133C-A	UWOPS87_ctrl_hmg124h_2.JPG.dat	1164.8	141.4	1.0	0.11	#NUM!	3.9E-04
YDL133W	YDL133W	UWOPS87_ctrl_hmg124h_2.JPG.dat	1174.8	262.0	1.0	0.22	#NUM!	2.4E-03
YDL134C	YDL134C	UWOPS87_ctrl_hmg124h_2.JPG.dat	1189.3	85.6	1.1	0.06	#NUM!	6.1E-05
YDL134C-A	YDL134C-A	UWOPS87_ctrl_hmg124h_2.JPG.dat	1296.5	199.7	1.2	0.19	#NUM!	1.1E-03
YDL135C	YDL135C	UWOPS87_ctrl_hmg124h_2.JPG.dat	1160.5	85.6	1.0	0.07	#NUM!	1.0E-04
YDL136W	YDL136W	UWOPS87_ctrl_hmg124h_2.JPG.dat	892.8	41.4	0.8	0.03	#NUM!	1.7E-05
YDL137W	YDL137W	UWOPS87_ctrl_hmg124h_2.JPG.dat	1023.8	58.4	0.9	0.06	#NUM!	8.5E-05
YDL138W	YDL138W	UWOPS87_ctrl_hmg124h_2.JPG.dat	1269.8	45.7	1.2	0.05	#NUM!	1.9E-05
YDL142C	YDL142C	UWOPS87_ctrl_hmg124h_2.JPG.dat	1076.3	59.3	1.0	0.04	#NUM!	2.3E-05
YDL144C	YDL144C	UWOPS87_ctrl_hmg124h_2.JPG.dat	1132.8	45.4	1.0	0.01	#NUM!	1.9E-05
YDL146W	YDL146W	UWOPS87_ctrl_hmg124h_2.JPG.dat	1236.0	51.2	1.1	0.01	#NUM!	4.5E-05
YDL149W	YDL149W	UWOPS87_ctrl_hmg124h_2.JPG.dat	1215.3	10.9	1.0	0.01	#NUM!	3.0E-07
YDL154W	YDL154W	UWOPS87_ctrl_hmg124h_2.JPG.dat	1225.8	69.1	1.1	0.07	#NUM!	6.5E-05
YDL155W	YDL155W	UWOPS87_ctrl_hmg124h_2.JPG.dat	981.3	84.2	0.8	0.02	#NUM!	1.9E-04
YDL156W	YDL156W	UWOPS87_ctrl_hmg124h_2.JPG.dat	1325.3	51.5	1.2	0.05	#NUM!	2.5E-05
YDL157C	YDL157C	UWOPS87_ctrl_hmg124h_2.JPG.dat	1117.8	85.5	0.9	0.08	#NUM!	1.5E-04
YDL159W	YDL159W	UWOPS87_ctrl_hmg124h_2.JPG.dat	0.0	0.0	0.0	0.00	#NUM!	
YDL160C	YDL160C	UWOPS87_ctrl_hmg124h_2.JPG.dat	1272.5	136.3	1.1	0.03	#NUM!	3.2E-04
YDL161W	YDL161W	UWOPS87_ctrl_hmg124h_2.JPG.dat	1093.0	56.6	1.0	0.06	#NUM!	5.4E-05
YDL162C	YDL162C	UWOPS87_ctrl_hmg124h_2.JPG.dat	1189.0	65.9	1.1	0.02	#NUM!	7.9E-05
YDL167C	YDL167C	UWOPS87_ctrl_hmg124h_2.JPG.dat	1319.8	117.9	1.2	0.03	#NUM!	2.3E-04
YDL168W	YDL168W	UWOPS87_ctrl_hmg124h_2.JPG.dat	1360.3	65.4	1.1	0.05	#NUM!	2.6E-05
YDL169C	YDL169C	UWOPS87_ctrl_hmg124h_2.JPG.dat	1401.8	30.9	1.1	0.03	#NUM!	5.6E-06
YDL170W	YDL170W	UWOPS87_ctrl_hmg124h_2.JPG.dat	1291.8	65.3	1.1	0.07	#NUM!	6.8E-05
YDL171C	YDL171C	UWOPS87_ctrl_hmg124h_2.JPG.dat	1107.8	118.1	1.0	0.10	#NUM!	3.0E-04
YDL172C	YDL172C	UWOPS87_ctrl_hmg124h_2.JPG.dat	1155.3	146.8	1.0	0.13	#NUM!	6.2E-04
YDL173W	YDL173W	UWOPS87_ctrl_hmg124h_2.JPG.dat	943.5	56.5	0.8	0.04	#NUM!	4.9E-05
YDL174C	YDL174C	UWOPS87_ctrl_hmg124h_2.JPG.dat	1325.5	69.6	1.1	0.08	#NUM!	8.7E-05
YDL175C	YDL175C	UWOPS87_ctrl_hmg124h_2.JPG.dat	1093.3	129.7	0.9	0.11	#NUM!	4.6E-04
YDL176W	YDL176W	UWOPS87_ctrl_hmg124h_2.JPG.dat	1267.3	34.2	1.1	0.03	#NUM!	6.4E-06
YDL177C	YDL177C	UWOPS87_ctrl_hmg124h_2.JPG.dat	1167.5	96.8	1.0	0.08	#NUM!	1.2E-04
YDL178W	YDL178W	UWOPS87_ctrl_hmg124h_2.JPG.dat	955.0	56.1	0.9	0.02	#NUM!	1.6E-04
YDL179W	YDL179W	UWOPS87_ctrl_hmg124h_2.JPG.dat	1302.5	173.6	1.1	0.12	#NUM!	4.2E-04
YDL180W	YDL180W	UWOPS87_ctrl_hmg124h_2.JPG.dat	1218.3	300.7	1.0	0.22	#NUM!	2.9E-03
YDL181W	YDL181W	UWOPS87_ctrl_hmg124h_2.JPG.dat	1097.5	30.4	1.0	0.05	#NUM!	3.7E-05
YDL182W	YDL182W	UWOPS87_ctrl_hmg124h_2.JPG.dat	990.0	18.5	0.9	0.04	#NUM!	3.0E-05
YDL183C	YDL183C	UWOPS87_ctrl_hmg124h_2.JPG.dat	1010.3	83.0	0.9	0.06	#NUM!	8.2E-05
YDL184C	YDL184C	UWOPS87_ctrl_hmg124h_2.JPG.dat	1216.8	21.5	1.0	0.04	#NUM!	2.2E-05
YOR202W	YOR202W	UWOPS87_ctrl_hmg124h_2.JPG.dat	1387.6	213.1	1.0	0.08	#NUM!	1.9E-296
1	1	UWOPS87_ctrl_hmg124h_3.JPG.dat	0.5	1.0	0.0	0.00	#NUM!	
2	2	UWOPS87_ctrl_hmg124h_3.JPG.dat	1396.8	211.7	1.3	0.22	#NUM!	1.4E-03
3	3	UWOPS87_ctrl_hmg124h_3.JPG.dat	1311.5	227.1	1.1	0.17	#NUM!	1.1E-03
4	4	UWOPS87_ctrl_hmg124h_3.JPG.dat	1262.3	61.0	1.0	0.01	#NUM!	3.1E-05

YDL185W	YDL185W	UWOPS87_ctrl_hmg124h_3.JPG.dat	1065.8	30.1	1.0	0.03	#NUM!	1.1E-05
YDL186W	YDL186W	UWOPS87_ctrl_hmg124h_3.JPG.dat	1005.3	36.4	0.9	0.04	#NUM!	2.1E-05
YDL187C	YDL187C	UWOPS87_ctrl_hmg124h_3.JPG.dat	1092.8	169.2	0.9	0.02	#NUM!	1.0E-04
YDL188C	YDL188C	UWOPS87_ctrl_hmg124h_3.JPG.dat	1001.8	23.5	0.9	0.03	#NUM!	1.1E-05
YDL189W	YDL189W	UWOPS87_ctrl_hmg124h_3.JPG.dat	1288.3	140.5	1.2	0.05	#NUM!	5.7E-04
YDL190C	YDL190C	UWOPS87_ctrl_hmg124h_3.JPG.dat	1138.5	61.8	1.0	0.05	#NUM!	2.7E-05
YDL191W	YDL191W	UWOPS87_ctrl_hmg124h_3.JPG.dat	1281.8	68.5	1.1	0.05	#NUM!	3.3E-05
YDL192W	YDL192W	UWOPS87_ctrl_hmg124h_3.JPG.dat	1160.5	60.4	1.0	0.06	#NUM!	6.9E-05
YDL194W	YDL194W	UWOPS87_ctrl_hmg124h_3.JPG.dat	1349.3	146.4	1.1	0.11	#NUM!	2.3E-04
YDL197C	YDL197C	UWOPS87_ctrl_hmg124h_3.JPG.dat	1186.0	191.4	0.9	0.04	#NUM!	6.0E-04
YDL199C	YDL199C	UWOPS87_ctrl_hmg124h_3.JPG.dat	1114.0	92.9	1.0	0.08	#NUM!	1.2E-04
YDL200C	YDL200C	UWOPS87_ctrl_hmg124h_3.JPG.dat	975.8	70.9	0.9	0.06	#NUM!	9.9E-05
YDL201W	YDL201W	UWOPS87_ctrl_hmg124h_3.JPG.dat	1161.0	125.5	1.1	0.10	#NUM!	2.1E-04
YDL203C	YDL203C	UWOPS87_ctrl_hmg124h_3.JPG.dat	1066.8	29.3	0.9	0.02	#NUM!	4.6E-06
YDL204W	YDL204W	UWOPS87_ctrl_hmg124h_3.JPG.dat	1295.0	60.9	1.1	0.02	#NUM!	1.0E-04
YDL206W	YDL206W	UWOPS87_ctrl_hmg124h_3.JPG.dat	1274.5	63.7	1.1	0.07	#NUM!	7.4E-05
YDL210W	YDL210W	UWOPS87_ctrl_hmg124h_3.JPG.dat	1295.5	109.9	1.1	0.11	#NUM!	3.0E-04
YDL211C	YDL211C	UWOPS87_ctrl_hmg124h_3.JPG.dat	1243.3	97.3	1.0	0.08	#NUM!	1.3E-04
YDL213C	YDL213C	UWOPS87_ctrl_hmg124h_3.JPG.dat	1078.5	73.6	0.9	0.07	#NUM!	1.0E-04
YDL214C	YDL214C	UWOPS87_ctrl_hmg124h_3.JPG.dat	1473.5	95.5	1.2	0.03	#NUM!	1.9E-04
YDL215C	YDL215C	UWOPS87_ctrl_hmg124h_3.JPG.dat	1449.5	49.0	1.2	0.06	#NUM!	3.3E-05
YDL216C	YDL216C	UWOPS87_ctrl_hmg124h_3.JPG.dat	1060.5	38.8	0.9	0.03	#NUM!	1.1E-05
YDL218W	YDL218W	UWOPS87_ctrl_hmg124h_3.JPG.dat	977.0	197.0	0.9	0.19	#NUM!	2.3E-03
YDL219W	YDL219W	UWOPS87_ctrl_hmg124h_3.JPG.dat	980.8	34.2	0.9	0.03	#NUM!	1.1E-05
YDL222C	YDL222C	UWOPS87_ctrl_hmg124h_3.JPG.dat	1266.3	93.4	1.1	0.08	#NUM!	9.8E-05
YDL223C	YDL223C	UWOPS87_ctrl_hmg124h_3.JPG.dat	1002.5	137.7	0.8	0.04	#NUM!	7.7E-04
YDL224C	YDL224C	UWOPS87_ctrl_hmg124h_3.JPG.dat	1194.8	37.1	1.0	0.03	#NUM!	9.6E-06
YDL226C	YDL226C	UWOPS87_ctrl_hmg124h_3.JPG.dat	1088.0	88.4	1.0	0.08	#NUM!	1.9E-04
YDL227C	YDL227C	UWOPS87_ctrl_hmg124h_3.JPG.dat	1163.5	81.0	1.0	0.07	#NUM!	1.0E-04
YDL229W	YDL229W	UWOPS87_ctrl_hmg124h_3.JPG.dat	1266.3	54.0	1.1	0.01	#NUM!	2.2E-05
YDL230W	YDL230W	UWOPS87_ctrl_hmg124h_3.JPG.dat	1319.5	8.9	1.1	0.02	#NUM!	2.1E-06
YDL231C	YDL231C	UWOPS87_ctrl_hmg124h_3.JPG.dat	1264.0	91.9	1.1	0.09	#NUM!	1.4E-04
YDL232W	YDL232W	UWOPS87_ctrl_hmg124h_3.JPG.dat	0.0	0.0	0.0	0.00	#NUM!	
YDL233W	YDL233W	UWOPS87_ctrl_hmg124h_3.JPG.dat	1264.3	97.7	1.2	0.11	#NUM!	2.1E-04
YDL234C	YDL234C	UWOPS87_ctrl_hmg124h_3.JPG.dat	1048.3	23.8	1.0	0.01	#NUM!	5.4E-05
YDL236W	YDL236W	UWOPS87_ctrl_hmg124h_3.JPG.dat	1342.3	96.8	1.2	0.10	#NUM!	1.5E-04
YDL237W	YDL237W	UWOPS87_ctrl_hmg124h_3.JPG.dat	1170.3	8.3	1.0	0.02	#NUM!	2.6E-06
YDL238C	YDL238C	UWOPS87_ctrl_hmg124h_3.JPG.dat	1461.8	86.6	1.2	0.09	#NUM!	1.0E-04
YDL239C	YDL239C	UWOPS87_ctrl_hmg124h_3.JPG.dat	1019.8	76.2	0.9	0.05	#NUM!	4.0E-05
YDL240W	YDL240W	UWOPS87_ctrl_hmg124h_3.JPG.dat	1063.5	63.8	0.9	0.01	#NUM!	3.0E-05
YDL241W	YDL241W	UWOPS87_ctrl_hmg124h_3.JPG.dat	1140.0	89.2	1.0	0.07	#NUM!	1.2E-04
YDL242W	YDL242W	UWOPS87_ctrl_hmg124h_3.JPG.dat	1222.0	64.5	1.0	0.05	#NUM!	2.5E-05
YDL243C	YDL243C	UWOPS87_ctrl_hmg124h_3.JPG.dat	1228.8	76.5	1.1	0.08	#NUM!	1.1E-04
YDR001C	YDR001C	UWOPS87_ctrl_hmg124h_3.JPG.dat	1058.5	55.5	1.0	0.04	#NUM!	1.8E-05
YDR003W	YDR003W	UWOPS87_ctrl_hmg124h_3.JPG.dat	1188.3	245.7	1.0	0.09	#NUM!	2.7E-03
YDR004W	YDR004W	UWOPS87_ctrl_hmg124h_3.JPG.dat	1163.3	132.1	1.1	0.13	#NUM!	4.6E-04
YDR005C	YDR005C	UWOPS87_ctrl_hmg124h_3.JPG.dat	1027.3	687.7	1.2	0.08	#NUM!	1.4E-03
YDR006C	YDR006C	UWOPS87_ctrl_hmg124h_3.JPG.dat	1410.8	38.8	1.2	0.01	#NUM!	1.0E-05
YDR007W	YDR007W	UWOPS87_ctrl_hmg124h_3.JPG.dat	1310.5	63.2	1.0	0.01	#NUM!	6.3E-05
YDR008C	YDR008C	UWOPS87_ctrl_hmg124h_3.JPG.dat	1139.8	60.9	1.0	0.01	#NUM!	2.0E-05
YDR009W	YDR009W	UWOPS87_ctrl_hmg124h_3.JPG.dat	1260.3	99.9	1.0	0.08	#NUM!	1.3E-04
YDR010C	YDR010C	UWOPS87_ctrl_hmg124h_3.JPG.dat	1229.5	95.2	1.0	0.08	#NUM!	1.1E-04
YDR011W	YDR011W	UWOPS87_ctrl_hmg124h_3.JPG.dat	1291.5	120.9	1.1	0.10	#NUM!	2.2E-04
YDR014W	YDR014W	UWOPS87_ctrl_hmg124h_3.JPG.dat	1241.3	62.6	1.0	0.01	#NUM!	4.7E-05
YDR015C	YDR015C	UWOPS87_ctrl_hmg124h_3.JPG.dat	1058.0	83.9	0.9	0.08	#NUM!	1.6E-04
YDR018C	YDR018C	UWOPS87_ctrl_hmg124h_3.JPG.dat	991.3	55.7	0.9	0.06	#NUM!	8.2E-05
YDR019C	YDR019C	UWOPS87_ctrl_hmg124h_3.JPG.dat	972.0	158.6	1.0	0.05	#NUM!	7.2E-04
YDR020C	YDR020C	UWOPS87_ctrl_hmg124h_3.JPG.dat	970.0	87.2	0.9	0.08	#NUM!	1.9E-04
YDR022C	YDR022C	UWOPS87_ctrl_hmg124h_3.JPG.dat	1168.8	97.7	1.0	0.08	#NUM!	1.4E-04
YDR024W	YDR024W	UWOPS87_ctrl_hmg124h_3.JPG.dat	1113.3	112.6	0.9	0.10	#NUM!	3.1E-04
YDR025W	YDR025W	UWOPS87_ctrl_hmg124h_3.JPG.dat	1343.0	84.3	1.1	0.07	#NUM!	7.0E-05
YDR048C	YDR048C	UWOPS87_ctrl_hmg124h_3.JPG.dat	1074.3	60.5	0.9	0.01	#NUM!	8.5E-05
YDR049W	YDR049W	UWOPS87_ctrl_hmg124h_3.JPG.dat	1093.5	27.9	1.0	0.03	#NUM!	1.0E-05
YDR051C	YDR051C	UWOPS87_ctrl_hmg124h_3.JPG.dat	1380.8	192.2	1.2	0.17	#NUM!	8.4E-04
YDR055W	YDR055W	UWOPS87_ctrl_hmg124h_3.JPG.dat	1095.3	164.6	0.9	0.15	#NUM!	1.1E-03
YDR056C	YDR056C	UWOPS87_ctrl_hmg124h_3.JPG.dat	1130.5	177.8	1.0	0.15	#NUM!	9.7E-04
YDR057W	YDR057W	UWOPS87_ctrl_hmg124h_3.JPG.dat	1273.8	128.9	1.1	0.08	#NUM!	8.2E-05
YDR058C	YDR058C	UWOPS87_ctrl_hmg124h_3.JPG.dat	1228.8	82.4	1.1	0.10	#NUM!	1.8E-04
YDR059C	YDR059C	UWOPS87_ctrl_hmg124h_3.JPG.dat	1137.8	147.5	1.0	0.10	#NUM!	2.6E-04
YDR061W	YDR061W	UWOPS87_ctrl_hmg124h_3.JPG.dat	1085.3	114.5	1.0	0.13	#NUM!	6.0E-04

YDR063W	YDR063W	UWOPS87_ctrl_hmg124h_3.JPG.dat	957.3	84.1	0.8	0.07	#NUM!	1.6E-04
YDR066C	YDR066C	UWOPS87_ctrl_hmg124h_3.JPG.dat	996.8	75.1	0.9	0.07	#NUM!	1.6E-04
YDR067C	YDR067C	UWOPS87_ctrl_hmg124h_3.JPG.dat	1064.8	39.6	0.9	0.04	#NUM!	2.3E-05
YDR068W	YDR068W	UWOPS87_ctrl_hmg124h_3.JPG.dat	1104.8	135.2	0.9	0.02	#NUM!	1.1E-04
YDR070C	YDR070C	UWOPS87_ctrl_hmg124h_3.JPG.dat	1023.5	81.1	0.9	0.02	#NUM!	1.3E-04
YDR071C	YDR071C	UWOPS87_ctrl_hmg124h_3.JPG.dat	951.3	669.3	1.1	0.21	#NUM!	1.2E-02
YDR072C	YDR072C	UWOPS87_ctrl_hmg124h_3.JPG.dat	981.8	129.9	0.9	0.02	#NUM!	1.9E-04
YDR073W	YDR073W	UWOPS87_ctrl_hmg124h_3.JPG.dat	1276.3	79.9	1.2	0.08	#NUM!	8.1E-05
YDR074W	YDR074W	UWOPS87_ctrl_hmg124h_3.JPG.dat	1409.3	81.1	1.3	0.06	#NUM!	2.9E-05
YDR075W	YDR075W	UWOPS87_ctrl_hmg124h_3.JPG.dat	1072.3	49.5	0.9	0.02	#NUM!	4.8E-06
YDR076W	YDR076W	UWOPS87_ctrl_hmg124h_3.JPG.dat	1145.5	29.9	1.0	0.04	#NUM!	1.9E-05
YDR077W	YDR077W	UWOPS87_ctrl_hmg124h_3.JPG.dat	1377.8	9.8	1.2	0.00	#NUM!	6.5E-07
YDR080W	YDR080W	UWOPS87_ctrl_hmg124h_3.JPG.dat	1160.8	21.5	1.0	0.01	#NUM!	1.5E-05
YDR083W	YDR083W	UWOPS87_ctrl_hmg124h_3.JPG.dat	880.5	17.2	0.8	0.02	#NUM!	5.7E-06
YDR084C	YDR084C	UWOPS87_ctrl_hmg124h_3.JPG.dat	1169.3	105.8	1.0	0.08	#NUM!	1.2E-04
YDR085C	YDR085C	UWOPS87_ctrl_hmg124h_3.JPG.dat	1022.5	68.5	0.9	0.06	#NUM!	7.8E-05
YDR089W	YDR089W	UWOPS87_ctrl_hmg124h_3.JPG.dat	1085.0	148.6	1.0	0.12	#NUM!	5.5E-04
YDR090C	YDR090C	UWOPS87_ctrl_hmg124h_3.JPG.dat	1109.3	219.1	1.0	0.20	#NUM!	2.2E-03
YDR092W	YDR092W	UWOPS87_ctrl_hmg124h_3.JPG.dat	1256.5	72.2	1.1	0.05	#NUM!	2.9E-05
YDR093W	YDR093W	UWOPS87_ctrl_hmg124h_3.JPG.dat	1014.0	72.6	0.9	0.08	#NUM!	1.4E-04
YDR094W	YDR094W	UWOPS87_ctrl_hmg124h_3.JPG.dat	1048.3	63.6	0.9	0.05	#NUM!	3.6E-05
YDR095C	YDR095C	UWOPS87_ctrl_hmg124h_3.JPG.dat	954.3	291.8	0.9	0.05	#NUM!	7.5E-04
YDR096W	YDR096W	UWOPS87_ctrl_hmg124h_3.JPG.dat	1547.5	75.3	1.3	0.05	#NUM!	1.4E-05
YDR097C	YDR097C	UWOPS87_ctrl_hmg124h_3.JPG.dat	1490.3	23.7	1.2	0.03	#NUM!	6.2E-06
YDR098C	YDR098C	UWOPS87_ctrl_hmg124h_3.JPG.dat	1111.5	152.6	1.0	0.12	#NUM!	5.8E-04
YDR099W	YDR099W	UWOPS87_ctrl_hmg124h_3.JPG.dat	1154.0	73.3	1.0	0.05	#NUM!	2.9E-05
YDR100W	YDR100W	UWOPS87_ctrl_hmg124h_3.JPG.dat	1089.8	120.2	0.9	0.11	#NUM!	4.3E-04
YDR101C	YDR101C	UWOPS87_ctrl_hmg124h_3.JPG.dat	829.3	93.9	0.7	0.09	#NUM!	5.3E-04
YDR102C	YDR102C	UWOPS87_ctrl_hmg124h_3.JPG.dat	1083.5	111.3	0.9	0.10	#NUM!	3.4E-04
YDR103W	YDR103W	UWOPS87_ctrl_hmg124h_3.JPG.dat	0.0	0.0	0.0	0.00	#NUM!	
YDR104C	YDR104C	UWOPS87_ctrl_hmg124h_3.JPG.dat	1101.0	63.4	1.0	0.05	#NUM!	4.3E-05
YDR105C	YDR105C	UWOPS87_ctrl_hmg124h_3.JPG.dat	993.0	66.6	1.0	0.07	#NUM!	9.5E-05
YDR107C	YDR107C	UWOPS87_ctrl_hmg124h_3.JPG.dat	1194.5	131.0	1.1	0.14	#NUM!	5.4E-04
YDR108W	YDR108W	UWOPS87_ctrl_hmg124h_3.JPG.dat	1176.8	35.6	1.0	0.03	#NUM!	8.3E-06
YDR109C	YDR109C	UWOPS87_ctrl_hmg124h_3.JPG.dat	1151.5	98.5	1.0	0.08	#NUM!	1.6E-04
YDR110W	YDR110W	UWOPS87_ctrl_hmg124h_3.JPG.dat	1137.3	30.2	1.0	0.01	#NUM!	1.3E-05
YDR111C	YDR111C	UWOPS87_ctrl_hmg124h_3.JPG.dat	1078.3	90.6	0.9	0.08	#NUM!	1.5E-04
YDR112W	YDR112W	UWOPS87_ctrl_hmg124h_3.JPG.dat	1138.0	122.5	1.0	0.02	#NUM!	7.1E-05
YDR116C	YDR116C	UWOPS87_ctrl_hmg124h_3.JPG.dat	1081.8	39.4	1.0	0.01	#NUM!	1.3E-05
YDR117C	YDR117C	UWOPS87_ctrl_hmg124h_3.JPG.dat	1178.8	125.6	1.0	0.10	#NUM!	2.6E-04
YDR119W	YDR119W	UWOPS87_ctrl_hmg124h_3.JPG.dat	989.3	46.4	0.9	0.04	#NUM!	2.4E-05
YDR120C	YDR120C	UWOPS87_ctrl_hmg124h_3.JPG.dat	1077.5	119.8	1.1	0.11	#NUM!	3.0E-04
YDR121W	YDR121W	UWOPS87_ctrl_hmg124h_3.JPG.dat	1176.0	36.3	1.1	0.04	#NUM!	1.1E-05
YDR122W	YDR122W	UWOPS87_ctrl_hmg124h_3.JPG.dat	1161.0	191.7	1.0	0.15	#NUM!	9.8E-04
YDR123C	YDR123C	UWOPS87_ctrl_hmg124h_3.JPG.dat	0.0	0.0	0.0	0.00	#NUM!	
YDR124W	YDR124W	UWOPS87_ctrl_hmg124h_3.JPG.dat	1349.5	148.3	1.1	0.14	#NUM!	4.8E-04
YDR125C	YDR125C	UWOPS87_ctrl_hmg124h_3.JPG.dat	1176.3	50.6	1.0	0.06	#NUM!	5.2E-05
YDR126W	YDR126W	UWOPS87_ctrl_hmg124h_3.JPG.dat	1222.0	24.3	1.0	0.02	#NUM!	3.2E-06
YDR127W	YDR127W	UWOPS87_ctrl_hmg124h_3.JPG.dat	0.0	0.0	0.0	0.00	#NUM!	
YDR128W	YDR128W	UWOPS87_ctrl_hmg124h_3.JPG.dat	1107.8	25.8	1.0	0.02	#NUM!	2.6E-06
YDR130C	YDR130C	UWOPS87_ctrl_hmg124h_3.JPG.dat	1185.8	120.4	1.0	0.10	#NUM!	2.2E-04
YDR131C	YDR131C	UWOPS87_ctrl_hmg124h_3.JPG.dat	1058.5	76.3	1.0	0.07	#NUM!	1.1E-04
YDR132C	YDR132C	UWOPS87_ctrl_hmg124h_3.JPG.dat	1296.5	91.0	1.3	0.02	#NUM!	7.3E-05
YDR133C	YDR133C	UWOPS87_ctrl_hmg124h_3.JPG.dat	895.3	59.7	0.8	0.04	#NUM!	4.6E-05
YDR134C	YDR134C	UWOPS87_ctrl_hmg124h_3.JPG.dat	912.8	158.7	0.8	0.14	#NUM!	1.5E-03
YDR135C	YDR135C	UWOPS87_ctrl_hmg124h_3.JPG.dat	1070.5	78.1	0.9	0.06	#NUM!	1.1E-04
YDR139C	YDR139C	UWOPS87_ctrl_hmg124h_3.JPG.dat	1240.8	83.7	1.0	0.06	#NUM!	7.5E-05
YDR142C	YDR142C	UWOPS87_ctrl_hmg124h_3.JPG.dat	1301.3	29.0	1.1	0.03	#NUM!	4.6E-06
YDR143C	YDR143C	UWOPS87_ctrl_hmg124h_3.JPG.dat	816.8	108.0	0.7	0.01	#NUM!	5.5E-05
YDR144C	YDR144C	UWOPS87_ctrl_hmg124h_3.JPG.dat	1235.5	25.8	1.0	0.03	#NUM!	9.0E-06
YDR146C	YDR146C	UWOPS87_ctrl_hmg124h_3.JPG.dat	1264.5	19.5	1.1	0.02	#NUM!	1.1E-06
YDR147W	YDR147W	UWOPS87_ctrl_hmg124h_3.JPG.dat	1009.0	24.3	0.9	0.03	#NUM!	7.8E-06
YDR148C	YDR148C	UWOPS87_ctrl_hmg124h_3.JPG.dat	1281.8	80.4	1.1	0.07	#NUM!	6.1E-05
YDR149C	YDR149C	UWOPS87_ctrl_hmg124h_3.JPG.dat	39.3	78.5	0.0	0.00	#NUM!	
YDR150W	YDR150W	UWOPS87_ctrl_hmg124h_3.JPG.dat	525.5	592.5	0.5	0.54	#NUM!	1.7E-01
YDR151C	YDR151C	UWOPS87_ctrl_hmg124h_3.JPG.dat	1060.8	114.5	0.9	0.10	#NUM!	3.1E-04
YDR152W	YDR152W	UWOPS87_ctrl_hmg124h_3.JPG.dat	1238.8	84.8	1.0	0.01	#NUM!	3.2E-05
YDR153C	YDR153C	UWOPS87_ctrl_hmg124h_3.JPG.dat	1083.0	113.6	0.9	0.10	#NUM!	3.7E-04
YDR154C	YDR154C	UWOPS87_ctrl_hmg124h_3.JPG.dat	1308.0	27.3	1.1	0.02	#NUM!	2.6E-06
YDR155C	YDR155C	UWOPS87_ctrl_hmg124h_3.JPG.dat	1333.3	27.7	1.2	0.03	#NUM!	4.7E-06

YDR156W	YDR156W	UWOPS87_ctrl_hmg124h_3.JPG.dat	1093.8	92.5	1.0	0.07	#NUM!	1.3E-04
YDR157W	YDR157W	UWOPS87_ctrl_hmg124h_3.JPG.dat	1180.8	145.0	1.0	0.12	#NUM!	4.0E-04
YDR158W	YDR158W	UWOPS87_ctrl_hmg124h_3.JPG.dat	1061.5	710.5	1.2	0.05	#NUM!	5.6E-04
YDR159W	YDR159W	UWOPS87_ctrl_hmg124h_3.JPG.dat	606.0	731.2	0.5	0.61	#NUM!	1.9E-01
YDR161W	YDR161W	UWOPS87_ctrl_hmg124h_3.JPG.dat	1029.3	102.8	0.9	0.07	#NUM!	1.2E-04
YDR162C	YDR162C	UWOPS87_ctrl_hmg124h_3.JPG.dat	1190.5	107.1	1.1	0.11	#NUM!	2.8E-04
YDR163W	YDR163W	UWOPS87_ctrl_hmg124h_3.JPG.dat	1073.5	53.3	1.0	0.06	#NUM!	7.9E-05
YDR165W	YDR165W	UWOPS87_ctrl_hmg124h_3.JPG.dat	1109.8	54.1	1.0	0.04	#NUM!	2.5E-05
YDR169C	YDR169C	UWOPS87_ctrl_hmg124h_3.JPG.dat	1103.0	59.8	1.0	0.07	#NUM!	1.1E-04
YDR171W	YDR171W	UWOPS87_ctrl_hmg124h_3.JPG.dat	1069.3	35.5	1.0	0.04	#NUM!	1.9E-05
YDR173C	YDR173C	UWOPS87_ctrl_hmg124h_3.JPG.dat	1282.8	130.0	1.2	0.15	#NUM!	5.8E-04
YDR174W	YDR174W	UWOPS87_ctrl_hmg124h_3.JPG.dat	1191.0	28.1	1.1	0.04	#NUM!	1.3E-05
YDR178W	YDR178W	UWOPS87_ctrl_hmg124h_3.JPG.dat	1170.3	38.6	1.1	0.02	#NUM!	7.4E-05
YDR179C	YDR179C	UWOPS87_ctrl_hmg124h_3.JPG.dat	1123.8	756.6	1.3	0.14	#NUM!	4.0E-03
YDR179W-	YDR179W-A	UWOPS87_ctrl_hmg124h_3.JPG.dat	1159.0	48.5	1.0	0.01	#NUM!	6.4E-05
YDR181C	YDR181C	UWOPS87_ctrl_hmg124h_3.JPG.dat	1161.0	61.8	1.0	0.05	#NUM!	4.0E-05
YDR183W	YDR183W	UWOPS87_ctrl_hmg124h_3.JPG.dat	1097.0	74.7	0.9	0.06	#NUM!	6.6E-05
YDR184C	YDR184C	UWOPS87_ctrl_hmg124h_3.JPG.dat	931.3	111.3	0.8	0.10	#NUM!	4.8E-04
YDR185C	YDR185C	UWOPS87_ctrl_hmg124h_3.JPG.dat	1005.3	105.5	0.9	0.09	#NUM!	3.1E-04
YDR186C	YDR186C	UWOPS87_ctrl_hmg124h_3.JPG.dat	1145.0	120.3	1.0	0.11	#NUM!	3.5E-04
YDR191W	YDR191W	UWOPS87_ctrl_hmg124h_3.JPG.dat	1445.5	109.0	1.2	0.09	#NUM!	1.4E-04
YDR192C	YDR192C	UWOPS87_ctrl_hmg124h_3.JPG.dat	1268.5	93.0	1.0	0.08	#NUM!	1.2E-04
YDR193W	YDR193W	UWOPS87_ctrl_hmg124h_3.JPG.dat	1204.0	53.8	1.0	0.05	#NUM!	3.5E-05
YDR198C	YDR198C	UWOPS87_ctrl_hmg124h_3.JPG.dat	1245.5	217.4	1.1	0.05	#NUM!	6.7E-04
YDR199W	YDR199W	UWOPS87_ctrl_hmg124h_3.JPG.dat	1053.8	187.7	1.0	0.16	#NUM!	1.1E-03
YDR202C	YDR202C	UWOPS87_ctrl_hmg124h_3.JPG.dat	1019.5	45.8	0.9	0.01	#NUM!	1.6E-05
YDR203W	YDR203W	UWOPS87_ctrl_hmg124h_3.JPG.dat	1174.3	188.6	1.1	0.17	#NUM!	9.0E-04
YDR205W	YDR205W	UWOPS87_ctrl_hmg124h_3.JPG.dat	1260.8	92.3	1.1	0.08	#NUM!	1.1E-04
YDR206W	YDR206W	UWOPS87_ctrl_hmg124h_3.JPG.dat	1164.3	62.9	1.0	0.06	#NUM!	4.5E-05
YDR207C	YDR207C	UWOPS87_ctrl_hmg124h_3.JPG.dat	321.5	373.4	0.3	0.32	#NUM!	1.8E-01
YDR209C	YDR209C	UWOPS87_ctrl_hmg124h_3.JPG.dat	1132.0	43.0	1.0	0.03	#NUM!	7.9E-06
YDR210W	YDR210W	UWOPS87_ctrl_hmg124h_3.JPG.dat	1133.0	71.4	1.0	0.02	#NUM!	1.1E-04
YDR213W	YDR213W	UWOPS87_ctrl_hmg124h_3.JPG.dat	1277.3	77.4	1.1	0.06	#NUM!	4.7E-05
YDR214W	YDR214W	UWOPS87_ctrl_hmg124h_3.JPG.dat	1140.0	43.9	1.0	0.03	#NUM!	6.1E-06
YDR215C	YDR215C	UWOPS87_ctrl_hmg124h_3.JPG.dat	1033.0	40.0	0.9	0.04	#NUM!	1.7E-05
YDR216W	YDR216W	UWOPS87_ctrl_hmg124h_3.JPG.dat	900.5	133.8	0.8	0.13	#NUM!	9.5E-04
YDR217C	YDR217C	UWOPS87_ctrl_hmg124h_3.JPG.dat	1231.0	62.1	1.1	0.02	#NUM!	1.5E-04
YDR218C	YDR218C	UWOPS87_ctrl_hmg124h_3.JPG.dat	1098.5	86.7	1.0	0.06	#NUM!	6.4E-05
YDR219C	YDR219C	UWOPS87_ctrl_hmg124h_3.JPG.dat	1106.8	50.6	1.0	0.01	#NUM!	2.3E-05
YDR220C	YDR220C	UWOPS87_ctrl_hmg124h_3.JPG.dat	1210.0	106.7	1.0	0.09	#NUM!	1.9E-04
YDR221W	YDR221W	UWOPS87_ctrl_hmg124h_3.JPG.dat	889.5	44.2	0.8	0.03	#NUM!	2.4E-05
YDR222W	YDR222W	UWOPS87_ctrl_hmg124h_3.JPG.dat	1363.8	171.5	1.3	0.01	#NUM!	1.2E-05
YDR223W	YDR223W	UWOPS87_ctrl_hmg124h_3.JPG.dat	1273.0	87.7	1.1	0.07	#NUM!	6.0E-05
YDR225W	YDR225W	UWOPS87_ctrl_hmg124h_3.JPG.dat	1043.3	56.2	0.9	0.04	#NUM!	3.0E-05
YDR227W	YDR227W	UWOPS87_ctrl_hmg124h_3.JPG.dat	0.0	0.0	0.0	0.00	#NUM!	
YDR229W	YDR229W	UWOPS87_ctrl_hmg124h_3.JPG.dat	1331.8	182.2	1.2	0.16	#NUM!	6.9E-04
YDR233C	YDR233C	UWOPS87_ctrl_hmg124h_3.JPG.dat	1092.0	92.4	1.0	0.02	#NUM!	1.3E-04
YDR234W	YDR234W	UWOPS87_ctrl_hmg124h_3.JPG.dat	903.8	52.6	0.9	0.06	#NUM!	9.3E-05
YDR239C	YDR239C	UWOPS87_ctrl_hmg124h_3.JPG.dat	868.8	70.3	0.8	0.09	#NUM!	3.2E-04
YDR241W	YDR241W	UWOPS87_ctrl_hmg124h_3.JPG.dat	1305.3	97.1	1.1	0.10	#NUM!	2.1E-04
YDR244W	YDR244W	UWOPS87_ctrl_hmg124h_3.JPG.dat	1395.5	94.8	1.2	0.03	#NUM!	1.6E-04
YDR245W	YDR245W	UWOPS87_ctrl_hmg124h_3.JPG.dat	1180.5	82.9	1.0	0.03	#NUM!	2.5E-04
YDR247W	YDR247W	UWOPS87_ctrl_hmg124h_3.JPG.dat	1262.0	104.8	1.1	0.10	#NUM!	2.1E-04
YDR248C	YDR248C	UWOPS87_ctrl_hmg124h_3.JPG.dat	1240.5	50.0	1.1	0.05	#NUM!	2.4E-05
YDR249C	YDR249C	UWOPS87_ctrl_hmg124h_3.JPG.dat	1258.5	37.2	1.1	0.05	#NUM!	2.9E-05
YDR250C	YDR250C	UWOPS87_ctrl_hmg124h_3.JPG.dat	1175.0	183.4	1.0	0.14	#NUM!	7.5E-04
YDR251W	YDR251W	UWOPS87_ctrl_hmg124h_3.JPG.dat	1096.5	197.4	0.9	0.16	#NUM!	1.3E-03
YDR252W	YDR252W	UWOPS87_ctrl_hmg124h_3.JPG.dat	1015.0	57.3	0.9	0.05	#NUM!	4.6E-05
YDR253C	YDR253C	UWOPS87_ctrl_hmg124h_3.JPG.dat	1099.8	83.5	1.0	0.02	#NUM!	7.6E-05
YDR254W	YDR254W	UWOPS87_ctrl_hmg124h_3.JPG.dat	1088.0	109.5	1.1	0.03	#NUM!	1.8E-04
YDR255C	YDR255C	UWOPS87_ctrl_hmg124h_3.JPG.dat	1123.0	77.6	1.0	0.07	#NUM!	8.7E-05
YDR256C	YDR256C	UWOPS87_ctrl_hmg124h_3.JPG.dat	1319.8	35.6	1.1	0.01	#NUM!	3.5E-05
YDR257C	YDR257C	UWOPS87_ctrl_hmg124h_3.JPG.dat	1084.8	128.3	0.9	0.11	#NUM!	5.4E-04
YDR258C	YDR258C	UWOPS87_ctrl_hmg124h_3.JPG.dat	1173.3	33.9	1.0	0.04	#NUM!	1.4E-05
YDR259C	YDR259C	UWOPS87_ctrl_hmg124h_3.JPG.dat	1172.8	125.8	1.0	0.04	#NUM!	4.7E-04
YDR260C	YDR260C	UWOPS87_ctrl_hmg124h_3.JPG.dat	1294.5	157.0	1.1	0.11	#NUM!	2.9E-04
YDR261C	YDR261C	UWOPS87_ctrl_hmg124h_3.JPG.dat	1228.0	162.2	1.0	0.12	#NUM!	4.1E-04
YDR262W	YDR262W	UWOPS87_ctrl_hmg124h_3.JPG.dat	1063.3	23.9	0.9	0.00	#NUM!	3.8E-06
YDR263C	YDR263C	UWOPS87_ctrl_hmg124h_3.JPG.dat	1119.5	138.6	1.0	0.12	#NUM!	4.1E-04
YDR265W	YDR265W	UWOPS87_ctrl_hmg124h_3.JPG.dat	981.5	36.6	0.9	0.05	#NUM!	4.8E-05

YDR266C	YDR266C	UWOPS87_ctrl_hmg124h_3.JPG.dat	988.8	24.9	1.0	0.03	#NUM!	6.9E-06
YDR270W	YDR270W	UWOPS87_ctrl_hmg124h_3.JPG.dat	1625.0	76.3	1.5	0.05	#NUM!	1.2E-05
YDR272W	YDR272W	UWOPS87_ctrl_hmg124h_3.JPG.dat	1179.3	44.5	1.0	0.02	#NUM!	1.6E-06
YDR273W	YDR273W	UWOPS87_ctrl_hmg124h_3.JPG.dat	1323.0	32.3	1.1	0.04	#NUM!	1.8E-05
YDR274C	YDR274C	UWOPS87_ctrl_hmg124h_3.JPG.dat	1294.3	44.4	1.1	0.05	#NUM!	3.1E-05
YDR275W	YDR275W	UWOPS87_ctrl_hmg124h_3.JPG.dat	1278.0	108.5	1.1	0.02	#NUM!	1.5E-04
YDR276C	YDR276C	UWOPS87_ctrl_hmg124h_3.JPG.dat	1235.5	36.8	1.1	0.02	#NUM!	2.0E-06
YDR277C	YDR277C	UWOPS87_ctrl_hmg124h_3.JPG.dat	1161.3	101.4	1.0	0.07	#NUM!	8.8E-05
YDR278C	YDR278C	UWOPS87_ctrl_hmg124h_3.JPG.dat	1204.0	135.6	1.1	0.03	#NUM!	3.0E-04
YDR279W	YDR279W	UWOPS87_ctrl_hmg124h_3.JPG.dat	1219.3	51.7	1.1	0.04	#NUM!	1.5E-05
YDR281C	YDR281C	UWOPS87_ctrl_hmg124h_3.JPG.dat	1028.3	19.2	0.9	0.01	#NUM!	2.2E-07
YDR282C	YDR282C	UWOPS87_ctrl_hmg124h_3.JPG.dat	990.8	77.1	0.9	0.07	#NUM!	1.0E-04
YDR284C	YDR284C	UWOPS87_ctrl_hmg124h_3.JPG.dat	1105.3	124.1	1.0	0.11	#NUM!	3.6E-04
YDR285W	YDR285W	UWOPS87_ctrl_hmg124h_3.JPG.dat	998.0	161.4	0.9	0.13	#NUM!	8.4E-04
YDR286C	YDR286C	UWOPS87_ctrl_hmg124h_3.JPG.dat	1264.0	98.3	1.1	0.08	#NUM!	1.4E-04
YDR287W	YDR287W	UWOPS87_ctrl_hmg124h_3.JPG.dat	1347.0	104.2	1.2	0.07	#NUM!	5.6E-05
YDR289C	YDR289C	UWOPS87_ctrl_hmg124h_3.JPG.dat	1079.3	68.1	0.9	0.07	#NUM!	1.0E-04
YDR291W	YDR291W	UWOPS87_ctrl_hmg124h_3.JPG.dat	1250.5	105.4	1.1	0.03	#NUM!	2.8E-04
YDR293C	YDR293C	UWOPS87_ctrl_hmg124h_3.JPG.dat	1248.8	130.2	1.1	0.10	#NUM!	1.9E-04
YDR294C	YDR294C	UWOPS87_ctrl_hmg124h_3.JPG.dat	1088.8	81.1	0.9	0.09	#NUM!	2.1E-04
YDR297W	YDR297W	UWOPS87_ctrl_hmg124h_3.JPG.dat	1327.0	69.9	1.1	0.02	#NUM!	8.3E-05
YDR304C	YDR304C	UWOPS87_ctrl_hmg124h_3.JPG.dat	1148.3	34.2	1.0	0.01	#NUM!	5.9E-05
YDR305C	YDR305C	UWOPS87_ctrl_hmg124h_3.JPG.dat	1070.5	105.3	1.0	0.10	#NUM!	2.9E-04
YDR306C	YDR306C	UWOPS87_ctrl_hmg124h_3.JPG.dat	1204.0	106.1	1.0	0.08	#NUM!	1.6E-04
YDR307W	YDR307W	UWOPS87_ctrl_hmg124h_3.JPG.dat	1264.8	188.1	1.1	0.15	#NUM!	7.3E-04
YDR309C	YDR309C	UWOPS87_ctrl_hmg124h_3.JPG.dat	1164.3	125.8	1.0	0.11	#NUM!	3.3E-04
YDR310C	YDR310C	UWOPS87_ctrl_hmg124h_3.JPG.dat	1169.5	95.1	1.0	0.08	#NUM!	1.3E-04
YDR312W	YDR312W	UWOPS87_ctrl_hmg124h_3.JPG.dat	1192.5	113.5	1.0	0.09	#NUM!	1.9E-04
YDR313C	YDR313C	UWOPS87_ctrl_hmg124h_3.JPG.dat	1366.0	100.1	1.1	0.09	#NUM!	1.3E-04
YDR314C	YDR314C	UWOPS87_ctrl_hmg124h_3.JPG.dat	1178.3	107.1	0.9	0.03	#NUM!	2.6E-04
YDR315C	YDR315C	UWOPS87_ctrl_hmg124h_3.JPG.dat	914.3	34.7	0.8	0.03	#NUM!	2.5E-05
YDR316W	YDR316W	UWOPS87_ctrl_hmg124h_3.JPG.dat	1410.5	58.0	1.2	0.08	#NUM!	7.9E-05
YDR317W	YDR317W	UWOPS87_ctrl_hmg124h_3.JPG.dat	1056.3	67.6	1.0	0.05	#NUM!	3.9E-05
YDR318W	YDR318W	UWOPS87_ctrl_hmg124h_3.JPG.dat	1109.8	44.3	1.1	0.05	#NUM!	2.5E-05
YDR319C	YDR319C	UWOPS87_ctrl_hmg124h_3.JPG.dat	1298.0	90.2	1.2	0.09	#NUM!	1.3E-04
YDR320C	YDR320C	UWOPS87_ctrl_hmg124h_3.JPG.dat	1005.8	162.3	0.9	0.14	#NUM!	9.4E-04
YDR321W	YDR321W	UWOPS87_ctrl_hmg124h_3.JPG.dat	1122.5	122.3	1.0	0.10	#NUM!	3.2E-04
YDR329C	YDR329C	UWOPS87_ctrl_hmg124h_3.JPG.dat	1337.5	120.9	1.1	0.10	#NUM!	2.1E-04
YDR330W	YDR330W	UWOPS87_ctrl_hmg124h_3.JPG.dat	1169.0	198.1	1.0	0.17	#NUM!	1.1E-03
YDR332W	YDR332W	UWOPS87_ctrl_hmg124h_3.JPG.dat	1182.0	41.7	1.0	0.04	#NUM!	1.1E-05
YDR333C	YDR333C	UWOPS87_ctrl_hmg124h_3.JPG.dat	1070.0	144.8	0.9	0.04	#NUM!	6.2E-04
YDR334W	YDR334W	UWOPS87_ctrl_hmg124h_3.JPG.dat	1023.3	34.7	0.9	0.03	#NUM!	7.0E-06
YDR335W	YDR335W	UWOPS87_ctrl_hmg124h_3.JPG.dat	1285.0	102.5	1.1	0.10	#NUM!	1.7E-04
YDR336W	YDR336W	UWOPS87_ctrl_hmg124h_3.JPG.dat	1102.3	45.3	1.0	0.05	#NUM!	3.0E-05
YDR338C	YDR338C	UWOPS87_ctrl_hmg124h_3.JPG.dat	1035.3	166.4	1.0	0.15	#NUM!	9.4E-04
YDR340W	YDR340W	UWOPS87_ctrl_hmg124h_3.JPG.dat	1028.3	26.6	0.9	0.03	#NUM!	9.1E-06
YDR344C	YDR344C	UWOPS87_ctrl_hmg124h_3.JPG.dat	1504.0	84.5	1.3	0.07	#NUM!	3.7E-05
YDR345C	YDR345C	UWOPS87_ctrl_hmg124h_3.JPG.dat	1028.5	82.4	0.9	0.07	#NUM!	1.4E-04
YDR346C	YDR346C	UWOPS87_ctrl_hmg124h_3.JPG.dat	1097.0	189.4	0.9	0.16	#NUM!	1.5E-03
YDR348C	YDR348C	UWOPS87_ctrl_hmg124h_3.JPG.dat	1046.0	237.8	0.9	0.20	#NUM!	2.9E-03
YDR349C	YDR349C	UWOPS87_ctrl_hmg124h_3.JPG.dat	1224.5	79.5	1.1	0.06	#NUM!	6.0E-05
YDR351W	YDR351W	UWOPS87_ctrl_hmg124h_3.JPG.dat	1072.0	79.0	0.9	0.06	#NUM!	7.3E-05
YDR352W	YDR352W	UWOPS87_ctrl_hmg124h_3.JPG.dat	1356.8	92.8	1.2	0.09	#NUM!	1.0E-04
YDR354W	YDR354W	UWOPS87_ctrl_hmg124h_3.JPG.dat	1017.8	85.1	0.9	0.08	#NUM!	2.1E-04
YDR357C	YDR357C	UWOPS87_ctrl_hmg124h_3.JPG.dat	1163.8	112.1	1.1	0.11	#NUM!	3.1E-04
YDR358W	YDR358W	UWOPS87_ctrl_hmg124h_3.JPG.dat	946.5	84.7	0.9	0.07	#NUM!	1.3E-04
YDR359C	YDR359C	UWOPS87_ctrl_hmg124h_3.JPG.dat	946.3	272.2	0.9	0.23	#NUM!	4.9E-03
YDR360W	YDR360W	UWOPS87_ctrl_hmg124h_3.JPG.dat	1067.0	85.8	1.0	0.02	#NUM!	1.9E-04
YDR363W	YDR363W	UWOPS87_ctrl_hmg124h_3.JPG.dat	1249.8	63.9	1.1	0.05	#NUM!	2.5E-05
YDR363W-	YDR363W-A	UWOPS87_ctrl_hmg124h_3.JPG.dat	1009.0	159.2	0.9	0.12	#NUM!	7.3E-04
YDR368W	YDR368W	UWOPS87_ctrl_hmg124h_3.JPG.dat	960.3	57.5	0.9	0.05	#NUM!	5.6E-05
YDR369C	YDR369C	UWOPS87_ctrl_hmg124h_3.JPG.dat	1048.3	34.2	0.9	0.03	#NUM!	1.2E-05
YDR370C	YDR370C	UWOPS87_ctrl_hmg124h_3.JPG.dat	1382.3	18.3	1.2	0.04	#NUM!	9.3E-06
YDR371W	YDR371W	UWOPS87_ctrl_hmg124h_3.JPG.dat	1168.5	135.9	1.1	0.02	#NUM!	1.2E-04
YDR372C	YDR372C	UWOPS87_ctrl_hmg124h_3.JPG.dat	1457.0	44.7	1.3	0.05	#NUM!	1.9E-05
YDR374C	YDR374C	UWOPS87_ctrl_hmg124h_3.JPG.dat	1057.3	47.1	1.0	0.05	#NUM!	4.1E-05
YDR375C	YDR375C	UWOPS87_ctrl_hmg124h_3.JPG.dat	0.0	0.0	0.0	0.00	#NUM!	
YDR378C	YDR378C	UWOPS87_ctrl_hmg124h_3.JPG.dat	0.0	0.0	0.0	0.00	#NUM!	
YDR379W	YDR379W	UWOPS87_ctrl_hmg124h_3.JPG.dat	1463.0	130.4	1.3	0.13	#NUM!	2.6E-04
YDR380W	YDR380W	UWOPS87_ctrl_hmg124h_3.JPG.dat	1064.8	228.9	0.9	0.18	#NUM!	2.0E-03

YDR382W	YDR382W	UWOPS87_ctrl_hmg124h_3.JPG.dat	0.0	0.0	0.0	0.00	#NUM!	
YDR383C	YDR383C	UWOPS87_ctrl_hmg124h_3.JPG.dat	1119.5	91.3	1.0	0.07	#NUM!	8.7E-05
YDR384C	YDR384C	UWOPS87_ctrl_hmg124h_3.JPG.dat	1145.5	74.6	1.0	0.09	#NUM!	1.7E-04
YDR385W	YDR385W	UWOPS87_ctrl_hmg124h_3.JPG.dat	1120.5	94.3	0.9	0.02	#NUM!	1.1E-04
YDR386W	YDR386W	UWOPS87_ctrl_hmg124h_3.JPG.dat	1171.8	95.3	1.0	0.08	#NUM!	1.4E-04
YDR387C	YDR387C	UWOPS87_ctrl_hmg124h_3.JPG.dat	1054.8	86.8	0.9	0.09	#NUM!	2.2E-04
YDR388W	YDR388W	UWOPS87_ctrl_hmg124h_3.JPG.dat	1139.5	38.1	1.0	0.04	#NUM!	1.3E-05
YDR389W	YDR389W	UWOPS87_ctrl_hmg124h_3.JPG.dat	908.5	118.2	0.9	0.11	#NUM!	5.0E-04
YDR391C	YDR391C	UWOPS87_ctrl_hmg124h_3.JPG.dat	1063.0	192.8	1.0	0.18	#NUM!	1.5E-03
YDR392W	YDR392W	UWOPS87_ctrl_hmg124h_3.JPG.dat	0.0	0.0	0.0	0.00	#NUM!	
YDR393W	YDR393W	UWOPS87_ctrl_hmg124h_3.JPG.dat	1268.0	46.7	1.1	0.03	#NUM!	5.6E-06
YDR395W	YDR395W	UWOPS87_ctrl_hmg124h_3.JPG.dat	1288.8	56.3	1.1	0.03	#NUM!	7.0E-06
YDR399W	YDR399W	UWOPS87_ctrl_hmg124h_3.JPG.dat	1076.0	68.8	0.9	0.07	#NUM!	1.4E-04
YDR400W	YDR400W	UWOPS87_ctrl_hmg124h_3.JPG.dat	1217.0	83.2	1.1	0.06	#NUM!	4.7E-05
YDR401W	YDR401W	UWOPS87_ctrl_hmg124h_3.JPG.dat	1064.5	39.8	0.9	0.04	#NUM!	2.3E-05
YDR402C	YDR402C	UWOPS87_ctrl_hmg124h_3.JPG.dat	908.5	71.4	0.8	0.02	#NUM!	1.3E-04
YDR403W	YDR403W	UWOPS87_ctrl_hmg124h_3.JPG.dat	1136.5	225.3	1.0	0.19	#NUM!	2.0E-03
YDR406W	YDR406W	UWOPS87_ctrl_hmg124h_3.JPG.dat	1177.0	96.2	1.0	0.03	#NUM!	2.6E-04
YDR408C	YDR408C	UWOPS87_ctrl_hmg124h_3.JPG.dat	1084.8	112.9	1.0	0.10	#NUM!	2.6E-04
YDR409W	YDR409W	UWOPS87_ctrl_hmg124h_3.JPG.dat	1488.3	79.4	1.4	0.07	#NUM!	3.1E-05
YDR410C	YDR410C	UWOPS87_ctrl_hmg124h_3.JPG.dat	0.0	0.0	0.0	0.00	#NUM!	
YDR411C	YDR411C	UWOPS87_ctrl_hmg124h_3.JPG.dat	1189.8	51.5	1.0	0.05	#NUM!	2.9E-05
YDR414C	YDR414C	UWOPS87_ctrl_hmg124h_3.JPG.dat	1611.8	89.9	1.3	0.03	#NUM!	1.3E-04
YDR415C	YDR415C	UWOPS87_ctrl_hmg124h_3.JPG.dat	1006.0	101.2	0.9	0.09	#NUM!	3.0E-04
YDR419W	YDR419W	UWOPS87_ctrl_hmg124h_3.JPG.dat	1070.8	114.5	0.9	0.10	#NUM!	2.9E-04
YDR420W	YDR420W	UWOPS87_ctrl_hmg124h_3.JPG.dat	1052.5	28.3	0.9	0.04	#NUM!	1.6E-05
YDR421W	YDR421W	UWOPS87_ctrl_hmg124h_3.JPG.dat	1092.3	22.3	1.0	0.03	#NUM!	5.6E-06
YDR422C	YDR422C	UWOPS87_ctrl_hmg124h_3.JPG.dat	1171.3	92.9	1.0	0.07	#NUM!	8.5E-05
YOR202W	YOR202W	UWOPS87_ctrl_hmg124h_3.JPG.dat	1314.0	216.0	1.0	0.11	#NUM!	3.3E-257
1	1	UWOPS87_ctrl_hmg124h_4.JPG.dat	1590.5	377.9	1.0	0.13	#NUM!	6.4E-04
2	2	UWOPS87_ctrl_hmg124h_4.JPG.dat	1316.0	116.1	1.0	0.01	#NUM!	5.3E-05
3	3	UWOPS87_ctrl_hmg124h_4.JPG.dat	1176.5	121.3	0.9	0.07	#NUM!	1.5E-04
4	4	UWOPS87_ctrl_hmg124h_4.JPG.dat	1446.5	112.1	1.0	0.05	#NUM!	3.0E-05
YDR423C	YDR423C	UWOPS87_ctrl_hmg124h_4.JPG.dat	1114.8	45.4	1.0	0.06	#NUM!	5.7E-05
YDR424C	YDR424C	UWOPS87_ctrl_hmg124h_4.JPG.dat	1072.3	49.5	0.9	0.06	#NUM!	8.0E-05
YDR425W	YDR425W	UWOPS87_ctrl_hmg124h_4.JPG.dat	1293.5	134.6	1.1	0.11	#NUM!	2.9E-04
YDR426C	YDR426C	UWOPS87_ctrl_hmg124h_4.JPG.dat	1255.8	137.8	1.1	0.03	#NUM!	2.5E-04
YDR428C	YDR428C	UWOPS87_ctrl_hmg124h_4.JPG.dat	1080.8	32.0	0.9	0.03	#NUM!	1.5E-05
YDR430C	YDR430C	UWOPS87_ctrl_hmg124h_4.JPG.dat	1209.8	122.1	1.0	0.10	#NUM!	2.7E-04
YDR431W	YDR431W	UWOPS87_ctrl_hmg124h_4.JPG.dat	1317.5	20.1	1.1	0.02	#NUM!	1.5E-06
YDR435C	YDR435C	UWOPS87_ctrl_hmg124h_4.JPG.dat	1167.8	49.3	1.0	0.02	#NUM!	4.2E-06
YDR436W	YDR436W	UWOPS87_ctrl_hmg124h_4.JPG.dat	1251.5	76.8	1.0	0.06	#NUM!	6.7E-05
YDR438W	YDR438W	UWOPS87_ctrl_hmg124h_4.JPG.dat	1280.0	28.3	1.0	0.03	#NUM!	8.7E-06
YDR439W	YDR439W	UWOPS87_ctrl_hmg124h_4.JPG.dat	1289.3	53.0	1.1	0.05	#NUM!	2.7E-05
YDR440W	YDR440W	UWOPS87_ctrl_hmg124h_4.JPG.dat	1267.8	110.5	1.2	0.10	#NUM!	1.6E-04
YDR441C	YDR441C	UWOPS87_ctrl_hmg124h_4.JPG.dat	1087.0	83.9	1.0	0.07	#NUM!	1.1E-04
YDR445C	YDR445C	UWOPS87_ctrl_hmg124h_4.JPG.dat	1350.5	48.7	1.2	0.04	#NUM!	1.1E-05
YDR446W	YDR446W	UWOPS87_ctrl_hmg124h_4.JPG.dat	1110.0	72.8	1.0	0.06	#NUM!	7.3E-05
YDR447C	YDR447C	UWOPS87_ctrl_hmg124h_4.JPG.dat	1009.8	62.5	0.9	0.05	#NUM!	7.1E-05
YDR451C	YDR451C	UWOPS87_ctrl_hmg124h_4.JPG.dat	1161.5	148.1	1.0	0.13	#NUM!	5.5E-04
YDR452W	YDR452W	UWOPS87_ctrl_hmg124h_4.JPG.dat	1119.8	54.2	0.9	0.05	#NUM!	3.4E-05
YDR453C	YDR453C	UWOPS87_ctrl_hmg124h_4.JPG.dat	1271.5	30.3	1.1	0.04	#NUM!	9.6E-06
YDR455C	YDR455C	UWOPS87_ctrl_hmg124h_4.JPG.dat	1163.3	28.8	1.0	0.00	#NUM!	7.0E-06
YDR458C	YDR458C	UWOPS87_ctrl_hmg124h_4.JPG.dat	1194.5	181.4	1.0	0.16	#NUM!	9.8E-04
YDR459C	YDR459C	UWOPS87_ctrl_hmg124h_4.JPG.dat	1118.3	82.2	0.9	0.02	#NUM!	1.3E-04
YDR463W	YDR463W	UWOPS87_ctrl_hmg124h_4.JPG.dat	1032.8	77.4	1.0	0.07	#NUM!	1.1E-04
YDR465C	YDR465C	UWOPS87_ctrl_hmg124h_4.JPG.dat	1094.5	50.8	1.0	0.04	#NUM!	1.8E-05
YDR466W	YDR466W	UWOPS87_ctrl_hmg124h_4.JPG.dat	1130.0	71.6	1.0	0.06	#NUM!	4.3E-05
YDR467C	YDR467C	UWOPS87_ctrl_hmg124h_4.JPG.dat	1031.3	112.4	0.9	0.10	#NUM!	3.5E-04
YDR469W	YDR469W	UWOPS87_ctrl_hmg124h_4.JPG.dat	1150.8	17.4	1.0	0.01	#NUM!	3.6E-08
YDR471W	YDR471W	UWOPS87_ctrl_hmg124h_4.JPG.dat	1257.0	108.2	1.2	0.00	#NUM!	5.6E-06
YDR474C	YDR474C	UWOPS87_ctrl_hmg124h_4.JPG.dat	1181.0	42.4	1.0	0.05	#NUM!	3.0E-05
YDR475C	YDR475C	UWOPS87_ctrl_hmg124h_4.JPG.dat	1055.0	106.8	1.0	0.10	#NUM!	3.1E-04
YDR476C	YDR476C	UWOPS87_ctrl_hmg124h_4.JPG.dat	1217.3	60.8	1.1	0.05	#NUM!	3.0E-05
YDR479C	YDR479C	UWOPS87_ctrl_hmg124h_4.JPG.dat	1114.3	48.3	1.0	0.05	#NUM!	4.3E-05
YDR480W	YDR480W	UWOPS87_ctrl_hmg124h_4.JPG.dat	996.8	95.3	0.9	0.08	#NUM!	1.7E-04
YDR481C	YDR481C	UWOPS87_ctrl_hmg124h_4.JPG.dat	1082.0	69.9	1.0	0.04	#NUM!	2.6E-05
YDR482C	YDR482C	UWOPS87_ctrl_hmg124h_4.JPG.dat	1263.8	64.6	1.1	0.04	#NUM!	1.0E-05
YDR483W	YDR483W	UWOPS87_ctrl_hmg124h_4.JPG.dat	1228.3	64.9	1.1	0.07	#NUM!	8.2E-05
YDR485C	YDR485C	UWOPS87_ctrl_hmg124h_4.JPG.dat	1031.8	22.3	0.9	0.03	#NUM!	1.2E-05

YDR486C	YDR486C	UWOPS87_ctrl_hmg124h_4.JPG.dat	0.0	0.0	0.0	0.00	#NUM!	
YDR488C	YDR488C	UWOPS87_ctrl_hmg124h_4.JPG.dat	1084.0	50.2	0.9	0.02	#NUM!	1.8E-04
YDR490C	YDR490C	UWOPS87_ctrl_hmg124h_4.JPG.dat	1018.5	357.0	1.0	0.02	#NUM!	8.5E-05
YDR491C	YDR491C	UWOPS87_ctrl_hmg124h_4.JPG.dat	1163.8	59.6	1.0	0.06	#NUM!	5.4E-05
YDR492W	YDR492W	UWOPS87_ctrl_hmg124h_4.JPG.dat	1251.0	83.5	1.1	0.01	#NUM!	5.8E-05
YDR494W	YDR494W	UWOPS87_ctrl_hmg124h_4.JPG.dat	1002.8	42.1	0.8	0.00	#NUM!	5.2E-06
YDR496C	YDR496C	UWOPS87_ctrl_hmg124h_4.JPG.dat	1024.0	12.4	0.9	0.01	#NUM!	1.3E-06
YDR497C	YDR497C	UWOPS87_ctrl_hmg124h_4.JPG.dat	1157.8	103.2	1.0	0.02	#NUM!	2.0E-04
YDR500C	YDR500C	UWOPS87_ctrl_hmg124h_4.JPG.dat	1205.8	94.8	1.1	0.07	#NUM!	9.3E-05
YDR501W	YDR501W	UWOPS87_ctrl_hmg124h_4.JPG.dat	1131.5	50.1	1.0	0.04	#NUM!	2.4E-05
YDR503C	YDR503C	UWOPS87_ctrl_hmg124h_4.JPG.dat	899.3	328.5	1.0	0.00	#NUM!	7.6E-06
YDR504C	YDR504C	UWOPS87_ctrl_hmg124h_4.JPG.dat	1125.3	66.7	1.0	0.05	#NUM!	3.7E-05
YDR505C	YDR505C	UWOPS87_ctrl_hmg124h_4.JPG.dat	1148.8	58.0	1.0	0.02	#NUM!	1.4E-04
YDR506C	YDR506C	UWOPS87_ctrl_hmg124h_4.JPG.dat	1150.0	65.8	1.0	0.06	#NUM!	6.2E-05
YDR508C	YDR508C	UWOPS87_ctrl_hmg124h_4.JPG.dat	1171.0	113.7	1.0	0.11	#NUM!	3.8E-04
YDR509W	YDR509W	UWOPS87_ctrl_hmg124h_4.JPG.dat	1006.8	69.8	0.9	0.06	#NUM!	1.0E-04
YDR511W	YDR511W	UWOPS87_ctrl_hmg124h_4.JPG.dat	1193.0	67.1	1.0	0.07	#NUM!	8.3E-05
YDR512C	YDR512C	UWOPS87_ctrl_hmg124h_4.JPG.dat	1119.5	72.1	1.0	0.02	#NUM!	1.4E-04
YDR513W	YDR513W	UWOPS87_ctrl_hmg124h_4.JPG.dat	1085.3	57.4	1.0	0.06	#NUM!	6.6E-05
YDR514C	YDR514C	UWOPS87_ctrl_hmg124h_4.JPG.dat	1258.3	21.4	1.2	0.03	#NUM!	4.3E-06
YDR516C	YDR516C	UWOPS87_ctrl_hmg124h_4.JPG.dat	1076.0	143.8	1.0	0.12	#NUM!	5.5E-04
YDR517W	YDR517W	UWOPS87_ctrl_hmg124h_4.JPG.dat	992.5	68.2	0.9	0.03	#NUM!	2.4E-04
YDR519W	YDR519W	UWOPS87_ctrl_hmg124h_4.JPG.dat	1337.8	53.7	1.2	0.05	#NUM!	1.7E-05
YDR520C	YDR520C	UWOPS87_ctrl_hmg124h_4.JPG.dat	968.0	38.6	0.9	0.01	#NUM!	1.3E-05
YDR522C	YDR522C	UWOPS87_ctrl_hmg124h_4.JPG.dat	1062.5	85.8	0.9	0.07	#NUM!	1.3E-04
YDR524C	YDR524C	UWOPS87_ctrl_hmg124h_4.JPG.dat	1044.8	67.4	0.9	0.06	#NUM!	7.7E-05
YDR525W	YDR525W	UWOPS87_ctrl_hmg124h_4.JPG.dat	1208.0	80.0	1.1	0.07	#NUM!	7.8E-05
YDR525W-	YDR525W-A	UWOPS87_ctrl_hmg124h_4.JPG.dat	997.5	100.7	1.0	0.03	#NUM!	2.9E-04
YDR528W	YDR528W	UWOPS87_ctrl_hmg124h_4.JPG.dat	1040.0	33.9	0.9	0.02	#NUM!	3.5E-06
YDR530C	YDR530C	UWOPS87_ctrl_hmg124h_4.JPG.dat	886.0	45.8	0.8	0.04	#NUM!	5.0E-05
YDR532C	YDR532C	UWOPS87_ctrl_hmg124h_4.JPG.dat	773.8	525.1	0.9	0.11	#NUM!	5.1E-03
YDR533C	YDR533C	UWOPS87_ctrl_hmg124h_4.JPG.dat	1119.8	126.3	1.0	0.11	#NUM!	3.9E-04
YDR534C	YDR534C	UWOPS87_ctrl_hmg124h_4.JPG.dat	1214.3	27.8	1.1	0.02	#NUM!	1.7E-06
YDR535C	YDR535C	UWOPS87_ctrl_hmg124h_4.JPG.dat	1119.8	53.4	0.9	0.01	#NUM!	2.3E-05
YDR536W	YDR536W	UWOPS87_ctrl_hmg124h_4.JPG.dat	1142.5	88.8	1.0	0.07	#NUM!	1.0E-04
YDR537C	YDR537C	UWOPS87_ctrl_hmg124h_4.JPG.dat	1131.5	6.6	1.0	0.01	#NUM!	3.1E-07
YDR538W	YDR538W	UWOPS87_ctrl_hmg124h_4.JPG.dat	1123.3	21.7	1.0	0.03	#NUM!	6.7E-06
YDR539W	YDR539W	UWOPS87_ctrl_hmg124h_4.JPG.dat	1079.0	34.7	1.0	0.03	#NUM!	7.6E-06
YDR540C	YDR540C	UWOPS87_ctrl_hmg124h_4.JPG.dat	824.3	550.5	1.0	0.03	#NUM!	3.2E-04
YDR541C	YDR541C	UWOPS87_ctrl_hmg124h_4.JPG.dat	1078.3	22.9	0.9	0.03	#NUM!	1.1E-05
YEL001C	YEL001C	UWOPS87_ctrl_hmg124h_4.JPG.dat	1190.8	31.1	1.1	0.01	#NUM!	8.0E-06
YEL003W	YEL003W	UWOPS87_ctrl_hmg124h_4.JPG.dat	1323.5	26.3	1.1	0.02	#NUM!	2.4E-06
YEL004W	YEL004W	UWOPS87_ctrl_hmg124h_4.JPG.dat	1050.0	62.5	0.9	0.01	#NUM!	4.2E-05
YEL005C	YEL005C	UWOPS87_ctrl_hmg124h_4.JPG.dat	1061.8	32.7	0.9	0.02	#NUM!	4.6E-06
YEL006W	YEL006W	UWOPS87_ctrl_hmg124h_4.JPG.dat	1373.3	36.7	1.1	0.03	#NUM!	3.5E-06
YEL007W	YEL007W	UWOPS87_ctrl_hmg124h_4.JPG.dat	1317.3	23.0	1.1	0.02	#NUM!	1.1E-06
YEL008W	YEL008W	UWOPS87_ctrl_hmg124h_4.JPG.dat	1342.0	94.2	1.1	0.07	#NUM!	6.0E-05
YEL010W	YEL010W	UWOPS87_ctrl_hmg124h_4.JPG.dat	1217.5	65.8	1.0	0.04	#NUM!	2.2E-05
YEL011W	YEL011W	UWOPS87_ctrl_hmg124h_4.JPG.dat	1162.0	43.2	1.0	0.03	#NUM!	6.5E-06
YEL012W	YEL012W	UWOPS87_ctrl_hmg124h_4.JPG.dat	856.5	615.6	0.7	0.51	#NUM!	6.9E-02
YEL013W	YEL013W	UWOPS87_ctrl_hmg124h_4.JPG.dat	957.8	56.0	0.8	0.05	#NUM!	5.7E-05
YEL014C	YEL014C	UWOPS87_ctrl_hmg124h_4.JPG.dat	771.5	515.4	1.0	0.04	#NUM!	6.9E-04
YEL015W	YEL015W	UWOPS87_ctrl_hmg124h_4.JPG.dat	1027.3	97.2	0.9	0.08	#NUM!	1.9E-04
YEL016C	YEL016C	UWOPS87_ctrl_hmg124h_4.JPG.dat	1024.5	22.9	0.9	0.02	#NUM!	1.7E-06
YEL017C-A	YEL017C-A	UWOPS87_ctrl_hmg124h_4.JPG.dat	1265.8	68.1	1.1	0.02	#NUM!	1.2E-04
YEL017W	YEL017W	UWOPS87_ctrl_hmg124h_4.JPG.dat	1281.0	29.6	1.1	0.02	#NUM!	9.3E-07
YEL020C	YEL020C	UWOPS87_ctrl_hmg124h_4.JPG.dat	1217.8	71.4	1.0	0.06	#NUM!	5.7E-05
YEL023C	YEL023C	UWOPS87_ctrl_hmg124h_4.JPG.dat	1125.3	54.1	1.0	0.05	#NUM!	3.4E-05
YEL025C	YEL025C	UWOPS87_ctrl_hmg124h_4.JPG.dat	1275.3	112.9	1.1	0.09	#NUM!	1.6E-04
YEL028W	YEL028W	UWOPS87_ctrl_hmg124h_4.JPG.dat	932.5	55.8	0.8	0.05	#NUM!	5.5E-05
YEL030W	YEL030W	UWOPS87_ctrl_hmg124h_4.JPG.dat	1093.0	71.7	1.0	0.06	#NUM!	7.7E-05
YEL031W	YEL031W	UWOPS87_ctrl_hmg124h_4.JPG.dat	854.5	273.5	0.7	0.23	#NUM!	8.2E-03
YEL033W	YEL033W	UWOPS87_ctrl_hmg124h_4.JPG.dat	750.0	14.4	0.7	0.02	#NUM!	8.3E-06
YEL037C	YEL037C	UWOPS87_ctrl_hmg124h_4.JPG.dat	1090.0	78.4	1.0	0.08	#NUM!	1.6E-04
YEL038W	YEL038W	UWOPS87_ctrl_hmg124h_4.JPG.dat	1204.3	66.5	1.1	0.08	#NUM!	9.2E-05
YEL039C	YEL039C	UWOPS87_ctrl_hmg124h_4.JPG.dat	1096.8	28.0	1.0	0.04	#NUM!	1.7E-05
YEL040W	YEL040W	UWOPS87_ctrl_hmg124h_4.JPG.dat	1060.5	47.9	0.9	0.03	#NUM!	1.0E-05
YEL041W	YEL041W	UWOPS87_ctrl_hmg124h_4.JPG.dat	1153.8	37.3	1.0	0.02	#NUM!	2.4E-06
YEL042W	YEL042W	UWOPS87_ctrl_hmg124h_4.JPG.dat	1059.3	52.6	0.9	0.05	#NUM!	4.6E-05
YEL043W	YEL043W	UWOPS87_ctrl_hmg124h_4.JPG.dat	1167.0	29.2	1.0	0.02	#NUM!	3.9E-06

YEL047C	YEL047C	UWOPS87_ctrl_hmg124h_4.JPG.dat	1034.8	110.7	1.0	0.10	#NUM!	2.7E-04
YEL048C	YEL048C	UWOPS87_ctrl_hmg124h_4.JPG.dat	1006.3	11.8	0.9	0.02	#NUM!	4.3E-06
YEL049W	YEL049W	UWOPS87_ctrl_hmg124h_4.JPG.dat	1285.0	28.9	1.2	0.02	#NUM!	1.5E-06
YEL052W	YEL052W	UWOPS87_ctrl_hmg124h_4.JPG.dat	1275.0	56.1	1.2	0.04	#NUM!	1.4E-05
YEL053C	YEL053C	UWOPS87_ctrl_hmg124h_4.JPG.dat	1221.5	68.5	1.1	0.04	#NUM!	2.1E-05
YEL056W	YEL056W	UWOPS87_ctrl_hmg124h_4.JPG.dat	1057.0	72.8	0.9	0.05	#NUM!	3.7E-05
YEL057C	YEL057C	UWOPS87_ctrl_hmg124h_4.JPG.dat	1311.8	41.0	1.2	0.06	#NUM!	3.2E-05
YEL059W	YEL059W	UWOPS87_ctrl_hmg124h_4.JPG.dat	1141.0	105.8	1.0	0.07	#NUM!	1.1E-04
YEL060C	YEL060C	UWOPS87_ctrl_hmg124h_4.JPG.dat	1172.8	59.9	1.0	0.05	#NUM!	3.5E-05
YEL061C	YEL061C	UWOPS87_ctrl_hmg124h_4.JPG.dat	1150.5	39.7	1.0	0.01	#NUM!	3.3E-05
YEL062W	YEL062W	UWOPS87_ctrl_hmg124h_4.JPG.dat	426.5	492.7	0.4	0.42	#NUM!	1.8E-01
YEL063C	YEL063C	UWOPS87_ctrl_hmg124h_4.JPG.dat	0.0	0.0	0.0	0.00	#NUM!	
YEL064C	YEL064C	UWOPS87_ctrl_hmg124h_4.JPG.dat	1201.8	77.9	1.0	0.07	#NUM!	1.0E-04
YEL065W	YEL065W	UWOPS87_ctrl_hmg124h_4.JPG.dat	1137.0	23.2	1.0	0.03	#NUM!	6.4E-06
YEL066W	YEL066W	UWOPS87_ctrl_hmg124h_4.JPG.dat	1034.8	51.5	1.0	0.01	#NUM!	1.8E-05
YEL067C	YEL067C	UWOPS87_ctrl_hmg124h_4.JPG.dat	1281.3	52.5	1.1	0.05	#NUM!	2.0E-05
YEL068C	YEL068C	UWOPS87_ctrl_hmg124h_4.JPG.dat	1025.8	97.4	0.9	0.08	#NUM!	1.6E-04
YEL071W	YEL071W	UWOPS87_ctrl_hmg124h_4.JPG.dat	982.5	135.4	1.0	0.02	#NUM!	1.4E-04
YER001W	YER001W	UWOPS87_ctrl_hmg124h_4.JPG.dat	1059.0	129.9	0.9	0.11	#NUM!	4.5E-04
YER002W	YER002W	UWOPS87_ctrl_hmg124h_4.JPG.dat	1087.3	50.1	1.0	0.05	#NUM!	4.5E-05
YER004W	YER004W	UWOPS87_ctrl_hmg124h_4.JPG.dat	1084.5	41.1	1.0	0.03	#NUM!	6.1E-06
YER005W	YER005W	UWOPS87_ctrl_hmg124h_4.JPG.dat	1211.3	102.7	1.1	0.04	#NUM!	3.7E-04
YER007C-A	YER007C-A	UWOPS87_ctrl_hmg124h_4.JPG.dat	1074.3	95.6	1.0	0.01	#NUM!	4.5E-05
YER007W	YER007W	UWOPS87_ctrl_hmg124h_4.JPG.dat	1295.8	42.2	1.1	0.03	#NUM!	4.5E-06
YER010C	YER010C	UWOPS87_ctrl_hmg124h_4.JPG.dat	1240.5	46.1	1.1	0.03	#NUM!	6.3E-06
YER011W	YER011W	UWOPS87_ctrl_hmg124h_4.JPG.dat	820.8	83.6	0.8	0.08	#NUM!	3.3E-04
YER016W	YER016W	UWOPS87_ctrl_hmg124h_4.JPG.dat	1036.0	62.6	1.0	0.05	#NUM!	3.7E-05
YER019C-A	YER019C-A	UWOPS87_ctrl_hmg124h_4.JPG.dat	1058.8	97.5	0.9	0.03	#NUM!	2.9E-04
YER019W	YER019W	UWOPS87_ctrl_hmg124h_4.JPG.dat	1185.8	156.4	1.1	0.15	#NUM!	7.2E-04
YER020W	YER020W	UWOPS87_ctrl_hmg124h_4.JPG.dat	1194.3	65.0	1.1	0.01	#NUM!	4.2E-05
YER024W	YER024W	UWOPS87_ctrl_hmg124h_4.JPG.dat	1192.3	82.1	1.1	0.08	#NUM!	1.2E-04
YER027C	YER027C	UWOPS87_ctrl_hmg124h_4.JPG.dat	1211.3	46.4	1.1	0.05	#NUM!	2.1E-05
YER028C	YER028C	UWOPS87_ctrl_hmg124h_4.JPG.dat	1234.0	8.6	1.1	0.00	#NUM!	6.5E-07
YER030W	YER030W	UWOPS87_ctrl_hmg124h_4.JPG.dat	1029.3	82.5	0.9	0.02	#NUM!	1.2E-04
YER031C	YER031C	UWOPS87_ctrl_hmg124h_4.JPG.dat	809.0	71.5	0.7	0.01	#NUM!	5.3E-05
YER032W	YER032W	UWOPS87_ctrl_hmg124h_4.JPG.dat	1130.0	47.4	1.0	0.04	#NUM!	1.8E-05
YER033C	YER033C	UWOPS87_ctrl_hmg124h_4.JPG.dat	1293.5	77.3	1.2	0.07	#NUM!	7.2E-05
YER034W	YER034W	UWOPS87_ctrl_hmg124h_4.JPG.dat	885.8	183.6	0.8	0.16	#NUM!	2.3E-03
YER035W	YER035W	UWOPS87_ctrl_hmg124h_4.JPG.dat	1101.5	147.1	1.0	0.13	#NUM!	6.8E-04
YER038W-.	YER038W-A	UWOPS87_ctrl_hmg124h_4.JPG.dat	1205.8	57.9	1.1	0.06	#NUM!	3.8E-05
YER039C	YER039C	UWOPS87_ctrl_hmg124h_4.JPG.dat	1214.8	85.7	1.0	0.07	#NUM!	7.5E-05
YER039C-A	YER039C-A	UWOPS87_ctrl_hmg124h_4.JPG.dat	1171.8	61.7	1.0	0.01	#NUM!	3.5E-05
YER041W	YER041W	UWOPS87_ctrl_hmg124h_4.JPG.dat	1225.0	87.8	1.1	0.07	#NUM!	7.4E-05
YER042W	YER042W	UWOPS87_ctrl_hmg124h_4.JPG.dat	1099.3	37.3	1.0	0.04	#NUM!	2.4E-05
YER044C-A	YER044C-A	UWOPS87_ctrl_hmg124h_4.JPG.dat	1254.5	63.2	1.2	0.07	#NUM!	5.3E-05
YER045C	YER045C	UWOPS87_ctrl_hmg124h_4.JPG.dat	1110.0	43.0	1.0	0.04	#NUM!	1.7E-05
YER046W	YER046W	UWOPS87_ctrl_hmg124h_4.JPG.dat	1049.0	23.7	0.9	0.00	#NUM!	4.6E-06
YER046W-.	YER046W-A	UWOPS87_ctrl_hmg124h_4.JPG.dat	1021.0	91.9	0.8	0.07	#NUM!	1.6E-04
YER047C	YER047C	UWOPS87_ctrl_hmg124h_4.JPG.dat	1197.5	26.3	1.0	0.01	#NUM!	9.2E-06
YER048C	YER048C	UWOPS87_ctrl_hmg124h_4.JPG.dat	1224.0	46.5	1.0	0.03	#NUM!	1.2E-05
YER049W	YER049W	UWOPS87_ctrl_hmg124h_4.JPG.dat	1182.0	50.9	0.9	0.05	#NUM!	3.5E-05
YER051W	YER051W	UWOPS87_ctrl_hmg124h_4.JPG.dat	1255.3	138.6	1.0	0.11	#NUM!	3.9E-04
YER052C	YER052C	UWOPS87_ctrl_hmg124h_4.JPG.dat	1307.0	62.1	1.0	0.04	#NUM!	2.0E-05
YER053C	YER053C	UWOPS87_ctrl_hmg124h_4.JPG.dat	1329.5	20.5	1.0	0.02	#NUM!	1.3E-06
YER054C	YER054C	UWOPS87_ctrl_hmg124h_4.JPG.dat	1439.3	75.9	1.1	0.07	#NUM!	6.4E-05
YER055C	YER055C	UWOPS87_ctrl_hmg124h_4.JPG.dat	1414.5	39.3	1.1	0.01	#NUM!	3.3E-05
YER056C	YER056C	UWOPS87_ctrl_hmg124h_4.JPG.dat	944.5	638.3	1.0	0.09	#NUM!	3.0E-03
YER056C-A	YER056C-A	UWOPS87_ctrl_hmg124h_4.JPG.dat	1485.8	228.5	1.2	0.17	#NUM!	8.7E-04
YER057C	YER057C	UWOPS87_ctrl_hmg124h_4.JPG.dat	995.5	69.4	0.9	0.06	#NUM!	6.8E-05
YER059W	YER059W	UWOPS87_ctrl_hmg124h_4.JPG.dat	1311.5	40.2	1.1	0.01	#NUM!	1.3E-05
YER060W	YER060W	UWOPS87_ctrl_hmg124h_4.JPG.dat	1271.8	112.6	1.1	0.03	#NUM!	2.6E-04
YER060W-.	YER060W-A	UWOPS87_ctrl_hmg124h_4.JPG.dat	1277.8	53.5	1.0	0.05	#NUM!	2.4E-05
YER061C	YER061C	UWOPS87_ctrl_hmg124h_4.JPG.dat	0.0	0.0	0.0	0.00	#NUM!	
YER062C	YER062C	UWOPS87_ctrl_hmg124h_4.JPG.dat	1096.3	77.1	0.9	0.06	#NUM!	8.9E-05
YER063W	YER063W	UWOPS87_ctrl_hmg124h_4.JPG.dat	1327.5	73.5	1.0	0.05	#NUM!	3.7E-05
YER064C	YER064C	UWOPS87_ctrl_hmg124h_4.JPG.dat	1070.8	86.7	0.9	0.08	#NUM!	2.0E-04
YER065C	YER065C	UWOPS87_ctrl_hmg124h_4.JPG.dat	1374.3	51.0	1.1	0.04	#NUM!	8.9E-06
YER066C-A	YER066C-A	UWOPS87_ctrl_hmg124h_4.JPG.dat	1319.0	44.3	1.1	0.01	#NUM!	4.4E-05
YER066W	YER066W	UWOPS87_ctrl_hmg124h_4.JPG.dat	1392.8	31.0	1.1	0.02	#NUM!	2.9E-06
YER067C-A	YER067C-A	UWOPS87_ctrl_hmg124h_4.JPG.dat	1232.0	47.1	1.1	0.04	#NUM!	1.6E-05

YER067W	YER067W	UWOPS87_ctrl_hmg124h_4.JPG.dat	1085.8	61.8	0.9	0.05	#NUM!	4.1E-05
YER069W	YER069W	UWOPS87_ctrl_hmg124h_4.JPG.dat	0.0	0.0	0.0	0.00	#NUM!	
YER071C	YER071C	UWOPS87_ctrl_hmg124h_4.JPG.dat	1290.3	68.3	1.1	0.06	#NUM!	4.9E-05
YER072W	YER072W	UWOPS87_ctrl_hmg124h_4.JPG.dat	1235.0	100.8	1.0	0.02	#NUM!	2.1E-04
YER073W	YER073W	UWOPS87_ctrl_hmg124h_4.JPG.dat	1274.5	24.3	1.0	0.02	#NUM!	1.8E-06
YER074W	YER074W	UWOPS87_ctrl_hmg124h_4.JPG.dat	1265.5	31.5	1.0	0.02	#NUM!	3.7E-06
YER075C	YER075C	UWOPS87_ctrl_hmg124h_4.JPG.dat	1219.0	119.7	1.1	0.01	#NUM!	5.6E-05
YER077C	YER077C	UWOPS87_ctrl_hmg124h_4.JPG.dat	1243.0	39.7	1.0	0.04	#NUM!	1.2E-05
YER078C	YER078C	UWOPS87_ctrl_hmg124h_4.JPG.dat	1262.5	15.9	1.0	0.01	#NUM!	4.3E-07
YER079W	YER079W	UWOPS87_ctrl_hmg124h_4.JPG.dat	1283.8	47.1	1.0	0.01	#NUM!	6.7E-05
YER080W	YER080W	UWOPS87_ctrl_hmg124h_4.JPG.dat	1078.5	121.9	0.9	0.10	#NUM!	3.8E-04
YER081W	YER081W	UWOPS87_ctrl_hmg124h_4.JPG.dat	1293.8	28.5	1.1	0.02	#NUM!	2.3E-06
YER083C	YER083C	UWOPS87_ctrl_hmg124h_4.JPG.dat	1107.0	26.0	0.9	0.01	#NUM!	1.6E-05
YER084W	YER084W	UWOPS87_ctrl_hmg124h_4.JPG.dat	1313.3	20.8	1.1	0.00	#NUM!	4.5E-06
YER085C	YER085C	UWOPS87_ctrl_hmg124h_4.JPG.dat	1251.8	44.1	1.0	0.04	#NUM!	1.6E-05
YER087C-A	YER087C-A	UWOPS87_ctrl_hmg124h_4.JPG.dat	1330.5	46.5	1.1	0.01	#NUM!	3.2E-05
YER088C	YER088C	UWOPS87_ctrl_hmg124h_4.JPG.dat	1241.5	27.1	1.0	0.01	#NUM!	2.9E-05
YER090W	YER090W	UWOPS87_ctrl_hmg124h_4.JPG.dat	1335.0	66.8	1.1	0.02	#NUM!	7.8E-05
YER091C	YER091C	UWOPS87_ctrl_hmg124h_4.JPG.dat	1196.3	56.6	1.0	0.02	#NUM!	8.2E-05
YER091C-A	YER091C-A	UWOPS87_ctrl_hmg124h_4.JPG.dat	1300.3	53.5	1.1	0.00	#NUM!	3.0E-06
YER092W	YER092W	UWOPS87_ctrl_hmg124h_4.JPG.dat	1085.5	101.8	0.9	0.08	#NUM!	2.0E-04
YER093C-A	YER093C-A	UWOPS87_ctrl_hmg124h_4.JPG.dat	1316.8	38.7	1.1	0.04	#NUM!	8.9E-06
YER095W	YER095W	UWOPS87_ctrl_hmg124h_4.JPG.dat	994.5	49.5	0.8	0.04	#NUM!	3.3E-05
YER096W	YER096W	UWOPS87_ctrl_hmg124h_4.JPG.dat	1278.3	64.8	1.1	0.05	#NUM!	3.2E-05
YER097W	YER097W	UWOPS87_ctrl_hmg124h_4.JPG.dat	1274.0	56.1	1.1	0.05	#NUM!	2.6E-05
YER098W	YER098W	UWOPS87_ctrl_hmg124h_4.JPG.dat	1348.5	27.9	1.1	0.01	#NUM!	2.3E-05
YER101C	YER101C	UWOPS87_ctrl_hmg124h_4.JPG.dat	1293.0	62.2	1.1	0.05	#NUM!	3.0E-05
YER106W	YER106W	UWOPS87_ctrl_hmg124h_4.JPG.dat	1284.0	39.6	1.0	0.04	#NUM!	1.4E-05
YER108C	YER108C	UWOPS87_ctrl_hmg124h_4.JPG.dat	1284.3	26.5	1.1	0.03	#NUM!	5.5E-06
YER109C	YER109C	UWOPS87_ctrl_hmg124h_4.JPG.dat	1305.0	64.0	1.1	0.02	#NUM!	9.0E-05
YER111C	YER111C	UWOPS87_ctrl_hmg124h_4.JPG.dat	1250.3	7.6	1.0	0.01	#NUM!	2.4E-07
YER113C	YER113C	UWOPS87_ctrl_hmg124h_4.JPG.dat	1241.3	100.4	1.0	0.08	#NUM!	1.4E-04
YER114C	YER114C	UWOPS87_ctrl_hmg124h_4.JPG.dat	1025.0	52.5	0.9	0.06	#NUM!	6.8E-05
YER115C	YER115C	UWOPS87_ctrl_hmg124h_4.JPG.dat	1219.8	9.3	1.1	0.01	#NUM!	3.6E-07
YER116C	YER116C	UWOPS87_ctrl_hmg124h_4.JPG.dat	1109.3	26.0	1.0	0.02	#NUM!	3.6E-06
YER117W	YER117W	UWOPS87_ctrl_hmg124h_4.JPG.dat	1349.8	64.6	1.2	0.01	#NUM!	9.1E-06
YER118C	YER118C	UWOPS87_ctrl_hmg124h_4.JPG.dat	1167.5	89.9	1.0	0.08	#NUM!	1.3E-04
YER119C	YER119C	UWOPS87_ctrl_hmg124h_4.JPG.dat	1298.8	49.9	1.1	0.03	#NUM!	8.1E-06
YER119C-A	YER119C-A	UWOPS87_ctrl_hmg124h_4.JPG.dat	1198.5	57.2	1.0	0.04	#NUM!	2.3E-05
YER120W	YER120W	UWOPS87_ctrl_hmg124h_4.JPG.dat	1275.3	58.4	1.1	0.06	#NUM!	4.9E-05
YER121W	YER121W	UWOPS87_ctrl_hmg124h_4.JPG.dat	1188.3	74.0	1.0	0.06	#NUM!	4.9E-05
YER123W	YER123W	UWOPS87_ctrl_hmg124h_4.JPG.dat	1129.8	101.8	1.0	0.07	#NUM!	1.2E-04
YER124C	YER124C	UWOPS87_ctrl_hmg124h_4.JPG.dat	1272.0	138.0	1.2	0.04	#NUM!	4.0E-04
YER128W	YER128W	UWOPS87_ctrl_hmg124h_4.JPG.dat	1313.3	170.7	1.2	0.17	#NUM!	6.9E-04
YER129W	YER129W	UWOPS87_ctrl_hmg124h_4.JPG.dat	1124.0	101.5	1.0	0.09	#NUM!	2.1E-04
YER130C	YER130C	UWOPS87_ctrl_hmg124h_4.JPG.dat	1073.0	92.2	1.0	0.08	#NUM!	1.7E-04
YER131W	YER131W	UWOPS87_ctrl_hmg124h_4.JPG.dat	936.3	33.2	0.9	0.03	#NUM!	1.3E-05
YER132C	YER132C	UWOPS87_ctrl_hmg124h_4.JPG.dat	1219.5	74.0	1.1	0.07	#NUM!	6.2E-05
YER134C	YER134C	UWOPS87_ctrl_hmg124h_4.JPG.dat	1094.0	131.6	1.0	0.12	#NUM!	4.8E-04
YER135C	YER135C	UWOPS87_ctrl_hmg124h_4.JPG.dat	1215.3	46.3	1.1	0.01	#NUM!	6.8E-05
YER137C	YER137C	UWOPS87_ctrl_hmg124h_4.JPG.dat	1124.5	75.2	1.0	0.07	#NUM!	1.1E-04
YER139C	YER139C	UWOPS87_ctrl_hmg124h_4.JPG.dat	1271.5	66.1	1.2	0.05	#NUM!	2.6E-05
YER140W	YER140W	UWOPS87_ctrl_hmg124h_4.JPG.dat	1221.5	58.8	1.1	0.02	#NUM!	6.7E-05
YER142C	YER142C	UWOPS87_ctrl_hmg124h_4.JPG.dat	765.0	519.2	0.9	0.12	#NUM!	5.3E-03
YER143W	YER143W	UWOPS87_ctrl_hmg124h_4.JPG.dat	1269.0	52.7	1.1	0.01	#NUM!	5.0E-05
YER144C	YER144C	UWOPS87_ctrl_hmg124h_4.JPG.dat	1296.5	21.4	1.0	0.02	#NUM!	1.2E-06
YER145C	YER145C	UWOPS87_ctrl_hmg124h_4.JPG.dat	1251.5	20.9	1.0	0.00	#NUM!	3.6E-07
YER149C	YER149C	UWOPS87_ctrl_hmg124h_4.JPG.dat	1276.3	87.4	1.0	0.07	#NUM!	9.9E-05
YER150W	YER150W	UWOPS87_ctrl_hmg124h_4.JPG.dat	1415.3	40.0	1.1	0.02	#NUM!	2.2E-06
YER151C	YER151C	UWOPS87_ctrl_hmg124h_4.JPG.dat	1269.8	42.7	1.0	0.03	#NUM!	1.1E-05
YER152C	YER152C	UWOPS87_ctrl_hmg124h_4.JPG.dat	1304.3	50.9	1.0	0.01	#NUM!	8.6E-06
YER153C	YER153C	UWOPS87_ctrl_hmg124h_4.JPG.dat	1079.8	727.9	1.1	0.10	#NUM!	2.5E-03
YER155C	YER155C	UWOPS87_ctrl_hmg124h_4.JPG.dat	962.3	35.8	0.8	0.03	#NUM!	1.8E-05
YER156C	YER156C	UWOPS87_ctrl_hmg124h_4.JPG.dat	1121.5	120.8	0.8	0.01	#NUM!	9.3E-05
YER158C	YER158C	UWOPS87_ctrl_hmg124h_4.JPG.dat	1332.5	228.3	1.1	0.17	#NUM!	1.2E-03
YER161C	YER161C	UWOPS87_ctrl_hmg124h_4.JPG.dat	961.3	32.9	0.9	0.03	#NUM!	1.6E-05
YER162C	YER162C	UWOPS87_ctrl_hmg124h_4.JPG.dat	1204.0	34.9	1.0	0.01	#NUM!	2.7E-05
YER163C	YER163C	UWOPS87_ctrl_hmg124h_4.JPG.dat	1090.0	49.6	0.9	0.04	#NUM!	2.3E-05
YER164W	YER164W	UWOPS87_ctrl_hmg124h_4.JPG.dat	1022.8	53.8	0.9	0.05	#NUM!	4.0E-05
YER166W	YER166W	UWOPS87_ctrl_hmg124h_4.JPG.dat	1117.8	55.5	0.9	0.01	#NUM!	7.7E-05

YER167W	YER167W	UWOPS87_ctrl_hmg124h_4.JPG.dat	1366.5	21.9	1.1	0.01	#NUM!	6.3E-07
YER170W	YER170W	UWOPS87_ctrl_hmg124h_4.JPG.dat	1292.0	55.2	1.0	0.04	#NUM!	1.8E-05
YER173W	YER173W	UWOPS87_ctrl_hmg124h_4.JPG.dat	1134.5	35.7	0.9	0.03	#NUM!	1.0E-05
YER174C	YER174C	UWOPS87_ctrl_hmg124h_4.JPG.dat	1175.0	11.0	1.0	0.01	#NUM!	1.2E-07
YER175C	YER175C	UWOPS87_ctrl_hmg124h_4.JPG.dat	1259.8	19.6	1.0	0.00	#NUM!	2.3E-06
YER176W	YER176W	UWOPS87_ctrl_hmg124h_4.JPG.dat	1357.0	113.6	1.1	0.01	#NUM!	1.3E-05
YER177W	YER177W	UWOPS87_ctrl_hmg124h_4.JPG.dat	1110.8	31.6	1.0	0.03	#NUM!	1.1E-05
YER178W	YER178W	UWOPS87_ctrl_hmg124h_4.JPG.dat	1086.3	66.4	0.9	0.05	#NUM!	5.4E-05
YER179W	YER179W	UWOPS87_ctrl_hmg124h_4.JPG.dat	995.0	80.6	0.9	0.07	#NUM!	1.7E-04
YER180C	YER180C	UWOPS87_ctrl_hmg124h_4.JPG.dat	1233.8	34.9	1.0	0.03	#NUM!	7.0E-06
YER181C	YER181C	UWOPS87_ctrl_hmg124h_4.JPG.dat	1259.8	20.3	1.0	0.01	#NUM!	5.5E-07
YER182W	YER182W	UWOPS87_ctrl_hmg124h_4.JPG.dat	1102.3	243.6	1.0	0.05	#NUM!	1.0E-03
YER183C	YER183C	UWOPS87_ctrl_hmg124h_4.JPG.dat	1192.8	67.5	1.0	0.06	#NUM!	5.5E-05
YER184C	YER184C	UWOPS87_ctrl_hmg124h_4.JPG.dat	1263.5	23.8	1.0	0.01	#NUM!	7.0E-07
YER185W	YER185W	UWOPS87_ctrl_hmg124h_4.JPG.dat	1136.0	60.0	1.0	0.05	#NUM!	3.7E-05
YER186C	YER186C	UWOPS87_ctrl_hmg124h_4.JPG.dat	1142.8	53.6	0.9	0.04	#NUM!	2.5E-05
YER187W	YER187W	UWOPS87_ctrl_hmg124h_4.JPG.dat	1242.8	40.7	1.0	0.04	#NUM!	1.3E-05
YER188W	YER188W	UWOPS87_ctrl_hmg124h_4.JPG.dat	1202.8	114.7	1.1	0.10	#NUM!	2.7E-04
YFL006W	YFL006W	UWOPS87_ctrl_hmg124h_4.JPG.dat	1203.5	45.6	1.0	0.03	#NUM!	1.0E-05
YFL011W	YFL011W	UWOPS87_ctrl_hmg124h_4.JPG.dat	1221.8	46.0	1.0	0.04	#NUM!	1.3E-05
YFL013W-/	YFL013W-A	UWOPS87_ctrl_hmg124h_4.JPG.dat	1144.0	118.8	1.0	0.10	#NUM!	2.9E-04
YFL014W	YFL014W	UWOPS87_ctrl_hmg124h_4.JPG.dat	1153.5	41.8	0.9	0.03	#NUM!	1.1E-05
YFL015C	YFL015C	UWOPS87_ctrl_hmg124h_4.JPG.dat	1314.5	62.7	1.1	0.05	#NUM!	3.1E-05
YFL018C	YFL018C	UWOPS87_ctrl_hmg124h_4.JPG.dat	418.5	640.0	0.1	0.15	#NUM!	4.2E-01
YFL019C	YFL019C	UWOPS87_ctrl_hmg124h_4.JPG.dat	1066.8	65.2	0.9	0.06	#NUM!	8.0E-05
YFL020C	YFL020C	UWOPS87_ctrl_hmg124h_4.JPG.dat	1087.3	25.8	0.9	0.02	#NUM!	3.7E-06
YFL021W	YFL021W	UWOPS87_ctrl_hmg124h_4.JPG.dat	1137.3	99.1	0.9	0.08	#NUM!	1.9E-04
YFL023W	YFL023W	UWOPS87_ctrl_hmg124h_4.JPG.dat	916.0	77.8	0.8	0.06	#NUM!	1.6E-04
YFL025C	YFL025C	UWOPS87_ctrl_hmg124h_4.JPG.dat	984.3	37.1	0.9	0.03	#NUM!	1.2E-05
YFL026W	YFL026W	UWOPS87_ctrl_hmg124h_4.JPG.dat	952.0	76.5	0.8	0.07	#NUM!	1.6E-04
YFL027C	YFL027C	UWOPS87_ctrl_hmg124h_4.JPG.dat	1124.0	63.9	1.0	0.05	#NUM!	4.9E-05
YFL028C	YFL028C	UWOPS87_ctrl_hmg124h_4.JPG.dat	1072.3	8.9	0.9	0.01	#NUM!	3.3E-07
YFL030W	YFL030W	UWOPS87_ctrl_hmg124h_4.JPG.dat	1249.0	10.8	1.0	0.01	#NUM!	2.2E-07
YFL031W	YFL031W	UWOPS87_ctrl_hmg124h_4.JPG.dat	1125.8	28.1	0.9	0.03	#NUM!	5.8E-06
YFL032W	YFL032W	UWOPS87_ctrl_hmg124h_4.JPG.dat	591.3	582.8	0.5	0.48	#NUM!	1.3E-01
YFL034C-A	YFL034C-A	UWOPS87_ctrl_hmg124h_4.JPG.dat	1172.0	75.2	0.9	0.06	#NUM!	6.8E-05
YFL034W	YFL034W	UWOPS87_ctrl_hmg124h_4.JPG.dat	1093.0	187.0	0.9	0.15	#NUM!	1.2E-03
YFL035C-B	YFL035C-B	UWOPS87_ctrl_hmg124h_4.JPG.dat	1266.5	29.6	1.0	0.03	#NUM!	4.1E-06
YFL036W	YFL036W	UWOPS87_ctrl_hmg124h_4.JPG.dat	1245.3	80.7	1.0	0.02	#NUM!	1.1E-04
YFL040W	YFL040W	UWOPS87_ctrl_hmg124h_4.JPG.dat	1045.8	42.6	1.0	0.04	#NUM!	2.4E-05
YFL041W	YFL041W	UWOPS87_ctrl_hmg124h_4.JPG.dat	1104.5	35.6	1.0	0.04	#NUM!	1.8E-05
YFL042C	YFL042C	UWOPS87_ctrl_hmg124h_4.JPG.dat	1157.3	80.6	1.0	0.07	#NUM!	6.8E-05
YFL043C	YFL043C	UWOPS87_ctrl_hmg124h_4.JPG.dat	967.8	30.3	0.9	0.01	#NUM!	1.2E-05
YFL044C	YFL044C	UWOPS87_ctrl_hmg124h_4.JPG.dat	1065.8	83.0	0.9	0.07	#NUM!	1.3E-04
YFL046W	YFL046W	UWOPS87_ctrl_hmg124h_4.JPG.dat	1028.3	14.2	0.9	0.01	#NUM!	1.7E-05
YFL047W	YFL047W	UWOPS87_ctrl_hmg124h_4.JPG.dat	1042.3	39.8	0.9	0.03	#NUM!	1.2E-05
YFL048C	YFL048C	UWOPS87_ctrl_hmg124h_4.JPG.dat	1278.0	49.6	1.1	0.01	#NUM!	4.0E-05
YFL049W	YFL049W	UWOPS87_ctrl_hmg124h_4.JPG.dat	1161.8	40.8	1.0	0.04	#NUM!	2.1E-05
YFL050C	YFL050C	UWOPS87_ctrl_hmg124h_4.JPG.dat	1075.8	147.3	0.9	0.14	#NUM!	9.6E-04
YFL051C	YFL051C	UWOPS87_ctrl_hmg124h_4.JPG.dat	1093.3	98.4	1.0	0.08	#NUM!	1.5E-04
YFL052W	YFL052W	UWOPS87_ctrl_hmg124h_4.JPG.dat	827.0	65.3	0.8	0.07	#NUM!	1.8E-04
YFL053W	YFL053W	UWOPS87_ctrl_hmg124h_4.JPG.dat	764.0	518.1	0.9	0.11	#NUM!	4.4E-03
YFL054C	YFL054C	UWOPS87_ctrl_hmg124h_4.JPG.dat	848.3	565.5	1.1	0.00	#NUM!	2.6E-06
YFL055W	YFL055W	UWOPS87_ctrl_hmg124h_4.JPG.dat	1140.5	31.8	1.0	0.03	#NUM!	6.1E-06
YFL056C	YFL056C	UWOPS87_ctrl_hmg124h_4.JPG.dat	1108.5	61.5	1.0	0.05	#NUM!	3.9E-05
YFR006W	YFR006W	UWOPS87_ctrl_hmg124h_4.JPG.dat	1101.8	59.8	1.0	0.05	#NUM!	4.0E-05
YFR007W	YFR007W	UWOPS87_ctrl_hmg124h_4.JPG.dat	1152.5	13.3	1.0	0.01	#NUM!	4.0E-07
YFR008W	YFR008W	UWOPS87_ctrl_hmg124h_4.JPG.dat	1204.0	50.1	1.0	0.02	#NUM!	7.3E-05
YFR009W	YFR009W	UWOPS87_ctrl_hmg124h_4.JPG.dat	714.8	480.2	0.9	0.08	#NUM!	2.3E-03
YFR010W	YFR010W	UWOPS87_ctrl_hmg124h_4.JPG.dat	724.8	500.8	0.9	0.15	#NUM!	9.3E-03
YFR011C	YFR011C	UWOPS87_ctrl_hmg124h_4.JPG.dat	1017.3	71.5	0.9	0.07	#NUM!	1.1E-04
YOR202W	YOR202W	UWOPS87_ctrl_hmg124h_4.JPG.dat	1341.1	186.4	1.0	0.08	#NUM!	0.0E+00
1	1	UWOPS87_ctrl_hmg124h_5.JPG.dat	1356.0	319.4	0.9	0.15	#NUM!	1.1E-03
2	2	UWOPS87_ctrl_hmg124h_5.JPG.dat	1216.3	204.1	1.0	0.13	#NUM!	6.9E-04
3	3	UWOPS87_ctrl_hmg124h_5.JPG.dat	1298.5	183.9	1.0	0.10	#NUM!	2.5E-04
4	4	UWOPS87_ctrl_hmg124h_5.JPG.dat	1270.8	139.1	1.0	0.06	#NUM!	6.6E-05
YFR012W	YFR012W	UWOPS87_ctrl_hmg124h_5.JPG.dat	1165.8	86.9	1.1	0.09	#NUM!	1.3E-04
YFR013W	YFR013W	UWOPS87_ctrl_hmg124h_5.JPG.dat	950.3	156.6	0.8	0.01	#NUM!	1.0E-04
YFR014C	YFR014C	UWOPS87_ctrl_hmg124h_5.JPG.dat	1044.5	58.1	1.0	0.05	#NUM!	3.1E-05
YFR015C	YFR015C	UWOPS87_ctrl_hmg124h_5.JPG.dat	1099.5	82.1	1.0	0.07	#NUM!	8.9E-05

YFR016C	YFR016C	UWOPS87_ctrl_hmg124h_5.JPG.dat	1132.8	35.4	1.0	0.02	#NUM!	3.9E-06
YFR017C	YFR017C	UWOPS87_ctrl_hmg124h_5.JPG.dat	1190.0	139.7	1.1	0.11	#NUM!	2.9E-04
YFR018C	YFR018C	UWOPS87_ctrl_hmg124h_5.JPG.dat	1315.0	26.2	1.1	0.02	#NUM!	1.1E-06
YFR019W	YFR019W	UWOPS87_ctrl_hmg124h_5.JPG.dat	1078.3	41.7	0.9	0.03	#NUM!	9.0E-06
YFR020W	YFR020W	UWOPS87_ctrl_hmg124h_5.JPG.dat	1256.3	20.1	1.1	0.00	#NUM!	1.3E-06
YFR021W	YFR021W	UWOPS87_ctrl_hmg124h_5.JPG.dat	1137.0	77.9	1.0	0.06	#NUM!	8.0E-05
YFR022W	YFR022W	UWOPS87_ctrl_hmg124h_5.JPG.dat	1216.0	143.0	1.1	0.11	#NUM!	3.1E-04
YFR023W	YFR023W	UWOPS87_ctrl_hmg124h_5.JPG.dat	1048.8	85.4	1.0	0.08	#NUM!	1.4E-04
YFR024C	YFR024C	UWOPS87_ctrl_hmg124h_5.JPG.dat	1104.0	58.4	0.9	0.05	#NUM!	3.0E-05
YFR024C-A	YFR024C-A	UWOPS87_ctrl_hmg124h_5.JPG.dat	1143.0	68.2	1.0	0.07	#NUM!	1.0E-04
YFR025C	YFR025C	UWOPS87_ctrl_hmg124h_5.JPG.dat	0.0	0.0	0.0	0.00	#NUM!	
YFR026C	YFR026C	UWOPS87_ctrl_hmg124h_5.JPG.dat	1359.5	66.1	1.1	0.06	#NUM!	5.0E-05
YFR030W	YFR030W	UWOPS87_ctrl_hmg124h_5.JPG.dat	0.0	0.0	0.0	0.00	#NUM!	
YFR031C-A	YFR031C-A	UWOPS87_ctrl_hmg124h_5.JPG.dat	1118.0	27.1	0.9	0.02	#NUM!	2.9E-06
YFR032C	YFR032C	UWOPS87_ctrl_hmg124h_5.JPG.dat	1190.5	49.5	1.0	0.04	#NUM!	2.2E-05
YFR032C-A	YFR032C-A	UWOPS87_ctrl_hmg124h_5.JPG.dat	1220.8	23.0	1.0	0.01	#NUM!	1.9E-05
YFR033C	YFR033C	UWOPS87_ctrl_hmg124h_5.JPG.dat	1164.3	16.1	0.9	0.01	#NUM!	5.8E-07
YFR034C	YFR034C	UWOPS87_ctrl_hmg124h_5.JPG.dat	1325.3	90.6	1.1	0.01	#NUM!	3.6E-05
YFR035C	YFR035C	UWOPS87_ctrl_hmg124h_5.JPG.dat	957.3	44.8	1.0	0.01	#NUM!	2.1E-05
YFR038W	YFR038W	UWOPS87_ctrl_hmg124h_5.JPG.dat	1367.0	85.8	1.2	0.09	#NUM!	1.1E-04
YFR039C	YFR039C	UWOPS87_ctrl_hmg124h_5.JPG.dat	1274.3	92.5	1.1	0.08	#NUM!	1.0E-04
YFR040W	YFR040W	UWOPS87_ctrl_hmg124h_5.JPG.dat	1113.8	69.4	0.9	0.05	#NUM!	4.2E-05
YFR041C	YFR041C	UWOPS87_ctrl_hmg124h_5.JPG.dat	1282.3	47.4	1.0	0.01	#NUM!	2.6E-05
YFR043C	YFR043C	UWOPS87_ctrl_hmg124h_5.JPG.dat	0.0	0.0	0.0	0.00	#NUM!	
YFR044C	YFR044C	UWOPS87_ctrl_hmg124h_5.JPG.dat	1265.0	58.3	1.0	0.01	#NUM!	2.9E-05
YFR045W	YFR045W	UWOPS87_ctrl_hmg124h_5.JPG.dat	1.3	2.5	0.0	0.00	#NUM!	
YFR046C	YFR046C	UWOPS87_ctrl_hmg124h_5.JPG.dat	1057.3	38.6	0.9	0.04	#NUM!	2.4E-05
YFR047C	YFR047C	UWOPS87_ctrl_hmg124h_5.JPG.dat	1000.5	47.1	0.9	0.04	#NUM!	3.2E-05
YFR048W	YFR048W	UWOPS87_ctrl_hmg124h_5.JPG.dat	983.8	36.2	0.9	0.05	#NUM!	4.8E-05
YFR049W	YFR049W	UWOPS87_ctrl_hmg124h_5.JPG.dat	1221.5	47.9	1.2	0.06	#NUM!	2.8E-05
YFR053C	YFR053C	UWOPS87_ctrl_hmg124h_5.JPG.dat	1153.8	163.5	1.0	0.13	#NUM!	6.3E-04
YFR054C	YFR054C	UWOPS87_ctrl_hmg124h_5.JPG.dat	1160.5	48.4	1.0	0.04	#NUM!	2.4E-05
YFR055W	YFR055W	UWOPS87_ctrl_hmg124h_5.JPG.dat	1155.8	49.1	0.9	0.04	#NUM!	2.3E-05
YFR056C	YFR056C	UWOPS87_ctrl_hmg124h_5.JPG.dat	1166.3	110.0	1.0	0.08	#NUM!	1.8E-04
YFR057W	YFR057W	UWOPS87_ctrl_hmg124h_5.JPG.dat	1201.3	93.8	1.0	0.02	#NUM!	1.5E-04
YGL002W	YGL002W	UWOPS87_ctrl_hmg124h_5.JPG.dat	1329.0	72.4	1.1	0.06	#NUM!	3.8E-05
YGL004C	YGL004C	UWOPS87_ctrl_hmg124h_5.JPG.dat	1105.5	67.1	0.9	0.02	#NUM!	9.0E-05
YGL005C	YGL005C	UWOPS87_ctrl_hmg124h_5.JPG.dat	1216.5	42.9	1.1	0.04	#NUM!	1.6E-05
YGL006W	YGL006W	UWOPS87_ctrl_hmg124h_5.JPG.dat	1077.8	37.2	1.0	0.04	#NUM!	1.4E-05
YGL007W	YGL007W	UWOPS87_ctrl_hmg124h_5.JPG.dat	881.8	192.8	0.7	0.05	#NUM!	1.8E-03
YGL009C	YGL009C	UWOPS87_ctrl_hmg124h_5.JPG.dat	0.0	0.0	0.0	0.00	#NUM!	
YGL010W	YGL010W	UWOPS87_ctrl_hmg124h_5.JPG.dat	1154.3	19.5	1.0	0.01	#NUM!	2.0E-05
YGL013C	YGL013C	UWOPS87_ctrl_hmg124h_5.JPG.dat	1086.0	101.1	1.0	0.02	#NUM!	1.5E-04
YGL014W	YGL014W	UWOPS87_ctrl_hmg124h_5.JPG.dat	1220.5	57.2	1.0	0.04	#NUM!	1.9E-05
YGL015C	YGL015C	UWOPS87_ctrl_hmg124h_5.JPG.dat	1069.8	99.2	0.9	0.09	#NUM!	2.2E-04
YGL016W	YGL016W	UWOPS87_ctrl_hmg124h_5.JPG.dat	1069.8	70.5	0.9	0.01	#NUM!	7.9E-05
YGL017W	YGL017W	UWOPS87_ctrl_hmg124h_5.JPG.dat	1197.0	96.0	1.1	0.03	#NUM!	2.5E-04
YGL019W	YGL019W	UWOPS87_ctrl_hmg124h_5.JPG.dat	1160.5	20.5	1.1	0.02	#NUM!	3.3E-06
YGL020C	YGL020C	UWOPS87_ctrl_hmg124h_5.JPG.dat	1131.0	60.5	1.0	0.05	#NUM!	4.3E-05
YGL021W	YGL021W	UWOPS87_ctrl_hmg124h_5.JPG.dat	1200.5	52.6	1.1	0.02	#NUM!	1.0E-04
YGL023C	YGL023C	UWOPS87_ctrl_hmg124h_5.JPG.dat	1057.3	20.7	1.0	0.02	#NUM!	1.4E-06
YGL024W	YGL024W	UWOPS87_ctrl_hmg124h_5.JPG.dat	1063.8	60.2	1.0	0.02	#NUM!	1.5E-04
YGL026C	YGL026C	UWOPS87_ctrl_hmg124h_5.JPG.dat	0.0	0.0	0.0	0.00	#NUM!	
YGL027C	YGL027C	UWOPS87_ctrl_hmg124h_5.JPG.dat	1227.3	43.8	1.1	0.04	#NUM!	1.4E-05
YGL028C	YGL028C	UWOPS87_ctrl_hmg124h_5.JPG.dat	1084.8	75.4	0.9	0.07	#NUM!	1.0E-04
YGL031C	YGL031C	UWOPS87_ctrl_hmg124h_5.JPG.dat	1290.8	47.6	1.1	0.04	#NUM!	1.7E-05
YGL032C	YGL032C	UWOPS87_ctrl_hmg124h_5.JPG.dat	1280.8	51.9	1.1	0.05	#NUM!	2.7E-05
YGL034C	YGL034C	UWOPS87_ctrl_hmg124h_5.JPG.dat	1223.3	72.8	1.1	0.06	#NUM!	5.9E-05
YGL035C	YGL035C	UWOPS87_ctrl_hmg124h_5.JPG.dat	1088.3	74.6	1.0	0.00	#NUM!	6.2E-07
YGL036W	YGL036W	UWOPS87_ctrl_hmg124h_5.JPG.dat	1125.8	13.6	1.0	0.01	#NUM!	7.5E-07
YGL039W	YGL039W	UWOPS87_ctrl_hmg124h_5.JPG.dat	1099.3	45.3	1.0	0.01	#NUM!	3.1E-05
YGL041C	YGL041C	UWOPS87_ctrl_hmg124h_5.JPG.dat	1157.5	68.8	1.0	0.05	#NUM!	3.0E-05
YGL043W	YGL043W	UWOPS87_ctrl_hmg124h_5.JPG.dat	999.5	71.1	0.9	0.02	#NUM!	9.6E-05
YGL045W	YGL045W	UWOPS87_ctrl_hmg124h_5.JPG.dat	1121.3	64.6	1.0	0.01	#NUM!	1.2E-05
YGL046W	YGL046W	UWOPS87_ctrl_hmg124h_5.JPG.dat	1204.3	57.6	1.1	0.05	#NUM!	2.6E-05
YGL050W	YGL050W	UWOPS87_ctrl_hmg124h_5.JPG.dat	1092.5	96.9	1.0	0.08	#NUM!	1.5E-04
YGL051W	YGL051W	UWOPS87_ctrl_hmg124h_5.JPG.dat	1040.5	118.9	0.9	0.11	#NUM!	4.2E-04
YGL053W	YGL053W	UWOPS87_ctrl_hmg124h_5.JPG.dat	1205.3	22.6	1.1	0.02	#NUM!	1.9E-06
YGL054C	YGL054C	UWOPS87_ctrl_hmg124h_5.JPG.dat	1056.3	64.0	1.0	0.06	#NUM!	7.7E-05
YGL056C	YGL056C	UWOPS87_ctrl_hmg124h_5.JPG.dat	1144.3	103.4	1.1	0.09	#NUM!	1.7E-04

YGL057C	YGL057C	UWOPS87_ctrl_hmg124h_5.JPG.dat	1071.0	48.7	1.0	0.04	#NUM!	2.7E-05
YGL058W	YGL058W	UWOPS87_ctrl_hmg124h_5.JPG.dat	1098.5	33.6	1.0	0.03	#NUM!	5.2E-06
YGL059W	YGL059W	UWOPS87_ctrl_hmg124h_5.JPG.dat	1079.8	86.0	0.9	0.08	#NUM!	1.5E-04
YGL060W	YGL060W	UWOPS87_ctrl_hmg124h_5.JPG.dat	1154.0	111.7	1.1	0.03	#NUM!	2.0E-04
YGL062W	YGL062W	UWOPS87_ctrl_hmg124h_5.JPG.dat	933.0	76.1	0.9	0.08	#NUM!	1.7E-04
YGL066W	YGL066W	UWOPS87_ctrl_hmg124h_5.JPG.dat	1056.3	154.4	1.0	0.15	#NUM!	9.6E-04
YGL067W	YGL067W	UWOPS87_ctrl_hmg124h_5.JPG.dat	1066.3	104.2	1.0	0.08	#NUM!	1.6E-04
YGL077C	YGL077C	UWOPS87_ctrl_hmg124h_5.JPG.dat	1191.5	40.0	1.1	0.01	#NUM!	3.6E-05
YGL078C	YGL078C	UWOPS87_ctrl_hmg124h_5.JPG.dat	971.5	14.0	0.9	0.02	#NUM!	3.9E-06
YGL079W	YGL079W	UWOPS87_ctrl_hmg124h_5.JPG.dat	1118.3	50.2	1.0	0.01	#NUM!	4.1E-05
YGL080W	YGL080W	UWOPS87_ctrl_hmg124h_5.JPG.dat	1093.0	37.0	1.0	0.01	#NUM!	3.8E-05
YGL081W	YGL081W	UWOPS87_ctrl_hmg124h_5.JPG.dat	1290.5	93.5	1.1	0.03	#NUM!	2.8E-04
YGL082W	YGL082W	UWOPS87_ctrl_hmg124h_5.JPG.dat	1304.0	62.4	1.1	0.06	#NUM!	5.4E-05
YGL083W	YGL083W	UWOPS87_ctrl_hmg124h_5.JPG.dat	1155.5	75.2	1.0	0.00	#NUM!	4.1E-06
YGL084C	YGL084C	UWOPS87_ctrl_hmg124h_5.JPG.dat	1091.5	61.9	1.0	0.01	#NUM!	2.4E-05
YGL085W	YGL085W	UWOPS87_ctrl_hmg124h_5.JPG.dat	1220.5	34.6	1.1	0.03	#NUM!	5.7E-06
YGL086W	YGL086W	UWOPS87_ctrl_hmg124h_5.JPG.dat	1181.0	13.4	1.0	0.00	#NUM!	9.1E-09
YGL087C	YGL087C	UWOPS87_ctrl_hmg124h_5.JPG.dat	1348.8	65.1	1.1	0.04	#NUM!	1.2E-05
YGL089C	YGL089C	UWOPS87_ctrl_hmg124h_5.JPG.dat	1339.3	58.8	1.1	0.04	#NUM!	1.0E-05
YGL090W	YGL090W	UWOPS87_ctrl_hmg124h_5.JPG.dat	1284.0	46.9	1.0	0.01	#NUM!	9.8E-06
YGL101W	YGL101W	UWOPS87_ctrl_hmg124h_5.JPG.dat	1254.5	64.7	1.0	0.04	#NUM!	2.4E-05
YGL104C	YGL104C	UWOPS87_ctrl_hmg124h_5.JPG.dat	1231.0	21.5	1.0	0.02	#NUM!	3.1E-06
YGL105W	YGL105W	UWOPS87_ctrl_hmg124h_5.JPG.dat	0.0	0.0	0.0	0.00	#NUM!	
YGL108C	YGL108C	UWOPS87_ctrl_hmg124h_5.JPG.dat	1305.3	66.7	1.1	0.05	#NUM!	3.1E-05
YGL109W	YGL109W	UWOPS87_ctrl_hmg124h_5.JPG.dat	1336.3	67.5	1.1	0.05	#NUM!	3.6E-05
YGL110C	YGL110C	UWOPS87_ctrl_hmg124h_5.JPG.dat	947.3	72.1	0.9	0.07	#NUM!	1.3E-04
YGL114W	YGL114W	UWOPS87_ctrl_hmg124h_5.JPG.dat	1126.5	62.7	1.1	0.04	#NUM!	2.1E-05
YGL115W	YGL115W	UWOPS87_ctrl_hmg124h_5.JPG.dat	814.8	641.8	0.7	0.57	#NUM!	8.6E-02
YGL117W	YGL117W	UWOPS87_ctrl_hmg124h_5.JPG.dat	1249.8	63.2	1.0	0.04	#NUM!	1.3E-05
YGL118C	YGL118C	UWOPS87_ctrl_hmg124h_5.JPG.dat	1345.8	51.5	1.1	0.03	#NUM!	7.5E-06
YGL121C	YGL121C	UWOPS87_ctrl_hmg124h_5.JPG.dat	1396.3	51.7	1.1	0.01	#NUM!	3.2E-05
YGL124C	YGL124C	UWOPS87_ctrl_hmg124h_5.JPG.dat	1344.5	86.2	1.1	0.08	#NUM!	1.0E-04
YGL125W	YGL125W	UWOPS87_ctrl_hmg124h_5.JPG.dat	1324.0	43.8	1.1	0.03	#NUM!	4.5E-06
YGL126W	YGL126W	UWOPS87_ctrl_hmg124h_5.JPG.dat	1258.3	58.9	1.1	0.05	#NUM!	2.3E-05
YGL127C	YGL127C	UWOPS87_ctrl_hmg124h_5.JPG.dat	372.8	433.6	0.3	0.39	#NUM!	1.8E-01
YGL131C	YGL131C	UWOPS87_ctrl_hmg124h_5.JPG.dat	1073.3	73.7	0.9	0.02	#NUM!	1.5E-04
YGL132W	YGL132W	UWOPS87_ctrl_hmg124h_5.JPG.dat	1049.0	38.5	1.0	0.05	#NUM!	2.8E-05
YGL133W	YGL133W	UWOPS87_ctrl_hmg124h_5.JPG.dat	1038.8	51.5	1.0	0.04	#NUM!	2.0E-05
YGL136C	YGL136C	UWOPS87_ctrl_hmg124h_5.JPG.dat	1175.5	40.9	1.0	0.04	#NUM!	1.1E-05
YGL138C	YGL138C	UWOPS87_ctrl_hmg124h_5.JPG.dat	1289.0	38.3	1.1	0.01	#NUM!	2.6E-05
YGL139W	YGL139W	UWOPS87_ctrl_hmg124h_5.JPG.dat	1314.3	32.4	1.1	0.03	#NUM!	5.8E-06
YGL140C	YGL140C	UWOPS87_ctrl_hmg124h_5.JPG.dat	1332.8	52.2	1.1	0.04	#NUM!	1.0E-05
YGL141W	YGL141W	UWOPS87_ctrl_hmg124h_5.JPG.dat	1048.0	40.6	0.9	0.03	#NUM!	1.6E-05
YGL144C	YGL144C	UWOPS87_ctrl_hmg124h_5.JPG.dat	1030.5	36.7	0.9	0.04	#NUM!	3.1E-05
YGL146C	YGL146C	UWOPS87_ctrl_hmg124h_5.JPG.dat	1276.3	52.2	1.1	0.05	#NUM!	1.9E-05
YGL147C	YGL147C	UWOPS87_ctrl_hmg124h_5.JPG.dat	1037.3	51.2	0.9	0.01	#NUM!	8.8E-05
YGL148W	YGL148W	UWOPS87_ctrl_hmg124h_5.JPG.dat	1077.0	22.7	1.0	0.02	#NUM!	2.5E-06
YGL149W	YGL149W	UWOPS87_ctrl_hmg124h_5.JPG.dat	997.3	43.6	1.0	0.05	#NUM!	3.9E-05
YGL151W	YGL151W	UWOPS87_ctrl_hmg124h_5.JPG.dat	881.8	83.8	0.8	0.07	#NUM!	1.8E-04
YGL152C	YGL152C	UWOPS87_ctrl_hmg124h_5.JPG.dat	1257.3	37.4	1.1	0.03	#NUM!	8.6E-06
YGL153W	YGL153W	UWOPS87_ctrl_hmg124h_5.JPG.dat	1166.3	81.1	1.0	0.06	#NUM!	7.3E-05
YGL154C	YGL154C	UWOPS87_ctrl_hmg124h_5.JPG.dat	1085.8	77.0	0.9	0.06	#NUM!	7.7E-05
YGL156W	YGL156W	UWOPS87_ctrl_hmg124h_5.JPG.dat	1235.3	128.7	1.0	0.11	#NUM!	2.9E-04
YGL157W	YGL157W	UWOPS87_ctrl_hmg124h_5.JPG.dat	1239.8	73.0	1.1	0.07	#NUM!	7.2E-05
YGL158W	YGL158W	UWOPS87_ctrl_hmg124h_5.JPG.dat	1089.8	60.6	1.0	0.05	#NUM!	3.1E-05
YGL159W	YGL159W	UWOPS87_ctrl_hmg124h_5.JPG.dat	1105.8	61.2	1.0	0.05	#NUM!	3.8E-05
YGL160W	YGL160W	UWOPS87_ctrl_hmg124h_5.JPG.dat	1133.8	40.7	1.0	0.04	#NUM!	1.3E-05
YGL161C	YGL161C	UWOPS87_ctrl_hmg124h_5.JPG.dat	1060.8	35.3	0.9	0.03	#NUM!	1.2E-05
YGL162W	YGL162W	UWOPS87_ctrl_hmg124h_5.JPG.dat	1017.3	99.4	1.0	0.10	#NUM!	2.8E-04
YGL163C	YGL163C	UWOPS87_ctrl_hmg124h_5.JPG.dat	1036.5	106.0	1.0	0.02	#NUM!	1.6E-04
YGL164C	YGL164C	UWOPS87_ctrl_hmg124h_5.JPG.dat	1301.3	18.1	1.1	0.02	#NUM!	1.0E-06
YGL165C	YGL165C	UWOPS87_ctrl_hmg124h_5.JPG.dat	1129.0	48.1	1.0	0.04	#NUM!	2.0E-05
YGL166W	YGL166W	UWOPS87_ctrl_hmg124h_5.JPG.dat	1062.5	63.3	0.9	0.05	#NUM!	5.6E-05
YGL167C	YGL167C	UWOPS87_ctrl_hmg124h_5.JPG.dat	1171.3	53.6	1.0	0.04	#NUM!	2.4E-05
YGL168W	YGL168W	UWOPS87_ctrl_hmg124h_5.JPG.dat	1098.3	39.5	1.0	0.03	#NUM!	1.1E-05
YGL170C	YGL170C	UWOPS87_ctrl_hmg124h_5.JPG.dat	1094.5	16.1	1.0	0.02	#NUM!	1.2E-06
YGL173C	YGL173C	UWOPS87_ctrl_hmg124h_5.JPG.dat	1134.0	22.8	1.0	0.02	#NUM!	2.2E-06
YGL174W	YGL174W	UWOPS87_ctrl_hmg124h_5.JPG.dat	0.0	0.0	0.0	0.00	#NUM!	
YGL175C	YGL175C	UWOPS87_ctrl_hmg124h_5.JPG.dat	865.5	578.9	1.0	0.04	#NUM!	6.1E-04
YGL176C	YGL176C	UWOPS87_ctrl_hmg124h_5.JPG.dat	1058.5	81.6	1.0	0.07	#NUM!	1.1E-04

YGL177W	YGL177W	UWOPS87_ctrl_hmg124h_5.JPG.dat	1110.3	131.1	1.0	0.12	#NUM!	4.2E-04
YGL179C	YGL179C	UWOPS87_ctrl_hmg124h_5.JPG.dat	1044.0	53.3	1.0	0.05	#NUM!	3.9E-05
YGL180W	YGL180W	UWOPS87_ctrl_hmg124h_5.JPG.dat	1293.8	58.0	1.2	0.01	#NUM!	1.4E-05
YGL181W	YGL181W	UWOPS87_ctrl_hmg124h_5.JPG.dat	1191.0	59.1	1.1	0.02	#NUM!	7.0E-05
YGL194C	YGL194C	UWOPS87_ctrl_hmg124h_5.JPG.dat	1244.5	16.4	1.1	0.01	#NUM!	8.8E-06
YGL195W	YGL195W	UWOPS87_ctrl_hmg124h_5.JPG.dat	1098.3	61.6	1.0	0.06	#NUM!	4.6E-05
YGL196W	YGL196W	UWOPS87_ctrl_hmg124h_5.JPG.dat	1130.3	68.2	1.0	0.02	#NUM!	9.7E-05
YGL197W	YGL197W	UWOPS87_ctrl_hmg124h_5.JPG.dat	1031.8	81.6	0.9	0.07	#NUM!	1.1E-04
YGL198W	YGL198W	UWOPS87_ctrl_hmg124h_5.JPG.dat	802.8	535.2	1.0	0.01	#NUM!	3.4E-05
YGL199C	YGL199C	UWOPS87_ctrl_hmg124h_5.JPG.dat	1026.0	123.0	0.8	0.02	#NUM!	2.1E-04
YGL202W	YGL202W	UWOPS87_ctrl_hmg124h_5.JPG.dat	1074.0	66.2	1.0	0.06	#NUM!	6.8E-05
YGL203C	YGL203C	UWOPS87_ctrl_hmg124h_5.JPG.dat	1074.8	83.8	1.0	0.08	#NUM!	1.5E-04
YGL205W	YGL205W	UWOPS87_ctrl_hmg124h_5.JPG.dat	1238.5	65.6	1.2	0.08	#NUM!	8.2E-05
YGL208W	YGL208W	UWOPS87_ctrl_hmg124h_5.JPG.dat	1196.3	136.4	1.1	0.12	#NUM!	3.7E-04
YGL209W	YGL209W	UWOPS87_ctrl_hmg124h_5.JPG.dat	1164.8	43.9	1.1	0.03	#NUM!	8.1E-06
YGL210W	YGL210W	UWOPS87_ctrl_hmg124h_5.JPG.dat	1112.0	121.5	1.0	0.10	#NUM!	3.0E-04
YGL211W	YGL211W	UWOPS87_ctrl_hmg124h_5.JPG.dat	1142.3	27.9	1.0	0.04	#NUM!	1.5E-05
YGL212W	YGL212W	UWOPS87_ctrl_hmg124h_5.JPG.dat	1082.3	62.3	0.9	0.06	#NUM!	8.2E-05
YGL213C	YGL213C	UWOPS87_ctrl_hmg124h_5.JPG.dat	1228.8	51.8	1.1	0.00	#NUM!	4.7E-07
YGL214W	YGL214W	UWOPS87_ctrl_hmg124h_5.JPG.dat	1171.5	37.3	1.0	0.02	#NUM!	3.9E-06
YGL215W	YGL215W	UWOPS87_ctrl_hmg124h_5.JPG.dat	1035.0	140.5	0.9	0.12	#NUM!	6.7E-04
YGL216W	YGL216W	UWOPS87_ctrl_hmg124h_5.JPG.dat	1015.0	67.2	1.0	0.07	#NUM!	1.2E-04
YGL217C	YGL217C	UWOPS87_ctrl_hmg124h_5.JPG.dat	1133.0	124.1	1.0	0.10	#NUM!	2.4E-04
YGL218W	YGL218W	UWOPS87_ctrl_hmg124h_5.JPG.dat	987.5	4.2	0.9	0.02	#NUM!	2.9E-06
YGL219C	YGL219C	UWOPS87_ctrl_hmg124h_5.JPG.dat	582.5	389.5	0.7	0.02	#NUM!	2.8E-04
YGL221C	YGL221C	UWOPS87_ctrl_hmg124h_5.JPG.dat	1186.5	44.4	1.0	0.03	#NUM!	5.2E-06
YGL222C	YGL222C	UWOPS87_ctrl_hmg124h_5.JPG.dat	1198.5	31.3	1.0	0.02	#NUM!	2.2E-06
YGL224C	YGL224C	UWOPS87_ctrl_hmg124h_5.JPG.dat	1216.5	66.1	1.0	0.05	#NUM!	2.7E-05
YGL226C-/	YGL226C-A	UWOPS87_ctrl_hmg124h_5.JPG.dat	1115.5	57.2	0.9	0.04	#NUM!	2.4E-05
YGL226W	YGL226W	UWOPS87_ctrl_hmg124h_5.JPG.dat	1266.5	23.7	1.0	0.03	#NUM!	4.9E-06
YGL227W	YGL227W	UWOPS87_ctrl_hmg124h_5.JPG.dat	1236.0	168.3	1.0	0.15	#NUM!	7.6E-04
YGL228W	YGL228W	UWOPS87_ctrl_hmg124h_5.JPG.dat	1025.0	52.0	0.9	0.04	#NUM!	3.0E-05
YGL229C	YGL229C	UWOPS87_ctrl_hmg124h_5.JPG.dat	1174.8	168.6	1.1	0.16	#NUM!	7.7E-04
YGL230C	YGL230C	UWOPS87_ctrl_hmg124h_5.JPG.dat	1152.5	64.9	1.0	0.06	#NUM!	5.6E-05
YGL231C	YGL231C	UWOPS87_ctrl_hmg124h_5.JPG.dat	1278.8	129.2	1.0	0.03	#NUM!	3.5E-04
YGL232W	YGL232W	UWOPS87_ctrl_hmg124h_5.JPG.dat	1354.0	7.9	1.1	0.00	#NUM!	7.2E-09
YGL234W	YGL234W	UWOPS87_ctrl_hmg124h_5.JPG.dat	1229.0	79.1	1.0	0.06	#NUM!	6.9E-05
YGL235W	YGL235W	UWOPS87_ctrl_hmg124h_5.JPG.dat	1362.3	84.2	1.1	0.07	#NUM!	8.2E-05
YGL236C	YGL236C	UWOPS87_ctrl_hmg124h_5.JPG.dat	1324.3	55.7	1.1	0.02	#NUM!	8.4E-05
YGL237C	YGL237C	UWOPS87_ctrl_hmg124h_5.JPG.dat	1361.0	57.2	1.1	0.05	#NUM!	2.2E-05
YGL241W	YGL241W	UWOPS87_ctrl_hmg124h_5.JPG.dat	1157.8	32.4	1.0	0.01	#NUM!	9.9E-06
YGL242C	YGL242C	UWOPS87_ctrl_hmg124h_5.JPG.dat	1268.0	56.1	1.1	0.02	#NUM!	1.0E-04
YGL243W	YGL243W	UWOPS87_ctrl_hmg124h_5.JPG.dat	1396.5	26.4	1.2	0.03	#NUM!	4.9E-06
YGL244W	YGL244W	UWOPS87_ctrl_hmg124h_5.JPG.dat	854.5	18.1	0.8	0.01	#NUM!	8.9E-07
YGL248W	YGL248W	UWOPS87_ctrl_hmg124h_5.JPG.dat	1138.5	49.4	1.0	0.04	#NUM!	1.4E-05
YGL249W	YGL249W	UWOPS87_ctrl_hmg124h_5.JPG.dat	1083.8	40.7	0.9	0.04	#NUM!	1.8E-05
YGL250W	YGL250W	UWOPS87_ctrl_hmg124h_5.JPG.dat	1330.8	43.5	1.1	0.04	#NUM!	1.8E-05
YGL251C	YGL251C	UWOPS87_ctrl_hmg124h_5.JPG.dat	1077.0	118.5	0.9	0.11	#NUM!	4.6E-04
YGL252C	YGL252C	UWOPS87_ctrl_hmg124h_5.JPG.dat	1189.0	74.1	1.0	0.05	#NUM!	3.7E-05
YGL253W	YGL253W	UWOPS87_ctrl_hmg124h_5.JPG.dat	1123.3	37.2	0.9	0.05	#NUM!	3.3E-05
YGL254W	YGL254W	UWOPS87_ctrl_hmg124h_5.JPG.dat	1221.3	136.8	1.1	0.10	#NUM!	2.4E-04
YGL255W	YGL255W	UWOPS87_ctrl_hmg124h_5.JPG.dat	1194.8	97.6	1.1	0.02	#NUM!	1.1E-04
YGL256W	YGL256W	UWOPS87_ctrl_hmg124h_5.JPG.dat	1098.8	29.8	1.0	0.02	#NUM!	2.0E-06
YGL257C	YGL257C	UWOPS87_ctrl_hmg124h_5.JPG.dat	1041.0	80.2	1.0	0.07	#NUM!	1.2E-04
YGL258W	YGL258W	UWOPS87_ctrl_hmg124h_5.JPG.dat	1285.3	21.8	1.2	0.01	#NUM!	4.1E-07
YGL259W	YGL259W	UWOPS87_ctrl_hmg124h_5.JPG.dat	1308.5	28.7	1.1	0.02	#NUM!	1.5E-06
YGL260W	YGL260W	UWOPS87_ctrl_hmg124h_5.JPG.dat	1252.3	181.5	1.1	0.15	#NUM!	8.5E-04
YGL261C	YGL261C	UWOPS87_ctrl_hmg124h_5.JPG.dat	1213.0	114.5	1.0	0.09	#NUM!	2.2E-04
YGL262W	YGL262W	UWOPS87_ctrl_hmg124h_5.JPG.dat	1250.5	35.9	1.0	0.03	#NUM!	8.6E-06
YGL263W	YGL263W	UWOPS87_ctrl_hmg124h_5.JPG.dat	1146.0	118.9	1.0	0.10	#NUM!	3.5E-04
YGR001C	YGR001C	UWOPS87_ctrl_hmg124h_5.JPG.dat	1145.5	56.7	1.0	0.05	#NUM!	2.8E-05
YGR003W	YGR003W	UWOPS87_ctrl_hmg124h_5.JPG.dat	1165.0	45.5	1.0	0.04	#NUM!	2.1E-05
YGR004W	YGR004W	UWOPS87_ctrl_hmg124h_5.JPG.dat	1195.0	47.0	1.1	0.01	#NUM!	6.0E-05
YGR007W	YGR007W	UWOPS87_ctrl_hmg124h_5.JPG.dat	1061.3	15.6	0.9	0.01	#NUM!	1.1E-06
YGR008C	YGR008C	UWOPS87_ctrl_hmg124h_5.JPG.dat	1054.5	46.9	1.0	0.01	#NUM!	4.4E-05
YGR010W	YGR010W	UWOPS87_ctrl_hmg124h_5.JPG.dat	1026.0	43.9	1.0	0.03	#NUM!	1.2E-05
YGR011W	YGR011W	UWOPS87_ctrl_hmg124h_5.JPG.dat	1280.3	58.2	1.1	0.05	#NUM!	2.7E-05
YGR012W	YGR012W	UWOPS87_ctrl_hmg124h_5.JPG.dat	1203.3	14.8	1.0	0.00	#NUM!	1.4E-06
YGR014W	YGR014W	UWOPS87_ctrl_hmg124h_5.JPG.dat	1309.3	49.0	1.1	0.04	#NUM!	1.6E-05
YGR015C	YGR015C	UWOPS87_ctrl_hmg124h_5.JPG.dat	1103.0	75.8	0.9	0.06	#NUM!	7.4E-05

YGR016W	YGR016W	UWOPS87_ctrl_hmg124h_5.JPG.dat	1161.8	86.8	1.0	0.08	#NUM!	1.5E-04
YGR017W	YGR017W	UWOPS87_ctrl_hmg124h_5.JPG.dat	1261.8	56.9	1.1	0.05	#NUM!	3.0E-05
YGR018C	YGR018C	UWOPS87_ctrl_hmg124h_5.JPG.dat	1087.0	35.4	1.0	0.03	#NUM!	8.5E-06
YGR019W	YGR019W	UWOPS87_ctrl_hmg124h_5.JPG.dat	1270.8	106.3	1.1	0.09	#NUM!	1.6E-04
YGR021W	YGR021W	UWOPS87_ctrl_hmg124h_5.JPG.dat	886.3	590.9	1.0	0.01	#NUM!	2.6E-05
YGR022C	YGR022C	UWOPS87_ctrl_hmg124h_5.JPG.dat	1207.3	85.6	1.1	0.08	#NUM!	9.9E-05
YGR023W	YGR023W	UWOPS87_ctrl_hmg124h_5.JPG.dat	1020.5	53.5	1.0	0.01	#NUM!	5.0E-05
YGR025W	YGR025W	UWOPS87_ctrl_hmg124h_5.JPG.dat	1069.3	214.2	0.9	0.02	#NUM!	1.9E-04
YGR026W	YGR026W	UWOPS87_ctrl_hmg124h_5.JPG.dat	1126.5	66.2	1.0	0.06	#NUM!	5.1E-05
YGR027C	YGR027C	UWOPS87_ctrl_hmg124h_5.JPG.dat	1126.8	66.4	1.0	0.01	#NUM!	6.5E-05
YGR028W	YGR028W	UWOPS87_ctrl_hmg124h_5.JPG.dat	1234.8	68.1	1.1	0.08	#NUM!	8.8E-05
YGR031W	YGR031W	UWOPS87_ctrl_hmg124h_5.JPG.dat	1022.5	61.4	0.9	0.07	#NUM!	1.0E-04
YGR032W	YGR032W	UWOPS87_ctrl_hmg124h_5.JPG.dat	1087.5	36.5	1.0	0.05	#NUM!	2.9E-05
YGR033C	YGR033C	UWOPS87_ctrl_hmg124h_5.JPG.dat	1152.5	74.6	1.0	0.02	#NUM!	8.9E-05
YGR034W	YGR034W	UWOPS87_ctrl_hmg124h_5.JPG.dat	1179.0	29.2	1.1	0.04	#NUM!	1.8E-05
YGR035C	YGR035C	UWOPS87_ctrl_hmg124h_5.JPG.dat	994.3	664.4	1.2	0.05	#NUM!	5.1E-04
YGR037C	YGR037C	UWOPS87_ctrl_hmg124h_5.JPG.dat	971.0	217.6	0.8	0.04	#NUM!	7.0E-04
YGR038W	YGR038W	UWOPS87_ctrl_hmg124h_5.JPG.dat	1104.3	76.1	1.0	0.05	#NUM!	3.4E-05
YGR039W	YGR039W	UWOPS87_ctrl_hmg124h_5.JPG.dat	1139.8	99.9	1.1	0.08	#NUM!	1.3E-04
YGR040W	YGR040W	UWOPS87_ctrl_hmg124h_5.JPG.dat	1078.3	14.6	1.0	0.02	#NUM!	1.8E-06
YGR041W	YGR041W	UWOPS87_ctrl_hmg124h_5.JPG.dat	915.5	104.7	0.8	0.10	#NUM!	4.7E-04
YGR042W	YGR042W	UWOPS87_ctrl_hmg124h_5.JPG.dat	1083.8	69.7	1.0	0.05	#NUM!	4.1E-05
YGR043C	YGR043C	UWOPS87_ctrl_hmg124h_5.JPG.dat	1059.3	101.0	1.0	0.03	#NUM!	2.3E-04
YGR044C	YGR044C	UWOPS87_ctrl_hmg124h_5.JPG.dat	1038.5	68.0	0.9	0.06	#NUM!	6.6E-05
YGR045C	YGR045C	UWOPS87_ctrl_hmg124h_5.JPG.dat	1204.8	239.8	1.1	0.20	#NUM!	1.9E-03
YGR049W	YGR049W	UWOPS87_ctrl_hmg124h_5.JPG.dat	1288.8	65.9	1.1	0.07	#NUM!	6.7E-05
YGR050C	YGR050C	UWOPS87_ctrl_hmg124h_5.JPG.dat	1099.0	75.6	0.9	0.02	#NUM!	2.2E-04
YGR051C	YGR051C	UWOPS87_ctrl_hmg124h_5.JPG.dat	1356.5	48.9	1.2	0.03	#NUM!	4.3E-06
YGR052W	YGR052W	UWOPS87_ctrl_hmg124h_5.JPG.dat	1047.3	78.2	1.0	0.06	#NUM!	7.5E-05
YGR053C	YGR053C	UWOPS87_ctrl_hmg124h_5.JPG.dat	1076.8	83.7	1.0	0.09	#NUM!	2.1E-04
YGR054W	YGR054W	UWOPS87_ctrl_hmg124h_5.JPG.dat	1215.8	101.1	1.1	0.09	#NUM!	1.5E-04
YGR058W	YGR058W	UWOPS87_ctrl_hmg124h_5.JPG.dat	1171.8	178.2	1.1	0.03	#NUM!	2.7E-04
YGR059W	YGR059W	UWOPS87_ctrl_hmg124h_5.JPG.dat	1117.8	32.2	1.0	0.01	#NUM!	1.4E-05
YGR066C	YGR066C	UWOPS87_ctrl_hmg124h_5.JPG.dat	1275.5	90.3	1.1	0.07	#NUM!	9.1E-05
YGR069W	YGR069W	UWOPS87_ctrl_hmg124h_5.JPG.dat	1200.0	81.8	1.0	0.05	#NUM!	3.8E-05
YGR070W	YGR070W	UWOPS87_ctrl_hmg124h_5.JPG.dat	1299.8	86.9	1.1	0.06	#NUM!	4.7E-05
YGR071C	YGR071C	UWOPS87_ctrl_hmg124h_5.JPG.dat	1356.3	88.7	1.1	0.06	#NUM!	5.1E-05
YGR072W	YGR072W	UWOPS87_ctrl_hmg124h_5.JPG.dat	1211.0	55.2	1.0	0.02	#NUM!	1.2E-04
YGR077C	YGR077C	UWOPS87_ctrl_hmg124h_5.JPG.dat	1226.8	96.3	1.0	0.07	#NUM!	9.2E-05
YGR078C	YGR078C	UWOPS87_ctrl_hmg124h_5.JPG.dat	1018.5	80.5	1.0	0.07	#NUM!	1.2E-04
YGR079W	YGR079W	UWOPS87_ctrl_hmg124h_5.JPG.dat	968.0	46.0	0.9	0.03	#NUM!	1.0E-05
YGR080W	YGR080W	UWOPS87_ctrl_hmg124h_5.JPG.dat	939.8	111.1	0.8	0.09	#NUM!	4.0E-04
YGR081C	YGR081C	UWOPS87_ctrl_hmg124h_5.JPG.dat	1080.3	84.0	0.9	0.07	#NUM!	1.1E-04
YGR084C	YGR084C	UWOPS87_ctrl_hmg124h_5.JPG.dat	1133.5	43.4	0.9	0.03	#NUM!	1.3E-05
YGR085C	YGR085C	UWOPS87_ctrl_hmg124h_5.JPG.dat	1146.0	25.9	0.9	0.02	#NUM!	1.4E-06
YGR086C	YGR086C	UWOPS87_ctrl_hmg124h_5.JPG.dat	1214.3	31.2	1.0	0.03	#NUM!	5.4E-06
YGR087C	YGR087C	UWOPS87_ctrl_hmg124h_5.JPG.dat	1304.8	19.9	1.1	0.03	#NUM!	4.2E-06
YGR088W	YGR088W	UWOPS87_ctrl_hmg124h_5.JPG.dat	1092.0	30.8	0.9	0.03	#NUM!	6.2E-06
YGR089W	YGR089W	UWOPS87_ctrl_hmg124h_5.JPG.dat	1089.8	14.7	0.9	0.02	#NUM!	2.4E-06
YGR092W	YGR092W	UWOPS87_ctrl_hmg124h_5.JPG.dat	702.3	484.9	0.8	0.14	#NUM!	9.6E-03
YGR093W	YGR093W	UWOPS87_ctrl_hmg124h_5.JPG.dat	958.3	82.2	0.9	0.06	#NUM!	8.5E-05
YGR096W	YGR096W	UWOPS87_ctrl_hmg124h_5.JPG.dat	970.3	38.6	0.9	0.04	#NUM!	2.4E-05
YGR097W	YGR097W	UWOPS87_ctrl_hmg124h_5.JPG.dat	1184.5	50.4	1.0	0.06	#NUM!	4.5E-05
YGR100W	YGR100W	UWOPS87_ctrl_hmg124h_5.JPG.dat	1206.3	65.0	1.0	0.06	#NUM!	6.6E-05
YGR101W	YGR101W	UWOPS87_ctrl_hmg124h_5.JPG.dat	1318.3	44.2	1.1	0.05	#NUM!	2.9E-05
YGR105W	YGR105W	UWOPS87_ctrl_hmg124h_5.JPG.dat	1280.0	176.4	1.1	0.04	#NUM!	3.9E-04
YGR106C	YGR106C	UWOPS87_ctrl_hmg124h_5.JPG.dat	1106.3	64.1	0.9	0.06	#NUM!	7.9E-05
YGR107W	YGR107W	UWOPS87_ctrl_hmg124h_5.JPG.dat	1217.5	56.0	1.0	0.01	#NUM!	1.1E-05
YGR108W	YGR108W	UWOPS87_ctrl_hmg124h_5.JPG.dat	1096.3	53.6	1.0	0.04	#NUM!	2.4E-05
YGR109C	YGR109C	UWOPS87_ctrl_hmg124h_5.JPG.dat	1114.5	23.2	1.0	0.02	#NUM!	3.5E-06
YGR110W	YGR110W	UWOPS87_ctrl_hmg124h_5.JPG.dat	1170.5	64.1	1.1	0.05	#NUM!	3.3E-05
YGR111W	YGR111W	UWOPS87_ctrl_hmg124h_5.JPG.dat	1056.3	48.6	1.0	0.04	#NUM!	1.6E-05
YGR117C	YGR117C	UWOPS87_ctrl_hmg124h_5.JPG.dat	1098.3	68.9	1.0	0.06	#NUM!	5.4E-05
YGR118W	YGR118W	UWOPS87_ctrl_hmg124h_5.JPG.dat	859.5	76.2	0.7	0.07	#NUM!	2.0E-04
YGR121C	YGR121C	UWOPS87_ctrl_hmg124h_5.JPG.dat	1328.3	55.8	1.1	0.05	#NUM!	2.9E-05
YGR123C	YGR123C	UWOPS87_ctrl_hmg124h_5.JPG.dat	1154.3	37.5	0.9	0.03	#NUM!	1.1E-05
YGR124W	YGR124W	UWOPS87_ctrl_hmg124h_5.JPG.dat	1243.8	86.0	1.0	0.07	#NUM!	8.5E-05
YGR125W	YGR125W	UWOPS87_ctrl_hmg124h_5.JPG.dat	1335.0	58.1	1.1	0.05	#NUM!	2.2E-05
YGR126W	YGR126W	UWOPS87_ctrl_hmg124h_5.JPG.dat	1093.5	35.4	0.9	0.04	#NUM!	1.9E-05
YGR127W	YGR127W	UWOPS87_ctrl_hmg124h_5.JPG.dat	1045.8	30.1	0.9	0.03	#NUM!	6.3E-06

YGR129W	YGR129W	UWOPS87_ctrl_hmg124h_5.JPG.dat	1092.8	50.1	1.0	0.05	#NUM!	3.6E-05
YGR130C	YGR130C	UWOPS87_ctrl_hmg124h_5.JPG.dat	1155.8	132.9	1.0	0.02	#NUM!	2.1E-04
YGR131W	YGR131W	UWOPS87_ctrl_hmg124h_5.JPG.dat	1016.5	49.6	1.0	0.01	#NUM!	4.7E-05
YGR132C	YGR132C	UWOPS87_ctrl_hmg124h_5.JPG.dat	1240.0	69.2	1.1	0.06	#NUM!	3.5E-05
YGR133W	YGR133W	UWOPS87_ctrl_hmg124h_5.JPG.dat	1110.8	24.1	0.9	0.02	#NUM!	2.5E-06
YGR134W	YGR134W	UWOPS87_ctrl_hmg124h_5.JPG.dat	1083.0	48.1	0.9	0.04	#NUM!	2.0E-05
YGR135W	YGR135W	UWOPS87_ctrl_hmg124h_5.JPG.dat	1158.5	120.1	1.0	0.10	#NUM!	3.0E-04
YGR136W	YGR136W	UWOPS87_ctrl_hmg124h_5.JPG.dat	1212.8	59.0	1.0	0.02	#NUM!	7.8E-05
YGR137W	YGR137W	UWOPS87_ctrl_hmg124h_5.JPG.dat	0.0	0.0	0.0	0.00	#NUM!	
YGR138C	YGR138C	UWOPS87_ctrl_hmg124h_5.JPG.dat	1056.8	53.0	0.9	0.04	#NUM!	2.0E-05
YGR139W	YGR139W	UWOPS87_ctrl_hmg124h_5.JPG.dat	1146.5	36.5	1.0	0.03	#NUM!	1.0E-05
YGR141W	YGR141W	UWOPS87_ctrl_hmg124h_5.JPG.dat	567.5	656.5	0.5	0.57	#NUM!	1.8E-01
YGR142W	YGR142W	UWOPS87_ctrl_hmg124h_5.JPG.dat	1332.0	74.1	1.1	0.06	#NUM!	4.5E-05
YGR143W	YGR143W	UWOPS87_ctrl_hmg124h_5.JPG.dat	998.0	90.3	1.0	0.09	#NUM!	2.3E-04
YGR144W	YGR144W	UWOPS87_ctrl_hmg124h_5.JPG.dat	1325.0	134.3	1.3	0.16	#NUM!	5.5E-04
YGR146C	YGR146C	UWOPS87_ctrl_hmg124h_5.JPG.dat	1061.8	49.0	1.0	0.06	#NUM!	5.8E-05
YGR148C	YGR148C	UWOPS87_ctrl_hmg124h_5.JPG.dat	1041.3	85.9	0.9	0.07	#NUM!	1.1E-04
YGR149W	YGR149W	UWOPS87_ctrl_hmg124h_5.JPG.dat	1090.3	94.9	1.0	0.09	#NUM!	1.8E-04
YGR151C	YGR151C	UWOPS87_ctrl_hmg124h_5.JPG.dat	1140.5	28.9	1.0	0.04	#NUM!	1.3E-05
YGR152C	YGR152C	UWOPS87_ctrl_hmg124h_5.JPG.dat	1017.5	16.8	0.9	0.01	#NUM!	9.3E-07
YGR153W	YGR153W	UWOPS87_ctrl_hmg124h_5.JPG.dat	1058.3	53.0	1.0	0.01	#NUM!	1.3E-05
YGR154C	YGR154C	UWOPS87_ctrl_hmg124h_5.JPG.dat	1078.8	7.4	1.0	0.02	#NUM!	1.5E-06
YGR157W	YGR157W	UWOPS87_ctrl_hmg124h_5.JPG.dat	0.0	0.0	0.0	0.00	#NUM!	
YGR161C	YGR161C	UWOPS87_ctrl_hmg124h_5.JPG.dat	1249.5	96.6	1.1	0.07	#NUM!	7.0E-05
YOR202W	YOR202W	UWOPS87_ctrl_hmg124h_5.JPG.dat	1293.3	203.5	1.0	0.09	#NUM!	8.3E-282
1	1	UWOPS87_ctrl_hmg124h_6.JPG.dat	1294.8	182.9	1.0	0.14	#NUM!	7.1E-04
2	2	UWOPS87_ctrl_hmg124h_6.JPG.dat	1151.0	184.6	1.0	0.12	#NUM!	5.0E-04
3	3	UWOPS87_ctrl_hmg124h_6.JPG.dat	961.5	168.8	0.8	0.05	#NUM!	1.2E-03
4	4	UWOPS87_ctrl_hmg124h_6.JPG.dat	994.5	196.0	0.9	0.14	#NUM!	1.2E-03
YGR163W	YGR163W	UWOPS87_ctrl_hmg124h_6.JPG.dat	918.5	38.4	0.9	0.05	#NUM!	3.5E-05
YGR164W	YGR164W	UWOPS87_ctrl_hmg124h_6.JPG.dat	1084.0	45.0	1.0	0.02	#NUM!	7.2E-05
YGR166W	YGR166W	UWOPS87_ctrl_hmg124h_6.JPG.dat	979.8	110.1	0.9	0.02	#NUM!	1.6E-04
YGR168C	YGR168C	UWOPS87_ctrl_hmg124h_6.JPG.dat	1076.8	117.7	1.0	0.02	#NUM!	8.9E-05
YGR169C	YGR169C	UWOPS87_ctrl_hmg124h_6.JPG.dat	1027.0	80.8	1.0	0.07	#NUM!	9.8E-05
YGR170W	YGR170W	UWOPS87_ctrl_hmg124h_6.JPG.dat	1077.8	25.0	1.0	0.01	#NUM!	7.0E-07
YGR173W	YGR173W	UWOPS87_ctrl_hmg124h_6.JPG.dat	994.8	120.7	0.9	0.04	#NUM!	7.1E-04
YGR174C	YGR174C	UWOPS87_ctrl_hmg124h_6.JPG.dat	1136.0	84.4	1.0	0.07	#NUM!	9.9E-05
YGR176W	YGR176W	UWOPS87_ctrl_hmg124h_6.JPG.dat	1043.0	74.6	0.9	0.07	#NUM!	9.5E-05
YGR177C	YGR177C	UWOPS87_ctrl_hmg124h_6.JPG.dat	1056.5	79.4	1.0	0.03	#NUM!	2.5E-04
YGR178C	YGR178C	UWOPS87_ctrl_hmg124h_6.JPG.dat	1208.8	89.6	1.1	0.08	#NUM!	1.0E-04
YGR181W	YGR181W	UWOPS87_ctrl_hmg124h_6.JPG.dat	1057.5	78.8	1.1	0.07	#NUM!	8.7E-05
YGR182C	YGR182C	UWOPS87_ctrl_hmg124h_6.JPG.dat	1210.8	122.7	1.1	0.11	#NUM!	2.2E-04
YGR183C	YGR183C	UWOPS87_ctrl_hmg124h_6.JPG.dat	1336.5	56.8	1.2	0.07	#NUM!	5.3E-05
YGR184C	YGR184C	UWOPS87_ctrl_hmg124h_6.JPG.dat	0.0	0.0	0.0	0.00	#NUM!	
YGR187C	YGR187C	UWOPS87_ctrl_hmg124h_6.JPG.dat	921.0	80.1	0.9	0.07	#NUM!	1.2E-04
YGR188C	YGR188C	UWOPS87_ctrl_hmg124h_6.JPG.dat	910.3	157.2	1.0	0.01	#NUM!	5.3E-05
YGR189C	YGR189C	UWOPS87_ctrl_hmg124h_6.JPG.dat	984.3	31.8	1.0	0.03	#NUM!	8.8E-06
YGR192C	YGR192C	UWOPS87_ctrl_hmg124h_6.JPG.dat	1164.0	33.2	1.1	0.01	#NUM!	2.5E-05
YGR193C	YGR193C	UWOPS87_ctrl_hmg124h_6.JPG.dat	1147.8	97.0	1.0	0.10	#NUM!	2.2E-04
YGR194C	YGR194C	UWOPS87_ctrl_hmg124h_6.JPG.dat	1104.0	138.6	1.1	0.02	#NUM!	1.7E-04
YGR196C	YGR196C	UWOPS87_ctrl_hmg124h_6.JPG.dat	925.3	44.1	0.9	0.04	#NUM!	2.6E-05
YGR197C	YGR197C	UWOPS87_ctrl_hmg124h_6.JPG.dat	1109.5	67.5	1.1	0.08	#NUM!	8.4E-05
YGR199W	YGR199W	UWOPS87_ctrl_hmg124h_6.JPG.dat	1069.8	138.7	1.0	0.14	#NUM!	6.8E-04
YGR200C	YGR200C	UWOPS87_ctrl_hmg124h_6.JPG.dat	1102.8	97.9	1.1	0.10	#NUM!	2.1E-04
YGR201C	YGR201C	UWOPS87_ctrl_hmg124h_6.JPG.dat	1023.5	136.9	1.0	0.12	#NUM!	5.2E-04
YGR202C	YGR202C	UWOPS87_ctrl_hmg124h_6.JPG.dat	1016.0	25.6	1.0	0.03	#NUM!	4.1E-06
YGR203W	YGR203W	UWOPS87_ctrl_hmg124h_6.JPG.dat	944.0	35.9	0.9	0.01	#NUM!	3.5E-05
YGR205W	YGR205W	UWOPS87_ctrl_hmg124h_6.JPG.dat	1102.0	28.3	1.1	0.04	#NUM!	9.6E-06
YGR206W	YGR206W	UWOPS87_ctrl_hmg124h_6.JPG.dat	1024.3	11.9	1.0	0.01	#NUM!	1.8E-07
YGR207C	YGR207C	UWOPS87_ctrl_hmg124h_6.JPG.dat	1111.0	132.9	1.1	0.01	#NUM!	6.5E-05
YGR208W	YGR208W	UWOPS87_ctrl_hmg124h_6.JPG.dat	1082.8	27.2	1.0	0.03	#NUM!	6.8E-06
YGR209C	YGR209C	UWOPS87_ctrl_hmg124h_6.JPG.dat	1105.3	54.0	1.1	0.02	#NUM!	9.9E-05
YGR210C	YGR210C	UWOPS87_ctrl_hmg124h_6.JPG.dat	817.8	561.3	1.1	0.16	#NUM!	6.1E-03
YGR212W	YGR212W	UWOPS87_ctrl_hmg124h_6.JPG.dat	1059.3	98.3	1.0	0.08	#NUM!	1.6E-04
YGR213C	YGR213C	UWOPS87_ctrl_hmg124h_6.JPG.dat	893.8	109.4	0.9	0.04	#NUM!	6.1E-04
YGR214W	YGR214W	UWOPS87_ctrl_hmg124h_6.JPG.dat	836.0	73.0	0.8	0.07	#NUM!	1.5E-04
YGR217W	YGR217W	UWOPS87_ctrl_hmg124h_6.JPG.dat	1079.8	35.5	1.1	0.01	#NUM!	1.0E-05
YGR221C	YGR221C	UWOPS87_ctrl_hmg124h_6.JPG.dat	1140.8	45.6	1.1	0.01	#NUM!	4.8E-05
YGR223C	YGR223C	UWOPS87_ctrl_hmg124h_6.JPG.dat	1105.8	53.9	1.1	0.01	#NUM!	5.3E-05
YGR224W	YGR224W	UWOPS87_ctrl_hmg124h_6.JPG.dat	1212.0	72.2	1.1	0.08	#NUM!	9.8E-05

YGR225W	YGR225W	UWOPS87_ctrl_hmg124h_6.JPG.dat	1200.5	21.8	1.1	0.01	#NUM!	1.1E-05
YGR226C	YGR226C	UWOPS87_ctrl_hmg124h_6.JPG.dat	1068.8	62.5	1.0	0.06	#NUM!	6.4E-05
YGR227W	YGR227W	UWOPS87_ctrl_hmg124h_6.JPG.dat	960.8	39.7	0.9	0.01	#NUM!	1.6E-05
YGR228W	YGR228W	UWOPS87_ctrl_hmg124h_6.JPG.dat	884.8	34.1	1.0	0.05	#NUM!	3.4E-05
YGR229C	YGR229C	UWOPS87_ctrl_hmg124h_6.JPG.dat	0.0	0.0	0.0	0.00	#NUM!	
YGR230W	YGR230W	UWOPS87_ctrl_hmg124h_6.JPG.dat	1089.3	109.3	1.1	0.10	#NUM!	2.3E-04
YGR231C	YGR231C	UWOPS87_ctrl_hmg124h_6.JPG.dat	1137.0	83.2	1.2	0.03	#NUM!	1.8E-04
YGR232W	YGR232W	UWOPS87_ctrl_hmg124h_6.JPG.dat	1073.3	50.2	1.1	0.01	#NUM!	1.2E-05
YGR233C	YGR233C	UWOPS87_ctrl_hmg124h_6.JPG.dat	898.0	53.0	0.9	0.04	#NUM!	3.2E-05
YGR234W	YGR234W	UWOPS87_ctrl_hmg124h_6.JPG.dat	990.8	91.8	1.0	0.08	#NUM!	1.4E-04
YGR235C	YGR235C	UWOPS87_ctrl_hmg124h_6.JPG.dat	1272.8	15.1	1.2	0.01	#NUM!	5.5E-07
YGR236C	YGR236C	UWOPS87_ctrl_hmg124h_6.JPG.dat	1242.8	45.9	1.1	0.05	#NUM!	1.8E-05
YGR237C	YGR237C	UWOPS87_ctrl_hmg124h_6.JPG.dat	1127.5	146.1	1.1	0.14	#NUM!	5.7E-04
YGR238C	YGR238C	UWOPS87_ctrl_hmg124h_6.JPG.dat	929.8	51.4	1.0	0.05	#NUM!	3.6E-05
YGR239C	YGR239C	UWOPS87_ctrl_hmg124h_6.JPG.dat	1241.5	43.9	1.3	0.04	#NUM!	1.2E-05
YGR241C	YGR241C	UWOPS87_ctrl_hmg124h_6.JPG.dat	793.5	535.1	1.0	0.09	#NUM!	2.9E-03
YGR242W	YGR242W	UWOPS87_ctrl_hmg124h_6.JPG.dat	1195.3	66.9	1.1	0.07	#NUM!	6.6E-05
YGR243W	YGR243W	UWOPS87_ctrl_hmg124h_6.JPG.dat	1080.5	97.8	1.0	0.10	#NUM!	2.1E-04
YGR244C	YGR244C	UWOPS87_ctrl_hmg124h_6.JPG.dat	1052.0	42.5	1.1	0.04	#NUM!	1.9E-05
YGR247W	YGR247W	UWOPS87_ctrl_hmg124h_6.JPG.dat	865.3	134.2	0.8	0.13	#NUM!	1.1E-03
YGR248W	YGR248W	UWOPS87_ctrl_hmg124h_6.JPG.dat	1075.0	116.9	1.1	0.04	#NUM!	5.0E-04
YGR249W	YGR249W	UWOPS87_ctrl_hmg124h_6.JPG.dat	1111.5	93.1	1.0	0.09	#NUM!	2.0E-04
YGR250C	YGR250C	UWOPS87_ctrl_hmg124h_6.JPG.dat	1229.0	27.9	1.1	0.03	#NUM!	4.2E-06
YGR256W	YGR256W	UWOPS87_ctrl_hmg124h_6.JPG.dat	963.3	145.5	0.9	0.13	#NUM!	6.8E-04
YGR259C	YGR259C	UWOPS87_ctrl_hmg124h_6.JPG.dat	952.3	59.8	1.0	0.06	#NUM!	7.5E-05
YGR260W	YGR260W	UWOPS87_ctrl_hmg124h_6.JPG.dat	974.5	95.7	1.0	0.04	#NUM!	4.3E-04
YGR261C	YGR261C	UWOPS87_ctrl_hmg124h_6.JPG.dat	1002.0	30.6	1.0	0.03	#NUM!	5.1E-06
YGR263C	YGR263C	UWOPS87_ctrl_hmg124h_6.JPG.dat	1049.8	142.0	1.1	0.04	#NUM!	5.1E-04
YGR266W	YGR266W	UWOPS87_ctrl_hmg124h_6.JPG.dat	1044.3	93.5	1.1	0.04	#NUM!	5.1E-04
YGR268C	YGR268C	UWOPS87_ctrl_hmg124h_6.JPG.dat	857.3	113.3	1.0	0.11	#NUM!	4.2E-04
YGR269W	YGR269W	UWOPS87_ctrl_hmg124h_6.JPG.dat	978.8	102.2	1.1	0.02	#NUM!	1.1E-04
YGR270W	YGR270W	UWOPS87_ctrl_hmg124h_6.JPG.dat	1053.5	68.3	1.1	0.02	#NUM!	8.9E-05
YGR275W	YGR275W	UWOPS87_ctrl_hmg124h_6.JPG.dat	999.5	156.2	1.0	0.13	#NUM!	6.4E-04
YGR279C	YGR279C	UWOPS87_ctrl_hmg124h_6.JPG.dat	852.0	76.6	0.9	0.07	#NUM!	1.7E-04
YGR281W	YGR281W	UWOPS87_ctrl_hmg124h_6.JPG.dat	971.5	26.6	1.1	0.01	#NUM!	3.6E-05
YGR282C	YGR282C	UWOPS87_ctrl_hmg124h_6.JPG.dat	997.0	32.5	1.1	0.02	#NUM!	2.8E-06
YGR283C	YGR283C	UWOPS87_ctrl_hmg124h_6.JPG.dat	981.0	83.8	0.9	0.02	#NUM!	1.1E-04
YGR284C	YGR284C	UWOPS87_ctrl_hmg124h_6.JPG.dat	1162.0	55.5	1.1	0.01	#NUM!	2.0E-05
YGR286C	YGR286C	UWOPS87_ctrl_hmg124h_6.JPG.dat	1231.3	103.1	1.1	0.09	#NUM!	1.3E-04
YGR287C	YGR287C	UWOPS87_ctrl_hmg124h_6.JPG.dat	893.8	79.2	0.9	0.08	#NUM!	1.9E-04
YGR288W	YGR288W	UWOPS87_ctrl_hmg124h_6.JPG.dat	1021.3	110.1	1.1	0.04	#NUM!	4.2E-04
YGR290W	YGR290W	UWOPS87_ctrl_hmg124h_6.JPG.dat	1019.8	35.4	1.0	0.03	#NUM!	1.2E-05
YGR295C	YGR295C	UWOPS87_ctrl_hmg124h_6.JPG.dat	1038.3	59.3	1.0	0.06	#NUM!	6.0E-05
YHL002W	YHL002W	UWOPS87_ctrl_hmg124h_6.JPG.dat	1258.8	14.4	1.2	0.02	#NUM!	6.3E-07
YHL003C	YHL003C	UWOPS87_ctrl_hmg124h_6.JPG.dat	1049.8	108.4	1.0	0.10	#NUM!	3.1E-04
YHL005C	YHL005C	UWOPS87_ctrl_hmg124h_6.JPG.dat	1029.0	46.2	0.9	0.04	#NUM!	2.5E-05
YHL006C	YHL006C	UWOPS87_ctrl_hmg124h_6.JPG.dat	1141.0	49.3	1.1	0.04	#NUM!	1.3E-05
YHL007C	YHL007C	UWOPS87_ctrl_hmg124h_6.JPG.dat	0.0	0.0	0.0	0.00	#NUM!	
YHL008C	YHL008C	UWOPS87_ctrl_hmg124h_6.JPG.dat	1105.0	75.3	1.1	0.07	#NUM!	9.3E-05
YHL009C	YHL009C	UWOPS87_ctrl_hmg124h_6.JPG.dat	1113.5	64.2	1.0	0.07	#NUM!	7.7E-05
YHL010C	YHL010C	UWOPS87_ctrl_hmg124h_6.JPG.dat	1101.8	49.4	1.1	0.03	#NUM!	8.9E-06
YHL012W	YHL012W	UWOPS87_ctrl_hmg124h_6.JPG.dat	1065.0	109.7	1.1	0.03	#NUM!	3.3E-04
YHL013C	YHL013C	UWOPS87_ctrl_hmg124h_6.JPG.dat	907.0	47.3	0.9	0.05	#NUM!	3.7E-05
YHL014C	YHL014C	UWOPS87_ctrl_hmg124h_6.JPG.dat	1080.0	38.4	1.1	0.05	#NUM!	3.4E-05
YHL016C	YHL016C	UWOPS87_ctrl_hmg124h_6.JPG.dat	892.3	149.2	0.9	0.14	#NUM!	1.1E-03
YHL017W	YHL017W	UWOPS87_ctrl_hmg124h_6.JPG.dat	1034.8	51.8	1.0	0.06	#NUM!	5.5E-05
YHL019C	YHL019C	UWOPS87_ctrl_hmg124h_6.JPG.dat	1030.0	30.4	0.9	0.02	#NUM!	2.2E-06
YHL020C	YHL020C	UWOPS87_ctrl_hmg124h_6.JPG.dat	896.0	81.4	0.9	0.07	#NUM!	1.7E-04
YHL021C	YHL021C	UWOPS87_ctrl_hmg124h_6.JPG.dat	901.8	41.8	1.0	0.05	#NUM!	4.4E-05
YHL022C	YHL022C	UWOPS87_ctrl_hmg124h_6.JPG.dat	901.3	102.2	0.8	0.01	#NUM!	2.2E-05
YHL023C	YHL023C	UWOPS87_ctrl_hmg124h_6.JPG.dat	910.8	141.7	0.8	0.05	#NUM!	1.4E-03
YHL024W	YHL024W	UWOPS87_ctrl_hmg124h_6.JPG.dat	1041.8	181.6	1.0	0.19	#NUM!	1.6E-03
YHL025W	YHL025W	UWOPS87_ctrl_hmg124h_6.JPG.dat	958.8	146.6	1.0	0.14	#NUM!	8.6E-04
YHL026C	YHL026C	UWOPS87_ctrl_hmg124h_6.JPG.dat	1009.5	66.6	1.0	0.06	#NUM!	5.6E-05
YHL027W	YHL027W	UWOPS87_ctrl_hmg124h_6.JPG.dat	931.8	59.3	0.9	0.05	#NUM!	4.3E-05
YHL028W	YHL028W	UWOPS87_ctrl_hmg124h_6.JPG.dat	965.5	117.1	0.9	0.10	#NUM!	3.7E-04
YHL029C	YHL029C	UWOPS87_ctrl_hmg124h_6.JPG.dat	955.3	29.2	0.9	0.02	#NUM!	5.0E-06
YHL030W	YHL030W	UWOPS87_ctrl_hmg124h_6.JPG.dat	1093.0	35.9	1.0	0.01	#NUM!	3.6E-05
YHL031C	YHL031C	UWOPS87_ctrl_hmg124h_6.JPG.dat	1046.8	46.9	1.0	0.05	#NUM!	2.7E-05
YHL032C	YHL032C	UWOPS87_ctrl_hmg124h_6.JPG.dat	864.5	33.6	0.9	0.02	#NUM!	5.2E-06

YHL033C	YHL033C	UWOPS87_ctrl_hmg124h_6.JPG.dat	837.3	239.6	0.8	0.25	#NUM!	6.9E-03
YHL034C	YHL034C	UWOPS87_ctrl_hmg124h_6.JPG.dat	1071.5	47.5	1.0	0.01	#NUM!	2.5E-05
YHL035C	YHL035C	UWOPS87_ctrl_hmg124h_6.JPG.dat	828.3	82.4	0.8	0.07	#NUM!	1.7E-04
YHL036W	YHL036W	UWOPS87_ctrl_hmg124h_6.JPG.dat	1076.0	45.0	1.1	0.03	#NUM!	3.7E-06
YHL037C	YHL037C	UWOPS87_ctrl_hmg124h_6.JPG.dat	1063.8	133.4	1.1	0.03	#NUM!	2.2E-04
YHL039W	YHL039W	UWOPS87_ctrl_hmg124h_6.JPG.dat	957.5	45.6	0.9	0.04	#NUM!	2.3E-05
YHL040C	YHL040C	UWOPS87_ctrl_hmg124h_6.JPG.dat	871.8	158.0	0.8	0.14	#NUM!	1.2E-03
YHL041W	YHL041W	UWOPS87_ctrl_hmg124h_6.JPG.dat	1147.3	57.7	1.0	0.05	#NUM!	2.5E-05
YHL042W	YHL042W	UWOPS87_ctrl_hmg124h_6.JPG.dat	1092.8	254.8	1.0	0.24	#NUM!	4.0E-03
YHL043W	YHL043W	UWOPS87_ctrl_hmg124h_6.JPG.dat	1101.8	34.5	1.1	0.05	#NUM!	2.3E-05
YHL044W	YHL044W	UWOPS87_ctrl_hmg124h_6.JPG.dat	675.3	452.1	1.0	0.06	#NUM!	1.0E-03
YHL045W	YHL045W	UWOPS87_ctrl_hmg124h_6.JPG.dat	870.5	45.7	0.9	0.02	#NUM!	1.2E-04
YHL046C	YHL046C	UWOPS87_ctrl_hmg124h_6.JPG.dat	1037.3	79.3	1.0	0.07	#NUM!	9.6E-05
YHL047C	YHL047C	UWOPS87_ctrl_hmg124h_6.JPG.dat	954.3	166.6	1.0	0.06	#NUM!	1.3E-03
YHR001W-	YHR001W-A	UWOPS87_ctrl_hmg124h_6.JPG.dat	818.0	36.4	0.8	0.04	#NUM!	2.3E-05
YHR003C	YHR003C	UWOPS87_ctrl_hmg124h_6.JPG.dat	1029.8	40.5	1.0	0.05	#NUM!	3.1E-05
YHR004C	YHR004C	UWOPS87_ctrl_hmg124h_6.JPG.dat	950.3	45.7	1.0	0.05	#NUM!	4.3E-05
YHR005C	YHR005C	UWOPS87_ctrl_hmg124h_6.JPG.dat	977.5	109.3	0.9	0.11	#NUM!	4.1E-04
YHR006W	YHR006W	UWOPS87_ctrl_hmg124h_6.JPG.dat	1271.8	13.1	1.2	0.01	#NUM!	4.4E-07
YHR009C	YHR009C	UWOPS87_ctrl_hmg124h_6.JPG.dat	921.8	59.0	0.9	0.05	#NUM!	5.4E-05
YHR012W	YHR012W	UWOPS87_ctrl_hmg124h_6.JPG.dat	766.5	33.8	0.8	0.01	#NUM!	3.0E-05
YHR014W	YHR014W	UWOPS87_ctrl_hmg124h_6.JPG.dat	910.3	76.3	0.9	0.03	#NUM!	3.8E-04
YHR015W	YHR015W	UWOPS87_ctrl_hmg124h_6.JPG.dat	648.5	434.0	0.8	0.05	#NUM!	1.3E-03
YHR016C	YHR016C	UWOPS87_ctrl_hmg124h_6.JPG.dat	1203.3	46.0	1.1	0.02	#NUM!	8.3E-05
YHR017W	YHR017W	UWOPS87_ctrl_hmg124h_6.JPG.dat	927.5	152.9	0.9	0.14	#NUM!	1.0E-03
YHR018C	YHR018C	UWOPS87_ctrl_hmg124h_6.JPG.dat	848.3	62.5	0.8	0.05	#NUM!	6.9E-05
YHR021C	YHR021C	UWOPS87_ctrl_hmg124h_6.JPG.dat	1045.8	62.7	1.0	0.06	#NUM!	4.9E-05
YHR021W-	YHR021W-A	UWOPS87_ctrl_hmg124h_6.JPG.dat	1170.3	9.4	1.1	0.01	#NUM!	4.8E-07
YHR022C	YHR022C	UWOPS87_ctrl_hmg124h_6.JPG.dat	756.3	514.3	0.9	0.11	#NUM!	4.5E-03
YHR025W	YHR025W	UWOPS87_ctrl_hmg124h_6.JPG.dat	1006.3	60.3	0.9	0.06	#NUM!	8.8E-05
YHR028C	YHR028C	UWOPS87_ctrl_hmg124h_6.JPG.dat	982.3	212.0	0.9	0.19	#NUM!	2.4E-03
YHR029C	YHR029C	UWOPS87_ctrl_hmg124h_6.JPG.dat	1007.8	70.9	1.0	0.07	#NUM!	8.8E-05
YHR030C	YHR030C	UWOPS87_ctrl_hmg124h_6.JPG.dat	1043.5	48.8	1.1	0.05	#NUM!	1.9E-05
YHR031C	YHR031C	UWOPS87_ctrl_hmg124h_6.JPG.dat	817.8	72.1	0.9	0.09	#NUM!	3.2E-04
YHR032W	YHR032W	UWOPS87_ctrl_hmg124h_6.JPG.dat	922.3	50.6	1.0	0.05	#NUM!	4.8E-05
YHR033W	YHR033W	UWOPS87_ctrl_hmg124h_6.JPG.dat	880.5	60.8	0.9	0.01	#NUM!	6.2E-05
YHR034C	YHR034C	UWOPS87_ctrl_hmg124h_6.JPG.dat	854.0	66.1	1.0	0.08	#NUM!	1.4E-04
YHR035W	YHR035W	UWOPS87_ctrl_hmg124h_6.JPG.dat	843.0	143.9	0.9	0.13	#NUM!	8.8E-04
YHR037W	YHR037W	UWOPS87_ctrl_hmg124h_6.JPG.dat	849.0	86.4	0.9	0.09	#NUM!	2.9E-04
YHR039C	YHR039C	UWOPS87_ctrl_hmg124h_6.JPG.dat	981.8	105.9	1.1	0.04	#NUM!	4.5E-04
YHR041C	YHR041C	UWOPS87_ctrl_hmg124h_6.JPG.dat	883.0	58.4	0.9	0.07	#NUM!	1.5E-04
YHR043C	YHR043C	UWOPS87_ctrl_hmg124h_6.JPG.dat	882.8	109.1	0.9	0.13	#NUM!	7.9E-04
YHR044C	YHR044C	UWOPS87_ctrl_hmg124h_6.JPG.dat	886.5	76.2	1.0	0.08	#NUM!	1.7E-04
YHR045W	YHR045W	UWOPS87_ctrl_hmg124h_6.JPG.dat	923.5	23.9	0.9	0.02	#NUM!	2.0E-06
YHR046C	YHR046C	UWOPS87_ctrl_hmg124h_6.JPG.dat	949.0	96.3	0.9	0.09	#NUM!	2.6E-04
YHR047C	YHR047C	UWOPS87_ctrl_hmg124h_6.JPG.dat	1118.5	85.0	1.1	0.10	#NUM!	2.0E-04
YHR048W	YHR048W	UWOPS87_ctrl_hmg124h_6.JPG.dat	1008.3	64.9	1.0	0.05	#NUM!	3.6E-05
YHR049C-/	YHR049C-A	UWOPS87_ctrl_hmg124h_6.JPG.dat	862.0	68.8	0.8	0.07	#NUM!	1.4E-04
YHR049W	YHR049W	UWOPS87_ctrl_hmg124h_6.JPG.dat	1163.5	66.4	1.1	0.06	#NUM!	5.3E-05
YHR050W	YHR050W	UWOPS87_ctrl_hmg124h_6.JPG.dat	1291.5	48.5	1.2	0.05	#NUM!	1.8E-05
YHR057C	YHR057C	UWOPS87_ctrl_hmg124h_6.JPG.dat	997.5	62.1	0.9	0.07	#NUM!	1.2E-04
YHR059W	YHR059W	UWOPS87_ctrl_hmg124h_6.JPG.dat	1080.5	82.0	1.0	0.08	#NUM!	1.6E-04
YHR061C	YHR061C	UWOPS87_ctrl_hmg124h_6.JPG.dat	1248.3	80.4	1.2	0.07	#NUM!	5.5E-05
YHR066W	YHR066W	UWOPS87_ctrl_hmg124h_6.JPG.dat	1018.8	154.3	1.0	0.14	#NUM!	7.9E-04
YHR067W	YHR067W	UWOPS87_ctrl_hmg124h_6.JPG.dat	865.5	68.7	0.9	0.07	#NUM!	1.5E-04
YHR073W	YHR073W	UWOPS87_ctrl_hmg124h_6.JPG.dat	884.8	41.8	0.8	0.04	#NUM!	3.2E-05
YHR075C	YHR075C	UWOPS87_ctrl_hmg124h_6.JPG.dat	1057.8	30.5	1.0	0.03	#NUM!	7.4E-06
YHR076W	YHR076W	UWOPS87_ctrl_hmg124h_6.JPG.dat	1195.3	40.3	1.1	0.04	#NUM!	9.5E-06
YHR077C	YHR077C	UWOPS87_ctrl_hmg124h_6.JPG.dat	1046.8	7.1	1.0	0.01	#NUM!	4.1E-08
YHR078W	YHR078W	UWOPS87_ctrl_hmg124h_6.JPG.dat	1179.5	35.6	1.1	0.03	#NUM!	5.4E-06
YHR079C	YHR079C	UWOPS87_ctrl_hmg124h_6.JPG.dat	1031.8	47.3	1.0	0.05	#NUM!	2.7E-05
YHR079C-E	YHR079C-B	UWOPS87_ctrl_hmg124h_6.JPG.dat	1086.5	37.1	1.0	0.03	#NUM!	7.5E-06
YHR080C	YHR080C	UWOPS87_ctrl_hmg124h_6.JPG.dat	1109.8	12.0	1.0	0.01	#NUM!	1.7E-07
YHR081W	YHR081W	UWOPS87_ctrl_hmg124h_6.JPG.dat	951.5	27.0	0.9	0.02	#NUM!	3.9E-06
YHR082C	YHR082C	UWOPS87_ctrl_hmg124h_6.JPG.dat	1089.0	104.8	1.1	0.02	#NUM!	7.9E-05
YHR086W	YHR086W	UWOPS87_ctrl_hmg124h_6.JPG.dat	1049.5	127.5	1.0	0.11	#NUM!	3.8E-04
YHR087W	YHR087W	UWOPS87_ctrl_hmg124h_6.JPG.dat	1039.3	155.3	0.9	0.14	#NUM!	8.7E-04
YHR092C	YHR092C	UWOPS87_ctrl_hmg124h_6.JPG.dat	981.5	67.7	0.9	0.06	#NUM!	8.1E-05
YHR093W	YHR093W	UWOPS87_ctrl_hmg124h_6.JPG.dat	1080.8	154.2	1.0	0.14	#NUM!	7.7E-04
YHR094C	YHR094C	UWOPS87_ctrl_hmg124h_6.JPG.dat	1084.8	53.3	1.1	0.05	#NUM!	2.6E-05

YHR095W	YHR095W	UWOPS87_ctrl_hmg124h_6.JPG.dat	971.8	40.2	0.9	0.04	#NUM!	2.1E-05
YHR096C	YHR096C	UWOPS87_ctrl_hmg124h_6.JPG.dat	1103.5	13.0	1.0	0.01	#NUM!	6.7E-07
YHR097C	YHR097C	UWOPS87_ctrl_hmg124h_6.JPG.dat	1112.8	44.5	1.0	0.01	#NUM!	8.4E-06
YHR103W	YHR103W	UWOPS87_ctrl_hmg124h_6.JPG.dat	1157.5	48.4	1.0	0.04	#NUM!	2.2E-05
YHR104W	YHR104W	UWOPS87_ctrl_hmg124h_6.JPG.dat	1127.8	80.0	1.0	0.07	#NUM!	8.9E-05
YHR105W	YHR105W	UWOPS87_ctrl_hmg124h_6.JPG.dat	1105.8	111.1	1.0	0.10	#NUM!	2.3E-04
YHR106W	YHR106W	UWOPS87_ctrl_hmg124h_6.JPG.dat	949.0	81.3	1.0	0.10	#NUM!	2.9E-04
YHR108W	YHR108W	UWOPS87_ctrl_hmg124h_6.JPG.dat	1197.3	23.1	1.1	0.01	#NUM!	1.7E-05
YHR109W	YHR109W	UWOPS87_ctrl_hmg124h_6.JPG.dat	979.8	52.0	0.9	0.02	#NUM!	1.2E-04
YHR110W	YHR110W	UWOPS87_ctrl_hmg124h_6.JPG.dat	1102.5	37.1	1.1	0.01	#NUM!	2.2E-05
YHR111W	YHR111W	UWOPS87_ctrl_hmg124h_6.JPG.dat	953.5	122.6	0.9	0.12	#NUM!	5.1E-04
YHR112C	YHR112C	UWOPS87_ctrl_hmg124h_6.JPG.dat	895.5	69.2	0.9	0.07	#NUM!	1.2E-04
YHR113W	YHR113W	UWOPS87_ctrl_hmg124h_6.JPG.dat	1006.5	45.5	1.0	0.01	#NUM!	3.3E-05
YHR114W	YHR114W	UWOPS87_ctrl_hmg124h_6.JPG.dat	1066.5	21.7	1.0	0.02	#NUM!	4.8E-06
YHR115C	YHR115C	UWOPS87_ctrl_hmg124h_6.JPG.dat	1208.8	94.9	1.1	0.09	#NUM!	1.5E-04
YHR116W	YHR116W	UWOPS87_ctrl_hmg124h_6.JPG.dat	1197.8	164.1	1.2	0.03	#NUM!	2.8E-04
YHR117W	YHR117W	UWOPS87_ctrl_hmg124h_6.JPG.dat	955.5	69.8	0.9	0.05	#NUM!	5.3E-05
YHR121W	YHR121W	UWOPS87_ctrl_hmg124h_6.JPG.dat	905.8	32.7	0.9	0.04	#NUM!	2.1E-05
YHR123W	YHR123W	UWOPS87_ctrl_hmg124h_6.JPG.dat	1080.0	108.0	1.0	0.09	#NUM!	2.0E-04
YHR124W	YHR124W	UWOPS87_ctrl_hmg124h_6.JPG.dat	979.0	76.2	0.9	0.08	#NUM!	1.8E-04
YHR125W	YHR125W	UWOPS87_ctrl_hmg124h_6.JPG.dat	804.8	115.8	0.7	0.02	#NUM!	2.3E-04
YHR126C	YHR126C	UWOPS87_ctrl_hmg124h_6.JPG.dat	1033.3	132.5	1.0	0.14	#NUM!	6.9E-04
YHR127W	YHR127W	UWOPS87_ctrl_hmg124h_6.JPG.dat	916.0	92.9	0.9	0.09	#NUM!	2.8E-04
YHR129C	YHR129C	UWOPS87_ctrl_hmg124h_6.JPG.dat	961.3	61.1	0.9	0.05	#NUM!	3.8E-05
YHR130C	YHR130C	UWOPS87_ctrl_hmg124h_6.JPG.dat	1170.5	49.4	1.1	0.01	#NUM!	4.4E-05
YHR131C	YHR131C	UWOPS87_ctrl_hmg124h_6.JPG.dat	1111.3	33.1	1.0	0.04	#NUM!	1.5E-05
YHR132C	YHR132C	UWOPS87_ctrl_hmg124h_6.JPG.dat	1008.0	160.7	0.9	0.14	#NUM!	8.7E-04
YHR132W-	YHR132W-A	UWOPS87_ctrl_hmg124h_6.JPG.dat	1045.5	72.6	1.0	0.07	#NUM!	7.7E-05
YHR133C	YHR133C	UWOPS87_ctrl_hmg124h_6.JPG.dat	837.5	97.7	0.9	0.03	#NUM!	2.6E-04
YHR134W	YHR134W	UWOPS87_ctrl_hmg124h_6.JPG.dat	788.0	530.7	1.0	0.09	#NUM!	2.8E-03
YHR135C	YHR135C	UWOPS87_ctrl_hmg124h_6.JPG.dat	859.5	164.2	0.7	0.05	#NUM!	1.9E-03
YHR136C	YHR136C	UWOPS87_ctrl_hmg124h_6.JPG.dat	993.3	50.2	1.0	0.05	#NUM!	4.1E-05
YHR137W	YHR137W	UWOPS87_ctrl_hmg124h_6.JPG.dat	967.0	88.1	1.0	0.10	#NUM!	2.9E-04
YHR138C	YHR138C	UWOPS87_ctrl_hmg124h_6.JPG.dat	1149.8	22.2	1.1	0.02	#NUM!	1.1E-06
YHR139C	YHR139C	UWOPS87_ctrl_hmg124h_6.JPG.dat	1015.0	48.9	1.0	0.05	#NUM!	3.0E-05
YHR139C-/	YHR139C-A	UWOPS87_ctrl_hmg124h_6.JPG.dat	1113.3	101.1	1.0	0.09	#NUM!	1.8E-04
YHR140W	YHR140W	UWOPS87_ctrl_hmg124h_6.JPG.dat	1133.3	57.4	1.0	0.05	#NUM!	3.9E-05
YHR142W	YHR142W	UWOPS87_ctrl_hmg124h_6.JPG.dat	1263.0	38.9	1.2	0.03	#NUM!	5.6E-06
YHR143W	YHR143W	UWOPS87_ctrl_hmg124h_6.JPG.dat	975.5	47.5	1.0	0.04	#NUM!	2.4E-05
YHR146W	YHR146W	UWOPS87_ctrl_hmg124h_6.JPG.dat	1015.5	29.1	1.0	0.06	#NUM!	4.4E-05
YHR150W	YHR150W	UWOPS87_ctrl_hmg124h_6.JPG.dat	588.8	397.9	0.8	0.10	#NUM!	5.4E-03
YHR151C	YHR151C	UWOPS87_ctrl_hmg124h_6.JPG.dat	1273.0	93.0	1.2	0.09	#NUM!	1.2E-04
YHR152W	YHR152W	UWOPS87_ctrl_hmg124h_6.JPG.dat	999.5	10.5	1.0	0.02	#NUM!	2.5E-06
YHR153C	YHR153C	UWOPS87_ctrl_hmg124h_6.JPG.dat	854.5	85.5	0.8	0.02	#NUM!	2.5E-04
YHR154W	YHR154W	UWOPS87_ctrl_hmg124h_6.JPG.dat	1177.8	62.1	1.1	0.03	#NUM!	8.0E-06
YHR155W	YHR155W	UWOPS87_ctrl_hmg124h_6.JPG.dat	1143.0	45.0	1.1	0.01	#NUM!	6.6E-07
YHR156C	YHR156C	UWOPS87_ctrl_hmg124h_6.JPG.dat	1095.5	30.8	1.0	0.04	#NUM!	2.0E-05
YHR157W	YHR157W	UWOPS87_ctrl_hmg124h_6.JPG.dat	1090.8	172.8	0.9	0.07	#NUM!	1.7E-03
YHR158C	YHR158C	UWOPS87_ctrl_hmg124h_6.JPG.dat	952.3	66.1	0.9	0.08	#NUM!	1.4E-04
YHR159W	YHR159W	UWOPS87_ctrl_hmg124h_6.JPG.dat	955.0	23.4	0.9	0.01	#NUM!	6.2E-05
YHR160C	YHR160C	UWOPS87_ctrl_hmg124h_6.JPG.dat	946.5	23.8	0.9	0.02	#NUM!	4.3E-06
YHR161C	YHR161C	UWOPS87_ctrl_hmg124h_6.JPG.dat	962.8	19.8	0.9	0.01	#NUM!	7.7E-08
YHR162W	YHR162W	UWOPS87_ctrl_hmg124h_6.JPG.dat	924.3	176.9	0.9	0.15	#NUM!	1.5E-03
YHR163W	YHR163W	UWOPS87_ctrl_hmg124h_6.JPG.dat	1016.0	89.4	1.0	0.10	#NUM!	2.8E-04
YHR167W	YHR167W	UWOPS87_ctrl_hmg124h_6.JPG.dat	1222.5	31.8	1.2	0.04	#NUM!	1.2E-05
YHR171W	YHR171W	UWOPS87_ctrl_hmg124h_6.JPG.dat	1023.8	64.6	1.0	0.05	#NUM!	3.8E-05
YHR176W	YHR176W	UWOPS87_ctrl_hmg124h_6.JPG.dat	1203.0	54.8	1.2	0.02	#NUM!	6.2E-05
YHR178W	YHR178W	UWOPS87_ctrl_hmg124h_6.JPG.dat	749.8	52.1	0.7	0.06	#NUM!	1.6E-04
YHR179W	YHR179W	UWOPS87_ctrl_hmg124h_6.JPG.dat	1070.3	97.1	1.0	0.08	#NUM!	1.5E-04
YHR180W	YHR180W	UWOPS87_ctrl_hmg124h_6.JPG.dat	1195.5	87.5	1.2	0.01	#NUM!	4.0E-05
YHR181W	YHR181W	UWOPS87_ctrl_hmg124h_6.JPG.dat	1028.0	228.7	1.0	0.22	#NUM!	2.8E-03
YHR182W	YHR182W	UWOPS87_ctrl_hmg124h_6.JPG.dat	1056.5	22.6	1.1	0.02	#NUM!	2.2E-06
YHR184W	YHR184W	UWOPS87_ctrl_hmg124h_6.JPG.dat	1103.5	44.5	1.1	0.04	#NUM!	1.3E-05
YHR185C	YHR185C	UWOPS87_ctrl_hmg124h_6.JPG.dat	974.3	108.2	0.9	0.10	#NUM!	3.3E-04
YHR189W	YHR189W	UWOPS87_ctrl_hmg124h_6.JPG.dat	1257.5	41.9	1.2	0.04	#NUM!	1.5E-05
YHR191C	YHR191C	UWOPS87_ctrl_hmg124h_6.JPG.dat	940.0	38.2	0.9	0.05	#NUM!	3.5E-05
YHR193C	YHR193C	UWOPS87_ctrl_hmg124h_6.JPG.dat	1039.0	47.5	1.0	0.04	#NUM!	2.0E-05
YHR194W	YHR194W	UWOPS87_ctrl_hmg124h_6.JPG.dat	1030.8	40.5	1.0	0.04	#NUM!	1.3E-05
YHR195W	YHR195W	UWOPS87_ctrl_hmg124h_6.JPG.dat	1061.3	59.1	1.0	0.04	#NUM!	2.2E-05
YHR198C	YHR198C	UWOPS87_ctrl_hmg124h_6.JPG.dat	1132.3	67.5	1.0	0.06	#NUM!	5.5E-05

YHR199C	YHR199C	UWOPS87_ctrl_hmg124h_6.JPG.dat	1070.0	27.2	1.0	0.00	#NUM!	4.4E-06
YHR200W	YHR200W	UWOPS87_ctrl_hmg124h_6.JPG.dat	1148.5	94.0	1.1	0.09	#NUM!	1.5E-04
YHR202W	YHR202W	UWOPS87_ctrl_hmg124h_6.JPG.dat	1144.8	45.7	1.1	0.02	#NUM!	7.6E-05
YHR203C	YHR203C	UWOPS87_ctrl_hmg124h_6.JPG.dat	847.3	18.0	0.8	0.00	#NUM!	6.9E-07
YHR204W	YHR204W	UWOPS87_ctrl_hmg124h_6.JPG.dat	1175.0	59.7	1.1	0.05	#NUM!	3.6E-05
YHR206W	YHR206W	UWOPS87_ctrl_hmg124h_6.JPG.dat	896.0	63.7	0.8	0.06	#NUM!	9.1E-05
YHR207C	YHR207C	UWOPS87_ctrl_hmg124h_6.JPG.dat	1132.3	14.1	1.1	0.01	#NUM!	2.4E-05
YHR209W	YHR209W	UWOPS87_ctrl_hmg124h_6.JPG.dat	1058.8	197.3	1.1	0.05	#NUM!	7.4E-04
YHR210C	YHR210C	UWOPS87_ctrl_hmg124h_6.JPG.dat	903.8	89.4	0.9	0.09	#NUM!	2.9E-04
YIL001W	YIL001W	UWOPS87_ctrl_hmg124h_6.JPG.dat	1214.3	15.2	1.1	0.01	#NUM!	5.1E-07
YIL002C	YIL002C	UWOPS87_ctrl_hmg124h_6.JPG.dat	1074.0	41.5	1.0	0.03	#NUM!	8.7E-06
YIL005W	YIL005W	UWOPS87_ctrl_hmg124h_6.JPG.dat	1221.3	19.2	1.1	0.02	#NUM!	1.2E-06
YIL006W	YIL006W	UWOPS87_ctrl_hmg124h_6.JPG.dat	1196.0	7.2	1.1	0.00	#NUM!	2.6E-06
YIL007C	YIL007C	UWOPS87_ctrl_hmg124h_6.JPG.dat	867.0	34.1	0.9	0.01	#NUM!	3.7E-05
YIL008W	YIL008W	UWOPS87_ctrl_hmg124h_6.JPG.dat	1230.0	15.2	1.2	0.03	#NUM!	4.0E-06
YIL009C-A	YIL009C-A	UWOPS87_ctrl_hmg124h_6.JPG.dat	0.0	0.0	0.0	0.00	#NUM!	
YIL010W	YIL010W	UWOPS87_ctrl_hmg124h_6.JPG.dat	1073.8	104.5	1.1	0.02	#NUM!	8.7E-05
YIL011W	YIL011W	UWOPS87_ctrl_hmg124h_6.JPG.dat	1021.0	41.6	1.0	0.04	#NUM!	1.6E-05
YIL012W	YIL012W	UWOPS87_ctrl_hmg124h_6.JPG.dat	880.8	105.9	0.8	0.04	#NUM!	6.3E-04
YIL013C	YIL013C	UWOPS87_ctrl_hmg124h_6.JPG.dat	1074.3	23.5	1.1	0.03	#NUM!	6.8E-06
YIL014W	YIL014W	UWOPS87_ctrl_hmg124h_6.JPG.dat	1045.8	9.5	1.0	0.03	#NUM!	5.7E-06
YIL015C-A	YIL015C-A	UWOPS87_ctrl_hmg124h_6.JPG.dat	1075.0	42.0	1.0	0.03	#NUM!	8.1E-06
YIL015W	YIL015W	UWOPS87_ctrl_hmg124h_6.JPG.dat	1165.8	32.9	1.0	0.03	#NUM!	8.0E-06
YIL016W	YIL016W	UWOPS87_ctrl_hmg124h_6.JPG.dat	1139.3	32.1	1.1	0.04	#NUM!	9.9E-06
YIL017C	YIL017C	UWOPS87_ctrl_hmg124h_6.JPG.dat	1133.0	96.0	1.2	0.11	#NUM!	1.9E-04
YIL020C	YIL020C	UWOPS87_ctrl_hmg124h_6.JPG.dat	1045.3	180.6	1.0	0.17	#NUM!	1.3E-03
YIL023C	YIL023C	UWOPS87_ctrl_hmg124h_6.JPG.dat	1131.8	130.3	1.1	0.11	#NUM!	3.1E-04
YIL024C	YIL024C	UWOPS87_ctrl_hmg124h_6.JPG.dat	1176.3	17.8	1.1	0.03	#NUM!	3.5E-06
YIL025C	YIL025C	UWOPS87_ctrl_hmg124h_6.JPG.dat	1143.8	16.4	1.1	0.02	#NUM!	1.1E-06
YIL027C	YIL027C	UWOPS87_ctrl_hmg124h_6.JPG.dat	939.3	86.9	0.9	0.02	#NUM!	1.4E-04
YIL028W	YIL028W	UWOPS87_ctrl_hmg124h_6.JPG.dat	1063.0	39.5	1.1	0.01	#NUM!	6.1E-05
YIL029C	YIL029C	UWOPS87_ctrl_hmg124h_6.JPG.dat	1051.3	126.6	1.0	0.11	#NUM!	4.1E-04
YIL030C	YIL030C	UWOPS87_ctrl_hmg124h_6.JPG.dat	1090.0	54.5	1.0	0.06	#NUM!	5.7E-05
YIL032C	YIL032C	UWOPS87_ctrl_hmg124h_6.JPG.dat	1026.3	176.2	0.9	0.15	#NUM!	1.2E-03
YIL034C	YIL034C	UWOPS87_ctrl_hmg124h_6.JPG.dat	1028.0	154.1	1.0	0.15	#NUM!	8.8E-04
YIL035C	YIL035C	UWOPS87_ctrl_hmg124h_6.JPG.dat	879.3	93.4	0.9	0.04	#NUM!	5.7E-04
YIL036W	YIL036W	UWOPS87_ctrl_hmg124h_6.JPG.dat	1120.3	69.8	1.1	0.07	#NUM!	6.4E-05
YIL037C	YIL037C	UWOPS87_ctrl_hmg124h_6.JPG.dat	1267.3	17.7	1.2	0.02	#NUM!	1.3E-06
YIL038C	YIL038C	UWOPS87_ctrl_hmg124h_6.JPG.dat	1131.8	81.3	1.1	0.07	#NUM!	6.8E-05
YIL039W	YIL039W	UWOPS87_ctrl_hmg124h_6.JPG.dat	809.0	33.4	0.8	0.01	#NUM!	5.8E-05
YIL040W	YIL040W	UWOPS87_ctrl_hmg124h_6.JPG.dat	1014.8	52.2	1.0	0.02	#NUM!	1.4E-04
YIL041W	YIL041W	UWOPS87_ctrl_hmg124h_6.JPG.dat	925.0	64.0	0.9	0.06	#NUM!	7.7E-05
YIL042C	YIL042C	UWOPS87_ctrl_hmg124h_6.JPG.dat	1116.8	110.7	1.0	0.09	#NUM!	1.9E-04
YIL043C	YIL043C	UWOPS87_ctrl_hmg124h_6.JPG.dat	1095.3	49.2	1.0	0.01	#NUM!	6.8E-05
YIL044C	YIL044C	UWOPS87_ctrl_hmg124h_6.JPG.dat	1024.8	105.7	1.0	0.10	#NUM!	3.1E-04
YIL045W	YIL045W	UWOPS87_ctrl_hmg124h_6.JPG.dat	833.0	47.6	0.9	0.06	#NUM!	7.8E-05
YIL047C	YIL047C	UWOPS87_ctrl_hmg124h_6.JPG.dat	901.8	73.1	0.9	0.09	#NUM!	2.4E-04
YIL049W	YIL049W	UWOPS87_ctrl_hmg124h_6.JPG.dat	1227.0	61.2	1.2	0.07	#NUM!	6.2E-05
YIL050W	YIL050W	UWOPS87_ctrl_hmg124h_6.JPG.dat	621.0	414.3	0.8	0.04	#NUM!	8.1E-04
YIL052C	YIL052C	UWOPS87_ctrl_hmg124h_6.JPG.dat	1031.0	40.1	1.0	0.01	#NUM!	3.9E-05
YIL053W	YIL053W	UWOPS87_ctrl_hmg124h_6.JPG.dat	1002.0	189.8	1.0	0.17	#NUM!	1.3E-03
YIL054W	YIL054W	UWOPS87_ctrl_hmg124h_6.JPG.dat	1170.5	77.2	1.1	0.06	#NUM!	4.2E-05
YIL055C	YIL055C	UWOPS87_ctrl_hmg124h_6.JPG.dat	1087.3	79.7	1.0	0.06	#NUM!	4.5E-05
YIL056W	YIL056W	UWOPS87_ctrl_hmg124h_6.JPG.dat	1039.5	83.5	1.0	0.07	#NUM!	9.4E-05
YIL057C	YIL057C	UWOPS87_ctrl_hmg124h_6.JPG.dat	1068.8	106.5	1.0	0.11	#NUM!	3.3E-04
YIL058W	YIL058W	UWOPS87_ctrl_hmg124h_6.JPG.dat	1127.0	138.3	1.1	0.05	#NUM!	5.5E-04
YIL059C	YIL059C	UWOPS87_ctrl_hmg124h_6.JPG.dat	852.0	14.7	0.9	0.04	#NUM!	3.1E-05
YOR202W	YOR202W	UWOPS87_ctrl_hmg124h_6.JPG.dat	1087.0	204.3	1.0	0.14	#NUM!	3.1E-232
1	1	UWOPS87_ctrl_hmg124h_7.JPG.dat	1692.0	290.4	1.1	0.05	#NUM!	2.5E-05
2	2	UWOPS87_ctrl_hmg124h_7.JPG.dat	1404.8	186.0	1.1	0.08	#NUM!	9.6E-05
3	3	UWOPS87_ctrl_hmg124h_7.JPG.dat	1258.3	153.9	0.9	0.06	#NUM!	9.5E-05
4	4	UWOPS87_ctrl_hmg124h_7.JPG.dat	1270.0	254.7	1.0	0.14	#NUM!	7.7E-04
YIL060W	YIL060W	UWOPS87_ctrl_hmg124h_7.JPG.dat	828.5	56.1	0.8	0.05	#NUM!	7.9E-05
YIL064W	YIL064W	UWOPS87_ctrl_hmg124h_7.JPG.dat	1061.5	110.0	0.9	0.10	#NUM!	3.2E-04
YIL065C	YIL065C	UWOPS87_ctrl_hmg124h_7.JPG.dat	815.3	544.3	0.9	0.03	#NUM!	3.8E-04
YIL066C	YIL066C	UWOPS87_ctrl_hmg124h_7.JPG.dat	1086.0	126.0	0.9	0.10	#NUM!	3.4E-04
YIL067C	YIL067C	UWOPS87_ctrl_hmg124h_7.JPG.dat	1152.5	64.1	0.9	0.02	#NUM!	1.2E-04
YIL070C	YIL070C	UWOPS87_ctrl_hmg124h_7.JPG.dat	1320.5	29.8	1.1	0.01	#NUM!	2.4E-05
YIL071C	YIL071C	UWOPS87_ctrl_hmg124h_7.JPG.dat	1035.8	21.2	0.9	0.00	#NUM!	4.6E-06
YIL072W	YIL072W	UWOPS87_ctrl_hmg124h_7.JPG.dat	1156.0	47.7	1.0	0.05	#NUM!	3.5E-05

YIL073C	YIL073C	UWOPS87_ctrl_hmg124h_7.JPG.dat	1149.3	258.9	0.9	0.20	#NUM!	2.8E-03
YIL074C	YIL074C	UWOPS87_ctrl_hmg124h_7.JPG.dat	1020.0	33.1	0.9	0.02	#NUM!	5.9E-06
YIL076W	YIL076W	UWOPS87_ctrl_hmg124h_7.JPG.dat	1149.5	87.7	1.0	0.06	#NUM!	7.7E-05
YIL077C	YIL077C	UWOPS87_ctrl_hmg124h_7.JPG.dat	943.0	61.7	0.9	0.06	#NUM!	9.4E-05
YIL079C	YIL079C	UWOPS87_ctrl_hmg124h_7.JPG.dat	1175.8	28.6	1.0	0.02	#NUM!	2.8E-06
YIL084C	YIL084C	UWOPS87_ctrl_hmg124h_7.JPG.dat	0.0	0.0	0.0	0.00	#NUM!	
YIL086C	YIL086C	UWOPS87_ctrl_hmg124h_7.JPG.dat	1258.0	29.8	1.0	0.03	#NUM!	6.0E-06
YIL087C	YIL087C	UWOPS87_ctrl_hmg124h_7.JPG.dat	1267.8	24.1	1.0	0.03	#NUM!	4.5E-06
YIL088C	YIL088C	UWOPS87_ctrl_hmg124h_7.JPG.dat	1118.5	97.3	0.9	0.08	#NUM!	2.1E-04
YIL089W	YIL089W	UWOPS87_ctrl_hmg124h_7.JPG.dat	1006.8	68.9	0.9	0.06	#NUM!	8.4E-05
YIL090W	YIL090W	UWOPS87_ctrl_hmg124h_7.JPG.dat	842.3	580.2	0.9	0.15	#NUM!	8.8E-03
YIL092W	YIL092W	UWOPS87_ctrl_hmg124h_7.JPG.dat	1343.3	73.6	1.0	0.05	#NUM!	3.5E-05
YIL093C	YIL093C	UWOPS87_ctrl_hmg124h_7.JPG.dat	1007.5	126.8	0.8	0.10	#NUM!	4.7E-04
YIL095W	YIL095W	UWOPS87_ctrl_hmg124h_7.JPG.dat	1071.0	75.2	0.9	0.07	#NUM!	1.2E-04
YIL096C	YIL096C	UWOPS87_ctrl_hmg124h_7.JPG.dat	935.3	34.4	0.9	0.00	#NUM!	6.9E-06
YIL097W	YIL097W	UWOPS87_ctrl_hmg124h_7.JPG.dat	1210.0	72.3	1.1	0.07	#NUM!	7.3E-05
YIL098C	YIL098C	UWOPS87_ctrl_hmg124h_7.JPG.dat	1143.5	27.1	1.0	0.02	#NUM!	3.1E-06
YIL099W	YIL099W	UWOPS87_ctrl_hmg124h_7.JPG.dat	1157.3	65.2	1.0	0.01	#NUM!	2.4E-05
YIL100W	YIL100W	UWOPS87_ctrl_hmg124h_7.JPG.dat	1158.8	26.4	1.0	0.02	#NUM!	2.6E-06
YIL101C	YIL101C	UWOPS87_ctrl_hmg124h_7.JPG.dat	1088.5	45.6	1.0	0.05	#NUM!	3.2E-05
YIL103W	YIL103W	UWOPS87_ctrl_hmg124h_7.JPG.dat	1058.3	43.1	1.0	0.04	#NUM!	2.2E-05
YIL105C	YIL105C	UWOPS87_ctrl_hmg124h_7.JPG.dat	1120.0	96.7	1.0	0.08	#NUM!	1.4E-04
YIL107C	YIL107C	UWOPS87_ctrl_hmg124h_7.JPG.dat	1199.5	71.3	1.0	0.06	#NUM!	7.8E-05
YIL108W	YIL108W	UWOPS87_ctrl_hmg124h_7.JPG.dat	1318.0	73.2	1.1	0.05	#NUM!	2.8E-05
YIL110W	YIL110W	UWOPS87_ctrl_hmg124h_7.JPG.dat	908.3	14.7	0.8	0.00	#NUM!	4.1E-06
YIL112W	YIL112W	UWOPS87_ctrl_hmg124h_7.JPG.dat	1047.5	70.6	1.0	0.06	#NUM!	4.8E-05
YIL113W	YIL113W	UWOPS87_ctrl_hmg124h_7.JPG.dat	1104.3	67.3	0.9	0.05	#NUM!	4.8E-05
YIL114C	YIL114C	UWOPS87_ctrl_hmg124h_7.JPG.dat	1118.0	18.2	1.0	0.02	#NUM!	3.4E-06
YIL116W	YIL116W	UWOPS87_ctrl_hmg124h_7.JPG.dat	1064.8	32.2	0.9	0.01	#NUM!	1.8E-05
YIL117C	YIL117C	UWOPS87_ctrl_hmg124h_7.JPG.dat	1148.3	46.0	1.0	0.03	#NUM!	9.3E-06
YIL119C	YIL119C	UWOPS87_ctrl_hmg124h_7.JPG.dat	1203.0	32.4	1.1	0.03	#NUM!	7.6E-06
YIL120W	YIL120W	UWOPS87_ctrl_hmg124h_7.JPG.dat	1101.5	71.6	1.0	0.07	#NUM!	1.1E-04
YIL123W	YIL123W	UWOPS87_ctrl_hmg124h_7.JPG.dat	1114.0	131.3	1.0	0.10	#NUM!	3.3E-04
YIL124W	YIL124W	UWOPS87_ctrl_hmg124h_7.JPG.dat	1338.3	74.2	1.1	0.02	#NUM!	1.5E-04
YIL128W	YIL128W	UWOPS87_ctrl_hmg124h_7.JPG.dat	1216.5	49.8	1.0	0.03	#NUM!	8.8E-06
YIL130W	YIL130W	UWOPS87_ctrl_hmg124h_7.JPG.dat	1021.5	109.0	0.9	0.09	#NUM!	2.6E-04
YIL132C	YIL132C	UWOPS87_ctrl_hmg124h_7.JPG.dat	887.0	30.5	0.8	0.00	#NUM!	4.2E-06
YIL133C	YIL133C	UWOPS87_ctrl_hmg124h_7.JPG.dat	1020.5	33.3	0.9	0.04	#NUM!	1.7E-05
YIL134W	YIL134W	UWOPS87_ctrl_hmg124h_7.JPG.dat	1325.5	30.5	1.2	0.03	#NUM!	3.4E-06
YIL135C	YIL135C	UWOPS87_ctrl_hmg124h_7.JPG.dat	1231.5	18.6	1.1	0.01	#NUM!	5.0E-07
YIL137C	YIL137C	UWOPS87_ctrl_hmg124h_7.JPG.dat	973.5	86.4	0.9	0.08	#NUM!	2.2E-04
YIL138C	YIL138C	UWOPS87_ctrl_hmg124h_7.JPG.dat	1147.0	37.8	1.0	0.04	#NUM!	1.2E-05
YIL139C	YIL139C	UWOPS87_ctrl_hmg124h_7.JPG.dat	1249.3	59.3	1.2	0.06	#NUM!	3.5E-05
YIL140W	YIL140W	UWOPS87_ctrl_hmg124h_7.JPG.dat	1151.8	34.1	1.0	0.03	#NUM!	4.9E-06
YIL141W	YIL141W	UWOPS87_ctrl_hmg124h_7.JPG.dat	1181.3	61.9	1.0	0.07	#NUM!	7.3E-05
YIL145C	YIL145C	UWOPS87_ctrl_hmg124h_7.JPG.dat	990.0	188.3	0.9	0.17	#NUM!	1.9E-03
YIL146C	YIL146C	UWOPS87_ctrl_hmg124h_7.JPG.dat	1275.8	74.6	1.1	0.05	#NUM!	2.0E-05
YIL148W	YIL148W	UWOPS87_ctrl_hmg124h_7.JPG.dat	895.0	41.4	0.9	0.05	#NUM!	5.2E-05
YIL149C	YIL149C	UWOPS87_ctrl_hmg124h_7.JPG.dat	1136.3	56.0	1.0	0.06	#NUM!	6.7E-05
YIL152W	YIL152W	UWOPS87_ctrl_hmg124h_7.JPG.dat	1125.0	39.3	1.0	0.04	#NUM!	1.3E-05
YIL153W	YIL153W	UWOPS87_ctrl_hmg124h_7.JPG.dat	1153.5	106.1	1.0	0.08	#NUM!	1.4E-04
YIL154C	YIL154C	UWOPS87_ctrl_hmg124h_7.JPG.dat	1084.0	39.7	1.0	0.04	#NUM!	2.0E-05
YIL155C	YIL155C	UWOPS87_ctrl_hmg124h_7.JPG.dat	1168.5	23.3	1.1	0.00	#NUM!	1.3E-06
YIL156W	YIL156W	UWOPS87_ctrl_hmg124h_7.JPG.dat	1182.8	104.5	1.1	0.10	#NUM!	1.8E-04
YIL157C	YIL157C	UWOPS87_ctrl_hmg124h_7.JPG.dat	1124.5	54.3	1.1	0.04	#NUM!	1.5E-05
YIL159W	YIL159W	UWOPS87_ctrl_hmg124h_7.JPG.dat	1104.8	76.9	1.0	0.05	#NUM!	4.4E-05
YIL160C	YIL160C	UWOPS87_ctrl_hmg124h_7.JPG.dat	1200.8	147.6	1.1	0.14	#NUM!	6.4E-04
YIL161W	YIL161W	UWOPS87_ctrl_hmg124h_7.JPG.dat	1178.3	128.3	1.0	0.11	#NUM!	2.9E-04
YIL162W	YIL162W	UWOPS87_ctrl_hmg124h_7.JPG.dat	954.0	84.5	0.9	0.03	#NUM!	4.6E-04
YIL163C	YIL163C	UWOPS87_ctrl_hmg124h_7.JPG.dat	1301.0	65.7	1.1	0.07	#NUM!	8.3E-05
YIL164C	YIL164C	UWOPS87_ctrl_hmg124h_7.JPG.dat	1338.0	69.0	1.1	0.07	#NUM!	6.5E-05
YIL165C	YIL165C	UWOPS87_ctrl_hmg124h_7.JPG.dat	1201.8	139.2	1.0	0.11	#NUM!	3.6E-04
YIL166C	YIL166C	UWOPS87_ctrl_hmg124h_7.JPG.dat	1216.0	82.8	1.1	0.07	#NUM!	6.3E-05
YIL167W	YIL167W	UWOPS87_ctrl_hmg124h_7.JPG.dat	925.0	83.8	0.8	0.06	#NUM!	8.6E-05
YIL168W	YIL168W	UWOPS87_ctrl_hmg124h_7.JPG.dat	1142.8	216.3	1.0	0.04	#NUM!	5.0E-04
YIL170W	YIL170W	UWOPS87_ctrl_hmg124h_7.JPG.dat	1166.5	266.2	1.0	0.06	#NUM!	1.3E-03
YIL173W	YIL173W	UWOPS87_ctrl_hmg124h_7.JPG.dat	1177.3	63.7	1.1	0.08	#NUM!	1.2E-04
YIR001C	YIR001C	UWOPS87_ctrl_hmg124h_7.JPG.dat	1141.3	105.8	1.0	0.10	#NUM!	2.9E-04
YIR002C	YIR002C	UWOPS87_ctrl_hmg124h_7.JPG.dat	1037.5	155.4	0.9	0.12	#NUM!	5.5E-04
YIR003W	YIR003W	UWOPS87_ctrl_hmg124h_7.JPG.dat	1293.8	23.3	1.2	0.03	#NUM!	6.3E-06

YIR005W	YIR005W	UWOPS87_ctrl_hmg124h_7.JPG.dat	1329.8	29.1	1.2	0.01	#NUM!	2.1E-05
YIR007W	YIR007W	UWOPS87_ctrl_hmg124h_7.JPG.dat	1189.5	21.8	1.0	0.03	#NUM!	6.3E-06
YIR009W	YIR009W	UWOPS87_ctrl_hmg124h_7.JPG.dat	1103.5	42.0	0.9	0.03	#NUM!	7.0E-06
YIR013C	YIR013C	UWOPS87_ctrl_hmg124h_7.JPG.dat	1357.8	73.4	1.1	0.06	#NUM!	5.0E-05
YIR014W	YIR014W	UWOPS87_ctrl_hmg124h_7.JPG.dat	1357.0	36.8	1.1	0.01	#NUM!	1.9E-05
YIR016W	YIR016W	UWOPS87_ctrl_hmg124h_7.JPG.dat	1239.0	46.8	1.0	0.04	#NUM!	1.6E-05
YIR017C	YIR017C	UWOPS87_ctrl_hmg124h_7.JPG.dat	1269.3	25.2	1.1	0.03	#NUM!	6.1E-06
YIR018W	YIR018W	UWOPS87_ctrl_hmg124h_7.JPG.dat	1303.3	31.6	1.0	0.03	#NUM!	5.2E-06
YIR019C	YIR019C	UWOPS87_ctrl_hmg124h_7.JPG.dat	1309.3	55.2	1.1	0.02	#NUM!	1.2E-04
YIR020C	YIR020C	UWOPS87_ctrl_hmg124h_7.JPG.dat	1317.0	27.3	1.1	0.03	#NUM!	3.7E-06
YIR020W-E	YIR020W-B	UWOPS87_ctrl_hmg124h_7.JPG.dat	1343.5	18.4	1.2	0.01	#NUM!	4.8E-05
YIR023W	YIR023W	UWOPS87_ctrl_hmg124h_7.JPG.dat	1155.5	63.6	1.0	0.04	#NUM!	1.3E-05
YIR024C	YIR024C	UWOPS87_ctrl_hmg124h_7.JPG.dat	1365.8	32.0	1.1	0.03	#NUM!	4.1E-06
YIR025W	YIR025W	UWOPS87_ctrl_hmg124h_7.JPG.dat	1283.5	36.2	1.0	0.03	#NUM!	5.5E-06
YIR027C	YIR027C	UWOPS87_ctrl_hmg124h_7.JPG.dat	1180.3	10.5	1.0	0.00	#NUM!	1.3E-06
YIR028W	YIR028W	UWOPS87_ctrl_hmg124h_7.JPG.dat	1190.0	22.0	1.0	0.00	#NUM!	3.4E-06
YIR029W	YIR029W	UWOPS87_ctrl_hmg124h_7.JPG.dat	1349.8	49.0	1.1	0.04	#NUM!	1.6E-05
YIR030C	YIR030C	UWOPS87_ctrl_hmg124h_7.JPG.dat	1196.3	24.6	1.0	0.00	#NUM!	4.0E-06
YIR031C	YIR031C	UWOPS87_ctrl_hmg124h_7.JPG.dat	1393.5	56.6	1.1	0.01	#NUM!	3.4E-05
YIR032C	YIR032C	UWOPS87_ctrl_hmg124h_7.JPG.dat	1421.0	38.0	1.1	0.03	#NUM!	8.0E-06
YIR033W	YIR033W	UWOPS87_ctrl_hmg124h_7.JPG.dat	1499.8	42.5	1.2	0.04	#NUM!	7.5E-06
YIR034C	YIR034C	UWOPS87_ctrl_hmg124h_7.JPG.dat	871.5	56.3	0.9	0.05	#NUM!	5.1E-05
YIR035C	YIR035C	UWOPS87_ctrl_hmg124h_7.JPG.dat	1252.5	47.0	1.1	0.03	#NUM!	4.2E-06
YIR036C	YIR036C	UWOPS87_ctrl_hmg124h_7.JPG.dat	1386.0	45.5	1.2	0.03	#NUM!	3.9E-06
YIR037W	YIR037W	UWOPS87_ctrl_hmg124h_7.JPG.dat	1139.0	26.0	1.0	0.02	#NUM!	2.1E-06
YIR038C	YIR038C	UWOPS87_ctrl_hmg124h_7.JPG.dat	1069.8	35.0	1.0	0.00	#NUM!	3.9E-06
YIR039C	YIR039C	UWOPS87_ctrl_hmg124h_7.JPG.dat	1109.8	23.4	1.0	0.03	#NUM!	5.6E-06
YIR042C	YIR042C	UWOPS87_ctrl_hmg124h_7.JPG.dat	1170.3	57.2	1.0	0.05	#NUM!	3.5E-05
YIR043C	YIR043C	UWOPS87_ctrl_hmg124h_7.JPG.dat	1187.8	44.1	1.0	0.02	#NUM!	3.1E-06
YIR044C	YIR044C	UWOPS87_ctrl_hmg124h_7.JPG.dat	1394.5	77.5	1.1	0.06	#NUM!	5.3E-05
YJL004C	YJL004C	UWOPS87_ctrl_hmg124h_7.JPG.dat	0.0	0.0	0.0	0.00	#NUM!	
YJL007C	YJL007C	UWOPS87_ctrl_hmg124h_7.JPG.dat	1218.3	113.1	1.0	0.03	#NUM!	3.0E-04
YJL012C	YJL012C	UWOPS87_ctrl_hmg124h_7.JPG.dat	1052.8	112.8	1.0	0.12	#NUM!	3.7E-04
YJL013C	YJL013C	UWOPS87_ctrl_hmg124h_7.JPG.dat	1116.8	87.4	1.0	0.07	#NUM!	1.0E-04
YJL016W	YJL016W	UWOPS87_ctrl_hmg124h_7.JPG.dat	1060.5	57.9	0.9	0.05	#NUM!	3.1E-05
YJL017W	YJL017W	UWOPS87_ctrl_hmg124h_7.JPG.dat	997.8	61.7	0.9	0.05	#NUM!	5.9E-05
YJL020C	YJL020C	UWOPS87_ctrl_hmg124h_7.JPG.dat	1152.0	26.3	1.1	0.01	#NUM!	1.0E-05
YJL021C	YJL021C	UWOPS87_ctrl_hmg124h_7.JPG.dat	1228.0	100.0	1.0	0.01	#NUM!	1.2E-05
YJL022W	YJL022W	UWOPS87_ctrl_hmg124h_7.JPG.dat	1115.3	87.7	1.0	0.03	#NUM!	2.3E-04
YJL023C	YJL023C	UWOPS87_ctrl_hmg124h_7.JPG.dat	1194.0	61.6	1.1	0.02	#NUM!	9.2E-05
YJL024C	YJL024C	UWOPS87_ctrl_hmg124h_7.JPG.dat	1351.0	44.0	1.1	0.04	#NUM!	1.5E-05
YJL027C	YJL027C	UWOPS87_ctrl_hmg124h_7.JPG.dat	1281.8	45.4	1.1	0.03	#NUM!	8.2E-06
YJL028W	YJL028W	UWOPS87_ctrl_hmg124h_7.JPG.dat	1079.5	83.3	1.0	0.02	#NUM!	8.7E-05
YJL030W	YJL030W	UWOPS87_ctrl_hmg124h_7.JPG.dat	904.0	129.9	0.9	0.12	#NUM!	7.2E-04
YJL036W	YJL036W	UWOPS87_ctrl_hmg124h_7.JPG.dat	1303.3	203.7	1.2	0.17	#NUM!	9.0E-04
YJL037W	YJL037W	UWOPS87_ctrl_hmg124h_7.JPG.dat	982.3	59.7	0.9	0.05	#NUM!	5.8E-05
YJL038C	YJL038C	UWOPS87_ctrl_hmg124h_7.JPG.dat	1100.3	46.2	1.0	0.04	#NUM!	1.9E-05
YJL042W	YJL042W	UWOPS87_ctrl_hmg124h_7.JPG.dat	1050.5	182.3	1.0	0.17	#NUM!	1.4E-03
YJL043W	YJL043W	UWOPS87_ctrl_hmg124h_7.JPG.dat	1064.8	96.7	1.0	0.08	#NUM!	1.8E-04
YJL044C	YJL044C	UWOPS87_ctrl_hmg124h_7.JPG.dat	1149.5	33.7	1.0	0.03	#NUM!	9.5E-06
YJL045W	YJL045W	UWOPS87_ctrl_hmg124h_7.JPG.dat	1049.8	112.5	1.0	0.10	#NUM!	2.9E-04
YJL046W	YJL046W	UWOPS87_ctrl_hmg124h_7.JPG.dat	0.0	0.0	0.0	0.00	#NUM!	
YJL047C	YJL047C	UWOPS87_ctrl_hmg124h_7.JPG.dat	1288.0	81.8	1.1	0.08	#NUM!	9.6E-05
YJL048C	YJL048C	UWOPS87_ctrl_hmg124h_7.JPG.dat	1005.5	119.6	0.9	0.11	#NUM!	4.7E-04
YJL049W	YJL049W	UWOPS87_ctrl_hmg124h_7.JPG.dat	933.0	77.3	0.9	0.08	#NUM!	1.6E-04
YJL051W	YJL051W	UWOPS87_ctrl_hmg124h_7.JPG.dat	1220.5	30.3	1.1	0.04	#NUM!	1.0E-05
YJL053W	YJL053W	UWOPS87_ctrl_hmg124h_7.JPG.dat	979.5	49.2	0.9	0.04	#NUM!	2.5E-05
YJL055W	YJL055W	UWOPS87_ctrl_hmg124h_7.JPG.dat	1000.0	25.1	0.9	0.01	#NUM!	1.7E-05
YJL057C	YJL057C	UWOPS87_ctrl_hmg124h_7.JPG.dat	1195.0	49.8	1.1	0.03	#NUM!	7.4E-06
YJL058C	YJL058C	UWOPS87_ctrl_hmg124h_7.JPG.dat	1137.0	56.1	1.0	0.06	#NUM!	5.5E-05
YJL059W	YJL059W	UWOPS87_ctrl_hmg124h_7.JPG.dat	1089.0	65.1	1.0	0.05	#NUM!	3.4E-05
YJL060W	YJL060W	UWOPS87_ctrl_hmg124h_7.JPG.dat	1187.5	70.9	1.1	0.06	#NUM!	3.6E-05
YJL062W	YJL062W	UWOPS87_ctrl_hmg124h_7.JPG.dat	824.0	271.7	0.9	0.08	#NUM!	3.1E-03
YJL064W	YJL064W	UWOPS87_ctrl_hmg124h_7.JPG.dat	671.5	449.4	0.8	0.05	#NUM!	1.2E-03
YJL065C	YJL065C	UWOPS87_ctrl_hmg124h_7.JPG.dat	1222.0	133.2	1.1	0.11	#NUM!	2.6E-04
YJL066C	YJL066C	UWOPS87_ctrl_hmg124h_7.JPG.dat	1069.3	66.3	0.9	0.01	#NUM!	8.0E-05
YJL067W	YJL067W	UWOPS87_ctrl_hmg124h_7.JPG.dat	1283.3	161.1	1.1	0.17	#NUM!	9.6E-04
YJL068C	YJL068C	UWOPS87_ctrl_hmg124h_7.JPG.dat	971.3	76.6	0.8	0.06	#NUM!	9.0E-05
YJL070C	YJL070C	UWOPS87_ctrl_hmg124h_7.JPG.dat	1255.5	123.1	1.0	0.04	#NUM!	5.7E-04
YJL071W	YJL071W	UWOPS87_ctrl_hmg124h_7.JPG.dat	0.0	0.0	0.0	0.00	#NUM!	

YJL073W	YJL073W	UWOPS87_ctrl_hmg124h_7.JPG.dat	1427.3	93.4	1.3	0.03	#NUM!	1.4E-04
YJL077C	YJL077C	UWOPS87_ctrl_hmg124h_7.JPG.dat	1204.5	161.0	1.1	0.12	#NUM!	4.1E-04
YJL078C	YJL078C	UWOPS87_ctrl_hmg124h_7.JPG.dat	1062.3	89.6	1.0	0.08	#NUM!	1.5E-04
YJL083W	YJL083W	UWOPS87_ctrl_hmg124h_7.JPG.dat	1011.5	154.7	0.9	0.12	#NUM!	6.0E-04
YJL084C	YJL084C	UWOPS87_ctrl_hmg124h_7.JPG.dat	840.5	34.0	0.8	0.04	#NUM!	3.4E-05
YJL088W	YJL088W	UWOPS87_ctrl_hmg124h_7.JPG.dat	1243.5	70.7	1.1	0.02	#NUM!	1.5E-04
YJL089W	YJL089W	UWOPS87_ctrl_hmg124h_7.JPG.dat	936.8	35.5	0.9	0.02	#NUM!	3.9E-06
YJL093C	YJL093C	UWOPS87_ctrl_hmg124h_7.JPG.dat	1208.0	51.3	1.2	0.07	#NUM!	5.2E-05
YJL094C	YJL094C	UWOPS87_ctrl_hmg124h_7.JPG.dat	1105.0	78.8	1.0	0.03	#NUM!	2.2E-04
YJL095W	YJL095W	UWOPS87_ctrl_hmg124h_7.JPG.dat	1247.5	404.4	1.1	0.38	#NUM!	8.8E-03
YJL098W	YJL098W	UWOPS87_ctrl_hmg124h_7.JPG.dat	1233.3	62.5	1.1	0.05	#NUM!	2.0E-05
YJL099W	YJL099W	UWOPS87_ctrl_hmg124h_7.JPG.dat	1019.5	122.9	0.9	0.12	#NUM!	5.8E-04
YJL100W	YJL100W	UWOPS87_ctrl_hmg124h_7.JPG.dat	1024.8	118.9	0.9	0.01	#NUM!	5.5E-05
YJL101C	YJL101C	UWOPS87_ctrl_hmg124h_7.JPG.dat	938.3	58.8	0.9	0.07	#NUM!	1.2E-04
YJL105W	YJL105W	UWOPS87_ctrl_hmg124h_7.JPG.dat	1098.0	163.3	0.9	0.02	#NUM!	1.5E-04
YJL108C	YJL108C	UWOPS87_ctrl_hmg124h_7.JPG.dat	1135.0	98.1	1.1	0.10	#NUM!	2.0E-04
YJL110C	YJL110C	UWOPS87_ctrl_hmg124h_7.JPG.dat	1056.8	53.5	1.0	0.06	#NUM!	5.1E-05
YJL112W	YJL112W	UWOPS87_ctrl_hmg124h_7.JPG.dat	1080.3	35.3	1.1	0.04	#NUM!	1.3E-05
YJL115W	YJL115W	UWOPS87_ctrl_hmg124h_7.JPG.dat	984.5	103.8	0.9	0.10	#NUM!	3.2E-04
YJL116C	YJL116C	UWOPS87_ctrl_hmg124h_7.JPG.dat	1267.8	111.3	1.2	0.10	#NUM!	1.7E-04
YJL121C	YJL121C	UWOPS87_ctrl_hmg124h_7.JPG.dat	0.0	0.0	0.0	0.00	#NUM!	
YJL122W	YJL122W	UWOPS87_ctrl_hmg124h_7.JPG.dat	1139.0	101.6	1.0	0.10	#NUM!	2.3E-04
YJL123C	YJL123C	UWOPS87_ctrl_hmg124h_7.JPG.dat	1163.3	89.8	1.1	0.09	#NUM!	1.6E-04
YJL124C	YJL124C	UWOPS87_ctrl_hmg124h_7.JPG.dat	830.3	24.4	0.8	0.03	#NUM!	1.2E-05
YJL126W	YJL126W	UWOPS87_ctrl_hmg124h_7.JPG.dat	1293.0	27.4	1.2	0.02	#NUM!	1.5E-06
YJL128C	YJL128C	UWOPS87_ctrl_hmg124h_7.JPG.dat	1081.8	171.0	0.9	0.03	#NUM!	4.7E-04
YJL130C	YJL130C	UWOPS87_ctrl_hmg124h_7.JPG.dat	0.0	0.0	0.0	0.00	#NUM!	
YJL131C	YJL131C	UWOPS87_ctrl_hmg124h_7.JPG.dat	994.3	37.7	0.9	0.04	#NUM!	1.5E-05
YJL132W	YJL132W	UWOPS87_ctrl_hmg124h_7.JPG.dat	930.0	88.3	1.0	0.08	#NUM!	1.4E-04
YJL133W	YJL133W	UWOPS87_ctrl_hmg124h_7.JPG.dat	1032.5	92.7	1.0	0.08	#NUM!	1.9E-04
YJL134W	YJL134W	UWOPS87_ctrl_hmg124h_7.JPG.dat	1202.5	30.9	1.1	0.03	#NUM!	6.4E-06
YJL135W	YJL135W	UWOPS87_ctrl_hmg124h_7.JPG.dat	1116.3	68.5	1.0	0.07	#NUM!	1.0E-04
YJL136C	YJL136C	UWOPS87_ctrl_hmg124h_7.JPG.dat	873.5	144.2	0.9	0.04	#NUM!	5.7E-04
YJL137C	YJL137C	UWOPS87_ctrl_hmg124h_7.JPG.dat	1086.0	13.6	1.0	0.02	#NUM!	1.1E-06
YJL138C	YJL138C	UWOPS87_ctrl_hmg124h_7.JPG.dat	924.8	113.5	0.9	0.11	#NUM!	4.7E-04
YJL139C	YJL139C	UWOPS87_ctrl_hmg124h_7.JPG.dat	1237.8	107.4	1.1	0.09	#NUM!	1.5E-04
YJL141C	YJL141C	UWOPS87_ctrl_hmg124h_7.JPG.dat	1374.8	92.2	1.2	0.08	#NUM!	9.9E-05
YJL142C	YJL142C	UWOPS87_ctrl_hmg124h_7.JPG.dat	1325.0	55.8	1.2	0.05	#NUM!	2.7E-05
YJL144W	YJL144W	UWOPS87_ctrl_hmg124h_7.JPG.dat	1216.0	140.3	1.1	0.02	#NUM!	8.0E-05
YJL145W	YJL145W	UWOPS87_ctrl_hmg124h_7.JPG.dat	1099.0	131.4	1.1	0.12	#NUM!	3.6E-04
YJL146W	YJL146W	UWOPS87_ctrl_hmg124h_7.JPG.dat	1175.8	24.2	1.0	0.01	#NUM!	1.8E-05
YJL147C	YJL147C	UWOPS87_ctrl_hmg124h_7.JPG.dat	1032.0	51.1	0.9	0.05	#NUM!	4.3E-05
YJL148W	YJL148W	UWOPS87_ctrl_hmg124h_7.JPG.dat	1073.3	51.0	1.0	0.06	#NUM!	5.9E-05
YJL149W	YJL149W	UWOPS87_ctrl_hmg124h_7.JPG.dat	1103.5	53.4	1.0	0.04	#NUM!	1.4E-05
YJL150W	YJL150W	UWOPS87_ctrl_hmg124h_7.JPG.dat	1217.3	39.9	1.1	0.04	#NUM!	1.5E-05
YJL151C	YJL151C	UWOPS87_ctrl_hmg124h_7.JPG.dat	1141.0	34.2	1.0	0.03	#NUM!	4.3E-06
YJL152W	YJL152W	UWOPS87_ctrl_hmg124h_7.JPG.dat	1105.8	38.9	0.9	0.04	#NUM!	1.8E-05
YJL153C	YJL153C	UWOPS87_ctrl_hmg124h_7.JPG.dat	1268.5	81.5	1.0	0.07	#NUM!	9.3E-05
YJL154C	YJL154C	UWOPS87_ctrl_hmg124h_7.JPG.dat	1213.3	38.4	1.0	0.02	#NUM!	3.0E-06
YJL155C	YJL155C	UWOPS87_ctrl_hmg124h_7.JPG.dat	1044.5	111.4	1.0	0.11	#NUM!	3.8E-04
YJL157C	YJL157C	UWOPS87_ctrl_hmg124h_7.JPG.dat	1284.0	29.4	1.2	0.02	#NUM!	2.1E-06
YJL158C	YJL158C	UWOPS87_ctrl_hmg124h_7.JPG.dat	1245.0	19.6	1.1	0.02	#NUM!	3.0E-06
YJL159W	YJL159W	UWOPS87_ctrl_hmg124h_7.JPG.dat	1096.5	15.9	1.0	0.01	#NUM!	7.1E-07
YJL160C	YJL160C	UWOPS87_ctrl_hmg124h_7.JPG.dat	958.5	40.9	0.9	0.03	#NUM!	1.7E-05
YJL161W	YJL161W	UWOPS87_ctrl_hmg124h_7.JPG.dat	916.8	35.0	0.8	0.03	#NUM!	1.5E-05
YJL162C	YJL162C	UWOPS87_ctrl_hmg124h_7.JPG.dat	1084.3	43.5	1.0	0.04	#NUM!	1.5E-05
YJL163C	YJL163C	UWOPS87_ctrl_hmg124h_7.JPG.dat	1075.0	39.1	1.0	0.04	#NUM!	1.7E-05
YJL164C	YJL164C	UWOPS87_ctrl_hmg124h_7.JPG.dat	1195.3	54.2	1.1	0.01	#NUM!	3.3E-05
YJL165C	YJL165C	UWOPS87_ctrl_hmg124h_7.JPG.dat	1089.0	19.6	0.9	0.00	#NUM!	8.8E-06
YJL168C	YJL168C	UWOPS87_ctrl_hmg124h_7.JPG.dat	1235.8	67.8	1.1	0.06	#NUM!	4.1E-05
YJL169W	YJL169W	UWOPS87_ctrl_hmg124h_7.JPG.dat	1061.0	38.4	0.9	0.01	#NUM!	1.7E-05
YJL170C	YJL170C	UWOPS87_ctrl_hmg124h_7.JPG.dat	1242.0	104.5	1.2	0.08	#NUM!	8.7E-05
YJL171C	YJL171C	UWOPS87_ctrl_hmg124h_7.JPG.dat	1353.0	64.6	1.2	0.06	#NUM!	2.8E-05
YJL172W	YJL172W	UWOPS87_ctrl_hmg124h_7.JPG.dat	1150.5	82.3	1.1	0.02	#NUM!	1.0E-04
YJL175W	YJL175W	UWOPS87_ctrl_hmg124h_7.JPG.dat	1126.8	26.3	1.0	0.03	#NUM!	5.3E-06
YJL176C	YJL176C	UWOPS87_ctrl_hmg124h_7.JPG.dat	1097.3	26.3	1.0	0.03	#NUM!	7.1E-06
YJL178C	YJL178C	UWOPS87_ctrl_hmg124h_7.JPG.dat	1087.3	121.8	1.0	0.04	#NUM!	6.8E-04
YJL181W	YJL181W	UWOPS87_ctrl_hmg124h_7.JPG.dat	1104.3	215.6	0.9	0.05	#NUM!	9.6E-04
YJL182C	YJL182C	UWOPS87_ctrl_hmg124h_7.JPG.dat	1034.8	30.1	1.0	0.03	#NUM!	7.1E-06
YJL183W	YJL183W	UWOPS87_ctrl_hmg124h_7.JPG.dat	1075.5	76.8	1.0	0.06	#NUM!	8.0E-05

YJL185C	YJL185C	UWOPS87_ctrl_hmg124h_7.JPG.dat	977.8	82.0	0.9	0.07	#NUM!	1.6E-04
YJL186W	YJL186W	UWOPS87_ctrl_hmg124h_7.JPG.dat	1137.3	170.5	0.9	0.03	#NUM!	2.5E-04
YJL187C	YJL187C	UWOPS87_ctrl_hmg124h_7.JPG.dat	1300.5	109.5	1.3	0.02	#NUM!	7.4E-05
YJL188C	YJL188C	UWOPS87_ctrl_hmg124h_7.JPG.dat	1236.0	67.0	1.0	0.05	#NUM!	3.6E-05
YJL190C	YJL190C	UWOPS87_ctrl_hmg124h_7.JPG.dat	1242.3	27.0	1.1	0.02	#NUM!	2.2E-06
YJL191W	YJL191W	UWOPS87_ctrl_hmg124h_7.JPG.dat	1256.8	49.7	1.1	0.05	#NUM!	2.2E-05
YJL192C	YJL192C	UWOPS87_ctrl_hmg124h_7.JPG.dat	1138.3	27.5	1.0	0.03	#NUM!	7.8E-06
YJL193W	YJL193W	UWOPS87_ctrl_hmg124h_7.JPG.dat	927.0	149.0	0.9	0.03	#NUM!	3.3E-04
YJL196C	YJL196C	UWOPS87_ctrl_hmg124h_7.JPG.dat	1034.3	19.3	1.0	0.01	#NUM!	9.0E-07
YJL197W	YJL197W	UWOPS87_ctrl_hmg124h_7.JPG.dat	906.8	73.5	0.8	0.07	#NUM!	1.6E-04
YJL198W	YJL198W	UWOPS87_ctrl_hmg124h_7.JPG.dat	1021.5	48.1	0.9	0.04	#NUM!	3.1E-05
YJL199C	YJL199C	UWOPS87_ctrl_hmg124h_7.JPG.dat	1256.5	27.4	1.1	0.02	#NUM!	1.4E-06
YJL201W	YJL201W	UWOPS87_ctrl_hmg124h_7.JPG.dat	1234.8	42.8	1.1	0.04	#NUM!	9.7E-06
YJL204C	YJL204C	UWOPS87_ctrl_hmg124h_7.JPG.dat	917.0	73.9	0.9	0.06	#NUM!	7.4E-05
YJL206C	YJL206C	UWOPS87_ctrl_hmg124h_7.JPG.dat	1016.8	21.1	1.0	0.02	#NUM!	4.2E-06
YJL206C-A	YJL206C-A	UWOPS87_ctrl_hmg124h_7.JPG.dat	993.3	38.2	1.0	0.03	#NUM!	4.9E-06
YJL207C	YJL207C	UWOPS87_ctrl_hmg124h_7.JPG.dat	1160.8	84.6	1.1	0.02	#NUM!	1.5E-04
YJL208C	YJL208C	UWOPS87_ctrl_hmg124h_7.JPG.dat	1017.8	148.1	0.9	0.12	#NUM!	6.2E-04
YJL210W	YJL210W	UWOPS87_ctrl_hmg124h_7.JPG.dat	1117.5	15.9	1.0	0.01	#NUM!	7.7E-07
YJL211C	YJL211C	UWOPS87_ctrl_hmg124h_7.JPG.dat	1302.5	65.9	1.2	0.07	#NUM!	5.6E-05
YJL212C	YJL212C	UWOPS87_ctrl_hmg124h_7.JPG.dat	928.8	51.7	0.9	0.05	#NUM!	5.7E-05
YJL213W	YJL213W	UWOPS87_ctrl_hmg124h_7.JPG.dat	1152.8	116.8	1.0	0.11	#NUM!	3.7E-04
YJL214W	YJL214W	UWOPS87_ctrl_hmg124h_7.JPG.dat	1152.8	67.2	1.1	0.06	#NUM!	5.5E-05
YJL215C	YJL215C	UWOPS87_ctrl_hmg124h_7.JPG.dat	1004.5	119.0	1.0	0.13	#NUM!	6.2E-04
YJL216C	YJL216C	UWOPS87_ctrl_hmg124h_7.JPG.dat	959.0	76.9	1.0	0.07	#NUM!	1.1E-04
YJL217W	YJL217W	UWOPS87_ctrl_hmg124h_7.JPG.dat	908.0	52.8	0.9	0.05	#NUM!	3.9E-05
YJL218W	YJL218W	UWOPS87_ctrl_hmg124h_7.JPG.dat	944.0	75.3	0.9	0.07	#NUM!	1.2E-04
YJR001W	YJR001W	UWOPS87_ctrl_hmg124h_7.JPG.dat	1338.8	66.8	1.2	0.06	#NUM!	2.9E-05
YJR003C	YJR003C	UWOPS87_ctrl_hmg124h_7.JPG.dat	1021.5	71.3	1.0	0.02	#NUM!	9.6E-05
YJR005W	YJR005W	UWOPS87_ctrl_hmg124h_7.JPG.dat	1295.0	71.1	1.2	0.02	#NUM!	1.2E-04
YJR008W	YJR008W	UWOPS87_ctrl_hmg124h_7.JPG.dat	949.3	103.6	0.9	0.10	#NUM!	3.5E-04
YJR009C	YJR009C	UWOPS87_ctrl_hmg124h_7.JPG.dat	1004.3	25.8	1.0	0.00	#NUM!	6.9E-07
YJR010C-A	YJR010C-A	UWOPS87_ctrl_hmg124h_7.JPG.dat	1230.3	87.0	1.1	0.07	#NUM!	7.5E-05
YJR010W	YJR010W	UWOPS87_ctrl_hmg124h_7.JPG.dat	1114.0	111.0	1.0	0.10	#NUM!	2.4E-04
YJR011C	YJR011C	UWOPS87_ctrl_hmg124h_7.JPG.dat	1012.5	56.5	1.0	0.06	#NUM!	5.4E-05
YJR014W	YJR014W	UWOPS87_ctrl_hmg124h_7.JPG.dat	858.3	41.3	0.9	0.01	#NUM!	4.0E-05
YJR015W	YJR015W	UWOPS87_ctrl_hmg124h_7.JPG.dat	871.3	30.2	0.8	0.03	#NUM!	1.4E-05
YJR019C	YJR019C	UWOPS87_ctrl_hmg124h_7.JPG.dat	1205.5	40.5	1.1	0.03	#NUM!	8.7E-06
YJR020W	YJR020W	UWOPS87_ctrl_hmg124h_7.JPG.dat	1226.3	90.9	1.1	0.08	#NUM!	1.2E-04
YJR021C	YJR021C	UWOPS87_ctrl_hmg124h_7.JPG.dat	1033.3	34.4	1.0	0.03	#NUM!	1.1E-05
YJR024C	YJR024C	UWOPS87_ctrl_hmg124h_7.JPG.dat	1188.5	33.1	1.1	0.02	#NUM!	2.7E-06
YJR025C	YJR025C	UWOPS87_ctrl_hmg124h_7.JPG.dat	955.0	40.8	0.9	0.01	#NUM!	8.2E-05
YJR026W	YJR026W	UWOPS87_ctrl_hmg124h_7.JPG.dat	1017.0	78.3	1.0	0.01	#NUM!	2.2E-05
YJR030C	YJR030C	UWOPS87_ctrl_hmg124h_7.JPG.dat	1038.5	52.7	0.9	0.04	#NUM!	3.2E-05
YJR031C	YJR031C	UWOPS87_ctrl_hmg124h_7.JPG.dat	1208.3	120.7	1.0	0.10	#NUM!	2.7E-04
YJR032W	YJR032W	UWOPS87_ctrl_hmg124h_7.JPG.dat	0.0	0.0	0.0	0.00	#NUM!	
YJR033C	YJR033C	UWOPS87_ctrl_hmg124h_7.JPG.dat	1020.8	32.5	1.0	0.01	#NUM!	2.6E-05
YJR035W	YJR035W	UWOPS87_ctrl_hmg124h_7.JPG.dat	1195.8	100.3	1.1	0.07	#NUM!	8.4E-05
YJR036C	YJR036C	UWOPS87_ctrl_hmg124h_7.JPG.dat	1197.5	57.1	1.1	0.06	#NUM!	5.1E-05
YJR037W	YJR037W	UWOPS87_ctrl_hmg124h_7.JPG.dat	1105.0	45.8	1.0	0.04	#NUM!	1.6E-05
YJR038C	YJR038C	UWOPS87_ctrl_hmg124h_7.JPG.dat	1049.5	42.4	1.0	0.01	#NUM!	2.6E-05
YJR040W	YJR040W	UWOPS87_ctrl_hmg124h_7.JPG.dat	1128.8	37.0	1.0	0.03	#NUM!	7.8E-06
YJR043C	YJR043C	UWOPS87_ctrl_hmg124h_7.JPG.dat	1143.8	72.5	1.0	0.06	#NUM!	5.5E-05
YJR047C	YJR047C	UWOPS87_ctrl_hmg124h_7.JPG.dat	1244.5	16.7	1.1	0.03	#NUM!	3.6E-06
YJR048W	YJR048W	UWOPS87_ctrl_hmg124h_7.JPG.dat	1274.5	30.2	1.0	0.01	#NUM!	8.0E-07
YJR049C	YJR049C	UWOPS87_ctrl_hmg124h_7.JPG.dat	1049.8	71.0	0.9	0.06	#NUM!	1.1E-04
YJR050W	YJR050W	UWOPS87_ctrl_hmg124h_7.JPG.dat	798.5	25.7	0.7	0.03	#NUM!	2.7E-05
YJR051W	YJR051W	UWOPS87_ctrl_hmg124h_7.JPG.dat	1222.8	87.6	1.2	0.09	#NUM!	1.1E-04
YJR052W	YJR052W	UWOPS87_ctrl_hmg124h_7.JPG.dat	1299.3	46.7	1.1	0.05	#NUM!	2.4E-05
YJR053W	YJR053W	UWOPS87_ctrl_hmg124h_7.JPG.dat	1118.0	65.2	1.0	0.06	#NUM!	5.7E-05
YJR054W	YJR054W	UWOPS87_ctrl_hmg124h_7.JPG.dat	1098.5	41.8	1.0	0.04	#NUM!	1.7E-05
YJR058C	YJR058C	UWOPS87_ctrl_hmg124h_7.JPG.dat	1139.0	45.8	1.0	0.04	#NUM!	1.8E-05
YJR059W	YJR059W	UWOPS87_ctrl_hmg124h_7.JPG.dat	1171.0	50.4	1.0	0.05	#NUM!	2.5E-05
YJR060W	YJR060W	UWOPS87_ctrl_hmg124h_7.JPG.dat	1193.3	13.1	1.0	0.01	#NUM!	8.0E-07
YJR061W	YJR061W	UWOPS87_ctrl_hmg124h_7.JPG.dat	1109.3	97.8	1.0	0.08	#NUM!	1.7E-04
YJR062C	YJR062C	UWOPS87_ctrl_hmg124h_7.JPG.dat	1100.3	136.9	1.0	0.03	#NUM!	4.1E-04
YJR069C	YJR069C	UWOPS87_ctrl_hmg124h_7.JPG.dat	1184.3	184.1	1.0	0.16	#NUM!	9.9E-04
YJR070C	YJR070C	UWOPS87_ctrl_hmg124h_7.JPG.dat	1248.8	35.6	1.1	0.04	#NUM!	1.1E-05
YJR073C	YJR073C	UWOPS87_ctrl_hmg124h_7.JPG.dat	1144.8	35.4	1.1	0.03	#NUM!	6.0E-06
YJR074W	YJR074W	UWOPS87_ctrl_hmg124h_7.JPG.dat	1025.3	59.2	0.9	0.04	#NUM!	2.1E-05

YJR075W	YJR075W	UWOPS87_ctrl_hmg124h_7.JPG.dat	1172.5	152.0	1.1	0.14	#NUM!	5.7E-04
YJR077C	YJR077C	UWOPS87_ctrl_hmg124h_7.JPG.dat	0.0	0.0	0.0	0.00	#NUM!	
YJR078W	YJR078W	UWOPS87_ctrl_hmg124h_7.JPG.dat	844.3	72.5	0.8	0.07	#NUM!	1.8E-04
YJR079W	YJR079W	UWOPS87_ctrl_hmg124h_7.JPG.dat	1088.5	41.1	1.0	0.04	#NUM!	1.6E-05
YJR080C	YJR080C	UWOPS87_ctrl_hmg124h_7.JPG.dat	0.0	0.0	0.0	0.00	#NUM!	
YJR082C	YJR082C	UWOPS87_ctrl_hmg124h_7.JPG.dat	1105.5	45.1	1.1	0.02	#NUM!	8.6E-05
YJR083C	YJR083C	UWOPS87_ctrl_hmg124h_7.JPG.dat	1164.3	43.4	1.1	0.04	#NUM!	1.0E-05
YJR084W	YJR084W	UWOPS87_ctrl_hmg124h_7.JPG.dat	962.5	15.4	0.9	0.02	#NUM!	1.8E-06
YJR087W	YJR087W	UWOPS87_ctrl_hmg124h_7.JPG.dat	1539.8	111.1	1.4	0.10	#NUM!	1.1E-04
YJR088C	YJR088C	UWOPS87_ctrl_hmg124h_7.JPG.dat	1126.8	153.4	1.1	0.16	#NUM!	8.9E-04
YJR090C	YJR090C	UWOPS87_ctrl_hmg124h_7.JPG.dat	337.8	46.8	0.3	0.04	#NUM!	6.9E-04
YJR091C	YJR091C	UWOPS87_ctrl_hmg124h_7.JPG.dat	1177.0	59.9	1.0	0.05	#NUM!	3.3E-05
YJR092W	YJR092W	UWOPS87_ctrl_hmg124h_7.JPG.dat	1141.3	56.9	1.0	0.02	#NUM!	1.6E-04
YJR094C	YJR094C	UWOPS87_ctrl_hmg124h_7.JPG.dat	1170.5	78.0	1.0	0.06	#NUM!	6.5E-05
YJR094W-A	YJR094W-A	UWOPS87_ctrl_hmg124h_7.JPG.dat	1046.8	33.5	0.9	0.03	#NUM!	6.9E-06
YJR095W	YJR095W	UWOPS87_ctrl_hmg124h_7.JPG.dat	1216.0	118.7	1.1	0.11	#NUM!	2.9E-04
YJR096W	YJR096W	UWOPS87_ctrl_hmg124h_7.JPG.dat	1030.8	57.1	0.9	0.02	#NUM!	1.5E-04
YJR097W	YJR097W	UWOPS87_ctrl_hmg124h_7.JPG.dat	1291.3	39.6	1.2	0.03	#NUM!	6.4E-06
YJR098C	YJR098C	UWOPS87_ctrl_hmg124h_7.JPG.dat	1478.8	174.0	1.4	0.03	#NUM!	1.5E-04
YJR099W	YJR099W	UWOPS87_ctrl_hmg124h_7.JPG.dat	1003.8	82.4	0.8	0.02	#NUM!	1.1E-04
YOR202W	YOR202W	UWOPS87_ctrl_hmg124h_7.JPG.dat	1297.3	221.0	1.0	0.12	#NUM!	2.7E-256
1	1	UWOPS87_ctrl_hmg124h_8.JPG.dat	1899.8	273.5	#NUM!		#NUM!	
2	2	UWOPS87_ctrl_hmg124h_8.JPG.dat	1374.0	231.1	1.1	0.16	#NUM!	7.9E-04
3	3	UWOPS87_ctrl_hmg124h_8.JPG.dat	1318.5	160.9	1.0	0.09	#NUM!	2.1E-04
4	4	UWOPS87_ctrl_hmg124h_8.JPG.dat	1261.3	116.8	1.0	0.05	#NUM!	4.2E-05
YJR100C	YJR100C	UWOPS87_ctrl_hmg124h_8.JPG.dat	1058.5	50.1	1.0	0.04	#NUM!	1.5E-05
YJR103W	YJR103W	UWOPS87_ctrl_hmg124h_8.JPG.dat	306.0	612.0	0.0	0.00	#NUM!	
YJR106W	YJR106W	UWOPS87_ctrl_hmg124h_8.JPG.dat	1220.8	74.7	1.0	0.07	#NUM!	1.1E-04
YJR107W	YJR107W	UWOPS87_ctrl_hmg124h_8.JPG.dat	1243.8	30.8	1.0	0.02	#NUM!	2.8E-06
YJR108W	YJR108W	UWOPS87_ctrl_hmg124h_8.JPG.dat	1364.0	25.5	1.1	0.02	#NUM!	1.6E-06
YJR109C	YJR109C	UWOPS87_ctrl_hmg124h_8.JPG.dat	1282.0	53.9	1.0	0.04	#NUM!	1.7E-05
YJR110W	YJR110W	UWOPS87_ctrl_hmg124h_8.JPG.dat	1220.0	45.1	1.0	0.04	#NUM!	1.5E-05
YJR111C	YJR111C	UWOPS87_ctrl_hmg124h_8.JPG.dat	1173.0	47.5	1.0	0.03	#NUM!	8.4E-06
YJR115W	YJR115W	UWOPS87_ctrl_hmg124h_8.JPG.dat	1267.3	58.2	1.0	0.04	#NUM!	1.5E-05
YJR116W	YJR116W	UWOPS87_ctrl_hmg124h_8.JPG.dat	1248.5	44.4	1.0	0.03	#NUM!	7.4E-06
YJR117W	YJR117W	UWOPS87_ctrl_hmg124h_8.JPG.dat	1325.8	136.1	1.1	0.11	#NUM!	2.7E-04
YJR118C	YJR118C	UWOPS87_ctrl_hmg124h_8.JPG.dat	857.5	104.3	0.8	0.10	#NUM!	4.7E-04
YJR119C	YJR119C	UWOPS87_ctrl_hmg124h_8.JPG.dat	1189.3	83.0	1.0	0.02	#NUM!	1.3E-04
YJR120W	YJR120W	UWOPS87_ctrl_hmg124h_8.JPG.dat	0.0	0.0	0.0	0.00	#NUM!	
YJR121W	YJR121W	UWOPS87_ctrl_hmg124h_8.JPG.dat	1212.3	44.7	1.1	0.01	#NUM!	3.4E-05
YJR124C	YJR124C	UWOPS87_ctrl_hmg124h_8.JPG.dat	1144.8	34.5	1.0	0.02	#NUM!	3.1E-06
YJR125C	YJR125C	UWOPS87_ctrl_hmg124h_8.JPG.dat	1251.3	34.5	1.0	0.03	#NUM!	9.6E-06
YJR126C	YJR126C	UWOPS87_ctrl_hmg124h_8.JPG.dat	1062.0	77.7	0.9	0.06	#NUM!	1.0E-04
YJR127C	YJR127C	UWOPS87_ctrl_hmg124h_8.JPG.dat	1082.0	66.3	0.9	0.06	#NUM!	7.9E-05
YJR128W	YJR128W	UWOPS87_ctrl_hmg124h_8.JPG.dat	1296.8	51.5	1.1	0.05	#NUM!	2.1E-05
YJR129C	YJR129C	UWOPS87_ctrl_hmg124h_8.JPG.dat	1253.8	75.9	1.1	0.01	#NUM!	5.7E-05
YJR130C	YJR130C	UWOPS87_ctrl_hmg124h_8.JPG.dat	1185.5	86.9	1.0	0.07	#NUM!	1.0E-04
YJR131W	YJR131W	UWOPS87_ctrl_hmg124h_8.JPG.dat	1171.0	144.7	1.1	0.15	#NUM!	6.2E-04
YJR133W	YJR133W	UWOPS87_ctrl_hmg124h_8.JPG.dat	1313.3	66.7	1.1	0.05	#NUM!	2.6E-05
YJR134C	YJR134C	UWOPS87_ctrl_hmg124h_8.JPG.dat	1194.8	74.9	1.0	0.06	#NUM!	6.9E-05
YJR135C	YJR135C	UWOPS87_ctrl_hmg124h_8.JPG.dat	1131.3	57.0	1.0	0.05	#NUM!	3.5E-05
YJR137C	YJR137C	UWOPS87_ctrl_hmg124h_8.JPG.dat	1142.0	54.5	1.0	0.05	#NUM!	2.8E-05
YJR139C	YJR139C	UWOPS87_ctrl_hmg124h_8.JPG.dat	935.3	326.5	0.8	0.27	#NUM!	1.1E-02
YJR140C	YJR140C	UWOPS87_ctrl_hmg124h_8.JPG.dat	809.0	539.6	0.9	0.02	#NUM!	1.8E-04
YJR142W	YJR142W	UWOPS87_ctrl_hmg124h_8.JPG.dat	1196.5	25.2	1.0	0.02	#NUM!	2.6E-06
YJR145C	YJR145C	UWOPS87_ctrl_hmg124h_8.JPG.dat	1075.0	61.0	0.9	0.05	#NUM!	4.4E-05
YJR146W	YJR146W	UWOPS87_ctrl_hmg124h_8.JPG.dat	1247.8	38.0	1.0	0.03	#NUM!	8.0E-06
YJR147W	YJR147W	UWOPS87_ctrl_hmg124h_8.JPG.dat	1218.3	28.7	1.0	0.02	#NUM!	3.8E-06
YJR148W	YJR148W	UWOPS87_ctrl_hmg124h_8.JPG.dat	1108.8	32.5	1.0	0.02	#NUM!	3.2E-06
YJR149W	YJR149W	UWOPS87_ctrl_hmg124h_8.JPG.dat	1260.3	132.4	1.0	0.10	#NUM!	2.8E-04
YJR150C	YJR150C	UWOPS87_ctrl_hmg124h_8.JPG.dat	1289.5	24.2	1.1	0.02	#NUM!	3.2E-06
YJR152W	YJR152W	UWOPS87_ctrl_hmg124h_8.JPG.dat	1112.0	41.6	0.9	0.04	#NUM!	1.8E-05
YJR153W	YJR153W	UWOPS87_ctrl_hmg124h_8.JPG.dat	1258.0	51.2	1.1	0.05	#NUM!	2.7E-05
YJR154W	YJR154W	UWOPS87_ctrl_hmg124h_8.JPG.dat	313.5	627.0	0.0	0.00	#NUM!	
YKL001C	YKL001C	UWOPS87_ctrl_hmg124h_8.JPG.dat	0.0	0.0	0.0	0.00	#NUM!	
YKL005C	YKL005C	UWOPS87_ctrl_hmg124h_8.JPG.dat	1245.0	61.4	1.1	0.05	#NUM!	3.4E-05
YKL006W	YKL006W	UWOPS87_ctrl_hmg124h_8.JPG.dat	0.0	0.0	0.0	0.00	#NUM!	
YKL007W	YKL007W	UWOPS87_ctrl_hmg124h_8.JPG.dat	1110.3	53.0	0.9	0.02	#NUM!	1.3E-04
YKL008C	YKL008C	UWOPS87_ctrl_hmg124h_8.JPG.dat	1282.8	103.3	1.1	0.08	#NUM!	1.2E-04
YKL009W	YKL009W	UWOPS87_ctrl_hmg124h_8.JPG.dat	985.0	72.4	0.9	0.07	#NUM!	9.9E-05

YKL010C	YKL010C	UWOPS87_ctrl_hmg124h_8.JPG.dat	1140.0	163.1	1.0	0.14	#NUM!	8.3E-04
YKL015W	YKL015W	UWOPS87_ctrl_hmg124h_8.JPG.dat	1228.8	88.8	1.0	0.08	#NUM!	1.1E-04
YKL017C	YKL017C	UWOPS87_ctrl_hmg124h_8.JPG.dat	1131.0	216.9	1.1	0.06	#NUM!	1.0E-03
YKL020C	YKL020C	UWOPS87_ctrl_hmg124h_8.JPG.dat	1173.5	79.2	1.0	0.07	#NUM!	9.0E-05
YKL023W	YKL023W	UWOPS87_ctrl_hmg124h_8.JPG.dat	1326.0	93.7	1.1	0.07	#NUM!	8.2E-05
YKL025C	YKL025C	UWOPS87_ctrl_hmg124h_8.JPG.dat	1312.0	54.5	1.1	0.05	#NUM!	2.1E-05
YKL026C	YKL026C	UWOPS87_ctrl_hmg124h_8.JPG.dat	1139.3	39.6	1.0	0.03	#NUM!	5.3E-06
YKL027W	YKL027W	UWOPS87_ctrl_hmg124h_8.JPG.dat	1137.3	48.8	1.0	0.04	#NUM!	2.6E-05
YKL029C	YKL029C	UWOPS87_ctrl_hmg124h_8.JPG.dat	0.0	0.0	0.0	0.00	#NUM!	
YKL030W	YKL030W	UWOPS87_ctrl_hmg124h_8.JPG.dat	1279.8	154.7	1.1	0.13	#NUM!	4.8E-04
YKL031W	YKL031W	UWOPS87_ctrl_hmg124h_8.JPG.dat	1302.3	118.3	1.2	0.12	#NUM!	2.3E-04
YKL032C	YKL032C	UWOPS87_ctrl_hmg124h_8.JPG.dat	1303.5	39.0	1.1	0.03	#NUM!	4.6E-06
YKL033W-/	YKL033W-A	UWOPS87_ctrl_hmg124h_8.JPG.dat	877.8	585.3	1.0	0.01	#NUM!	2.9E-05
YKL034W	YKL034W	UWOPS87_ctrl_hmg124h_8.JPG.dat	1217.0	27.3	1.0	0.02	#NUM!	3.8E-06
YKL037W	YKL037W	UWOPS87_ctrl_hmg124h_8.JPG.dat	1182.8	31.1	1.0	0.03	#NUM!	5.0E-06
YKL038W	YKL038W	UWOPS87_ctrl_hmg124h_8.JPG.dat	1256.5	20.2	1.0	0.02	#NUM!	1.3E-06
YKL039W	YKL039W	UWOPS87_ctrl_hmg124h_8.JPG.dat	1238.5	49.9	1.0	0.05	#NUM!	2.6E-05
YKL040C	YKL040C	UWOPS87_ctrl_hmg124h_8.JPG.dat	1270.0	83.6	1.1	0.07	#NUM!	7.7E-05
YKL041W	YKL041W	UWOPS87_ctrl_hmg124h_8.JPG.dat	1229.3	65.6	1.1	0.01	#NUM!	4.1E-05
YKL043W	YKL043W	UWOPS87_ctrl_hmg124h_8.JPG.dat	1245.3	110.0	1.1	0.10	#NUM!	2.1E-04
YKL044W	YKL044W	UWOPS87_ctrl_hmg124h_8.JPG.dat	1117.3	27.6	1.0	0.03	#NUM!	5.1E-06
YKL046C	YKL046C	UWOPS87_ctrl_hmg124h_8.JPG.dat	1092.3	71.2	1.0	0.07	#NUM!	7.6E-05
YKL047W	YKL047W	UWOPS87_ctrl_hmg124h_8.JPG.dat	1087.0	69.3	1.0	0.07	#NUM!	8.7E-05
YKL048C	YKL048C	UWOPS87_ctrl_hmg124h_8.JPG.dat	1135.8	68.6	1.0	0.05	#NUM!	2.9E-05
YKL050C	YKL050C	UWOPS87_ctrl_hmg124h_8.JPG.dat	1217.5	17.4	1.1	0.00	#NUM!	2.9E-06
YKL051W	YKL051W	UWOPS87_ctrl_hmg124h_8.JPG.dat	1154.3	82.7	1.0	0.02	#NUM!	1.5E-04
YKL053C-A	YKL053C-A	UWOPS87_ctrl_hmg124h_8.JPG.dat	1264.3	83.2	1.1	0.07	#NUM!	6.8E-05
YKL053W	YKL053W	UWOPS87_ctrl_hmg124h_8.JPG.dat	1131.0	43.7	1.0	0.01	#NUM!	6.5E-05
YKL055C	YKL055C	UWOPS87_ctrl_hmg124h_8.JPG.dat	1228.3	70.7	1.1	0.05	#NUM!	2.6E-05
YKL056C	YKL056C	UWOPS87_ctrl_hmg124h_8.JPG.dat	1067.5	45.5	1.0	0.05	#NUM!	4.3E-05
YKL061W	YKL061W	UWOPS87_ctrl_hmg124h_8.JPG.dat	997.3	99.7	0.9	0.03	#NUM!	3.2E-04
YKL062W	YKL062W	UWOPS87_ctrl_hmg124h_8.JPG.dat	1159.5	38.8	1.0	0.01	#NUM!	2.9E-05
YKL063C	YKL063C	UWOPS87_ctrl_hmg124h_8.JPG.dat	1023.5	26.2	0.9	0.04	#NUM!	1.5E-05
YKL064W	YKL064W	UWOPS87_ctrl_hmg124h_8.JPG.dat	1177.5	88.0	1.0	0.05	#NUM!	3.4E-05
YKL065C	YKL065C	UWOPS87_ctrl_hmg124h_8.JPG.dat	0.0	0.0	0.0	0.00	#NUM!	
YKL066W	YKL066W	UWOPS87_ctrl_hmg124h_8.JPG.dat	1328.8	61.0	1.1	0.05	#NUM!	2.2E-05
YKL067W	YKL067W	UWOPS87_ctrl_hmg124h_8.JPG.dat	1213.0	27.4	1.0	0.03	#NUM!	8.3E-06
YKL068W	YKL068W	UWOPS87_ctrl_hmg124h_8.JPG.dat	1200.0	38.8	1.0	0.03	#NUM!	5.0E-06
YKL069W	YKL069W	UWOPS87_ctrl_hmg124h_8.JPG.dat	953.5	648.1	1.0	0.12	#NUM!	4.4E-03
YKL070W	YKL070W	UWOPS87_ctrl_hmg124h_8.JPG.dat	1289.8	73.2	1.0	0.05	#NUM!	3.4E-05
YKL071W	YKL071W	UWOPS87_ctrl_hmg124h_8.JPG.dat	1316.8	57.0	1.1	0.00	#NUM!	6.8E-06
YKL072W	YKL072W	UWOPS87_ctrl_hmg124h_8.JPG.dat	1344.8	14.8	1.0	0.02	#NUM!	1.9E-06
YKL073W	YKL073W	UWOPS87_ctrl_hmg124h_8.JPG.dat	1285.0	47.7	1.1	0.01	#NUM!	4.5E-05
YKL074C	YKL074C	UWOPS87_ctrl_hmg124h_8.JPG.dat	694.3	466.0	0.9	0.06	#NUM!	1.6E-03
YKL075C	YKL075C	UWOPS87_ctrl_hmg124h_8.JPG.dat	1117.5	119.6	1.0	0.10	#NUM!	2.2E-04
YKL076C	YKL076C	UWOPS87_ctrl_hmg124h_8.JPG.dat	565.3	652.9	0.5	0.58	#NUM!	1.8E-01
YKL077W	YKL077W	UWOPS87_ctrl_hmg124h_8.JPG.dat	1115.8	87.9	1.0	0.07	#NUM!	1.3E-04
YKL079W	YKL079W	UWOPS87_ctrl_hmg124h_8.JPG.dat	1223.0	54.2	1.0	0.05	#NUM!	2.7E-05
YKL081W	YKL081W	UWOPS87_ctrl_hmg124h_8.JPG.dat	1171.8	67.1	1.0	0.06	#NUM!	4.8E-05
YKL084W	YKL084W	UWOPS87_ctrl_hmg124h_8.JPG.dat	1236.8	49.9	1.0	0.01	#NUM!	5.2E-05
YKL085W	YKL085W	UWOPS87_ctrl_hmg124h_8.JPG.dat	1166.0	44.7	1.0	0.05	#NUM!	2.7E-05
YKL086W	YKL086W	UWOPS87_ctrl_hmg124h_8.JPG.dat	1259.0	47.2	1.0	0.02	#NUM!	7.9E-05
YKL090W	YKL090W	UWOPS87_ctrl_hmg124h_8.JPG.dat	1283.8	123.6	1.1	0.09	#NUM!	2.0E-04
YKL091C	YKL091C	UWOPS87_ctrl_hmg124h_8.JPG.dat	1220.5	73.3	1.0	0.06	#NUM!	4.4E-05
YKL092C	YKL092C	UWOPS87_ctrl_hmg124h_8.JPG.dat	986.0	63.3	0.9	0.06	#NUM!	7.5E-05
YKL093W	YKL093W	UWOPS87_ctrl_hmg124h_8.JPG.dat	1057.8	78.4	1.0	0.05	#NUM!	4.1E-05
YKL094W	YKL094W	UWOPS87_ctrl_hmg124h_8.JPG.dat	1029.3	71.5	0.9	0.06	#NUM!	1.0E-04
YKL096W	YKL096W	UWOPS87_ctrl_hmg124h_8.JPG.dat	1245.8	59.5	1.1	0.01	#NUM!	9.0E-06
YKL096W-/	YKL096W-A	UWOPS87_ctrl_hmg124h_8.JPG.dat	1210.0	19.4	1.1	0.01	#NUM!	8.0E-06
YKL097C	YKL097C	UWOPS87_ctrl_hmg124h_8.JPG.dat	1184.0	64.5	1.0	0.05	#NUM!	4.3E-05
YKL098W	YKL098W	UWOPS87_ctrl_hmg124h_8.JPG.dat	1281.0	60.4	1.1	0.05	#NUM!	2.7E-05
YKL100C	YKL100C	UWOPS87_ctrl_hmg124h_8.JPG.dat	1067.3	48.2	0.9	0.02	#NUM!	9.6E-05
YKL101W	YKL101W	UWOPS87_ctrl_hmg124h_8.JPG.dat	1183.3	187.4	1.0	0.16	#NUM!	1.1E-03
YKL102C	YKL102C	UWOPS87_ctrl_hmg124h_8.JPG.dat	1179.8	70.7	1.0	0.06	#NUM!	5.8E-05
YKL103C	YKL103C	UWOPS87_ctrl_hmg124h_8.JPG.dat	1149.0	51.9	1.0	0.05	#NUM!	2.9E-05
YKL105C	YKL105C	UWOPS87_ctrl_hmg124h_8.JPG.dat	1151.0	123.6	1.1	0.11	#NUM!	3.3E-04
YKL106W	YKL106W	UWOPS87_ctrl_hmg124h_8.JPG.dat	319.3	638.5	0.0	0.00	#NUM!	
YKL107W	YKL107W	UWOPS87_ctrl_hmg124h_8.JPG.dat	1179.5	85.4	1.0	0.08	#NUM!	1.6E-04
YKL109W	YKL109W	UWOPS87_ctrl_hmg124h_8.JPG.dat	1016.8	678.8	1.1	0.03	#NUM!	3.1E-04
YKL110C	YKL110C	UWOPS87_ctrl_hmg124h_8.JPG.dat	1104.3	30.6	0.9	0.03	#NUM!	6.7E-06

YKL113C	YKL113C	UWOPS87_ctrl_hmg124h_8.JPG.dat	1131.8	31.9	1.0	0.03	#NUM!	1.2E-05
YKL114C	YKL114C	UWOPS87_ctrl_hmg124h_8.JPG.dat	1194.0	8.1	1.0	0.01	#NUM!	2.1E-07
YKL115C	YKL115C	UWOPS87_ctrl_hmg124h_8.JPG.dat	1084.5	98.8	0.9	0.08	#NUM!	1.8E-04
YKL116C	YKL116C	UWOPS87_ctrl_hmg124h_8.JPG.dat	1265.5	51.9	1.1	0.04	#NUM!	2.1E-05
YKL117W	YKL117W	UWOPS87_ctrl_hmg124h_8.JPG.dat	1203.8	84.1	1.0	0.06	#NUM!	7.4E-05
YKL120W	YKL120W	UWOPS87_ctrl_hmg124h_8.JPG.dat	1191.0	30.8	1.0	0.00	#NUM!	2.1E-06
YKL121W	YKL121W	UWOPS87_ctrl_hmg124h_8.JPG.dat	1055.0	63.3	1.0	0.02	#NUM!	1.5E-04
YKL123W	YKL123W	UWOPS87_ctrl_hmg124h_8.JPG.dat	1049.0	93.2	0.9	0.08	#NUM!	1.5E-04
YKL124W	YKL124W	UWOPS87_ctrl_hmg124h_8.JPG.dat	1141.3	129.2	1.0	0.02	#NUM!	7.9E-05
YKL127W	YKL127W	UWOPS87_ctrl_hmg124h_8.JPG.dat	1246.3	10.7	1.1	0.01	#NUM!	3.1E-08
YKL128C	YKL128C	UWOPS87_ctrl_hmg124h_8.JPG.dat	1224.3	19.5	1.0	0.02	#NUM!	2.1E-06
YKL129C	YKL129C	UWOPS87_ctrl_hmg124h_8.JPG.dat	1190.8	86.4	1.0	0.03	#NUM!	2.1E-04
YKL130C	YKL130C	UWOPS87_ctrl_hmg124h_8.JPG.dat	209.8	419.5	0.0	0.00	#NUM!	
YKL131W	YKL131W	UWOPS87_ctrl_hmg124h_8.JPG.dat	1213.8	66.8	1.0	0.05	#NUM!	2.8E-05
YKL132C	YKL132C	UWOPS87_ctrl_hmg124h_8.JPG.dat	1094.8	44.4	0.9	0.04	#NUM!	1.7E-05
YKL133C	YKL133C	UWOPS87_ctrl_hmg124h_8.JPG.dat	1286.3	72.3	1.1	0.06	#NUM!	4.6E-05
YKL136W	YKL136W	UWOPS87_ctrl_hmg124h_8.JPG.dat	1215.0	72.1	1.0	0.06	#NUM!	5.3E-05
YKL137W	YKL137W	UWOPS87_ctrl_hmg124h_8.JPG.dat	1003.8	58.2	0.9	0.06	#NUM!	6.0E-05
YKL139W	YKL139W	UWOPS87_ctrl_hmg124h_8.JPG.dat	1058.3	92.2	0.9	0.07	#NUM!	1.1E-04
YKL140W	YKL140W	UWOPS87_ctrl_hmg124h_8.JPG.dat	1255.0	94.7	1.1	0.08	#NUM!	1.1E-04
YKL142W	YKL142W	UWOPS87_ctrl_hmg124h_8.JPG.dat	1202.5	30.8	1.0	0.03	#NUM!	4.2E-06
YKL146W	YKL146W	UWOPS87_ctrl_hmg124h_8.JPG.dat	1191.0	50.3	1.0	0.04	#NUM!	2.0E-05
YKL147C	YKL147C	UWOPS87_ctrl_hmg124h_8.JPG.dat	1174.5	57.9	1.0	0.04	#NUM!	2.3E-05
YKL148C	YKL148C	UWOPS87_ctrl_hmg124h_8.JPG.dat	1260.8	50.8	1.0	0.04	#NUM!	1.3E-05
YKL149C	YKL149C	UWOPS87_ctrl_hmg124h_8.JPG.dat	1241.5	56.4	1.0	0.01	#NUM!	2.2E-05
YKL150W	YKL150W	UWOPS87_ctrl_hmg124h_8.JPG.dat	1173.3	37.1	1.0	0.03	#NUM!	6.5E-06
YKL151C	YKL151C	UWOPS87_ctrl_hmg124h_8.JPG.dat	1228.3	117.2	1.0	0.03	#NUM!	3.3E-04
YKL156W	YKL156W	UWOPS87_ctrl_hmg124h_8.JPG.dat	1128.5	36.0	1.0	0.01	#NUM!	4.2E-05
YKL157W	YKL157W	UWOPS87_ctrl_hmg124h_8.JPG.dat	1092.8	49.4	1.0	0.04	#NUM!	1.3E-05
YKL158W	YKL158W	UWOPS87_ctrl_hmg124h_8.JPG.dat	1067.5	70.8	1.0	0.06	#NUM!	7.0E-05
YKL159C	YKL159C	UWOPS87_ctrl_hmg124h_8.JPG.dat	1118.8	93.2	1.0	0.09	#NUM!	2.0E-04
YKL160W	YKL160W	UWOPS87_ctrl_hmg124h_8.JPG.dat	1046.3	45.9	0.9	0.01	#NUM!	5.3E-05
YKL161C	YKL161C	UWOPS87_ctrl_hmg124h_8.JPG.dat	1104.3	50.3	1.0	0.01	#NUM!	3.9E-05
YKL162C	YKL162C	UWOPS87_ctrl_hmg124h_8.JPG.dat	1028.0	27.9	0.9	0.04	#NUM!	1.7E-05
YKL163W	YKL163W	UWOPS87_ctrl_hmg124h_8.JPG.dat	1070.3	31.0	0.9	0.02	#NUM!	1.4E-06
YKL164C	YKL164C	UWOPS87_ctrl_hmg124h_8.JPG.dat	1201.3	68.5	1.1	0.05	#NUM!	3.8E-05
YKL166C	YKL166C	UWOPS87_ctrl_hmg124h_8.JPG.dat	1079.5	59.0	1.0	0.01	#NUM!	2.3E-05
YKL167C	YKL167C	UWOPS87_ctrl_hmg124h_8.JPG.dat	1179.0	112.3	1.1	0.09	#NUM!	1.9E-04
YKL168C	YKL168C	UWOPS87_ctrl_hmg124h_8.JPG.dat	858.0	572.2	1.0	0.02	#NUM!	8.4E-05
YKL171W	YKL171W	UWOPS87_ctrl_hmg124h_8.JPG.dat	1137.0	76.3	1.0	0.07	#NUM!	8.8E-05
YKL174C	YKL174C	UWOPS87_ctrl_hmg124h_8.JPG.dat	1263.5	32.3	1.0	0.01	#NUM!	2.4E-05
YKL175W	YKL175W	UWOPS87_ctrl_hmg124h_8.JPG.dat	1171.3	42.8	0.9	0.01	#NUM!	1.7E-05
YKL176C	YKL176C	UWOPS87_ctrl_hmg124h_8.JPG.dat	1114.8	88.9	0.9	0.03	#NUM!	2.5E-04
YKL177W	YKL177W	UWOPS87_ctrl_hmg124h_8.JPG.dat	1313.8	98.5	1.0	0.02	#NUM!	1.7E-04
YKL178C	YKL178C	UWOPS87_ctrl_hmg124h_8.JPG.dat	1255.8	96.7	1.0	0.07	#NUM!	1.1E-04
YKL179C	YKL179C	UWOPS87_ctrl_hmg124h_8.JPG.dat	1388.5	82.2	1.1	0.01	#NUM!	1.5E-05
YKL183W	YKL183W	UWOPS87_ctrl_hmg124h_8.JPG.dat	1093.5	15.0	0.9	0.02	#NUM!	3.6E-06
YKL184W	YKL184W	UWOPS87_ctrl_hmg124h_8.JPG.dat	1080.8	103.7	0.8	0.02	#NUM!	2.6E-04
YKL185W	YKL185W	UWOPS87_ctrl_hmg124h_8.JPG.dat	1322.5	173.2	1.0	0.13	#NUM!	5.8E-04
YKL187C	YKL187C	UWOPS87_ctrl_hmg124h_8.JPG.dat	1295.0	93.4	1.1	0.07	#NUM!	8.1E-05
YKL188C	YKL188C	UWOPS87_ctrl_hmg124h_8.JPG.dat	1019.0	132.0	1.0	0.12	#NUM!	4.6E-04
YKL190W	YKL190W	UWOPS87_ctrl_hmg124h_8.JPG.dat	872.5	582.1	1.0	0.02	#NUM!	1.6E-04
YKL191W	YKL191W	UWOPS87_ctrl_hmg124h_8.JPG.dat	1246.8	50.2	1.0	0.02	#NUM!	7.0E-05
YKL197C	YKL197C	UWOPS87_ctrl_hmg124h_8.JPG.dat	1258.8	126.6	1.1	0.11	#NUM!	2.8E-04
YKL198C	YKL198C	UWOPS87_ctrl_hmg124h_8.JPG.dat	925.3	620.5	1.0	0.07	#NUM!	1.6E-03
YKL199C	YKL199C	UWOPS87_ctrl_hmg124h_8.JPG.dat	1168.5	44.6	1.0	0.04	#NUM!	1.9E-05
YKL200C	YKL200C	UWOPS87_ctrl_hmg124h_8.JPG.dat	1339.3	33.9	1.1	0.03	#NUM!	4.4E-06
YKL201C	YKL201C	UWOPS87_ctrl_hmg124h_8.JPG.dat	1181.3	91.5	1.0	0.01	#NUM!	6.6E-05
YKL202W	YKL202W	UWOPS87_ctrl_hmg124h_8.JPG.dat	1023.3	253.8	1.0	0.03	#NUM!	2.5E-04
YKL204W	YKL204W	UWOPS87_ctrl_hmg124h_8.JPG.dat	1261.3	207.7	1.0	0.17	#NUM!	1.2E-03
YKL205W	YKL205W	UWOPS87_ctrl_hmg124h_8.JPG.dat	1150.8	71.2	1.0	0.06	#NUM!	6.1E-05
YKL206C	YKL206C	UWOPS87_ctrl_hmg124h_8.JPG.dat	1172.0	35.8	1.1	0.01	#NUM!	2.2E-05
YKL207W	YKL207W	UWOPS87_ctrl_hmg124h_8.JPG.dat	1263.8	24.3	1.1	0.02	#NUM!	2.8E-06
YKL208W	YKL208W	UWOPS87_ctrl_hmg124h_8.JPG.dat	1277.3	21.7	1.1	0.01	#NUM!	2.2E-07
YKL211C	YKL211C	UWOPS87_ctrl_hmg124h_8.JPG.dat	1203.0	63.0	1.0	0.06	#NUM!	4.8E-05
YKL212W	YKL212W	UWOPS87_ctrl_hmg124h_8.JPG.dat	1151.3	37.5	1.0	0.00	#NUM!	1.7E-06
YKL213C	YKL213C	UWOPS87_ctrl_hmg124h_8.JPG.dat	1176.0	62.4	1.0	0.06	#NUM!	5.5E-05
YKL214C	YKL214C	UWOPS87_ctrl_hmg124h_8.JPG.dat	1085.8	52.9	0.9	0.04	#NUM!	2.6E-05
YKL215C	YKL215C	UWOPS87_ctrl_hmg124h_8.JPG.dat	1033.3	148.4	0.9	0.12	#NUM!	6.5E-04
YKL216W	YKL216W	UWOPS87_ctrl_hmg124h_8.JPG.dat	1248.0	38.4	1.0	0.02	#NUM!	2.5E-06

YKL217W	YKL217W	UWOPS87_ctrl_hmg124h_8.JPG.dat	1190.8	89.7	1.0	0.07	#NUM!	1.1E-04
YKL218C	YKL218C	UWOPS87_ctrl_hmg124h_8.JPG.dat	1191.5	171.2	0.9	0.05	#NUM!	8.2E-04
YKL220C	YKL220C	UWOPS87_ctrl_hmg124h_8.JPG.dat	1112.8	98.8	1.0	0.10	#NUM!	2.3E-04
YKL221W	YKL221W	UWOPS87_ctrl_hmg124h_8.JPG.dat	1198.5	145.5	1.0	0.12	#NUM!	4.3E-04
YKL222C	YKL222C	UWOPS87_ctrl_hmg124h_8.JPG.dat	1191.0	27.6	1.0	0.03	#NUM!	4.4E-06
YKR001C	YKR001C	UWOPS87_ctrl_hmg124h_8.JPG.dat	1185.8	48.4	1.0	0.04	#NUM!	1.6E-05
YKR003W	YKR003W	UWOPS87_ctrl_hmg124h_8.JPG.dat	1118.0	58.1	1.0	0.05	#NUM!	4.2E-05
YKR005C	YKR005C	UWOPS87_ctrl_hmg124h_8.JPG.dat	1214.8	63.4	1.0	0.06	#NUM!	4.9E-05
YKR007W	YKR007W	UWOPS87_ctrl_hmg124h_8.JPG.dat	1213.8	64.8	1.0	0.06	#NUM!	5.2E-05
YKR009C	YKR009C	UWOPS87_ctrl_hmg124h_8.JPG.dat	1198.0	42.3	1.0	0.04	#NUM!	1.2E-05
YKR010C	YKR010C	UWOPS87_ctrl_hmg124h_8.JPG.dat	1136.5	30.3	1.0	0.02	#NUM!	4.2E-06
YKR011C	YKR011C	UWOPS87_ctrl_hmg124h_8.JPG.dat	1283.8	37.3	1.1	0.03	#NUM!	4.9E-06
YKR012C	YKR012C	UWOPS87_ctrl_hmg124h_8.JPG.dat	1189.5	136.0	0.9	0.03	#NUM!	4.4E-04
YKR013W	YKR013W	UWOPS87_ctrl_hmg124h_8.JPG.dat	1185.5	46.2	1.1	0.05	#NUM!	2.4E-05
YKR014C	YKR014C	UWOPS87_ctrl_hmg124h_8.JPG.dat	1320.8	76.9	1.1	0.07	#NUM!	7.7E-05
YKR015C	YKR015C	UWOPS87_ctrl_hmg124h_8.JPG.dat	1238.8	107.5	1.0	0.09	#NUM!	1.7E-04
YKR016W	YKR016W	UWOPS87_ctrl_hmg124h_8.JPG.dat	1140.3	19.4	0.9	0.00	#NUM!	2.9E-06
YKR017C	YKR017C	UWOPS87_ctrl_hmg124h_8.JPG.dat	1124.3	48.9	0.9	0.04	#NUM!	2.8E-05
YKR018C	YKR018C	UWOPS87_ctrl_hmg124h_8.JPG.dat	1248.0	67.5	1.0	0.05	#NUM!	3.9E-05
YKR019C	YKR019C	UWOPS87_ctrl_hmg124h_8.JPG.dat	1243.0	101.5	1.0	0.09	#NUM!	1.7E-04
YKR020W	YKR020W	UWOPS87_ctrl_hmg124h_8.JPG.dat	298.5	597.0	0.0	0.00	#NUM!	
YKR021W	YKR021W	UWOPS87_ctrl_hmg124h_8.JPG.dat	1262.5	167.3	1.1	0.13	#NUM!	5.6E-04
YKR023W	YKR023W	UWOPS87_ctrl_hmg124h_8.JPG.dat	1216.3	59.9	1.0	0.05	#NUM!	4.1E-05
YKR024C	YKR024C	UWOPS87_ctrl_hmg124h_8.JPG.dat	1081.5	128.3	0.9	0.10	#NUM!	4.2E-04
YKR026C	YKR026C	UWOPS87_ctrl_hmg124h_8.JPG.dat	1034.5	38.7	1.0	0.03	#NUM!	5.7E-06
YKR027W	YKR027W	UWOPS87_ctrl_hmg124h_8.JPG.dat	1208.3	37.7	1.0	0.04	#NUM!	2.2E-05
YKR028W	YKR028W	UWOPS87_ctrl_hmg124h_8.JPG.dat	1092.8	39.2	0.9	0.01	#NUM!	1.9E-05
YKR029C	YKR029C	UWOPS87_ctrl_hmg124h_8.JPG.dat	1203.8	69.5	1.1	0.02	#NUM!	7.3E-05
YKR030W	YKR030W	UWOPS87_ctrl_hmg124h_8.JPG.dat	1261.8	59.7	1.1	0.05	#NUM!	3.0E-05
YKR031C	YKR031C	UWOPS87_ctrl_hmg124h_8.JPG.dat	1164.0	55.6	1.0	0.04	#NUM!	2.1E-05
YKR032W	YKR032W	UWOPS87_ctrl_hmg124h_8.JPG.dat	943.5	630.1	1.0	0.03	#NUM!	2.9E-04
YKR033C	YKR033C	UWOPS87_ctrl_hmg124h_8.JPG.dat	1060.5	166.4	0.9	0.14	#NUM!	1.1E-03
YKR034W	YKR034W	UWOPS87_ctrl_hmg124h_8.JPG.dat	1128.3	100.7	1.0	0.08	#NUM!	1.5E-04
YKR035C	YKR035C	UWOPS87_ctrl_hmg124h_8.JPG.dat	1114.5	71.0	1.0	0.06	#NUM!	6.0E-05
YKR035W-A	YKR035W-A	UWOPS87_ctrl_hmg124h_8.JPG.dat	1093.0	54.6	0.9	0.05	#NUM!	4.2E-05
YKR036C	YKR036C	UWOPS87_ctrl_hmg124h_8.JPG.dat	1019.5	27.1	1.0	0.04	#NUM!	1.4E-05
YKR039W	YKR039W	UWOPS87_ctrl_hmg124h_8.JPG.dat	1110.5	60.2	1.0	0.05	#NUM!	4.6E-05
YKR040C	YKR040C	UWOPS87_ctrl_hmg124h_8.JPG.dat	1201.0	126.0	1.0	0.11	#NUM!	2.8E-04
YKR041W	YKR041W	UWOPS87_ctrl_hmg124h_8.JPG.dat	1098.8	24.3	1.0	0.02	#NUM!	4.5E-06
YKR042W	YKR042W	UWOPS87_ctrl_hmg124h_8.JPG.dat	1191.3	41.3	1.0	0.02	#NUM!	7.4E-05
YKR043C	YKR043C	UWOPS87_ctrl_hmg124h_8.JPG.dat	1264.5	26.6	1.1	0.03	#NUM!	5.3E-06
YKR044W	YKR044W	UWOPS87_ctrl_hmg124h_8.JPG.dat	1226.0	69.2	1.0	0.01	#NUM!	1.0E-05
YKR045C	YKR045C	UWOPS87_ctrl_hmg124h_8.JPG.dat	1282.5	19.8	1.1	0.02	#NUM!	1.2E-06
YKR046C	YKR046C	UWOPS87_ctrl_hmg124h_8.JPG.dat	1165.3	94.2	1.0	0.08	#NUM!	1.2E-04
YKR047W	YKR047W	UWOPS87_ctrl_hmg124h_8.JPG.dat	1041.8	56.5	0.9	0.04	#NUM!	2.9E-05
YKR048C	YKR048C	UWOPS87_ctrl_hmg124h_8.JPG.dat	1107.3	56.0	1.0	0.05	#NUM!	4.7E-05
YKR049C	YKR049C	UWOPS87_ctrl_hmg124h_8.JPG.dat	1177.3	23.4	1.0	0.01	#NUM!	4.0E-07
YKR050W	YKR050W	UWOPS87_ctrl_hmg124h_8.JPG.dat	1132.0	28.0	0.9	0.01	#NUM!	4.3E-07
YKR051W	YKR051W	UWOPS87_ctrl_hmg124h_8.JPG.dat	1405.3	103.5	1.1	0.09	#NUM!	1.2E-04
YKR052C	YKR052C	UWOPS87_ctrl_hmg124h_8.JPG.dat	1200.0	94.5	1.0	0.07	#NUM!	1.2E-04
YKR053C	YKR053C	UWOPS87_ctrl_hmg124h_8.JPG.dat	1347.3	27.2	1.1	0.03	#NUM!	3.7E-06
YKR054C	YKR054C	UWOPS87_ctrl_hmg124h_8.JPG.dat	546.3	295.4	0.4	0.24	#NUM!	3.5E-02
YKR055W	YKR055W	UWOPS87_ctrl_hmg124h_8.JPG.dat	1382.3	72.3	1.1	0.06	#NUM!	3.7E-05
YKR056W	YKR056W	UWOPS87_ctrl_hmg124h_8.JPG.dat	1260.3	35.9	1.0	0.03	#NUM!	6.6E-06
YKR057W	YKR057W	UWOPS87_ctrl_hmg124h_8.JPG.dat	906.5	57.3	0.7	0.04	#NUM!	4.8E-05
YKR058W	YKR058W	UWOPS87_ctrl_hmg124h_8.JPG.dat	1416.3	101.7	1.1	0.08	#NUM!	9.7E-05
YKR059W	YKR059W	UWOPS87_ctrl_hmg124h_8.JPG.dat	1004.0	121.2	0.8	0.09	#NUM!	3.6E-04
YKR060W	YKR060W	UWOPS87_ctrl_hmg124h_8.JPG.dat	1031.8	114.3	1.0	0.12	#NUM!	4.6E-04
YKR061W	YKR061W	UWOPS87_ctrl_hmg124h_8.JPG.dat	1061.0	69.7	1.0	0.07	#NUM!	9.1E-05
YKR064W	YKR064W	UWOPS87_ctrl_hmg124h_8.JPG.dat	0.0	0.0	0.0	0.00	#NUM!	
YKR065C	YKR065C	UWOPS87_ctrl_hmg124h_8.JPG.dat	1207.0	59.5	1.0	0.01	#NUM!	2.2E-05
YKR066C	YKR066C	UWOPS87_ctrl_hmg124h_8.JPG.dat	1264.5	59.8	1.1	0.05	#NUM!	2.5E-05
YKR067W	YKR067W	UWOPS87_ctrl_hmg124h_8.JPG.dat	1234.3	37.1	1.0	0.03	#NUM!	5.7E-06
YKR069W	YKR069W	UWOPS87_ctrl_hmg124h_8.JPG.dat	1198.8	38.9	1.0	0.03	#NUM!	1.2E-05
YKR070W	YKR070W	UWOPS87_ctrl_hmg124h_8.JPG.dat	1219.8	69.5	1.0	0.06	#NUM!	5.9E-05
YKR072C	YKR072C	UWOPS87_ctrl_hmg124h_8.JPG.dat	1196.0	6.5	1.0	0.00	#NUM!	6.9E-07
YKR074W	YKR074W	UWOPS87_ctrl_hmg124h_8.JPG.dat	1115.0	61.5	0.9	0.01	#NUM!	5.5E-05
YKR076W	YKR076W	UWOPS87_ctrl_hmg124h_8.JPG.dat	1270.5	95.8	1.1	0.08	#NUM!	1.1E-04
YKR077W	YKR077W	UWOPS87_ctrl_hmg124h_8.JPG.dat	977.3	61.7	1.0	0.01	#NUM!	6.1E-05
YKR078W	YKR078W	UWOPS87_ctrl_hmg124h_8.JPG.dat	1120.5	92.9	1.0	0.02	#NUM!	8.6E-05

YKR080W	YKR080W	UWOPS87_ctrl_hmg124h_8.JPG.dat	1113.3	110.4	1.0	0.10	#NUM!	2.6E-04
YKR082W	YKR082W	UWOPS87_ctrl_hmg124h_8.JPG.dat	804.5	357.1	0.7	0.30	#NUM!	2.0E-02
YKR084C	YKR084C	UWOPS87_ctrl_hmg124h_8.JPG.dat	1003.8	34.7	0.9	0.02	#NUM!	6.8E-06
YKR087C	YKR087C	UWOPS87_ctrl_hmg124h_8.JPG.dat	1244.0	41.8	1.1	0.04	#NUM!	1.3E-05
YKR088C	YKR088C	UWOPS87_ctrl_hmg124h_8.JPG.dat	1245.3	46.3	1.0	0.04	#NUM!	2.1E-05
YKR089C	YKR089C	UWOPS87_ctrl_hmg124h_8.JPG.dat	1257.8	64.7	1.1	0.05	#NUM!	2.2E-05
YKR090W	YKR090W	UWOPS87_ctrl_hmg124h_8.JPG.dat	1286.8	44.2	1.1	0.03	#NUM!	8.3E-06
YKR091W	YKR091W	UWOPS87_ctrl_hmg124h_8.JPG.dat	1201.8	40.8	1.0	0.03	#NUM!	5.3E-06
YKR092C	YKR092C	UWOPS87_ctrl_hmg124h_8.JPG.dat	1111.5	63.3	0.9	0.06	#NUM!	7.3E-05
YKR093W	YKR093W	UWOPS87_ctrl_hmg124h_8.JPG.dat	962.8	18.3	0.9	0.02	#NUM!	3.1E-06
YKR094C	YKR094C	UWOPS87_ctrl_hmg124h_8.JPG.dat	1183.8	87.9	1.1	0.02	#NUM!	1.2E-04
YKR095W	YKR095W	UWOPS87_ctrl_hmg124h_8.JPG.dat	1123.8	84.9	1.0	0.08	#NUM!	1.3E-04
YKR096W	YKR096W	UWOPS87_ctrl_hmg124h_8.JPG.dat	1095.5	35.6	1.0	0.01	#NUM!	1.3E-05
YKR097W	YKR097W	UWOPS87_ctrl_hmg124h_8.JPG.dat	1257.8	119.9	1.1	0.01	#NUM!	4.9E-05
YKR098C	YKR098C	UWOPS87_ctrl_hmg124h_8.JPG.dat	1206.0	144.0	1.1	0.02	#NUM!	9.1E-05
YKR099W	YKR099W	UWOPS87_ctrl_hmg124h_8.JPG.dat	1135.8	82.5	0.9	0.07	#NUM!	1.1E-04
YKR100C	YKR100C	UWOPS87_ctrl_hmg124h_8.JPG.dat	1234.5	63.6	1.1	0.01	#NUM!	4.4E-05
YKR101W	YKR101W	UWOPS87_ctrl_hmg124h_8.JPG.dat	1221.0	40.2	1.0	0.03	#NUM!	9.6E-06
YKR102W	YKR102W	UWOPS87_ctrl_hmg124h_8.JPG.dat	1236.5	41.3	1.0	0.03	#NUM!	1.0E-05
YKR103W	YKR103W	UWOPS87_ctrl_hmg124h_8.JPG.dat	1224.5	38.6	1.0	0.03	#NUM!	8.6E-06
YKR105C	YKR105C	UWOPS87_ctrl_hmg124h_8.JPG.dat	1154.0	67.6	1.1	0.05	#NUM!	3.5E-05
YKR106W	YKR106W	UWOPS87_ctrl_hmg124h_8.JPG.dat	1297.0	35.3	1.1	0.01	#NUM!	3.9E-05
YLL001W	YLL001W	UWOPS87_ctrl_hmg124h_8.JPG.dat	1069.8	71.3	0.9	0.06	#NUM!	7.4E-05
YLL002W	YLL002W	UWOPS87_ctrl_hmg124h_8.JPG.dat	436.0	505.4	0.4	0.42	#NUM!	1.8E-01
YLL005C	YLL005C	UWOPS87_ctrl_hmg124h_8.JPG.dat	1234.0	13.8	1.0	0.01	#NUM!	4.2E-07
YLL006W	YLL006W	UWOPS87_ctrl_hmg124h_8.JPG.dat	1217.0	57.3	1.0	0.05	#NUM!	3.0E-05
YLL007C	YLL007C	UWOPS87_ctrl_hmg124h_8.JPG.dat	1226.8	44.7	1.0	0.04	#NUM!	1.4E-05
YLL009C	YLL009C	UWOPS87_ctrl_hmg124h_8.JPG.dat	1198.5	157.6	1.0	0.13	#NUM!	6.5E-04
YLL010C	YLL010C	UWOPS87_ctrl_hmg124h_8.JPG.dat	1225.0	52.7	1.0	0.04	#NUM!	1.2E-05
YLL012W	YLL012W	UWOPS87_ctrl_hmg124h_8.JPG.dat	1244.3	73.7	1.0	0.07	#NUM!	8.7E-05
YLL013C	YLL013C	UWOPS87_ctrl_hmg124h_8.JPG.dat	1263.8	33.2	1.1	0.03	#NUM!	8.1E-06
YLL014W	YLL014W	UWOPS87_ctrl_hmg124h_8.JPG.dat	1085.0	56.0	1.0	0.06	#NUM!	5.3E-05
YLL015W	YLL015W	UWOPS87_ctrl_hmg124h_8.JPG.dat	1151.3	35.8	1.0	0.01	#NUM!	6.2E-05
YLL016W	YLL016W	UWOPS87_ctrl_hmg124h_8.JPG.dat	1264.5	27.8	1.1	0.03	#NUM!	8.1E-06
YLL017W	YLL017W	UWOPS87_ctrl_hmg124h_8.JPG.dat	1159.3	48.1	1.0	0.04	#NUM!	2.0E-05
YLL019C	YLL019C	UWOPS87_ctrl_hmg124h_8.JPG.dat	1131.5	71.4	1.0	0.01	#NUM!	5.5E-05
YLL020C	YLL020C	UWOPS87_ctrl_hmg124h_8.JPG.dat	859.3	576.4	1.0	0.07	#NUM!	1.8E-03
YLL021W	YLL021W	UWOPS87_ctrl_hmg124h_8.JPG.dat	1216.3	39.9	1.0	0.03	#NUM!	9.7E-06
YLL023C	YLL023C	UWOPS87_ctrl_hmg124h_8.JPG.dat	1189.5	21.3	1.0	0.02	#NUM!	1.8E-06
YLL024C	YLL024C	UWOPS87_ctrl_hmg124h_8.JPG.dat	1137.5	38.8	1.0	0.03	#NUM!	7.4E-06
YLL025W	YLL025W	UWOPS87_ctrl_hmg124h_8.JPG.dat	1258.8	78.3	1.1	0.08	#NUM!	1.0E-04
YLL026W	YLL026W	UWOPS87_ctrl_hmg124h_8.JPG.dat	1155.8	92.6	1.0	0.07	#NUM!	8.8E-05
YLL028W	YLL028W	UWOPS87_ctrl_hmg124h_8.JPG.dat	1055.8	71.6	1.0	0.08	#NUM!	1.4E-04
YLL029W	YLL029W	UWOPS87_ctrl_hmg124h_8.JPG.dat	1140.5	55.6	1.1	0.02	#NUM!	1.0E-04
YLL032C	YLL032C	UWOPS87_ctrl_hmg124h_8.JPG.dat	1126.0	61.4	1.0	0.06	#NUM!	5.7E-05
YLL038C	YLL038C	UWOPS87_ctrl_hmg124h_8.JPG.dat	1188.5	21.0	1.0	0.02	#NUM!	1.6E-06
YLL039C	YLL039C	UWOPS87_ctrl_hmg124h_8.JPG.dat	1169.5	36.3	1.0	0.01	#NUM!	4.5E-05
YLL040C	YLL040C	UWOPS87_ctrl_hmg124h_8.JPG.dat	1289.3	69.3	1.1	0.05	#NUM!	2.8E-05
YLL041C	YLL041C	UWOPS87_ctrl_hmg124h_8.JPG.dat	1294.0	50.6	1.1	0.05	#NUM!	2.2E-05
YLL042C	YLL042C	UWOPS87_ctrl_hmg124h_8.JPG.dat	1231.3	73.5	1.0	0.07	#NUM!	6.9E-05
YLL043W	YLL043W	UWOPS87_ctrl_hmg124h_8.JPG.dat	1306.0	75.7	1.1	0.07	#NUM!	5.3E-05
YLL044W	YLL044W	UWOPS87_ctrl_hmg124h_8.JPG.dat	1070.8	13.4	0.9	0.01	#NUM!	8.3E-08
YLL045C	YLL045C	UWOPS87_ctrl_hmg124h_8.JPG.dat	965.0	50.7	0.8	0.02	#NUM!	2.3E-04
YOR202W	YOR202W	UWOPS87_ctrl_hmg124h_8.JPG.dat	1324.6	191.6	1.0	0.08	#NUM!	6.9E-304
1	1	UWOPS87_ctrl_hmg124h_9.JPG.dat	1684.8	389.0	#NUM!		#NUM!	
2	2	UWOPS87_ctrl_hmg124h_9.JPG.dat	1252.3	132.2	1.2	0.12	#NUM!	2.6E-04
3	3	UWOPS87_ctrl_hmg124h_9.JPG.dat	1111.8	165.8	1.0	0.11	#NUM!	3.4E-04
4	4	UWOPS87_ctrl_hmg124h_9.JPG.dat	1022.5	57.3	0.9	0.02	#NUM!	4.4E-06
YLL046C	YLL046C	UWOPS87_ctrl_hmg124h_9.JPG.dat	971.3	98.7	0.9	0.11	#NUM!	3.8E-04
YLL047W	YLL047W	UWOPS87_ctrl_hmg124h_9.JPG.dat	1182.0	109.1	1.1	0.11	#NUM!	2.7E-04
YLL048C	YLL048C	UWOPS87_ctrl_hmg124h_9.JPG.dat	1122.3	81.2	1.1	0.08	#NUM!	1.2E-04
YLL049W	YLL049W	UWOPS87_ctrl_hmg124h_9.JPG.dat	1053.5	277.4	1.0	0.25	#NUM!	4.4E-03
YLL051C	YLL051C	UWOPS87_ctrl_hmg124h_9.JPG.dat	987.5	159.9	0.9	0.14	#NUM!	1.0E-03
YLL052C	YLL052C	UWOPS87_ctrl_hmg124h_9.JPG.dat	1282.8	49.0	1.1	0.04	#NUM!	1.3E-05
YLL053C	YLL053C	UWOPS87_ctrl_hmg124h_9.JPG.dat	1084.8	34.5	0.9	0.03	#NUM!	1.2E-05
YLL054C	YLL054C	UWOPS87_ctrl_hmg124h_9.JPG.dat	1146.0	130.6	1.0	0.12	#NUM!	4.3E-04
YLL055W	YLL055W	UWOPS87_ctrl_hmg124h_9.JPG.dat	1191.0	33.7	1.0	0.03	#NUM!	4.3E-06
YLL056C	YLL056C	UWOPS87_ctrl_hmg124h_9.JPG.dat	1040.8	22.1	0.9	0.02	#NUM!	3.7E-06
YLL057C	YLL057C	UWOPS87_ctrl_hmg124h_9.JPG.dat	1056.5	37.9	1.0	0.04	#NUM!	1.3E-05
YLL058W	YLL058W	UWOPS87_ctrl_hmg124h_9.JPG.dat	1057.3	142.0	1.1	0.14	#NUM!	5.9E-04

YLL059C	YLL059C	UWOPS87_ctrl_hmg124h_9.JPG.dat	857.0	78.5	0.9	0.08	#NUM!	1.8E-04
YLL060C	YLL060C	UWOPS87_ctrl_hmg124h_9.JPG.dat	969.8	124.7	1.0	0.11	#NUM!	3.8E-04
YLL061W	YLL061W	UWOPS87_ctrl_hmg124h_9.JPG.dat	1027.0	73.5	1.0	0.06	#NUM!	6.0E-05
YLL062C	YLL062C	UWOPS87_ctrl_hmg124h_9.JPG.dat	1094.8	88.5	1.1	0.09	#NUM!	1.6E-04
YLL063C	YLL063C	UWOPS87_ctrl_hmg124h_9.JPG.dat	1066.0	81.1	1.0	0.02	#NUM!	1.8E-04
YLR001C	YLR001C	UWOPS87_ctrl_hmg124h_9.JPG.dat	1172.3	41.2	1.1	0.04	#NUM!	1.2E-05
YLR003C	YLR003C	UWOPS87_ctrl_hmg124h_9.JPG.dat	1289.8	52.2	1.2	0.05	#NUM!	1.9E-05
YLR004C	YLR004C	UWOPS87_ctrl_hmg124h_9.JPG.dat	1022.8	84.0	0.9	0.09	#NUM!	2.5E-04
YLR006C	YLR006C	UWOPS87_ctrl_hmg124h_9.JPG.dat	1053.5	89.8	1.0	0.00	#NUM!	1.4E-06
YLR011W	YLR011W	UWOPS87_ctrl_hmg124h_9.JPG.dat	1028.8	95.3	1.0	0.09	#NUM!	2.1E-04
YLR012C	YLR012C	UWOPS87_ctrl_hmg124h_9.JPG.dat	798.0	98.7	0.8	0.10	#NUM!	5.1E-04
YLR013W	YLR013W	UWOPS87_ctrl_hmg124h_9.JPG.dat	1065.5	105.7	1.0	0.04	#NUM!	4.2E-04
YLR014C	YLR014C	UWOPS87_ctrl_hmg124h_9.JPG.dat	1145.5	137.9	1.1	0.14	#NUM!	4.9E-04
YLR015W	YLR015W	UWOPS87_ctrl_hmg124h_9.JPG.dat	1161.5	25.0	1.1	0.03	#NUM!	4.4E-06
YLR016C	YLR016C	UWOPS87_ctrl_hmg124h_9.JPG.dat	949.3	73.5	0.9	0.02	#NUM!	1.1E-04
YLR017W	YLR017W	UWOPS87_ctrl_hmg124h_9.JPG.dat	992.5	47.7	0.9	0.03	#NUM!	8.9E-06
YLR018C	YLR018C	UWOPS87_ctrl_hmg124h_9.JPG.dat	1013.5	27.8	0.9	0.04	#NUM!	2.5E-05
YLR019W	YLR019W	UWOPS87_ctrl_hmg124h_9.JPG.dat	1054.3	10.4	1.0	0.03	#NUM!	6.0E-06
YLR020C	YLR020C	UWOPS87_ctrl_hmg124h_9.JPG.dat	1171.8	89.7	1.1	0.06	#NUM!	5.4E-05
YLR021W	YLR021W	UWOPS87_ctrl_hmg124h_9.JPG.dat	1252.5	100.5	1.2	0.02	#NUM!	1.1E-04
YLR023C	YLR023C	UWOPS87_ctrl_hmg124h_9.JPG.dat	1202.8	162.4	1.2	0.17	#NUM!	8.4E-04
YLR024C	YLR024C	UWOPS87_ctrl_hmg124h_9.JPG.dat	921.0	52.1	1.0	0.05	#NUM!	4.9E-05
YLR028C	YLR028C	UWOPS87_ctrl_hmg124h_9.JPG.dat	1131.3	100.4	1.1	0.10	#NUM!	1.8E-04
YLR030W	YLR030W	UWOPS87_ctrl_hmg124h_9.JPG.dat	995.5	143.3	1.0	0.13	#NUM!	5.7E-04
YLR031W	YLR031W	UWOPS87_ctrl_hmg124h_9.JPG.dat	854.0	197.0	0.8	0.19	#NUM!	3.3E-03
YLR032W	YLR032W	UWOPS87_ctrl_hmg124h_9.JPG.dat	1073.3	52.8	1.0	0.05	#NUM!	2.6E-05
YLR034C	YLR034C	UWOPS87_ctrl_hmg124h_9.JPG.dat	1115.3	33.9	1.0	0.03	#NUM!	4.1E-06
YLR035C	YLR035C	UWOPS87_ctrl_hmg124h_9.JPG.dat	1048.0	29.6	1.0	0.03	#NUM!	6.7E-06
YLR036C	YLR036C	UWOPS87_ctrl_hmg124h_9.JPG.dat	1005.5	55.4	0.9	0.06	#NUM!	6.3E-05
YLR037C	YLR037C	UWOPS87_ctrl_hmg124h_9.JPG.dat	1229.0	25.9	1.1	0.00	#NUM!	4.4E-07
YLR038C	YLR038C	UWOPS87_ctrl_hmg124h_9.JPG.dat	1315.3	20.6	1.2	0.01	#NUM!	2.2E-07
YLR039C	YLR039C	UWOPS87_ctrl_hmg124h_9.JPG.dat	948.3	17.2	0.9	0.01	#NUM!	8.1E-07
YLR040C	YLR040C	UWOPS87_ctrl_hmg124h_9.JPG.dat	923.3	57.6	0.9	0.01	#NUM!	6.6E-05
YLR041W	YLR041W	UWOPS87_ctrl_hmg124h_9.JPG.dat	983.8	45.0	1.0	0.05	#NUM!	3.0E-05
YLR042C	YLR042C	UWOPS87_ctrl_hmg124h_9.JPG.dat	916.5	48.0	0.9	0.04	#NUM!	3.0E-05
YLR043C	YLR043C	UWOPS87_ctrl_hmg124h_9.JPG.dat	1087.8	21.7	1.0	0.00	#NUM!	5.6E-06
YLR044C	YLR044C	UWOPS87_ctrl_hmg124h_9.JPG.dat	1116.5	29.0	1.1	0.03	#NUM!	4.6E-06
YLR046C	YLR046C	UWOPS87_ctrl_hmg124h_9.JPG.dat	1013.0	42.5	0.9	0.04	#NUM!	2.4E-05
YLR047C	YLR047C	UWOPS87_ctrl_hmg124h_9.JPG.dat	1098.5	25.5	1.0	0.01	#NUM!	1.9E-05
YLR048W	YLR048W	UWOPS87_ctrl_hmg124h_9.JPG.dat	1199.5	26.9	1.1	0.03	#NUM!	5.5E-06
YLR049C	YLR049C	UWOPS87_ctrl_hmg124h_9.JPG.dat	1019.8	77.4	0.9	0.07	#NUM!	1.2E-04
YLR050C	YLR050C	UWOPS87_ctrl_hmg124h_9.JPG.dat	945.5	78.0	0.9	0.03	#NUM!	3.4E-04
YLR053C	YLR053C	UWOPS87_ctrl_hmg124h_9.JPG.dat	1000.3	80.3	1.0	0.08	#NUM!	1.2E-04
YLR054C	YLR054C	UWOPS87_ctrl_hmg124h_9.JPG.dat	939.8	63.9	1.0	0.07	#NUM!	9.2E-05
YLR055C	YLR055C	UWOPS87_ctrl_hmg124h_9.JPG.dat	1112.0	81.6	1.1	0.08	#NUM!	9.1E-05
YLR056W	YLR056W	UWOPS87_ctrl_hmg124h_9.JPG.dat	981.8	125.5	1.0	0.11	#NUM!	4.4E-04
YLR057W	YLR057W	UWOPS87_ctrl_hmg124h_9.JPG.dat	1039.5	39.5	1.0	0.05	#NUM!	2.9E-05
YLR058C	YLR058C	UWOPS87_ctrl_hmg124h_9.JPG.dat	998.0	14.3	1.0	0.02	#NUM!	3.6E-06
YLR059C	YLR059C	UWOPS87_ctrl_hmg124h_9.JPG.dat	913.0	127.1	0.8	0.11	#NUM!	6.3E-04
YLR061W	YLR061W	UWOPS87_ctrl_hmg124h_9.JPG.dat	1231.8	59.1	1.1	0.06	#NUM!	4.5E-05
YLR062C	YLR062C	UWOPS87_ctrl_hmg124h_9.JPG.dat	1035.3	116.1	0.9	0.03	#NUM!	4.3E-04
YLR063W	YLR063W	UWOPS87_ctrl_hmg124h_9.JPG.dat	940.8	629.1	1.1	0.06	#NUM!	9.2E-04
YLR064W	YLR064W	UWOPS87_ctrl_hmg124h_9.JPG.dat	1086.5	119.7	1.0	0.13	#NUM!	5.0E-04
YLR065C	YLR065C	UWOPS87_ctrl_hmg124h_9.JPG.dat	778.8	88.8	0.8	0.09	#NUM!	4.1E-04
YLR070C	YLR070C	UWOPS87_ctrl_hmg124h_9.JPG.dat	962.5	50.2	1.0	0.05	#NUM!	4.8E-05
YLR072W	YLR072W	UWOPS87_ctrl_hmg124h_9.JPG.dat	1007.3	56.4	1.0	0.06	#NUM!	5.6E-05
YLR073C	YLR073C	UWOPS87_ctrl_hmg124h_9.JPG.dat	932.3	159.1	0.9	0.15	#NUM!	1.1E-03
YLR077W	YLR077W	UWOPS87_ctrl_hmg124h_9.JPG.dat	1001.8	68.3	1.0	0.08	#NUM!	1.2E-04
YLR079W	YLR079W	UWOPS87_ctrl_hmg124h_9.JPG.dat	1004.3	102.9	1.0	0.10	#NUM!	2.6E-04
YLR080W	YLR080W	UWOPS87_ctrl_hmg124h_9.JPG.dat	1080.0	66.0	1.0	0.06	#NUM!	6.7E-05
YLR081W	YLR081W	UWOPS87_ctrl_hmg124h_9.JPG.dat	1103.5	122.3	1.0	0.11	#NUM!	2.8E-04
YLR082C	YLR082C	UWOPS87_ctrl_hmg124h_9.JPG.dat	1223.0	125.8	1.3	0.01	#NUM!	1.3E-05
YLR083C	YLR083C	UWOPS87_ctrl_hmg124h_9.JPG.dat	1177.0	135.3	1.2	0.01	#NUM!	2.3E-05
YLR084C	YLR084C	UWOPS87_ctrl_hmg124h_9.JPG.dat	974.5	35.5	0.9	0.00	#NUM!	1.2E-06
YLR085C	YLR085C	UWOPS87_ctrl_hmg124h_9.JPG.dat	904.3	60.1	0.8	0.05	#NUM!	5.4E-05
YLR087C	YLR087C	UWOPS87_ctrl_hmg124h_9.JPG.dat	935.0	116.3	0.9	0.02	#NUM!	2.1E-04
YLR089C	YLR089C	UWOPS87_ctrl_hmg124h_9.JPG.dat	1033.0	80.5	1.0	0.06	#NUM!	7.9E-05
YLR090W	YLR090W	UWOPS87_ctrl_hmg124h_9.JPG.dat	1014.8	162.3	1.0	0.15	#NUM!	1.0E-03
YLR091W	YLR091W	UWOPS87_ctrl_hmg124h_9.JPG.dat	1049.5	78.0	0.9	0.01	#NUM!	5.3E-05
YLR092W	YLR092W	UWOPS87_ctrl_hmg124h_9.JPG.dat	1232.8	82.3	1.2	0.07	#NUM!	6.1E-05

YLR093C	YLR093C	UWOPS87_ctrl_hmg124h_9.JPG.dat	1086.8	73.3	1.0	0.07	#NUM!	1.1E-04
YLR094C	YLR094C	UWOPS87_ctrl_hmg124h_9.JPG.dat	1212.8	63.9	1.0	0.06	#NUM!	4.1E-05
YLR095C	YLR095C	UWOPS87_ctrl_hmg124h_9.JPG.dat	1069.8	83.5	0.9	0.08	#NUM!	1.5E-04
YLR096W	YLR096W	UWOPS87_ctrl_hmg124h_9.JPG.dat	1095.5	125.0	1.0	0.11	#NUM!	4.2E-04
YLR097C	YLR097C	UWOPS87_ctrl_hmg124h_9.JPG.dat	1215.0	106.3	1.1	0.10	#NUM!	2.2E-04
YLR098C	YLR098C	UWOPS87_ctrl_hmg124h_9.JPG.dat	1227.5	97.3	1.2	0.03	#NUM!	1.6E-04
YLR099C	YLR099C	UWOPS87_ctrl_hmg124h_9.JPG.dat	1181.5	135.8	1.2	0.16	#NUM!	5.7E-04
YLR102C	YLR102C	UWOPS87_ctrl_hmg124h_9.JPG.dat	959.8	80.9	1.0	0.02	#NUM!	7.8E-05
YLR104W	YLR104W	UWOPS87_ctrl_hmg124h_9.JPG.dat	921.0	68.1	0.9	0.06	#NUM!	6.9E-05
YLR107W	YLR107W	UWOPS87_ctrl_hmg124h_9.JPG.dat	661.8	119.9	0.6	0.12	#NUM!	1.9E-03
YLR108C	YLR108C	UWOPS87_ctrl_hmg124h_9.JPG.dat	861.3	34.6	0.9	0.04	#NUM!	3.1E-05
YLR109W	YLR109W	UWOPS87_ctrl_hmg124h_9.JPG.dat	1079.3	87.4	1.0	0.07	#NUM!	8.3E-05
YLR110C	YLR110C	UWOPS87_ctrl_hmg124h_9.JPG.dat	1053.0	109.0	1.0	0.10	#NUM!	3.0E-04
YLR111W	YLR111W	UWOPS87_ctrl_hmg124h_9.JPG.dat	1155.8	40.2	1.0	0.02	#NUM!	9.2E-05
YLR112W	YLR112W	UWOPS87_ctrl_hmg124h_9.JPG.dat	1034.5	114.1	0.9	0.09	#NUM!	2.3E-04
YLR113W	YLR113W	UWOPS87_ctrl_hmg124h_9.JPG.dat	1091.8	154.2	1.0	0.02	#NUM!	2.0E-04
YLR114C	YLR114C	UWOPS87_ctrl_hmg124h_9.JPG.dat	1004.5	30.8	1.0	0.04	#NUM!	1.7E-05
YLR118C	YLR118C	UWOPS87_ctrl_hmg124h_9.JPG.dat	920.5	159.0	1.0	0.17	#NUM!	1.5E-03
YLR119W	YLR119W	UWOPS87_ctrl_hmg124h_9.JPG.dat	1122.5	117.3	1.2	0.11	#NUM!	2.4E-04
YLR120C	YLR120C	UWOPS87_ctrl_hmg124h_9.JPG.dat	800.8	79.3	0.8	0.10	#NUM!	4.5E-04
YLR121C	YLR121C	UWOPS87_ctrl_hmg124h_9.JPG.dat	1074.0	59.6	1.0	0.07	#NUM!	7.0E-05
YLR122C	YLR122C	UWOPS87_ctrl_hmg124h_9.JPG.dat	944.3	105.7	0.9	0.10	#NUM!	3.4E-04
YLR123C	YLR123C	UWOPS87_ctrl_hmg124h_9.JPG.dat	1004.3	47.8	1.0	0.02	#NUM!	8.2E-05
YLR124W	YLR124W	UWOPS87_ctrl_hmg124h_9.JPG.dat	1063.5	73.7	0.9	0.02	#NUM!	1.0E-04
YLR125W	YLR125W	UWOPS87_ctrl_hmg124h_9.JPG.dat	1087.3	40.0	1.0	0.02	#NUM!	9.9E-07
YLR126C	YLR126C	UWOPS87_ctrl_hmg124h_9.JPG.dat	1075.5	60.5	1.0	0.07	#NUM!	9.1E-05
YLR128W	YLR128W	UWOPS87_ctrl_hmg124h_9.JPG.dat	1095.8	160.5	1.0	0.14	#NUM!	7.2E-04
YLR130C	YLR130C	UWOPS87_ctrl_hmg124h_9.JPG.dat	997.3	155.4	1.0	0.14	#NUM!	8.6E-04
YLR131C	YLR131C	UWOPS87_ctrl_hmg124h_9.JPG.dat	1026.0	63.0	1.1	0.02	#NUM!	1.6E-04
YLR133W	YLR133W	UWOPS87_ctrl_hmg124h_9.JPG.dat	848.8	67.5	0.9	0.06	#NUM!	1.1E-04
YLR134W	YLR134W	UWOPS87_ctrl_hmg124h_9.JPG.dat	1205.0	93.4	1.2	0.10	#NUM!	1.5E-04
YLR135W	YLR135W	UWOPS87_ctrl_hmg124h_9.JPG.dat	892.8	66.6	0.9	0.00	#NUM!	4.7E-06
YLR136C	YLR136C	UWOPS87_ctrl_hmg124h_9.JPG.dat	1213.0	105.2	1.2	0.02	#NUM!	7.8E-05
YLR137W	YLR137W	UWOPS87_ctrl_hmg124h_9.JPG.dat	970.3	171.7	1.0	0.05	#NUM!	9.5E-04
YLR138W	YLR138W	UWOPS87_ctrl_hmg124h_9.JPG.dat	884.8	593.7	1.1	0.08	#NUM!	1.6E-03
YLR142W	YLR142W	UWOPS87_ctrl_hmg124h_9.JPG.dat	1097.0	73.2	1.0	0.08	#NUM!	1.2E-04
YLR143W	YLR143W	UWOPS87_ctrl_hmg124h_9.JPG.dat	1090.3	65.4	1.0	0.05	#NUM!	3.3E-05
YLR144C	YLR144C	UWOPS87_ctrl_hmg124h_9.JPG.dat	1071.8	212.4	1.0	0.20	#NUM!	2.1E-03
YLR146C	YLR146C	UWOPS87_ctrl_hmg124h_9.JPG.dat	972.3	40.3	0.9	0.01	#NUM!	2.9E-05
YLR149C	YLR149C	UWOPS87_ctrl_hmg124h_9.JPG.dat	985.5	55.8	1.0	0.06	#NUM!	4.7E-05
YLR150W	YLR150W	UWOPS87_ctrl_hmg124h_9.JPG.dat	1003.3	54.8	1.0	0.02	#NUM!	1.1E-04
YLR151C	YLR151C	UWOPS87_ctrl_hmg124h_9.JPG.dat	939.5	69.1	0.9	0.07	#NUM!	1.3E-04
YLR152C	YLR152C	UWOPS87_ctrl_hmg124h_9.JPG.dat	1200.0	51.2	1.1	0.06	#NUM!	3.3E-05
YLR154C	YLR154C	UWOPS87_ctrl_hmg124h_9.JPG.dat	1126.5	16.3	1.1	0.02	#NUM!	1.2E-06
YLR164W	YLR164W	UWOPS87_ctrl_hmg124h_9.JPG.dat	1051.0	46.6	1.0	0.05	#NUM!	3.0E-05
YLR165C	YLR165C	UWOPS87_ctrl_hmg124h_9.JPG.dat	1021.8	45.1	0.9	0.04	#NUM!	2.7E-05
YLR168C	YLR168C	UWOPS87_ctrl_hmg124h_9.JPG.dat	1194.0	93.9	1.1	0.09	#NUM!	1.6E-04
YLR169W	YLR169W	UWOPS87_ctrl_hmg124h_9.JPG.dat	1126.5	117.0	1.1	0.04	#NUM!	3.7E-04
YLR170C	YLR170C	UWOPS87_ctrl_hmg124h_9.JPG.dat	1054.5	45.9	1.0	0.05	#NUM!	3.6E-05
YLR171W	YLR171W	UWOPS87_ctrl_hmg124h_9.JPG.dat	1131.0	46.4	1.1	0.04	#NUM!	1.2E-05
YLR172C	YLR172C	UWOPS87_ctrl_hmg124h_9.JPG.dat	911.5	37.4	0.9	0.01	#NUM!	1.1E-05
YLR173W	YLR173W	UWOPS87_ctrl_hmg124h_9.JPG.dat	977.8	108.9	1.0	0.03	#NUM!	3.0E-04
YLR174W	YLR174W	UWOPS87_ctrl_hmg124h_9.JPG.dat	1198.5	55.6	1.2	0.05	#NUM!	2.2E-05
YLR176C	YLR176C	UWOPS87_ctrl_hmg124h_9.JPG.dat	1025.8	27.3	1.0	0.03	#NUM!	1.1E-05
YLR177W	YLR177W	UWOPS87_ctrl_hmg124h_9.JPG.dat	1004.0	37.4	1.0	0.01	#NUM!	4.1E-05
YLR178C	YLR178C	UWOPS87_ctrl_hmg124h_9.JPG.dat	1089.3	66.8	1.0	0.06	#NUM!	4.6E-05
YLR179C	YLR179C	UWOPS87_ctrl_hmg124h_9.JPG.dat	1238.3	52.5	1.1	0.03	#NUM!	4.7E-06
YLR180W	YLR180W	UWOPS87_ctrl_hmg124h_9.JPG.dat	1140.5	106.9	1.0	0.09	#NUM!	1.6E-04
YLR181C	YLR181C	UWOPS87_ctrl_hmg124h_9.JPG.dat	1321.3	16.0	1.2	0.03	#NUM!	6.2E-06
YLR182W	YLR182W	UWOPS87_ctrl_hmg124h_9.JPG.dat	125.5	251.0	0.0	0.00	#NUM!	
YLR183C	YLR183C	UWOPS87_ctrl_hmg124h_9.JPG.dat	1008.0	77.7	1.0	0.06	#NUM!	8.3E-05
YLR184W	YLR184W	UWOPS87_ctrl_hmg124h_9.JPG.dat	612.3	41.7	0.6	0.04	#NUM!	7.6E-05
YLR185W	YLR185W	UWOPS87_ctrl_hmg124h_9.JPG.dat	593.3	49.5	0.6	0.05	#NUM!	1.9E-04
YLR187W	YLR187W	UWOPS87_ctrl_hmg124h_9.JPG.dat	964.3	69.2	1.0	0.06	#NUM!	7.5E-05
YLR188W	YLR188W	UWOPS87_ctrl_hmg124h_9.JPG.dat	1213.8	88.8	1.2	0.09	#NUM!	1.3E-04
YLR189C	YLR189C	UWOPS87_ctrl_hmg124h_9.JPG.dat	982.3	36.2	1.0	0.04	#NUM!	1.3E-05
YLR190W	YLR190W	UWOPS87_ctrl_hmg124h_9.JPG.dat	977.3	130.7	1.0	0.12	#NUM!	5.0E-04
YLR191W	YLR191W	UWOPS87_ctrl_hmg124h_9.JPG.dat	1257.0	44.1	1.2	0.06	#NUM!	3.1E-05
YLR192C	YLR192C	UWOPS87_ctrl_hmg124h_9.JPG.dat	1055.5	60.7	1.0	0.06	#NUM!	5.6E-05
YLR193C	YLR193C	UWOPS87_ctrl_hmg124h_9.JPG.dat	1099.5	118.6	1.0	0.03	#NUM!	2.6E-04

YLR194C	YLR194C	UWOPS87_ctrl_hmg124h_9.JPG.dat	1029.5	43.5	1.0	0.04	#NUM!	2.0E-05
YLR199C	YLR199C	UWOPS87_ctrl_hmg124h_9.JPG.dat	1148.5	110.9	1.1	0.09	#NUM!	1.9E-04
YLR200W	YLR200W	UWOPS87_ctrl_hmg124h_9.JPG.dat	923.5	104.5	0.9	0.09	#NUM!	2.5E-04
YLR205C	YLR205C	UWOPS87_ctrl_hmg124h_9.JPG.dat	1123.5	124.2	1.1	0.13	#NUM!	5.3E-04
YLR206W	YLR206W	UWOPS87_ctrl_hmg124h_9.JPG.dat	931.8	84.4	0.9	0.07	#NUM!	1.4E-04
YLR207W	YLR207W	UWOPS87_ctrl_hmg124h_9.JPG.dat	1068.8	103.7	1.0	0.11	#NUM!	3.0E-04
YLR209C	YLR209C	UWOPS87_ctrl_hmg124h_9.JPG.dat	1055.5	91.5	1.0	0.01	#NUM!	1.8E-05
YLR210W	YLR210W	UWOPS87_ctrl_hmg124h_9.JPG.dat	1227.8	118.6	1.1	0.12	#NUM!	3.1E-04
YLR211C	YLR211C	UWOPS87_ctrl_hmg124h_9.JPG.dat	1132.5	90.2	1.0	0.09	#NUM!	1.7E-04
YLR213C	YLR213C	UWOPS87_ctrl_hmg124h_9.JPG.dat	1314.8	209.3	1.2	0.19	#NUM!	9.3E-04
YLR214W	YLR214W	UWOPS87_ctrl_hmg124h_9.JPG.dat	1106.8	67.6	1.0	0.06	#NUM!	6.3E-05
YLR216C	YLR216C	UWOPS87_ctrl_hmg124h_9.JPG.dat	1057.5	48.7	1.0	0.05	#NUM!	3.2E-05
YLR217W	YLR217W	UWOPS87_ctrl_hmg124h_9.JPG.dat	1132.3	100.8	1.2	0.12	#NUM!	2.7E-04
YLR218C	YLR218C	UWOPS87_ctrl_hmg124h_9.JPG.dat	1127.5	171.5	1.2	0.18	#NUM!	9.8E-04
YLR219W	YLR219W	UWOPS87_ctrl_hmg124h_9.JPG.dat	983.8	187.2	1.0	0.18	#NUM!	1.7E-03
YLR220W	YLR220W	UWOPS87_ctrl_hmg124h_9.JPG.dat	1109.3	117.5	1.1	0.12	#NUM!	3.5E-04
YLR221C	YLR221C	UWOPS87_ctrl_hmg124h_9.JPG.dat	921.3	54.5	0.9	0.05	#NUM!	3.9E-05
YLR224W	YLR224W	UWOPS87_ctrl_hmg124h_9.JPG.dat	1039.8	43.4	1.0	0.01	#NUM!	3.3E-05
YLR225C	YLR225C	UWOPS87_ctrl_hmg124h_9.JPG.dat	1169.0	25.0	1.1	0.03	#NUM!	5.5E-06
YLR228C	YLR228C	UWOPS87_ctrl_hmg124h_9.JPG.dat	1151.5	25.7	1.0	0.01	#NUM!	8.3E-06
YLR231C	YLR231C	UWOPS87_ctrl_hmg124h_9.JPG.dat	924.8	66.7	0.9	0.06	#NUM!	1.0E-04
YLR232W	YLR232W	UWOPS87_ctrl_hmg124h_9.JPG.dat	1252.5	70.2	1.1	0.07	#NUM!	5.7E-05
YLR233C	YLR233C	UWOPS87_ctrl_hmg124h_9.JPG.dat	0.0	0.0	0.0	0.00	#NUM!	
YLR234W	YLR234W	UWOPS87_ctrl_hmg124h_9.JPG.dat	894.3	43.6	0.9	0.05	#NUM!	3.5E-05
YLR235C	YLR235C	UWOPS87_ctrl_hmg124h_9.JPG.dat	1152.3	58.5	1.2	0.06	#NUM!	2.9E-05
YLR236C	YLR236C	UWOPS87_ctrl_hmg124h_9.JPG.dat	953.8	34.4	0.9	0.04	#NUM!	2.5E-05
YLR237W	YLR237W	UWOPS87_ctrl_hmg124h_9.JPG.dat	1107.5	134.3	1.1	0.13	#NUM!	5.2E-04
YLR239C	YLR239C	UWOPS87_ctrl_hmg124h_9.JPG.dat	610.0	411.6	0.6	0.40	#NUM!	5.9E-02
YLR241W	YLR241W	UWOPS87_ctrl_hmg124h_9.JPG.dat	984.3	26.7	1.0	0.03	#NUM!	8.2E-06
YLR242C	YLR242C	UWOPS87_ctrl_hmg124h_9.JPG.dat	87.5	175.0	0.0	0.00	#NUM!	
YLR246W	YLR246W	UWOPS87_ctrl_hmg124h_9.JPG.dat	1217.3	25.3	1.1	0.03	#NUM!	3.6E-06
YLR247C	YLR247C	UWOPS87_ctrl_hmg124h_9.JPG.dat	983.8	72.4	0.9	0.07	#NUM!	1.4E-04
YLR248W	YLR248W	UWOPS87_ctrl_hmg124h_9.JPG.dat	1079.3	70.6	1.0	0.05	#NUM!	4.9E-05
YLR250W	YLR250W	UWOPS87_ctrl_hmg124h_9.JPG.dat	1088.8	99.0	1.0	0.09	#NUM!	2.1E-04
YLR251W	YLR251W	UWOPS87_ctrl_hmg124h_9.JPG.dat	1037.8	144.4	0.9	0.03	#NUM!	3.1E-04
YLR252W	YLR252W	UWOPS87_ctrl_hmg124h_9.JPG.dat	1094.3	57.0	1.2	0.02	#NUM!	5.9E-05
YLR253W	YLR253W	UWOPS87_ctrl_hmg124h_9.JPG.dat	1028.8	131.9	1.0	0.13	#NUM!	6.1E-04
YLR254C	YLR254C	UWOPS87_ctrl_hmg124h_9.JPG.dat	988.3	42.1	1.0	0.05	#NUM!	3.2E-05
YLR255C	YLR255C	UWOPS87_ctrl_hmg124h_9.JPG.dat	964.5	45.3	0.9	0.04	#NUM!	2.0E-05
YLR257W	YLR257W	UWOPS87_ctrl_hmg124h_9.JPG.dat	1052.5	89.9	1.1	0.02	#NUM!	7.1E-05
YLR258W	YLR258W	UWOPS87_ctrl_hmg124h_9.JPG.dat	1054.3	91.4	1.0	0.07	#NUM!	1.0E-04
YLR261C	YLR261C	UWOPS87_ctrl_hmg124h_9.JPG.dat	1143.8	39.3	1.0	0.05	#NUM!	2.6E-05
YLR262C	YLR262C	UWOPS87_ctrl_hmg124h_9.JPG.dat	942.5	43.7	0.9	0.05	#NUM!	4.7E-05
YLR262C-A	YLR262C-A	UWOPS87_ctrl_hmg124h_9.JPG.dat	989.5	21.4	0.9	0.01	#NUM!	2.8E-07
YLR263W	YLR263W	UWOPS87_ctrl_hmg124h_9.JPG.dat	993.3	114.2	0.9	0.10	#NUM!	3.4E-04
YLR264W	YLR264W	UWOPS87_ctrl_hmg124h_9.JPG.dat	545.8	337.7	0.5	0.34	#NUM!	5.0E-02
YLR265C	YLR265C	UWOPS87_ctrl_hmg124h_9.JPG.dat	910.8	88.4	1.0	0.03	#NUM!	3.0E-04
YLR266C	YLR266C	UWOPS87_ctrl_hmg124h_9.JPG.dat	1042.8	69.9	1.0	0.07	#NUM!	9.2E-05
YLR267W	YLR267W	UWOPS87_ctrl_hmg124h_9.JPG.dat	984.0	104.4	1.0	0.09	#NUM!	2.5E-04
YLR268W	YLR268W	UWOPS87_ctrl_hmg124h_9.JPG.dat	1171.8	64.2	1.1	0.07	#NUM!	7.2E-05
YLR269C	YLR269C	UWOPS87_ctrl_hmg124h_9.JPG.dat	1219.5	73.3	1.1	0.08	#NUM!	9.8E-05
YLR271W	YLR271W	UWOPS87_ctrl_hmg124h_9.JPG.dat	1137.0	114.1	1.0	0.09	#NUM!	1.9E-04
YLR273C	YLR273C	UWOPS87_ctrl_hmg124h_9.JPG.dat	1086.3	84.8	1.0	0.08	#NUM!	1.5E-04
YLR278C	YLR278C	UWOPS87_ctrl_hmg124h_9.JPG.dat	1051.0	67.3	1.0	0.07	#NUM!	1.1E-04
YLR279W	YLR279W	UWOPS87_ctrl_hmg124h_9.JPG.dat	1055.3	103.3	0.9	0.08	#NUM!	1.7E-04
YLR280C	YLR280C	UWOPS87_ctrl_hmg124h_9.JPG.dat	1110.3	71.0	1.0	0.06	#NUM!	4.1E-05
YLR281C	YLR281C	UWOPS87_ctrl_hmg124h_9.JPG.dat	1009.8	188.6	0.9	0.01	#NUM!	9.1E-05
YLR282C	YLR282C	UWOPS87_ctrl_hmg124h_9.JPG.dat	1025.8	95.1	1.0	0.04	#NUM!	4.2E-04
YLR283W	YLR283W	UWOPS87_ctrl_hmg124h_9.JPG.dat	1039.8	139.3	1.0	0.14	#NUM!	6.8E-04
YLR284C	YLR284C	UWOPS87_ctrl_hmg124h_9.JPG.dat	1037.5	231.8	1.0	0.22	#NUM!	2.5E-03
YLR285W	YLR285W	UWOPS87_ctrl_hmg124h_9.JPG.dat	963.8	115.2	0.9	0.11	#NUM!	4.6E-04
YLR286C	YLR286C	UWOPS87_ctrl_hmg124h_9.JPG.dat	1163.0	103.9	1.1	0.10	#NUM!	1.9E-04
YLR287C	YLR287C	UWOPS87_ctrl_hmg124h_9.JPG.dat	1219.8	28.6	1.1	0.01	#NUM!	1.2E-05
YLR287C-A	YLR287C-A	UWOPS87_ctrl_hmg124h_9.JPG.dat	1273.8	26.7	1.1	0.01	#NUM!	2.1E-05
YLR289W	YLR289W	UWOPS87_ctrl_hmg124h_9.JPG.dat	1160.5	53.8	1.1	0.05	#NUM!	2.4E-05
YLR290C	YLR290C	UWOPS87_ctrl_hmg124h_9.JPG.dat	1279.5	45.0	1.2	0.05	#NUM!	2.1E-05
YLR292C	YLR292C	UWOPS87_ctrl_hmg124h_9.JPG.dat	1007.0	114.1	0.9	0.04	#NUM!	6.5E-04
YLR294C	YLR294C	UWOPS87_ctrl_hmg124h_9.JPG.dat	1005.5	48.2	1.0	0.01	#NUM!	6.9E-05
YLR296W	YLR296W	UWOPS87_ctrl_hmg124h_9.JPG.dat	997.0	58.9	1.1	0.06	#NUM!	5.2E-05
YLR297W	YLR297W	UWOPS87_ctrl_hmg124h_9.JPG.dat	978.8	63.1	1.0	0.06	#NUM!	5.8E-05

YLR299W	YLR299W	UWOPS87_ctrl_hmg124h_9.JPG.dat	993.0	93.7	1.0	0.11	#NUM!	3.0E-04
YLR300W	YLR300W	UWOPS87_ctrl_hmg124h_9.JPG.dat	1025.8	61.3	1.0	0.06	#NUM!	4.4E-05
YLR303W	YLR303W	UWOPS87_ctrl_hmg124h_9.JPG.dat	1036.0	66.9	1.0	0.07	#NUM!	7.3E-05
YLR306W	YLR306W	UWOPS87_ctrl_hmg124h_9.JPG.dat	1095.3	40.4	1.0	0.01	#NUM!	5.4E-05
YLR307W	YLR307W	UWOPS87_ctrl_hmg124h_9.JPG.dat	1092.8	89.5	1.0	0.08	#NUM!	1.3E-04
YLR309C	YLR309C	UWOPS87_ctrl_hmg124h_9.JPG.dat	950.3	36.4	0.9	0.01	#NUM!	1.6E-05
YLR311C	YLR311C	UWOPS87_ctrl_hmg124h_9.JPG.dat	1032.0	106.3	0.9	0.03	#NUM!	3.4E-04
YLR312C	YLR312C	UWOPS87_ctrl_hmg124h_9.JPG.dat	1043.3	67.3	1.0	0.06	#NUM!	5.4E-05
YLR313C	YLR313C	UWOPS87_ctrl_hmg124h_9.JPG.dat	1090.3	166.2	1.1	0.16	#NUM!	9.7E-04
YLR315W	YLR315W	UWOPS87_ctrl_hmg124h_9.JPG.dat	958.0	32.5	1.0	0.01	#NUM!	1.2E-05
YLR318W	YLR318W	UWOPS87_ctrl_hmg124h_9.JPG.dat	1229.5	205.5	1.2	0.20	#NUM!	1.4E-03
YLR319C	YLR319C	UWOPS87_ctrl_hmg124h_9.JPG.dat	1056.5	38.3	1.0	0.04	#NUM!	1.8E-05
YLR320W	YLR320W	UWOPS87_ctrl_hmg124h_9.JPG.dat	974.8	44.2	0.9	0.04	#NUM!	2.1E-05
YLR324W	YLR324W	UWOPS87_ctrl_hmg124h_9.JPG.dat	950.0	69.7	0.9	0.02	#NUM!	1.1E-04
YLR325C	YLR325C	UWOPS87_ctrl_hmg124h_9.JPG.dat	1172.0	22.0	1.1	0.02	#NUM!	2.1E-06
YLR326W	YLR326W	UWOPS87_ctrl_hmg124h_9.JPG.dat	1050.5	93.2	0.9	0.08	#NUM!	1.7E-04
YLR327C	YLR327C	UWOPS87_ctrl_hmg124h_9.JPG.dat	1160.0	106.7	1.1	0.11	#NUM!	2.7E-04
YLR328W	YLR328W	UWOPS87_ctrl_hmg124h_9.JPG.dat	969.3	26.4	0.9	0.02	#NUM!	1.4E-06
YLR329W	YLR329W	UWOPS87_ctrl_hmg124h_9.JPG.dat	1003.3	75.1	0.9	0.06	#NUM!	7.9E-05
YLR330W	YLR330W	UWOPS87_ctrl_hmg124h_9.JPG.dat	1147.8	35.1	1.1	0.06	#NUM!	4.7E-05
YLR332W	YLR332W	UWOPS87_ctrl_hmg124h_9.JPG.dat	924.3	78.2	1.0	0.09	#NUM!	2.0E-04
YLR333C	YLR333C	UWOPS87_ctrl_hmg124h_9.JPG.dat	911.5	52.3	0.9	0.04	#NUM!	3.0E-05
YLR334C	YLR334C	UWOPS87_ctrl_hmg124h_9.JPG.dat	1138.3	157.5	1.2	0.16	#NUM!	7.9E-04
YLR335W	YLR335W	UWOPS87_ctrl_hmg124h_9.JPG.dat	1111.5	48.3	1.1	0.05	#NUM!	2.6E-05
YLR337C	YLR337C	UWOPS87_ctrl_hmg124h_9.JPG.dat	1075.3	448.9	1.1	0.45	#NUM!	1.8E-02
YLR338W	YLR338W	UWOPS87_ctrl_hmg124h_9.JPG.dat	212.3	417.9	0.0	0.01	#NUM!	4.2E-01
YLR341W	YLR341W	UWOPS87_ctrl_hmg124h_9.JPG.dat	954.3	154.3	0.9	0.14	#NUM!	1.2E-03
YLR342W	YLR342W	UWOPS87_ctrl_hmg124h_9.JPG.dat	1084.8	27.3	1.0	0.02	#NUM!	3.7E-06
YLR343W	YLR343W	UWOPS87_ctrl_hmg124h_9.JPG.dat	1104.0	62.1	1.0	0.05	#NUM!	4.4E-05
YLR344W	YLR344W	UWOPS87_ctrl_hmg124h_9.JPG.dat	1172.8	131.8	1.1	0.13	#NUM!	4.3E-04
YLR345W	YLR345W	UWOPS87_ctrl_hmg124h_9.JPG.dat	1101.5	118.3	1.0	0.04	#NUM!	4.1E-04
YLR346C	YLR346C	UWOPS87_ctrl_hmg124h_9.JPG.dat	885.0	33.5	0.9	0.00	#NUM!	5.7E-07
YLR348C	YLR348C	UWOPS87_ctrl_hmg124h_9.JPG.dat	981.3	96.2	1.0	0.09	#NUM!	2.1E-04
YLR349W	YLR349W	UWOPS87_ctrl_hmg124h_9.JPG.dat	1128.8	204.8	1.2	0.03	#NUM!	1.9E-04
YLR350W	YLR350W	UWOPS87_ctrl_hmg124h_9.JPG.dat	1198.0	73.8	1.2	0.02	#NUM!	8.6E-05
YLR351C	YLR351C	UWOPS87_ctrl_hmg124h_9.JPG.dat	997.5	67.9	1.0	0.07	#NUM!	8.6E-05
YLR352W	YLR352W	UWOPS87_ctrl_hmg124h_9.JPG.dat	1144.5	79.5	1.1	0.06	#NUM!	4.0E-05
YLR353W	YLR353W	UWOPS87_ctrl_hmg124h_9.JPG.dat	1222.3	43.6	1.1	0.04	#NUM!	1.1E-05
YLR354C	YLR354C	UWOPS87_ctrl_hmg124h_9.JPG.dat	1159.0	45.1	1.0	0.04	#NUM!	1.2E-05
YLR356W	YLR356W	UWOPS87_ctrl_hmg124h_9.JPG.dat	1173.5	49.5	1.0	0.03	#NUM!	6.6E-06
YLR357W	YLR357W	UWOPS87_ctrl_hmg124h_9.JPG.dat	155.3	195.5	0.1	0.18	#NUM!	2.1E-01
YLR360W	YLR360W	UWOPS87_ctrl_hmg124h_9.JPG.dat	958.0	79.6	0.9	0.08	#NUM!	1.7E-04
YLR361C	YLR361C	UWOPS87_ctrl_hmg124h_9.JPG.dat	903.8	58.6	0.9	0.05	#NUM!	5.1E-05
YLR362W	YLR362W	UWOPS87_ctrl_hmg124h_9.JPG.dat	175.8	351.5	0.0	0.00	#NUM!	
YLR363C	YLR363C	UWOPS87_ctrl_hmg124h_9.JPG.dat	1044.3	153.0	1.1	0.16	#NUM!	1.0E-03
YLR364W	YLR364W	UWOPS87_ctrl_hmg124h_9.JPG.dat	1213.5	36.8	1.2	0.04	#NUM!	1.4E-05
YLR365W	YLR365W	UWOPS87_ctrl_hmg124h_9.JPG.dat	995.8	20.6	1.0	0.03	#NUM!	9.6E-06
YLR366W	YLR366W	UWOPS87_ctrl_hmg124h_9.JPG.dat	1076.0	37.5	1.0	0.03	#NUM!	4.0E-06
YLR367W	YLR367W	UWOPS87_ctrl_hmg124h_9.JPG.dat	1083.0	33.6	1.0	0.03	#NUM!	1.1E-05
YLR368W	YLR368W	UWOPS87_ctrl_hmg124h_9.JPG.dat	1099.8	10.0	1.0	0.02	#NUM!	3.9E-06
YLR370C	YLR370C	UWOPS87_ctrl_hmg124h_9.JPG.dat	1214.3	48.3	1.1	0.03	#NUM!	4.6E-06
YLR371W	YLR371W	UWOPS87_ctrl_hmg124h_9.JPG.dat	1096.0	110.5	1.1	0.03	#NUM!	1.9E-04
YLR372W	YLR372W	UWOPS87_ctrl_hmg124h_9.JPG.dat	1022.3	61.9	1.0	0.05	#NUM!	2.8E-05
YLR373C	YLR373C	UWOPS87_ctrl_hmg124h_9.JPG.dat	977.8	23.7	1.0	0.04	#NUM!	1.5E-05
YLR374C	YLR374C	UWOPS87_ctrl_hmg124h_9.JPG.dat	1156.0	94.7	1.2	0.12	#NUM!	2.6E-04
YLR375W	YLR375W	UWOPS87_ctrl_hmg124h_9.JPG.dat	1006.8	77.6	1.0	0.07	#NUM!	9.0E-05
YLR376C	YLR376C	UWOPS87_ctrl_hmg124h_9.JPG.dat	952.8	44.0	0.9	0.05	#NUM!	5.9E-05
YLR377C	YLR377C	UWOPS87_ctrl_hmg124h_9.JPG.dat	1313.5	32.1	1.2	0.03	#NUM!	6.1E-06
YLR380W	YLR380W	UWOPS87_ctrl_hmg124h_9.JPG.dat	1047.0	35.2	1.0	0.01	#NUM!	4.0E-05
YLR381W	YLR381W	UWOPS87_ctrl_hmg124h_9.JPG.dat	1197.8	55.3	1.1	0.06	#NUM!	4.3E-05
YLR384C	YLR384C	UWOPS87_ctrl_hmg124h_9.JPG.dat	1018.3	164.3	0.9	0.06	#NUM!	1.5E-03
YLR385C	YLR385C	UWOPS87_ctrl_hmg124h_9.JPG.dat	998.8	142.5	0.9	0.14	#NUM!	9.2E-04
YLR386W	YLR386W	UWOPS87_ctrl_hmg124h_9.JPG.dat	670.5	484.5	0.6	0.44	#NUM!	6.7E-02
YLR387C	YLR387C	UWOPS87_ctrl_hmg124h_9.JPG.dat	1148.3	146.3	1.1	0.12	#NUM!	3.7E-04
YLR388W	YLR388W	UWOPS87_ctrl_hmg124h_9.JPG.dat	771.0	208.2	0.7	0.05	#NUM!	1.4E-03
YLR389C	YLR389C	UWOPS87_ctrl_hmg124h_9.JPG.dat	1024.8	52.9	1.0	0.04	#NUM!	1.6E-05
YLR390W	YLR390W	UWOPS87_ctrl_hmg124h_9.JPG.dat	1013.5	123.4	1.0	0.03	#NUM!	4.2E-04
YLR390W-A	YLR390W-A	UWOPS87_ctrl_hmg124h_9.JPG.dat	1075.0	56.1	1.0	0.01	#NUM!	3.2E-05
YLR391W	YLR391W	UWOPS87_ctrl_hmg124h_9.JPG.dat	974.5	27.0	0.9	0.02	#NUM!	5.2E-06
YLR392C	YLR392C	UWOPS87_ctrl_hmg124h_9.JPG.dat	1069.0	46.5	1.0	0.01	#NUM!	6.8E-05

YLR393W	YLR393W	UWOPS87_ctrl_hmg124h_9.JPG.dat	1054.0	7.5	0.9	0.00	#NUM!		4.0E-06
YLR394W	YLR394W	UWOPS87_ctrl_hmg124h_9.JPG.dat	1053.3	71.7	0.9	0.07	#NUM!		9.0E-05
YLR395C	YLR395C	UWOPS87_ctrl_hmg124h_9.JPG.dat	1034.5	94.5	0.9	0.08	#NUM!		1.9E-04
YLR398C	YLR398C	UWOPS87_ctrl_hmg124h_9.JPG.dat	1083.5	95.2	1.0	0.08	#NUM!		1.5E-04
YLR400W	YLR400W	UWOPS87_ctrl_hmg124h_9.JPG.dat	973.0	74.9	0.9	0.07	#NUM!		1.2E-04
YLR401C	YLR401C	UWOPS87_ctrl_hmg124h_9.JPG.dat	938.0	26.2	1.0	0.00	#NUM!		5.7E-06
YLR402W	YLR402W	UWOPS87_ctrl_hmg124h_9.JPG.dat	778.3	28.8	0.8	0.03	#NUM!		1.5E-05
YLR404W	YLR404W	UWOPS87_ctrl_hmg124h_9.JPG.dat	825.0	39.9	0.9	0.04	#NUM!		3.1E-05
YLR405W	YLR405W	UWOPS87_ctrl_hmg124h_9.JPG.dat	1038.5	60.1	1.0	0.06	#NUM!		6.4E-05
YLR406C	YLR406C	UWOPS87_ctrl_hmg124h_9.JPG.dat	1031.5	88.3	1.1	0.09	#NUM!		1.6E-04
YLR407W	YLR407W	UWOPS87_ctrl_hmg124h_9.JPG.dat	1004.8	73.1	1.0	0.07	#NUM!		8.0E-05
YLR408C	YLR408C	UWOPS87_ctrl_hmg124h_9.JPG.dat	1092.5	41.0	1.0	0.03	#NUM!		9.6E-06
YLR410W	YLR410W	UWOPS87_ctrl_hmg124h_9.JPG.dat	965.8	77.3	0.9	0.08	#NUM!		1.5E-04
YLR412W	YLR412W	UWOPS87_ctrl_hmg124h_9.JPG.dat	1141.3	174.7	1.1	0.17	#NUM!		9.3E-04
YLR413W	YLR413W	UWOPS87_ctrl_hmg124h_9.JPG.dat	1008.5	90.7	1.0	0.09	#NUM!		2.2E-04
YLR414C	YLR414C	UWOPS87_ctrl_hmg124h_9.JPG.dat	1298.0	128.3	1.2	0.13	#NUM!		3.2E-04
YOR202W	YOR202W	UWOPS87_ctrl_hmg124h_9.JPG.dat	1201.9	183.5	1.0	0.10	#NUM!		5.3E-276
1	1	Y55_Ator08_hmg124h_1.JPG.dat	1085.0	33.9	1.1	0.03	0.070	0.033	8.3E-06
2	2	Y55_Ator08_hmg124h_1.JPG.dat	905.5	113.2	1.0	0.11	0.002	0.114	4.6E-04
3	3	Y55_Ator08_hmg124h_1.JPG.dat	844.8	51.8	1.0	0.06	-0.018	0.059	6.3E-05
4	4	Y55_Ator08_hmg124h_1.JPG.dat	827.0	66.6	0.9	0.06	-0.003	0.061	8.2E-05
YAL002W	YAL002W	Y55_Ator08_hmg124h_1.JPG.dat	814.8	25.4	1.0	0.03	0.010	0.030	7.5E-06
YAL004W	YAL004W	Y55_Ator08_hmg124h_1.JPG.dat	728.0	15.6	0.9	0.02	-0.095	0.024	4.6E-06
YAL005C	YAL005C	Y55_Ator08_hmg124h_1.JPG.dat	724.5	54.9	0.9	0.07	-0.012	0.072	1.3E-04
YAL007C	YAL007C	Y55_Ator08_hmg124h_1.JPG.dat	788.3	56.3	1.0	0.07	0.050	0.070	8.5E-05
YAL008W	YAL008W	Y55_Ator08_hmg124h_1.JPG.dat	881.5	79.2	1.1	0.03	0.035	0.029	2.4E-04
YAL010C	YAL010C	Y55_Ator08_hmg124h_1.JPG.dat	738.8	51.4	1.0	0.06	-0.001	0.064	7.5E-05
YAL011W	YAL011W	Y55_Ator08_hmg124h_1.JPG.dat	480.5	24.4	0.6	0.01	-0.129	0.009	7.1E-05
YAL013W	YAL013W	Y55_Ator08_hmg124h_1.JPG.dat	551.5	61.9	0.7	0.03	-0.079	0.026	5.3E-04
YAL014C	YAL014C	Y55_Ator08_hmg124h_1.JPG.dat	845.8	43.4	1.0	0.01	-0.050	0.013	6.0E-05
YAL015C	YAL015C	Y55_Ator08_hmg124h_1.JPG.dat	871.3	17.9	1.0	0.02	-0.065	0.023	3.2E-06
YAL017W	YAL017W	Y55_Ator08_hmg124h_1.JPG.dat	934.0	62.9	1.1	0.08	0.019	0.081	1.1E-04
YAL018C	YAL018C	Y55_Ator08_hmg124h_1.JPG.dat	884.0	164.8	1.1	0.20	0.133	0.197	1.7E-03
YAL019W	YAL019W	Y55_Ator08_hmg124h_1.JPG.dat	818.5	11.9	1.0	0.01	-0.040	0.013	6.5E-07
YAL020C	YAL020C	Y55_Ator08_hmg124h_1.JPG.dat	677.0	32.8	0.8	0.00	-0.117	0.004	6.8E-06
YAL022C	YAL022C	Y55_Ator08_hmg124h_1.JPG.dat	748.0	44.0	1.0	0.06	-0.043	0.060	6.7E-05
YAL023C	YAL023C	Y55_Ator08_hmg124h_1.JPG.dat	823.0	6.5	1.1	0.01	0.065	0.007	6.7E-08
YAL026C	YAL026C	Y55_Ator08_hmg124h_1.JPG.dat	582.3	27.0	0.8	0.04	-0.025	0.039	3.4E-05
YAL027W	YAL027W	Y55_Ator08_hmg124h_1.JPG.dat	822.8	26.5	1.1	0.03	0.036	0.035	9.4E-06
YAL028W	YAL028W	Y55_Ator08_hmg124h_1.JPG.dat	844.3	23.9	1.1	0.03	-0.076	0.034	8.8E-06
YAL029C	YAL029C	Y55_Ator08_hmg124h_1.JPG.dat	844.5	23.7	1.0	0.02	-0.023	0.023	3.5E-06
YAL030W	YAL030W	Y55_Ator08_hmg124h_1.JPG.dat	888.0	24.2	1.0	0.02	0.003	0.023	3.1E-06
YAL031C	YAL031C	Y55_Ator08_hmg124h_1.JPG.dat	848.8	82.6	1.0	0.10	0.125	0.104	3.2E-04
YAL034C	YAL034C	Y55_Ator08_hmg124h_1.JPG.dat	752.3	85.5	0.9	0.10	-0.148	0.103	3.8E-04
YAL036C	YAL036C	Y55_Ator08_hmg124h_1.JPG.dat	732.8	116.8	1.0	0.03	0.052	0.033	3.7E-04
YAL037W	YAL037W	Y55_Ator08_hmg124h_1.JPG.dat	828.5	81.4	1.1	0.09	-0.047	0.090	1.7E-04
YAL040C	YAL040C	Y55_Ator08_hmg124h_1.JPG.dat	738.8	73.0	1.0	0.09	0.015	0.093	2.3E-04
YAL042W	YAL042W	Y55_Ator08_hmg124h_1.JPG.dat	743.5	36.3	1.0	0.06	-0.012	0.056	4.7E-05
YAL043C-A	YAL043C-A	Y55_Ator08_hmg124h_1.JPG.dat	711.3	72.6	1.0	0.10	-0.040	0.102	3.0E-04
YAL045C	YAL045C	Y55_Ator08_hmg124h_1.JPG.dat	770.0	37.4	1.0	0.02	-0.008	0.018	1.0E-04
YAL049C	YAL049C	Y55_Ator08_hmg124h_1.JPG.dat	803.0	74.1	1.0	0.09	0.023	0.092	1.9E-04
YAL051W	YAL051W	Y55_Ator08_hmg124h_1.JPG.dat	798.8	58.7	1.0	0.07	-0.011	0.073	1.1E-04
YAL053W	YAL053W	Y55_Ator08_hmg124h_1.JPG.dat	855.5	54.1	1.0	0.06	0.019	0.055	4.3E-05
YAL054C	YAL054C	Y55_Ator08_hmg124h_1.JPG.dat	796.3	47.5	1.0	0.05	0.026	0.051	3.9E-05
YAL055W	YAL055W	Y55_Ator08_hmg124h_1.JPG.dat	880.0	41.3	1.1	0.05	-0.024	0.049	2.7E-05
YAL056W	YAL056W	Y55_Ator08_hmg124h_1.JPG.dat	746.5	38.1	0.9	0.05	-0.077	0.054	5.0E-05
YAL058C-A	YAL058C-A	Y55_Ator08_hmg124h_1.JPG.dat	803.0	62.7	1.0	0.08	0.064	0.084	1.5E-04
YAL058W	YAL058W	Y55_Ator08_hmg124h_1.JPG.dat	778.8	35.1	1.1	0.04	0.102	0.045	2.1E-05
YAL059W	YAL059W	Y55_Ator08_hmg124h_1.JPG.dat	756.8	31.3	1.1	0.04	-0.014	0.043	1.9E-05
YAL060W	YAL060W	Y55_Ator08_hmg124h_1.JPG.dat	634.5	154.2	1.0	0.02	-0.016	0.022	1.5E-04
YAL061W	YAL061W	Y55_Ator08_hmg124h_1.JPG.dat	677.8	57.2	1.0	0.03	-0.005	0.030	3.1E-04
YAL062W	YAL062W	Y55_Ator08_hmg124h_1.JPG.dat	755.3	50.1	1.0	0.02	0.064	0.021	1.4E-04
YAL064C-A	YAL064C-A	Y55_Ator08_hmg124h_1.JPG.dat	834.3	35.5	1.0	0.04	0.013	0.043	1.9E-05
YAL065C	YAL065C	Y55_Ator08_hmg124h_1.JPG.dat	823.3	26.5	1.0	0.04	-0.033	0.036	1.3E-05
YAL066W	YAL066W	Y55_Ator08_hmg124h_1.JPG.dat	733.3	39.6	0.9	0.04	0.007	0.043	2.8E-05
YAL067C	YAL067C	Y55_Ator08_hmg124h_1.JPG.dat	867.3	65.1	1.1	0.09	0.125	0.092	1.7E-04
YAL068C	YAL068C	Y55_Ator08_hmg124h_1.JPG.dat	803.3	30.3	1.0	0.03	-0.037	0.033	9.7E-06
YAR002C-A	YAR002C-A	Y55_Ator08_hmg124h_1.JPG.dat	911.8	117.0	1.2	0.15	0.159	0.149	5.4E-04
YAR002W	YAR002W	Y55_Ator08_hmg124h_1.JPG.dat	582.3	153.6	0.8	0.21	-0.249	0.207	4.5E-03
YAR003W	YAR003W	Y55_Ator08_hmg124h_1.JPG.dat	402.3	71.2	0.6	0.10	-0.187	0.097	1.3E-03

YAR014C	YAR014C	Y55_Ator08_hmg124h_1.JPG.dat	643.3	131.0	0.9	0.20	-0.104	0.202	2.7E-03
YAR015W	YAR015W	Y55_Ator08_hmg124h_1.JPG.dat	622.5	170.0	0.9	0.24	-0.182	0.243	5.7E-03
YAR018C	YAR018C	Y55_Ator08_hmg124h_1.JPG.dat	564.5	129.2	0.8	0.17	-0.263	0.168	3.0E-03
YAR020C	YAR020C	Y55_Ator08_hmg124h_1.JPG.dat	678.8	106.8	0.9	0.13	-0.105	0.135	1.1E-03
YAR023C	YAR023C	Y55_Ator08_hmg124h_1.JPG.dat	852.3	57.8	1.0	0.00	0.054	0.005	6.9E-06
YAR027W	YAR027W	Y55_Ator08_hmg124h_1.JPG.dat	803.8	38.9	1.0	0.02	-0.004	0.018	1.0E-04
YAR028W	YAR028W	Y55_Ator08_hmg124h_1.JPG.dat	706.5	48.9	0.9	0.01	-0.074	0.009	3.2E-05
YAR029W	YAR029W	Y55_Ator08_hmg124h_1.JPG.dat	760.8	63.7	1.0	0.09	-0.007	0.086	1.8E-04
YAR030C	YAR030C	Y55_Ator08_hmg124h_1.JPG.dat	595.8	73.9	0.8	0.10	-0.179	0.098	5.5E-04
YAR031W	YAR031W	Y55_Ator08_hmg124h_1.JPG.dat	791.8	92.3	1.1	0.11	0.088	0.106	2.6E-04
YAR035W	YAR035W	Y55_Ator08_hmg124h_1.JPG.dat	634.0	25.3	0.9	0.03	0.037	0.034	1.6E-05
YAR037W	YAR037W	Y55_Ator08_hmg124h_1.JPG.dat	696.0	43.6	1.0	0.04	0.018	0.041	2.1E-05
YAR040C	YAR040C	Y55_Ator08_hmg124h_1.JPG.dat	794.3	73.5	1.1	0.02	0.088	0.016	7.2E-05
YAR042W	YAR042W	Y55_Ator08_hmg124h_1.JPG.dat	682.5	105.7	1.0	0.05	-0.029	0.054	1.0E-03
YAR043C	YAR043C	Y55_Ator08_hmg124h_1.JPG.dat	461.5	99.0	0.6	0.12	-0.084	0.121	2.4E-03
YAR044W	YAR044W	Y55_Ator08_hmg124h_1.JPG.dat	518.0	25.6	0.6	0.04	-0.062	0.041	7.5E-05
YAR047C	YAR047C	Y55_Ator08_hmg124h_1.JPG.dat	586.8	118.4	0.7	0.05	-0.162	0.052	2.1E-03
YAR050W	YAR050W	Y55_Ator08_hmg124h_1.JPG.dat	872.0	64.8	1.0	0.08	-0.008	0.075	1.1E-04
YBL001C	YBL001C	Y55_Ator08_hmg124h_1.JPG.dat	712.8	147.2	0.9	0.19	-0.050	0.186	2.6E-03
YBL003C	YBL003C	Y55_Ator08_hmg124h_1.JPG.dat	855.5	53.2	1.1	0.08	0.033	0.077	1.1E-04
YBL005W	YBL005W	Y55_Ator08_hmg124h_1.JPG.dat	791.3	48.6	1.0	0.06	-0.009	0.057	5.1E-05
YBL007C	YBL007C	Y55_Ator08_hmg124h_1.JPG.dat	803.3	89.8	1.1	0.04	-0.029	0.041	4.9E-04
YBL008W	YBL008W	Y55_Ator08_hmg124h_1.JPG.dat	805.8	36.4	1.0	0.05	-0.003	0.050	3.4E-05
YBL009W	YBL009W	Y55_Ator08_hmg124h_1.JPG.dat	783.0	79.7	1.0	0.10	-0.191	0.101	3.2E-04
YBL010C	YBL010C	Y55_Ator08_hmg124h_1.JPG.dat	801.3	96.8	1.0	0.11	0.040	0.112	4.3E-04
YBL011W	YBL011W	Y55_Ator08_hmg124h_1.JPG.dat	835.3	63.4	1.0	0.07	-0.064	0.066	8.9E-05
YBL013W	YBL013W	Y55_Ator08_hmg124h_1.JPG.dat	947.8	74.2	1.1	0.08	-0.012	0.078	1.1E-04
YBL015W	YBL015W	Y55_Ator08_hmg124h_1.JPG.dat	887.3	13.9	1.2	0.06	-0.091	0.055	2.8E-05
YBL016W	YBL016W	Y55_Ator08_hmg124h_1.JPG.dat	857.0	116.5	1.1	0.14	0.128	0.143	6.7E-04
YBL017C	YBL017C	Y55_Ator08_hmg124h_1.JPG.dat	789.8	15.8	1.0	0.02	0.005	0.017	1.6E-06
YBL019W	YBL019W	Y55_Ator08_hmg124h_1.JPG.dat	875.3	14.4	1.1	0.02	0.056	0.019	1.3E-06
YBL021C	YBL021C	Y55_Ator08_hmg124h_1.JPG.dat	720.0	61.6	0.9	0.08	-0.120	0.078	1.6E-04
YBL024W	YBL024W	Y55_Ator08_hmg124h_1.JPG.dat	779.0	27.4	1.0	0.04	0.062	0.038	1.4E-05
YBL025W	YBL025W	Y55_Ator08_hmg124h_1.JPG.dat	674.5	21.2	0.9	0.04	0.024	0.035	1.6E-05
YBL027W	YBL027W	Y55_Ator08_hmg124h_1.JPG.dat	137.0	272.0	0.0	0.00	0.001	0.002	4.2E-01
YBL028C	YBL028C	Y55_Ator08_hmg124h_1.JPG.dat	785.5	55.7	1.0	0.07	-0.068	0.072	1.1E-04
YBL029W	YBL029W	Y55_Ator08_hmg124h_1.JPG.dat	896.8	37.4	1.1	0.05	0.062	0.049	2.6E-05
YBL031W	YBL031W	Y55_Ator08_hmg124h_1.JPG.dat	225.8	451.5	0.0	0.00	0.000	0.000	
YBL032W	YBL032W	Y55_Ator08_hmg124h_1.JPG.dat	224.5	449.0	0.0	0.00	0.000	0.000	
YBL036C	YBL036C	Y55_Ator08_hmg124h_1.JPG.dat	839.8	14.1	1.0	0.02	0.072	0.018	1.6E-06
YBL037W	YBL037W	Y55_Ator08_hmg124h_1.JPG.dat	904.0	90.2	1.1	0.02	0.117	0.016	6.8E-05
YBL039C	YBL039C	Y55_Ator08_hmg124h_1.JPG.dat	832.0	92.1	1.0	0.11	0.081	0.113	3.8E-04
YBL042C	YBL042C	Y55_Ator08_hmg124h_1.JPG.dat	856.3	46.4	1.1	0.02	0.105	0.016	7.2E-05
YBL043W	YBL043W	Y55_Ator08_hmg124h_1.JPG.dat	773.0	40.8	1.0	0.01	-0.062	0.010	3.5E-05
YBL046W	YBL046W	Y55_Ator08_hmg124h_1.JPG.dat	746.8	54.2	1.0	0.07	-0.105	0.070	9.7E-05
YBL047C	YBL047C	Y55_Ator08_hmg124h_1.JPG.dat	741.0	77.8	1.0	0.02	0.031	0.017	9.1E-05
YBL048W	YBL048W	Y55_Ator08_hmg124h_1.JPG.dat	795.3	31.1	1.0	0.04	-0.078	0.044	2.4E-05
YBL049W	YBL049W	Y55_Ator08_hmg124h_1.JPG.dat	776.3	62.5	0.9	0.08	-0.036	0.083	1.9E-04
YBL051C	YBL051C	Y55_Ator08_hmg124h_1.JPG.dat	852.0	132.4	0.9	0.05	-0.053	0.050	1.0E-03
YBL052C	YBL052C	Y55_Ator08_hmg124h_1.JPG.dat	793.5	51.9	0.9	0.06	0.003	0.062	8.9E-05
YBL053W	YBL053W	Y55_Ator08_hmg124h_1.JPG.dat	793.8	54.9	1.0	0.07	-0.018	0.070	1.0E-04
YBL054W	YBL054W	Y55_Ator08_hmg124h_1.JPG.dat	890.3	86.0	1.1	0.10	0.150	0.097	2.0E-04
YBL055C	YBL055C	Y55_Ator08_hmg124h_1.JPG.dat	821.3	13.6	1.0	0.01	-0.050	0.014	6.5E-07
YBL056W	YBL056W	Y55_Ator08_hmg124h_1.JPG.dat	720.5	39.6	0.9	0.04	-0.036	0.045	3.0E-05
YBL057C	YBL057C	Y55_Ator08_hmg124h_1.JPG.dat	732.5	29.6	1.0	0.05	-0.016	0.048	3.1E-05
YBL059W	YBL059W	Y55_Ator08_hmg124h_1.JPG.dat	703.0	58.2	1.0	0.02	0.026	0.019	1.1E-04
YBL060W	YBL060W	Y55_Ator08_hmg124h_1.JPG.dat	670.3	42.1	0.9	0.05	0.007	0.053	5.1E-05
YBL061C	YBL061C	Y55_Ator08_hmg124h_1.JPG.dat	764.0	29.3	1.0	0.03	-0.023	0.026	4.6E-06
YBL062W	YBL062W	Y55_Ator08_hmg124h_1.JPG.dat	776.5	61.8	1.0	0.06	-0.093	0.064	7.7E-05
YBL063W	YBL063W	Y55_Ator08_hmg124h_1.JPG.dat	832.5	21.5	1.0	0.03	-0.006	0.029	6.7E-06
YBL064C	YBL064C	Y55_Ator08_hmg124h_1.JPG.dat	863.0	23.2	1.0	0.01	0.065	0.011	3.9E-05
YBL065W	YBL065W	Y55_Ator08_hmg124h_1.JPG.dat	854.0	53.8	1.1	0.06	0.090	0.064	6.1E-05
YBL066C	YBL066C	Y55_Ator08_hmg124h_1.JPG.dat	785.0	46.6	0.9	0.06	-0.102	0.059	6.6E-05
YBL067C	YBL067C	Y55_Ator08_hmg124h_1.JPG.dat	711.3	92.9	0.9	0.12	0.112	0.116	6.0E-04
YBL068W	YBL068W	Y55_Ator08_hmg124h_1.JPG.dat	777.8	57.0	1.0	0.07	0.085	0.072	9.6E-05
YBL069W	YBL069W	Y55_Ator08_hmg124h_1.JPG.dat	720.3	60.4	1.0	0.08	0.024	0.083	1.6E-04
YBL070C	YBL070C	Y55_Ator08_hmg124h_1.JPG.dat	695.8	71.0	1.0	0.04	-0.025	0.036	4.1E-04
YBL071C	YBL071C	Y55_Ator08_hmg124h_1.JPG.dat	714.3	54.9	1.0	0.08	0.003	0.080	1.3E-04
YBL072C	YBL072C	Y55_Ator08_hmg124h_1.JPG.dat	443.0	259.9	0.6	0.35	-0.403	0.349	4.2E-02
YBL075C	YBL075C	Y55_Ator08_hmg124h_1.JPG.dat	775.5	108.4	1.1	0.04	0.012	0.043	5.5E-04

YBL078C	YBL078C	Y55_Ator08_hmg124h_1.JPG.dat	755.3	70.2	0.9	0.09	-0.113	0.090	2.5E-04
YBL079W	YBL079W	Y55_Ator08_hmg124h_1.JPG.dat	794.5	103.0	1.0	0.12	-0.081	0.123	5.7E-04
YBL081W	YBL081W	Y55_Ator08_hmg124h_1.JPG.dat	833.5	134.7	1.0	0.05	0.028	0.053	1.0E-03
YBL082C	YBL082C	Y55_Ator08_hmg124h_1.JPG.dat	997.0	109.2	1.2	0.13	0.040	0.129	3.3E-04
YBL083C	YBL083C	Y55_Ator08_hmg124h_1.JPG.dat	800.3	74.4	1.0	0.09	-0.066	0.091	2.0E-04
YBL085W	YBL085W	Y55_Ator08_hmg124h_1.JPG.dat	759.0	31.3	1.0	0.03	-0.038	0.028	5.5E-06
YBL086C	YBL086C	Y55_Ator08_hmg124h_1.JPG.dat	510.3	20.0	0.7	0.03	-0.085	0.034	2.8E-05
YBL087C	YBL087C	Y55_Ator08_hmg124h_1.JPG.dat	642.5	26.0	0.9	0.00	0.049	0.005	8.5E-06
YBL088C	YBL088C	Y55_Ator08_hmg124h_1.JPG.dat	591.0	36.5	0.8	0.05	-0.252	0.048	4.9E-05
YBL089W	YBL089W	Y55_Ator08_hmg124h_1.JPG.dat	722.5	45.6	1.0	0.03	0.065	0.025	2.1E-04
YBL091C	YBL091C	Y55_Ator08_hmg124h_1.JPG.dat	830.5	25.6	1.1	0.02	0.047	0.023	2.9E-06
YBL091C-A	YBL091C-A	Y55_Ator08_hmg124h_1.JPG.dat	831.5	9.3	1.0	0.00	0.012	0.002	1.7E-06
YBL094C	YBL094C	Y55_Ator08_hmg124h_1.JPG.dat	910.5	45.6	1.1	0.05	0.109	0.054	3.1E-05
YBL095W	YBL095W	Y55_Ator08_hmg124h_1.JPG.dat	863.5	66.5	1.1	0.07	0.060	0.075	8.8E-05
YBL096C	YBL096C	Y55_Ator08_hmg124h_1.JPG.dat	707.5	166.8	0.9	0.20	-0.159	0.201	3.2E-03
YBL098W	YBL098W	Y55_Ator08_hmg124h_1.JPG.dat	864.3	13.3	1.1	0.01	-0.024	0.008	1.6E-05
YBL100C	YBL100C	Y55_Ator08_hmg124h_1.JPG.dat	701.3	15.5	0.9	0.01	-0.078	0.009	2.8E-07
YBL101C	YBL101C	Y55_Ator08_hmg124h_1.JPG.dat	569.5	175.7	0.8	0.26	-0.239	0.259	8.6E-03
YBL102W	YBL102W	Y55_Ator08_hmg124h_1.JPG.dat	690.0	78.2	1.0	0.11	-0.103	0.111	4.1E-04
YBL103C	YBL103C	Y55_Ator08_hmg124h_1.JPG.dat	840.8	72.4	1.2	0.08	0.122	0.076	7.6E-05
YBL104C	YBL104C	Y55_Ator08_hmg124h_1.JPG.dat	748.3	76.5	1.0	0.10	0.091	0.098	2.7E-04
YBL106C	YBL106C	Y55_Ator08_hmg124h_1.JPG.dat	748.3	38.5	1.0	0.04	-0.019	0.035	1.3E-05
YBL107C	YBL107C	Y55_Ator08_hmg124h_1.JPG.dat	861.3	30.6	1.0	0.01	-0.033	0.007	1.4E-05
YBR001C	YBR001C	Y55_Ator08_hmg124h_1.JPG.dat	950.8	111.5	1.2	0.13	0.094	0.134	4.2E-04
YBR005W	YBR005W	Y55_Ator08_hmg124h_1.JPG.dat	891.0	53.5	1.1	0.06	-0.002	0.059	4.7E-05
YBR006W	YBR006W	Y55_Ator08_hmg124h_1.JPG.dat	847.5	64.4	1.0	0.09	0.019	0.085	1.8E-04
YBR007C	YBR007C	Y55_Ator08_hmg124h_1.JPG.dat	742.5	99.0	0.9	0.11	0.048	0.114	5.6E-04
YBR008C	YBR008C	Y55_Ator08_hmg124h_1.JPG.dat	806.3	43.3	1.0	0.04	0.081	0.044	2.3E-05
YBR009C	YBR009C	Y55_Ator08_hmg124h_1.JPG.dat	784.0	32.6	1.0	0.04	0.059	0.041	1.9E-05
YBR010W	YBR010W	Y55_Ator08_hmg124h_1.JPG.dat	789.3	46.2	1.0	0.06	0.024	0.058	5.7E-05
YBR012C	YBR012C	Y55_Ator08_hmg124h_1.JPG.dat	822.8	36.2	1.0	0.05	0.028	0.046	2.5E-05
YBR013C	YBR013C	Y55_Ator08_hmg124h_1.JPG.dat	795.8	52.3	1.0	0.06	-0.066	0.062	7.3E-05
YBR014C	YBR014C	Y55_Ator08_hmg124h_1.JPG.dat	758.3	60.4	0.9	0.07	-0.035	0.071	1.4E-04
YBR015C	YBR015C	Y55_Ator08_hmg124h_1.JPG.dat	762.0	101.3	0.9	0.04	-0.020	0.039	6.0E-04
YBR016W	YBR016W	Y55_Ator08_hmg124h_1.JPG.dat	796.8	37.7	0.9	0.08	-0.128	0.076	1.5E-04
YBR018C	YBR018C	Y55_Ator08_hmg124h_1.JPG.dat	837.3	41.7	1.0	0.06	0.002	0.057	5.6E-05
YBR019C	YBR019C	Y55_Ator08_hmg124h_1.JPG.dat	836.8	51.7	1.0	0.01	0.074	0.015	6.8E-05
YBR020W	YBR020W	Y55_Ator08_hmg124h_1.JPG.dat	821.0	33.4	1.0	0.01	0.056	0.012	4.7E-05
YBR022W	YBR022W	Y55_Ator08_hmg124h_1.JPG.dat	723.8	71.7	0.9	0.10	-0.009	0.097	3.5E-04
YBR023C	YBR023C	Y55_Ator08_hmg124h_1.JPG.dat	600.5	175.1	0.7	0.21	-0.042	0.213	6.2E-03
YBR024W	YBR024W	Y55_Ator08_hmg124h_1.JPG.dat	802.8	30.0	1.0	0.00	-0.007	0.003	2.8E-06
YBR025C	YBR025C	Y55_Ator08_hmg124h_1.JPG.dat	812.0	69.3	1.0	0.04	0.066	0.037	4.3E-04
YBR026C	YBR026C	Y55_Ator08_hmg124h_1.JPG.dat	0.0	0.0	0.0	0.00	0.000	0.000	
YBR027C	YBR027C	Y55_Ator08_hmg124h_1.JPG.dat	998.0	34.9	1.1	0.04	0.037	0.042	1.5E-05
YBR028C	YBR028C	Y55_Ator08_hmg124h_1.JPG.dat	942.8	42.3	1.0	0.04	-0.005	0.040	1.7E-05
YBR030W	YBR030W	Y55_Ator08_hmg124h_1.JPG.dat	839.5	48.1	0.9	0.01	-0.027	0.009	2.8E-05
YBR031W	YBR031W	Y55_Ator08_hmg124h_1.JPG.dat	742.3	65.3	0.9	0.08	-0.057	0.076	1.8E-04
YBR032W	YBR032W	Y55_Ator08_hmg124h_1.JPG.dat	873.8	40.1	1.1	0.05	0.022	0.047	2.5E-05
YBR033W	YBR033W	Y55_Ator08_hmg124h_1.JPG.dat	843.0	75.5	1.0	0.10	0.048	0.099	2.4E-04
YBR034C	YBR034C	Y55_Ator08_hmg124h_1.JPG.dat	724.0	53.6	0.9	0.07	-0.161	0.069	1.2E-04
YBR036C	YBR036C	Y55_Ator08_hmg124h_1.JPG.dat	626.3	20.8	0.8	0.03	-0.119	0.026	9.2E-06
YBR040W	YBR040W	Y55_Ator08_hmg124h_1.JPG.dat	802.5	52.8	1.0	0.07	0.012	0.072	8.8E-05
YBR041W	YBR041W	Y55_Ator08_hmg124h_1.JPG.dat	703.5	34.7	0.9	0.05	-0.123	0.053	5.7E-05
YBR042C	YBR042C	Y55_Ator08_hmg124h_1.JPG.dat	779.8	3.3	0.9	0.00	-0.023	0.002	9.2E-07
YBR043C	YBR043C	Y55_Ator08_hmg124h_1.JPG.dat	886.0	41.2	1.0	0.05	0.017	0.046	2.5E-05
YBR044C	YBR044C	Y55_Ator08_hmg124h_1.JPG.dat	956.0	15.5	1.1	0.01	0.058	0.014	6.8E-07
YBR045C	YBR045C	Y55_Ator08_hmg124h_1.JPG.dat	939.0	34.6	1.1	0.05	0.133	0.049	2.7E-05
YBR046C	YBR046C	Y55_Ator08_hmg124h_1.JPG.dat	830.5	64.7	1.0	0.09	0.035	0.086	1.9E-04
YBR047W	YBR047W	Y55_Ator08_hmg124h_1.JPG.dat	833.5	128.9	1.0	0.15	-0.023	0.151	9.1E-04
YBR048W	YBR048W	Y55_Ator08_hmg124h_1.JPG.dat	657.8	75.1	0.8	0.02	-0.061	0.022	2.9E-04
YBR050C	YBR050C	Y55_Ator08_hmg124h_1.JPG.dat	844.0	49.4	1.1	0.05	0.014	0.048	2.5E-05
YBR051W	YBR051W	Y55_Ator08_hmg124h_1.JPG.dat	769.0	38.3	1.0	0.06	-0.048	0.059	5.6E-05
YBR052C	YBR052C	Y55_Ator08_hmg124h_1.JPG.dat	724.5	62.5	1.0	0.02	0.013	0.019	1.2E-04
YBR053C	YBR053C	Y55_Ator08_hmg124h_1.JPG.dat	800.0	20.2	1.0	0.02	0.036	0.023	3.1E-06
YBR054W	YBR054W	Y55_Ator08_hmg124h_1.JPG.dat	753.3	22.9	0.9	0.01	-0.113	0.008	2.6E-05
YBR056W	YBR056W	Y55_Ator08_hmg124h_1.JPG.dat	842.8	55.0	1.0	0.02	-0.020	0.018	1.1E-04
YBR057C	YBR057C	Y55_Ator08_hmg124h_1.JPG.dat	870.5	45.7	1.0	0.01	-0.023	0.013	6.2E-05
YBR058C	YBR058C	Y55_Ator08_hmg124h_1.JPG.dat	830.5	56.4	1.0	0.07	-0.030	0.069	9.9E-05
YBR059C	YBR059C	Y55_Ator08_hmg124h_1.JPG.dat	814.3	96.0	1.0	0.12	0.050	0.116	4.6E-04
YBR061C	YBR061C	Y55_Ator08_hmg124h_1.JPG.dat	888.5	32.9	1.1	0.04	-0.004	0.043	1.6E-05

YBR062C	YBR062C	Y55_Ator08_hmg124h_1.JPG.dat	796.5	41.3	1.0	0.06	-0.087	0.062	6.4E-05
YBR063C	YBR063C	Y55_Ator08_hmg124h_1.JPG.dat	825.0	56.8	1.1	0.07	0.043	0.070	7.2E-05
YBR064W	YBR064W	Y55_Ator08_hmg124h_1.JPG.dat	742.5	40.7	1.0	0.05	-0.026	0.053	4.1E-05
YBR065C	YBR065C	Y55_Ator08_hmg124h_1.JPG.dat	783.0	72.4	1.1	0.11	0.047	0.107	2.6E-04
YBR066C	YBR066C	Y55_Ator08_hmg124h_1.JPG.dat	703.0	41.1	1.0	0.05	-0.011	0.051	4.2E-05
YBR067C	YBR067C	Y55_Ator08_hmg124h_1.JPG.dat	810.5	20.5	1.0	0.02	0.056	0.020	2.0E-06
YBR068C	YBR068C	Y55_Ator08_hmg124h_1.JPG.dat	824.0	33.6	1.0	0.04	-0.013	0.037	1.4E-05
YBR069C	YBR069C	Y55_Ator08_hmg124h_1.JPG.dat	861.8	15.2	1.0	0.02	-0.014	0.023	3.3E-06
YBR071W	YBR071W	Y55_Ator08_hmg124h_1.JPG.dat	780.8	58.0	0.9	0.06	0.011	0.063	8.2E-05
YBR072W	YBR072W	Y55_Ator08_hmg124h_1.JPG.dat	786.3	57.1	0.9	0.07	-0.040	0.066	1.0E-04
YBR073W	YBR073W	Y55_Ator08_hmg124h_1.JPG.dat	847.0	116.8	1.0	0.14	0.016	0.143	7.5E-04
YBR074W	YBR074W	Y55_Ator08_hmg124h_1.JPG.dat	843.3	68.9	1.0	0.07	0.027	0.073	9.6E-05
YBR075W	YBR075W	Y55_Ator08_hmg124h_1.JPG.dat	747.5	53.9	1.0	0.01	-0.024	0.014	6.4E-05
YBR076W	YBR076W	Y55_Ator08_hmg124h_1.JPG.dat	737.5	59.9	1.0	0.08	0.051	0.079	1.4E-04
YBR077C	YBR077C	Y55_Ator08_hmg124h_1.JPG.dat	770.5	16.2	1.1	0.02	-0.098	0.020	1.8E-06
YBR082C	YBR082C	Y55_Ator08_hmg124h_1.JPG.dat	722.3	13.7	0.9	0.01	0.031	0.009	3.3E-05
YBR083W	YBR083W	Y55_Ator08_hmg124h_1.JPG.dat	810.0	42.2	1.0	0.05	0.001	0.055	4.2E-05
YBR084C-A	YBR084C-A	Y55_Ator08_hmg124h_1.JPG.dat	735.5	18.2	0.9	0.02	0.037	0.022	4.3E-06
YBR084W	YBR084W	Y55_Ator08_hmg124h_1.JPG.dat	850.8	25.6	1.0	0.03	0.035	0.030	7.8E-06
YBR090C	YBR090C	Y55_Ator08_hmg124h_1.JPG.dat	837.5	42.0	1.0	0.01	-0.052	0.012	5.0E-05
YBR090C-A	YBR090C-A	Y55_Ator08_hmg124h_1.JPG.dat	894.3	54.5	1.1	0.01	-0.054	0.006	1.1E-05
YBR092C	YBR092C	Y55_Ator08_hmg124h_1.JPG.dat	714.3	156.3	0.9	0.19	-0.154	0.185	2.6E-03
YBR093C	YBR093C	Y55_Ator08_hmg124h_1.JPG.dat	690.3	26.7	0.8	0.03	0.032	0.034	1.8E-05
YBR094W	YBR094W	Y55_Ator08_hmg124h_1.JPG.dat	771.5	67.3	1.0	0.09	-0.001	0.094	2.3E-04
YBR095C	YBR095C	Y55_Ator08_hmg124h_1.JPG.dat	903.3	12.9	1.2	0.01	0.081	0.006	8.6E-06
YBR098W	YBR098W	Y55_Ator08_hmg124h_1.JPG.dat	729.3	39.9	1.0	0.02	0.006	0.019	1.4E-04
YBR099C	YBR099C	Y55_Ator08_hmg124h_1.JPG.dat	760.0	25.7	1.0	0.01	0.090	0.006	1.1E-05
YBR100W	YBR100W	Y55_Ator08_hmg124h_1.JPG.dat	787.3	38.9	1.0	0.02	0.015	0.015	8.4E-05
YBR101C	YBR101C	Y55_Ator08_hmg124h_1.JPG.dat	824.5	54.7	1.0	0.07	-0.056	0.070	1.0E-04
YBR103W	YBR103W	Y55_Ator08_hmg124h_1.JPG.dat	717.3	30.0	0.8	0.04	-0.162	0.036	2.3E-05
YBR104W	YBR104W	Y55_Ator08_hmg124h_1.JPG.dat	762.8	37.7	0.9	0.04	-0.042	0.044	3.2E-05
YBR105C	YBR105C	Y55_Ator08_hmg124h_1.JPG.dat	642.8	431.9	1.0	0.07	-0.107	0.071	1.8E-03
YBR106W	YBR106W	Y55_Ator08_hmg124h_1.JPG.dat	769.8	34.1	0.9	0.04	0.030	0.043	3.0E-05
YBR107C	YBR107C	Y55_Ator08_hmg124h_1.JPG.dat	899.5	21.1	1.1	0.02	0.010	0.015	8.2E-07
YBR108W	YBR108W	Y55_Ator08_hmg124h_1.JPG.dat	828.5	60.9	1.0	0.02	0.022	0.022	1.7E-04
YBR111C	YBR111C	Y55_Ator08_hmg124h_1.JPG.dat	801.0	45.1	1.0	0.05	0.015	0.051	3.9E-05
YBR113W	YBR113W	Y55_Ator08_hmg124h_1.JPG.dat	814.3	106.1	0.9	0.05	-0.025	0.051	9.8E-04
YBR114W	YBR114W	Y55_Ator08_hmg124h_1.JPG.dat	842.8	29.6	1.0	0.01	0.030	0.007	1.6E-05
YBR115C	YBR115C	Y55_Ator08_hmg124h_1.JPG.dat	771.0	42.2	0.9	0.05	-0.014	0.047	3.7E-05
YBR116C	YBR116C	Y55_Ator08_hmg124h_1.JPG.dat	938.3	13.9	1.1	0.02	-0.025	0.020	2.0E-06
YBR119W	YBR119W	Y55_Ator08_hmg124h_1.JPG.dat	835.5	80.4	0.9	0.08	-0.044	0.085	2.2E-04
YBR121C	YBR121C	Y55_Ator08_hmg124h_1.JPG.dat	0.0	0.0	0.0	0.00	0.000	0.000	
YBR122C	YBR122C	Y55_Ator08_hmg124h_1.JPG.dat	844.8	25.3	1.0	0.03	-0.038	0.033	1.1E-05
YBR125C	YBR125C	Y55_Ator08_hmg124h_1.JPG.dat	872.8	24.9	1.0	0.03	0.064	0.034	1.1E-05
YBR126C	YBR126C	Y55_Ator08_hmg124h_1.JPG.dat	750.0	81.5	0.9	0.09	-0.159	0.088	2.7E-04
YBR128C	YBR128C	Y55_Ator08_hmg124h_1.JPG.dat	855.5	40.5	1.1	0.04	0.053	0.041	1.7E-05
YBR129C	YBR129C	Y55_Ator08_hmg124h_1.JPG.dat	828.5	32.1	1.0	0.05	0.052	0.053	3.7E-05
YBR130C	YBR130C	Y55_Ator08_hmg124h_1.JPG.dat	860.5	45.8	1.1	0.04	-0.022	0.042	1.7E-05
YBR131W	YBR131W	Y55_Ator08_hmg124h_1.JPG.dat	622.8	73.9	0.8	0.10	-0.204	0.100	5.9E-04
YBR132C	YBR132C	Y55_Ator08_hmg124h_1.JPG.dat	938.3	28.9	1.1	0.03	-0.034	0.032	6.9E-06
YBR134W	YBR134W	Y55_Ator08_hmg124h_1.JPG.dat	1006.5	53.7	1.1	0.04	0.002	0.041	1.3E-05
YBR137W	YBR137W	Y55_Ator08_hmg124h_1.JPG.dat	931.5	50.8	1.0	0.04	-0.079	0.043	2.2E-05
YBR138C	YBR138C	Y55_Ator08_hmg124h_1.JPG.dat	962.0	22.8	1.0	0.02	-0.054	0.021	2.4E-06
YBR139W	YBR139W	Y55_Ator08_hmg124h_1.JPG.dat	934.5	73.4	1.1	0.08	0.105	0.080	1.0E-04
YBR141C	YBR141C	Y55_Ator08_hmg124h_1.JPG.dat	852.8	25.5	1.0	0.02	-0.067	0.021	2.8E-06
YBR144C	YBR144C	Y55_Ator08_hmg124h_1.JPG.dat	769.8	32.5	0.9	0.01	-0.116	0.014	7.7E-05
YBR145W	YBR145W	Y55_Ator08_hmg124h_1.JPG.dat	878.5	72.9	1.1	0.09	0.040	0.092	1.6E-04
YBR146W	YBR146W	Y55_Ator08_hmg124h_1.JPG.dat	719.8	65.4	0.9	0.02	-0.106	0.017	1.2E-04
YBR147W	YBR147W	Y55_Ator08_hmg124h_1.JPG.dat	835.3	18.1	1.1	0.02	0.033	0.019	1.5E-06
YBR148W	YBR148W	Y55_Ator08_hmg124h_1.JPG.dat	862.5	31.3	1.1	0.04	0.065	0.039	1.2E-05
YBR149W	YBR149W	Y55_Ator08_hmg124h_1.JPG.dat	863.0	25.7	1.1	0.03	0.038	0.032	7.9E-06
YBR150C	YBR150C	Y55_Ator08_hmg124h_1.JPG.dat	877.5	37.1	1.0	0.04	-0.017	0.041	1.8E-05
YBR151W	YBR151W	Y55_Ator08_hmg124h_1.JPG.dat	899.3	29.7	1.0	0.03	0.060	0.028	6.1E-06
YBR156C	YBR156C	Y55_Ator08_hmg124h_1.JPG.dat	914.8	63.0	1.0	0.07	-0.016	0.072	1.0E-04
YBR157C	YBR157C	Y55_Ator08_hmg124h_1.JPG.dat	874.8	43.5	1.0	0.05	0.073	0.046	2.5E-05
YBR158W	YBR158W	Y55_Ator08_hmg124h_1.JPG.dat	878.3	46.9	1.0	0.05	-0.040	0.046	2.5E-05
YBR159W	YBR159W	Y55_Ator08_hmg124h_1.JPG.dat	845.8	84.4	1.0	0.10	0.006	0.104	3.0E-04
YBR161W	YBR161W	Y55_Ator08_hmg124h_1.JPG.dat	852.0	61.0	1.1	0.07	-0.005	0.072	8.3E-05
YBR162C	YBR162C	Y55_Ator08_hmg124h_1.JPG.dat	860.8	71.8	1.1	0.10	-0.046	0.100	1.9E-04
YBR162W-	YBR162W-A	Y55_Ator08_hmg124h_1.JPG.dat	799.8	54.9	1.1	0.06	0.034	0.058	4.5E-05

YBR164C	YBR164C	Y55_Ator08_hmg124h_1.JPG.dat	708.0	46.6	0.9	0.06	-0.086	0.057	6.3E-05
YBR165W	YBR165W	Y55_Ator08_hmg124h_1.JPG.dat	811.5	21.5	1.0	0.03	0.101	0.028	6.0E-06
YBR166C	YBR166C	Y55_Ator08_hmg124h_1.JPG.dat	815.5	38.6	1.0	0.06	0.036	0.061	6.8E-05
YBR168W	YBR168W	Y55_Ator08_hmg124h_1.JPG.dat	874.0	15.0	1.0	0.00	0.107	0.003	3.1E-06
YBR169C	YBR169C	Y55_Ator08_hmg124h_1.JPG.dat	912.8	26.0	1.0	0.01	-0.008	0.015	8.0E-07
YBR170C	YBR170C	Y55_Ator08_hmg124h_1.JPG.dat	807.0	33.0	1.0	0.04	-0.020	0.042	2.2E-05
YBR171W	YBR171W	Y55_Ator08_hmg124h_1.JPG.dat	934.3	70.5	1.1	0.03	0.032	0.025	1.9E-04
YBR172C	YBR172C	Y55_Ator08_hmg124h_1.JPG.dat	806.0	26.3	1.0	0.03	-0.103	0.034	1.1E-05
YBR174C	YBR174C	Y55_Ator08_hmg124h_1.JPG.dat	749.3	37.6	1.0	0.05	-0.011	0.050	3.9E-05
YBR175W	YBR175W	Y55_Ator08_hmg124h_1.JPG.dat	666.0	55.9	0.9	0.07	-0.042	0.071	1.3E-04
YBR176W	YBR176W	Y55_Ator08_hmg124h_1.JPG.dat	718.5	34.8	1.0	0.05	-0.008	0.048	3.1E-05
YBR177C	YBR177C	Y55_Ator08_hmg124h_1.JPG.dat	741.3	17.7	1.0	0.03	0.023	0.031	7.7E-06
YBR178W	YBR178W	Y55_Ator08_hmg124h_1.JPG.dat	735.3	45.2	1.0	0.05	-0.041	0.052	4.3E-05
YBR180W	YBR180W	Y55_Ator08_hmg124h_1.JPG.dat	853.8	27.9	1.1	0.04	0.093	0.037	1.1E-05
YBR181C	YBR181C	Y55_Ator08_hmg124h_1.JPG.dat	759.0	29.6	0.9	0.01	0.088	0.011	5.1E-05
YBR182C	YBR182C	Y55_Ator08_hmg124h_1.JPG.dat	838.5	54.3	1.0	0.06	-0.015	0.058	5.6E-05
YBR183W	YBR183W	Y55_Ator08_hmg124h_1.JPG.dat	938.8	54.3	1.1	0.06	0.137	0.058	4.0E-05
YBR184W	YBR184W	Y55_Ator08_hmg124h_1.JPG.dat	824.5	104.0	0.9	0.12	0.001	0.115	4.9E-04
YBR185C	YBR185C	Y55_Ator08_hmg124h_1.JPG.dat	877.8	45.8	1.1	0.02	0.074	0.015	6.8E-05
YBR186W	YBR186W	Y55_Ator08_hmg124h_1.JPG.dat	786.8	53.5	1.0	0.06	-0.033	0.062	6.6E-05
YBR187W	YBR187W	Y55_Ator08_hmg124h_1.JPG.dat	760.3	36.6	1.0	0.05	-0.024	0.046	2.5E-05
YBR188C	YBR188C	Y55_Ator08_hmg124h_1.JPG.dat	752.8	52.4	1.0	0.08	0.020	0.079	1.2E-04
YBR189W	YBR189W	Y55_Ator08_hmg124h_1.JPG.dat	0.0	0.0	0.0	0.00	0.000	0.000	
YBR194W	YBR194W	Y55_Ator08_hmg124h_1.JPG.dat	833.8	32.2	1.0	0.00	0.068	0.005	6.7E-06
YBR195C	YBR195C	Y55_Ator08_hmg124h_1.JPG.dat	830.8	19.6	1.0	0.02	-0.002	0.019	1.7E-06
YBR197C	YBR197C	Y55_Ator08_hmg124h_1.JPG.dat	914.0	42.6	1.1	0.05	0.020	0.053	3.3E-05
YBR199W	YBR199W	Y55_Ator08_hmg124h_1.JPG.dat	817.0	34.2	0.9	0.04	-0.067	0.040	2.0E-05
YBR200W	YBR200W	Y55_Ator08_hmg124h_1.JPG.dat	0.0	0.0	0.0	0.00	0.000	0.000	
YBR201W	YBR201W	Y55_Ator08_hmg124h_1.JPG.dat	908.0	37.5	1.0	0.01	-0.022	0.011	3.5E-05
YBR203W	YBR203W	Y55_Ator08_hmg124h_1.JPG.dat	872.8	59.1	1.0	0.07	0.092	0.070	8.4E-05
YBR204C	YBR204C	Y55_Ator08_hmg124h_1.JPG.dat	763.5	23.7	1.0	0.03	-0.044	0.034	1.2E-05
YBR205W	YBR205W	Y55_Ator08_hmg124h_1.JPG.dat	775.0	92.6	1.0	0.12	-0.025	0.123	4.6E-04
YBR206W	YBR206W	Y55_Ator08_hmg124h_1.JPG.dat	654.5	102.3	0.9	0.14	-0.096	0.140	1.1E-03
YBR207W	YBR207W	Y55_Ator08_hmg124h_1.JPG.dat	752.3	50.6	1.0	0.07	0.015	0.066	7.7E-05
YBR208C	YBR208C	Y55_Ator08_hmg124h_1.JPG.dat	844.3	52.3	1.1	0.06	-0.001	0.062	5.4E-05
YBR209W	YBR209W	Y55_Ator08_hmg124h_1.JPG.dat	864.5	96.5	1.1	0.12	0.003	0.118	3.9E-04
YBR210W	YBR210W	Y55_Ator08_hmg124h_1.JPG.dat	948.8	54.1	1.1	0.06	-0.026	0.065	5.5E-05
YBR212W	YBR212W	Y55_Ator08_hmg124h_1.JPG.dat	906.0	49.3	1.1	0.02	0.074	0.017	8.1E-05
YBR213W	YBR213W	Y55_Ator08_hmg124h_1.JPG.dat	0.0	0.0	0.0	0.00	0.000	0.000	
YBR214W	YBR214W	Y55_Ator08_hmg124h_1.JPG.dat	918.3	61.7	1.1	0.02	-0.036	0.020	1.1E-04
YBR215W	YBR215W	Y55_Ator08_hmg124h_1.JPG.dat	911.3	14.4	1.0	0.00	0.078	0.003	2.8E-06
YBR216C	YBR216C	Y55_Ator08_hmg124h_1.JPG.dat	891.5	80.9	1.1	0.09	0.011	0.094	1.8E-04
YBR217W	YBR217W	Y55_Ator08_hmg124h_1.JPG.dat	795.0	23.4	1.0	0.01	-0.043	0.011	3.9E-05
YBR218C	YBR218C	Y55_Ator08_hmg124h_1.JPG.dat	814.3	58.7	1.0	0.07	-0.007	0.071	9.7E-05
YBR219C	YBR219C	Y55_Ator08_hmg124h_1.JPG.dat	786.8	39.0	0.9	0.02	-0.052	0.019	1.3E-04
YBR220C	YBR220C	Y55_Ator08_hmg124h_1.JPG.dat	797.5	60.7	0.9	0.07	-0.078	0.070	1.1E-04
YBR221C	YBR221C	Y55_Ator08_hmg124h_1.JPG.dat	906.8	31.8	1.0	0.03	0.008	0.034	9.9E-06
YBR222C	YBR222C	Y55_Ator08_hmg124h_1.JPG.dat	915.3	12.6	1.0	0.01	0.026	0.013	6.5E-07
YBR223C	YBR223C	Y55_Ator08_hmg124h_1.JPG.dat	948.3	26.4	1.1	0.04	0.016	0.038	1.3E-05
YOR202W	YOR202W	Y55_Ator08_hmg124h_1.JPG.dat	877.2	153.6	1.0	0.16	0.004	0.161	5.4E-221
1	1	Y55_Ator08_hmg124h_10.JPG.dat	0.0	0.0	0.0	0.00	0.000	0.000	
2	2	Y55_Ator08_hmg124h_10.JPG.dat	733.5	57.6	1.2	0.13	-0.163	0.126	3.6E-04
3	3	Y55_Ator08_hmg124h_10.JPG.dat	779.0	104.0	1.1	0.11	0.033	0.113	3.1E-04
4	4	Y55_Ator08_hmg124h_10.JPG.dat	819.5	93.9	1.0	0.12	0.081	0.119	4.0E-04
YLR415C	YLR415C	Y55_Ator08_hmg124h_10.JPG.dat	729.5	48.7	1.0	0.01	0.030	0.006	1.2E-05
YLR416C	YLR416C	Y55_Ator08_hmg124h_10.JPG.dat	732.5	68.4	1.0	0.06	-0.031	0.061	5.5E-05
YLR418C	YLR418C	Y55_Ator08_hmg124h_10.JPG.dat	471.5	28.0	0.7	0.03	-0.084	0.028	1.9E-05
YLR420W	YLR420W	Y55_Ator08_hmg124h_10.JPG.dat	620.3	56.3	0.8	0.02	-0.090	0.024	2.7E-04
YLR421C	YLR421C	Y55_Ator08_hmg124h_10.JPG.dat	857.5	155.8	1.2	0.18	0.309	0.181	9.9E-04
YLR422W	YLR422W	Y55_Ator08_hmg124h_10.JPG.dat	827.0	69.2	1.1	0.13	0.037	0.130	4.3E-04
YLR423C	YLR423C	Y55_Ator08_hmg124h_10.JPG.dat	771.8	70.4	1.0	0.12	-0.043	0.124	4.6E-04
YLR425W	YLR425W	Y55_Ator08_hmg124h_10.JPG.dat	677.8	58.4	0.9	0.07	-0.194	0.069	1.2E-04
YLR426W	YLR426W	Y55_Ator08_hmg124h_10.JPG.dat	0.0	0.0	0.0	0.00	0.000	0.000	
YLR427W	YLR427W	Y55_Ator08_hmg124h_10.JPG.dat	791.3	137.2	1.1	0.16	0.049	0.157	8.9E-04
YLR428C	YLR428C	Y55_Ator08_hmg124h_10.JPG.dat	783.8	30.9	1.1	0.01	-0.076	0.006	1.0E-05
YLR429W	YLR429W	Y55_Ator08_hmg124h_10.JPG.dat	685.0	16.4	1.0	0.01	-0.063	0.008	2.5E-05
YLR431C	YLR431C	Y55_Ator08_hmg124h_10.JPG.dat	722.3	57.0	1.0	0.08	-0.016	0.083	1.4E-04
YLR432W	YLR432W	Y55_Ator08_hmg124h_10.JPG.dat	730.5	172.2	1.0	0.24	0.010	0.240	3.3E-03
YLR433C	YLR433C	Y55_Ator08_hmg124h_10.JPG.dat	779.0	77.7	1.1	0.10	0.038	0.103	2.4E-04
YLR434C	YLR434C	Y55_Ator08_hmg124h_10.JPG.dat	718.8	29.4	1.0	0.04	-0.026	0.044	2.7E-05

YLR435W	YLR435W	Y55_Ator08_hmg124h_10.JPG.dat	556.0	39.4	0.7	0.06	-0.098	0.058	1.3E-04
YLR436C	YLR436C	Y55_Ator08_hmg124h_10.JPG.dat	715.0	43.2	1.0	0.02	-0.064	0.021	1.5E-04
YLR437C	YLR437C	Y55_Ator08_hmg124h_10.JPG.dat	859.3	80.7	1.1	0.10	0.000	0.096	1.9E-04
YLR438W	YLR438W	Y55_Ator08_hmg124h_10.JPG.dat	935.0	116.8	1.1	0.13	-0.059	0.132	4.2E-04
YLR441C	YLR441C	Y55_Ator08_hmg124h_10.JPG.dat	806.3	34.9	1.0	0.03	0.284	0.030	6.4E-06
YLR442C	YLR442C	Y55_Ator08_hmg124h_10.JPG.dat	627.3	44.4	0.9	0.07	0.078	0.070	1.4E-04
YLR443W	YLR443W	Y55_Ator08_hmg124h_10.JPG.dat	719.3	41.0	1.0	0.01	0.032	0.006	1.0E-05
YLR444C	YLR444C	Y55_Ator08_hmg124h_10.JPG.dat	626.8	49.8	0.9	0.07	-0.022	0.065	1.0E-04
YLR445W	YLR445W	Y55_Ator08_hmg124h_10.JPG.dat	673.0	50.2	0.9	0.07	-0.040	0.070	1.1E-04
YLR446W	YLR446W	Y55_Ator08_hmg124h_10.JPG.dat	796.3	39.8	1.1	0.05	-0.094	0.053	3.3E-05
YLR448W	YLR448W	Y55_Ator08_hmg124h_10.JPG.dat	755.0	9.6	1.0	0.02	0.190	0.021	2.5E-06
YLR449W	YLR449W	Y55_Ator08_hmg124h_10.JPG.dat	672.5	80.3	0.9	0.11	0.090	0.111	5.0E-04
YLR450W	YLR450W	Y55_Ator08_hmg124h_10.JPG.dat	0.0	0.0	0.0	0.00	0.000	0.000	
YLR451W	YLR451W	Y55_Ator08_hmg124h_10.JPG.dat	884.8	76.5	1.1	0.08	0.319	0.084	1.4E-04
YLR452C	YLR452C	Y55_Ator08_hmg124h_10.JPG.dat	753.0	74.2	0.9	0.03	-0.119	0.026	2.5E-04
YLR453C	YLR453C	Y55_Ator08_hmg124h_10.JPG.dat	822.0	133.6	1.1	0.16	0.070	0.158	8.7E-04
YLR454W	YLR454W	Y55_Ator08_hmg124h_10.JPG.dat	630.8	33.4	0.9	0.04	0.115	0.044	3.1E-05
YLR455W	YLR455W	Y55_Ator08_hmg124h_10.JPG.dat	718.5	112.6	0.9	0.01	-0.110	0.015	8.3E-05
YLR456W	YLR456W	Y55_Ator08_hmg124h_10.JPG.dat	909.8	129.4	1.1	0.02	0.043	0.017	7.2E-05
YLR460C	YLR460C	Y55_Ator08_hmg124h_10.JPG.dat	906.3	78.5	1.1	0.11	0.109	0.107	2.3E-04
YLR461W	YLR461W	Y55_Ator08_hmg124h_10.JPG.dat	769.3	49.4	0.9	0.06	0.057	0.063	8.0E-05
YML001W	YML001W	Y55_Ator08_hmg124h_10.JPG.dat	0.0	0.0	0.0	0.00	0.000	0.000	
YML002W	YML002W	Y55_Ator08_hmg124h_10.JPG.dat	820.5	27.5	1.0	0.01	0.051	0.007	1.7E-05
YML003W	YML003W	Y55_Ator08_hmg124h_10.JPG.dat	743.3	31.0	0.9	0.04	-0.180	0.042	2.9E-05
YML004C	YML004C	Y55_Ator08_hmg124h_10.JPG.dat	940.3	53.5	1.0	0.06	0.265	0.059	5.1E-05
YML005W	YML005W	Y55_Ator08_hmg124h_10.JPG.dat	778.0	104.7	0.8	0.12	-0.118	0.116	6.8E-04
YML006C	YML006C	Y55_Ator08_hmg124h_10.JPG.dat	922.8	39.1	1.1	0.04	0.118	0.044	1.7E-05
YML007W	YML007W	Y55_Ator08_hmg124h_10.JPG.dat	742.5	9.0	1.0	0.01	0.072	0.013	5.7E-07
YML008C	YML008C	Y55_Ator08_hmg124h_10.JPG.dat	685.5	62.5	1.0	0.08	0.032	0.079	1.5E-04
YML009c	YML009c	Y55_Ator08_hmg124h_10.JPG.dat	745.3	138.2	1.0	0.16	-0.029	0.164	1.3E-03
YML010C-I	YML010C-B	Y55_Ator08_hmg124h_10.JPG.dat	836.0	13.6	1.0	0.02	0.050	0.017	1.4E-06
YML011C	YML011C	Y55_Ator08_hmg124h_10.JPG.dat	841.8	25.5	1.0	0.04	0.164	0.039	1.5E-05
YML012W	YML012W	Y55_Ator08_hmg124h_10.JPG.dat	0.0	0.0	0.0	0.00	0.000	0.000	
YML013C-/	YML013C-A	Y55_Ator08_hmg124h_10.JPG.dat	790.8	35.8	1.0	0.04	-0.207	0.040	1.9E-05
YML013W	YML013W	Y55_Ator08_hmg124h_10.JPG.dat	758.5	19.7	0.9	0.03	-0.012	0.032	1.2E-05
YML016C	YML016C	Y55_Ator08_hmg124h_10.JPG.dat	885.0	13.5	1.0	0.02	-0.056	0.015	9.2E-07
YML017W	YML017W	Y55_Ator08_hmg124h_10.JPG.dat	966.8	68.0	1.1	0.08	-0.014	0.076	9.3E-05
YML018C	YML018C	Y55_Ator08_hmg124h_10.JPG.dat	772.0	81.2	1.0	0.10	-0.044	0.105	3.5E-04
YML019W	YML019W	Y55_Ator08_hmg124h_10.JPG.dat	774.5	27.2	1.1	0.04	-0.065	0.037	1.2E-05
YML020W	YML020W	Y55_Ator08_hmg124h_10.JPG.dat	776.8	109.8	1.1	0.15	0.096	0.153	7.4E-04
YML021C	YML021C	Y55_Ator08_hmg124h_10.JPG.dat	848.5	76.0	1.1	0.11	0.104	0.110	2.8E-04
YML022W	YML022W	Y55_Ator08_hmg124h_10.JPG.dat	845.8	40.8	1.0	0.05	-0.045	0.048	2.7E-05
YML026C	YML026C	Y55_Ator08_hmg124h_10.JPG.dat	846.0	31.4	1.1	0.03	0.049	0.034	9.4E-06
YML027W	YML027W	Y55_Ator08_hmg124h_10.JPG.dat	800.5	27.5	1.0	0.03	0.003	0.033	1.0E-05
YML028W	YML028W	Y55_Ator08_hmg124h_10.JPG.dat	818.5	31.1	1.0	0.04	0.079	0.036	1.2E-05
YML029W	YML029W	Y55_Ator08_hmg124h_10.JPG.dat	884.3	25.3	1.1	0.03	0.221	0.034	7.7E-06
YML030W	YML030W	Y55_Ator08_hmg124h_10.JPG.dat	898.0	61.7	1.1	0.07	-0.077	0.073	8.4E-05
YML032C	YML032C	Y55_Ator08_hmg124h_10.JPG.dat	679.5	453.0	1.1	0.02	0.420	0.019	9.8E-05
YML033W	YML033W	Y55_Ator08_hmg124h_10.JPG.dat	731.0	33.4	1.0	0.06	-0.014	0.055	5.2E-05
YML034W	YML034W	Y55_Ator08_hmg124h_10.JPG.dat	697.5	72.8	0.9	0.03	-0.013	0.029	3.3E-04
YML035C	YML035C	Y55_Ator08_hmg124h_10.JPG.dat	914.8	122.6	1.2	0.17	0.472	0.169	7.1E-04
YML035C-/	YML035C-A	Y55_Ator08_hmg124h_10.JPG.dat	654.8	40.1	0.8	0.06	-0.034	0.062	1.1E-04
YML036W	YML036W	Y55_Ator08_hmg124h_10.JPG.dat	706.3	28.8	0.9	0.01	-0.045	0.006	1.8E-05
YML037C	YML037C	Y55_Ator08_hmg124h_10.JPG.dat	713.3	67.3	0.9	0.08	-0.015	0.078	1.7E-04
YML038C	YML038C	Y55_Ator08_hmg124h_10.JPG.dat	824.0	91.2	1.0	0.10	0.058	0.098	2.3E-04
YML041C	YML041C	Y55_Ator08_hmg124h_10.JPG.dat	928.3	84.6	1.1	0.05	0.066	0.046	5.5E-04
YML042W	YML042W	Y55_Ator08_hmg124h_10.JPG.dat	830.0	47.0	1.1	0.06	0.041	0.058	4.1E-05
YML047C	YML047C	Y55_Ator08_hmg124h_10.JPG.dat	879.5	74.0	1.1	0.08	0.116	0.082	1.0E-04
YML048W	YML048W	Y55_Ator08_hmg124h_10.JPG.dat	762.0	61.7	1.0	0.07	-0.022	0.069	9.6E-05
YML048W-	YML048W-A	Y55_Ator08_hmg124h_10.JPG.dat	807.8	75.2	1.1	0.10	0.123	0.102	2.3E-04
YML051W	YML051W	Y55_Ator08_hmg124h_10.JPG.dat	723.0	46.1	1.0	0.05	-0.006	0.050	3.3E-05
YML052W	YML052W	Y55_Ator08_hmg124h_10.JPG.dat	686.5	51.1	1.0	0.06	-0.037	0.062	6.9E-05
YML053C	YML053C	Y55_Ator08_hmg124h_10.JPG.dat	737.3	37.3	1.1	0.02	-0.003	0.015	6.5E-05
YML054C	YML054C	Y55_Ator08_hmg124h_10.JPG.dat	667.5	16.4	1.0	0.01	-0.044	0.011	4.1E-05
YML055W	YML055W	Y55_Ator08_hmg124h_10.JPG.dat	654.5	15.4	0.9	0.03	0.032	0.027	6.2E-06
YML056C	YML056C	Y55_Ator08_hmg124h_10.JPG.dat	706.0	47.3	1.0	0.01	0.029	0.013	5.1E-05
YML057W	YML057W	Y55_Ator08_hmg124h_10.JPG.dat	735.5	148.3	1.0	0.17	-0.043	0.173	1.4E-03
YML058C-/	YML058C-A	Y55_Ator08_hmg124h_10.JPG.dat	815.0	116.3	1.1	0.14	0.108	0.141	6.1E-04
YML058W	YML058W	Y55_Ator08_hmg124h_10.JPG.dat	712.5	101.4	1.0	0.13	-0.058	0.133	7.4E-04
YML059C	YML059C	Y55_Ator08_hmg124h_10.JPG.dat	771.8	54.7	1.0	0.06	0.026	0.063	6.5E-05

YML060W	YML060W	Y55_Ator08_hmg124h_10.JPG.dat	792.8	30.1	1.0	0.06	-0.003	0.057	4.7E-05
YML062C	YML062C	Y55_Ator08_hmg124h_10.JPG.dat	722.0	60.7	1.0	0.01	0.085	0.012	4.3E-05
YML063W	YML063W	Y55_Ator08_hmg124h_10.JPG.dat	183.3	366.5	0.0	0.00	0.000	0.000	
YML066C	YML066C	Y55_Ator08_hmg124h_10.JPG.dat	699.8	44.0	1.0	0.07	0.018	0.066	8.0E-05
YML067C	YML067C	Y55_Ator08_hmg124h_10.JPG.dat	829.0	79.4	1.2	0.04	0.138	0.041	3.6E-04
YML068W	YML068W	Y55_Ator08_hmg124h_10.JPG.dat	544.0	378.4	1.0	0.17	0.130	0.171	9.3E-03
YML070W	YML070W	Y55_Ator08_hmg124h_10.JPG.dat	368.0	427.5	0.5	0.57	0.016	0.574	1.8E-01
YML071C	YML071C	Y55_Ator08_hmg124h_10.JPG.dat	641.8	433.1	1.1	0.10	0.033	0.097	2.4E-03
YML072C	YML072C	Y55_Ator08_hmg124h_10.JPG.dat	713.3	91.8	1.0	0.02	0.461	0.022	1.7E-04
YML074C	YML074C	Y55_Ator08_hmg124h_10.JPG.dat	0.0	0.0	0.0	0.00	0.000	0.000	
YML075C	YML075C	Y55_Ator08_hmg124h_10.JPG.dat	0.0	0.0	0.0	0.00	0.000	0.000	
YML076C	YML076C	Y55_Ator08_hmg124h_10.JPG.dat	414.3	489.9	0.5	0.62	0.084	0.619	1.9E-01
YML081C-/	YML081C-A	Y55_Ator08_hmg124h_10.JPG.dat	408.8	472.2	0.5	0.63	-0.018	0.631	1.8E-01
YML090W	YML090W	Y55_Ator08_hmg124h_10.JPG.dat	756.8	33.2	1.1	0.04	0.049	0.037	1.0E-05
YML094W	YML094W	Y55_Ator08_hmg124h_10.JPG.dat	0.0	0.0	0.0	0.00	0.000	0.000	
YML095C	YML095C	Y55_Ator08_hmg124h_10.JPG.dat	703.5	148.7	0.9	0.07	-0.133	0.068	1.9E-03
YML096W	YML096W	Y55_Ator08_hmg124h_10.JPG.dat	750.0	37.3	1.0	0.06	0.044	0.058	4.9E-05
YML097C	YML097C	Y55_Ator08_hmg124h_10.JPG.dat	0.0	0.0	0.0	0.00	0.000	0.000	
YML099C	YML099C	Y55_Ator08_hmg124h_10.JPG.dat	757.8	51.8	1.0	0.06	-0.110	0.064	6.5E-05
YML100W	YML100W	Y55_Ator08_hmg124h_10.JPG.dat	872.8	39.9	1.2	0.01	0.027	0.006	9.9E-06
YML100W-	YML100W-A	Y55_Ator08_hmg124h_10.JPG.dat	892.0	86.7	1.1	0.12	0.031	0.117	3.1E-04
YML101C	YML101C	Y55_Ator08_hmg124h_10.JPG.dat	859.3	63.4	1.0	0.02	-0.125	0.017	9.5E-05
YML102C-/	YML102C-A	Y55_Ator08_hmg124h_10.JPG.dat	166.8	333.5	0.0	0.00	0.000	0.000	
YML102W	YML102W	Y55_Ator08_hmg124h_10.JPG.dat	699.5	20.5	1.0	0.01	0.036	0.014	8.8E-07
YML103C	YML103C	Y55_Ator08_hmg124h_10.JPG.dat	546.3	364.5	1.0	0.02	-0.062	0.021	1.5E-04
YML104C	YML104C	Y55_Ator08_hmg124h_10.JPG.dat	714.8	16.7	1.0	0.03	-0.148	0.035	1.2E-05
YML106W	YML106W	Y55_Ator08_hmg124h_10.JPG.dat	0.0	0.0	0.0	0.00	0.000	0.000	
YML107C	YML107C	Y55_Ator08_hmg124h_10.JPG.dat	891.5	43.3	1.1	0.05	0.225	0.051	2.7E-05
YML108W	YML108W	Y55_Ator08_hmg124h_10.JPG.dat	852.3	47.3	1.1	0.06	-0.192	0.058	4.6E-05
YML109W	YML109W	Y55_Ator08_hmg124h_10.JPG.dat	717.8	71.7	0.9	0.09	-0.111	0.089	2.6E-04
YML113W	YML113W	Y55_Ator08_hmg124h_10.JPG.dat	786.5	13.9	0.9	0.01	-0.013	0.014	8.9E-07
YML116W	YML116W	Y55_Ator08_hmg124h_10.JPG.dat	933.0	20.5	1.1	0.03	0.046	0.033	8.0E-06
YML117W	YML117W	Y55_Ator08_hmg124h_10.JPG.dat	967.3	37.5	1.1	0.04	0.072	0.043	1.8E-05
YML117W-	YML117W-A	Y55_Ator08_hmg124h_10.JPG.dat	952.8	84.6	1.1	0.02	0.140	0.019	8.9E-05
YML118W	YML118W	Y55_Ator08_hmg124h_10.JPG.dat	718.0	43.2	0.9	0.05	-0.073	0.054	5.5E-05
YML119W	YML119W	Y55_Ator08_hmg124h_10.JPG.dat	760.3	160.8	1.0	0.02	-0.052	0.019	1.3E-04
YML120C	YML120C	Y55_Ator08_hmg124h_10.JPG.dat	853.3	103.9	1.2	0.13	0.193	0.126	3.5E-04
YML121W	YML121W	Y55_Ator08_hmg124h_10.JPG.dat	933.0	70.3	1.2	0.09	0.113	0.091	1.3E-04
YML122C	YML122C	Y55_Ator08_hmg124h_10.JPG.dat	689.5	39.9	0.8	0.05	-0.109	0.048	5.0E-05
YML123C	YML123C	Y55_Ator08_hmg124h_10.JPG.dat	821.8	82.9	1.0	0.10	0.240	0.100	2.6E-04
YML124C	YML124C	Y55_Ator08_hmg124h_10.JPG.dat	800.3	49.0	1.0	0.06	0.038	0.058	5.4E-05
YML128C	YML128C	Y55_Ator08_hmg124h_10.JPG.dat	812.5	27.9	1.0	0.04	-0.027	0.039	1.7E-05
YML131W	YML131W	Y55_Ator08_hmg124h_10.JPG.dat	834.3	26.6	1.0	0.01	0.008	0.009	3.1E-05
YMR002W	YMR002W	Y55_Ator08_hmg124h_10.JPG.dat	941.5	38.8	1.1	0.01	0.144	0.014	5.6E-05
YMR003W	YMR003W	Y55_Ator08_hmg124h_10.JPG.dat	878.3	41.5	1.1	0.01	-0.001	0.013	4.9E-05
YMR004W	YMR004W	Y55_Ator08_hmg124h_10.JPG.dat	790.8	69.7	1.0	0.09	-0.025	0.088	1.6E-04
YMR006C	YMR006C	Y55_Ator08_hmg124h_10.JPG.dat	710.8	37.9	1.0	0.05	-0.136	0.053	3.9E-05
YMR007W	YMR007W	Y55_Ator08_hmg124h_10.JPG.dat	735.3	100.7	1.0	0.13	0.002	0.128	5.8E-04
YMR008C	YMR008C	Y55_Ator08_hmg124h_10.JPG.dat	895.5	56.5	1.1	0.07	0.092	0.075	8.1E-05
YMR009W	YMR009W	Y55_Ator08_hmg124h_10.JPG.dat	698.0	38.7	0.9	0.05	-0.142	0.047	4.4E-05
YMR010W	YMR010W	Y55_Ator08_hmg124h_10.JPG.dat	694.3	20.0	0.9	0.02	0.025	0.025	6.4E-06
YMR011W	YMR011W	Y55_Ator08_hmg124h_10.JPG.dat	815.0	14.3	1.0	0.02	-0.052	0.020	2.2E-06
YMR012W	YMR012W	Y55_Ator08_hmg124h_10.JPG.dat	823.8	52.4	1.0	0.07	0.007	0.068	8.1E-05
YMR015C	YMR015C	Y55_Ator08_hmg124h_10.JPG.dat	760.8	27.8	0.9	0.03	0.080	0.028	7.6E-06
YMR016C	YMR016C	Y55_Ator08_hmg124h_10.JPG.dat	807.0	36.9	1.0	0.01	0.002	0.011	4.1E-05
YMR017W	YMR017W	Y55_Ator08_hmg124h_10.JPG.dat	710.3	44.2	0.9	0.06	-0.088	0.061	9.0E-05
YMR018W	YMR018W	Y55_Ator08_hmg124h_10.JPG.dat	728.8	25.5	1.0	0.04	-0.005	0.036	1.3E-05
YMR019W	YMR019W	Y55_Ator08_hmg124h_10.JPG.dat	706.3	17.7	0.9	0.01	-0.037	0.009	2.8E-05
YMR020W	YMR020W	Y55_Ator08_hmg124h_10.JPG.dat	703.5	61.3	0.9	0.01	-0.129	0.012	6.1E-05
YMR021C	YMR021C	Y55_Ator08_hmg124h_10.JPG.dat	886.5	47.6	1.1	0.06	0.027	0.064	5.1E-05
YMR022W	YMR022W	Y55_Ator08_hmg124h_10.JPG.dat	753.0	66.9	1.0	0.07	-0.011	0.068	9.5E-05
YMR023C	YMR023C	Y55_Ator08_hmg124h_10.JPG.dat	859.5	37.5	1.1	0.05	0.046	0.049	2.5E-05
YMR025W	YMR025W	Y55_Ator08_hmg124h_10.JPG.dat	871.8	68.5	1.1	0.07	0.104	0.074	8.2E-05
YMR026C	YMR026C	Y55_Ator08_hmg124h_10.JPG.dat	831.3	36.3	1.1	0.05	0.011	0.051	3.0E-05
YMR027W	YMR027W	Y55_Ator08_hmg124h_10.JPG.dat	687.0	7.1	0.9	0.03	-0.046	0.027	7.4E-06
YMR029C	YMR029C	Y55_Ator08_hmg124h_10.JPG.dat	720.0	43.5	0.9	0.08	-0.074	0.075	1.4E-04
YMR030W	YMR030W	Y55_Ator08_hmg124h_10.JPG.dat	740.3	19.9	1.0	0.02	-0.045	0.021	2.8E-06
YMR031C	YMR031C	Y55_Ator08_hmg124h_10.JPG.dat	751.0	29.9	1.0	0.05	0.057	0.052	3.4E-05
YMR031W	YMR031W-A	Y55_Ator08_hmg124h_10.JPG.dat	699.5	7.1	1.0	0.01	-0.031	0.013	7.6E-07
YMR034C	YMR034C	Y55_Ator08_hmg124h_10.JPG.dat	725.3	15.4	1.0	0.03	-0.055	0.028	5.9E-06

YMR035W	YMR035W	Y55_Ator08_hmg124h_10.JPG.dat	766.0	122.3	1.1	0.16	0.060	0.157	8.8E-04
YMR036C	YMR036C	Y55_Ator08_hmg124h_10.JPG.dat	861.8	101.2	1.2	0.14	0.105	0.136	4.1E-04
YMR037C	YMR037C	Y55_Ator08_hmg124h_10.JPG.dat	704.5	135.2	0.9	0.18	0.021	0.178	1.8E-03
YMR038C	YMR038C	Y55_Ator08_hmg124h_10.JPG.dat	986.5	77.9	1.3	0.10	0.399	0.103	1.4E-04
YMR039C	YMR039C	Y55_Ator08_hmg124h_10.JPG.dat	888.3	25.3	1.2	0.05	0.141	0.050	2.3E-05
YMR040W	YMR040W	Y55_Ator08_hmg124h_10.JPG.dat	687.5	61.5	0.9	0.09	-0.133	0.092	3.1E-04
YMR041C	YMR041C	Y55_Ator08_hmg124h_10.JPG.dat	841.5	93.2	1.1	0.13	0.046	0.125	4.5E-04
YMR042W	YMR042W	Y55_Ator08_hmg124h_10.JPG.dat	829.0	40.0	1.1	0.05	-0.048	0.052	3.1E-05
YMR044W	YMR044W	Y55_Ator08_hmg124h_10.JPG.dat	705.3	30.0	1.1	0.04	-0.081	0.036	1.0E-05
YMR048W	YMR048W	Y55_Ator08_hmg124h_10.JPG.dat	670.8	35.2	0.9	0.05	-0.121	0.050	3.9E-05
YMR052C-,	YMR052C-A	Y55_Ator08_hmg124h_10.JPG.dat	884.0	167.0	1.2	0.23	0.249	0.235	1.8E-03
YMR052W	YMR052W	Y55_Ator08_hmg124h_10.JPG.dat	709.3	58.2	1.0	0.03	-0.046	0.031	3.5E-04
YMR053C	YMR053C	Y55_Ator08_hmg124h_10.JPG.dat	757.8	119.8	1.0	0.04	-0.128	0.036	4.6E-04
YMR054W	YMR054W	Y55_Ator08_hmg124h_10.JPG.dat	790.5	19.3	1.1	0.02	-0.005	0.024	3.4E-06
YMR055C	YMR055C	Y55_Ator08_hmg124h_10.JPG.dat	706.0	19.1	0.9	0.02	-0.117	0.021	3.2E-06
YMR056C	YMR056C	Y55_Ator08_hmg124h_10.JPG.dat	874.0	135.6	1.1	0.17	0.104	0.173	9.5E-04
YMR057C	YMR057C	Y55_Ator08_hmg124h_10.JPG.dat	886.0	141.7	1.1	0.18	0.097	0.179	1.1E-03
YMR058W	YMR058W	Y55_Ator08_hmg124h_10.JPG.dat	189.3	378.5	0.0	0.00	0.000	0.000	
YMR060C	YMR060C	Y55_Ator08_hmg124h_10.JPG.dat	553.3	365.0	1.0	0.06	-0.036	0.061	1.4E-03
YMR063W	YMR063W	Y55_Ator08_hmg124h_10.JPG.dat	671.3	46.2	0.9	0.06	-0.071	0.060	7.3E-05
YMR065W	YMR065W	Y55_Ator08_hmg124h_10.JPG.dat	713.3	49.6	1.0	0.07	-0.025	0.070	9.2E-05
YMR067C	YMR067C	Y55_Ator08_hmg124h_10.JPG.dat	752.8	27.9	1.1	0.04	0.013	0.045	2.1E-05
YMR068W	YMR068W	Y55_Ator08_hmg124h_10.JPG.dat	705.3	45.5	0.9	0.01	-0.136	0.012	5.8E-05
YMR070W	YMR070W	Y55_Ator08_hmg124h_10.JPG.dat	886.3	98.9	1.2	0.14	0.250	0.135	4.2E-04
YMR073C	YMR073C	Y55_Ator08_hmg124h_10.JPG.dat	826.8	76.0	1.1	0.10	0.020	0.096	2.0E-04
YMR075C-,	YMR075C-A	Y55_Ator08_hmg124h_10.JPG.dat	664.8	43.0	0.8	0.02	-0.140	0.021	2.2E-04
YMR075W	YMR075W	Y55_Ator08_hmg124h_10.JPG.dat	668.3	30.4	0.8	0.03	-0.123	0.032	1.4E-05
YMR078C	YMR078C	Y55_Ator08_hmg124h_10.JPG.dat	782.3	90.4	0.9	0.12	0.161	0.118	5.4E-04
YMR080C	YMR080C	Y55_Ator08_hmg124h_10.JPG.dat	961.0	45.5	1.2	0.00	0.091	0.004	3.4E-06
YMR085W	YMR085W	Y55_Ator08_hmg124h_10.JPG.dat	869.0	169.1	1.1	0.20	0.072	0.199	1.6E-03
YMR086C-,	YMR086C-A	Y55_Ator08_hmg124h_10.JPG.dat	709.0	8.8	1.0	0.00	-0.056	0.005	8.1E-06
YMR086W	YMR086W	Y55_Ator08_hmg124h_10.JPG.dat	747.0	46.3	1.1	0.07	0.001	0.070	7.6E-05
YMR087W	YMR087W	Y55_Ator08_hmg124h_10.JPG.dat	534.8	69.5	0.7	0.09	-0.266	0.086	4.3E-04
YMR088C	YMR088C	Y55_Ator08_hmg124h_10.JPG.dat	805.0	28.8	1.0	0.01	-0.038	0.007	1.5E-05
YMR092C	YMR092C	Y55_Ator08_hmg124h_10.JPG.dat	746.3	22.6	0.9	0.03	-0.096	0.030	9.2E-06
YMR095C	YMR095C	Y55_Ator08_hmg124h_10.JPG.dat	816.0	33.8	1.0	0.04	-0.102	0.042	1.9E-05
YMR096W	YMR096W	Y55_Ator08_hmg124h_10.JPG.dat	765.8	32.4	1.0	0.01	0.152	0.008	2.4E-05
YMR099C	YMR099C	Y55_Ator08_hmg124h_10.JPG.dat	808.5	60.6	1.0	0.06	-0.099	0.063	7.4E-05
YMR100W	YMR100W	Y55_Ator08_hmg124h_10.JPG.dat	941.3	35.5	1.1	0.04	-0.066	0.038	1.3E-05
YMR101C	YMR101C	Y55_Ator08_hmg124h_10.JPG.dat	942.3	89.4	1.0	0.09	0.019	0.089	1.7E-04
YMR102C	YMR102C	Y55_Ator08_hmg124h_10.JPG.dat	849.0	37.3	1.0	0.04	-0.031	0.039	1.5E-05
YMR103C	YMR103C	Y55_Ator08_hmg124h_10.JPG.dat	720.8	47.9	0.9	0.02	-0.023	0.017	1.1E-04
YMR104C	YMR104C	Y55_Ator08_hmg124h_10.JPG.dat	629.0	34.3	0.9	0.02	0.081	0.016	1.0E-04
YMR105C	YMR105C	Y55_Ator08_hmg124h_10.JPG.dat	899.3	171.9	1.2	0.25	0.117	0.252	2.3E-03
YMR106C	YMR106C	Y55_Ator08_hmg124h_10.JPG.dat	814.8	47.9	1.0	0.06	-0.010	0.061	5.5E-05
YMR107W	YMR107W	Y55_Ator08_hmg124h_10.JPG.dat	842.3	41.2	1.0	0.01	-0.137	0.010	3.2E-05
YMR109W	YMR109W	Y55_Ator08_hmg124h_10.JPG.dat	834.8	40.1	1.1	0.05	-0.127	0.052	3.1E-05
YMR110C	YMR110C	Y55_Ator08_hmg124h_10.JPG.dat	816.8	35.1	1.1	0.06	0.141	0.061	5.3E-05
YMR111C	YMR111C	Y55_Ator08_hmg124h_10.JPG.dat	855.3	18.8	1.1	0.03	0.007	0.026	4.2E-06
YMR114C	YMR114C	Y55_Ator08_hmg124h_10.JPG.dat	869.8	21.2	1.0	0.02	0.103	0.019	1.7E-06
YMR115W	YMR115W	Y55_Ator08_hmg124h_10.JPG.dat	877.0	68.1	1.0	0.08	0.122	0.075	1.1E-04
YMR116C	YMR116C	Y55_Ator08_hmg124h_10.JPG.dat	724.8	85.4	0.9	0.10	-0.026	0.099	3.4E-04
YMR119W	YMR119W	Y55_Ator08_hmg124h_10.JPG.dat	667.3	15.5	0.9	0.03	0.056	0.026	5.6E-06
YMR119W	YMR119W-A	Y55_Ator08_hmg124h_10.JPG.dat	647.0	25.3	0.9	0.05	-0.061	0.054	5.3E-05
YMR120C	YMR120C	Y55_Ator08_hmg124h_10.JPG.dat	791.5	123.5	1.0	0.15	0.053	0.148	7.8E-04
YMR121C	YMR121C	Y55_Ator08_hmg124h_10.JPG.dat	832.8	30.4	1.0	0.05	0.044	0.050	3.1E-05
YMR122C	YMR122C	Y55_Ator08_hmg124h_10.JPG.dat	777.5	24.1	1.0	0.03	-0.009	0.027	5.9E-06
YMR123W	YMR123W	Y55_Ator08_hmg124h_10.JPG.dat	572.5	39.4	0.7	0.06	0.341	0.057	1.3E-04
YMR124W	YMR124W	Y55_Ator08_hmg124h_10.JPG.dat	829.3	18.9	1.1	0.01	-0.090	0.011	2.8E-07
YMR126C	YMR126C	Y55_Ator08_hmg124h_10.JPG.dat	779.3	10.9	1.0	0.02	-0.042	0.024	4.0E-06
YMR127C	YMR127C	Y55_Ator08_hmg124h_10.JPG.dat	798.0	20.0	0.9	0.00	0.032	0.004	7.1E-06
YMR129W	YMR129W	Y55_Ator08_hmg124h_10.JPG.dat	801.0	78.9	1.0	0.09	0.018	0.091	2.3E-04
YMR130W	YMR130W	Y55_Ator08_hmg124h_10.JPG.dat	658.0	22.5	0.9	0.01	-0.010	0.014	8.4E-05
YMR132C	YMR132C	Y55_Ator08_hmg124h_10.JPG.dat	748.0	73.7	1.1	0.10	0.039	0.100	2.3E-04
YMR133W	YMR133W	Y55_Ator08_hmg124h_10.JPG.dat	660.5	24.5	0.9	0.02	-0.010	0.024	5.6E-06
YMR135C	YMR135C	Y55_Ator08_hmg124h_10.JPG.dat	850.3	85.4	1.1	0.10	0.016	0.095	1.9E-04
YMR135W	YMR135W-A	Y55_Ator08_hmg124h_10.JPG.dat	898.5	132.0	1.1	0.16	0.031	0.162	8.3E-04
YMR136W	YMR136W	Y55_Ator08_hmg124h_10.JPG.dat	803.0	50.3	1.0	0.06	0.013	0.062	6.5E-05
YMR137C	YMR137C	Y55_Ator08_hmg124h_10.JPG.dat	800.5	62.0	1.0	0.08	0.099	0.077	1.3E-04
YMR138W	YMR138W	Y55_Ator08_hmg124h_10.JPG.dat	799.5	32.6	1.0	0.01	-0.143	0.008	2.0E-05

YMR139W	YMR139W	Y55_Ator08_hmg124h_10.JPG.dat	778.3	50.4	1.0	0.02	0.048	0.016	9.6E-05
YMR140W	YMR140W	Y55_Ator08_hmg124h_10.JPG.dat	868.8	16.7	1.1	0.03	0.120	0.026	4.0E-06
YMR141C	YMR141C	Y55_Ator08_hmg124h_10.JPG.dat	908.0	88.6	1.2	0.03	0.117	0.033	2.6E-04
YMR143W	YMR143W	Y55_Ator08_hmg124h_10.JPG.dat	715.0	22.3	1.0	0.00	-0.062	0.005	7.8E-06
YMR144W	YMR144W	Y55_Ator08_hmg124h_10.JPG.dat	762.5	68.8	1.1	0.10	0.041	0.098	2.2E-04
YMR145C	YMR145C	Y55_Ator08_hmg124h_10.JPG.dat	715.8	19.5	1.0	0.04	-0.067	0.043	2.4E-05
YMR147W	YMR147W	Y55_Ator08_hmg124h_10.JPG.dat	701.8	36.8	1.0	0.03	-0.022	0.033	1.1E-05
YMR148W	YMR148W	Y55_Ator08_hmg124h_10.JPG.dat	736.5	26.6	1.0	0.05	0.076	0.048	2.9E-05
YMR152W	YMR152W	Y55_Ator08_hmg124h_10.JPG.dat	875.0	90.6	1.2	0.14	0.081	0.137	3.9E-04
YMR153C-A	YMR153C-A	Y55_Ator08_hmg124h_10.JPG.dat	769.8	153.3	1.0	0.19	0.155	0.192	1.7E-03
YMR153W	YMR153W	Y55_Ator08_hmg124h_10.JPG.dat	667.3	49.2	0.9	0.03	0.072	0.025	2.5E-04
YMR154C	YMR154C	Y55_Ator08_hmg124h_10.JPG.dat	734.8	21.4	0.9	0.03	-0.038	0.030	8.8E-06
YMR155W	YMR155W	Y55_Ator08_hmg124h_10.JPG.dat	610.3	48.5	0.8	0.06	-0.011	0.060	1.2E-04
YMR156C	YMR156C	Y55_Ator08_hmg124h_10.JPG.dat	738.0	100.1	1.0	0.13	-0.033	0.130	6.9E-04
YMR157C	YMR157C	Y55_Ator08_hmg124h_10.JPG.dat	761.5	33.3	1.0	0.05	0.013	0.050	4.0E-05
YMR158C-	YMR158C-B	Y55_Ator08_hmg124h_10.JPG.dat	823.5	85.4	1.1	0.14	0.029	0.142	5.2E-04
YMR158W	YMR158W-A	Y55_Ator08_hmg124h_10.JPG.dat	765.8	11.7	1.1	0.00	0.207	0.003	2.9E-06
YMR159C	YMR159C	Y55_Ator08_hmg124h_10.JPG.dat	789.3	105.8	1.1	0.15	0.194	0.149	6.6E-04
YMR160W	YMR160W	Y55_Ator08_hmg124h_10.JPG.dat	712.3	41.0	1.0	0.02	0.005	0.021	1.4E-04
YMR161W	YMR161W	Y55_Ator08_hmg124h_10.JPG.dat	649.8	53.6	1.0	0.02	0.066	0.017	1.0E-04
YMR162C	YMR162C	Y55_Ator08_hmg124h_10.JPG.dat	896.3	110.5	1.1	0.03	0.032	0.031	2.5E-04
YMR163C	YMR163C	Y55_Ator08_hmg124h_10.JPG.dat	775.5	62.0	1.1	0.03	0.056	0.029	2.5E-04
YMR164C	YMR164C	Y55_Ator08_hmg124h_10.JPG.dat	805.8	12.3	1.0	0.02	-0.040	0.017	1.2E-06
YMR166C	YMR166C	Y55_Ator08_hmg124h_10.JPG.dat	824.5	88.2	1.1	0.12	0.069	0.119	3.8E-04
YMR167W	YMR167W	Y55_Ator08_hmg124h_10.JPG.dat	713.0	31.8	0.9	0.04	0.020	0.045	3.3E-05
YMR169c	YMR169c	Y55_Ator08_hmg124h_10.JPG.dat	902.8	56.7	1.1	0.07	0.003	0.066	5.5E-05
YMR170C	YMR170C	Y55_Ator08_hmg124h_10.JPG.dat	736.5	130.6	1.0	0.17	0.124	0.166	1.3E-03
YMR171C	YMR171C	Y55_Ator08_hmg124h_10.JPG.dat	759.5	32.8	1.1	0.02	0.125	0.016	7.2E-05
YMR172C-A	YMR172C-A	Y55_Ator08_hmg124h_10.JPG.dat	728.3	52.6	1.0	0.07	0.146	0.074	1.0E-04
YMR172W	YMR172W	Y55_Ator08_hmg124h_10.JPG.dat	647.8	63.4	0.9	0.08	0.110	0.085	2.3E-04
YMR173W	YMR173W	Y55_Ator08_hmg124h_10.JPG.dat	742.5	159.6	0.9	0.06	0.007	0.056	1.3E-03
YMR173W	YMR173W-A	Y55_Ator08_hmg124h_10.JPG.dat	887.5	135.8	1.2	0.18	0.081	0.179	9.9E-04
YMR174C	YMR174C	Y55_Ator08_hmg124h_10.JPG.dat	821.3	67.0	1.1	0.08	0.052	0.085	1.3E-04
YMR175w	YMR175w	Y55_Ator08_hmg124h_10.JPG.dat	792.5	33.8	1.0	0.04	-0.052	0.044	2.3E-05
YMR176W	YMR176W	Y55_Ator08_hmg124h_10.JPG.dat	837.0	22.8	1.0	0.03	0.106	0.031	7.5E-06
YMR177W	YMR177W	Y55_Ator08_hmg124h_10.JPG.dat	972.8	27.8	1.1	0.01	0.113	0.012	3.8E-05
YMR178W	YMR178W	Y55_Ator08_hmg124h_10.JPG.dat	752.0	66.4	0.9	0.09	-0.093	0.089	2.7E-04
YMR179W	YMR179W	Y55_Ator08_hmg124h_10.JPG.dat	798.3	57.8	1.1	0.08	-0.017	0.080	1.2E-04
YMR180C	YMR180C	Y55_Ator08_hmg124h_10.JPG.dat	636.3	18.2	0.9	0.01	0.171	0.006	1.7E-05
YMR181C	YMR181C	Y55_Ator08_hmg124h_10.JPG.dat	685.5	19.1	1.0	0.01	0.095	0.006	1.3E-05
YMR182C	YMR182C	Y55_Ator08_hmg124h_10.JPG.dat	825.5	102.8	1.2	0.02	0.315	0.023	1.3E-04
YMR183C	YMR183C	Y55_Ator08_hmg124h_10.JPG.dat	810.0	84.6	1.0	0.10	0.047	0.101	2.5E-04
YMR186W	YMR186W	Y55_Ator08_hmg124h_10.JPG.dat	772.0	23.1	1.0	0.03	-0.114	0.034	1.2E-05
YMR187C	YMR187C	Y55_Ator08_hmg124h_10.JPG.dat	709.5	22.1	0.9	0.03	-0.026	0.031	1.1E-05
YMR188C	YMR188C	Y55_Ator08_hmg124h_10.JPG.dat	902.3	85.0	1.1	0.11	0.309	0.114	2.9E-04
YMR189W	YMR189W	Y55_Ator08_hmg124h_10.JPG.dat	907.8	57.6	1.1	0.01	-0.067	0.015	5.8E-05
YMR190C	YMR190C	Y55_Ator08_hmg124h_10.JPG.dat	0.0	0.0	0.0	0.00	0.000	0.000	
YMR191W	YMR191W	Y55_Ator08_hmg124h_10.JPG.dat	961.0	39.6	1.1	0.04	0.082	0.036	9.4E-06
YMR192W	YMR192W	Y55_Ator08_hmg124h_10.JPG.dat	790.8	80.9	1.0	0.09	-0.049	0.091	1.9E-04
YMR193C-A	YMR193C-A	Y55_Ator08_hmg124h_10.JPG.dat	650.3	15.8	1.0	0.02	0.145	0.023	3.6E-06
YMR194W	YMR194W	Y55_Ator08_hmg124h_10.JPG.dat	685.8	24.6	1.0	0.04	0.139	0.042	2.1E-05
YMR195W	YMR195W	Y55_Ator08_hmg124h_10.JPG.dat	722.8	89.9	0.9	0.13	-0.068	0.127	6.6E-04
YMR196W	YMR196W	Y55_Ator08_hmg124h_10.JPG.dat	834.5	27.7	1.1	0.03	-0.089	0.027	4.7E-06
YMR198W	YMR198W	Y55_Ator08_hmg124h_10.JPG.dat	763.5	64.1	1.0	0.09	-0.071	0.089	2.1E-04
YMR199W	YMR199W	Y55_Ator08_hmg124h_10.JPG.dat	860.3	30.3	1.1	0.01	-0.103	0.010	2.8E-05
YMR201C	YMR201C	Y55_Ator08_hmg124h_10.JPG.dat	633.3	422.7	1.1	0.02	-0.046	0.024	1.8E-04
YMR202W	YMR202W	Y55_Ator08_hmg124h_10.JPG.dat	855.0	24.1	1.0	0.04	0.012	0.038	1.4E-05
YMR204C	YMR204C	Y55_Ator08_hmg124h_10.JPG.dat	858.5	26.2	1.0	0.03	-0.069	0.032	8.7E-06
YMR205C	YMR205C	Y55_Ator08_hmg124h_10.JPG.dat	721.0	82.2	0.9	0.11	0.115	0.112	5.8E-04
YMR206W	YMR206W	Y55_Ator08_hmg124h_10.JPG.dat	727.5	101.7	0.9	0.02	-0.076	0.022	1.8E-04
YMR207C	YMR207C	Y55_Ator08_hmg124h_10.JPG.dat	740.8	78.1	1.1	0.13	0.444	0.126	4.1E-04
YMR209C	YMR209C	Y55_Ator08_hmg124h_10.JPG.dat	714.5	29.4	1.0	0.02	-0.062	0.022	2.9E-06
YMR210W	YMR210W	Y55_Ator08_hmg124h_10.JPG.dat	828.5	50.4	1.0	0.07	-0.012	0.072	9.0E-05
YMR214W	YMR214W	Y55_Ator08_hmg124h_10.JPG.dat	981.0	25.0	1.2	0.04	0.197	0.044	1.2E-05
YMR215W	YMR215W	Y55_Ator08_hmg124h_10.JPG.dat	830.8	40.0	1.0	0.06	-0.224	0.063	6.0E-05
YMR216C	YMR216C	Y55_Ator08_hmg124h_10.JPG.dat	786.0	9.2	1.0	0.02	0.191	0.020	2.1E-06
YMR219W	YMR219W	Y55_Ator08_hmg124h_10.JPG.dat	842.5	50.9	1.1	0.06	0.033	0.062	5.6E-05
YMR221C	YMR221C	Y55_Ator08_hmg124h_10.JPG.dat	934.3	12.3	1.1	0.02	-0.034	0.023	2.3E-06
YMR222C	YMR222C	Y55_Ator08_hmg124h_10.JPG.dat	760.8	46.2	0.9	0.05	-0.026	0.051	4.7E-05
YMR223W	YMR223W	Y55_Ator08_hmg124h_10.JPG.dat	970.0	53.0	1.2	0.07	0.126	0.069	5.1E-05

YMR224C	YMR224C	Y55_Ator08_hmg124h_10.JPG.dat	694.3	38.3	1.0	0.02	0.033	0.015	8.2E-05
YMR225C	YMR225C	Y55_Ator08_hmg124h_10.JPG.dat	687.8	23.0	1.0	0.01	-0.033	0.012	4.9E-05
YMR226C	YMR226C	Y55_Ator08_hmg124h_10.JPG.dat	812.3	66.2	1.1	0.10	-0.144	0.095	1.9E-04
YMR230W	YMR230W	Y55_Ator08_hmg124h_10.JPG.dat	761.3	52.0	0.9	0.06	0.100	0.065	8.8E-05
YMR232W	YMR232W	Y55_Ator08_hmg124h_10.JPG.dat	807.5	57.1	1.0	0.07	0.067	0.070	9.1E-05
YMR233W	YMR233W	Y55_Ator08_hmg124h_10.JPG.dat	857.0	52.0	1.1	0.02	0.104	0.022	1.4E-04
YMR234W	YMR234W	Y55_Ator08_hmg124h_10.JPG.dat	857.5	12.2	1.1	0.01	0.076	0.006	1.0E-05
YMR237W	YMR237W	Y55_Ator08_hmg124h_10.JPG.dat	799.8	82.6	1.0	0.11	0.059	0.107	3.4E-04
YMR238W	YMR238W	Y55_Ator08_hmg124h_10.JPG.dat	752.8	32.3	0.9	0.03	-0.071	0.033	1.2E-05
YMR241W	YMR241W	Y55_Ator08_hmg124h_10.JPG.dat	813.0	112.7	1.0	0.15	-0.080	0.151	8.7E-04
YMR243C	YMR243C	Y55_Ator08_hmg124h_10.JPG.dat	0.0	0.0	0.0	0.00	0.000	0.000	
YMR244C-,	YMR244C-A	Y55_Ator08_hmg124h_10.JPG.dat	740.3	23.8	1.0	0.03	-0.005	0.031	8.2E-06
YOR202W	YOR202W	Y55_Ator08_hmg124h_10.JPG.dat	819.2	104.5	1.0	0.09	0.010	0.088	3.7E-287
1	1	Y55_Ator08_hmg124h_11.JPG.dat	890.0	168.1	1.1	0.02	0.190	0.020	1.0E-04
2	2	Y55_Ator08_hmg124h_11.JPG.dat	741.3	78.1	1.0	0.07	-0.030	0.068	9.5E-05
3	3	Y55_Ator08_hmg124h_11.JPG.dat	702.5	56.5	1.0	0.05	0.082	0.049	3.7E-05
4	4	Y55_Ator08_hmg124h_11.JPG.dat	694.8	71.3	1.0	0.09	0.069	0.088	2.1E-04
YJR109C	YJR109C	Y55_Ator08_hmg124h_11.JPG.dat	861.0	52.6	1.0	0.07	0.046	0.065	7.0E-05
YMR244W	YMR244W	Y55_Ator08_hmg124h_11.JPG.dat	0.0	0.0	0.0	0.00	0.000	0.000	
YMR245W	YMR245W	Y55_Ator08_hmg124h_11.JPG.dat	718.0	69.4	1.0	0.08	-0.091	0.083	1.8E-04
YMR246W	YMR246W	Y55_Ator08_hmg124h_11.JPG.dat	880.0	24.0	1.1	0.04	-0.006	0.037	1.0E-05
YMR247C	YMR247C	Y55_Ator08_hmg124h_11.JPG.dat	802.5	36.9	1.0	0.05	0.027	0.048	3.2E-05
YMR250W	YMR250W	Y55_Ator08_hmg124h_11.JPG.dat	792.0	38.8	1.0	0.05	0.025	0.049	3.5E-05
YMR251W	YMR251W	Y55_Ator08_hmg124h_11.JPG.dat	705.0	13.4	0.9	0.02	-0.092	0.020	3.1E-06
YMR251W	YMR251W-A	Y55_Ator08_hmg124h_11.JPG.dat	770.3	53.1	1.0	0.02	0.064	0.020	1.4E-04
YMR252C	YMR252C	Y55_Ator08_hmg124h_11.JPG.dat	858.5	35.9	1.1	0.04	0.025	0.043	1.8E-05
YMR253C	YMR253C	Y55_Ator08_hmg124h_11.JPG.dat	838.5	67.9	1.1	0.03	0.035	0.033	3.1E-04
YMR254C	YMR254C	Y55_Ator08_hmg124h_11.JPG.dat	831.3	81.2	1.1	0.09	-0.081	0.093	1.9E-04
YMR255W	YMR255W	Y55_Ator08_hmg124h_11.JPG.dat	697.0	32.5	1.0	0.04	0.048	0.035	1.2E-05
YMR256C	YMR256C	Y55_Ator08_hmg124h_11.JPG.dat	0.0	0.0	0.0	0.00	0.000	0.000	
YMR258C	YMR258C	Y55_Ator08_hmg124h_11.JPG.dat	852.0	49.9	1.1	0.06	0.016	0.056	4.2E-05
YMR259C	YMR259C	Y55_Ator08_hmg124h_11.JPG.dat	882.5	38.3	1.0	0.04	-0.007	0.038	1.4E-05
YMR261C	YMR261C	Y55_Ator08_hmg124h_11.JPG.dat	832.8	44.9	0.9	0.06	-0.110	0.058	6.5E-05
YMR262W	YMR262W	Y55_Ator08_hmg124h_11.JPG.dat	874.5	49.9	1.0	0.06	0.056	0.056	4.7E-05
YMR263W	YMR263W	Y55_Ator08_hmg124h_11.JPG.dat	1054.0	20.7	1.3	0.04	0.138	0.038	7.9E-06
YMR264W	YMR264W	Y55_Ator08_hmg124h_11.JPG.dat	889.8	13.1	1.1	0.01	0.044	0.008	1.2E-07
YMR265C	YMR265C	Y55_Ator08_hmg124h_11.JPG.dat	876.0	21.0	1.0	0.04	0.038	0.036	1.2E-05
YMR266W	YMR266W	Y55_Ator08_hmg124h_11.JPG.dat	880.8	33.0	1.0	0.03	-0.028	0.032	8.1E-06
YMR269W	YMR269W	Y55_Ator08_hmg124h_11.JPG.dat	834.0	20.7	1.0	0.03	0.114	0.033	9.8E-06
YMR271C	YMR271C	Y55_Ator08_hmg124h_11.JPG.dat	702.5	46.6	1.0	0.05	-0.153	0.049	3.5E-05
YMR272C	YMR272C	Y55_Ator08_hmg124h_11.JPG.dat	750.5	73.8	1.0	0.03	0.012	0.029	3.0E-04
YMR273C	YMR273C	Y55_Ator08_hmg124h_11.JPG.dat	791.5	76.0	1.0	0.08	0.015	0.085	1.8E-04
YMR274C	YMR274C	Y55_Ator08_hmg124h_11.JPG.dat	718.8	73.1	0.8	0.03	-0.202	0.032	5.2E-04
YMR275C	YMR275C	Y55_Ator08_hmg124h_11.JPG.dat	866.3	19.8	1.0	0.02	-0.097	0.023	3.6E-06
YMR276W	YMR276W	Y55_Ator08_hmg124h_11.JPG.dat	870.8	40.5	1.0	0.05	-0.042	0.048	2.8E-05
YMR278W	YMR278W	Y55_Ator08_hmg124h_11.JPG.dat	765.3	29.9	0.9	0.04	-0.112	0.036	1.7E-05
YMR279C	YMR279C	Y55_Ator08_hmg124h_11.JPG.dat	808.8	64.2	1.0	0.08	-0.011	0.081	1.6E-04
YMR280C	YMR280C	Y55_Ator08_hmg124h_11.JPG.dat	875.0	20.9	1.0	0.03	0.061	0.028	5.0E-06
YMR282C	YMR282C	Y55_Ator08_hmg124h_11.JPG.dat	0.0	0.0	0.0	0.00	0.000	0.000	
YMR283C	YMR283C	Y55_Ator08_hmg124h_11.JPG.dat	823.3	83.5	1.1	0.02	0.066	0.022	1.3E-04
YMR284W	YMR284W	Y55_Ator08_hmg124h_11.JPG.dat	631.3	18.9	0.9	0.01	-0.062	0.008	2.3E-05
YMR285C	YMR285C	Y55_Ator08_hmg124h_11.JPG.dat	730.5	24.4	0.9	0.03	-0.052	0.028	7.2E-06
YMR289W	YMR289W	Y55_Ator08_hmg124h_11.JPG.dat	843.3	110.5	1.0	0.12	0.173	0.116	4.2E-04
YMR291W	YMR291W	Y55_Ator08_hmg124h_11.JPG.dat	809.3	57.8	0.9	0.06	0.023	0.065	9.1E-05
YMR292W	YMR292W	Y55_Ator08_hmg124h_11.JPG.dat	917.3	68.3	1.0	0.02	0.019	0.016	8.0E-05
YMR294W	YMR294W	Y55_Ator08_hmg124h_11.JPG.dat	830.8	84.0	1.0	0.09	0.085	0.092	2.4E-04
YMR294W	YMR294W-A	Y55_Ator08_hmg124h_11.JPG.dat	847.5	42.4	1.0	0.05	-0.040	0.055	4.4E-05
YMR295C	YMR295C	Y55_Ator08_hmg124h_11.JPG.dat	902.8	35.4	1.1	0.04	-0.010	0.039	1.4E-05
YMR297W	YMR297W	Y55_Ator08_hmg124h_11.JPG.dat	918.0	26.4	1.1	0.03	0.010	0.030	6.1E-06
YMR299C	YMR299C	Y55_Ator08_hmg124h_11.JPG.dat	0.0	0.0	0.0	0.00	0.000	0.000	
YMR300C	YMR300C	Y55_Ator08_hmg124h_11.JPG.dat	745.5	29.4	1.0	0.04	0.050	0.039	1.7E-05
YMR302C	YMR302C	Y55_Ator08_hmg124h_11.JPG.dat	702.5	46.2	1.0	0.07	-0.188	0.067	8.4E-05
YMR303C	YMR303C	Y55_Ator08_hmg124h_11.JPG.dat	732.8	83.6	1.0	0.13	0.135	0.126	6.3E-04
YMR304C-,	YMR304C-A	Y55_Ator08_hmg124h_11.JPG.dat	811.5	15.0	1.0	0.02	0.048	0.018	1.8E-06
YMR304W	YMR304W	Y55_Ator08_hmg124h_11.JPG.dat	806.0	22.5	0.9	0.03	-0.023	0.029	7.6E-06
YMR305C	YMR305C	Y55_Ator08_hmg124h_11.JPG.dat	875.8	36.9	1.0	0.02	-0.046	0.016	8.5E-05
YMR306C-,	YMR306C-A	Y55_Ator08_hmg124h_11.JPG.dat	0.0	0.0	0.0	0.00	0.000	0.000	
YMR306W	YMR306W	Y55_Ator08_hmg124h_11.JPG.dat	759.3	35.1	0.9	0.04	-0.023	0.040	2.3E-05
YMR307W	YMR307W	Y55_Ator08_hmg124h_11.JPG.dat	782.3	24.8	0.9	0.03	0.003	0.033	1.1E-05
YMR310C	YMR310C	Y55_Ator08_hmg124h_11.JPG.dat	848.0	44.2	1.0	0.06	0.105	0.061	5.6E-05

YMR311C	YMR311C	Y55_Ator08_hmg124h_11.JPG.dat	758.8	47.4	1.0	0.06	-0.094	0.058	5.5E-05
YMR312W	YMR312W	Y55_Ator08_hmg124h_11.JPG.dat	762.3	29.5	1.0	0.03	-0.093	0.030	6.5E-06
YMR313C	YMR313C	Y55_Ator08_hmg124h_11.JPG.dat	720.3	18.4	1.1	0.02	0.088	0.025	3.4E-06
YMR315W	YMR315W	Y55_Ator08_hmg124h_11.JPG.dat	823.5	132.1	1.0	0.17	-0.108	0.166	1.1E-03
YMR316C-.	YMR316C-A	Y55_Ator08_hmg124h_11.JPG.dat	851.3	39.0	1.0	0.04	-0.002	0.041	1.9E-05
YMR316C-	YMR316C-B	Y55_Ator08_hmg124h_11.JPG.dat	871.3	12.1	1.0	0.01	-0.173	0.007	1.4E-05
YMR316W	YMR316W	Y55_Ator08_hmg124h_11.JPG.dat	875.0	17.2	1.0	0.01	0.136	0.015	8.0E-07
YMR317W	YMR317W	Y55_Ator08_hmg124h_11.JPG.dat	864.0	27.5	1.0	0.04	0.059	0.039	1.4E-05
YMR318C	YMR318C	Y55_Ator08_hmg124h_11.JPG.dat	823.0	32.6	1.0	0.04	0.127	0.038	1.4E-05
YMR319C	YMR319C	Y55_Ator08_hmg124h_11.JPG.dat	771.8	23.8	1.0	0.02	-0.070	0.024	4.6E-06
YMR320W	YMR320W	Y55_Ator08_hmg124h_11.JPG.dat	694.0	31.7	0.9	0.05	-0.089	0.052	5.7E-05
YMR322C	YMR322C	Y55_Ator08_hmg124h_11.JPG.dat	711.5	34.0	0.9	0.04	-0.044	0.040	2.1E-05
YMR326C	YMR326C	Y55_Ator08_hmg124h_11.JPG.dat	734.3	52.5	1.0	0.01	-0.014	0.006	1.2E-05
YNL001W	YNL001W	Y55_Ator08_hmg124h_11.JPG.dat	806.5	15.5	1.0	0.01	-0.071	0.014	7.2E-07
YNL003C	YNL003C	Y55_Ator08_hmg124h_11.JPG.dat	679.3	139.7	1.0	0.03	0.078	0.027	2.5E-04
YNL004W	YNL004W	Y55_Ator08_hmg124h_11.JPG.dat	764.8	55.9	1.0	0.06	0.010	0.056	4.9E-05
YNL008C	YNL008C	Y55_Ator08_hmg124h_11.JPG.dat	804.8	79.1	1.0	0.09	0.113	0.094	2.0E-04
YNL009W	YNL009W	Y55_Ator08_hmg124h_11.JPG.dat	887.0	62.1	1.1	0.09	0.111	0.090	1.4E-04
YNL010W	YNL010W	Y55_Ator08_hmg124h_11.JPG.dat	693.8	57.3	0.9	0.02	0.004	0.023	2.1E-04
YNL012W	YNL012W	Y55_Ator08_hmg124h_11.JPG.dat	774.0	40.6	1.0	0.05	0.083	0.047	2.7E-05
YNL013C	YNL013C	Y55_Ator08_hmg124h_11.JPG.dat	775.3	30.0	1.0	0.03	-0.007	0.031	7.7E-06
YNL015W	YNL015W	Y55_Ator08_hmg124h_11.JPG.dat	719.5	38.1	1.0	0.04	-0.114	0.044	2.4E-05
YNL016W	YNL016W	Y55_Ator08_hmg124h_11.JPG.dat	743.5	14.0	1.1	0.03	0.055	0.027	4.9E-06
YNL020C	YNL020C	Y55_Ator08_hmg124h_11.JPG.dat	700.5	15.9	1.0	0.02	-0.044	0.023	3.6E-06
YNL021W	YNL021W	Y55_Ator08_hmg124h_11.JPG.dat	639.0	58.3	0.9	0.02	-0.116	0.022	2.1E-04
YNL022C	YNL022C	Y55_Ator08_hmg124h_11.JPG.dat	714.3	35.5	1.0	0.01	-0.077	0.013	5.1E-05
YNL023C	YNL023C	Y55_Ator08_hmg124h_11.JPG.dat	599.0	23.9	0.8	0.04	-0.195	0.039	3.2E-05
YNL024C	YNL024C	Y55_Ator08_hmg124h_11.JPG.dat	699.5	27.0	0.9	0.03	-0.110	0.032	1.2E-05
YNL027W	YNL027W	Y55_Ator08_hmg124h_11.JPG.dat	766.5	41.2	0.9	0.05	-0.045	0.049	3.7E-05
YNL028W	YNL028W	Y55_Ator08_hmg124h_11.JPG.dat	844.8	46.4	1.0	0.05	0.010	0.055	4.2E-05
YNL029C	YNL029C	Y55_Ator08_hmg124h_11.JPG.dat	899.3	17.0	1.1	0.02	0.035	0.022	2.3E-06
YNL030W	YNL030W	Y55_Ator08_hmg124h_11.JPG.dat	809.5	65.2	1.0	0.08	-0.018	0.085	1.6E-04
YNL031C	YNL031C	Y55_Ator08_hmg124h_11.JPG.dat	849.5	17.7	1.0	0.02	-0.009	0.022	2.7E-06
YNL032W	YNL032W	Y55_Ator08_hmg124h_11.JPG.dat	768.5	5.1	1.0	0.01	-0.047	0.008	1.5E-07
YNL034W	YNL034W	Y55_Ator08_hmg124h_11.JPG.dat	847.5	33.0	1.1	0.04	0.164	0.038	1.2E-05
YNL035C	YNL035C	Y55_Ator08_hmg124h_11.JPG.dat	815.3	43.5	1.1	0.06	0.014	0.064	5.4E-05
YNL037C	YNL037C	Y55_Ator08_hmg124h_11.JPG.dat	732.0	24.7	1.0	0.03	-0.022	0.025	4.3E-06
YNL040W	YNL040W	Y55_Ator08_hmg124h_11.JPG.dat	869.5	42.4	1.1	0.06	-0.116	0.055	3.4E-05
YNL041C	YNL041C	Y55_Ator08_hmg124h_11.JPG.dat	833.5	100.4	1.0	0.03	0.095	0.032	3.1E-04
YNL043C	YNL043C	Y55_Ator08_hmg124h_11.JPG.dat	863.5	54.5	1.0	0.07	0.108	0.065	7.8E-05
YNL044W	YNL044W	Y55_Ator08_hmg124h_11.JPG.dat	877.8	33.3	1.0	0.04	0.040	0.036	1.4E-05
YNL045W	YNL045W	Y55_Ator08_hmg124h_11.JPG.dat	891.0	34.8	1.0	0.03	0.072	0.034	1.0E-05
YNL046W	YNL046W	Y55_Ator08_hmg124h_11.JPG.dat	847.0	40.1	1.0	0.01	0.028	0.008	2.1E-05
YNL049C	YNL049C	Y55_Ator08_hmg124h_11.JPG.dat	818.0	30.3	1.0	0.03	-0.106	0.027	6.0E-06
YNL050C	YNL050C	Y55_Ator08_hmg124h_11.JPG.dat	832.0	27.7	1.0	0.00	0.032	0.004	6.1E-06
YNL051W	YNL051W	Y55_Ator08_hmg124h_11.JPG.dat	794.0	36.2	1.0	0.05	-0.086	0.046	3.2E-05
YNL052W	YNL052W	Y55_Ator08_hmg124h_11.JPG.dat	662.3	63.3	0.9	0.09	-0.231	0.087	2.8E-04
YNL054W	YNL054W	Y55_Ator08_hmg124h_11.JPG.dat	714.8	54.7	1.0	0.08	0.048	0.084	1.6E-04
YNL056W	YNL056W	Y55_Ator08_hmg124h_11.JPG.dat	830.3	25.7	1.1	0.03	-0.008	0.030	6.6E-06
YNL057W	YNL057W	Y55_Ator08_hmg124h_11.JPG.dat	907.8	92.2	1.1	0.11	0.028	0.107	2.7E-04
YNL058C	YNL058C	Y55_Ator08_hmg124h_11.JPG.dat	961.5	32.7	1.1	0.01	0.028	0.014	5.1E-05
YNL063W	YNL063W	Y55_Ator08_hmg124h_11.JPG.dat	903.5	27.3	1.0	0.03	0.036	0.033	8.9E-06
YNL064C	YNL064C	Y55_Ator08_hmg124h_11.JPG.dat	839.8	29.0	1.0	0.04	0.066	0.038	1.6E-05
YNL065W	YNL065W	Y55_Ator08_hmg124h_11.JPG.dat	731.8	20.0	0.9	0.02	-0.113	0.023	5.4E-06
YNL066W	YNL066W	Y55_Ator08_hmg124h_11.JPG.dat	767.5	111.7	1.0	0.05	-0.125	0.050	8.3E-04
YNL067W	YNL067W	Y55_Ator08_hmg124h_11.JPG.dat	885.8	37.3	1.1	0.05	0.069	0.049	2.4E-05
YNL068C	YNL068C	Y55_Ator08_hmg124h_11.JPG.dat	726.0	26.2	1.0	0.02	-0.006	0.015	8.3E-05
YNL069C	YNL069C	Y55_Ator08_hmg124h_11.JPG.dat	903.8	65.3	1.2	0.08	0.108	0.076	6.9E-05
YNL070W	YNL070W	Y55_Ator08_hmg124h_11.JPG.dat	834.3	31.0	1.0	0.02	-0.001	0.024	3.8E-06
YNL071W	YNL071W	Y55_Ator08_hmg124h_11.JPG.dat	821.8	47.8	1.0	0.05	-0.089	0.046	3.1E-05
YNL072W	YNL072W	Y55_Ator08_hmg124h_11.JPG.dat	940.0	56.8	1.1	0.02	0.048	0.022	1.4E-04
YNL074C	YNL074C	Y55_Ator08_hmg124h_11.JPG.dat	899.5	35.7	1.0	0.04	0.010	0.038	1.4E-05
YNL076W	YNL076W	Y55_Ator08_hmg124h_11.JPG.dat	866.8	67.7	1.0	0.01	-0.049	0.015	7.6E-05
YNL077W	YNL077W	Y55_Ator08_hmg124h_11.JPG.dat	793.0	37.9	0.9	0.01	-0.045	0.005	1.1E-05
YNL078W	YNL078W	Y55_Ator08_hmg124h_11.JPG.dat	914.3	28.5	1.0	0.01	0.017	0.013	5.5E-05
YNL079C	YNL079C	Y55_Ator08_hmg124h_11.JPG.dat	639.0	76.6	0.8	0.03	0.021	0.032	5.0E-04
YNL080C	YNL080C	Y55_Ator08_hmg124h_11.JPG.dat	559.8	377.7	1.0	0.09	-0.172	0.093	3.1E-03
YNL081C	YNL081C	Y55_Ator08_hmg124h_11.JPG.dat	695.3	179.4	1.1	0.06	0.030	0.062	1.1E-03
YNL082W	YNL082W	Y55_Ator08_hmg124h_11.JPG.dat	782.3	24.5	1.1	0.03	0.004	0.029	5.7E-06
YNL083W	YNL083W	Y55_Ator08_hmg124h_11.JPG.dat	779.3	44.6	1.0	0.01	0.154	0.007	1.9E-05

YNL085W	YNL085W	Y55_Ator08_hmg124h_11.JPG.dat	939.5	23.6	1.1	0.02	0.069	0.025	3.0E-06
YNL087W	YNL087W	Y55_Ator08_hmg124h_11.JPG.dat	739.5	106.5	0.9	0.03	-0.075	0.030	3.4E-04
YNL089C	YNL089C	Y55_Ator08_hmg124h_11.JPG.dat	863.8	19.5	1.0	0.02	-0.047	0.020	2.1E-06
YNL090W	YNL090W	Y55_Ator08_hmg124h_11.JPG.dat	844.0	11.0	1.0	0.01	-0.045	0.014	7.1E-07
YNL091W	YNL091W	Y55_Ator08_hmg124h_11.JPG.dat	856.3	55.9	1.0	0.02	-0.035	0.015	7.5E-05
YNL092W	YNL092W	Y55_Ator08_hmg124h_11.JPG.dat	878.0	64.5	1.1	0.02	0.146	0.024	1.6E-04
YNL093W	YNL093W	Y55_Ator08_hmg124h_11.JPG.dat	854.3	52.8	1.1	0.07	0.072	0.070	7.5E-05
YNL094W	YNL094W	Y55_Ator08_hmg124h_11.JPG.dat	606.0	75.0	0.8	0.02	-0.299	0.021	2.4E-04
YNL095C	YNL095C	Y55_Ator08_hmg124h_11.JPG.dat	648.3	26.0	0.9	0.01	0.006	0.012	5.1E-05
YNL097C	YNL097C	Y55_Ator08_hmg124h_11.JPG.dat	772.8	32.4	1.0	0.05	-0.114	0.046	2.5E-05
YNL098C	YNL098C	Y55_Ator08_hmg124h_11.JPG.dat	936.0	31.4	1.1	0.04	0.062	0.042	1.5E-05
YNL099C	YNL099C	Y55_Ator08_hmg124h_11.JPG.dat	804.0	13.5	0.9	0.01	-0.009	0.015	1.1E-06
YNL100W	YNL100W	Y55_Ator08_hmg124h_11.JPG.dat	866.8	4.9	1.0	0.01	0.088	0.009	2.1E-07
YNL101W	YNL101W	Y55_Ator08_hmg124h_11.JPG.dat	913.5	56.9	1.1	0.07	0.024	0.075	9.4E-05
YNL104C	YNL104C	Y55_Ator08_hmg124h_11.JPG.dat	802.5	53.7	1.0	0.07	0.063	0.069	1.0E-04
YNL105W	YNL105W	Y55_Ator08_hmg124h_11.JPG.dat	871.3	43.4	1.1	0.06	0.005	0.062	5.3E-05
YNL106C	YNL106C	Y55_Ator08_hmg124h_11.JPG.dat	905.3	41.2	1.1	0.06	0.070	0.060	4.3E-05
YNL107W	YNL107W	Y55_Ator08_hmg124h_11.JPG.dat	775.3	79.7	1.0	0.09	0.109	0.095	2.3E-04
YNL108C	YNL108C	Y55_Ator08_hmg124h_11.JPG.dat	684.0	57.9	0.9	0.08	-0.091	0.082	1.9E-04
YNL115C	YNL115C	Y55_Ator08_hmg124h_11.JPG.dat	724.8	30.0	1.0	0.04	-0.110	0.040	1.6E-05
YNL116W	YNL116W	Y55_Ator08_hmg124h_11.JPG.dat	769.0	39.1	1.0	0.05	0.019	0.053	3.7E-05
YNL117W	YNL117W	Y55_Ator08_hmg124h_11.JPG.dat	750.8	56.2	1.0	0.06	0.020	0.059	6.2E-05
YNL119W	YNL119W	Y55_Ator08_hmg124h_11.JPG.dat	754.3	50.6	1.0	0.02	-0.060	0.016	8.4E-05
YNL120C	YNL120C	Y55_Ator08_hmg124h_11.JPG.dat	801.3	59.2	1.1	0.01	0.040	0.015	6.3E-05
YNL121C	YNL121C	Y55_Ator08_hmg124h_11.JPG.dat	820.5	89.5	1.0	0.13	0.106	0.127	5.0E-04
YNL122C	YNL122C	Y55_Ator08_hmg124h_11.JPG.dat	741.3	27.0	0.9	0.01	-0.061	0.011	4.3E-05
YNL123W	YNL123W	Y55_Ator08_hmg124h_11.JPG.dat	730.0	72.9	1.0	0.02	-0.019	0.024	1.9E-04
YNL125C	YNL125C	Y55_Ator08_hmg124h_11.JPG.dat	788.5	31.2	1.0	0.05	0.084	0.047	2.6E-05
YNL127W	YNL127W	Y55_Ator08_hmg124h_11.JPG.dat	763.3	30.6	1.1	0.05	0.018	0.047	2.4E-05
YNL128W	YNL128W	Y55_Ator08_hmg124h_11.JPG.dat	708.8	30.9	1.0	0.05	0.007	0.050	3.3E-05
YNL129W	YNL129W	Y55_Ator08_hmg124h_11.JPG.dat	669.3	36.8	0.9	0.01	-0.046	0.014	7.6E-05
YNL130C	YNL130C	Y55_Ator08_hmg124h_11.JPG.dat	713.3	26.6	1.0	0.06	0.028	0.058	5.9E-05
YNL134C	YNL134C	Y55_Ator08_hmg124h_11.JPG.dat	733.8	37.7	0.9	0.01	0.023	0.009	3.3E-05
YNL135C	YNL135C	Y55_Ator08_hmg124h_11.JPG.dat	701.0	46.6	0.9	0.06	0.067	0.056	7.0E-05
YNL136W	YNL136W	Y55_Ator08_hmg124h_11.JPG.dat	812.8	132.8	1.1	0.04	0.037	0.041	4.9E-04
YNL140C	YNL140C	Y55_Ator08_hmg124h_11.JPG.dat	790.8	53.4	1.0	0.05	-0.061	0.047	3.1E-05
YNL141W	YNL141W	Y55_Ator08_hmg124h_11.JPG.dat	930.3	49.5	1.2	0.05	0.022	0.050	2.2E-05
YNL142W	YNL142W	Y55_Ator08_hmg124h_11.JPG.dat	817.5	96.8	1.0	0.10	0.099	0.101	2.7E-04
YNL143C	YNL143C	Y55_Ator08_hmg124h_11.JPG.dat	786.8	23.7	1.0	0.04	-0.065	0.041	2.1E-05
YNL144C	YNL144C	Y55_Ator08_hmg124h_11.JPG.dat	910.8	77.4	1.2	0.03	0.009	0.032	2.4E-04
YNL145W	YNL145W	Y55_Ator08_hmg124h_11.JPG.dat	857.8	28.7	1.1	0.03	-0.031	0.028	4.7E-06
YNL146W	YNL146W	Y55_Ator08_hmg124h_11.JPG.dat	748.3	53.1	1.1	0.00	0.043	0.004	5.1E-06
YNL153C	YNL153C	Y55_Ator08_hmg124h_11.JPG.dat	681.8	40.4	1.0	0.05	0.038	0.050	4.0E-05
YNL154C	YNL154C	Y55_Ator08_hmg124h_11.JPG.dat	808.3	54.4	1.1	0.06	0.065	0.064	6.1E-05
YNL155W	YNL155W	Y55_Ator08_hmg124h_11.JPG.dat	856.3	56.5	1.1	0.06	0.005	0.059	5.0E-05
YNL156C	YNL156C	Y55_Ator08_hmg124h_11.JPG.dat	852.3	33.4	1.0	0.04	0.032	0.036	1.2E-05
YNL157W	YNL157W	Y55_Ator08_hmg124h_11.JPG.dat	808.5	27.5	1.0	0.03	-0.075	0.028	6.7E-06
YNL159C	YNL159C	Y55_Ator08_hmg124h_11.JPG.dat	761.5	30.7	0.9	0.04	-0.120	0.040	2.2E-05
YNL162W	YNL162W	Y55_Ator08_hmg124h_11.JPG.dat	809.5	77.5	1.0	0.04	0.088	0.037	4.2E-04
YNL164C	YNL164C	Y55_Ator08_hmg124h_11.JPG.dat	814.5	3.9	1.0	0.01	-0.008	0.009	2.3E-07
YNL165W	YNL165W	Y55_Ator08_hmg124h_11.JPG.dat	764.3	35.9	0.9	0.05	0.114	0.047	3.5E-05
YNL166C	YNL166C	Y55_Ator08_hmg124h_11.JPG.dat	822.0	95.4	1.0	0.13	0.040	0.125	4.8E-04
YNL167C	YNL167C	Y55_Ator08_hmg124h_11.JPG.dat	732.8	52.0	1.1	0.07	0.019	0.068	7.4E-05
YNL168C	YNL168C	Y55_Ator08_hmg124h_11.JPG.dat	720.3	31.0	1.0	0.06	0.010	0.059	5.5E-05
YNL169C	YNL169C	Y55_Ator08_hmg124h_11.JPG.dat	823.0	263.9	1.0	0.35	0.064	0.345	9.0E-03
YNL170W	YNL170W	Y55_Ator08_hmg124h_11.JPG.dat	884.3	93.4	1.0	0.03	-0.070	0.026	2.2E-04
YNL171C	YNL171C	Y55_Ator08_hmg124h_11.JPG.dat	0.5	1.0	0.0	0.00	0.000	0.000	
YNL173C	YNL173C	Y55_Ator08_hmg124h_11.JPG.dat	914.3	54.4	1.1	0.07	0.043	0.066	6.0E-05
YNL175C	YNL175C	Y55_Ator08_hmg124h_11.JPG.dat	801.5	52.2	1.0	0.06	0.097	0.059	5.9E-05
YNL176C	YNL176C	Y55_Ator08_hmg124h_11.JPG.dat	809.3	40.3	1.0	0.04	0.022	0.043	2.2E-05
YNL179C	YNL179C	Y55_Ator08_hmg124h_11.JPG.dat	443.3	426.2	0.5	0.52	0.011	0.516	1.3E-01
YNL183C	YNL183C	Y55_Ator08_hmg124h_11.JPG.dat	705.3	142.1	0.9	0.17	-0.078	0.175	2.1E-03
YNL187W	YNL187W	Y55_Ator08_hmg124h_11.JPG.dat	810.5	85.6	1.0	0.03	-0.043	0.034	3.9E-04
YNL190W	YNL190W	Y55_Ator08_hmg124h_11.JPG.dat	647.8	23.1	0.9	0.02	0.026	0.025	5.0E-06
YNL191W	YNL191W	Y55_Ator08_hmg124h_11.JPG.dat	622.0	40.7	0.8	0.05	-0.026	0.054	7.5E-05
YNL192W	YNL192W	Y55_Ator08_hmg124h_11.JPG.dat	775.8	66.5	0.9	0.07	-0.179	0.072	1.2E-04
YNL193W	YNL193W	Y55_Ator08_hmg124h_11.JPG.dat	954.3	21.7	1.1	0.02	-0.012	0.024	2.7E-06
YNL194C	YNL194C	Y55_Ator08_hmg124h_11.JPG.dat	735.0	41.1	0.9	0.01	-0.005	0.013	7.5E-05
YNL195C	YNL195C	Y55_Ator08_hmg124h_11.JPG.dat	865.0	33.7	1.0	0.00	0.010	0.002	1.9E-06
YNL196C	YNL196C	Y55_Ator08_hmg124h_11.JPG.dat	770.0	26.3	0.9	0.04	0.030	0.036	1.6E-05

YNL197C	YNL197C	Y55_Ator08_hmg124h_11.JPG.dat	619.3	233.6	0.7	0.28	0.102	0.279	1.3E-02
YNL198C	YNL198C	Y55_Ator08_hmg124h_11.JPG.dat	732.8	103.4	0.9	0.12	-0.037	0.119	6.9E-04
YNL199C	YNL199C	Y55_Ator08_hmg124h_11.JPG.dat	840.0	53.1	1.1	0.01	0.070	0.011	3.5E-05
YNL200C	YNL200C	Y55_Ator08_hmg124h_11.JPG.dat	823.0	63.4	1.1	0.07	-0.057	0.071	7.8E-05
YNL201C	YNL201C	Y55_Ator08_hmg124h_11.JPG.dat	736.3	50.0	1.1	0.07	0.094	0.071	8.2E-05
YNL202W	YNL202W	Y55_Ator08_hmg124h_11.JPG.dat	726.0	27.5	1.0	0.02	-0.046	0.025	4.9E-06
YNL203C	YNL203C	Y55_Ator08_hmg124h_11.JPG.dat	864.8	44.4	1.1	0.02	-0.015	0.016	7.6E-05
YNL204C	YNL204C	Y55_Ator08_hmg124h_11.JPG.dat	823.0	41.1	1.0	0.06	0.079	0.059	6.2E-05
YNL205C	YNL205C	Y55_Ator08_hmg124h_11.JPG.dat	844.0	28.0	1.0	0.01	-0.010	0.010	3.2E-05
YNL206C	YNL206C	Y55_Ator08_hmg124h_11.JPG.dat	831.0	52.4	1.0	0.07	0.147	0.073	1.1E-04
YNL208W	YNL208W	Y55_Ator08_hmg124h_11.JPG.dat	870.8	43.9	1.1	0.05	0.038	0.048	2.5E-05
YNL211C	YNL211C	Y55_Ator08_hmg124h_11.JPG.dat	830.8	21.9	1.0	0.03	-0.085	0.025	4.4E-06
YNL212W	YNL212W	Y55_Ator08_hmg124h_11.JPG.dat	842.3	20.4	1.0	0.02	-0.070	0.020	2.0E-06
YNL214W	YNL214W	Y55_Ator08_hmg124h_11.JPG.dat	777.3	79.5	1.0	0.09	-0.050	0.090	1.9E-04
YNL215W	YNL215W	Y55_Ator08_hmg124h_11.JPG.dat	726.8	57.6	1.0	0.01	-0.022	0.010	3.4E-05
YNL217W	YNL217W	Y55_Ator08_hmg124h_11.JPG.dat	720.0	17.8	1.1	0.02	0.045	0.022	2.5E-06
YNL218W	YNL218W	Y55_Ator08_hmg124h_11.JPG.dat	872.0	64.8	1.2	0.03	-0.037	0.026	1.7E-04
YNL219C	YNL219C	Y55_Ator08_hmg124h_11.JPG.dat	989.5	46.2	1.2	0.06	0.155	0.063	4.1E-05
YNL223W	YNL223W	Y55_Ator08_hmg124h_11.JPG.dat	839.3	18.1	1.0	0.02	0.031	0.018	1.7E-06
YNL224C	YNL224C	Y55_Ator08_hmg124h_11.JPG.dat	835.8	14.6	1.0	0.02	-0.012	0.016	1.1E-06
YNL226W	YNL226W	Y55_Ator08_hmg124h_11.JPG.dat	837.3	35.7	1.0	0.04	-0.051	0.044	2.2E-05
YNL227C	YNL227C	Y55_Ator08_hmg124h_11.JPG.dat	828.0	36.0	1.1	0.02	-0.057	0.015	7.2E-05
YNL228W	YNL228W	Y55_Ator08_hmg124h_11.JPG.dat	808.0	43.2	1.0	0.05	0.018	0.049	3.1E-05
YNL229C	YNL229C	Y55_Ator08_hmg124h_11.JPG.dat	672.5	35.4	0.9	0.02	0.078	0.017	1.2E-04
YNL230C	YNL230C	Y55_Ator08_hmg124h_11.JPG.dat	727.8	41.8	1.0	0.06	-0.009	0.055	5.0E-05
YNL231C	YNL231C	Y55_Ator08_hmg124h_11.JPG.dat	657.8	59.5	0.9	0.08	-0.053	0.083	2.0E-04
YNL233W	YNL233W	Y55_Ator08_hmg124h_11.JPG.dat	591.3	31.5	0.9	0.05	-0.148	0.051	5.3E-05
YNL234W	YNL234W	Y55_Ator08_hmg124h_11.JPG.dat	881.0	101.7	1.2	0.12	0.057	0.122	3.2E-04
YNL235C	YNL235C	Y55_Ator08_hmg124h_11.JPG.dat	777.8	38.5	1.0	0.05	0.227	0.051	4.0E-05
YNL236W	YNL236W	Y55_Ator08_hmg124h_11.JPG.dat	832.8	34.3	1.0	0.04	-0.012	0.039	1.4E-05
YNL237W	YNL237W	Y55_Ator08_hmg124h_11.JPG.dat	837.0	31.0	1.0	0.01	-0.044	0.009	2.7E-05
YNL238W	YNL238W	Y55_Ator08_hmg124h_11.JPG.dat	471.5	35.1	0.6	0.05	-0.038	0.045	1.2E-04
YNL239W	YNL239W	Y55_Ator08_hmg124h_11.JPG.dat	801.0	59.0	1.0	0.02	-0.052	0.016	8.2E-05
YNL241C	YNL241C	Y55_Ator08_hmg124h_11.JPG.dat	607.3	65.7	0.8	0.01	-0.086	0.009	4.2E-05
YNL242W	YNL242W	Y55_Ator08_hmg124h_11.JPG.dat	691.0	29.9	0.9	0.05	-0.072	0.048	3.8E-05
YNL246W	YNL246W	Y55_Ator08_hmg124h_11.JPG.dat	658.8	38.4	0.9	0.05	-0.030	0.052	5.0E-05
YNL249C	YNL249C	Y55_Ator08_hmg124h_11.JPG.dat	727.8	19.4	1.0	0.04	-0.064	0.037	1.4E-05
YNL253W	YNL253W	Y55_Ator08_hmg124h_11.JPG.dat	699.8	19.2	1.0	0.02	0.077	0.016	9.2E-07
YNL254C	YNL254C	Y55_Ator08_hmg124h_11.JPG.dat	754.0	22.6	1.0	0.06	0.013	0.059	5.2E-05
YNL255C	YNL255C	Y55_Ator08_hmg124h_11.JPG.dat	691.8	53.5	0.9	0.06	0.051	0.059	7.4E-05
YNL257C	YNL257C	Y55_Ator08_hmg124h_11.JPG.dat	762.3	29.4	1.0	0.02	-0.005	0.018	1.7E-06
YNL259C	YNL259C	Y55_Ator08_hmg124h_11.JPG.dat	890.8	72.9	1.1	0.07	-0.033	0.073	7.9E-05
YNL265C	YNL265C	Y55_Ator08_hmg124h_11.JPG.dat	814.5	39.9	1.0	0.01	-0.108	0.010	3.5E-05
YNL266W	YNL266W	Y55_Ator08_hmg124h_11.JPG.dat	818.3	86.1	1.0	0.10	-0.036	0.099	2.6E-04
YNL268W	YNL268W	Y55_Ator08_hmg124h_11.JPG.dat	888.0	76.1	1.1	0.11	0.135	0.112	2.9E-04
YNL270C	YNL270C	Y55_Ator08_hmg124h_11.JPG.dat	834.0	27.7	1.0	0.04	-0.119	0.038	1.5E-05
YNL271C	YNL271C	Y55_Ator08_hmg124h_11.JPG.dat	742.3	18.5	0.9	0.03	-0.077	0.032	1.1E-05
YNL273W	YNL273W	Y55_Ator08_hmg124h_11.JPG.dat	794.8	29.1	1.0	0.06	0.043	0.057	5.1E-05
YNL274C	YNL274C	Y55_Ator08_hmg124h_11.JPG.dat	748.5	38.7	1.0	0.04	0.077	0.037	1.4E-05
YNL275W	YNL275W	Y55_Ator08_hmg124h_11.JPG.dat	704.0	32.9	1.0	0.05	0.042	0.051	3.6E-05
YNL277W	YNL277W	Y55_Ator08_hmg124h_11.JPG.dat	756.0	27.0	1.0	0.04	-0.044	0.040	1.7E-05
YNL278W	YNL278W	Y55_Ator08_hmg124h_11.JPG.dat	814.5	82.7	1.0	0.10	-0.060	0.099	2.5E-04
YNL279W	YNL279W	Y55_Ator08_hmg124h_11.JPG.dat	857.0	41.3	1.1	0.02	0.062	0.016	7.7E-05
YNL280C	YNL280C	Y55_Ator08_hmg124h_11.JPG.dat	843.8	18.5	1.0	0.02	0.004	0.016	1.1E-06
YNL281W	YNL281W	Y55_Ator08_hmg124h_11.JPG.dat	808.3	44.6	1.0	0.05	0.047	0.054	4.6E-05
YNL283C	YNL283C	Y55_Ator08_hmg124h_11.JPG.dat	739.3	30.7	0.9	0.04	-0.210	0.039	2.1E-05
YNL285W	YNL285W	Y55_Ator08_hmg124h_11.JPG.dat	815.3	40.5	1.0	0.05	-0.027	0.051	3.8E-05
YNL286W	YNL286W	Y55_Ator08_hmg124h_11.JPG.dat	864.5	30.0	1.1	0.04	0.036	0.036	1.1E-05
YNL288W	YNL288W	Y55_Ator08_hmg124h_11.JPG.dat	853.8	25.4	1.1	0.03	0.025	0.035	9.8E-06
YNL289W	YNL289W	Y55_Ator08_hmg124h_11.JPG.dat	768.5	20.4	1.1	0.01	0.094	0.009	2.6E-05
YNL291C	YNL291C	Y55_Ator08_hmg124h_11.JPG.dat	591.8	18.7	0.9	0.01	-0.094	0.006	1.5E-05
YNL292W	YNL292W	Y55_Ator08_hmg124h_11.JPG.dat	764.5	69.1	1.0	0.09	-0.035	0.095	2.3E-04
YNL293W	YNL293W	Y55_Ator08_hmg124h_11.JPG.dat	840.0	54.7	1.0	0.06	-0.081	0.058	4.8E-05
YNL294C	YNL294C	Y55_Ator08_hmg124h_11.JPG.dat	845.0	23.4	1.0	0.01	0.005	0.011	3.8E-05
YNL295W	YNL295W	Y55_Ator08_hmg124h_11.JPG.dat	868.5	5.4	1.0	0.01	-0.013	0.015	8.2E-07
YNL296W	YNL296W	Y55_Ator08_hmg124h_11.JPG.dat	610.5	407.7	1.0	0.04	-0.059	0.036	4.6E-04
YNL297C	YNL297C	Y55_Ator08_hmg124h_11.JPG.dat	805.3	26.7	1.0	0.04	-0.173	0.040	1.8E-05
[truncated: 6,885,880 more chars]
